# Performance thresholds, efficiency, and access equity in junior girls’ tennis: longitudinal analysis and bayesian forecasting of ITF rankings (2004–2029)

**DOI:** 10.1186/s13102-026-01542-x

**Published:** 2026-01-23

**Authors:** Michal Bozděch

**Affiliations:** Department of Physical Education and Social Sciences, Faculty of Sports Studies, Masaryk University, Brno, Czech Republic

**Keywords:** Performance benchmarks, Percentile thresholds, Talent identification, Load management, Geographic access equity

## Abstract

**Background:**

Percentile-based thresholds in the International Tennis Federation (ITF) junior ranking system are widely used to gauge competitive standing, yet little is known about their long-term dynamics, systemic biases, and predictive validity. Total Ranking Points (TRP) reflect cumulative achievement but are affected by tournament volume and access. Points per Event (PPE) have therefore been proposed as a complementary efficiency-based metric. The age at which thresholds are reached and the geographic distribution of top performers are critical for understanding developmental timing and equity of access.

**Objectives:**

This study aimed to (1) quantify and forecast cut-off thresholds (P_90_, P_75_, P_50_) categories using TRP and PPE; (2) examine the age of players achieving these benchmarks; (3) assess geographic representation at P_90_; and (4) test whether trajectories display linear stability or accelerating progression.

**Methods:**

A longitudinal dataset of 8,413 junior female players ranked in ITF year-end lists (2004–2024) was analysed. TRP were obtained from ITF records, while PPE were calculated as TRP divided by sanctioned tournament entries. Annual percentile cut-offs were derived using binary logistic regression. Geographic equity was assessed via country counts, Shannon diversity, and a sensitivity criterion (≥ 2 players per country). Player ages were calculated from birth year and tested with regression and Kendall’s τ. Bayesian Prophet models forecast 2025–2029 thresholds, configured for changepoint detection.

**Results:**

TRP thresholds rose exponentially, while PPE trajectories were more variable. P_90_ age remained stable at 16 years, whereas P_75_ and P_50_ trended older. Geographic access at P_90_ was stable but concentrated, with only 5–10 countries represented annually and repeated multi-player presence limited to a few nations (e.g., USA, Russia, Czechia). Forecasts indicated TRP cut-offs will continue to rise through 2029, with P_90_ exceeding 1,400 points, while PPE trends remained less stable.

**Conclusions:**

The ITF junior girls’ ranking system shows accelerating cumulative thresholds (TRP) alongside less predictable efficiency-based patterns (PPE). Elite entry age remains consistently young, broader tiers are ageing, and access to P_90_ is dominated by a small group of nations. Percentile thresholds therefore capture systemic competitiveness more than intrinsic ability, offering valuable but context-dependent insights for planning and equity in talent development.

**Supplementary Information:**

The online version contains supplementary material available at 10.1186/s13102-026-01542-x.

## Introduction

Analysing long-term patterns in the International Tennis Federation (ITF) rankings and evaluating junior female players’ progression offers critical insights into the factors shaping development and long-term success in elite tennis. Tennis players typically begin structured training between ages 7 and 10 and may enter the professional circuit in their late teens, while peak performance often occurs between 22 and 24 years [1–3]. This discrepancy underscores the need for sustained developmental planning beyond the junior years, including targeted coaching, injury prevention, efficiency-oriented performance monitoring, systematic evaluation of performance thresholds, and attention to access equity within the competitive system.

The junior-to-senior transition is particularly complex for female athletes. Only a small proportion of international U18 players progress to the upper tiers of the WTA rankings [4], with systematic reviews indicating that about 10% of U17/U18 juniors reach professional level Participation in higher-tier junior tournaments, as well as higher frequency of competitive engagement, substantially increases these chances [5]. Early systematic training and specialisation further enhance the likelihood of success: each additional year of structured practice raises the probability of attaining top rankings [6, 7], and early competitive engagement is linked to a ~ 20% increase in the chance of reaching elite status [6].

Within the ITF system, Total Ranking Points (TRP)—a cumulative metric reflecting points earned across sanctioned tournaments—have long served as the main indicator of competitiveness. While TRP capture the breadth of engagement, reliance solely on cumulative totals has been questioned for failing to account for efficiency or equity dimensions [8]. To address this, the present study introduces Points per Event (PPE), defined as TRP divided by tournament entries. PPE provides an efficiency-oriented perspective on point accumulation and may help differentiate players who achieve higher yields per tournament from those whose ranking progression is driven primarily by participation volume [5]. Considered together, TRP and PPE allow performance to be interpreted not only as cumulative achievement but also as an efficiency-dependent process that may be shaped by developmental timing and differential access to competition.

The relevance of PPE is further substantiated by several strands of performance-development literature. Talent identification frameworks emphasise the importance of efficiency-based indicators, noting that high-performing juniors frequently exhibit superior point-yield relative to match exposure rather than relying solely on participation volume [5, 8]. Moreover, studies on the relative age effect have demonstrated that early-maturing players often accumulate ranking points through increased competitive opportunity rather than superior efficiency, underscoring the need for metrics that disentangle performance quality from participation-driven advantages [9–11]. Finally, the access and equity literature highlights that unequal tournament availability can inflate cumulative point totals without necessarily reflecting underlying competitive proficiency [5, 8, 9, 12]. In combination, these perspectives provide a theoretical basis for PPE as a more discriminating indicator of performance efficiency within heterogeneous developmental and structural environments.

In addition to cumulative achievement and efficiency, two further dimensions warrant consideration. First, age distributions across percentile thresholds (P_90_, P_75_, P_50_) provide insights into developmental timing, early specialisation, and delayed maturation. Prior research suggests that successful juniors and successful seniors often represent disparate populations [11], and early specialisation is linked to both performance gains and risks of overuse injuries [8–10]. Second, geographic access equity examines whether opportunities at the top of junior tennis are broadly distributed across nations or concentrated in a small group of dominant countries [13].

Percentile thresholds (P_90_, P_75_, P_50_) thus provide an analytical framework to track competitive advantage across the junior years [14]. Yet these benchmarks may be distorted by unequal access to tournaments and resources, favouring players with greater logistical and financial support [15]. Moreover, the rising demands of junior tennis raise concerns about athletes’ ability to balance heavy competition schedules with physiological recovery [9, 10, 12]. Injury-related withdrawals are particularly prevalent among female players, reflecting the escalating strain on both juniors and professionals [16].

This multidimensional framework provides a richer understanding of competitive attainment in junior female tennis and allows evaluation of systemic dynamics over time [17–19].

The study pursued four primary objectives: (1) to quantify annual performance thresholds (P_90_, P_75_, P_50_) based on cumulative achievement (TRP) and efficiency-adjusted output (PPE); (2) to examine whether long-term percentile trajectories exhibit linear stability or accelerating, non-stationary development; (3) to analyse age distributions across percentile tiers to identify patterns of early versus delayed competitive progression; and (4) to assess geographic access equity by evaluating the concentration or diversification of nations represented within the top performance strata. Together, these objectives provide a multidimensional framework for evaluating systemic dynamics within junior female tennis.

This study offers several novel contributions to the literature. First, it leverages a 20-year longitudinal dataset, providing one of the most comprehensive examinations of systemic performance trajectories in junior female tennis. Second, it applies Bayesian Prophet forecasting—a method rarely used in this field—to model non-linear and potentially non-stationary trends in percentile thresholds. Third, it integrates the PPE framework as an efficiency-based complement to cumulative TRP, addressing longstanding concerns about participation-driven inflation in junior rankings. Finally, the inclusion of a geographic access-equity analysis extends prior work by quantifying structural imbalances in opportunity distribution across nations. Together, these contributions address gaps in existing research and offer a multidimensional perspective on competitive progression within the ITF junior system.

## Methods

### Study design & data source

This study employed a longitudinal observational design, combining retrospective analysis of historical performance data with prospective forecasting using Bayesian time-series methods. The primary aim was to examine longitudinal shifts in performance standards within junior female tennis over a twenty-year period from 2004 to 2024, complemented by forecasting future trends in these performance thresholds. Data were drawn from junior female competitors participating in the ITF World Tennis Tour Juniors (WTTJ). While the WTTJ (formerly ITF Junior Circuit) categorises tournaments into several tiers (J10, J30, J60, J100, J200, J300, and J500) based on the allocated ranking points, tier-specific distinctions were intentionally omitted from the analytical framework of this study.

Instead, this analysis aggregated players’ year-end Total Ranking Points (TRP), as publicly provided by ITF records (available at https://www.itftennis.com/), thereby generating a universal indicator of competitive achievement across athletes. In addition, Points per Event (PPE) was defined as the ratio of a player’s year-end Total Ranking Points (TRP) to the number of ITF-sanctioned tournament entries within the same calendar year: PPE_*i*_,_*y*_ = TRP_*i*_,_*y*_ / Events_*i*_,_*y*_. In this formulation, Events were counted as the total number of eligible tournament entries (singles and doubles combined) within the WTTJ calendar year. An entry was considered an event if (i) an ITF tournament identifier was provided in the year-end records, (ii) ranking points were officially credited (including walkovers and retirements), and (iii) the competition was completed within the boundaries of the calendar year (1 January–31 December). Qualifying draws were included when points were awarded under the ITF schedule. For players with zero eligible events, PPE was coded as NA and such cases were excluded from PPE-based models to avoid division by zero. Combining singles and doubles entries in the PPE denominator aligns with the ITF’s unified point-allocation framework and reflects the cumulative competitive load faced by junior athletes; sensitivity checks indicated that separating singles and doubles would not materially alter PPE-based threshold estimates.

The final dataset (supplementary material, S1 File) comprised 8,413 player-year observations, meaning that each athlete appears once for every year in which she was listed in the year-end WTTJ rankings. Because many players compete across multiple seasons, the dataset does not represent 8,413 unique individuals but rather the full set of annual performance entries used to construct longitudinal time series. In accordance with the ethical standards stated in the Declaration of Helsinki, all data utilised in this study were anonymised prior to analysis to safeguard the identities of individual athletes. Given that this study relied solely on anonymised public data provided by the ITF, no further ethical approvals were necessitated.

### Participants and inclusion criteria

The sample population included all junior female tennis players officially listed in the ITF year-end rankings from 2004 to 2024. Inclusion criteria were defined exclusively by the presence of the athletes in the published ranking as of December 31st for each respective year in the observed timeframe. Importantly, no exclusion criteria based on performance level were applied; thus, all players, irrespective of their achieved points—including those with zero Total Ranking Points—were retained to ensure an unbiased representation of the competitive landscape. To maintain analytical clarity and homogeneity, the dataset was explicitly restricted to junior female players; data from male-only or mixed-gender events were purposefully excluded from the present study.

### Variables and operational definitions

Performance thresholds were defined using annual percentile-based rankings—specifically the 90th (P_90_), 75th (P_75_), and 50th (P_50_) percentiles—calculated annually across the observed period. These percentiles represented the minimum year-end ranking points required to classify players into their respective performance tiers within the ITF junior ranking structure, thus facilitating the construction of performance-threshold time series. Thresholds were calculated separately for TRP and PPE, enabling parallel assessment of absolute and efficiency-adjusted indicators of competitive performance.

Access equity was operationalised as the geographical distribution of players attaining P_90_ status across the study period (2004–2024, excluding COVID-19 years 2020–2021). Three complementary metrics were employed: (1) the number and proportion of countries with at least one player in P_90_ each year; (2) the Shannon diversity index of country representation within P_90_, additionally expressed as the effective number of countries (exp(Shannon)); and (3) a sensitivity analysis applying a stricter criterion requiring ≥ 2 players per country to enter P_90_. For each year, both absolute counts and relative shares were derived, and repeated appearances of countries with ≥ 2 players were explicitly documented. In addition, for the ≥ 2-player criterion, annual values of the Shannon index and effective number were calculated to provide a comparable measure of diversity under this stricter threshold, ensuring methodological consistency with the primary analysis. Together, these measures allowed robust assessment of geographic access equity and its temporal stability.

In addition, player age was operationalised as the difference between the calendar year of ranking (Year) and the player’s reported year of birth. For each percentile group (P_90_, P_75_, P_50_), annual age distributions were calculated and summarised using means and standard deviations. This enabled the study to capture not only performance thresholds (TRP, PPE) but also the age-related context of players attaining these competitive benchmarks.

### Statistical analysis

To determine the likelihood that a specific TRP or PPE value corresponded to each percentile (P_90_, P_75_, P_50_), binary logistic regression models were employed. In these models, the dependent variable was dichotomously coded (0, 1), indicating whether a player exceeded the relevant performance percentile threshold. The independent predictor variable comprised the athlete’s TRP or PPE. Model validity and statistical significance were evaluated using an alpha level (α) of 0.05, and explanatory power was quantified via Nagelkerke’s R². Models meeting both the statistical significance criterion (*p* < .05) and demonstrating sufficient explanatory strength (Nagelkerke’s R² > 0.40) were considered valid and retained. The unusually high Nagelkerke R² values observed for TRP-based models reflect the quasi-deterministic structure of percentile classification within the ITF ranking system, where cumulative point totals map closely onto percentile membership; thus, these models serve primarily as inferential tools for estimating thresholds rather than for discovering novel predictive relationships. Because percentile rank is defined directly from the TRP distribution, the logistic models do not test a causal or predictive relationship. Their sole purpose is to estimate the numeric cut-off values that best separate percentile groups. Thus, the regressions serve as threshold-identification tools rather than as correlation tests. Coefficient estimates from these models (*β*₀, *β*₁) facilitated the computation of yearly threshold values necessary to achieve respective percentile rankings for both metrics.

Although percentile cut-off values can be derived directly from the empirical distribution, logistic regression was used because it provides an inferentially robust method for estimating threshold values from the underlying point–percentile relationship. This approach reduces sensitivity to distributional irregularities, allows uncertainty to be quantified through model coefficients, and enables threshold estimation even under skewed or heavy-tailed score distributions.

All logistic models were specified using a logit link function, with percentile membership coded dichotomously (0 = below threshold; 1 = at or above threshold). Class balance reflected the expected proportions for each percentile (P_90_ ≈ 10%, P_75_ ≈ 25%, P_50_ ≈ 50%). The predictor variable consisted solely of the athlete’s TRP or PPE, producing a simple, reproducible model structure for each year.

Model performance was evaluated using standard metrics including classification accuracy and AUC, which consistently indicated satisfactory discrimination (typically AUC > 0.80 for TRP-based models).

For access equity metrics, descriptive summaries were computed annually, followed by statistical evaluation of temporal trends. Both parametric (linear regression) and non-parametric (Kendall’s τ) approaches were applied to test for monotonic changes across the study period. Sensitivity analyses with ≥ 2 players per country were used to validate robustness. The same combination of linear regression and Kendall’s τ was also applied to assess temporal trends in player age distributions across percentile groups (P_90_, P_75_, P_50_), ensuring that age-related dynamics were formally evaluated rather than only described.

Missing data were minimal and handled conservatively: PPE values equal to zero-event cases were coded as NA and excluded from efficiency-specific models, and no outlier removal was performed because extreme values represented genuine competitive outcomes within the ITF structure.

### Forecasting procedures

Before conducting predictive analyses, the assumptions intrinsic to the Prophet forecasting model were thoroughly verified. Time-series stationarity—pertaining specifically to annual cut-off thresholds—was assessed using the Dickey-Fuller unit root test. Results consistently indicated non-stationarity for all examined percentiles (P_90_, P_75_, and P_50_). Prophet was selected for its capacity to model non-linear, non-stationary, and structurally complex data through trend decomposition and automatic changepoint detection, using a Bayesian framework for parameter estimation [20].

Additionally, residual independence—another vital assumption for robust forecasting—was validated through examination of autocorrelation functions (ACF). The absence of statistically significant autocorrelations among residuals verified compliance with this assumption.

Each percentile-specific performance time series was independently forecasted using Prophet, configured to detect a maximum of 19 (PPE) or 21 (TRP) changepoints within the first 80% of each data series (changepoint range = 0.8). To regularise changepoint detection and prevent model overfitting, a Laplace prior scale (τ = 0.05) was applied. Parameter estimation procedures utilised Markov Chain Monte Carlo (MCMC) methods with 2,000 sampling iterations. Forecast uncertainties were expressed using both 80% prediction intervals and 95% Bayesian credible intervals. Importantly, data from years significantly impacted by the COVID-19 pandemic (2020 and 2021) were explicitly excluded due to marked disruptions in tournament scheduling and player participation, thereby enhancing the predictive stability and validity of future threshold estimations.

In the present study, the Prophet model was used to forecast performance thresholds for the P_90_, P_75_, and P_50_ categories over a five-year horizon (2025–2029). Forecasts were derived from training data spanning 2004–2019 and 2022–2024, with missing years (2020 and 2021) excluded as noted above. A supplementary sensitivity analysis demonstrated that including the COVID-19 seasons (2020–2021) substantially widened prediction intervals and produced incoherent trend estimates, confirming that excluding these years yields more stable and interpretable forecasts. Separate forecasts were generated for TRP and PPE to enable direct comparison of cumulative and efficiency-adjusted performance trajectories. In addition, both linear and exponential curve fits were compared against the historical cut-off series (excluding 2020–2021) to evaluate whether performance thresholds followed a constant or accelerating trajectory over time.

### Software and tools used

All statistical and forecasting analyses—including descriptive statistics, binary logistic regression, and time-series modelling—were conducted using JASP (Version 0.19.1, University of Amsterdam) and the R statistical environment via RStudio (Version 4.4.3, Posit Software, PBC). Forecasts were generated using the *prophet* package in R.

## Results

According to descriptive statistics from the WTTJ year-end dataset (Table 1), the average Total Ranking Points (TRP) among female participants in 2024 was 997.3 (median: 872.6; SD: 567.3; min: 195.5; max: 3511.0). The distribution of TRP scores was positively skewed (1.6), with a small number of players achieving disproportionately high totals. Across the entire 2004–2024 period, skewness values ranged from 0.9 to 5.5 and kurtosis values from 0.5 to 40.7, with markedly inflated values during the COVID-19-affected years (2020–2021). These findings indicate that while the majority of athletes clustered within moderate scoring ranges, the distribution was consistently shaped by a minority of exceptionally high-performing players.

**Table 1:** Descriptive statistics of total ranking points (TRP), points per event (PPE), and percentile thresholds (P_90_, P_75_, P_50_) in junior female tennis (2004–2024)

Year	Percentile (*n*)			*n*	Total Ranking Points (TRP)							Points per Event (PPE)						
	*P*_90_	*P*_75_	*P*_50_		Med	M	SD	Skew.	Kurt.	Min	Max	Med	M	SD	Skewn.	Kurt.	Min	Max
2004	10	24	48	96	380.0	425.9	238.8	1.7	3.5	75.0	1415.0	15.0	18.1	13.2	2.3	6.6	3.8	74.7
2005	9	24	48	82	427.5	494.5	300.8	2.5	10.5	72.5	2146.3	18.1	23.8	25.3	4.9	28.9	3.8	195.1
2006	10	23	46	91	393.8	458.4	271.3	2.5	11.3	75.0	2013.8	17.3	21.8	17.0	2.5	7.7	2.4	100.7
2007	9	21	42	84	379.4	448.9	230.9	1.3	2.1	52.5	1341.3	14.7	20.3	17.3	3.2	13.1	3.3	111.8
2008	9	22	43	85	413.8	444.6	210.2	1.2	1.5	100.0	1082.5	16.4	19.9	16.1	4.0	21.3	5.3	123.4
2009	9	21	41	82	428.8	463.9	243.2	1.5	4.5	45.0	1553.8	17.9	21.3	14.3	2.0	5.3	1.5	82.5
2010	9	22	44	88	409.4	465.4	234.0	1.3	2.1	118.8	1395.0	15.8	21.2	14.4	2.0	4.4	6.6	82.1
2011	9	21	42	84	398.8	434.3	236.3	1.4	2.6	67.5	1345.0	14.4	20.5	19.9	3.2	11.4	3.4	118.2
2012	9	22	44	87	402.5	463.8	223.7	1.5	3.5	135.0	1430.0	15.3	19.9	14.6	3.1	13.3	5.0	102.8
2013	9	23	45	89	470.0	511.7	278.8	2.4	9.5	170.0	2017.5	16.5	23.0	20.1	3.1	12.6	4.6	126.1
2014	10	25	50	100	450.9	502.2	221.9	0.9	0.5	92.5	1190.0	17.1	22.1	15.8	2.4	8.3	4.0	107.3
2015	10	23	46	92	414.7	482.7	222.3	1.0	0.7	37.5	1168.8	18.7	22.3	12.5	1.0	1.1	2.2	67.9
2016	9	22	44	87	461.3	493.4	259.9	1.5	3.2	97.5	1566.3	16.7	22.8	17.7	2.2	4.4	4.1	83.3
2017	9	23	45	90	452.2	481.7	228.5	2.0	6.9	66.3	1588.8	17.0	20.1	12.7	1.9	4.8	3.3	77.3
2018	9	23	45	90	629.4	824.5	572.5	1.9	3.7	140.0	3100.0	22.8	37.6	40.3	2.4	5.1	7.6	183.8
2019	12	28	56	111	643.8	710.7	416.6	1.6	3.5	32.8	2356.3	22.5	31.7	37.1	5.6	41.3	2.7	335.5
2020	352	879	1757	3514	14.5	78.3	187.5	5.4	40.7	0.8	2595.5	1.4	4.1	12.9	21.2	664.5	0.03	482.5
2021	314	785	1569	3137	14.3	77.3	190.9	5.5	40.3	0.8	2367.3	1.7	5.4	16.1	12.7	232.7	0.03	371.9
2022	10	25	50	100	839.9	940.3	499.0	2.6	10.1	292.0	3578.0	32.2	40.7	35.0	3.5	15.1	10.1	224.9
2023	12	29	58	116	856.1	971.1	578.8	1.7	4.2	110.5	3659.3	29.4	43.4	49.5	4.6	28.2	4.1	406.6
2024	11	27	54	108	872.6	997.3	567.3	1.6	3.7	195.5	3511.0	29.0	39.0	30.8	2.6	8.6	9.3	177.9

For Points per Event (PPE), the distribution showed somewhat greater interannual variability compared to TRP. In 2024, the average PPE among female players was 39.0 (median: 29.0; SD: 30.8; min: 9.3; max: 177.9). The distribution was positively skewed (2.6) with a kurtosis of 8.6, indicating a minority of highly efficient players who accumulated exceptionally high average points per tournament relative to the majority. Across the 2004–2024 period, PPE values exhibited a gradual upward trend until 2017, followed by sharp inflation in 2018–2019 (means of 37.6 and 31.7, respectively), reflecting unusually high point yields in those years. The COVID-19-affected years (2020–2021) produced extreme distortions, with means collapsing to 4.1 and 5.4, skewness soaring to 21.2 and 12.7, and kurtosis inflating to 664.5 and 232.7, respectively—illustrating a highly unequal distribution where very few players scored non-zero points. Post-pandemic recovery (2022–2024) saw PPE levels stabilise at historically high values (means between 39.0 and 43.4), with skewness (2.6–4.6) and kurtosis (8.6–28.2) moderating relative to the COVID peak, but still reflecting persistent right-tailed distributions.

Taken together, these descriptive results indicate that PPE, while similarly skewed as TRP, tends to produce less extreme heavy tails under normal competitive conditions, thereby offering a more balanced metric of player efficiency. However, both metrics remain sensitive to systemic disruptions, as exemplified by the COVID-19 pandemic. Percentile-based thresholds (P_90_, P_75_, P_50_) for TRP, and analogously for PPE, are detailed in Identifying Performance cut-off Thresholds.

### Identifying performance cut-off thresholds

For each analysed year (2004–2024), binary logistic regression was conducted to estimate the optimal cut-off thresholds for the three performance index categories (P_90_, P_75_, and P_50_). The results for Total Ranking Points (TRP) are summarised in S1 Table, demonstrating that all constructed models (*n* = 63; 21 years × 3 P_*i*_ categories) were statistically significant (*p* < .001), with Nagelkerke’s R² values ranging from 0.803 to 0.999, indicating strong to near-perfect explanatory power across the entire sample. The regression coefficient β₁ was positive in all models, confirming a consistent relationship between increased Total Ranking Points and a higher probability of belonging to a more elite performance percentile. This finding suggests that players who accumulated more points by year-end were significantly more likely to meet or exceed percentile-specific performance thresholds, supporting the validity of ranking points as a performance discriminator. The very high Nagelkerke R² values observed for TRP-based models (often exceeding 0.80) reflect the quasi-deterministic relationship between cumulative point totals and percentile membership in the ITF ranking system. These models therefore function primarily as tools for threshold estimation rather than as predictive classifiers.

As an illustrative example, the logistic regression model constructed for the year 2024 aimed to estimate the probability of a player being classified within the P_50_ category. According to descriptive statistics from the WTTJ year-end dataset, the model identified a threshold value of approximately 871.42 points, above which players had a high probability of inclusion within this performance tier (TOP 50% players).

**Fig. 1:**
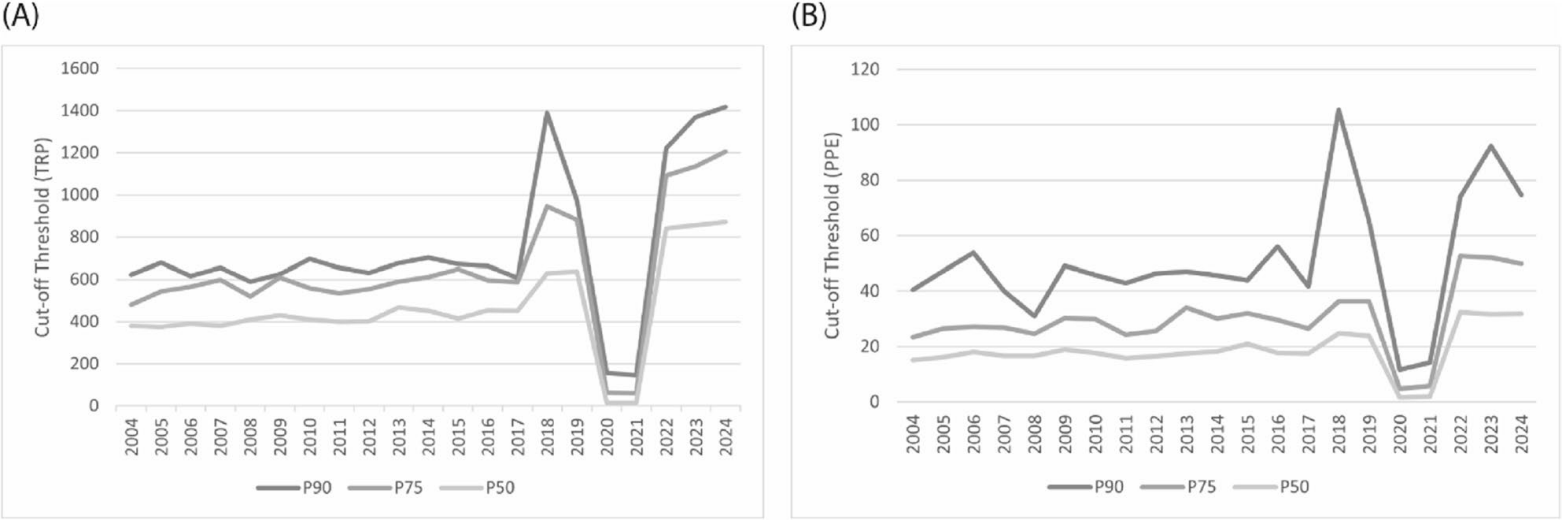
Historical development of percentile cut-off thresholds (P_90_, P_75_, P_50_) based on (**A**) Total Ranking Points and (**B**) Points per Event, 2004–2024

As illustrated in Fig. 1A, which depicts cut-off thresholds derived from Total Ranking Points (TRP), certain thresholds appeared notably close in specific years. For instance, the gap between the P_90_ and P_75_ thresholds was minimal in 2009, amounting to only 15.2 TRP. By contrast, the most pronounced disparity between these two percentiles was observed in 2018, when the difference reached 443.2 TRP. A comparable pattern emerged in the P_90_–P_50_ comparison, with the largest gap of 759.9 TRP likewise occurring in 2018. Excluding the COVID-19-affected years (2020 and 2021), the smallest differences between these performance tiers were identified in 2017 and 2008, with point gaps of 153.8 and 180.3 TRP, respectively. These findings further underscore a widening performance gradient in recent years (2022–2024), reflecting increasing competitiveness and stratification within junior tennis.

Analysis of percentile gaps derived from Points per Event (PPE) in Fig. 1B revealed substantial variability across the observation period. The narrowest gap between the P_90_ and P_75_ thresholds occurred in 2008, with a difference of only 6.5 PPE, followed by similarly small separations in 2020 (8.4 PPE) and 2013 (12.9 PPE). In contrast, the widest disparity between these two percentiles was observed in 2018, amounting to 68.9 PPE. A comparable pattern emerged in the P_90_–P_50_ comparison, with the smallest gap detected in 2008 (14.3 PPE) and the most pronounced difference again in 2018, when the separation reached 80.6 PPE. Taken together, these findings suggest an oscillating pattern over the study period, characterised by intermittent contractions (e.g., 2008, 2013, 2020) interspersed with phases of widening stratification (most notably 2018 and 2023). This dynamic underscore the shifting competitive density within junior tennis, where short-term equilibria are periodically disrupted by surges in performance differentiation.

Complementary analyses were performed using Points per Event (PPE) are summarised in S2 Table, an efficiency-adjusted measure reflecting average points obtained per tournament. Similar to TRP, all logistic regression models based on PPE (*n* = 63; 21 years × 3 P_*i*_ categories) were statistically significant (*p* < .001), with Nagelkerke’s R² values ranging from 0.380 to 0.900, indicating moderate to strong explanatory strength across years and percentiles. The regression coefficient β₁ was positive in every case, confirming that higher PPE values substantially increased the likelihood of achieving elite performance classification. This demonstrates that not only cumulative points but also per-event efficiency serves as a robust predictor of percentile membership.

For instance, the logistic regression model for 2024 targeting the P_50_ category identified a PPE cut-off of approximately 31.8 points per event. This implies that players who achieved an average point yield above this threshold per event were highly likely to rank among the top 50th percentile, regardless of the total number of tournaments entered. Notably, the 2024 cut-off for P_50_ (31.8 PPE) exceeded the cut-offs that characterised the more demanding P_25_ category during the earlier period 2004–2012, underscoring the substantial upward shift in performance requirements over time. Complementary performance metrics corroborated the adequacy of these classification models. TRP-based logistic regressions demonstrated excellent discrimination and high classification accuracy across years, whereas PPE-based models showed moderate-to-good discrimination. Calibration checks did not indicate systematic bias, supporting the use of these models as reliable tools for estimating percentile-specific cut-offs rather than as complex predictive classifiers.

Together, these results underscore that both TRP and PPE represent valid discriminators of elite performance thresholds within junior female tennis. While TRP reflects the cumulative achievement across a season, PPE provides additional insight into the efficiency of point accumulation per event. The convergence of findings across both indicators strengthens the robustness of percentile-based thresholds and ensures that interpretations of competitive standing are not solely driven by differential exposure to tournament participation.

**Fig. 2:**
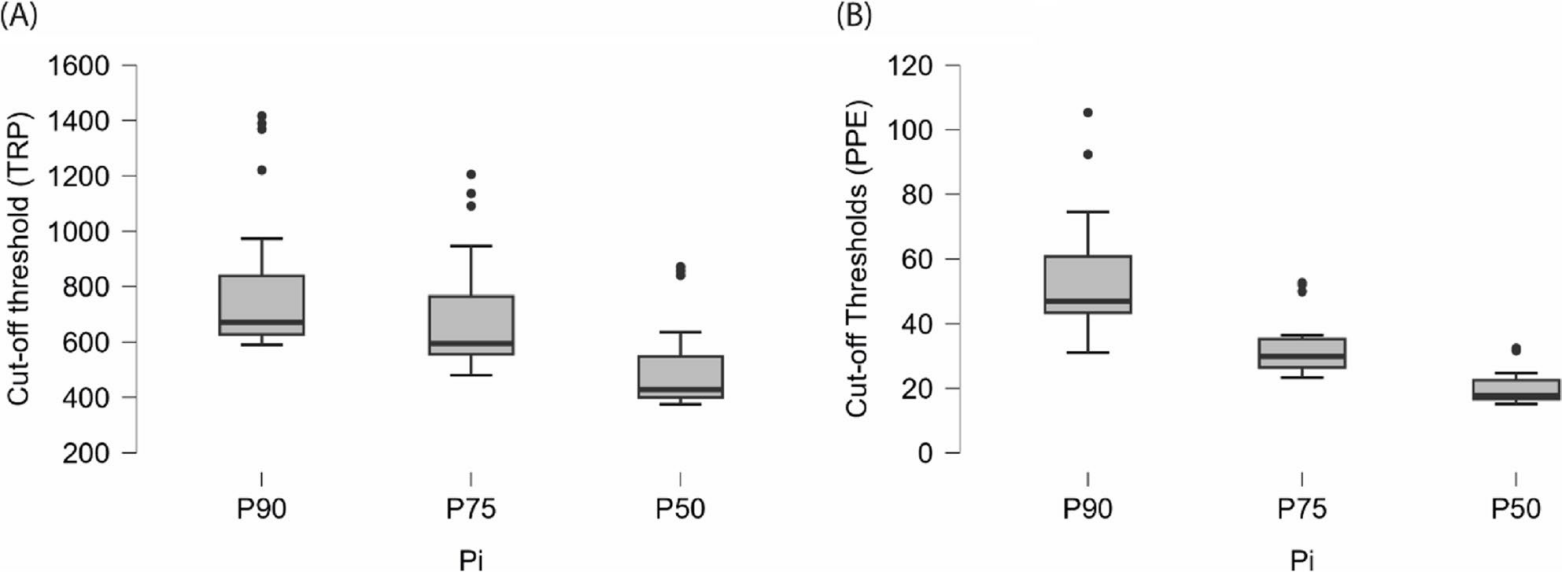
Cut-off Thresholds Based on (**A**) Total Ranking Points (TRP) and (**B**) Points per Event (PPE)

Descriptive statistics of the empirically derived cut-off thresholds for TRP and PPE (2004–2024) are provided in S3 Table, illustrating the central tendency, variability, and distributional range across all three percentiles. In addition, Fig. 2 presents boxplot visualisations of these distributions, with Panel A displaying TRP thresholds and Panel B showing PPE thresholds. Together, these supplementary materials provide a concise overview of the distributional properties of both indicators, offering a transparent baseline for subsequent forecasting analyses.

### Age of players across performance percentiles

Descriptive analyses of player ages (S4 Table) indicated that the median age of athletes attaining the P_90_ threshold remained consistently stable at 16 years across the entire study period, with only minor annual fluctuations between 16 and 17 years. In contrast, broader performance tiers exhibited a gradual increase in age. For P_75_, the median age oscillated between 16 and 17 years, with interquartile ranges (IQRs) generally spanning one year, suggesting slightly older athletes entering the upper quartile of performance over time. A similar but more pronounced pattern was observed for P_50_, where the median age more frequently reached 17 years, and the IQR confirmed that the majority of players within this group were clustered between 16 and 17 years. These distributions collectively suggest that while the very top of the performance pyramid (P_90_) is consistently achieved by relatively younger athletes, the broader groups (P_75_ and P_50_) increasingly comprise older junior players.

Trend analyses corroborated these descriptive findings. Linear regression results showed no statistically significant temporal trend in P_90_ age (slope = 0.014, *p* = .464; Kendall’s τ = 0.165, *p* = .394), confirming the stability of the median age at the highest performance threshold. Conversely, both P_75_ (slope = 0.037, *p* = .047; Kendall’s τ = 0.432, *p* = .026) and P_50_ (slope = 0.044, *p* = .011; Kendall’s τ = 0.485, *p* = .014) displayed significant upward trends, indicating a gradual aging of players achieving these thresholds over time. The explanatory power of these models was modest for P_75_ (R² = 0.213) and stronger for P_50_ (R² = 0.322), supporting the conclusion that this trend is particularly robust in the broader competitive base.

Together, these results suggest a differentiated age dynamic across performance levels in junior female tennis: the highest-performing athletes (P_90_) maintain stable entry ages, whereas progressively older age profiles emerge when broader percentile groups (P_75_ and P_50_) are considered. This finding highlights a structural feature of the junior tennis system, where early talent maturity is most critical for accessing the elite tier, while broader competitive success is increasingly achievable at older junior ages.

### Geographic access equity

Annual distributions of active countries, the number and share of countries with ≥ 1 player in P_90_, Shannon diversity index, the corresponding effective number of countries, and the list of countries with ≥ 2 players in P_90_ are summarised in Table 2.

**Table 2:** Geographic access to P_90_ in junior female tennis (2004–2024, excluding 2020–2021)

Year	Countries (total)	Countries in *P*_90_ [*n*]	Countries in *P*_90_ [%]	Shannon index	Effective number (exp(Shannon)	Countries (≥ 2 players in *P*_90_) [*n*]	Countries (≥ 2players in *P*_90_)
2004	35	8	22.86	2.03	7.58	2	Belarus (2); Chinese Taipei (2)
2005	31	8	25.81	2.04	7.72	1	Romania (2)
2006	37	10	27.03	2.30	10.00	0	—
2007	34	6	17.65	1.58	4.86	1	Russia (4)
2008	35	7	20.00	1.83	6.24	1	Romania (3)
2009	37	8	21.62	2.04	7.72	1	Australia (2)
2010	40	8	20.00	2.04	7.72	1	Czechia (2)
2011	36	9	25.00	2.20	9.00	0	—
2012	45	8	17.78	2.04	7.72	1	Canada (2)
2013	33	6	18.18	1.74	5.67	3	Czechia (2); Russia (2); USA (2)
2014	34	9	26.47	2.16	8.71	1	Spain (2)
2015	32	7	21.88	1.83	6.26	2	USA (3); Canada (2)
2016	38	5	13.16	1.46	4.33	2	Russia (3); USA (3)
2017	39	8	20.51	2.04	7.72	1	Colombia (2)
2018	38	6	15.79	1.68	5.35	2	China. P.r. (3); USA (2)
2019	43	8	18.60	1.98	7.24	3	USA (3); France (2); Russia (2)
2022	39	8	20.51	2.03	7.58	2	Czechia (2); USA (2)
2023	32	9	28.13	2.09	8.12	2	Czechia (3); Japan (2)
2024	39	8	20.51	1.97	7.19	2	USA (3); Great Britain (2)

Across 2004–2024, the number of countries with ≥ 1 player in P_90_ ranged from 5 to 10 per year, corresponding to 13–28% of all active countries in a given year. The Shannon diversity of the P_90_ composition varied between 1.46 and 2.30, i.e., an effective number of countries of roughly 4.3–10.0. A sensitivity analysis requiring ≥ 2 P_90_ players per country yielded 0–3 countries per year, producing the same qualitative pattern. Notably, countries that appeared multiple times with ≥ 2 representatives included the USA (7 years; 36.8%), Russia (5 years; 36.3%), Czechia (4 years; 21.1%), Romania (2 years; 10.5%), and Canada (2 years; 10.5%) over the 19-year observation period. Linear trend tests did not indicate systematic changes over time for any metric (all *p* ≥ .49; Kendall’s τ: all *p* ≥ .55). Together, these results suggest stable geographic access and composition of the female P_90_ across the study period, once COVID-19 years are excluded.

### Tournament participation and point accumulation efficiency

To further interpret the percentile thresholds identified for 2024, the average number of combined singles and doubles tournaments required to achieve each cut-off value was estimated. This analysis was based on the observed mean point gains per tournament in each performance tier. To reach the P_90_ cut-off of 1416.50 points, a player with the average efficiency of 104.39 ± 47.87 points per tournament would require participation in approximately 13.57 tournaments. To attain the P_75_ threshold of 1205.11 points, a player averaging 76.18 ± 40.03 points per event would need about 15.81 tournaments. For the P_50_ threshold of 871.42 points, the required number of events would rise to approximately 15.44 tournaments, assuming a mean gain of 56.44 ± 35.12 points per tournament. These approximations highlight that percentile attainment is not strictly a function of tournament frequency but rather of performance yield per event. Players in higher percentiles achieve their ranking status with fewer events due to markedly greater point efficiency. This finding reinforces the practical value of developing targeted competitive schedules and maximising performance at each tournament entry. It also illustrates how cut-off benchmarks can be reverse-engineered into strategic planning goals for players and coaches alike.

According to descriptive statistics for the 2024 season (Table 3), the mean Points per Event (PPE) across all junior female participants was 39.00 ± 30.83, with an average of 28.8 tournaments entered. When expressed as Implied Total Points—calculated as PPE multiplied by the combined number of singles and doubles tournaments per player—the overall mean reached 997.29 ± 567.25, closely aligning with observed TRP values. Importantly, subgroup analyses revealed clear performance gradients across percentiles. Players in the P_90_ tier achieved an average PPE of 104.39 ± 47.87 and required only 24.45 tournaments to accumulate an implied total of 2285.3 ± 517.28 points. In contrast, players in the P_75_ category averaged 76.18 ± 40.03 points per event, resulting in 1763.43 ± 555.37 implied total points across 25.44 tournaments. The P_50_ group exhibited the lowest efficiency, with 56.44 ± 35.12 points per event and an implied total of 1401.56 ± 537.11, despite playing a slightly higher average of 27.89 tournaments.

**Table 3:** Descriptive statistics of tournament Participation, point Accumulation, and implied performance outcomes across percentiles (2024 Season)

Category	Avg. Points/Tournament, PPE [mean ± SD]	Avg. No. of Tournaments [mean ± SD]	Total Points [mean ± SD]	Implied Total Points [mean ± SD]
Total	39.00 ± 30.83	28.8 ± 8.84	997.29 ± 567.28	997.29 ± 567.25
- P_90_	104.39 ± 47.87	24.45 ± 7.01	2285.30 ± 517.28	2285.3 ± 517.28
- P_75_	76.18 ± 40.03	25.44 ± 6.14	1763.43 ± 555.37	1763.43 ± 555.37
- P_50_	56.44 ± 35.12	27.89 ± 7.62	1401.56 ± 537.11	1401.56 ± 537.11

These results highlight two key findings. First, PPE values provide a sensitive indicator of performance efficiency, distinguishing players not merely by tournament volume but by the quality of point accumulation per entry. Second, the analysis demonstrates that athletes at higher percentiles achieve substantially greater point totals with fewer tournaments, underscoring the strategic value of maximising per-event performance. The consistency between implied totals and actual TRP further validates PPE as a robust complementary performance metric in evaluating access to and attainment of elite junior tennis benchmarks.

### Forecasting of Cut-off thresholds based on total ranking points (TRP)

The objective of this section was to forecast future cut-off threshold values for the P_90_, P_75_, and P_50_ categories over the forthcoming five-year period (2025–2029) using the Prophet model. To ensure the validity and stability of the forecasts, data from the COVID-19-affected seasons (2020 and 2021) were intentionally excluded, as these years exhibited substantial irregularities in tournament scheduling and player participation.

**Fig. 3:**
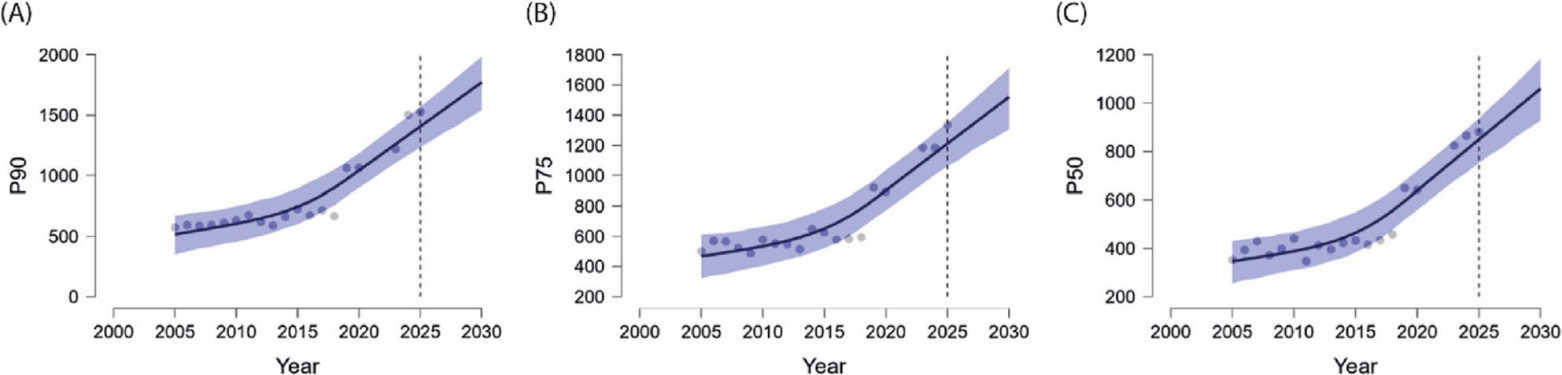
Forecasted Development of Total Ranking Points (TRP) Among Junior Female Tennis Players Across Three Percentile Categories for the Seasons 2025 to 2029. **A** Predicted P_90_ TRP cut-off values representing the top 10% of performers; (**B**) predicted P_75_ TRP cut-off values corresponding to the top quartile; and (**C**) predicted P_50_ TRP cut-off values indicating the median performance level. Bayesian Prophet modelling was used to generate projections based on historical TRP data from 2004 to 2019 and 2022 to 2024. Observed TRP data points are shown as grey dots; solid black lines represent posterior means; and shaded blue areas indicate 95% Bayesian credible intervals. Dashed vertical lines mark the start of the forecasted period

Table 4 summarises the posterior mean estimates, associated credible intervals, and convergence diagnostics (R-hat and effective sample size, ESS) for the key parameters of the forecasting models—namely, the growth rate (*k*), offset (*m*), and residual variance (*δ*)—across all three performance percentiles. These parameters offer critical insight into the projected dynamics of ITF junior ranking thresholds for girls, revealing consistent patterns of positive growth and stable model behaviour across the examined categories. Taken together, the low residual variance, narrow 95% credible intervals of the posterior parameters, and favourable convergence diagnostics (R-hat ≈ 1.00; high effective sample sizes) indicate that the model yields stable posterior estimates. The uncertainty associated with the resulting forecasts is visualised in the prediction intervals presented in Fig. 3, emphasising that the projected TRP thresholds should be interpreted as probabilistic ranges rather than exact point estimates. A supplementary sensitivity analysis demonstrated that including the COVID-19 seasons (2020–2021) substantially widened the 95% credible intervals and reduced the temporal coherence of the fitted trend, further supporting their exclusion for the purpose of deriving stable and interpretable long-term forecasts.

**Table 4:** Posterior summary statistics of forecasting model parameters for total ranking points (TRP) Cut-Off thresholds

Parameter	*P*_i_	Mean	SD	95% CI		*R*-hat	ESS (bulk)	ESS (tail)
				Lower	Upper			
Growth rate (*k*)	P_90_	0.219	0.183	-0.132	0.588	1.001	1621	2016
	P_75_	0.189	0.190	-0.167	0.576	1.002	1160	1608
	P_50_	0.185	0.186	-0.158	0.573	1.002	1204	1366
Offset (*m*)	P_90_	0.338	0.043	0.246	0.414	1.002	1646	1750
	P_75_	0.349	0.045	0.247	0.429	1.001	1434	1561
	P_50_	0.393	0.044	0.295	0.467	1.003	1240	1461
Residual variance (δ)	P_90_	0.071	0.020	0.042	0.118	1.002	1273	1898
	P_75_	0.073	0.021	0.043	0.121	1.002	1471	2299
	P_50_	0.068	0.021	0.039	0.117	1.001	1323	1845

The parameter Growth rate (*k*), which reflects the projected rate of change in the forecasted trends for the cut-off thresholds, exhibited positive posterior mean values across all P_*i*_ categories: 0.219 (SD = 0.183) for P_90_, 0.189 (SD = 0.190) for P_75_, and 0.185 (SD = 0.186) for P_50_. Although the 95% credible intervals marginally included zero in each case (e.g., − 0.132 to 0.588 for P_90_), the overall direction of the estimates suggests a continued upward trend in the performance thresholds for junior female players. This implies that progressively higher ITF ranking points will likely be required to reach elite percentile categories in the coming years, reinforcing the intensification of competitive standards.

The Offset (*m*) parameter, which represents the baseline level or intercept of the forecasted trajectory, also returned positive posterior means for all performance categories: 0.338 (SD = 0.043) for P_90_, 0.349 (SD = 0.045) for P_75_, and 0.393 (SD = 0.044) for P_50_. The corresponding 95% credible intervals for each percentile (e.g., 0.246 to 0.414 for P_90_) were entirely above zero, indicating that baseline values for performance thresholds are expected to remain robust. From a practical standpoint, this suggests that even in the absence of extraordinary performance surges, players will need to sustain or improve their ranking-point totals in order to remain within or advance toward higher percentile categories.

The Residual variance (*δ*) values were relatively low and consistent across categories—0.071 (SD = 0.020) for P_90_, 0.073 (SD = 0.021) for P_75_, and 0.069 (SD = 0.021) for P_50_—indicating well-fitted models with minimal unexplained fluctuation. Additionally, convergence diagnostics for all parameters, including R-hat (≈ 1.001–1.003) and effective sample sizes (ESS), confirmed model stability and reliability across all chains.

Changepoint analysis for P_90_ (TRP) identified a total of 14 changepoints distributed between 2005 and 2018 across the time series. Although not all changepoints yielded statistically significant deviations in the growth rate (as indicated by 95% credible intervals overlapping zero), the majority of them exhibited positive mean changes in slope (*δ*), particularly from 2011 onwards. This pattern suggests multiple localised periods of acceleration in performance thresholds among junior female players. Such periods may be attributed to shifts in competitive density, structural adjustments in the ITF tournament hierarchy, or strategic changes in player development practices. Importantly, the repeated identification of changepoints over consecutive years (e.g., 2011–2015) reinforces the hypothesis of sustained upward pressure on ranking standards throughout the examined period.

The concentration of changepoints between 2005 and 2018 corresponds to several well-documented structural shifts within the ITF junior system, including expansions of the tournament calendar, modifications to point allocations across event tiers, and increasing globalisation of the junior circuit. In particular, the year 2018 marked a substantial reform of the ITF junior ranking structure: point scales were redesigned to place greater emphasis on performance at high-tier events (Grand Slams, Grade A, Grade 1), point values for advanced rounds were markedly increased, and a global conversion to the new point table produced an abrupt upward shift in ranking totals. Although the underlying counting rules (six best results and one quarter of doubles points) remained unchanged, the elevated point values effectively inflated overall scores. These adjustments likely contributed to local accelerations in the competitive landscape, producing the identifiable slope changes captured by the model.

Taken together, the upward shift in the growth rate (*k*), strong positive offset (*m*), and low residual variance (*δ*) all indicate that the cut-off thresholds for achieving P_90_, P_75_, and P_50_ status in junior female tennis —when measured by Total Ranking Points (TRP)— are expected to continue their upward trajectory through 2029. These forecasted developments are presented visually in Fig. 3, illustrating the predicted evolution of performance standards across the three percentile categories.

After excluding data points from the COVID-19 period (2020 and 2021), the remaining historical TRP series displayed a stronger fit to an exponential rather than a linear trend, as indicated by the coefficient of determination (R²). The exponential models demonstrated high explanatory power across all percentile categories: P_90_ (R² = 0.693), P_75_ (R² = 0.816), and P_50_ (R² = 0.842). These values suggest that the evolution of TRP-based performance thresholds among junior female players has not followed a linear progression, but rather an accelerating trajectory—particularly pronounced within the median and upper-middle tiers. This pattern is consistent with a broader intensification of competitive standards over time and supports the use of flexible, non-linear forecasting approaches. For transparency, exploratory model comparisons (AIC/BIC and residual-based fit indices) consistently favoured exponential over linear specifications, providing further evidence that the long-term evolution of TRP thresholds follows an accelerating rather than a constant rate of change.

### Forecasting of Cut-off thresholds based on points per event (PPE)

Table 5 summarises the posterior mean estimates, standard deviations, and 95% credible intervals for the key parameters of the forecasting models—growth rate (*k*), offset (*m*), and residual variance (δ)—across the three performance percentiles (P_90_, P_75_, P_50_) when using Points per Event (PPE) as the performance indicator. Convergence diagnostics (R-hat ≈ 1.001–1.003 and ESS values well above 1,000) confirmed model stability and reliability across all chains.

**Table 5:** Posterior summary statistics of forecasting model parameters for points per event (PPE) Cut-Off thresholds

Parameter	*P*_i_	Mean	SD	95% CI		*R*-hat	ESS (bulk)	ESS (tail)
				Lower	Upper			
Growth rate (*k*)	P_90_	0.203	0.239	-0.264	0.666	1.001	2099	1954
	P_75_	0.232	0.256	-0.297	0.719	1.003	1889	2213
	P_50_	0.216	0.252	-0.275	0.733	1.001	1641	2201
Offset (*m*)	P_90_	0.375	0.098	0.178	0.571	1.000	2688	2511
	P_75_	0.447	0.108	0.238	0.660	1.001	2664	2627
	P_50_	0.461	0.109	0.245	0.675	1.002	2065	2506
Residual variance (*δ*)	P_90_	0.217	0.038	0.158	0.304	1.001	3797	2366
	P_75_	0.237	0.043	0.172	0.336	1.002	4255	2131
	P_50_	0.249	0.044	0.180	0.352	1.001	3587	2355

The growth rate (*k*) exhibited positive posterior mean values across all performance percentiles (P_90_: 0.203, P_75_: 0.232, P_50_: 0.216), although the associated 95% credible intervals included zero in all cases. This pattern indicates a generally upward trend in forecasted PPE thresholds but with moderate statistical uncertainty regarding the precise rate of increase. Nevertheless, the consistency of positive means across percentiles suggests that achieving higher percentile rankings will require slightly higher average points per event in forthcoming seasons.

The offset parameter (*m*) returned robust positive values across all categories (P_90_: 0.375, P_75_: 0.447, P_50_: 0.461), with 95% credible intervals entirely above zero. This finding indicates a stable baseline level of PPE thresholds, implying that performance standards in terms of efficiency per event are unlikely to decrease. Instead, players will need to maintain or slightly elevate their average points per event to remain competitive at the respective percentile levels.

Residual variance (δ) values were generally low but slightly higher than those observed for TRP models, particularly in the P_75_ (0.237) and P_50_ (0.249) categories. These values suggest that while the forecasting models fit the PPE data adequately, greater unexplained variability remains when performance is expressed on a per-event basis compared to aggregate point totals.

The PPE-based model for P_90_ also identified 14 changepoints (out of a maximum of 19 possible), distributed predominantly between 2005 and 2018. Estimated changes in growth rate (δ) ranged from 0.0001 (2006) to 0.0178 (2011), with predominantly positive mean values during the period 2009–2015. This pattern suggests localised accelerations in the growth of PPE thresholds at the upper performance tier. However, as all 95% credible intervals overlapped zero, none of these changes reached clear statistical significance. Overall, the results indicate a stable long-term trend, characterised by only modest year-to-year fluctuations in growth dynamics at the P_90_ level.

**Fig. 4:**
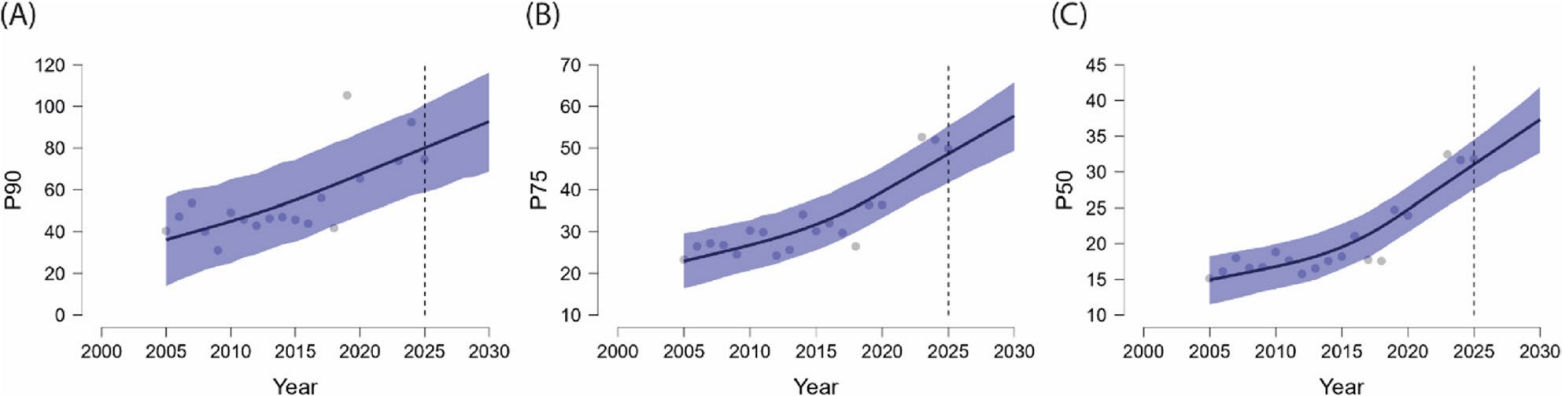
Forecasted Development of Points per Event (PPE) Among Junior Female Tennis Players Across Three Percentile Categories for the Seasons 2025 to 2029. **A** Predicted P_90_ PPE cut-off values representing the top 10% of performers; (**B**) predicted P_75_ PPE cut-off values corresponding to the top quartile; and (**C**) predicted P_50_ PPE cut-off values indicating the median performance level. Bayesian Prophet modelling was used to generate projections based on historical TRP data from 2004 to 2019 and 2022 to 2024. Observed PPE data points are shown as grey dots; solid black lines represent posterior means; and shaded blue areas indicate 95% Bayesian credible intervals. Dashed vertical lines mark the start of the forecasted period

Taken together, the upward shift in the growth rate (*k*), strong positive offset (*m*), and low residual variance (*δ*) all indicate that the cut-off thresholds for achieving P_90_, P_75_, and P_50_ status in junior female tennis —when measured by Points per Event (PPE)— are expected to continue their upward trajectory through 2029 (similar to Fig. 3). These forecasted developments are presented visually in Fig. 4, illustrating the predicted evolution of performance standards across the three percentile categories.

Taken together, the forecasting analysis indicates that PPE thresholds across the P_90_, P_75_, and P_50_ categories are expected to follow a gradually rising trajectory over the 2025–2029 period, though with slightly greater year-to-year variability than TRP. These findings suggest that average efficiency per tournament entry will remain a relevant and increasingly demanding benchmark for junior players aspiring to reach or maintain elite percentile status.

After excluding data points from the COVID-19 period (2020 and 2021), the remaining historical PPE series showed only a weak fit to an exponential model, with coefficients of determination (R²) markedly lower than those observed for TRP. Specifically, the explanatory power was limited across all percentile categories: P_90_ (R² = 0.232), P_75_ (R² = 0.271), and P_50_ (R² = 0.233). These values indicate that the evolution of PPE-based performance thresholds among junior female players did not follow a clear accelerating trajectory, but rather reflected substantial variability and noise within the data. The weak model fit suggests that PPE is less reliable for capturing long-term structural patterns in competitive intensity, underscoring the need for caution when applying non-linear forecasting approaches to this variable.

When comparing forecasting results based on P_90_ Total Ranking Points (TRP) and Points per Event (PPE), several structural differences emerge. TRP models displayed stronger and more frequent changepoint dynamics, reflecting abrupt shifts in competitive intensity across the observed period. By contrast, PPE thresholds evolved more gradually, with only minor, statistically non-significant local accelerations concentrated primarily between 2009 and 2015. From an applied perspective, TRP captures the cumulative advantage of frequent tournament participation, while PPE highlights efficiency on a per-event basis. The combination of both indicators thus provides a complementary view: TRP emphasises the accumulation of opportunities, whereas PPE underlines consistency and quality of performance at each entry. Together, they form a more comprehensive framework for assessing future performance thresholds in junior tennis.

## Discussion

### Performance benchmarks and classification

The ITF ranking points and their associated performance percentiles (P_90_, P_75_, P_50_) provide meaningful benchmarks in junior female tennis. These thresholds serve dual functions: they allow longitudinal tracking of systemic competitiveness while also offering individualised targets for player development [2, 21]. Percentile-based classification facilitates a more nuanced assessment of competitive positioning compared to absolute ranks or raw point totals, especially in contexts where access to tournaments and resources may be uneven [22]. In the present study, two complementary indicators were employed: Total Ranking Points (TRP), representing cumulative achievement, and Points per Event (PPE), reflecting efficiency of point accumulation per tournament entry.

The present study confirmed that statistically robust logistic regression models can reliably estimate whether a given ITF point total is sufficient to reach specific performance percentiles by the end of the competitive season. For TRP, all models demonstrated very high explanatory power (Nagelkerke’s R² ≥ 0.803), whereas for PPE the explanatory strength was somewhat lower but still significant across years (R² = 0.380–0.900). These results align with prior research validating logistic approaches for classification within ranking systems [23]. The findings indicate that percentile membership can be validly discriminated not only by cumulative point totals but also by per-event efficiency, thereby broadening the methodological toolkit available for evaluating junior competitive performance.

### Forecasting insights and systemic trends

Forecasting results supported the notion that junior female tennis has experienced non-linear development over the last two decades, with exponential rather than linear growth in cut-off thresholds. When TRP was employed, exponential models provided strong fits (R² up to 0.842), and Prophet forecasts projected that by 2029 the expected cut-off thresholds for P_90_, P_75_, and P_50_ will reach approximately 1413, 1203, and 889 points, respectively. By contrast, PPE trajectories displayed weaker exponential fits (R² ≤ 0.271) and greater year-to-year variability, suggesting that efficiency-based benchmarks rise more gently and less predictably over time. Nevertheless, while percentile thresholds (TRP) have risen exponentially and are forecasted to continue increasing, analogous research in Olympic disciplines has shown that human performance improvements in certain events are approaching stagnation or physiological limits [13, 24]. This suggests that the escalation of thresholds in junior tennis may not be unlimited but could eventually plateau as natural boundaries of human performance are encountered. This observation is consistent with recent research showing that post-pandemic shifts in training regimes, travel, and tournament access have accelerated competitive stratification [8]. Taken together, TRP captures the intensification of cumulative achievement, while PPE highlights efficiency gains that differentiate higher-performing athletes even when the number of tournaments played remains comparable.

The identification of 14 changepoints in each percentile trajectory (TRP)—concentrated primarily between 2005 and 2018—suggests periods of structural evolution in the WTTJ system. These inflection points likely correspond to broader developments in junior tennis, including the internationalisation of tournament calendars, revised point structures, and growing participation density and competitiveness [19]. Such changepoint patterns align with observed structural transitions in other competitive systems [25]. Comparable though weaker changepoint dynamics were observed when PPE was used, with most accelerations modest and statistically inconclusive, further confirming that cumulative measures are more sensitive to systemic restructuring than efficiency-based ones.

Although Prophet projected that P_90_ TRP thresholds may exceed ~ 1,400 points by 2029, these values must be interpreted in light of the logistical and structural constraints of the ITF junior system. Ranking points are earned exclusively through competitive participation, and junior players face practical limits on annual tournament volume due to travel demands, age-based workload recommendations, and regulatory caps. In the 2024 dataset, P_90_ athletes participated in an average of 24.45 tournaments and achieved a mean PPE of 104.39 points per event. At this efficiency level, reaching a threshold of ~ 1,400 TRP would require only 13–15 tournaments, meaning that further increases in TRP could arise through efficiency gains alone without necessitating increases in tournament volume. However, if elite juniors are already operating near their performance ceilings in the majority of events, additional tournament entries may be needed to support further TRP growth. These considerations highlight that Prophet forecasts reflect systemic momentum rather than guaranteed attainability and may overestimate future thresholds if tournament availability, scheduling density, or player workload constraints limit athletes’ capacity to accumulate ranking points.

A marked inflation in percentile thresholds in 2018 is attributable to a major ITF reform of the junior ranking system implemented that year. The point tables were restructured to place greater emphasis on high-tier events (Grand Slams, Grade A and Grade 1), with substantially increased point values for advanced rounds and a global conversion of all existing player totals to the new scale. Although the counting rules (six best singles results and one quarter of doubles points) remained unchanged, the recalibration of point allocations produced an abrupt upward shift in TRP across the junior circuit. As a result, TRP values from 2018 onwards are not directly comparable to those of earlier years on a raw-point basis, and inflationary discontinuities should be understood as artefacts of systemic restructuring rather than sudden changes in competitive quality.

### Equity and access

Despite this, it is important to acknowledge potential structural biases in the ITF junior ranking system that may influence point accumulation. While the data do not indicate significant differences in tournament volume between groups, access to tournaments may still vary based on external factors. Geographic location, financial resources, parental support, and federation-level assistance can limit some athletes’ ability to travel and compete internationally. Consequently, certain players may be underrepresented in higher percentile categories—not due to lower tennis proficiency but because of reduced access to ranking opportunities. This access-related bias may distort the true competitive hierarchy, elevating athletes who are more frequently exposed to the international circuit irrespective of relative ability. Addressing this structural inequity remains essential to fostering a fair and merit-based developmental pathway in junior tennis [15]. The geographic equity analysis presented here confirmed that access to P_90_ remains concentrated, with only 5–10 countries represented per year (13–28% of all active nations), and multi-player representation (≥ 2 athletes per country) repeatedly restricted to a small subset of nations such as the USA, Russia, and Czechia. No systematic trend towards diversification was identified, underscoring the persistence of opportunity gaps across the observation period.

### Developmental trends and age structure

Age-related analyses provided further insight into structural dynamics. While the median age of athletes reaching the P_90_ threshold remained stable at 16 years across the entire period, significant upward trends were observed for P_75_ and P_50_ groups, indicating that broader tiers are increasingly populated by older players. This pattern suggests that early breakthroughs remain critical for accessing the elite tier, whereas pathways into broader competitive strata may accommodate delayed development. This observation is consistent with systematic evidence showing that peak performance ages vary substantially across sports, with tennis players generally peaking earlier than endurance athletes but still exhibiting a progressive ageing trend in recent decades [1]. Moreover, longitudinal syntheses have demonstrated that successful juniors and successful seniors often constitute two disparate populations, with most early achievers failing to replicate equivalent success at senior level [11]. Such findings have implications for talent identification and support strategies, highlighting the importance of monitoring both early- and late-developing athletes.

The continuous upward trend in cut-off thresholds has critical implications for the design and implementation of development pathways. High-performing junior female athletes increasingly require early exposure to international-level events, targeted periodisation, and support systems capable of sustaining competitive momentum [5]. Forecasting tools such as those demonstrated in this study can aid national federations and training centres in anticipating the demands of future seasons, allowing for more efficient resource allocation and long-term planning. At the same time, the dual consideration of TRP and PPE suggests that development strategies must address not only the pursuit of cumulative points but also the optimisation of per-event efficiency. Reverse-engineering of percentile cut-offs into implied tournament counts and required PPE yields indicates that players in the highest percentiles secure their status with fewer tournaments due to substantially greater efficiency. Such insights can be translated into more rational calendar design and load management, mitigating the risks of over-scheduling while maintaining competitive positioning. This proactive, data-driven approach aligns with the need for adaptive strategies in an increasingly dynamic and globalised junior tennis ecosystem [17].

### Methodological limitations

Although the present study provides a detailed account of long-term performance dynamics in junior female tennis, several methodological constraints must be acknowledged. First, both Total Ranking Points (TRP) and Points per Event (PPE) represent system-generated metrics whose values depend not only on competitive quality but also on structural features of the ITF ecosystem, including participation volume, geographic access, and strategic scheduling. As such, percentile thresholds derived from TRP and PPE should be interpreted as approximations of systemic competitiveness rather than intrinsic measures of athlete ability. TRP in particular is sensitive to changes in the calendar structure, point tables, or the geographic distribution of events, which may inflate or suppress scores independently of underlying performance capacity. Similar concerns have been highlighted in comparative sport systems, where performance benchmarks have risen historically but now approach structural or physiological ceilings [13, 24, 26].

Second, while PPE provides an efficiency-oriented perspective on point accumulation, combining singles and doubles entries introduces a structural bias, as players may rely disproportionately on one competition format. This aggregation may obscure meaningful heterogeneity in developmental pathways, especially given that singles and doubles demand distinct tactical, physical, and partnership-related skill sets. Treating all WTTJ tiers as analytically equivalent introduces further simplification, as selective participation in high-value events may elevate ranking outcomes independently of genuine improvements in competitive quality.

Third, the logistic regression models used to identify percentile cut-offs exhibit quasi-deterministic behaviour due to the strong mathematical coupling between ranking points and percentile membership. The unusually high R² values observed in TRP-based models therefore reflect structural determinism rather than predictive strength. Consistent with the forecasting and performance-modelling literature [27], these models should be interpreted primarily as inferential tools for estimating thresholds rather than as classifiers capable of capturing novel performance determinants.

Finally, although the Bayesian Prophet model is well suited for non-linear, non-stationary time series, its predictive capacity remains sensitive to structural perturbations. The ITF’s 2018 ranking reform, which introduced substantial changes to point allocation and the weighting of high-tier events, and the COVID-19 disruptions illustrate how institutional and exogenous shocks can affect model stability. Prophet extrapolates trends under the assumption of piecewise-linear or logistic structure and thus captures the momentum of the ranking system itself rather than the true developmental trajectory of athletes. Forecasts should therefore be viewed as probabilistic projections bounded by systemic constraints, rather than as definitive estimates of future performance [13, 24, 26].

### Practical implications and applied recommendations

The present findings yield several actionable insights for federations, coaches, and athletes. Reverse-engineering of percentile thresholds demonstrates that, in the 2024 season, a typical P_90_ performance profile could be achieved with approximately 14 international tournaments per year combined with an average PPE of around 100 points per event. Analogously, P_75_ and P50 thresholds were attainable with approximately 16 tournaments and PPE values of about 75 and 55 points per event, respectively. While these benchmarks derive from a single season and should not be interpreted deterministically, they offer concrete reference points for designing age-appropriate international schedules and for calibrating federation support related to travel, competition exposure, and developmental planning.

For coaches, PPE provides a complementary indicator to cumulative TRP, allowing the evaluation of per-event efficiency and supporting more targeted load management strategies. Emphasising quality of competitive performance rather than high-volume scheduling may reduce the risk of burnout and injury while promoting sustainable long-term development. For athletes and parents, percentile-based targets offer transparent markers of progress and can help set realistic expectations regarding the competitive demands of the ITF junior system.

### Directions for future research

Future investigations should pursue longitudinal tracking from junior to senior levels to assess whether percentile attainment—particularly P_90_—predicts progression into WTA rankings or professional success. Such validation against independent performance indicators, including win–loss ratios, match-level statistics, and strength of opposition, would strengthen the interpretability of percentile thresholds and forecasting models [27]. Further, differentiating singles and doubles trajectories may reveal role-specific developmental patterns that are obscured in aggregated PPE measures. Incorporating surface-specific and tier-specific effects could clarify how tournament category (e.g., J100 vs. J500) moderates efficiency, access, and competitive progression.

Given the documented geographic inequities in access to high-value events, future studies should also examine cost–access relationships and the financial barriers that may constrain participation for athletes from resource-limited regions. Finally, hybrid forecasting frameworks that integrate Prophet with neural architectures or include exogenous covariates—such as maturation markers, technical–tactical profiles, or psycho-social characteristics—may provide more holistic and practically relevant models for understanding talent development in junior female tennis [8–10].

## Conclusion

This study aimed to evaluate long-term performance dynamics in junior female tennis by analysing cumulative achievement (TRP), efficiency per event (PPE), age distributions, and geographic access across twenty seasons of ITF data. The results showed that TRP-based percentile thresholds have risen non-linearly with clear accelerations, while PPE-based thresholds displayed greater variability and weaker structural trends. Elite entry age (P_90_) remained stable at 16 years, whereas broader tiers showed gradual ageing, and geographic access to P90 remained persistently concentrated within a small group of nations. A key limitation is that both TRP and PPE reflect systemic features of the ITF ranking architecture and are therefore sensitive to structural reforms and participation inequities. From a policy perspective, TRP should inform planning of cumulative competitive load, while PPE should guide efficiency-based performance management and event scheduling. Providing players and federations with clear, reverse-engineered benchmarks may help optimise developmental pathways and mitigate access-related disparities in international junior tennis.

## Supplementary Information

Supplementary Material 1.

Supplementary Material 2.

Supplementary Material 3.

## Data Availability

The datasets analysed during the current study are available in the ITF World Tennis Tour Juniors online repository (https://www.itftennis.com/), and aggregated datasets used for analysis are openly available in Figshare at DOI:10.6084/m9.figshare.30256252. Supplementary S1–S4Tables (additional calculations for extended information), together with the S1_file containing the raw dataset, are also provided in the Figshare repository.

## Abbreviations

CI: Credible Interval
ESS: Effective Sample Size
ITF: International Tennis Federation
J10, J30, J60, J100, J200, J300, J500: Tournament categories within the ITF World Tennis Tour Juniors
M: Mean
Med: Median
MCMC: Markov Chain Monte Carlo
NA: Not Applicable
P_90_, P_75_, P_50_: Percentile thresholds (90th, 75th, 50th )
PPE: Points per Event
SD: Standard Deviation
TRP: Total Ranking Points
WTA: Women’s Tennis Association
WTTJ: World Tennis Tour Juniors
