# Supplementary material for: Performance thresholds, efficiency, and access equity in junior girls’ tennis: longitudinal analysis and bayesian forecasting of ITF rankings (2004–2029)
Source: BMC Sports Sci Med Rehabil. 2026 Jan 23;18:90. doi: 10.1186/s13102-026-01542-x (PMC12910927; doi:10.1186/s13102-026-01542-x)
Supplement: Supplementary file 1 — Supplementary Material 1. [file 13102_2026_1542_MOESM1_ESM.pdf]

Performance Thresholds, Efficiency, and Access Equity in Junior Girls' Tennis: Longitudinal Analysis and Bayesian Forecasting of ITF Rankings (2004–2029)

Supplementary Material (File 1, Table 1-4)

Supplementary Table 1.

Binary logistic regression results and empirically derived cut-off thresholds for Total Ranking Points (TRP) across performance percentiles (P_{90}, P_{75}, P_{50}), 2004–2024.

Year	P_i	β_0 (intercept)	β_1 (TRP coefficient)	*p*	Nagelkerke R^2	Cut-off TRP
2004	P90	-17.40	0.03	<.001	.817	621.50
2004	P75	-3981.58	8.28	<.001	.999	480.63
2004	P50	-1282.52	3.38	<.001	.990	380.01
2005	P90	-25.82	0.04	<.001	.928	679.53
2005	P75	-2099.88	3.86	<.001	.999	544.43
2005	P50	-2339.22	6.24	<.001	.999	374.99
2006	P90	-22.15	0.04	<.001	.848	615.25
2006	P75	-1059.69	1.88	<.001	.999	564.26
2006	P50	-2043.24	5.23	<.001	.999	390.68
2007	P90	-24.86	0.04	<.001	.869	654.29
2007	P75	-4937.10	8.25	<.001	.999	598.15
2007	P50	-8485.70	22.37	<.001	.999	379.38
2008	P90	-238.48	0.40	<.001	.999	590.29
2008	P75	-11954.65	23.02	<.001	.999	519.38
2008	P50	-1838.46	4.48	<.001	.999	410.00
2009	P90	-18.13	0.03	<.001	.803	625.21
2009	P75	-3823.45	6.27	<.001	.999	609.99
2009	P50	-5312.63	12.39	<.001	.999	428.78
2010	P90	-11.85	0.02	<.001	.803	696.76
2010	P75	-1700.26	3.05	<.001	.999	558.01
2010	P50	-1606.15	3.92	<.001	.999	409.31
2011	P90	-18.33	0.03	<.001	.868	654.68
2011	P75	-280.85	5.23	<.001	.999	535.64
2011	P50	-4779.55	11.99	<.001	.999	398.76
2012	P90	-95.36	0.15	<.001	.948	631.49
2012	P75	-2455.85	4.43	<.001	.999	554.99
2012	P50	-9091.45	22.62	<.001	.999	401.87
2013	P90	-31.82	0.05	<.001	.946	677.00
2013	P75	-5631.36	9.56	<.001	.999	589.05
2013	P50	-2385.41	5.11	<.001	.999	466.90
2014	P90	-61.21	0.09	<.001	.951	703.54
2014	P75	-7312.16	11.96	<.001	.999	611.23
2014	P50	-19359.12	42.93	<.001	.999	45.94
2015	P90	-313.10	0.47	<.001	.999	671.90
2015	P75	-3128.99	4.83	<.001	.999	647.82
2015	P50	-2374.63	5.73	<.001	.999	414.71
2016	P90	-23.90	0.04	<.001	.910	664.00
2016	P75	-2126.55	3.57	<.001	.999	595.34
2016	P50	-1057.89	2.33	<.001	.999	453.64
2017	P90	-59.99	0.10	<.001	.922	605.99
2017	P75	-5731.29	9.75	<.001	.999	587.82
2017	P50	-4425.21	9.79	<.001	.999	452.20
2018	P90	-11.11	0.01	<.001	.857	1389.25
2018	P75	-1186.35	1.25	<.001	.999	946.05

2018	P50	-4813.21	7.65	<.001	.999	629.34
2019	P90	-14.60	0.02	<.001	.811	973.13
2019	P75	-488.28	0.55	<.001	.999	882.97
2019	P50	-1514.47	2.38	<.001	.999	636.87

For each calendar year between 2004 and 2024, separate binary logistic regression models were fitted to estimate the probability that a given value of Total Ranking Points (TRP) would correspond to inclusion within each performance percentile category (P_{90}, P_{75}, and P_{50}).

In all models, the dependent variable was coded dichotomously, where a value of 1 indicated that a player reached the specified percentile threshold in the respective year, and a value of 0 indicated that the player did not reach the threshold. TRP served as the continuous independent variable.

The coefficients β_0 (intercept) and β_1 (slope) describe the log-odds relationship between TRP and the probability of achieving the respective percentile category. Model significance was evaluated using Wald tests, and overall model fit was assessed using Nagelkerke's R^2.

The reported cut-off TRP values represent empirically derived threshold points corresponding to a predicted probability of classification within the given percentile category, calculated from the estimated regression coefficients. These thresholds indicate the minimum number of ranking points required to achieve P_{90}, P_{75}, or P_{50} status in a given year.

All models were statistically significant (*p* < 0.001) and demonstrated substantial explanatory power (Nagelkerke's R^2 ranging from .634 to .999).

Supplementary Table 2.

Binary logistic regression results and empirically derived cut-off thresholds for Points per Event (PPE) across performance percentiles (P_{90}, P_{75}, P_{50}), 2004–2024.

Year	P_i	β_0 (Intercept)	β_1 (PPE coefficient)	*p*	Nagelkerke R^2	Cut-off PPE
2004	P90	-17.40	0.03	<.001	.817	621.50
2004	P75	-3981.58	8.28	<.001	.999	480.63
2004	P50	-1282.52	3.38	<.001	.990	380.01
2005	P90	-25.82	0.04	<.001	.928	679.53
2005	P75	-2099.88	3.86	<.001	.999	544.43
2005	P50	-2339.22	6.24	<.001	.999	374.99
2006	P90	-22.15	0.04	<.001	.848	615.25
2006	P75	-1059.69	1.88	<.001	.999	564.26
2006	P50	-2043.24	5.23	<.001	.999	390.68
2007	P90	-24.86	0.04	<.001	.869	654.29
2007	P75	-4937.10	8.25	<.001	.999	598.15
2007	P50	-8485.70	22.37	<.001	.999	379.38
2008	P90	-238.48	0.40	<.001	.999	590.29
2008	P75	-11954.65	23.02	<.001	.999	519.38
2008	P50	-1838.46	4.48	<.001	.999	410.00
2009	P90	-18.13	0.03	<.001	.803	625.21
2009	P75	-3823.45	6.27	<.001	.999	609.99
2009	P50	-5312.63	12.39	<.001	.999	428.78
2010	P90	-11.85	0.02	<.001	.803	696.76
2010	P75	-1700.26	3.05	<.001	.999	558.01
2010	P50	-1606.15	3.92	<.001	.999	409.31
2011	P90	-18.33	0.03	<.001	.868	654.68
2011	P75	-280.85	5.23	<.001	.999	535.64
2011	P50	-4779.55	11.99	<.001	.999	398.76
2012	P90	-95.36	0.15	<.001	.948	631.49
2012	P75	-2455.85	4.43	<.001	.999	554.99
2012	P50	-9091.45	22.62	<.001	.999	401.87
2013	P90	-31.82	0.05	<.001	.946	677.00
2013	P75	-5631.36	9.56	<.001	.999	589.05
2013	P50	-2385.41	5.11	<.001	.999	466.90
2014	P90	-61.21	0.09	<.001	.951	703.54
2014	P75	-7312.16	11.96	<.001	.999	611.23
2014	P50	-19359.12	42.93	<.001	.999	45.94
2015	P90	-313.10	0.47	<.001	.999	671.90
2015	P75	-3128.99	4.83	<.001	.999	647.82
2015	P50	-2374.63	5.73	<.001	.999	414.71
2016	P90	-23.90	0.04	<.001	.910	664.00
2016	P75	-2126.55	3.57	<.001	.999	595.34
2016	P50	-1057.89	2.33	<.001	.999	453.64
2017	P90	-59.99	0.10	<.001	.922	605.99
2017	P75	-5731.29	9.75	<.001	.999	587.82
2017	P50	-4425.21	9.79	<.001	.999	452.20
2018	P90	-11.11	0.01	<.001	.857	1389.25
2018	P75	-1186.35	1.25	<.001	.999	946.05
2018	P50	-4813.21	7.65	<.001	.999	629.34
2019	P90	-14.60	0.02	<.001	.811	973.13
2019	P75	-488.28	0.55	<.001	.999	882.97
2019	P50	-1514.47	2.38	<.001	.999	636.87

For each calendar year between 2004 and 2024, separate binary logistic regression models were estimated to examine the probability that a given value of Points per Event (PPE) would correspond to inclusion within each performance percentile category (P_{90}, P_{75}, and P_{50}).

In all models, the dependent variable was coded dichotomously, where a value of 1 indicated that a player reached the specified percentile threshold in the respective year, and a value of 0 indicated that the player did not reach the threshold. PPE, defined as the ratio of total ranking points accumulated to the number of tournaments played within the given season, served as the continuous independent variable.

The regression coefficients β_0 (intercept) and β_1 (slope) describe the log-odds relationship between PPE and the likelihood of achieving the respective percentile category. Statistical significance was evaluated using Wald tests, while overall model fit was assessed using Nagelkerke's R^2.

The reported cut-off PPE values represent empirically derived efficiency thresholds corresponding to a predicted probability of classification within the given percentile category, calculated from the estimated regression coefficients. These thresholds indicate the minimum level of points efficiency per tournament required to achieve P_{90}, P_{75}, or P_{50} status in a given year.

All estimated models were statistically significant ($p < 0.001$) and demonstrated strong explanatory power, with Nagelkerke's R^2 values ranging from moderate to near-perfect fit across the analysed years.

Supplementary Table 3.

Descriptive statistics of empirically derived cut-off thresholds for Total Ranking Points (TRP) and Points per Event (PPE) across performance percentiles (P_{90}, P_{75}, P_{50}), 2004–2024.

	TRP			PPE		
	**P_{90}**	**P_{75}**	**P_{50}**	**P_{90}**	**P_{75}**	**P_{50}**
n	19	19	19	19	19	19
**Mean**	813.67	697.77	507.67	54.89	32.54	20.42
**SD**	296.98	229.52	171.20	19.19	9.25	5.71
**Min**	590.29	480.64	374.99	31.07	23.32	15.12
**Max**	1416.50	1205.11	871.42	105.33	52.63	32.43
**Q_1**	628.35	556.50	400.31	43.43	26.49	16.66
**Med**	671.90	595.34	428.78	47.00	29.88	17.74
**Q_3**	838.34	765.39	548.12	60.70	35.25	22.50

This table summarises descriptive statistics of the empirically derived annual cut-off thresholds for both Total Ranking Points (TRP) and Points per Event (PPE) across the three analysed performance percentile categories (P_{90}, P_{75}, and P_{50}) over the period 2004–2024.

Cut-off thresholds represent the minimum values of TRP or PPE required to achieve classification within the respective percentile category in a given year, as estimated using binary logistic regression models (see Supplementary Tables S1 and S2). Descriptive statistics were computed across annual cut-off values to characterise the central tendency, variability, and distributional shape of performance thresholds over time.

Skewness and kurtosis provide information on the asymmetry and tail behaviour of the cut-off distributions. Positive skewness indicates a concentration of lower threshold values with occasional years requiring substantially higher performance standards, while elevated kurtosis reflects the presence of extreme threshold values in specific seasons.

Years affected by substantial disruptions to the international junior competition calendar due to the COVID-19 pandemic (2020 and 2021) may contribute disproportionately to distributional irregularities and should therefore be interpreted with caution.

Supplementary Table 4.

Age characteristics of players within performance percentiles (P_{90}, P_{75}, P_{50}) in junior female tennis, 2004–2024 (excluding COVID-19 affected seasons).

Year	P_i	*n*	Median	Q_1	Q_3	IQR	min	max
2004	P90	10	16.0	15.25	17	1.75	15	18
2004	P75	24	16.0	15.00	17	2.00	14	18
2004	P50	48	16.0	15.75	17	1.25	14	18
2005	P90	9	16.0	16.00	16	0.00	15	17
2005	P75	24	16.0	15.75	17	1.25	15	18
2005	P50	48	16.0	16.00	17	1.00	14	18
2006	P90	10	16.0	16.00	16	0.00	15	17
2006	P75	23	16.0	16.00	17	1.00	15	18
2006	P50	46	16.0	16.00	17	1.00	14	18
2007	P90	9	16.0	16.00	17	1.00	16	17
2007	P75	21	16.0	16.00	17	1.00	14	18
2007	P50	42	17.0	16.00	17	1.00	14	18
2008	P90	9	17.0	16.00	17	1.00	14	18
2008	P75	22	17.0	16.00	17	1.00	14	18
2008	P50	43	17.0	16.00	17	1.00	14	18
2009	P90	9	16.0	16.00	17	1.00	15	18
2009	P75	21	16.0	16.00	17	1.00	14	18
2009	P50	41	16.0	16.00	17	1.00	14	18
2010	P90	9	17.0	16.00	17	1.00	15	18
2010	P75	22	16.5	16.00	17	1.00	15	18
2010	P50	44	16.0	16.00	17	1.00	15	18
2011	P90	9	17.0	16.00	17	1.00	15	18
2011	P75	21	17.0	16.00	17	1.00	15	18
2011	P50	42	17.0	16.00	17	1.00	15	18
2012	P90	9	16.0	15.00	17	2.00	15	18
2012	P75	22	17.0	16.00	17	1.00	15	18
2012	P50	44	17.0	16.00	17	1.00	15	18
2013	P90	9	16.0	16.00	17	1.00	15	18
2013	P75	23	17.0	16.00	17	1.00	15	18
2013	P50	45	17.0	16.00	17	1.00	15	18
2014	P90	10	17.0	16.25	17	0.75	15	17
2014	P75	25	17.0	16.00	17	1.00	15	18
2014	P50	50	17.0	16.00	17	1.00	14	18
2015	P90	10	16.5	16.00	17	1.00	15	17
2015	P75	23	17.0	16.00	17	1.00	14	18
2015	P50	46	17.0	16.00	17	1.00	14	18
2016	P90	9	16.0	16.00	17	1.00	15	17
2016	P75	22	16.0	16.00	17	1.00	15	18
2016	P50	44	16.0	16.00	17	1.00	15	18
2017	P90	9	16.0	16.00	17	1.00	15	18
2017	P75	23	17.0	16.00	17	1.00	15	18
2017	P50	45	17.0	16.00	17	1.00	15	18
2018	P90	9	17.0	16.00	17	1.00	14	18
2018	P75	23	17.0	16.00	17	1.00	14	18
2018	P50	45	17.0	16.00	17	1.00	14	18
2019	P90	12	17.0	15.75	17	1.25	14	18
2019	P75	28	17.0	16.00	17	1.00	14	18
2019	P50	56	17.0	16.00	17	1.00	14	18
2022	P90	10	17.0	16.00	17	1.00	15	17
2022	P75	25	17.0	16.00	17	1.00	15	18
2022	P50	50	17.0	16.25	17	0.75	15	18
2023	P90	12	16.0	15.00	17	2.00	14	18
2023	P75	29	16.0	15.00	17	2.00	14	18
2023	P50	58	17.0	16.00	17	1.00	14	18
2024	P90	11	16.0	16.00	17	1.00	15	17
2024	P75	27	17.0	16.00	18	2.00	15	18
2024	P50	54	17.0	16.00	17	1.00	15	18

This table presents annual descriptive age characteristics of junior female players classified within the P_{90}, P_{75}, and P_{50} performance percentiles, based on year-end ITF junior rankings between 2004 and 2024. The analysis summarises the age distribution of players who achieved each percentile category in a given season.

For each year and percentile category, the table reports the sample size (n), median age, first and third quartiles (Q_1, Q_3), interquartile range (IQR), and observed minimum and maximum ages. Age was calculated as the player's chronological age at the end of the respective competitive year.

The percentile categories (P_{90}, P_{75}, and P_{50}) indicate whether a player achieved inclusion within the top 10%, 25%, or 50% of the year-end performance distribution, respectively, based on Total Ranking Points. Players could contribute to different percentile categories across years, depending on their annual ranking outcomes.

Data from the 2020 and 2021 seasons were excluded from this table due to substantial disruptions to the international junior competition calendar caused by the COVID-19 pandemic, which resulted in atypical participation patterns and incomplete age distributions.
