# Supplementary material for: Performance thresholds, efficiency, and access equity in junior girls’ tennis: longitudinal analysis and bayesian forecasting of ITF rankings (2004–2029)
Source: BMC Sports Sci Med Rehabil. 2026 Jan 23;18:90. doi: 10.1186/s13102-026-01542-x (PMC12910927; doi:10.1186/s13102-026-01542-x)
Supplement: Supplementary file 3 — Supplementary Material 3. [file 13102_2026_1542_MOESM3_ESM.pdf]

Supplementary File 1.

Raw year-end ITF junior ranking data used for longitudinal analyses (2

no.	P10	P25	P50	ITF Ranking	Player name	Year of Birth
1	1	1	1	1	Joaofonseca	2006
2	1	1	1	2	Rodrigopacheco mendez	2005
3	1	1	1	3	Joelschwaerzler	2006
4	1	1	1	4	Yaroslavdemin	2005
5	1	1	1	5	Cooperwilliams	2005
6	1	1	1	6	Henrysearle	2006
7	1	1	1	7	Federicocina	2007
8	1	1	1	8	Juan Carlosprado angelo	2005
9	1	1	1	9	Darwinblanch	2007
10	1	1	1	10	Iliyanradulov	2005
11	1	1	1	11	Yizhou	2005
12	1	1	1	12	Brankodjuric	2005
13	1	1	1	13	Nicolaibudkov kjaer	2006
14	0	1	1	14	Arthurgea	2005
15	0	1	1	15	Tomaszberkieta	2006
16	0	1	1	16	Federicobondioli	2005
17	0	1	1	17	Reisakamoto	2006
18	0	1	1	18	Oliverojakaar	2005
19	0	1	1	19	Alexanderfrusina	2005
20	0	1	1	20	Sebastianeriksson	2005
21	0	1	1	21	Kaylanbigun	2006
22	0	1	1	22	Danilpanarin	2005
23	0	1	1	23	Alejandromelero kretzer	2005
24	0	1	1	24	Alexanderrazeghi	2006
25	0	1	1	25	Adhithyaganesan	2005
26	0	1	1	26	Maximmrva	2007
27	0	1	1	27	Gabrielevulpitta	2005
28	0	1	1	28	Oliverbonding	2007
29	0	1	1	29	Jakubfilip	2005
30	0	1	1	30	Jangjunkim	2006
31	0	1	1	31	Petrbrunclik	2006
32	0	0	1	32	Charliecamus	2006
33	0	0	1	33	Lassepoertner	2005
34	0	0	1	34	Maxdahlin	2005
35	0	0	1	35	Redabennani	2007
36	0	0	1	36	Royhorovitz	2006
37	0	0	1	37	Tianhuizhang	2006
38	0	0	1	38	Pavlemarinkov	2006
39	0	0	1	39	Janhrazdil	2005
40	0	0	1	40	Pedrorodrigues	2006
41	0	0	1	41	Adrianodzhenev	2005
42	0	0	1	42	Thijsboogaard	2008
43	0	0	1	43	Haydenjones	2006
44	0	0	1	44	Lorenzosciahbasi	2005
45	0	0	1	45	Carlo Albertocaniato	2005
46	0	0	1	46	Abelforger	2005

47	0	0	1	47	Viktorfrydrych	2006
48	0	0	1	48	Timofeiderepasko	2007
49	0	0	1	49	Patrickschoen	2005
50	0	0	1	50	Yuvannandal	2005
51	0	0	1	51	Fabiode michele	2005
52	0	0	1	52	Charlierobertson	2006
53	0	0	1	53	Daniilsarksian	2006
54	0	0	1	54	Theopapamalamis	2006
55	0	0	1	55	Thomasfaurel	2006
56	0	0	1	56	Atakankarahan	2006
57	0	0	1	57	Hoyoungroh	2006
58	0	0	1	58	Paulbarbier gazeu	2005
59	0	0	1	59	Emiliendemanet	2005
60	0	0	1	60	Kevinedengren	2005
61	0	0	1	61	Meesrottgering	2007
62	0	0	1	62	Maxwellexsted	2007
63	0	0	0	63	Matickriznik	2005
64	0	0	0	64	Donghyunhwang	2007
65	0	0	0	65	Maxschoenhaus	2007
66	0	0	0	66	Cooperwoestendick	2006
67	0	0	0	67	Gustavo Ribeirode almeida	2006
68	0	0	0	68	Aryanshah	2005
69	0	0	0	69	Albertsaar	2005
70	0	0	0	70	Vitkalina	2005
71	0	0	0	71	Markusmolder	2005
72	0	0	0	72	Ignacioparisca	2005
73	0	0	0	73	Luis Josenakamine	2006
74	0	0	0	74	Hayatomatsuoka	2005
75	0	0	0	75	Seungminpark	2005
76	0	0	0	76	Nicolasoliveira	2006
77	0	0	0	77	Keeganrice	2006
78	0	0	0	78	Vukradjenovic	2005
79	0	0	0	79	Filipporomano	2005
80	0	0	0	80	Maximozeitune	2006
81	0	0	0	81	Fernandocavallo	2005
82	0	0	0	82	Jaggerleach	2007
83	0	0	0	83	Ilyas Miladfahim	2005
84	0	0	0	84	Tiagopires	2005
85	0	0	0	85	Alejosanchez quilez	2005
86	0	0	0	86	Segundogoity zapico	2005
87	0	0	0	87	Volodymyriakubenko	2005
88	0	0	0	88	Kylekang	2005
89	0	0	0	89	Rogerpascual ferra	2006
90	0	0	0	90	Ruslantiukaev	2005
91	0	0	0	91	Hughwinter	2006
92	0	0	0	92	Mihai Alexandrucoman	2005
93	0	0	0	93	Henriquecostanzi de brito	2005
94	0	0	0	94	Amirmilushev	2005
95	0	0	0	95	Davidfix	2005
96	0	0	0	96	Migueltobon	2006

97	0	0	0	97	Cyrusmahjoob	2005
98	0	0	0	98	Yutatomida	2006
99	0	0	0	99	Luis Felipeferraz sandoval carva	2005
100	0	0	0	100	Duncanchan	2005
101	0	0	0	101	Matthias Uwekask	2005
102	0	0	0	102	Andriizimnokh	2005
103	0	0	0	103	Zoranludoski	2005
104	0	0	0	104	Rafaelsegado esteve	2006
105	0	0	0	105	Luccaguercio	2006
106	0	0	0	106	Adityagovila	2006
107	0	0	0	107	Albertpedrico kravtsov	2005
108	0	0	0	108	Thanaphatboosarawongse	2006
109	0	0	0	109	Stilesbrockett	2006
110	0	0	0	110	Manasdhamne	2007
111	0	0	0	111	Calvinbaierl	2007
112	0	0	0	112	Victor Hugoremondy pagotto	2005
113	0	0	0	113	Carel Aubrielngounoue	2008
114	0	0	0	114	Alejandroarcila	2007
115	0	0	0	115	Iannismiletich	2005
116	0	0	0	116	Gonzalozeitune	2005
117	0	0	0	117	Meecahbigun	2006
118	0	0	0	118	Jeremyzhang	2005
119	0	0	0	119	Satorunakajima	2005
120	0	0	0	120	Reiyahattori	2005
121	0	0	0	121	Cornelius Alexandershalmi	2005
122	0	0	0	122	Felipevirgili berini	2005
123	0	0	0	123	Takamasamishiro	2005
124	0	0	0	124	Shumuto	2006
125	1	1	1	1	Gilles Arnaudbailly	2005
126	1	1	1	2	Martinlandaluce	2006
127	1	1	1	3	Gerardcampana lee	2004
128	1	1	1	4	Danielvallejo	2004
129	1	1	1	5	Nisheshbasavareddy	2005
130	1	1	1	6	Rodrigopacheco mendez	2005
131	1	1	1	7	Kilianfeldbausch	2005
132	1	1	1	8	Yaroslavdemin	2005
133	1	1	1	9	Gonzalobueno	2004
134	1	1	1	10	Lautaromidon	2004
135	1	1	1	11	Gabrieldebru	2005
136	1	1	1	12	Dinoprizmic	2005
137	0	1	1	13	Alexanderblockx	2005
138	0	1	1	14	Edasbutvilas	2004
139	0	1	1	15	Jakubmensik	2005
140	0	1	1	16	Cooperwilliams	2005
141	0	1	1	17	Jakubnicod	2004
142	0	1	1	18	Colemanwong	2004
143	0	1	1	19	Arthurgea	2005
144	0	1	1	20	Borartnak	2004
145	0	1	1	21	Paulinchauspe	2004
146	0	1	1	22	Hynekbarton	2004

147	0	1	1	23	Martynpawelski	2004
148	0	1	1	24	Juan Carlosprado angelo	2005
149	0	1	1	25	Michaelzheng	2004
150	0	1	1	26	Antoineghibaudo	2005
151	0	1	1	27	Devinbadenhorst	2004
152	0	1	1	28	Ignaciobuse	2004
153	0	1	1	29	Ozanbaris	2004
154	0	0	1	30	Reisakamoto	2006
155	0	0	1	31	Hayatomatsuoka	2005
156	0	0	1	32	Peternad	2004
157	0	0	1	33	Paulbarbier gazeu	2005
158	0	0	1	34	Alexmichelsen	2004
159	0	0	1	35	Danilpanarin	2005
160	0	0	1	36	Peter Benjaminprivara	2004
161	0	0	1	37	Pedrorodenas	2004
162	0	0	1	38	Leanidboika	2004
163	0	0	1	39	Joaofonseca	2006
164	0	0	1	40	Jackloutit	2004
165	0	0	1	41	Haydenjones	2006
166	0	0	1	42	Jadenweekes	2004
167	0	0	1	43	Milipoljicak	2004
168	0	0	1	44	Kevinedengren	2005
169	0	0	1	45	Nicholasgodsick	2004
170	0	0	1	46	Dylandietrich	2004
171	0	0	1	47	Learnertien	2005
172	0	0	1	48	Mikabrunold	2004
173	0	0	1	49	Alejandromelero kretzer	2005
174	0	0	1	50	Martin Antoniovergara del puer	2004
175	0	0	1	51	Aidankim	2004
176	0	0	1	52	Tiagopires	2005
177	0	0	1	53	Jeremyjin	2004
178	0	0	1	54	Alexanderfrusina	2005
179	0	0	1	55	Jonahbraswell	2004
180	0	0	1	56	Vojtechpetr	2005
181	0	0	1	57	Charliecamus	2006
182	0	0	0	58	Yannikrahman	2004
183	0	0	0	59	Luis Carlosalvarez valdes	2004
184	0	0	0	60	Janhrazdil	2005
185	0	0	0	61	Aleksapisaric	2005
186	0	0	0	62	Kaylanbigun	2006
187	0	0	0	63	Brankodjuric	2005
188	0	0	0	64	Jonathanirwanto	2004
189	0	0	0	65	Lennon Roarkjones	2005
190	0	0	0	66	Federicocina	2007
191	0	0	0	67	Hoyoungroh	2006
192	0	0	0	68	Tanapattnirundorn	2004
193	0	0	0	69	Atakankarahan	2006
194	0	0	0	70	Patrickschoen	2005
195	0	0	0	71	Edwardwinter	2004
196	0	0	0	72	Davidfix	2005

197	0	0	0	73	Sebastianeriksson	2005
198	0	0	0	74	Volodymyriakubenko	2005
199	0	0	0	75	Constantinoskoshis	2004
200	0	0	0	76	Kentamiyoshi	2004
201	0	0	0	77	Connor Henryvan schalkwyk	2004
202	0	0	0	78	Alejandrobancalari	2004
203	0	0	0	79	Yannikkelm	2005
204	0	0	0	80	Alexanderrazeghi	2006
205	0	0	0	81	Yanakimilev	2004
206	0	0	0	82	Jelanisarr	2004
207	0	0	0	83	Phoenixweir	2005
208	0	0	0	84	Asahiharazaki	2004
209	0	0	0	85	Adhithyaganesan	2005
210	0	0	0	86	Lorenzoferri	2004
211	0	0	0	87	Duncanchan	2005
212	0	0	0	88	Reiyahattori	2005
213	0	0	0	89	Togantokac	2004
214	0	0	0	90	Tiagopereira	2004
215	0	0	0	91	Patrickbrady	2004
216	0	0	0	92	Alessiobasile	2004
217	0	0	0	93	Quangduong	2006
218	0	0	0	94	Lucapow	2005
219	0	0	0	95	Thanaphatboosarawongse	2006
220	0	0	0	96	Boryszgola	2004
221	0	0	0	97	Maxbatyutenko	2005
222	0	0	0	98	Julesleroux	2005
223	0	0	0	99	Olafpieczkowski	2004
224	0	0	0	100	Danielverbeek	2004
225	0	0	0	101	Ivansodan	2004
226	0	0	0	102	Liamgavrielides	2004
227	0	0	0	103	Giammarcogandolfi	2004
228	0	0	0	104	Benjamintorrealba	2005
229	0	0	0	105	Kurtmiller	2004
230	0	0	0	106	Niccolociavarella	2004
231	0	0	0	107	Juan Manuella serna	2004
232	0	0	0	108	Evanwen	2004
233	0	0	0	109	Viliusgaubas	2004
234	0	0	0	110	Takamasamishiro	2005
235	0	0	0	111	Kalinivanovski	2004
236	0	0	0	112	Matthewrankin	2004
237	0	0	0	113	Danieleminighini	2004
238	0	0	0	114	Woobinshin	2004
239	1	1	1	1	Junchengshang	2005
240	1	1	1	2	Danielrincon	2003
241	1	1	1	3	Samirbanerjee	2003
242	1	1	1	4	Brunokuzuhara	2004
243	1	1	1	5	Viacheslavbielinskyi	2003
244	1	1	1	6	Victorlilov	2004
245	1	1	1	7	Milipoljicak	2004
246	1	1	1	8	Danielvallejo	2004

247	1	1	1	9	Saschagueymard wayenburg	2003
248	1	1	1	10	Jeromekym	2003
249	1	1	1	11	Jakubmensik	2005
250	1	1	1	12	Lucavan assche	2004
251	1	1	1	13	Marklajal	2003
252	1	1	1	14	Gonzalobueno	2004
253	1	1	1	15	Leoborg	2003
254	1	1	1	16	Ignaciobuse	2004
255	1	1	1	17	Alejandromanzanera pertusa	2003
256	1	1	1	18	Gabrieldebru	2005
257	1	1	1	19	Edasbutvilas	2004
258	1	1	1	20	Jackpinnington jones	2003
259	1	1	1	21	Arthurfils	2004
260	1	1	1	22	Philipsekulic	2003
261	1	1	1	23	Olafpieczkowski	2004
262	1	1	1	24	Ethanquinn	2004
263	1	1	1	25	Pedroboscardin dias	2003
264	1	1	1	26	Giovannimpetshi perricard	2003
265	1	1	1	27	Danielmerida	2004
266	1	1	1	28	Rodrigopacheco mendez	2005
267	1	1	1	29	Kalinivanovski	2004
268	1	1	1	30	Jackanthrop	2003
269	1	1	1	31	Kilianfeldbausch	2005
270	1	1	1	32	Pyotrnesterov	2003
271	1	1	1	33	Colemanwong	2004
272	1	1	1	34	Viliusgaubas	2004
273	1	1	1	35	Ozanbaris	2004
274	1	1	1	36	Peter Benjaminprivara	2004
275	1	1	1	37	Dinoprizmic	2005
276	1	1	1	38	Alexanderbernard	2003
277	1	1	1	39	Alvaroguillen meza	2003
278	1	1	1	40	Orelkimhi	2003
279	1	1	1	41	Borartnak	2004
280	1	1	1	42	Lautaromidon	2004
281	1	1	1	43	Seancuenin	2004
282	1	1	1	44	Aleksanderorlikowski	2003
283	1	1	1	45	Makskasnikowski	2003
284	1	1	1	46	Erikarutiunian	2004
285	1	1	1	47	Sebastiandominko	2003
286	1	1	1	48	Daniilostapenkov	2003
287	1	1	1	49	Ryancolby	2003
288	1	1	1	50	Gerardcampana lee	2004
289	1	1	1	51	Konstantinzhzhenov	2004
290	1	1	1	52	Maxwestphal	2003
291	1	1	1	53	Abedallahshelbayh	2003
292	1	1	1	54	Juan Carlosprado angelo	2005
293	1	1	1	55	Max Hansrehberg	2003
294	1	1	1	56	Philipflorig	2003
295	1	1	1	57	Liamgavrielides	2004
296	1	1	1	58	Neoniedner	2003

297	1	1	1	59	Iliasimakin	2003
298	1	1	1	60	Alexanderblockx	2005
299	1	1	1	61	Adamjurajda	2003
300	1	1	1	62	Matthew Williamdonald	2004
301	1	1	1	63	Joao Victorcouto loureiro	2003
302	1	1	1	64	Vojtechpetr	2005
303	1	1	1	65	Arturkukasian	2004
304	1	1	1	66	Miguelperez pena	2003
305	1	1	1	67	Connor Henryvan schalkwyk	2004
306	1	1	1	68	Juan Manuella serna	2004
307	1	1	1	69	Daliblanch	2003
308	1	1	1	70	Jakubnicod	2004
309	1	1	1	71	Filippieczonka	2004
310	1	1	1	72	Maximshin	2003
311	1	1	1	73	Walidahouda	2003
312	1	1	1	74	Lukamikrut	2004
313	1	1	1	75	Yassinedlimi	2003
314	1	1	1	76	Stefanpopovic	2003
315	1	1	1	77	Pierre Yvesbailly	2003
316	1	1	1	78	Boryszgola	2004
317	1	1	1	79	Igorkudriashov	2003
318	1	1	1	80	Mehdibenchakroun	2003
319	1	1	1	81	Jadenweekes	2004
320	1	1	1	82	Woobinshin	2004
321	1	1	1	83	Luimaxted	2003
322	1	1	1	84	Aidankim	2004
323	1	1	1	85	Danieleminighini	2004
324	1	1	1	86	Martinkatz	2003
325	1	1	1	87	Germansamofalov	2003
326	1	1	1	88	Gilles Arnaudbailly	2005
327	1	1	1	89	Mikabrunold	2004
328	1	1	1	90	Hynekbarton	2004
329	1	1	1	91	Pedrorodenas	2004
330	1	1	1	92	Johan Alexanderrodriguez rodri	2003
331	1	1	1	93	Maxbatyutenko	2005
332	1	1	1	94	Maximzhukov	2005
333	1	1	1	95	Cooperwilliams	2005
334	1	1	1	96	Constantinoskoshis	2004
335	1	1	1	97	Nishantdabas	2003
336	1	1	1	98	Ronellouck	2004
337	1	1	1	99	Miguelgomes	2003
338	1	1	1	100	Robinbertrand	2003
339	1	1	1	101	Arthurgea	2005
340	1	1	1	102	Mikhailgorokhov	2004
341	1	1	1	103	Marcelkamrowski	2003
342	1	1	1	104	Nikolaynedelchev	2003
343	1	1	1	105	Luis Carlosalvarez valdes	2004
344	1	1	1	106	Yannikrahman	2004
345	1	1	1	107	Maximilianhomberg	2003
346	1	1	1	108	Azumavisaya	2003

347	1	1	1	109	Togantokac	2004
348	1	1	1	110	Tanapattnirundorn	2004
349	1	1	1	111	Ezequielmonferrer	2003
350	1	1	1	112	Paulinchauspe	2004
351	1	1	1	113	Michaelminasyan	2003
352	1	1	1	114	Markotopo	2003
353	1	1	1	115	Sebastiansec	2003
354	1	1	1	116	Henriquerocha	2004
355	1	1	1	117	Isacstroemberg	2003
356	1	1	1	118	Gianlucaballotta	2004
357	1	1	1	119	Yaroslavdemin	2005
358	1	1	1	120	Lennon Roarkjones	2005
359	1	1	1	121	Alessiobasile	2004
360	1	1	1	122	Nicholasgodsick	2004
361	1	1	1	123	Markoandrejic	2003
362	1	1	1	124	Bradenshick	2003
363	1	1	1	125	Matthewrankin	2004
364	1	1	1	126	Leanidboika	2004
365	1	1	1	127	Charleliecosnet	2003
366	1	1	1	128	Dinkodinev	2004
367	1	1	1	129	Dylandietrich	2004
368	1	1	1	130	Christopherli	2003
369	1	1	1	131	Peterbuldorini	2004
370	1	1	1	132	Jamesmccabe	2003
371	1	1	1	133	Jaydentempleman	2003
372	1	1	1	134	Derrickchen	2003
373	1	1	1	135	Jay Dylanhara friend	2003
374	1	1	1	136	Jankobierski	2004
375	1	1	1	137	Martin Antoniovergara del puer	2004
376	1	1	1	138	Francisco Tomasgeschwind	2003
377	1	1	1	139	Michaelzheng	2004
378	1	1	1	140	Noahmerre	2004
379	1	1	1	141	Chiragduhan	2004
380	1	1	1	142	Amandahiya	2004
381	1	1	1	143	Kurtmiller	2004
382	1	1	1	144	Benjaminkittay	2003
383	1	1	1	145	Andrejnedic	2004
384	1	1	1	146	Antoineghibaudo	2005
385	1	1	1	147	Patrickschoen	2005
386	1	1	1	148	Aleksapisaric	2005
387	1	1	1	149	Luckoenig	2003
388	1	1	1	150	Antonarzhankin	2003
389	1	1	1	151	Stepanmruzek	2004
390	1	1	1	152	Brankodjuric	2005
391	1	1	1	153	Peternad	2004
392	1	1	1	154	Jaimefaria	2003
393	1	1	1	155	Gabrielepiraino	2003
394	1	1	1	156	Alexmichelsen	2004
395	1	1	1	157	Timothyphung	2003
396	1	1	1	158	Louisbowden	2004

397	1	1	1	159	Sebastiangorzny	2004
398	1	1	1	160	Artempodorozhnyi	2003
399	1	1	1	161	Amirkhamzanasridinov	2005
400	1	1	1	162	Luca- Julianhotze	2003
401	1	1	1	163	Mehdisadaoui	2003
402	1	1	1	164	Martynpawelski	2004
403	1	1	1	165	Enriquepena	2004
404	1	1	1	166	Giulioperego	2003
405	1	1	1	167	Janhrazdil	2005
406	1	1	1	168	Tiagopires	2005
407	1	1	1	169	Thibaultverdier	2003
408	1	1	1	170	Stylianoschristodoulou	2004
409	1	1	1	171	Jeremyjin	2004
410	1	1	1	172	Patrickbrady	2004
411	1	1	1	173	Mikalaibankou	2005
412	1	1	1	174	Danilpanarin	2005
413	1	1	1	175	Niccolociavarella	2004
414	1	1	1	176	Ivansodan	2004
415	1	1	1	177	nicolasnino mendoza	2004
416	1	1	1	178	Coltonsmith	2003
417	1	1	1	179	Lorenzoferri	2004
418	1	1	1	180	Markostakusic	2003
419	1	1	1	181	Maciejkos	2004
420	1	1	1	182	Ellisshort	2003
421	1	1	1	183	Danielsiniakov	2003
422	1	1	1	184	Prestonstearns	2004
423	1	1	1	185	Marianotammaro	2004
424	1	1	1	186	Santiagogiamichelle	2003
425	1	1	1	187	Giammarcogandolfi	2004
426	1	1	1	188	Josephphillips	2005
427	1	1	1	189	Danielverbeek	2004
428	1	1	1	190	Lorenzoesquici	2003
429	1	1	1	191	Alexanderrazeghi	2006
430	1	1	1	192	Matickriznik	2005
431	1	1	1	193	Aidanmayo	2003
432	1	1	1	194	Jonahbraswell	2004
433	1	1	1	195	Illyamaksymchuk	2004
434	1	1	1	196	Baransoyler	2004
435	1	1	1	197	Shaybar menahem	2003
436	1	1	1	198	Filipapltauer	2004
437	1	1	1	199	Dragos Nicolaecazacu	2004
438	1	1	1	200	Valentinbasel	2004
439	1	1	1	201	Devinbadenhorst	2004
440	1	1	1	202	Armandosotelo	2004
441	1	1	1	203	Seandaryabeigi	2004
442	1	1	1	204	Ainiussabaliauskas	2003
443	1	1	1	205	Alexanderfrusina	2005
444	1	1	1	206	Yanakimilev	2004
445	1	1	1	207	Mihai Alexandrucoman	2005
446	1	1	1	208	Ardaazkara	2003

447	1	1	1	209	Theodorosmitsakos	2004
448	1	1	1	210	Melchiordelloye	2003
449	1	1	1	211	Eric Davidverdes	2004
450	1	1	1	212	Williamjansen	2004
451	1	1	1	213	Camilocano gomez	2004
452	1	1	1	214	Kentamiyoshi	2004
453	1	1	1	215	Nicolas Iankotzen	2003
454	1	1	1	216	Sebastianeriksson	2005
455	1	1	1	217	Edwardwinter	2004
456	1	1	1	218	Nikolazekic	2004
457	1	1	1	219	Vitkalina	2005
458	1	1	1	220	Germanmingaleev	2004
459	1	1	1	221	Zoranludoski	2005
460	1	1	1	222	Marc Othmanktiri	2003
461	1	1	1	223	Amirmilushev	2005
462	1	1	1	224	Iliyanradulov	2005
463	1	1	1	225	Denimyadav	2004
464	1	1	1	226	Quangduong	2006
465	1	1	1	227	Yuvannandal	2005
466	1	1	1	228	Jakubprachar	2003
467	1	1	1	229	Gianlucacitadini	2004
468	1	1	1	230	Sam Kyddbaumgartner	2004
469	1	1	1	231	Tiagopereira	2004
470	1	1	1	232	Wissamabderrahman	2003
471	1	1	1	233	Adrianodzhenev	2005
472	1	1	1	234	Alejandrobancalari	2004
473	1	1	1	235	Alejandrolicea	2004
474	1	1	1	236	Lukasvelik	2005
475	1	1	1	237	Mikalipp	2003
476	1	1	1	238	Filipporomano	2005
477	1	1	1	239	Nathancox	2003
478	1	1	1	240	Emilianoaguilera guerrero	2003
479	1	1	1	241	Sahebsodhi	2004
480	1	1	1	242	Zaneford	2004
481	1	1	1	243	Martinlandaluce	2006
482	1	1	1	244	Joao Eduardoschiessl	2004
483	1	1	1	245	Rushilkhosla	2006
484	1	1	1	246	Vladimirbazilevskiy	2005
485	1	1	1	247	Matthiasujvary	2004
486	1	1	1	248	Paulbarbier gazeu	2005
487	1	1	1	249	Oliverojakaar	2005
488	1	1	1	250	Matejdodig	2005
489	1	1	1	251	Lucapow	2005
490	1	1	1	252	Berkbugarikj	2003
491	1	1	1	253	Alexanderorlov	2004
492	1	1	1	254	Sameral tori	2003
493	1	1	1	255	Grigoryshebekin	2005
494	1	1	1	256	Eskanderguryev	2004
495	1	1	1	257	Pablotrochu	2003
496	1	1	1	258	Wilson Oswaleleigbinovia	2003

497	1	1	1	259	Davidfix	2005
498	1	1	1	260	Venedikttsirikidze	2004
499	1	1	1	261	Jackloutit	2004
500	1	1	1	262	Ekanshkumar	2003
501	1	1	1	263	Petarteodorovic	2003
502	1	1	1	264	Evanlee	2003
503	1	1	1	265	Hans Christianbustiman gonzale	2003
504	1	1	1	266	Michalkrajci	2005
505	1	1	1	267	Learnertien	2005
506	1	1	1	268	Romaingales	2004
507	1	1	1	269	Yehongyu	2004
508	1	1	1	270	Idrisshaddouch	2004
509	1	1	1	271	Birtanduran	2004
510	1	1	1	272	Daniilfelker	2004
511	1	1	1	273	Taymal azmeh	2004
512	1	1	1	274	Abelforger	2005
513	1	1	1	275	Tokyranaivo	2003
514	1	1	1	276	Nicolaskobelt	2004
515	1	1	1	277	Phoenixweir	2005
516	1	1	1	278	Jangjunkim	2006
517	1	1	1	279	Lukajovanovic	2004
518	1	1	1	280	Dominiquerolland	2004
519	1	1	1	281	Hayatomatsuoka	2005
520	1	1	1	282	Benjamintorrealba	2005
521	1	1	1	283	Loanlestir	2005
522	1	1	1	284	Ivanburnashov	2004
523	1	1	1	285	Attila Dominikboros	2003
524	1	1	1	286	Federicocina	2007
525	1	1	1	287	Victortosetto	2004
526	1	1	1	288	Kokoroisomura	2003
527	1	1	1	289	Paulwerren	2004
528	1	1	1	290	Karimibrahim	2003
529	1	1	1	291	Zacharyviiala	2005
530	1	1	1	292	Amansharma	2003
531	1	1	1	293	Nicolasvillalon valdes	2004
532	1	1	1	294	Andreasscott	2003
533	1	1	1	295	Johnkim	2004
534	1	1	1	296	Vlad Cristianbreazu	2003
535	1	1	1	297	Mateodel pino	2003
536	0	1	1	298	Simonmyslivec	2004
537	0	1	1	299	Haydenjones	2006
538	0	1	1	300	Photosphotiades	2003
539	0	1	1	301	Andrincasanova	2004
540	0	1	1	302	Fredericvogeli	2003
541	0	1	1	303	Meecahbigun	2006
542	0	1	1	304	Patrykstokowski	2003
543	0	1	1	305	Yamatosueoka	2003
544	0	1	1	306	Alejandromelero kretzer	2005
545	0	1	1	307	Williamnolan	2003
546	0	1	1	308	Georgiosnouchakis	2003

547	0	1	1	309	Hadyel kordy	2005
548	0	1	1	310	Aayush Pbhat	2005
549	0	1	1	311	Gabrielmatuszewski	2004
550	0	1	1	312	Jelanisarr	2004
551	0	1	1	313	Cesarbouchelaghem	2004
552	0	1	1	314	Paulo Guillermoramos martin	2003
553	0	1	1	315	Freddymurray	2003
554	0	1	1	316	Mansdahlberg	2003
555	0	1	1	317	Siwanatauytayakul	2004
556	0	1	1	318	Juan Diegorojas mora	2003
557	0	1	1	319	Danielbagnolini	2003
558	0	1	1	320	Hamadmedjedovic	2003
559	0	1	1	321	Andriizimnokh	2005
560	0	1	1	322	Viktormarkov	2005
561	0	1	1	323	Semenaginskiy	2005
562	0	1	1	324	Lucasbrown	2004
563	0	1	1	325	Oliverolsson	2004
564	0	1	1	326	Manfredigraziani	2004
565	0	1	1	327	Asahiharazaki	2004
566	0	1	1	328	Mariomartinez serrano	2004
567	0	1	1	329	Federicobondioli	2005
568	0	1	1	330	Jonaszdziopak	2003
569	0	1	1	331	Franciscosinopoli	2004
570	0	1	1	332	Jagger Zhen Chihong patzwald	2003
571	0	1	1	333	Kylekang	2005
572	0	1	1	334	Connorkruger	2003
573	0	1	1	335	Santiagoarredondo delgado	2003
574	0	1	1	336	Veljkokrstic	2003
575	0	1	1	337	Luis Felipemiguel	2004
576	0	1	1	338	Dmitrydolzhenkov	2004
577	0	1	1	339	Mitsuki Wei Kangleong	2004
578	0	1	1	340	Ahmedzaki	2005
579	0	1	1	341	Olivertarvet	2003
580	0	1	1	342	Diogomorais	2004
581	0	1	1	343	Joelschwaerzler	2006
582	0	1	1	344	Lucioratti	2004
583	0	1	1	345	Massimogiunta	2004
584	0	1	1	346	Abdurahmansaidmuhamedov	2003
585	0	1	1	347	Hod'abalo isakpadio	2005
586	0	1	1	348	Elmermoeller	2003
587	0	1	1	349	Pijusvaitiekunas	2003
588	0	1	1	350	Maximusjones	2004
589	0	1	1	351	Nathan Anthonybarki	2004
590	0	1	1	352	Nikitasidorov	2003
591	0	1	1	353	Jacksonarmistead	2004
592	0	1	1	354	Islamorynbasar	2004
593	0	1	1	355	Raffaelemariani	2004
594	0	1	1	356	Kyvanrietkerk	2005
595	0	1	1	357	Petrbrunclik	2006
596	0	1	1	358	Hamzael amine	2004

597	0	1	1	359	Miguelavendano cadena	2005
598	0	1	1	360	Maxencerivet	2003
599	0	1	1	361	Freddieridout	2004
600	0	1	1	362	Corbancrowther	2003
601	0	1	1	363	Matijasamardzic	2004
602	0	1	1	364	Kaerihernandez mariona	2003
603	0	1	1	365	Husseinel tawil	2003
604	0	1	1	366	Hugocoquelin	2005
605	0	1	1	367	Charliecamus	2006
606	0	1	1	368	Cosmerolland de ravel	2004
607	0	1	1	369	Josueguzman	2005
608	0	1	1	370	Yousseflabbene	2004
609	0	1	1	371	Saveliyivanov	2004
610	0	1	1	372	Theopapamalamis	2006
611	0	1	1	373	Toprakavcibasi	2004
612	0	1	1	374	Maximilianwuelfing	2004
613	0	1	1	375	Ilyamukhin	2005
614	0	1	1	376	Manvydasbalciunas	2006
615	0	1	1	377	Sebastiencauhape	2006
616	0	1	1	378	Norbertmarosik	2004
617	0	1	1	379	Giorgiotabacco	2003
618	0	1	1	380	Billyblaydes	2003
619	0	1	1	381	Henryren	2004
620	0	1	1	382	Anronel	2004
621	0	1	1	383	Giacomonosei	2005
622	0	1	1	384	Woohyukchang	2005
623	0	1	1	385	Stefansimeunovic	2004
624	0	1	1	386	Jakubjupa	2004
625	0	1	1	387	Alfredalmasi	2003
626	0	1	1	388	Vukradjenovic	2005
627	0	1	1	389	Natand'hulst	2003
628	0	1	1	390	Lucboulier santelices	2003
629	0	1	1	391	Ruslantiukaev	2005
630	0	1	1	392	Rohanmurali	2004
631	0	1	1	393	Nikitafilin	2006
632	0	1	1	394	Arthuro'sullivan	2004
633	0	1	1	395	Joelpierleoni	2003
634	0	1	1	396	Yerassylyerdilda	2005
635	0	1	1	397	Santiagonavarro	2003
636	0	1	1	398	Aljazjeran	2003
637	0	1	1	399	Yannikkelm	2005
638	0	1	1	400	Edson Alejandrosanchez elizond	2003
639	0	1	1	401	Jakubfilip	2005
640	0	1	1	402	Jonathanirwanto	2004
641	0	1	1	403	Duncanchan	2005
642	0	1	1	404	Aryanshah	2005
643	0	1	1	405	Takumiwatanabe	2005
644	0	1	1	406	Danielkhazime	2005
645	0	1	1	407	Mohamed Aminoueslati	2004
646	0	1	1	408	Stefanregalia	2005

647	0	1	1	409	Yunseokjang	2003
648	0	1	1	410	Kacperszymkowiak	2004
649	0	1	1	411	Anarghaganguly	2004
650	0	1	1	412	Lucasphanthala	2004
651	0	1	1	413	Markusmolder	2005
652	0	1	1	414	Ethan Yi Hangleung	2004
653	0	1	1	415	Juho Ericbiggs	2004
654	0	1	1	416	Maigao	2003
655	0	1	1	417	Timothycarlsson seger	2003
656	0	1	1	418	Theoherrmann	2005
657	0	1	1	419	Aristarkhsafonov	2003
658	0	1	1	420	Adamkresina	2004
659	0	1	1	421	Lucanardi	2003
660	0	1	1	422	Adityanandal	2003
661	0	1	1	423	Lucapreda	2006
662	0	1	1	424	Jiayangdong	2004
663	0	1	1	425	Yutatomida	2006
664	0	1	1	426	Kentanakamura	2004
665	0	1	1	427	Kajquirijns	2004
666	0	1	1	428	Aliyazdani	2003
667	0	1	1	429	Christian Andre Shengliew	2004
668	0	1	1	430	Ange-Kevinkoua	2004
669	0	1	1	431	Lucalemaitre vilchis	2005
670	0	1	1	432	Nikolaibarsukov	2006
671	0	1	1	433	Reiyahattori	2005
672	0	1	1	434	Henrysearle	2006
673	0	1	1	435	Pavlosparson	2003
674	0	1	1	436	Youssefsadek	2003
675	0	1	1	437	Guillaumejacob	2004
676	0	1	1	438	Alexanderedley	2003
677	0	1	1	439	Joaofonseca	2006
678	0	1	1	440	Benjamin Ignaciotorres fernand	2003
679	0	1	1	441	Freekvan donselaar	2003
680	0	1	1	442	Williamjucha	2004
681	0	1	1	443	Itaybity wekselman	2004
682	0	1	1	444	Otsomartikainen	2006
683	0	1	1	445	Royhorovitz	2006
684	0	1	1	446	Daniilsarksian	2006
685	0	1	1	447	Dmitriiburtsev	2006
686	0	1	1	448	Ivankremenchutskyi	2004
687	0	1	1	449	Fadibidan	2006
688	0	1	1	450	Rogerpascual ferra	2006
689	0	1	1	451	Kasrarahmani	2006
690	0	1	1	452	Poonthongkomopisut	2005
691	0	1	1	453	Eriktkalenko	2005
692	0	1	1	454	Suphawatsaeoui	2005
693	0	1	1	455	Fatihsari	2003
694	0	1	1	456	Gustavsdambins	2005
695	0	1	1	457	Nejcskorjanc	2004
696	0	1	1	458	Dimitrispaliouras	2003

697	0	1	1	459	Heziheng	2004
698	0	1	1	460	Adamantiosstavrakas	2003
699	0	1	1	461	Julianlozano	2003
700	0	1	1	462	Leomatthysen	2006
701	0	1	1	463	Etiennemarty	2004
702	0	1	1	464	Estebandavila gasser	2004
703	0	1	1	465	Matheus Amorimde lima	2005
704	0	1	1	466	Matei Victorpalcau	2003
705	0	1	1	467	Joshuanuyts	2004
706	0	1	1	468	Williamzulch	2003
707	0	1	1	469	Omarsuarez berrezueta	2004
708	0	1	1	470	Matthewrutter	2003
709	0	1	1	471	Joantorres espinosa	2004
710	0	1	1	472	Volodymyriakubenko	2005
711	0	1	1	473	Aryanlakshmanan	2005
712	0	1	1	474	Woodamsong	2003
713	0	1	1	475	Tiagoboschmans	2005
714	0	1	1	476	Maxstenzer	2006
715	0	1	1	477	Majpremzl	2004
716	0	1	1	478	Yuataka	2003
717	0	1	1	479	Adriancontreras	2003
718	0	1	1	480	Mateuszkulakowski	2003
719	0	1	1	481	Min Jaekim	2004
720	0	1	1	482	Eyalshyovitz	2004
721	0	1	1	483	Jamessim	2003
722	0	1	1	484	Tuncayduran	2004
723	0	1	1	485	Davidschwarc	2005
724	0	1	1	486	Saidakbarov	2004
725	0	1	1	487	Henrylamchinniah	2005
726	0	1	1	488	Pavelpetrov	2004
727	0	1	1	489	Artiomdorofeev	2004
728	0	1	1	490	Inigodiaz	2003
729	0	1	1	491	Nicholassteiglehner	2004
730	0	1	1	492	Theodoretruwit	2003
731	0	1	1	493	Braydenmichna	2004
732	0	1	1	494	William Yovannyribero duarte	2003
733	0	1	1	495	Juan Jose Germanvargas gutier	2003
734	0	1	1	496	Taentawan Taddeomajoli magg	2003
735	0	1	1	497	Eliezergonzalez	2004
736	0	1	1	498	Danialrakhmatullayev	2006
737	0	1	1	499	Segundogoity zapico	2005
738	0	1	1	500	Emiliendemanet	2005
739	0	1	1	501	Alin Mihaifatu	2004
740	0	1	1	502	Gabrielevulpitta	2005
741	0	1	1	503	Ismoilhayriddinov	2004
742	0	1	1	504	Alecbraund	2004
743	0	1	1	505	Georg Eduardisraelan	2005
744	0	1	1	506	Matthewjohnstone	2004
745	0	1	1	507	Kolganovkirill	2004
746	0	1	1	508	Piotrczaczkowski	2005

747	0	1	1	509	Hoyoungroh	2006
748	0	1	1	510	Kylehughes	2005
749	0	1	1	511	Wiljenkins	2004
750	0	1	1	512	Alfonsridstrom	2005
751	0	1	1	513	Idosamimyan	2005
752	0	1	1	514	gian carlosrodriguez	2004
753	0	1	1	515	Jamesvan herzeele	2003
754	0	1	1	516	Julesleroux	2005
755	0	1	1	517	Davidsspaks	2004
756	0	1	1	518	Shingomasuda	2005
757	0	1	1	519	Gregorhausberger	2003
758	0	1	1	520	Pietsteveker	2004
759	0	1	1	521	Iegoropanasenko	2004
760	0	1	1	522	Christopheclement	2004
761	0	1	1	523	Danielstens	2005
762	0	1	1	524	Tamerlanazizov	2004
763	0	1	1	525	Adhithyaganesan	2005
764	0	1	1	526	Sebastiancarpio	2003
765	0	1	1	527	Jacopobilardo	2004
766	0	1	1	528	RODRIGObraunstein	2003
767	0	1	1	529	Lukasjanousek	2004
768	0	1	1	530	Galemersic potocnik	2003
769	0	1	1	530	Alenmujakic	2003
770	0	1	1	532	Aleksanderchayka	2004
771	0	1	1	533	Lunobrul	2004
772	0	1	1	534	Jamesedley	2003
773	0	1	1	535	Valdemar Frederikpape	2003
774	0	1	1	536	Tomas Danielvalencia navech	2003
775	0	1	1	537	Patrikjurina	2005
776	0	1	1	538	Sasharozin	2005
777	0	1	1	539	Tsihonfedorov	2005
778	0	1	1	540	Pierofernandez moreno	2004
779	0	1	1	541	Piotrsiekanowicz	2004
780	0	1	1	542	Carlholder	2006
781	0	1	1	543	Isaacrondon gonzalez	2003
782	0	1	1	544	Fayzullomirzaganiyev	2003
783	0	1	1	545	Alexanderdespoja	2005
784	0	1	1	546	Antonkastsenich	2004
785	0	1	1	547	Vladimirpopov	2003
786	0	1	1	548	Muniananth mani	2004
787	0	1	1	549	Rafaelymer	2005
788	0	1	1	550	Maceosimpson	2003
789	0	1	1	551	Samscherer	2004
790	0	1	1	552	Kaylanbigun	2006
791	0	1	1	553	Romanvenger	2006
792	0	1	1	554	Adidagan	2003
793	0	1	1	555	Emmanueljebutu	2004
794	0	1	1	556	Alexandru Raresgiurea	2004
795	0	1	1	557	Florentlimani	2004
796	0	1	1	558	Hugosoares	2005

797	0	1	1	559	Viktorfrydrych	2006
798	0	1	1	560	Alexkhamrotau	2004
799	0	1	1	561	Henriquepetiz	2003
800	0	1	1	562	Valentinfaure	2005
801	0	1	1	563	RONINlotlikar	2004
802	0	1	1	564	Edouardaubert	2005
803	0	1	1	565	Matiasrojas	2004
804	0	1	1	566	Fabionestola	2005
805	0	1	1	567	Seydinaandre	2003
806	0	1	1	568	Jackloge	2004
807	0	1	1	569	Calin Teodorstirbu	2006
808	0	1	1	570	Zachstephens	2006
809	0	1	1	571	Radovanmichalik	2003
810	0	1	1	572	Naoyahonda	2006
811	0	1	1	573	Tiagosilva	2004
812	0	1	1	574	Roykeegan	2005
813	0	1	1	575	Romeohadzimehmedovic	2005
814	0	1	1	576	Yutooki	2005
815	0	1	1	577	Denzelseetso	2005
816	0	1	1	578	Garvbahl	2004
817	0	1	1	579	Filipposastreinidis	2003
818	0	1	1	580	Redabennani	2007
819	0	1	1	581	Juliandesousa	2003
820	0	1	1	582	Mihnea Petruzaharia	2003
821	0	1	1	583	Julianalonso	2005
822	0	1	1	584	Fnunidunjianzan	2004
823	0	1	1	585	Emiliobravo	2004
824	0	1	1	586	Denyszamaraiev	2003
825	0	1	1	587	Eduardomorais	2003
826	0	1	1	588	Adamduda	2004
827	0	1	1	589	Albertsaar	2005
828	0	1	1	590	Takamasamishiro	2005
829	0	1	1	591	Alexanderaney	2004
830	0	1	1	592	Aditsinha	2003
831	0	1	1	593	Mostafamohamed	2005
832	0	1	1	594	Ikersevilla	2005
833	0	1	1	595	Tomaszberkieta	2006
834	0	1	1	596	Kanebonsach ganley	2004
835	0	1	1	597	Jacobolar	2007
836	0	1	1	598	Tommyczaplinski	2004
837	0	1	1	599	Lukevuletic	2003
838	0	1	1	600	Aleksandarmitric	2005
839	0	1	1	601	FELIPEpinzon moreno	2004
840	0	1	1	602	Juan Pablopatino velasco	2004
841	0	1	1	603	Egorkhotchenkov	2003
842	0	1	1	604	Gokberksaritas	2003
843	0	1	1	605	Miguel Angelalonso	2003
844	0	1	1	606	Lylezaloumis	2004
845	0	1	1	607	Carlosguerrero alvarez	2004
846	0	1	1	608	Myko Yohann Himadkouame	2003

847	0	1	1	609	Dominicducariu	2005
848	0	1	1	610	Rodrigo Nicolasmontes de oca [illegible]	2003
849	0	1	1	611	Adriandibildox	2003
850	0	1	1	612	Pedroliborio	2003
851	0	1	1	613	Freddyblaydes	2005
852	0	1	1	614	Lambrospantzaris	2003
853	0	1	1	615	Adrienberrut	2005
854	0	1	1	616	Carlos Joaquinramos membrive	2003
855	0	1	1	617	Reiniskupfers	2004
856	0	1	1	618	Princegandonou	2003
857	0	1	1	619	Martenmurrand	2003
858	0	1	1	620	Alejandrojuan mano	2005
859	0	1	1	621	Desmondayaaba	2004
860	0	1	1	622	Maximest-hilaire	2003
861	0	1	1	623	Indreksoome	2003
862	0	1	1	624	Andream'chich	2005
863	0	1	1	625	Mihailivanov	2004
864	0	1	1	626	Landonardila	2004
865	0	1	1	627	Rajatathamcharoensatit	2005
866	0	1	1	628	Oldrichvejsicky	2004
867	0	1	1	629	Liamdrover-mattinen	2005
868	0	1	1	630	Tomasquesada perez	2004
869	0	1	1	631	Atakankarahan	2006
870	0	1	1	632	Luisfrancisco	2003
871	0	1	1	633	Patrikmeszaros	2005
872	0	1	1	634	Jose Alessandrohernandez frias	2005
873	0	1	1	635	Piotrkusiewicz	2003
874	0	1	1	636	Goranzgola	2005
875	0	1	1	637	Maxdahlin	2005
876	0	1	1	638	Oliverhague	2005
877	0	1	1	639	Maximedubouch	2003
878	0	1	1	640	Michaelbassem sobhy	2004
879	0	1	1	641	Williamnorton	2005
880	0	1	1	642	Chan Heehan	2005
881	0	1	1	643	Mattiassouthcombe	2003
882	0	1	1	644	Bon Loukarstens	2005
883	0	1	1	645	Petarjovanovic	2003
884	0	1	1	646	Iliavedmed	2005
885	0	1	1	647	Andreameduri	2005
886	0	1	1	648	Michael Dylanjimenez	2006
887	0	1	1	649	Nikhilniranjan	2003
888	0	1	1	650	Kevinedengren	2005
889	0	1	1	651	Arturkhairutdinov	2004
890	0	1	1	652	Cooperwhite	2003
891	0	1	1	653	Matei Cristianonofrei	2005
892	0	1	1	654	Alvaropedraza garcia	2004
893	0	1	1	655	Kirtanpatel	2004
894	0	1	1	656	Gurtrakhtenberg	2006
895	0	1	1	657	Thanaphatboosarawongse	2006
896	0	1	1	658	Gillianosmont	2003

897	0	1	1	659	Ethan Denzelsibanda	2004
898	0	1	1	660	Lucianoalcocer	2004
899	0	1	1	661	Danielmilardovic	2004
900	0	1	1	662	Timofeiderepasko	2007
901	0	1	1	663	Stefanskobelev	2004
902	0	1	1	664	Markomesarovic	2004
903	0	1	1	665	Jungheeyou	2005
904	0	1	1	666	Matteocovato	2005
905	0	1	1	667	Danirkaldybekov	2006
906	0	1	1	668	Rohanloubser	2004
907	0	1	1	669	Lorenzocarboni	2006
908	0	1	1	670	Marselznuderl	2003
909	0	1	1	671	Samuelreeve	2004
910	0	1	1	672	Santiagopadilla cote	2005
911	0	1	1	673	Luisfernandez	2003
912	0	1	1	674	Jansebesta	2003
913	0	1	1	675	Grantlothringer	2003
914	0	1	1	676	Pavlemarinkov	2006
915	0	1	1	677	Nikhilmukherjee	2005
916	0	1	1	678	Igormandou berranger	2005
917	0	1	1	679	Antoinenivault	2003
918	0	1	1	680	Romankazantsev	2004
919	0	1	1	681	Evgeniosvasilakis	2005
920	0	1	1	682	Diegoortiz gamonal	2003
921	0	1	1	683	Maanavjain	2003
922	0	1	1	684	Satorunakajima	2005
923	0	1	1	685	Eliasshokry	2004
924	0	1	1	686	Yahorslizevich	2003
925	0	1	1	687	Nikitafilatov	2003
926	0	1	1	688	Alexanderwatanabe eriksson	2003
927	0	1	1	689	Victor Milarealves	2004
928	0	1	1	690	Hugohashimoto	2003
929	0	1	1	691	Ericpadgham	2003
930	0	1	1	692	Maximdontsov	2004
931	0	1	1	693	Amorjasika	2004
932	0	1	1	694	Victor Barrosfernandes	2003
933	0	1	1	695	Manasdhamne	2007
934	0	1	1	696	Stepanbaum	2003
935	0	1	1	697	Miguellarrea sicco	2005
936	0	1	1	698	Ignacioparisca	2005
937	0	1	1	699	Iliyasmaratuly	2006
938	0	1	1	700	Parispouatcha	2006
939	0	1	1	701	Dominykasstonkus	2004
940	0	1	1	702	Jeidensaidizand	2004
941	0	1	1	703	Juan Davidduque ramirez	2003
942	0	1	1	704	Hlibaharyshev	2004
943	0	1	1	705	Danielhammond	2004
944	0	1	1	706	Patrickjozwicki	2005
945	0	1	1	707	Sasamarkovic	2005
946	0	1	1	708	Paubosch munoz	2005

947	0	1	1	709	Lorantgyori	2006
948	0	1	1	710	Rashednawaf	2005
949	0	1	1	711	Kael Shalinshah	2004
950	0	1	1	712	Patrikoplustil	2005
951	0	1	1	713	Oleggussakov	2004
952	0	1	1	714	Thomas Nicholasgadecki	2006
953	0	1	1	715	Lucalo nardo	2004
954	0	1	1	716	Vineethmutyala	2006
955	0	1	1	717	Henrikbladelius	2004
956	0	1	1	718	Goncalomarques	2006
957	0	1	1	719	Kosukemorita	2004
958	0	1	1	720	Aaronventura	2003
959	0	1	1	721	Tobiaswallin	2004
960	0	1	1	722	Zianvanderstappen	2004
961	0	1	1	723	Tillbrunner	2004
962	0	1	1	724	Kaseschinnerer	2006
963	0	1	1	725	Joshmanuel	2005
964	0	1	1	726	Kwok Shun Dassonchan	2005
965	0	1	1	727	Alexanderchernomaz	2004
966	0	1	1	728	Richardbiagiotti	2003
967	0	1	1	729	Benjaminscott	2004
968	0	1	1	730	Benweintraub	2003
969	0	1	1	731	Reiyakomagata	2003
970	0	1	1	732	Arslanbekaitkulov	2004
971	0	1	1	733	Jakubkroslak	2003
972	0	1	1	734	Emiliozielkowski	2004
973	0	1	1	735	Oscarpinto sansano	2003
974	0	1	1	736	Baltazarwiger nordas	2003
975	0	1	1	737	Marcelomarino hidalgo	2004
976	0	1	1	738	Diegoduran	2004
977	0	1	1	739	Jean-Christianmorandais	2003
978	0	1	1	740	Alekseimokrov	2005
979	0	1	1	741	Mikhaildubrouski	2003
980	0	1	1	742	Matthieuchambonniere	2004
981	0	0	1	743	Jandimitrijevic	2003
982	0	0	1	744	Diegojarry fillol	2003
983	0	0	1	745	Keremozlale	2005
984	0	0	1	746	Izanorgiles garcia retamero	2004
985	0	0	1	747	Radomirtomic	2004
986	0	0	1	748	Josephtownes	2004
987	0	0	1	749	Suleyman Denizgursoy	2004
988	0	0	1	750	Georgelazarov	2005
989	0	0	1	751	Grigoriigordeev	2003
990	0	0	1	752	Amitvales	2005
991	0	0	1	753	Maximecontet	2005
992	0	0	1	754	Min Seongkim	2004
993	0	0	1	755	Nahuel Nicolaseli	2005
994	0	0	1	756	Williamalsterback	2003
995	0	0	1	757	Rohanbelday	2006
996	0	0	1	758	Erdener	2004

997	0	0	1	759	Jacob Kailiangshen	2007
998	0	0	1	760	Felipelopez comper	2003
999	0	0	1	761	Dhruvhirpara	2003
1000	0	0	1	762	Shuannontricerri	2003
1001	0	0	1	763	Maximillianrelic	2003
1002	0	0	1	764	Arseniykhokhlov	2004
1003	0	0	1	765	Dakshprasad	2005
1004	0	0	1	766	Jose Emilianomontilla garces	2003
1005	0	0	1	767	Henriquecostanzi de brito	2005
1006	0	0	1	768	Constantinosdjakouris	2004
1007	0	0	1	769	Gonzalozeitune	2005
1008	0	0	1	770	Kiyan Santinokasemi	2003
1009	0	0	1	771	Danielcrowie	2004
1010	0	0	1	772	Enricobaldisserri	2004
1011	0	0	1	773	Markomaksimovic	2006
1012	0	0	1	774	Caseyhoole	2003
1013	0	0	1	775	Jonahgethin	2004
1014	0	0	1	776	Tsegaigebremeskel	2004
1015	0	0	1	777	Manuellazic	2005
1016	0	0	1	778	Lukanovakovic	2004
1017	0	0	1	779	Reisakamoto	2006
1018	0	0	1	780	Gustavo Ribeirode almeida	2006
1019	0	0	1	781	Samuelkyjaci	2004
1020	0	0	1	782	Chetannaamadike	2005
1021	0	0	1	783	Milesgroom	2003
1022	0	0	1	784	Rafaelbotran neutze	2006
1023	0	0	1	785	Andremegrabian	2005
1024	0	0	1	786	Johnchin	2003
1025	0	0	1	787	Ismailelshafei	2004
1026	0	0	1	788	Dylancharlap	2005
1027	0	0	1	789	Carlescordoba	2006
1028	0	0	1	790	Adityagovila	2006
1029	0	0	1	791	Thomasfaurel	2006
1030	0	0	1	792	Nunopinheiro	2004
1031	0	0	1	793	Nikmikovic	2004
1032	0	0	1	794	Soogiri	2005
1033	0	0	1	795	Agriyayadav	2005
1034	0	0	1	796	Aljazkirbis	2004
1035	0	0	1	797	Timhammes	2003
1036	0	0	1	798	Andreykozlov	2005
1037	0	0	1	799	Diegoherrera	2006
1038	0	0	1	800	Nicojaeger	2003
1039	0	0	1	801	Davisrolis	2005
1040	0	0	1	802	Francisco Javierborbolla	2003
1041	0	0	1	803	Sylgaxherri	2004
1042	0	0	1	804	Daksh Ashokagarwal	2004
1043	0	0	1	805	Iannisfernandes	2005
1044	0	0	1	806	Arturaiukhanov	2006
1045	0	0	1	807	Iustinbelea	2003
1046	0	0	1	808	Josephoyebog jr	2007

1047	0	0	1	809	Vascoleote prata	2003
1048	0	0	1	810	Olegkolninov	2005
1049	0	0	1	811	Jaysonmbogoro	2004
1050	0	0	1	812	nicolasrecoura	2004
1051	0	0	1	813	Yaronamorule	2004
1052	0	0	1	814	Aarav Samrathada	2007
1053	0	0	1	815	Oskargrzegorzewski	2005
1054	0	0	1	816	Francescocordova	2005
1055	0	0	1	817	Samyarelyasi	2004
1056	0	0	1	818	BUSHANhaobam	2006
1057	0	0	1	819	Amiralighavam	2006
1058	0	0	1	820	Ivanpenev	2003
1059	0	0	1	821	Wianroothman	2004
1060	0	0	1	822	Andrewdelgado	2005
1061	0	0	1	823	Masatoperera	2003
1062	0	0	1	824	Nicholasheng	2003
1063	0	0	1	825	Rafaelsegado esteve	2006
1064	0	0	1	826	Davidpetrovic	2004
1065	0	0	1	827	Taurasmickus	2004
1066	0	0	1	828	Franciscocastro	2007
1067	0	0	1	829	Darius Florinpop	2004
1068	0	0	1	830	Taewanjoo	2005
1069	0	0	1	831	Amirasylkozhaev	2006
1070	0	0	1	832	Andreicuceu	2003
1071	0	0	1	833	Gokalpayar	2003
1072	0	0	1	834	Jaishnavshinde	2005
1073	0	0	1	835	Geniinoue	2003
1074	0	0	1	836	Dragomirdraganov	2004
1075	0	0	1	837	Pedrorodrigues	2006
1076	0	0	1	838	Chotiwatnaen-udorn	2004
1077	0	0	1	839	Adampanaras	2003
1078	0	0	1	840	Nikolajovanovic	2004
1079	0	0	1	841	Luigibove	2004
1080	0	0	1	842	Caymus Hei Tungchoi	2004
1081	0	0	1	843	Evangeloskypriotis	2003
1082	0	0	1	844	Usmankushimo	2004
1083	0	0	1	845	Saba Georgenanobashvili	2004
1084	0	0	1	846	Hoanmouret	2005
1085	0	0	1	847	Nikolasfedasko	2005
1086	0	0	1	848	Krystofsvojgr	2006
1087	0	0	1	849	Derickominde	2003
1088	0	0	1	850	Victorkuznetsov	2005
1089	0	0	1	851	Tobiasleitner	2005
1090	0	0	1	852	Zinovarouhas-poole	2004
1091	0	0	1	853	Alexde gabriele	2004
1092	0	0	1	854	MONILlotlikar	2004
1093	0	0	1	855	Sooyoungyang	2004
1094	0	0	1	856	Aaronyu	2005
1095	0	0	1	857	Danielbaquero	2005
1096	0	0	1	858	Alejandroverdasco	2004

1097	0	0	1	859	Larswagenaar	2005
1098	0	0	1	860	Thomascardona herrera	2004
1099	0	0	1	861	Pablo Marceltorres almeida	2004
1100	0	0	1	862	Luis Felipeferraz sandoval carva	2005
1101	0	0	1	863	Thomasdrapeau	2004
1102	0	0	1	864	Tomaszlatohlavek	2004
1103	0	0	1	865	Mukhtarandu	2004
1104	0	0	1	866	Georgerussell	2004
1105	0	0	1	867	Kanikajayathilake	2004
1106	0	0	1	868	Artemcherepin	2004
1107	0	0	1	869	Eduardonunez del prado flores	2004
1108	0	0	1	870	Jamiediack	2006
1109	0	0	1	871	Philipprein	2004
1110	0	0	1	872	Yakupuzunel	2005
1111	0	0	1	873	Louiscloud	2003
1112	0	0	1	874	Blazejsopolinski	2003
1113	0	0	1	875	Jorgeruiz	2005
1114	0	0	1	876	Michaelemmanuel	2004
1115	0	0	1	877	Hayden Khoomenon	2005
1116	0	0	1	878	Sandroszic	2003
1117	0	0	1	879	Henrique Hamamotoushizima	2005
1118	0	0	1	880	Nikolajsgalkins	2003
1119	0	0	1	881	Alejosanchez quilez	2005
1120	0	0	1	882	Luis Josenakamine	2006
1121	0	0	1	883	Yegorbilyy	2005
1122	0	0	1	884	Alanheughebaert	2004
1123	0	0	1	885	Finnemslie-robson	2004
1124	0	0	1	886	Alejandrojacome jaramillo	2003
1125	0	0	1	887	Benedictbadza	2005
1126	0	0	1	888	Emiliovila	2003
1127	0	0	1	889	Nicolasphilibert	2005
1128	0	0	1	890	Eneskocu	2005
1129	0	0	1	891	Cesarmahecha	2006
1130	0	0	1	892	Cornelius Alexandershalmi	2005
1131	0	0	1	893	Pavelkelo	2003
1132	0	0	1	894	Jakubjedrzejczak	2006
1133	0	0	1	895	Giuseppecorsaro	2004
1134	0	0	1	896	Tarasignatiuk	2003
1135	0	0	1	897	Markusgiersiepen garcia	2005
1136	0	0	1	898	Julienimbert	2005
1137	0	0	1	899	Valentinvelazquez	2003
1138	0	0	1	900	Alexanderushakov	2004
1139	0	0	1	901	Zigasvec	2006
1140	0	0	1	902	Siddharthalama	2005
1141	0	0	1	903	Danielzimmermann	2005
1142	0	0	1	904	Max Shun Wangkwok	2005
1143	0	0	1	905	Christianteszari	2004
1144	0	0	1	906	Diegocueto de romana	2005
1145	0	0	1	907	Lachlanbrain	2003
1146	0	0	1	908	Umut Kaanyenilmez	2003

1147	0	0	1	909	Georgiosvalaris	2004
1148	0	0	1	910	Paulo Hugoetchecoin	2005
1149	0	0	1	911	Gianlucacadenasso	2004
1150	0	0	1	912	Semenvoronin	2003
1151	0	0	1	913	Clementkubiak	2005
1152	0	0	1	913	Supasetsincharoenwattana	2004
1153	0	0	1	915	Vardanmanukyan	2004
1154	0	0	1	916	Mihailosavic	2003
1155	0	0	1	917	Abhishekthorat	2006
1156	0	0	1	918	Yunestalavar	2004
1157	0	0	1	919	Benjamingusic wan	2007
1158	0	0	1	920	Nicolasoliveira	2006
1159	0	0	1	921	Oskarjansson	2004
1160	0	0	1	922	Matheuscury bueres	2003
1161	0	0	1	923	Uladzislaustrugach	2004
1162	0	0	1	924	Simonknezevic	2005
1163	0	0	1	925	Shogosanada	2005
1164	0	0	1	926	Ervinmesic	2004
1165	0	0	1	927	Nachoserra sanchez	2004
1166	0	0	1	928	Robertguna	2005
1167	0	0	1	929	Roanjones	2003
1168	0	0	1	930	Kaua Emanuelpereira dos santo	2005
1169	0	0	1	931	Alimounir	2003
1170	0	0	1	932	Brendan Nicholashendrata	2005
1171	0	0	1	933	Stefansimeunovic	2005
1172	0	0	1	934	Oscarbain moreno de vega	2004
1173	0	0	1	935	Dorianvasung	2004
1174	0	0	1	936	Yumaeda	2005
1175	0	0	1	937	Edwardvo	2003
1176	0	0	1	938	Andrenemeth	2006
1177	0	0	1	939	Oribaron	2004
1178	0	0	1	940	Nikoslehmann	2005
1179	0	0	1	941	Makarelesin	2005
1180	0	0	1	942	Thiago Horaciomessa	2004
1181	0	0	1	943	Nisheshbasavareddy	2005
1182	0	0	1	944	Hiirosakamoto	2005
1183	0	0	1	945	Pedro Antoniosavelli cardoso	2003
1184	0	0	1	946	Filipavdeev	2006
1185	0	0	1	946	Tobiaswirlend	2003
1186	0	0	1	948	Lechgontar	2003
1187	0	0	1	949	Dmitrybessonov	2006
1188	0	0	1	950	Ernestorequena	2003
1189	0	0	1	951	Richardantoni	2005
1190	0	0	1	952	Benjaminmartin	2005
1191	0	0	1	953	jimschmitter	2004
1192	0	0	1	954	Hyunwoonseo	2005
1193	0	0	1	955	Brunofernandez	2004
1194	0	0	1	956	Sebastianfigueira	2004
1195	0	0	1	957	Dario Alexandruciobotaru	2004
1196	0	0	1	958	Juan Davidrobayo arias	2005

1197	0	0	1	959	Fernandobulnes garrorena	2004
1198	0	0	1	960	Tygengoldammer	2005
1199	0	0	1	961	Batuataseven	2004
1200	0	0	1	962	Hughwinter	2006
1201	0	0	1	963	Oleksandrguriev	2005
1202	0	0	1	964	Parvnage	2003
1203	0	0	1	965	Shumuto	2006
1204	0	0	1	966	Cesarcruz	2006
1205	0	0	1	967	Piet Luispinter	2005
1206	0	0	1	968	Pascaltylek	2004
1207	0	0	1	969	Marekpazdera	2005
1208	0	0	1	970	Federicogarbero	2005
1209	0	0	1	971	Abubakariyakubu-lea	2006
1210	0	0	1	972	Pepijnbastiaansen	2005
1211	0	0	1	973	Alejandrohernandez	2004
1212	0	0	1	974	Andreastimini	2006
1213	0	0	1	975	Dhillonvirdee oakley	2003
1214	0	0	1	976	Muriloantunes burckhardt	2004
1215	0	0	1	977	Jankumstat	2007
1216	0	0	1	978	Joaquinguilleme	2006
1217	0	0	1	979	Milogazivoda	2003
1218	0	0	1	980	Fernandolimon rumlova	2005
1219	0	0	1	981	Alimetwalli	2004
1220	0	0	1	982	Gabriellosiewicz	2004
1221	0	0	1	983	Ivanperez martin	2004
1222	0	0	1	984	Razkatz	2003
1223	0	0	1	985	Mikaeelwoodman	2003
1224	0	0	1	986	Dorianley	2005
1225	0	0	1	987	Jamesconnel	2003
1226	0	0	1	988	Aymen Abderrahmeneali mous	2003
1227	0	0	1	989	Filiptatera	2003
1228	0	0	1	990	Juanestevez	2005
1229	0	0	1	991	Omarali	2004
1230	0	0	1	992	Damiancraddock	2004
1231	0	0	1	993	Sanjithdevineni	2003
1232	0	0	1	994	Meesrottgering	2007
1233	0	0	1	995	Jamesmcpherson	2003
1234	0	0	1	996	Fabiode michele	2005
1235	0	0	1	997	Shrikeshavmurugesan	2006
1236	0	0	1	998	Amiromarkhanov	2007
1237	0	0	1	999	Olegsyrovatskiy	2003
1238	0	0	1	1000	Mustafadustov	2004
1239	0	0	1	1001	Martinpestana	2004
1240	0	0	1	1002	Ofekshimanov	2005
1241	0	0	1	1003	David Tonyedawariye	2004
1242	0	0	1	1004	Mihail Mario Alexandrupop	2004
1243	0	0	1	1005	Kristianjanci	2004
1244	0	0	1	1006	Juliusaugunas	2005
1245	0	0	1	1007	Charlierobertson	2006
1246	0	0	1	1008	Michalwlodarczyk	2004

1247	0	0	1	1009	Alejandro Josegandini	2003
1248	0	0	1	1010	Nemanjastefanovic	2004
1249	0	0	1	1011	Dhananjayathreya	2005
1250	0	0	1	1012	Natsukiyamamoto	2005
1251	0	0	1	1013	Octavecaillat	2006
1252	0	0	1	1014	Karlnagel-heyer	2006
1253	0	0	1	1015	Lukasmaskow	2003
1254	0	0	1	1016	Josemurariu	2005
1255	0	0	1	1017	Oscar Andresperez gil	2004
1256	0	0	1	1018	Matthewbrowning	2004
1257	0	0	1	1019	Egorkirakosov	2004
1258	0	0	1	1020	Haoduan	2005
1259	0	0	1	1021	Darwinblanch	2007
1260	0	0	1	1022	Emildapcevic	2005
1261	0	0	1	1023	Marcoscastellanos	2005
1262	0	0	1	1024	Piotrandrzejewski	2005
1263	0	0	1	1025	Vitaliyzatsepin	2006
1264	0	0	1	1026	Jiricizek	2005
1265	0	0	1	1027	Torusrawat	2003
1266	0	0	1	1028	Emiliosanchez bronzetti	2004
1267	0	0	1	1029	Asafschwartz	2004
1268	0	0	1	1030	Johngabelic	2003
1269	0	0	1	1031	Donghyunhwang	2007
1270	0	0	1	1032	Jiangyunhan	2004
1271	0	0	1	1033	Jaytemming	2005
1272	0	0	1	1034	Niccolofestini mira	2004
1273	0	0	1	1035	Maksgrygolowicz	2003
1274	0	0	1	1036	Lucasbritez risso	2003
1275	0	0	1	1036	Alessiotramontin	2003
1276	0	0	1	1038	Joshuacheng	2005
1277	0	0	1	1039	Rafaelsbeghen sabio	2004
1278	0	0	1	1040	Paolo Benedictobonaguro la ro	2005
1279	0	0	1	1041	Alex-Andreojassalu	2003
1280	0	0	1	1042	Ernestoruy sanchez pavon	2004
1281	0	0	1	1043	Hyeokmin	2004
1282	0	0	1	1044	Kaua Lopescressoni	2005
1283	0	0	1	1045	Casparjalili mikkelsen	2003
1284	0	0	1	1046	Davidsimeonov	2005
1285	0	0	1	1047	Gabrieliusguzauskas	2003
1286	0	0	1	1048	Haroldvan raemdonck	2005
1287	0	0	1	1049	Leonardobastos	2005
1288	0	0	1	1050	Uladzislautarasevich	2004
1289	0	0	1	1051	Aleshrdina	2003
1290	0	0	1	1052	Migueltobon	2006
1291	0	0	1	1053	Elliotjarosz	2003
1292	0	0	1	1054	Lazardimitrijevic	2005
1293	0	0	1	1055	Sebastian Andresstipanov	2005
1294	0	0	1	1056	Gordan Franmileusnic	2003
1295	0	0	1	1057	Stepanzhoromsky	2005
1296	0	0	1	1058	Diegoquispe-kim	2005

1297	0	0	1	1059	Matteolataste	2004
1298	0	0	1	1060	magdeldin	2004
1299	0	0	1	1061	Glebkuharev	2003
1300	0	0	1	1062	Kostiantynmantach	2006
1301	0	0	1	1063	Giovannibranchetti	2003
1302	0	0	1	1064	Gabrielgaray	2003
1303	0	0	1	1065	Artemchernykh	2005
1304	0	0	1	1066	Haesunlee	2004
1305	0	0	1	1067	Lassepoertner	2005
1306	0	0	1	1068	Dusanbaranec	2003
1307	0	0	1	1069	Ericli	2004
1308	0	0	1	1070	Ajaymalik	2003
1309	0	0	1	1071	Wyattobrien	2005
1310	0	0	1	1072	Josefzapletal	2003
1311	0	0	1	1072	Mujtabaali-khan	2003
1312	0	0	1	1074	Bekzodnematov	2005
1313	0	0	1	1075	Antonioprat	2004
1314	0	0	1	1076	Markopavicic	2004
1315	0	0	1	1077	Nielsratiu	2005
1316	0	0	1	1078	Devaharshithneelam	2005
1317	0	0	1	1079	Jaden Dewandakatan	2006
1318	0	0	1	1080	Cristiancare	2005
1319	0	0	1	1081	caniceabua	
1320	0	0	1	1082	Daniilpiatrouski	2003
1321	0	0	1	1083	Beomchanoh	2004
1322	0	0	1	1084	Giovannivitali	2004
1323	0	0	1	1084	Maxdickey	2003
1324	0	0	1	1086	Calvinwang	2006
1325	0	0	1	1087	Juankim	2006
1326	0	0	1	1088	Jankusy	2005
1327	0	0	1	1089	Peterpolitis	2003
1328	0	0	1	1090	owenmegargee	2004
1329	0	0	1	1091	Aramnoroozian	2003
1330	0	0	1	1092	Angel Josuemaleno rosario	2003
1331	0	0	1	1093	Sergiocallejon hernando	2004
1332	0	0	1	1094	Lorenzosciahbasi	2005
1333	0	0	1	1095	Pavelmarinov	2006
1334	0	0	1	1096	Kristofkincses	2003
1335	0	0	1	1097	Enrique Mariadoldan sisa	2004
1336	0	0	1	1098	Cesarpalafox	2003
1337	0	0	1	1099	Alexanderchang	2003
1338	0	0	1	1099	Albert Porsmyrhansen	2003
1339	0	0	1	1099	Sachinpalta	2003
1340	0	0	1	1099	Waleedqadir	2004
1341	0	0	1	1099	Elistephenson	2004
1342	0	0	1	1104	Ilyamaltsev	2007
1343	0	0	1	1105	Toamaeda	2006
1344	0	0	1	1106	Evanwen	2004
1345	0	0	1	1107	Janisgraski	2006
1346	0	0	1	1108	Dhruvtangri	2003

1347	0	0	1	1109	Kianvakili	2003
1348	0	0	1	1110	Juan Eduardoballesteros	2005
1349	0	0	1	1111	Enriquelaennec molins	2005
1350	0	0	1	1112	Artemalekseychuk	2005
1351	0	0	1	1113	Michaelkouame	2006
1352	0	0	1	1114	Gabrielschenekenberg	2004
1353	0	0	1	1115	Andresdelgadillo	2006
1354	0	0	1	1116	Adithyasuresh	2005
1355	0	0	1	1117	Paulopocasangre	2004
1356	0	0	1	1118	Pedropinto	2005
1357	0	0	1	1119	Mohammad parsaafsari	2004
1358	0	0	1	1120	Adriandaniel	2003
1359	0	0	1	1121	Benhudson	2005
1360	0	0	1	1122	Ondrejkratochvil	2004
1361	0	0	1	1123	Sachagrandvincent	2005
1362	0	0	1	1124	Teunrozenberg	2003
1363	0	0	1	1125	Borisdurovic	2005
1364	0	0	1	1126	Jonashahn	2007
1365	0	0	1	1127	Pavellagutin	2005
1366	0	0	1	1128	Patrikhomola	2004
1367	0	0	1	1129	Issamjonsharifov	2003
1368	0	0	1	1130	Bastiaanweststrate	2003
1369	0	0	1	1131	Mohammadalkotop	2005
1370	0	0	1	1131	Denispetak	2006
1371	0	0	1	1133	Humzanoor	2005
1372	0	0	1	1134	Matejkajzer	2005
1373	0	0	1	1135	Aleksakrivokapic	2003
1374	0	0	1	1136	Deepmunim	2004
1375	0	0	1	1137	Karansingh	2003
1376	0	0	1	1138	Semenbatin	2005
1377	0	0	1	1139	Zhangeralmashev	2003
1378	0	0	1	1140	Romanvaletov	2005
1379	0	0	1	1141	Tomasmarin	2006
1380	0	0	1	1142	Gabrielberner	2004
1381	0	0	1	1142	Meethrebarot	2005
1382	0	0	1	1144	Markuslazarcik	2005
1383	0	0	1	1145	Rayenhermassi	2004
1384	0	0	1	1146	Edoardosantoni	2005
1385	0	0	1	1147	Nikolakeremedchiev	2003
1386	0	0	1	1148	Ricardodutra	2004
1387	0	0	1	1149	Patrykpalyszko	2004
1388	0	0	1	1150	Alejandrobravo diaz	2005
1389	0	0	1	1151	Gerardhenning	2004
1390	0	0	1	1152	Aoiooka	2006
1391	0	0	1	1153	Louislarue	2004
1392	0	0	1	1154	Gerardplanelles ripoll	2003
1393	0	0	1	1155	Adrianquiros monterrey	2005
1394	0	0	1	1156	Benitomassacri	2003
1395	0	0	1	1157	Philipkamara	2004
1396	0	0	1	1158	Flynn Tjarkbaumert	2005

1397	0	0	1	1159	Takuramhwandagara	2006
1398	0	0	1	1160	Eytanmichaeli	2003
1399	0	0	1	1160	Carlroothman	2003
1400	0	0	1	1160	Tajhibbert	2004
1401	0	0	1	1163	Minjoonkim	2004
1402	0	0	1	1164	Filippomartini	2003
1403	0	0	1	1165	Davidasenov	2006
1404	0	0	1	1166	Ioannisvon meyenburg	2004
1405	0	0	1	1167	Christian Anthonysalmeron	2006
1406	0	0	1	1168	Alyel rafie	2004
1407	0	0	1	1169	William Chasethompson	2004
1408	0	0	1	1169	Stephaniserath	2004
1409	0	0	1	1171	Viacheslavbaranenkov	2005
1410	0	0	1	1172	Maemalige	2006
1411	0	0	1	1173	Levgorb	2006
1412	0	0	1	1173	Lucasschurdevin	2003
1413	0	0	1	1175	Felipevirgili berini	2005
1414	0	0	1	1176	Nyovan dyck	2005
1415	0	0	1	1177	Tinostro	2003
1416	0	0	1	1178	Davidekpenyong	2005
1417	0	0	1	1179	Georgechaidemenos	2004
1418	0	0	1	1180	Felipeiser	2004
1419	0	0	1	1181	Daniilssnaiders	2003
1420	0	0	1	1182	Daniilryzhankou	2006
1421	0	0	1	1183	Yonibidny	2004
1422	0	0	1	1184	Se Hyunkim	2006
1423	0	0	1	1185	Taekgyujeong	2004
1424	0	0	1	1186	Mustaphael natour	2004
1425	0	0	1	1187	Lukasramer olofsson	2003
1426	0	0	1	1188	Danieleaugenti	2004
1427	0	0	1	1189	Petrpanacek	2005
1428	0	0	1	1190	Georgelouw	2004
1429	0	0	1	1191	Edwardstan	2006
1430	0	0	1	1192	Eduardobribiesca ponce	2004
1431	0	0	1	1193	Aleksandartomas	2003
1432	0	0	1	1194	Rudyceccon	2004
1433	0	0	1	1195	Thomasmathys	2004
1434	0	0	1	1196	Albertpedrico kravtsov	2005
1435	0	0	1	1197	Rafaelde la vega olascoaga	2005
1436	0	0	1	1197	Alvarogarcia	2005
1437	0	0	1	1199	Hayatokurihara	2004
1438	0	0	1	1200	Danielpersson	2003
1439	0	0	1	1201	Williambartram	2004
1440	0	0	1	1202	Dong Geonkim	2005
1441	0	0	1	1203	Seungminpark	2005
1442	0	0	1	1204	William Tristanlee iii	2004
1443	0	0	1	1204	Mateocastaneda	2006
1444	0	0	1	1206	Ivoisqueiro	2004
1445	0	0	1	1207	Filiphorak	2006
1446	0	0	1	1208	Ahmetatabay	2005

1447	0	0	1	1209	Kennethadjokatse	2004
1448	0	0	1	1210	Tsz Himchan	2004
1449	0	0	1	1211	Diego Josesegovia perez	2003
1450	0	0	1	1212	Hermannnsahno ndonfack	2005
1451	0	0	1	1213	Huasendong	2004
1452	0	0	1	1214	Jhoan Sebastianvaquero	2004
1453	0	0	1	1215	Naimbenito fernandez	2005
1454	0	0	1	1216	Germanodiehl setti	2004
1455	0	0	1	1217	Amarhuseinovic	2007
1456	0	0	1	1218	Lucasandrade da silva	2005
1457	0	0	1	1219	Erickats	2003
1458	0	0	1	1220	Pannawatsuttisomboon	2006
1459	0	0	1	1221	Henryjefferson	2006
1460	0	0	1	1222	Teomasera	2005
1461	0	0	1	1223	Maximus Zavierwong	2007
1462	0	0	1	1224	Nicolastrebuchet	2005
1463	0	0	1	1225	Viktorblom	2003
1464	0	0	1	1226	Sergio Alejandrogonzalez ibane	2003
1465	0	0	1	1227	Billchan	2006
1466	0	0	1	1228	Andrescastellanos carrillo	2004
1467	0	0	1	1229	Loannmassard	2005
1468	0	0	1	1230	Thiagodrozdowski	2006
1469	0	0	1	1231	Alexanderpatane	2003
1470	0	0	1	1232	Aricotoulas	2005
1471	0	0	1	1233	Gabrielmiranda	2003
1472	0	0	1	1234	Anthonyweingarten	2003
1473	0	0	1	1235	Matthias Uwekask	2005
1474	0	0	1	1235	Jose Davidsegovia perez	2006
1475	0	0	1	1237	Lovreerceg	2003
1476	0	0	1	1238	Georgigeorgiev	2006
1477	0	0	1	1239	Santiagoferrero	2004
1478	0	0	1	1240	Gaborhornung	2005
1479	0	0	1	1241	Giannilopez	2004
1480	0	0	1	1242	Juan Pablovaldez guzon	2005
1481	0	0	1	1243	Radu Danielflorea	2004
1482	0	0	1	1244	Alfredogallegos	2005
1483	0	0	1	1245	Filipbojko	2004
1484	0	0	1	1246	Andreasloizas	2006
1485	0	0	1	1247	Clementbergantz	2006
1486	0	0	1	1248	Luis Enriqueperez penaloza	2004
1487	0	0	1	1249	Thomaskano	2005
1488	0	0	1	1250	Matyaszavadil	
1489	0	0	1	1251	Benjaminkreynes	2006
1490	0	0	1	1252	nikolapuntaric	2004
1491	0	0	1	1253	Ching Kitfong	2003
1492	0	0	1	1254	Joshuakim	2005
1493	0	0	1	1255	Zhakaskozbak	2006
1494	0	0	1	1256	Saveliishpita	2005
1495	0	0	1	1257	Vladislavabramov	2004
1496	0	0	1	1258	Jamesdelgado	2003

1497	0	0	1	1259	Kevinfu	2003
1498	0	0	1	1260	Tymurbieldiugin	2005
1499	0	0	1	1261	Pierrengijol carre	2004
1500	0	0	1	1262	Noavukadin	2004
1501	0	0	1	1262	Jakedembo	2008
1502	0	0	1	1262	Marcusschoeman	2005
1503	0	0	1	1265	Josipsimundza	2004
1504	0	0	1	1266	Raghavsundararaman	2004
1505	0	0	1	1266	Davidleon mantilla	2003
1506	0	0	1	1268	Oscar Andresmartinez de freita	2006
1507	0	0	1	1269	Donigashi	2004
1508	0	0	1	1270	Darcynicholls	2003
1509	0	0	1	1271	Justinengel	2007
1510	0	0	1	1272	Marshallhulett	2004
1511	0	0	1	1273	Jaccoarends	2003
1512	0	0	1	1273	Daanmeijer	2005
1513	0	0	1	1273	Jeremyzhang	2005
1514	0	0	1	1276	Nikitasafonov	2004
1515	0	0	1	1277	Lukehooper	2006
1516	0	0	1	1278	Ivovercik	2003
1517	0	0	1	1279	Samanrezaeyan	2004
1518	0	0	1	1280	Jayfairclough	2005
1519	0	0	1	1281	Linusvidehult	2003
1520	0	0	1	1281	Alenbill	2003
1521	0	0	1	1283	Lucasmarionneau	2006
1522	0	0	1	1284	Filiphanzelin	2006
1523	0	0	1	1285	Stephane Pierre-Jokamendje	2006
1524	0	0	1	1286	Ivanvladimirov	2005
1525	0	0	1	1287	Rodrigodeleu	2003
1526	0	0	1	1288	Lenniloukkola	2005
1527	0	0	1	1289	Gabrieljornert	2004
1528	0	0	1	1290	Bryce Nakashimanakashima	2004
1529	0	0	1	1291	Valentinpena	2003
1530	0	0	1	1292	Sebastiansorger	2005
1531	0	0	1	1293	Felixalopaeus	2007
1532	0	0	1	1294	Niklasgrunewald	2004
1533	0	0	1	1295	Martimmarujo	2003
1534	0	0	1	1296	Lukasescher	2005
1535	0	0	1	1297	Pumiphatsukkho	2006
1536	0	0	1	1298	Lukasrais	2005
1537	0	0	1	1299	Ariyojavadi	2004
1538	0	0	1	1300	Arunavamajumder	2005
1539	0	0	1	1301	Bartlomiejros	2004
1540	0	0	1	1302	Fernandocavallo	2005
1541	0	0	1	1303	Levsholosh	2004
1542	0	0	1	1304	Bogdandesy	2005
1543	0	0	1	1305	Mikowassermann	2003
1544	0	0	1	1305	Yumadaijima	2003
1545	0	0	1	1307	Willmayew	2004
1546	0	0	1	1308	Musasani	2003

1547	0	0	1	1309	Shumatsuoka	2003
1548	0	0	1	1309	Stepanboytsov	2004
1549	0	0	1	1311	Rethin Pranavsenthil kumar	2007
1550	0	0	1	1312	Shionitsusaki	2007
1551	0	0	1	1313	Owenlien	2007
1552	0	0	1	1314	Facundoperlov	2004
1553	0	0	1	1315	Santiago Adrianvedia villegas	2004
1554	0	0	1	1316	Alexihebicka	2005
1555	0	0	1	1317	Murilogracio martins	2005
1556	0	0	1	1318	Gabrielgomez	2004
1557	0	0	1	1319	Yamatoyukawa	2006
1558	0	0	1	1320	Zamaanmoledina	2003
1559	0	0	1	1321	Shanetapera	2004
1560	0	0	1	1322	Vanjahodzic	2003
1561	0	0	1	1323	Aurelienschmitt	2005
1562	0	0	1	1324	Luis Felipeazurduy castellanos	2003
1563	0	0	1	1325	Francescoscarpa	2004
1564	0	0	1	1326	Emilmatikainen	2005
1565	0	0	1	1327	Samuelheredia	2006
1566	0	0	1	1328	Oliverthoeny	2004
1567	0	0	1	1329	Eyalshumilov	2006
1568	0	0	1	1330	Ottoleikola	2003
1569	0	0	1	1331	Abdullahadnan	2003
1570	0	0	1	1332	Santiagosoto	2006
1571	0	0	1	1333	Romankonovalov	2004
1572	0	0	1	1334	Santiago Arturoflores moreno	2005
1573	0	0	1	1335	Davidmanu	2005
1574	0	0	1	1336	Karoltomanski	2003
1575	0	0	1	1337	Matteoceradelli	2005
1576	0	0	1	1338	Arturibragimov	2005
1577	0	0	1	1339	Andreizubkov	2004
1578	0	0	1	1340	Jacques-Kaiinoue	2004
1579	0	0	1	1341	Vojtechvales	2006
1580	0	0	1	1342	Kabirchhabria	2004
1581	0	0	1	1343	Tim Danielstemler	2003
1582	0	0	1	1344	Arjunbalakrishnan	2005
1583	0	0	1	1345	Pablopaternostro	2005
1584	0	0	1	1346	Andreyshutov	2003
1585	0	0	1	1347	Roberto Alejandroleal	2006
1586	0	0	1	1348	Walkerallen	2005
1587	0	0	1	1349	VINHHIENtruong	2004
1588	0	0	1	1350	Khomtatkitthachok	2004
1589	0	0	1	1351	Ching Wahwai	2003
1590	0	0	1	1352	Carlos Eduardomorante	2005
1591	0	0	1	1353	Kyranmagimay	2004
1592	0	0	1	1354	Amitshamash	2005
1593	0	0	1	1355	Aki Zubenrawat	2006
1594	0	0	1	1356	Rodrigo Alonsotabormina araya	2003
1595	0	0	1	1356	Itayfeigin	2003
1596	0	0	1	1356	Juanmaaccossatto	2003

1597	0	0	1	1359	Wongwarunwongcharoen	2006
1598	0	0	1	1360	Albertoblumenschein	2004
1599	0	0	1	1361	Mahmut Berabinici	2004
1600	0	0	1	1362	Maxwellexsted	2007
1601	0	0	1	1363	Nicolas Marcelotrigo lopez	2003
1602	0	0	1	1364	Vadymrogynskyi	2004
1603	0	0	1	1365	Marknawa	2006
1604	0	0	1	1366	Juan Josemorales gutierrez	2005
1605	0	0	1	1367	Amartahirovic	2004
1606	0	0	1	1368	Juan Camiloperez gongora	2003
1607	0	0	1	1369	Henrilyons	2003
1608	0	0	1	1370	Majdiben abdelwehid	2004
1609	0	0	1	1371	Eldinhuseinovic	2003
1610	0	0	1	1371	Joseph franklinjimoh	2007
1611	0	0	1	1373	Matisroche	2004
1612	0	0	1	1373	Rasmusegehus	2003
1613	0	0	1	1373	Christianfellin	2003
1614	0	0	1	1376	Paytonyoung	2004
1615	0	0	1	1377	Abdelrahmanargoun	2005
1616	0	0	1	1378	Andresriveros-tejada puerta	2005
1617	0	0	1	1379	Stefansaudrins	2006
1618	0	0	1	1380	Koloskincses	2007
1619	0	0	1	1380	Jettleong	2004
1620	0	0	1	1382	Yardencauly	2005
1621	0	0	1	1383	Santiagoflyckt	2004
1622	0	0	1	1384	Ioan Alexandruteglas	2003
1623	0	0	1	1385	Ognjenmilic	2007
1624	0	0	1	1386	Rawiyosef	2003
1625	0	0	1	1386	Emmettpotter	2005
1626	0	0	1	1388	Kui Chi Antoniolee	2007
1627	0	0	1	1389	Skythavornchaisophon	2005
1628	0	0	1	1390	Angelmalinowski	2004
1629	0	0	1	1391	Marcos Leechan baratau	2005
1630	0	0	1	1392	Jamisavolainen	2005
1631	0	0	1	1393	Tyswitzer	2003
1632	0	0	1	1394	Guyvorwerk	2006
1633	0	0	1	1395	Tudorbatin	2007
1634	0	0	1	1396	Nicolasrakela herrera	2004
1635	0	0	1	1397	SETHwong	2006
1636	0	0	1	1397	Tannerpovey	2005
1637	0	0	1	1399	Jihoonkang	2005
1638	0	0	1	1399	Daniloraicevic	2003
1639	0	0	1	1401	Andrescortes	2003
1640	0	0	1	1402	Tymofiykhrystyuk	2004
1641	0	0	1	1402	Guilhermerickli da matta	2004
1642	0	0	1	1402	Gianlucakunz	2005
1643	0	0	1	1405	Igorvisnevskij	2003
1644	0	0	1	1406	Duncanmccall	2005
1645	0	0	1	1407	Batsomimarobela	2004
1646	0	0	1	1408	Artjomsafanasjevs	2005

1647	0	0	1	1409	Roshufujioka	2005
1648	0	0	1	1410	Omermann	2003
1649	0	0	1	1410	Haydenloffler	2004
1650	0	0	1	1412	Rikitakahata	2004
1651	0	0	1	1413	Tamasfoldi	2003
1652	0	0	1	1414	Mitchelllee	2006
1653	0	0	1	1415	Sebastiangarcia escorcia	2005
1654	0	0	1	1416	Luc Anandaceuca	2005
1655	0	0	1	1417	Ismailsafwat	2005
1656	0	0	1	1418	Mikel Josugarmendia de la igles	2004
1657	0	0	1	1419	Felipede dios	2003
1658	0	0	1	1420	Lucaebenriter	2003
1659	0	0	1	1421	Garethong	2004
1660	0	0	1	1421	Darkhanzhakanger	2006
1661	0	0	1	1423	Loganstoltz	2003
1662	0	0	1	1424	Axeleriksson	2004
1663	0	0	1	1425	Pietropampanin	2003
1664	0	0	1	1426	Erictripathi	2003
1665	0	0	1	1427	Akarshgaonkar	2004
1666	0	0	1	1428	Hudsonbeaudoin	2003
1667	0	0	1	1429	Alexcairo	2004
1668	0	0	1	1430	Temirlanmekhtiev	2005
1669	0	0	1	1431	Juan Ignaciolombisano	2003
1670	0	0	1	1432	Alexandernordal clausen	2006
1671	0	0	1	1433	Alexandredacorte	2006
1672	0	0	1	1434	Sebastian Andresrondon gomez	2003
1673	0	0	1	1435	Omarfakhoury	2003
1674	0	0	1	1436	Botondkisantal	2005
1675	0	0	1	1436	Dzhordzhobinda	2005
1676	0	0	1	1438	Albertogarcia garcia	2004
1677	0	0	1	1439	Mareknano	2006
1678	0	0	1	1439	Martinmazev	2004
1679	0	0	1	1441	Nour Salahibrahim	2004
1680	0	0	1	1442	Marvinkao	2004
1681	0	0	1	1443	Ivandreycopp	2003
1682	0	0	1	1443	Firasben chouikha	2003
1683	0	0	1	1445	Alexandru Cristiandumitru	2004
1684	0	0	1	1446	Christopherlam	2004
1685	0	0	1	1447	Levlutkov	2005
1686	0	0	1	1447	Petarbelosevic	2003
1687	0	0	1	1449	Ghouse Alifaisal	2003
1688	0	0	1	1449	Christopher Koseiono	2006
1689	0	0	1	1451	Christophgrant	2005
1690	0	0	1	1452	Noahregas luis	2004
1691	0	0	1	1452	Alejandroarcila	2007
1692	0	0	1	1454	Aldorandazzo	2005
1693	0	0	1	1455	Carel Pieterwessel	2004
1694	0	0	1	1456	Markusvainikka	2006
1695	0	0	1	1457	Marceldzielo	2004
1696	0	0	1	1457	Yassinhamed	2005

1697	0	0	1	1459	Samuelrovai	2004
1698	0	0	1	1460	Visheshpatel	2004
1699	0	0	1	1461	Matthewgriscti	2003
1700	0	0	1	1462	Jordanedwards	2003
1701	0	0	1	1463	Christian Felipewinstead	2003
1702	0	0	1	1464	Jonaskucera	2006
1703	0	0	1	1465	OKALAhagoua valentin brayan a	2003
1704	0	0	1	1466	Petrospieros	2003
1705	0	0	1	1467	Paternekouadio	2005
1706	0	0	1	1467	Jiholee	2005
1707	0	0	1	1469	Laurentjulia calac	2004
1708	0	0	1	1469	Alexandrede villepin	2003
1709	0	0	1	1471	Royleshem	2004
1710	0	0	1	1472	Cyrusmahjoob	2005
1711	0	0	1	1473	Felipe Joserivadeneira gallegos	2006
1712	0	0	1	1474	Andreifiraru	2005
1713	0	0	1	1475	Christophernorlin	2004
1714	0	0	1	1476	Sergejtopic	2006
1715	0	0	1	1477	Ivanpauliuchenka	2003
1716	0	0	1	1478	Jeronimobonoris	2003
1717	0	0	1	1479	Diegolaguna	2004
1718	0	0	1	1479	Davidde hoyos rodriguez	2004
1719	0	0	1	1481	Georgiiarakelian	2005
1720	0	0	1	1482	Moritzanterist	2004
1721	0	0	1	1483	Noahmueller	2005
1722	0	0	0	1484	Markomilosavljevic	2006
1723	0	0	0	1485	Ignacioquiroga	2003
1724	0	0	0	1486	Zurycohen	2005
1725	0	0	0	1487	Jose Antoniovelasco	2004
1726	0	0	0	1488	Yoavbloch	2004
1727	0	0	0	1488	Dhanushpatel	2004
1728	0	0	0	1488	Pablorobledo hoyos	2007
1729	0	0	0	1491	Heerakvora	2003
1730	0	0	0	1492	Dusanrsovac	2003
1731	0	0	0	1493	Tudorenea	2003
1732	0	0	0	1494	Luis Miguelsaraiva	2005
1733	0	0	0	1495	Boburmadaminov	2005
1734	0	0	0	1496	Soutayoshida	2004
1735	0	0	0	1496	Felipepolach	2004
1736	0	0	0	1498	Anthonywright	2004
1737	0	0	0	1499	Vuk Davidstramput	2003
1738	0	0	0	1500	Leonidaslazarides	2005
1739	0	0	0	1500	Konstantin Gadekruger	2005
1740	0	0	0	1502	Patriciosepulveda garza	2004
1741	0	0	0	1503	Ammartahoon	2006
1742	0	0	0	1504	Cristobalfuentes	2004
1743	0	0	0	1505	Raphaelpichot	2004
1744	0	0	0	1506	Tunanergizoglu	2004
1745	0	0	0	1507	Alessandroversteegh	2005
1746	0	0	0	1508	Leophijidvijan	2005

1747	0	0	0	1509	Timeogaillard	2006
1748	0	0	0	1510	Haitamaboufirassi	2005
1749	0	0	0	1511	Manricromero	2004
1750	0	0	0	1512	Chandanshivaraj	2005
1751	0	0	0	1512	Duhyuneum	2003
1752	0	0	0	1514	Zanestevens	2006
1753	0	0	0	1515	Sergiotintore	2004
1754	0	0	0	1516	Steffanvan weldam	2004
1755	0	0	0	1517	Eldinnokic	2004
1756	0	0	0	1518	Robertgaliullin	2006
1757	0	0	0	1519	Juliusleino	2004
1758	0	0	0	1520	Naruebodeeboonyawat	2005
1759	0	0	0	1521	Idogeva	2003
1760	0	0	0	1522	Niksaarsic	2004
1761	0	0	0	1522	Sturle Hoffskigelstrand	2005
1762	0	0	0	1524	Youssefkadiri hassani	2003
1763	0	0	0	1525	Oliverking	2006
1764	0	0	0	1525	Jean-Nicolasleblanc	2004
1765	0	0	0	1527	Simonkuszynski	2004
1766	0	0	0	1527	Qwynquittner	2004
1767	0	0	0	1527	Faycelkaddour	2003
1768	0	0	0	1530	Miguel Angelde rueda medina	2004
1769	0	0	0	1531	Martin Alejandrobarbera	2003
1770	0	0	0	1532	FEDERICO HERNANgiovanniello	2003
1771	0	0	0	1532	Mersedeli	2004
1772	0	0	0	1532	Martindespodov	2004
1773	0	0	0	1532	Phopthumsriwong	2008
1774	0	0	0	1536	Edvards Teodorsliepins	2003
1775	0	0	0	1537	Antonioayerbe streubel	2004
1776	0	0	0	1538	YUKIkaratsu	2005
1777	0	0	0	1538	Hamzaeltoukhi	2006
1778	0	0	0	1540	Aryangiri	2005
1779	0	0	0	1541	Tim Thomasgauntlett	2003
1780	0	0	0	1541	Zandervan orshoven	2005
1781	0	0	0	1543	Andresurquiola montellano	2004
1782	0	0	0	1544	Artyomastafyev	2003
1783	0	0	0	1545	Vitohorvat	2003
1784	0	0	0	1546	Adamfarag-cao	2007
1785	0	0	0	1546	Wiktorkosowski	2004
1786	0	0	0	1546	Oferhacohen	2003
1787	0	0	0	1549	Kamonpanyakornthadpong	2007
1788	0	0	0	1550	Tsubasanakamura	2004
1789	0	0	0	1550	Boxiongzhang	2007
1790	0	0	0	1550	Adam Gaborszoke	2004
1791	0	0	0	1550	Juan Ignaciozamora	2004
1792	0	0	0	1550	Guntinunsootinun-opart	2003
1793	0	0	0	1555	Ryuseimiyazato	2006
1794	0	0	0	1556	Stefanochappuis	2005
1795	0	0	0	1557	Ahmedmonsef	2006
1796	0	0	0	1558	Danielcardy	2004

1797	0	0	0	1559	Davidcerne	2004
1798	0	0	0	1560	Sotaminami	2003
1799	0	0	0	1561	Nicolasdonoso	2004
1800	0	0	0	1562	Alecsondeschamps	2003
1801	0	0	0	1562	Filipekrohn da silva	2003
1802	0	0	0	1564	Niccolodessi	2004
1803	0	0	0	1565	Glibsekachov	2006
1804	0	0	0	1566	Taonamhwandagara	2003
1805	0	0	0	1566	Ludvighede	2007
1806	0	0	0	1568	Michalcurlej	2004
1807	0	0	0	1569	Janis Raffaelsimmen	2006
1808	0	0	0	1570	Mathiastempel	2004
1809	0	0	0	1571	Clandestine Kudakwashendiring	2006
1810	0	0	0	1572	Youssefboubaker	2004
1811	0	0	0	1572	Vladislavgorbatenko	2003
1812	0	0	0	1574	Andybrisdon	2004
1813	0	0	0	1575	Christotimini	2004
1814	0	0	0	1576	Eren matthewcheung	2006
1815	0	0	0	1577	Romainandres	2007
1816	0	0	0	1578	Adavaitmantri	2006
1817	0	0	0	1578	Tomsorin	2004
1818	0	0	0	1578	Sebastianbohrt	2004
1819	0	0	0	1578	Matteolavizzari	2005
1820	0	0	0	1578	Luisching	2003
1821	0	0	0	1578	Marcmajdandzic	2005
1822	0	0	0	1578	Christophergarde	2005
1823	0	0	0	1578	Ligalkirshenboim	2005
1824	0	0	0	1586	Connorgibson	2003
1825	0	0	0	1587	Deancooper kirby	2003
1826	0	0	0	1587	Jakubstrzelecki	2004
1827	0	0	0	1589	Kristiantumbas kajgo	2005
1828	0	0	0	1589	Shingisbayseit	2005
1829	0	0	0	1591	Ruslanserazhetdinov	2005
1830	0	0	0	1592	Samuelguzon	2005
1831	0	0	0	1593	Diegoschtulmann	2005
1832	0	0	0	1594	Rayenzribi	2003
1833	0	0	0	1595	Mathieuderache	2004
1834	0	0	0	1596	Arsentiyyakovlev	2003
1835	0	0	0	1596	Andreilantsov	2004
1836	0	0	0	1596	David Cristiancarteputreda	2007
1837	0	0	0	1596	Sehyeongkim	2006
1838	0	0	0	1600	Pranavkarthik	2005
1839	0	0	0	1600	Pranavkarthik	2005
1840	0	0	0	1602	Daniilstepanov	2006
1841	0	0	0	1603	Arslanbektemirhanov	2003
1842	0	0	0	1604	Luccac pignaton	2006
1843	0	0	0	1604	Nikoladjosic	2007
1844	0	0	0	1606	Cooperwilkinson	2004
1845	0	0	0	1607	Ruslansfilipenko	2004
1846	0	0	0	1607	Selimkhouaja	2003

1847	0	0	0	1609	Noahgabriel	2005
1848	0	0	0	1610	Rikuogasawara	2004
1849	0	0	0	1610	Hugoouzilou	2003
1850	0	0	0	1610	Franciscoduran	2006
1851	0	0	0	1613	TAKUMIkatsuda	
1852	0	0	0	1614	Mostafanoureldine	2006
1853	0	0	0	1614	Vivekkolluru	2004
1854	0	0	0	1614	Juan Diegohernandez hernande	2003
1855	0	0	0	1617	Mikhailkorkunov	2003
1856	0	0	0	1618	Matsherremans	2004
1857	0	0	0	1619	Yahir Emilianomoheno	2003
1858	0	0	0	1620	Lachlanvickery	2004
1859	0	0	0	1621	Patrikhinz	2006
1860	0	0	0	1621	Andrewchang	2003
1861	0	0	0	1621	Andreassillaste	2004
1862	0	0	0	1621	Wimansawongkhan	2004
1863	0	0	0	1621	Nicolasgarcia longo	2005
1864	0	0	0	1621	Sarthakgandhi	2003
1865	0	0	0	1621	Krishangraghuvanshi	2005
1866	0	0	0	1621	Ikerurribarrens ramirez	2004
1867	0	0	0	1629	Dennybao	2006
1868	0	0	0	1630	Tarikbajrovic	2004
1869	0	0	0	1631	Harrylazell	2003
1870	0	0	0	1631	Peterkalocsai	2003
1871	0	0	0	1633	Lukajanuashvili	2005
1872	0	0	0	1633	Guglielmoverdese	2006
1873	0	0	0	1633	Samuelnyamekye	2003
1874	0	0	0	1636	Juwanbae	2005
1875	0	0	0	1637	Halelashoosh	2006
1876	0	0	0	1638	Apivichrojpiboonphan	2005
1877	0	0	0	1639	Garenspicka	2004
1878	0	0	0	1639	Jonatansztasak	2003
1879	0	0	0	1641	Shokioshita	2004
1880	0	0	0	1641	Daniel Josepinzon pinto	2004
1881	0	0	0	1643	Adamgroves	2003
1882	0	0	0	1644	Sebastianaranzeta boneta	2003
1883	0	0	0	1645	Stefanvedovelli	2005
1884	0	0	0	1646	Milanristov	2005
1885	0	0	0	1646	Thomasaston	2005
1886	0	0	0	1646	Ibragimturgambekov	2005
1887	0	0	0	1649	Cho Kong Ethanluk	2005
1888	0	0	0	1649	Simondaune	2006
1889	0	0	0	1651	Lukekoh	2007
1890	0	0	0	1652	Zacharypellouchoud	2003
1891	0	0	0	1653	Youssefihab mokhles	2004
1892	0	0	0	1653	Kuang Qingxu	2006
1893	0	0	0	1655	Agustin Eduardocuellar lorberg	2003
1894	0	0	0	1655	Moritzpieper	2004
1895	0	0	0	1655	Bencejuhasz	2006
1896	0	0	0	1655	Calumjestin	2004

1897	0	0	0	1655	Petergombas	2003
1898	0	0	0	1655	Emonvan loben sels	2004
1899	0	0	0	1655	Adammajchrzak	2005
1900	0	0	0	1655	Anasbennour dit sahli	2006
1901	0	0	0	1663	Bogdanborodin	2006
1902	0	0	0	1664	Masondace	2004
1903	0	0	0	1664	Sichengli	2005
1904	0	0	0	1666	Davidjavadov	2006
1905	0	0	0	1667	Harrypugh	2006
1906	0	0	0	1668	Andresserrano	2004
1907	0	0	0	1669	Moritzschroeter	2003
1908	0	0	0	1669	Jose Andresperez	2004
1909	0	0	0	1671	Raducojocaru	2006
1910	0	0	0	1671	Bruno Carlosbacigalupi carrizos	2003
1911	0	0	0	1673	Vladimirholban	2005
1912	0	0	0	1673	Lucabluett	2005
1913	0	0	0	1675	Lorenzoangelini	2006
1914	0	0	0	1675	Noaforste dinell	2005
1915	0	0	0	1677	Rahulsameer	2004
1916	0	0	0	1678	Harrisongradin	2004
1917	0	0	0	1679	Hugomagnusson	2004
1918	0	0	0	1680	Mudiwamunyimani	2006
1919	0	0	0	1681	Muhammadabdulsalam	2004
1920	0	0	0	1681	Danielerapagnetta	2006
1921	0	0	0	1681	Markostoilkovski	2005
1922	0	0	0	1684	Alanabdullin	2006
1923	0	0	0	1684	Ollenoltorp	2004
1924	0	0	0	1686	Ahmedamin	2005
1925	0	0	0	1687	Filipecosta	2005
1926	0	0	0	1688	Tomonobuinagi	2006
1927	0	0	0	1688	Yuitosasaki	2005
1928	0	0	0	1690	Geonwoolee	2005
1929	0	0	0	1690	Jose Franciscocalderon	2003
1930	0	0	0	1690	Markonikolic	2005
1931	0	0	0	1690	Boburmirzoekubjanov	2005
1932	0	0	0	1694	Filipvacula	2005
1933	0	0	0	1694	Ondrejvalek	2003
1934	0	0	0	1694	Augustgustafson	2003
1935	0	0	0	1694	Lorenzocomino	2006
1936	0	0	0	1694	Ryotaigarashi	2006
1937	0	0	0	1699	Jonagitschel	2003
1938	0	0	0	1700	Zavanfahim	2003
1939	0	0	0	1700	Zavanfahim	2003
1940	0	0	0	1700	Ilyas Miladfahim	2005
1941	0	0	0	1700	Omarfarahat	2005
1942	0	0	0	1704	Inigovillacampa goni	2004
1943	0	0	0	1704	Viggogrondahl	2003
1944	0	0	0	1704	Nikolaossfyrakis	2004
1945	0	0	0	1704	Niklasjensen	2004
1946	0	0	0	1704	Berentan	2004

1947	0	0	0	1704	Tomasbenitez	2004
1948	0	0	0	1704	Sui Lunwang	2005
1949	0	0	0	1711	Kartikparhar	2003
1950	0	0	0	1712	Kanangasimov	2005
1951	0	0	0	1713	Jakubgogulski	2005
1952	0	0	0	1714	Ion Arcasastraciuc	2003
1953	0	0	0	1714	Lucascouy	2005
1954	0	0	0	1716	Kevinkovacs	2003
1955	0	0	0	1716	Erikkovacs	2003
1956	0	0	0	1718	Abasskenneh	2007
1957	0	0	0	1718	Danylomaslennikov	2005
1958	0	0	0	1718	victorarrese mata	2004
1959	0	0	0	1718	Joshuadous karpenschif	2003
1960	0	0	0	1718	Mykhailomossur	2003
1961	0	0	0	1718	Arystanbekkaparov	2003
1962	0	0	0	1718	Maleksharaf	2004
1963	0	0	0	1718	Zachariaszrytelewski	2004
1964	0	0	0	1718	Noahwheen	2003
1965	0	0	0	1718	Wenbobai	2007
1966	0	0	0	1728	Boscolee	2007
1967	0	0	0	1728	Ethansilva	2003
1968	0	0	0	1728	Kittinaeb iem	2004
1969	0	0	0	1728	Franmitrovic	2003
1970	0	0	0	1728	Robertbauer	2003
1971	0	0	0	1728	Nikolakolyachev	2003
1972	0	0	0	1728	Patrykstankiewicz	2004
1973	0	0	0	1728	Thomasgerbaud	2004
1974	0	0	0	1728	Krishanhooda	2003
1975	0	0	0	1728	Filipkana	2004
1976	0	0	0	1728	Adamduan	2003
1977	0	0	0	1728	Agustinbaroni	2004
1978	0	0	0	1728	Edvardssnepsts	2003
1979	0	0	0	1728	Hurshvardhansingh	2006
1980	0	0	0	1728	Tomascortez	2005
1981	0	0	0	1728	Paulcolin	2003
1982	0	0	0	1728	Carsonbaker	2005
1983	0	0	0	1745	Tomaslopez salazar	2003
1984	0	0	0	1745	Rodrigosanchez hurtado	2003
1985	0	0	0	1747	Mikapetkovic	2006
1986	0	0	0	1747	Jakubnovak	2003
1987	0	0	0	1747	Cooperwoestendick	2006
1988	0	0	0	1747	Willemroosenschoon	2003
1989	0	0	0	1751	Hiteshchauhan	2007
1990	0	0	0	1751	Enzolima	2003
1991	0	0	0	1753	Danielwessels	2003
1992	0	0	0	1753	Mateo Luisalvarez sarmiento	2005
1993	0	0	0	1755	Benjaminpoblete martinez	2003
1994	0	0	0	1755	Adrianluddeckens	2003
1995	0	0	0	1757	Aaronrittberger	2003
1996	0	0	0	1758	Gabrielticona sainz	2006

1997	0	0	0	1758	Boracankaya	2006
1998	0	0	0	1758	samuelorellana orellana	2004
1999	0	0	0	1758	Mathisjouanneau	2004
2000	0	0	0	1762	Eliaspeter	2003
2001	0	0	0	1763	Leonnikolic	2003
2002	0	0	0	1763	Alexandervisser	2004
2003	0	0	0	1763	Luckydanjuma	2007
2004	0	0	0	1763	Tomgonindard	2003
2005	0	0	0	1763	Kaimilburn	2007
2006	0	0	0	1763	Einarikilpinen	2004
2007	0	0	0	1763	Gabrielelicha navas	2003
2008	0	0	0	1770	Oscar Gabrielmorales barrera	2005
2009	0	0	0	1771	Bernardoperalta	2003
2010	0	0	0	1772	Jamesbeaven	2004
2011	0	0	0	1773	Maximmichaels	2005
2012	0	0	0	1773	Abrahamortiz	2003
2013	0	0	0	1773	Joshuakhoo	2005
2014	0	0	0	1776	Tomnoltorp	2006
2015	0	0	0	1777	Maramatshoayombekov	2004
2016	0	0	0	1778	PARSAfakhrara	2004
2017	0	0	0	1779	Yannickmaser	2004
2018	0	0	0	1779	Jaydenpakkiri	2005
2019	0	0	0	1779	Andreaschapides	2003
2020	0	0	0	1782	Jun Hwanlee	2003
2021	0	0	0	1782	Borrisekani essono	2004
2022	0	0	0	1782	Venkat Rishibatlanki	2007
2023	0	0	0	1782	Jovansinadinovic	2003
2024	0	0	0	1786	Dan Vasileduta	2004
2025	0	0	0	1786	Oskaripaldanius	2007
2026	0	0	0	1786	Fryderyklechno-wasiutynski	2005
2027	0	0	0	1789	Maxalopaeus	2005
2028	0	0	0	1790	Theomottier	2005
2029	0	0	0	1791	Leocohen-bacrie	2006
2030	0	0	0	1791	Jonathanatana onana	2003
2031	0	0	0	1791	Taichiwakamatsu	2006
2032	0	0	0	1791	Alexanderskripaev	2005
2033	0	0	0	1791	Harryleruth	2005
2034	0	0	0	1791	Oskarstumpf	2003
2035	0	0	0	1791	Bricepatoux	2004
2036	0	0	0	1791	Gabriel Eduardotorres arevalo	2004
2037	0	0	0	1791	Janklimas	2006
2038	0	0	0	1791	Dujemarkovina	2007
2039	0	0	0	1791	Jonathanatana onana	2003
2040	0	0	0	1802	Bryan Lap Yinhui	2005
2041	0	0	0	1803	Axelreich pena	2004
2042	0	0	0	1804	Adamibrahim	2003
2043	0	0	0	1804	Dong Hwankim	2003
2044	0	0	0	1806	Marcelungerboeck	2004
2045	0	0	0	1806	Cristianmarton	2005
2046	0	0	0	1808	Bojanradoman	2003

2047	0	0	0	1808	Gautams venkatraman	2007
2048	0	0	0	1808	Arnavnemali	2005
2049	0	0	0	1808	Karimbennani	2007
2050	0	0	0	1808	Danielshemesh	2003
2051	0	0	0	1813	Lars Goranverwerft	2003
2052	0	0	0	1814	Iakovromanovich	2003
2053	0	0	0	1815	Grovervargas fernandez	2004
2054	0	0	0	1816	Viktorboban	2004
2055	0	0	0	1817	Ignacioruiz marquinez	2007
2056	0	0	0	1817	Theodornystrom	2004
2057	0	0	0	1817	Lameckbagerbaseh	2006
2058	0	0	0	1817	Umitsarifi	2005
2059	0	0	0	1821	Chaserobinson	2004
2060	0	0	0	1821	Abdullah Amr Mohamedgenedy	2003
2061	0	0	0	1821	Benediktszerencsits	2005
2062	0	0	0	1821	Rainer Erikschestak	2006
2063	0	0	0	1821	Iliyaavramov	2004
2064	0	0	0	1821	Brianbozemoj	2003
2065	0	0	0	1821	Tristanflander	2004
2066	0	0	0	1821	Justinlyons	2004
2067	0	0	0	1821	Yujiroonuma	2004
2068	0	0	0	1821	Ettore Romeobaggio danesi	2006
2069	0	0	0	1831	francescode martinez	2005
2070	0	0	0	1831	ELISHA FOLARINolowogbile	2003
2071	0	0	0	1831	Ho Wangleung	2004
2072	0	0	0	1831	Teanobalducci	2005
2073	0	0	0	1831	Kohshiishibashi	2007
2074	0	0	0	1831	Efe Boranguler	2004
2075	0	0	0	1831	Sebastianococola	2006
2076	0	0	0	1838	Egorgorin	2007
2077	0	0	0	1839	Oliverhadley	2004
2078	0	0	0	1840	Sebastiancastro loedel	2004
2079	0	0	0	1840	Marcusmasarcin	2004
2080	0	0	0	1840	Sachaouzilou	2003
2081	0	0	0	1843	Agustingaia	2004
2082	0	0	0	1844	Andriyporitskyy	2005
2083	0	0	0	1845	Mateoquintero castillo	2003
2084	0	0	0	1845	Felipepagnacco	2005
2085	0	0	0	1845	Alejandroraffo	2003
2086	0	0	0	1845	jungminhwang	2004
2087	0	0	0	1845	Aarondawson	2004
2088	0	0	0	1845	Kossi Andredogbefu	2005
2089	0	0	0	1845	Jinpengtang	2006
2090	0	0	0	1852	Noaheisenberg	2003
2091	0	0	0	1852	Zamanimoyo	2003
2092	0	0	0	1854	Denismaijorov	2005
2093	0	0	0	1855	Pierremouesca	2004
2094	0	0	0	1855	Jaspercamehn	2006
2095	0	0	0	1855	Cameron Austinchang	2003
2096	0	0	0	1858	Mimaltmerjou	2004

2097	0	0	0	1858	Mateogalvez	2005
2098	0	0	0	1860	Seon Junji	2004
2099	0	0	0	1860	RYOmurakami	2004
2100	0	0	0	1862	Kar Longng	2003
2101	0	0	0	1862	Mattponchet	2004
2102	0	0	0	1862	Andre-Remijakobi	2003
2103	0	0	0	1865	Andrejdjokic	2003
2104	0	0	0	1865	Georgstrasch	2006
2105	0	0	0	1865	Morganesegodo	2004
2106	0	0	0	1865	Matthewdubas	2006
2107	0	0	0	1865	Filippomazzola	2005
2108	0	0	0	1870	Carlos Adrianalcala alarcon	2003
2109	0	0	0	1870	Gregoriode gasper	2004
2110	0	0	0	1870	Eyad Reda Ezzatmohamed sher	2008
2111	0	0	0	1870	Artemkhuda	2005
2112	0	0	0	1870	Hassanbaroudi	2003
2113	0	0	0	1870	Luis Octaviobulcao	2004
2114	0	0	0	1870	Gauthierprince	2004
2115	0	0	0	1877	Raphaelvaksmann	2006
2116	0	0	0	1878	Egetopkoc	2006
2117	0	0	0	1879	Samuellockett	2005
2118	0	0	0	1879	Eliasanwar	2004
2119	0	0	0	1879	Samuelborjesson	2005
2120	0	0	0	1882	Arturyurkevich	2005
2121	0	0	0	1882	Alfrendovogelaar	2004
2122	0	0	0	1884	Rodolfocuevas guerrero	2005
2123	0	0	0	1884	sahilprakash	2006
2124	0	0	0	1884	Thomasbevan	2005
2125	0	0	0	1887	Markomilosavljevic	2004
2126	0	0	0	1887	Shonmor	2005
2127	0	0	0	1887	Alaatrifi	2007
2128	0	0	0	1890	Louaymakke	2006
2129	0	0	0	1890	Silhollemans	2003
2130	0	0	0	1890	Sachamaes	2003
2131	0	0	0	1893	Roccopiatti	2004
2132	0	0	0	1893	Gianlucacarlini	2005
2133	0	0	0	1895	Danielmarincas	2005
2134	0	0	0	1895	Mateuszlange	2006
2135	0	0	0	1895	Gakuokuma	2005
2136	0	0	0	1895	Vitalybelanov	2004
2137	0	0	0	1899	Santiagoserrano huidobro	2005
2138	0	0	0	1899	Mustafasancaklioglu	2004
2139	0	0	0	1899	Mustafasancaklioglu	2004
2140	0	0	0	1902	Dorianjuszczak	2005
2141	0	0	0	1902	Ahmedelkarmoty	2006
2142	0	0	0	1902	Alvaropeiro serrano	2004
2143	0	0	0	1902	Denisivenin	2006
2144	0	0	0	1902	Wynnshioi	2005
2145	0	0	0	1902	Troydelgado	2004
2146	0	0	0	1908	Christopher Robinrootare	2004

2147	0	0	0	1908	Malharrajpal	2004
2148	0	0	0	1908	Brendanloh	2006
2149	0	0	0	1908	Patriciomedrano	2004
2150	0	0	0	1912	Pawatpunnaket	2004
2151	0	0	0	1912	Aril Kayratuna	2006
2152	0	0	0	1912	Aleksamisic	2004
2153	0	0	0	1915	Mariocazares tipan	2004
2154	0	0	0	1915	Alexandermirkov	2005
2155	0	0	0	1915	Nilbibard grau	2004
2156	0	0	0	1915	Jordan Georgegeorgiev	2006
2157	0	0	0	1915	Pedroalmeida melli	2004
2158	0	0	0	1915	Irakliskalanov	2007
2159	0	0	0	1915	Hirainagashima	2005
2160	0	0	0	1915	Jakubsvoboda	2003
2161	0	0	0	1915	Kiruthik Kalyaanannur senthil k	2003
2162	0	0	0	1915	Nourfathalla	2006
2163	0	0	0	1925	Razaghaus	2003
2164	0	0	0	1925	Juan Luisreding	2006
2165	0	0	0	1925	Etiennelee	2005
2166	0	0	0	1925	Alonsoroca	2005
2167	0	0	0	1925	Rodrigovarillas	2006
2168	0	0	0	1925	Marcelomifano pinto	2004
2169	0	0	0	1925	Ferdinandkloesters	2005
2170	0	0	0	1925	Mickaelkaouk	2008
2171	0	0	0	1925	Artturonkainen	2005
2172	0	0	0	1925	Louis-Loulangenaken	2006
2173	0	0	0	1925	Filippoverdese	2004
2174	0	0	0	1925	Mathisseidl	2005
2175	0	0	0	1937	abdelrhmanattalla	2006
2176	0	0	0	1937	Rafaelgracie	2006
2177	0	0	0	1937	Milosvujasinovic	2003
2178	0	0	0	1937	Kylemckay	2005
2179	0	0	0	1941	Valtterivare	2003
2180	0	0	0	1941	Michalosomanski	2003
2181	0	0	0	1941	Jacobibain	2003
2182	0	0	0	1941	Victorpaganetti	2003
2183	0	0	0	1945	Eduard Silviuschipor	2004
2184	0	0	0	1945	Matthewcizmarik	2006
2185	0	0	0	1947	Alexpadro parra	2004
2186	0	0	0	1947	Victorkimpel	2006
2187	0	0	0	1947	Nathantrouve	2006
2188	0	0	0	1947	Tarmrojanasoonthon	2003
2189	0	0	0	1947	Jackchapman	2006
2190	0	0	0	1947	Luccalafrance	2005
2191	0	0	0	1947	Anargyrospanagiotou	2006
2192	0	0	0	1947	Mateoleon quinones	2005
2193	0	0	0	1947	Alessiovasquez gehrke	2003
2194	0	0	0	1947	Rometmoosar	2005
2195	0	0	0	1947	Lukabutera	2004
2196	0	0	0	1947	Agustinyap binet	2003

2197	0	0	0	1947	Stewartaronson	2005
2198	0	0	0	1947	Gabriele Thomasbrancatelli	2003
2199	0	0	0	1947	Berktunali	2003
2200	0	0	0	1947	Henrihaupt	2006
2201	0	0	0	1947	Alihatem	2007
2202	0	0	0	1947	Sebastianmedica sandoval	2005
2203	0	0	0	1947	Javierguzman	2003
2204	0	0	0	1947	Andresrodriguez	2003
2205	0	0	0	1947	Zakvince	2003
2206	0	0	0	1968	Alecgaudin	2004
2207	0	0	0	1968	Vatsalrukhaiyar	2004
2208	0	0	0	1968	Gustavodel porto rubio	2004
2209	0	0	0	1968	Oscarbaumgartner	2006
2210	0	0	0	1968	Achilefs Rafailkousaridis	2005
2211	0	0	0	1973	Oryanzohar	2003
2212	0	0	0	1973	Jakubwlodarczyk	2005
2213	0	0	0	1975	Ilyasavelev	2005
2214	0	0	0	1975	Gustavo Adolfopacheco	2003
2215	0	0	0	1977	Lwazingwenya	2004
2216	0	0	0	1977	Juan pablolua llamas	2004
2217	0	0	0	1977	Diegoandrade padilla	2005
2218	0	0	0	1977	Hassanammar	2006
2219	0	0	0	1977	Ianmayew	2006
2220	0	0	0	1977	Ali Sherifahmed	2005
2221	0	0	0	1983	Christian Seanwong	2006
2222	0	0	0	1983	Diegomadrigal	2005
2223	0	0	0	1983	Raphael Niiankrah	2006
2224	0	0	0	1983	Aleksandarnikolov	2004
2225	0	0	0	1983	Anastasismandalenakis	2004
2226	0	0	0	1983	Loicyoung	2005
2227	0	0	0	1989	Maximcazac	2004
2228	0	0	0	1989	Yarinaebi	2003
2229	0	0	0	1989	ABUBAKARyusuf	2005
2230	0	0	0	1989	Kaitonelson	2005
2231	0	0	0	1989	Alexanderkarman	2003
2232	0	0	0	1989	Giacomocrisostomo	2003
2233	0	0	0	1989	Artempugach	2005
2234	0	0	0	1989	Stephenslocombe	2003
2235	0	0	0	1989	Carlo Albertocaniato	2005
2236	0	0	0	1989	Filipstojanovski	2006
2237	0	0	0	1989	Fredrik Kroghsolheim	2006
2238	0	0	0	1989	Paolorosati	2003
2239	0	0	0	1989	Artempugach	2005
2240	0	0	0	1989	Alexanderkarman	2003
2241	0	0	0	2003	Gianmariamigliardi	2004
2242	0	0	0	2003	Philiporloff	2005
2243	0	0	0	2003	Mohamed Nasserabouzied	2007
2244	0	0	0	2003	Belalelsharkawy	2005
2245	0	0	0	2003	Steyndippenaar	2003
2246	0	0	0	2003	Mikhailrimskii	2004

2247	0	0	0	2003	Timotheelustig	2007
2248	0	0	0	2010	Danilaterebkov	2004
2249	0	0	0	2010	Ahmedmohamed	2003
2250	0	0	0	2010	Maxymkulish	2005
2251	0	0	0	2013	Juan Daviddiaz rincon	2005
2252	0	0	0	2013	Ilyaignatov	2005
2253	0	0	0	2013	Jaidutta	2006
2254	0	0	0	2013	Loucabertone	2003
2255	0	0	0	2013	Ginorondon	2005
2256	0	0	0	2013	Drisssedrati	2005
2257	0	0	0	2013	Luismarquez	2004
2258	0	0	0	2013	Eduardtsaturyan	2005
2259	0	0	0	2013	Martindoskocil	2007
2260	0	0	0	2013	hamidisrar gul	2006
2261	0	0	0	2023	Krishkarpe	2005
2262	0	0	0	2023	Rushikeshsonawane	2008
2263	0	0	0	2025	Mattiaricci	2005
2264	0	0	0	2025	Ivangoncharov	2005
2265	0	0	0	2025	ZhengQingji	2005
2266	0	0	0	2025	Hon Chunlee	2003
2267	0	0	0	2025	Felixbalshaw	2006
2268	0	0	0	2025	Danielwatson	2004
2269	0	0	0	2031	Kylepiqueras	2006
2270	0	0	0	2031	Artemmaltsev	2004
2271	0	0	0	2033	Kai-Lucaampaw	2006
2272	0	0	0	2033	Ethancarr	2004
2273	0	0	0	2033	Arjungohad	2004
2274	0	0	0	2036	Mengezi Denzel Awakhiwesibar	2007
2275	0	0	0	2036	Matyasriha	2004
2276	0	0	0	2038	Damianchng	2005
2277	0	0	0	2038	Youssefelshafei	2007
2278	0	0	0	2038	Jackmartuccio	2004
2279	0	0	0	2038	Adhiritawal	2005
2280	0	0	0	2038	Mawuko Francois-Xaviergakpo	2007
2281	0	0	0	2038	Lucienmichel	2004
2282	0	0	0	2038	Hiromasakoyama	2007
2283	0	0	0	2038	Masharinawaf	2007
2284	0	0	0	2038	Akashkarekatte	2004
2285	0	0	0	2038	Gryffinminor	2004
2286	0	0	0	2038	Amour Kelly Djidjohogandonou	2003
2287	0	0	0	2038	Lucianobarbarino	2005
2288	0	0	0	2038	Skandhaprasanna rao	2005
2289	0	0	0	2038	Juliusbult	2003
2290	0	0	0	2038	Hugosalmeron	2003
2291	0	0	0	2038	Hong Kiulau	2006
2292	0	0	0	2054	Tillfremeaux	2005
2293	0	0	0	2054	Rokostipetic	2003
2294	0	0	0	2054	Tywunderlich	2004
2295	0	0	0	2054	Matejagojkovic	2005
2296	0	0	0	2054	Albertoaltur fernandez	2005

2297	0	0	0	2054	Ianbracks	2006
2298	0	0	0	2054	Johannrakotomaharo	2003
2299	0	0	0	2054	Jontygiesen	2006
2300	0	0	0	2054	Matejmuransky	2004
2301	0	0	0	2054	Martindelnido	2005
2302	0	0	0	2064	Fortunejoseph	2005
2303	0	0	0	2064	Ryusukeinuzuka	2005
2304	0	0	0	2064	JOAQUINflores	2005
2305	0	0	0	2064	Howardchan	2007
2306	0	0	0	2064	Yoavlevy	2003
2307	0	0	0	2069	Luisutrilla	2004
2308	0	0	0	2069	Joel Markgibson	2004
2309	0	0	0	2071	Min Jiechua	2007
2310	0	0	0	2071	Arda Edizgul	2004
2311	0	0	0	2071	Jeong-Ankim	2003
2312	0	0	0	2071	Davidreutlinger	2005
2313	0	0	0	2071	Axelhuysmans	2006
2314	0	0	0	2071	Iannismiletich	2005
2315	0	0	0	2071	Abraariqbal	2003
2316	0	0	0	2071	Ilgizkamchibekov	2004
2317	0	0	0	2079	Alexandervlahos	2006
2318	0	0	0	2079	Keeganrice	2006
2319	0	0	0	2079	Michaelcaspanello	2004
2320	0	0	0	2079	Antoninorattenni	2004
2321	0	0	0	2079	Amitshahar	2005
2322	0	0	0	2084	Maxhein	2005
2323	0	0	0	2084	Moritzlesjak	2005
2324	0	0	0	2084	Kyubinsakong	2004
2325	0	0	0	2087	Lawrenceivanow	2005
2326	0	0	0	2087	Leonardtramolay	2005
2327	0	0	0	2087	Sergioramirez	2005
2328	0	0	0	2087	Benjamin Ignacioherrera deluc	2003
2329	0	0	0	2087	Mart Jankaak	2004
2330	0	0	0	2087	Valdemardubin	2005
2331	0	0	0	2087	Damirabdusamadov	2006
2332	0	0	0	2087	Patrickfindlay	2004
2333	0	0	0	2087	Ramiro Nicolaschristensen	2005
2334	0	0	0	2087	Pavlorezoglazov	2004
2335	0	0	0	2097	Hlibkovalenko	2007
2336	0	0	0	2097	Danilmulintsev	2004
2337	0	0	0	2097	Eyaltoledano	2004
2338	0	0	0	2097	Ryancarter	2004
2339	0	0	0	2097	Ryancarter	2004
2340	0	0	0	2097	Eyaltoledano	2004
2341	0	0	0	2097	Ethangaubert	2003
2342	0	0	0	2104	Victormayer	2006
2343	0	0	0	2104	Razvanaghion	2003
2344	0	0	0	2104	Kelyanbrinon	2004
2345	0	0	0	2104	Colecozens	2005
2346	0	0	0	2104	Wenzelgraski	2003

2347	0	0	0	2104	Pierre-Alexandreboivin	2005
2348	0	0	0	2104	Matthewovervelde	2005
2349	0	0	0	2111	Stjepanivanek	2004
2350	0	0	0	2111	Erikschiessl	2004
2351	0	0	0	2111	Cowenlau	2005
2352	0	0	0	2111	Thaddeussim	2006
2353	0	0	0	2111	Daniilzhibul	2003
2354	0	0	0	2111	Tomasallen	2004
2355	0	0	0	2111	Jakeriordan	2004
2356	0	0	0	2111	Chanathornkangtong	2003
2357	0	0	0	2111	Williamrejchtman vinciguerra	2007
2358	0	0	0	2111	Jordangancho	2003
2359	0	0	0	2111	Tylerwaddock	2004
2360	0	0	0	2111	Ethan Matthewkoh	2008
2361	0	0	0	2111	Jobjapelj	2004
2362	0	0	0	2111	Tiago Valentinpszybylski	2005
2363	0	0	0	2111	RONAN TASHINGAmtisi	2007
2364	0	0	0	2111	Ethankhun	2006
2365	0	0	0	2111	Aadarshtripathi	2003
2366	0	0	0	2111	Chrisspyrou	2006
2367	0	0	0	2111	Boburkhonturgunov	2003
2368	0	0	0	2111	Kristiankjellberg	2005
2369	0	0	0	2111	Francisco Joserossi	2003
2370	0	0	0	2111	Kyleerwee	2005
2371	0	0	0	2111	Philippbosse	2004
2372	0	0	0	2111	Dong Kyulee	2005
2373	0	0	0	2111	Abhaychhabra	2004
2374	0	0	0	2111	Amir Mahdifarokhpour	2007
2375	0	0	0	2111	Estebanortiz	2005
2376	0	0	0	2111	Fabriciososa pons	2003
2377	0	0	0	2111	Dante Carlosteramo	2004
2378	0	0	0	2111	Maitamunyimani	2008
2379	0	0	0	2111	Felix Chun Heilam	2007
2380	0	0	0	2111	Marlon Fabrizzioleon eguizabal	2004
2381	0	0	0	2111	Josephblack	2006
2382	0	0	0	2111	Herrick Thomaslegaspi	2004
2383	0	0	0	2111	Aleksandardaskalovic	2006
2384	0	0	0	2111	kokishibata	2003
2385	0	0	0	2111	Marcboada subirana	2003
2386	0	0	0	2111	Yassinelaroussy	2006
2387	0	0	0	2111	Antoinehernandez	2004
2388	0	0	0	2111	Maximilianontiveros	2006
2389	0	0	0	2111	tatwong	2005
2390	0	0	0	2111	Omarverduzco	2005
2391	0	0	0	2111	SAMUELallais	2003
2392	0	0	0	2111	Marcsoriano sanchez	2004
2393	0	0	0	2111	Adambojkovic	2005
2394	0	0	0	2111	WEPIA Narcisse Andrebessebro	2006
2395	0	0	0	2111	Diego Eduardomunoz navia	2006
2396	0	0	0	2111	Mukilramanan	2003

2397	0	0	0	2111	Songrit Davidchanbubpha	2003
2398	0	0	0	2111	Devanshpunjabi	2007
2399	0	0	0	2111	Simonbachler pacheco	2004
2400	0	0	0	2111	Takuyakobayashi	2004
2401	0	0	0	2111	Nahuelfernandez	2003
2402	0	0	0	2111	Adelghanim	2005
2403	0	0	0	2111	cristobalvalenzuela	2005
2404	0	0	0	2111	Teetawattavachphongsri	2007
2405	0	0	0	2111	Emanuel Graukristensen	2007
2406	0	0	0	2111	Ibrahimkandil	2005
2407	0	0	0	2111	Yukiasai	2005
2408	0	0	0	2111	Arthurattrill	2007
2409	0	0	0	2171	Francky Martialhoimian	2006
2410	0	0	0	2171	Gunukkang	2004
2411	0	0	0	2171	Chang Hunlee	2003
2412	0	0	0	2171	Ryanwatt	2006
2413	0	0	0	2171	Antoniovoljavec	2006
2414	0	0	0	2171	ADAMsun	2006
2415	0	0	0	2171	Tobeylock	2004
2416	0	0	0	2171	Leonschmidt	2005
2417	0	0	0	2171	Christosglavas	2006
2418	0	0	0	2171	Sarvarazamatov	2006
2419	0	0	0	2171	Julappagari Anuragreddy	2003
2420	0	0	0	2171	Alex Santinonunez vera	2007
2421	0	0	0	2171	Robengavani	2007
2422	0	0	0	2171	Rudyquan	2006
2423	0	0	0	2171	Angelvladov	2006
2424	0	0	0	2171	Luis Eduardoarellano rojas	2005
2425	0	0	0	2171	Constantinradu	2006
2426	0	0	0	2171	Ivanshchepilov	2003
2427	0	0	0	2171	Yankodjoed	2005
2428	0	0	0	2171	Romankharlamov	2007
2429	0	0	0	2171	Ryanaugusto dos santos	2003
2430	0	0	0	2171	Joannadal vives	2004
2431	0	0	0	2171	Dan Iulianpasca	2003
2432	0	0	0	2171	Maksimlavygin	2005
2433	0	0	0	2171	Nielsvillard	2005
2434	0	0	0	2171	Harutasumi	2004
2435	0	0	0	2171	Artemiykukushkin	2004
2436	0	0	0	2198	Devkanbargimath	2007
2437	0	0	0	2198	Nicolasgarnier	2006
2438	0	0	0	2198	Filipstidell	2005
2439	0	0	0	2198	Filipstidell	2005
2440	0	0	0	2202	Joaomaia	2004
2441	0	0	0	2202	Stylianospapamichael	2005
2442	0	0	0	2202	Thomasgiles	2005
2443	0	0	0	2202	Pabloharo maya	2004
2444	0	0	0	2206	Georgiobidan	2008
2445	0	0	0	2206	Adhamghobashy	2005
2446	0	0	0	2206	Viktorkirov	2006

2447	0	0	0	2206	Nikolamarinov	2006
2448	0	0	0	2206	Sergeypetrov	2005
2449	0	0	0	2206	Nickyousefian	2006
2450	0	0	0	2206	Simonnorrman	2005
2451	0	0	0	2206	Brunoloeffler	2003
2452	0	0	0	2206	Noahbrownrigg	2006
2453	0	0	0	2206	Alexlindqvist	2003
2454	0	0	0	2206	Guillermo Alfonsobennaton	2005
2455	0	0	0	2206	Ibrahim Furkandeniz	2005
2456	0	0	0	2206	Leonidpoliakov	2004
2457	0	0	0	2206	Omaansh Choudharysaharia	2006
2458	0	0	0	2206	Augustin Maximemiramontes	2005
2459	0	0	0	2206	Ezequielarce gonzalez	2003
2460	0	0	0	2206	Masonrobins	2004
2461	0	0	0	2206	Aryannanda	2006
2462	0	0	0	2206	Imrankhaitbaev	2006
2463	0	0	0	2206	Williamclifford	2004
2464	0	0	0	2206	Maurocupelli	2005
2465	0	0	0	2227	Simon Andresojeda viloria	2004
2466	0	0	0	2227	Tizianotocon garcia	2004
2467	0	0	0	2227	Suleimanibrahim	2005
2468	0	0	0	2227	Sampidoux	2006
2469	0	0	0	2231	Dhanunaiphukesorn	2006
2470	0	0	0	2231	Carlloeffler	2007
2471	0	0	0	2231	Danieljauss	2004
2472	0	0	0	2231	Erkutaydin	2006
2473	0	0	0	2231	Baransivasli	2006
2474	0	0	0	2231	Richard Nii Okaiokine	2004
2475	0	0	0	2231	Joaobenvenutti	2004
2476	0	0	0	2231	Malagushi	2004
2477	0	0	0	2231	Breno Rossettourvaneja	2004
2478	0	0	0	2231	Omarmounir	2006
2479	0	0	0	2231	Krisjanissulcs	2005
2480	0	0	0	2231	Dominikvonbank	2004
2481	0	0	0	2243	Gregorgottein	2005
2482	0	0	0	2243	Andrejradojicic	2004
2483	0	0	0	2243	Arielbouskila	2004
2484	0	0	0	2243	Ferditangal	2004
2485	0	0	0	2243	Albertopuello	2006
2486	0	0	0	2248	Sampazoki	2007
2487	0	0	0	2248	Fedorsosedkin	2006
2488	0	0	0	2248	Andrejrajak	2005
2489	0	0	0	2248	Santiagomuhala	2006
2490	0	0	0	2248	Kobediaz	2004
2491	0	0	0	2248	Albertwidell	2006
2492	0	0	0	2248	Ivanmezentsev	2005
2493	0	0	0	2248	Loagosaleshando	2003
2494	0	0	0	2256	Ishmael Nii Norteydowuona	2004
2495	0	0	0	2256	Albertsegura onichtchenko	2003
2496	0	0	0	2256	Juan Joseycaza amador	2003

2497	0	0	0	2256	Lucianopardo	2003
2498	0	0	0	2256	Majnajvirt kolaric	2003
2499	0	0	0	2256	Louisvanhuyse	2005
2500	0	0	0	2256	Punyaenagpal	2006
2501	0	0	0	2256	Oleksandrponomar	2006
2502	0	0	0	2264	Tassoalves moreira	2003
2503	0	0	0	2264	Adamdellala	2005
2504	0	0	0	2264	Akbarshokhshukurov	2003
2505	0	0	0	2264	Adamgestinger	2003
2506	0	0	0	2264	Danylorekun	2005
2507	0	0	0	2264	Benoittorcq	2005
2508	0	0	0	2264	Konstantinshauraks	2005
2509	0	0	0	2264	Bilalasim	2006
2510	0	0	0	2264	Antoniorodriguez rodriguez	2004
2511	0	0	0	2264	Vishalgautam	2003
2512	0	0	0	2264	Raghavharsh	2006
2513	0	0	0	2264	Erkinraimov	2005
2514	0	0	0	2264	Villemaadamsoo	2005
2515	0	0	0	2264	kartiksaxena	2003
2516	0	0	0	2264	Amirrezaarmioun	2004
2517	0	0	0	2264	Gabrielpiqueras	2006
2518	0	0	0	2264	Drikusde jager	2005
2519	0	0	0	2264	Danielblumaier	2005
2520	0	0	0	2282	Filipmarinkovic	2005
2521	0	0	0	2282	Davidminnivaliev	2003
2522	0	0	0	2282	Andreybeketov	2003
2523	0	0	0	2282	Lucasfoix	2004
2524	0	0	0	2282	Jeong Yukim	2004
2525	0	0	0	2282	Federicoscotuzzi	2004
2526	0	0	0	2288	Taymurmuhammad	2006
2527	0	0	0	2288	Arjunshah	2003
2528	0	0	0	2288	ATICHAPHATcharatviroch	2006
2529	0	0	0	2288	Natthachaimultha	2004
2530	0	0	0	2288	Danismijatovic	2005
2531	0	0	0	2288	Maximmrva	2007
2532	0	0	0	2288	Jurajobtulovic	2004
2533	0	0	0	2288	Vihaaniyer	2005
2534	0	0	0	2296	Dmitriyparintsev	2004
2535	0	0	0	2296	Prajwaltewari	2005
2536	0	0	0	2296	Julianplatzer	2006
2537	0	0	0	2296	Arthusde la bassetiere	2003
2538	0	0	0	2296	Milesphijidvijan	2008
2539	0	0	0	2296	Arthusde la bassetiere	2003
2540	0	0	0	2296	Briancapalnean	2004
2541	0	0	0	2296	Brycemurphy	2004
2542	0	0	0	2296	Andrekock	2003
2543	0	0	0	2296	Leonberg	2003
2544	0	0	0	2296	Winstonzhang	2005
2545	0	0	0	2296	Sviatoslavkoliadynskyi	2006
2546	0	0	0	2296	Zakariaachour	2005

2547	0	0	0	2296	Milesphijidvijan	2008
2548	0	0	0	2296	Julianplatzer	2006
2549	0	0	0	2296	Kristianthomas	2005
2550	0	0	0	2296	Rudythorogood	2005
2551	0	0	0	2313	Andresde los rios kolejewska	2004
2552	0	0	0	2313	Napathuadcharoen	2005
2553	0	0	0	2313	Leostenlund	2006
2554	0	0	0	2313	Willroberts	2005
2555	0	0	0	2313	Arseniynazarov	2004
2556	0	0	0	2313	Bryantmorales	2005
2557	0	0	0	2313	Arnavyadav	2005
2558	0	0	0	2313	Kacperwereszka	2003
2559	0	0	0	2313	Simeonlatinchev	2004
2560	0	0	0	2313	Davidtotikashvili	2004
2561	0	0	0	2313	Arjunpremkumar	2004
2562	0	0	0	2313	Tomchapman	2006
2563	0	0	0	2313	Gabriel Augustocarvalho	2004
2564	0	0	0	2313	Antonioverno	2004
2565	0	0	0	2313	Juliuskostopoulos	2003
2566	0	0	0	2313	Nithilan Erickinattuvilai	2004
2567	0	0	0	2313	Rafaelzard	2006
2568	0	0	0	2313	Oscar Fernandotrujillo hernand	2004
2569	0	0	0	2313	Bogdanzakharov	2004
2570	0	0	0	2313	Filipegrebentsov	2004
2571	0	0	0	2313	Anupbangargi	2003
2572	0	0	0	2313	Kazumakimura	2006
2573	0	0	0	2313	Haithemrabbouche	2004
2574	0	0	0	2313	Gianluca Valentinobocanegra r	2005
2575	0	0	0	2313	Egorkrivenyshev	2003
2576	0	0	0	2313	Raahimagha	2004
2577	0	0	0	2313	Yessinekerouat	2005
2578	0	0	0	2313	Nikolacvetanovic	2003
2579	0	0	0	2313	Raffaelebarba	2004
2580	0	0	0	2313	Julientremolieres	2005
2581	0	0	0	2313	Fresksylhasi	2004
2582	0	0	0	2313	Sami Zebkhan	2004
2583	0	0	0	2313	Ahtialkio	2003
2584	0	0	0	2313	Edoardolanza cariccio	2004
2585	0	0	0	2313	YAHAYAsani inabo	2007
2586	0	0	0	2313	Chika Janez Calebnwaozuzu	2006
2587	0	0	0	2349	Maximkhorozov	2003
2588	0	0	0	2349	Egorpleshivtsev	2007
2589	0	0	0	2349	Paoloangeli	2007
2590	0	0	0	2349	Duartemestre	2005
2591	0	0	0	2349	Teunmantel	2006
2592	0	0	0	2349	Anthonycater	2003
2593	0	0	0	2349	Roccovalente	2004
2594	0	0	0	2349	Trisztangubi	2005
2595	0	0	0	2349	Federicopieckenstainer	2004
2596	0	0	0	2349	Kornelhalmos	2006

2597	0	0	0	2349	ARNAVkapoor	2004
2598	0	0	0	2349	Robin Maximustrono	2005
2599	0	0	0	2349	Jose Mariomarquez	2004
2600	0	0	0	2349	Lukasrieber	2004
2601	0	0	0	2349	Chasefralick	2005
2602	0	0	0	2349	Alexandroszervos	2003
2603	0	0	0	2349	Connorsmillie	2004
2604	0	0	0	2349	Pietro Augustolavoratori	2004
2605	0	0	0	2349	Ho Wanli	2006
2606	0	0	0	2349	Lukasmarjanovic	2006
2607	0	0	0	2349	Fabriziovillegas villarroel	2003
2608	0	0	0	2349	Vidmohar	2006
2609	0	0	0	2349	Ethanmaan	2005
2610	0	0	0	2349	Ka Chun Oscarleung	2006
2611	0	0	0	2349	Aleksanderfranko	2007
2612	0	0	0	2349	Ethangottschalk	2005
2613	0	0	0	2349	Joaquincabello	2004
2614	0	0	0	2349	Harrisoncare	2007
2615	0	0	0	2377	Micheledi pierro	2004
2616	0	0	0	2377	Eytanmiessen	2006
2617	0	0	0	2377	Alexanderfrantzis	2005
2618	0	0	0	2377	Nicohipfl	2006
2619	0	0	0	2377	Maximusdussault	2007
2620	0	0	0	2377	Williamlevasseur	2004
2621	0	0	0	2377	Russellsoohoo	2003
2622	0	0	0	2377	Filipgustafsson	2005
2623	0	0	0	2377	Santiagocastellanos donoso	2003
2624	0	0	0	2377	Aaronmatikkala	2003
2625	0	0	0	2377	Tomraveh	2003
2626	0	0	0	2377	Peterdragar	2003
2627	0	0	0	2377	Valentinvasquez	2004
2628	0	0	0	2377	Linusolausson	2005
2629	0	0	0	2377	Patcharasuklian	2005
2630	0	0	0	2392	Lionel Berimyuynjaff	2003
2631	0	0	0	2392	Baptistelaggia	2006
2632	0	0	0	2392	Temirlanaslan	2006
2633	0	0	0	2392	Victortauson	2005
2634	0	0	0	2392	Damianpiekarz	2005
2635	0	0	0	2392	Wadehermanus	2003
2636	0	0	0	2398	Carlos Eduardocarrascosa gonz	2004
2637	0	0	0	2398	Ritviknadikude	2004
2638	0	0	0	2398	Jacksontreacy	2006
2639	0	0	0	2398	Sofyanelnaqa	2006
2640	0	0	0	2398	Carlos Eduardocarrascosa gonz	2004
2641	0	0	0	2398	Kianmohammadi	2004
2642	0	0	0	2398	Mariorichmagui	2006
2643	0	0	0	2398	Lukashansson	2003
2644	0	0	0	2398	Santiagosolares ballerstaedt	2005
2645	0	0	0	2398	Andreaspolichronopoulos	2005
2646	0	0	0	2398	Viktorvan craenenbroeck	2006

2647	0	0	0	2398	Stilesbrockett	2006
2648	0	0	0	2398	Mathisheng	2007
2649	0	0	0	2398	Oliverbonding	2007
2650	0	0	0	2398	Negadezzat	2005
2651	0	0	0	2398	Tusharmittal	2004
2652	0	0	0	2398	Arthurlaborde	2004
2653	0	0	0	2398	Egealtas	2006
2654	0	0	0	2398	Filipcebic	2003
2655	0	0	0	2398	Caheerwarik	2006
2656	0	0	0	2398	Furkankaynarca	2005
2657	0	0	0	2398	Kazumasamatsuda	2005
2658	0	0	0	2398	Chi Wang Jerrycheng	2004
2659	0	0	0	2398	Lucadobronauteanu	2003
2660	0	0	0	2398	Vandadgheibi	2003
2661	0	0	0	2398	ABUBAKARaliyu	2005
2662	0	0	0	2398	Thiotshivhase	2003
2663	0	0	0	2398	Samuelmaia	2005
2664	0	0	0	2398	Itaivasershtein	2004
2665	0	0	0	2398	Filipkosinski	2007
2666	0	0	0	2398	Aryamanrajeev	2003
2667	0	0	0	2398	Viktorhockl	2003
2668	0	0	0	2398	Nikitapetrusenko	2003
2669	0	0	0	2398	SAIDUsani inabo	2005
2670	0	0	0	2398	Pijusreklevicius	2006
2671	0	0	0	2398	Alessiopergola	2006
2672	0	0	0	2398	Radu Davidturcanu	2006
2673	0	0	0	2398	Ritviknadikude	2004
2674	0	0	0	2398	Aldiyarabzhan	2005
2675	0	0	0	2398	Joaomartins	2004
2676	0	0	0	2398	Noahgauthier	2005
2677	0	0	0	2398	Jeffersontemgoua	2005
2678	0	0	0	2398	Miguelmoret	2004
2679	0	0	0	2398	Enzokohlmann de freitas	2006
2680	0	0	0	2398	Tzu Lichang	2003
2681	0	0	0	2398	Jose Migueluzcategui	2004
2682	0	0	0	2398	Nelsonramos ponce	2004
2683	0	0	0	2398	Eliasruusuvuori	2004
2684	0	0	0	2398	Toros Erosyakincam	2006
2685	0	0	0	2398	Lucasmock	2006
2686	0	0	0	2398	Ryotabata	2008
2687	0	0	0	2398	Tsz Fuwong	2005
2688	0	0	0	2398	Javieralonso	2006
2689	0	0	0	2398	Tomaskratochvil	2006
2690	0	0	0	2398	Kiankarimi reykandeh	2005
2691	0	0	0	2398	Gregoriohidalgo	2005
2692	0	0	0	2398	Matveygreschner	2004
2693	0	0	0	2398	Lukarupnjak	2004
2694	0	0	0	2398	Filip Gabrielbara	2005
2695	0	0	0	2398	Hugocochlin	2004
2696	0	0	0	2398	Kailashnath	2005

2697	0	0	0	2398	Yeong Junejeong	2003
2698	0	0	0	2398	Dannyyassine	2006
2699	0	0	0	2398	Daoudafallah	2006
2700	0	0	0	2398	Mathieudussaubat	2004
2701	0	0	0	2398	Seonungcho	2003
2702	0	0	0	2464	Andrei Florin Teodorrobitu	2003
2703	0	0	0	2464	Enmanuelmunoz	2005
2704	0	0	0	2464	Alejandro Javierflores natusch	2004
2705	0	0	0	2464	Connorchurch	2006
2706	0	0	0	2464	Hae Jinlee	2004
2707	0	0	0	2464	Adammuller	2004
2708	0	0	0	2464	Albertonordio	2004
2709	0	0	0	2464	Lucamindrut	2006
2710	0	0	0	2464	Nikitafiadutsik	2004
2711	0	0	0	2464	Josephmazingham	2007
2712	0	0	0	2464	Gabrielmoroder	2004
2713	0	0	0	2464	Eliashoxha	2004
2714	0	0	0	2464	Alinbutnaru	2006
2715	0	0	0	2464	Omarmorsy	2006
2716	0	0	0	2464	Teerapatkhantiweerawat	2007
2717	0	0	0	2464	Francoisvan wyk	2004
2718	0	0	0	2464	Alekseipechenkin	2004
2719	0	0	0	2464	Hasan Basaransimsek	2004
2720	0	0	0	2464	Asger Emilildor	2003
2721	0	0	0	2483	Gauthamsanthosh	2007
2722	0	0	0	2483	Lukabojicic ono	2005
2723	0	0	0	2483	Dastanzhakanger	2006
2724	0	0	0	2483	Luisbaumeister	2004
2725	0	0	0	2483	Seungikpark	2003
2726	0	0	0	2483	Jamesashworth	2005
2727	0	0	0	2483	Rayan Sajjidkoothrat	2007
2728	0	0	0	2483	Mateonavarro	2003
2729	0	0	0	2483	Danielkotka	2004
2730	0	0	0	2483	Joaquinperez casamayor	2004
2731	0	0	0	2493	Mihailbrovinschi	2004
2732	0	0	0	2493	Stjepananisic	2003
2733	0	0	0	2493	Pedrode la riva bueno	2003
2734	0	0	0	2493	antoinerenard	2004
2735	0	0	0	2493	Ivankizilov	2005
2736	0	0	0	2493	Hikarutakahashi	2007
2737	0	0	0	2493	Karloklaic	2004
2738	0	0	0	2493	Erikchobanyan	2004
2739	0	0	0	2493	Valentinwerner	2003
2740	0	0	0	2493	Edinmulalic	2005
2741	0	0	0	2493	Rares Andreigolescu	2006
2742	0	0	0	2493	Skandermezouar	2005
2743	0	0	0	2493	Pavelvysotin	2007
2744	0	0	0	2493	Miguelrotunno perretti	2004
2745	0	0	0	2493	Victorclairacq	2006
2746	0	0	0	2493	Ming Lun Arnoldcheng	2005

2747	0	0	0	2493	Erikzhakiyanov	2004
2748	0	0	0	2493	Andresjimenez	2004
2749	0	0	0	2493	Juan Arturoespanol moreno	2003
2750	0	0	0	2493	Chadvaldes	2006
2751	0	0	0	2493	Brenoferrari maciel	2004
2752	0	0	0	2493	Keldibekkozhoev	2005
2753	0	0	0	2493	Arya Ganapathykallambella	2007
2754	0	0	0	2493	Erikchobanyan	2004
2755	0	0	0	2493	Cesarotondo	2005
2756	0	0	0	2493	Eivindtandberg	2003
2757	0	0	0	2493	Radinfahimi	2008
2758	0	0	0	2493	Juan Carlosfuentes vasquez	2005
2759	0	0	0	2493	Diegorojas	2003
2760	0	0	0	2493	Tomasalvarado crexell	2005
2761	0	0	0	2493	Kirillkalennik	2006
2762	0	0	0	2493	Thomasdrayton	2003
2763	0	0	0	2493	Jose Santiagotorrez	2003
2764	0	0	0	2493	Farhaanpatrawala	2003
2765	0	0	0	2493	Eusebio Andresdiaz garcia	2004
2766	0	0	0	2493	Shepardgregory	2004
2767	0	0	0	2493	Nathanslattery	2004
2768	0	0	0	2493	Xavicalduch	2004
2769	0	0	0	2493	Noahriccitelli	2005
2770	0	0	0	2493	Nikodembarcik	2005
2771	0	0	0	2493	Enzocrevelaro	2005
2772	0	0	0	2493	Brianburke alcantara	2005
2773	0	0	0	2493	Sachaamoyel	2006
2774	0	0	0	2493	Krisharora	2006
2775	0	0	0	2493	Alejandrouribe quintero	2004
2776	0	0	0	2493	yuyaando	2007
2777	0	0	0	2493	Dominikknutson	2003
2778	0	0	0	2493	Veljkocvetkovic	2004
2779	0	0	0	2493	Ernestopico	2006
2780	0	0	0	2493	Pedropetroski rocha	2006
2781	0	0	0	2493	Hikarutakahashi	2007
2782	0	0	0	2493	Miguelngounou simo	2008
2783	0	0	0	2493	Santiagoordonez achinte	2004
2784	0	0	0	2493	Felixtazvivinga	2005
2785	0	0	0	2493	Perawatsukjai	2007
2786	0	0	0	2493	Tommasoiotti	2004
2787	0	0	0	2493	Vitorcosta goncalves	2004
2788	0	0	0	2493	Egorsergeev	2004
2789	0	0	0	2493	Alibeheshti	2003
2790	0	0	0	2493	HIROMUNEuchida	2007
2791	0	0	0	2493	Diegorodriguez	2004
2792	0	0	0	2493	Kacperkrolikowski	2005
2793	0	0	0	2493	Calebaryettey	2006
2794	0	0	0	2493	Martincimra	2004
2795	0	0	0	2493	Santiagoserdan	2004
2796	0	0	0	2493	Markomileticki	2005

2797	0	0	0	2493	Vincentvohl	2005
2798	0	0	0	2493	Kalanisavage	2005
2799	0	0	0	2493	Zyadhamed	2003
2800	0	0	0	2493	Vincenzopassariello	2003
2801	0	0	0	2493	Stjepananisic	2003
2802	0	0	0	2493	Miguel Angelsarria mondragon	2004
2803	0	0	0	2493	Armandgarabedian	2006
2804	0	0	0	2493	Andertarrio ponte	2005
2805	0	0	0	2493	Marianodedura-palomero	2005
2806	0	0	0	2493	Michelanumendem	2007
2807	0	0	0	2493	Savvakrivoshchekov	2003
2808	0	0	0	2493	antoinerenard	2004
2809	0	0	0	2493	Abdulhanan	2005
2810	0	0	0	2493	Cezar Gabrielpapoe	2007
2811	0	0	0	2493	Alessandromondazzi	2007
2812	0	0	0	2493	Rodrigofernandes	2006
2813	0	0	0	2493	Gabrielwojcicki	2005
2814	0	0	0	2493	Egormichurin	2004
2815	0	0	0	2493	Harissahman	2003
2816	0	0	0	2493	Ahurarezaee	2003
2817	0	0	0	2493	Onderbalci	2005
2818	0	0	0	2493	Keremyilmaz	2007
2819	0	0	0	2493	Santiagotrujillo garcia	2007
2820	0	0	0	2493	Jun Yeolbae	2003
2821	0	0	0	2493	Velizariliev	2005
2822	0	0	0	2493	Derin Canatacan	2005
2823	0	0	0	2493	Caydenwang	2006
2824	0	0	0	2493	Felipedreer	2006
2825	0	0	0	2493	Jasondavid	2006
2826	0	0	0	2493	Mikhailshataev	2005
2827	0	0	0	2493	Gerardodelgado	2006
2828	0	0	0	2493	Karloklaic	2004
2829	0	0	0	2493	Diego Josebsereni rebolledo	2006
2830	0	0	0	2493	Ivankizilov	2005
2831	0	0	0	2493	Jettgrech	2007
2832	0	0	0	2493	Nikolalulic	2006
2833	0	0	0	2493	Chinmay Mitulpatel	2005
2834	0	0	0	2493	Azizmoulahi	2005
2835	0	0	0	2493	Senthenreddy	2005
2836	0	0	0	2493	Andreade marchi	2007
2837	0	0	0	2493	ljubistambuk ljubi	2005
2838	0	0	0	2493	Ivailodechev	2003
2839	0	0	0	2493	Farhaanpatrawala	2003
2840	0	0	0	2493	Kacperkrolikowski	2005
2841	0	0	0	2493	Thiagoguglieri	2006
2842	0	0	0	2493	Markomileticki	2005
2843	0	0	0	2493	Brianburke alcantara	2005
2844	0	0	0	2493	Marianodedura-palomero	2005
2845	0	0	0	2493	Gerardodelgado	2006
2846	0	0	0	2493	Jacobborch-nielsen	2004

2847	0	0	0	2493	Cianmikkelsen	2004
2848	0	0	0	2493	Sachaamoyel	2006
2849	0	0	0	2493	Alibeheshti	2003
2850	0	0	0	2493	Andertarrio ponte	2005
2851	0	0	0	2493	Vitorcosta goncalves	2004
2852	0	0	0	2493	Felipedreer	2006
2853	0	0	0	2493	Santiagoserdan	2004
2854	0	0	0	2493	Chinmay Mitulpatel	2005
2855	0	0	0	2493	Armandgarabedian	2006
2856	0	0	0	2493	Nikolalulic	2006
2857	0	0	0	2493	Mehrabmalmir	2007
2858	0	0	0	2493	Rodrigofernandes	2006
2859	0	0	0	2493	Albertoleon-ponte	2004
2860	0	0	0	2493	Bernardomunk mesa	2006
2861	0	0	0	2493	Isaacyang lay	2004
2862	0	0	0	2493	Radinfahimi	2008
2863	0	0	0	2493	Leogutjahr	2005
2864	0	0	0	2493	Leonardotagliavini	2004
2865	0	0	0	2493	Brenoferrari maciel	2004
2866	0	0	0	2493	Nicolaibudkov kjaer	2006
2867	0	0	0	2629	Jose Miguelroa rodriguez	2003
2868	0	0	0	2629	Maxrichardson	2007
2869	0	0	0	2629	Christian Manuelgalarza guilart	2004
2870	0	0	0	2629	Felixdiedrichs	2004
2871	0	0	0	2629	Axeledstrom	2005
2872	0	0	0	2629	Oabonakoobatlile	2004
2873	0	0	0	2629	Dominickmosejczuk	2007
2874	0	0	0	2629	Marktaranov	2004
2875	0	0	0	2629	Nicolaspinzon moreno	2003
2876	0	0	0	2629	Hendrikkatmuk	2004
2877	0	0	0	2629	Amitweizman	2004
2878	0	0	0	2629	Rolandrisko	2003
2879	0	0	0	2629	Angushoward	2005
2880	0	0	0	2629	Filipmarinovic	2004
2881	0	0	0	2629	Michaelkokkinis	2004
2882	0	0	0	2629	Aleksandarsolak	2005
2883	0	0	0	2629	Mihneamaftei	2006
2884	0	0	0	2629	Joaogoncalves	2003
2885	0	0	0	2629	Metekoseoglu	2004
2886	0	0	0	2629	Noahpawirodirjo	2005
2887	0	0	0	2629	Alexanderokonkwo	2005
2888	0	0	0	2629	Yehorteperev	2003
2889	0	0	0	2629	Flynncaruana	2004
2890	0	0	0	2629	Tristanmesserli	2003
2891	0	0	0	2629	Carlosgamboa	2005
2892	0	0	0	2629	Derenyigin	2006
2893	0	0	0	2629	OSCARflores	2005
2894	0	0	0	2629	Riccardocampagnola	2004
2895	0	0	0	2629	Rafaelcandelas ruiz	2004
2896	0	0	0	2629	Brooklynbamburac	2005

2897	0	0	0	2629	Leonhuck	2004
2898	0	0	0	2629	Damian Alexandrucucu	2005
2899	0	0	0	2629	Jiplangerhuizen	2006
2900	0	0	0	2629	Jaovan schalkwyk	2005
2901	0	0	0	2629	Vanshnandal	2006
2902	0	0	0	2629	Finnburridge	2005
2903	0	0	0	2629	Roccomendez	2005
2904	0	0	0	2629	Pepijnlangras	2005
2905	0	0	0	2629	Xaviercalvelo	2006
2906	0	0	0	2629	Tatendamutetwa	2003
2907	0	0	0	2629	Lukasauer jimenez	2004
2908	0	0	0	2629	Kodaikato	2003
2909	0	0	0	2629	mohammadsaadeddin	2004
2910	0	0	0	2629	Oswaldo Alejandroreyes tirado	2007
2911	0	0	0	2629	Ahmedabdelaziz	2005
2912	0	0	0	2629	Pirmdhararag	2005
2913	0	0	0	2629	Arnoguillet	2006
2914	0	0	0	2629	Carlosbraun simo	2004
2915	0	0	0	2629	Lukavuglar	2004
2916	0	0	0	2629	Andyhernandez	2003
2917	0	0	0	2629	Jackcurtis	2005
2918	0	0	0	2629	Norbertcernat	2006
2919	0	0	0	2629	Markomiljanic	2003
2920	0	0	0	2629	Danielpopescu	2004
2921	0	0	0	2629	Christianceuca	2003
2922	0	0	0	2629	Leofortier-gariepy	2004
2923	0	0	0	2629	Alirezagolzad	2004
2924	0	0	0	2629	Markkneiss	2003
2925	0	0	0	2629	Kokou Thomasametowossi	2004
2926	0	0	0	2629	Danielkrige	2005
2927	0	0	0	2629	Setthawutsinpibarn	2004
2928	0	0	0	2629	Thomaskennedy	2003
2929	0	0	0	2629	Tomas Agustingonzalez	2003
2930	0	0	0	2629	Tawananyashamanzvera	2004
2931	0	0	0	2629	Lucastopforth	2007
2932	0	0	0	2629	Filipsemancik	2004
2933	0	0	0	2629	Igorkubalka	2005
2934	0	0	0	2629	Emmanueloudensi	2005
2935	0	0	0	2629	Codyatkinson	2008
2936	0	0	0	2629	Andyallen	2005
2937	0	0	0	2629	Silas Patrikscherrer	2004
2938	0	0	0	2629	Alexandrudamian	2004
2939	0	0	0	2629	Brandonvu	2006
2940	0	0	0	2629	Rohanhazratwala	2007
2941	0	0	0	2629	Setthawutsinpibarn	2004
2942	0	0	0	2629	Silas Patrikscherrer	2004
2943	0	0	0	2629	Tomas Agustingonzalez	2003
2944	0	0	0	2629	Tonylai	2003
2945	0	0	0	2629	Hendrikkatmuk	2004
2946	0	0	0	2629	Vladimircuc	2003

2947	0	0	0	2629	Christianceuca	2003
2948	0	0	0	2629	Alvarorodes	2003
2949	0	0	0	2629	Jackcurtis	2005
2950	0	0	0	2629	Jasonhuang	2006
2951	0	0	0	2629	Filipsemancik	2004
2952	0	0	0	2629	Axeledstrom	2005
2953	0	0	0	2715	Sklarphillips	2007
2954	0	0	0	2715	Azeezaleem azeez similoluwa	2003
2955	0	0	0	2715	Vitaliyhorovoy	2005
2956	0	0	0	2715	Adrian Cristiangheorghe	2003
2957	0	0	0	2715	Allankrueger	2003
2958	0	0	0	2715	Mikhailsilvestrov	2005
2959	0	0	0	2715	Luiz Felipegobbi brandao	2005
2960	0	0	0	2715	Benjaminhyde-smith	2004
2961	0	0	0	2715	Luisgarcia paez	2006
2962	0	0	0	2715	Ahmad Naelqureshi	2006
2963	0	0	0	2715	Piekebouman	2003
2964	0	0	0	2715	Giorgigiorgadze	2003
2965	0	0	0	2715	Peterkucera	2003
2966	0	0	0	2715	Maximollamas castellanos	2006
2967	0	0	0	2715	Pablomartinez gomez	2006
2968	0	0	0	2715	Owennguyen	2007
2969	0	0	0	2715	Williamelmers	2005
2970	0	0	0	2715	Juan Estebantrujillo hernandez	2006
2971	0	0	0	2715	Karimgazizov	2004
2972	0	0	0	2715	Haktangarayev	2003
2973	0	0	0	2715	Ralphbartlett	2004
2974	0	0	0	2715	Andrisoome	2005
2975	0	0	0	2715	Izanalmazan valiente	2006
2976	0	0	0	2715	Esteveagusti	2004
2977	0	0	0	2715	Rupertgeorge	2004
2978	0	0	0	2715	Rodrigoochoa ibarguengoitia	2005
2979	0	0	0	2715	Edoardobrustia	2003
2980	0	0	0	2715	Simonede luigi	2004
2981	0	0	0	2715	Konstantinegogeliya	2005
2982	0	0	0	2715	Viktorfraenkel	2005
2983	0	0	0	2715	Gabrielporras	2005
2984	0	0	0	2715	Danielbien	2006
2985	0	0	0	2715	Vicentepreto	2005
2986	0	0	0	2715	Eduardosanchez jover	2005
2987	0	0	0	2715	Nicolasrobert	2006
2988	0	0	0	2715	Cooperdillon	2005
2989	0	0	0	2715	Emilianojorquera	2003
2990	0	0	0	2715	Bastiendanicher	2006
2991	0	0	0	2715	Hugode grande	2006
2992	0	0	0	2715	Wenxugu	2004
2993	0	0	0	2715	Egorpishchelev	2006
2994	0	0	0	2715	Danhosono	2005
2995	0	0	0	2715	Mohamed Belalsoliman	2004
2996	0	0	0	2715	Ramonstrassmann	2003

2997	0	0	0	2715	Yeonsujeong	2007
2998	0	0	0	2715	Iliecazac	2006
2999	0	0	0	2715	Christiancapacci	2005
3000	0	0	0	2715	Gian Carlonavarro	2004
3001	0	0	0	2715	Santiagobaez bolanos	2004
3002	0	0	0	2715	Filippocaporali	2005
3003	0	0	0	2715	Daniilblinov	2005
3004	0	0	0	2715	Muhammad Haider Alirizwan	2007
3005	0	0	0	2715	Tristanreiff	2003
3006	0	0	0	2715	Andrealibre	2003
3007	0	0	0	2715	Simonmalis	2003
3008	0	0	0	2715	Christoszonias	2004
3009	0	0	0	2715	Nicolafilippi	2003
3010	0	0	0	2715	Axelvalavaara	2004
3011	0	0	0	2715	Adrienducruet	2006
3012	0	0	0	2715	Cesargarcia	2004
3013	0	0	0	2715	Bakhtiyorisroilov	2004
3014	0	0	0	2715	Bardiaziyaei	2004
3015	0	0	0	2715	Adamvasir	2004
3016	0	0	0	2715	Victordominguez torro	2004
3017	0	0	0	2715	Man Hoyap	2004
3018	0	0	0	2715	Lukaskrupa	2006
3019	0	0	0	2715	Curtis Hong Tsengtan	2005
3020	0	0	0	2715	Wooinlee	2006
3021	0	0	0	2715	Harryrock	2003
3022	0	0	0	2715	Giandurrer	2004
3023	0	0	0	2715	Giorgivachnadze	2004
3024	0	0	0	2715	Kriishtyagi	2007
3025	0	0	0	2715	Alejandrogonzalez	2005
3026	0	0	0	2715	Rishivandhansadeeshkumar	2006
3027	0	0	0	2715	Mohamed Mostafamoens	2004
3028	0	0	0	2790	Diegovega	2006
3029	0	0	0	2790	Kostiantynaksonov	2005
3030	0	0	0	2790	Maximiliankolk	2005
3031	0	0	0	2790	Curziomanucci	2005
3032	0	0	0	2790	Tomokisotoike	2006
3033	0	0	0	2790	Alanjesudason	2003
3034	0	0	0	2790	Bjorn Cristianoasbjornrod valdi	2006
3035	0	0	0	2790	Huzaima Abdulrehman	2005
3036	0	0	0	2790	Carlos Manuelgarcia guzman	2006
3037	0	0	0	2790	Patrick Oscarcrespo lima	2005
3038	0	0	0	2790	Henryde keulenaer	2005
3039	0	0	0	2790	Tomokisotoike	2006
3040	0	0	0	2790	Carlos Manuelgarcia guzman	2006
3041	0	0	0	2790	Luka Takumistula	2004
3042	0	0	0	2790	Kiarashrasamnezhad	2003
3043	0	0	0	2790	Mathislaffont	2004
3044	0	0	0	2790	RENEmorales	2004
3045	0	0	0	2790	Navidrezarowshanghias	2004
3046	0	0	0	2790	Noahcoghlan	2006

3047	0	0	0	2790	Tiagocueto bon-betemps	2006
3048	0	0	0	2790	Ivanivanov	2004
3049	0	0	0	2790	Nikhilmittal	2003
3050	0	0	0	2790	Jackwong	2004
3051	0	0	0	2790	Mohamed Sanadbouhlel	2006
3052	0	0	0	2790	Mirceamortasifu	2004
3053	0	0	0	2790	Andrearossi principe	2006
3054	0	0	0	2790	ARYAbhattacharya	2005
3055	0	0	0	2790	Naisdridi	2007
3056	0	0	0	2790	Joaoteixeira	2005
3057	0	0	0	2790	Maximiliankolk	2005
3058	0	0	0	2790	Ronitkarki	2007
3059	0	0	0	2790	Joelmartin perez	2003
3060	0	0	0	2790	Adamkaczmarek	2004
3061	0	0	0	2790	Mario Alejandrovirreira toro	2003
3062	0	0	0	2790	Fabiangarvik	2006
3063	0	0	0	2790	Yanghopark	2006
3064	0	0	0	2790	Bjorn Cristianoasbjornrod valdi	2006
3065	0	0	0	2790	Henryde keulenaer	2005
3066	0	0	0	2790	Carlosmanaure	2005
3067	0	0	0	2790	Yukikani	2005
3068	0	0	0	2790	Nicojamison	2004
3069	0	0	0	2790	Patrick Oscarcrespo lima	2005
3070	0	0	0	2790	Zoheirfardoun	2006
3071	0	0	0	2790	Brendanjayaprakash	2005
3072	0	0	0	2790	Nikitatsiareshka	2005
3073	0	0	0	2790	Harrylumb	2006
3074	0	0	0	2790	Samandarabdumalikov	2003
3075	0	0	0	2790	Yudaii wai	2004
3076	0	0	0	2790	Naokiwatanabe	2005
3077	0	0	0	2790	Balsadjokovic	2006
3078	0	0	0	2790	Vorshevdanila	2005
3079	0	0	0	2790	Hudsonbaxter	2005
3080	0	0	0	2790	Pietrocasciola	2006
3081	0	0	0	2790	Juhokim	2006
3082	0	0	0	2790	Dragosbucurescu	2005
3083	0	0	0	2790	MICHAELnekepeme	2006
3084	0	0	0	2790	RYOsunami	2004
3085	0	0	0	2790	Yusuketodoroki	2005
3086	0	0	0	2790	Krittamaetthammakun	2008
3087	0	0	0	2790	Zacharyriley	2005
3088	0	0	0	2790	Lukavojvodic	2005
3089	0	0	0	2790	Noahschmiedel	2005
3090	0	0	0	2790	Haraldsrozkalns	2004
3091	0	0	0	2790	Bugraozkoc	2006
3092	0	0	0	2790	Naoyakomoda	2004
3093	0	0	0	2790	Andreirogatskii	2007
3094	0	0	0	2790	Rodrigo Sebastianmio sernaque	2003
3095	0	0	0	2790	Ojente Friday Azamataojente	2006
3096	0	0	0	2790	Janbako	2005

3097	0	0	0	2790	Vitaliihnidets	2006
3098	0	0	0	2790	Zaidmashni	2006
3099	0	0	0	2790	Alanjesudason	2003
3100	0	0	0	2790	Jose Andresandrade aspiazu	2004
3101	0	0	0	2790	Victorarribas gutierrez	2003
3102	0	0	0	2790	Stefanozanette perez	2006
3103	0	0	0	2790	jibriladamu	2006
3104	0	0	0	2790	Danielekango	2009
3105	0	0	0	2790	Jackdeveney	2005
3106	0	0	0	2790	Nicolasdispas	2006
3107	0	0	0	2790	Markobanicevic	2005
3108	0	0	0	2790	SANTIAGO MARTINenriquez bo	2006
3109	0	0	0	2790	Raul Rokitagarcia	2004
3110	0	0	0	2790	Nicolasarredondo	2006
3111	0	0	0	2790	Ethan Lovrehirons	2005
3112	0	0	0	2790	Jose Andresarandia	2003
3113	0	0	0	2790	Adamsingleton	2004
3114	0	0	0	2790	Sihookim	2005
3115	0	0	0	2790	Nisanshrestha	2003
3116	0	0	0	2790	Mathyspicard	2007
3117	0	0	0	2790	Tibomeermans	2007
3118	0	0	0	2790	Angelivanov	2004
3119	0	0	0	2790	Branislavkrstevski	2004
3120	0	0	0	2790	Davistaylor	2004
3121	0	0	0	2790	Leonardokolbe	2005
3122	0	0	0	2790	Amarkolic	2005
3123	0	0	0	2790	Gregoryvrel nagel	2007
3124	0	0	0	2790	Diegovega	2006
3125	0	0	0	2790	Nicolassteden	2005
3126	0	0	0	2790	Cabrel Jordankenfack azankia	2005
3127	0	0	0	2790	Demirhalisdemir	2007
3128	0	0	0	2790	Pavelsavelov	2006
3129	0	0	0	2790	Pavelcherevatyy	2005
3130	0	0	0	2790	Juan Cruzcollardin	2005
3131	0	0	0	2790	Alexanderpenagos	2005
3132	0	0	0	2790	Ilyagrishkov	2006
3133	0	0	0	2790	Vibhuchallagundla	2005
3134	0	0	0	2790	Linuslagerbohm	2007
3135	0	0	0	2790	Deniskrakovsky	2006
3136	0	0	0	2790	Ilyareznikov	2005
3137	0	0	0	2790	Curziomanucci	2005
3138	0	0	0	2790	Mikayelavanesyan	2008
3139	0	0	0	2790	Nicojamison	2004
3140	0	0	0	2790	Naokiwatanabe	2005
3141	0	0	0	2790	RYOsunami	2004
3142	0	0	0	2790	Naoyakomoda	2004
3143	0	0	0	2790	Darkokolevski	2004
3144	0	0	0	2790	RENEmorales	2004
3145	0	0	0	2790	Jackwong	2004
3146	0	0	0	2790	Lucabenaibbouche	2005

3147	0	0	0	2790	Ronitkarki	2007
3148	0	0	0	2790	Stefanozanette perez	2006
3149	0	0	0	2790	Rodrigo Sebastianmio sernaque	2003
3150	0	0	0	2790	Angelivanov	2004
3151	0	0	0	2790	Vorshevdanila	2005
3152	0	0	0	2790	Krittamaetthammakun	2008
3153	0	0	0	2790	Kiarashrasamnezhad	2003
3154	0	0	0	2790	Nicolasarredondo	2006
3155	0	0	0	2790	Sebastianbaumann	2007
3156	0	0	0	2790	Olivernaude	2004
3157	0	0	0	2790	Mohamed Sanadbouhlel	2006
3158	0	0	0	2790	Yassinhassan	2006
3159	0	0	0	2790	Mehmet Eminadiguezel	2004
3160	0	0	0	2790	Lukavojvodic	2005
3161	0	0	0	2790	Janbako	2005
3162	0	0	0	2790	Salvadorprice	2005
3163	0	0	0	2790	Victor Alvaresde castro rocha	2007
3164	0	0	0	2790	Fabiangarvik	2006
3165	0	0	0	2790	Zoheirfardoun	2006
3166	0	0	0	2790	Noahcoghlan	2006
3167	0	0	0	2790	ARYAbhattacharya	2005
3168	0	0	0	2790	Mirceamortasifu	2004
3169	0	0	0	2790	Masanarisohda	2005
3170	0	0	0	2790	Mouadhdhaouadi	2007
3171	0	0	0	2790	Tiagocueto bon-betemps	2006
3172	0	0	0	2790	Ivanivanov	2004
3173	0	0	0	2790	Joaoteixeira	2005
3174	0	0	0	2790	Anthonymoukarzel	2006
3175	0	0	0	2790	Rayenhouas	2006
3176	0	0	0	2790	ANTOINEdidout	2005
3177	0	0	0	2790	Carlosmanaure	2005
3178	0	0	0	2790	Huzaima Abdulrehman	2005
3179	0	0	0	2790	Naisdridi	2007
3180	0	0	0	2790	Bogdanvalentov	2004
3181	0	0	0	2790	Victorarribas gutierrez	2003
3182	0	0	0	2790	Raul Rokitagarcia	2004
3183	0	0	0	2790	Tomokisotoike	2006
3184	0	0	0	2790	Adamkaczmarek	2004
3185	0	0	0	2790	NIKILdosuza	2006
3186	0	0	0	2790	makitakei	2007
3187	0	0	0	2790	jibriladamu	2006
3188	0	0	0	2790	Jean carlofranco	2005
3189	0	0	0	2790	Luisagullo menarguez	2005
3190	0	0	0	2790	Cabrel Jordankenfack azankia	2005
3191	0	0	0	2790	Yanghopark	2006
3192	0	0	0	2790	Brendanjayaprakash	2005
3193	0	0	0	2790	Hudsonbaxter	2005
3194	0	0	0	2790	Zacharyriley	2005
3195	0	0	0	2790	Ojente Friday Azamataojente	2006
3196	0	0	0	2790	Abelsaura	2005

3197	0	0	0	2790	Mathislaffont	2004
3198	0	0	0	2790	Joelmartin perez	2003
3199	0	0	0	2790	Yukikani	2005
3200	0	0	0	2790	Yudaii wai	2004
3201	0	0	0	2790	MICHAELnekepeme	2006
3202	0	0	0	2790	Danielekango	2009
3203	0	0	0	2790	Alessandrosciacca	2006
3204	1	1	1	1	Haroldmayot	2002
3205	1	1	1	2	Holgerrune	2003
3206	1	1	1	3	Dominicstricker	2002
3207	1	1	1	4	Shintaromochizuki	2003
3208	1	1	1	5	Arthurcazaux	2002
3209	1	1	1	6	Leandroriedi	2002
3210	1	1	1	7	Martindamm	2003
3211	1	1	1	8	Natanrodrigues	2002
3212	1	1	1	9	Karlisozolins	2002
3213	1	1	1	10	Lucianodarderi	2002
3214	1	1	1	11	Hanwenli	2002
3215	1	1	1	12	Arthurfery	2002
3216	1	1	1	13	Juan Bautistatorres	2002
3217	1	1	1	14	Peterfajta	2002
3218	1	1	1	15	Khololwammontsi	2002
3219	1	1	1	16	Jeffreyvon der schulenburg	2002
3220	1	1	1	17	Timolegout	2002
3221	1	1	1	18	Gustavoheide	2002
3222	1	1	1	19	Flaviocobolli	2002
3223	1	1	1	20	Guyden ouden	2002
3224	1	1	1	21	Martinbreysach	2002
3225	1	1	1	22	Tobykodat	2003
3226	1	1	1	23	Daliblanch	2003
3227	1	1	1	24	Lilianmarmousez	2002
3228	1	1	1	25	Roman Andresburruchaga	2002
3229	1	1	1	26	Alexbarrena	2002
3230	1	1	1	27	Jackpinnington jones	2003
3231	1	1	1	28	Daliborsvrcina	2002
3232	1	1	1	29	Martinkrumich	2002
3233	1	1	1	30	Lukasneumayer	2002
3234	1	1	1	31	Pierre Yvesbailly	2003
3235	1	1	1	32	Raphaelcollignon	2002
3236	1	1	1	33	Terenceatmane	2002
3237	1	1	1	34	Felixgill	2002
3238	1	1	1	35	Eliakimcoulibaly	2002
3239	1	1	1	36	Giovannimpetshi perricard	2003
3240	1	1	1	37	Hamadmedjedovic	2003
3241	1	1	1	38	Seancuenin	2004
3242	1	1	1	39	Jeromekym	2003
3243	1	1	1	40	Nicholas Davidionel	2002
3244	1	1	1	41	Shunsukemitsui	2002
3245	1	1	1	42	Yunchaoketebu	2002
3246	1	1	1	43	Makskasnikowski	2003

3247	1	1	1	44	Arthurfils	2004
3248	1	1	1	45	Alexanderhoogmartens	2002
3249	1	1	1	46	Brunooliveira	2002
3250	1	1	1	47	Junchengshang	2005
3251	1	1	1	48	Mansdahlberg	2003
3252	1	1	1	49	Mikolajlorens	2002
3253	1	1	1	50	Colemanwong	2004
3254	1	1	1	51	Egoragafonov	2002
3255	1	1	1	52	Diego Augustobarreto sanchez	2002
3256	1	1	1	53	Maratsharipov	2002
3257	1	1	1	54	Lucanardi	2003
3258	1	1	1	55	Pedroboscardin dias	2003
3259	1	1	1	56	Alexanderbernard	2003
3260	1	1	1	57	Samuelvincent ruggeri	2002
3261	1	1	1	58	Brunokuzuhara	2004
3262	1	1	1	59	Elmermoeller	2003
3263	1	1	1	60	Lucavan assche	2004
3264	1	1	1	61	Aleksanderorlikowski	2003
3265	1	1	1	62	Romainfaucon	2002
3266	1	1	1	63	Mariomansilla diez	2002
3267	1	1	1	64	Zsomborvelcz	2002
3268	1	1	1	65	Leonardomalgaroli	2002
3269	1	1	1	66	Sebastiangima	2002
3270	1	1	1	67	Ilyasnitari	2002
3271	1	1	1	68	Rafael Alfonsode alba valdes	2002
3272	1	1	1	69	Pengyulu	2002
3273	1	1	1	70	Markotopo	2003
3274	1	1	1	71	Maxwiskandt	2002
3275	1	1	1	72	Aidanmayo	2003
3276	1	1	1	73	Maxwestphal	2003
3277	1	1	1	74	Lorenzoclaverie	2002
3278	1	1	1	75	Axelgarcian	2002
3279	1	1	1	76	Jackanthrop	2003
3280	1	1	1	77	Maxwellmckennon	2002
3281	1	1	1	78	Devjavia	2002
3282	1	1	1	79	Kokoroisomura	2003
3283	1	1	1	80	Matteogigante	2002
3284	1	1	1	81	Pablollamas ruiz	2002
3285	1	1	1	82	Biagiogramaticopolo	2002
3286	1	1	1	83	Mehdisadaoui	2003
3287	1	1	1	84	Patornhanchaikul	2002
3288	1	1	1	85	Tobysamuel	2002
3289	1	1	1	86	Carlosalcaraz	2003
3290	1	1	1	87	Danielvallejo	2004
3291	1	1	1	88	Pyotrnesterov	2003
3292	1	1	1	89	Igorkudriashov	2003
3293	1	1	1	90	Maxalcala gurri	2002
3294	1	1	1	91	Nikitakashirin	2002
3295	1	1	1	92	Yuzhang	2002
3296	1	1	1	93	Philipsekulic	2003

3297	1	1	1	94	Lorenzorottoli	2002
3298	1	1	1	95	Michalnovansky	2002
3299	1	1	1	96	Maikelde boes	2002
3300	1	1	1	97	Chendong	2002
3301	1	1	1	98	Konstantinzhzhenov	2004
3302	1	1	1	99	Evgenyphilippov	2002
3303	1	1	1	100	Damiensalvestre	2002
3304	1	1	1	101	Martinkatz	2003
3305	1	1	1	102	Francescomaestrelli	2002
3306	1	1	1	103	Mikalipp	2003
3307	1	1	1	104	Viacheslavbielinskyi	2003
3308	1	1	1	105	Jean Marcmalkowski	2002
3309	1	1	1	106	Santiagode la fuente	2002
3310	1	1	1	107	Abedallahshelbayh	2003
3311	1	1	1	108	Samirbanerjee	2003
3312	1	1	1	109	Simeonterziev	2002
3313	1	1	1	110	Benjaminkittay	2003
3314	1	1	1	111	Mateogomez	2002
3315	1	1	1	112	Joaquimalmeida	2002
3316	1	1	1	113	Joao Victorcouto loureiro	2003
3317	1	1	1	114	Joshualapadat	2002
3318	1	1	1	115	Alibekkachmazov	2002
3319	1	1	1	116	Noahlopez	2002
3320	1	1	1	117	Angelguerrero melgar	2002
3321	1	1	1	118	Juan Sebastianzabala vargas	2002
3322	1	1	1	119	Luimaxted	2003
3323	1	1	1	120	Szymonkielan	2002
3324	1	1	1	121	Danielrincon	2003
3325	1	1	1	122	Nicolaszanellato	2002
3326	1	1	1	123	Ozanbaris	2004
3327	1	1	1	124	Max Hansrehberg	2003
3328	1	1	1	125	Milipoljicak	2004
3329	1	1	1	126	Joshuadous karpenschif	2003
3330	1	1	1	127	Markoandrejic	2003
3331	1	1	1	128	Iirovasa	2002
3332	1	1	1	129	Mikhailgorokhov	2004
3333	1	1	1	130	Svyatoslavgulin	2002
3334	1	1	1	131	Victorlilov	2004
3335	1	1	1	132	Juan Sebastiandominguez colla	2002
3336	1	1	1	133	Saschagueymard wayenburg	2003
3337	1	1	1	134	Leoborg	2003
3338	1	1	1	135	Benitosanchez martinez	2002
3339	1	1	1	136	Lukapetrovic	2002
3340	1	1	1	137	Danielibragimov	2002
3341	1	1	1	138	Peter Benjaminprivara	2004
3342	1	1	1	139	Rostislavhalfinger	2002
3343	1	1	1	140	Stijnpel	2002
3344	1	1	1	141	Yamatosueoka	2003
3345	1	1	1	142	Cashhanzlik	2002
3346	1	1	1	143	Adamjurajda	2003

3347	1	1	1	144	Lukaspalovic	2002
3348	1	1	1	145	Han-Chihlin	2002
3349	1	1	1	146	Jessede jager	2002
3350	1	1	1	147	Alexbulte	2002
3351	1	1	1	148	Marc Othmanktiri	2003
3352	1	1	1	149	Corbancrowther	2003
3353	1	1	1	150	Robinbertrand	2003
3354	1	1	1	151	Germansamofalov	2003
3355	1	1	1	152	Orelkimhi	2003
3356	1	1	1	153	Aissabenchakroun	2002
3357	1	1	1	154	Haoyuanhuang	2002
3358	1	1	1	155	Jaszaszajrych	2002
3359	1	1	1	156	Albertoorso	2002
3360	1	1	1	157	Matthew Williamdonald	2004
3361	1	1	1	158	Valerioaboian	2002
3362	1	1	1	159	Anthonygenov	2002
3363	1	1	1	160	Stefanpopovic	2003
3364	1	1	1	161	Williamnolan	2003
3365	1	1	1	162	Franciscollanes	2002
3366	1	1	1	163	Inakimontes-de la torre	2002
3367	1	1	1	164	Michael Franciseala	2002
3368	1	1	1	165	Aleksandrmyagkov	2002
3369	1	1	1	166	Marklajal	2003
3370	1	1	1	167	Bohuadong	2002
3371	1	1	1	168	Zanekhan	2002
3372	1	1	1	169	Aristarkhsafonov	2003
3373	1	1	1	170	Stefanleustian	2002
3374	1	1	1	171	Adrienburdet	2002
3375	1	1	1	172	Henriquerocha	2004
3376	1	1	1	173	Aleksandrkalinin	2002
3377	1	1	1	174	Bornadevald	2002
3378	1	1	1	175	Constantinoskoshis	2004
3379	1	1	1	176	Tokyranaivo	2003
3380	1	1	1	177	Mariogonzalez fernandez	2002
3381	1	1	1	178	Alvaroguillen meza	2003
3382	1	1	1	179	Yunseokjang	2003
3383	1	1	1	180	Petarjovanovic	2003
3384	1	1	1	181	Ezequielmonferrer	2003
3385	1	1	1	182	Ondrejhorak	2002
3386	1	1	1	183	Markostakusic	2003
3387	1	1	1	184	Petarteodorovic	2003
3388	1	1	1	185	Connorkrug	2002
3389	1	1	1	186	Miguelgomes	2003
3390	1	1	1	187	Jamestracy	2002
3391	1	1	1	188	Valentinlapalu	2002
3392	1	1	1	189	Vitekhorak	2002
3393	1	1	1	190	Jakubmensik	2005
3394	1	1	1	191	Dawidtaczala	2002
3395	1	1	1	192	Caseyhoole	2003
3396	1	1	1	193	Jerryroddick	2002

3397	1	1	1	194	Brunonhavene	2002
3398	1	1	1	195	Lukaspokorny	2002
3399	1	1	1	196	Philipflorig	2003
3400	1	1	1	197	Marcelkamrowski	2003
3401	1	1	1	198	Artempodorozhnyi	2003
3402	1	1	1	199	Thibaultverdier	2003
3403	1	1	1	200	Elyesmarouani	2002
3404	1	1	1	201	Jingu	2002
3405	1	1	1	202	Jakekrug	2002
3406	1	1	1	203	Mehdibenchakroun	2003
3407	1	1	1	204	Wissamabderrahman	2003
3408	1	1	1	205	Nisheshbasavareddy	2005
3409	1	1	1	206	Linuscarlsson halldin	2002
3410	1	1	1	207	Mathisdebru	2002
3411	1	1	1	208	Danielsiniakov	2003
3412	1	1	1	209	Dong Jukim	2002
3413	1	1	1	210	Borartnak	2004
3414	1	1	1	211	Yassinedlimi	2003
3415	1	1	1	212	Rodrigopacheco mendez	2005
3416	1	1	1	213	Emilianogonzalez	2002
3417	1	1	1	214	Pabloalemany	2002
3418	1	1	1	215	Antonshepp	2002
3419	1	1	1	216	Ammarelamin	2002
3420	1	1	1	217	Thijmenloof	2002
3421	1	1	1	218	Pedroaraujo	2002
3422	1	1	1	219	Fonsvan sambeek	2002
3423	1	1	1	220	Reecefalck	2002
3424	1	1	1	221	Woobinshin	2004
3425	1	1	1	222	Matejgalik	2002
3426	1	1	1	223	Aditsinha	2003
3427	1	1	1	224	Aidankim	2004
3428	1	1	1	225	Faustotabacco	2002
3429	1	1	1	226	Daniellinkuns-morozovs	2002
3430	1	1	1	227	Jamesmccabe	2003
3431	1	1	1	228	Derrickchen	2003
3432	1	1	1	229	Antoineghibaudo	2005
3433	1	1	1	230	Gabrielhurtado	2002
3434	1	1	1	231	Bekhzodrasamatov	2002
3435	1	1	1	232	Ilyalebedev	2003
3436	1	1	1	233	Dinkodinev	2004
3437	1	1	1	234	Johan Alexanderrodriguez rodri	2003
3438	1	1	1	235	Maximilianhomberg	2003
3439	1	1	1	236	Yan Chengchen	2002
3440	1	1	1	237	Azizdadabaev	2002
3441	1	1	1	238	Maximzhukov	2005
3442	1	1	1	239	Cooperwhite	2003
3443	1	1	1	240	Piotrpawlak	2002
3444	1	1	1	241	Tanapattnirundorn	2004
3445	1	1	1	242	Nicolascortes campos	2002
3446	1	1	1	243	Murphycassone	2002

3447	1	1	1	244	Miguelperez pena	2003
3448	1	1	1	245	Amauryraynel	2002
3449	1	1	1	246	Guntinunsootinun-opart	2003
3450	1	1	1	247	Xavimatas ortega	2002
3451	1	1	1	248	Kalinivanovski	2004
3452	1	1	1	249	Oskar Brostrompoulsen	2002
3453	1	1	1	250	Leoraquin	2003
3454	1	1	1	251	Maximemareschal-hay	2002
3455	1	1	1	252	Fnunidunjianzan	2004
3456	1	1	1	253	Giulioperego	2003
3457	1	1	1	254	Lautaromidon	2004
3458	1	1	1	255	Danielmerida	2004
3459	1	1	1	256	Sambossem	2002
3460	1	1	1	257	Reiyakomagata	2003
3461	1	1	1	258	Lukamikrut	2004
3462	1	1	1	259	Bradenshick	2003
3463	1	1	1	260	Marcio Silvamarcio silva	2002
3464	1	1	1	261	Danilozernoy	2002
3465	1	1	1	262	Devinbadenhorst	2004
3466	1	1	1	263	Giorgiotabacco	2003
3467	1	1	1	264	Sylvestremonnou	2002
3468	1	1	1	265	Enzoaguiard	2002
3469	1	1	1	266	Petrbar biryukov	2002
3470	1	1	1	267	Gilles Arnaudbailly	2005
3471	1	1	1	268	Peteralam	2002
3472	1	1	1	269	Samuelpaquette	2002
3473	1	1	1	270	Hikarutakeda	2002
3474	1	1	1	271	Uditgogoi	2004
3475	1	1	1	272	Lazarmilicevic	2002
3476	1	1	1	273	Azumavisaya	2003
3477	1	1	1	274	Codie Schalkvan schalkwyk	2002
3478	1	1	1	275	Nivzuckerman	2002
3479	1	1	1	276	Ilyatiraspolsky	2002
3480	1	1	1	277	Iliasimakin	2003
3481	1	1	1	278	Tai Leonardsach	2002
3482	1	1	1	279	Nikolaynedelchev	2003
3483	1	1	1	280	Sameral tori	2003
3484	1	1	1	281	Carlroothman	2003
3485	1	1	1	282	Matthewbosancic	2002
3486	1	1	1	283	NAN HSUNlin	2003
3487	1	1	1	284	Richardgama	2003
3488	1	1	1	285	Olafpieczkowski	2004
3489	1	1	1	286	Matasvasiliauskas	2002
3490	1	1	1	287	Noritakakoizumi	2003
3491	1	1	1	288	Hunterheck	2002
3492	1	1	1	289	Jipvan assendelft	2002
3493	1	1	1	290	Matiasiturbe	2002
3494	1	1	1	291	Sebastianabboud	2002
3495	1	1	1	292	Alyshokeir	2002
3496	1	1	1	293	Alankam	2002

3497	1	1	1	294	Togantokac	2004
3498	1	1	1	295	Kaiyoteshima	2002
3499	1	1	1	296	Ardaazkara	2003
3500	1	1	1	297	Zacharysvajda	2002
3501	1	1	1	298	Filippieczonka	2004
3502	1	1	1	299	Guanglinwen	2003
3503	1	1	1	300	Victoriomarquiselli	2002
3504	1	1	1	301	Coltonsmith	2003
3505	1	1	1	302	Billysuarez	2002
3506	1	1	1	303	Vojtechpetr	2005
3507	1	1	1	304	Conorgannon	2002
3508	1	1	1	305	Tomasluis	2002
3509	1	1	1	306	Benshelton	2002
3510	1	1	1	307	Kamilkozlowski	2002
3511	1	1	1	308	Alvin Nicholastudorica	2002
3512	1	1	1	309	Edasbutvilas	2004
3513	1	1	1	310	Edson Alejandrosanchez elizond	2003
3514	1	1	1	311	Karimibrahim	2003
3515	1	1	1	312	Aryaanbhatia	2002
3516	1	1	1	313	Maxbasing	2002
3517	1	1	1	314	Pijusvaitiekunas	2003
3518	1	1	1	315	Nishantdabas	2003
3519	1	1	1	316	Shaoyunliu	2002
3520	1	1	1	317	Valentinode pellegrin	2002
3521	1	1	1	318	Franciscolamas villarroel	2003
3522	1	1	1	319	Mario Albertoduron garza	2002
3523	1	1	1	320	Mihai Alexandrucoman	2005
3524	0	1	1	321	Geniinoue	2003
3525	0	1	1	322	Taymal azmeh	2004
3526	0	1	1	323	Majpremzl	2004
3527	0	1	1	324	Taiyouenoyama	2003
3528	0	1	1	325	Erikarutiunian	2004
3529	0	1	1	326	Yousseflabbene	2004
3530	0	1	1	327	Radu Mihaipapoe	2002
3531	0	1	1	328	Felixnordby	2002
3532	0	1	1	329	Damienlaporte	2002
3533	0	1	1	330	Jakubnicod	2004
3534	0	1	1	331	Woodamsong	2003
3535	0	1	1	332	Arturkukasian	2004
3536	0	1	1	333	Habibstambouli	2002
3537	0	1	1	334	Clementchauvin	2002
3538	0	1	1	335	Maksimstarostin	2002
3539	0	1	1	336	Muhammadshoaib	2002
3540	0	1	1	337	Berkbugarikj	2003
3541	0	1	1	338	Mathiasmusil	2002
3542	0	1	1	339	Emilianoaguilera guerrero	2003
3543	0	1	1	340	Ekanshkumar	2003
3544	0	1	1	341	Yannikrahman	2004
3545	0	1	1	342	Juan Martinfumeaux buenaven	2002
3546	0	1	1	343	Matiasgaedechens	2002

3547	0	1	1	344	Younesdjouder	2003
3548	0	1	1	345	Dmitrybezborodov	2002
3549	0	1	1	346	Natebonetto	2003
3550	0	1	1	347	Venedikttsirikidze	2004
3551	0	1	1	348	Marcelpolitowicz	2002
3552	0	1	1	349	Samuelsanchez	2002
3553	0	1	1	350	Charalamposfouzas	2002
3554	0	1	1	351	Rikutoyamaguchi	2002
3555	0	1	1	352	Seonungcho	2003
3556	0	1	1	353	Youssefsadek	2003
3557	0	1	1	354	Xiaofeiwang	2003
3558	0	1	1	355	Kentanakamura	2004
3559	0	1	1	356	Samscherer	2004
3560	0	1	1	357	Suphawatsaeoui	2005
3561	0	1	1	358	Denislavprodanov	2002
3562	0	1	1	359	Andreasscott	2003
3563	0	1	1	360	Samuilkonov	2002
3564	0	1	1	361	Aleksilofman	2002
3565	0	1	1	362	Fredericvogeli	2003
3566	0	1	1	363	Pedrorodenas	2004
3567	0	1	1	364	Noahmartens	2002
3568	0	1	1	365	Nickbeamish	2002
3569	0	1	1	366	Edwardwinter	2004
3570	0	1	1	367	Jamesvan herzeele	2003
3571	0	1	1	368	Eytanmichaeli	2003
3572	0	1	1	369	Markdancuart	2002
3573	0	1	1	370	Mikaeelwoodman	2003
3574	0	1	1	371	Joelpierleoni	2003
3575	0	1	1	372	Kyle Lok Yintang	2002
3576	0	1	1	373	Galemersic potocnik	2003
3577	0	1	1	374	Joao Eduardoschiessl	2004
3578	0	1	1	375	Casperchristensen	2002
3579	0	1	1	376	Martynpawelski	2004
3580	0	1	1	377	Petrkapuskin	2002
3581	0	1	1	378	Jorgeplans	2002
3582	0	1	1	379	Patrickjoss	2002
3583	0	1	1	380	Junyitang	2002
3584	0	1	1	381	Manuelmouilleron salvo	2003
3585	0	1	1	382	Uddayvirsingh	2002
3586	0	1	1	383	Christiankuehne	2002
3587	0	1	1	384	Clementmainguy	2003
3588	0	1	1	385	Asaffriedler	2002
3589	0	1	1	386	Danielwebb	2002
3590	0	1	1	387	Neoniedner	2003
3591	0	1	1	388	Lorenzoesquici	2003
3592	0	1	1	389	Maximusjones	2004
3593	0	1	1	390	Michaelminasyan	2003
3594	0	1	1	391	Stianklaassen	2003
3595	0	1	1	392	Charleliecosnet	2003
3596	0	1	1	393	Grigoriigordeev	2003

3597	0	1	1	394	Tiagotorres	2002
3598	0	1	1	395	Noahmerre	2004
3599	0	1	1	396	Ronellouck	2004
3600	0	1	1	397	Hanamichicarvajal suazo	2002
3601	0	1	1	398	Dylansalton	2002
3602	0	1	1	399	Timothyphung	2003
3603	0	1	1	400	Dominikbuzonics	2002
3604	0	1	1	401	Melchiordelloye	2003
3605	0	1	1	402	Walidahouda	2003
3606	0	1	1	403	Dominikrys	2002
3607	0	1	1	404	William Yovannyribero duarte	2003
3608	0	1	1	405	Aleksandartomas	2003
3609	0	1	1	406	Tomaspinho	2002
3610	0	1	1	407	Spencerbrachman	2002
3611	0	1	1	408	Assilboussayri	2002
3612	0	1	1	409	Alejandromanzanera pertusa	2003
3613	0	1	1	410	Juan Carlosprado angelo	2005
3614	0	1	1	411	Maximdontsov	2004
3615	0	1	1	412	Mikhailkniazeu	2002
3616	0	1	1	413	Dominikjakovljevic	2002
3617	0	1	1	414	Ainiussabaliauskas	2003
3618	0	1	1	415	Gonzalobueno	2004
3619	0	1	1	416	Jerallyasin	2002
3620	0	1	1	417	Gerardplanelles ripoll	2003
3621	0	1	1	418	ADITYA VARDHANroy chowdhu	2002
3622	0	1	1	419	Daniilssnaiders	2003
3623	0	1	1	420	Dinoprizmic	2005
3624	0	1	1	421	Aaroncohen	2002
3625	0	1	1	422	Matiasolivero vidal	2002
3626	0	1	1	423	Vasildimitrov	2002
3627	0	1	1	424	Paulinchauspe	2004
3628	0	1	1	425	Photosphotiades	2003
3629	0	1	1	426	Matthewrutter	2003
3630	0	1	1	427	Mitsuki Wei Kangleong	2004
3631	0	1	1	428	Lucasbrown	2004
3632	0	1	1	429	Hadyel kordy	2005
3633	0	1	1	430	Glebkuharev	2003
3634	0	1	1	431	Alessiotramontin	2003
3635	0	1	1	432	Carlos Alejandrosolares midenc	2002
3636	0	1	1	433	Pavlosparson	2003
3637	0	1	1	434	Marcusmcdaniel	2002
3638	0	1	1	435	Alexfinkelstein	2002
3639	0	1	1	436	Maximehillhouse	2003
3640	0	1	1	437	Pablotrochu	2003
3641	0	1	1	438	Amansharma	2003
3642	0	1	1	439	Marinojakic	2002
3643	0	1	1	440	Daniilostapenkov	2003
3644	0	1	1	441	Ellisshort	2003
3645	0	1	1	442	Frankjonker	2002
3646	0	1	1	443	Johnchin	2003

3647	0	1	1	444	Arslanbektemirhanov	2003
3648	0	1	1	445	Daisukeinagawa	2002
3649	0	1	1	446	Jaydentempleman	2003
3650	0	1	1	447	Javier Eduardomatos	2002
3651	0	1	1	448	Connor Henryvan schalkwyk	2004
3652	0	1	1	449	Agustin Eduardocuellar lorberg	2003
3653	0	1	1	450	Lennon Roarkjones	2005
3654	0	1	1	451	Dragos Nicolaecazacu	2004
3655	0	1	1	452	Antonarzhankin	2003
3656	0	1	1	453	Ryancolby	2003
3657	0	1	1	454	Jose Antoniotria	2002
3658	0	1	1	455	Abayomi Oluseyiphilips	2002
3659	0	1	1	456	Luciussoller	2002
3660	0	1	1	457	Hamzael amine	2004
3661	0	1	1	458	Karl Kazumalee	2002
3662	0	1	1	459	Jakubprachar	2003
3663	0	1	1	460	Lucboulier santelices	2003
3664	0	1	1	461	Mathisbondaz	2002
3665	0	1	1	462	Artjommatus	2002
3666	0	1	1	463	Kai Shiangyang	2003
3667	0	1	1	464	Christopherfok kow	2002
3668	0	1	1	465	Ivanpauliuchenka	2003
3669	0	1	1	466	Jiayangdong	2004
3670	0	1	1	467	Mattiassouthcombe	2003
3671	0	1	1	468	Aaryanzaveri	2002
3672	0	1	1	469	Jungwonpark	2002
3673	0	1	1	470	Woohyuksim	2003
3674	0	1	1	471	Arthurgea	2005
3675	0	1	1	472	Boryszgola	2004
3676	0	1	1	473	Noahgampel	2002
3677	0	1	1	474	Diego Nora Rosagobis de castro	2002
3678	0	1	1	475	Yehongyu	2004
3679	0	1	1	476	Myko Yohann Himadkouame	2003
3680	0	1	1	477	Huzaifa Abdulrehman	2002
3681	0	1	1	478	Gabrielepiraino	2003
3682	0	1	1	479	Gabrielmatuszewski	2004
3683	0	1	1	480	Denis Constantinspiridon	2002
3684	0	1	1	481	Derekpham	2004
3685	0	1	1	482	Rikumiyata	2002
3686	0	1	1	483	Taentawan Taddeomajoli magg	2003
3687	0	1	1	484	Jankobierski	2004
3688	0	1	1	485	Jaroslavsmedek	2002
3689	0	1	1	486	Creditchaiyarin	2003
3690	0	1	1	487	Filipposastreinidis	2003
3691	0	1	1	488	Chengtang	2003
3692	0	1	1	489	Piotrkusiewicz	2003
3693	0	1	1	490	Viliusgaubas	2004
3694	0	1	1	491	Volodymyrzakharov	2002
3695	0	1	1	492	Blazvidovic	2002
3696	0	1	1	493	Harrylazell	2003

3697	0	1	1	494	Veljkokrstic	2003
3698	0	1	1	495	Giacomorevelli	2002
3699	0	1	1	496	Majdiben abdelwehid	2004
3700	0	1	1	497	Jackloutit	2004
3701	0	1	1	498	Alin Mihaifatu	2004
3702	0	1	1	499	Baransoyler	2004
3703	0	1	1	500	Francisco Tomasgeschwind	2003
3704	0	1	1	501	Kristiankubik	2002
3705	0	1	1	502	Liamliles	2002
3706	0	1	1	503	Sanjithdevineni	2003
3707	0	1	1	504	Estebanpenagos	2002
3708	0	1	1	505	Hynekbarton	2004
3709	0	1	1	506	Maximest-hilaire	2003
3710	0	1	1	507	Ivan Noelhornung	2002
3711	0	1	1	508	Timothycarlsson seger	2003
3712	0	1	1	509	Mikalaibankou	2005
3713	0	1	1	510	Nahuel Nicolaseli	2005
3714	0	1	1	511	Gabrielgeneroso	2003
3715	0	1	1	512	RODRIGObraunstein	2003
3716	0	1	1	513	Alexandergaponenko	2002
3717	0	1	1	514	Nikhilniranjan	2003
3718	0	1	1	515	Brenoferreira marques	2002
3719	0	1	1	516	Arturkhairutdinov	2004
3720	0	1	1	517	Shaybar menahem	2003
3721	0	1	1	518	Edvards Teodorsliepins	2003
3722	0	1	1	519	Oskarantinheimo	2002
3723	0	1	1	520	Germanmingaleev	2004
3724	0	1	1	521	Jonathanbaron	2002
3725	0	1	1	522	Gunawantrismuwantara	2003
3726	0	1	1	523	Peternad	2004
3727	0	1	1	524	Dragos Petrepopeanga	2002
3728	0	1	1	525	Isacstroemberg	2003
3729	0	1	1	526	Alexanderknox jones	2003
3730	0	1	1	527	Heziheng	2004
3731	0	1	1	528	Alenmujakic	2003
3732	0	1	1	529	Evangeloskypriotis	2003
3733	0	1	1	530	Janhrazdil	2005
3734	0	1	1	531	Georg Eduardisraelan	2005
3735	0	1	1	532	Aljazjeran	2003
3736	0	1	1	533	Sandeepvm	2003
3737	0	1	1	534	Elijahporitzky	2003
3738	0	1	1	535	Michaelbassem sobhy	2004
3739	0	1	1	536	Mykhailomossur	2003
3740	0	1	1	537	Amandahiya	2004
3741	0	1	1	538	Jamesoncorsillo	2002
3742	0	1	1	539	Ignaciobuse	2004
3743	0	1	1	540	Tzu Lichang	2003
3744	0	1	1	541	Lorenzomusetti	2002
3745	0	1	1	542	Alexandrkoliev	2002
3746	0	1	1	543	Sashatrkulja	2002

3747	0	1	1	544	Adamduan	2003
3748	0	1	1	545	Hugohashimoto	2003
3749	0	1	1	546	Jakubkatuch	2002
3750	0	1	1	547	Kajquirijns	2004
3751	0	1	1	548	Niroopvallabhaneni	2002
3752	0	1	1	549	Santiagorendon buitrago	2002
3753	0	1	1	550	Mohamed Aminoueslati	2004
3754	0	1	1	551	Danielbagnolini	2003
3755	0	1	1	552	Nedimsuko	2002
3756	0	1	1	553	Yuchenpan	2002
3757	0	1	1	554	Lucamcmanus	2002
3758	0	1	1	555	Eshaanlumba	2002
3759	0	1	1	556	Carlosguerrero alvarez	2004
3760	0	1	1	557	Nikolazekic	2004
3761	0	1	1	558	Artemgerasimchuk	2002
3762	0	1	1	559	Tim Thomasgauntlett	2003
3763	0	1	1	560	Brankodjuric	2005
3764	0	1	1	561	Lucasbritez risso	2003
3765	0	1	1	562	Daniloraicevic	2003
3766	0	1	1	563	Danijalmuminovic	2002
3767	0	1	1	564	Luckoenig	2003
3768	0	1	1	565	Ivan Karloban	2002
3769	0	1	1	566	Dusanrsovac	2003
3770	0	1	1	567	Edwardvo	2003
3771	0	1	1	568	Rodrigo Nicolasmontes de oca	2003
3772	0	1	1	569	Egorkhotchenkov	2003
3773	0	1	1	570	Etiennemarty	2004
3774	0	1	1	571	Gabrieliusguzauskas	2003
3775	0	1	1	572	Jay Dylanhara friend	2003
3776	0	1	1	573	Damiennezar	2002
3777	0	1	1	574	Ivanpenev	2003
3778	0	1	1	575	Matei Victorpalcau	2003
3779	0	1	1	576	Pietropampanin	2003
3780	0	1	1	577	Mikaberghaus	2002
3781	0	1	1	578	Muhammed Kaanerel	2002
3782	0	1	1	579	Yu-Chenchiu	2002
3783	0	1	1	580	Chiragduhan	2004
3784	0	1	1	581	Oscarpinto sansano	2003
3785	0	1	1	582	Ethan Denzelsibanda	2004
3786	0	1	1	583	Ruslantiukaev	2005
3787	0	1	1	584	Danieleminighini	2004
3788	0	1	1	585	Tiagopereira	2004
3789	0	1	1	586	owenmegargee	2004
3790	0	1	1	587	Jansebesta	2003
3791	0	1	1	588	Jaishvinsidana	2002
3792	0	1	1	589	Christopherheck	2002
3793	0	1	1	590	Stylianoschristodoulou	2004
3794	0	1	1	591	Janwajdemajer	2002
3795	0	1	1	592	Zhangeralmashev	2003
3796	0	1	1	593	Jadenweekes	2004

3797	0	1	1	594	Pablomasjuan ginel	2002
3798	0	1	1	595	Santiagoperez casamayor	2002
3799	0	1	1	596	Patrickbrady	2004
3800	0	1	1	597	Dragomirdraganov	2004
3801	0	1	1	598	Matthewgarcia	2002
3802	0	1	1	599	Alexanderfrusina	2005
3803	0	1	1	600	Nathan Anthonybarki	2004
3804	0	1	1	601	Barneyfitzpatrick	2002
3805	0	1	1	602	Cooperwilliams	2005
3806	0	1	1	603	Bastiaanweststrate	2003
3807	0	1	1	604	Richardmallory	2002
3808	0	1	1	605	Ha Minh Ducvu	2003
3809	0	1	1	606	Sebastiandominko	2003
3810	0	1	1	607	Vladimirbazilevskiy	2005
3811	0	1	1	608	Matthewshearer	2002
3812	0	1	1	609	Freekvan donselaar	2003
3813	0	1	1	610	nicolasnino mendoza	2004
3814	0	1	1	611	Joaoduleba	2002
3815	0	1	1	612	Abdurahmansaidmuhamedov	2003
3816	0	1	1	613	Ibrokhimjonurinov	2002
3817	0	1	1	614	Michael Felixfrank	2002
3818	0	1	1	615	Maxencerivet	2003
3819	0	1	1	616	Vlad Cristianbreazu	2003
3820	0	1	1	617	Alejandro Josegandini	2003
3821	0	1	1	618	Jaimefaria	2003
3822	0	1	1	619	Diegodigraci	2002
3823	0	1	1	620	Erikkovacs	2003
3824	0	1	1	621	Mikowassermann	2003
3825	0	1	1	622	Jian Keong Takeshikoey	2002
3826	0	1	1	623	Jakubostajewski	2002
3827	0	1	1	624	Williamjucha	2004
3828	0	1	1	625	Julianlozano	2003
3829	0	1	1	626	Oriolfillat gimenez	2002
3830	0	1	1	627	Bartnouws	2002
3831	0	1	1	628	Billyblaydes	2003
3832	0	1	1	629	Facundoyunis	2002
3833	0	1	1	630	Alejandrosanchez gonzalez	2002
3834	0	1	1	631	Vincentthiel	2002
3835	0	1	1	632	Evanwen	2004
3836	0	1	1	633	Oleksiyshestakov	2002
3837	0	1	1	634	Hyeondeokyoon	2003
3838	0	1	1	635	Daikiyoshimura	2002
3839	0	1	1	636	Alexander Georgmandma	2002
3840	0	1	1	637	Pedroliborio	2003
3841	0	1	1	638	Tomasmoretto	2003
3842	0	1	1	639	Kevinkovacs	2003
3843	0	1	1	640	Peerapatdedthonglang	2002
3844	0	1	1	641	Peterkalocsai	2003
3845	0	1	1	642	Kosukemorita	2004
3846	0	1	1	643	Denimyadav	2004

3847	0	1	1	644	Filipporomano	2005
3848	0	1	1	645	Yassineben jemaa	2002
3849	0	1	1	646	Jeremyjin	2004
3850	0	1	1	647	Loanlestir	2005
3851	0	1	1	648	Alexandrede villepin	2003
3852	0	1	1	649	Andreybeketov	2003
3853	0	1	1	650	Lukasmaskow	2003
3854	0	1	1	651	Oliverolsson	2004
3855	0	1	1	652	Dhruvtangri	2003
3856	0	1	1	653	Noavukadin	2004
3857	0	1	1	654	Mark Owenendler	2002
3858	0	1	1	655	Liamgavrielides	2004
3859	0	1	1	656	Franciscopini	2003
3860	0	1	1	657	Evanlee	2003
3861	0	1	1	658	Vascoleote prata	2003
3862	0	1	1	659	Jiaxima	2003
3863	0	1	1	660	Pedro Javieralvarez prado	2003
3864	0	1	1	661	Rashednawaf	2005
3865	0	1	1	662	Hyeoung Jinkim	2003
3866	0	1	1	663	Minjoonkim	2004
3867	0	1	1	664	Sebastiansec	2003
3868	0	1	1	665	Keeanshah	2002
3869	0	1	1	666	Akhilendranindrabalan	2002
3870	0	1	1	667	Martin Antoniovergara del pue	2004
3871	0	1	1	668	Adidagan	2003
3872	0	1	1	669	Benjamin Ignaciotorres fernanc	2003
3873	0	1	1	670	Samuelreeve	2004
3874	0	1	1	671	Tyswitzer	2003
3875	0	1	1	672	Lucacastagnola	2002
3876	0	1	1	673	Nikitasidorov	2003
3877	0	1	1	674	Kilianfeldbausch	2005
3878	0	1	1	675	Jakubjupa	2004
3879	0	1	1	676	Anronel	2004
3880	0	1	1	677	Krishanhooda	2003
3881	0	1	1	678	Kentamiyoshi	2004
3882	0	1	1	679	Marianotammaro	2004
3883	0	1	1	680	Itaybity wekselman	2004
3884	0	1	1	681	Ernesto Gabrielmealla	2002
3885	0	1	1	682	Oscartejera	2002
3886	0	1	1	683	Tomaslanik	2002
3887	0	1	1	684	Yu Shianghuang	2003
3888	0	1	1	685	Charliemiller	2002
3889	0	1	1	686	Veselinkiselkov	2002
3890	0	1	1	687	Christopherli	2003
3891	0	1	1	688	Attila Dominikboros	2003
3892	0	1	1	689	Haktangarayev	2003
3893	0	1	1	690	Ajaymalik	2003
3894	0	1	1	691	Samirhamza reguig	2002
3895	0	1	1	692	Arnavpathange	2002
3896	0	1	1	693	Henriquefukushima	2003

3897	0	1	1	694	Tomasgomez gomez	2002
3898	0	1	1	695	Alessiobasile	2004
3899	0	1	1	696	Theogabbanini	2002
3900	0	1	1	697	Louisskupien	2002
3901	0	1	1	698	Maigao	2003
3902	0	1	1	699	Vencen'tcha	2002
3903	0	1	1	700	Maximgroysman	2002
3904	0	1	1	701	Vladislavgorbatenko	2003
3905	0	1	1	702	Lukajovanovic	2004
3906	0	1	1	703	Yaroslavdemin	2005
3907	0	1	1	704	Eliseykalashnikov	2002
3908	0	1	1	705	Aleksakrivokapic	2003
3909	0	1	1	706	Leroyyong	2004
3910	0	1	1	707	Jonasgreif	2002
3911	0	1	1	708	Nicolasjadoun	2002
3912	0	1	1	709	Tatendamutetwa	2003
3913	0	1	1	710	Janpucalka	2002
3914	0	1	1	711	Maximshin	2003
3915	0	1	1	712	Gianlucaballotta	2004
3916	0	1	1	713	Williamjansen	2004
3917	0	1	1	714	Aramnoroozian	2003
3918	0	1	1	715	Kaipomarshall	2002
3919	0	1	1	716	Kaancepel	2002
3920	0	1	1	717	Eliottroche	2002
3921	0	1	1	718	Calinpostea	2002
3922	0	1	1	719	Garvbahl	2004
3923	0	1	1	720	Maximolucentini	2003
3924	0	1	1	721	Quinnsnyder	2002
3925	0	1	1	722	Alexpadro parra	2004
3926	0	1	1	723	Amorjasika	2004
3927	0	1	1	724	Alexanderblockx	2005
3928	0	1	1	725	Maxgraham	2002
3929	0	1	1	726	Tudor Vladvasilache	2002
3930	0	1	1	727	Razhaviv	2002
3931	0	1	1	728	Kodjo Charlesalipoe-tchotchodj	2002
3932	0	1	1	729	Jackmccarthy	2002
3933	0	1	1	730	Gustavoschwebel	2002
3934	0	1	1	731	Thibautgadilhe	2002
3935	0	1	1	732	Nikitafilatov	2003
3936	0	1	1	733	Christian Andre Shengliew	2004
3937	0	1	1	734	Leopuljic	2002
3938	0	1	1	735	Kirillkistanov	2002
3939	0	1	1	736	Gianlucamarin	2003
3940	0	1	1	737	Tomparis	2002
3941	0	1	1	738	Adamkresina	2004
3942	0	1	1	739	Emiliobravo	2004
3943	0	1	1	740	Markusmolder	2005
3944	0	1	1	741	Abd Elrahmanhassan	2003
3945	0	1	1	742	Drazenpetrovic	2002
3946	0	1	1	743	Yangxuanli	2003

3947	0	1	1	744	Renato Estebangonzalez jorque	2002
3948	0	1	1	745	Karlokrolo	2002
3949	0	1	1	746	Shomakita	2003
3950	0	1	1	747	Oliverojakaar	2005
3951	0	1	1	748	Gokalpayar	2003
3952	0	1	1	749	Lisandrurodriguez	2002
3953	0	1	1	750	Deniskozlov	2003
3954	0	1	1	751	Vanshbhagtani	2002
3955	0	1	1	752	Marcelomarino hidalgo	2004
3956	0	1	1	753	Derickominde	2003
3957	0	1	1	754	Charliepenman	2002
3958	0	1	1	755	Lunobrul	2004
3959	0	1	1	756	Stefanskobelev	2004
3960	0	1	1	757	Paulcolin	2003
3961	0	1	1	758	Yuataka	2003
3962	0	1	1	759	Davidbagrinovskiy	2002
3963	0	1	1	760	Florianarmand	2003
3964	0	1	1	761	Martenmurrand	2003
3965	0	1	1	762	Igormelik agamalov	2002
3966	0	1	1	763	souleymaneouattara nafiralo	2003
3967	0	1	1	764	Ivansodan	2004
3968	0	1	1	765	Zacheryfoster	2002
3969	0	1	1	766	Tristansarap	2003
3970	0	1	1	767	Egortsarapkin	2003
3971	0	1	1	768	Miguel Angelalonso	2003
3972	0	1	1	769	Robertomiceli	2002
3973	0	1	1	770	Hans Christianbustiman gonzale	2003
3974	0	1	1	771	Mihailosavic	2003
3975	0	1	1	772	Min Jaekim	2004
3976	0	1	1	773	Marcelloserafini	2002
3977	0	1	1	774	Ioanniskountourakis	2002
3978	0	1	1	775	Maciejkos	2004
3979	0	1	1	776	Emiliovila	2003
3980	0	1	1	777	Malanqidu	2002
3981	0	1	1	778	Mateodel pino	2003
3982	0	1	1	779	Amitvales	2005
3983	0	1	1	780	Lucioratti	2004
3984	0	1	1	781	Guillaumedalmasso	2002
3985	0	1	1	782	Jacobbradshaw	2002
3986	0	1	1	783	Nathanhoarau	2002
3987	0	1	1	784	Williamcooksey	2003
3988	0	1	1	785	Andresteinbach	2003
3989	0	1	1	786	Erboleldiyar uulu	2002
3990	0	1	1	787	Samuelreichbach	2003
3991	0	1	1	788	Karoltomanski	2003
3992	0	1	1	789	Adamheinonen	2002
3993	0	1	1	790	Junhyeonlee	2002
3994	0	1	1	791	Tuomasrikkonen	2002
3995	0	1	1	792	Tarasignatiuk	2003
3996	0	1	1	793	Sushantdabas	2003

3997	0	1	1	794	Francisco Javierborbolla	2003
3998	0	1	1	795	Daniilromanov	2002
3999	0	1	1	796	Alexanderchernomaz	2004
4000	0	1	1	797	Ryotakaneda	2003
4001	0	1	1	798	Joshuamiller	2002
4002	0	1	1	799	Niccolociavarella	2004
4003	0	1	1	800	Felipelopez comper	2003
4004	0	0	1	801	Santiagogiamichelle	2003
4005	0	0	1	802	Simonmalis	2003
4006	0	0	1	803	Dong Hwankim	2003
4007	0	0	1	804	Bryanaguiar kuntz	2002
4008	0	0	1	805	Zhanzheng	2003
4009	0	0	1	806	Joseftruhlar	2003
4010	0	0	1	807	Armandosotelo	2004
4011	0	0	1	808	Sebastiandefilippis	2002
4012	0	0	1	809	Vladimirpopov	2003
4013	0	0	1	810	Eduardomorais	2003
4014	0	0	1	811	Seydinaandre	2003
4015	0	0	1	812	Rohanloubser	2004
4016	0	0	1	813	Islamorynbasar	2004
4017	0	0	1	814	Maxbatyutenko	2005
4018	0	0	1	815	Connorkruger	2003
4019	0	0	1	816	Nauvaldo Jatiagatra	2003
4020	0	0	1	817	Musamohammed	2002
4021	0	0	1	818	Franmitrovic	2003
4022	0	0	1	819	Cesarbouchelaghem	2004
4023	0	0	1	820	Matthewrobinson	2002
4024	0	0	1	821	Michaelyounan	2002
4025	0	0	1	822	Jakubsvoboda	2003
4026	0	0	1	823	Moritzstoeger	2002
4027	0	0	1	824	Valentinbasel	2004
4028	0	0	1	825	Filipmandovic	2003
4029	0	0	1	826	Alexanderwatanabe eriksson	2003
4030	0	0	1	827	Gabrieledatei	2003
4031	0	0	1	828	Dylanandrianaly	2002
4032	0	0	1	829	Guillaumejacob	2004
4033	0	0	1	830	Richardbiagiotti	2003
4034	0	0	1	831	Luisfrancisco	2003
4035	0	0	1	832	Olimjonnabiev	2002
4036	0	0	1	833	Gunukkang	2004
4037	0	0	1	834	Alessiovasquez gehrke	2003
4038	0	0	1	835	Jettleong	2004
4039	0	0	1	836	Romaingales	2004
4040	0	0	1	837	Matheuscosta paiva	2002
4041	0	0	1	838	Khomtatkitthachok	2004
4042	0	0	1	839	Vladislavtasenkov	2002
4043	0	0	1	840	Alejandrolicea	2004
4044	0	0	1	841	Antonstendal	2003
4045	0	0	1	842	Davidminnivaliev	2003
4046	0	0	1	843	Pedro Antoniosavelli cardoso	2003

4047	0	0	1	844	Matthewrankin	2004
4048	0	0	1	845	Jamessim	2003
4049	0	0	1	846	Mertarabaci	2002
4050	0	0	1	847	Luis Carlosalvarez valdes	2004
4051	0	0	1	848	Boopathysakthivel	2002
4052	0	0	1	849	Derinacaroglu	2003
4053	0	0	1	850	Victor Agustincortes flores	2002
4054	0	0	1	851	Pedrofranca	2002
4055	0	0	1	852	Mateoberta	2003
4056	0	0	1	853	Berktunali	2003
4057	0	0	1	854	Kaylanbigun	2006
4058	0	0	1	855	Zigakovacic	2002
4059	0	0	1	856	Paulbarbier gazeu	2005
4060	0	0	1	857	Enriquepena	2004
4061	0	0	1	858	Jaysonmbogoro	2004
4062	0	0	1	859	Jun Hahyun	2003
4063	0	0	1	860	Artursoghoyan	2002
4064	0	0	1	861	Dawidwisniewski	2002
4065	0	0	1	862	Ryan Atetorandiek	2002
4066	0	0	1	863	Henrique Silvestregomes	2002
4067	0	0	1	864	Adityabalsekar	2002
4068	0	0	1	865	Ryukitsutsumi	2003
4069	0	0	1	866	Berk Ulasenc	2002
4070	0	0	1	867	Illyamaksymchuk	2004
4071	0	0	1	868	Tule khanh duy	2002
4072	0	0	1	869	Myroslavbondar	2002
4073	0	0	1	870	Bogdanseleznev	2002
4074	0	0	1	871	Diegoduran	2004
4075	0	0	1	872	Saiprakashgoli	2002
4076	0	0	1	873	Goranzivotic	2002
4077	0	0	1	874	Olivertarvet	2003
4078	0	0	1	875	Freddieridout	2004
4079	0	0	1	876	Tarmrojanasoonthon	2003
4080	0	0	1	877	Danieltorres munoz	2003
4081	0	0	1	878	Miloskarol	2002
4082	0	0	1	879	Leonberg	2003
4083	0	0	1	880	Abdullah Amr Mohamedgened	2003
4084	0	0	1	881	Rahuldesai	2003
4085	0	0	1	882	Thomasnavarro	2002
4086	0	0	1	883	Jacobibain	2003
4087	0	0	1	884	Georgiosnouchakis	2003
4088	0	0	1	885	Radovanmichalik	2003
4089	0	0	1	886	Juan Diegorojas mora	2003
4090	0	0	1	887	Mattiabernardi	2002
4091	0	0	1	888	Alejandroturriziani alvarez	2003
4092	0	0	1	889	Chunlam	2002
4093	0	0	1	890	Denyszamaraiev	2003
4094	0	0	1	891	Taehajang	2002
4095	0	0	1	892	Tsihonfedorov	2005
4096	0	0	1	893	Lucasgonzalez ramirez	2003

4097	0	0	1	894	Alenbill	2003
4098	0	0	1	895	Alimounir	2003
4099	0	0	1	896	Yu Hanhsieh	2004
4100	0	0	1	897	Emmanueljebutu	2004
4101	0	0	1	898	Luis Felipemiguel	2004
4102	0	0	1	899	Kaerihernandez mariona	2003
4103	0	0	1	900	Henadzihrabavets	2002
4104	0	0	1	901	Yen-Hsunlin	2002
4105	0	0	1	902	Roanjones	2003
4106	0	0	1	903	Alexandru Filipbucurescu	2002
4107	0	0	1	904	Guilhermesousa severin	2002
4108	0	0	1	905	Maxwellsmith	2002
4109	0	0	1	906	Michaelstrestian stamati	2002
4110	0	0	1	907	Toganmakzume	2002
4111	0	0	1	908	Tauheedbrowning	2002
4112	0	0	1	909	Lucky Candrakurniawan	2003
4113	0	0	1	910	Aryanesmaeilpourfallahi	2002
4114	0	0	1	911	Juan Manuella serna	2004
4115	0	0	1	912	Juan Jose Germanvargas gutier	2003
4116	0	0	1	913	Jakobcadonau	2002
4117	0	0	1	914	Je Wonjeon	2003
4118	0	0	1	915	Robertsjaricevskis	2002
4119	0	0	1	916	Oskarieerola	2002
4120	0	0	1	917	Rodrigodeleu	2003
4121	0	0	1	918	Thomas Georgebrun	2002
4122	0	0	1	919	Griffindaehnke	2002
4123	0	0	1	920	Haithemrabbouche	2004
4124	0	0	1	921	Rohanmehra	2002
4125	0	0	1	922	Ishaanravichander	2002
4126	0	0	1	923	Vladimircherkovets	2002
4127	0	0	1	924	Williamjesser	2002
4128	0	0	1	925	Artiomdorofeev	2004
4129	0	0	1	926	Asahiharazaki	2004
4130	0	0	1	927	Ping Chenlin	2002
4131	0	0	1	928	Valentin Manuelgreco	2003
4132	0	0	1	929	Yerassylyerdilda	2005
4133	0	0	1	930	Jacksonarmistead	2004
4134	0	0	1	931	Omermann	2003
4135	0	0	1	932	Rudyceccon	2004
4136	0	0	1	933	Stefansimeunovic	2004
4137	0	0	1	934	Nicolasmoser	2002
4138	0	0	1	935	Idrisshaddouch	2004
4139	0	0	1	936	Jansimonsson	2003
4140	0	0	1	937	Husseinel tawil	2003
4141	0	0	1	938	Lucasphanthala	2004
4142	0	0	1	939	Andrejnedic	2004
4143	0	0	1	940	Dogeonpark	2003
4144	0	0	1	941	Zyadhamed	2003
4145	0	0	1	942	Gerardcampana lee	2004
4146	0	0	1	943	Dimitrijezubac	2002

4147	0	0	1	944	Ahmedkamil	2002
4148	0	0	1	944	Rohanbelday	2006
4149	0	0	1	946	Ke Chentseng	2002
4150	0	0	1	947	Saidakbarov	2004
4151	0	0	1	948	Milanmacura	2002
4152	0	0	1	949	Grigoryshebekin	2005
4153	0	0	1	950	Artemcherepin	2004
4154	0	0	1	951	Keitaduclos	2003
4155	0	0	1	952	Illyasarafyn	2002
4156	0	0	1	953	Eric Davidverdes	2004
4157	0	0	1	954	Kaitaodani	2002
4158	0	0	1	955	Josephphillips	2005
4159	0	0	1	956	Arthusde la bassetiere	2003
4160	0	0	1	957	Stefanandrei	2002
4161	0	0	1	958	Sandroszic	2003
4162	0	0	1	959	Mateoguerineau	2002
4163	0	0	1	960	Wen-Yichou	2004
4164	0	0	1	961	Enriquegarcia	2002
4165	0	0	1	962	Aleksamardjonovic	2002
4166	0	0	1	963	Egorshestakov	2002
4167	0	0	1	964	Adrianluddeckens	2003
4168	0	0	1	965	Prachayabaibong	2002
4169	0	0	1	966	Mathieujosserand	2002
4170	0	0	1	967	Davidqariaqus	2002
4171	0	0	1	968	Chuan Yuchiu	2004
4172	0	0	1	969	Keremozlale	2005
4173	0	0	1	970	Ugniusbickauskis	2002
4174	0	0	1	971	Franciscosinopoli	2004
4175	0	0	1	972	Dylandietrich	2004
4176	0	0	1	973	Mathisjouanneau	2004
4177	0	0	1	974	Chen-Entsai	2002
4178	0	0	1	975	Fatihsari	2003
4179	0	0	1	976	Lovreerceg	2003
4180	0	0	1	977	Kolganovkirill	2004
4181	0	0	1	978	Yitaocao	2003
4182	0	0	1	979	Filippodi perna	2002
4183	0	0	1	980	Hugosalmeron	2003
4184	0	0	1	981	Giovannibranchetti	2003
4185	0	0	1	982	Zixuanli	2002
4186	0	0	1	983	Shogosanada	2005
4187	0	0	1	984	Roykeegan	2005
4188	0	0	1	985	Takumiwatanabe	2005
4189	0	0	1	986	Jae Myeonglee	2002
4190	0	0	1	987	Idogeva	2003
4191	0	0	1	988	Giammarcogandolfi	2004
4192	0	0	1	989	Nicholasgodsick	2004
4193	0	0	1	990	Qiulinwang	2002
4194	0	0	1	991	Jonaszdziopak	2003
4195	0	0	1	992	Remidugardin	2002
4196	0	0	1	993	Mihaelmihaylov	2002

4197	0	0	1	994	Santiagonavarro	2003
4198	0	0	1	995	Patrykstokowski	2003
4199	0	0	1	996	Iustinbelea	2003
4200	0	0	1	997	Lucasschurdevin	2003
4201	0	0	1	998	Lucapow	2005
4202	0	0	1	999	Wiljenkins	2004
4203	0	0	1	1000	Meecahbigun	2006
4204	0	0	1	1001	Maximkhorozov	2003
4205	0	0	1	1002	Archiegraham	2003
4206	0	0	1	1003	Andrescortes	2003
4207	0	0	1	1004	Maximilianwuelfing	2004
4208	0	0	1	1005	Ming-Chiehchiu	2002
4209	0	0	1	1006	Jesseden hartog	2002
4210	0	0	1	1007	Lukakalenic	2002
4211	0	0	1	1008	Guangsenwen	2003
4212	0	0	1	1009	Kareemabdul hakim	2004
4213	0	0	1	1010	Jeroenboer	2002
4214	0	0	1	1011	Thiago Horaciomessa	2004
4215	0	0	1	1012	Min Seongkim	2004
4216	0	0	1	1013	Stepanmruzek	2004
4217	0	0	1	1014	Matteocovato	2005
4218	0	0	1	1015	Tomasquesada perez	2004
4219	0	0	1	1016	Louiefletcher	2003
4220	0	0	1	1017	Paultheate	2002
4221	0	0	1	1018	Gianlucacitadini	2004
4222	0	0	1	1019	Brycerobinson	2002
4223	0	0	1	1020	Stefanoliokossis	2003
4224	0	0	1	1021	Juan Davidduque ramirez	2003
4225	0	0	1	1022	Cameron Austinchang	2003
4226	0	0	1	1023	Nachoserra sanchez	2004
4227	0	0	1	1024	Poldel castillo	2003
4228	0	0	1	1025	Alecbraund	2004
4229	0	0	1	1026	Luis Guilhermecupertino de sar	2003
4230	0	0	1	1027	Aymen Abderrahmeneali mous	2003
4231	0	0	1	1028	Jiangyunhan	2004
4232	0	0	1	1029	Alex-Andreojassalu	2003
4233	0	0	1	1030	Alfredalmasi	2003
4234	0	0	1	1031	Diegonavarro	2002
4235	0	0	1	1032	Arifahresi	2002
4236	0	0	1	1033	Matthias Uwekask	2005
4237	0	0	1	1034	Nicolasvillalon valdes	2004
4238	0	0	1	1035	Leanidboika	2004
4239	0	0	1	1036	Timothylim	2002
4240	0	0	1	1037	Leonardobiasiolo	2002
4241	0	0	1	1038	Quinnvandecasteele	2002
4242	0	0	1	1039	Yumadaijima	2003
4243	0	0	1	1040	Imransibille	2002
4244	0	0	1	1041	Theodorosmitsakos	2004
4245	0	0	1	1042	Maximilianborisov	2002
4246	0	0	1	1043	Danielkhazime	2005

4247	0	0	1	1044	Zamaanmoledina	2003
4248	0	0	1	1045	Henriquepetiz	2003
4249	0	0	1	1046	Mikhailkorkunov	2003
4250	0	0	1	1047	Siwanatauytayakul	2004
4251	0	0	1	1048	Isfandiyormakhmudov	2002
4252	0	0	1	1049	Lachlanrobertson	2004
4253	0	0	1	1050	Semenvoronin	2003
4254	0	0	1	1051	Anapattimangkul	2002
4255	0	0	1	1052	Nikolai Charleshensberg	2002
4256	0	0	1	1053	Gabrielecamilli	2002
4257	0	0	1	1054	Guilhermezotin oliveira	2002
4258	0	0	1	1055	Juliandesousa	2003
4259	0	0	1	1056	Danielverbeek	2004
4260	0	0	1	1057	Haesunlee	2004
4261	0	0	1	1058	Samandarabdumalikov	2003
4262	0	0	1	1059	Ericpadgham	2003
4263	0	0	1	1060	Taonamhwandagara	2003
4264	0	0	1	1061	Aleksapisaric	2005
4265	0	0	1	1062	Harryrock	2003
4266	0	0	1	1063	Ryomamishiro	2003
4267	0	0	1	1064	Natand'hulst	2003
4268	0	0	1	1065	caniceabua	
4269	0	0	1	1066	Jagger Zhen Chihong patzwald	2003
4270	0	0	1	1067	Leonbenedict	2002
4271	0	0	1	1068	Milogazivoda	2003
4272	0	0	1	1069	Marceaufouilhoux	2002
4273	0	0	1	1070	Gustavo Antoniorivas ramirez	2002
4274	0	0	1	1071	Antonkastsenich	2004
4275	0	0	1	1072	Martinpestana	2004
4276	0	0	1	1073	Aljazkirbis	2004
4277	0	0	1	1074	Yassinkarim	2002
4278	0	0	1	1075	Adriandibildox	2003
4279	0	0	1	1076	Emirmologean	2002
4280	0	0	1	1077	Rui Ninghuang	2004
4281	0	0	1	1078	Ottoleikola	2003
4282	0	0	1	1079	Hwijinjung	2002
4283	0	0	1	1080	Simonsedliak	2002
4284	0	0	1	1081	Jandimitrijevic	2003
4285	0	0	1	1082	Guilhermerickli da matta	2004
4286	0	0	1	1083	Gabrielebombara	2002
4287	0	0	1	1084	Mikhailmalakhovich	2002
4288	0	0	1	1085	Ethanquinn	2004
4289	0	0	1	1086	Sebastiantrigosso mercado	2002
4290	0	0	1	1087	Brendan Nicholashendrata	2005
4291	0	0	1	1088	Yakovzhornist	2003
4292	0	0	1	1089	Henryren	2004
4293	0	0	1	1090	Matijasamardzic	2004
4294	0	0	1	1091	Yi-Xianghuang	2002
4295	0	0	1	1092	Yannikkelm	2005
4296	0	0	1	1093	Janprihoda	2002

4297	0	0	1	1094	Olegsyrovatskiy	2003
4298	0	0	1	1095	Gabrieldebru	2005
4299	0	0	1	1096	Julienfabre	2002
4300	0	0	1	1097	Yanickschneider	2002
4301	0	0	1	1098	Robertkorelov	2003
4302	0	0	1	1099	Daniilfelker	2004
4303	0	0	1	1100	Jacksonboone	2002
4304	0	0	1	1101	Theoherrmann	2005
4305	0	0	1	1102	Brianbozemoj	2003
4306	0	0	1	1103	Tobiassmoliner	2002
4307	0	0	1	1104	Diegoschtulmann	2005
4308	0	0	1	1105	Nicolasherrero cuesta	2002
4309	0	0	1	1106	Selimkhouaja	2003
4310	0	0	1	1107	Mohamed Aliabibsi	2002
4311	0	0	1	1108	Alejandrollanos mccallan	2002
4312	0	0	1	1109	Noaheisenberg	2003
4313	0	0	1	1110	Jonathanda silva	2002
4314	0	0	1	1111	Baderalabdullah	2002
4315	0	0	1	1112	Valentinvelazquez	2003
4316	0	0	1	1113	Gokberksaritas	2003
4317	0	0	1	1114	Lucafe d'ostiani	2002
4318	0	0	1	1115	Noamcohen	2002
4319	0	0	1	1116	Matthieuchambonniere	2004
4320	0	0	1	1117	Yumaeda	2005
4321	0	0	1	1118	Daniilzhibul	2003
4322	0	0	1	1119	Eliaspeter	2003
4323	0	0	1	1120	Ollesonesson lidholt	2002
4324	0	0	1	1121	Felipemikkelsen	2002
4325	0	0	1	1122	Songrit Davidchanbubpha	2003
4326	0	0	1	1122	Eitaishikawa	2002
4327	0	0	1	1124	Satorunakajima	2005
4328	0	0	1	1125	Alexanderpatane	2003
4329	0	0	1	1126	Nicolaskobelt	2004
4330	0	0	1	1127	Viniciuspereira rodrigues	2003
4331	0	0	1	1128	Luca- Julianhotze	2003
4332	0	0	1	1129	Benweintraub	2003
4333	0	0	1	1130	Jaredphillips	2002
4334	0	0	1	1131	Diogomorais	2004
4335	0	0	1	1132	Santiagopadilla cote	2005
4336	0	0	1	1133	Basilisvoltirakis	2003
4337	0	0	1	1134	Jacobdubas	2005
4338	0	0	1	1135	Birtanduran	2004
4339	0	0	1	1136	Matias Eliasjuri	2004
4340	0	0	1	1137	Aleksandarljubojevic	2002
4341	0	0	1	1138	Callumgale	2002
4342	0	0	1	1139	Ryotarokoshiba	2002
4343	0	0	1	1140	Valerijsvorobjovs	2002
4344	0	0	1	1141	Noahregas luis	2004
4345	0	0	1	1142	Levlevyatov	2002
4346	0	0	1	1143	Pierrelieutaud	2004

4347	0	0	1	1144	Sukann	2002
4348	0	0	1	1145	Emilpristauz	2002
4349	0	0	1	1145	Harusonton	2003
4350	0	0	1	1147	Pavelkelo	2003
4351	0	0	1	1148	Danilaishchanka	2002
4352	0	0	1	1149	Savelirachalouski	2004
4353	0	0	1	1150	Kacperszymkowiak	2004
4354	0	0	1	1151	Willmayew	2004
4355	0	0	1	1152	Mouadnaoui	2002
4356	0	0	1	1153	Kyleweekes	2002
4357	0	0	1	1154	Dusanmilanovic	2002
4358	0	0	1	1155	Yoshinobunakatsuji	2002
4359	0	0	1	1156	Paulogutierrez ruperti	2002
4360	0	0	1	1157	Rayenhermassi	2004
4361	0	0	1	1158	Davidtesic	2002
4362	0	0	1	1158	Jonatansztasak	2003
4363	0	0	1	1160	Johanncoetzee	2002
4364	0	0	1	1161	Tinashetaruberekera	2002
4365	0	0	1	1162	Viktorjovic	2002
4366	0	0	1	1163	Polotin	2002
4367	0	0	1	1164	Stepanbaum	2003
4368	0	0	1	1165	Lucaparenti	2003
4369	0	0	1	1166	Danielpersson	2003
4370	0	0	1	1167	Ikerurribarrens ramirez	2004
4371	0	0	1	1168	Simonevaccari	2002
4372	0	0	1	1169	Giannibiagianti	2002
4373	0	0	1	1170	Ahmedzaki	2005
4374	0	0	1	1171	Yonibidny	2004
4375	0	0	1	1172	Martinbenes	2004
4376	0	0	1	1173	Rayenzribi	2003
4377	0	0	1	1174	Christopheclement	2004
4378	0	0	1	1174	Darius Florinpop	2004
4379	0	0	1	1176	Aayush Pbhat	2005
4380	0	0	1	1177	Nicolafilippi	2003
4381	0	0	1	1178	Darcynicholls	2003
4382	0	0	1	1179	Andremegrabian	2005
4383	0	0	1	1180	Williambinnie	2002
4384	0	0	1	1181	Fayzullomirzaganiyev	2003
4385	0	0	1	1182	Hayatoshimizu	2004
4386	0	0	1	1183	Marianokpodeziaou	2004
4387	0	0	1	1184	Ahmedhassairi	2005
4388	0	0	1	1185	Andreaschapides	2003
4389	0	0	1	1186	Noahmalige	2002
4390	0	0	1	1187	Filipposzachiotis	2002
4391	0	0	1	1187	Axeleriksson	2004
4392	0	0	1	1189	Asahiyamanaka	2003
4393	0	0	1	1190	Yifengli	2004
4394	0	0	1	1191	Danielwessels	2003
4395	0	0	1	1192	Stephenslocombe	2003
4396	0	0	1	1193	Pierofernandez moreno	2004

4397	0	0	1	1194	Peterpolitis	2003
4398	0	0	1	1195	Jean-Christianmorandais	2003
4399	0	0	1	1196	Juliuskostopoulos	2003
4400	0	0	1	1197	Kylekang	2005
4401	0	0	1	1198	Siddharthjadli	2002
4402	0	0	1	1198	Theopapamalamis	2006
4403	0	0	1	1200	Marselznuderl	2003
4404	0	0	1	1201	Alessandrocadena	2002
4405	0	0	1	1202	Gustavsdambins	2005
4406	0	0	1	1203	muhammad ali akbarramadhan	2002
4407	0	0	1	1204	Ivankremenchutskyi	2004
4408	0	0	1	1205	Kianvakili	2003
4409	0	0	1	1206	Pierreboullenger	2002
4410	0	0	1	1207	Andyhernandez	2003
4411	0	0	1	1208	Gillianosmont	2003
4412	0	0	1	1209	Jose Emilianomontilla garces	2003
4413	0	0	1	1210	Mihavetrih	2002
4414	0	0	1	1211	Tahamesselmani	2003
4415	0	0	1	1212	Alejandroverdasco	2004
4416	0	0	1	1212	Thomascardona herrera	2004
4417	0	0	1	1214	Nicolasgoenaga gonzalez	2002
4418	0	0	1	1215	Luis Fernandoreis	2002
4419	0	0	1	1216	Vanjahodzic	2003
4420	0	0	1	1217	Casparjalili mikkelsen	2003
4421	0	0	1	1218	Dmitrydolzhenkov	2004
4422	0	0	1	1219	Fabionestola	2005
4423	0	0	1	1220	Luca-Danielconstantin	2003
4424	0	0	1	1221	Gjorgjijankulovski	2002
4425	0	0	1	1222	Krishpatel	2002
4426	0	0	1	1223	Danielpaveska	2002
4427	0	0	1	1224	Federicobondioli	2005
4428	0	0	1	1224	Martinlandaluce	2006
4429	0	0	1	1226	Dusanbaranec	2003
4430	0	0	1	1227	Davidschwarc	2005
4431	0	0	1	1228	Lucianoalcocer	2004
4432	0	0	1	1229	Nicokleber	2002
4433	0	0	1	1230	Oscarlacides	2004
4434	0	0	1	1231	Matthewharper	2003
4435	0	0	1	1232	Stefanbianchet	2002
4436	0	0	1	1233	Kaiwenjiang	2002
4437	0	0	1	1234	Amirmohammadkachabi	2002
4438	0	0	1	1235	Benjamintorrealba	2005
4439	0	0	1	1236	Andrewpereverzev	2002
4440	0	0	1	1237	Manavsreekumar	2002
4441	0	0	1	1238	Matthewjohnstone	2004
4442	0	0	1	1239	Rifqy Sukma Ramadhansumiars	2002
4443	0	0	1	1240	Arystanbekkaparov	2003
4444	0	0	1	1241	Kai-iwang	2004
4445	0	0	1	1242	Bautistarodriguez aguilar	2002
4446	0	0	1	1243	Shuannontricerri	2003

4447	0	0	1	1243	Nicolaspinzon moreno	2003
4448	0	0	1	1245	Joaoferreira	2002
4449	0	0	1	1246	Aryangiri	2005
4450	0	0	1	1247	Paul Brunelwamba ngouadjio	2003
4451	0	0	1	1248	Leonardogagliani	2003
4452	0	0	1	1249	Adrianodzhenev	2005
4453	0	0	1	1250	Alex Cosminpavel	2002
4454	0	0	1	1251	Jonasgundacker	2002
4455	0	0	1	1252	Tobiaswallin	2004
4456	0	0	1	1253	Jayzwinkels	2002
4457	0	0	1	1254	Chukwudumebismarty	2002
4458	0	0	1	1255	Yahorslizevich	2003
4459	0	0	1	1256	Lucateboul	2002
4460	0	0	1	1257	Lukasjanousek	2004
4461	0	0	1	1258	Quoc Uyvo	2004
4462	0	0	1	1259	Zivmoravia	2002
4463	0	0	1	1260	Toprakavcibasi	2004
4464	0	0	1	1261	Lucalemaitre vilchis	2005
4465	0	0	1	1262	Danielsancho arbizu	2003
4466	0	0	1	1263	Ethanmuza	2003
4467	0	0	1	1263	Henrikbladelius	2004
4468	0	0	1	1265	Stephankortenhof	2002
4469	0	0	1	1266	Maanavjain	2003
4470	0	0	1	1267	Luisfernandez	2003
4471	0	0	1	1268	Kanwatanabe	2002
4472	0	0	1	1269	Johannrakotomaharo	2003
4473	0	0	1	1270	Ghouse Alifaisal	2003
4474	0	0	1	1271	Ethanfarlie	2002
4475	0	0	1	1272	Lorenzoferri	2004
4476	0	0	1	1273	Zavanfahim	2003
4477	0	0	1	1274	Sachaorlowski	2004
4478	0	0	1	1275	Deancooper kirby	2003
4479	0	0	1	1276	Alvarochavez	2002
4480	0	0	1	1277	Iliyanradulov	2005
4481	0	0	1	1277	Abdelrahmanargoun	2005
4482	0	0	1	1279	Riccardotrione	2002
4483	0	0	1	1280	Yassinetazi	2002
4484	0	0	1	1281	Arjunsriram	2002
4485	0	0	1	1282	Amineel kholti	2002
4486	0	0	1	1283	CHIA-CHIEHlin	2003
4487	0	0	1	1284	Danilpanarin	2005
4488	0	0	1	1285	Mikailalimli	2003
4489	0	0	1	1286	Lambrospantzaris	2003
4490	0	0	1	1287	Joshuanuyts	2004
4491	0	0	1	1288	Williamalsterback	2003
4492	0	0	1	1289	Elwingauffin	2004
4493	0	0	1	1290	Rajatathamcharoensatit	2005
4494	0	0	1	1291	Willemroosenschoon	2003
4495	0	0	1	1291	Juan Ignaciolombisano	2003
4496	0	0	1	1291	Efecomu	2002

4497	0	0	1	1291	Benedictbadza	2005
4498	0	0	1	1295	Jose Franciscogonzalez noriega	2002
4499	0	0	1	1296	Faycelkaddour	2003
4500	0	0	1	1297	Mihnea Petruzaharia	2003
4501	0	0	1	1298	Tsimafeikuksau	2002
4502	0	0	1	1299	Indreksoome	2003
4503	0	0	1	1300	Tsegaigebremeskel	2004
4504	0	0	1	1301	Davidfix	2005
4505	0	0	1	1302	Nathanjattavapornvanit	2002
4506	0	0	1	1303	Kilianmaitre	2002
4507	0	0	1	1304	Louiscloud	2003
4508	0	0	1	1305	Nikolakeremedchiev	2003
4509	0	0	1	1305	Matiasmontanes tutzo	2002
4510	0	0	1	1307	Lukarupnjak	2004
4511	0	0	1	1308	Kevinedengren	2005
4512	0	0	1	1309	Pedromolero	2002
4513	0	0	1	1310	Aleyalkheshen	2003
4514	0	0	1	1311	Adisaginayev	2002
4515	0	0	1	1312	Kael Shalinshah	2004
4516	0	0	1	1313	Ofekshimanov	2005
4517	0	0	1	1314	Sooyoungyang	2004
4518	0	0	1	1315	Paul Arminalbrecht	2002
4519	0	0	1	1316	Alexanderde sousa	2002
4520	0	0	1	1317	Gregorhausberger	2003
4521	0	0	1	1318	Micheal Oluwatosinosewa	2002
4522	0	0	1	1319	gun hyoungkim	2002
4523	0	0	1	1320	Ethandunn	2003
4524	0	0	1	1321	Agustin Ivandamiano	2002
4525	0	0	1	1321	Juho Ericbiggs	2004
4526	0	0	1	1323	Jakenaylor	2002
4527	0	0	1	1324	Jasinjakupi	2002
4528	0	0	1	1325	Pablomontanes gadea	2002
4529	0	0	1	1326	Otsomartikainen	2006
4530	0	0	1	1327	Matveialeksandrov	2002
4531	0	0	1	1328	Lucaebenriter	2003
4532	0	0	1	1329	Diegoortiz gamonal	2003
4533	0	0	1	1330	Wenfuli	2004
4534	0	0	1	1331	Iakovromanovich	2003
4535	0	0	1	1332	Yanakimilev	2004
4536	0	0	1	1333	Alexganchev	2002
4537	0	0	1	1334	Mersedeli	2004
4538	0	0	1	1335	Siravitsudnate	2002
4539	0	0	1	1336	Gabriel L Vjohnson	2003
4540	0	0	1	1337	Zubinomar	2002
4541	0	0	1	1338	Clementkubiak	2005
4542	0	0	1	1338	Jeidensaidizand	2004
4543	0	0	1	1340	Tobiashajek	2002
4544	0	0	1	1341	Saudalhogbani	2003
4545	0	0	1	1342	Lucaszancheta	2002
4546	0	0	1	1343	Matejdodig	2005

4547	0	0	1	1344	Razvanaghion	2003
4548	0	0	1	1345	Ahmedatef	2002
4549	0	0	1	1346	Petarbelosevic	2003
4550	0	0	1	1347	Quintanvan wijk	2002
4551	0	0	1	1348	Omarali	2004
4552	0	0	1	1349	Pierredjaroueh	2003
4553	0	0	1	1350	Enricobaldisserri	2004
4554	0	0	1	1351	Christianteszari	2004
4555	0	0	1	1352	Jakefellows	2002
4556	0	0	1	1353	Lazardanic	2003
4557	0	0	1	1354	Hayden Khoomenon	2005
4558	0	0	1	1355	Matisamier	2002
4559	0	0	1	1356	Harukiomori cowie	2002
4560	0	0	1	1357	SHU-LUNchang	2002
4561	0	0	1	1358	Stewartaronson	2005
4562	0	0	1	1359	Antonsarunic	2002
4563	0	0	1	1360	Romeohadzimehmedovic	2005
4564	0	0	1	1361	Ethancook	2003
4565	0	0	1	1362	Mathistremblay	2002
4566	0	0	1	1363	Igorvisnevskij	2003
4567	0	0	1	1364	Trinitygrear	2002
4568	0	0	1	1365	Achillesbelkovics	2003
4569	0	0	1	1366	Vedranradonjanin	2002
4570	0	0	1	1367	Kylerhugo	2003
4571	0	0	1	1367	Lucalink	2003
4572	0	0	1	1369	Vadymkonovchuk	2003
4573	0	0	1	1370	Linghaozhang	2003
4574	0	0	1	1371	Arslanbekaitkulov	2004
4575	0	0	1	1372	Danyilshcheblykin	2002
4576	0	0	1	1373	Sebastienliu chung ming	2002
4577	0	0	1	1374	Tiagopires	2005
4578	0	0	1	1375	Adithyasuresh	2005
4579	0	0	1	1376	Samhodges	2002
4580	0	0	1	1377	Jacoblowen	2002
4581	0	0	1	1378	Alibekaidaraly	2002
4582	0	0	1	1379	Alejandroguevara garcia	2003
4583	0	0	1	1380	Talgoodman	2002
4584	0	0	1	1381	Charlescourteau	2002
4585	0	0	1	1382	Andrewchang	2003
4586	0	0	1	1383	Alexandroszervos	2003
4587	0	0	1	1384	Youssefberrajah	2002
4588	0	0	1	1385	Yin-Chihlan	2004
4589	0	0	1	1386	Jellekorthuis	2002
4590	0	0	1	1387	Raphaelmujan	2002
4591	0	0	1	1388	Mikhailgalkin	2002
4592	0	0	1	1389	Valdemar Frederikpape	2003
4593	0	0	1	1390	Patrikjurina	2005
4594	0	0	1	1391	Luca Emanuelknese	2002
4595	0	0	1	1392	Erdener	2004
4596	0	0	1	1393	Victortosetto	2004

4597	0	0	1	1394	Harrycollins	2002
4598	0	0	1	1395	Hayatomatsuoka	2005
4599	0	0	1	1396	Nicholaskanazirev	2002
4600	0	0	1	1397	Federicoserena	2002
4601	0	0	1	1398	Jakesweeney	2002
4602	0	0	1	1399	Justastrainauskas	2002
4603	0	0	1	1400	Caymus Hei Tungchoi	2004
4604	0	0	1	1400	Volodymyriakubenko	2005
4605	0	0	1	1402	Tristanbouquier	2003
4606	0	0	1	1403	Daanvan dijk	2003
4607	0	0	1	1404	Reiyahattori	2005
4608	0	0	1	1405	Alanheughebaert	2004
4609	0	0	1	1405	Gauthiercharollais	2003
4610	0	0	1	1405	Juan Camiloperez gongora	2003
4611	0	0	1	1405	Pranavikkurthy	2003
4612	0	0	1	1405	Karansingh	2003
4613	0	0	1	1410	Chotiwatnaen-udorn	2004
4614	0	0	1	1411	Julesleroux	2005
4615	0	0	1	1412	Gabrielschenekenberg	2004
4616	0	0	1	1413	JOEmisrahi	2004
4617	0	0	1	1414	ALANgonzalez	2004
4618	0	0	1	1415	Khaledfrikha	2006
4619	0	0	1	1416	Benitomassacri	2003
4620	0	0	1	1417	Markojovanovic	2003
4621	0	0	1	1418	Paolorosati	2003
4622	0	0	1	1419	Jorge Eduardopastor arrieta	2002
4623	0	0	1	1420	Paulopocasangre	2004
4624	0	0	1	1421	Juliusratt	2002
4625	0	0	1	1422	FELIPEpinzon moreno	2004
4626	0	0	1	1423	Carel Pieterwessel	2004
4627	0	0	1	1424	Inakicabrera bello	2002
4628	0	0	1	1425	Sanidbrdar	2002
4629	0	0	1	1425	Anarghaganguly	2004
4630	0	0	1	1427	Ivansidorov	2003
4631	0	0	1	1428	Lukakurtovic	2003
4632	0	0	1	1429	Furkankarabulut	2002
4633	0	0	1	1430	Elias Julianwerner	2003
4634	0	0	1	1431	Martinmachala	2003
4635	0	0	1	1432	Richmondarmaah	2002
4636	0	0	1	1433	Adilkarakayev	2003
4637	0	0	1	1434	Nikolajsgalkins	2003
4638	0	0	1	1435	Petarbasic palkovic	2003
4639	0	0	1	1436	Nilsandersson	2002
4640	0	0	1	1437	Jonagitschel	2003
4641	0	0	1	1438	Yevheniibondarenko	2003
4642	0	0	1	1439	Thai Nguyentat	2002
4643	0	0	1	1440	Braydenmichna	2004
4644	0	0	1	1441	Nicojaeger	2003
4645	0	0	1	1442	Heerakvora	2003
4646	0	0	1	1443	Dinkarahodza	2003

4647	0	0	1	1444	Alexanderkarman	2003
4648	0	0	1	1445	Alyel rafie	2004
4649	0	0	1	1446	Viggogrondahl	2003
4650	0	0	1	1447	Pablo Luisbalafas	2002
4651	0	0	1	1447	Mikabrunold	2004
4652	0	0	1	1449	Jackloge	2004
4653	0	0	1	1450	Nikitapertsev	2002
4654	0	0	1	1451	Nadimmotaghedi	2002
4655	0	0	1	1452	Junhyukchang	2002
4656	0	0	1	1453	Yizhou	2005
4657	0	0	1	1454	Yi changlin	2003
4658	0	0	1	1455	Dominykasstonkus	2004
4659	0	0	1	1456	Danielabernethy	2003
4660	0	0	1	1457	Andrebaltazar	2002
4661	0	0	1	1458	Samueldickson	2003
4662	0	0	1	1459	Danielstens	2005
4663	0	0	1	1460	Rossweibull	2002
4664	0	0	1	1460	Firasben chouikha	2003
4665	0	0	1	1462	Vitkalina	2005
4666	0	0	1	1463	Mohamed Rayengmati	2003
4667	0	0	1	1464	Emirsutekin	2002
4668	0	0	1	1465	Yu-Shunlai	2004
4669	0	0	1	1466	Ping-Hochen	2002
4670	0	0	1	1467	Oldrichvejsicky	2004
4671	0	0	1	1468	Yoshinorikobayashi	2002
4672	0	0	1	1468	Motoinaka	2002
4673	0	0	1	1470	Mariomartinez serrano	2004
4674	0	0	1	1471	Johnkim	2004
4675	0	0	1	1472	Sampras Victorrakotondrainibe	2004
4676	0	0	1	1473	Victor Barrosfernandes	2003
4677	0	0	1	1474	Alexkhamrotau	2004
4678	0	0	1	1475	Muhammaddossani	2002
4679	0	0	1	1475	Matheusprust gelbcke	2002
4680	0	0	1	1477	Danialrakhmatullayev	2006
4681	0	0	1	1478	Artemmaltsev	2004
4682	0	0	1	1479	Azizbekniyazov	2003
4683	0	0	1	1480	Alexandervasilyuk	2002
4684	0	0	1	1481	Poliskoursaros	2002
4685	0	0	1	1482	Tommyharrison	2003
4686	0	0	1	1483	Diegomillas	2003
4687	0	0	1	1484	Matsherremans	2004
4688	0	0	1	1485	Aki Zubenrawat	2006
4689	0	0	1	1486	ziangli	2002
4690	0	0	1	1487	Hoyoungroh	2006
4691	0	0	1	1488	Alexgomez goyette	2002
4692	0	0	1	1489	Naoyahonda	2006
4693	0	0	1	1490	Samuelepieri	2002
4694	0	0	1	1491	Lachlanbrain	2003
4695	0	0	1	1492	Filipapltauer	2004
4696	0	0	1	1493	Bon Loukarstens	2005

4697	0	0	1	1494	Leonardorossi	2002
4698	0	0	1	1495	Joaofonseca	2006
4699	0	0	1	1496	Andreicuceu	2003
4700	0	0	1	1497	Lukasvelik	2005
4701	0	0	1	1498	Oskarstumpf	2003
4702	0	0	1	1499	Danilamoroz	2002
4703	0	0	1	1499	Asger Emilildor	2003
4704	0	0	1	1501	FOTIOS ANDREASantoniadis	2002
4705	0	0	1	1502	Lathantreacy	2003
4706	0	0	1	1503	Mohamed Belalsoliman	2004
4707	0	0	1	1504	Ismoilhayriddinov	2004
4708	0	0	1	1505	Yegorbilyy	2005
4709	0	0	1	1506	Eugenemurat	2003
4710	0	0	1	1507	Matisde sousa lecorre	2002
4711	0	0	1	1508	Elliotaltman	2002
4712	0	0	1	1509	Samyprot	2004
4713	0	0	1	1510	Supasetsincharoenwattana	2004
4714	0	0	1	1511	Filiptatera	2003
4715	0	0	1	1512	Cesar Augustohenao manrique	2003
4716	0	0	1	1513	Markomesarovic	2004
4717	0	0	1	1514	Azariahrusher	2004
4718	0	0	1	1514	Siddharthalama	2005
4719	0	0	1	1514	Julius Christianseifert	2003
4720	0	0	1	1514	Ericliao	2003
4721	0	0	1	1518	Benjaminscott	2004
4722	0	0	1	1518	Thorbensannen	2002
4723	0	0	1	1520	Enriquelaennec molins	2005
4724	0	0	1	1521	Alexeystarchenkov	2002
4725	0	0	1	1522	Vojtechburian	2003
4726	0	0	1	1523	Santiagoarredondo delgado	2003
4727	0	0	1	1523	Oscar Andresperez gil	2004
4728	0	0	1	1525	Alexanderorlov	2004
4729	0	0	1	1526	Montygraveney	2002
4730	0	0	1	1527	Philippemercelina	2002
4731	0	0	1	1527	Kristofkincses	2003
4732	0	0	1	1527	Nishanthbasavareddy	2002
4733	0	0	1	1530	Nikolastvedt	2002
4734	0	0	1	1531	Alexanderlee	2002
4735	0	0	1	1532	Zacharywhite	2002
4736	0	0	1	1533	Luigicastelletti	2002
4737	0	0	1	1534	Amirmilushev	2005
4738	0	0	1	1535	Juliuszherok	2003
4739	0	0	1	1536	Linusvidehult	2003
4740	0	0	1	1537	Dorianley	2005
4741	0	0	1	1538	Alexandrever hulst	2002
4742	0	0	1	1539	Kanikajayathilake	2004
4743	0	0	1	1540	Oliversaarinen	2002
4744	0	0	1	1540	Davidtomic	2002
4745	0	0	1	1540	Zacharymurphy	2004
4746	0	0	1	1543	Alexandru Raresgiurea	2004

4747	0	0	1	1544	Danielivanov	2004
4748	0	0	1	1545	Johnlasanajak	2004
4749	0	0	1	1546	Skandermezouar	2005
4750	0	0	1	1547	Dylanbruce	2004
4751	0	0	1	1547	Tajhibbert	2004
4752	0	0	1	1549	Valentinfaure	2005
4753	0	0	1	1550	Hon Chunlee	2003
4754	0	0	1	1550	Ruzelfathullin	2002
4755	0	0	1	1552	Mikhaildubrouski	2003
4756	0	0	1	1553	Yunestalavar	2004
4757	0	0	1	1553	Soutaoomura	2002
4758	0	0	1	1555	Zalankhan	2003
4759	0	0	1	1556	Vitalybelanov	2004
4760	0	0	1	1556	Idosamimyan	2005
4761	0	0	1	1556	Kiyan Santinokasemi	2003
4762	0	0	1	1556	Tegar Abdi Satriowibowo	2003
4763	0	0	1	1560	Alexanderedley	2003
4764	0	0	1	1561	Matthewgriscti	2003
4765	0	0	1	1561	Matheocoupu moison	2002
4766	0	0	1	1563	Ivanla cava	2003
4767	0	0	1	1563	Gabrielfriday	2002
4768	0	0	1	1565	Arturivanenko	2003
4769	0	0	1	1566	Lautarosanchez	2004
4770	0	0	1	1567	Danielbaquero	2005
4771	0	0	1	1568	Antonio Matteoiaquinto	2003
4772	0	0	1	1569	Anthonyerbrech	2003
4773	0	0	1	1569	Antoniosinopoli	2002
4774	0	0	1	1571	Seyeed Mohammad Emanhose	2002
4775	0	0	1	1571	Jamesedley	2003
4776	0	0	1	1573	Ivanburnashov	2004
4777	0	0	1	1574	Isseiaoki	2002
4778	0	0	1	1574	Haoduan	2005
4779	0	0	1	1576	Cesarpalafox	2003
4780	0	0	1	1577	Iliavedmed	2005
4781	0	0	1	1578	Calin Teodorstirbu	2006
4782	0	0	1	1579	Williamzulch	2003
4783	0	0	1	1580	Maxbakken	2002
4784	0	0	1	1581	Tomsaporta	2002
4785	0	0	1	1582	Antonioli	2003
4786	0	0	1	1583	Natsukiyamamoto	2005
4787	0	0	1	1584	Davidverkhovskiy	2002
4788	0	0	1	1585	Yanismoundir	2002
4789	0	0	1	1585	Nejcskorjanc	2004
4790	0	0	1	1585	Keijumiyake	2002
4791	0	0	1	1588	Aidendrover-mattinen	2003
4792	0	0	1	1588	Timzelikovsky	2002
4793	0	0	1	1590	Danielmarcano	2002
4794	0	0	1	1591	Tunanergizoglu	2004
4795	0	0	1	1591	Jhoan Sebastianvaquero	2004
4796	0	0	1	1593	Jonahgethin	2004

4797	0	0	1	1593	Erkintootoonchi moghadam	2002
4798	0	0	1	1595	Oliverhall	2002
4799	0	0	1	1596	Jun Hwanlee	2003
4800	0	0	1	1597	Aarav Samrathada	2007
4801	0	0	1	1598	Danieltam	2002
4802	0	0	1	1599	Gianfrancopanta	2002
4803	0	0	0	1600	Arthurgirault	2002
4804	0	0	0	1601	Jirikrouzek	2002
4805	0	0	0	1602	Filipekrohn da silva	2003
4806	0	0	0	1603	Christopheleblanc	2003
4807	0	0	0	1603	Artembabaiev	2003
4808	0	0	0	1605	Fabiantaracena	2002
4809	0	0	0	1605	Florianledoux	2002
4810	0	0	0	1607	Brunoloeffler	2003
4811	0	0	0	1608	Desmondayaaba	2004
4812	0	0	0	1608	Jirijindacek	2003
4813	0	0	0	1610	Dhruvhirpara	2003
4814	0	0	0	1610	Shreejitsen	2003
4815	0	0	0	1612	Igormandou berranger	2005
4816	0	0	0	1612	Itayfeigin	2003
4817	0	0	0	1612	Yakupuzunel	2005
4818	0	0	0	1615	Umit Egecankus	2002
4819	0	0	0	1616	Qinglihao	2004
4820	0	0	0	1616	Jeong-Ankim	2003
4821	0	0	0	1618	Gabrielconstantino	2003
4822	0	0	0	1619	Lefteriapostolou	2002
4823	0	0	0	1620	Rodionmaksiuta	2002
4824	0	0	0	1621	Kevinfu	2003
4825	0	0	0	1621	Damiancraddock	2004
4826	0	0	0	1623	Noelpall	2004
4827	0	0	0	1624	Danielkong	2005
4828	0	0	0	1625	Peterbuldorini	2004
4829	0	0	0	1626	Akbarshokhshukurov	2003
4830	0	0	0	1627	Tomaslopez salazar	2003
4831	0	0	0	1628	Pavlemarinkov	2006
4832	0	0	0	1628	Nikitavoronov	2003
4833	0	0	0	1628	Miloszsroka	2003
4834	0	0	0	1631	Fedimlika	2004
4835	0	0	0	1632	Tsubasakoga	2002
4836	0	0	0	1633	Hugocardinaud	2004
4837	0	0	0	1634	Moeinmoazam	2002
4838	0	0	0	1635	Taraspakharenko	2003
4839	0	0	0	1636	Boyaohuang	2002
4840	0	0	0	1637	Yessinekerouat	2005
4841	0	0	0	1637	Richarddulganov	2002
4842	0	0	0	1637	Ahtialkio	2003
4843	0	0	0	1640	Davidleon moros	2003
4844	0	0	0	1641	Mykolaszilinskas	2002
4845	0	0	0	1641	Patrikmeszaros	2005
4846	0	0	0	1641	hamidisrar gul	2006

4847	0	0	0	1641	Issamjonsharifov	2003
4848	0	0	0	1641	Jamieforrest	2003
4849	0	0	0	1641	Dominicducariu	2005
4850	0	0	0	1641	Matteofuschillo	2003
4851	0	0	0	1641	Samuel Agbesi Oseiantwi	2002
4852	0	0	0	1649	Raunaksood	2002
4853	0	0	0	1649	Peterdragar	2003
4854	0	0	0	1651	Farzanamiri	2003
4855	0	0	0	1652	Lucadobronauteanu	2003
4856	0	0	0	1653	Fabiangusic	2002
4857	0	0	0	1654	Jakelam hau ching	2004
4858	0	0	0	1655	Eliassefiani	2004
4859	0	0	0	1656	Michael Dylanjimenez	2006
4860	0	0	0	1656	Tomas Danielvalencia navech	2003
4861	0	0	0	1658	Razkatz	2003
4862	0	0	0	1658	Aashutoshkulakarni prachet	2002
4863	0	0	0	1658	Yong Joonpark	2002
4864	0	0	0	1658	Juliusleino	2004
4865	0	0	0	1662	Matickriznik	2005
4866	0	0	0	1663	Stefanregalia	2005
4867	0	0	0	1664	Hai Truongvo	2003
4868	0	0	0	1664	Ryuichironakano	2002
4869	0	0	0	1666	Princegandonou	2003
4870	0	0	0	1667	RISHIpaventhan	2002
4871	0	0	0	1668	Justinkuo	2004
4872	0	0	0	1669	Alessandrosant'angelo	2003
4873	0	0	0	1670	Antonifabre	2003
4874	0	0	0	1670	Jonathanberling	2002
4875	0	0	0	1672	Matthewbrowning	2004
4876	0	0	0	1673	Takuramhwandagara	2006
4877	0	0	0	1674	Zi Huichen	2003
4878	0	0	0	1675	Angeloclervil	2002
4879	0	0	0	1675	Naruseminami	2002
4880	0	0	0	1677	Maximedubouch	2003
4881	0	0	0	1677	Jakaprotic zakelj	2003
4882	0	0	0	1677	Albertsaar	2005
4883	0	0	0	1677	Ivanshchepilov	2003
4884	0	0	0	1681	Marcoscastellanos	2005
4885	0	0	0	1682	Pedrode la riva bueno	2003
4886	0	0	0	1682	Rasmusegehus	2003
4887	0	0	0	1684	Laurentschroeder	2002
4888	0	0	0	1685	Markusstions	2002
4889	0	0	0	1686	CHETANprashant gadiyar	2002
4890	0	0	0	1686	Wilson Oswaleleigbinovia	2003
4891	0	0	0	1686	Pumiphatsukkho	2006
4892	0	0	0	1686	Paulovalarezo lingen	2002
4893	0	0	0	1690	Phoenixweir	2005
4894	0	0	0	1690	Sam Kyddbaumgartner	2004
4895	0	0	0	1692	Kirillplatonov	2003
4896	0	0	0	1693	Dimtriosbinopoulos	2002

4897	0	0	0	1694	Dmitriiivanov	2003
4898	0	0	0	1694	Alexanderstokbroekx	2002
4899	0	0	0	1694	Petrospieros	2003
4900	0	0	0	1697	Paoloflores	2002
4901	0	0	0	1697	Riccardotomassetti	2002
4902	0	0	0	1697	Haodajiang	2004
4903	0	0	0	1700	Arseniykhokhlov	2004
4904	0	0	0	1701	Patrickschoen	2005
4905	0	0	0	1702	Santiagosperanza	2002
4906	0	0	0	1703	Visheshpatel	2004
4907	0	0	0	1704	Adamantiosstavrakas	2003
4908	0	0	0	1705	Nithilan Erickinattuvilai	2004
4909	0	0	0	1705	Georgelazarov	2005
4910	0	0	0	1707	Subhan Binsalik	2002
4911	0	0	0	1707	Nathan Akiramatsuguma	2003
4912	0	0	0	1707	Rohamabbassi	2002
4913	0	0	0	1707	Torusrawat	2003
4914	0	0	0	1707	Arnovan droogenbroeck	2003
4915	0	0	0	1712	Jonahbraswell	2004
4916	0	0	0	1713	Alihabib	2002
4917	0	0	0	1713	Liamspiers	2002
4918	0	0	0	1715	Benjaminheynold	2002
4919	0	0	0	1716	Florentlimani	2004
4920	0	0	0	1716	Goranzgola	2005
4921	0	0	0	1716	Denzelseetso	2005
4922	0	0	0	1719	Atakankarahan	2006
4923	0	0	0	1720	Younesadnane	2002
4924	0	0	0	1720	Marco Antoniosommer siviero	2002
4925	0	0	0	1720	Umut Kaanyenilmez	2003
4926	0	0	0	1720	Aleksanderchayka	2004
4927	0	0	0	1724	Massimogiunta	2004
4928	0	0	0	1725	Omarknani	2005
4929	0	0	0	1726	CHENliang	2002
4930	0	0	0	1727	Yuvannandal	2005
4931	0	0	0	1728	Hyeokmin	2004
4932	0	0	0	1728	Stylianospoutis	2002
4933	0	0	0	1728	Vladislavgavrilov	2003
4934	0	0	0	1731	Kyvanrietkerk	2005
4935	0	0	0	1732	Christian Felipewinstead	2003
4936	0	0	0	1733	Gabriel Mauricionolasco pozo	2002
4937	0	0	0	1733	Jovansinadinovic	2003
4938	0	0	0	1733	Albertsegura onichtchenko	2003
4939	0	0	0	1736	Sebastiansorger	2005
4940	0	0	0	1736	Mehdibelmahi	2002
4941	0	0	0	1738	Gauthierprince	2004
4942	0	0	0	1738	Nathancox	2003
4943	0	0	0	1738	Filipstipic	2002
4944	0	0	0	1741	Dario Alexandruciobotaru	2004
4945	0	0	0	1742	Luigibove	2004
4946	0	0	0	1742	Hassanbaroudi	2003

4947	0	0	0	1744	Stjepananisic	2003
4948	0	0	0	1745	Reiniskupfers	2004
4949	0	0	0	1745	Ji Hoonkong	2002
4950	0	0	0	1745	Alexandermarinov	2002
4951	0	0	0	1748	Ianfreire	2002
4952	0	0	0	1748	Ahmedmohamed	2003
4953	0	0	0	1750	Chen-Yulu	2004
4954	0	0	0	1751	Aronpilbart	2004
4955	0	0	0	1752	Seankamyshev	2002
4956	0	0	0	1753	Tobiaswirlend	2003
4957	0	0	0	1753	Zoharyaniv	2005
4958	0	0	0	1755	Nikolamitrovic	2002
4959	0	0	0	1756	Matissebobichon	2002
4960	0	0	0	1756	Arseniynazarov	2004
4961	0	0	0	1756	Stefanod'agostino	2003
4962	0	0	0	1756	Saveliyivanov	2004
4963	0	0	0	1760	Luis Eduardoplatas	2002
4964	0	0	0	1760	Jelanisarr	2004
4965	0	0	0	1760	Karimal-amin	2003
4966	0	0	0	1760	Alexcairo	2004
4967	0	0	0	1760	Yujiroonuma	2004
4968	0	0	0	1760	Matasbruzas	2002
4969	0	0	0	1760	Tomgrosjean	2002
4970	0	0	0	1760	Christosnikoloulis	2002
4971	0	0	0	1768	Eldinhuseinovic	2003
4972	0	0	0	1768	Lucasolivier	2003
4973	0	0	0	1768	Matthewearle	2002
4974	0	0	0	1768	Taurasmickus	2004
4975	0	0	0	1772	Yoavlevy	2003
4976	0	0	0	1772	Shinnosukeiwamoto	2004
4977	0	0	0	1774	Erikchobanyan	2004
4978	0	0	0	1774	Albanlahoreau	2003
4979	0	0	0	1776	Niccolodessi	2004
4980	0	0	0	1776	Marco Antonioazar	2003
4981	0	0	0	1776	Vittoriofaletti	2002
4982	0	0	0	1779	Razvanperial	2002
4983	0	0	0	1780	Muhammad Rizalmuzaqir	2002
4984	0	0	0	1780	Jonathan Zacharylaforest	2002
4985	0	0	0	1782	Omarfakhoury	2003
4986	0	0	0	1783	Nazarfedoryshyn	2004
4987	0	0	0	1783	Matthiasujvary	2004
4988	0	0	0	1783	Zaneford	2004
4989	0	0	0	1783	Williamhatzis	2003
4990	0	0	0	1783	Mateonavarro	2003
4991	0	0	0	1783	yoontaeklee	2003
4992	0	0	0	1789	Jankopcik	2003
4993	0	0	0	1790	Andreatiburzio	2004
4994	0	0	0	1791	Markuskosak	2003
4995	0	0	0	1792	Jaovan schalkwyk	2005
4996	0	0	0	1793	Diego Josesegovia perez	2003

4997	0	0	0	1793	Ruslanserazhetdinov	2005
4998	0	0	0	1793	Lucasperrang	2003
4999	0	0	0	1793	Oliviermancewicz	2003
5000	0	0	0	1797	Mustaphabenmira	2002
5001	0	0	0	1797	Qwynquittner	2004
5002	0	0	0	1797	Tombolton	2002
5003	0	0	0	1800	Aleksanderzdzuj	2002
5004	0	0	0	1801	Nickmanopoulos	2002
5005	0	0	0	1802	Herrick Thomaslegaspi	2004
5006	0	0	0	1803	Samchawla	2003
5007	0	0	0	1803	Marc Antoinegaillard	2002
5008	0	0	0	1803	Emilmatikainen	2005
5009	0	0	0	1803	Gheorghe Claudiuschinteie	2002
5010	0	0	0	1803	Igorkurbatov	2002
5011	0	0	0	1808	lucagiordano	2002
5012	0	0	0	1809	Grigoriygubarev	2002
5013	0	0	0	1809	Lucasbitzer	2003
5014	0	0	0	1809	Vanshkapoor	2002
5015	0	0	0	1809	Benjaminfernandez	2002
5016	0	0	0	1813	Davidleon mantilla	2003
5017	0	0	0	1814	Josipsimundza	2004
5018	0	0	0	1815	Maksgrygolowicz	2003
5019	0	0	0	1815	Davidsspaks	2004
5020	0	0	0	1817	Zianvanderstappen	2004
5021	0	0	0	1817	Michaelwalser	2002
5022	0	0	0	1817	Ching Wahwai	2003
5023	0	0	0	1817	Alekseimokrov	2005
5024	0	0	0	1817	Hyunwoonseo	2005
5025	0	0	0	1817	Gabrielzissis	2002
5026	0	0	0	1817	Jose Miguelfernandez martinez	2003
5027	0	0	0	1817	Leonnikolic	2003
5028	0	0	0	1817	Federicoruggeri	2002
5029	0	0	0	1817	Chan Heehan	2005
5030	0	0	0	1817	Rudra Himendubhatt	2002
5031	0	0	0	1828	Mohammadalkotop	2005
5032	0	0	0	1828	Aleksandartemov	2002
5033	0	0	0	1828	Nikitazavhorodko	2003
5034	0	0	0	1831	magdeldin	2004
5035	0	0	0	1832	Nirskoletsky	2003
5036	0	0	0	1833	Javierhazouri celorio	2002
5037	0	0	0	1834	Pavelpetrov	2004
5038	0	0	0	1834	Benjamin Thomasgeorge	2002
5039	0	0	0	1834	Tinostro	2003
5040	0	0	0	1834	Aldan Jakobuscoetzee	2002
5041	0	0	0	1834	Andreastimini	2006
5042	0	0	0	1834	Majunli	2005
5043	0	0	0	1840	Finnemslie-robson	2004
5044	0	0	0	1840	Gabrieledolce	2002
5045	0	0	0	1842	Ziadaboufirassi	2002
5046	0	0	0	1843	Davidpolo	2002

5047	0	0	0	1843	Mathieuderache	2004
5048	0	0	0	1845	Lucalo nardo	2004
5049	0	0	0	1845	Markrubin	2003
5050	0	0	0	1845	Zinovarouhas-poole	2004
5051	0	0	0	1845	Danielhammond	2004
5052	0	0	0	1845	Oliverfiallo	2003
5053	0	0	0	1845	Omarfarahat	2005
5054	0	0	0	1851	Hanni Facundoelias	2002
5055	0	0	0	1852	Joaquinperez casamayor	2004
5056	0	0	0	1853	Andreykozlov	2005
5057	0	0	0	1853	Teunrozenberg	2003
5058	0	0	0	1853	Valentinpena	2003
5059	0	0	0	1853	Hod'abalo isakpadio	2005
5060	0	0	0	1853	Jordigarcia mestre	2002
5061	0	0	0	1858	Eyaltoledano	2004
5062	0	0	0	1858	Nicholasheng	2003
5063	0	0	0	1858	Marcmajdandzic	2005
5064	0	0	0	1858	Dominiksimunovic	2002
5065	0	0	0	1858	Jameso'sullivan	2003
5066	0	0	0	1858	Daanmeijer	2005
5067	0	0	0	1864	Oliverrichards	2002
5068	0	0	0	1864	Tsholofelotsiang	2002
5069	0	0	0	1866	Martimsimoes	2003
5070	0	0	0	1866	Artemalekseychuk	2005
5071	0	0	0	1866	Filipegrebentsov	2004
5072	0	0	0	1869	Maxdickey	2003
5073	0	0	0	1869	Mateuszkulakowski	2003
5074	0	0	0	1871	Luis Diegoleon	2003
5075	0	0	0	1871	Juan Fernandocontreras medin	2002
5076	0	0	0	1871	Andyzhu	2003
5077	0	0	0	1874	Dhillonvirdee oakley	2003
5078	0	0	0	1874	Jakubkroslak	2003
5079	0	0	0	1874	Abelforger	2005
5080	0	0	0	1874	Mattia Marcomauriziano abum	2002
5081	0	0	0	1878	Chanathornkangtong	2003
5082	0	0	0	1878	Robertschorr	2002
5083	0	0	0	1878	Botondkisantal	2005
5084	0	0	0	1878	Federicoinfante santana	2002
5085	0	0	0	1882	Robertzhang	2005
5086	0	0	0	1883	Benjaminmartin	2005
5087	0	0	0	1883	Mustafasancaklioglu	2004
5088	0	0	0	1883	Levgorb	2006
5089	0	0	0	1886	Quang Truongtran	2002
5090	0	0	0	1887	Ignaciomontes cuellar	2002
5091	0	0	0	1888	Jacopodenitto	2002
5092	0	0	0	1888	Antoinemarleau	2002
5093	0	0	0	1888	Savaradovic	2002
5094	0	0	0	1888	RYOmurakami	2004
5095	0	0	0	1892	Kartikparhar	2003
5096	0	0	0	1892	Tamasfoldi	2003

5097	0	0	0	1892	Jangjunkim	2006
5098	0	0	0	1895	Paytonyoung	2004
5099	0	0	0	1896	Eudy Franciscoledesma merced	2002
5100	0	0	0	1897	Georgigeorgiev	2006
5101	0	0	0	1897	Grantlumkong	2003
5102	0	0	0	1897	Mohamed Malekcherif	2004
5103	0	0	0	1897	Ervinmesic	2004
5104	0	0	0	1897	Grantlumkong	2003
5105	0	0	0	1902	David Tonyedawariye	2004
5106	0	0	0	1902	Sohrobamiryavari	2003
5107	0	0	0	1902	Pelayorodriguez	2003
5108	0	0	0	1902	Woohyukchang	2005
5109	0	0	0	1902	Miguellarrea sicco	2005
5110	0	0	0	1902	Seungikpark	2003
5111	0	0	0	1908	Marshallhulett	2004
5112	0	0	0	1909	Andre-Remijakobi	2003
5113	0	0	0	1909	Tienthinhtran	2002
5114	0	0	0	1911	Possawatakarapan	2002
5115	0	0	0	1912	Viktorgrigorov	2002
5116	0	0	0	1912	Lars Goranverwerft	2003
5117	0	0	0	1912	Balintdobak	2002
5118	0	0	0	1912	Raffaelebarba	2004
5119	0	0	0	1912	Ismailelshafei	2004
5120	0	0	0	1917	DANIILchumakov	2002
5121	0	0	0	1917	Tomraveh	2003
5122	0	0	0	1917	Lorantgyori	2006
5123	0	0	0	1917	Malharrajpal	2004
5124	0	0	0	1921	Eliasanwar	2004
5125	0	0	0	1922	Lasha Giorgikikvidze	2002
5126	0	0	0	1922	Quentinlamothe	2004
5127	0	0	0	1924	Romankonovalov	2004
5128	0	0	0	1924	Andreameduri	2005
5129	0	0	0	1924	Shingisbayseit	2005
5130	0	0	0	1924	Abbossadikov	2003
5131	0	0	0	1928	Eldinnokic	2004
5132	0	0	0	1928	Andrei Florin Teodorrobitu	2003
5133	0	0	0	1930	Gerardhenning	2004
5134	0	0	0	1930	Lylezaloumis	2004
5135	0	0	0	1930	Mahmut Berabinici	2004
5136	0	0	0	1930	Musasani	2003
5137	0	0	0	1930	Sebastianzabala castrillon	2003
5138	0	0	0	1930	Lukevuletic	2003
5139	0	0	0	1930	Nikitabochkarev	2004
5140	0	0	0	1937	Mehdimezghani	2004
5141	0	0	0	1937	Silhollemans	2003
5142	0	0	0	1937	Gregoriohidalgo	2005
5143	0	0	0	1937	Joelstevens	2003
5144	0	0	0	1937	Ethan Yi Hangleung	2004
5145	0	0	0	1942	Christoszonias	2004
5146	0	0	0	1942	Majnajvirt kolaric	2003

5147	0	0	0	1942	Kavisharatnayake	2002
5148	0	0	0	1945	Lucascosta de lima	2003
5149	0	0	0	1945	Chun-Pohuang	2003
5150	0	0	0	1947	Maxforman	2002
5151	0	0	0	1948	Duvan Santiagotorres villamil	2004
5152	0	0	0	1948	Sebastian Andresrondon gomez	2003
5153	0	0	0	1948	Fedorrichter	2003
5154	0	0	0	1948	Juanjorodriguez garceran	2003
5155	0	0	0	1952	Lucamatheiowetz	2002
5156	0	0	0	1952	Marcomigliorati	2003
5157	0	0	0	1952	Raffaelschaer	2002
5158	0	0	0	1952	Davidtivanovac	2002
5159	0	0	0	1952	Kristiantumbas kajgo	2005
5160	0	0	0	1952	Dimitrispaliouras	2003
5161	0	0	0	1952	Tylerbowers	2002
5162	0	0	0	1959	Marknielsen	2004
5163	0	0	0	1959	Aryanlakshmanan	2005
5164	0	0	0	1959	Fabriziovillegas villarroel	2003
5165	0	0	0	1959	Maximus Zavierwong	2007
5166	0	0	0	1959	Nikolacvetanovic	2003
5167	0	0	0	1959	Gianroccode filippo	2002
5168	0	0	0	1959	Alexanderushakov	2004
5169	0	0	0	1959	Lukekoh	2007
5170	0	0	0	1967	Sami Zebkhan	2004
5171	0	0	0	1967	Claudio Renardilumanau	2004
5172	0	0	0	1967	Nicolasphilibert	2005
5173	0	0	0	1970	Jasonespitalier noel	2002
5174	0	0	0	1971	Adammuller	2004
5175	0	0	0	1971	Williamkesterson	2002
5176	0	0	0	1971	Jakubnovak	2003
5177	0	0	0	1971	Yu-Hsiangchou	2004
5178	0	0	0	1971	Kyubinsakong	2004
5179	0	0	0	1971	Svencorbinais	2002
5180	0	0	0	1971	ZhenChangqin	2003
5181	0	0	0	1971	Maximilianosandoval dominguez	2002
5182	0	0	0	1979	Alexanderfrantzis	2005
5183	0	0	0	1979	Javier Alejandroromero de jesu	2003
5184	0	0	0	1979	Pablopaternostro	2005
5185	0	0	0	1982	Timofeystepanov	2003
5186	0	0	0	1982	Gregorgrebensek	2003
5187	0	0	0	1982	Nikmikovic	2004
5188	0	0	0	1982	Nilesrachal	2002
5189	0	0	0	1982	Albertogrimaldi	2002
5190	0	0	0	1982	Kristofferdalbak	2004
5191	0	0	0	1982	Llucmir anglada	2003
5192	0	0	0	1982	Leonbabic	2004
5193	0	0	0	1982	Lukanovakovic	2004
5194	0	0	0	1982	Hendrikkatmuk	2004
5195	0	0	0	1992	Vandadgheibi	2003
5196	0	0	0	1992	Thompsonthomu	2006

5197	0	0	0	1992	Hugosanchez	2002
5198	0	0	0	1995	Aaronjinks	2002
5199	0	0	0	1995	Burak Canyilmaz	2002
5200	0	0	0	1997	Viacheslavovchinnikov	2002
5201	0	0	0	1997	Andreifiraru	2005
5202	0	0	0	1997	Trisztangubi	2005
5203	0	0	0	2000	Timhammes	2003
5204	0	0	0	2000	Jianhaosheng	2002
5205	0	0	0	2000	Gordan Franmileusnic	2003
5206	0	0	0	2000	Lambertruland	2003
5207	0	0	0	2000	Nuriddinshagiev	2002
5208	0	0	0	2000	Billybougoure	2004
5209	0	0	0	2000	Timhammes	2003
5210	0	0	0	2000	Ethancarr	2004
5211	0	0	0	2000	Borenzheng	2004
5212	0	0	0	2009	Emildapcevic	2005
5213	0	0	0	2009	Benjaminbuck	2002
5214	0	0	0	2009	Yosriabdelmoula	2006
5215	0	0	0	2009	Justus Alexandreagbo	2002
5216	0	0	0	2013	Hoanmouret	2005
5217	0	0	0	2013	Duhyuneum	2003
5218	0	0	0	2013	Giuliovecchiarelli	2003
5219	0	0	0	2013	Luismarquez	2004
5220	0	0	0	2017	Patrickwheat-lindsey	2002
5221	0	0	0	2018	Ahmedel dardeery	2002
5222	0	0	0	2019	Arsentiyyakovlev	2003
5223	0	0	0	2019	Mikolajkonczal	2003
5224	0	0	0	2021	Garethong	2004
5225	0	0	0	2021	Pierre Antoinetailleu	2003
5226	0	0	0	2021	Rodrigodiaz	2002
5227	0	0	0	2021	Yigit Cengizbinici	2003
5228	0	0	0	2021	Naruebodeeboonyawat	2005
5229	0	0	0	2021	Georgeoganov	2002
5230	0	0	0	2021	Srijansingh	2002
5231	0	0	0	2021	Dantedespontin	2002
5232	0	0	0	2021	Fabiovarella	2003
5233	0	0	0	2021	Vatsalrukhaiyar	2004
5234	0	0	0	2021	Jamisavolainen	2005
5235	0	0	0	2032	Steyndippenaar	2003
5236	0	0	0	2032	Andretoomvali	2003
5237	0	0	0	2032	Tristanmesserli	2003
5238	0	0	0	2032	Kristoferjohnson	2002
5239	0	0	0	2036	Josephtownes	2004
5240	0	0	0	2036	Davidsimeonov	2005
5241	0	0	0	2036	Marvin Mang Hinlee	2003
5242	0	0	0	2039	Nikitakim	2004
5243	0	0	0	2039	Robinsonle meur	2003
5244	0	0	0	2041	Ariyojavadi	2004
5245	0	0	0	2041	Mateoquintero castillo	2003
5246	0	0	0	2041	Salimmekki berrada	2003

5247	0	0	0	2041	Yahiaelsheikh	2003
5248	0	0	0	2041	Oribaron	2004
5249	0	0	0	2041	Daniel Emiliotevez arias	2003
5250	0	0	0	2047	Victorpaganetti	2003
5251	0	0	0	2047	Grantlothringer	2003
5252	0	0	0	2049	Filipzilkay	2003
5253	0	0	0	2049	Alessandro Nicolasbizzarri	2004
5254	0	0	0	2051	Davidjovic	2002
5255	0	0	0	2051	Harissahman	2003
5256	0	0	0	2051	Karimfendri	2005
5257	0	0	0	2051	Mehdiabdelmoula	2002
5258	0	0	0	2051	Markopavicic	2004
5259	0	0	0	2051	Ilyas Miladfahim	2005
5260	0	0	0	2051	Julienpenzlin	2002
5261	0	0	0	2051	Oferhacohen	2003
5262	0	0	0	2059	Adityachopra	2004
5263	0	0	0	2059	Petergombas	2003
5264	0	0	0	2059	Achilefs Rafailkousaridis	2005
5265	0	0	0	2059	Jeong Yukim	2004
5266	0	0	0	2059	Nelsonramos ponce	2004
5267	0	0	0	2059	Stanislavvalent	2002
5268	0	0	0	2059	Tsz Himchan	2004
5269	0	0	0	2059	Piotrsiekanowicz	2004
5270	0	0	0	2059	Aneish Josesawney	2003
5271	0	0	0	2059	Deepakvayara	2002
5272	0	0	0	2059	kartiksaxena	2003
5273	0	0	0	2059	Adam Gaborszoke	2004
5274	0	0	0	2059	Dominikkoszegi	2005
5275	0	0	0	2059	Thomaspaulsell	2002
5276	0	0	0	2059	Kirillmishkin	2002
5277	0	0	0	2059	Thehan Sanjayawijemanne	2003
5278	0	0	0	2059	Shivendraperera	2003
5279	0	0	0	2059	Darrenmartis	2002
5280	0	0	0	2059	Shumatsuoka	2003
5281	0	0	0	2059	Maximilianoventura	2002
5282	0	0	0	2059	Roccopiatti	2004
5283	0	0	0	2059	Timothyyeung packer	2004
5284	0	0	0	2081	Devaharshithneelam	2005
5285	0	0	0	2081	Omarabdelmoula	2005
5286	0	0	0	2081	Drisssedrati	2005
5287	0	0	0	2084	Carlos Joaquinramos membrive	2003
5288	0	0	0	2084	Jose Miguelroa rodriguez	2003
5289	0	0	0	2084	Siavashshirdel	2002
5290	0	0	0	2084	Yeong Junejeong	2003
5291	0	0	0	2084	Andrejjovanovic	2003
5292	0	0	0	2089	Michaelagwi	2003
5293	0	0	0	2089	Ioannisvon meyenburg	2004
5294	0	0	0	2091	Felixalopaeus	2007
5295	0	0	0	2091	Akashkarekatte	2004
5296	0	0	0	2091	Skythavornchaisophon	2005

5297	0	0	0	2091	Dhilvanherath	2003
5298	0	0	0	2091	Rahulsameer	2004
5299	0	0	0	2091	Chibinyang	2003
5300	0	0	0	2091	Giorgigiorgadze	2003
5301	0	0	0	2091	Sebastiancarpio	2003
5302	0	0	0	2091	Fernandolimon rumlova	2005
5303	0	0	0	2091	Jun Yeolbae	2003
5304	0	0	0	2091	Peteranastasakis	2004
5305	0	0	0	2091	Chibinyang	2003
5306	0	0	0	2091	Felixalopaeus	2007
5307	0	0	0	2091	Rahulsameer	2004
5308	0	0	0	2091	Dhilvanherath	2003
5309	0	0	0	2091	Abdullahunknown	2003
5310	0	0	0	2091	Jun Yeolbae	2003
5311	0	0	0	2091	Pranjaltiwary	2002
5312	0	0	0	2091	Briancapalnean	2004
5313	0	0	0	2091	Fernandolimon rumlova	2005
5314	0	0	0	2091	Oscarrozados	2003
5315	0	0	0	2091	ELISHA FOLARINolowogbile	2003
5316	0	0	0	2113	Runchengli	2003
5317	0	0	0	2113	Tianhuizhang	2006
5318	0	0	0	2113	Mertramadan	2002
5319	0	0	0	2113	Uladzislautarasevich	2004
5320	0	0	0	2117	Jeansibel	2002
5321	0	0	0	2117	Ondrejvalek	2003
5322	0	0	0	2117	Zoranludoski	2005
5323	0	0	0	2117	Svenmoser	2002
5324	0	0	0	2121	Donovanspigner	2003
5325	0	0	0	2121	Giannilopez	2004
5326	0	0	0	2121	Santiago Adrianvedia villegas	2004
5327	0	0	0	2121	Oskar Erikhakonen	2002
5328	0	0	0	2121	Eliseoalcalde	2003
5329	0	0	0	2121	Alexandergilisen	2002
5330	0	0	0	2121	Axelbergdahl	2002
5331	0	0	0	2121	Adamibrahim	2003
5332	0	0	0	2121	Valentinvasquez	2004
5333	0	0	0	2121	Orhan Gurumur	2003
5334	0	0	0	2121	Filippomartini	2003
5335	0	0	0	2121	Timurchsherbakov	2003
5336	0	0	0	2121	Gyun Seopsim	2002
5337	0	0	0	2121	Gian Carlonavarro	2004
5338	0	0	0	2121	Andrewimmink	2003
5339	0	0	0	2121	Sotaminami	2003
5340	0	0	0	2121	Nikolajovanovic	2004
5341	0	0	0	2121	Eduardonunez del prado flores	2004
5342	0	0	0	2121	Viktormarkov	2005
5343	0	0	0	2121	Karolszczap	2003
5344	0	0	0	2141	Eunjaekim	2003
5345	0	0	0	2141	Izanorgiles garcia retamero	2004
5346	0	0	0	2141	Pabloharo maya	2004

5347	0	0	0	2141	Vuk Davidstramput	2003
5348	0	0	0	2141	Vincentturina	2002
5349	0	0	0	2146	Danielmilardovic	2004
5350	0	0	0	2146	Javier Ernestoaranda alvarez	2003
5351	0	0	0	2146	Joaquinzenteno	2002
5352	0	0	0	2146	Jose Franciscogonzalez grunspa	2002
5353	0	0	0	2146	Lorenzomacioce	2003
5354	0	0	0	2146	Maikelvillalona	2004
5355	0	0	0	2146	Miguel Angelrios	2003
5356	0	0	0	2146	Manuel Ramonespinal plaza	2004
5357	0	0	0	2146	Danielprickett	2004
5358	0	0	0	2146	Rafaelde la vega olascoaga	2005
5359	0	0	0	2146	Omarverduzco	2005
5360	0	0	0	2157	Markomilosavljevic	2004
5361	0	0	0	2157	Zacharyviiala	2005
5362	0	0	0	2157	Tilenkovac	2002
5363	0	0	0	2157	Adarsh Nagatippabhatla	2002
5364	0	0	0	2157	Tillbrunner	2004
5365	0	0	0	2157	Christiangonzalez	2004
5366	0	0	0	2157	Nikwastcoat	2003
5367	0	0	0	2157	Andrincasanova	2004
5368	0	0	0	2157	Jobjapelj	2004
5369	0	0	0	2157	Jbpickard	2003
5370	0	0	0	2157	Artemstarikov	2002
5371	0	0	0	2157	Yaojiezeng	2002
5372	0	0	0	2157	Simonmyslivec	2004
5373	0	0	0	2157	Lucasandrade da silva	2005
5374	0	0	0	2157	Giovannimanfrini	2005
5375	0	0	0	2157	Lukasramer olofsson	2003
5376	0	0	0	2157	Stjepanivanek	2004
5377	0	0	0	2157	Kamran Curtismcintosh-ross	2004
5378	0	0	0	2157	Jean-Nicolasleblanc	2004
5379	0	0	0	2157	Antoninorattenni	2004
5380	0	0	0	2157	Vikasdeo	2002
5381	0	0	0	2157	Matejajanicijevic	2003
5382	0	0	0	2157	Paufanlo munuera	2002
5383	0	0	0	2157	Mihai Viorellungu	2004
5384	0	0	0	2157	Lukegeduldt	2002
5385	0	0	0	2157	Winsteveker	2003
5386	0	0	0	2157	Jamesalbarracin	2004
5387	0	0	0	2157	Georgiseverov	2002
5388	0	0	0	2157	Matthewpitts	2002
5389	0	0	0	2157	Viktorhockl	2003
5390	0	0	0	2157	Simoneguercio	2002
5391	0	0	0	2157	Ruiwakisaka	2003
5392	0	0	0	2157	Haroldhuens	2002
5393	0	0	0	2157	Lucapreda	2006
5394	0	0	0	2157	Kurtmiller	2004
5395	0	0	0	2157	Liamkrall	2002
5396	0	0	0	2157	Seandaryabeigi	2004

5397	0	0	0	2157	Nejczunic	2003
5398	0	0	0	2157	Federicocina	2007
5399	0	0	0	2157	Esahhayat	2002
5400	0	0	0	2157	Samiozzor	2003
5401	0	0	0	2157	Jakubfilip	2005
5402	0	0	0	2157	Radu Danielflorea	2004
5403	0	0	0	2157	Changxiangwang	2004
5404	0	0	0	2157	Viktorhockl	2003
5405	0	0	0	2202	Masatoperera	2003
5406	0	0	0	2202	Juliendando	2002
5407	0	0	0	2202	Filibertofumagalli	2002
5408	0	0	0	2202	Learnertien	2005
5409	0	0	0	2202	andresdel bosque fanti	2003
5410	0	0	0	2202	Samuelnyamekye	2003
5411	0	0	0	2202	Juan Andresferreira ortiz	2004
5412	0	0	0	2202	Sakitange	2004
5413	0	0	0	2202	Nantprachuabmoh	2004
5414	0	0	0	2202	Owissa Makutaannas	2002
5415	0	0	0	2202	GERARDoltra guiral	2002
5416	0	0	0	2202	Andresde los rios kolejewska	2004
5417	0	0	0	2202	Leonardobastos	2005
5418	0	0	0	2202	Spencerjohnson	2003
5419	0	0	0	2202	Guillermorojas	2002
5420	0	0	0	2202	Tamasurban	2004
5421	0	0	0	2202	Ilyakarpov	2002
5422	0	0	0	2202	Santiagohirsch	2002
5423	0	0	0	2202	Fabianrodriguez	2002
5424	0	0	0	2202	Mihailivanov	2004
5425	0	0	0	2202	Stefanpetrovic	2002
5426	0	0	0	2202	Salvadorprice	2005
5427	0	0	0	2202	Diegoserra	2002
5428	0	0	0	2202	Hudsonrivera	2004
5429	0	0	0	2202	Arun Venkatguruswamy	2002
5430	0	0	0	2202	Diegocueto de romana	2005
5431	0	0	0	2228	Dariuseftekhar	2002
5432	0	0	0	2228	Clementmayoral	2003
5433	0	0	0	2228	Jamesjohnson	2002
5434	0	0	0	2228	Johngabelic	2003
5435	0	0	0	2228	Aoiooka	2006
5436	0	0	0	2228	Alexandru Cristiandumitru	2004
5437	0	0	0	2228	Eduardosanchez-carrion	2002
5438	0	0	0	2228	Hugosoares	2005
5439	0	0	0	2228	Hectortalens serra	2002
5440	0	0	0	2237	Zacherylim	2002
5441	0	0	0	2237	Johndransfeld	2002
5442	0	0	0	2237	Elliotjarosz	2003
5443	0	0	0	2237	Danielyizhaki	2003
5444	0	0	0	2237	Cartermorgan	2002
5445	0	0	0	2237	Mikealcarpenter	2002
5446	0	0	0	2237	Shouryaverma	2002

5447	0	0	0	2244	Facundoperlov	2004
5448	0	0	0	2244	Joaquinguilleme	2006
5449	0	0	0	2244	Jonespratama	2003
5450	0	0	0	2244	Emirtoglukdemir	2006
5451	0	0	0	2244	Pedrorapalo ceva	2003
5452	0	0	0	2244	Takiadachi	2003
5453	0	0	0	2244	Jaywong	2002
5454	0	0	0	2244	Diegoguzman	2002
5455	0	0	0	2244	Tomascostanzo	2002
5456	0	0	0	2244	Mohamad Yamannaghnagh	2004
5457	0	0	0	2244	Seok Woojung	2002
5458	0	0	0	2244	Martinmuedini	2002
5459	0	0	0	2244	Kylegriffith	2003
5460	0	0	0	2244	Carlosrullan zarraga	2002
5461	0	0	0	2244	Abdusamadusmanov	2002
5462	0	0	0	2244	Adriandaniel	2003
5463	0	0	0	2244	Oabonakoobatlile	2004
5464	0	0	0	2244	Francoisvan wyk	2004
5465	0	0	0	2244	ATICHAPHATcharatviroch	2006
5466	0	0	0	2244	Marcojalalian	2003
5467	0	0	0	2264	Brandonsuryana	2002
5468	0	0	0	2264	Daniilpiatrouski	2003
5469	0	0	0	2264	Maximkozlov	2002
5470	0	0	0	2264	Antoinevicaire	2002
5471	0	0	0	2268	Lin Entan	2004
5472	0	0	0	2268	Fryderyklechno-wasiutynski	2005
5473	0	0	0	2268	Richard Nii Okaiokine	2004
5474	0	0	0	2268	Lorenzobielli	2003
5475	0	0	0	2268	Jakeriordan	2004
5476	0	0	0	2268	Muhamedademovic	2003
5477	0	0	0	2268	Stepanzhoromsky	2005
5478	0	0	0	2268	Sohmanakagomi	2002
5479	0	0	0	2268	Mateimre	2004
5480	0	0	0	2277	Giuseppebonaiuti	2003
5481	0	0	0	2277	Yutanaka	2003
5482	0	0	0	2277	Mihail Mario Alexandrupop	2004
5483	0	0	0	2277	Dan Vasileduta	2004
5484	0	0	0	2277	Paulwerren	2004
5485	0	0	0	2282	SETHwong	2006
5486	0	0	0	2282	Sergio Alejandrogonzalez ibane	2003
5487	0	0	0	2282	Poonthongkomopisut	2005
5488	0	0	0	2282	Med Forkanelebdi	2003
5489	0	0	0	2282	Jamesmcpherson	2003
5490	0	0	0	2282	Daniilsarksian	2006
5491	0	0	0	2282	Dusantrunic	2002
5492	0	0	0	2282	Samuelmotazed	2003
5493	0	0	0	2282	Nestorlopushynskyi	2002
5494	0	0	0	2282	Gakutatanaka	2004
5495	0	0	0	2282	Louisvanhuyse	2005
5496	0	0	0	2282	Raahimagha	2004

5497	0	0	0	2282	Lorenzosciahbasi	2005
5498	0	0	0	2282	Filipcebic	2003
5499	0	0	0	2282	RUBItoba	2002
5500	0	0	0	2282	Aryaheidari	2002
5501	0	0	0	2282	Alejandroesquivel	2003
5502	0	0	0	2282	MIN-HUNGkao	2002
5503	0	0	0	2282	Dhanushpatel	2004
5504	0	0	0	2282	Louisvanhuyse	2005
5505	0	0	0	2282	Med Forkanelebdi	2003
5506	0	0	0	2282	Aryaheidari	2002
5507	0	0	0	2282	Meherbouars	2004
5508	0	0	0	2305	Anisramchane	2002
5509	0	0	0	2306	OJOfredrick	2002
5510	0	0	0	2306	Emmanuelcottle	2004
5511	0	0	0	2306	Tomasnader	2002
5512	0	0	0	2306	Daksh Ashokagarwal	2004
5513	0	0	0	2306	Muhammad Huzaifakhan	2006
5514	0	0	0	2306	Kobediaz	2004
5515	0	0	0	2306	Pannawatsuttisomboon	2006
5516	0	0	0	2306	ULUGBEKabdurakhmonov	2003
5517	0	0	0	2306	Manfredigraziani	2004
5518	0	0	0	2306	Andreyshutov	2003
5519	0	0	0	2306	Lucas Alexanderlai fat fur	2004
5520	0	0	0	2306	Thomasbevan	2005
5521	0	0	0	2306	Francky Martialhoimian	2006
5522	0	0	0	2306	Alialshati	2002
5523	0	0	0	2306	Malobarthez	2003
5524	0	0	0	2306	Joshmanuel	2005
5525	0	0	0	2306	Karelblom	2003
5526	0	0	0	2306	Owenlien	2007
5527	0	0	0	2306	Dmitrytrunov	2002
5528	0	0	0	2306	Nyovan dyck	2005
5529	0	0	0	2306	Loicschmitt	2004
5530	0	0	0	2306	Louisjulien	2002
5531	0	0	0	2306	Colincaldwell	2003
5532	0	0	0	2306	Nunopinheiro	2004
5533	0	0	0	2306	Fadibidan	2006
5534	0	0	0	2306	Nikitabortnichek	2004
5535	0	0	0	2306	Diegoquispe-kim	2005
5536	0	0	0	2306	Artemchernykh	2005
5537	0	0	0	2306	Davidtotikashvili	2004
5538	0	0	0	2306	Clovisbenichou	2003
5539	0	0	0	2306	Chathuryanilaweera	2004
5540	0	0	0	2306	Angelherreros roldan	2003
5541	0	0	0	2338	Silviomencaglia	2004
5542	0	0	0	2338	Stijnpaardekooper	2003
5543	0	0	0	2338	Rolandrisko	2003
5544	0	0	0	2338	Pavelgula	2004
5545	0	0	0	2338	Breno Rossettourvaneja	2004
5546	0	0	0	2343	Yahiael-bagoury	2003

5547	0	0	0	2343	Benjaminzuniga garcia	2002
5548	0	0	0	2343	Dwaineduncan	2003
5549	0	0	0	2343	Azadrahman	2002
5550	0	0	0	2343	Mohamed Mostafamoens	2004
5551	0	0	0	2343	Luis Gustavomoreira	2004
5552	0	0	0	2343	Joaogoncalves	2003
5553	0	0	0	2343	Hectordubuc	2002
5554	0	0	0	2343	Mohamed Hedibraham	2004
5555	0	0	0	2343	Anthony Brysoncook	2004
5556	0	0	0	2343	Tomgonindard	2003
5557	0	0	0	2343	Rafaelymer	2005
5558	0	0	0	2343	Leonardo Julianquintanilla dava	2002
5559	0	0	0	2356	Haydenloffler	2004
5560	0	0	0	2356	Ginoromano	2002
5561	0	0	0	2356	Gassergasser alaa seif el din	2002
5562	0	0	0	2356	Mohamed Hamzaessid	2002
5563	0	0	0	2356	Danilazakharov	2002
5564	0	0	0	2356	Alessandrod'anna	2002
5565	0	0	0	2356	Loueybenyoussef	2004
5566	0	0	0	2356	Karimgazizov	2004
5567	0	0	0	2356	Batuataseven	2004
5568	0	0	0	2356	Elbertbarr	2002
5569	0	0	0	2356	Janjadlovsky	2003
5570	0	0	0	2367	Ioan Alexandruchirita	2002
5571	0	0	0	2367	UCHEemeruwa	2002
5572	0	0	0	2367	Se Hyunkim	2006
5573	0	0	0	2367	Diegobravetti	2003
5574	0	0	0	2367	Karolmalirz	2002
5575	0	0	0	2367	Fresksylhasi	2004
5576	0	0	0	2367	Daniyaldarken	2003
5577	0	0	0	2367	Waritwannawitchate	2003
5578	0	0	0	2367	Alexandrachour	2003
5579	0	0	0	2367	Juan Cruzalcon	2003
5580	0	0	0	2367	Arturolangle	2002
5581	0	0	0	2367	Youssefkadiri hassani	2003
5582	0	0	0	2367	Alimetwalli	2004
5583	0	0	0	2367	Apivichrojpiboonphan	2005
5584	0	0	0	2367	Daniel Estebansanchez rojas	2003
5585	0	0	0	2367	Aaronventura	2003
5586	0	0	0	2367	Angel Fabrizio Antoniorengel ca	2003
5587	0	0	0	2367	Wenxugu	2004
5588	0	0	0	2367	Benedekszombathelyi	2003
5589	0	0	0	2367	Maxalopaeus	2005
5590	0	0	0	2367	Matthewcizmarik	2006
5591	0	0	0	2367	Damianmuller	2003
5592	0	0	0	2367	Camilocano gomez	2004
5593	0	0	0	2367	Max Shun Wangkwok	2005
5594	0	0	0	2367	Aljontalatayod	2002
5595	0	0	0	2367	Iqbalmohd khan	2002
5596	0	0	0	2367	Kuan Changhuang	2003

5597	0	0	0	2367	Liamdrover-mattinen	2005
5598	0	0	0	2367	Berdiermavlonov	2003
5599	0	0	0	2367	Fahadaldousari	2002
5600	0	0	0	2367	Luisbaumeister	2004
5601	0	0	0	2367	Joshuacheng	2005
5602	0	0	0	2367	Adampanaras	2003
5603	0	0	0	2367	Zamanimoyo	2003
5604	0	0	0	2367	Shanetapera	2004
5605	0	0	0	2367	Youssefkadiri hassani	2003
5606	0	0	0	2367	Waritwannawitchate	2003
5607	0	0	0	2367	Alexandrachour	2003
5608	0	0	0	2367	Joshuacheng	2005
5609	0	0	0	2367	Arturolangle	2002
5610	0	0	0	2367	Max Shun Wangkwok	2005
5611	0	0	0	2367	Alimetwalli	2004
5612	0	0	0	2367	Aaronventura	2003
5613	0	0	0	2367	Fahadaldousari	2002
5614	0	0	0	2367	Kuan Changhuang	2003
5615	0	0	0	2367	Luisbaumeister	2004
5616	0	0	0	2367	Joantorres espinosa	2004
5617	0	0	0	2414	Alexanderdespoja	2005
5618	0	0	0	2414	Davidmanu	2005
5619	0	0	0	2414	Gabriellutsky	2002
5620	0	0	0	2414	Donigashi	2004
5621	0	0	0	2414	Alexhan	2003
5622	0	0	0	2414	Erickats	2003
5623	0	0	0	2414	Dallas Glennramsay	2002
5624	0	0	0	2414	Sebastian Rauljunghanns dietze	2002
5625	0	0	0	2414	Benjaminlortie	2002
5626	0	0	0	2414	Danielzimmermann	2005
5627	0	0	0	2414	Daniilkakhniuk	2002
5628	0	0	0	2425	Simonkladiva	2002
5629	0	0	0	2425	Joao Pedro de Favariengel	2002
5630	0	0	0	2425	Michailchatzigeorgiou	2002
5631	0	0	0	2425	Sebastiangorzny	2004
5632	0	0	0	2425	Samyarelyasi	2004
5633	0	0	0	2425	Zhipengsong	2002
5634	0	0	0	2425	Tudorenea	2003
5635	0	0	0	2425	Sixtocabello	2003
5636	0	0	0	2425	Mikhailrimskii	2004
5637	0	0	0	2425	Davidkennerley	2002
5638	0	0	0	2425	Tatsuyatsuchiyama	2002
5639	0	0	0	2425	Yukiyoshida	2002
5640	0	0	0	2437	Mikolajlis	2003
5641	0	0	0	2437	Kittinaeb iem	2004
5642	0	0	0	2437	Samuelwinter	2003
5643	0	0	0	2437	Blazejsopolinski	2003
5644	0	0	0	2437	Penghanzhang	2004
5645	0	0	0	2437	Sangheonlee	2003
5646	0	0	0	2437	Diegoramos	2003

5647	0	0	0	2444	Fedorpopovic	2002
5648	0	0	0	2444	Sasharozin	2005
5649	0	0	0	2444	Rohanmurali	2004
5650	0	0	0	2444	Kirtanpatel	2004
5651	0	0	0	2444	Min Kijo	2003
5652	0	0	0	2444	Milanristov	2005
5653	0	0	0	2444	Priyanshuchoudhary	2002
5654	0	0	0	2444	Taewanjoo	2005
5655	0	0	0	2444	Ziadhemida	2003
5656	0	0	0	2444	Beomchanoh	2004
5657	0	0	0	2444	Marknawa	2006
5658	0	0	0	2444	Guidofeldberg	2002
5659	0	0	0	2444	Anesukwirirai	2002
5660	0	0	0	2444	Edgardo Manuelangara	2004
5661	0	0	0	2444	Santiagoserdan	2004
5662	0	0	0	2444	Diegolaguna	2004
5663	0	0	0	2444	Agriyayadav	2005
5664	0	0	0	2444	Ryoheimiyake	2004
5665	0	0	0	2444	Ioannisbechlivanidis	2004
5666	0	0	0	2444	Simonknezevic	2005
5667	0	0	0	2444	Stylianospapamichael	2005
5668	0	0	0	2444	Sebastiencauhape	2006
5669	0	0	0	2444	SANTIAGO MARTINenriquez bo	2006
5670	0	0	0	2444	Koloskincses	2007
5671	0	0	0	2444	Bertinkarenzi	2002
5672	0	0	0	2444	Nikhilmukherjee	2005
5673	0	0	0	2444	Matteobonfiglio	2003
5674	0	0	0	2444	Albert Porsmyrhansen	2003
5675	0	0	0	2444	Danielcordero	2004
5676	0	0	0	2444	Hrishikeshparashar	2003
5677	0	0	0	2444	Aamishpal	2002
5678	0	0	0	2444	Valtterivare	2003
5679	0	0	0	2444	Santiagoflores salazar	2003
5680	0	0	0	2444	Ebolum Pastornwokolo	2003
5681	0	0	0	2444	Marcellmarki	2005
5682	0	0	0	2444	Martindespodov	2004
5683	0	0	0	2444	Simone Marcocavalleri	2003
5684	0	0	0	2444	Vadymrogynskyi	2004
5685	0	0	0	2444	Kazumasamatsuda	2005
5686	0	0	0	2444	Pascaltylek	2004
5687	0	0	0	2444	Lorenzogallo	2003
5688	0	0	0	2444	Dayronzuniga	2002
5689	0	0	0	2444	Alexlindqvist	2003
5690	0	0	0	2444	Matthewrobert	2002
5691	0	0	0	2444	Bukhosi Jordanmoyo	2004
5692	0	0	0	2444	Ognjenpavlovic	2002
5693	0	0	0	2444	Rodrigosanchez hurtado	2003
5694	0	0	0	2444	Juan pablolua llamas	2004
5695	0	0	0	2444	Oscaroberoi jensen	2004
5696	0	0	0	2444	Alvaropedraza garcia	2004

5697	0	0	0	2444	Mohamed Saidounis	2003
5698	0	0	0	2444	Tunatuygun	2004
5699	0	0	0	2444	Oscarhernandez gonzalez	2002
5700	0	0	0	2444	Sergejdrljaca	2003
5701	0	0	0	2444	Veljkocvetkovic	2004
5702	0	0	0	2444	Hugo Joaquimbabakian	2003
5703	0	0	0	2444	Josemurariu	2005
5704	0	0	0	2444	Oscarhernandez gonzalez	2002
5705	0	0	0	2444	Danieltriantafyllou	2002
5706	0	0	0	2444	Alvaropedraza garcia	2004
5707	0	0	0	2444	Oscaroberoi jensen	2004
5708	0	0	0	2444	Marcellmarki	2005
5709	0	0	0	2444	Ioannisbechlivanidis	2004
5710	0	0	0	2444	Henryriccioni	2003
5711	0	0	0	2444	Mohamed Saidounis	2003
5712	0	0	0	2444	Filippocarpi	2003
5713	0	0	0	2444	SANTIAGO MARTINenriquez bo	2006
5714	0	0	0	2444	FEDERICO HERNANgiovanniello	2003
5715	0	0	0	2444	Mohammadkatibeh	2002
5716	0	0	0	2444	Kazumasamatsuda	2005
5717	0	0	0	2444	Noahbecker	2003
5718	0	0	0	2444	Eliezergonzalez	2004
5719	0	0	0	2444	Pascaltylek	2004
5720	0	0	0	2444	Archieturner	2002
5721	0	0	0	2444	Aamishpal	2002
5722	0	0	0	2444	Ebolum Pastornwokolo	2003
5723	0	0	0	2444	Rohanmurali	2004
5724	0	0	0	2444	Alexlindqvist	2003
5725	0	0	0	2444	Santiagoflores salazar	2003
5726	0	0	0	2444	Curtseifert	2002
5727	0	0	0	2444	Rawiyosef	2003
5728	0	0	0	2444	Gakuokuma	2005
5729	0	0	0	2444	Danielcordero	2004
5730	0	0	0	2444	Priyanshuchoudhary	2002
5731	0	0	0	2444	Taiyokurata	2003
5732	0	0	0	2444	Cyrusmahjoob	2005
5733	0	0	0	2444	Lorenzogallo	2003
5734	0	0	0	2444	Luke Connorheron	2003
5735	0	0	0	2444	Wyattobrien	2005
5736	0	0	0	2444	Pawatpunnaket	2004
5737	0	0	0	2444	Simone Marcocavalleri	2003
5738	0	0	0	2444	Tunatuygun	2004
5739	0	0	0	2444	Antoinerambour	2004
5740	0	0	0	2444	Enmanuelmunoz	2005
5741	0	0	0	2444	Bukhosi Jordanmoyo	2004
5742	0	0	0	2444	Suleyman Denizgursoy	2004
5743	0	0	0	2444	Jingsongwang	2003
5744	0	0	0	2444	Bertinkarenzi	2002
5745	0	0	0	2444	Ramiro Nicolasrincon hernande	2002
5746	0	0	0	2444	Slobodancejovic	2002

5747	0	0	0	2444	Masakikanehara	2002
5748	0	0	0	2444	Ognjenpavlovic	2002
5749	0	0	0	2444	Mateomatulovich	2003
5750	0	0	0	2444	Kai En Matthewlim	2004
5751	0	0	0	2444	Davidjohn	2002
5752	0	0	0	2444	Karimchedly	2004
5753	0	0	0	2444	Josemoreno	2002
5754	0	0	0	2444	Nadirsargsyan	2003
5755	0	0	0	2444	Suleimanibrahim	2005
5756	0	0	0	2444	Sergejdrljaca	2003
5757	0	0	0	2444	Alfredode marinis	2002
5758	0	0	0	2444	Beomchanoh	2004
5759	0	0	0	2444	Asafschwartz	2004
5760	0	0	0	2444	Mohamedabdellaoui maane	2004
5761	0	0	0	2444	Alfonsridstrom	2005
5762	0	0	0	2444	Ryoheimiyake	2004
5763	0	0	0	2444	Antonioayerbe streubel	2004
5764	0	0	0	2444	Thomasdrayton	2003
5765	0	0	0	2444	Chatthitiboonprakong	2004
5766	0	0	0	2563	Markostoilkovski	2005
5767	0	0	0	2563	Bendugmore	2002
5768	0	0	0	2563	Aleksamisic	2004
5769	0	0	0	2563	Danylopustovoitenko	2002
5770	0	0	0	2563	Dorianvasung	2004
5771	0	0	0	2563	Walkerallen	2005
5772	0	0	0	2563	Leonidaslazarides	2005
5773	0	0	0	2563	Alfredhagejard	2002
5774	0	0	0	2563	Maxxwilliam	2003
5775	0	0	0	2563	Gregor Mariuskliimask	2002
5776	0	0	0	2563	Akmalimamov	2002
5777	0	0	0	2563	Tristanreiff	2003
5778	0	0	0	2563	Tomslabbert	2002
5779	0	0	0	2563	Maleksharaf	2004
5780	0	0	0	2563	Bentomarelim araujo	2003
5781	0	0	0	2563	Viktoryanev	2002
5782	0	0	0	2563	Jose Ignacioblum granados	2003
5783	0	0	0	2563	Christotimini	2004
5784	0	0	0	2563	Ishmael Nii Norteydowuona	2004
5785	0	0	0	2563	Noahburton	2002
5786	0	0	0	2563	Robin Mickbucher	2002
5787	0	0	0	2563	Hugocoquelin	2005
5788	0	0	0	2585	Viliussauciunas	2002
5789	0	0	0	2585	Benjamin Ignacioherrera delucc	2003
5790	0	0	0	2585	Dmytriiierokhin	2003
5791	0	0	0	2585	Ilbinnam	2003
5792	0	0	0	2585	Takuyakobayashi	2004
5793	0	0	0	2585	Maximehenny	2002
5794	0	0	0	2585	Moritzschroeter	2003
5795	0	0	0	2585	Jin Youngkim	2002
5796	0	0	0	2585	KAI-CHUNlin	2004

5797	0	0	0	2585	Ethanielwaterschoot	2003
5798	0	0	0	2585	Leomatthysen	2006
5799	0	0	0	2585	Julientremolieres	2005
5800	0	0	0	2597	Heotae gyeong	2003
5801	0	0	0	2597	Manuellazic	2005
5802	0	0	0	2597	Raja Azanahmed sajjid	2004
5803	0	0	0	2597	Giovan Leonlumenta	2004
5804	0	0	0	2597	Martin Matiasgaviria arboleda	2003
5805	0	0	0	2597	Tylerwaddock	2004
5806	0	0	0	2597	Bardiaziyaei	2004
5807	0	0	0	2597	Daniilegorov	2003
5808	0	0	0	2597	Bojanradoman	2003
5809	0	0	0	2597	Coopergriffiths	2002
5810	0	0	0	2597	Ibrokhimfayzullaev	2003
5811	0	0	0	2597	Mattponchet	2004
5812	0	0	0	2597	Carlosferrer	2003
5813	0	0	0	2597	Dakotagibson	2004
5814	0	0	0	2597	Jaydenharrison	2002
5815	0	0	0	2597	Kim Johannesniethammer	2003
5816	0	0	0	2597	Dmitriyparintsev	2004
5817	0	0	0	2597	Billchan	2006
5818	0	0	0	2597	Malagushi	2004
5819	0	0	0	2597	Soo Hyunkang	2003
5820	0	0	0	2597	Alejandroraffo	2003
5821	0	0	0	2597	Constantinogarcia ortiz	2002
5822	0	0	0	2597	Sylgaxherri	2004
5823	0	0	0	2597	Valentinwerner	2003
5824	0	0	0	2597	Norbertmarosik	2004
5825	0	0	0	2597	Alirezagolzad	2004
5826	0	0	0	2597	Berentan	2004
5827	0	0	0	2597	Abdulhanan	2005
5828	0	0	0	2597	Samedgar	2002
5829	0	0	0	2597	Daniel Josepinzon pinto	2004
5830	0	0	0	2597	Manuellazic	2005
5831	0	0	0	2597	Younghacho	2002
5832	0	0	0	2597	Ching Kitfong	2003
5833	0	0	0	2597	Jaimesigauque	2002
5834	0	0	0	2597	Henrikvillanger	2004
5835	0	0	0	2597	Robertgaliullin	2006
5836	0	0	0	2597	Kennethprete	2002
5837	0	0	0	2597	Giovan Leonlumenta	2004
5838	0	0	0	2597	Mohammednabihi	2004
5839	0	0	0	2597	Brieucfisette	2002
5840	0	0	0	2597	Amirkhamzanasridinov	2005
5841	0	0	0	2597	Lukaplosnic	2005
5842	0	0	0	2597	Danirkaldybekov	2006
5843	0	0	0	2597	Roshufujioka	2005
5844	0	0	0	2597	Mustafadustov	2004
5845	0	0	0	2597	Nazarposikira	2004
5846	0	0	0	2597	Ainurgarifullin	2003

5847	0	0	0	2597	Tobias Bokudsk	2002
5848	0	0	0	2597	Seanlee	2003
5849	0	0	0	2597	Kasperbyskata	2002
5850	0	0	0	2597	Yaronamorule	2004
5851	0	0	0	2597	Ivocimino	2003
5852	0	0	0	2597	Manuelmesmer	2002
5853	0	0	0	2597	Juan Daviddiaz rincon	2005
5854	0	0	0	2597	Michalwlodarczyk	2004
5855	0	0	0	2597	Christopherlam	2004
5856	0	0	0	2597	Andres Mauriciosaavedra aldan	2004
5857	0	0	0	2597	Aaronmatikkala	2003
5858	0	0	0	2597	Zoranstastny	2003
5859	0	0	0	2597	Bradywisdom	2003
5860	0	0	0	2597	Maxymkulish	2005
5861	0	0	0	2597	Yashnagdev	2002
5862	0	0	0	2597	Pablobesse	2002
5863	0	0	0	2597	Clementbergantz	2006
5864	0	0	0	2597	Marcosnavas gallardo	2002
5865	0	0	0	2597	Ivantrevisiol	2002
5866	0	0	0	2597	Charltondottin	2002
5867	0	0	0	2597	Ahmad Naelqureshi	2006
5868	0	0	0	2597	Raphaelpichot	2004
5869	0	0	0	2597	Ethanschiffman	2004
5870	0	0	0	2597	Baltazarwiger nordas	2003
5871	0	0	0	2597	Artyomastafyev	2003
5872	0	0	0	2597	Stuisaravia obando	2002
5873	0	0	0	2597	Adamlevi	2002
5874	0	0	0	2597	Lukecarey	2003
5875	0	0	0	2597	Jasonfogle	2002
5876	0	0	0	2597	Giacomonosei	2005
5877	0	0	0	2597	Eldonlee	2007
5878	0	0	0	2597	Marioantonio morales	2003
5879	0	0	0	2597	Ilyaterebkov	2006
5880	0	0	0	2597	Tomyquijada rios	2003
5881	0	0	0	2597	Vladislavkebets	2002
5882	0	0	0	2597	Arturibragimov	2005
5883	0	0	0	2597	Nickolaymarakhtanov	2002
5884	0	0	0	2597	Julesjolivier	2003
5885	0	0	0	2597	Williambartram	2004
5886	0	0	0	2597	Ole Tiniusstave lepsoe	2002
5887	0	0	0	2597	Ahmedbousslema	2003
5888	0	0	0	2597	Antonio Josechavez chavez	2005
5889	0	0	0	2597	Kornelhalmos	2006
5890	0	0	0	2597	Matiasrojas	2004
5891	0	0	0	2597	Adamibnelkaid	2004
5892	0	0	0	2597	Marinjamnicki	2002
5893	0	0	0	2597	Sergeivukasinovic	2005
5894	0	0	0	2597	Andrei Cristianbutnaru	2002
5895	0	0	0	2597	Andres Ignaciovaldez arze	2002
5896	0	0	0	2597	Lawrenceivanow	2005

5897	0	0	0	2597	Tymofiykhrystyuk	2004
5898	0	0	0	2597	Wianroothman	2004
5899	0	0	0	2597	Alessandrococcioli	2002
5900	0	0	0	2597	Zakharmartsinkevich	2003
5901	0	0	0	2597	Bekzodnematov	2005
5902	0	0	0	2597	JeanPaulsantamaria	2003
5903	0	0	0	2597	Joshuaoboniye	2004
5904	0	0	0	2597	Mohamed Nasserabouzied	2007
5905	0	0	0	2597	Matiasrojas	2004
5906	0	0	0	2597	Tymofiykhrystyuk	2004
5907	0	0	0	2597	Daniilegorov	2003
5908	0	0	0	2597	Cesarmahecha	2006
5909	0	0	0	2597	Danirkaldybekov	2006
5910	0	0	0	2597	Coopergriffiths	2002
5911	0	0	0	2597	Ole Tiniusstave lepsoe	2002
5912	0	0	0	2597	Inigodiaz	2003
5913	0	0	0	2597	Ilyaterebkov	2006
5914	0	0	0	2597	Billchan	2006
5915	0	0	0	2597	Younghacho	2002
5916	0	0	0	2597	Suvratmall	2002
5917	0	0	0	2597	Soo Hyunkang	2003
5918	0	0	0	2597	Miguelsimao	2003
5919	0	0	0	2597	Andrei Cristianbutnaru	2002
5920	0	0	0	2597	Eduardo Adriancama san marti	2003
5921	0	0	0	2597	Artyomastafyev	2003
5922	0	0	0	2597	Aryamanrajeev	2003
5923	0	0	0	2597	Valentinwerner	2003
5924	0	0	0	2597	Masihmohkamkar	2004
5925	0	0	0	2597	Mattponchet	2004
5926	0	0	0	2597	Haykmikayelyan	2004
5927	0	0	0	2597	Henrikvillanger	2004
5928	0	0	0	2597	Hidekimatsuoka	2003
5929	0	0	0	2597	Raja Azanahmed sajjid	2004
5930	0	0	0	2597	Rudyquan	2006
5931	0	0	0	2597	Kim Johannesniethammer	2003
5932	0	0	0	2597	Einarikilpinen	2004
5933	0	0	0	2597	Maxymkulish	2005
5934	0	0	0	2597	Tomokihanahara	2005
5935	0	0	0	2597	Sergeivukasinovic	2005
5936	0	0	0	2597	Yosvanimaturell	2003
5937	0	0	0	2597	Wongwarunwongcharoen	2006
5938	0	0	0	2597	Omidasghari	2002
5939	0	0	0	2597	Dmitriyparintsev	2004
5940	0	0	0	2597	Yosmanimaturell	2003
5941	0	0	0	2597	Raphaelpichot	2004
5942	0	0	0	2597	Aleksababic	2004
5943	0	0	0	2597	Bekzodnematov	2005
5944	0	0	0	2597	Danielgruen	2002
5945	0	0	0	2597	Zhuyuanli	2004
5946	0	0	0	2597	Joshuakhoo	2005

5947	0	0	0	2597	Norbertmarosik	2004
5948	0	0	0	2597	Daikimorikawa	2005
5949	0	0	0	2597	Mustafadustov	2004
5950	0	0	0	2597	Juan Arturoespanol moreno	2003
5951	0	0	0	2597	Brieucfisette	2002
5952	0	0	0	2597	Stuisaravia obando	2002
5953	0	0	0	2597	Giovannerecchia	2006
5954	0	0	0	2597	Andres Ignaciovaldez arze	2002
5955	0	0	0	2597	Lukaplosnic	2005
5956	0	0	0	2597	Tomyquijada rios	2003
5957	0	0	0	2597	Christopherlam	2004
5958	0	0	0	2597	Joshuaoboniye	2004
5959	0	0	0	2597	Tobias Bokudsk	2002
5960	0	0	0	2597	Antonio Josechavez chavez	2005
5961	0	0	0	2597	Gabrielelicha navas	2003
5962	0	0	0	2597	Jorgecatala ortiz	2003
5963	0	0	0	2597	Jaimesigauque	2002
5964	0	0	0	2597	Omaansh Choudharysaharia	2006
5965	0	0	0	2597	Bardiaziyaei	2004
5966	0	0	0	2597	Alexlepine	2003
5967	0	0	0	2597	Amirkhamzanasridinov	2005
5968	0	0	0	2597	Alfonso Alejandrozerpa contrer	2002
5969	0	0	0	2597	Dhruvverma	2003
5970	0	0	0	2597	Doriantremblay	2002
5971	0	0	0	2597	Alirezagolzad	2004
5972	0	0	0	2597	Angilbalaoing	2004
5973	0	0	0	2597	Sylgaxherri	2004
5974	0	0	0	2597	Oskaripaldanius	2007
5975	0	0	0	2597	Berentan	2004
5976	0	0	0	2597	Bright Nii Norteinortey	2004
5977	0	0	0	2597	Juan Luisreding	2006
5978	0	0	0	2597	Guillermo Davidrodriguez pinill	2005
5979	0	0	0	2597	Arturibragimov	2005
5980	0	0	0	2597	Leliu	2004
5981	0	0	0	2597	Manuelmesmer	2002
5982	0	0	0	2597	raflyzulkarnaaen	2004
5983	0	0	0	2597	BUSHANhaobam	2006
5984	0	0	0	2597	Vladislavkebets	2002
5985	0	0	0	2597	Josueguzman	2005
5986	0	0	0	2597	Francisco Tomasbertolino	2005
5987	0	0	0	2597	Michaelemmanuel	2004
5988	0	0	0	2597	Hoi Man Kevinwong	2002
5989	0	0	0	2597	Jaydenharrison	2002
5990	0	0	0	2597	Josephmagutshwa	2005
5991	0	0	0	2597	Alessandrococcioli	2002
5992	0	0	0	2597	Zakharmartsinkevich	2003
5993	0	0	0	2597	Cesarcruz	2006
5994	0	0	0	2597	Parichsavetvanich	2006
5995	0	0	0	2597	Drikusde jager	2005
5996	0	0	0	2597	Marinjamnicki	2002

5997	0	0	0	2597	Peter Ochejohn	2005
5998	0	0	0	2597	Adamlevi	2002
5999	0	0	0	2597	Arian Ranggadesvianto	2003
6000	0	0	0	2597	Andrekock	2003
6001	0	0	0	2597	Ante Petarercegovic	2003
6002	0	0	0	2597	Giacomonosei	2005
6003	0	0	0	2597	Adamduda	2004
6004	0	0	0	2597	Adamduda	2004
6005	0	0	0	2597	Alahaouas	2004
6006	0	0	0	2597	Trevormasi	2002
6007	0	0	0	2597	Ivancenturion	2003
6008	0	0	0	2597	Bojanradoman	2003
6009	0	0	0	2597	Oybekmirzoshamsutdinov	2005
6010	0	0	0	2597	Julianojompolsky	2002
6011	0	0	0	2597	Sergeivukasinovic	2005
6012	0	0	0	2809	Williammclennaghan	2002
6013	0	0	0	2809	Saschajankovic	2002
6014	0	0	0	2809	Davidgauberg	2003
6015	0	0	0	2809	Coopergordon	2004
6016	0	0	0	2809	Jose Andresperez	2004
6017	0	0	0	2809	Alejandrosantana	2002
6018	0	0	0	2809	Apollonpapapanagiotou	2003
6019	0	0	0	2809	Jairdangibbs	2005
6020	0	0	0	2809	Jovanslijepcevic	2002
6021	0	0	0	2809	Francescocastellano	2003
6022	0	0	0	2809	Meesrottgering	2007
6023	0	0	0	2809	Marcopennelli	2003
6024	0	0	0	2809	Weitaoxu	2005
6025	0	0	0	2809	Raffaelemariani	2004
6026	0	0	0	2809	Man Hoyap	2004
6027	0	0	0	2809	Mateuszlange	2006
6028	0	0	0	2809	Thomaskennedy	2003
6029	0	0	0	2809	Davidcerne	2004
6030	0	0	0	2809	NEKTARIOSvlahos	2002
6031	0	0	0	2809	Randyfunez rivera	2003
6032	0	0	0	2809	Markdrozdetskii	2002
6033	0	0	0	2809	Andrejdjokic	2003
6034	0	0	0	2809	Armanzamani	2002
6035	0	0	0	2809	Sebastiangarcia escorcia	2005
6036	0	0	0	2809	Nikitasafonov	2004
6037	0	0	0	2809	Henriklindeman	2002
6038	0	0	0	2809	Carlos Manuelalvarez castillo	2003
6039	0	0	0	2809	Thomasaston	2005
6040	0	0	0	2809	Andres Felipebonilla perez	2002
6041	0	0	0	2809	Gustavhasslegren	2003
6042	0	0	0	2809	Igortikhonov	2002
6043	0	0	0	2809	Miguelmartin cano	2002
6044	0	0	0	2809	Tintran	2003
6045	0	0	0	2809	Adriapol torres	2004
6046	0	0	0	2809	Inigobarcelo	2003

6047	0	0	0	2809	Andrealibre	2003
6048	0	0	0	2809	Eliashoxha	2004
6049	0	0	0	2809	Rainuustalo	2002
6050	0	0	0	2809	Aidancarter	2002
6051	0	0	0	2809	Orlandoguerrero	2003
6052	0	0	0	2809	Nickolasrosa	2002
6053	0	0	0	2809	Duncanmccall	2005
6054	0	0	0	2809	Mehdielloumi	2004
6055	0	0	0	2809	Nimrodnemes	2002
6056	0	0	0	2809	Ioannisstamoulos	2003
6057	0	0	0	2809	Enriconurme	2003
6058	0	0	0	2809	Thibaultdorval	2002
6059	0	0	0	2809	Derekwinsnes	2004
6060	0	0	0	2809	Simonjavorsek	2003
6061	0	0	0	2809	Faressaid	2003
6062	0	0	0	2809	alexanderkotarski	2005
6063	0	0	0	2809	Lukasescher	2005
6064	0	0	0	2809	Shauryasood	2002
6065	0	0	0	2809	Martimmarujo	2003
6066	0	0	0	2809	Ottorosin	2003
6067	0	0	0	2809	Francescomineo mineo	2002
6068	0	0	0	2809	Noahgabriel	2005
6069	0	0	0	2809	Andrestenorio	2004
6070	0	0	0	2809	Samverkaeren	2002
6071	0	0	0	2809	Jon Williamkarlstad	2003
6072	0	0	0	2809	Kristofferstahlbrand	2002
6073	0	0	0	2809	Ivanperez martin	2004
6074	0	0	0	2809	Jaccoarends	2003
6075	0	0	0	2809	Eirinaiosioannou eirinaios	2004
6076	0	0	0	2809	Vinkobrkic	2003
6077	0	0	0	2809	Majmiklavec	2003
6078	0	0	0	2809	Mahdibehboodi	2002
6079	0	0	0	2809	ChengLinli	2003
6080	0	0	0	2809	Mathiaslangaard	2003
6081	0	0	0	2809	Noaforste dinell	2005
6082	0	0	0	2809	Erikzhakiyanov	2004
6083	0	0	0	2809	Cornelius Kee Hungsia	2003
6084	0	0	0	2809	Mattiasselde	2002
6085	0	0	0	2809	Alejandrocaceres	2003
6086	0	0	0	2809	Jakubfederowicz	2002
6087	0	0	0	2809	Petrpanacek	2005
6088	0	0	0	2809	Timurmukhtarulin	2002
6089	0	0	0	2809	Adrijanhrzic	2002
6090	0	0	0	2809	Mahmoudfathallah	2003
6091	0	0	0	2809	Maksymjezierski	2003
6092	0	0	0	2809	Shidoito	2003
6093	0	0	0	2809	Sebastianeriksson	2005
6094	0	0	0	2809	Carlos Danielvillamizar vargas	2002
6095	0	0	0	2892	Aryanshah	2005
6096	0	0	0	2892	Jean-Christophe Lbaril	2003

6097	0	0	0	2892	Nikolaibarsukov	2006
6098	0	0	0	2892	Josehernandez saracho	2002
6099	0	0	0	2892	Hanupatel	2005
6100	0	0	0	2892	Vaclavschneider	2002
6101	0	0	0	2892	Talalalabdullah	2003
6102	0	0	0	2892	Alejandrobravo diaz	2005
6103	0	0	0	2892	Luis Albertorivas	2002
6104	0	0	0	2892	Jesus Guillermorodriguez fonse	2002
6105	0	0	0	2892	Halelashoosh	2006
6106	0	0	0	2892	Jamilracho	2002
6107	0	0	0	2892	Tassoalves moreira	2003
6108	0	0	0	2892	Andres Alejandroandrade fierro	2003
6109	0	0	0	2892	Nicolasbettini	2002
6110	0	0	0	2892	Luccapinto	2003
6111	0	0	0	2892	Geonwoolee	2005
6112	0	0	0	2892	Semenkinner	2004
6113	0	0	0	2892	Benjaminpoblete martinez	2003
6114	0	0	0	2892	Egorkrivenyshev	2003
6115	0	0	0	2892	Alexanderlee	2003
6116	0	0	0	2892	Arnavnemali	2005
6117	0	0	0	2892	Hiirosakamoto	2005
6118	0	0	0	2892	Dunstan Alijahleslie	2003
6119	0	0	0	2892	Eduardogutierrez	2003
6120	0	0	0	2892	Araizmalik	2002
6121	0	0	0	2892	Kunakornkangtong	2005
6122	0	0	0	2892	Nathanvaldez	2003
6123	0	0	0	2892	Edgarmayorga calva	2004
6124	0	0	0	2892	Talalalabdullah	2003
6125	0	0	0	2892	Amitshamash	2005
6126	0	0	0	2892	Krittamatesodsong	2005
6127	0	0	0	2892	Panathonsooksod	2004
6128	0	0	0	2892	Nikolaibarsukov	2006
6129	0	0	0	2892	Wadehermanus	2003
6130	0	0	0	2892	Markojevremovic	2002
6131	0	0	0	2892	Alejandrobravo diaz	2005
6132	0	0	0	2892	Luis Albertorivas	2002
6133	0	0	0	2892	Marcusmasarcin	2004
6134	0	0	0	2892	Ziedtalbi	2002
6135	0	0	0	2892	Quangduong	2006
6136	0	0	0	2892	Alexballinger	2002
6137	0	0	0	2892	Gabrieljornert	2004
6138	0	0	0	2892	Aryanshah	2005
6139	0	0	0	2892	Amitoj Singhsachdeva	2003
6140	0	0	0	2892	Oliverbyskata	2005
6141	0	0	0	2892	Danurey	2003
6142	0	0	0	2892	Kristianjanci	2004
6143	0	0	0	2892	Jordangancho	2003
6144	0	0	0	2892	Michaelbakay	2002
6145	0	0	0	2892	Hanupatel	2005
6146	0	0	0	2892	Mohammad Erfandarvish nouri	2002

6147	0	0	0	2892	Vaclavschneider	2002
6148	0	0	0	2892	Djordjekurcubic	2002
6149	0	0	0	2892	Dimitrijekocic	2002
6150	0	0	0	2892	Artemsalomatov	2003
6151	0	0	0	2892	Jacopobilardo	2004
6152	0	0	0	2892	Barakbar	2004
6153	0	0	0	2892	Hasan Basaransimsek	2004
6154	0	0	0	2892	Josehernandez saracho	2002
6155	0	0	0	2892	Alejandrorodriguez siquier	2003
6156	0	0	0	2892	Raffaellopapajcik	2003
6157	0	0	0	2892	Markvisontai	2002
6158	0	0	0	2892	Amaan Adiltezabwala	2002
6159	0	0	0	2956	Yaroslavsharyy	2004
6160	0	0	0	2956	Mirkolagasio	2003
6161	0	0	0	2956	Stefanopapagno	2003
6162	0	0	0	2956	Arthuro'sullivan	2004
6163	0	0	0	2956	Samyakbajracharya	2003
6164	0	0	0	2956	Jing Zhuchen	2002
6165	0	0	0	2956	Jing Kechen	2002
6166	0	0	0	2956	Mohamed Alisghaier	2005
6167	0	0	0	2956	Allankrueger	2003
6168	0	0	0	2956	Andreivoican	2004
6169	0	0	0	2956	Joaomaia	2004
6170	0	0	0	2956	Niccolofestini mira	2004
6171	0	0	0	2956	Andriidenysenko	2004
6172	0	0	0	2956	Nikitatyatkov	2002
6173	0	0	0	2956	Seisaitoh	2002
6174	0	0	0	2956	Soutayoshida	2004
6175	0	0	0	2956	Aleshrdina	2003
6176	0	0	0	2956	Hodcohen	2002
6177	0	0	0	2956	Rasulnurmukhamedov	2003
6178	0	0	0	2956	Ethanblot	2003
6179	0	0	0	2956	Igordanich	2002
6180	0	0	0	2956	Nikjurjevcic	2002
6181	0	0	0	2956	Lazarignjovic	2002
6182	0	0	0	2956	Csanadnyaradi	2002
6183	0	0	0	2956	Marioradev	2002
6184	0	0	0	2956	Urosjovic	2002
6185	0	0	0	2956	Teomancelik	2003
6186	0	0	0	2956	Lukatodorovic	2002
6187	0	0	0	2956	Alexandersviridochkin	2003
6188	0	0	0	2956	Illyarevenko	2002
6189	0	0	0	2956	Filippomorroni	2003
6190	0	0	0	2956	Ciprian Andreidavid	2002
6191	0	0	0	2956	Mihakastelec	2002
6192	0	0	0	2956	Tanakornsrirat	2002
6193	0	0	0	2956	Neelgarud	2002
6194	0	0	0	2956	Kennethlegacy	2002
6195	0	0	0	2956	Fidanagushi	2002
6196	0	0	0	2956	Robertorombola	2003

6197	0	0	0	2956	Stavroshadjivarnava	2003
6198	0	0	0	2956	Ethanterblanche	2002
6199	0	0	0	2956	Maceosimpson	2003
6200	0	0	0	2956	Harshvardhanchopra	2002
6201	0	0	0	2956	Artjomsafanasjevs	2005
6202	0	0	0	2956	Nicolastrebuchet	2005
6203	0	0	0	2956	Goncalomarques	2006
6204	0	0	0	2956	Eliegadilhe	2004
6205	0	0	0	2956	Tiagoboschmans	2005
6206	0	0	0	2956	Hanyangliu	2003
6207	0	0	0	2956	Gianlucabeaujon	2003
6208	0	0	0	2956	Sidneydollar	2002
6209	0	0	0	2956	Anuthwijesinghe	2004
6210	0	0	0	2956	Davisrolis	2005
6211	0	0	0	2956	Markuslazarcik	2005
6212	0	0	0	2956	Maximcazac	2004
6213	0	0	0	2956	Sergeypetrov	2005
6214	0	0	0	2956	Freddymurray	2003
6215	0	0	0	2956	Aeneasschaub	2002
6216	0	0	0	2956	Vanjavojinovic	2003
6217	0	0	0	2956	Ivanlevitsky	2003
6218	0	0	0	2956	Alexanderasenov	2002
6219	0	0	0	2956	Eugeniogonzalez fitzmaurice	2003
6220	0	0	0	2956	Sementsyrenov	2002
6221	0	0	0	2956	Parakrambakiwala	2002
6222	0	0	0	2956	Romankazantsev	2004
6223	0	0	0	2956	Micheleribecai	2003
6224	0	0	0	2956	Vladislavabramov	2004
6225	0	0	0	2956	Nicholas Eleazarwidyaputra	2002
6226	0	0	0	2956	Oryanzohar	2003
6227	0	0	0	2956	Tarikbajrovic	2004
6228	0	0	0	2956	Alexandruandrian	2003
6229	0	0	0	2956	Peterkucera	2003
6230	0	0	0	2956	Mustaphael natour	2004
6231	0	0	0	2956	Mihailbrovinschi	2004
6232	0	0	0	2956	Andrijastoskovic	2002
6233	0	0	0	2956	Constantinosdjakouris	2004
6234	0	0	0	2956	Mihajlostanojevic	2005
6235	0	0	0	2956	Navidrezarowshanghias	2004
6236	0	0	0	2956	Christianparr	2002
6237	0	0	0	2956	Andersparr	2002
6238	0	0	0	2956	Mikkelballisager	2003
6239	0	0	0	2956	Allandimov	2005
6240	0	0	0	2956	Thanosbenos	2003
6241	0	0	0	2956	Maksymneverov	2002
6242	0	0	0	2956	Oleksandrguriev	2005
6243	0	0	0	2956	Siddharth Reddyvaada	2004
6244	0	0	0	2956	Flynn Tjarkbaumert	2005
6245	0	0	0	2956	Ioanniskassis	2003
6246	0	0	0	2956	Josemarino	2002

6247	0	0	0	2956	Zurycohen	2005
6248	0	0	0	2956	Dhanyashah	2003
6249	0	0	0	2956	Tommasopace	2002
6250	0	0	0	2956	Demidgorbachev	2007
6251	0	0	0	2956	Pabloriva	2002
6252	0	0	0	2956	Hugho'sullivan	2003
6253	0	0	0	2956	Jingyikaipan	2002
6254	0	0	0	2956	Sai Bhairaavkmannju	2003
6255	0	0	0	2956	Royleshem	2004
6256	0	0	0	2956	Niklasgrunewald	2004
6257	0	0	0	2956	LIAMsheppard	2003
6258	0	0	0	2956	Roryhogan	2002
6259	0	0	0	2956	Iraklisgerousis	2002
6260	0	0	0	2956	KRITANTAsarma	2003
6261	0	0	0	2956	Alexanderharris	2002
6262	0	0	0	2956	Kianmohammadi	2004
6263	0	0	0	2956	Zhiyuanwei	2002
6264	0	0	0	2956	Seungminpark	2005
6265	0	0	0	2956	Hae Jinlee	2004
6266	0	0	0	2956	Albertkirsten	2002
6267	0	0	0	2956	Md Juelrana	2002
6268	0	0	0	2956	Othmanbezzaz	2005
6269	0	0	0	2956	Mohamedalami	2002
6270	0	0	0	2956	Antreasevlavis	2003
6271	0	0	0	2956	Oleksandrponomar	2006
6272	0	0	0	2956	Pedrocressoni	2003
6273	0	0	0	2956	Bruno Carlosbacigalupi carrizos	2003
6274	0	0	0	2956	Robertopinargote palma	2002
6275	0	0	0	2956	Enzobordini garutti	2003
6276	0	0	0	2956	Mario Alejandrovirreira toro	2003
6277	0	0	0	2956	Bernardoperalta	2003
6278	0	0	0	2956	Rodrigo Alonsotabormina araya	2003
6279	0	0	0	2956	Henriquebaggio	2003
6280	0	0	0	2956	Ezequielarce gonzalez	2003
6281	0	0	0	2956	Jose Luisclaro	2003
6282	0	0	0	2956	Matias Danielacosta pino	2004
6283	0	0	0	2956	Aril Kayratuna	2006
6284	0	0	0	2956	Omarhamzaoui	2005
6285	0	0	0	2956	Rafaelsegado esteve	2006
6286	0	0	0	2956	Masakiyamada	2003
6287	0	0	0	2956	Marlonomdahl	2002
6288	0	0	0	2956	Arielbouskila	2004
6289	0	0	0	2956	Gabrielberner	2004
6290	0	0	0	2956	Pablocruz morales	2002
6291	0	0	0	2956	Napathuadcharoen	2005
6292	0	0	0	2956	Shouryamanik	2002
6293	0	0	0	2956	Isaacsmith	2003
6294	0	0	0	2956	Muniananth mani	2004
6295	0	0	0	2956	Taymurmuhammad	2006
6296	0	0	0	2956	Jose Migueluzcategui	2004

6297	0	0	0	2956	Enzocisternino	2003
6298	0	0	0	2956	Alex Javieraguiar	2003
6299	0	0	0	2956	Carlorosellini	2002
6300	0	0	0	2956	Alejandroestrada	2002
6301	0	0	0	2956	Michaelhappy	2004
6302	0	0	0	2956	Aidanbyrne	2004
6303	0	0	0	2956	Charliecowan	2004
6304	0	0	0	2956	Vivekkolluru	2004
6305	0	0	0	2956	Jaden Dewandakatan	2006
6306	0	0	0	2956	Andreyalexeev	2003
6307	0	0	0	2956	Alexeygavrilin	2003
6308	0	0	0	2956	Kodaikato	2003
6309	0	0	0	2956	Jose Armandosequeira sanchez	2003
6310	0	0	0	2956	Aaronrittberger	2003
6311	0	0	0	2956	Sibusisoqame	2004
6312	0	0	0	2956	KAIhitomi	2003
6313	0	0	0	2956	Enricochicone recchia	2002
6314	0	0	0	2956	Jose Antoniomartinez toral	2002
6315	0	0	0	2956	Balazsguenther	2005
6316	0	0	0	2956	Yunzhang	2005
6317	0	0	0	2956	Jingshenwang	2003
6318	0	0	0	2956	Alejandroacebal	2004
6319	0	0	0	2956	Patrickfindlay	2004
6320	0	0	0	2956	Shreyarora	2003
6321	0	0	0	2956	Ryanlyn	2004
6322	0	0	0	2956	Jamesbradley	2004
6323	0	0	0	2956	Sachagrandvincent	2005
6324	0	0	0	2956	Bekhruzmirjalilov	2005
6325	0	0	0	2956	Mateogalvez	2005
6326	0	0	0	2956	Lanja Ny Ainarakotozandriny	2003
6327	0	0	0	2956	Santiagobaez bolanos	2004
6328	0	0	0	2956	anthonyburrows	2005
6329	0	0	0	2956	Yu-techou	2003
6330	0	0	0	2956	Wooinlee	2006
6331	0	0	0	2956	Matejamilosevic	2004
6332	0	0	0	2956	Alexmarino	2004
6333	0	0	0	2956	Faiznasyam	2003
6334	0	0	0	2956	Jeff Gatiensodji	2004
6335	0	0	0	2956	Takamasamishiro	2005
6336	0	0	0	2956	Hasheeshkumar	2004
6337	0	0	0	2956	Sebastianfigueira	2004
6338	0	0	0	2956	Pablopenagos	2005
6339	0	0	0	2956	Luis Ignaciorosselot	2002
6340	0	0	0	2956	Paultruong	2004
6341	0	0	0	2956	Saimchouldry	2004
6342	0	0	0	2956	Arunavamajumder	2005
6343	0	0	0	2956	Nicolas Rafaelgoldberg alviani	2002
6344	0	0	0	2956	Thiagodrozdowski	2006
6345	0	0	0	2956	Lucas Ramiroaugotr	2002
6346	0	0	0	2956	Artembek	2003

6347	0	0	0	2956	Andreaspetsalis	2002
6348	0	0	0	2956	Mohammad parsaafsari	2004
6349	0	0	0	2956	Jamesmackenzie	2003
6350	0	0	0	2956	Siddharth Srinivastirupati	2004
6351	0	0	0	2956	Alejandrobancalari	2004
6352	0	0	0	2956	Gustavo Adolfopacheco	2003
6353	0	0	0	2956	Rhajzonrankins	2002
6354	0	0	0	2956	SAMUELallais	2003
6355	0	0	0	2956	Seon Junji	2004
6356	0	0	0	2956	Shantanupowar	2002
6357	0	0	0	2956	Arjunshah	2003
6358	0	0	0	2956	Andres Rodolfoovalle najera	2004
6359	0	0	0	2956	Peytonwilliams	2004
6360	0	0	0	2956	Egorrybakov	2006
6361	0	0	0	2956	Mariorichmagui	2006
6362	0	0	0	2956	Muhammadreza falevi	2002
6363	0	0	0	2956	Brandonbeta	2004
6364	0	0	0	2956	Augustus Tetteytetteh	2003
6365	0	0	0	2956	Zacharysands	2005
6366	0	0	0	2956	Harrypugh	2006
6367	0	0	0	2956	Jackchapman	2006
6368	0	0	0	2956	Mahatir Muhammadkhan	2005
6369	0	0	0	2956	Clandestine Kudakwashendiring	2006
6370	0	0	0	2956	Santiagoflyckt	2004
6371	0	0	0	2956	Naokiwatanabe	2005
6372	0	0	0	2956	Andrasszilagyi	2002
6373	0	0	0	2956	Eliasruusuvuori	2004
6374	0	0	0	2956	Atahanniyazi	2006
6375	0	0	0	2956	Dhanunaiphukesorn	2006
6376	0	0	0	2956	Mahmoud salimgannouni	2007
6377	0	0	0	2956	Mohamed Sanadbouhlel	2006
6378	0	0	0	2956	Evgeniysoushkevich	2003
6379	0	0	0	2956	Benjaminayuba	2004
6380	0	0	0	2956	Wareatigona	2003
6381	0	0	0	2956	Rafaelkimati lima	2003
6382	0	0	0	2956	Louisboisdenghien	2002
6383	0	0	0	2956	Kokimasaki	2004
6384	0	0	0	2956	Nicolas Eliasandonie bono	2004
6385	0	0	0	2956	Cooperwilkinson	2004
6386	0	0	0	2956	Ivananhelov	2003
6387	0	0	0	2956	Nicholasshabelsky	2003
6388	0	0	0	2956	Conradbeneke	2005
6389	0	0	0	2956	TSAIchuang ju	2002
6390	0	0	0	2956	Kossi Andredogbefu	2005
6391	0	0	0	2956	Gerald Billkruger	2005
6392	0	0	0	2956	Luis Enriqueperez penaloza	2004
6393	0	0	0	2956	Dzhordzhobinda	2005
6394	0	0	0	2956	Stepanmatsuk	2004
6395	0	0	0	2956	Ritviknadikude	2004
6396	0	0	0	2956	Ivanwularu	2003

6397	0	0	0	2956	Ernestorequena	2003
6398	0	0	0	2956	Ryanfox	2003
6399	0	0	0	2956	Omarsuarez berrezueta	2004
6400	0	0	0	2956	Pratyushmohanty	2002
6401	1	1	1	1	Thiago Agustintirante	2001
6402	1	1	1	2	Holgerrune	2003
6403	1	1	1	3	Jonasforejtek	2001
6404	1	1	1	4	Shintaromochizuki	2003
6405	1	1	1	5	Haroldmayot	2002
6406	1	1	1	6	Martindamm	2003
6407	1	1	1	7	Emilionava	2001
6408	1	1	1	8	Valentinroyer	2001
6409	1	1	1	9	Tobykodat	2003
6410	1	1	1	10	Shunsukemitsui	2002
6411	1	1	1	11	Jirilehecka	2001
6412	0	1	1	12	Jeffreyvon der schulenburg	2002
6413	0	1	1	13	Arthurcazaux	2002
6414	0	1	1	14	Leandroriedi	2002
6415	0	1	1	15	Dominicstricker	2002
6416	0	1	1	16	Liamdraxl	2001
6417	0	1	1	17	Khololwammontsi	2002
6418	0	1	1	18	Flaviocobolli	2002
6419	0	1	1	19	Gauthieronclin	2001
6420	0	1	1	20	Daliborsvrcina	2002
6421	0	1	1	21	Juan Bautistatorres	2002
6422	0	1	1	22	Illyabeloborodko	2001
6423	0	1	1	23	Petermakk	2001
6424	0	1	1	24	Eliakimcoulibaly	2002
6425	0	1	1	25	Cannonkingsley	2001
6426	0	1	1	26	Karlisozolins	2002
6427	0	0	1	27	Hanwenli	2002
6428	0	0	1	28	Tristanschoolkate	2001
6429	0	0	1	29	Yunchaoketebu	2002
6430	0	0	1	30	Nicolasalvarez varona	2001
6431	0	0	1	31	Matheuspucinelli de almeida	2001
6432	0	0	1	32	Matteoarnaldi	2001
6433	0	0	1	33	Arthurfery	2002
6434	0	0	1	34	Alexanderzgirovsky	2001
6435	0	0	1	35	Carlosalcaraz	2003
6436	0	0	1	36	Tylerzink	2001
6437	0	0	1	37	Kevinchahoud	2001
6438	0	0	1	38	Pablollamas ruiz	2002
6439	0	0	1	39	Wojciechmarek	2001
6440	0	0	1	40	Timolegout	2002
6441	0	0	1	41	Tahabaadi	2001
6442	0	0	1	42	Roman Andresburruchaga	2002
6443	0	0	1	43	Eliotspizzirri	2001
6444	0	0	1	44	Andrewpaulson	2001
6445	0	0	1	45	Nicholas Davidionel	2002
6446	0	0	1	46	Mikolajlorens	2002

6447	0	0	1	47	Daliblanch	2003
6448	0	0	1	48	Keisukesaitoh	2001
6449	0	0	1	49	Lukasneumayer	2002
6450	0	0	1	50	Harrywendelken	2001
6451	0	0	1	51	Francescopassaro	2001
6452	0	0	0	52	Stijnpel	2002
6453	0	0	0	53	Danesweeny	2001
6454	0	0	0	54	Ericvanshelboim	2001
6455	0	0	0	55	Youcefrihane	2001
6456	0	0	0	56	Sebastiangima	2002
6457	0	0	0	57	Williamgrant	2001
6458	0	0	0	58	Sebastiannothhaft	2001
6459	0	0	0	59	Giovannimpetshi perricard	2003
6460	0	0	0	60	Alejo Lorenzolingua lavallen	2001
6461	0	0	0	61	Taiyoyamanaka	2001
6462	0	0	0	62	Natanrodrigues	2002
6463	0	0	0	63	Maxwiskandt	2002
6464	0	0	0	64	Blubaker	2001
6465	0	0	0	65	Lilianmarmousez	2002
6466	0	0	0	66	Ryuheiazuma	2001
6467	0	0	0	67	Ninoehrenschneider	2001
6468	0	0	0	68	Andrewdale	2001
6469	0	0	0	69	Aidanmayo	2003
6470	0	0	0	70	Baptisteanselmo	2001
6471	0	0	0	71	Chendong	2002
6472	0	0	0	72	Nini Gabrieldica	2001
6473	0	0	0	73	Martinbreysach	2002
6474	0	0	0	74	Stefanstorch	2001
6475	0	0	0	75	Rinkyhijikata	2001
6476	0	0	0	76	Brunooliveira	2002
6477	0	0	0	77	Gian Lucatanner	2001
6478	0	0	0	78	Lorispourroy	2001
6479	0	0	0	79	Andresmartin	2001
6480	0	0	0	80	Benitosanchez martinez	2002
6481	0	0	0	81	Cashhanzlik	2002
6482	0	0	0	82	Lodewijkweststrate	2001
6483	0	0	0	83	Blaisebicknell	2001
6484	0	0	0	84	Ryomamatsushita	2001
6485	0	0	0	85	Joao Victorcouto loureiro	2003
6486	0	0	0	86	Joubertklopper	2001
6487	0	0	0	87	Markotopo	2003
6488	0	0	0	88	Kokoroisomura	2003
6489	0	0	0	89	Pedroboscardin dias	2003
6490	0	0	0	90	Ottovirtanen	2001
6491	0	0	0	91	Lucianodarderi	2002
6492	0	0	0	92	Adrienburdet	2002
6493	0	0	0	93	Tomleblanc claverie cazassus	2001
6494	0	0	0	94	Franciscollanes	2002
6495	0	0	0	95	Angelguerrero melgar	2002
6496	0	0	0	96	Cezarcretu	2001

6497	0	0	0	97	Jamesstory	2001
6498	0	0	0	98	Christianlerby	2001
6499	0	0	0	99	Manuelmouilleron salvo	2003
6500	0	0	0	100	Milledgecossu	2001
6501	0	0	0	101	Charlmorgan	2001
6502	0	0	0	102	Phuong Vannguyen	2001
6503	1	1	1	1	Chun Hsintseng	2001
6504	1	1	1	2	Hugogaston	2000
6505	1	1	1	3	Sebastianbaez	2000
6506	1	1	1	4	Adrianandreev	2001
6507	1	1	1	5	Brandonnakashima	2001
6508	1	1	1	6	Facundodiaz acosta	2000
6509	1	1	1	7	Nicolasmejia	2000
6510	1	1	1	8	Lorenzomusetti	2002
6511	1	1	1	9	Sebastiankorda	2000
6512	1	1	1	10	Gilbertklier junior	2000
6513	1	1	1	11	Ottovirtanen	2001
6514	0	1	1	12	Jackdraper	2001
6515	0	1	1	13	Filip Cristianjianu	2001
6516	0	1	1	14	Rinkyhijikata	2001
6517	0	1	1	15	Naokitajima	2000
6518	0	1	1	16	Nicolasalvarez varona	2001
6519	0	1	1	17	Clementtabur	2000
6520	0	1	1	18	Gauthieronclin	2001
6521	0	1	1	19	Nickhardt	2000
6522	0	1	1	20	Yankierel	2000
6523	0	1	1	21	Daliborsvrcina	2002
6524	0	1	1	22	Antonmatusevich	2001
6525	0	1	1	23	Carloslopez montagud	2000
6526	0	1	1	24	Jonasforejtek	2001
6527	0	1	1	25	Taomu	2000
6528	0	1	1	26	Ondrejstyler	2000
6529	0	1	1	27	Arnaudbovy	2000
6530	0	1	1	28	Cannonkingsley	2001
6531	0	0	1	29	Valentinroyer	2001
6532	0	0	1	30	Deneywassermann	2001
6533	0	0	1	31	Arthurcazaux	2002
6534	0	0	1	32	Damienwenger	2000
6535	0	0	1	33	Tristanboyer	2001
6536	0	0	1	34	Emilionava	2001
6537	0	0	1	35	Haroldmayot	2002
6538	0	0	1	36	Liamdraxl	2001
6539	0	0	1	37	Aidanmchugh	2000
6540	0	0	1	38	Sergeyfomin	2001
6541	0	0	1	39	Eliotspizzirri	2001
6542	0	0	1	40	Zanekhan	2002
6543	0	0	1	41	Jesperde jong	2000
6544	0	0	1	42	Keisukesaitoh	2001
6545	0	0	1	43	Tylerzink	2001
6546	0	0	1	44	Danielmichalski	2000

6547	0	0	1	45	Mateusalves	2001
6548	0	0	1	46	Santiagode la fuente	2002
6549	0	0	1	47	Govindnanda	2001
6550	0	0	1	48	Giuliozeppieri	2001
6551	0	0	1	49	Alejo Lorenzolingua lavallen	2001
6552	0	0	1	50	Lucianotacchi	2001
6553	0	0	1	51	Drewbaird	2000
6554	0	0	1	52	Taiseiichikawa	2000
6555	0	0	1	53	Rayho	2000
6556	0	0	1	54	Natanrodrigues	2002
6557	0	0	1	55	Jacobfearnley	2001
6558	0	0	0	56	Harrywendelken	2001
6559	0	0	0	57	Seonyonghan	2001
6560	0	0	0	58	Timofeiskatov	2001
6561	0	0	0	59	Henryvon der schulenburg	2000
6562	0	0	0	60	Louisherman	2001
6563	0	0	0	61	Philiphenning	2000
6564	0	0	0	62	Shunsukemitsui	2002
6565	0	0	0	63	Joao Lucasreis da silva	2000
6566	0	0	0	64	Siddhantbanthia	2000
6567	0	0	0	65	Lodewijkweststrate	2001
6568	0	0	0	66	Wojciechmarek	2001
6569	0	0	0	67	Tahabaadi	2001
6570	0	0	0	68	Leopoldzima	2000
6571	0	0	0	69	Matheuspucinelli de almeida	2001
6572	0	0	0	70	Treyhilderbrand	2000
6573	0	0	0	71	Justinschlageter	2001
6574	0	0	0	72	Alexandrbinda	2001
6575	0	0	0	73	Admirkalender	2001
6576	0	0	0	74	Davidetortora	2000
6577	0	0	0	75	Juan Manuelcerundolo	2001
6578	0	0	0	76	Tobykodat	2003
6579	0	0	0	77	Juan Bautistatorres	2002
6580	0	0	0	78	Ki Lungng	2001
6581	0	0	0	79	Henrisquire	2000
6582	0	0	0	80	Roman Andresburruchaga	2002
6583	0	0	0	81	Igorgimenez	2000
6584	0	0	0	82	Borasengul	2001
6585	0	0	0	83	Filipkolasinski	2000
6586	0	0	0	84	Christianlerby	2001
6587	0	0	0	85	Cezarcretu	2001
6588	0	0	0	86	Keenanmayo	2000
6589	0	0	0	87	Titouandroguet	2001
6590	0	0	0	88	Rokohorvat	2001
6591	0	0	0	89	Mathyserhard	2001
6592	0	0	0	90	Lilianmarmousez	2002
6593	0	0	0	91	Sebastianrodriguez	2001
6594	0	0	0	92	Minjongpark	2001
6595	0	0	0	93	Williamwoodall	2000
6596	0	0	0	94	Thiago Agustintirante	2001

6597	0	0	0	95	Daniilglinka	2000
6598	0	0	0	96	Joaoferreira	2001
6599	0	0	0	97	Ronanjachuck	2001
6600	0	0	0	98	Dostanbektashbulatov	2000
6601	0	0	0	99	Arthur Craigpantino	2001
6602	0	0	0	100	Williamgrant	2001
6603	0	0	0	101	Christian Didierchin	2000
6604	0	0	0	102	Alexanderlee	2002
6605	0	0	0	103	Erikgrevelius	2000
6606	0	0	0	104	Andrewfenty	2000
6607	0	0	0	105	Brandonperez	2000
6608	0	0	0	106	Stefanpalosi	2000
6609	0	0	0	107	Mateobarreiros reyes	2000
6610	0	0	0	108	Murphycassone	2002
6611	0	0	0	109	Neelrajesh	2000
6612	1	1	1	1	Axelgeller	1999
6613	1	1	1	2	Yibingwu	1999
6614	1	1	1	3	Alexeipopyrin	1999
6615	1	1	1	4	Timofeiskatov	2001
6616	1	1	1	5	Emilruusuvuori	1999
6617	1	1	1	6	Zsomborpiros	1999
6618	1	1	1	7	Markomiladinovic	2000
6619	1	1	1	8	Jurijrodionov	1999
6620	1	1	1	9	Yutashimizu	1999
6621	1	1	1	10	Uisungpark	2000
6622	1	1	1	11	Sebastianbaez	2000
6623	0	1	1	12	Corentinmoutet	1999
6624	0	1	1	13	Hugogaston	2000
6625	0	1	1	14	Sebastiankorda	2000
6626	0	1	1	15	Trentbryde	1999
6627	0	1	1	16	Chun Hsintseng	2001
6628	0	1	1	17	Olivercrawford	1999
6629	0	1	1	18	Yu Hsiouhsu	1999
6630	0	1	1	19	Nicolasmejia	2000
6631	0	1	1	20	Thiagoseyboth wild	2000
6632	0	1	1	21	Michaelvrbensky	1999
6633	0	1	1	22	Constantinbittoun kouzmine	1999
6634	0	1	1	23	Juan Manuelcerundolo	2001
6635	0	1	1	24	Thiago Agustintirante	2001
6636	0	1	1	25	Alexeyzakharov	2000
6637	0	1	1	26	Andrewfenty	2000
6638	0	0	1	27	Matteomartineau	1999
6639	0	0	1	28	Ondrejstyler	2000
6640	0	0	1	29	Naokitajima	2000
6641	0	0	1	30	Patrickkypson	1999
6642	0	0	1	31	Tomasmachac	2000
6643	0	0	1	32	Yshaioliel	2000
6644	0	0	1	33	Alan Fernandorubio fierros	1999
6645	0	0	1	34	Tristanboyer	2001
6646	0	0	1	35	Zizoubergs	1999

6647	0	0	1	36	Alexandrerotsaert	1999
6648	0	0	1	37	Stefanpalosi	2000
6649	0	0	1	38	Duartevale	1999
6650	0	0	1	39	Vasilkirkov	1999
6651	0	0	1	40	Gianniross	1999
6652	0	0	1	41	Alafiaayeni	1999
6653	0	0	1	42	Dannythomas	1999
6654	0	0	1	43	Georgeloffhagen	2001
6655	0	0	1	44	Samriffice	1999
6656	0	0	1	45	Juan Pablograssi mazzuchi	1999
6657	0	0	1	46	Drewbaird	2000
6658	0	0	1	47	Nickhardt	2000
6659	0	0	1	48	Meliosefstathiou	1999
6660	0	0	1	49	Aidanmchugh	2000
6661	0	0	1	50	Carloslopez montagud	2000
6662	0	0	1	51	Francescoforti	1999
6663	0	0	1	52	Simoncarr	1999
6664	0	0	0	53	Joao Lucasreis da silva	2000
6665	0	0	0	54	Matheuspucinelli de almeida	2001
6666	0	0	0	55	Rudolfmolleker	2000
6667	0	0	0	56	Danadded	1999
6668	0	0	0	57	Mohamed Alibellalouna	1999
6669	0	0	0	58	Filip Cristianjianu	2001
6670	0	0	0	59	Taiseiichikawa	2000
6671	0	0	0	60	Briancernoch	1999
6672	0	0	0	61	Siddhantbanthia	2000
6673	0	0	0	62	Nikolayvylegzhanin	2000
6674	0	0	0	63	Artemdubrivnyy	1999
6675	0	0	0	64	Igorgimenez	2000
6676	0	0	0	65	Ryannijboer	1999
6677	0	0	0	66	Thomasbosancic	2000
6678	0	0	0	67	Leonidsheyngezikht	2000
6679	0	0	0	68	Jackdraper	2001
6680	0	0	0	69	James Kenttrotter	1999
6681	0	0	0	70	Sangeetsridhar	2000
6682	0	0	0	71	Gilbertklier junior	2000
6683	0	0	0	72	Benard Brunonkomba	1999
6684	0	0	0	73	Axelnefve	2000
6685	0	0	0	74	Barnabysmith	1999
6686	0	0	0	75	Jesperde jong	2000
6687	0	0	0	76	Brianshi	2000
6688	0	0	0	77	Seonyonghan	2001
6689	0	0	0	78	Shinjihazawa	1999
6690	0	0	0	79	Maxencebroville	1999
6691	0	0	0	80	Philiphenning	2000
6692	0	0	0	81	Mikhailsokolovskiy	1999
6693	0	0	0	82	Jaimee Floydangele	2000
6694	0	0	0	83	Valentinroyer	2001
6695	0	0	0	84	Tylerzink	2001
6696	0	0	0	85	Matiassoto	1999

6697	0	0	0	86	Alexhernandez	1999
6698	0	0	0	87	Emilehudd	2000
6699	0	0	0	88	Ryangoetz	1999
6700	0	0	0	89	Mattiafrinzi	1999
6701	0	0	0	90	Sergio Luishernandez ramirez	1999
6702	0	0	0	91	Leopoldzima	2000
6703	0	0	0	92	Ajeetrai	1999
6704	0	0	0	93	Bang Shuoyin	1999
6705	0	0	0	94	Tomaskopczynski	2000
6706	0	0	0	95	Toruhorie	1999
6707	0	0	0	96	Brandonperez	2000
6708	0	0	0	97	Lukasgreif	1999
6709	0	0	0	98	Diegoriviere padilha	2000
6710	0	0	0	99	Boriskozlov	2000
6711	0	0	0	100	Gabrieldecamps	1999
6712	0	0	0	101	Markmandlik	2001
6713	0	0	0	102	Garrettjohns	2000
6714	0	0	0	103	Anujwatane	2000
6715	1	1	1	1	Miomirkecmanovic	1999
6716	1	1	1	2	Stefanostsitsipas	1998
6717	1	1	1	3	Yosukewatanuki	1998
6718	1	1	1	4	Felixauger-aliassime	2000
6719	1	1	1	5	Ulisesblanch	1998
6720	1	1	1	6	Yibingwu	1999
6721	1	1	1	7	Geoffreyblancaneaux	1998
6722	1	1	1	8	Jurabekkarimov	1998
6723	1	1	1	9	Benjaminsigouin	1999
6724	0	1	1	10	Nicolakuhn	2000
6725	0	1	1	11	Alexde minaur	1999
6726	0	1	1	12	Yutashimizu	1999
6727	0	1	1	13	Kennethraisma	1998
6728	0	1	1	14	Jurijrodionov	1999
6729	0	1	1	15	Johnmcnally	1998
6730	0	1	1	16	Marvinmoeller	1999
6731	0	1	1	17	Alejandrodavidovich fokina	1999
6732	0	1	1	18	Yshaioliel	2000
6733	0	1	1	19	Louiswessels	1998
6734	0	1	1	20	Tomas Martinetcheverry	1999
6735	0	1	1	21	Liamcaruana	1998
6736	0	1	1	22	Duartevale	1999
6737	0	1	1	23	Rudolfmolleker	2000
6738	0	1	1	24	Genaro Albertoolivieri	1998
6739	0	1	1	25	Gabrieldecamps	1999
6740	0	1	1	26	Alexeipopyrin	1999
6741	0	0	1	27	Juan Carlosaguilar	1998
6742	0	0	1	28	JJwolf	1998
6743	0	0	1	29	Ryan Jamesstorrie	1998
6744	0	0	1	30	Youssefhossam	1998
6745	0	0	1	31	Khumoyunsultanov	1998
6746	0	0	1	32	Naokitajima	2000

6747	0	0	1	33	Chien Hsunlo	1998
6748	0	0	1	34	Franciscovittar	1999
6749	0	0	1	35	Toruhorie	1999
6750	0	0	1	36	Danadded	1999
6751	0	0	1	37	Kacperzuk	1999
6752	0	0	1	38	Elliotbenchetrit	1998
6753	0	0	1	39	Samriffice	1999
6754	0	0	1	40	Piotrmatuszewski	1998
6755	0	0	1	41	Ergikirkin	1999
6756	0	0	1	42	Lingxizhao	2001
6757	0	0	1	43	Trentbryde	1999
6758	0	0	1	44	Vasilkirkov	1999
6759	0	0	1	45	Olivercrawford	1999
6760	0	0	1	46	Blakeellis	1999
6761	0	0	1	47	Albertolim	1999
6762	0	0	1	48	Felipemeligeni alves	1998
6763	0	0	1	49	Gianniross	1999
6764	0	0	1	50	Zsomborpiros	1999
6765	0	0	1	51	Juan Martinjalif	1999
6766	0	0	1	52	Alastairgray	1998
6767	0	0	0	53	Alexiscanter	1998
6768	0	0	0	54	Constantinbittoun kouzmine	1999
6769	0	0	0	55	Evanfurness	1998
6770	0	0	0	56	Eduardguell	1998
6771	0	0	0	57	Mikhailsokolovskiy	1999
6772	0	0	0	58	Shinjihazawa	1999
6773	0	0	0	59	Finnbass	1999
6774	0	0	0	60	Matteomartineau	1999
6775	0	0	0	61	Louistessa	1998
6776	0	0	0	62	Nathanponwith	1998
6777	0	0	0	63	Anthony Jackietang	1998
6778	0	0	0	64	Vlad Andreidancu	1999
6779	0	0	0	65	Levkazakov	1998
6780	0	0	0	66	Patrickkypson	1999
6781	0	0	0	67	Seppecuypers	1998
6782	0	0	0	68	Matevalkusz	1998
6783	0	0	0	69	Alexandrerotsaert	1999
6784	0	0	0	70	Alan Fernandorubio fierros	1999
6785	0	0	0	71	Alafiaayeni	1999
6786	0	0	0	72	Riccardobalzerani	1998
6787	0	0	0	73	Lukasklein	1998
6788	0	0	0	74	Tung-Linwu	1998
6789	0	0	0	75	Briancernoch	1999
6790	0	0	0	76	Mattiassiimar	1998
6791	0	0	0	77	Ezekielclark	1998
6792	0	0	0	78	Thiagoseyboth wild	2000
6793	0	0	0	79	Lucaskoelle	1998
6794	0	0	0	80	Chengzelyu	1998
6795	0	0	0	81	James Kenttrotter	1999
6796	0	0	0	82	Jayclarke	1998

6797	0	0	0	83	Sebastianarcila	1998
6798	0	0	0	84	Pavelkotov	1998
6799	0	0	0	85	Corradosummaria	1998
6800	0	0	0	86	Brandonholt	1998
6801	0	0	0	87	Keenanmayo	2000
6802	0	0	0	88	Jack Mingjielin	1999
6803	0	0	0	89	Ewanmoore	1998
6804	0	0	0	90	Andres Gabrielciurletti	1998
6805	1	1	1	1	Taylorfritz	1997
6806	1	1	1	2	Casperruud	1998
6807	1	1	1	3	Matevalkusz	1998
6808	1	1	1	4	Seongchanhong	1997
6809	1	1	1	5	Tomasbarrios vera	1997
6810	1	1	1	6	Orlandoluz	1998
6811	1	1	1	7	Mikaelymer	1998
6812	1	1	1	8	Miomirkecmanovic	1999
6813	1	1	1	9	Williamblumberg	1998
6814	1	1	1	10	Alvarolopez san martin	1997
6815	1	1	1	11	Corentindenolly	1997
6816	0	1	1	12	Akirasantillan	1997
6817	0	1	1	13	Nam Hoangly	1997
6818	0	1	1	14	Stefanostsitsipas	1998
6819	0	1	1	15	Yun Seongchung	1998
6820	0	1	1	16	Alexde minaur	1999
6821	0	1	1	17	Manuelpena lopez	1998
6822	0	1	1	18	Alexmolcan	1997
6823	0	1	1	19	Albertolim	1999
6824	0	1	1	20	Michaelmmoh	1998
6825	0	1	1	21	Francocapalbo	1997
6826	0	1	1	22	Jurabekkarimov	1998
6827	0	1	1	23	Jayclarke	1998
6828	0	1	1	24	Patrikniklas-salminen	1997
6829	0	1	1	25	Ulisesblanch	1998
6830	0	1	1	26	Genaro Albertoolivieri	1998
6831	0	0	1	27	Ugohumbert	1998
6832	0	0	1	28	Youssefhossam	1998
6833	0	0	1	29	Nathanponwith	1998
6834	0	0	1	30	Andreapellegrino	1997
6835	0	0	1	31	Yosukewatanuki	1998
6836	0	0	1	32	Geoffreyblancaneaux	1998
6837	0	0	1	33	Benjaminsigouin	1999
6838	0	0	1	34	Yibingwu	1999
6839	0	0	1	35	Kennethraisma	1998
6840	0	0	1	36	Denisshapovalov	1999
6841	0	0	1	37	Lukasklein	1998
6842	0	0	1	38	Denisklok	1998
6843	0	0	1	39	Tung-Linwu	1998
6844	0	0	1	40	Felipecunha silva	1997
6845	0	0	1	41	Geronimoespin busleiman	1997
6846	0	0	1	42	Alexanderbublik	1997

6847	0	0	1	43	Chan-Yeongoh	1998
6848	0	0	1	44	Nunoborges	1997
6849	0	0	1	45	Gian Marcomoroni	1998
6850	0	0	1	46	Huberthurkacz	1997
6851	0	0	1	47	Samriffice	1999
6852	0	0	1	48	Yusuketakahashi	1997
6853	0	0	1	49	Sumitnagal	1997
6854	0	0	1	50	Benjaminhannestad	1997
6855	0	0	1	51	Timsandkaulen	1997
6856	0	0	1	52	Alexandarlazarov	1997
6857	0	0	0	53	Igormarcondes	1997
6858	0	0	0	54	Alexeipopyrin	1999
6859	0	0	0	55	Rentatokuda	1998
6860	0	0	0	56	Eduardguell	1998
6861	0	0	0	57	Mattiassiimar	1998
6862	0	0	0	58	Alexeyaleshchev	1998
6863	0	0	0	59	Louiswessels	1998
6864	0	0	0	60	Sorafukuda	1997
6865	0	0	0	61	Yshaioliel	2000
6866	0	0	0	62	Marcpolmans	1997
6867	0	0	0	63	Juan Joserosas	1997
6868	0	0	0	64	Markoosmakcic	1998
6869	0	0	0	65	Michaldembek	1997
6870	0	0	0	66	Federicobonacia	1997
6871	0	0	0	67	Emilreinberg	1997
6872	0	0	0	68	Yuyaito	1997
6873	0	0	0	69	Vasilkirkov	1999
6874	0	0	0	70	Chien Hsunlo	1998
6875	0	0	0	71	Marcomosciatti	1997
6876	0	0	0	72	Jakedelaney	1997
6877	0	0	0	73	Ergikirkin	1999
6878	0	0	0	74	Piotrmatuszewski	1998
6879	0	0	0	75	Azizdougaz	1997
6880	0	0	0	76	Felipemeligeni alves	1998
6881	0	0	0	77	Liamcaruana	1998
6882	0	0	0	78	Ezekielclark	1998
6883	0	0	0	79	Eerovasa	1997
6884	0	0	0	80	Lucaskoelle	1998
6885	0	0	0	81	Bjornthomson	1997
6886	0	0	0	82	Gabrieldecamps	1999
6887	0	0	0	83	Alexiscanter	1998
6888	0	0	0	84	Ryan Jamesstorrie	1998
6889	0	0	0	85	Tomas Martinetcheverry	1999
6890	0	0	0	86	Brunobritez	1998
6891	0	0	0	87	Bogdanbobrov	1997
6892	0	0	0	88	Juan Carlosaguilar	1998
6893	0	0	0	89	Julianocleppo	1997
6894	0	0	0	90	Constantinbittoun kouzmine	1999
6895	0	0	0	91	Catalinmateas	1997
6896	0	0	0	92	Danielnolan	1997

6897	0	0	0	93	Nathanperrone	1998
6898	0	0	0	94	Trentbryde	1999
6899	0	0	0	95	Elliotbenchetrit	1998
6900	0	0	0	96	Alejandrotabilo	1997
6901	0	0	0	97	Gabrielroveri sidney	1997
6902	0	0	0	98	Mirkomartinez	1998
6903	0	0	0	99	Johannikles	1997
6904	0	0	0	100	Jumpeiyamasaki	1997
6905	0	0	0	101	Vasishtcheruku	1999
6906	0	0	0	102	Jakevan emburgh	1998
6907	0	0	0	103	Sameerkumar	1997
6908	0	0	0	104	Rafaellima wagner	1998
6909	1	1	1	1	Andreyrublev	1997
6910	1	1	1	2	Orlandoluz	1998
6911	1	1	1	3	Stefankozlov	1998
6912	1	1	1	4	Quentinhalys	1996
6913	1	1	1	5	Jaumemunar	1997
6914	1	1	1	6	Taylorfritz	1997
6915	1	1	1	7	Omarjasika	1997
6916	1	1	1	8	Yun Seongchung	1998
6917	1	1	1	9	Michaelmmoh	1998
6918	1	1	1	10	Johan Sebastientatlot	1996
6919	0	1	1	11	Naokinakagawa	1996
6920	0	1	1	12	Duckheelee	1998
6921	0	1	1	13	Marcelozormann	1996
6922	0	1	1	14	Kamilmajchrzak	1996
6923	0	1	1	15	Seongchanhong	1997
6924	0	1	1	16	Matiaszukas	1996
6925	0	1	1	17	Janchoinski	1996
6926	0	1	1	18	Jumpeiyamasaki	1997
6927	0	1	1	19	Romansafiullin	1997
6928	0	1	1	20	Mikaelymer	1998
6929	0	1	1	21	Nicolasalvarez	1996
6930	0	1	1	22	Petroschrysochos	1996
6931	0	1	1	23	Corentindenolly	1997
6932	0	1	1	24	Timvan rijthoven	1997
6933	0	1	1	25	Alexrybakov	1997
6934	0	0	1	26	Joaomenezes	1996
6935	0	0	1	27	Janzielinski	1996
6936	0	0	1	28	franciscobahamonde nicastro	1996
6937	0	0	1	29	Francestiafoe	1998
6938	0	0	1	30	Bogdanbobrov	1997
6939	0	0	1	31	Pedromartinez	1997
6940	0	0	1	32	Juan Joserosas	1997
6941	0	0	1	33	Stefanostsitsipas	1998
6942	0	0	1	34	Chan-Yeongoh	1998
6943	0	0	1	35	Clementgeens	1996
6944	0	0	1	36	Akirasantillan	1997
6945	0	0	1	37	Andrebiro	1996
6946	0	0	1	38	Harrybourchier	1996

6947	0	0	1	39	Henrikwiersholm	1997
6948	0	0	1	40	Lucasmiedler	1996
6949	0	0	1	41	Huberthurkacz	1997
6950	0	0	1	42	Danielappelgren	1996
6951	0	0	1	43	Nicolaefrunza	1997
6952	0	0	1	44	Sorafukuda	1997
6953	0	0	1	45	Alexanderbublik	1997
6954	0	0	1	46	Logansmith	1996
6955	0	0	1	47	Matteoberrettini	1996
6956	0	0	1	48	Ninoserdarusic	1996
6957	0	0	1	49	Alejandrotabilo	1997
6958	0	0	0	50	Dennisuspensky	1996
6959	0	0	0	51	Lloydharris	1997
6960	0	0	0	52	Miomirkecmanovic	1999
6961	0	0	0	53	Marcpolmans	1997
6962	0	0	0	54	Petriamachkine	1996
6963	0	0	0	55	Ryotaromatsumura	1996
6964	0	0	0	56	Corentinmoutet	1999
6965	0	0	0	57	Jordiarconada	1996
6966	0	0	0	58	Nam Hoangly	1997
6967	0	0	0	59	Martinblasko	1996
6968	0	0	0	60	Alexmolcan	1997
6969	0	0	0	61	Jurabekkarimov	1998
6970	0	0	0	62	Sameerkumar	1997
6971	0	0	0	63	Rafaelmatos	1996
6972	0	0	0	64	Johanneshaerteis	1996
6973	0	0	0	65	Petarconkic	1996
6974	0	0	0	66	Jakedelaney	1997
6975	0	0	0	67	Borispokotilov	1997
6976	0	0	0	68	Weiqiangzheng	1996
6977	0	0	0	69	Daniilmedvedev	1996
6978	0	0	0	70	Benjaminhannestad	1997
6979	0	0	0	71	Andreapellegrino	1997
6980	0	0	0	72	Gabrielroveri sidney	1997
6981	0	0	0	73	Markoosmakcic	1998
6982	0	0	0	74	Francocapalbo	1997
6983	0	0	0	75	Alexandersendegeya	1996
6984	0	0	0	76	Wei-Delin	1996
6985	0	0	0	77	Yusuketakahashi	1997
6986	0	0	0	78	Ku Keonkang	1997
6987	0	0	0	79	Eerovasa	1997
6988	0	0	0	80	Luisvalero	1996
6989	0	0	0	81	Makotoochi	1996
6990	0	0	0	82	Filippobaldi	1996
6991	0	0	0	83	Ulisesblanch	1998
6992	0	0	0	84	Clementlarriere	1997
6993	0	0	0	85	Peterbertran	1996
6994	0	0	0	86	Robertlevine	1997
6995	0	0	0	87	Rentatokuda	1998
6996	0	0	0	88	Emilreinberg	1997

6997	0	0	0	89	Igormarcondes	1997
6998	0	0	0	90	Alexiscanter	1998
6999	0	0	0	91	Jack Wvan slyke	1997
7000	0	0	0	92	Walkerduncan	1996
7001	0	0	0	93	Oscarjanglin	1997
7002	0	0	0	94	Alexanderlebedev	1997
7003	0	0	0	95	Simon Friissondergaard	1996
7004	0	0	0	96	Lucaskoelle	1998
7005	0	0	0	97	Danielhobart	1998
7006	1	1	1	1	Alexanderzverev	1997
7007	1	1	1	2	Bornacoric	1996
7008	1	1	1	3	Gianluigiquinzi	1996
7009	1	1	1	4	Nikolamilojevic	1995
7010	1	1	1	5	Stefankozlov	1998
7011	1	1	1	6	Cristiangarin	1996
7012	1	1	1	7	Johan Sebastientatlot	1996
7013	1	1	1	8	Francestiafoe	1998
7014	1	1	1	9	Michaelmmoh	1998
7015	1	1	1	10	Filippobaldi	1996
7016	0	1	1	11	Pedrocachin	1995
7017	0	1	1	12	Nickkyrgios	1995
7018	0	1	1	13	Romansafiullin	1997
7019	0	1	1	14	Quentinhalys	1996
7020	0	1	1	15	Guillermonunez	1995
7021	0	1	1	16	Maximehamou	1995
7022	0	1	1	17	Nicolasjarry	1995
7023	0	1	1	18	Daniilmedvedev	1996
7024	0	1	1	19	Naokinakagawa	1996
7025	0	1	1	20	Kamilmajchrzak	1996
7026	0	1	1	21	Duckheelee	1998
7027	0	1	1	22	Andreyrublev	1997
7028	0	1	1	23	Laslodjere	1995
7029	0	1	1	24	Karenkhachanov	1996
7030	0	1	1	25	Enzocouacaud	1995
7031	0	0	1	26	Jorge Brianpanta	1995
7032	0	0	1	27	Clementgeens	1996
7033	0	0	1	28	Yoshihitonishioka	1995
7034	0	0	1	29	Lucasmiedler	1996
7035	0	0	1	30	Jumpeiyamasaki	1997
7036	0	0	1	31	Marcelozormann	1996
7037	0	0	1	32	Hyeonchung	1996
7038	0	0	1	33	Cameronnorrie	1995
7039	0	0	1	34	Lucasgomez	1995
7040	0	0	1	35	Maximilianmarterer	1995
7041	0	0	1	36	Waynemontgomery	1995
7042	0	0	1	37	Bradleymousley	1996
7043	0	0	1	38	franciscobahamonde nicastro	1996
7044	0	0	1	39	Benjaminbonzi	1996
7045	0	0	1	40	Stefanonapolitano	1995
7046	0	0	1	41	Kenonishi	1996

7047	0	0	1	42	Lucacorinteli	1995
7048	0	0	1	43	Martinredlicki	1995
7049	0	0	1	44	Harrybourchier	1996
7050	0	0	1	45	Seongchanhong	1997
7051	0	0	1	46	Simon Friissondergaard	1996
7052	0	0	1	47	Rafaelmatos	1996
7053	0	0	1	48	Young Seokkim	1995
7054	0	0	1	49	Ku Keonkang	1997
7055	0	0	1	50	Hanneswagner	1995
7056	0	0	0	51	Lukebambridge	1995
7057	0	0	0	52	Johanneshaerteis	1996
7058	0	0	0	53	Omarjasika	1997
7059	0	0	0	54	Maximejanvier	1996
7060	0	0	0	55	Joaomenezes	1996
7061	0	0	0	56	Skandermansouri	1995
7062	0	0	0	57	Jaime Ignaciogalleguillos	1995
7063	0	0	0	58	Gabriel Vellinhohocevar	1996
7064	0	0	0	59	Spencerpapa	1995
7065	0	0	0	60	Paulwoerner	1996
7066	0	0	0	61	Noahrubin	1996
7067	0	0	0	62	Diegopedraza	1995
7068	0	0	0	63	Matteodonati	1995
7069	0	0	0	64	Joaowalendowsky	1995
7070	0	0	0	65	Danielwindahl	1996
7071	0	0	0	66	Bastianmalla	1996
7072	0	0	0	67	Weiqiangzheng	1996
7073	0	0	0	68	Sorafukuda	1997
7074	0	0	0	69	Yusuketakahashi	1997
7075	0	0	0	70	Jonnyo'mara	1995
7076	0	0	0	71	Takashisaito	1995
7077	0	0	0	72	Danielkerznerman	1996
7078	0	0	0	73	Tommymylnikov	1995
7079	0	0	0	74	Cemilkel	1995
7080	0	0	0	75	Juan Pablovarillas	1995
7081	0	0	0	76	Wishayatrongcharoenchaikul	1995
7082	0	0	0	77	Alexandremuller	1997
7083	0	0	0	78	Matejmaruscak	1995
7084	0	0	0	79	Dennisuspensky	1996
7085	0	0	0	80	Luisvalero	1996
7086	0	0	0	81	Rogeliosiller	1995
7087	0	0	0	82	Mackenziemcdonald	1995
7088	0	0	0	83	Adrianortiz	1995
7089	0	0	0	84	Alexandersendegeya	1996
7090	0	0	0	85	Jordiarconada	1996
7091	0	0	0	86	Guy Orlyiradukunda	1996
7092	0	0	0	87	Julianzlobinsky	1996
7093	0	0	0	88	Hugodi feo	1995
7094	0	0	0	89	Henrikwiersholm	1997
7095	0	0	0	90	Eliasymer	1996
7096	0	0	0	91	Leonardocivita-telles	1995

7097	0	0	0	92	Alannunez aguilera	1995
7098	0	0	0	93	Rafael Antoniocoutinho	1996
7099	0	0	0	94	Braydenschnur	1995
7100	0	0	0	95	Marcelotebet	1996
7101	0	0	0	96	Mazenosama	1995
7102	0	0	0	97	Luke Jacobgamble	1995
7103	0	0	0	98	Javierrestrepo	1996
7104	0	0	0	99	Ayedzatar	1996
7105	0	0	0	100	Andrestorres	1996
7106	1	1	1	1	Filippeliwo	1994
7107	1	1	1	2	Kimmercoppejans	1994
7108	1	1	1	3	Gianluigiquinzi	1996
7109	1	1	1	4	Nickkyrgios	1995
7110	1	1	1	5	Lukesaville	1994
7111	1	1	1	6	Fredericoferreira silva	1995
7112	1	1	1	7	Laslodjere	1995
7113	1	1	1	8	Nikolamilojevic	1995
7114	1	1	1	9	Kaichiuchida	1994
7115	1	1	1	10	Eliasymer	1996
7116	1	1	1	11	Mateo Nicolasmartinez	1994
7117	0	1	1	12	Noahrubin	1996
7118	0	1	1	13	Cristiangarin	1996
7119	0	1	1	14	Liambroady	1994
7120	0	1	1	15	Stefanonapolitano	1995
7121	0	1	1	16	Hyeonchung	1996
7122	0	1	1	17	Mitchellkrueger	1994
7123	0	1	1	18	Waynemontgomery	1995
7124	0	1	1	19	Maximilianmarterer	1995
7125	0	1	1	20	Bornacoric	1996
7126	0	1	1	21	Thai-Sonkwiatkowski	1995
7127	0	1	1	22	Spencerpapa	1995
7128	0	1	1	23	Juan Ignaciogalarza	1994
7129	0	1	1	24	Mackenziemcdonald	1995
7130	0	1	1	25	Jordanthompson	1994
7131	0	1	1	26	Danielmasur	1994
7132	0	1	1	27	Maxde vroome	1994
7133	0	0	1	28	Yoshihitonishioka	1995
7134	0	0	1	29	Joshuaward-hibbert	1994
7135	0	0	1	30	Matteodonati	1995
7136	0	0	1	31	Gabrielfriedrich	1994
7137	0	0	1	32	Hugodi feo	1995
7138	0	0	1	33	Kyleedmund	1995
7139	0	0	1	34	Lukebambridge	1995
7140	0	0	1	35	Pedjakrstin	1994
7141	0	0	1	36	Mikaeltorpegaard	1994
7142	0	0	1	37	Filipbergevi	1994
7143	0	0	1	38	Mathiasbourgue	1994
7144	0	0	1	39	Braydenschnur	1995
7145	0	0	1	40	Pedrocachin	1995
7146	0	0	1	41	Danielsantos	1994

7147	0	0	1	42	Kevinkaczynski	1994
7148	0	0	1	43	Laurentlokoli	1994
7149	0	0	1	44	Maximehamou	1995
7150	0	0	1	45	Antondesyatnik	1995
7151	0	0	1	46	Juliencagnina	1994
7152	0	0	1	47	Pietrolicciardi	1994
7153	0	0	1	48	Karimhossam	1994
7154	0	0	1	49	Frankomiocic	1995
7155	0	0	1	50	Enzocouacaud	1995
7156	0	0	1	51	Temurismailov	1995
7157	0	0	1	52	Quentinhalys	1996
7158	0	0	1	53	Cameronnorrie	1995
7159	0	0	0	54	Johan Sebastientatlot	1996
7160	0	0	0	55	Ku Keonkang	1997
7161	0	0	0	56	Jorge Brianpanta	1995
7162	0	0	0	57	Stefankozlov	1998
7163	0	0	0	58	Jayandrijic	1995
7164	0	0	0	59	Herkkopollanen	1994
7165	0	0	0	60	Evanhoyt	1995
7166	0	0	0	61	Vaclavsafranek	1994
7167	0	0	0	62	Lucasgomez	1995
7168	0	0	0	63	Martinredlicki	1995
7169	0	0	0	64	Omarjasika	1997
7170	0	0	0	65	Hanneswagner	1995
7171	0	0	0	66	Jakuboravec	1995
7172	0	0	0	67	Lukasmugevicius	1994
7173	0	0	0	68	Andrewharris	1994
7174	0	0	0	69	Guillermonunez	1995
7175	0	0	0	70	Federicomaccari	1994
7176	0	0	0	71	Bradleymousley	1996
7177	0	0	0	72	Hugo Cesardojas	1994
7178	0	0	0	73	Bar Tzufbotzer	1994
7179	0	0	0	74	Alexandrefavrot	1995
7180	0	0	0	75	Cemilkel	1995
7181	0	0	0	76	Orram-harel	1995
7182	0	0	0	77	Danielkerznerman	1996
7183	0	0	0	78	Poltoledo bague	1994
7184	0	0	0	79	Lucacorinteli	1995
7185	0	0	0	80	Michaelmmoh	1998
7186	0	0	0	81	Connorfarren	1994
7187	0	0	0	82	Rickymedinilla	1995
7188	0	0	0	83	Naokinakagawa	1996
7189	0	0	0	84	Kenonishi	1996
7190	0	0	0	85	Markoskalovelonis	1994
7191	0	0	0	86	Diegopedraza	1995
7192	0	0	0	87	Treystrobel	1994
7193	0	0	0	88	Franzsydow	1995
7194	0	0	0	89	Evgenykarlovskiy	1994
7195	0	0	0	90	Thiennguyen hoang	1995
7196	0	0	0	91	Alexioshalebian	1994

7197	0	0	0	92	Chi-Shanjao	1995
7198	0	0	0	93	Jareddonaldson	1996
7199	0	0	0	94	Hassanndayishimiye	1994
7200	0	0	0	95	Simeonrossier	1995
7201	0	0	0	96	Arjunkadhe	1994
7202	0	0	0	97	Thiagomonteiro	1994
7203	0	0	0	98	Mateusbrisot silva	1994
7204	0	0	0	99	Enzopy	1995
7205	0	0	0	100	Patrikfabian	1994
7206	0	0	0	101	Guilhermehadlich	1994
7207	0	0	0	102	Alannunez aguilera	1995
7208	0	0	0	103	Tomhill	1995
7209	0	0	0	104	Bernardocasares	1994
7210	0	0	0	105	Joshuapage	1994
7211	1	1	1	1	Jirivesely	1993
7212	1	1	1	2	Lukesaville	1994
7213	1	1	1	3	Olivergolding	1993
7214	1	1	1	4	Dominicthiem	1993
7215	1	1	1	5	Bjornfratangelo	1993
7216	1	1	1	6	Hugodellien	1993
7217	1	1	1	7	Thiagomonteiro	1994
7218	1	1	1	8	Filiphoransky	1993
7219	1	1	1	9	Liambroady	1994
7220	1	1	1	10	Patrickofner	1993
7221	0	1	1	11	Robinkern	1993
7222	0	1	1	12	Kaichiuchida	1994
7223	0	1	1	13	Mitchellkrueger	1994
7224	0	1	1	14	Joao Pedrosorgi	1993
7225	0	1	1	15	Matiassborowitz	1993
7226	0	1	1	16	Andrewwhittington	1993
7227	0	1	1	17	Nikolamilojevic	1995
7228	0	1	1	18	Georgemorgan	1993
7229	0	1	1	19	Juliencagnina	1994
7230	0	1	1	20	Brunosant'anna	1993
7231	0	1	1	21	Kyleedmund	1995
7232	0	1	1	22	Diegohidalgo	1993
7233	0	1	1	23	Maximdubarenco	1993
7234	0	1	1	24	Marcosgiron	1993
7235	0	0	1	25	Adampavlasek	1994
7236	0	0	1	26	Kimmercoppejans	1994
7237	0	0	1	27	Xingao	1994
7238	0	0	1	28	Nickkyrgios	1995
7239	0	0	1	29	Matepavic	1993
7240	0	0	1	30	Karimhossam	1994
7241	0	0	1	31	Yaraslavshyla	1993
7242	0	0	1	32	Enzocouacaud	1995
7243	0	0	1	33	Jesonpatrombon	1993
7244	0	0	1	34	Filippeliwo	1994
7245	0	0	1	35	Andresartunedo martinavarro	1993
7246	0	0	1	36	Macstyslinger	1993

7247	0	0	1	37	Pedjakrstin	1994
7248	0	0	1	38	Vladyslavmanafov	1993
7249	0	0	1	39	Fredericoferreira silva	1995
7250	0	0	1	40	Benjaminugarte	1993
7251	0	0	1	41	Gonzalolama	1993
7252	0	0	1	42	Marco Aurelionunez	1993
7253	0	0	1	43	Julianlenz	1993
7254	0	0	1	44	Aslankaratsev	1993
7255	0	0	1	45	Lucaspouille	1994
7256	0	0	1	46	Matedelic	1993
7257	0	0	1	47	Waynemontgomery	1995
7258	0	0	1	48	Mikijankovic	1994
7259	0	0	0	49	Jorisde loore	1993
7260	0	0	0	50	Hassanndayishimiye	1994
7261	0	0	0	51	Gianluigiquinzi	1996
7262	0	0	0	52	Joshuaward-hibbert	1994
7263	0	0	0	53	Connorfarren	1994
7264	0	0	0	54	Kevinkaczynski	1994
7265	0	0	0	55	Patrikfabian	1994
7266	0	0	0	56	Evgenykarlovskiy	1994
7267	0	0	0	57	Laurentlokoli	1994
7268	0	0	0	58	Seanberman	1993
7269	0	0	0	59	Dennisnovak	1993
7270	0	0	0	60	Jadengrinter	1993
7271	0	0	0	61	Oriolroca batalla	1993
7272	0	0	0	62	Marekjaloviec	1994
7273	0	0	0	63	Alexioshalebian	1994
7274	0	0	0	64	Benjaminlock	1993
7275	0	0	0	65	Dennisnovikov	1993
7276	0	0	0	66	Nikrazborsek	1993
7277	0	0	0	67	Gabriel Vicentinipereira	1993
7278	0	0	0	68	Axelalvarez llamas	1993
7279	0	0	0	69	Shanevinsant	1993
7280	0	0	0	70	Danielsantos	1994
7281	0	0	0	71	Rishabhdevrajshekar raman	1993
7282	0	0	0	72	Lukebambridge	1995
7283	0	0	0	73	Jorge Brianpanta	1995
7284	0	0	0	74	Benwagland	1993
7285	0	0	0	75	Jae Hwankim	1994
7286	0	0	0	76	Dantegennaro	1993
7287	0	0	0	77	Stefanlindmark	1993
7288	0	0	0	78	Bernardocasares	1994
7289	0	0	0	79	Thiennguyen hoang	1995
7290	0	0	0	80	Samhutt	1994
7291	0	0	0	81	Danielskripnik	1993
7292	0	0	0	82	Emmett Aegger	1993
7293	0	0	0	83	Lukasvrnak	1993
7294	0	0	0	84	Karuesell	1993
7295	0	0	0	85	Jonathancornish	1994
7296	0	0	0	86	Juan Pablomurra	1993

7297	0	0	0	87	Evanhoyt	1995
7298	0	0	0	88	Franciscozambon	1993
7299	0	0	0	89	Luispatino	1993
7300	0	0	0	90	Rodrigoencinas	1994
7301	0	0	0	91	Treystrobel	1994
7302	0	0	0	92	Richarddel nunzio	1993
7303	0	0	0	93	Jenshauser	1993
7304	0	0	0	94	Kazumakawachi	1994
7305	0	0	0	95	Diogorocha	1994
7306	0	0	0	96	Vitorgalvao	1993
7307	1	1	1	1	Juan Sebastiangomez	1992
7308	1	1	1	2	Martonfucsovics	1992
7309	1	1	1	3	Jirivesely	1993
7310	1	1	1	4	Damirdzumhur	1992
7311	1	1	1	5	Dominicthiem	1993
7312	1	1	1	6	Tiagofernandes	1993
7313	1	1	1	7	Robertoquiroz	1992
7314	1	1	1	8	Duilioberetta	1992
7315	1	1	1	9	Agustinvelotti	1992
7316	1	1	1	10	Matepavic	1993
7317	0	1	1	11	Jamesduckworth	1992
7318	0	1	1	12	Mikhailbiryukov	1992
7319	0	1	1	13	Renzoolivo	1992
7320	0	1	1	14	Georgemorgan	1993
7321	0	1	1	15	Olivergolding	1993
7322	0	1	1	16	Matezsiga	1992
7323	0	1	1	17	Bowenouyang	1992
7324	0	1	1	18	Victorbaluda	1992
7325	0	1	1	19	Yasutakauchiyama	1992
7326	0	1	1	20	Deniskudla	1992
7327	0	1	1	21	Alessandrocolella	1992
7328	0	1	1	22	Jannicklupescu	1993
7329	0	1	1	23	Benwagland	1993
7330	0	1	1	24	Filiphoransky	1993
7331	0	0	1	25	Hugodellien	1993
7332	0	0	1	26	Jozefkovalik	1992
7333	0	0	1	27	Jesonpatrombon	1993
7334	0	0	1	28	Mathiasbourgue	1994
7335	0	0	1	29	Ricardorodriguez	1993
7336	0	0	1	30	Andrewwhittington	1993
7337	0	0	1	31	Tarodaniel	1993
7338	0	0	1	32	Peterheller	1992
7339	0	0	1	33	Shanevinsant	1993
7340	0	0	1	34	Justineleveld	1992
7341	0	0	1	35	Matthiaswunner	1993
7342	0	0	1	36	Chuhanwang	1992
7343	0	0	1	37	Guilhermeclezar	1992
7344	0	0	1	38	Dennisnovikov	1993
7345	0	0	1	39	Johnmorrissey	1992
7346	0	0	1	40	Federicocoria	1992

7347	0	0	1	41	Alexanderrumyantsev	1992
7348	0	0	1	42	Danewebb	1992
7349	0	0	1	43	Sambarry	1992
7350	0	0	1	44	Ashleyhewitt	1992
7351	0	0	1	45	Diegogaleano	1992
7352	0	0	1	46	Giammarcomicolani	1992
7353	0	0	1	47	Nickchappell	1992
7354	0	0	1	48	Luispatino	1993
7355	0	0	0	49	Tobiasblomgren	1992
7356	0	0	0	50	Karuesell	1993
7357	0	0	0	51	Mitchellkrueger	1994
7358	0	0	0	52	Vitorgalvao	1993
7359	0	0	0	53	Francis Caseyalcantara	1992
7360	0	0	0	54	Robinkern	1993
7361	0	0	0	55	Kevinkrawietz	1992
7362	0	0	0	56	Raymondsarmiento	1992
7363	0	0	0	57	Liang-Chihuang	1992
7364	0	0	0	58	Facundomena	1992
7365	0	0	0	59	Benmclachlan	1992
7366	0	0	0	60	Patrickelias	1993
7367	0	0	0	61	Gregoirebarrere	1994
7368	0	0	0	62	Romainarneodo	1992
7369	0	0	0	63	Xingao	1994
7370	0	0	0	64	Sai Kartiknakireddi	1993
7371	0	0	0	65	Mitchellfrank	1992
7372	0	0	0	66	Ahmedtriki	1992
7373	0	0	0	67	Alexiscarlos	1992
7374	0	0	0	68	Jeamoonlee	1993
7375	0	0	0	69	Diegoacosta	1992
7376	0	0	0	70	Brunosemenzato	1992
7377	0	0	0	71	Marco Aurelionunez	1993
7378	0	0	0	72	Brunosant'anna	1993
7379	0	0	0	73	Robertcarter	1993
7380	0	0	0	74	Matiassborowitz	1993
7381	0	0	0	75	Sebastianlopez	1992
7382	0	0	0	76	Evansong	1992
7383	0	0	0	77	Pavelkrainik	1992
7384	0	0	0	78	Micklescure	1993
7385	0	0	0	79	Maciejsmola	1993
7386	0	0	0	80	Mickekontinen	1992
7387	0	0	0	81	Jadengrinter	1993
7388	0	0	0	82	Michaelzhu	1992
7389	0	0	0	83	Marcocecchinato	1992
7390	0	0	0	84	Benjaminugarte	1993
7391	0	0	0	85	Darianking	1992
7392	0	0	0	86	Gabrielflores ruiz	1992
7393	0	0	0	87	Nikolaihaessig	1992
7394	0	0	0	88	Feliperios	1992
7395	0	0	0	89	Bartoszsawicki	1992
7396	0	0	0	90	Jackcarpenter	1992

7397	0	0	0	91	Tomallen	1992
7398	0	0	0	92	Sebastianlavie	1992
7399	0	0	0	93	Cristianchavez	1992
7400	0	0	0	94	Cedrickcommin	1993
7401	0	0	0	95	Ivanconejero	1993
7402	1	1	1	1	Danielberta	1992
7403	1	1	1	2	Giannimina	1992
7404	1	1	1	3	Agustinvelotti	1992
7405	1	1	1	4	Jasonkubler	1993
7406	1	1	1	5	Julienobry	1991
7407	1	1	1	6	Mitchellfrank	1992
7408	1	1	1	7	Liang-Chihuang	1992
7409	1	1	1	8	Andreacollarini	1992
7410	1	1	1	9	Pierre-Huguesherbert	1991
7411	0	1	1	10	Andreykuznetsov	1991
7412	0	1	1	11	Facundoarguello	1992
7413	0	1	1	12	Kevinkrawietz	1992
7414	0	1	1	13	Cheng-Penghsieh	1991
7415	0	1	1	14	Deniskudla	1992
7416	0	1	1	15	Davidsouto	1992
7417	0	1	1	16	Dominikschulz	1992
7418	0	1	1	17	Arthurde greef	1992
7419	0	1	1	18	Guilhermeclezar	1992
7420	0	1	1	19	Duilioberetta	1992
7421	0	1	1	20	Yasutakauchiyama	1992
7422	0	1	1	21	Federicogaio	1992
7423	0	1	1	22	Hiroyasuehara	1991
7424	0	1	1	23	Filiphoransky	1993
7425	0	0	1	24	Tennyssandgren	1991
7426	0	0	1	25	Shuichisekiguchi	1991
7427	0	0	1	26	Henrilaaksonen	1992
7428	0	0	1	27	Yannikreuter	1991
7429	0	0	1	28	Tiagofernandes	1993
7430	0	0	1	29	Richardbecker	1991
7431	0	0	1	30	Josepereira	1991
7432	0	0	1	31	Kevinkonfederak	1991
7433	0	0	1	32	Evanking	1992
7434	0	0	1	33	Dinomarcan	1991
7435	0	0	1	34	Jozefkovalik	1992
7436	0	0	1	35	Israel Alexanderore	1992
7437	0	0	1	36	Renzoolivo	1992
7438	0	0	1	37	Julenuriguen	1991
7439	0	0	1	38	Stanislavpoplavskyy	1991
7440	0	0	1	39	Sebastianlavie	1992
7441	0	0	1	40	Alexandros-Ferdinandosgeorgo	1991
7442	0	0	1	41	Nicolaasscholtz	1991
7443	0	0	1	42	Sandroehrat	1991
7444	0	0	1	43	Christianlindell	1991
7445	0	0	1	44	Mickekontinen	1992
7446	0	0	1	45	Johnmorrissey	1992

7447	0	0	0	46	Francis Caseyalcantara	1992
7448	0	0	0	47	Patrikbrydolf	1991
7449	0	0	0	48	Harryfowler	1991
7450	0	0	0	49	Jordancox	1992
7451	0	0	0	50	Mikhailbiryukov	1992
7452	0	0	0	51	Jackcarpenter	1992
7453	0	0	0	52	Olivergolding	1993
7454	0	0	0	53	Alessandrobega	1991
7455	0	0	0	54	Tristan-Samuelweissborn	1991
7456	0	0	0	55	Benmclachlan	1992
7457	0	0	0	56	Maximilianneuchrist	1991
7458	0	0	0	57	Matteocivarolo	1992
7459	0	0	0	58	Adrienpuget	1991
7460	0	0	0	59	Johannes Robertvan overbeek	1992
7461	0	0	0	60	Rikimclachlan	1991
7462	0	0	0	61	Karim-Mohamedmaamoun	1991
7463	0	0	0	62	Emanuelemolina	1991
7464	0	0	0	63	Laurent-Olivierdaxhelet	1991
7465	0	0	0	64	Sudanwasitaram	1991
7466	0	0	0	65	Dominikwirlend	1991
7467	0	0	0	66	Ahmedel menshawy	1991
7468	0	0	0	67	Carlosboluda-purkiss	1993
7469	0	0	0	68	Gaetande lovinfosse	1991
7470	0	0	0	69	Marindraganja	1991
7471	0	0	0	70	Zacharywhite	1991
7472	0	0	0	71	Francescopicco	1991
7473	0	0	0	72	Alfredomoreno	1991
7474	0	0	0	73	Pavelkrainik	1992
7475	0	0	0	74	Andreivasilevski	1991
7476	0	0	0	75	Radimurbanek	1991
7477	0	0	0	76	Emiliogomez	1991
7478	0	0	0	77	Mikelislibietis	1992
7479	0	0	0	78	Vinayak Sharmakaza	1991
7480	0	0	0	79	Matthieuvierin	1991
7481	0	0	0	80	Pablocarreno busta	1991
7482	0	0	0	81	Jamesmarsalek	1992
7483	0	0	0	82	Facundomena	1992
7484	0	0	0	83	Tomfarquharson	1992
7485	0	0	0	84	Gabrieldias	1991
7486	0	0	0	85	Eduardodischinger	1992
7487	0	0	0	86	Brunosemenzato	1992
7488	0	0	0	87	Rodneycarey	1992
7489	0	0	0	88	Davidsofaer	1991
7490	0	0	0	89	Germansanchez delfin	1991
7491	0	0	0	90	Jose Gerardomeza paniagua	1991
7492	1	1	1	1	Tsung-Huayang	1991
7493	1	1	1	2	Yukibhambri	1992
7494	1	1	1	3	Bernardtomic	1992
7495	1	1	1	4	Henrikontinen	1990
7496	1	1	1	5	Cedrik-Marcelstebe	1990

7497	1	1	1	6	Guillaumerufin	1990
7498	1	1	1	7	Cesarramirez	1990
7499	1	1	1	8	Josepereira	1991
7500	1	1	1	9	Davidgoffin	1990
7501	1	1	1	10	Mirzabasic	1991
7502	0	1	1	11	Juanvazquez-valenzuela	1991
7503	0	1	1	12	Marceloarevalo	1990
7504	0	1	1	13	Filipkrajinovic	1992
7505	0	1	1	14	Chasebuchanan	1991
7506	0	1	1	15	Marcuswillis	1990
7507	0	1	1	16	Henriquecunha	1990
7508	0	1	1	17	Christopherrungkat	1990
7509	0	1	1	18	Bradleyklahn	1990
7510	0	1	1	19	Marindraganja	1991
7511	0	1	1	20	Alexandrefolie	1990
7512	0	1	1	21	Nikolausmoser	1990
7513	0	1	1	22	Hirokimoriya	1990
7514	0	1	1	23	Alexeigrigorov	1990
7515	0	1	1	24	Jarmerejenkins	1990
7516	0	1	1	25	Borutpuc	1991
7517	0	0	1	26	Danielsmethurst	1990
7518	0	0	1	27	Kittiphongwachiramanowong	1990
7519	0	0	1	28	Liang-Chihuang	1992
7520	0	0	1	29	Devinbritton	1991
7521	0	0	1	30	Adrienpuget	1991
7522	0	0	1	31	Andrewthomas	1990
7523	0	0	1	32	Hiroyasuehara	1991
7524	0	0	1	33	Peerakitsiributwong	1990
7525	0	0	1	34	Alessandrogiannessi	1990
7526	0	0	1	35	Dinomarcan	1991
7527	0	0	1	36	Danepropoggia	1990
7528	0	0	1	37	Cheng-Penghsieh	1991
7529	0	0	1	38	Blazrola	1990
7530	0	0	1	39	Jaredeaston	1990
7531	0	0	1	40	Tennyssandgren	1991
7532	0	0	1	41	Danielcox	1990
7533	0	0	1	42	Diwu	1991
7534	0	0	1	43	Juan-Carlosspir	1990
7535	0	0	1	44	Josehernandez-fernandez	1990
7536	0	0	1	45	Tristan-Samuelweissborn	1991
7537	0	0	1	46	Alexanderlobkov	1990
7538	0	0	1	47	Ryanharrison	1992
7539	0	0	1	48	Harryfowler	1991
7540	0	0	1	49	Milosraonic	1990
7541	0	0	0	50	Cristobalsaavedra-corvalan	1990
7542	0	0	0	51	Takanyigaranganga	1990
7543	0	0	0	52	Julenuriguen	1991
7544	0	0	0	53	Tytrombetta	1990
7545	0	0	0	54	Julienobry	1991
7546	0	0	0	55	Evanking	1992

7547	0	0	0	56	Giacomomiccini	1992
7548	0	0	0	57	Jaan-Frederikbrunken	1990
7549	0	0	0	58	Ashleywatling	1990
7550	0	0	0	59	Michaelthompson	1990
7551	0	0	0	60	Timvan terheijden	1990
7552	0	0	0	61	Mikhailbiryukov	1992
7553	0	0	0	62	Ignaciomartire	1990
7554	0	0	0	63	Neilpauffley	1990
7555	0	0	0	64	Yannikreuter	1991
7556	0	0	0	65	Alexsanders	1991
7557	0	0	0	66	Axelmichon	1990
7558	0	0	0	67	James Boseal	1991
7559	0	0	0	68	Alexllompart	1990
7560	0	0	0	69	Sudanwasitaram	1991
7561	0	0	0	70	Danielevans	1990
7562	0	0	0	71	Pierre-Huguesherbert	1991
7563	0	0	0	72	Grantive	1991
7564	0	0	0	73	Jordancox	1992
7565	0	0	0	74	Christopherdiaz-figueroa	1990
7566	0	0	0	75	Johannes Robertvan overbeek	1992
7567	0	0	0	76	Giladben zvi	1990
7568	0	0	0	77	Vasekpospisil	1990
7569	0	0	0	78	Vladzimirkruk	1991
7570	0	0	0	79	Tomislavjotovski	1990
7571	0	0	0	80	Niallangus	1990
7572	0	0	0	81	Mattreid	1990
7573	0	0	0	82	Davidsouto	1992
7574	0	0	0	83	Kelseystevenson	1990
7575	0	0	0	84	Mauricioastorga	1991
7576	0	0	0	85	Jose Gerardomeza paniagua	1991
7577	0	0	0	86	Alastairbarnes	1991
7578	0	0	0	87	Lorenzopapasidero	1990
7579	0	0	0	88	Markverryth	1991
7580	0	0	0	89	Marinmilan	1990
7581	0	0	0	90	Maximilianneuchrist	1991
7582	0	0	0	91	Thomasknights	1990
7583	0	0	0	92	Erikcrepaldi	1990
7584	0	0	0	93	Matthewshort	1990
7585	0	0	0	94	Tadayukilonghi	1990
7586	0	0	0	95	Andrevidaller	1991
7587	0	0	0	96	Andresbucaro	1990
7588	0	0	0	97	Samgarforth-bles	1990
7589	1	1	1	1	Ricardasberankis	1990
7590	1	1	1	2	Uladzimirignatik	1990
7591	1	1	1	3	Matteotrevisan	1989
7592	1	1	1	4	Jonathaneysseric	1990
7593	1	1	1	5	Fernandoromboli	1989
7594	1	1	1	6	Stephanepiro	1989
7595	1	1	1	7	Thomasfabbiano	1989
7596	1	1	1	8	Romanjebavy	1989

7597	1	1	1	9	Ricardourzua-rivera	1989
7598	1	1	1	10	John-Patricksmith	1989
7599	1	1	1	11	Henriquecunha	1990
7600	0	1	1	12	Jerzyjanowicz	1990
7601	0	1	1	13	Gastaoelias	1990
7602	0	1	1	14	Grigordimitrov	1991
7603	0	1	1	15	Guillermorivera-aranguiz	1989
7604	0	1	1	16	Stephendonald	1989
7605	0	1	1	17	Gregjones	1989
7606	0	1	1	18	Radualbot	1989
7607	0	1	1	19	Rhynewilliams	1991
7608	0	1	1	20	Danielevans	1990
7609	0	1	1	21	Cesarramirez	1990
7610	0	1	1	22	Bernardtomic	1992
7611	0	1	1	23	Andrewthomas	1990
7612	0	1	1	24	Jeromeinzerillo	1990
7613	0	1	1	25	Ryanharrison	1992
7614	0	1	1	26	Thomasschoorel	1989
7615	0	0	1	27	Jarmerejenkins	1990
7616	0	0	1	28	Davidrice	1989
7617	0	0	1	29	Hsien-Yinpeng	1989
7618	0	0	1	30	Daniel-Alejandrolopez cassaccia	1989
7619	0	0	1	31	Andreikaratchenia	1989
7620	0	0	1	32	Aljazbedene	1989
7621	0	0	1	33	Kellendamico	1989
7622	0	0	1	34	Patricioalvarado	1989
7623	0	0	1	35	Kittiphongwachiramanowong	1990
7624	0	0	1	36	Alejandrogonzalez	1989
7625	0	0	1	37	Cristobalsaavedra-corvalan	1990
7626	0	0	1	38	Fredericde fays	1989
7627	0	0	1	39	Markverryth	1991
7628	0	0	1	40	Janezsemrajc	1989
7629	0	0	1	41	Jaredeaston	1990
7630	0	0	1	42	Damianhume	1989
7631	0	0	1	43	Ilijamartinoski	1989
7632	0	0	1	44	Alexeigrigorov	1990
7633	0	0	1	45	Guillaumerufin	1990
7634	0	0	1	46	Bradleyklahn	1990
7635	0	0	1	47	Brendanmckenzie	1990
7636	0	0	1	48	Hirokimoriya	1990
7637	0	0	1	49	Christopherrungkat	1990
7638	0	0	1	50	Jirikosler	1989
7639	0	0	1	51	Evgenydonskoy	1990
7640	0	0	1	52	Danielcox	1990
7641	0	0	0	53	Lorenzopapasidero	1990
7642	0	0	0	54	Johnnyhamui	1989
7643	0	0	0	55	Dragos Cristianmirtea	1989
7644	0	0	0	56	Nikitazotov	1989
7645	0	0	0	57	Eugenbrazdil	1989
7646	0	0	0	58	Silviodadic	1990

7647	0	0	0	59	Borutpuc	1991
7648	0	0	0	60	Soong-Jaecho	1990
7649	0	0	0	61	Marceloarevalo	1990
7650	0	0	0	62	Devinbritton	1991
7651	0	0	0	63	Roybruggeling	1989
7652	0	0	0	64	Perakiatsiriluethaiwattana	1989
7653	0	0	0	65	Bence-Gyulatoth	1989
7654	0	0	0	66	Giacomomiccini	1992
7655	0	0	0	67	Axelmichon	1990
7656	0	0	0	68	Harriheliovaara	1989
7657	0	0	0	69	Rupeshroy	1989
7658	0	0	0	70	Tytrombetta	1990
7659	0	0	0	71	Xanderspong	1990
7660	0	0	0	72	Ilijavucic	1990
7661	0	0	0	73	Chasebuchanan	1991
7662	0	0	0	74	Neilpauffley	1990
7663	0	0	0	75	Austinkrajicek	1990
7664	0	0	0	76	Henrikontinen	1990
7665	0	0	0	77	Rodrigoperez	1989
7666	0	0	0	78	Alessandrogiannessi	1990
7667	0	0	0	79	Jantavcar	1990
7668	0	0	0	80	Graemedyce	1989
7669	0	0	0	81	Josehernandez-fernandez	1990
7670	0	0	0	82	Giladben zvi	1990
7671	0	0	0	83	Mateuszkecki	1990
7672	0	0	0	84	Timvan terheijden	1990
7673	0	0	0	85	Lukasomen	1990
7674	0	0	0	86	Marindraganja	1991
7675	0	0	0	87	Drewdaniel	1989
7676	0	0	0	88	Robertomaytin	1989
7677	0	0	0	89	Lazarekukhalashvili	1991
7678	0	0	0	90	Andrejmartin	1989
7679	0	0	0	91	Thomasknights	1990
7680	0	0	0	92	Vladimirzinyakov	1990
7681	0	0	0	93	Kevinbotti	1989
7682	0	0	0	94	Loforamiaramanana	1989
7683	0	0	0	95	Alin-Mihaiconstantin	1990
7684	0	0	0	96	Alexllompart	1990
7685	0	0	0	97	Goranrozic	1989
7686	0	0	0	98	Kyle-Joshuadandan	1989
7687	0	0	0	99	Nassimslilam	1990
7688	0	0	0	100	Santiagonieto	1989
7689	0	0	0	101	Mateuszszmigiel	1989
7690	0	0	0	102	Farisakhazzan	1990
7691	0	0	0	103	Adamel mihdawy	1989
7692	0	0	0	104	Yannickyoshizawa	1990
7693	1	1	1	1	Thiemode bakker	1988
7694	1	1	1	2	Nicolassantos	1988
7695	1	1	1	3	Martinklizan	1989
7696	1	1	1	4	Jonathaneysseric	1990

7697	1	1	1	5	Petru-Alexandruluncanu	1989
7698	1	1	1	6	Dusanlojda	1988
7699	1	1	1	7	Alexandresidorenko	1988
7700	1	1	1	8	Lukabelic	1988
7701	1	1	1	9	Kellendamico	1989
7702	1	1	1	10	Pavelchekhov	1988
7703	0	1	1	11	Romanjebavy	1989
7704	0	1	1	12	Arturchernov	1988
7705	0	1	1	13	Gregjones	1989
7706	0	1	1	14	Pedrosousa	1988
7707	0	1	1	15	Albertramos-vinolas	1988
7708	0	1	1	16	Rubenbemelmans	1988
7709	0	1	1	17	Jaakpoldma	1988
7710	0	1	1	18	Danieldutra da silva	1988
7711	0	1	1	19	Andrejmartin	1989
7712	0	1	1	20	Bassambeidas	1988
7713	0	1	1	21	Sanamsingh	1988
7714	0	1	1	22	Jose-Robertovelasco	1989
7715	0	1	1	23	Danilaarsenov	1989
7716	0	0	1	24	Hsin-Hanlee	1988
7717	0	0	1	25	Nathanielschnugg	1988
7718	0	0	1	26	Hanspodlipnik-castillo	1988
7719	0	0	1	27	Michalkonecny	1989
7720	0	0	1	28	Marcingawron	1988
7721	0	0	1	29	Antonioveic	1988
7722	0	0	1	30	Jeevannedunchezhiyan	1988
7723	0	0	1	31	Matteotrevisan	1989
7724	0	0	1	32	Thomasfabbiano	1989
7725	0	0	1	33	Peterpolansky	1988
7726	0	0	1	34	Ricardasberankis	1990
7727	0	0	1	35	Shoaida	1988
7728	0	0	1	36	Jamiehunt	1988
7729	0	0	1	37	Ivansergeyev	1988
7730	0	0	1	38	Daniel-Alejandrolopez cassaccia	1989
7731	0	0	1	39	Gastaoelias	1990
7732	0	0	1	40	Fernandoromboli	1989
7733	0	0	1	41	Dennislajola	1989
7734	0	0	1	42	Rupeshroy	1989
7735	0	0	1	43	Peterlucassen	1988
7736	0	0	1	44	Emilianomassa	1988
7737	0	0	1	45	Robertomaytin	1989
7738	0	0	1	46	Ricardourzua-rivera	1989
7739	0	0	0	47	Harriheliovaara	1989
7740	0	0	0	48	Mateuszkecki	1990
7741	0	0	0	49	Robertobautista agut	1988
7742	0	0	0	50	Nikolamektic	1988
7743	0	0	0	51	Johnnyhamui	1989
7744	0	0	0	52	Clintbowles	1988
7745	0	0	0	53	Guillermorivera-aranguiz	1989
7746	0	0	0	54	Yanbai	1989

7747	0	0	0	55	Jean-Micheldurango	1988
7748	0	0	0	56	John-Patricksmith	1989
7749	0	0	0	57	Philipbester	1988
7750	0	0	0	58	Jurgenzopp	1988
7751	0	0	0	59	Stephendonald	1989
7752	0	0	0	60	Fumiakikita	1989
7753	0	0	0	61	Joellindner	1989
7754	0	0	0	62	Vedransiljegovic	1988
7755	0	0	0	63	Rasidwinklaar	1988
7756	0	0	0	64	Waelkilani	1988
7757	0	0	0	65	Jun-Chaoxu	1988
7758	0	0	0	66	Kevinbotti	1989
7759	0	0	0	67	Robinroshardt	1988
7760	0	0	0	68	Christianvitulli	1988
7761	0	0	0	69	Alejandrogonzalez	1989
7762	0	0	0	70	Yuichisugita	1988
7763	0	0	0	71	Parisgemouchidis	1988
7764	0	0	0	72	Brydanklein	1989
7765	0	0	0	73	Edgarrodriguez	1988
7766	0	0	0	74	Stephanepiro	1989
7767	0	0	0	75	Vladimirzinyakov	1990
7768	0	0	0	76	Graemedyce	1989
7769	0	0	0	77	Dylansessagesimi	1989
7770	0	0	0	78	Yoannre	1988
7771	0	0	0	79	Rasmusmoller	1988
7772	0	0	0	80	Ivananikanov	1988
7773	0	0	0	81	Austenchilds	1989
7774	0	0	0	82	Jameslemke	1988
7775	0	0	0	83	Drewdaniel	1989
7776	0	0	0	84	Ivanendara	1988
7777	0	0	0	85	Christopherracz	1988
7778	0	0	0	86	Joshuamilton	1989
7779	0	0	0	87	Jirikosler	1989
7780	0	0	0	88	Jeromeinzerillo	1990
7781	0	0	0	89	Yannmarti	1988
7782	0	0	0	90	Davidrice	1989
7783	0	0	0	91	Davidsimon	1988
7784	1	1	1	1	Donaldyoung	1989
7785	1	1	1	2	Marincilic	1988
7786	1	1	1	3	Ryansweeting	1987
7787	1	1	1	4	Jeremychardy	1987
7788	1	1	1	5	Leonardomayer	1987
7789	1	1	1	6	Robinhaase	1987
7790	1	1	1	7	Robinroshardt	1988
7791	1	1	1	8	Nielsdesein	1987
7792	1	1	1	9	Thiemode bakker	1988
7793	0	1	1	10	Andremiele	1987
7794	0	1	1	11	Carstenball	1987
7795	0	1	1	12	Sun-Yongkim	1987
7796	0	1	1	13	Pieroluisi	1987

7797	0	1	1	14	Raonycarvalho	1987
7798	0	1	1	15	Antalvan der duim	1987
7799	0	1	1	16	Dusanlojda	1988
7800	0	1	1	17	Samquerrey	1987
7801	0	1	1	18	Evgenykirillov	1987
7802	0	1	1	19	Petarjelenic	1987
7803	0	1	1	20	Andreashaider-maurer	1987
7804	0	1	1	21	Andreaarnaboldi	1987
7805	0	1	1	22	Philipbester	1988
7806	0	0	1	23	Jeevannedunchezhiyan	1988
7807	0	0	1	24	Kellendamico	1989
7808	0	0	1	25	Kevinbotti	1989
7809	0	0	1	26	Sergeibubka	1987
7810	0	0	1	27	Shoaida	1988
7811	0	0	1	28	Vivekshokeen	1987
7812	0	0	1	29	Jesselevine	1987
7813	0	0	1	30	Alexandersadecky	1987
7814	0	0	1	31	Davidnavarrete	1987
7815	0	0	1	32	Valeryrudnev	1988
7816	0	0	1	33	Aljoschathron	1987
7817	0	0	1	34	Emilianomassa	1988
7818	0	0	1	35	Luis-Henriquegrangeiro	1987
7819	0	0	1	36	Keinishikori	1989
7820	0	0	1	37	Michaelshabaz	1987
7821	0	0	1	38	Jameslemke	1988
7822	0	0	1	39	Jochenschoettler	1987
7823	0	0	1	40	Lukaslacko	1987
7824	0	0	1	41	Pavelchekhov	1988
7825	0	0	1	42	Parisgemouchidis	1988
7826	0	0	1	43	Holdenseguso	1988
7827	0	0	1	44	Timothyneilly	1987
7828	0	0	0	45	Jamiehunt	1988
7829	0	0	0	46	Martinsayer	1987
7830	0	0	0	47	Alexclayton	1987
7831	0	0	0	48	Romanrecarte	1987
7832	0	0	0	49	Shuheiuzawa	1988
7833	0	0	0	50	Peterpolansky	1988
7834	0	0	0	51	Kentotakeuchi	1987
7835	0	0	0	52	Abdullahmaqdes	1987
7836	0	0	0	53	Tristanfarron-mahon	1987
7837	0	0	0	54	Nathanielschnugg	1988
7838	0	0	0	55	Timsmyczek	1987
7839	0	0	0	56	Mattbruch	1987
7840	0	0	0	57	Patricknicholls	1989
7841	0	0	0	58	Dennislajola	1989
7842	0	0	0	59	Anasfattar	1987
7843	0	0	0	60	Martinkamenik	1988
7844	0	0	0	61	Dylanarnould	1988
7845	0	0	0	62	Andrewcoelho	1987
7846	0	0	0	63	Jurgensstrydom	1987

7847	0	0	0	64	Albertogonzalez	1987
7848	0	0	0	65	Alexandrdolgopolov	1988
7849	0	0	0	66	Pavolcervenak	1987
7850	0	0	0	67	Andrewkennaugh	1987
7851	0	0	0	68	Martinkillemose	1987
7852	0	0	0	69	Yannmarti	1988
7853	0	0	0	70	Leorosenberg	1989
7854	0	0	0	71	Marcusfugate	1988
7855	0	0	0	72	Johnnyhamui	1989
7856	0	0	0	73	Miguel-Angelreyes-varela	1987
7857	0	0	0	74	Bryankoniecko	1987
7858	0	0	0	75	Yuichisugita	1988
7859	0	0	0	76	Pedrograber-anguita	1988
7860	0	0	0	77	Toddley	1988
7861	0	0	0	78	Spencervegosen	1988
7862	0	0	0	79	Thomasfabbiano	1989
7863	0	0	0	80	Sergueitarasevitch	1987
7864	0	0	0	81	Yannickthomet	1988
7865	0	0	0	82	Alejandromoreno figueroa	1988
7866	0	0	0	83	Mylesblake	1987
7867	0	0	0	84	Juricagrubisic	1988
7868	0	0	0	85	Nathanrosenfeld	1987
7869	0	0	0	86	Gustavoloza	1987
7870	0	0	0	87	Jose-Carlostolentino	1987
7871	0	0	0	88	Jean-Yvesaubone	1988
7872	1	1	1	1	Gaelmonfils	1986
7873	1	1	1	2	Eduardoschwank	1986
7874	1	1	1	3	Brendanevans	1986
7875	1	1	1	4	Woong-Sunjun	1986
7876	1	1	1	5	Sun-Yongkim	1987
7877	1	1	1	6	Mischazverev	1987
7878	1	1	1	7	Pabloandujar	1986
7879	1	1	1	8	Scottoudsema	1986
7880	1	1	1	9	Lukaslacko	1987
7881	1	1	1	10	Rafaelarevalo	1986
7882	1	1	1	11	Chu Huanyi	1987
7883	0	1	1	12	Viktortroicki	1986
7884	0	1	1	13	Donaldyoung	1989
7885	0	1	1	14	Coenvan keulen	1986
7886	0	1	1	15	Fabiofognini	1987
7887	0	1	1	16	Jeremychardy	1987
7888	0	1	1	17	Guillermoalcaide	1986
7889	0	1	1	18	Igorsijsling	1987
7890	0	1	1	19	Timothyneilly	1987
7891	0	1	1	20	Sergeibubka	1987
7892	0	1	1	21	Scovillejenkins	1986
7893	0	1	1	22	Jamiebaker	1986
7894	0	1	1	23	Juan Martindel potro	1988
7895	0	1	1	24	Karanrastogi	1986
7896	0	1	1	25	Sebastianrieschick	1986

7897	0	1	1	26	Nikitakryvonos	1986
7898	0	0	1	27	Robinhaase	1987
7899	0	0	1	28	Mileskasiri	1986
7900	0	0	1	29	Timsmyczek	1987
7901	0	0	1	30	Thomazbellucci	1987
7902	0	0	1	31	Kevinanderson	1986
7903	0	0	1	32	Phillipsimmonds	1986
7904	0	0	1	33	Tomrushby	1986
7905	0	0	1	34	Nielsdesein	1987
7906	0	0	1	35	Williamward	1986
7907	0	0	1	36	Gianlucanaso	1987
7908	0	0	1	37	Davidnavarrete	1987
7909	0	0	1	38	Jamiemurray	1986
7910	0	0	1	39	GDjones	1986
7911	0	0	1	40	Juan-Pabloamado	1986
7912	0	0	1	41	Frankoskugor	1987
7913	0	0	1	42	Janmarek	1987
7914	0	0	1	43	Andreaarnaboldi	1987
7915	0	0	1	44	Marincilic	1988
7916	0	0	1	45	Remkode rijke	1986
7917	0	0	1	46	Evgenykirillov	1987
7918	0	0	1	47	Joelkerley	1986
7919	0	0	1	48	Aleksandrnedovyesov	1987
7920	0	0	1	49	Mylesblake	1987
7921	0	0	1	50	Josselinouanna	1986
7922	0	0	1	51	Erikchvojka	1986
7923	0	0	0	52	Aljoschathron	1987
7924	0	0	0	53	Sergueitarasevitch	1987
7925	0	0	0	54	Petarjelenic	1987
7926	0	0	0	55	Tusharliberhan	1986
7927	0	0	0	56	Andremiele	1987
7928	0	0	0	57	Tristanfarron-mahon	1987
7929	0	0	0	58	Michaelshabaz	1987
7930	0	0	0	59	Alexanderkrasnorutskiy	1987
7931	0	0	0	60	Mohammedsalifu	1986
7932	0	0	0	61	Gregoryouellette	1986
7933	0	0	0	62	Fariskhatib	1987
7934	0	0	0	63	Divijsharan	1986
7935	0	0	0	64	Vilimvisak	1986
7936	0	0	0	65	Petermiklusicak	1986
7937	0	0	0	66	Lachlanferguson	1986
7938	0	0	0	67	Raonycarvalho	1987
7939	0	0	0	68	Vahidmirzadeh	1986
7940	0	0	0	69	Antalvan der duim	1987
7941	0	0	0	70	Martinfischer	1986
7942	0	0	0	71	Luisdiaz-barriga	1986
7943	0	0	0	72	Navdeepsingh	1986
7944	0	0	0	73	Brunorosa	1986
7945	0	0	0	74	Pieroluisi	1987
7946	0	0	0	75	Takanobufujii	1987

7947	0	0	0	76	Mattbruch	1987
7948	0	0	0	77	Davidgalic	1986
7949	0	0	0	78	Michaeljohnson	1987
7950	0	0	0	79	Juhopaukku	1986
7951	0	0	0	80	Syrymabdukhalikov	1987
7952	0	0	0	81	Fabricemartin	1986
7953	0	0	0	82	Philipposwald	1986
7954	0	0	0	83	Chriseaton	1987
7955	0	0	0	84	Renandelsin	1986
7956	0	0	0	85	Chrischirico	1987
7957	0	0	0	86	Deniszivkovic	1987
7958	0	0	0	87	Fredericomarques	1986
7959	0	0	0	88	Danielmuller	1986
7960	0	0	0	89	Michaelsamara	1986
7961	0	0	0	90	Richardwire	1987
7962	0	0	0	91	Marcspicijaric	1986
7963	0	0	0	92	Jean-Yvesaubone	1988
7964	0	0	0	93	Robertcameron	1986
7965	0	0	0	94	Lorenzocava	1986
7966	0	0	0	95	Ryosekiguchi	1986
7967	0	0	0	96	Jose-Carlostolentino	1987
7968	0	0	0	97	Naokisato	1986
7969	0	0	0	98	Pierre-Ludovicduclos	1986
7970	0	0	0	99	Danielvallverdu	1986
7971	0	0	0	100	Vascoantunes	1986
7972	0	0	0	101	Nathanrosenfeld	1987
7973	1	1	1	1	Emersonjones	2008
7974	1	1	1	2	Ivajovic	2007
7975	1	1	1	3	Tyra Caterinagrant	2008
7976	1	1	1	4	Mikastojsavljevic	2008
7977	1	1	1	5	Jelinevandromme	2007
7978	1	1	1	6	Wakanasonobe	2008
7979	1	1	1	7	Teodorakostovic	2007
7980	1	1	1	8	Kristinapenickova	2009
7981	1	1	1	9	Terezakrejcova	2008
7982	1	1	1	10	Elizarayaneva	2007
7983	1	1	1	11	Minggexu	2007
7984	0	1	1	12	Rositsadencheva	2007
7985	0	1	1	13	Antoniavergara rivera	2006
7986	0	1	1	14	Sonjazhiyenbayeva	2006
7987	0	1	1	15	Alenakovackova	2008
7988	0	1	1	16	Ivaivanova	2006
7989	0	1	1	17	Kaitlynrolls	2007
7990	0	1	1	18	Vendulavaldmannova	2007
7991	0	1	1	19	Hannahklugman	2009
7992	0	1	1	20	Mikabuchnik	2007
7993	0	1	1	21	Monikastankiewicz	2006
7994	0	1	1	22	Mayucrossley	2006
7995	0	1	1	23	Yelyzavetakotliar	2007
7996	0	1	1	24	Vittoriapaganetti	2006

7997	0	1	1	25	Malakel allami	2006
7998	0	1	1	26	Juliepastikova	2008
7999	0	1	1	27	Eliskatichackova	2006
8000	0	0	1	28	Juliastusek	2008
8001	0	0	1	29	Theafrodin	2008
8002	0	0	1	30	Emilysartz-lunde	2006
8003	0	0	1	31	Leanilsson	2008
8004	0	0	1	32	Luna Mariacinalli	2008
8005	0	0	1	33	Mayaiyengar	2007
8006	0	0	1	34	Reinagoto	2007
8007	0	0	1	35	Noemibasiletti	2006
8008	0	0	1	36	Alanasubasic	2007
8009	0	0	1	37	Joyde zeeuw	2006
8010	0	0	1	38	Kseniaefremova	2009
8011	0	0	1	39	Laimavladson	2007
8012	0	0	1	40	Sarah Melanyfajmonova	2007
8013	0	0	1	41	Annikapenickova	2009
8014	0	0	1	42	Aspenschuman	2007
8015	0	0	1	43	Sonjazhenikhova	2008
8016	0	0	1	44	Yoanakonstantinova	2007
8017	0	0	1	45	Nadialagaev	2008
8018	0	0	1	46	Victoria Luizabarros	2009
8019	0	0	1	47	Kanonsawashiro	2008
8020	0	0	1	48	Nellietaraba wallberg	2007
8021	0	0	1	49	Miapohankova	2008
8022	0	0	1	50	Lillitagger	2008
8023	0	0	1	51	Christashamcneil	2006
8024	0	0	1	52	Shannonlam	2008
8025	0	0	1	53	Rose Marienijkamp	2006
8026	0	0	1	54	Sol Ailinlarraya guidi	2008
8027	0	0	0	55	Clairean	2007
8028	0	0	0	56	Eleejahinisan	2008
8029	0	0	0	57	Emmadong	2007
8030	0	0	0	58	Daphneempetshi perricard	2008
8031	0	0	0	59	Adakumru	2008
8032	0	0	0	60	Capucinejauffret	2008
8033	0	0	0	61	Mariagolovina	2007
8034	0	0	0	62	Margotphanthala	2007
8035	0	0	0	63	Charoesquiva banuls	2008
8036	0	0	0	64	Leenafriedman	2007
8037	0	0	0	65	Florajohnson	2007
8038	0	0	0	66	Oliviacarneiro	2006
8039	0	0	0	67	Julietapareja	2009
8040	0	0	0	68	Yuyaoli	2007
8041	0	0	0	69	Shihotsujioka	2007
8042	0	0	0	70	Yuliyaperapekhina	2008
8043	0	0	0	71	Victoriamilovanova	2007
8044	0	0	0	72	Anna Mariafedotova	2007
8045	0	0	0	73	Gaiamaduzzi	2006
8046	0	0	0	74	Lucieurbanova	2006

8047	0	0	0	75	Janahossam salah	2007
8048	0	0	0	76	Meritxellteixido garcia	2007
8049	0	0	0	77	Anitatu	2009
8050	0	0	0	78	Yiruchen	2007
8051	0	0	0	79	Monikaekstrand	2007
8052	0	0	0	80	Allegrakorpanec davies	2007
8053	0	0	0	81	Krishamahendran	2007
8054	0	0	0	82	Dunevaissaud	2007
8055	0	0	0	83	Hikariyamamoto	2009
8056	0	0	0	84	Dariaegorova	2007
8057	0	0	0	85	Amysucha	2007
8058	0	0	0	86	Laurabrunkel	2006
8059	0	0	0	87	Anastasiiafirman	2006
8060	0	0	0	88	Mariiadrobysheva	2007
8061	0	0	0	89	Marieslamenikova	2007
8062	0	0	0	90	Trinetravijayakumar	2006
8063	0	0	0	91	Isabellasvahn	2006
8064	0	0	0	92	Maayarajeshwaran revathi	2009
8065	0	0	0	93	Alyssajames	2008
8066	0	0	0	94	Junhanzhang	2007
8067	0	0	0	95	Asylzhanarystanbekova	2006
8068	0	0	0	96	Katefakih	2006
8069	0	0	0	97	Agustinagrassi	2007
8070	0	0	0	98	Eva Mariaionescu	2007
8071	0	0	0	99	Eliskaforejtkova	2008
8072	0	0	0	100	Gabiapaskauskas	2006
8073	0	0	0	101	Jinshuxia	2007
8074	0	0	0	102	Ichinohorikawa	2007
8075	0	0	0	103	Valentinasteiner	2006
8076	0	0	0	104	Miaslama	2006
8077	0	0	0	105	Gaeuljang	2006
8078	0	0	0	106	Gloriananahum	2006
8079	0	0	0	107	Mayadutta	2006
8080	0	0	0	108	Oliviacenter	2006
8081	1	1	1	1	Alinakorneeva	2007
8082	1	1	1	2	Clerviengounoue	2006
8083	1	1	1	3	Renatajamrichova	2007
8084	1	1	1	4	Laurasamson	2008
8085	1	1	1	5	Luccianaperez alarcon	2005
8086	1	1	1	6	Sarasaito	2006
8087	1	1	1	7	Kaitlinquevedo	2006
8088	1	1	1	8	Sayakaishii	2005
8089	1	1	1	9	Hannahklugman	2009
8090	1	1	1	10	Terezavalentova	2007
8091	1	1	1	11	Emersonjones	2008
8092	1	1	1	12	Alenakovackova	2008
8093	0	1	1	13	Anastasiiagureva	2005
8094	0	1	1	14	Tyra Caterinagrant	2008
8095	0	1	1	15	Enakoike	2006
8096	0	1	1	16	Charoesquiva banuls	2008

8097	0	1	1	17	Federicaurgesi	2005
8098	0	1	1	18	Minggexu	2007
8099	0	1	1	19	Teodorakostovic	2007
8100	0	1	1	20	Wakanasonobe	2008
8101	0	1	1	21	Vladamincheva	2007
8102	0	1	1	22	Mayucrossley	2006
8103	0	1	1	23	Alisaoktiabreva	2008
8104	0	1	1	24	Rebeccamunk mortensen	2005
8105	0	1	1	25	Maragae	2005
8106	0	1	1	26	Ivaivanova	2006
8107	0	1	1	27	Kristianasidorova	2006
8108	0	1	1	28	Mayajoint	2006
8109	0	1	1	29	Luna Mariacinalli	2008
8110	0	0	1	30	Elizarayaneva	2007
8111	0	0	1	31	Ranah Akuastoiber	2005
8112	0	0	1	32	Gloriananahum	2006
8113	0	0	1	33	Ayael aouni	2005
8114	0	0	1	34	Ninavargova	2005
8115	0	0	1	35	Rositsadencheva	2007
8116	0	0	1	36	Hayukinoshita	2006
8117	0	0	1	37	Mikabuchnik	2007
8118	0	0	1	38	Alessandrateodosescu	2006
8119	0	0	1	39	Monikastankiewicz	2006
8120	0	0	1	40	Vittoriapaganetti	2006
8121	0	0	1	41	Francescapace	2005
8122	0	0	1	42	Emilysartz-lunde	2006
8123	0	0	1	43	Oliviacarneiro	2006
8124	0	0	1	44	Alanishamilton	2007
8125	0	0	1	45	Alexiaharmon	2005
8126	0	0	1	46	Antoniavergara rivera	2006
8127	0	0	1	47	Mikastojsavljevic	2008
8128	0	0	1	48	Noemibasiletti	2006
8129	0	0	1	49	Zuzannapawlikowska	2005
8130	0	0	1	50	Yoanakonstantinova	2007
8131	0	0	1	51	Kristynatomajkova	2005
8132	0	0	1	52	Malakel allami	2006
8133	0	0	1	53	Ela Nalamilic	2006
8134	0	0	1	54	Ekaterinaperelygina	2006
8135	0	0	1	55	Elena Ruxandrabertea	2006
8136	0	0	1	56	Asylzhanarystanbekova	2006
8137	0	0	1	57	Miaslama	2006
8138	0	0	1	58	Arianaanazagasty-pursoo	2006
8139	0	0	0	59	Yelyzavetakotliar	2007
8140	0	0	0	60	Francescagandolfi	2006
8141	0	0	0	61	Katefakih	2006
8142	0	0	0	62	Shihotsujioka	2007
8143	0	0	0	63	Sonjazhiyenbayeva	2006
8144	0	0	0	64	Emma Ottaviaghirardato	2005
8145	0	0	0	65	Gaiamaduzzi	2006
8146	0	0	0	66	Lucianamoyano	2005

8147	0	0	0	67	Tatumevans	2005
8148	0	0	0	68	Josydaems	2006
8149	0	0	0	69	Tamarakostic	2006
8150	0	0	0	70	Malwinarowinska	2005
8151	0	0	0	71	Yujinkim	2005
8152	0	0	0	72	Roisingilheany	2005
8153	0	0	0	73	Gabiapaskauskas	2006
8154	0	0	0	74	Melisaercan	2005
8155	0	0	0	75	Liamoved	2005
8156	0	0	0	76	Darjasuvirdjonkova	2005
8157	0	0	0	77	Cara Mariamester	2005
8158	0	0	0	78	Theadorarabman	2005
8159	0	0	0	79	Lilytaylor	2006
8160	0	0	0	80	Gretagreco lucchina	2005
8161	0	0	0	81	Hephzibaholuwadare	2007
8162	0	0	0	82	Margotphanthala	2007
8163	0	0	0	83	Oliviacenter	2006
8164	0	0	0	84	Valeriaray	2005
8165	0	0	0	85	Mayadutta	2006
8166	0	0	0	86	Anastasiiafirman	2006
8167	0	0	0	87	Christashamcneil	2006
8168	0	0	0	88	Daphneempetshi perricard	2008
8169	0	0	0	89	Nikoladaubnerova	2005
8170	0	0	0	90	Anna Linnpuls	2006
8171	0	0	0	91	Shannonlam	2008
8172	0	0	0	92	Laurabrunkel	2006
8173	0	0	0	93	Sol Ailinlarraya guidi	2008
8174	0	0	0	94	Eva Mariaionescu	2007
8175	0	0	0	95	Ellamcdonald	2005
8176	0	0	0	96	Krishamahendran	2007
8177	0	0	0	97	Anyamurthy	2006
8178	0	0	0	98	Naonishino	2005
8179	0	0	0	99	Yichenzhao	2006
8180	0	0	0	100	Ashtonbowers	2005
8181	0	0	0	101	Naomixu	2006
8182	0	0	0	102	Taylorgoetz	2005
8183	0	0	0	103	Capucinejauffret	2008
8184	0	0	0	104	Anastasiiagrechkina	2005
8185	0	0	0	105	Imogenhaddad	2005
8186	0	0	0	106	Aiyanupbay	2006
8187	0	0	0	107	Ameliesmejkalova	2005
8188	0	0	0	108	Carmen Andreeaherea	2005
8189	0	0	0	109	Onyuchoi	2006
8190	0	0	0	110	Eleejahinisan	2008
8191	0	0	0	111	Elliedaniels	2006
8192	0	0	0	112	Gaiagreco	2006
8193	0	0	0	113	Gabriellamikaul	2006
8194	0	0	0	114	Jessicabernales	2006
8195	0	0	0	115	Ekaterinaivanova	2006
8196	0	0	0	116	Martinamarica	2005

8197	1	1	1	1	Luciehavlickova	2005
8198	1	1	1	2	Sofiacostoulas	2005
8199	1	1	1	3	Livhovde	2005
8200	1	1	1	4	Celinenaef	2005
8201	1	1	1	5	Sarasaito	2006
8202	1	1	1	6	Victoriamboko	2006
8203	1	1	1	7	Terezavalentova	2007
8204	1	1	1	8	Clerviengounoue	2006
8205	1	1	1	9	Ellamcdonald	2005
8206	1	1	1	10	Nikoladaubnerova	2005
8207	0	1	1	11	Taylahpreston	2005
8208	0	1	1	12	Sayakaishii	2005
8209	0	1	1	13	Lucianamoyano	2005
8210	0	1	1	14	Ninavargova	2005
8211	0	1	1	15	Lucaudvardy	2005
8212	0	1	1	16	Ellaseidel	2005
8213	0	1	1	17	Luccianaperez alarcon	2005
8214	0	1	1	18	Kaitlinquevedo	2006
8215	0	1	1	19	Ranah Akuastoiber	2005
8216	0	1	1	20	Minggexu	2007
8217	0	1	1	21	Qavialopez	2005
8218	0	1	1	22	Anastasiyalopata	2004
8219	0	1	1	23	Renatajamrichova	2007
8220	0	1	1	24	Yarabartashevich	2005
8221	0	1	1	25	Annabellexu	2004
8222	0	0	1	26	Luciapeyre	2005
8223	0	0	1	27	Kaylacross	2005
8224	0	0	1	28	Hayukinoshita	2006
8225	0	0	1	29	Miakupres	2004
8226	0	0	1	30	Johanne Christinesvendsen	2004
8227	0	0	1	31	Ivajovic	2007
8228	0	0	1	32	Ameliesmejkalova	2005
8229	0	0	1	33	Tatumevans	2005
8230	0	0	1	34	Yu-Yunli	2004
8231	0	0	1	35	Arianaanazagasty-pursoo	2006
8232	0	0	1	36	Carolinakuhl	2005
8233	0	0	1	37	Miaslama	2006
8234	0	0	1	38	Madeleinejessup	2005
8235	0	0	1	39	Ela Nalamilic	2006
8236	0	0	1	40	Irinabalus	2005
8237	0	0	1	41	Dominikasalkova	2004
8238	0	0	1	42	Theadorarabman	2005
8239	0	0	1	43	Joelle Lilly Sophiesteur	2004
8240	0	0	1	44	Alexisblokhina	2004
8241	0	0	1	45	Hannevandewinkel	2004
8242	0	0	1	46	Naomixu	2006
8243	0	0	1	47	Darjasuvirdjonkova	2005
8244	0	0	1	48	Raquelgonzalez vilar	2004
8245	0	0	1	49	Charlottekempenaers-pocz	2004
8246	0	0	1	50	Alevtinaibragimova	2005

8247	0	0	0	51	Weronikaewald	2006
8248	0	0	0	52	Juliestruplova	2004
8249	0	0	0	53	Melisaercan	2005
8250	0	0	0	54	Avakrug	2005
8251	0	0	0	55	Ahmaniguichard	2005
8252	0	0	0	56	Valeriaray	2005
8253	0	0	0	57	Shrutiahlawat	2006
8254	0	0	0	58	Malwinarowinska	2005
8255	0	0	0	59	Kristynatomajkova	2005
8256	0	0	0	60	Anyamurthy	2006
8257	0	0	0	61	Hephzibaholuwadare	2007
8258	0	0	0	62	Malakel allami	2006
8259	0	0	0	63	Isabellelacy	2006
8260	0	0	0	64	Aruzhansagandikova	2004
8261	0	0	0	65	Giorgiapedone	2004
8262	0	0	0	66	Ameliawaligora	2005
8263	0	0	0	67	Sonyamacavei	2004
8264	0	0	0	68	Angellaokutoyi	2004
8265	0	0	0	69	Gabriellabroadfoot	2004
8266	0	0	0	70	Sandugashkenzhibayeva	2005
8267	0	0	0	71	Anastasiiagureva	2005
8268	0	0	0	72	Elliedaniels	2006
8269	0	0	0	73	Aysegulmert	2004
8270	0	0	0	74	Yichenzhao	2006
8271	0	0	0	75	Rebeccamunk mortensen	2005
8272	0	0	0	76	Zaralarke	2005
8273	0	0	0	77	Barboramichalkova	2006
8274	0	0	0	78	Isis Louisevan den broek	2005
8275	0	0	0	79	Ekaterinakhayrutdinova	2004
8276	0	0	0	80	Annaparadisi	2005
8277	0	0	0	81	Amelievan impe	2004
8278	0	0	0	82	Lucijaciric bagaric	2004
8279	0	0	0	83	Michaelalaki	2005
8280	0	0	0	84	Chelseafontenel	2004
8281	0	0	0	85	Denislavaglushkova	2004
8282	0	0	0	86	Imogenhaddad	2005
8283	0	0	0	87	Meshkatolzahrasafi	2004
8284	0	0	0	88	Danabaidaulet	2005
8285	0	0	0	89	Gloriananahum	2006
8286	0	0	0	90	Ayael aouni	2005
8287	0	0	0	91	Tijanasretenovic	2005
8288	0	0	0	92	Natachaschou	2004
8289	0	0	0	93	Sageloudon	2005
8290	0	0	0	94	Zhanelrustemova	2004
8291	0	0	0	95	Anacandiotto	2004
8292	0	0	0	96	Emilysartz-lunde	2006
8293	0	0	0	97	Martinamarica	2005
8294	0	0	0	98	Denisevalente	2004
8295	0	0	0	99	Patricijapaukstyte	2004
8296	0	0	0	100	Shannonlam	2008

8297	1	1	1	1	Petramarcinko	2005
8298	1	1	1	2	Lindafruhvirtova	2005
8299	1	1	1	3	Dianashnaider	2004
8300	1	1	1	4	Brendafruhvirtova	2007
8301	1	1	1	5	Victoriajimenez kasintseva	2005
8302	1	1	1	6	Kristinadmitruk	2003
8303	1	1	1	7	Kseniazaytseva	2004
8304	1	1	1	8	Robinmontgomery	2004
8305	1	1	1	9	Anemintegi del olmo	2003
8306	1	1	1	10	Alexandraeala	2005
8307	1	1	1	11	Solanasierra	2004
8308	1	1	1	12	Clerviengounoue	2006
8309	1	1	1	13	Michaelalaki	2005
8310	1	1	1	14	Mirraandreeva	2007
8311	1	1	1	15	Lindanoskova	2004
8312	1	1	1	16	Nataliaszabanin	2003
8313	1	1	1	17	Johanne Christinesvendsen	2004
8314	1	1	1	18	Nikolabartunkova	2006
8315	1	1	1	19	Maraguth	2003
8316	1	1	1	20	Sofiacostoulas	2005
8317	1	1	1	21	Celinenaef	2005
8318	1	1	1	22	Lindaklimovicova	2004
8319	1	1	1	23	Yarabartashevich	2005
8320	1	1	1	24	Madisonsieg	2003
8321	1	1	1	25	Lucijaciric bagaric	2004
8322	1	1	1	26	Barborapalicova	2004
8323	1	1	1	27	Oceanebabel	2004
8324	1	1	1	28	Nastasjaschunk	2003
8325	1	1	1	29	Hannevandewinkel	2004
8326	1	1	1	30	Livhovde	2005
8327	1	1	1	31	Juliamiddendorf	2003
8328	1	1	1	32	Victoriamboko	2006
8329	1	1	1	33	Polinaiatcenko	2003
8330	1	1	1	34	Laurahietaranta	2004
8331	1	1	1	35	Reesebrantmeier	2004
8332	1	1	1	36	Elvinakalieva	2003
8333	1	1	1	37	Elenapridankina	2005
8334	1	1	1	38	Anastasiiagureva	2005
8335	1	1	1	39	Kristynatomajkova	2005
8336	1	1	1	40	Marinastakusic	2004
8337	1	1	1	41	Alinashcherbinina	2003
8338	1	1	1	42	Tilwithdi girolami	2003
8339	1	1	1	43	Sebastiannascilipoti	2003
8340	1	1	1	44	Qavialopez	2005
8341	1	1	1	45	Kaylacross	2005
8342	1	1	1	46	Biancabehulova	2003
8343	1	1	1	47	Alexisblokhina	2004
8344	1	1	1	48	Luciehavlickova	2005
8345	1	1	1	49	Annabellexu	2004
8346	1	1	1	50	Ashlynkrueger	2004

8347	1	1	1	51	Chelseafontenel	2004
8348	1	1	1	52	Dominikasalkova	2004
8349	1	1	1	53	Joelle Lilly Sophiesteur	2004
8350	1	1	1	54	Ekaterinakhayrutdinova	2004
8351	1	1	1	55	Denislavaglushkova	2004
8352	1	1	1	56	Juliagarcia	2003
8353	1	1	1	57	Radkazelnickova	2003
8354	1	1	1	58	Sarabejlek	2006
8355	1	1	1	59	Ranah Akuastoiber	2005
8356	1	1	1	60	Carolinakuhl	2005
8357	1	1	1	61	Amelievan impe	2004
8358	1	1	1	62	Miakupres	2004
8359	1	1	1	63	Tijanasretenovic	2005
8360	1	1	1	64	Erikaandreeva	2004
8361	1	1	1	65	Laiapetretic	2004
8362	1	1	1	66	Evialinalaskevich	2004
8363	1	1	1	67	Oksanaselekhmeteva	2003
8364	1	1	1	68	Sarasaito	2006
8365	1	1	1	69	Alexandrayepifanova	2003
8366	1	1	1	70	Mariasholokhova	2004
8367	1	1	1	71	Nicolerivkin	2003
8368	1	1	1	72	Lolaradivojevic	2005
8369	1	1	1	73	Tatianaprozorova	2003
8370	1	1	1	74	Aysegulmert	2004
8371	1	1	1	75	Luccianaperez alarcon	2005
8372	1	1	1	76	Irinabalus	2005
8373	1	1	1	77	Ancatodoni	2004
8374	1	1	1	78	Ayael aouni	2005
8375	1	1	1	79	Angellaokutoyi	2004
8376	1	1	1	80	Alinagranwehr	2003
8377	1	1	1	81	Ellaseidel	2005
8378	1	1	1	82	Aruzhansagandikova	2004
8379	1	1	1	83	Valenciaxu	2003
8380	1	1	1	84	Raquelgonzalez vilar	2004
8381	1	1	1	85	Ameliesmejkalova	2005
8382	1	1	1	86	Krystalblanch	2004
8383	1	1	1	87	Ekaterinamaklakova	2004
8384	1	1	1	88	Patricijapaukstyte	2004
8385	1	1	1	89	Annazyryanova	2004
8386	1	1	1	90	Kirapavlova	2004
8387	1	1	1	91	Lucianamoyano	2005
8388	1	1	1	92	Amarissa Kiaratoth	2003
8389	1	1	1	93	Terezavalentova	2007
8390	1	1	1	94	Elizabethcoleman	2003
8391	1	1	1	95	Ninavargova	2005
8392	1	1	1	96	Ioanazvonaru	2004
8393	1	1	1	97	Elizavetagavrilova	2003
8394	1	1	1	98	Katjawiersholm	2004
8395	1	1	1	99	Nikoladaubnerova	2005
8396	1	1	1	100	Melisaercan	2005

8397	1	1	1	101	Giorgiapedone	2004
8398	1	1	1	102	Polinakudermetova	2003
8399	1	1	1	103	Matildamutavdzic	2004
8400	1	1	1	104	Shrutiahlawat	2006
8401	1	1	1	105	Gracieepps	2004
8402	1	1	1	106	Alevtinaibragimova	2005
8403	1	1	1	107	Juliestruplova	2004
8404	1	1	1	108	Luciapeyre	2005
8405	1	1	1	109	Maomushika	2005
8406	1	1	1	110	Miaslama	2006
8407	1	1	1	111	Virginiaferrara	2004
8408	1	1	1	112	Meshkatolzahrasafi	2004
8409	1	1	1	113	Pimradajattavapornvanit	2003
8410	1	1	1	114	Leyla Fiorellabritez risso	2004
8411	1	1	1	115	Avakrug	2005
8412	1	1	1	116	Ameliawaligora	2005
8413	1	1	1	117	Lucaudvardy	2005
8414	1	1	1	118	Sabinazeynalova	2004
8415	1	1	1	119	Ozlemuslu	2004
8416	1	1	1	120	Olivialincer	2004
8417	1	1	1	121	Kaitlinquevedo	2006
8418	1	1	1	122	Laura Isabelputz	2003
8419	1	1	1	123	Vanessa Ioana Georgianapopa t	2004
8420	1	1	1	124	Natachaschou	2004
8421	1	1	1	125	Alejandracruz	2003
8422	1	1	1	126	Meihasegawa	2003
8423	1	1	1	127	Reececarter	2004
8424	1	1	1	128	Nahiaberecoechea	2004
8425	1	1	1	129	Rebeccamunk mortensen	2005
8426	1	1	1	130	Anastasiiagrechkina	2005
8427	1	1	1	131	Sayakaishii	2005
8428	1	1	1	132	Sonyamacavei	2004
8429	1	1	1	133	Feryelben hassen	2004
8430	1	1	1	134	Anacandiotto	2004
8431	1	1	1	135	Tatyananikolenko	2004
8432	1	1	1	136	Katerynalazarenko	2004
8433	1	1	1	137	Larasmejkal	2004
8434	1	1	1	138	Katerinadimitrova	2004
8435	1	1	1	139	Sandugashkenzhibayeva	2005
8436	1	1	1	140	Celinesimunyu	2004
8437	1	1	1	141	Miaristic	2006
8438	1	1	1	142	Malwinarowinska	2005
8439	1	1	1	143	Julianamunhoz	2003
8440	1	1	1	144	Dashaplekhanova	2004
8441	1	1	1	145	Madeleinejessup	2005
8442	1	1	1	146	Dariashadchneva	2006
8443	1	1	1	147	Hayukinoshita	2006
8444	1	1	1	148	Denisevalente	2004
8445	1	1	1	149	Heikejanse van vuuren	2004
8446	1	1	1	150	Danabaidaulet	2005

8447	1	1	1	151	Tealukic	2004
8448	1	1	1	152	Anastasiyalopata	2004
8449	1	1	1	153	Karolinakozakova	2005
8450	1	1	1	154	Marianamanyoma velasquez	2003
8451	1	1	1	155	Alinakhvatova	2004
8452	1	1	1	156	Elisabethjones	2004
8453	1	1	1	157	Klaudijabubelyte	2003
8454	1	1	1	158	Yu-Yunli	2004
8455	1	1	1	159	Tamarakostic	2006
8456	1	1	1	160	Taylahpreston	2005
8457	1	1	1	161	Annaparadisi	2005
8458	1	1	1	162	Tatumevans	2005
8459	1	1	1	163	Dimitrapavlou	2004
8460	1	1	1	164	Matildepaoletti	2003
8461	1	1	1	165	Maria Oliviacastedo	2003
8462	1	1	1	166	Aliciadudeney	2003
8463	1	1	1	167	Adapiestrzynska	2004
8464	1	1	1	168	Zuzannakubacha	2004
8465	1	1	1	169	Juliettebovy	2004
8466	1	1	1	170	Sarasvetac	2004
8467	1	1	1	171	Kyliebilchev	2003
8468	1	1	1	172	Jerminesherif	2005
8469	1	1	1	173	Isis Louisevan den broek	2005
8470	1	1	1	174	Yichenzhao	2006
8471	1	1	1	175	Carolinagomez	2004
8472	1	1	1	176	Nevenacarton	2003
8473	1	1	1	177	Dariayesypchuk	2005
8474	1	1	1	178	Iuliiaiudenko	2004
8475	1	1	1	179	Suhithamaruri	2005
8476	1	1	1	180	Sarasuchankova	2003
8477	1	1	1	181	Victoriamatasova	2004
8478	1	1	1	182	Darjavidmanova	2003
8479	1	1	1	183	Elisewagle	2003
8480	1	1	1	184	Emilijatverijonaite	2004
8481	1	1	1	185	Maria Fernandamartinez herna	2004
8482	1	1	1	186	Tania Isabelandrade sabando	2005
8483	1	1	1	187	Marievogt	2005
8484	1	1	1	188	Alexiajacobs	2005
8485	1	1	1	189	Zhanelrustemova	2004
8486	1	1	1	190	Ashtonbowers	2005
8487	1	1	1	191	Martynaostrzygalo	2005
8488	1	1	1	192	Antoniaruzic	2003
8489	1	1	1	193	Liamoved	2005
8490	1	1	1	194	Xeniabandurowska	2004
8491	1	1	1	195	Sonjazhiyenbayeva	2006
8492	1	1	1	196	Lanlanatararudee	2004
8493	1	1	1	197	Magdalenaswierczynska	2004
8494	1	1	1	198	Rozaliagruszczynska	2004
8495	1	1	1	199	Talianeilson-gatenby	2005
8496	1	1	1	200	Mariiahlahola	2004

8497	1	1	1	201	Minggexu	2007
8498	1	1	1	202	Evashaw	2005
8499	1	1	1	203	Cara Mariamester	2005
8500	1	1	1	204	Martelambrecht	2004
8501	1	1	1	205	Luciallinares domingo	2003
8502	1	1	1	206	Mariasantos	2004
8503	1	1	1	207	Annakhaykis	2004
8504	1	1	1	208	Enakoike	2006
8505	1	1	1	209	Erikamatsuda	2003
8506	1	1	1	210	Mathildengijol carre	2007
8507	1	1	1	211	Liel Marliesrothensteiner	2005
8508	1	1	1	212	Divnaratkovic	2004
8509	1	1	1	213	Daniellaben-abraham	2004
8510	1	1	1	214	Pannabartha	2004
8511	1	1	1	215	Sasha Nataliechimedza	2007
8512	1	1	1	216	Lisapigato	2003
8513	1	1	1	217	Federicaurgesi	2005
8514	1	1	1	218	Yelyzavetakotliar	2007
8515	1	1	1	219	Malakel allami	2006
8516	1	1	1	220	Paulinafranco martinessi	2004
8517	1	1	1	221	Ellamcdonald	2005
8518	1	1	1	222	Alexiaharmon	2005
8519	1	1	1	223	Martasoriano santiago	2006
8520	1	1	1	224	Janakolodynska	2003
8521	1	1	1	225	Mariyasinitsyna	2003
8522	1	1	1	226	Arianaanazagasty-pursoo	2006
8523	1	1	1	227	Anitasahdiieva	2004
8524	1	1	1	228	Leighvan zyl	2004
8525	1	1	1	229	Anaelleleclercq	2003
8526	1	1	1	230	Naomixu	2006
8527	1	1	1	231	Lucieurbanova	2006
8528	1	1	1	232	Ahmaniguichard	2005
8529	1	1	1	233	Kristynahranacova	2004
8530	1	1	1	234	Erkezhanarystanbekova	2004
8531	1	1	1	235	Jovanagrujic	2006
8532	1	1	1	236	Stefaniabojica	2005
8533	1	1	1	237	Suzaniepretorius	2004
8534	1	1	1	238	Rose Marienijkamp	2006
8535	1	1	1	239	Emmavalletta	2004
8536	1	1	1	240	Josydaems	2006
8537	1	1	1	241	Weronikaewald	2006
8538	1	1	1	242	Daiannehayashida	2003
8539	1	1	1	243	Alexandrashubladze	2005
8540	1	1	1	244	Emmacharney	2003
8541	1	1	1	245	Bertapassola	2004
8542	1	1	1	246	Maeliemonfils	2005
8543	1	1	1	247	Annasedysheva	2005
8544	1	1	1	248	Clarinelerby	2003
8545	1	1	1	249	Isabellelacy	2006
8546	1	1	1	250	Roisingilheany	2005

8547	1	1	1	251	Andreaobradovic	2004
8548	1	1	1	252	Yoanakonstantinova	2007
8549	1	1	1	253	Zuzannakolonus	2005
8550	1	1	1	254	Scarlettnicholson	2006
8551	1	1	1	255	Carolinaxavier laydner	2005
8552	1	1	1	256	Kimmihance	2003
8553	1	1	1	257	Sophiellewellyn	2006
8554	1	1	1	258	Maria Sarapopa	2005
8555	1	1	1	259	Annasemenova	2004
8556	1	1	1	260	Charlottekempenaers-pocz	2004
8557	1	1	1	261	Danacharintceva	2005
8558	1	1	1	262	Salakthipounmuang	2005
8559	1	1	1	263	Claudiade las heras armenteras	2003
8560	1	1	1	264	Rufaromagarira	2003
8561	1	1	1	265	Charlotteowensby	2003
8562	1	1	1	266	Elizabethtindera	2003
8563	1	1	1	267	Dariakhomutsianskaya	2004
8564	1	1	1	268	Noamajor	2005
8565	1	1	1	269	Sarahhamner	2003
8566	1	1	1	270	Kristianasidorova	2006
8567	1	1	1	271	Melisasenli	2004
8568	1	1	1	272	Vladamincheva	2007
8569	1	1	1	273	Junebjork	2005
8570	1	1	1	274	Riabhakta	2005
8571	1	1	1	275	Leyla Niluferelmas	2005
8572	1	1	1	276	Nomanoha akugue	2003
8573	1	1	1	277	Maryloveedwards	2005
8574	1	1	1	278	Mariabondarenko	2003
8575	1	1	1	279	Polinapavlova	2003
8576	1	1	1	280	Noraknezevic	2005
8577	1	1	1	281	Mialiepert	2004
8578	1	1	1	282	Sarahiliev	2006
8579	1	1	1	283	Lilytaylor	2006
8580	1	1	1	284	Melodiecollard	2003
8581	1	1	1	285	Catherineaulia	2004
8582	1	1	1	286	Anastasiaabbagnato	2003
8583	1	1	1	287	Vladislavaandreevskaya	2005
8584	1	1	1	288	Salmadrugdova	2004
8585	1	1	1	289	Gabriellabroadfoot	2004
8586	1	1	1	290	Gabrielacortes flores	2004
8587	1	1	1	291	Sara Valentinacruz bonilla	2003
8588	1	1	1	292	Cadencebrace	2005
8589	1	1	1	293	Alinakorneeva	2007
8590	1	1	1	294	Elliedaniels	2006
8591	1	1	1	295	Elizavetamorozova	2004
8592	1	1	1	296	Umabakaityte	2004
8593	1	1	1	297	Renatajamrichova	2007
8594	1	1	1	298	Laylashoukry	2006
8595	1	1	1	299	Valeriaray	2005
8596	1	1	1	300	Arinavalitova	2004

8597	1	1	1	301	Carmen Andreeaherea	2005
8598	1	1	1	302	Klaramilicevic	2003
8599	1	1	1	303	Martinamarica	2005
8600	1	1	1	304	Reshmamaruri	2003
8601	1	1	1	305	Francescapace	2005
8602	1	1	1	306	Maragae	2005
8603	1	1	1	307	Elaplatenikova	2004
8604	1	1	1	308	Maria Carolinaferreira turchett	2004
8605	1	1	1	309	Taylorgoetz	2005
8606	1	1	1	310	Brianaszabo	2005
8607	1	1	1	311	Ana Carmenzamburek	2003
8608	1	1	1	312	Sofiianagornaia	2003
8609	1	1	1	313	Vaishnaviadkar	2004
8610	1	1	1	314	Vandavargova	2003
8611	0	1	1	315	Yasminanigametzyanova	2005
8612	0	1	1	316	Elizavetamasnaia	2005
8613	0	1	1	317	Leabathellier	2005
8614	0	1	1	318	Nastasijanesterovic	2007
8615	0	1	1	319	Miomushika	2005
8616	0	1	1	320	Sarasarinova	2005
8617	0	1	1	321	Klio Mariaioannou	2005
8618	0	1	1	322	Zaralarke	2005
8619	0	1	1	323	Karinaltori	2006
8620	0	1	1	324	Florajohnson	2007
8621	0	1	1	325	Martinapavissich	2006
8622	0	1	1	326	Alessandracaceres	2004
8623	0	1	1	327	Chiaradi genova	2004
8624	0	1	1	328	Sanjanasirimalla	2004
8625	0	1	1	329	Emabubalo	2004
8626	0	1	1	330	Aninalanz	2005
8627	0	1	1	331	Emilijapranyte	2005
8628	0	1	1	332	Elisabethiila	2004
8629	0	1	1	333	Karsynevans	2005
8630	0	1	1	334	Masha Alexandravrsalovic gime	2004
8631	0	1	1	335	Melissaboyden	2003
8632	0	1	1	336	Isabellachhiv	2005
8633	0	1	1	337	Flaviebrugnone	2003
8634	0	1	1	338	Annelinbakker	2005
8635	0	1	1	339	Aleksandragabrovska	2006
8636	0	1	1	340	Katarinapavlechova	2005
8637	0	1	1	341	Mayaiyengar	2007
8638	0	1	1	342	Tiantsoa Sarahrakotomanga raj	2005
8639	0	1	1	343	Lauracilekova	2005
8640	0	1	1	344	Priska Madelynnugroho	2003
8641	0	1	1	345	Alionafalei	2004
8642	0	1	1	346	Sue Yantan	2006
8643	0	1	1	347	Fiorelladuran montano	2004
8644	0	1	1	348	Marianareding	2005
8645	0	1	1	349	Flohelsen	2005
8646	0	1	1	350	Rumagaikaiwari	2005

8647	0	1	1	351	Luca Victoriavocke	2004
8648	0	1	1	352	Sarapantelic	2005
8649	0	1	1	353	Valerygynina	2005
8650	0	1	1	354	Liisavarul	2004
8651	0	1	1	355	Vivianyang	2005
8652	0	1	1	356	Evaelbaz	2004
8653	0	1	1	357	Wardaait el bachir	2004
8654	0	1	1	358	Stellaremander	2005
8655	0	1	1	359	Inesbekrar	2003
8656	0	1	1	360	Astridlew yan foon	2005
8657	0	1	1	361	Glorianananahum	2006
8658	0	1	1	362	Rebeccalynn	2004
8659	0	1	1	363	Kseniaagureeva	2004
8660	0	1	1	364	Lilyfairclough	2005
8661	0	1	1	365	Marystoiana	2003
8662	0	1	1	366	Sarahtatu	2004
8663	0	1	1	367	Marentesijbesma	2004
8664	0	1	1	368	Dariaegorova	2007
8665	0	1	1	369	Danaguzman	2003
8666	0	1	1	370	Carlottamoccia	2003
8667	0	1	1	371	Carlapacot	2005
8668	0	1	1	372	Janastojanova	2005
8669	0	1	1	373	Sarahmueller	2004
8670	0	1	1	374	Loesebeling koning	2006
8671	0	1	1	375	Asylzhanarystanbekova	2006
8672	0	1	1	376	Manalennaciri	2005
8673	0	1	1	377	Matildemorais	2004
8674	0	1	1	378	Theadorarabman	2005
8675	0	1	1	379	Honokaumeda	2004
8676	0	1	1	380	Mariagarcia cid	2005
8677	0	1	1	381	Vilmakrebs hyllested	2005
8678	0	1	1	382	Pia Marijarebec	2004
8679	0	1	1	383	Nataliafantini	2006
8680	0	1	1	384	Ekuayouri	2005
8681	0	1	1	385	Hannah Kaileshen	2004
8682	0	1	1	386	Elsajacquemot	2003
8683	0	1	1	387	Hibaheni	2006
8684	0	1	1	388	Catalinapadilla udaeta	2005
8685	0	1	1	389	Evabusaad	2005
8686	0	1	1	390	Carlagalmiche	2004
8687	0	1	1	391	Ana Fabiaholweg bustillos	2004
8688	0	1	1	392	Luanaavelar	2003
8689	0	1	1	393	Sashasitue	2006
8690	0	1	1	394	Durusoke	2006
8691	0	1	1	395	Gretegull	2006
8692	0	1	1	396	Katrinpavkova	2005
8693	0	1	1	397	Fatima Ingrid Amarthakeita	2003
8694	0	1	1	398	Victoriamilovanova	2007
8695	0	1	1	399	Arinabulatova	2006
8696	0	1	1	400	Deborahdominguez collado	2005

8697	0	1	1	401	Valeriacarreno	2004
8698	0	1	1	402	Oliviacarneiro	2006
8699	0	1	1	403	Aminazeghlouli	2007
8700	0	1	1	404	Paulacembranos	2004
8701	0	1	1	405	Sarahvan emst	2004
8702	0	1	1	406	Tizianarossini	2004
8703	0	1	1	407	Vichitrapornvimuktananda	2005
8704	0	1	1	408	Lucienguyen tan	2003
8705	0	1	1	409	Tanyaradzwamidzi	2006
8706	0	1	1	410	Anjanayar	2005
8707	0	1	1	411	Maybaranes	2005
8708	0	1	1	412	Elena Ruxandrabertea	2006
8709	0	1	1	413	Alessiamartinez	2004
8710	0	1	1	414	Ela Nalamilic	2006
8711	0	1	1	415	Nataliatrigosso	2006
8712	0	1	1	416	Kiramatushkina	2006
8713	0	1	1	417	Brinasulin	2004
8714	0	1	1	419	Alexandrazimmer	2006
8715	0	1	1	420	Magdalenasmekalova	2006
8716	0	1	1	421	Elizavetashebekina	2003
8717	0	1	1	422	Alianzack	2006
8718	0	1	1	423	Sageloudon	2005
8719	0	1	1	424	Violetamartinez	2004
8720	0	1	1	425	Deannechoo	2003
8721	0	1	1	426	Antoniavergara rivera	2006
8722	0	1	1	427	Emilysartz-lunde	2006
8723	0	1	1	428	Margaritatoshkova	2004
8724	0	1	1	429	Arunkumarlakshmi prabha	2005
8725	0	1	1	430	Maria Paulavargas triana	2005
8726	0	1	1	431	Yeonwooku	2003
8727	0	1	1	432	Claracwajgenbaum	2005
8728	0	1	1	433	Aiyanupbay	2006
8729	0	1	1	434	Isseypurser	2004
8730	0	1	1	435	Barboramichalkova	2006
8731	0	1	1	436	Tianyudong	2005
8732	0	1	1	437	Palomagoldsmith weinreich	2004
8733	0	1	1	438	Stefaniwebb	2005
8734	0	1	1	439	Alexandraazarko	2006
8735	0	1	1	440	Joyde zeeuw	2006
8736	0	1	1	441	Miachudejova	2003
8737	0	1	1	442	Shihotsujioka	2007
8738	0	1	1	443	Maria Luisaoliveira	2005
8739	0	1	1	444	Anagrubor	2004
8740	0	1	1	445	Emmarizzetto	2004
8741	0	1	1	446	Sarahl'allier	2005
8742	0	1	1	447	Gretagreco lucchina	2005
8743	0	1	1	448	Eleonoraalvisi	2003
8744	0	1	1	449	Tessa Johannabrockmann	2005
8745	0	1	1	450	Rositsadencheva	2007
8746	0	1	1	451	Gabrielarondon	2005

8747	0	1	1	452	Matildejorge	2004
8748	0	1	1	453	Olgamishenina	2003
8749	0	1	1	454	Romanelongueville	2005
8750	0	1	1	455	Mandegarfarzami	2006
8751	0	1	1	456	Danielarivera	2004
8752	0	1	1	457	Saraakid	2003
8753	0	1	1	458	Aleksandrasimeva	2004
8754	0	1	1	459	Serafimashastova	2004
8755	0	1	1	460	Nataliavela zangroniz	2004
8756	0	1	1	461	Darjasuvirdjonkova	2005
8757	0	1	1	462	Kittimolnar	2006
8758	0	1	1	463	Mayadrozd	2004
8759	0	1	1	464	Milenajevtovic	2004
8760	0	1	1	465	Sophia Natalyabaranov	2004
8761	0	1	1	466	Vittoriapaganetti	2006
8762	0	1	1	467	Elizabetebrutane	2007
8763	0	1	1	468	Aubreynisbet	2005
8764	0	1	1	469	Ellieblackford	2006
8765	0	1	1	470	Anastasiabozova	2005
8766	0	1	1	471	Petjadrame	2004
8767	0	1	1	472	Laurakoralnik	2004
8768	0	1	1	473	Nataliaperez	2006
8769	0	1	1	474	Sonalpatil	2005
8770	0	1	1	475	Oliviabergler	2005
8771	0	1	1	476	Lourdesayala	2006
8772	0	1	1	477	Emmatothova	2005
8773	0	1	1	478	Bridgetstammel	2003
8774	0	1	1	479	Micaelaponce serrano	2003
8775	0	1	1	480	Valeska Alexandrasan martin ra	2005
8776	0	1	1	481	Kristiboro	2004
8777	0	1	1	482	Karinarizvanova	2003
8778	0	1	1	483	Leenametwally	2005
8779	0	1	1	484	Luciepawlak	2006
8780	0	1	1	485	Mayucrossley	2006
8781	0	1	1	486	Rutujachaphalkar	2004
8782	0	1	1	487	Ranimrassil	2006
8783	0	1	1	488	Annakantor	2004
8784	0	1	1	489	Fatima Yareligutierrez martinez	2004
8785	0	1	1	490	Larastojanovski	2005
8786	0	1	1	491	Sofiacohen perovani	2006
8787	0	1	1	492	Laviniamorreale	2005
8788	0	1	1	493	Gaeuljang	2006
8789	0	1	1	494	Bianca Joliefernandez	2004
8790	0	1	1	495	Elizarayaneva	2007
8791	0	1	1	496	Sophieschouten	2004
8792	0	1	1	497	Anyamurthy	2006
8793	0	1	1	498	Marinamarkina	2006
8794	0	1	1	499	Miasaveljic	2005
8795	0	1	1	500	Ecegencer	2006
8796	0	1	1	501	Mariiamasiianskaia	2007

8797	0	1	1	502	Elizabethtkachenko	2004
8798	0	1	1	503	Maddyzampardo	2004
8799	0	1	1	504	Bojanapozder	2003
8800	0	1	1	505	Suanatucakovic	2004
8801	0	1	1	506	Viktoriaveleva	2004
8802	0	1	1	507	Madhurimasawant	2005
8803	0	1	1	508	Elinazakharova	2003
8804	0	1	1	509	Zuzannafrankowska	2004
8805	0	1	1	510	Juliastusek	2008
8806	0	1	1	511	Darigarustemova	2006
8807	0	1	1	512	Alessiacau	2005
8808	0	1	1	513	Valerija Maijakargina	2006
8809	0	1	1	514	Kaatcoppez	2005
8810	0	1	1	515	Yevagaliievska	2007
8811	0	1	1	516	Lidiiarasskovskaia	2004
8812	0	1	1	517	Valeriiakrokhotina	2005
8813	0	1	1	518	Yilinyan	2005
8814	0	1	1	519	Arinavaraksina	2007
8815	0	1	1	520	Athinapitta	2005
8816	0	1	1	521	Janahossam salah	2007
8817	0	1	1	522	Kaitlynrolls	2007
8818	0	1	1	523	Tomokokato	2003
8819	0	1	1	524	Carolinaalonso	2007
8820	0	1	1	525	Maria Sofiamadrid rocca	2004
8821	0	1	1	526	Dianade simone	2005
8822	0	1	1	527	Jo-Leensaw	2007
8823	0	1	1	528	Gyeong Seolee	2005
8824	0	1	1	529	Tapaneeboonyawat	2005
8825	0	1	1	530	Ingkardyussebay	2007
8826	0	1	1	531	Emmapenne	2004
8827	0	1	1	532	Elenilazaridou	2004
8828	0	1	1	533	Rinkomatsuda	2005
8829	0	1	1	534	Karinajumazhanova	2006
8830	0	1	1	535	Marianazegada	2003
8831	0	1	1	536	Na Hyunkang	2004
8832	0	1	1	537	Polinaskopintseva	2005
8833	0	1	1	538	Sophiewilliams	2004
8834	0	1	1	539	Anastasiyazholdakova	2004
8835	0	1	1	540	Amirabargaoui	2004
8836	0	1	1	541	Mayabetzalel	2004
8837	0	1	1	542	Violettaborodina	2004
8838	0	1	1	543	Martinamarkov	2004
8839	0	1	1	544	Tamaryaffe	2004
8840	0	1	1	545	Isabellabenavides jaramillo	2005
8841	0	1	1	546	Mikabuchnik	2007
8842	0	1	1	547	Vivianovrootsky	2004
8843	0	1	1	548	Dariagorska	2005
8844	0	1	1	549	Tadiwanashemauchi	2006
8845	0	1	1	550	Violaturini	2005
8846	0	1	1	551	Dunjamaric	2006

8847	0	1	1	552	Stadfanynformi	2004
8848	0	1	1	553	Klaravukicevic	2003
8849	0	1	1	554	Mariacastano	2006
8850	0	1	1	555	Mernarefaat	2004
8851	0	1	1	556	Dorsacheraghi	2005
8852	0	1	1	557	Kristinapaskauskas	2004
8853	0	1	1	558	Masaviriant	2005
8854	0	1	1	559	Meerajesudason	2005
8855	0	1	1	560	Melis Aydauyar	2003
8856	0	1	1	561	Anna Marieweissheim	2004
8857	0	1	1	562	Carolanndelaunay	2004
8858	0	1	1	563	Athenarosas-pacifico	2006
8859	0	1	1	564	Victoriaosuigwe	2006
8860	0	1	1	565	Sofia Cristinataborga rollano	2003
8861	0	1	1	566	Jacquelinesoloveychik	2004
8862	0	1	1	567	Noemibasiletti	2006
8863	0	1	1	568	Mariapazospina	2006
8864	0	1	1	569	Ann Akashaceuca	2006
8865	0	1	1	570	Yujinkim	2005
8866	0	1	1	571	Annavashchenko	2006
8867	0	1	1	572	Alicebattesti	2005
8868	0	1	1	573	Naledimanyube	2004
8869	0	1	1	574	Ximenamorales araneda	2004
8870	0	1	1	575	Valeriiaivanovskaia	2004
8871	0	1	1	576	Esthervyrlan	2006
8872	0	1	1	577	Priscilajanikian	2003
8873	0	1	1	578	Shaniceroignot	2007
8874	0	1	1	579	Darinakamenoff	2005
8875	0	1	1	580	Valentinaponce serrano	2006
8876	0	1	1	581	Zuzannapawlikowska	2005
8877	0	1	1	582	Dariiamonakhova	2003
8878	0	1	1	583	Petramihokova	2004
8879	0	1	1	584	Mafaldaguedes	2004
8880	0	1	1	585	Morganpyrz	2004
8881	0	1	1	586	Valeriiasimakova	2006
8882	0	1	1	587	Sarahrokusek	2006
8883	0	1	1	588	Moranemeyers	2005
8884	0	1	1	589	Andreade bernardo	2006
8885	0	1	1	590	Jelenavico	2006
8886	0	1	1	591	Vanesa Dalmadanko	2005
8887	0	1	1	592	Zoedu pasquier jensen	2005
8888	0	1	1	593	Leontinastojanovic	2005
8889	0	1	1	594	Amelie Clairemontalvo lundy	2004
8890	0	1	1	595	Emmakamper	2004
8891	0	1	1	596	Jadeotway	2003
8892	0	1	1	597	Patricia Georgianagoina	2005
8893	0	1	1	598	Mayadutta	2006
8894	0	1	1	599	Laurarahnel	2006
8895	0	1	1	600	Ginafeistel	2003
8896	0	1	1	601	Elenamicic	2004

8897	0	1	1	602	Riyauboveja	2003
8898	0	1	1	603	Orlyogilvy	2005
8899	0	1	1	604	Ariannazucchini	2003
8900	0	1	1	605	Dianakhaydarshina	2005
8901	0	1	1	606	Anastasiasafta	2004
8902	0	1	1	607	Aminasalibayeva	2006
8903	0	1	1	608	Jordinacegarra ramirez	2004
8904	0	1	1	609	Xintongzhou	2005
8905	0	1	1	610	Analanisek	2005
8906	0	1	1	611	Fatmaidrizovic	2004
8907	0	1	1	612	Vivianmiller	2004
8908	0	1	1	613	Likaperesypkina	2005
8909	0	1	1	614	Miatsoukalas	2004
8910	0	1	1	615	Carmenjuan bru	2005
8911	0	1	1	616	Gaiamaduzzi	2006
8912	0	1	1	617	Diana Mariailie	2006
8913	0	1	1	618	Hannahchambers	2004
8914	0	1	1	619	Olgamolchanova	2004
8915	0	1	1	620	Nikitavishwase	2004
8916	0	1	1	621	Gabriella Vannessalindgren	2006
8917	0	1	1	622	Cleohutchinson	2007
8918	0	1	1	623	Katherinehui	2005
8919	0	1	1	624	Ekaterinaperelygina	2006
8920	0	1	1	625	Anna Linnpuls	2006
8921	0	1	1	626	Nicolealfaro	2005
8922	0	1	1	627	Jennylim	2005
8923	0	1	1	628	Kennedygibbs	2004
8924	0	1	1	629	Kamonwanyodpetch	2007
8925	0	1	1	630	Varvaraaltukhova	2006
8926	0	1	1	631	Inesfaltinger	2003
8927	0	1	1	632	Miotisoarasendra andriananter	2006
8928	0	1	1	633	Stefanyashalakhova	2004
8929	0	1	1	634	Victoriasasinka	2004
8930	0	1	1	635	Carlamarkus	2006
8931	0	1	1	636	Asemshoybek	2005
8932	0	1	1	637	Kristinavolgapkina	2004
8933	0	1	1	638	Pimmadathongkum	2006
8934	0	1	1	639	Aidaoviedo	2006
8935	0	1	1	640	Zaradarken	2007
8936	0	1	1	641	Didenazpolat	2004
8937	0	1	1	642	Jadesolacole	2006
8938	0	1	1	643	Tyralithiby	2006
8939	0	1	1	644	Salmafarhat	2004
8940	0	1	1	645	Laviniatanasie	2003
8941	0	1	1	646	Sthefanyrodrigues de lima	2006
8942	0	1	1	647	Laurasamson	2008
8943	0	1	1	648	Sofiiapaladi	2005
8944	0	1	1	649	Dharaniniroshan	2004
8945	0	1	1	650	Mariamibrahim	2005
8946	0	1	1	651	Abbykelliher	2007

8947	0	1	1	652	Isabellekouzmanov	2004
8948	0	1	1	653	Elenakarner	2003
8949	0	1	1	654	Antoniasarria	2005
8950	0	1	1	655	Carolina Isabelchiatti reynoso	2004
8951	0	1	1	656	Roseseccia	2004
8952	0	1	1	657	Soo Hajang	2004
8953	0	1	1	658	Mayajoint	2006
8954	0	1	1	659	Melissazeh	2004
8955	0	1	1	660	Shahzodaartikbaeva	2008
8956	0	1	1	661	Saramilanese	2004
8957	0	1	1	662	Anastasiiafirman	2006
8958	0	1	1	663	Charlottekeitel	2005
8959	0	1	1	664	Abhilashabista	2003
8960	0	1	1	665	Nicolinesartz-lunde	2004
8961	0	1	1	666	Edanumanoglu	2005
8962	0	1	1	667	Addison Yangcomiskey	2007
8963	0	1	1	668	Umayrahashimova	2003
8964	0	1	1	669	Patriciagrigoras	2006
8965	0	1	1	670	Tildejagare	2004
8966	0	1	1	671	Isabellarivera ortiz	2004
8967	0	1	1	672	Marilouisevan zyl	2006
8968	0	1	1	673	Ariannaparadas	2006
8969	0	1	1	674	Salonitamang	2004
8970	0	1	1	675	Victoriabervid	2008
8971	0	1	1	676	Beatrisezeltina	2007
8972	0	1	1	677	Hephzibaholuwadare	2007
8973	0	1	1	678	Monanarita	2006
8974	0	1	1	679	Elysetse	2004
8975	0	1	1	680	Giulialesa	2006
8976	0	1	1	681	Andrea Camilarobles cuellar	2006
8977	0	1	1	682	Sapircohen	2007
8978	0	1	1	683	Seinmyoung	2005
8979	0	1	1	684	Katemansfield	2004
8980	0	1	1	685	Germanydavis	2005
8981	0	1	1	686	Taylawilmot	2005
8982	0	1	1	687	Vasilissakupriyanova	2006
8983	0	1	1	688	Sashamotlagh	2005
8984	0	1	1	689	Bohoussou Blanche Lili-bellemi	2005
8985	0	1	1	690	Leahkuruvilla	2004
8986	0	1	1	691	Ceciliacosta	2006
8987	0	1	1	692	Mille Johannamoerk	2006
8988	0	1	1	693	Yunokitahara	2006
8989	0	1	1	694	Lyafernandez	2007
8990	0	1	1	695	Juliafliegner	2003
8991	0	1	1	696	Evawoydt	2005
8992	0	1	1	697	Michelleyeo	2005
8993	0	1	1	698	Thamarafrasser kawaratani	2005
8994	0	1	1	699	Jadepsonka	2005
8995	0	1	1	700	Rebekakern	2003
8996	0	1	1	701	Joodyelkady	2006

8997	0	1	1	702	Johannahiesmair	2005
8998	0	1	1	703	Chui Keileung	2003
8999	0	1	1	704	Theresastabauer	2006
9000	0	1	1	705	Eleanayu	2004
9001	0	1	1	705	Polawygonowska	2003
9002	0	1	1	707	Sainadeshpande	2005
9003	0	1	1	708	Jelenacvijanovic	2005
9004	0	1	1	709	Laimavladson	2007
9005	0	1	1	710	Andreapalazon	2004
9006	0	1	1	711	Annikaplaninsek	2003
9007	0	1	1	712	MARIA DOMINIQUEfuenzalida	2006
9008	0	1	1	713	Man Wai Chlorisli	2005
9009	0	1	1	714	Saoirsebreen	2004
9010	0	1	1	715	Mariataranova	2005
9011	0	1	1	716	Ekaterinaivanova	2006
9012	0	1	1	717	Raissapetrovskaya	2005
9013	0	1	1	718	Scarlettperkins	2005
9014	0	1	1	719	Erinmckenzie	2005
9015	0	1	1	720	Ksenijarage	2006
9016	0	1	1	721	Dunevaissaud	2007
9017	0	1	1	722	Soniapieczynska	2005
9018	0	1	1	723	Sofiiamykhailets	2007
9019	0	1	1	724	Anastasiiavashchenko	2004
9020	0	1	1	725	Orianaizarra	2004
9021	0	1	1	726	Marinaskegro	2004
9022	0	1	1	727	Monikastankiewicz	2006
9023	0	1	1	728	Valentinetalaki	2006
9024	0	1	1	729	Ivasepa	2004
9025	0	1	1	730	BoYoungjeong	2003
9026	0	1	1	731	Ameliabissett	2003
9027	0	1	1	732	Josieusereau	2005
9028	0	1	1	733	Brittdu pree	2007
9029	0	1	1	734	Chloenoel	2004
9030	0	1	1	735	Daryazykova	2005
9031	0	1	1	736	Anikalemus maldonado	2005
9032	0	1	1	737	Meriemben ezzedine	2006
9033	0	1	1	738	Kurumitamura	2004
9034	0	1	1	739	Lilly Paulineschultz	2004
9035	0	1	1	740	Mirjanajovanovic	2004
9036	0	1	1	741	Amelie Justinehejtmanek	2007
9037	0	1	1	742	Jadehaller	2004
9038	0	1	1	743	Catherinewalker	2005
9039	0	1	1	744	Abbymason	2003
9040	0	1	1	745	Vesagjinaj	2004
9041	0	1	1	746	Viktoriehazmukova	2005
9042	0	1	1	747	Bellabergkvist larsson	2006
9043	0	1	1	748	Riotsuda	2006
9044	0	1	1	749	Naonishino	2005
9045	0	1	1	750	Jaquelynogunwale	2005
9046	0	1	1	751	Raquelcaballero chica	2005

9047	0	1	1	752	Erikabosaia	2006
9048	0	1	1	753	Haniaabouelsaad	2003
9049	0	1	1	754	Andra Izabellasirbu	2005
9050	0	1	1	755	Mariaconstantinou	2003
9051	0	1	1	756	Arinaarifullina	2006
9052	0	1	1	757	Joulia Cristianacirstea	2006
9053	0	1	1	758	Nikastrasek	2003
9054	0	1	1	759	Tejsavixxx	2008
9055	0	1	1	760	Elizabeteklavinska	2003
9056	0	1	1	761	Deliagaillard	2006
9057	0	1	1	762	Mariasysoeva	2006
9058	0	1	1	763	Nataliakoppova	2006
9059	0	1	1	764	Kei Yaucheung	2004
9060	0	1	1	765	Valeriaavdeeva	2004
9061	0	1	1	766	Yifeihuang	2007
9062	0	1	1	767	Chandreboshoff	2004
9063	0	1	1	768	Alessandrateodosescu	2006
9064	0	1	1	769	Emmafantusis	2006
9065	0	1	1	770	Claudiagasparovic	2004
9066	0	1	1	771	Adeolagreatorex	2004
9067	0	1	1	772	Anastasiayashchenko	2006
9068	0	1	1	773	Ivajovic	2007
9069	0	1	1	774	Yoshinofujita	2005
9070	0	1	1	775	Tabithaholmes	2005
9071	0	1	1	776	Lisenrinman	2005
9072	0	1	1	777	Marianacampino	2003
9073	0	1	1	778	Ademiamankeldi	2006
9074	0	1	1	779	Yaellevaissaud	2004
9075	0	1	1	780	Shachflieberman	2003
9076	0	1	1	781	Mariapukhina	2003
9077	0	1	1	782	Agnesmitteregger	2004
9078	0	1	1	783	Michelacastro zunino	2006
9079	0	1	1	784	Hanael deeb	2004
9080	0	1	1	785	Margotphanthala	2007
9081	0	1	1	786	Africaburillo berezak	2007
9082	0	0	1	787	Leydengames	2003
9083	0	0	1	787	Kaitlyncarnicella	2005
9084	0	0	1	787	Bayleysheinin	2003
9085	0	0	1	790	Emmasun	2004
9086	0	0	1	790	Kashishbote	2004
9087	0	0	1	790	Anne Christinelutkemeyer obre	2004
9088	0	0	1	793	Constancelevivier	2005
9089	0	0	1	794	Britmartin	2006
9090	0	0	1	795	Lakshanyavishwanath	2003
9091	0	0	1	796	Chelseachakanyuka	2006
9092	0	0	1	797	Marianthichristoforidou	2005
9093	0	0	1	798	Tyra Caterinagrant	2008
9094	0	0	1	799	Imogenhaddad	2005
9095	0	0	1	800	Zuzanavancelova	2004
9096	0	0	1	801	Emeliegabran	2003

9097	0	0	1	802	Hayleeconway	2006
9098	0	0	1	803	Vendulavaldmannova	2007
9099	0	0	1	804	Aliciaowegi	2004
9100	0	0	1	805	Sofiamikhailova	2005
9101	0	0	1	806	Indiralepage	2006
9102	0	0	1	807	Marieslamenikova	2007
9103	0	0	1	808	Meritxellboix molla	2004
9104	0	0	1	809	Raphaelleleroux	2006
9105	0	0	1	810	Leaaschenberger	2006
9106	0	0	1	811	Magdalenanunez pureco	2003
9107	0	0	1	812	Ericajessel	2006
9108	0	0	1	813	Camiliasamel druz	2006
9109	0	0	1	814	Emmajouy	2005
9110	0	0	1	815	Mariaaraoz-gosn	2006
9111	0	0	1	816	Mariagalatescu	2006
9112	0	0	1	817	Emmadong	2007
9113	0	0	1	818	Taisiayakunina	2004
9114	0	0	1	819	Ya Yiyang	2004
9115	0	0	1	820	Varvarapanshina	2006
9116	0	0	1	821	Satiaubakirova	2006
9117	0	0	1	822	Baylenbrown	2004
9118	0	0	1	823	Olgagolas	2003
9119	0	0	1	824	Viktorialackova	2005
9120	0	0	1	825	An Sofiebeckers	2004
9121	0	0	1	826	Norazuvela	2005
9122	0	0	1	827	Anet Angelikakoskel	2003
9123	0	0	1	828	Caterinasalas	2004
9124	0	0	1	829	Vanesasuarez	2005
9125	0	0	1	830	Mikaylaplitt	2004
9126	0	0	1	831	Daryavelikova	2004
9127	0	0	1	832	Shadiaamado	2003
9128	0	0	1	833	Indiafenieys	2005
9129	0	0	1	834	Francescagandolfi	2006
9130	0	0	1	835	Elenikarantali	2006
9131	0	0	1	836	Tildestromquist	2004
9132	0	0	1	837	Ozi-oiza Josephineyakubu	2005
9133	0	0	1	838	Margheritamarcon	2005
9134	0	0	1	839	Shumanwu	2004
9135	0	0	1	840	Martina Mariabovio	2005
9136	0	0	1	841	Alexandraiordache	2003
9137	0	0	1	842	Hannatsitavets	2005
9138	0	0	1	843	Sarahliu	2004
9139	0	0	1	844	Leoniemoeller	2004
9140	0	0	1	845	Shonanakano	2004
9141	0	0	1	846	Sun Minha	2004
9142	0	0	1	847	Maria Helenasodre	2004
9143	0	0	1	848	Avabruno	2005
9144	0	0	1	849	Kseniiasaenko	2005
9145	0	0	1	850	Samanthanunez	2005
9146	0	0	1	851	Nataliesyrova	2006

9147	0	0	1	852	Edaarli	2004
9148	0	0	1	853	Petrajanigova	2005
9149	0	0	1	854	Kasiatreiber	2003
9150	0	0	1	855	Gabrielafelix da silva	2006
9151	0	0	1	856	Emma Ottaviaghirardato	2005
9152	0	0	1	857	Lindaziets segura	2005
9153	0	0	1	858	Gloriaalogo piqueras	2004
9154	0	0	1	859	Sin Yuche	2005
9155	0	0	1	860	Camila Ceciliamiranda jove	2004
9156	0	0	1	861	Elettragradassi	2005
9157	0	0	1	862	Valentinasteiner	2006
9158	0	0	1	863	Anjalirathi	2006
9159	0	0	1	864	Terezapolakova	2004
9160	0	0	1	865	Gaiagreco	2006
9161	0	0	1	866	Annapinaieva	2004
9162	0	0	1	867	Ninaplihal	2004
9163	0	0	1	868	Isabellasvahn	2006
9164	0	0	1	869	Marcelalopez	2004
9165	0	0	1	870	Kelly Loanavargas gonzalez	2004
9166	0	0	1	871	Irinaartemova	2004
9167	0	0	1	872	Ceylinipek	2006
9168	0	0	1	873	Matiacristiani	2004
9169	0	0	1	874	Asliozcan	2004
9170	0	0	1	875	Bianca Elenabarbulescu	2003
9171	0	0	1	876	Givenroach	2005
9172	0	0	1	877	Hannatelzynska	2005
9173	0	0	1	878	Yasminezzat	2003
9174	0	0	1	879	Manuelagomez gomez	2004
9175	0	0	1	880	Despoinamastrodima	2004
9176	0	0	1	880	Tiannarangan	2006
9177	0	0	1	882	Noelle Vivianfaber	2004
9178	0	0	1	883	Millieskelton	2005
9179	0	0	1	884	Sofiasnitko	2005
9180	0	0	1	885	Carlebotha	2005
9181	0	0	1	886	Lunadinoto fernandez	2006
9182	0	0	1	887	Tamarasramkova	2006
9183	0	0	1	888	Annakobayashi	2005
9184	0	0	1	889	Karolinakrajmer	2006
9185	0	0	1	890	Chiaradavi	2006
9186	0	0	1	891	Andrearoots	2005
9187	0	0	1	892	Irinawenger	2005
9188	0	0	1	892	Isabelamocelin romanichen	2004
9189	0	0	1	894	Kristinakudryavtseva	2003
9190	0	0	1	895	Danielarubio	2005
9191	0	0	1	896	Alinavasilenko	2004
9192	0	0	1	897	Sydniratliff	2003
9193	0	0	1	898	Pearliezhang	2006
9194	0	0	1	899	Hannahsmith	2004
9195	0	0	1	900	Rubycooling	2007
9196	0	0	1	901	Kyarabelhadj	2005

9197	0	0	1	902	Adrianableichner	2005
9198	0	0	1	903	Reneezhang	2006
9199	0	0	1	904	Ana Paulachavez sanchez	2004
9200	0	0	1	905	Tamrynbrown	2005
9201	0	0	1	906	Arinaoreshchenkova	2005
9202	0	0	1	907	Malakasalem	2005
9203	0	0	1	908	Yoanadudova	2004
9204	0	0	1	909	Carlafity	2006
9205	0	0	1	910	Helin Alessandraalvarez prado	2005
9206	0	0	1	911	Alessandrasimone	2003
9207	0	0	1	912	Serenamanca	2007
9208	0	0	1	913	Rahmajendoubi	2005
9209	0	0	1	914	Romicohen	2004
9210	0	0	1	915	Dilnazmashabayeva	2006
9211	0	0	1	916	Nalediraguin	2006
9212	0	0	1	917	Zofiacubonova	2004
9213	0	0	1	918	Navyavadlamudi	2004
9214	0	0	1	919	Mirthemoens	2006
9215	0	0	1	920	Maria Dacianaciubotaru	2005
9216	0	0	1	921	Sakonakaoka	2005
9217	0	0	1	922	Stellajurina	2005
9218	0	0	1	923	Rubyyoung	2006
9219	0	0	1	924	Emmapedretti	2004
9220	0	0	1	925	Mariacharl	2003
9221	0	0	1	926	Pierinalescher	2005
9222	0	0	1	927	Palinasaulevich	2004
9223	0	0	1	928	Hannahread	2006
9224	0	0	1	929	Alinalaptieva	2005
9225	0	0	1	930	Ulyanaromanova	2005
9226	0	0	1	931	Alessandracristiani	2006
9227	0	0	1	932	Morganmccarthy	2004
9228	0	0	1	933	Kamornwankornsila	2006
9229	0	0	1	934	Samgrosjean	2006
9230	0	0	1	935	Philippafaerber	2007
9231	0	0	1	936	Tabethtadoum	2004
9232	0	0	1	937	Teazivic	2005
9233	0	0	1	938	Lizanneboyer	2004
9234	0	0	1	939	Aline Midorida silva	2004
9235	0	0	1	940	Lok Shutang	2006
9236	0	0	1	941	Aglayafedorova	2004
9237	0	0	1	942	Laurafuchs	2005
9238	0	0	1	943	Alisabaranovska	2005
9239	0	0	1	944	Biancabulat	2005
9240	0	0	1	945	Marianazottoli	2005
9241	0	0	1	946	Anastasijaadeikyte	2004
9242	0	0	1	947	Alicesoulie	2006
9243	0	0	1	948	Barbaramartinez	2003
9244	0	0	1	949	Sofia Lorenmunoz munoz	2006
9245	0	0	1	950	Minseokim	2004
9246	0	0	1	951	Parniaroknabadi	2003

9247	0	0	1	952	Nataliacastaneda guerrero	2005
9248	0	0	1	953	Ashleykatz	2006
9249	0	0	1	954	Lorintilipman	2003
9250	0	0	1	955	Emersonjones	2008
9251	0	0	1	956	Annakaraseva	2005
9252	0	0	1	957	Reyacoe	2005
9253	0	0	1	958	Teanikcevic	2005
9254	0	0	1	959	Ifigeneiakafka	2005
9255	0	0	1	960	Naomihagi	2007
9256	0	0	1	961	Ana Paulamartinez mora	2003
9257	0	0	1	962	Mihaelatsoneva	2004
9258	0	0	1	963	Polinagumeniuk	2007
9259	0	0	1	964	Yaraalhaqbani	2004
9260	0	0	1	965	Reinagoto	2007
9261	0	0	1	966	Anastasiapettersson	2006
9262	0	0	1	967	Amaheecharrier	2004
9263	0	0	1	968	Kellyarends	2003
9264	0	0	1	969	Marysidelnyk	2008
9265	0	0	1	970	Christinekruger	2003
9266	0	0	1	971	Klaravaja	2006
9267	0	0	1	972	Ioanadragomir	2004
9268	0	0	1	973	Susanasouhrada	2006
9269	0	0	1	974	Vaidamatuseviciute	2006
9270	0	0	1	975	Elenakim	2003
9271	0	0	1	976	Ninacostalas	2007
9272	0	0	1	977	Elizavetalobanova	2006
9273	0	0	1	978	Radazolotareva	2008
9274	0	0	1	979	Sabrinakalandarov	2003
9275	0	0	1	980	Daryakharlanova	2006
9276	0	0	1	981	Loisboisson	2003
9277	0	0	1	982	Nishithasaravanan	2004
9278	0	0	1	983	Sofia Janewong enqi	2006
9279	0	0	1	984	Carlatanguy	2006
9280	0	0	1	985	Lauragaldano	2006
9281	0	0	1	986	Jaynaclemens	2004
9282	0	0	1	987	Allabetina	2003
9283	0	0	1	988	Nourhassan moustafa	2005
9284	0	0	1	989	Defnecirpanli	2004
9285	0	0	1	990	Cezarapencea	2004
9286	0	0	1	991	Mettesteegmans	2006
9287	0	0	1	992	Evazabolotnaia	2007
9288	0	0	1	993	Nandinidixit	2005
9289	0	0	1	994	Paulinajurkowska	2005
9290	0	0	1	995	Nellyvighova	2004
9291	0	0	1	996	Dainheekim	2004
9292	0	0	1	997	Florencefohnen	2005
9293	0	0	1	998	Sadescharlin	2005
9294	0	0	1	999	Sofiiazhylchuk	2005
9295	0	0	1	1000	Laurabrunkel	2006
9296	0	0	1	1001	Anamartinez vaquero	2006

9297	0	0	1	1002	Nairagevorkian	2006
9298	0	0	1	1003	Inescosta	2005
9299	0	0	1	1004	Nelajandova	2007
9300	0	0	1	1005	Inelamprecht	2003
9301	0	0	1	1006	Aadainna	2004
9302	0	0	1	1007	Kenishamoning	2006
9303	0	0	1	1008	Rheamakesar	2006
9304	0	0	1	1009	Jasminmakela	2004
9305	0	0	1	1010	Josefafuenzalida	2005
9306	0	0	1	1011	Lauranadaska	2004
9307	0	0	1	1012	Sharvanishrestha	2005
9308	0	0	1	1013	Rominazeynali sourmanabadi	2005
9309	0	0	1	1014	Loubaudouin	2004
9310	0	0	1	1015	Ivaivanova	2006
9311	0	0	1	1016	Riyahdugar	2004
9312	0	0	1	1017	Chiarajerolimov	2006
9313	0	0	1	1018	Sanneschalekamp	2003
9314	0	0	1	1019	Liyapencheva	2004
9315	0	0	1	1020	Emmabelluomini	2004
9316	0	0	1	1021	Ada Pinarakarabacak	2005
9317	0	0	1	1022	Maayanlaron	2007
9318	0	0	1	1023	Idajohansson	2004
9319	0	0	1	1024	Maria Valentinasalek rivero	2005
9320	0	0	1	1025	Paulinafarchetto	2005
9321	0	0	1	1026	Kirabataikina	2007
9322	0	0	1	1027	Tildahessleryd	2006
9323	0	0	1	1028	Alaiarubio perez	2004
9324	0	0	1	1029	Aimeebrown	2003
9325	0	0	1	1030	Kannchayachungwatana	2007
9326	0	0	1	1031	Aleksandrawegrzyn	2004
9327	0	0	1	1032	Nisabulut	2006
9328	0	0	1	1033	Anastasialim	2004
9329	0	0	1	1034	Kaili Demiteso	2006
9330	0	0	1	1035	Katiecodd	2004
9331	0	0	1	1035	Andreanova	2003
9332	0	0	1	1037	Anelyakildiyarova	2006
9333	0	0	1	1038	Camilaquirarte	2006
9334	0	0	1	1039	Chahanabudhbhatti	2005
9335	0	0	1	1040	Monikawojcik	2005
9336	0	0	1	1041	Mariamatia	2005
9337	0	0	1	1042	Benthede bresser	2005
9338	0	0	1	1043	Lineabajraliu	2007
9339	0	0	1	1044	Artemisaslanisvili	2006
9340	0	0	1	1045	Punninkovapitukted	2003
9341	0	0	1	1045	Marleylambert	2004
9342	0	0	1	1047	Emmacohen	2005
9343	0	0	1	1048	Veda Rajuprapurna	2005
9344	0	0	1	1049	Anastasijaignjatic	2006
9345	0	0	1	1050	Aliceferlito	2005
9346	0	0	1	1051	Aleksandra Sophialopez savos	2004

9347	0	0	1	1052	Reesecalvo	2007
9348	0	0	1	1053	Arinababenko	2004
9349	0	0	1	1054	Maria Carolinadepablos ochoa	2005
9350	0	0	1	1055	Yonthatadoum	2006
9351	0	0	1	1056	Magdalenanavratilova	2005
9352	0	0	1	1057	Vladahaetcaia	2004
9353	0	0	1	1058	Sonyakovalenko	2005
9354	0	0	1	1059	Tamaraermakova	2007
9355	0	0	1	1060	Kristynadamaskova	2005
9356	0	0	1	1061	Nadialagaev	2008
9357	0	0	1	1062	Liviaairoldi	2005
9358	0	0	1	1063	Smiltebakyte	2004
9359	0	0	1	1064	Valeriasan miguel soria	2004
9360	0	0	1	1065	Aivaschmitz	2004
9361	0	0	1	1066	Mariamgabr	2005
9362	0	0	1	1067	Madisonschwarz	2004
9363	0	0	1	1068	Alexia Laviniapuiac	2005
9364	0	0	1	1069	Thessyntondele zinga	2006
9365	0	0	1	1070	Emilijazdravkovic	2005
9366	0	0	1	1071	Nataliaturczuk	2004
9367	0	0	1	1072	Reesemiller	2005
9368	0	0	1	1073	Melissayakup pour	2004
9369	0	0	1	1074	Elifalbayrak	2004
9370	0	0	1	1075	Leticiatarocco	2005
9371	0	0	1	1076	Klarakajabova	2004
9372	0	0	1	1077	Renatanaka	2005
9373	0	0	1	1078	Amodininaik	2006
9374	0	0	1	1079	Valentinatorres da silva	2004
9375	0	0	1	1080	Eva Mariaionescu	2007
9376	0	0	1	1081	Tanishakantam	2007
9377	0	0	1	1082	Tsitsi Clairemahere	2003
9378	0	0	1	1083	Onsboubaker	2005
9379	0	0	1	1084	Sanagarakani	2003
9380	0	0	1	1085	Linegreyling	2004
9381	0	0	1	1086	Nadiarodewald	2006
9382	0	0	1	1087	Anastasiasimonov	2004
9383	0	0	1	1088	Aarni Reddyyellu	2004
9384	0	0	1	1089	Anjastankovic	2005
9385	0	0	1	1090	Sofyakashchenko	2005
9386	0	0	1	1091	Jiayiliu	2004
9387	0	0	1	1092	Sophiafuller	2006
9388	0	0	1	1093	Lakshmigowda	2005
9389	0	0	1	1094	Camillabossi	2003
9390	0	0	1	1094	Anastasiyafeklistova	2003
9391	0	0	1	1096	Emmalindh	2005
9392	0	0	1	1097	Sofiablanco	2003
9393	0	0	1	1097	Sofiafortuno	2003
9394	0	0	1	1099	Sofiapavlova	2005
9395	0	0	1	1100	Lenacouto	2007
9396	0	0	1	1101	Ivalakic	2008

9397	0	0	1	1102	Elleedryer	2004
9398	0	0	1	1103	Fatemesadatzanjani	2004
9399	0	0	1	1104	Ariannade bernardo	2004
9400	0	0	1	1105	Emilyniers	2004
9401	0	0	1	1106	Mariamiroshnichenko	2006
9402	0	0	1	1107	Daria Stefaniamalaescu	2005
9403	0	0	1	1108	Habibaelanany	2006
9404	0	0	1	1109	Neratesankic	2006
9405	0	0	1	1110	Amaliawiddowson	2008
9406	0	0	1	1111	Valeriacenteno mendoza	2004
9407	0	0	1	1112	Rawansherif	2004
9408	0	0	1	1113	Ominahonvalihanova	2004
9409	0	0	1	1114	Verayesenkova	2004
9410	0	0	1	1115	Juliapeer	2005
9411	0	0	1	1116	Anaisfernandez laaksonen	2005
9412	0	0	1	1117	Kahooyama	2005
9413	0	0	1	1118	Pavitraparikh	2005
9414	0	0	1	1119	Mariana Isabelhiguita barraza	2007
9415	0	0	1	1120	Clairechan	2004
9416	0	0	1	1121	Onyuchoi	2006
9417	0	0	1	1122	Alinanesmianovych	2005
9418	0	0	1	1123	Rennesingla	2004
9419	0	0	1	1124	Nicole Robertaserraglio	2004
9420	0	0	1	1125	Natalieblock	2004
9421	0	0	1	1126	Zlatachinnova	2007
9422	0	0	1	1127	Katerinamandelikova	2003
9423	0	0	1	1127	Mikadagan fruchtman	2003
9424	0	0	1	1127	Sibleycharles	2005
9425	0	0	1	1127	Ameliahoner	2003
9426	0	0	1	1127	Alicexu	2004
9427	0	0	1	1132	Ivakuzmanoska	2004
9428	0	0	1	1132	Juliettenask	2003
9429	0	0	1	1134	Florianapopovici	2004
9430	0	0	1	1135	Imankhan	2003
9431	0	0	1	1136	Ioana Teodorasava	2005
9432	0	0	1	1137	Yanamykytyn	2004
9433	0	0	1	1137	Florentinedekkers	2004
9434	0	0	1	1139	Danielapiani	2007
9435	0	0	1	1140	Mariianikitash	2004
9436	0	0	1	1141	Juliarocha de macedo moraes	2003
9437	0	0	1	1142	Sophiawang	2004
9438	0	0	1	1142	Nellietaraba wallberg	2007
9439	0	0	1	1144	Luciaaranda arcas	2004
9440	0	0	1	1145	Natalijaiani	2005
9441	0	0	1	1146	Lucywebber	2003
9442	0	0	1	1147	Rianna Ioanacoreisa	2004
9443	0	0	1	1148	Luanapaiva	2006
9444	0	0	1	1149	Arianesouligny	2004
9445	0	0	1	1150	Audreytong	2006
9446	0	0	1	1151	Gretafenyves	2005

9447	0	0	1	1152	Polinabolotova	2006
9448	0	0	1	1153	Kamilapoplawska	2005
9449	0	0	1	1154	Avawilliamson	2006
9450	0	0	1	1155	Alexandramagia	2006
9451	0	0	1	1156	Maaikevanschoenbeek	2006
9452	0	0	1	1157	Eun Jilee	2004
9453	0	0	1	1158	Yeong-Eunryu	2004
9454	0	0	1	1159	Ellakordik	2003
9455	0	0	1	1160	Mariastudennikova	2004
9456	0	0	1	1161	Mariagell	2006
9457	0	0	1	1162	Sophiestanton	2007
9458	0	0	1	1163	Evamuller uhan	2005
9459	0	0	1	1164	Clemencethouard	2005
9460	0	0	1	1165	Oliwiaorlinska	2005
9461	0	0	1	1166	Brooklynolson	2006
9462	0	0	1	1167	Bertamiret avante	2004
9463	0	0	1	1168	Eddamamedova	2006
9464	0	0	1	1169	Milicapopovski	2006
9465	0	0	1	1170	Lironloiter	2006
9466	0	0	1	1171	Ayakakoga	2004
9467	0	0	1	1172	Catherinegagnon	2006
9468	0	0	1	1173	Audreywijono	2004
9469	0	0	1	1174	Sudeince	2004
9470	0	0	1	1175	Gargipawar	2003
9471	0	0	1	1176	Mia Sophiemueller	2004
9472	0	0	1	1177	Karakatkoilybaeva	2006
9473	0	0	1	1178	Sofiiasuslova	2004
9474	0	0	1	1179	Tahliakokkinis	2008
9475	0	0	1	1180	Hannahnagpal	2005
9476	0	0	1	1181	Nicolematukutire	2005
9477	0	0	1	1182	Miawainwright	2007
9478	0	0	1	1183	Majabajorek	2004
9479	0	0	1	1184	Aislingo'connor	2003
9480	0	0	1	1185	Olgasorochkina	2004
9481	0	0	1	1185	Isabellacolmenares b	2004
9482	0	0	1	1187	Mariellagelbke	2005
9483	0	0	1	1188	SANJANAdevineni	2007
9484	0	0	1	1189	TOWAKIamita	2006
9485	0	0	1	1190	Anjapetkovic	2005
9486	0	0	1	1191	Mayaboonyaaroonnet	2007
9487	0	0	1	1192	Carolinalima de oliveira	2003
9488	0	0	1	1193	Marissaminetti	2005
9489	0	0	1	1194	Ninakyselova	2006
9490	0	0	1	1195	Piperfreeman	2004
9491	0	0	1	1196	Ameliebrooks	2006
9492	0	0	1	1197	Noadujardin	2003
9493	0	0	1	1197	Yuranakagawa	2003
9494	0	0	1	1199	Kayonishimura	2006
9495	0	0	1	1200	Rachelhermanova	2003
9496	0	0	1	1201	Esterfesiuk	2004

9497	0	0	1	1202	Alexis Larachua	2005
9498	0	0	1	1203	Patriciekubikova	2004
9499	0	0	1	1204	Marlenefoerster	2004
9500	0	0	1	1205	Salomefluri	2005
9501	0	0	1	1206	REBECCAekpenyong	2004
9502	0	0	1	1207	Ekaterinasuvorova	2005
9503	0	0	1	1208	Juliagonzalez romero	2005
9504	0	0	1	1209	Andreakrejzova	2004
9505	0	0	1	1210	Beverleynyangon	2007
9506	0	0	1	1211	Valentinamozas pavon	2004
9507	0	0	1	1212	Sofiapinto	2004
9508	0	0	1	1213	Niyatikukreti	2006
9509	0	0	1	1214	Marcelladessolis	2005
9510	0	0	1	1215	Marianajimenez bustillo	2004
9511	0	0	1	1216	Luciamartinez gomez	2003
9512	0	0	1	1217	Iremkurt	2007
9513	0	0	1	1218	Dilaraokur	2006
9514	0	0	1	1219	Cristinaramos sierra	2006
9515	0	0	1	1220	Bhaktishah	2003
9516	0	0	1	1221	Nathaliemokhtar	2003
9517	0	0	1	1222	Iulia Andreeaionescu	2005
9518	0	0	1	1223	Cristinadiaz adrover	2005
9519	0	0	1	1224	Brunamelato	2006
9520	0	0	1	1225	Daniellagalvez isaacs	2003
9521	0	0	1	1226	Isabellaventer	2003
9522	0	0	1	1227	Larissa Cristinasilva	2005
9523	0	0	1	1228	Loisnewberry	2007
9524	0	0	1	1229	Hau Ching Rosalynchan	2008
9525	0	0	1	1230	Anetajunova	2006
9526	0	0	1	1231	Ekaterinadoroshenko	2005
9527	0	0	1	1232	Paolalopez	2006
9528	0	0	1	1233	Victorialazarova	2004
9529	0	0	1	1234	Annetterobertson	2004
9530	0	0	1	1235	Se-Yeonyang	2005
9531	0	0	1	1236	Maria Fernandahernandez	2004
9532	0	0	1	1237	Gabriellevillegas	2007
9533	0	0	1	1238	Patriciaungure rogozoveca	2005
9534	0	0	1	1239	Evaalvarez sande	2004
9535	0	0	1	1240	Danielalozano	2005
9536	0	0	1	1241	Chloeschwarz	2004
9537	0	0	1	1242	Danielalivson	2005
9538	0	0	1	1243	Yunakadowaki	2004
9539	0	0	1	1244	Varvaraalexeyeva	2005
9540	0	0	1	1244	Isabelladaher vaz castro	2005
9541	0	0	1	1246	Mariaustic	2007
9542	0	0	1	1247	Adibitton	2007
9543	0	0	1	1247	PUSHTIladdha	2006
9544	0	0	1	1249	Brendayoris	2004
9545	0	0	1	1250	Juaeshin	2004
9546	0	0	1	1251	Sirenawaas	2006

9547	0	0	1	1252	Annakislyak	2007
9548	0	0	1	1253	Senemocal	2004
9549	0	0	1	1254	katerinamelicharova	2008
9550	0	0	1	1255	Pawineeruamrak	2003
9551	0	0	1	1256	Yoanaradulova	2005
9552	0	0	1	1257	Vittoriabaccino	2004
9553	0	0	1	1258	Anastasiagolubkova	2004
9554	0	0	1	1259	Morganjordaan	2008
9555	0	0	1	1260	Anastasiaradovanovic	2004
9556	0	0	1	1261	Kseniiapiskareva	2006
9557	0	0	1	1262	Caylinele grange	2003
9558	0	0	1	1263	Yenzokuhledladla	2007
9559	0	0	1	1264	Biancabernardes	2006
9560	0	0	1	1265	Aelyarai	2006
9561	0	0	1	1266	Stefanisandoiu shtein	2005
9562	0	0	1	1267	Hlael-sakka	2003
9563	0	0	1	1268	Hetveechaudhari	2004
9564	0	0	1	1269	Stylianiroussopoulou	2003
9565	0	0	1	1270	Sofiyazueva	2004
9566	0	0	1	1271	Josephinekunz	2006
9567	0	0	1	1272	Jessieculley	2005
9568	0	0	1	1273	Ophelieboullay	2008
9569	0	0	1	1274	Zalimorris	2004
9570	0	0	1	1274	Myroslavazelenchuk	2004
9571	0	0	1	1276	Lilitha Sithenkosindungane	2005
9572	0	0	1	1277	Victoriapohle	2007
9573	0	0	1	1278	Nathaliamossambani	2003
9574	0	0	1	1279	Panatchakornsuanarsa	2003
9575	0	0	1	1280	Serafimabobrovnikova	2006
9576	0	0	1	1281	Eszterberenyi	2005
9577	0	0	1	1282	Giojang	2005
9578	0	0	1	1283	Basmalaaboutaleb	2004
9579	0	0	1	1284	Annademirtzi	2007
9580	0	0	1	1285	Annatheasara	2006
9581	0	0	1	1285	Ivanovalucero moreta	2004
9582	0	0	1	1287	Enabaltic	2003
9583	0	0	1	1288	Fabianalabrador	2006
9584	0	0	1	1289	Valentinade sousa	2004
9585	0	0	1	1290	Milinasubkhankulova	2007
9586	0	0	1	1291	Sarahmessenlechner	2007
9587	0	0	1	1292	Mayfadida	2007
9588	0	0	1	1293	Tildalarsson	2006
9589	0	0	1	1294	Sarah Elizabethpartida garcia	2006
9590	0	0	1	1295	ALEXIAestrada	2007
9591	0	0	1	1296	Sashikempster	2003
9592	0	0	1	1296	Maelleleclercq	2004
9593	0	0	1	1298	Marinabulbarella	2005
9594	0	0	1	1299	Noemiedadoun	2006
9595	0	0	1	1300	Marianazurita berdecio	2005
9596	0	0	1	1301	Astamiettinen	2004

9597	0	0	1	1302	Hrudayashah	2003
9598	0	0	1	1302	Evelyn Alessandragomez	2003
9599	0	0	1	1304	Sheena Jademasuda karrasch	2003
9600	0	0	1	1304	Gabrielaskrabalova	2005
9601	0	0	1	1306	Marianneangel	2008
9602	0	0	1	1307	Mariagabriel	2006
9603	0	0	1	1308	Emmakuusisto	2005
9604	0	0	1	1309	Khushalimodi	2005
9605	0	0	1	1310	Koronayasherugara	2007
9606	0	0	1	1311	Naghelygarcia galian	2005
9607	0	0	1	1312	Oceannelopez	2004
9608	0	0	1	1313	Isabellaquintero garcia	2005
9609	0	0	1	1314	Tsehaydriscoll	2005
9610	0	0	1	1314	Akarimatsuno	2003
9611	0	0	1	1316	Jane Ling Chunwong	2007
9612	0	0	1	1317	Malakahmed adel	2006
9613	0	0	1	1318	Sierraberry	2004
9614	0	0	1	1318	Barbaratrocel	2006
9615	0	0	1	1320	Billurgonlusen	2004
9616	0	0	1	1320	Kit Yi Kayeau-yeung	2003
9617	0	0	1	1322	Nicoletrofimczyk	2006
9618	0	0	1	1323	Carolinraschdorf	2006
9619	0	0	1	1324	Ronjascheuermann	2004
9620	0	0	1	1325	Aliyamusabekova	2004
9621	0	0	1	1326	Ayamatsunaga	2005
9622	0	0	1	1327	Chisatokanemaki	2006
9623	0	0	1	1328	Alexavolkov	2005
9624	0	0	1	1329	Antoniettabocache martins	2006
9625	0	0	1	1330	Divine Dasamnweke	2004
9626	0	0	1	1330	Heykarodriguez	2005
9627	0	0	1	1332	Erica Kannahagi	2005
9628	0	0	1	1333	Katalinapetrova	2006
9629	0	0	1	1334	Ariliboshoff	2005
9630	0	0	1	1335	Sarahdev	2003
9631	0	0	1	1336	Vladakozak	2006
9632	0	0	1	1336	Shizukasakurada	2004
9633	0	0	1	1338	Martynapiechowicz	2005
9634	0	0	1	1339	mazamesso gracetchamoussa	2006
9635	0	0	1	1340	Miapopovic	2004
9636	0	0	1	1341	Averyjennings	2006
9637	0	0	1	1342	Cynthiawanjala	2005
9638	0	0	1	1342	Munisaashurbekova	2005
9639	0	0	1	1344	Aleksandralobanova	2005
9640	0	0	1	1345	Georginahays	2006
9641	0	0	1	1346	Toyin Shewaasogba	2003
9642	0	0	1	1347	Laracharles	2004
9643	0	0	1	1348	Denisahanakova	2004
9644	0	0	1	1349	Shihomi Li Xuanleong	2007
9645	0	0	1	1350	Carlarodier begue	2006
9646	0	0	1	1351	Pipercharney	2005

9647	0	0	1	1352	Ji Woochoi	2004
9648	0	0	1	1353	Sarah-Annewong anqi	2006
9649	0	0	1	1354	Lanastanisic	2004
9650	0	0	1	1354	Juliebousseau	2003
9651	0	0	1	1356	Paolaueno dalmonico	2006
9652	0	0	1	1357	Pariyakornsrisuriyapatkul	2007
9653	0	0	1	1358	Tanushripandey	2004
9654	0	0	1	1359	Riyasachdeva	2007
9655	0	0	1	1359	Alaraayaz	2005
9656	0	0	1	1361	Miraykonar	2003
9657	0	0	1	1362	Georgiakalamaris	2006
9658	0	0	1	1363	Tabathasuarez	2005
9659	0	0	1	1364	Lenathomas iglesias	2006
9660	0	0	1	1365	Elenamercioiu	2005
9661	0	0	1	1366	Margaritaokhendovskaya	2006
9662	0	0	1	1367	Anastasiazhilina	2003
9663	0	0	1	1368	Lenabeckx	2004
9664	0	0	1	1369	Sukhmannibhandari	2005
9665	0	0	1	1370	Gabiapaskauskas	2006
9666	0	0	1	1371	Valentinarodas carcamo	2005
9667	0	0	1	1372	Anastasiarozhkova	2007
9668	0	0	1	1373	Dartavorslava	2004
9669	0	0	1	1374	Noemibabikova	2004
9670	0	0	1	1375	Petekomada	2004
9671	0	0	1	1375	Javieraverdugo	2004
9672	0	0	1	1377	Melisaugur	2007
9673	0	0	1	1378	Vickyveloso	2005
9674	0	0	1	1379	Shahnozamarimova	2006
9675	0	0	1	1380	Samirade stefano	2004
9676	0	0	1	1380	Larapfeifer	2004
9677	0	0	1	1382	Martinaalcaino vergara	2005
9678	0	0	1	1383	Mariaaldea wesselly	2005
9679	0	0	1	1384	Carlaverdu azorin	2006
9680	0	0	1	1385	Sofiadaryaie	2004
9681	0	0	1	1386	Varvaradegtiareva	2004
9682	0	0	1	1387	Giorgiaroselli	2004
9683	0	0	1	1388	Laraeroglu	2005
9684	0	0	1	1389	Leannserna	2004
9685	0	0	1	1390	Alanasubasic	2007
9686	0	0	1	1391	Elisabethdunac	2005
9687	0	0	1	1391	VICTORIApapadopoulou	2004
9688	0	0	1	1391	Rosealix vidal	2005
9689	0	0	1	1394	Adrianapellizzari	2005
9690	0	0	1	1395	Rikkede koning	2003
9691	0	0	1	1396	Camilamordiz perez	2005
9692	0	0	1	1397	Angelesrodriguez-rizo	2006
9693	0	0	1	1398	Linasarhan	2005
9694	0	0	1	1399	Nikoletaparetzoglou	2005
9695	0	0	1	1400	Assaraphonwannapho	2006
9696	0	0	1	1400	Justynajodlowska	2004

9697	0	0	1	1402	Sofiagiraldo zamorano	2005
9698	0	0	1	1403	DESTINYokhinaye	2006
9699	0	0	1	1404	Haramkim	2005
9700	0	0	1	1405	Charnellefozo	2008
9701	0	0	1	1406	Vasilisaborisova	2006
9702	0	0	1	1407	Renenaishtut nikishov	2007
9703	0	0	1	1408	Emma Janebarclay	2003
9704	0	0	1	1409	Nataly Miroslavovaninova	2004
9705	0	0	1	1410	Adelalatalova	2005
9706	0	0	1	1411	Adrianatkachenko	2007
9707	0	0	1	1412	Isabellarighi	2004
9708	0	0	1	1413	Nardinesalem	2007
9709	0	0	1	1414	Mariaandrienko	2004
9710	0	0	1	1415	Madaramarkevica	2006
9711	0	0	1	1415	Emmaleitner	2006
9712	0	0	1	1417	Juliadaroszewska	2005
9713	0	0	1	1418	Erinmaguire	2005
9714	0	0	1	1418	Annaegorova	2005
9715	0	0	1	1420	Saumrita Jagatdeoverma	2007
9716	0	0	1	1420	Ivanagombitova	2004
9717	0	0	1	1422	Mekhalamanna	2004
9718	0	0	1	1423	Abigailelham	2007
9719	0	0	1	1424	Juliacaffarena	2006
9720	0	0	1	1424	Preranakoirala	2003
9721	0	0	1	1426	Allegrakorpanec davies	2007
9722	0	0	1	1427	Ohunene Comfortyakubu	2008
9723	0	0	1	1428	Anjalikotecha	2005
9724	0	0	1	1428	Anastasiahuijsegoms	2008
9725	0	0	1	1430	Benedettasensi	2005
9726	0	0	1	1431	Lucyquinlan	2007
9727	0	0	1	1432	Zeyneperbakan	2006
9728	0	0	1	1433	Maria Piavivenzio	2005
9729	0	0	1	1434	Andjelalazarevic	2005
9730	0	0	1	1435	Ching Laamlai	2007
9731	0	0	1	1435	Faridamoussa	2006
9732	0	0	1	1437	Elayener	2006
9733	0	0	1	1438	Lujanalegre	2005
9734	0	0	1	1439	Kaavyapalani	2003
9735	0	0	1	1439	Carmenlopez martinez	2005
9736	0	0	1	1439	Emilybaek	2006
9737	0	0	1	1442	YUUWAorisaka	2006
9738	0	0	1	1443	Ji Hyunkang	2005
9739	0	0	1	1443	Danielaben hamo	2004
9740	0	0	1	1445	Fabriziacambria	2004
9741	0	0	1	1445	Victoriagomez o'hayon	2003
9742	0	0	1	1445	MARYgodwin	2004
9743	0	0	1	1445	Briannabaldi	2005
9744	0	0	1	1449	Nadezdakhalturina	2004
9745	0	0	1	1449	Brooke Lynnschafer	2005
9746	0	0	1	1451	Amalgati	2003

9747	0	0	1	1452	Aishidas	2008
9748	0	0	1	1453	Francescamaguina bunikowska	2007
9749	0	0	1	1454	Carlotabalseiro	2007
9750	0	0	1	1455	Liepasataite	2006
9751	0	0	1	1455	Sofiavelarde	2003
9752	0	0	1	1457	Nadineabid	2006
9753	0	0	1	1458	Elizavetagnilozubova	2004
9754	0	0	1	1459	Rebeccaamerio	2005
9755	0	0	1	1459	Jadegroen	2004
9756	0	0	1	1459	Mehranehzohourian	2007
9757	0	0	1	1462	Nahyeongcho	2007
9758	0	0	1	1463	Ceciliaalcobe garibay	2003
9759	0	0	1	1464	Kseniamamontova	2003
9760	0	0	1	1464	Dariaartemyeva	2004
9761	0	0	1	1466	Aryanabartlett	2005
9762	0	0	1	1467	Marinade diego quinones	2004
9763	0	0	1	1468	Millelangaas	2003
9764	0	0	1	1469	Lia Kaishikimosimann	2004
9765	0	0	1	1469	Noelani Sarateso	2007
9766	0	0	1	1471	Naishasrivastav	2004
9767	0	0	1	1472	Xenialogvin	2006
9768	0	0	1	1473	Sofiacardenas galvis	2003
9769	0	0	1	1474	Leanneabouhamad	2007
9770	0	0	1	1475	Ana Mariatornean	2004
9771	0	0	1	1476	Arianajativa riofrio	2004
9772	0	0	1	1476	Sarraben amor	2004
9773	0	0	1	1476	Sofiaiantosca	2004
9774	0	0	1	1479	Lexingtonreed	2006
9775	0	0	1	1479	Sudiptakumar	2003
9776	0	0	1	1481	Andra Mariasimion	2006
9777	0	0	1	1482	Carlotafarres carreter	2004
9778	0	0	1	1482	Ewaczapulak	2004
9779	0	0	1	1484	Elif Tuanagunal	2005
9780	0	0	1	1485	Gianocta	2006
9781	0	0	1	1486	Elizabethivanov	2007
9782	0	0	1	1487	Annapetkovic	2005
9783	0	0	1	1488	Eevaristola	2003
9784	0	0	1	1489	Quintyvan dongen	2005
9785	0	0	1	1490	Judithattias	2005
9786	0	0	1	1491	Emilybriggs	2007
9787	0	0	1	1491	Dianabadalyan	2008
9788	0	0	1	1493	Solenygonzalez merida	2005
9789	0	0	1	1493	Kairatan	2007
9790	0	0	1	1495	Camillefisette	2006
9791	0	0	1	1496	Albamartinez	2006
9792	0	0	1	1497	Estergonosova	2006
9793	0	0	1	1498	Maftunabonukahramonova	2005
9794	0	0	1	1499	Mariagolovina	2007
9795	0	0	1	1500	Luciahradecka	2007
9796	0	0	1	1501	Marribera orquin	2004

9797	0	0	1	1502	Saraalba verastegui	2006
9798	0	0	1	1502	Claraschoen	2006
9799	0	0	1	1504	Ustiniyalekomtseva	2006
9800	0	0	1	1505	Laurapliksa	2003
9801	0	0	1	1506	Pornnapathongjumradsin	2008
9802	0	0	1	1507	Nourshafei	2005
9803	0	0	1	1508	Mehermishra	2005
9804	0	0	1	1509	Ha Yeongryu	2003
9805	0	0	1	1510	Juliaviesi santoro pereira	2006
9806	0	0	1	1511	Anlinxie	2005
9807	0	0	1	1512	Sofyagapankova	2006
9808	0	0	1	1513	Kupakwashe Diliakanyore	2007
9809	0	0	1	1514	Tsz Lamyeung	2007
9810	0	0	1	1515	Emiliiamargolina	2004
9811	0	0	1	1516	Margaritaandreiuk	2003
9812	0	0	1	1517	Likabolkvadze	2005
9813	0	0	1	1518	Lucianamendoza	2005
9814	0	0	1	1519	Alexandrabiot	2005
9815	0	0	1	1519	Aokanagasawa	2005
9816	0	0	1	1521	Danielgoldstein	2006
9817	0	0	1	1521	Kagisoledwaba	2004
9818	0	0	1	1523	ISABELLAlechuga franco	2005
9819	0	0	1	1523	Marielafortuny	2005
9820	0	0	1	1525	Clairejaramba	2004
9821	0	0	1	1526	Natalierainke	2004
9822	0	0	1	1526	MARYudoffa	2005
9823	0	0	1	1528	Vladlenamartyunina	2007
9824	0	0	1	1529	Elizavetasabirova	2006
9825	0	0	1	1530	Saraborkop	2006
9826	0	0	1	1531	Veronikapodobed	2004
9827	0	0	1	1532	Katjamarkus	2004
9828	0	0	1	1533	Anna-Raphaelleserghi	2006
9829	0	0	1	1533	Anastasiauspenskaia	2003
9830	0	0	1	1535	Daniela Tarakotara	2005
9831	0	0	1	1536	Helenhernandez	2005
9832	0	0	1	1537	Nedarahimkhani	2007
9833	0	0	1	1537	Angel Lilybean	2006
9834	0	0	1	1539	Olairla-bartyzel	2006
9835	0	0	1	1539	Duru Yagmurdurgun	2005
9836	0	0	1	1541	Karolinamrazikova	2004
9837	0	0	1	1542	Pilarbeveraggi lespiau	2006
9838	0	0	1	1542	Agostinaandreazzini	2004
9839	0	0	1	1544	Mariagarcia	2006
9840	0	0	1	1545	Karenverduzco	2005
9841	0	0	1	1546	Paloma Florenciacaceres villalb	2004
9842	0	0	1	1546	Nicolelukesova	2006
9843	0	0	1	1548	Leonierabl	2007
9844	0	0	1	1549	Reka Katamatko	2003
9845	0	0	1	1550	Yeseniiaovcharova	2005
9846	0	0	1	1551	Nina Marcelachavez vicente	2006

9847	0	0	1	1552	Lolatavcar	2004
9848	0	0	1	1553	Daria Mariamunteanu	2003
9849	0	0	1	1553	Jesychea	2006
9850	0	0	1	1555	Juliebedard	2005
9851	0	0	1	1556	Nataliazaman	2006
9852	0	0	1	1556	Yleymi Lugianamuelle valdez	2008
9853	0	0	1	1556	Emilyvelasco	2006
9854	0	0	1	1559	Avroravolkova	2004
9855	0	0	1	1560	Amalizillmann	2007
9856	0	0	1	1561	Ana Sofiahernandez	2004
9857	0	0	1	1562	Mancalampret	2003
9858	0	0	1	1562	Nataliemala	2006
9859	0	0	1	1564	Kenzyabdelaal	2005
9860	0	0	1	1565	Zeynep Nazdara	2005
9861	0	0	1	1566	Miagarber	2005
9862	0	0	1	1567	Lolade leon	2004
9863	0	0	1	1567	Puckapornjirachavala	2004
9864	0	0	1	1569	Eleftheriakousoula	2004
9865	0	0	1	1570	Maria Josezarza mojoli	2005
9866	0	0	0	1570	Derenozel	2005
9867	0	0	0	1572	Taramihaljevic	2006
9868	0	0	0	1573	Ibifuroclement	2005
9869	0	0	0	1573	Erykakruk	2005
9870	0	0	0	1575	Michelleirigoyen	2005
9871	0	0	0	1575	Elinebex	2007
9872	0	0	0	1577	Dariabibanina	2007
9873	0	0	0	1578	Ninasozaonova	2007
9874	0	0	0	1578	Ariela Paulinapadilla segovia	2005
9875	0	0	0	1580	Sofiamazzucato	2005
9876	0	0	0	1581	Anna Sofiahaseth	2004
9877	0	0	0	1582	Dylangelber	2007
9878	0	0	0	1583	Kavyakhirwar	2003
9879	0	0	0	1583	Isabellaasenov	2005
9880	0	0	0	1585	Selyarakki	2004
9881	0	0	0	1586	Janelospanova	2006
9882	0	0	0	1587	Victoriachernova	2005
9883	0	0	0	1588	Mayaalexandrova	2005
9884	0	0	0	1589	Maddisonricardo	2004
9885	0	0	0	1590	Ximenacano	2004
9886	0	0	0	1591	Adrianacortes	2005
9887	0	0	0	1592	Talimalykhin	2004
9888	0	0	0	1593	Naroaaranzabal	2005
9889	0	0	0	1593	Ayabychenko	2005
9890	0	0	0	1595	Ornellade fazio	2006
9891	0	0	0	1595	Charlottetse	2004
9892	0	0	0	1595	Sydneystark	2007
9893	0	0	0	1595	Riokoumekuni	2007
9894	0	0	0	1595	Caterinaassi	2005
9895	0	0	0	1595	Eugenia Jazminceinos	2006
9896	0	0	0	1595	Mariaivankovic	2006

9897	0	0	0	1602	Hyunyeelee	2007
9898	0	0	0	1602	Jananiramesh	2004
9899	0	0	0	1602	Miayamakita	2007
9900	0	0	0	1605	Julietapalma	2005
9901	0	0	0	1606	Gresibajri	2006
9902	0	0	0	1607	Milenamayorova	2005
9903	0	0	0	1608	Nicolenikoli	2005
9904	0	0	0	1608	Emilylederer	2007
9905	0	0	0	1610	Jemmacave	2004
9906	0	0	0	1611	Adlalopez	2008
9907	0	0	0	1611	Zoyalevina	2006
9908	0	0	0	1613	Annelieke Saustrupsteenhuisen	2005
9909	0	0	0	1614	Candelaaparisi gonzalez-vallarir	2005
9910	0	0	0	1614	Anetapoborilova	2004
9911	0	0	0	1616	Soniabenlhassen	2004
9912	0	0	0	1617	Chiaratalleri	2005
9913	0	0	0	1617	Elsachaumette	2005
9914	0	0	0	1619	Julianasperb	2005
9915	0	0	0	1620	Jingyili	2003
9916	0	0	0	1621	Karolinagolda	2006
9917	0	0	0	1622	Daria Ioanapastoric	2004
9918	0	0	0	1623	Giannaoboniye	2006
9919	0	0	0	1624	Lizidolidze	2005
9920	0	0	0	1624	Kenzienguyen	2007
9921	0	0	0	1624	Sabrinalin	2007
9922	0	0	0	1624	E Wenwee	2006
9923	0	0	0	1624	Petrakonjikusic	2007
9924	0	0	0	1629	Lejla Hanagolubovic	2006
9925	0	0	0	1629	Frederikke Albertepape	2006
9926	0	0	0	1631	Mariabylina	2005
9927	0	0	0	1631	Deborah Li Junlim	2005
9928	0	0	0	1633	Maria Paularamos garcia	2006
9929	0	0	0	1634	Anna Lorielemongo toumbou	2003
9930	0	0	0	1635	Li Hsinlin	2005
9931	0	0	0	1635	GUADALUPEardao	2005
9932	0	0	0	1637	Leonardakajkic	2003
9933	0	0	0	1637	Alyssareguer	2006
9934	0	0	0	1637	Batipa Saidathponthy	2008
9935	0	0	0	1640	Deepshikasriram	2003
9936	0	0	0	1641	VALERIAguerrero	2004
9937	0	0	0	1641	Aliesiareva	2006
9938	0	0	0	1643	Neleniermann	2005
9939	0	0	0	1643	Charlottegisclon	2003
9940	0	0	0	1645	Martynamackiewicz	2006
9941	0	0	0	1646	Oliviasmith	2004
9942	0	0	0	1647	Paulaschaefer	2006
9943	0	0	0	1648	Sarahhartel	2006
9944	0	0	0	1648	Mirianagalietta	2005
9945	0	0	0	1650	Stefaniyapushkar	2007
9946	0	0	0	1651	Carakorhonen	2005

9947	0	0	0	1652	Scarlettehetherington	2008
9948	0	0	0	1653	Andjelijamarkovic	2006
9949	0	0	0	1654	Anna Lenaebster	2004
9950	0	0	0	1654	Alenakasatkina	2005
9951	0	0	0	1656	Norasvensson	2005
9952	0	0	0	1656	Swetasamanta	2005
9953	0	0	0	1658	Denizturan	2006
9954	0	0	0	1658	Luciakrivosudska	2003
9955	0	0	0	1660	Jieonoh	2005
9956	0	0	0	1661	Adelisakahriman	2005
9957	0	0	0	1661	Haruneshigemasu	2004
9958	0	0	0	1663	Dominikapodhajecka	2006
9959	0	0	0	1664	Taldekel	2003
9960	0	0	0	1665	Marieweckerle	2003
9961	0	0	0	1666	Wakanasonobe	2008
9962	0	0	0	1666	Selahstibbins	2004
9963	0	0	0	1666	Marelieraath	2006
9964	0	0	0	1666	Brookeblack	2007
9965	0	0	0	1666	Shannonlam	2008
9966	0	0	0	1666	Kenzael akili	2007
9967	0	0	0	1666	Gretazawels	2005
9968	0	0	0	1673	Silvanafigueroa teusa	2004
9969	0	0	0	1674	Audreyaulia	2007
9970	0	0	0	1674	Sinafackelmann	2004
9971	0	0	0	1676	Janarefaat	2005
9972	0	0	0	1677	Laurasparnina	2007
9973	0	0	0	1678	Alexandrapanagiotidou	2007
9974	0	0	0	1679	Annafrost	2006
9975	0	0	0	1679	Hanabecirovic novak	2003
9976	0	0	0	1679	Sama Hassanhindawy	2007
9977	0	0	0	1682	Alyssaperdomo	2004
9978	0	0	0	1682	Sofiaciobica	2005
9979	0	0	0	1684	Giuliadal pozzo	2005
9980	0	0	0	1684	Alexandra Karinpersson	2005
9981	0	0	0	1686	Rebekaantal	2005
9982	0	0	0	1687	Sofiiakrasikova	2003
9983	0	0	0	1687	Amybloch	2004
9984	0	0	0	1689	Cheridarley	2003
9985	0	0	0	1690	Poudima Anne-Marie Bre-Naam	2008
9986	0	0	0	1690	Barbarastraszewska	2006
9987	0	0	0	1690	Carlagrignac	2007
9988	0	0	0	1693	Zehrasuko	2005
9989	0	0	0	1694	Aissatouasaah	2005
9990	0	0	0	1694	Milenagevorgyan	2003
9991	0	0	0	1694	Monique Eradinechague tapind	2004
9992	0	0	0	1697	Lauradukic	2004
9993	0	0	0	1698	Natalieong	2007
9994	0	0	0	1699	Jelenagojkovic	2005
9995	0	0	0	1700	Dianayanotovskaya	2003
9996	0	0	0	1700	Erikadi muzio	2004

9997	0	0	0	1700	Erikadi muzio	2004
9998	0	0	0	1703	Summerlee	2007
9999	0	0	0	1704	Paulinewalpot	2004
10000	0	0	0	1705	Sarasadadinovic	2004
10001	0	0	0	1705	Jolenefernandes	2006
10002	0	0	0	1705	Ayse Bilgesuyurdakul	2004
10003	0	0	0	1708	Ireneacevedo vallecillo	2004
10004	0	0	0	1709	Kenzy Sherifelhamrawy	2006
10005	0	0	0	1709	Sabrinaolimjanova	2006
10006	0	0	0	1709	Tereziaperhacova	2004
10007	0	0	0	1709	Daryagneidina	2005
10008	0	0	0	1713	Beatricericci	2003
10009	0	0	0	1713	Lilly Rosetengbe	2004
10010	0	0	0	1713	Angelinamelgar escobar	2004
10011	0	0	0	1713	Sophiasappa	2005
10012	0	0	0	1713	Julialovqvist	2003
10013	0	0	0	1713	Annagrigoreva	2003
10014	0	0	0	1713	Jasaroka	2004
10015	0	0	0	1713	Midoricastillo meza	2003
10016	0	0	0	1713	Biborkanagy bekesi	2004
10017	0	0	0	1713	Sofiaberdecio flores	2005
10018	0	0	0	1713	Anjamenn	2005
10019	0	0	0	1713	Dayanaravelasco	2007
10020	0	0	0	1713	Eira Gabriellealegria	2006
10021	0	0	0	1713	Carolaschaudinn	2005
10022	0	0	0	1727	Victorialinkovski	2005
10023	0	0	0	1728	Niliatfi	2007
10024	0	0	0	1728	Ranabozdag	2005
10025	0	0	0	1730	Naa Anyemamckorley	2003
10026	0	0	0	1730	Korinaroso	2005
10027	0	0	0	1732	Ceciliemaeland	2006
10028	0	0	0	1733	Justina Mariagonzalez daniele	2005
10029	0	0	0	1734	Leticiamatuszewski	2005
10030	0	0	0	1735	Eliciakim	2005
10031	0	0	0	1736	Alizabaerrohim	2005
10032	0	0	0	1737	Naa shikamckorley	2007
10033	0	0	0	1737	Tracyampah	2007
10034	0	0	0	1739	Biancamolnar	2006
10035	0	0	0	1740	Julietungamirai	2005
10036	0	0	0	1741	Laurashelekhova	2007
10037	0	0	0	1742	Janakareisova	2006
10038	0	0	0	1742	Alinazepfel	2003
10039	0	0	0	1744	Lilienthieking	2006
10040	0	0	0	1745	Pauline Gretabruns	2005
10041	0	0	0	1746	Samanthavelasquez rivertte	2005
10042	0	0	0	1746	Michelamoore	2005
10043	0	0	0	1748	Fernandacastillo huerta	2003
10044	0	0	0	1748	Mariebrard vidot	2005
10045	0	0	0	1750	Kristinasamardzic	2003
10046	0	0	0	1751	Valeriegomez	2006

10047	0	0	0	1751	Mariashmakova	2007
10048	0	0	0	1751	Magdalenakaminska	2005
10049	0	0	0	1751	Karolinavalentova	2007
10050	0	0	0	1751	Amberdano	2008
10051	0	0	0	1751	Learupnjak	2004
10052	0	0	0	1751	Leanachanove	2004
10053	0	0	0	1751	Monicapadron castaldo	2005
10054	0	0	0	1751	Anastasia Aleksandranikolova	2005
10055	0	0	0	1760	Zhansayabakytzhan	2005
10056	0	0	0	1761	Lamijaavdic	2005
10057	0	0	0	1761	Rikokikawada	2006
10058	0	0	0	1761	Nataliazamora caprirolo	2003
10059	0	0	0	1761	Ireneriva	2004
10060	0	0	0	1761	Patricijacechanaviciute	2006
10061	0	0	0	1761	Sofiacorte real	2006
10062	0	0	0	1767	Elianakook	2007
10063	0	0	0	1768	Ayamimaehama	2004
10064	0	0	0	1769	Daryakornilova	2003
10065	0	0	0	1770	Jovanapetrovic	2007
10066	0	0	0	1770	Dariabutsko	2003
10067	0	0	0	1770	Aminasadikova	2003
10068	0	0	0	1770	Danijeladanilovic	2003
10069	0	0	0	1770	Zahraarthur	2004
10070	0	0	0	1770	Varvaradiachenko	2006
10071	0	0	0	1770	Natalialezniak	2004
10072	0	0	0	1770	Rozannevan der schyff	2005
10073	0	0	0	1770	Carolinaazadinho	2003
10074	0	0	0	1770	Daphneempetshi perricard	2008
10075	0	0	0	1770	Gaurimangaonkar	2006
10076	0	0	0	1770	Shravanisachin khawale	2005
10077	0	0	0	1770	Elizadunbar	2005
10078	0	0	0	1770	Mariabakhtina	2006
10079	0	0	0	1770	Ruvarashemagarira	2007
10080	0	0	0	1770	Stefanieschmid	2005
10081	0	0	0	1786	VALENTINAgarces vergara	2004
10082	0	0	0	1787	Jo-Yeechan	2004
10083	0	0	0	1787	Lilyaraiss	2005
10084	0	0	0	1787	Annaortiz gasco	2005
10085	0	0	0	1787	Vieradeeperm	2004
10086	0	0	0	1787	Micaelarosignuolo	2004
10087	0	0	0	1787	HIMARIhayashi	2006
10088	0	0	0	1787	Catalinarobert	2004
10089	0	0	0	1787	Danielaglanzer	2003
10090	0	0	0	1787	Sachisharma	2003
10091	0	0	0	1787	Evelinatakhmazyan	2006
10092	0	0	0	1787	Ksenija-Anavujacic	2004
10093	0	0	0	1787	Emelinehonfoga	2003
10094	0	0	0	1799	Malanniavashkevich	2004
10095	0	0	0	1799	Desireparn	2005
10096	0	0	0	1799	Nisancan	2007

10097	0	0	0	1802	Roselidaasumwa	2004
10098	0	0	0	1803	ROSALINE LUMakombo	2005
10099	0	0	0	1803	Camerondidion	2004
10100	0	0	0	1803	Jeninaishii	2003
10101	0	0	0	1803	Valeria Isabelteran bastidas	2005
10102	0	0	0	1807	Darahross	2004
10103	0	0	0	1808	Madeleinejoffe	2005
10104	0	0	0	1808	Seo Hyeonjang	2004
10105	0	0	0	1810	Teahchavez	2005
10106	0	0	0	1811	Stefaniyaanikina	2006
10107	0	0	0	1812	Chenel	2007
10108	0	0	0	1812	Inesbollecker	2006
10109	0	0	0	1812	Kudzai Erin Patiencechapepa	2008
10110	0	0	0	1812	Patriciacano	2003
10111	0	0	0	1812	Andreaperez bordon	2004
10112	0	0	0	1812	Juminkong	2004
10113	0	0	0	1812	Aditigulati	2007
10114	0	0	0	1819	Eminalagarija	2005
10115	0	0	0	1820	Sofiascatola	2006
10116	0	0	0	1820	Alexicleveland	2005
10117	0	0	0	1820	Emilydeming	2007
10118	0	0	0	1820	Odetapanasa	2008
10119	0	0	0	1820	Veronikakarimbayeva	2004
10120	0	0	0	1825	Mariamauad de souza	2004
10121	0	0	0	1826	Wanyizhang	2003
10122	0	0	0	1826	Verasekerina	2004
10123	0	0	0	1826	Sophiacedeno	2007
10124	0	0	0	1829	Dariadiatlova	2005
10125	0	0	0	1829	Kayleighlim	2007
10126	0	0	0	1829	Kimkuehbauer	2007
10127	0	0	0	1829	Nazli Denizsaka	2004
10128	0	0	0	1833	Bridgetmihulka	2007
10129	0	0	0	1834	Dariashubina	2006
10130	0	0	0	1835	Jianibrahim	2007
10131	0	0	0	1836	Polinakrumkachev	2006
10132	0	0	0	1837	Arianakolbassina	2007
10133	0	0	0	1837	Sophia Desireepiech	2003
10134	0	0	0	1837	Lianacammilleri	2004
10135	0	0	0	1837	Luciacassidy	2006
10136	0	0	0	1841	Sofiavillarroel llobet	2003
10137	0	0	0	1842	Mariamfarouk	2006
10138	0	0	0	1843	Milenaciocan	2008
10139	0	0	0	1843	Zeynepilker	2005
10140	0	0	0	1843	Martinapuvill	2005
10141	0	0	0	1843	Millie Maematthews	2003
10142	0	0	0	1847	Ha Yoonson	2007
10143	0	0	0	1848	Kenzyramy	2005
10144	0	0	0	1849	Eva Mariedesvignes	2008
10145	0	0	0	1849	Pimmadalim	2007
10146	0	0	0	1851	Krystallim	2007

10147	0	0	0	1852	Neneuemura	2004
10148	0	0	0	1852	Lerr Minlim	2004
10149	0	0	0	1852	Beatrizguerra	2006
10150	0	0	0	1852	Oliviacenter	2006
10151	0	0	0	1852	Katerynarudenko	2003
10152	0	0	0	1852	Libusehavlova	2005
10153	0	0	0	1852	Juliearmingol	2007
10154	0	0	0	1852	Joanmadi	2004
10155	0	0	0	1852	Emma Timmneergaard	2006
10156	0	0	0	1852	Luciagale	2008
10157	0	0	0	1862	Anyatkachyk	2006
10158	0	0	0	1862	Kaedetajima	2006
10159	0	0	0	1864	Yuen Tunglam	2005
10160	0	0	0	1864	Stefaniaghiorghita	2004
10161	0	0	0	1864	Amyleather	2003
10162	0	0	0	1867	Laviniatorselli	2004
10163	0	0	0	1867	Kesmatel tawil	2006
10164	0	0	0	1869	Gloriajovic	2007
10165	0	0	0	1869	Valeriaguerrero barberan	2005
10166	0	0	0	1871	Lola Emiliebean	2008
10167	0	0	0	1872	KESARINunsiri	2004
10168	0	0	0	1872	Kotisthamodak	2004
10169	0	0	0	1872	Milleechug	2006
10170	0	0	0	1875	Arisasabzezar	2005
10171	0	0	0	1875	Ghaliaguizani	2006
10172	0	0	0	1877	Pelincolak	2005
10173	0	0	0	1878	Maria Gabrielamejia	2005
10174	0	0	0	1878	Mayraarce gonzalez	2005
10175	0	0	0	1880	Valeriehutkova	2007
10176	0	0	0	1881	Alexiatrasca	2004
10177	0	0	0	1882	Conleyraidt	2005
10178	0	0	0	1882	Malakarafat	2004
10179	0	0	0	1882	Nuppupalm	2007
10180	0	0	0	1885	Rasitarkulnongdang	2005
10181	0	0	0	1885	Lourdes Belenluccatto	2005
10182	0	0	0	1885	Piapetelinsek	2006
10183	0	0	0	1885	Dianasmelansky	2003
10184	0	0	0	1885	Aleksandrakiseleva	2005
10185	0	0	0	1885	Nawalyoussef	2006
10186	0	0	0	1885	Wansaikasikampaiboon	2005
10187	0	0	0	1885	SUPANIDnopparith	2005
10188	0	0	0	1885	Adithiare	2003
10189	0	0	0	1885	Dogaakyurek	2003
10190	0	0	0	1885	esternankulange	2004
10191	0	0	0	1885	Saanvitak	2004
10192	0	0	0	1885	Malenacanon	2003
10193	0	0	0	1885	Tarasladakovic	2005
10194	0	0	0	1899	Amelierosadoro	2005
10195	0	0	0	1899	Vanshika Maria Vaizvasanth vai	2008
10196	0	0	0	1899	Sofiacarpi	2006

10197	0	0	0	1899	Sofiacarpi	2006
10198	0	0	0	1899	Vanshika Maria Vaizvasanth vai	2008
10199	0	0	0	1899	Anasavkovic	2006
10200	0	0	0	1899	Clairezhang	2007
10201	0	0	0	1906	NAGA ROSHNEarunkumar	2004
10202	0	0	0	1907	Habiba Mohamed Yousryahme	2007
10203	0	0	0	1908	Lok Yanwong	2006
10204	0	0	0	1909	Gabrielamiteva	2005
10205	0	0	0	1909	Victoria Sofiamadail	2007
10206	0	0	0	1909	Darianavera	2005
10207	0	0	0	1909	Ingazege	2005
10208	0	0	0	1909	Divabhatia	2006
10209	0	0	0	1914	Alicebolton	2005
10210	0	0	0	1914	Pamelabadillo hernandez	2006
10211	0	0	0	1914	Michaelasvobodova	2007
10212	0	0	0	1914	Fredrikarunstrom	2007
10213	0	0	0	1914	Dorotejapetrovic	2003
10214	0	0	0	1914	Sara Isabelsnyder	2003
10215	0	0	0	1920	Kseniasmirnova	2007
10216	0	0	0	1920	Ellaidzikowski	2006
10217	0	0	0	1920	Lounazoppas	2007
10218	0	0	0	1920	Kristinatai	2007
10219	0	0	0	1924	Victoriasavvides	2004
10220	0	0	0	1924	Vidhijani	2004
10221	0	0	0	1926	Tatiananovikova	2008
10222	0	0	0	1927	Lidialaskova	2003
10223	0	0	0	1927	Camila Moirataborga rollano	2004
10224	0	0	0	1927	Ivanacastillo hurtado	2007
10225	0	0	0	1930	Elenasamuelsson	2005
10226	0	0	0	1930	Mariabreidy	2006
10227	0	0	0	1930	Hailey Robyntroup	2005
10228	0	0	0	1933	Aidapasonova	2005
10229	0	0	0	1933	Sofikozeikidze	2004
10230	0	0	0	1933	Natsukiyamagami	2007
10231	0	0	0	1933	Loladecoux	2004
10232	0	0	0	1933	Kristynapaul	2007
10233	0	0	0	1933	Victoriakapcia	2006
10234	0	0	0	1933	Martahalbersztadt	2005
10235	0	0	0	1933	Priyanshichauhan	2005
10236	0	0	0	1941	Asimasazan	2005
10237	0	0	0	1941	Annatanaka	2003
10238	0	0	0	1941	Inesvandekerckhove	2007
10239	0	0	0	1941	Milicastosovic	2006
10240	0	0	0	1941	Mariataneva	2004
10241	0	0	0	1941	Heleneschnack	2007
10242	0	0	0	1941	Ekaterinanekhaeva	2004
10243	0	0	0	1948	Lararussiniello	2005
10244	0	0	0	1948	Georgia Andrianastrati	2005
10245	0	0	0	1948	Yanakuznetsova	2006
10246	0	0	0	1948	Patrycjaniewiadomska	2003

10247	0	0	0	1948	DIANA GABRIELAcraciun	2006
10248	0	0	0	1953	Emiliadurska	2004
10249	0	0	0	1953	Enija Paulaaploka	2005
10250	0	0	0	1955	Nikolmircheva	2004
10251	0	0	0	1955	Michelleconnor salazar	2004
10252	0	0	0	1957	Sheanna Sarahmutthumani	2006
10253	0	0	0	1958	Lillypiveteau	2007
10254	0	0	0	1958	Tatyanachsherbakova	2004
10255	0	0	0	1958	Emmavirtanen	2006
10256	0	0	0	1961	Nishithamaarka	2007
10257	0	0	0	1961	Anastasijaarsic	2006
10258	0	0	0	1963	Reginaurbiola armenta	2003
10259	0	0	0	1964	Ashiramurray	2004
10260	0	0	0	1964	Oliviacollins	2006
10261	0	0	0	1966	Klarasameliakova	2004
10262	0	0	0	1966	KISSMELcedano lapaix	2004
10263	0	0	0	1968	Editcosmo	2006
10264	0	0	0	1968	Nicolesifuentes	2006
10265	0	0	0	1968	Diana Sofializarazo	2006
10266	0	0	0	1971	Anaitarutyunyan	2003
10267	0	0	0	1971	Estefaniagarcia	2003
10268	0	0	0	1971	Noralouko	2006
10269	0	0	0	1971	Coumba Ben Mamadouniangad	2004
10270	0	0	0	1971	Wozukomdlulwa	2007
10271	0	0	0	1971	Yukikawa	2006
10272	0	0	0	1977	Matildeercoli	2006
10273	0	0	0	1977	Alissia Aleksandrakivi	2005
10274	0	0	0	1977	Aimeereynoso	2004
10275	0	0	0	1977	Alexiagonzalez galino	2007
10276	0	0	0	1981	Nicolbenesova	2004
10277	0	0	0	1982	Pimlaphatlim	2008
10278	0	0	0	1982	Juliananieto	2004
10279	0	0	0	1982	Ellao'malley	2003
10280	0	0	0	1982	Thaniya Saraigogulamanda	2008
10281	0	0	0	1982	Sonjazhenikhova	2008
10282	0	0	0	1982	Laismartins	2004
10283	0	0	0	1982	Liz Mariecalderon	2003
10284	0	0	0	1982	Camilamontoya galvis	2006
10285	0	0	0	1982	Cheyennehoukes	2003
10286	0	0	0	1982	Kamilaslovakova	2005
10287	0	0	0	1982	Lisa Liayssel	2003
10288	0	0	0	1982	Kamandafsharzadeh	2004
10289	0	0	0	1982	Livavevere	2005
10290	0	0	0	1982	Mahrazrazavian	2004
10291	0	0	0	1996	Angelinavoloshchuk	2007
10292	0	0	0	1996	Vaniadotse	2003
10293	0	0	0	1996	Daniela Andreearosca	2003
10294	0	0	0	1996	Juliaklimovicz	2003
10295	0	0	0	1996	Tainaternes cordeiro	2004
10296	0	0	0	1996	Arianagogulina	2008

10297	0	0	0	1996	Vaniadotse	2003
10298	0	0	0	2003	Livsumak	2004
10299	0	0	0	2004	Vanessa Ioananocaci	2007
10300	0	0	0	2004	Yanameermans	2005
10301	0	0	0	2004	Erikaweil	2004
10302	0	0	0	2007	Sarahejtmankova	2004
10303	0	0	0	2007	Juliaglagoleva	2005
10304	0	0	0	2007	Beatrizortega	2004
10305	0	0	0	2010	Alisa Biancaapostol	2005
10306	0	0	0	2011	Ritagefaizy	2003
10307	0	0	0	2011	Shuk Ying Haileyng	2005
10308	0	0	0	2011	Serenapereyra giubbani	2004
10309	0	0	0	2011	Lovisavalentinsson	2004
10310	0	0	0	2011	Carolinewernli	2003
10311	0	0	0	2011	Kittikaaeb iem	2004
10312	0	0	0	2011	Alinafedulova	2003
10313	0	0	0	2011	Saraconde	2007
10314	0	0	0	2011	Miokozaki	2004
10315	0	0	0	2011	Filippalarsson	2003
10316	0	0	0	2011	Noemieoliveras	2006
10317	0	0	0	2011	Nastaranchoobineh	2003
10318	0	0	0	2011	Sanviahluwalia	2004
10319	0	0	0	2011	Samatchayaorungrote	2005
10320	0	0	0	2011	Fridaweijerman	2004
10321	0	0	0	2011	Aida Mariamitrache	2004
10322	0	0	0	2011	Eszterkis czako	2006
10323	0	0	0	2011	Katieoliver	2006
10324	0	0	0	2011	Wirinruendhawin	2007
10325	0	0	0	2011	Selin Lidyasepken	2003
10326	0	0	0	2011	Kynadecruy	2005
10327	0	0	0	2011	Freja Bloevbjerg	2005
10328	0	0	0	2011	Celeste Juan Yingyeh	2008
10329	0	0	0	2011	Leanilsson	2008
10330	0	0	0	2011	Aliciamartial	2004
10331	0	0	0	2011	Lizmartinez	2005
10332	0	0	0	2011	Rehakipsang	2007
10333	0	0	0	2011	Namihorie	2004
10334	0	0	0	2011	Faithurasa	2006
10335	0	0	0	2011	Ichinohorikawa	2007
10336	0	0	0	2011	Xuanyuchen	2007
10337	0	0	0	2042	Kai Ning Chanyang	2008
10338	0	0	0	2042	Amirakaztugan	2006
10339	0	0	0	2042	Tajrajugic	2003
10340	0	0	0	2045	Audreynewman	2006
10341	0	0	0	2045	Havanakadi	2007
10342	0	0	0	2045	Susannabrass	2004
10343	0	0	0	2045	Farah Haithamkassem	2005
10344	0	0	0	2045	Lidiaencheva	2006
10345	0	0	0	2045	Emakovacevic	2006
10346	0	0	0	2051	Karolinalincer	2008

10347	0	0	0	2051	Beracah Confianceatana mbam	2005
10348	0	0	0	2051	Maria de la Pazalberto	2006
10349	0	0	0	2054	Sandra Isabelaarguelles	2007
10350	0	0	0	2054	Fernandasandoval	2006
10351	0	0	0	2054	Francesca Andreafeodorov	2003
10352	0	0	0	2054	Anastasiyaskorupska	2004
10353	0	0	0	2054	Siennaleeson	2005
10354	0	0	0	2054	Elenagrekul	2004
10355	0	0	0	2054	Maryamfarrouh	
10356	0	0	0	2061	Mariaeduarda lages	2004
10357	0	0	0	2061	Elisadavalos donoso	2003
10358	0	0	0	2063	Axellevan beurden	2007
10359	0	0	0	2063	Sajidsheeza	2006
10360	0	0	0	2063	FAITHameh	2005
10361	0	0	0	2063	Friederikenolte	2006
10362	0	0	0	2063	SALAMATharuna	2006
10363	0	0	0	2063	Kristinanordikyan	2005
10364	0	0	0	2063	Chloelomans	2006
10365	0	0	0	2063	Melinechaumeil	2004
10366	0	0	0	2063	Aliceklinteby	2004
10367	0	0	0	2063	Sai Harshitaaadivi	2005
10368	0	0	0	2063	Blancapico navarro	2005
10369	0	0	0	2063	Polalangiewicz	2005
10370	0	0	0	2063	Lilypradkin	2006
10371	0	0	0	2063	Zahrasuleman	2004
10372	0	0	0	2077	Nataliepaterova	2005
10373	0	0	0	2078	Serenahatakka	2005
10374	0	0	0	2078	Mara Sophieburger	2005
10375	0	0	0	2078	Sofiyavolkova	2006
10376	0	0	0	2078	Aleksandrastevanovic	2007
10377	0	0	0	2078	Shannonbovy	2006
10378	0	0	0	2078	Saniya Manojmasand	2003
10379	0	0	0	2078	Adajane dealcantara	2005
10380	0	0	0	2078	Sophiade michelle	2004
10381	0	0	0	2078	Marielapluutus	2004
10382	0	0	0	2078	Luciarizza	2003
10383	0	0	0	2078	Yelenakelleher	2006
10384	0	0	0	2078	Juliettetrunet	2007
10385	0	0	0	2078	Diyatamse	2006
10386	0	0	0	2091	Kristannagjomakaj	2005
10387	0	0	0	2091	Milabadics	2004
10388	0	0	0	2091	Amikebotha	2006
10389	0	0	0	2094	Naomiwidiaman	2004
10390	0	0	0	2094	Alexakaratancheva	2007
10391	0	0	0	2094	Doramiskovic	2007
10392	0	0	0	2094	Seo Yeonpark	2003
10393	0	0	0	2094	Viktoriiasamarina	2007
10394	0	0	0	2099	Tsvetaradeva	2004
10395	0	0	0	2099	Emmakast	2006
10396	0	0	0	2099	Feyza Ebraraydin	2004

10397	0	0	0	2099	Feyza Ebraraydin	2004
10398	0	0	0	2099	Fernandarain contreras	2004
10399	0	0	0	2104	Klaudiehutkova	2004
10400	0	0	0	2104	Miasarcev	2005
10401	0	0	0	2104	Valentinaferrarini	2003
10402	0	0	0	2107	Marijanamajetic	2004
10403	0	0	0	2107	Kashmiramanohar	2003
10404	0	0	0	2109	Eun Bilee	2005
10405	0	0	0	2109	Sahanasanjeev	2006
10406	0	0	0	2109	Anastasiakavounov	2006
10407	0	0	0	2109	Ameliatye	2005
10408	0	0	0	2109	Elinaxler	2005
10409	0	0	0	2109	Miruthula Mahalakshmikrishna	2004
10410	0	0	0	2109	Carolinaguerrero	2007
10411	0	0	0	2109	Jordana Victoriasalcedo franklin	2006
10412	0	0	0	2109	Janapremovic	2007
10413	0	0	0	2109	Ameiasorey	2007
10414	0	0	0	2119	Sreenidhi Reddyamireddy	2005
10415	0	0	0	2119	Vitoriamaximiliano	2005
10416	0	0	0	2119	Diana Vanessadiaz daza	2007
10417	0	0	0	2119	Valentinavargas	2007
10418	0	0	0	2119	Viktorialorentzon	2005
10419	0	0	0	2119	Dariaraimondo	2007
10420	0	0	0	2119	Sultanamavric	2007
10421	0	0	0	2119	Tiadarrag	2007
10422	0	0	0	2119	EMMAmoratalla sanz	2004
10423	0	0	0	2119	Itzarogel	2004
10424	0	0	0	2119	Ivajankovic	2006
10425	0	0	0	2119	Gretapetrillo	2007
10426	0	0	0	2119	Celestinacedillo - vayson de pra	2006
10427	0	0	0	2119	Sukhivenkatesh	2007
10428	0	0	0	2119	Patriciawamba	2004
10429	0	0	0	2119	Irenefuster hidalgo	2006
10430	0	0	0	2119	Sofiacanto hurtado	2005
10431	0	0	0	2119	Antoniade melo ferrarini	2005
10432	0	0	0	2119	MARTINAacebedo bonocore	2006
10433	0	0	0	2138	Meghankumria	2004
10434	0	0	0	2138	DANIELAchaves	2004
10435	0	0	0	2140	Sabrinasanchez	2003
10436	0	0	0	2140	Andrea Marlenelandaeta quinte	2007
10437	0	0	0	2140	Elenjantacova	2005
10438	0	0	0	2140	Jasmineluse	2006
10439	0	0	0	2140	Marinahristova	2003
10440	0	0	0	2140	Camilaibarra paolone	2003
10441	0	0	0	2146	Laragrulovic	2003
10442	0	0	0	2147	Chelseastergiopoulos	2006
10443	0	0	0	2147	Ivadaneva	2005
10444	0	0	0	2147	Anastasiyadyadchenko	2004
10445	0	0	0	2147	Ekaterinalazareva	2005
10446	0	0	0	2147	Shirachiche	2004

10447	0	0	0	2152	Louchalard	2005
10448	0	0	0	2152	Chems Dohazeghlouli	2004
10449	0	0	0	2152	Golnesaakbari	2007
10450	0	0	0	2152	Nikolanikolajeva	2005
10451	0	0	0	2152	Defneerbakan	2006
10452	0	0	0	2152	Isabellagibson	2006
10453	0	0	0	2152	Hannavasconez teran	2004
10454	0	0	0	2152	Annalisesmith	2005
10455	0	0	0	2152	Sofyaostrovskaya	2003
10456	0	0	0	2152	Sofiyalebedeva	2003
10457	0	0	0	2152	Bella-Gracemelov	2004
10458	0	0	0	2152	Sofjanalivajko	2006
10459	0	0	0	2152	Sarahsuljadzic	2006
10460	0	0	0	2152	Anamarijakovacevic	2003
10461	0	0	0	2152	Karolinaskowronska	2004
10462	0	0	0	2152	Hanasoltani	2007
10463	0	0	0	2152	Aldenrussell	2007
10464	0	0	0	2152	Sarahibrahimagic	2004
10465	0	0	0	2170	Karinadunda	2005
10466	0	0	0	2170	Cherysh Endurancehenry	2005
10467	0	0	0	2172	Andreeapavel	2004
10468	0	0	0	2172	Theadonarski	2004
10469	0	0	0	2172	Karina Ioanafilipescu	2008
10470	0	0	0	2172	Clarasobius	2004
10471	0	0	0	2172	Kimmartin	2007
10472	0	0	0	2172	Ana Paolamercado cervantes	2004
10473	0	0	0	2172	Leandranizetic	2003
10474	0	0	0	2179	Flaviascata	2006
10475	0	0	0	2179	Valeriiashvetsova	2007
10476	0	0	0	2179	Theodoradegelow	2004
10477	0	0	0	2179	Leajakic	2004
10478	0	0	0	2179	Ninakwakman	2005
10479	0	0	0	2179	Polinazaytseva	2006
10480	0	0	0	2179	Praewajongjit	2004
10481	0	0	0	2179	Kiramalakhova	2007
10482	0	0	0	2179	Terezakrenkova	2004
10483	0	0	0	2179	Lrunralisebusadee	2005
10484	0	0	0	2179	Nataliestasny	
10485	0	0	0	2179	Beathrizmaciel	2004
10486	0	0	0	2179	Tamannatakoria	2006
10487	0	0	0	2179	Maria Cristinapurizaca	2006
10488	0	0	0	2179	Sofiabeltran arratia	2005
10489	0	0	0	2179	Min Youngpark	2006
10490	0	0	0	2179	Kristynakostelna	2004
10491	0	0	0	2179	Phoebepeus	2004
10492	0	0	0	2179	Papitchayaisaro	2007
10493	0	0	0	2179	Thaismitlmeier rollano	2005
10494	0	0	0	2179	Deborasamanovica	2006
10495	0	0	0	2179	Deepalakshmivanaraja	2003
10496	0	0	0	2179	Kamolaamanova	2005

10497	0	0	0	2179	Aleksandrabortnichek	2008
10498	0	0	0	2179	Kristynakostelna	2004
10499	0	0	0	2179	Praifachanchaimongkon	2006
10500	0	0	0	2179	Inesrodrigues	2004
10501	0	0	0	2179	Angelinablinova	2004
10502	0	0	0	2179	Thaismitlmeier rollano	2005
10503	0	0	0	2179	Deborasamanovica	2006
10504	0	0	0	2179	Phoebepeus	2004
10505	0	0	0	2179	Hitakamya Singhnarwal	2005
10506	0	0	0	2179	Isabellalynch	2003
10507	0	0	0	2179	Antonellamartins xavier	2005
10508	0	0	0	2179	Athenakomodromou	2004
10509	0	0	0	2179	Kamolaamanova	2005
10510	0	0	0	2179	Raeganpine	2003
10511	0	0	0	2179	Cheuk Yu Rachelkan	2007
10512	0	0	0	2179	Angelinada silva guggenbuehler	2005
10513	0	0	0	2179	Rotemcohen	2006
10514	0	0	0	2179	Flaviascata	2006
10515	0	0	0	2179	Audreymoutama	2004
10516	0	0	0	2179	Ghaidajeribi	2004
10517	0	0	0	2179	Theodoradegelow	2004
10518	0	0	0	2179	Eminacabro	2005
10519	0	0	0	2179	Sofiaarellano	2003
10520	0	0	0	2179	Nicoleteodosescu	2003
10521	0	0	0	2179	Ninakwakman	2005
10522	0	0	0	2179	Terezakrenkova	2004
10523	0	0	0	2179	Riadernikovic	2005
10524	0	0	0	2179	Hee Wonju	2008
10525	0	0	0	2179	Papitchayaisaro	2007
10526	0	0	0	2179	AhKyungkim	2008
10527	0	0	0	2179	Kahyeonhan	2007
10528	0	0	0	2179	Laylasakr	2007
10529	0	0	0	2179	Mariafedenko	2005
10530	0	0	0	2179	Seromy Valentinasicha ticona	2007
10531	0	0	0	2179	Jillian Louiseroa	2003
10532	0	0	0	2179	Zixin Xinxinzhu	2007
10533	0	0	0	2179	Emilse Lujanruiz	2004
10534	0	0	0	2179	Tanakagarikai	2006
10535	0	0	0	2179	Mirabellebrettkelly	2003
10536	0	0	0	2179	Mahinarangiwarren	2007
10537	0	0	0	2179	Danna Katalinaquintero cubillos	2004
10538	0	0	0	2179	Arielmadatali	2005
10539	0	0	0	2179	Carolinabohrer martins	2007
10540	0	0	0	2179	Ashtyncason	2006
10541	0	0	0	2179	Nataliestasny	
10542	0	0	0	2179	Zofiastrzyzewska	2006
10543	0	0	0	2179	Dafnekremers	2005
10544	0	0	0	2179	OURANIAsfrintzeri	2005
10545	0	0	0	2179	Akanshadhuwe	2008
10546	0	0	0	2179	Anitamohrekesh	2007

10547	0	0	0	2179	Fatmamornagui	2004
10548	0	0	0	2179	Tianyaozhang	2007
10549	0	0	0	2179	Sarayyli-piipari	2005
10550	0	0	0	2179	Rirakosaka	2008
10551	0	0	0	2179	MERIEMmakhlouf	2007
10552	0	0	0	2179	Trinityang	2008
10553	0	0	0	2179	Katerinashabashkevich	2008
10554	0	0	0	2179	Christashamcneil	2006
10555	0	0	0	2179	Alliebittner	2006
10556	0	0	0	2179	Macarenacannoni	2003
10557	0	0	0	2179	Vanessa Kabukieakuaku	2002
10558	0	0	0	2179	Lawrenciaaghoghorbia	2003
10559	0	0	0	2264	Annaperelman	2007
10560	0	0	0	2264	Silviaalejo	2005
10561	0	0	0	2264	Rinnahokonjo	2007
10562	0	0	0	2264	LAURAvillanueva morillo	2005
10563	0	0	0	2264	Monikatemkova	2003
10564	0	0	0	2264	Mansivadyala	2006
10565	0	0	0	2264	Cayetanagay	2006
10566	0	0	0	2264	Eleejahinisan	2008
10567	0	0	0	2264	Saekaneko	2007
10568	0	0	0	2264	Deborahabiodun	2004
10569	0	0	0	2264	Ekaterinastrizhkova	2004
10570	0	0	0	2264	Elisabethbirkevold	2005
10571	0	0	0	2264	Clairean	2007
10572	0	0	0	2264	Ziyigao	2005
10573	0	0	0	2264	Ellasowman	2006
10574	0	0	0	2264	Hinatachiba	2006
10575	0	0	0	2264	Lou Agatheetienne	2007
10576	0	0	0	2264	Anastasiakouchnareva	2003
10577	0	0	0	2264	Leavon kozierowski	2006
10578	0	0	0	2264	Josefinefalster	2005
10579	0	0	0	2264	Kailabarksdale	2004
10580	0	0	0	2264	Elenabocchi	2004
10581	0	0	0	2264	Shubhangee Laxmishah	2006
10582	0	0	0	2264	Anastasiasizykh	2005
10583	0	0	0	2264	Margauxtulet-jost	2006
10584	0	0	0	2264	Yaqihuang	2004
10585	0	0	0	2264	Tejatirunelveli	2004
10586	0	0	0	2264	Giadarossi	2005
10587	0	0	0	2264	Jadefeneon	2003
10588	0	0	0	2264	Maddalenagiordano	2003
10589	0	0	0	2294	Tak Sumfung	2008
10590	0	0	0	2294	Zehracanbaz	2003
10591	0	0	0	2294	CECILIAmanitto	2005
10592	0	0	0	2294	Ruthroura llaverias	2006
10593	0	0	0	2294	Laurerazet	2004
10594	0	0	0	2294	Valeria Amorocho Hernandezar	2007
10595	0	0	0	2294	Beyzayaniklar	2004
10596	0	0	0	2294	Ineshomberg fernandez	2006

10597	0	0	0	2302	Emmaanderson	2005
10598	0	0	0	2302	Amelsammari	2004
10599	0	0	0	2302	Milanadejanovic	2007
10600	0	0	0	2302	Luisa Silvestre da Rosasilvestre	2006
10601	0	0	0	2302	Raquel Alexasanchez elizondo	2005
10602	0	0	0	2307	Viktoriyasankiyska	2005
10603	0	0	0	2307	Vihanashresta	2004
10604	0	0	0	2307	Nataliecinkova	2005
10605	0	0	0	2307	Raja Sarvagnyakilaru	2007
10606	0	0	0	2307	Elenaondobo	2006
10607	0	0	0	2307	Ruvarashe Lyndahshoko	2005
10608	0	0	0	2307	Emilialehtinen	2007
10609	0	0	0	2307	Tina Precioustankia asongalem	2005
10610	0	0	0	2307	Camilacorrales	2004
10611	0	0	0	2307	Valerialazo	2004
10612	0	0	0	2307	Sabrina Alejandrasolis encinas	2005
10613	0	0	0	2307	Ngai Fancheung	2006
10614	0	0	0	2307	Anastasiabertacchi	2006
10615	0	0	0	2307	Zuzanna Izabelawitkowska	2005
10616	0	0	0	2321	Seo Hyeonpark	2004
10617	0	0	0	2322	Anna Mariatottrup	2005
10618	0	0	0	2322	Polinastarkova	2007
10619	0	0	0	2322	Aimeehulett	2004
10620	0	0	0	2322	Celineabsawi	2007
10621	0	0	0	2322	Jaelschwarz	2004
10622	0	0	0	2322	Andrasutherland	2004
10623	0	0	0	2322	Mariameltaweel	2006
10624	0	0	0	2322	Kyrashetty	2005
10625	0	0	0	2322	Nicolecarpenter	2003
10626	0	0	0	2322	Hui Onchoi	2005
10627	0	0	0	2322	Anamariakurtsikashvili	2005
10628	0	0	0	2322	Taleiatuatao	2005
10629	0	0	0	2322	Francescasimpson	2007
10630	0	0	0	2322	Jessicamatthews	2004
10631	0	0	0	2322	isabellaochoa	2005
10632	0	0	0	2337	Ameliatarasova	2003
10633	0	0	0	2338	Georgiaplunkett	2004
10634	0	0	0	2338	Aurorapedwell	2005
10635	0	0	0	2338	Rosiespeed	2006
10636	0	0	0	2338	Sohinisanjay mohanty	2008
10637	0	0	0	2338	Paularodriguez gregoris	2004
10638	0	0	0	2338	Lisettealjas	2006
10639	0	0	0	2338	dianaivanikhina	2003
10640	0	0	0	2338	Neliasolovei	2006
10641	0	0	0	2338	Rachaelphelan	2004
10642	0	0	0	2338	Shahdhabib	2006
10643	0	0	0	2338	Prakritishrestha	2004
10644	0	0	0	2349	Janadenisenko	2007
10645	0	0	0	2349	Estellenajean	2003
10646	0	0	0	2349	Stsefaniyashabanava	2004

10647	0	0	0	2349	Lunaivkovic	2006
10648	0	0	0	2349	Nicolaschoeman	2007
10649	0	0	0	2349	Sarahmildren	2008
10650	0	0	0	2355	Sasafelixova	2006
10651	0	0	0	2355	Jeannememe	2008
10652	0	0	0	2355	Valerygomez	2006
10653	0	0	0	2355	Saskiasoome	2005
10654	0	0	0	2355	Sofijazarina	2006
10655	0	0	0	2355	Sarvinbazgir	2006
10656	0	0	0	2355	Milica Vasilikimilosavljevic	2005
10657	0	0	0	2355	Seinakishimoto	2006
10658	0	0	0	2355	PAMELAchazaro croda	2003
10659	0	0	0	2355	Hetanuutinen	2003
10660	0	0	0	2355	Jaeahlee	2007
10661	0	0	0	2355	Sofiajohnson	2004
10662	0	0	0	2355	Mishelyfernandez	2005
10663	0	0	0	2355	Anarellabrito	2007
10664	0	0	0	2355	Sofia Mantuanibuhr	2004
10665	0	0	0	2355	Arghavansepehr	2007
10666	0	0	0	2355	UTAasakura	2006
10667	0	0	0	2355	Alexamartinez mendoza	2005
10668	0	0	0	2355	Alexandradaradur	2005
10669	0	0	0	2355	Mariakalyakina	2006
10670	0	0	0	2355	Sierraausman	2005
10671	0	0	0	2355	Sachenka Valeskarios romero	2007
10672	0	0	0	2377	Isha Shreeshah	2005
10673	0	0	0	2377	Mariaantonova	2005
10674	0	0	0	2377	Sofiabenhaddouch	2006
10675	0	0	0	2377	Gracebiot	2005
10676	0	0	0	2377	Wing Yan Emilychan	2004
10677	0	0	0	2377	Rosearnastairmand	2005
10678	0	0	0	2377	Aidaeissa	2005
10679	0	0	0	2377	Berklie Simmeringsimmering	2007
10680	0	0	0	2385	Yvonnesubev	2005
10681	0	0	0	2385	Farahheddar	2006
10682	0	0	0	2385	Kellyhope	2004
10683	0	0	0	2385	Farahhassan assad hassan abda	2008
10684	0	0	0	2385	Clarissablomqvist	2004
10685	0	0	0	2385	Giovannapiedemonte	2005
10686	0	0	0	2385	Annakvizhinadze	2004
10687	0	0	0	2385	Paulinavillegas	2005
10688	0	0	0	2385	Mariageorgiadou	2003
10689	0	0	0	2385	Amelijamatjunina	2003
10690	0	0	0	2385	Gaeunlee	2004
10691	0	0	0	2385	Bonniesmeltz	2007
10692	0	0	0	2397	Ruramainyamakura	2006
10693	0	0	0	2397	Yuliyaklimova	2006
10694	0	0	0	2397	Anastasiiasergienko	2005
10695	0	0	0	2397	Silvia Mariacostache	2003
10696	0	0	0	2397	Saniyasingh	2004

10697	0	0	0	2397	Prishabaliga	2003
10698	0	0	0	2397	Anastasiiasergienko	2005
10699	0	0	0	2397	Silvia Mariacostache	2003
10700	0	0	0	2397	Yuliyaklimova	2006
10701	0	0	0	2397	Gerdareinaru	2003
10702	0	0	0	2397	Alinamokrynska	2006
10703	0	0	0	2397	Carlikruger	2006
10704	0	0	0	2397	Saniyasingh	2004
10705	0	0	0	2397	Ruramainyamakura	2006
10706	0	0	0	2411	Martinecarroll	2004
10707	0	0	0	2411	Tak Yufung	2003
10708	0	0	0	2411	Sarafina Oliviahansen	2005
10709	0	0	0	2411	Cristinetchanichev	2003
10710	0	0	0	2411	Nataliapapshev	2006
10711	0	0	0	2411	Thi Lienmouret	2008
10712	0	0	0	2411	Claudia Alexaalvarado lara	2004
10713	0	0	0	2411	Annawise	2005
10714	0	0	0	2411	Sabiramohamed	2003
10715	0	0	0	2411	Ximenaku	2004
10716	0	0	0	2411	Lauraozola	2006
10717	0	0	0	2411	Josefinehardelin	2007
10718	0	0	0	2411	Laurenseye	2007
10719	0	0	0	2411	Amna Aliqayum	2008
10720	0	0	0	2411	Polinasleptsova	2007
10721	0	0	0	2411	Leilaepstein	2003
10722	0	0	0	2411	Biancadumitru	2005
10723	0	0	0	2411	Viktoriezidelova	2006
10724	0	0	0	2411	Agamgamliel	2005
10725	0	0	0	2411	Zeyneppolat	2008
10726	0	0	0	2411	Andreavargas lau	2005
10727	0	0	0	2411	Paulinelerminiaux	2007
10728	0	0	0	2411	Malikaamirgalieva	2007
10729	0	0	0	2411	Joshua Kelly Farnyiyukbuin	2003
10730	0	0	0	2411	Jilhassinger	2007
10731	0	0	0	2411	Chrystallopez	2008
10732	0	0	0	2411	Aminakadic	2005
10733	0	0	0	2411	Rachellee	2006
10734	0	0	0	2411	Ariadnazegarra	2004
10735	0	0	0	2411	Isabellawong	2007
10736	0	0	0	2411	Gretchenwolfe	
10737	0	0	0	2411	Shimreenahamed	2005
10738	0	0	0	2411	Larabakhaya	2003
10739	0	0	0	2411	Julianafigueroa teusa	2006
10740	0	0	0	2411	Zuzanatrebunova	2004
10741	0	0	0	2411	Adelagemzicka	2005
10742	0	0	0	2411	Mariapinto	2006
10743	0	0	0	2411	Ameliaciesielczyk	2006
10744	0	0	0	2411	Yariange Angelibello barrera	2006
10745	0	0	0	2411	Giuliabizzari	2006
10746	0	0	0	2411	Elaine Alfonsinamendoza ortiz	2004

10747	0	0	0	2411	Durubekaroglu	2005
10748	0	0	0	2411	Maitree Diksharout	2004
10749	0	0	0	2411	Alexandrawarchausky	2004
10750	0	0	0	2411	Kanitaselimanovic	2004
10751	0	0	0	2411	Marimovafarida	2004
10752	0	0	0	2457	Anagutierrez	2004
10753	0	0	0	2457	Sonia Rae Jia Enloke	2008
10754	0	0	0	2457	Juliabortoluzzi bianchin bordign	2005
10755	0	0	0	2457	Daryazubtsouskaya	2003
10756	0	0	0	2457	Majapawelska	2008
10757	0	0	0	2457	Claudinaaiassa	2003
10758	0	0	0	2457	Katesmith	2006
10759	0	0	0	2457	Anamariznuderl	2005
10760	0	0	0	2457	Ximenagarzon martinez	2005
10761	0	0	0	2457	Riamehta	2004
10762	0	0	0	2457	Siddhikhot	2005
10763	0	0	0	2457	Reneedorval	2005
10764	0	0	0	2457	Beatrizcosta	2003
10765	0	0	0	2457	Laratorres	2003
10766	0	0	0	2457	Kristinanikiforova	2005
10767	0	0	0	2457	Nemha Sarahkispotta	2007
10768	0	0	0	2457	Cirkelinelundberg	2003
10769	0	0	0	2457	Scarlettsmeltz	2008
10770	0	0	0	2457	Danielaporges	2003
10771	0	0	0	2457	Isabellawalker	2008
10772	0	0	0	2457	Mollyrobinson	2007
10773	0	0	0	2457	Ananyavartak	2003
10774	0	0	0	2479	Amysucha	2007
10775	0	0	0	2479	Faithsadgrove	2005
10776	0	0	0	2479	Mia Sofiagarcia escorcia	2005
10777	0	0	0	2479	Evasegato	2006
10778	0	0	0	2479	Petramirkovic	2006
10779	0	0	0	2479	Izabellepersson	2003
10780	0	0	0	2479	Stephanieyakoff	2005
10781	0	0	0	2479	Tia Bonitajakupovic-kljako	2006
10782	0	0	0	2479	Steffyjullian	2003
10783	0	0	0	2479	Learibourdouille	2006
10784	0	0	0	2479	Bentebierma	2004
10785	0	0	0	2490	Rosiesterk	2003
10786	0	0	0	2490	Vladislavagerasenko	2004
10787	0	0	0	2490	Ioana Sanzianalung	2005
10788	0	0	0	2490	Cecilie Agerbekfridthjof	2007
10789	0	0	0	2494	Sofiahadab	2007
10790	0	0	0	2494	Zaaraomar	2004
10791	0	0	0	2494	Norakhediri	2005
10792	0	0	0	2494	Maria Luisa Cusmabloot	2005
10793	0	0	0	2494	Kushivenkatesh	2005
10794	0	0	0	2494	Varvarabernovich	2006
10795	0	0	0	2494	AMANY JAURESSE NELLYkouam	2004
10796	0	0	0	2494	Ajabroqi	2005

10797	0	0	0	2494	Ayaeddaoudi	2006
10798	0	0	0	2494	MARIA SOFIAvaldespino lopez	2003
10799	0	0	0	2494	Anastasijastojanovska	2003
10800	0	0	0	2494	Maria Luisa Cusmabloot	2005
10801	0	0	0	2494	Letiziamigani	2004
10802	0	0	0	2494	Martinagenis salas	2006
10803	0	0	0	2494	Tamiranashe Hopewellshoko	2007
10804	0	0	0	2494	Franziskakremerskothen	2006
10805	0	0	0	2494	Andreapineda	2004
10806	0	0	0	2494	Varvarabernovich	2006
10807	0	0	0	2494	Norakhediri	2005
10808	0	0	0	2494	Anoushkasingh	2005
10809	0	0	0	2494	Sofiahadab	2007
10810	0	0	0	2494	Thitareetumthong	2003
10811	0	0	0	2494	Kylasteyn	2004
10812	0	0	0	2494	Yee Tingchan	2005
10813	0	0	0	2494	Brigittegomez	2007
10814	0	0	0	2494	Maria Gaiameneguzzo	2005
10815	0	0	0	2494	Mafaldaalmeida	2006
10816	0	0	0	2494	Pak Yingkok	2006
10817	0	0	0	2494	Zaaraomar	2004
10818	0	0	0	2494	Hamsmetwali	2006
10819	0	0	0	2494	Alexandragombitova	2004
10820	0	0	0	2494	Mariiapetrova	2005
10821	0	0	0	2494	Felinevan eijkelenburg	2005
10822	0	0	0	2494	AMANY JAURESSE NELLYkouam	2004
10823	0	0	0	2494	Medinayessenaliyeva	2006
10824	0	0	0	2494	Anghelinamocanu	2006
10825	0	0	0	2494	chakiradermane	2003
10826	0	0	0	2494	Giulianacunza	2006
10827	0	0	0	2494	Eliskatichackova	2006
10828	0	0	0	2494	Ivanna Carolinade gregorio per	2006
10829	0	0	0	2494	Miapopovic	2004
10830	0	0	0	2494	Riomaeda	2005
10831	0	0	0	2494	Sara Sofiasanchez molano	2007
10832	0	0	0	2494	Seo Youngkim	2004
10833	0	0	0	2494	Mediamesgary	2004
10834	0	0	0	2494	Asalsadat kiaei	2008
10835	0	0	0	2494	Alexiapatru	2006
10836	0	0	0	2494	Jayabertossa	2007
10837	0	0	0	2494	Annastewart	2005
10838	0	0	0	2494	Rocioamoros navarro	2006
10839	0	0	0	2494	Florafarkaslaki hints	2007
10840	0	0	0	2494	Ajabroqi	2005
10841	0	0	0	2494	Radharaja	2005
10842	0	0	0	2494	Sofia Mariapapachristou	2006
10843	0	0	0	2494	Kushivenkatesh	2005
10844	0	0	0	2494	Oliviagiotis	2007
10845	0	0	0	2494	Serenagar	2003
10846	0	0	0	2494	Leilaakhmetova	2007

10847	0	0	0	2494	Milanatomashevich	2007
10848	0	0	0	2494	Toffeeghorab	2007
10849	0	0	0	2494	Raquel Alejandrasantos marmo	2005
10850	0	0	0	2494	Safienboulonois	2006
10851	0	0	0	2494	kokomiema	2007
10852	0	0	0	2494	EIRINI MARIAtsakiri	2007
10853	0	0	0	2494	Shalomsalvi	2003
10854	0	0	0	2494	Nataliaalvarez canedo	2004
10855	0	0	0	2494	Natalidavidova	2006
10856	0	0	0	2494	Emmadvorackova	2006
10857	0	0	0	2494	Ellyleis	2007
10858	0	0	0	2494	Iidaheinonen	2007
10859	0	0	0	2494	Alyssaahn	2006
10860	0	0	0	2494	Aleksandrakushnareva	2005
10861	0	0	0	2494	Melissaduarte	
10862	0	0	0	2494	Sophia tran ngoc nhihuynh	2006
10863	0	0	0	2494	Anasantisteban	2006
10864	0	0	0	2494	Katherinedibbs	2004
10865	0	0	0	2494	Nayumasubba	2003
10866	0	0	0	2571	Warapornmuangpho	2005
10867	0	0	0	2571	Tuhinasambhus	2006
10868	0	0	0	2571	Summerhupfeld	2008
10869	0	0	0	2571	Abide Shelabeouzi	2007
10870	0	0	0	2571	Alexandragerasimova	2006
10871	0	0	0	2571	Sarahcopley	2007
10872	0	0	0	2571	Valentinasuvirdjonkova	2008
10873	0	0	0	2571	Nadezhdapetkova	2004
10874	0	0	0	2571	Marijadjurasic	2003
10875	0	0	0	2571	STELLAde blende	2007
10876	0	0	0	2571	Emmazheng	2005
10877	0	0	0	2571	Maria Ceciliagomez yanes	2004
10878	0	0	0	2571	Kasandra Leonidjekic	2004
10879	0	0	0	2571	Ananyamanchanda	2004
10880	0	0	0	2571	Radhika Rajeshmahajan	2004
10881	0	0	0	2571	Cameliahadab	2007
10882	0	0	0	2587	Xeniaeremenko	2005
10883	0	0	0	2587	Isabellatemenugov	2006
10884	0	0	0	2587	Alizeyarwood	2007
10885	0	0	0	2587	Polinakratyuk	2007
10886	0	0	0	2587	Charlottepikkaart	2006
10887	0	0	0	2587	Ji Eunlee	2005
10888	0	0	0	2587	Andrea Alejandrada costa rojas	2005
10889	0	0	0	2594	Sofiasrebnick	2006
10890	0	0	0	2594	Andrejagagilaite	2005
10891	0	0	0	2594	Evrayildirim	2004
10892	0	0	0	2594	Marina Nonato Figueiredoribei	2005
10893	0	0	0	2594	Maiawarlies paredes	2005
10894	0	0	0	2594	Natalia Gabrielanavarro ramire	2003
10895	0	0	0	2594	Evelinavaisberg	2006
10896	0	0	0	2594	Kiarazabkova	2007

10897	0	0	0	2594	Marina Nonato Figueiredoribei	2005
10898	0	0	0	2594	Susannahsu	2007
10899	0	0	0	2594	Albinakakenova	2008
10900	0	0	0	2594	Evelinavaisberg	2006
10901	0	0	0	2594	Sooinlee	2006
10902	0	0	0	2594	Savannasalahuddin-wright	2008
10903	0	0	0	2594	Nicolemolenberg	2007
10904	0	0	0	2594	Selmamoustaadil	2006
10905	0	0	0	2594	Charlottevan zonneveld	2004
10906	0	0	0	2594	Kimikocooper	2008
10907	0	0	0	2594	Kiarazabkova	2007
10908	0	0	0	2594	Consueloalarcon	2006
10909	0	0	0	2594	Karinayurchenko	2004
10910	0	0	0	2594	Ireneaguirre	2005
10911	0	0	0	2594	Durukuscu	2006
10912	0	0	0	2594	Danielaaguilar	2006
10913	0	0	0	2594	Silverbijlsma	2007
10914	0	0	0	2594	Sofiasrebnick	2006
10915	0	0	0	2594	Feidiabreu	2004
10916	0	0	0	2594	Ariba Imranidrees	2006
10917	0	0	0	2594	Maria Isabelmorales reyes	2005
10918	0	0	0	2594	Tamarabachmann	2004
10919	0	0	0	2594	Lornapujals	2007
10920	0	0	0	2594	Denizdilek	2007
10921	0	0	0	2594	Husnakathrada	2003
10922	0	0	0	2594	Ximenasenties	2006
10923	0	0	0	2594	Kristinabogomazova	2006
10924	0	0	0	2594	Yevheniiazelenska	2007
10925	0	0	0	2594	Alejandravazquez	2004
10926	0	0	0	2594	Renadayman maamoun	2005
10927	0	0	0	2594	Ozgeyuksel	2006
10928	0	0	0	2594	katrinesadze	2004
10929	0	0	0	2594	Alexandrakala	2004
10930	0	0	0	2594	Lourdes Mariaorozco	2006
10931	0	0	0	2594	Carinasyrtveit	2005
10932	0	0	0	2594	Zuzannaszmyd	2004
10933	0	0	0	2594	Ana Sofiabeccar	2005
10934	0	0	0	2594	Athinagrigoriadou	2007
10935	0	0	0	2594	Parichavan	2005
10936	0	0	0	2594	Emelinemoreaux	2005
10937	0	0	0	2594	Ellasalonen	2003
10938	0	0	0	2594	Hannahhasegawa	2007
10939	0	0	0	2594	Hinatafurutani	2006
10940	0	0	0	2594	Ariadna Sayanafernandez ceval	2006
10941	0	0	0	2594	Carolinamesquita	2006
10942	0	0	0	2594	Nataliaalves de lima	2006
10943	0	0	0	2594	Sophiemartin	2004
10944	0	0	0	2594	Ximenahernandez	2006
10945	0	0	0	2594	Elisabourdeilletas	2006
10946	0	0	0	2594	Juliettema	2003

10947	0	0	0	2594	Hastinouriaskarabad	2006
10948	0	0	0	2594	Aisanakojonazarova	2006
10949	0	0	0	2594	Polinakorolova	2007
10950	0	0	0	2594	Anastasiasamets	2005
10951	0	0	0	2594	Milenacanellas	2003
10952	0	0	0	2594	Magdalenabaniak	2006
10953	0	0	0	2594	Annaasadullina	2007
10954	0	0	0	2594	D'Estreecolalancia	2005
10955	0	0	0	2594	Samanthaimbo nloga	2003
10956	0	0	0	2594	Ugnevingyte	2007
10957	0	0	0	2594	Dinalipovski	2007
10958	0	0	0	2594	Ninaandreoni	2006
10959	0	0	0	2594	Rachelcranley	2003
10960	0	0	0	2594	Kristinalee	2005
10961	0	0	0	2594	Shaniceweekes	2004
10962	0	0	0	2594	Tiakaukenas	2006
10963	0	0	0	2594	Severinedeppner	2007
10964	0	0	0	2594	Evrayildirim	2004
10965	0	0	0	2594	Claudia Valentinavalderrama te	2005
10966	0	0	0	2594	Avaschueller	2006
10967	0	0	0	2594	Amelijasupulniece	2004
10968	0	0	0	2594	Cristina Mariacorrales nieves sa	2006
10969	0	0	0	2594	Leaerenda	2005
10970	0	0	0	2594	Alessandrasikharulidze	2007
10971	0	0	0	2594	Aleksandrarozkalne	2008
10972	0	0	0	2594	Ema Albinablahova	2003
10973	0	0	0	2594	Zaraburns	2005
10974	0	0	0	2594	Mariangelnunez	2005
10975	0	0	0	2594	Mahyaabedini	2007
10976	0	0	0	2594	Kureahayasaka	2008
10977	0	0	0	2594	Lisemaury	2006
10978	0	0	0	2594	Chizitemokonjo	2005
10979	0	0	0	2594	Manamitanaka	2004
10980	0	0	0	2594	Dinaraalloyarova	2003
10981	0	0	0	2594	Sofiamatiushova	2003
10982	0	0	0	2594	Angelina Beatricestancu	2006
10983	0	0	0	2594	Isabelleturra karam	2006
10984	0	0	0	2594	Camilacardona-carballosa	2006
10985	0	0	0	2594	Venetagencheva	2006
10986	0	0	0	2594	Cenanliu	2005
10987	0	0	0	2594	Andrejagagilaite	2005
10988	0	0	0	2594	Alessiapopescu	2004
10989	0	0	0	2594	Millafalster	2003
10990	0	0	0	2594	Paulinaalvarez	2003
10991	0	0	0	2594	Seyedeh Arshinkhadem	2004
10992	0	0	0	2594	Leahbaroudi	2007
10993	0	0	0	2594	Orianagrott	2003
10994	0	0	0	2594	Annalottahoworka	2007
10995	0	0	0	2594	Sumejjaisic	2003
10996	0	0	0	2594	Yelyzavetachainykova	2006

10997	0	0	0	2594	Millafalster	2003
10998	0	0	0	2594	Rachelcranley	2003
10999	0	0	0	2594	Silverbijlsma	2007
11000	0	0	0	2594	Mahyaabedini	2007
11001	0	0	0	2594	Katebrzostek	2003
11002	0	0	0	2594	Marianachaves	2006
11003	0	0	0	2594	Cenanliu	2005
11004	0	0	0	2594	Isabelleturra karam	2006
11005	0	0	0	2594	Emilielugon moulin	2004
11006	0	0	0	2594	Amelijasupulniece	2004
11007	0	0	0	2594	Charlottevan zonneveld	2004
11008	0	0	0	2594	Seyedeh Arshinkhadem	2004
11009	0	0	0	2594	Shriyapadmanabhan	2006
11010	0	0	0	2594	Valentinamerku	2006
11011	0	0	0	2594	Annalottahoworka	2007
11012	0	0	0	2594	Kaitlyn Leighramduth	2006
11013	0	0	0	2594	Sofiamatiushova	2003
11014	0	0	0	2594	Parneetkaur	2004
11015	0	0	0	2594	Luisaschruff	2005
11016	0	0	0	2594	Emmakarasova	2006
11017	0	0	0	2594	Yasminealjane	2007
11018	0	0	0	2594	Alkminigiannakogiorgou	2004
11019	0	0	0	2594	Erikafreire	2003
11020	0	0	0	2594	Sabinapintea	2006
11021	0	0	0	2594	Alhonda	2007
11022	0	0	0	2594	Valentinalanatta	2006
11023	0	0	0	2594	Paulinaalvarez	2003
11024	0	0	0	2594	REINE SIRRIakombo	2003
11025	0	0	0	2594	Artinagashi	2007
11026	0	0	0	2594	Anastasiyakolyada	2007
11027	0	0	0	2594	Skyebentum-siripi	2005
11028	0	0	0	2594	Isabellaortiz plata	2007
11029	0	0	0	2594	Anelle Margarettait	2006
11030	0	0	0	2594	Sarahjemail	2007
11031	0	0	0	2594	Mariebannmeyer	2006
11032	0	0	0	2594	kimiasagheb tehrani	2007
11033	0	0	0	2594	Annaslabous	
11034	0	0	0	2594	Catharina Sophiaandrade cardo	2006
11035	0	0	0	2594	Manamitanaka	2004
11036	0	0	0	2594	Fernandaaguerri	2006
11037	0	0	0	2594	Sofia Magalibarreto scholz	2007
11038	0	0	0	2594	Luciagallegos	2007
11039	0	0	0	2594	Sophiemartin	2004
11040	0	0	0	2594	Anikaracho akl	2005
11041	0	0	0	2594	Zainab Aliraja	2008
11042	0	0	0	2594	asaljavidan	2005
11043	0	0	0	2594	Emmamason	2006
11044	0	0	0	2594	Adyapoudyal	2008
11045	0	0	0	2594	Alexandra Valentinacedeno her	2005
11046	0	0	0	2594	Townsendpenney	2006

11047	0	0	0	2594	Eloisenewberry	2008
11048	0	0	0	2594	Lauralisboa dib vieira	2006
11049	0	0	0	2594	Samikshasarkar	2006
11050	0	0	0	2594	Ozieraahmad	2005
11051	0	0	0	2594	Caitlinbaker	2005
11052	0	0	0	2757	Rosaliecarrier-dulac	2005
11053	0	0	0	2757	Majaradisic	2003
11054	0	0	0	2757	Hanarose	2005
11055	0	0	0	2757	RINKAhari	2005
11056	0	0	0	2757	Michellekorpys	2004
11057	0	0	0	2757	Natashachidzwondo	2007
11058	0	0	0	2757	Oliviabrown	2006
11059	0	0	0	2757	Alizeepoulin	2006
11060	0	0	0	2757	Emamravcova	2006
11061	0	0	0	2757	Ernabrdarevic	2003
11062	0	0	0	2757	Annabelleloutit	2006
11063	0	0	0	2757	Shorouk mohamedelasuty	2006
11064	0	0	0	2757	Heewonkil	2004
11065	0	0	0	2757	Peytoncapuano	2005
11066	0	0	0	2757	Alanishamilton	2007
11067	0	0	0	2757	Clemencemercier	2008
11068	0	0	0	2757	Ioana Mariaabrudan	2005
11069	0	0	0	2757	Angelamarnika	2004
11070	0	0	0	2757	Lojainkamel	2007
11071	0	0	0	2757	Isabellaaguiar	2006
11072	0	0	0	2757	Aaliyahhohmann	2003
11073	0	0	0	2757	Rosariohormazabal	2003
11074	0	0	0	2757	Marian Camilaangel velasquez	2004
11075	0	0	0	2757	Melissa Nanjalamwakha	2008
11076	0	0	0	2757	Denissearinez guilarte	2005
11077	0	0	0	2757	Nouraymetwally	2005
11078	0	0	0	2757	Morigrylak	2005
11079	0	0	0	2757	Risaueno	2007
11080	0	0	0	2757	Emmavanderheyden	2003
11081	0	0	0	2757	Gabrielestrolyte	2004
11082	0	0	0	2757	Manuellapersson	2005
11083	0	0	0	2757	Valentinauribe arriaga	2007
11084	0	0	0	2757	Anca Stefaniasauciuc	2006
11085	0	0	0	2757	Claranilsson	2006
11086	0	0	0	2757	Addisonbowman	2006
11087	0	0	0	2757	Crissy Alexiatudora	2004
11088	0	0	0	2757	nataliewagner	2003
11089	0	0	0	2757	Gabriellarawles	2007
11090	0	0	0	2757	Emma Andreatutoveanu	2006
11091	0	0	0	2757	Mercylineoresha	2007
11092	0	0	0	2757	Sonjawedenski	2008
11093	0	0	0	2757	Rotemsabag	2005
11094	0	0	0	2757	Hannaserhel	2003
11095	0	0	0	2757	Annaprotsenko	2003
11096	0	0	0	2757	Gabrielladanielsson	2004

11097	0	0	0	2757	Morigrylak	2005
11098	0	0	0	2757	Natashachidzwondo	2007
11099	0	0	0	2757	Annabelleganescu	2008
11100	0	0	0	2757	Nataliemachkova	2005
11101	0	0	0	2757	Jennipher L Mugalamugala	2008
11102	0	0	0	2757	Emma Andreatutoveanu	2006
11103	0	0	0	2757	Ernabrdarevic	2003
11104	0	0	0	2757	Gabriellalindley	2005
11105	0	0	0	2757	Angelaganeva	2005
11106	0	0	0	2757	Peytoncapuano	2005
11107	0	0	0	2757	LINDAserena	2006
11108	0	0	0	2757	Dianaloseva	2007
11109	0	0	0	2757	Alisaozgun	2007
11110	0	0	0	2757	Rosaliecarrier-dulac	2005
11111	0	0	0	2757	Hanagamracy	2003
11112	0	0	0	2757	Cecilycortes	2006
11113	0	0	0	2757	Gretamurzsa	2005
11114	0	0	0	2757	Emamravcova	2006
11115	0	0	0	2757	Madeleinereed	2006
11116	0	0	0	2757	Sabinahasancic	2006
11117	0	0	0	2757	Emiliaschmidt	2004
11118	0	0	0	2757	Ioana Mariaabrudan	2005
11119	0	0	0	2757	Vyomaabhaskar	2003
11120	0	0	0	2757	Juliemyatovic	2007
11121	0	0	0	2757	Michellekorpys	2004
11122	0	0	0	2757	Anca Stefaniasauciuc	2006
11123	0	0	0	2757	Rileycrowder	2007
11124	0	0	0	2757	Annagymnander	2003
11125	0	0	0	2757	Gabrielladanielsson	2004
11126	0	0	0	2757	Sonjawedenski	2008
11127	0	0	0	2757	Charlottabuss	2004
11128	0	0	0	2757	nataliewagner	2003
11129	0	0	0	2757	Chutnareeboonklang	2004
11130	0	0	0	2757	Denissearinez guilarte	2005
11131	0	0	0	2757	Emilijasausverde	2004
11132	0	0	0	2757	Claranilsson	2006
11133	0	0	0	2757	Ornella Mariamiranda semiglia	2005
11134	0	0	0	2757	Majaradisic	2003
11135	0	0	0	2757	Gabriellarawles	2007
11136	0	0	0	2757	Yositasriporn	2007
11137	0	0	0	2757	Ella Rainenielsen	2005
11138	0	0	0	2757	Rosariohormazabal	2003
11139	0	0	0	2757	Adeliyamukhutdinova	2003
11140	0	0	0	2757	Evelinaanastasi	2006
11141	0	0	0	2757	Stoneycooks	2007
11142	0	0	0	2757	Kristinakuksina	2004
11143	0	0	0	2757	Rafaelaplatsiota	2003
11144	0	0	0	2757	Nouraymetwally	2005
11145	0	0	0	2757	Lindaskvrndova	2004
11146	0	0	0	2757	Thekliachristodoulou	2004

11147	0	0	0	2757	Daryavolkova	2003
11148	0	0	0	2757	Juliaacioly	2004
11149	0	0	0	2757	Reginadavalos	2005
11150	0	0	0	2757	Briana Mariaspataru	2005
11151	0	0	0	2757	Justinebretnacher	2007
11152	0	0	0	2757	Rotemsabag	2005
11153	0	0	0	2757	Tumikgosiemang	2006
11154	0	0	0	2757	Polyanikolova	2004
11155	0	0	0	2757	Tomimain	2003
11156	0	0	0	2757	Alexismelov	2004
11157	0	0	0	2757	Nataliebartova	2005
11158	0	0	0	2757	STACYchepkemei yego	2008
11159	0	0	0	2864	ETOROemmanuel bassey	2007
11160	0	0	0	2864	Mayrasama	2006
11161	0	0	0	2864	Anasabikova	2004
11162	0	0	0	2864	Scendyhenry	2008
11163	0	0	0	2864	Emmavasilenko	2007
11164	0	0	0	2864	Rayanashah	2005
11165	0	0	0	2864	Emiliajarvinen	2004
11166	0	0	0	2864	EMMANUELglory	2008
11167	0	0	0	2864	Eceboylu	2006
11168	0	0	0	2864	Sarah Danieladaza obando	2004
11169	0	0	0	2864	Zeynepkocak	2004
11170	0	0	0	2864	Gretarizzetto	2007
11171	0	0	0	2864	Hlasaber	2004
11172	0	0	0	2864	Lucianabejarano cuellar	2004
11173	0	0	0	2864	SUNMISOLAokajare	2005
11174	0	0	0	2864	Andrea Paolaandrade fierro	2007
11175	0	0	0	2864	Knoozfouad	2005
11176	0	0	0	2864	Eugeniazozaya menendez	2007
11177	0	0	0	2864	Miamayerova	2003
11178	0	0	0	2864	MARIA FERNANDA CALFATduar	2004
11179	0	0	0	2864	Evelinazhuk	2007
11180	0	0	0	2864	Minjichoi	2007
11181	0	0	0	2864	Katyabalin	2004
11182	0	0	0	2864	Barborakanova	2006
11183	0	0	0	2864	Lauravarsamaki	2005
11184	0	0	0	2864	MARIA PIAgonzalez	2006
11185	0	0	0	2864	Karolasuviste	2004
11186	0	0	0	2864	Kazukioya	2004
11187	0	0	0	2864	Dariagiansante	2003
11188	0	0	0	2864	Daniela Valentinatijaro	2006
11189	0	0	0	2864	Charlyegger	2004
11190	0	0	0	2864	Riannaalame	2007
11191	0	0	0	2864	Sandracastedo	2006
11192	0	0	0	2864	Valeriacasusol	2007
11193	0	0	0	2864	Nanakasato	2006
11194	0	0	0	2864	Petrakoszo	2006
11195	0	0	0	2864	Vitoriasolis urdiales	2004
11196	0	0	0	2864	Tadisachamisa	2005

11197	0	0	0	2864	Aliciacelorio morales	2006
11198	0	0	0	2864	Yoanamoneva	2005
11199	0	0	0	2864	Kaiakruus	2005
11200	0	0	0	2864	Ivarafailovic	2006
11201	0	0	0	2864	Eceboylu	2006
11202	0	0	0	2864	Anasabikova	2004
11203	0	0	0	2864	Minjichoi	2007
11204	0	0	0	2864	Daniela Valentinatijaro	2006
11205	0	0	0	2864	Tadisachamisa	2005
11206	0	0	0	2864	Ellenkatzer	2007
11207	0	0	0	2864	Parisingh	2005
11208	0	0	0	2864	Scendyhenry	2008
11209	0	0	0	2864	Gretarizzetto	2007
11210	0	0	0	2864	Petrakoszo	2006
11211	0	0	0	2864	Sarasimonova	2003
11212	0	0	0	2864	Emmavasilenko	2007
11213	0	0	0	2864	Emiliajarvinen	2004
11214	0	0	0	2864	Defneursan	2005
11215	0	0	0	2864	Zeynepkocak	2004
11216	0	0	0	2864	Veronikanovakova	2006
11217	0	0	0	2864	Veena Mariamnazar	2005
11218	0	0	0	2864	Miamayerova	2003
11219	0	0	0	2864	Lucianabejarano cuellar	2004
11220	0	0	0	2864	Vitoriasolis urdiales	2004
11221	0	0	0	2864	Dariagiansante	2003
11222	0	0	0	2864	Rayanashah	2005
11223	0	0	0	2864	Faustineliope	2004
11224	0	0	0	2864	Maxine Sophiekammerer	2005
11225	0	0	0	2864	Karolasuviste	2004
11226	0	0	0	2864	Emilycallahan	2003
11227	0	0	0	2864	Lianadvalishvili	2003
11228	0	0	0	2864	Charlyegger	2004
11229	0	0	0	2864	Stellasimeonova	2003
11230	0	0	0	2935	Lottanurminen	2004
11231	0	0	0	2935	Amandamenendez thomasson	2003
11232	0	0	0	2935	Irmamartynovskaia	2004
11233	0	0	0	2935	Jovananikolic	2003
11234	0	0	0	2935	Sofiakanarskaya	2004
11235	0	0	0	2935	Natalijanikolic	2004
11236	0	0	0	2935	Hristinaljuba	2004
11237	0	0	0	2935	Constancaazinhaga	2004
11238	0	0	0	2935	Yasminimamniyazova	2004
11239	0	0	0	2935	Juliettedoukhan	2004
11240	0	0	0	2935	Anizeikidze	2003
11241	0	0	0	2935	Denisetorrealba	2003
11242	0	0	0	2935	Matildecoen	2004
11243	0	0	0	2935	Enaarnautovic	2004
11244	0	0	0	2935	Victoriagrymalska	2005
11245	0	0	0	2935	Hanasokolovic	2004
11246	0	0	0	2935	Juliazlateva	2005

11247	0	0	0	2935	Celiabastin	2004
11248	0	0	0	2935	Laranedeljkovic	2004
11249	0	0	0	2935	Nataliakowalczyk	2004
11250	0	0	0	2935	Min Kyojung	2004
11251	0	0	0	2935	Simonadeletic	2005
11252	0	0	0	2935	Arisakobayashi	2005
11253	0	0	0	2935	Sangeeprieto	2004
11254	0	0	0	2935	Manuelaramirez correa	2004
11255	0	0	0	2935	Luisacabrera	2005
11256	0	0	0	2935	Sophiaaray	2003
11257	0	0	0	2935	Isabellaherrera pilonieta	2004
11258	0	0	0	2935	Abbycandelier	2005
11259	0	0	0	2935	Martakurmaz	2006
11260	0	0	0	2935	Da-Eunchoi	2004
11261	0	0	0	2935	Jin-Solyang	2004
11262	0	0	0	2935	Yumakomatsu	2004
11263	0	0	0	2935	Emmatagger	2006
11264	0	0	0	2935	Hoi Yiu Michellewong	2005
11265	0	0	0	2935	Annatereshchenko	2005
11266	0	0	0	2935	Aleksandraaverianova	2005
11267	0	0	0	2935	Yuliiakhmara	2005
11268	0	0	0	2935	Olesyaborozdenko	2004
11269	0	0	0	2935	Danicanedeljkovic	2005
11270	0	0	0	2935	Nutsatsintsadze	2005
11271	0	0	0	2935	Fortamorina	2005
11272	0	0	0	2935	Hanasonton	2005
11273	0	0	0	2935	Seojinoh	2003
11274	0	0	0	2935	Ekaterinaburenina	2004
11275	0	0	0	2935	Lilienlasakova	2005
11276	0	0	0	2935	Zlatabartanusz	2007
11277	0	0	0	2935	Evdokiiaartamonova	2007
11278	0	0	0	2935	Chulapatsathutham	2005
11279	0	0	0	2935	Marineboutros	2006
11280	0	0	0	2935	Lindaajimi	2006
11281	0	0	0	2935	Nicollyferreira	2003
11282	0	0	0	2935	Maria Angeortega mateu	2003
11283	0	0	0	2935	Sanjivanikutwal	2004
11284	0	0	0	2935	MAHIpanchal	2003
11285	0	0	0	2935	Munashebera	2005
11286	0	0	0	2935	Ranhyadiabate	2005
11287	0	0	0	2935	Sotiriakatsali	2006
11288	0	0	0	2935	Nicolebaccala righetto	2005
11289	0	0	0	2935	Martinabeltraminelli	2006
11290	0	0	0	2935	Blancatedeschi	2006
11291	0	0	0	2935	Nikolakoprivova	2006
11292	0	0	0	2935	Gabriella Victorianoa	2004
11293	0	0	0	2935	Flaviamonteverde	2006
11294	0	0	0	2935	Ana Mihaelagavrilescu	2004
11295	0	0	0	2935	Darianazarenko	2004
11296	0	0	0	2935	Laurabuchs	2005

11297	0	0	0	2935	Aimeefrey	2004
11298	0	0	0	2935	Paigegilbert	2003
11299	0	0	0	2935	Maohiguchi	2007
11300	0	0	0	2935	Yaelle Rosinaetter	2004
11301	0	0	0	2935	Simoanerodgers	2004
11302	0	0	0	2935	Ishanederman	2005
11303	0	0	0	2935	Jimena Maytepalomo villa	2004
11304	0	0	0	2935	Yuet Tungchiu	2005
11305	0	0	0	2935	Mfranco salcedo	2003
11306	0	0	0	2935	Yennlee	2006
11307	0	0	0	2935	Danielacruzado fretel	2004
11308	0	0	0	2935	Simonesteyn	2004
11309	0	0	0	2935	Mathilde Mosegaardravnholt	2005
11310	0	0	0	2935	ryanwuhrman	2005
11311	0	0	0	2935	Sofia Isabelancieta cortez	2004
11312	0	0	0	2935	Vittoriabenedetti	2006
11313	0	0	0	2935	Mariatsironi	2006
11314	0	0	0	2935	Janasvobodova	2008
11315	0	0	0	2935	Ariana Fernandarodriguez	2005
11316	0	0	0	2935	Galateaferro	2007
11317	0	0	0	2935	Karolinarajca	2005
11318	0	0	0	2935	Melissalino airoldi	2006
11319	0	0	0	2935	Tatiana Elenagitopoulou	2006
11320	0	0	0	2935	Karolinaheiderova	2006
11321	0	0	0	2935	Cristiana Nicoletatodoni	2006
11322	0	0	0	2935	Ibenlyme	2005
11323	0	0	0	2935	Cinthyapuetate	2004
11324	0	0	0	2935	Isabelladanielsson	2006
11325	0	0	0	2935	Mancipal	2005
11326	0	0	0	2935	Sadjocoulibaly	2004
11327	0	0	0	2935	Tomirismussina	2006
11328	0	0	0	2935	Tairaabildayeva	2006
11329	0	0	0	2935	Rokayazaki	2007
11330	0	0	0	2935	Valentinazambrano soto	2005
11331	0	0	0	2935	Stanislavashchekotur	2005
11332	0	0	0	2935	Giulia Sophystefan	2003
11333	0	0	0	2935	Lynnkader	2004
11334	0	0	0	2935	Miakrsteva	2003
11335	0	0	0	2935	Heung Tungcheng	2007
11336	0	0	0	2935	Bhumikatripathi	2004
11337	0	0	0	2935	Kelly Marierichter	2005
11338	0	0	0	2935	Iriannademetriou	2005
11339	0	0	0	2935	Natsukiirie	2004
11340	0	0	0	2935	Kseniapanteleeva	2005
11341	0	0	0	2935	Carolmota oliveira	2004
11342	0	0	0	2935	Genesis Alexandragonzalez pati	2005
11343	0	0	0	2935	Cleliadrudi lombardi	2004
11344	0	0	0	2935	Aisha Dolkergurung	2006
11345	0	0	0	2935	Lararossetto diniz de souza	2004
11346	0	0	0	2935	Luiza Caixetaparreira	2003

11347	0	0	0	2935	Nimishadhakal	2006
11348	0	0	0	2935	Reginaruiz	2003
11349	0	0	0	2935	Kristinamikhailova	2005
11350	0	0	0	2935	Maraia Paulamanrique	2004
11351	0	0	0	2935	Isabellaarana rios	2004
11352	0	0	0	2935	Maigoto	2007
11353	0	0	0	2935	Naomikarki	2005
11354	0	0	0	2935	Kannasoeda	2006
11355	0	0	0	2935	Saijayanibanerjee	2007
11356	0	0	0	2935	Marinaadati	2005
11357	0	0	0	2935	Hatshepsutassanova	2006
11358	0	0	0	2935	Hannahhafez	2007
11359	0	0	0	2935	Isabellezhu	2005
11360	0	0	0	2935	Nicolewuelfing	2004
11361	0	0	0	2935	Mary Boycedeatherage	2006
11362	0	0	0	2935	Karoline Mariasantos de oliveir	2005
11363	0	0	0	2935	Hannakoprowska	2006
11364	0	0	0	2935	Kristinabelkova	2007
11365	0	0	0	2935	Sameekshaannem	2006
11366	0	0	0	2935	Kameamedora	2005
11367	0	0	0	2935	Fleurmaris	2005
11368	0	0	0	2935	Corneliakack	2005
11369	0	0	0	2935	Barbara Stephanieolvera robles	2007
11370	0	0	0	2935	Anastasiyabelobrova	2008
11371	0	0	0	2935	Katelynvu	2003
11372	0	0	0	2935	Polinaburenina	2006
11373	0	0	0	2935	Nadyalyekontseva	2004
11374	0	0	0	2935	Sunirathapa	2008
11375	0	0	0	2935	Maria Josehernandez diaz	2005
11376	0	0	0	2935	Zeliebretin	2008
11377	0	0	0	2935	Marie Amandinereyes	2005
11378	0	0	0	2935	RAFFAELLAsalas	2005
11379	0	0	0	2935	Chiarasobenes	2005
11380	0	0	0	2935	Katherin Beatrizchacon quitian	2005
11381	0	0	0	2935	Honorikoyama	2006
11382	0	0	0	2935	VALERIA MARGARITAmora alva	2006
11383	0	0	0	2935	YI LAMlu	2007
11384	0	0	0	2935	Yebinkang	2008
11385	0	0	0	2935	Polinamarakhtanova	2006
11386	0	0	0	2935	Nataliasousa salazar	2006
11387	0	0	0	2935	Mayaeastburg	2007
11388	0	0	0	2935	Nehirkaragoz	2005
11389	0	0	0	2935	Meritxellteixido garcia	2007
11390	0	0	0	2935	Luciagomez delgado	2005
11391	0	0	0	2935	Kirstinhailey	
11392	0	0	0	2935	Catarinamelleiro	2003
11393	0	0	0	2935	Fatima Aliraja	2007
11394	0	0	0	2935	Mahugnon Eliazahonfoga	2006
11395	0	0	0	2935	Lidia Alejandrasuarez gonzalez	2004
11396	0	0	0	2935	Irinaalfonso	

11397	0	0	0	2935	Andreagonzalez	2004
11398	0	0	0	2935	Jessicahess	
11399	0	0	0	2935	Maria Eduarda Knolseisen Mora	2007
11400	0	0	0	2935	Anna Luciarodriguez coniglione	2006
11401	0	0	0	2935	Sophiemcdonald	2005
11402	0	0	0	2935	Mahikakhanna	2007
11403	0	0	0	2935	Athinaschlepphorst	2007
11404	0	0	0	2935	Lizmeylmoreira	2007
11405	0	0	0	2935	Sophiasingh	2007
11406	0	0	0	2935	Emiradiabate	2007
11407	0	0	0	2935	Juliepastikova	2008
11408	0	0	0	2935	SREE TANVIdasari	2007
11409	0	0	0	2935	Valeriaganina	2004
11410	0	0	0	2935	Katina Estefaniazepeda barrios	2007
11411	0	0	0	2935	Maria Pazdiaz	2003
11412	0	0	0	2935	Maria Eduardamalvestiti	2003
11413	0	0	0	2935	Rishitha Reddybasireddy	2008
11414	0	0	0	2935	Sofiyakhegay	2007
11415	0	0	0	2935	Sayurihata	2006
11416	0	0	0	2935	Beatrizlima ibrahim	2005
11417	0	0	0	2935	Nishareybuapetch	2006
11418	0	0	0	2935	Alessiascanga	2005
11419	0	0	0	2935	Serene Orphelianguimbis ntove	2006
11420	0	0	0	2935	Carolinatroiano	2005
11421	0	0	0	2935	Pimchanokjantapreechapong	2006
11422	0	0	0	2935	Annikameier vestermann	2004
11423	0	0	0	2935	Beatrizferreira	2004
11424	0	0	0	2935	KALINA-MAYstefanov	2008
11425	0	0	0	2935	Madelynnludwig	2007
11426	0	0	0	2935	Andreinatejada	2004
11427	0	0	0	2935	Erikajafar	2006
11428	0	0	0	2935	AIKOarakawa	2008
11429	0	0	0	2935	Maria Teodoraniculescu	2005
11430	0	0	0	2935	Audreyouellet	2005
11431	0	0	0	2935	Stefaniyayakubovskaya	2006
11432	0	0	0	2935	Marcelaolvera	2005
11433	0	0	0	2935	Shakiravarese	2006
11434	1	1	1	1	Elsajacquemot	2003
11435	1	1	1	2	Victoriajimenez kasintseva	2005
11436	1	1	1	3	Alexandraeala	2005
11437	1	1	1	4	Dianeparry	2002
11438	1	1	1	5	Dariasnigur	2002
11439	1	1	1	6	Polinakudermetova	2003
11440	1	1	1	7	Robinmontgomery	2004
11441	1	1	1	8	Alexandravecic	2002
11442	1	1	1	9	Kristinadmitruk	2003
11443	1	1	1	10	Alexanoel	2002
11444	1	1	1	11	Kamillabartone	2002
11445	1	1	1	12	Weronikabaszak	2002
11446	1	1	1	13	Oksanaselekhmeteva	2003

11447	1	1	1	14	Alinacharaeva	2002
11448	1	1	1	15	Mariabondarenko	2003
11449	1	1	1	16	Leylahfernandez	2002
11450	1	1	1	17	Priska Madelynnugroho	2003
11451	1	1	1	18	Lindafruhvirtova	2005
11452	1	1	1	19	Danaguzman	2003
11453	1	1	1	20	Matildamutavdzic	2004
11454	1	1	1	21	Lindanoskova	2004
11455	1	1	1	22	Hong Yi Codywong	2002
11456	1	1	1	23	Zhuoxuanbai	2002
11457	1	1	1	24	Mell Elizabethreasco gonzalez	2002
11458	1	1	1	25	Sofiacostoulas	2005
11459	1	1	1	26	Dianashnaider	2004
11460	1	1	1	27	Janakolodynska	2003
11461	1	1	1	28	Qinwenzheng	2002
11462	1	1	1	29	Alexandrayepifanova	2003
11463	1	1	1	30	Matildepaoletti	2003
11464	1	1	1	31	Melaniadelai	2002
11465	1	1	1	32	Elvinakalieva	2003
11466	1	1	1	33	Oceanebabel	2004
11467	1	1	1	34	Guillerminagrant	2002
11468	1	1	1	35	Darjavidmanova	2003
11469	1	1	1	36	Melodiecollard	2003
11470	1	1	1	37	Alionafalei	2004
11471	1	1	1	38	Mai Napattnirundorn	2002
11472	1	1	1	39	Elizabethcoleman	2003
11473	1	1	1	40	Aubanedroguet	2002
11474	1	1	1	41	Madisonsieg	2003
11475	1	1	1	42	Natsumikawaguchi	2002
11476	1	1	1	43	Leyreromero gormaz	2002
11477	1	1	1	44	Elinaavanesyan	2002
11478	1	1	1	45	Anemintegi del olmo	2003
11479	1	1	1	46	Nataliaszabanin	2003
11480	1	1	1	47	Zivafalkner	2002
11481	1	1	1	48	Juliagarcia	2003
11482	1	1	1	49	Erikaandreeva	2004
11483	1	1	1	50	Celia Bellemohr	2002
11484	1	1	1	51	Evialinalaskevich	2004
11485	1	1	1	52	Sebastiannascilipoti	2003
11486	1	1	1	53	Anageller	2002
11487	1	1	1	54	Dayeonback	2002
11488	1	1	1	55	Amarissa Kiaratoth	2003
11489	1	1	1	56	Selenajanicijevic	2002
11490	1	1	1	57	Romanacisovska	2002
11491	1	1	1	58	Maraguth	2003
11492	1	1	1	59	Laurahietaranta	2004
11493	1	1	1	60	Juliaavdeeva	2002
11494	1	1	1	61	Beatricericci	2003
11495	1	1	1	62	Eleonoraalvisi	2003
11496	1	1	1	63	Jessicabouzas maneiro	2002

11497	1	1	1	64	Juliebelgraver	2002
11498	1	1	1	65	Lisapigato	2003
11499	1	1	1	66	Ya Yiyang	2004
11500	1	1	1	67	Evalys	2002
11501	1	1	1	68	Taliagibson	2004
11502	1	1	1	69	Radkazelnickova	2003
11503	1	1	1	70	Yeonwooku	2003
11504	1	1	1	71	Katrinascott	2004
11505	1	1	1	72	Barborapalicova	2004
11506	1	1	1	73	Ashlynkrueger	2004
11507	1	1	1	74	Alinashcherbinina	2003
11508	1	1	1	75	Giuliamorlet	2002
11509	1	1	1	76	Tina Nadinesmith	2002
11510	1	1	1	77	Sohyunpark	2002
11511	1	1	1	78	Lucienguyen tan	2003
11512	1	1	1	79	Tarawuerth	2002
11513	1	1	1	80	Polinaiatcenko	2003
11514	1	1	1	81	Hannevandewinkel	2004
11515	1	1	1	82	Laurenanzalotta kynoch	2002
11516	1	1	1	83	Savannahbroadus	2002
11517	1	1	1	84	Angelinawirges	2002
11518	1	1	1	85	Amarnibanks	2002
11519	1	1	1	86	Sabinazeynalova	2004
11520	1	1	1	87	Jadabui	2002
11521	1	1	1	88	Pialovric	2002
11522	1	1	1	89	Erikamatsuda	2003
11523	1	1	1	90	Liubovkostenko	2003
11524	1	1	1	91	Anaelleleclercq	2003
11525	1	1	1	92	Alessandrasimone	2003
11526	1	1	1	93	Darjasemenistaja	2002
11527	1	1	1	94	Tatyananikolenko	2004
11528	1	1	1	95	Dariafrayman	2002
11529	1	1	1	96	Nastasjaschunk	2003
11530	1	1	1	97	Oliviagadecki	2002
11531	1	1	1	98	Indiahoughton	2002
11532	1	1	1	99	Mariasholokhova	2004
11533	1	1	1	100	Juliamiddendorf	2003
11534	1	1	1	101	Annabellexu	2004
11535	1	1	1	102	Petramarcinko	2005
11536	1	1	1	103	Tatianaprozorova	2003
11537	1	1	1	104	Zongyuli	2003
11538	1	1	1	105	Rominaccuno	2002
11539	1	1	1	106	Solanasierra	2004
11540	1	1	1	107	Flaviebrugnone	2003
11541	1	1	1	108	Mariatimofeeva	2003
11542	1	1	1	109	Meihasegawa	2003
11543	1	1	1	110	Ameliabissett	2003
11544	1	1	1	111	Sofia Elenacabezas dominguez	2002
11545	1	1	1	112	Ekaterinamaklakova	2004
11546	1	1	1	113	Fatima Ingrid Amarthakeita	2003

11547	1	1	1	114	Sofia Camilarojas	2002
11548	1	1	1	115	Alejandracruz	2003
11549	1	1	1	116	Biancabehulova	2003
11550	1	1	1	117	Jennadefalco	2002
11551	1	1	1	118	Amystevens	2002
11552	1	1	1	119	Hinainoue	2003
11553	1	1	1	120	Pimradajattavapornvanit	2003
11554	1	1	1	121	Nicolerivkin	2003
11555	1	1	1	122	Juanalarranaga	2002
11556	1	1	1	123	Marketapanackova	2002
11557	1	1	1	124	Tilwithdi girolami	2003
11558	1	1	1	125	Sakiimamura	2002
11559	1	1	1	126	Luciehavlickova	2005
11560	1	1	1	127	Isabellekouzmanov	2004
11561	1	1	1	128	Noakrznaric uygungul	2003
11562	1	1	1	129	Lanmi	2004
11563	1	1	1	130	Lolaradivojevic	2005
11564	1	1	1	131	Jasmineconway	2004
11565	1	1	1	132	Helenepellicano	2002
11566	1	1	1	133	Klaramilicevic	2003
11567	1	1	1	134	Sophieluescher	2002
11568	1	1	1	135	Martacustic	2002
11569	1	1	1	136	Reesebrantmeier	2004
11570	1	1	1	137	Jiaqiwang	2002
11571	1	1	1	138	Janicetjen	2002
11572	1	1	1	139	Yasminekabbaj	2004
11573	1	1	1	140	Alinagranwehr	2003
11574	1	1	1	141	Evashaw	2005
11575	1	1	1	142	Michaelalaki	2005
11576	1	1	1	143	Lindaklimovicova	2004
11577	1	1	1	144	Maria Oliviacastedo	2003
11578	1	1	1	145	Charlottekempenaers-pocz	2004
11579	1	1	1	146	Hibahshaikh	2002
11580	1	1	1	147	Alexisblokhina	2004
11581	1	1	1	148	Melisaercan	2005
11582	1	1	1	149	Barakat Oyinlomoquadre	2003
11583	1	1	1	150	Esztermeri	2002
11584	1	1	1	151	Inesbekrar	2003
11585	1	1	1	152	Kirapavlova	2004
11586	1	1	1	153	Skyler Marie Gracegrishuk	2002
11587	1	1	1	154	Polawygonowska	2003
11588	1	1	1	155	Jimar Geraldinegerald gonzalez	2002
11589	1	1	1	156	Nomanoha akugue	2003
11590	1	1	1	157	Valenciaxu	2003
11591	1	1	1	158	Antoniaruzic	2003
11592	1	1	1	159	Nadong	2003
11593	1	1	1	160	Yu-Yunli	2004
11594	1	1	1	161	Annerlygeorgopoulos	2003
11595	1	1	1	162	Yarabartashevich	2005
11596	1	1	1	163	Kaylacross	2005

11597	1	1	1	164	Alexiaharmon	2005
11598	1	1	1	165	Shavitkimchi	2002
11599	1	1	1	166	Amelievan impe	2004
11600	1	1	1	167	Laura Isabelputz	2003
11601	1	1	1	168	Denislavaglushkova	2004
11602	1	1	1	169	Patricijaspaka	2002
11603	1	1	1	170	Anchisachanta	2002
11604	1	1	1	171	Nevenacarton	2003
11605	1	1	1	172	Elenakarner	2003
11606	1	1	1	173	Camilasoares	2003
11607	1	1	1	174	Evagartseva	2003
11608	1	1	1	175	Anastasiiagureva	2005
11609	1	1	1	176	Miarepac	2004
11610	1	1	1	177	Yujiahuang	2002
11611	1	1	1	178	BoYoungjeong	2003
11612	1	1	1	179	Camillazanolini	2003
11613	1	1	1	180	Avacatanzarite	2002
11614	1	1	1	181	Lanlanatararudee	2004
11615	1	1	1	182	Clarinelerby	2003
11616	1	1	1	183	Kaileyevans	2003
11617	1	1	1	184	Taramalik	2002
11618	1	1	1	185	Yelizavetakarlova	2003
11619	1	1	1	186	Kuan Yilee	2002
11620	1	1	1	187	Ekaterinakhayrutdinova	2004
11621	1	1	1	188	Fangrantian	2003
11622	1	1	1	189	Tijanasretenovic	2005
11623	1	1	1	190	Elenapridankina	2005
11624	1	1	1	191	Rebeccalynn	2004
11625	1	1	1	192	Dashaplekhanova	2004
11626	1	1	1	193	Yu chintsai	2003
11627	1	1	1	194	Maomushika	2005
11628	1	1	1	195	Kseniazaytseva	2004
11629	1	1	1	196	Miakupres	2004
11630	1	1	1	197	Daiannehayashida	2003
11631	1	1	1	198	Vipashamehra	2002
11632	1	1	1	199	Melis Aydauyar	2003
11633	1	1	1	200	Yasminezzat	2003
11634	1	1	1	201	Miomushika	2005
11635	1	1	1	202	Matildejorge	2004
11636	1	1	1	203	Wiemboubaker	2003
11637	1	1	1	204	Emmajackson	2002
11638	1	1	1	205	Melissaboyden	2003
11639	1	1	1	206	Ana Paulanaranjo martinez kob	2002
11640	1	1	1	207	Nikolabartunkova	2006
11641	1	1	1	208	Zuzannakubacha	2004
11642	1	1	1	209	Klaudijabubelyte	2003
11643	1	1	1	210	Carolinakuhl	2005
11644	1	1	1	211	Nadezdakhalturina	2004
11645	1	1	1	212	Sabinerutlauka	2002
11646	1	1	1	213	Fionaarrese mata	2002

11647	1	1	1	214	Mikadagan fruchtman	2003
11648	1	1	1	215	Katerinamandelikova	2003
11649	1	1	1	216	Sarasuchankova	2003
11650	1	1	1	217	Punninkovapitukted	2003
11651	1	1	1	218	Asiaserafini	2003
11652	1	1	1	219	Anastasiyafeklistova	2003
11653	1	1	1	220	Laiapetretic	2004
11654	1	1	1	221	Juliettebovy	2004
11655	1	1	1	222	Anna Lorielemongo toumbou	2003
11656	1	1	1	223	Manamiukita	2002
11657	1	1	1	224	Salakthipounmuang	2005
11658	1	1	1	225	Marinastakusic	2004
11659	1	1	1	226	Sijiawei	2003
11660	1	1	1	227	Haniaabouelsaad	2003
11661	1	1	1	228	Aishaniyonkuru	2002
11662	1	1	1	229	Dimitrapavlou	2004
11663	1	1	1	230	Saraziodato	2002
11664	1	1	1	231	Kyliebilchev	2003
11665	1	1	1	232	Sara Valentinacruz bonilla	2003
11666	1	1	1	233	Feryelben hassen	2004
11667	1	1	1	234	Mariacharl	2003
11668	1	1	1	235	Sofiarocchetti	2002
11669	1	1	1	236	Dariashalamanova	2002
11670	1	1	1	237	Clerviengounoue	2006
11671	1	1	1	238	Chelseafontenel	2004
11672	1	1	1	239	Dariakrasnova	2002
11673	1	1	1	240	Kimmihance	2003
11674	1	1	1	241	Juliestruplova	2004
11675	1	1	1	242	Dakotafordham	2002
11676	1	1	1	243	Natasharussell	2002
11677	1	1	1	244	Jaedanbrown	2002
11678	1	1	1	245	Sarabejlek	2006
11679	1	1	1	246	Fatmaidrizovic	2004
11680	1	1	1	247	Vanessa Ioana Georgianapopa t	2004
11681	1	1	1	248	Elzatomase	2002
11682	1	1	1	249	Rozaliagruszczynska	2004
11683	1	1	1	250	Giannapielet	2002
11684	1	1	1	251	Elizavetashebekina	2003
11685	1	1	1	252	Yvonnazuffova	2002
11686	1	1	1	253	Charlotteowensby	2003
11687	1	1	1	254	Ninaradovanovic	2003
11688	1	1	1	255	Loisboisson	2003
11689	1	1	1	256	Ozlemuslu	2004
11690	1	1	1	257	Elizavetagavrilova	2003
11691	1	1	1	258	Ancatodoni	2004
11692	1	1	1	259	Joelle Lilly Sophiesteur	2004
11693	1	1	1	260	Charlottechavatipon	2002
11694	1	1	1	261	Ariannazucchini	2003
11695	1	1	1	262	Dariakhomutsianskaya	2004
11696	1	1	1	263	Saraakid	2003

11697	1	1	1	264	Qavialopez	2005
11698	1	1	1	265	TIANMImi	2004
11699	1	1	1	266	Violettaborodina	2004
11700	1	1	1	267	Priscilajanikian	2003
11701	1	1	1	268	Vandavargova	2003
11702	1	1	1	269	Giuliamartinelli	2004
11703	1	1	1	270	Dominikasalkova	2004
11704	1	1	1	271	Angellaokutoyi	2004
11705	1	1	1	272	Emelinede witte	2002
11706	1	1	1	273	Ana Paulajimenez coello	2002
11707	1	1	1	274	Sofiamendonca	2002
11708	1	1	1	275	Nanarikatsumi	2003
11709	1	1	1	276	Tshegofatsotsiang	2002
11710	1	1	1	277	Laravan der merwe	2002
11711	1	1	1	278	Julianamunhoz	2003
11712	1	1	1	279	Mariapetrovic	2002
11713	1	1	1	280	Victoriaborodulina	2002
11714	1	1	1	281	Danielladimitrova	2002
11715	1	1	1	282	Polinapavlova	2003
11716	1	1	1	283	Kristynalavickova	2002
11717	1	1	1	284	Aysegulmert	2004
11718	1	1	1	285	Elisewagle	2003
11719	1	1	1	286	Lucaudvardy	2005
11720	1	1	1	287	Sashawood	2002
11721	1	1	1	288	Hoi Ki Jennywong	2002
11722	1	1	1	289	Ioanazvonaru	2004
11723	1	1	1	290	Ellahaavisto	2002
11724	1	1	1	291	Kristynatomajkova	2005
11725	1	1	1	292	Giorgiapedone	2004
11726	1	1	1	293	Nellyknezkova	2003
11727	1	1	1	294	Fionacrawley	2002
11728	1	1	1	295	Irinadshandshgava	2002
11729	1	1	1	296	Lucijaciric bagaric	2004
11730	1	1	1	297	Miaslama	2006
11731	1	1	1	298	Kristinavolgapkina	2004
11732	1	1	1	299	Supapitchkuearum	2002
11733	1	1	1	300	Alevtinaibragimova	2005
11734	1	1	1	301	Isabeloliveira	2002
11735	1	1	1	302	Ameliawaligora	2005
11736	1	1	1	303	Anastasiyakomar	2003
11737	1	1	1	304	Annazyryanova	2004
11738	1	1	1	305	Julinefayard	2003
11739	1	1	1	306	Katjawiersholm	2004
11740	1	1	1	307	Maelleleclercq	2004
11741	1	1	1	308	Salmaloudili	2002
11742	1	1	1	309	Brendafruhvirtova	2007
11743	1	1	1	310	Victoriamboko	2006
11744	1	1	1	311	Annagrigoreva	2003
11745	1	1	1	312	Natashasengphrachanh	2002
11746	1	1	1	313	Lucajanosi	2002

11747	1	1	1	314	Sayakaishii	2005
11748	1	1	1	315	Leenabennetto	2003
11749	1	1	1	316	Nahiaberecoechea	2004
11750	1	1	1	317	Delienkleinhans	2002
11751	1	1	1	318	Celinenaef	2005
11752	1	1	1	319	Chia Yitsao	2003
11753	1	1	1	320	Aruzhansagandikova	2004
11754	1	1	1	321	Jadeotway	2003
11755	1	1	1	322	Xichenzhao	2003
11756	1	1	1	323	Luciallinares domingo	2003
11757	1	1	1	324	Melodyhefti	2002
11758	1	1	1	325	Leyla Fiorellabritez risso	2004
11759	1	1	1	326	Isabellaweatherhead	2002
11760	1	1	1	327	Anastasiiasobolieva	2004
11761	1	1	1	328	Sarahhamner	2003
11762	1	1	1	329	Anouckvrancken peeters	2003
11763	1	1	1	330	Vivianyang	2005
11764	1	1	1	331	Martynaostrzygalo	2005
11765	1	1	1	332	Sarasvetac	2004
11766	1	1	1	333	Gabrielamacias	2002
11767	1	1	1	334	Luciapeyre	2005
11768	1	1	1	335	Mariashusharina	2002
11769	1	1	1	336	Zijunjiang	2005
11770	1	1	1	337	Sabrinakalandarov	2003
11771	1	1	1	338	Tamarigagoshidze	2003
11772	1	1	1	339	Emmaaucagne	2003
11773	1	1	1	340	Eleanayu	2004
11774	1	1	1	341	Katerinadimitrova	2004
11775	1	1	1	342	Ericadi battista	2002
11776	1	1	1	343	Arianageerlings	2005
11777	1	1	1	344	Amandinemonnot	2002
11778	1	1	1	345	Kiraramorioka	2003
11779	1	1	1	346	Avakrug	2005
11780	1	1	1	347	Bentespee	2003
11781	1	1	1	348	Cara Mariamester	2005
11782	1	1	1	349	Danielletuhten	2004
11783	1	1	1	350	Setoranormurodova	2002
11784	1	1	1	351	Sarahmueller	2004
11785	1	1	1	352	Jerminesherif	2005
11786	0	1	1	353	Elenamicic	2004
11787	0	1	1	354	Tomokokato	2003
11788	0	1	1	355	Sheena Jademasuda karrasch	2003
11789	0	1	1	356	Aitiyaguliaixirefu	2003
11790	0	1	1	357	Denisehrdinkova	2003
11791	0	1	1	358	Nikolpalecek	2003
11792	0	1	1	359	Roopabains	2002
11793	0	1	1	360	Carolanndelaunay	2004
11794	0	1	1	361	Corinaspasojevic	2002
11795	0	1	1	362	Lissikubre	2002
11796	0	1	1	363	Marianamanyoma velasquez	2003

11797	0	1	1	364	Ayael aouni	2005
11798	0	1	1	365	Catherineaulia	2004
11799	0	1	1	366	Hwiwonwi	2002
11800	0	1	1	367	Kseniaagureeva	2004
11801	0	1	1	368	Claudiade las heras armenteras	2003
11802	0	1	1	369	Maeliemonfils	2005
11803	0	1	1	370	Florentinedekkers	2004
11804	0	1	1	371	Xin Tongwang	2004
11805	0	1	1	372	Naroaaranzabal	2005
11806	0	1	1	373	Ninonatsvlishvili	2003
11807	0	1	1	374	Isabelaalvarez mercante	2002
11808	0	1	1	375	Viktoryiakanapatskaya	2002
11809	0	1	1	376	Johanne Christinesvendsen	2004
11810	0	1	1	377	Nikitavishwase	2004
11811	0	1	1	378	Mariadzemeshkevich	2003
11812	0	1	1	379	Lizettereding	2002
11813	0	1	1	380	Patricijapaukstyte	2004
11814	0	1	1	381	Minorkamiranda	2002
11815	0	1	1	382	Agnesmitteregger	2004
11816	0	1	1	383	Conniema	2003
11817	0	1	1	384	Elainechervinsky	2003
11818	0	1	1	385	Tanishakashyap	2002
11819	0	1	1	386	Ranimrassil	2006
11820	0	1	1	387	Arinavalitova	2004
11821	0	1	1	388	Alinatikhonova	2002
11822	0	1	1	389	Kristinakudryavtseva	2003
11823	0	1	1	390	Carolinagomez	2004
11824	0	1	1	391	Romarycardenas rifka	2002
11825	0	1	1	392	Qianyuliu	2004
11826	0	1	1	393	Aleksandrawierzbowska	2002
11827	0	1	1	394	Koharuniimi	2002
11828	0	1	1	395	Gabrielacortes flores	2004
11829	0	1	1	396	Anastasiakovaleva	2002
11830	0	1	1	397	Mayapitts	2003
11831	0	1	1	398	Gabriellasoliman	2003
11832	0	1	1	399	Mariiahlahola	2004
11833	0	1	1	400	Renatafarima	2002
11834	0	1	1	401	Maria Camilatorres murcia	2002
11835	0	1	1	402	Selin Lidyasepken	2003
11836	0	1	1	403	Vivianovrootsky	2004
11837	0	1	1	404	Sinjakraus	2002
11838	0	1	1	405	Matildemariani	2002
11839	0	1	1	406	Anarodrigues	2002
11840	0	1	1	407	Kyliecollins	2002
11841	0	1	1	408	Francescacurmi	2002
11842	0	1	1	409	Camillabossi	2003
11843	0	1	1	410	Bianca Joliefernandez	2004
11844	0	1	1	411	Jenniferkida	2002
11845	0	1	1	412	Sarahdev	2003
11846	0	1	1	413	Hanshi	2005

11847	0	1	1	414	Miaristic	2006
11848	0	1	1	415	Victoriamatasova	2004
11849	0	1	1	416	Tealukic	2004
11850	0	1	1	417	Marethaburger	2002
11851	0	1	1	418	Ameliesmejkalova	2005
11852	0	1	1	419	Gabriellabroadfoot	2004
11853	0	1	1	420	Yifansun	2004
11854	0	1	1	421	Raquelgonzalez vilar	2004
11855	0	1	1	422	Zoebillon	2002
11856	0	1	1	423	Miachudejova	2003
11857	0	1	1	424	Milanazhabrailova	2003
11858	0	1	1	425	Dariyaradulova	2003
11859	0	1	1	426	Chao-Yiwang	2002
11860	0	1	1	427	Sophieschouten	2004
11861	0	1	1	428	Lucianamoyano	2005
11862	0	1	1	429	Sarahiliev	2006
11863	0	1	1	430	Semraaksu	2003
11864	0	1	1	431	Elenatrencheva	2002
11865	0	1	1	432	Ekaterinavinnik	2002
11866	0	1	1	433	Isabellacolmenares b	2004
11867	0	1	1	434	Salmadrugdova	2004
11868	0	1	1	435	Terezavalentova	2007
11869	0	1	1	436	Yajingcao	2002
11870	0	1	1	437	Maria Doloreslopez martinez	2003
11871	0	1	1	438	Andreanova	2003
11872	0	1	1	439	Madeleinejessup	2005
11873	0	1	1	440	Anagrubor	2004
11874	0	1	1	441	Dariayesypchuk	2005
11875	0	1	1	442	Kyokakubo	2004
11876	0	1	1	443	Yujinkim	2005
11877	0	1	1	444	Miyukakimoto	2002
11878	0	1	1	445	Dariabutsko	2003
11879	0	1	1	446	Nicolekiefer	2002
11880	0	1	1	447	Thamonpanjonglertrakul	2003
11881	0	1	1	448	Shioriito	2002
11882	0	1	1	449	Aleksandrasimeva	2004
11883	0	1	1	450	Xinyuehu	2002
11884	0	1	1	451	Anastasiaabbagnato	2003
11885	0	1	1	452	Chloenoel	2004
11886	0	1	1	453	Elizavetamorozova	2004
11887	0	1	1	454	Anitasahdiieva	2004
11888	0	1	1	455	Stefanijapolischuk	2002
11889	0	1	1	456	Leyla Niluferelmas	2005
11890	0	1	1	457	Vladlenabokova	2002
11891	0	1	1	458	Mariyasinitsyna	2003
11892	0	1	1	459	Sofyaostrovskaya	2003
11893	0	1	1	460	Hlael-sakka	2003
11894	0	1	1	461	Anastasiiagrechkina	2005
11895	0	1	1	462	Melisasenli	2004
11896	0	1	1	463	Marieweckerle	2003

11897	0	1	1	464	Le Yiliu	2003
11898	0	1	1	465	Annaparadisi	2005
11899	0	1	1	466	Meshkatolzahrasafi	2004
11900	0	1	1	467	Evaelbaz	2004
11901	0	1	1	468	Sarah Lisaaubertin	2002
11902	0	1	1	469	Nathaliemokhtar	2003
11903	0	1	1	470	Patriciekubikova	2004
11904	0	1	1	471	Valeriacarreno	2004
11905	0	1	1	472	Anastasiaastakhova	2002
11906	0	1	1	473	Gabriellaprice	2003
11907	0	1	1	474	Tildestromquist	2004
11908	0	1	1	475	Heddagurholt	2002
11909	0	1	1	476	Xeniabandurowska	2004
11910	0	1	1	477	Bianca Elenabarbulescu	2003
11911	0	1	1	478	Sarasaito	2006
11912	0	1	1	479	Ana Carmenzamburek	2003
11913	0	1	1	480	Natalijasenic	2003
11914	0	1	1	481	Sarah-Maudefortin	2002
11915	0	1	1	482	Raluca Mariatudorache	2002
11916	0	1	1	483	Luca Victoriavocke	2004
11917	0	1	1	484	Annagross	2002
11918	0	1	1	485	Brooklynolson	2006
11919	0	1	1	486	Hannahchambers	2004
11920	0	1	1	487	Sonyamacavei	2004
11921	0	1	1	488	Zdenasafarova	2003
11922	0	1	1	489	Jenniferriester	2002
11923	0	1	1	490	Avelinasayfetdinova	2002
11924	0	1	1	491	Ellaseidel	2005
11925	0	1	1	492	Alinakhvatova	2004
11926	0	1	1	493	Deannechoo	2003
11927	0	1	1	494	Danabaidaulet	2005
11928	0	1	1	495	Anastasiyalopata	2004
11929	0	1	1	496	Dariasoldatova	2002
11930	0	1	1	497	Cadencebrace	2005
11931	0	1	1	498	Milenajevtovic	2004
11932	0	1	1	499	Mayabetzalel	2004
11933	0	1	1	500	Sandugashkenzhibayeva	2005
11934	0	1	1	501	Karolinakozakova	2005
11935	0	1	1	502	Katerynalazarenko	2004
11936	0	1	1	503	Leighvan zyl	2004
11937	0	1	1	504	Simonaogescu	2002
11938	0	1	1	505	Jeongmoon	2002
11939	0	1	1	506	Juliesappl	2002
11940	0	1	1	507	Catherine Isabelledenysiewicz s	2003
11941	0	1	1	508	Min Chaekim	2002
11942	0	1	1	509	Kokomikoyama	2002
11943	0	1	1	510	Josefa Rosariofernandez	2002
11944	0	1	1	511	Klaranovakova	2002
11945	0	1	1	512	Anacandiotto	2004
11946	0	1	1	513	Maria Julianaparra romero	2002

11947	0	1	1	514	Karinajumazhanova	2006
11948	0	1	1	515	Anjanayar	2005
11949	0	1	1	516	Yuhanliu	2003
11950	0	1	1	517	Abbymason	2003
11951	0	1	1	518	Larawalker	2003
11952	0	1	1	519	Carolinaxavier laydner	2005
11953	0	1	1	520	Jeanettemireles	2003
11954	0	1	1	521	Hsuanhuang	2002
11955	0	1	1	522	Krystalblanch	2004
11956	0	1	1	523	Vanessaersoz	2002
11957	0	1	1	524	Sanjanasirimalla	2004
11958	0	1	1	525	Anet Angelikakoskel	2003
11959	0	1	1	526	Emilijatverijonaite	2004
11960	0	1	1	527	Liakaratancheva	2003
11961	0	1	1	528	Maria Sarapopa	2005
11962	0	1	1	529	Dinaraalloyarova	2003
11963	0	1	1	530	Izabellepersson	2003
11964	0	1	1	531	Virginiaferrara	2004
11965	0	1	1	532	Ranah Akuastoiber	2005
11966	0	1	1	533	Ekaterinatikhonko	2003
11967	0	1	1	534	Isabellepapazyan	2002
11968	0	1	1	535	Qinglinzang	2004
11969	0	1	1	536	Denisevalente	2004
11970	0	1	1	537	Smritibhasin	2002
11971	0	1	1	538	Sophiewilliams	2004
11972	0	1	1	539	Vichitrapornvimuktananda	2005
11973	0	1	1	540	Yujiaoche	2003
11974	0	1	1	541	Ralitsaalexandrova	2002
11975	0	1	1	542	Marianacampino	2003
11976	0	1	1	543	Miamack	2004
11977	0	1	1	544	Jademarie	2004
11978	0	1	1	545	Hanagamracy	2003
11979	0	1	1	546	Vasilisalisina	2003
11980	0	1	1	547	Tereziepluharova	2002
11981	0	1	1	548	Kellyarends	2003
11982	0	1	1	549	Astridcirotte	2003
11983	0	1	1	550	Kamiliashailina	2002
11984	0	1	1	551	Sandeepti Singhrao	2003
11985	0	1	1	552	Meng Yichen	2003
11986	0	1	1	553	Weronikaewald	2006
11987	0	1	1	554	Evaprokopenko	2002
11988	0	1	1	555	Elenakraleva	2002
11989	0	1	1	556	Noraknezevic	2005
11990	0	1	1	557	Gracieepps	2004
11991	0	1	1	558	Violetamartinez	2004
11992	0	1	1	559	Gretaschieroni	2002
11993	0	1	1	560	Yuxuanyang	2004
11994	0	1	1	561	Ninadanisova	2003
11995	0	1	1	562	Keren	2005
11996	0	1	1	563	Francesca Andreafeodorov	2003

11997	0	1	1	564	Maria Fernandamartinez herna	2004
11998	0	1	1	565	Annasemenova	2004
11999	0	1	1	566	Reshmamaruri	2003
12000	0	1	1	567	Faustazykute	2002
12001	0	1	1	568	Divnaratkovic	2004
12002	0	1	1	569	Karinarizvanova	2003
12003	0	1	1	570	Gabrielagiraldo	2002
12004	0	1	1	571	Margaritaandreiuk	2003
12005	0	1	1	572	Mihaelatsoneva	2004
12006	0	1	1	573	Chiaragirelli	2002
12007	0	1	1	574	Isabellakruger	2005
12008	0	1	1	575	Noadujardin	2003
12009	0	1	1	576	Sageloudon	2005
12010	0	1	1	577	Mingqiaoyang	2002
12011	0	1	1	578	Oliviasymons	2002
12012	0	1	1	579	Sarazalukar	2002
12013	0	1	1	580	Viktoriaveleva	2004
12014	0	1	1	581	Mialyranaivo	2003
12015	0	1	1	582	Alisabudanova	2002
12016	0	1	1	583	Stellaremander	2005
12017	0	1	1	584	Lundakumhom	2002
12018	0	1	1	585	Allurazamarripa	2002
12019	0	1	1	586	Jadepsonka	2005
12020	0	1	1	587	Mariyapolishchuk	2002
12021	0	1	1	588	Sofiablanco	2003
12022	0	1	1	589	Paulacembranos	2004
12023	0	1	1	590	Iuliiatrifonova	2003
12024	0	1	1	591	Jenniferruggeri	2003
12025	0	1	1	592	Alexandrapetric	2004
12026	0	1	1	593	Carolplakk	2002
12027	0	1	1	594	Maria Ceciliaaguiar	2002
12028	0	1	1	595	Mariyadolzhenko	2005
12029	0	1	1	596	Ji Woochoi	2004
12030	0	1	1	597	Karynakachur	2003
12031	0	1	1	598	Namieisago	2002
12032	0	1	1	599	Carlagalmiche	2004
12033	0	1	1	600	Cheridarley	2003
12034	0	1	1	601	Reececarter	2004
12035	0	1	1	602	Danielaglanzer	2003
12036	0	1	1	603	Charlotterussell	2002
12037	0	1	1	604	Paulinafranco martinessi	2004
12038	0	1	1	605	Emabolebruchova	2003
12039	0	1	1	606	Luanaavelar	2003
12040	0	1	1	607	Agatacerna	2004
12041	0	1	1	608	Christinekruger	2003
12042	0	1	1	609	Anikajaskova	2003
12043	0	1	1	610	Yataweechimcham	2002
12044	0	1	1	611	Maria Inesfonte	2002
12045	0	1	1	612	Maryamahmad	2002
12046	0	1	1	613	Jillian Louiseroa	2003

12047	0	1	1	614	Majadodik	2002
12048	0	1	1	615	Ninavargova	2005
12049	0	1	1	616	Olgamishenina	2003
12050	0	1	1	617	Helenastevic	2002
12051	0	1	1	618	Yuranakagawa	2003
12052	0	1	1	619	Taragorinsek	2002
12053	0	1	1	620	Michelle Kellytikhonko	2003
12054	0	1	1	621	Carlottamoccia	2003
12055	0	1	1	622	Alliegretkowski	2003
12056	0	1	1	623	Yizhenzheng	2003
12057	0	1	1	624	Yimingdang	2004
12058	0	1	1	625	Pawineeruamrak	2003
12059	0	1	1	626	Wei Ningfang	2002
12060	0	1	1	627	Veronikasolovyeva	2003
12061	0	1	1	628	Wing Kalin	2002
12062	0	1	1	629	Umabakaityte	2004
12063	0	1	1	630	Mengkeli	2004
12064	0	1	1	631	Leahkuruvilla	2004
12065	0	1	1	632	Fatima Yareligutierrez martinez	2004
12066	0	1	1	633	Kseniaaleshina	2003
12067	0	1	1	634	Tjasaklevisar	2002
12068	0	1	1	635	Emmacharney	2003
12069	0	1	1	636	Xintongzhou	2005
12070	0	1	1	637	Defnecirpanli	2004
12071	0	1	1	638	Ilinca Dalinaamariei	2002
12072	0	1	1	639	Mavieosterreicher	2002
12073	0	1	1	640	Chui Keileung	2003
12074	0	1	1	641	Nikastrasek	2003
12075	0	1	1	642	Arianaarseneault	2002
12076	0	1	1	643	Hibaheni	2006
12077	0	1	1	644	Sophia Natalyabaranov	2004
12078	0	1	1	645	Laviniatanasie	2003
12079	0	1	1	646	Xiaoyanxue	2004
12080	0	1	1	647	Darjasuvirdjonkova	2005
12081	0	1	1	648	Prernavichare	2002
12082	0	1	1	649	Suanatucakovic	2004
12083	0	1	1	650	Stephaniesanchez	2002
12084	0	1	1	651	Andreaobradovic	2004
12085	0	1	1	652	Skiakung	2003
12086	0	1	1	653	Maryloveedwards	2005
12087	0	1	1	654	Fang Anlin	2005
12088	0	1	1	655	Ninastankovska	2002
12089	0	1	1	656	Valeriiaivanovskaia	2004
12090	0	1	1	657	Valentinamutilba	2002
12091	0	1	1	658	Liisavarul	2004
12092	0	1	1	659	Tarazoppas	2004
12093	0	1	1	660	Sofiyachekhlystova	2002
12094	0	1	1	661	Elenakorokozidi	2003
12095	0	1	1	662	Kristinapaskauskas	2004
12096	0	1	1	663	Vanesa Dalmadanko	2005

12097	0	1	1	664	Chiarafornasieri	2003
12098	0	1	1	665	Jasmineadams	2002
12099	0	1	1	666	Anastasiyazholdakova	2004
12100	0	1	1	667	Hannavinahradava	2003
12101	0	1	1	668	Deborahmuratovic	2003
12102	0	1	1	669	Stefaniabojica	2005
12103	0	1	1	670	Rachelgailis	2004
12104	0	1	1	671	Alexandraiordache	2003
12105	0	1	1	672	Stefaniefrias da silva	2003
12106	0	1	1	673	Karolinanovakova	2002
12107	0	1	1	674	Sarasakaneko	2003
12108	0	1	1	675	Andrea Mariaartimedi	2003
12109	0	1	1	676	Adapiestrzynska	2004
12110	0	1	1	677	Malwinarowinska	2005
12111	0	1	1	678	Olgagolas	2003
12112	0	1	1	679	Camillaraggi	2002
12113	0	1	1	680	Rebeccamunk mortensen	2005
12114	0	1	1	681	Mbalilanga	2002
12115	0	1	1	682	Orynastoliarchuk	2002
12116	0	1	1	683	Silvia Mariacostache	2003
12117	0	1	1	684	Lucianablatter	2003
12118	0	1	1	685	Laylashoukry	2006
12119	0	1	1	686	Aubreynisbet	2005
12120	0	1	1	687	Klaravukicevic	2003
12121	0	1	1	688	Dorsacheraghi	2005
12122	0	1	1	689	Federicaurgesi	2005
12123	0	1	1	690	Dariazelinskaya	2004
12124	0	1	1	691	Dianalemanova	2005
12125	0	1	1	692	Louisewikander	2003
12126	0	1	1	693	Calissadellabarca	2003
12127	0	1	1	694	Honokaumeda	2004
12128	0	1	1	695	Federicasacco	2002
12129	0	1	1	696	Mariajosehernandez coronado	2002
12130	0	1	1	697	Gauribhagia	2002
12131	0	1	1	698	Cora Stefaniebarber	2002
12132	0	1	1	699	Zhanelrustemova	2004
12133	0	1	1	700	Daniquehavermans	2002
12134	0	1	1	701	Petjadrame	2004
12135	0	1	1	702	Liamoved	2005
12136	0	1	1	703	Darialopatetska	2003
12137	0	1	1	704	Kseniakhlynova	2002
12138	0	1	1	705	Dariarepina	2003
12139	0	1	1	706	Arinababkova	2003
12140	0	1	1	707	Riabhakta	2005
12141	0	1	1	708	Shachflieberman	2003
12142	0	1	1	709	Nikoladaubnerova	2005
12143	0	1	1	710	Elenifasoula	2002
12144	0	1	1	711	Eleanorbaglow	2002
12145	0	1	1	712	Ine Soltveitstange	2002
12146	0	1	1	713	Serafimashastova	2004

12147	0	1	1	714	Leydengames	2003
12148	0	1	1	715	Minatoglukdemir	2003
12149	0	1	1	716	Adajane dealcantara	2005
12150	0	1	1	717	Jackelinelopez	2002
12151	0	1	1	718	Arieljohnson	2003
12152	0	1	1	719	Mary Gracearmistead	2002
12153	0	1	1	720	Inesfaltinger	2003
12154	0	1	1	721	Magdalenaswierczynska	2004
12155	0	1	1	722	Jiminkwon	2003
12156	0	1	1	723	Amelie-Christinjanssen	2003
12157	0	1	1	724	Johannastrom	2003
12158	0	1	1	725	Amelie Clairemontalvo lundy	2004
12159	0	1	1	726	Malikaamarathe	2003
12160	0	1	1	727	Carsontanguilig	2003
12161	0	1	1	728	Yaellevaissaud	2004
12162	0	1	1	729	Iuliiaiudenko	2004
12163	0	1	1	730	Claracwajgenbaum	2005
12164	0	1	1	731	Vladislavapetrovskaia	2003
12165	0	1	1	732	Manalennaciri	2005
12166	0	1	1	733	Annachekanskaya	2003
12167	0	1	1	734	Oliviamalm	2002
12168	0	1	1	735	Carolinewernli	2003
12169	0	1	1	736	Laraschneider	2002
12170	0	1	1	737	Luccianaperez alarcon	2005
12171	0	1	1	738	Emmavalletta	2004
12172	0	1	1	739	Jaquelynogunwale	2005
12173	0	1	1	740	Haoyanwu	2004
12174	0	1	1	741	Mananchayasawangkaew	2002
12175	0	1	1	742	Wardaait el bachir	2004
12176	0	1	1	743	Jagmeetkaur	2002
12177	0	1	1	744	Abhilashabista	2003
12178	0	1	1	745	Rufaromagarira	2003
12179	0	1	1	746	Xinxinyao	2003
12180	0	1	1	747	Lilly Paulineschultz	2004
12181	0	1	1	748	Graceo'donnell	2002
12182	0	1	1	749	Vanessaong	2002
12183	0	1	1	750	Danaissabayeva	2002
12184	0	1	1	751	Mariaberlanga bandera	2003
12185	0	1	1	752	Sihyeongcho	2002
12186	0	1	1	753	Ilaydabaykan	2002
12187	0	1	1	754	Amyleather	2003
12188	0	1	1	755	Aljabelinger	2002
12189	0	1	1	756	Albarey garcia	2002
12190	0	1	1	757	Elizavetadementyeva	2002
12191	0	1	1	758	Vaishnaviadkar	2004
12192	0	1	1	759	Ludmilakareisova	2002
12193	0	1	1	760	Emilijazdravkovic	2005
12194	0	1	1	761	Fiorelladuran montano	2004
12195	0	1	1	762	Mahtakhanloo	2002
12196	0	1	1	763	Jessicaplazas	2002

12197	0	1	1	764	Mary Jeanbrumfield	2002
12198	0	1	1	765	Kristenborland	2002
12199	0	1	1	766	Alessiamartinez	2004
12200	0	1	1	767	Anadinu	2002
12201	0	1	1	768	Martinamarkov	2004
12202	0	1	1	769	Daryazubtsouskaya	2003
12203	0	1	1	770	Polinashitikova	2002
12204	0	1	1	771	Kamilaneradova	2002
12205	0	1	1	772	Olgamolchanova	2004
12206	0	1	1	773	Tarachilton	2002
12207	0	1	1	774	Ceciliaalcobe garibay	2003
12208	0	1	1	775	Leabathellier	2005
12209	0	1	1	776	Dainheekim	2004
12210	0	1	1	777	Jenaila Roseprulla	2005
12211	0	1	1	778	Hannatsitavets	2005
12212	0	1	1	779	Anastasiaberezov	2003
12213	0	1	1	780	Nataliaperez	2006
12214	0	1	1	781	Jingyiwang	2003
12215	0	1	1	782	Shuoranwang	2002
12216	0	1	1	783	Inesstephani	2002
12217	0	1	1	784	Katrinpavkova	2005
12218	0	1	1	785	Ke Syuanjhang	2002
12219	0	1	1	786	Jessicaportwig	2002
12220	0	1	1	787	Chiaradi tommaso	2002
12221	0	1	1	788	Ana Karenguadiana campos	2002
12222	0	1	1	789	Martinasmolenova	2003
12223	0	1	1	790	Elisabethjones	2004
12224	0	1	1	791	Jessica Christawirahadipoernon	2003
12225	0	1	1	792	Sarinareinertsen	2002
12226	0	1	1	793	Avahrastar	2002
12227	0	1	1	794	Ana Paulachavez sanchez	2004
12228	0	1	1	795	Audreymoutama	2004
12229	0	1	1	796	Karenmarthiens	2004
12230	0	1	1	797	Mafaldaguedes	2004
12231	0	1	1	798	Li Hsinlin	2005
12232	0	1	1	799	Hyeseoshin	2002
12233	0	1	1	800	Rubina Martade ponti	2002
12234	0	1	1	801	Vineethamummadi	2002
12235	0	1	1	802	Klio Mariaioannou	2005
12236	0	1	1	803	Coraliedebruyne	2004
12237	0	1	1	804	Lauraduhl	2002
12238	0	1	1	805	Elisabethiila	2004
12239	0	1	1	806	Jessieculley	2005
12240	0	1	1	807	Meritxellboix molla	2004
12241	0	1	1	808	Linda Claireeloundou nga	2002
12242	0	1	1	809	Elysetse	2004
12243	0	1	1	810	Victoriahu	2002
12244	0	1	1	811	Hagarramadan	2002
12245	0	1	1	812	Marineszostak	2002
12246	0	1	1	813	Pannabartha	2004

12247	0	1	1	814	Valerygynina	2005
12248	0	1	1	815	Hollyfischer	2003
12249	0	1	1	816	Ekaterinanikiforova	2002
12250	0	1	1	817	Athinapitta	2005
12251	0	1	1	818	Sanneschalekamp	2003
12252	0	1	1	819	Ana Paulamartinez mora	2003
12253	0	1	1	820	Irenamuradyan	2002
12254	0	1	1	821	Isabellarivera ortiz	2004
12255	0	1	1	822	Charlotteflorent	2003
12256	0	1	1	823	Anjalikakurera	2004
12257	0	1	1	824	Erkezhanarystanbekova	2004
12258	0	1	1	825	Maria Fernandamenezes	2002
12259	0	1	1	826	Sevilyuldasheva	2002
12260	0	1	1	827	Natachaschou	2004
12261	0	1	1	828	Tiantsoa Sarahrakotomanga raj	2005
12262	0	1	1	829	Sofia Isabelteran rios	2002
12263	0	1	1	830	Lorenamedeiros cardoso	2003
12264	0	1	1	831	Jingyili	2003
12265	0	1	1	832	Imogenhaddad	2005
12266	0	1	1	833	Lucywebber	2003
12267	0	1	1	834	Jiayuxu	2003
12268	0	1	1	835	Deborahdominguez collado	2005
12269	0	1	1	836	Annasedysheva	2005
12270	0	1	1	837	Isseypurser	2004
12271	0	1	1	838	Aglayafedorova	2004
12272	0	1	1	839	Flohelsen	2005
12273	0	1	1	840	Andreale	2003
12274	0	1	1	841	Constancelevivier	2005
12275	0	1	1	842	Sofiiasuslova	2004
12276	0	1	1	843	Amalgati	2003
12277	0	1	1	844	Sarahl'allier	2005
12278	0	1	1	845	Kanamihayashige	2004
12279	0	1	1	846	Aislingo'connor	2003
12280	0	1	1	847	Givenroach	2005
12281	0	1	1	848	Tiziana Marieschomburg	2003
12282	0	1	1	849	Elenamalykh	2003
12283	0	1	1	850	Anastasiarakita	2002
12284	0	1	1	851	Andjelapetrovic	2003
12285	0	1	1	852	Na Hyunkang	2004
12286	0	1	1	853	Santastrombach	2002
12287	0	1	1	854	Ivasepa	2004
12288	0	1	1	855	Dairacardenas	2002
12289	0	1	1	856	Samanthamartinez	2003
12290	0	1	1	857	Carolinacoletti	2003
12291	0	1	1	858	Charlottehaas	2004
12292	0	1	1	859	Yunakadowaki	2004
12293	0	1	1	860	Miraykonar	2003
12294	0	1	1	861	Annalytneva	2002
12295	0	1	1	862	Miekevan deventer	2002
12296	0	1	1	863	Ellamcdonald	2005

12297	0	1	1	864	Marianazegada	2003
12298	0	1	1	865	Taylahpreston	2005
12299	0	1	1	866	Millie Maematthews	2003
12300	0	1	1	867	Rachelhanford	2002
12301	0	1	1	868	Mirraandreeva	2007
12302	0	1	1	869	Dilettacherubini	2002
12303	0	1	1	870	Daria Andreeaconstantin	2002
12304	0	1	1	871	Daria Mariamunteanu	2003
12305	0	1	1	872	Valerija Maijakargina	2006
12306	0	1	1	873	Mariamibrahim	2005
12307	0	1	1	874	Marisaschmidt	2002
12308	0	1	1	875	Junebjork	2005
12309	0	1	1	876	Oliviarook	2003
12310	0	1	1	877	Sarahvan emst	2004
12311	0	1	1	878	Evrayildirim	2004
12312	0	1	1	879	Federicatrevisan	2002
12313	0	0	1	880	Maragae	2005
12314	0	0	1	881	Angelinalinnikova	2004
12315	0	0	1	882	Dorkadrahota szabo	2002
12316	0	0	1	883	Anna Sofiahaseth	2004
12317	0	0	1	884	Jennylim	2005
12318	0	0	1	885	Julieschalch	2003
12319	0	0	1	886	Chyaraonia seke	2004
12320	0	0	1	887	Rachelhermanova	2003
12321	0	0	1	888	Tizianarossini	2004
12322	0	0	1	889	Daniellaben-abraham	2004
12323	0	0	1	890	Raquelcaballero chica	2005
12324	0	0	1	891	Charlottecartledge	2002
12325	0	0	1	892	Sofiapizzoni	2003
12326	0	0	1	893	Xinyinong	2004
12327	0	0	1	894	Hsing-Jusung	2002
12328	0	0	1	895	Naonishino	2005
12329	0	0	1	896	Alicjaformella	2003
12330	0	0	1	897	Klaranelander	2002
12331	0	0	1	898	Rionobata	2003
12332	0	0	1	899	Saoirsebreen	2004
12333	0	0	1	900	Oluchukwu Jennifernkili	2002
12334	0	0	1	901	Sashikempster	2003
12335	0	0	1	902	Noamajor	2005
12336	0	0	1	903	Amilajusufbegovic	2002
12337	0	0	1	904	Helenekereselidze	2003
12338	0	0	1	905	Andreaquiroz	2002
12339	0	0	1	906	Teazivic	2005
12340	0	0	1	907	Heikejanse van vuuren	2004
12341	0	0	1	908	YeHuazhou	2003
12342	0	0	1	909	Alinatsyurpalevych	2002
12343	0	0	1	910	Zuzannaszczepanska	2002
12344	0	0	1	911	Valentinaascarrunz	2002
12345	0	0	1	912	Emmajouy	2005
12346	0	0	1	913	Bohoussou Blanche Lili-bellemi	2005

12347	0	0	1	914	Mariasantos	2004
12348	0	0	1	915	Petekomada	2004
12349	0	0	1	916	Nefelizafeiri	2002
12350	0	0	1	917	Laurapasterk	2003
12351	0	0	1	918	Kseniabrich	2002
12352	0	0	1	919	Alexiajacobs	2005
12353	0	0	1	920	Mariapopova	2005
12354	0	0	1	921	Tsehaydriscoll	2005
12355	0	0	1	922	Martelambrecht	2004
12356	0	0	1	923	Artemispados	2003
12357	0	0	1	924	Ha Yeongryu	2003
12358	0	0	1	925	Rinonokuwaki	2004
12359	0	0	1	926	Serenapereyra giubbani	2004
12360	0	0	1	927	Rosaleenalioussef	2003
12361	0	0	1	928	Dianayanotovskaya	2003
12362	0	0	1	929	Tara Katarinamilic	2002
12363	0	0	1	930	Annaparkhomenko	2002
12364	0	0	1	931	Despoinamastrodima	2004
12365	0	0	1	932	Adelmalgonusova	2004
12366	0	0	1	933	Elliepittman	2002
12367	0	0	1	934	Mariastudennikova	2004
12368	0	0	1	935	Hephzibaholuwadare	2007
12369	0	0	1	936	Camila Ceciliamiranda jove	2004
12370	0	0	1	937	Itahabekovic	2002
12371	0	0	1	938	Elaplatenikova	2004
12372	0	0	1	939	Andreapineda	2004
12373	0	0	1	940	Yi Chinwu	2002
12374	0	0	1	941	Olivialincer	2004
12375	0	0	1	942	Zuzannakolonus	2005
12376	0	0	1	943	Larasmejkal	2004
12377	0	0	1	944	Pia Marijarebec	2004
12378	0	0	1	945	Darinakamenoff	2005
12379	0	0	1	946	Emilieelde	2002
12380	0	0	1	947	Rioosoegawa	2004
12381	0	0	1	948	Sofia Cristinataborga rollano	2003
12382	0	0	1	949	Sofiasokhetskaya	2002
12383	0	0	1	950	Rosealix vidal	2005
12384	0	0	1	951	Suzaniepretorius	2004
12385	0	0	1	952	Palinasaulevich	2004
12386	0	0	1	953	Elinazakharova	2003
12387	0	0	1	954	Mariaconstantinou	2003
12388	0	0	1	955	Iuliiaasanova	2002
12389	0	0	1	956	Aiyanupbay	2006
12390	0	0	1	957	Vanesasuarez	2005
12391	0	0	1	958	Moniquebarry	2002
12392	0	0	1	959	Karinhamilton	2002
12393	0	0	1	960	Luciamartinez gomez	2003
12394	0	0	1	961	Mernarefaat	2004
12395	0	0	1	962	Hozianekitambala	2003
12396	0	0	1	963	Kamornwankornsila	2006

12397	0	0	1	964	Cassidymataia	2004
12398	0	0	1	965	Takhminazhanatova	2002
12399	0	0	1	966	Dartadalecka	2002
12400	0	0	1	967	Annikaplaninsek	2003
12401	0	0	1	968	Sofiafortuno	2003
12402	0	0	1	969	Charlottegisclon	2003
12403	0	0	1	970	Riyauboveja	2003
12404	0	0	1	971	Zlatayankovskaya	2003
12405	0	0	1	972	Ling Hsuanwei	2002
12406	0	0	1	973	Onsboubaker	2005
12407	0	0	1	974	Sarapaunovic	2002
12408	0	0	1	975	Juliazhu	2005
12409	0	0	1	976	Norhanhesham	2002
12410	0	0	1	977	Malakasalem	2005
12411	0	0	1	978	Lolamarandel	2002
12412	0	0	1	979	Arinasorokina	2003
12413	0	0	1	980	Brianaszabo	2005
12414	0	0	1	981	Narindra Corrineranaivo	2003
12415	0	0	1	982	Valeriiacherfus	2002
12416	0	0	1	983	Lucierabiot	2002
12417	0	0	1	984	Mia Raevican	2003
12418	0	0	1	985	Ginevraparentini vallega monte	2003
12419	0	0	1	986	Hannahsmith	2004
12420	0	0	1	987	Ma Carmencita Isabelacarlos	2004
12421	0	0	1	988	Bhaktiparwani	2002
12422	0	0	1	989	Edaarli	2004
12423	0	0	1	990	Annakhaykis	2004
12424	0	0	1	991	Mariebrard vidot	2005
12425	0	0	1	992	eunchaekim	2003
12426	0	0	1	993	Emilybi	2003
12427	0	0	1	994	Leilanyipunesso	2004
12428	0	0	1	995	Dogaakyurek	2003
12429	0	0	1	996	Dariamikhaylova	2003
12430	0	0	1	997	Celinesimunyu	2004
12431	0	0	1	998	Laurakoralnik	2004
12432	0	0	1	999	Soo Hajang	2004
12433	0	0	1	1000	Caylinele grange	2003
12434	0	0	1	1001	Tabithaholmes	2005
12435	0	0	1	1002	Emmaeerola	2002
12436	0	0	1	1003	Mariarizzolo	2002
12437	0	0	1	1004	Emabubalo	2004
12438	0	0	1	1005	Nadyadhaneswara	2002
12439	0	0	1	1006	Ninaplihal	2004
12440	0	0	1	1007	Daniellagalvez isaacs	2003
12441	0	0	1	1008	Nishithasaravanan	2004
12442	0	0	1	1009	Marijanamajetic	2004
12443	0	0	1	1010	Elsachaumette	2005
12444	0	0	1	1011	Annakobayashi	2005
12445	0	0	1	1012	Peppiramstedt	2002
12446	0	0	1	1013	Danielaramos castro	2002

12447	0	0	1	1014	Isabelpascual montalvo	2002
12448	0	0	1	1015	Yevaavetisyan	2002
12449	0	0	1	1016	Zorikamorgan	2004
12450	0	0	1	1017	Emiliavalentinsson	2002
12451	0	0	1	1018	Daniela Andreearosca	2003
12452	0	0	1	1019	Tyralithiby	2006
12453	0	0	1	1020	Brinasulin	2004
12454	0	0	1	1021	Erika Wichincheng	2002
12455	0	0	1	1022	Sashasitue	2006
12456	0	0	1	1023	Ayuishibashi	2002
12457	0	0	1	1024	Ioana Dariamacos	2003
12458	0	0	1	1025	Enabaltic	2003
12459	0	0	1	1026	Sophia Desireepiech	2003
12460	0	0	1	1027	Linegreyling	2004
12461	0	0	1	1028	Sarasarinova	2005
12462	0	0	1	1029	Anaisfernandez laaksonen	2005
12463	0	0	1	1030	Mariianikitash	2004
12464	0	0	1	1031	Reneezhang	2006
12465	0	0	1	1032	Elysiawan	2002
12466	0	0	1	1033	Rahmajendoubi	2005
12467	0	0	1	1034	Marthamakantasi	2002
12468	0	0	1	1035	Sophiawang	2004
12469	0	0	1	1036	Lorintilipman	2003
12470	0	0	1	1037	Stelapeeva	2004
12471	0	0	1	1038	Pilartraiber	2002
12472	0	0	1	1039	Tuanatinaztepe	2002
12473	0	0	1	1040	Makaylaloubser	2002
12474	0	0	1	1041	Rochellepienaar	2002
12475	0	0	1	1042	Janavanik	2003
12476	0	0	1	1043	Vitoriabarandas	2002
12477	0	0	1	1044	Polinapaunova	2003
12478	0	0	1	1045	Ayakakoga	2004
12479	0	0	1	1046	Shonanakano	2004
12480	0	0	1	1047	Nevenakolarevic	2003
12481	0	0	1	1048	Mariamatia	2005
12482	0	0	1	1049	Tejatirunelveli	2004
12483	0	0	1	1050	Sofiajohnson	2004
12484	0	0	1	1051	Nanakakijima	2003
12485	0	0	1	1052	Amandaoliveira	2005
12486	0	0	1	1053	Emmyyaron	2002
12487	0	0	1	1054	Denisetorrealba	2003
12488	0	0	1	1055	Milenasumarokova	2002
12489	0	0	1	1056	Reka Katamatko	2003
12490	0	0	1	1057	Talianeilson-gatenby	2005
12491	0	0	1	1058	Asylzhanarystanbekova	2006
12492	0	0	1	1059	Majaradenkovic	2002
12493	0	0	1	1060	Sofia Antonellacaldera	2003
12494	0	0	1	1061	Ela Nalamilic	2006
12495	0	0	1	1062	Taylahlawless	2003
12496	0	0	1	1063	Carlapacot	2005

12497	0	0	1	1064	Karinagustowska	2003
12498	0	0	1	1065	Billurgonlusen	2004
12499	0	0	1	1066	Learupnjak	2004
12500	0	0	1	1067	Elizavetamasnaia	2005
12501	0	0	1	1068	Sofiyalebedeva	2003
12502	0	0	1	1069	Janastojanova	2005
12503	0	0	1	1070	Emmashasteen	2003
12504	0	0	1	1071	Yiwendanzhu	2002
12505	0	0	1	1072	Beaudeleu	2002
12506	0	0	1	1073	Yuyunchen	2005
12507	0	0	1	1074	Matildemorais	2004
12508	0	0	1	1075	Basmalaaboutaleb	2004
12509	0	0	1	1076	Oliviascattini	2002
12510	0	0	1	1077	Amaliastuger	2003
12511	0	0	1	1078	Veda Rajuprapurna	2005
12512	0	0	1	1079	Maria Sofiamadrid rocca	2004
12513	0	0	1	1080	Chen Chenliao	2002
12514	0	0	1	1081	Polinakhartsii	2002
12515	0	0	1	1082	Daryazykova	2005
12516	0	0	1	1083	Warisaralee	2002
12517	0	0	1	1084	Josydaems	2006
12518	0	0	1	1085	Jingyang	2003
12519	0	0	1	1086	Marcelalopez	2004
12520	0	0	1	1087	Victoriasasinka	2004
12521	0	0	1	1088	Rawansherif	2004
12522	0	0	1	1089	Elsaboisson	2003
12523	0	0	1	1090	Ayakahasegawa	2004
12524	0	0	1	1091	Zeinashaaban	2003
12525	0	0	1	1092	Younakim	2002
12526	0	0	1	1093	Tamarakostic	2006
12527	0	0	1	1094	Maria Dacianaciubotaru	2005
12528	0	0	1	1095	Catherinewalker	2005
12529	0	0	1	1096	Bochra Rehabmebarki	2004
12530	0	0	1	1097	Silviamartinez jimenez	2002
12531	0	0	1	1098	Talimalykhin	2004
12532	0	0	1	1099	Taragancev	2002
12533	0	0	1	1100	Katerynarublevska	2003
12534	0	0	1	1101	Theodoraxantava	2003
12535	0	0	1	1102	Sofiacardenas galvis	2003
12536	0	0	1	1103	Nadineabid	2006
12537	0	0	1	1104	Teanikcevic	2005
12538	0	0	1	1105	Hetveechaudhari	2004
12539	0	0	1	1106	Sarahliu	2004
12540	0	0	1	1107	Emeliegabran	2003
12541	0	0	1	1108	Miamarjanovic	2003
12542	0	0	1	1109	Andjelalopicic	2003
12543	0	0	1	1110	Elyangelicaalas	2002
12544	0	0	1	1111	Suhithamaruri	2005
12545	0	0	1	1112	Nicolealfaro	2005
12546	0	0	1	1113	Abigailrencheli	2002

12547	0	0	1	1114	Carringtonhayes	2003
12548	0	0	1	1115	Liliamahbouli	2004
12549	0	0	1	1116	Pauline Gretabruns	2005
12550	0	0	1	1117	Darialavrichenko	2003
12551	0	0	1	1118	Isabellabarrera aguirre	2003
12552	0	0	1	1119	Lidiagonzalez	2003
12553	0	0	1	1120	Melissayakup pour	2004
12554	0	0	1	1121	Andreaburguete beltran	2003
12555	0	0	1	1122	Klaramaciejewska	2004
12556	0	0	1	1123	Milenagevorgyan	2003
12557	0	0	1	1124	Indiannaspink	2002
12558	0	0	1	1125	Vaniadotse	2003
12559	0	0	1	1126	Katemansfield	2004
12560	0	0	1	1127	Larabakhaya	2003
12561	0	0	1	1128	Anastasiyaabramovich	2003
12562	0	0	1	1129	Palomahennicaux	2004
12563	0	0	1	1130	Stsefaniyashabanava	2004
12564	0	0	1	1131	Dariiamonakhova	2003
12565	0	0	1	1132	Ghaidajeribi	2004
12566	0	0	1	1133	Anna Marieweissheim	2004
12567	0	0	1	1134	Mancipal	2005
12568	0	0	1	1135	Johannabernhold	2004
12569	0	0	1	1136	Senatakebe	2002
12570	0	0	1	1137	Zi Wenmu	2002
12571	0	0	1	1138	Manuella Peguyeloundou nga	2002
12572	0	0	1	1139	Elenakim	2003
12573	0	0	1	1140	Loubaudouin	2004
12574	0	0	1	1141	Yuliazhytelna	2003
12575	0	0	1	1142	Emmapenne	2004
12576	0	0	1	1143	Sue Yantan	2006
12577	0	0	1	1144	Astridlew yan foon	2005
12578	0	0	1	1145	Sifabutcher	2002
12579	0	0	1	1146	Hindsemlali	2002
12580	0	0	1	1147	Camilaibarra paolone	2003
12581	0	0	1	1148	Sydniratliff	2003
12582	0	0	1	1149	Luciapinkova	2003
12583	0	0	1	1150	Camillagennaro	2003
12584	0	0	1	1151	weronikaejsmont	2002
12585	0	0	1	1152	Yoanakonstantinova	2007
12586	0	0	1	1153	Hannahread	2006
12587	0	0	1	1154	Darinaprecechtelova	2005
12588	0	0	1	1155	Chloeyoo	2003
12589	0	0	1	1156	Elifalbayrak	2004
12590	0	0	1	1157	Franceantezana dorado	2002
12591	0	0	1	1158	Onyuchoi	2006
12592	0	0	1	1159	Annakantor	2004
12593	0	0	1	1160	Bhaktishah	2003
12594	0	0	1	1161	Phoebepeus	2004
12595	0	0	1	1162	Beydabaykal	2003
12596	0	0	1	1163	Sadescharlin	2005

12597	0	0	1	1164	Martinamarusinova	2003
12598	0	0	1	1165	Eleanorschuster	2003
12599	0	0	1	1166	Alyssareguer	2006
12600	0	0	1	1167	Hannahsmith	2003
12601	0	0	1	1168	Mi Seongsim	2002
12602	0	0	1	1169	Orlyogilvy	2005
12603	0	0	1	1170	Marijasemenistaja	2002
12604	0	0	1	1171	Cyansun	2002
12605	0	0	1	1172	Tadiwanashemauchi	2006
12606	0	0	1	1173	Idajohansson	2004
12607	0	0	1	1174	Kristinakuksina	2004
12608	0	0	1	1175	Eunseokim	2002
12609	0	0	1	1176	Valeriavinces	2002
12610	0	0	1	1177	Vanjavidanovic	2002
12611	0	0	1	1178	Mariamikhailova	2002
12612	0	0	1	1179	Ginafeistel	2003
12613	0	0	1	1180	Jinzhang	2004
12614	0	0	1	1181	Inelamprecht	2003
12615	0	0	1	1182	Sofia Marianicolae	2002
12616	0	0	1	1183	Shadiaamado	2003
12617	0	0	1	1184	Anna Laurahinka	2002
12618	0	0	1	1185	Nataliafantini	2006
12619	0	0	1	1186	Laranedeljkovic	2004
12620	0	0	1	1187	Livhovde	2005
12621	0	0	1	1188	Diananekhorosheva	2002
12622	0	0	1	1189	Violaturini	2005
12623	0	0	1	1190	Dianalukyanova	2002
12624	0	0	1	1191	Ximenamorales araneda	2004
12625	0	0	1	1192	Rikkede koning	2003
12626	0	0	1	1193	Hyeongjuhan	2003
12627	0	0	1	1195	Nicole Isabelladiscenza orozco	2003
12628	0	0	1	1196	Whitneyking	2002
12629	0	0	1	1197	Renatajamrichova	2007
12630	0	0	1	1198	Kyarabelhadj	2005
12631	0	0	1	1199	Mia Sophiemueller	2004
12632	0	0	1	1200	Haramkim	2005
12633	0	0	1	1201	Juliettedaries	2002
12634	0	0	1	1202	Anikalemus maldonado	2005
12635	0	0	1	1203	Iuliamarko	2002
12636	0	0	1	1204	Alexandranielsen	2002
12637	0	0	1	1205	Haruneshigemasu	2004
12638	0	0	1	1206	Tatianalipatova	2003
12639	0	0	1	1207	Dharaniniroshan	2004
12640	0	0	1	1208	Madisonsmith	2005
12641	0	0	1	1209	Rianna Ioanacoreisa	2004
12642	0	0	1	1210	Hollystaff	2002
12643	0	0	1	1211	Nehavelaga	2002
12644	0	0	1	1212	Muskanmahajan	2002
12645	0	0	1	1213	Deborah Li Junlim	2005
12646	0	0	1	1214	Monikawojcik	2005

12647	0	0	1	1215	AIyamaguchi	2002
12648	0	0	1	1216	Hanael deeb	2004
12649	0	0	1	1217	Karinahofbauer	2004
12650	0	0	1	1218	Alexandraazarko	2006
12651	0	0	1	1219	Mirelashkumbova	2003
12652	0	0	1	1220	Feliciahalim	2003
12653	0	0	1	1221	Kittimolnar	2006
12654	0	0	1	1222	Yihanyang	2002
12655	0	0	1	1223	Vanesasulcova	2004
12656	0	0	1	1224	Anatamanika	2003
12657	0	0	1	1225	Analanisek	2005
12658	0	0	1	1225	Shuchichen	2004
12659	0	0	1	1227	Amirabargaoui	2004
12660	0	0	1	1228	Yoanadudova	2004
12661	0	0	1	1229	Selmaabdellaoui maane	2006
12662	0	0	1	1230	Preranakoirala	2003
12663	0	0	1	1230	Alicesoulie	2006
12664	0	0	1	1230	Patriciagrigoras	2006
12665	0	0	1	1233	Emmatothova	2005
12666	0	0	1	1234	Milastanojevic	2003
12667	0	0	1	1235	Adelamoravcova	2003
12668	0	0	1	1236	Gargipawar	2003
12669	0	0	1	1237	Viktoria Kleerkliimand	2002
12670	0	0	1	1238	Nicole Robertaserraglio	2004
12671	0	0	1	1239	Mara Sophieburger	2005
12672	0	0	1	1240	Katherinehui	2005
12673	0	0	1	1241	Benedettasensi	2005
12674	0	0	1	1242	Miatsoukalas	2004
12675	0	0	1	1243	Lidiiarasskovskaia	2004
12676	0	0	1	1244	Zixuanzeng	2004
12677	0	0	1	1245	Daryakornilova	2003
12678	0	0	1	1246	Martasoriano santiago	2006
12679	0	0	1	1247	Karsynevans	2005
12680	0	0	1	1248	Elizabethstrongina	2004
12681	0	0	1	1249	Nellyvighova	2004
12682	0	0	1	1250	Valentinade sousa	2004
12683	0	0	1	1250	Martinarivero	2002
12684	0	0	1	1252	Irodamakhamatjonova	2004
12685	0	0	1	1253	Polinaskopintseva	2005
12686	0	0	1	1254	Mariamauad de souza	2004
12687	0	0	1	1255	Marimovafarida	2004
12688	0	0	1	1256	Magdalenanunez pureco	2003
12689	0	0	1	1257	Maribellazamarripa	2002
12690	0	0	1	1258	Liviaairoldi	2005
12691	0	0	1	1259	Kaatcoppez	2005
12692	0	0	1	1260	Alejandraferrer rodriguez	2002
12693	0	0	1	1261	Vesagjinaj	2004
12694	0	0	1	1262	Kit Yi Kayeau-yeung	2003
12695	0	0	1	1263	jiyoungseo	2002
12696	0	0	1	1264	Zehrasuko	2005

12697	0	0	1	1265	Nalandateixeira da silva	2002
12698	0	0	1	1266	Emmaebona	2003
12699	0	0	1	1267	Amybloch	2004
12700	0	0	1	1268	Blankademicheli	2005
12701	0	0	1	1269	Marianareding	2005
12702	0	0	1	1270	Matiacristiani	2004
12703	0	0	1	1271	Aimeebrown	2003
12704	0	0	1	1272	Amritashanmugam	2003
12705	0	0	1	1273	Man Wai Chlorisli	2005
12706	0	0	1	1274	Erica Kannahagi	2005
12707	0	0	1	1275	Zan Ninglim	2005
12708	0	0	1	1275	Kholisa Sitimaisaroh	2006
12709	0	0	1	1277	Helenamohamed	2002
12710	0	0	1	1278	Yu Huacheng	2003
12711	0	0	1	1279	Gloriaalogo piqueras	2004
12712	0	0	1	1280	Yitongzhang	2002
12713	0	0	1	1281	Stefanalazar	2002
12714	0	0	1	1282	Clairechan	2004
12715	0	0	1	1283	Salmafarhat	2004
12716	0	0	1	1284	Reneevan bommel	2002
12717	0	0	1	1285	Marianaramirez nieto	2002
12718	0	0	1	1286	Nourshafei	2005
12719	0	0	1	1287	Chloechong zhi jing	2003
12720	0	0	1	1288	Vimbai Heidimoyo	2002
12721	0	0	1	1289	Hui-Gyeongkim	2002
12722	0	0	1	1290	Selyarakki	2004
12723	0	0	1	1291	Carmenlopez martinez	2005
12724	0	0	1	1292	Nikanovikova	2002
12725	0	0	1	1293	qingyangzhang	2004
12726	0	0	1	1294	Amaheecharrier	2004
12727	0	0	1	1295	Margaritatoshkova	2004
12728	0	0	1	1296	Amelsammari	2004
12729	0	0	1	1297	Annapanchartek	2004
12730	0	0	1	1298	Bertamiret avante	2004
12731	0	0	1	1299	En-Chiwang	2004
12732	0	0	1	1300	Sofiianagornaia	2003
12733	0	0	1	1301	Yasminanigametzyanova	2005
12734	0	0	1	1302	Yimeizhao	2004
12735	0	0	1	1303	Sofibrich	2002
12736	0	0	1	1304	Yuinaigarashi	2003
12737	0	0	1	1305	Giojang	2005
12738	0	0	1	1306	Emmaraducanu	2002
12739	0	0	1	1306	Misha Alizaberry	2002
12740	0	0	1	1308	Brunagebaili sampaio	2002
12741	0	0	1	1309	Mikaylafaure	2005
12742	0	0	1	1310	Yishuanglin	2004
12743	0	0	1	1311	Alexandracentra	2002
12744	0	0	1	1312	Marystoiana	2003
12745	0	0	1	1312	Jeonghaoh	2002
12746	0	0	1	1314	Coumba Ben Mamadouniangad	2004

12747	0	0	1	1315	Beatrisspasova	2002
12748	0	0	1	1316	Eva-Mariariml	2002
12749	0	0	1	1317	Inesbachir el bouhali	2002
12750	0	0	1	1318	Annetterobertson	2004
12751	0	0	1	1319	Mayarukosuev	2003
12752	0	0	1	1320	Zi Yihuang	2003
12753	0	0	1	1321	Petramiszczak	2002
12754	0	0	1	1322	Ada Pinarakarabacak	2005
12755	0	0	1	1323	Meekamarshall	2004
12756	0	0	1	1324	Aimaruyama	2003
12757	0	0	1	1325	Zohaasim	2002
12758	0	0	1	1326	Jiatonghan	2004
12759	0	0	1	1327	Danielanuno	2003
12760	0	0	1	1328	Polinakoneva	2008
12761	0	0	1	1329	Alicewidell	2003
12762	0	0	1	1330	Ashtonbowers	2005
12763	0	0	1	1331	Angelamendoza	2002
12764	0	0	1	1332	Maybaranes	2005
12765	0	0	1	1333	Eun Jilee	2004
12766	0	0	1	1334	Maryiastsetsevich	2004
12767	0	0	1	1335	Sofiazaprianov	2003
12768	0	0	1	1336	Stefaniwebb	2005
12769	0	0	1	1337	Rutujachaphalkar	2004
12770	0	0	1	1338	Kelly Loanavargas gonzalez	2004
12771	0	0	1	1339	Kenzyramy	2005
12772	0	0	1	1340	Elenaraffaeta	2002
12773	0	0	1	1341	Michelleyeo	2005
12774	0	0	1	1342	Victoriaosuigwe	2006
12775	0	0	1	1343	Britmartin	2006
12776	0	0	1	1344	Gabrielleleslie	2003
12777	0	0	1	1345	Clemencethouard	2005
12778	0	0	1	1346	Maria Piavivenzio	2005
12779	0	0	1	1347	Carolinefawzy	2002
12780	0	0	1	1348	Nourhassan moustafa	2005
12781	0	0	1	1349	Hannah Seen Eanyip	2003
12782	0	0	1	1350	Lindsayzink	2003
12783	0	0	1	1351	Anjapetkovic	2005
12784	0	0	1	1352	Leticiatarocco	2005
12785	0	0	1	1353	Kittikaaeb iem	2004
12786	0	0	1	1354	Norakhediri	2005
12787	0	0	1	1355	Isabellaventer	2003
12788	0	0	1	1356	Lorelyzmarruffo	2003
12789	0	0	1	1357	Reginaurbiola armenta	2003
12790	0	0	1	1358	Evabusaad	2005
12791	0	0	1	1359	Ntokozozungu	2002
12792	0	0	1	1360	Nattanichakaewcharoenwong	2003
12793	0	0	1	1361	Irynalysykh	2003
12794	0	0	1	1362	Sainadeshpande	2005
12795	0	0	1	1363	Viktoriehazmukova	2005
12796	0	0	1	1364	Stefaniapantaia	2003

12797	0	0	1	1365	Erikaweil	2004
12798	0	0	1	1366	Jessicadhome-casanova	2003
12799	0	0	1	1367	Mariaandrienko	2004
12800	0	0	1	1368	Isabellamartensson	2002
12801	0	0	1	1369	Alessandracaceres	2004
12802	0	0	1	1370	Amulikagottiparthy	2002
12803	0	0	1	1371	Seojinoh	2003
12804	0	0	1	1372	Alessandracristiani	2006
12805	0	0	1	1373	Shumanwu	2004
12806	0	0	1	1374	Zuzannafrankowska	2004
12807	0	0	1	1375	Rikokamiikura	2003
12808	0	0	1	1376	Danielalivson	2005
12809	0	0	1	1377	Miasaveljic	2005
12810	0	0	1	1378	Naledimanyube	2004
12811	0	0	1	1379	Evelinakontareva	2003
12812	0	0	1	1380	Alenakasatkina	2005
12813	0	0	1	1381	Emilijapranyte	2005
12814	0	0	1	1382	Martinamarica	2005
12815	0	0	1	1383	Valeriasilva benitez	2004
12816	0	0	1	1384	Hannah Kaileshen	2004
12817	0	0	1	1385	Ishitasingh	2002
12818	0	0	1	1386	Vladakozak	2006
12819	0	0	1	1387	Dianasmelansky	2003
12820	0	0	1	1388	Leenametwally	2005
12821	0	0	1	1389	Karenverduzco	2005
12822	0	0	1	1389	Vladislavaandreevskaya	2005
12823	0	0	1	1391	Mara Anastasiailiescu	2002
12824	0	0	1	1392	Dorotejapetrovic	2003
12825	0	0	1	1393	Anastasiamegrabian	2002
12826	0	0	1	1394	Anastasiyadyadchenko	2004
12827	0	0	1	1395	Danashakirova	2002
12828	0	0	1	1396	Elizabeteklavinska	2003
12829	0	0	1	1397	Bianca Liaantican gonzalez	2002
12830	0	0	1	1398	Thessyntondele zinga	2006
12831	0	0	1	1399	Stefanyashalakhova	2004
12832	0	0	1	1400	Chandrikajoshi	2002
12833	0	0	1	1401	Senemocal	2004
12834	0	0	1	1402	Katriinsaar	2002
12835	0	0	1	1403	Mariyakrasakova	2002
12836	0	0	1	1404	Anastasiapetrova	2002
12837	0	0	1	1405	Kaylasolagnier	2002
12838	0	0	1	1406	Kianamokhtari	2002
12839	0	0	1	1407	Irinawenger	2005
12840	0	0	1	1408	Carriebeckman	2002
12841	0	0	1	1409	Julianarodriguez	2004
12842	0	0	1	1410	Lerr Minlim	2004
12843	0	0	1	1411	Danielarivera	2004
12844	0	0	1	1412	Merlinasarno	2003
12845	0	0	1	1413	Lin Xintan	2002
12846	0	0	1	1414	Veranikapryts	2002

12847	0	0	1	1415	Lisakung	2002
12848	0	0	1	1416	Hadilnazih	2002
12849	0	0	1	1417	Heeraeim	2004
12850	0	0	1	1418	Mariabreidy	2006
12851	0	0	1	1419	Tapaneeboonyawat	2005
12852	0	0	1	1420	Solenygonzalez merida	2005
12853	0	0	1	1421	Sophyadevas	2006
12854	0	0	1	1422	Michelleweinstock	2004
12855	0	0	1	1423	Iantsatianaratovondrahona	2005
12856	0	0	1	1424	Carolina Isabelchiatti reynoso	2004
12857	0	0	1	1425	Hiromisekiguchi	2003
12858	0	0	1	1426	Emilycallahan	2003
12859	0	0	1	1427	Florianapopovici	2004
12860	0	0	1	1428	Karinaltori	2006
12861	0	0	1	1429	Paulacorbalan	2002
12862	0	0	1	1430	Zalimorris	2004
12863	0	0	1	1430	Mialiepert	2004
12864	0	0	1	1432	Jihoshin	2002
12865	0	0	1	1433	Sarapantelic	2005
12866	0	0	1	1434	Juliariera	2002
12867	0	0	1	1435	Marribera orquin	2004
12868	0	0	1	1435	Aureliariga	2004
12869	0	0	1	1437	Valieriiakornieva	2003
12870	0	0	1	1438	YuXuanluo	2002
12871	0	0	1	1439	Aleksandrazlatarova	2002
12872	0	0	1	1439	Sydneystone	2005
12873	0	0	1	1441	Sofia Nahiaragarcia	2003
12874	0	0	1	1442	Aryanabartlett	2005
12875	0	0	1	1443	Zoedu pasquier jensen	2005
12876	0	0	1	1444	Juliezytova	2003
12877	0	0	1	1445	Jadehaller	2004
12878	0	0	1	1446	Faustineliope	2004
12879	0	0	1	1447	Ottaviamassetti	2003
12880	0	0	1	1448	Ekaterinaleksina	2005
12881	0	0	1	1449	Anastasiiavorobiova	2004
12882	0	0	1	1450	Jasminmakela	2004
12883	0	0	1	1451	Dunjamaric	2006
12884	0	0	1	1452	Natsuminumano	2002
12885	0	0	1	1453	Isis Louisevan den broek	2005
12886	0	0	1	1454	Martynazaremba	2003
12887	0	0	1	1454	Clarissablomqvist	2004
12888	0	0	1	1456	Valentinalargacha gonzalez	2002
12889	0	0	1	1457	Ritagefaizy	2003
12890	0	0	1	1458	Souhilaaouni	2004
12891	0	0	1	1459	Paranutlumjeaktest	2003
12892	0	0	1	1460	Nicoleteodosescu	2003
12893	0	0	1	1461	Zeynep Nazdara	2005
12894	0	0	1	1462	Angelina Joyhug	2002
12895	0	0	1	1463	Valentinarodas carcamo	2005
12896	0	0	1	1464	Elenilazaridou	2004

12897	0	0	1	1465	Tanyaradzwamidzi	2006
12898	0	0	1	1466	Kristynakostelna	2004
12899	0	0	1	1467	Adelismagylova	2003
12900	0	0	1	1468	Raquel Alexasanchez elizondo	2005
12901	0	0	1	1469	Cartika Wisesha Dewilarasati	2002
12902	0	0	1	1470	Annabelschoen	2002
12903	0	0	1	1471	Cristinetchanichev	2003
12904	0	0	1	1471	Tinali	2004
12905	0	0	1	1473	Soniabenlhassen	2004
12906	0	0	1	1474	Lindasalvi	2002
12907	0	0	1	1475	Roseseccia	2004
12908	0	0	1	1476	Annabeldavis	2003
12909	0	0	1	1477	Katiatuliakova	2004
12910	0	0	1	1478	Marjoriesouza	2003
12911	0	0	1	1479	Alisanadeikina	2003
12912	0	0	1	1480	Reanakashima	2003
12913	0	0	1	1481	Miriamgondorova	2003
12914	0	0	1	1482	Mandegarfarzami	2006
12915	0	0	1	1483	Feierhu	2004
12916	0	0	1	1484	Sophiafornaris	2002
12917	0	0	1	1485	Terezadejnozkova	2002
12918	0	0	1	1486	Lakshanyavishwanath	2003
12919	0	0	1	1487	Bertapassola	2004
12920	0	0	1	1488	Chahdtourkhani	2007
12921	0	0	1	1489	Emmastauber	2003
12922	0	0	1	1490	Alexandrakornilova	2003
12923	0	0	1	1491	Leticiaferrari luiz	2002
12924	0	0	1	1492	YASHASWINI KRISHNA REDDYn	2005
12925	0	0	1	1493	Rennesingla	2004
12926	0	0	1	1494	Tessmenten	2002
12927	0	0	1	1494	dariana Joseprato pernalete	2003
12928	0	0	1	1496	Lamijaavdic	2005
12929	0	0	1	1497	Velizarafileva	2003
12930	0	0	1	1498	Shirleyliang	2002
12931	0	0	1	1499	Ana Paolamercado cervantes	2004
12932	0	0	1	1500	Annaprotsenko	2003
12933	0	0	1	1501	Shanelleiaconi	2004
12934	0	0	1	1502	Carmen Andreeaherea	2005
12935	0	0	1	1503	Juliafliegner	2003
12936	0	0	1	1504	Fatemesadatzanjani	2004
12937	0	0	1	1505	Angelinafolts	2003
12938	0	0	1	1506	Hayukinoshita	2006
12939	0	0	1	1507	Juliaandreach	2002
12940	0	0	1	1508	Saraho'connor	2002
12941	0	0	1	1509	Alinafedulova	2003
12942	0	0	1	1510	Sydjipeszynski	2004
12943	0	0	1	1511	Alinakorneeva	2007
12944	0	0	1	1512	Dariashadchneva	2006
12945	0	0	1	1513	Allabetina	2003
12946	0	0	1	1514	Oferkivity	2002

12947	0	0	1	1515	Adrianabarney castro	2002
12948	0	0	1	1516	Gretegull	2006
12949	0	0	1	1517	KISSMELcedano lapaix	2004
12950	0	0	1	1518	Nataliavela zangroniz	2004
12951	0	0	1	1519	Sofiamatiushova	2003
12952	0	0	1	1520	Ozi-oiza Josephineyakubu	2005
12953	0	0	1	1521	Margaritaokhendovskaya	2006
12954	0	0	1	1521	Ekaterinasuvorova	2005
12955	0	0	1	1523	Hrudayashah	2003
12956	0	0	1	1524	Aimeereynoso	2004
12957	0	0	1	1525	Brunacasasampere escoda	2002
12958	0	0	1	1526	Darynashoshyna	2005
12959	0	0	1	1527	Arinaarifullina	2006
12960	0	0	1	1528	Elizabetebrutane	2007
12961	0	0	1	1529	Mayaiyengar	2007
12962	0	0	1	1530	Lia Kaishikimosimann	2004
12963	0	0	1	1531	Chiaradesia d'abarno	2003
12964	0	0	1	1531	Ginevragottardi	2004
12965	0	0	1	1533	Jacquelinesoloveychik	2004
12966	0	0	1	1533	Anastasiyaskorupska	2004
12967	0	0	1	1533	Astridschmits	2004
12968	0	0	1	1536	Daryashauha	2003
12969	0	0	1	1537	Jou-Chenchen	2003
12970	0	0	1	1538	Arinabulatova	2006
12971	0	0	1	1539	Ximenacano	2004
12972	0	0	1	1540	Paulinaalvarez	2003
12973	0	0	1	1541	Samanthabuyckx	2002
12974	0	0	1	1542	Keirajirasook	2003
12975	0	0	1	1543	Arinasuchkova	2003
12976	0	0	1	1544	Aleiade buretel	2002
12977	0	0	1	1545	Asimasazan	2005
12978	0	0	1	1546	Maria Claudiavalladares	2003
12979	0	0	1	1546	Alisyagladkova	2003
12980	0	0	1	1548	Freialawrence	2003
12981	0	0	1	1549	Sharlehanekom	2003
12982	0	0	1	1550	Malaikarapolu	2003
12983	0	0	1	1550	Annazhang	2002
12984	0	0	1	1552	Luisameyer auf der heide	2002
12985	0	0	1	1553	Mayadrozd	2004
12986	0	0	1	1554	Mayucrossley	2006
12987	0	0	1	1555	Isabellabolivar garces	2002
12988	0	0	1	1556	Elsawan	2003
12989	0	0	1	1557	Dunevaissaud	2007
12990	0	0	1	1558	Smillakjaergaard	2002
12991	0	0	1	1559	Hlasaber	2004
12992	0	0	1	1560	Mariamfarouk	2006
12993	0	0	1	1561	Tamaryaffe	2004
12994	0	0	1	1562	Zhenghuaxiao	2002
12995	0	0	1	1562	Alexandrasilna	2002
12996	0	0	1	1564	Audreywijono	2004

12997	0	0	1	1565	Rafaelaplatsiota	2003
12998	0	0	1	1566	Andra Izabellasirbu	2005
12999	0	0	1	1567	Rheasingh	2002
13000	0	0	1	1568	Martinapavissich	2006
13001	0	0	1	1568	Vladamincheva	2007
13002	0	0	1	1568	Gaeuljang	2006
13003	0	0	1	1571	Stefanisandoiu shtein	2005
13004	0	0	1	1572	Maximaellenberg	2002
13005	0	0	1	1573	Maryjoecrisologo	2002
13006	0	0	1	1574	Ghaliaguizani	2006
13007	0	0	1	1575	Malakel allami	2006
13008	0	0	1	1576	Caitlynportela	2002
13009	0	0	1	1577	Kanyatamonyodsanit	2004
13010	0	0	1	1578	Maria Luisaoliveira	2005
13011	0	0	1	1579	Mariaantonova	2005
13012	0	0	1	1580	Magdalenasmekalova	2006
13013	0	0	1	1581	Aurorapedwell	2005
13014	0	0	1	1582	Aglayazaynieva	2002
13015	0	0	1	1583	Arinastanchik	2004
13016	0	0	1	1584	Yufeiren	2006
13017	0	0	1	1585	Akanksha Dileepnitture	2003
13018	0	0	1	1586	Zuzanavancelova	2004
13019	0	0	1	1587	Sarahtatu	2004
13020	0	0	1	1588	Vasiliki Magdalinisamara	2003
13021	0	0	1	1589	Luciarizza	2003
13022	0	0	1	1590	Mikabuchnik	2007
13023	0	0	1	1591	Claratauson	2002
13024	0	0	1	1592	Sze Xuanlim	2005
13025	0	0	1	1593	Kasiatreiber	2003
13026	0	0	1	1594	Roselidaasumwa	2004
13027	0	0	1	1595	Martinapetrovic	2003
13028	0	0	1	1596	Danielarubio	2005
13029	0	0	1	1597	Irinaartemova	2004
13030	0	0	1	1598	Sofiiavdovychenko	2003
13031	0	0	1	1599	Senkadeletic	2003
13032	0	0	1	1600	Julianacarton	2003
13033	0	0	1	1601	Chloeweekes	2003
13034	0	0	1	1602	Charlotte Mosbaekellegaard	2003
13035	0	0	1	1603	Irawandeewajee	2002
13036	0	0	1	1604	Petramihokova	2004
13037	0	0	1	1605	Patrycjaniewiadomska	2003
13038	0	0	1	1606	Sophiazaslow	2003
13039	0	0	1	1606	Lucindagatsiounis	2004
13040	0	0	1	1606	Annelinbakker	2005
13041	0	0	1	1606	Charlottehoward	2002
13042	0	0	1	1610	Sofiapavlova	2005
13043	0	0	1	1611	Fridabostrand	2002
13044	0	0	1	1611	Gabrielamiteva	2005
13045	0	0	1	1613	Korneliahuncak	2003
13046	0	0	1	1614	Dinaraenaleeva	2003

13047	0	0	1	1615	Valeriaguerrero barberan	2005
13048	0	0	1	1615	Teahchavez	2005
13049	0	0	1	1615	Ecegencer	2006
13050	0	0	1	1615	Anoukkoevermans	2004
13051	0	0	1	1615	Kundana Sribandaru	2005
13052	0	0	1	1620	Karolasuviste	2004
13053	0	0	1	1621	Alexandrajewitt	2003
13054	0	0	1	1622	Aninalanz	2005
13055	0	0	1	1623	Emmavanderheyden	2003
13056	0	0	1	1624	Maxiduncan	2002
13057	0	0	1	1625	Mariageorgiadou	2003
13058	0	0	1	1626	Kenzyabdelaal	2005
13059	0	0	1	1627	Mayajoint	2006
13060	0	0	1	1628	Lydiavlachou	2002
13061	0	0	1	1628	Loanmonnet	2003
13062	0	0	1	1628	Tamrynbrown	2005
13063	0	0	1	1631	Oliviabergler	2005
13064	0	0	1	1632	Vladalenskaya-bogomolova	2003
13065	0	0	1	1633	Sonjazhiyenbayeva	2006
13066	0	0	1	1634	Emmapedretti	2004
13067	0	0	1	1635	Mirjanajovanovic	2004
13068	0	0	1	1636	Reyacoe	2005
13069	0	0	1	1637	Taylawilmot	2005
13070	0	0	1	1638	Lok Shutang	2006
13071	0	0	1	1639	Andrearoots	2005
13072	0	0	1	1640	Barbaramartinez	2003
13073	0	0	1	1641	Hanaotsubo	2003
13074	0	0	1	1642	Sudeince	2004
13075	0	0	1	1642	Elysiapool	2002
13076	0	0	1	1642	Masha Alexandravrsalovic gime	2004
13077	0	0	1	1642	Wanyizhang	2003
13078	0	0	1	1646	Ellasimmons	2003
13079	0	0	1	1647	Maftunabonukahramonova	2005
13080	0	0	1	1648	Maria Biancaandrei	2003
13081	0	0	1	1649	Alejandraramirez	2002
13082	0	0	1	1650	Viktorialackova	2005
13083	0	0	1	1651	Sofiamikhailova	2005
13084	0	0	1	1652	Sophiawurm	2003
13085	0	0	1	1653	Isabelleexsted	2002
13086	0	0	1	1653	Alessiacau	2005
13087	0	0	1	1655	Camiliasamel druz	2006
13088	0	0	1	1656	Ivannia Carolinafernandez vald	2002
13089	0	0	1	1657	Pimmadathongkum	2006
13090	0	0	1	1658	AMANY JAURESSE NELLYkouam	2004
13091	0	0	1	1658	Elif Sanemkaradag	2004
13092	0	0	1	1660	Karolinabartusek	2003
13093	0	0	1	1660	Taiminashiku	2002
13094	0	0	1	1662	Julietahonrubia andrino	2002
13095	0	0	1	1663	Ana Fabiaholweg bustillos	2004
13096	0	0	1	1664	Stylianiroussopoulou	2003

13097	0	0	1	1665	Riadernikovic	2005
13098	0	0	1	1665	Carlottamencaglia	2003
13099	0	0	1	1667	Lia Isabellaespinal	2002
13100	0	0	1	1668	Katarinaneff	2003
13101	0	0	1	1669	Valerialloclla	2003
13102	0	0	1	1670	Micaelaponce serrano	2003
13103	0	0	1	1671	Tania Isabelandrade sabando	2005
13104	0	0	1	1671	Jannahelnemr	2003
13105	0	0	1	1673	Zehracanbaz	2003
13106	0	0	1	1674	Louisavoelz	2004
13107	0	0	1	1675	Carolinaazadinho	2003
13108	0	0	1	1676	Sara Isabelsnyder	2003
13109	0	0	1	1677	Andreea Alexandralila	2003
13110	0	0	1	1678	Isadorawalber machado	2002
13111	0	0	1	1678	Jamilahsnells	2002
13112	0	0	1	1678	Ayshecan	2002
13113	0	0	1	1681	Gyeong Seolee	2005
13114	0	0	1	1682	Scarlettperkins	2005
13115	0	0	1	1683	Alinazepfel	2003
13116	0	0	1	1683	Laurenstein	2002
13117	0	0	1	1685	Anggi Dwihidayati	2003
13118	0	0	1	1686	Ewasamberger	2002
13119	0	0	1	1686	Moranemeyers	2005
13120	0	0	1	1686	Viktoryiazhadzinskaya	2003
13121	0	0	1	1689	Ninadarmatiova	2003
13122	0	0	1	1690	Reneesharma	2003
13123	0	0	1	1691	VALERIAguerrero	2004
13124	0	0	1	1692	Megandavies	2002
13125	0	0	1	1693	Ademiamankeldi	2006
13126	0	0	1	1694	Robertade la garza velazquez	2003
13127	0	0	1	1695	Liel Marliesrothensteiner	2005
13128	0	0	1	1696	Minseokim	2004
13129	0	0	1	1697	Elisarigazio	2003
13130	0	0	1	1698	Hannaserhel	2003
13131	0	0	1	1699	Adithiare	2003
13132	0	0	1	1700	Morganmccarthy	2004
13133	0	0	1	1701	Sarahejtmankova	2004
13134	0	0	1	1702	Mariaverdu gonzalez	2002
13135	0	0	1	1703	Emiliejust-bomholt	2005
13136	0	0	1	1704	Linahachem	2004
13137	0	0	1	1704	Ifigeneiakafka	2005
13138	0	0	1	1706	Terezevevere	2002
13139	0	0	1	1706	Annakoltunova	2002
13140	0	0	1	1708	Ioanadragomir	2004
13141	0	0	1	1709	Elenamercioiu	2005
13142	0	0	1	1709	Emmiewright	2003
13143	0	0	1	1709	Angelinadaniukova	2003
13144	0	0	1	1709	Caterinasalas	2004
13145	0	0	1	1709	Emmakuusisto	2005
13146	0	0	1	1714	Lakshmigowda	2005

13147	0	0	1	1714	Ninavicic	2002
13148	0	0	1	1716	Arwaboussayri	2003
13149	0	0	1	1716	Sasha Nataliechimedza	2007
13150	0	0	1	1718	Ananyakothakota	2002
13151	0	0	1	1719	Gabrielacarvajal melendez	2002
13152	0	0	1	1720	Millieskelton	2005
13153	0	0	1	1720	Dianade simone	2005
13154	0	0	1	1720	Yu Jiezhang	2003
13155	0	0	1	1723	Tamarabajagic	2002
13156	0	0	1	1724	Madeliefhageman	2003
13157	0	0	1	1725	Annasyrova	2002
13158	0	0	1	1726	Alinavasilenko	2004
13159	0	0	1	1727	Sun Wooyoun	2003
13160	0	0	1	1728	Anitabertoloni	2002
13161	0	0	1	1729	Dora Eszterbaranyai	2003
13162	0	0	1	1730	Sapirsela	2002
13163	0	0	1	1730	Rebekakern	2003
13164	0	0	1	1732	Isabela Ioanadima	2002
13165	0	0	1	1733	Ana Mariagomez	2003
13166	0	0	1	1734	Isabelpetri bere	2002
13167	0	0	1	1735	VICTORIApapadopoulou	2004
13168	0	0	1	1736	Naa Anyemamckorley	2003
13169	0	0	1	1737	Avroravolkova	2004
13170	0	0	1	1737	Lucieurbanova	2006
13171	0	0	1	1739	Anastasiasafta	2004
13172	0	0	1	1740	Parniaroknabadi	2003
13173	0	0	1	1740	Brendayoris	2004
13174	0	0	1	1742	Yang Ping Anli	2002
13175	0	0	1	1742	Niken Ferlyanakusumaningtyas	2003
13176	0	0	1	1742	Se-Yeonyang	2005
13177	0	0	1	1745	Tiannarangan	2006
13178	0	0	1	1746	Ekaterinaperelygina	2006
13179	0	0	1	1746	Elif Tuanagunal	2005
13180	0	0	1	1748	Arabellamoen	2002
13181	0	0	1	1748	Irinabalus	2005
13182	0	0	1	1750	Mireiafehr	2003
13183	0	0	1	1751	Masaviriant	2005
13184	0	0	1	1752	Alishamoldakhmetova	2002
13185	0	0	1	1752	Evagoncharov	2003
13186	0	0	1	1754	Varisaramuisuk	2003
13187	0	0	1	1754	Darianazarenko	2004
13188	0	0	1	1756	Seo Yeonpark	2003
13189	0	0	1	1757	Madalenapeneda	2002
13190	0	0	1	1758	Rachelkrzyzak	2003
13191	0	0	0	1758	Darigarustemova	2006
13192	0	0	0	1760	Cherysh Endurancehenry	2005
13193	0	0	0	1760	Victoriasavvides	2004
13194	0	0	0	1762	SUPANIDnopparith	2005
13195	0	0	0	1763	Urszulanebelska	2002
13196	0	0	0	1763	Valeriamonko	2002

13197	0	0	0	1763	Sofiavelarde	2003
13198	0	0	0	1763	Yumuwatanabe	2003
13199	0	0	0	1763	Leannserna	2004
13200	0	0	0	1768	Roaaelatrebi	2003
13201	0	0	0	1769	Umayrahashimova	2003
13202	0	0	0	1770	Selenabird	2005
13203	0	0	0	1771	Vladahaetcaia	2004
13204	0	0	0	1772	Valeriiakrokhotina	2005
13205	0	0	0	1773	Cosima Trinitycalinescu	2002
13206	0	0	0	1774	Sinafackelmann	2004
13207	0	0	0	1775	Sudiptakumar	2003
13208	0	0	0	1776	Alaraayaz	2005
13209	0	0	0	1777	Riyahdugar	2004
13210	0	0	0	1777	Shreyachakraborty	2002
13211	0	0	0	1779	Juliebedard	2005
13212	0	0	0	1779	Beatrice Laurapirtac	2003
13213	0	0	0	1781	Anastasiabreshenkova	2005
13214	0	0	0	1782	Evelyn Alessandragomez	2003
13215	0	0	0	1783	Jemmacarbis	2002
13216	0	0	0	1784	Lindaajimi	2006
13217	0	0	0	1785	Elenagrekul	2004
13218	0	0	0	1785	Ameliatarasova	2003
13219	0	0	0	1787	Barboramichalkova	2006
13220	0	0	0	1788	Zhansayabakytzhan	2005
13221	0	0	0	1788	Hetanuutinen	2003
13222	0	0	0	1790	Hui Yiisoo	2005
13223	0	0	0	1791	Janakareisova	2006
13224	0	0	0	1791	Martinecarroll	2004
13225	0	0	0	1793	Kurumitamura	2004
13226	0	0	0	1794	Rosemarywatanabe	2002
13227	0	0	0	1794	Carolinalima de oliveira	2003
13228	0	0	0	1796	Oleksandradobranos	2002
13229	0	0	0	1796	Cambellebouchard	2003
13230	0	0	0	1798	Nataliamichta	2004
13231	0	0	0	1799	Sara Alejandraseivane torres	2002
13232	0	0	0	1800	Tiffany Clairenocos	2005
13233	0	0	0	1801	Jovanagrujic	2006
13234	0	0	0	1801	Anna Linnpuls	2006
13235	0	0	0	1803	Carolinaburgos	2002
13236	0	0	0	1803	Valentinetalaki	2006
13237	0	0	0	1803	Constanzamillas	2002
13238	0	0	0	1806	Elisadavalos donoso	2003
13239	0	0	0	1806	Maria Josearbelaez galindo	2003
13240	0	0	0	1806	Vanshitapathania	2002
13241	0	0	0	1809	Camilaluri iturbi	2002
13242	0	0	0	1810	Aleksandra Sophialopez savos	2004
13243	0	0	0	1811	Momokonishio	2002
13244	0	0	0	1812	Laviniatorselli	2004
13245	0	0	0	1813	Emiliahartman	2003
13246	0	0	0	1813	Anastasiyakostromitina	2002

13247	0	0	0	1815	Talikrasnopolski	2002
13248	0	0	0	1816	Ameliarajecki	2002
13249	0	0	0	1817	Nonnoadegawa	2004
13250	0	0	0	1818	Haileymurphy	2004
13251	0	0	0	1819	Sararhallab	2003
13252	0	0	0	1820	Sophiellewellyn	2006
13253	0	0	0	1821	Zi Yingruan	2006
13254	0	0	0	1822	Laurarahnel	2006
13255	0	0	0	1823	Kamonwanyodpetch	2007
13256	0	0	0	1824	Laure Linemonnoyer	2003
13257	0	0	0	1824	Jesutoyosiadeusi	2002
13258	0	0	0	1826	Alexandra Ralucabotin	2002
13259	0	0	0	1826	Emmarizzetto	2004
13260	0	0	0	1828	Leontinastojanovic	2005
13261	0	0	0	1828	Vilmakrebs hyllested	2005
13262	0	0	0	1830	Francinapuig llompart	2002
13263	0	0	0	1831	Reginaruiz	2003
13264	0	0	0	1832	Maitree Diksharout	2004
13265	0	0	0	1833	Saramikaca	2003
13266	0	0	0	1834	Lucero Noelimonsalvo	2003
13267	0	0	0	1834	Lujanalegre	2005
13268	0	0	0	1836	Fatima Andreanavarro pacheco	2002
13269	0	0	0	1837	Carlebotha	2005
13270	0	0	0	1838	Sawssanbouhlel	2003
13271	0	0	0	1838	Maria Fernandade la torre ugar	2002
13272	0	0	0	1840	Natalijanikolic	2004
13273	0	0	0	1841	Piperfreeman	2004
13274	0	0	0	1842	Ritiagarwal	2002
13275	0	0	0	1843	Nur Rosida Megahadiati	2002
13276	0	0	0	1844	Nataliacastaneda guerrero	2005
13277	0	0	0	1845	Nansitoskova	2002
13278	0	0	0	1846	Ronnienaor	2003
13279	0	0	0	1846	Ana Sofiaangeles	2002
13280	0	0	0	1848	Sofiashing	2002
13281	0	0	0	1849	Juliagonzalez romero	2005
13282	0	0	0	1850	Carlacabero del hierro	2003
13283	0	0	0	1850	Larastojanovski	2005
13284	0	0	0	1850	Swetasamanta	2005
13285	0	0	0	1850	Laura Sofiaruiz ramirez	2004
13286	0	0	0	1850	Avasadaoui	2005
13287	0	0	0	1855	Polinaramenskaja	2002
13288	0	0	0	1856	Lizanneboyer	2004
13289	0	0	0	1857	Francescaaguirre	2002
13290	0	0	0	1858	Nikolruseva	2003
13291	0	0	0	1859	Clarapenido	2002
13292	0	0	0	1860	Valentinaloretz	2003
13293	0	0	0	1861	Ariannacastellanos calderon	2003
13294	0	0	0	1861	ISABELLAlechuga franco	2005
13295	0	0	0	1863	Melissazeh	2004
13296	0	0	0	1864	Tianyudong	2005

13297	0	0	0	1865	Emma Janebarclay	2003
13298	0	0	0	1865	Janarefaat	2005
13299	0	0	0	1867	Meganbasson	2002
13300	0	0	0	1867	Nourgueblaoui	2004
13301	0	0	0	1869	Anastasiasizykh	2005
13302	0	0	0	1870	Evaalvarez sande	2004
13303	0	0	0	1870	Sanagarakani	2003
13304	0	0	0	1872	Danielacarreto	2002
13305	0	0	0	1873	Elizabethjurna	2005
13306	0	0	0	1874	Francescapace	2005
13307	0	0	0	1874	Cirkelinelundberg	2003
13308	0	0	0	1874	Monikatemkova	2003
13309	0	0	0	1874	Chloeschwarz	2004
13310	0	0	0	1874	Jessica-Jadehaylo	2004
13311	0	0	0	1874	Kaedetajima	2006
13312	0	0	0	1880	Mancalampret	2003
13313	0	0	0	1881	Sophiarogers	2003
13314	0	0	0	1882	Katarinaliedbeck	2003
13315	0	0	0	1883	Laurawipfli	2003
13316	0	0	0	1884	Maria Eduardade goes fontes d	2002
13317	0	0	0	1885	Karolinamazurek	2003
13318	0	0	0	1886	Piakranholdt	2002
13319	0	0	0	1886	Marwahakimi	2003
13320	0	0	0	1886	Sofiamazzucato	2005
13321	0	0	0	1886	Yingqunsun	2006
13322	0	0	0	1886	Allisonzipoli	2003
13323	0	0	0	1886	Yashnayellayi	2002
13324	0	0	0	1892	Linangassi saqout	2005
13325	0	0	0	1892	Lidialaskova	2003
13326	0	0	0	1894	Lauradukic	2004
13327	0	0	0	1894	Maria Andrealora meza	2004
13328	0	0	0	1894	Sofiakanarskaya	2004
13329	0	0	0	1894	Hannahpinto	2002
13330	0	0	0	1898	Isabelabarona estrada	2003
13331	0	0	0	1898	Yoshinofujita	2005
13332	0	0	0	1900	Kristinasamardzic	2003
13333	0	0	0	1901	Ana Julissade mata	2002
13334	0	0	0	1902	Nadafouad	2003
13335	0	0	0	1903	Claudiagasparovic	2004
13336	0	0	0	1904	Vanessakahkesh	2003
13337	0	0	0	1905	Filippavale frogner	2002
13338	0	0	0	1906	Althea Fayong	2003
13339	0	0	0	1906	Alisevindiga	2002
13340	0	0	0	1908	Sashanelson	2002
13341	0	0	0	1908	Vidhijani	2004
13342	0	0	0	1910	Saheraleem	2003
13343	0	0	0	1910	Zahrasuleman	2004
13344	0	0	0	1910	Biborkanagy bekesi	2004
13345	0	0	0	1910	Chelseachakanyuka	2006
13346	0	0	0	1910	Maria Paulavargas triana	2005

13347	0	0	0	1915	Finaritraandriamadison	2002
13348	0	0	0	1915	Noelle Vivianfaber	2004
13349	0	0	0	1917	Daryayelfimova	2003
13350	0	0	0	1917	Arinagamretkaia	2003
13351	0	0	0	1919	Annalajos	2004
13352	0	0	0	1920	Mirthemoens	2006
13353	0	0	0	1921	Fernandacastillo huerta	2003
13354	0	0	0	1922	Sarapistol	2005
13355	0	0	0	1923	Lilyfairclough	2005
13356	0	0	0	1923	Yilinyan	2005
13357	0	0	0	1925	Emilyniers	2004
13358	0	0	0	1925	Aarni Reddyyellu	2004
13359	0	0	0	1925	Seoyoungkim	2002
13360	0	0	0	1928	Laurerazet	2004
13361	0	0	0	1929	Simonadeletic	2005
13362	0	0	0	1930	Emmalindh	2005
13363	0	0	0	1930	Maria Joselopez	2006
13364	0	0	0	1930	Elanmnatsakanov	2002
13365	0	0	0	1930	Carolinalopez	2006
13366	0	0	0	1934	Ira Mehernissarawat	2002
13367	0	0	0	1935	Anna Lenaebster	2004
13368	0	0	0	1936	Iratiurbaneja salazar	2002
13369	0	0	0	1936	Kanikasivaraman	2003
13370	0	0	0	1938	Alexandralesnikova	2002
13371	0	0	0	1939	Maria Josezarza mojoli	2005
13372	0	0	0	1939	Aidaeissa	2005
13373	0	0	0	1941	Katarinazuzul	2002
13374	0	0	0	1942	Johannahiesmair	2005
13375	0	0	0	1943	Dareenelleithy	2002
13376	0	0	0	1944	Yuliavedischeva	2003
13377	0	0	0	1944	Ximenagarzon martinez	2005
13378	0	0	0	1946	Anikaseneviratne	2002
13379	0	0	0	1947	Carrolina Marthasanjaya	2002
13380	0	0	0	1948	Elizabetverizova	2002
13381	0	0	0	1949	Natalia Geraldinesalinas chapar	2002
13382	0	0	0	1950	Salonitamang	2004
13383	0	0	0	1950	Emmaanderson	2005
13384	0	0	0	1952	Palomagoldsmith weinreich	2004
13385	0	0	0	1953	Myaraiss	2004
13386	0	0	0	1953	Milankrish	2003
13387	0	0	0	1955	Estefaniagarcia	2003
13388	0	0	0	1955	Luyizadadvani	2002
13389	0	0	0	1957	Cocobosman	2003
13390	0	0	0	1958	Fabriziacambria	2004
13391	0	0	0	1959	Antonia Pazramos zapata	2002
13392	0	0	0	1960	Sissiaxelsson	2002
13393	0	0	0	1961	Jiatongcai	2006
13394	0	0	0	1961	Audreytong	2006
13395	0	0	0	1961	Ninagulbransen	2002
13396	0	0	0	1961	Valentinaborda	2003

13397	0	0	0	1961	Alianzack	2006
13398	0	0	0	1961	REBECCAekpenyong	2004
13399	0	0	0	1967	Laiapasini	2002
13400	0	0	0	1968	Anna-Lizakus	2002
13401	0	0	0	1968	Juliananieto	2004
13402	0	0	0	1968	Gloriananahum	2006
13403	0	0	0	1968	Francescagandolfi	2006
13404	0	0	0	1968	Sofiaifailova	2003
13405	0	0	0	1968	Tatianaloglo	2006
13406	0	0	0	1968	Anastasiabozova	2005
13407	0	0	0	1968	Dariajula	2002
13408	0	0	0	1968	Alessiapopescu	2004
13409	0	0	0	1977	Linanikolova	2002
13410	0	0	0	1977	Sarahmillard	2003
13411	0	0	0	1979	Shirachiche	2004
13412	0	0	0	1980	Helenaramos rocha e silva	2002
13413	0	0	0	1980	Emmazheng	2005
13414	0	0	0	1982	Rotemcohen	2006
13415	0	0	0	1983	Mariamella	2002
13416	0	0	0	1983	Jadefeneon	2003
13417	0	0	0	1985	Roisingilheany	2005
13418	0	0	0	1986	Arinababenko	2004
13419	0	0	0	1986	Giovanaoliveira	2002
13420	0	0	0	1986	Dogaturkmen	2002
13421	0	0	0	1986	Pearljansz	2002
13422	0	0	0	1986	Alicia Anndaly	2004
13423	0	0	0	1986	Valeriyabichurina	2002
13424	0	0	0	1986	Ceylinipek	2006
13425	0	0	0	1986	Paularumpf	2003
13426	0	0	0	1994	Kayarand	2002
13427	0	0	0	1994	Isabelleoikawa torres	2005
13428	0	0	0	1996	Emilielugon moulin	2004
13429	0	0	0	1996	Laragrulovic	2003
13430	0	0	0	1996	Jordynmcbride	2002
13431	0	0	0	1996	Neratesankic	2006
13432	0	0	0	1996	Alisakummel	2002
13433	0	0	0	1996	Luciasantos sanchez	2002
13434	0	0	0	1996	Javieraverdugo	2004
13435	0	0	0	1996	Laragrulovic	2003
13436	0	0	0	1996	Meerajesudason	2005
13437	0	0	0	1996	Neratesankic	2006
13438	0	0	0	1996	Luciasantos sanchez	2002
13439	0	0	0	1996	Emmapersson	2003
13440	0	0	0	1996	Allison Nicoleisaacs	2002
13441	0	0	0	1996	Millehaagensen	2002
13442	0	0	0	1996	Hanabecirovic novak	2003
13443	0	0	0	1996	Alisakummel	2002
13444	0	0	0	1996	Jordynmcbride	2002
13445	0	0	0	1996	Amyhuang	2002
13446	0	0	0	2014	Duru Yagmurdurgun	2005

13447	0	0	0	2014	Isabellaasenov	2005
13448	0	0	0	2014	Jordinacegarra ramirez	2004
13449	0	0	0	2014	Milicabratic	2004
13450	0	0	0	2014	Tildejagare	2004
13451	0	0	0	2019	Sapircohen	2007
13452	0	0	0	2020	isabellaochoa	2005
13453	0	0	0	2021	Huayizhang	2004
13454	0	0	0	2021	Erinmckenzie	2005
13455	0	0	0	2021	Labikadurab	2007
13456	0	0	0	2021	Aminasalibayeva	2006
13457	0	0	0	2025	Majabajorek	2004
13458	0	0	0	2025	Sofiyazueva	2004
13459	0	0	0	2025	Tullyrichter	2002
13460	0	0	0	2028	Malaylack Delilahpathummakur	2006
13461	0	0	0	2028	Beloslavaveleva	2003
13462	0	0	0	2028	Carlafity	2006
13463	0	0	0	2031	Adele Mariefernandez lecaroz	2002
13464	0	0	0	2031	Laurenrha	2003
13465	0	0	0	2033	Boluwatifebakre	2003
13466	0	0	0	2034	Martynaszpakowska	2003
13467	0	0	0	2034	Dariagorska	2005
13468	0	0	0	2036	Ishikaagarwal	2002
13469	0	0	0	2036	Lizbadde	2003
13470	0	0	0	2036	Yasminealjane	2007
13471	0	0	0	2039	Candiceballarin	2002
13472	0	0	0	2039	Mikaylaplitt	2004
13473	0	0	0	2039	Shawntebeale	2002
13474	0	0	0	2039	Matyldaburylo	2004
13475	0	0	0	2039	Gretafenyves	2005
13476	0	0	0	2039	Emamiyaura	2002
13477	0	0	0	2039	Ester Katerinapataki	2004
13478	0	0	0	2039	Mariagalatescu	2006
13479	0	0	0	2039	Justine Hannahmaneja	2004
13480	0	0	0	2048	Annaliolivelle	2002
13481	0	0	0	2049	Steffyjullian	2003
13482	0	0	0	2050	Eloiseswarbrick	2002
13483	0	0	0	2050	Shayfadlon	2004
13484	0	0	0	2052	Ellielogotheti	2002
13485	0	0	0	2053	Inesoliveira	2003
13486	0	0	0	2054	Kahonieda	2003
13487	0	0	0	2054	Tishyakhandelwal	2005
13488	0	0	0	2056	Juliettebegin	2003
13489	0	0	0	2057	Esterfesiuk	2004
13490	0	0	0	2057	Jagodawroblewska	2004
13491	0	0	0	2057	Athenarosas-pacifico	2006
13492	0	0	0	2060	Louiseratiu	2002
13493	0	0	0	2061	Gracehaugen	2003
13494	0	0	0	2062	Sarinahmaduro	2003
13495	0	0	0	2062	Nayumasubba	2003
13496	0	0	0	2064	Lisa Mariedugec	2003

13497	0	0	0	2065	Mariapukhina	2003
13498	0	0	0	2065	Anaveroux	2004
13499	0	0	0	2065	Savinijayasuriya	2002
13500	0	0	0	2065	Rominazeynali sourmanabadi	2005
13501	0	0	0	2065	Zhenghuitian	2002
13502	0	0	0	2065	Isabellabenavides jaramillo	2005
13503	0	0	0	2065	Sangeeprieto	2004
13504	0	0	0	2072	Victorialazarova	2004
13505	0	0	0	2073	Aniqueengelbrecht	2002
13506	0	0	0	2074	Evelinamartirosyan	2003
13507	0	0	0	2074	Aliciaowegi	2004
13508	0	0	0	2074	Pierinalescher	2005
13509	0	0	0	2077	Mariiamasiianskaia	2007
13510	0	0	0	2077	Larahrascanec	2003
13511	0	0	0	2077	Andreaocampo	2003
13512	0	0	0	2077	Sofiafranco salcedo	2002
13513	0	0	0	2077	Joanmadi	2004
13514	0	0	0	2077	Clarisseaussert	2002
13515	0	0	0	2077	Kenzingtonmann	2003
13516	0	0	0	2077	Charlottekeitel	2005
13517	0	0	0	2077	Mariamiroshnichenko	2006
13518	0	0	0	2077	Noemieoliveras	2006
13519	0	0	0	2077	Andjelalazarevic	2005
13520	0	0	0	2077	Miotisoarasendra andriananter	2006
13521	0	0	0	2077	Antoniaaragosa	2002
13522	0	0	0	2077	Charagrammatikaki	2003
13523	0	0	0	2077	Jelenavico	2006
13524	0	0	0	2077	Sarah Li-Annchan	2005
13525	0	0	0	2077	Hyeon jian	2003
13526	0	0	0	2094	Durusoke	2006
13527	0	0	0	2094	Anna mariahayek	2003
13528	0	0	0	2094	Lize-Maribeer	2004
13529	0	0	0	2094	Saniya Manojmasand	2003
13530	0	0	0	2094	Sun Minha	2004
13531	0	0	0	2094	Angelinagnezdilova	2002
13532	0	0	0	2100	Agamgamliel	2005
13533	0	0	0	2101	Rinkomatsuda	2005
13534	0	0	0	2102	Terezakrenkova	2004
13535	0	0	0	2103	Elizavetamladentseva	2003
13536	0	0	0	2103	Alinakostina	2005
13537	0	0	0	2103	Jessica Sueli	2005
13538	0	0	0	2103	Peyton Lynnanderson	2004
13539	0	0	0	2107	Sofiapinto	2004
13540	0	0	0	2107	Kristynahranacova	2004
13541	0	0	0	2109	Michelleirigoyen	2005
13542	0	0	0	2110	Vieradeeperm	2004
13543	0	0	0	2111	Rekaborsos	2003
13544	0	0	0	2111	Sarraben amor	2004
13545	0	0	0	2111	Tsitsi Clairemahere	2003
13546	0	0	0	2111	Leahgonzales edwards	2004

13547	0	0	0	2111	Chavana Shree Shemallela sree	2002
13548	0	0	0	2111	Melisabecerra gonzalez	2003
13549	0	0	0	2117	Ritatrocado	2004
13550	0	0	0	2118	Hannahlevy	2003
13551	0	0	0	2119	Zoeyasan	2003
13552	0	0	0	2119	Carolinaalonso	2007
13553	0	0	0	2119	Claudiasanchez quesada	2002
13554	0	0	0	2119	Didenazpolat	2004
13555	0	0	0	2123	Judithperello saavedra	2002
13556	0	0	0	2123	Mingfangzhu	2002
13557	0	0	0	2123	Leilaepstein	2003
13558	0	0	0	2123	Evamuller uhan	2005
13559	0	0	0	2127	Mebaeosaka	2004
13560	0	0	0	2127	Juliakuszynski	2002
13561	0	0	0	2127	Michelamontori	2002
13562	0	0	0	2127	Miaangelinaruja	2003
13563	0	0	0	2127	Mariakoroleva	2002
13564	0	0	0	2127	Ioanadumitrescu	2002
13565	0	0	0	2127	Ellieblackford	2006
13566	0	0	0	2127	Laurabrunkel	2006
13567	0	0	0	2127	Kahooyama	2005
13568	0	0	0	2127	Julietungamirai	2005
13569	0	0	0	2127	Solfaga	2004
13570	0	0	0	2138	Ameliijaswaffer-selff	2003
13571	0	0	0	2139	Rachelerimondini	2002
13572	0	0	0	2140	Rinkaoyama	2003
13573	0	0	0	2140	Nina Kaursidhu	2005
13574	0	0	0	2142	Beatrizcosta	2003
13575	0	0	0	2142	Kristinazamecnikova	2005
13576	0	0	0	2144	Chahdberriri	2005
13577	0	0	0	2144	Aaliyahhohmann	2003
13578	0	0	0	2144	Alessandrateodosescu	2006
13579	0	0	0	2144	Angelinaflachs	2005
13580	0	0	0	2144	Elisamaestri	2002
13581	0	0	0	2149	Laetitiarizk	2003
13582	0	0	0	2150	Lunadinoto fernandez	2006
13583	0	0	0	2150	Maria Elenamayet	2002
13584	0	0	0	2152	Ekaterinabremel	2002
13585	0	0	0	2153	Didibredberg canizares	2004
13586	0	0	0	2153	Alexiatrasca	2004
13587	0	0	0	2153	Melinamehrani	2003
13588	0	0	0	2156	Yunfan-shao	2004
13589	0	0	0	2157	Eleftheriakousoula	2004
13590	0	0	0	2158	Sajidsheeza	2006
13591	0	0	0	2158	Toyin Shewaasogba	2003
13592	0	0	0	2160	Charlottewijkamp	2003
13593	0	0	0	2160	Taisiasliusarenko	2002
13594	0	0	0	2160	Emiliaroslund	2002
13595	0	0	0	2160	Neyaraweerawansa	2003
13596	0	0	0	2164	Ominahonvalihanova	2004

13597	0	0	0	2164	Edanumanoglu	2005
13598	0	0	0	2166	Skylarmoody	2005
13599	0	0	0	2166	Stefanieauer	2002
13600	0	0	0	2166	Kiaracvetkovic	2003
13601	0	0	0	2169	Lydiampesmpalta	2005
13602	0	0	0	2169	Adakrajewska	2002
13603	0	0	0	2169	Alda Lizandramoreno	2003
13604	0	0	0	2169	Taniamunteanu	2002
13605	0	0	0	2169	Kei Yaucheung	2004
13606	0	0	0	2169	Elizavetachochia	2004
13607	0	0	0	2169	Ulajamar	2005
13608	0	0	0	2176	Danacharintceva	2005
13609	0	0	0	2176	Mayadutta	2006
13610	0	0	0	2176	Lentetaljard	2003
13611	0	0	0	2176	Patriciacano	2003
13612	0	0	0	2176	Nataliapapshev	2006
13613	0	0	0	2176	Mayabattle	2003
13614	0	0	0	2176	Samanthanunez	2005
13615	0	0	0	2183	Kellyhope	2004
13616	0	0	0	2184	Jia Yidong	2004
13617	0	0	0	2185	Varvaradegtiareva	2004
13618	0	0	0	2185	Emma Ceciliesorensen	2002
13619	0	0	0	2185	Kanitaselimanovic	2004
13620	0	0	0	2185	Scarlettnicholson	2006
13621	0	0	0	2185	Silvanarivero	2002
13622	0	0	0	2185	Zirongmu	2002
13623	0	0	0	2191	Ashleymckenzie	2003
13624	0	0	0	2191	Rubyfraser	2003
13625	0	0	0	2191	Tanvikhaasaravanan	2002
13626	0	0	0	2191	Ameek Kiranbatth	2002
13627	0	0	0	2195	Elenasalazar roca	2002
13628	0	0	0	2195	Romanelongueville	2005
13629	0	0	0	2195	Ashleykennedy	2003
13630	0	0	0	2195	Yangqu	2003
13631	0	0	0	2199	Maria Alejandrasandoval espin	2002
13632	0	0	0	2200	A youngjang	2003
13633	0	0	0	2201	Tajdazalokar	2004
13634	0	0	0	2201	Tajdazalokar	2004
13635	0	0	0	2201	Nildaikizoglu	2004
13636	0	0	0	2204	Nardinesalem	2007
13637	0	0	0	2204	Anaserrano	2003
13638	0	0	0	2204	Emmagretzky	2003
13639	0	0	0	2204	Katalinapetrova	2006
13640	0	0	0	2208	Gabrielarondon	2005
13641	0	0	0	2208	Lauren Chisholmchisholm	2003
13642	0	0	0	2208	Liliyadimova	2002
13643	0	0	0	2208	Melekhelal	2006
13644	0	0	0	2208	Giannaoboniye	2006
13645	0	0	0	2213	Verasekerina	2004
13646	0	0	0	2214	Karakatkoilybaeva	2006

13647	0	0	0	2214	Lauragarcia astudillo	2003
13648	0	0	0	2216	Gisellegarcia hurtado	2002
13649	0	0	0	2217	Ivarafailovic	2006
13650	0	0	0	2217	Miapopovic	2004
13651	0	0	0	2217	Sophiemartin	2004
13652	0	0	0	2217	Makennadoyle	2003
13653	0	0	0	2217	Amelijasupulniece	2004
13654	0	0	0	2217	Isabellaciancaglini	2002
13655	0	0	0	2217	Luziaobermeier	2002
13656	0	0	0	2217	Asliozcan	2004
13657	0	0	0	2217	Michaelastephens	2002
13658	0	0	0	2217	Katerynarudenko	2003
13659	0	0	0	2217	Laiagiralt	2003
13660	0	0	0	2217	Millelangaas	2003
13661	0	0	0	2217	Kaitlynnhart	2003
13662	0	0	0	2217	Jufe-Anncocoy	2005
13663	0	0	0	2217	Mariativanova	2005
13664	0	0	0	2217	Celinewan min kee	2002
13665	0	0	0	2217	Saanvitak	2004
13666	0	0	0	2234	Verayesenkova	2004
13667	0	0	0	2234	Laetitiaperona	2005
13668	0	0	0	2234	Sofiagiraldo zamorano	2005
13669	0	0	0	2234	Shradhachhabra	2002
13670	0	0	0	2234	Ksenijarage	2006
13671	0	0	0	2234	Danielgoldstein	2006
13672	0	0	0	2234	Panatchakornsuanarsa	2003
13673	0	0	0	2234	Evecostantini	2003
13674	0	0	0	2234	Sadjocoulibaly	2004
13675	0	0	0	2234	Paulasanchez rodriguez	2003
13676	0	0	0	2234	Lauravarsamaki	2005
13677	0	0	0	2234	Tanishakantam	2007
13678	0	0	0	2246	Karolinakrajmer	2006
13679	0	0	0	2246	Susanasouhrada	2006
13680	0	0	0	2246	Eunhookim	2003
13681	0	0	0	2246	Viktoriawiandt	2002
13682	0	0	0	2246	Nazarenavizcaino	2003
13683	0	0	0	2246	Renatanaka	2005
13684	0	0	0	2252	Nisabulut	2006
13685	0	0	0	2252	Vanjagudelj	2002
13686	0	0	0	2252	chakiradermane	2003
13687	0	0	0	2252	Priyanshibhandari	2003
13688	0	0	0	2252	Leanachanove	2004
13689	0	0	0	2257	Leoniehoppe	2002
13690	0	0	0	2258	Imankhan	2003
13691	0	0	0	2258	Livayildiz	2002
13692	0	0	0	2258	Annaortiz gasco	2005
13693	0	0	0	2258	Lisaryan	2004
13694	0	0	0	2258	Marinaskegro	2004
13695	0	0	0	2258	Emilialubenova	2005
13696	0	0	0	2258	Aethielle Louisekelkboom	2002

13697	0	0	0	2258	Aidapasonova	2005
13698	0	0	0	2258	Katerinagygalova	2003
13699	0	0	0	2258	Leoniemoeller	2004
13700	0	0	0	2258	Gabrielahrubanova	2003
13701	0	0	0	2258	Constantinatembrioti	2006
13702	0	0	0	2258	Danicanedeljkovic	2005
13703	0	0	0	2258	Valentinasteiner	2006
13704	0	0	0	2272	Srihithajaligama	2005
13705	0	0	0	2272	Varvarapopova	2004
13706	0	0	0	2272	Kamolaamanova	2005
13707	0	0	0	2272	Daryavolkova	2003
13708	0	0	0	2272	Renee Marieacena	2002
13709	0	0	0	2272	HYERIMjung	2003
13710	0	0	0	2278	Camillekiss	2002
13711	0	0	0	2278	Iuliabucea	2005
13712	0	0	0	2280	Isabella Nalanialvarez fu	2005
13713	0	0	0	2280	Hong Kiukwok	2002
13714	0	0	0	2282	Laurelpolman	2004
13715	0	0	0	2282	Sahradennehy	2002
13716	0	0	0	2282	Makhiraalimova	2003
13717	0	0	0	2282	Nikolmircheva	2004
13718	0	0	0	2286	Yevakramarova	2004
13719	0	0	0	2286	Sarasimonova	2003
13720	0	0	0	2288	Juliettebahri	2005
13721	0	0	0	2288	Alannadi francesco	2003
13722	0	0	0	2288	Gaeunlee	2004
13723	0	0	0	2288	Tanatswamusabaeka	2005
13724	0	0	0	2288	Hayajudeh	2003
13725	0	0	0	2288	Hanasonton	2005
13726	0	0	0	2288	Suminkim	2002
13727	0	0	0	2288	Maleehakhalid	2004
13728	0	0	0	2288	Anastasijastojanovska	2003
13729	0	0	0	2288	Ulianakarmalina	2002
13730	0	0	0	2288	mionayasukawa	2004
13731	0	0	0	2288	Juliaviesi santoro pereira	2006
13732	0	0	0	2288	Saramorais	2003
13733	0	0	0	2288	Daniellao'neill garcia	2004
13734	0	0	0	2302	Melinechaumeil	2004
13735	0	0	0	2302	Anastasiatsouchnenko	2003
13736	0	0	0	2302	Annademirtzi	2007
13737	0	0	0	2302	Soniaskobkareva	2003
13738	0	0	0	2302	Manuelagomez gomez	2004
13739	0	0	0	2302	Tanyananda	2003
13740	0	0	0	2302	Mariiamaksimova	2003
13741	0	0	0	2309	Shantalauzcategui	2002
13742	0	0	0	2309	Sashamotlagh	2005
13743	0	0	0	2309	Paulinaloretz	2003
13744	0	0	0	2309	Shylakhattar	2004
13745	0	0	0	2309	Loladecoux	2004
13746	0	0	0	2309	Juliettedoukhan	2004

13747	0	0	0	2309	Alexandramaksimova	2003
13748	0	0	0	2309	Jyun Yilee	2003
13749	0	0	0	2309	Sarahintrocaso	2003
13750	0	0	0	2309	Pimmadalim	2007
13751	0	0	0	2309	Juliaorlova	2003
13752	0	0	0	2309	Lrunralisebusadee	2005
13753	0	0	0	2309	YUN-YUNtsai	2004
13754	0	0	0	2309	Oceannelopez	2004
13755	0	0	0	2309	Shanayanaik	2002
13756	0	0	0	2309	Elizavetacherednyk	2004
13757	0	0	0	2309	Ananyavartak	2003
13758	0	0	0	2326	Liliminich	2002
13759	0	0	0	2326	FARIDAelhalaby	2005
13760	0	0	0	2328	Aminaarnaout	2004
13761	0	0	0	2328	Annemarielazar	2002
13762	0	0	0	2328	seonghyeonson	2003
13763	0	0	0	2328	Leonoroliveira	2002
13764	0	0	0	2332	Veronikaudina	2005
13765	0	0	0	2333	Asyasinitsyna	2002
13766	0	0	0	2333	Clairemachisa	2004
13767	0	0	0	2333	Annakalabukhova	2002
13768	0	0	0	2333	Sahrabouchaala	2002
13769	0	0	0	2337	Mariamgabr	2005
13770	0	0	0	2337	Jaynaclemens	2004
13771	0	0	0	2337	Diyoraalzhanova	2002
13772	0	0	0	2340	Maria Fernandapadilla jaimes	2003
13773	0	0	0	2340	Samanthaimbo nloga	2003
13774	0	0	0	2340	Janetimbo nloga	2002
13775	0	0	0	2343	Yoanamoneva	2005
13776	0	0	0	2343	Louisehosking	2002
13777	0	0	0	2343	Fernandacota	2002
13778	0	0	0	2343	katrinesadze	2004
13779	0	0	0	2343	Salomefluri	2005
13780	0	0	0	2343	Silaak	2004
13781	0	0	0	2343	Ariadnabriones ginesta	2002
13782	0	0	0	2343	luanaseyboth wild	2002
13783	0	0	0	2343	Chin-tinghuang	2005
13784	0	0	0	2352	Shuangliang	2003
13785	0	0	0	2352	Anastasiasereda	2005
13786	0	0	0	2352	Kairatan	2007
13787	0	0	0	2352	Mathildelangevin	2002
13788	0	0	0	2352	NAGA ROSHNEarunkumar	2004
13789	0	0	0	2352	Valeriaray	2005
13790	0	0	0	2352	Kristiboro	2004
13791	0	0	0	2352	Carmenjuan bru	2005
13792	0	0	0	2352	Julianegillespie	2002
13793	0	0	0	2352	Janapremovic	2007
13794	0	0	0	2352	Mayaeastburg	2007
13795	0	0	0	2352	Larabauerberg	2003
13796	0	0	0	2352	Marinamarkina	2006

13797	0	0	0	2352	Shriaatturu	2002
13798	0	0	0	2352	Marie Laurencedenis	2004
13799	0	0	0	2352	Aokanagasawa	2005
13800	0	0	0	2352	Erykakruk	2005
13801	0	0	0	2352	Juliakaemmerer	2005
13802	0	0	0	2352	Alisonazarak	2003
13803	0	0	0	2352	Amandamenendez thomasson	2003
13804	0	0	0	2372	Galaarangio	2005
13805	0	0	0	2372	Adelisakahriman	2005
13806	0	0	0	2372	Saragonzalez	2003
13807	0	0	0	2372	Nicolewuelfing	2004
13808	0	0	0	2372	Lilyaraiss	2005
13809	0	0	0	2372	Livsumak	2004
13810	0	0	0	2378	Lottaheiskanen	2002
13811	0	0	0	2378	Nadiaabad	2003
13812	0	0	0	2378	Stellajurina	2005
13813	0	0	0	2381	Mia Vukicamilojevic	2003
13814	0	0	0	2381	Sibleycharles	2005
13815	0	0	0	2381	Emilieleroy	2004
13816	0	0	0	2381	Mahrazrazavian	2004
13817	0	0	0	2381	Milabadics	2004
13818	0	0	0	2381	Bridgethan	2003
13819	0	0	0	2381	Annarahman	2005
13820	0	0	0	2381	Tak Yufung	2003
13821	0	0	0	2381	Sofia Janewong enqi	2006
13822	0	0	0	2381	Miagarber	2005
13823	0	0	0	2381	Kaedehatano	2002
13824	0	0	0	2381	Shira Rachelsivan	2005
13825	0	0	0	2381	Marissaminetti	2005
13826	0	0	0	2381	Qian Erqiu	2002
13827	0	0	0	2381	Chulapatsathutham	2005
13828	0	0	0	2381	Zoelfanny Wahyuningrumsaput	2002
13829	0	0	0	2381	Mayaboonyaaroonnet	2007
13830	0	0	0	2381	Nourdib	2002
13831	0	0	0	2381	Renatacastillo	2002
13832	0	0	0	2381	Rhythmaswal	2002
13833	0	0	0	2401	Morganpyrz	2004
13834	0	0	0	2402	Charlottetse	2004
13835	0	0	0	2402	Lauramair	2003
13836	0	0	0	2402	Alexandrastepanova	2004
13837	0	0	0	2402	Dariazhurid	2006
13838	0	0	0	2402	Aleksandragabrovska	2006
13839	0	0	0	2402	Omolayobamidele	2002
13840	0	0	0	2402	Jordangrayson	2003
13841	0	0	0	2402	Chisatokanemaki	2006
13842	0	0	0	2402	Rhonacook	2002
13843	0	0	0	2402	Melina Mariamaruca	2002
13844	0	0	0	2402	Silvia Alysiapomarolli	2003
13845	0	0	0	2402	Yeong-Eunryu	2004
13846	0	0	0	2402	Liyapencheva	2004

13847	0	0	0	2415	Saraikeba	2002
13848	0	0	0	2415	Brittvan zeelst	2003
13849	0	0	0	2415	Lilianadrukerova	2002
13850	0	0	0	2415	Baylenbrown	2004
13851	0	0	0	2415	Luiza Caixetaparreira	2003
13852	0	0	0	2415	Terezapolakova	2004
13853	0	0	0	2415	Ekaterinaivanova	2006
13854	0	0	0	2415	Brianastraut	2002
13855	0	0	0	2415	Patricia Georgianagoina	2005
13856	0	0	0	2424	Yuxuanli	2002
13857	0	0	0	2424	Sophiegreiner	2003
13858	0	0	0	2424	Rosariolouro	2002
13859	0	0	0	2424	Carol Vanesamora camacho	2003
13860	0	0	0	2424	VALENTINAgarces vergara	2004
13861	0	0	0	2424	Paolaprieto	2003
13862	0	0	0	2424	Claudia Alexaalvarado lara	2004
13863	0	0	0	2424	Jacquelineschnabel	2004
13864	0	0	0	2424	Sai Diyabalaji	2003
13865	0	0	0	2433	Sunskrithidamera	2003
13866	0	0	0	2433	Giannarosato	2003
13867	0	0	0	2433	Gaiagreco	2006
13868	0	0	0	2433	Anastasijaignjatic	2006
13869	0	0	0	2433	Mille Johannamoerk	2006
13870	0	0	0	2433	Rodianoudogbessi	2005
13871	0	0	0	2433	Emilysartz-lunde	2006
13872	0	0	0	2433	Neriyayona	2004
13873	0	0	0	2433	Desireparn	2005
13874	0	0	0	2433	Petramirkovic	2006
13875	0	0	0	2433	Josieusereau	2005
13876	0	0	0	2433	Nathaliamossambani	2003
13877	0	0	0	2433	Blankaslukova	2003
13878	0	0	0	2433	Asmaben abdallah	2006
13879	0	0	0	2433	Annguerry	2004
13880	0	0	0	2433	Fridahoungbo	2003
13881	0	0	0	2433	Nedamilenkovic	2004
13882	0	0	0	2433	Nikolkedun	2007
13883	0	0	0	2433	Anne Christinelutkemeyer obre	2004
13884	0	0	0	2433	Amandabikker	2003
13885	0	0	0	2433	Sagakoch	2003
13886	0	0	0	2433	Melisaugur	2007
13887	0	0	0	2433	Valeriacenteno mendoza	2004
13888	0	0	0	2433	Maria Fernandagouveia	2002
13889	0	0	0	2433	Isadora Fernandamuller	2002
13890	0	0	0	2433	Meganlang-gould	2002
13891	0	0	0	2433	Myroslavazelenchuk	2004
13892	0	0	0	2433	Kseniaminaeva	2004
13893	0	0	0	2433	Isabellanicholson	2003
13894	0	0	0	2433	Farhataleen qamar	2002
13895	0	0	0	2433	Marentesijbesma	2004
13896	0	0	0	2433	Tajrajugic	2003

13897	0	0	0	2433	Sotiriakatsali	2006
13898	0	0	0	2433	Aminakadic	2005
13899	0	0	0	2433	Valeriaschad	2002
13900	0	0	0	2433	Olwynryan bovey	2003
13901	0	0	0	2433	Kotisthamodak	2004
13902	0	0	0	2433	Mienkeviljoen	2003
13903	0	0	0	2433	Lydiazafeiri	2003
13904	0	0	0	2433	Emmanamnieks	2005
13905	0	0	0	2433	Emiliiamargolina	2004
13906	0	0	0	2433	Nadjanonkovic	2002
13907	0	0	0	2433	Valeriiatroitckaia	2006
13908	0	0	0	2433	Sirikongara	2003
13909	0	0	0	2433	Maria Carolinaferreira turchett	2004
13910	0	0	0	2433	Kristinanikiforova	2005
13911	0	0	0	2433	Lakshmi Sahithi Reddyvutukuru	2002
13912	0	0	0	2433	Anna Mariatottrup	2005
13913	0	0	0	2433	Camilaromero oller	2002
13914	0	0	0	2433	Tatianamuzykantskaya	2004
13915	0	0	0	2433	Niehuatian	2006
13916	0	0	0	2433	Mackenziewhite	2002
13917	0	0	0	2433	Ishitadahutia	2004
13918	0	0	0	2433	Nataliamamay	2002
13919	0	0	0	2433	Clarade santiago vinaixa	2002
13920	0	0	0	2488	Vidisha Reddypallamreddy	2002
13921	0	0	0	2488	Likaperesypkina	2005
13922	0	0	0	2488	Bala Gayathri Snigdhaangam	2002
13923	0	0	0	2488	Sarahibrahimagic	2004
13924	0	0	0	2488	Sakonakaoka	2005
13925	0	0	0	2488	Karim Lissetcarreras lampert	2002
13926	0	0	0	2488	Camillegrimoux	2006
13927	0	0	0	2488	Capucinesanoner	2002
13928	0	0	0	2488	Keesalee young	2004
13929	0	0	0	2488	Anishaapte	2002
13930	0	0	0	2488	Kotoitakeshi	2005
13931	0	0	0	2488	Annetteklettenberg	2004
13932	0	0	0	2488	Alexia Iuliamarginean	2006
13933	0	0	0	2488	Mayraarce gonzalez	2005
13934	0	0	0	2488	Jeninaishii	2003
13935	0	0	0	2488	Almaorenstain	2005
13936	0	0	0	2488	Anishaapte	2002
13937	0	0	0	2488	Vidisha Reddypallamreddy	2002
13938	0	0	0	2488	Annemariecristmann	2003
13939	0	0	0	2488	Julialintu	2006
13940	0	0	0	2488	Ayunanakazato	2007
13941	0	0	0	2488	Andrea Alejandranoguera men	2007
13942	0	0	0	2488	Karim Lissetcarreras lampert	2002
13943	0	0	0	2488	Capucinesanoner	2002
13944	0	0	0	2488	Cydneycrumbie	2003
13945	0	0	0	2488	Sakonakaoka	2005
13946	0	0	0	2488	Mayraarce gonzalez	2005

13947	0	0	0	2488	Catalinapadilla udaeta	2005
13948	0	0	0	2488	Annetteklettenberg	2004
13949	0	0	0	2488	Oliviasmith	2004
13950	0	0	0	2488	Kailabarksdale	2004
13951	0	0	0	2488	Alexia Iuliamarginean	2006
13952	0	0	0	2488	Likaperesypkina	2005
13953	0	0	0	2488	Cristinapescucci	2003
13954	0	0	0	2488	Mariasysoeva	2006
13955	0	0	0	2488	Sarahibrahimagic	2004
13956	0	0	0	2524	Stellaerlund	2002
13957	0	0	0	2524	Kennedygibbs	2004
13958	0	0	0	2524	Jaelschwarz	2004
13959	0	0	0	2524	Margaritachouliara	2002
13960	0	0	0	2524	Gabrielafelix da silva	2006
13961	0	0	0	2524	Maria Isabelpereira	2002
13962	0	0	0	2524	Kristinamikhailova	2005
13963	0	0	0	2531	Vittoriamodesti	2002
13964	0	0	0	2531	Vasileiagriva	2002
13965	0	0	0	2531	Elena Giovannagiessler	2002
13966	0	0	0	2531	Lafilissajoao	2002
13967	0	0	0	2531	Amandamiller	2002
13968	0	0	0	2531	Giuliapaterno	2003
13969	0	0	0	2531	Annasidorenko	2005
13970	0	0	0	2538	Naomixu	2006
13971	0	0	0	2538	Zvikomborero Teresa Chelsemt	2006
13972	0	0	0	2538	Taisiayakunina	2004
13973	0	0	0	2538	Charlotte Rutendozhuwakini	2002
13974	0	0	0	2538	Michelacastro zunino	2006
13975	0	0	0	2538	Pariyakornsrisuriyapatkul	2007
13976	0	0	0	2538	Mariellagelbke	2005
13977	0	0	0	2538	Chiarafiorenza	2003
13978	0	0	0	2538	Boglarkacsordas	2002
13979	0	0	0	2538	Taraphan	2006
13980	0	0	0	2538	Maddisonricardo	2004
13981	0	0	0	2538	Anastasijaadeikyte	2004
13982	0	0	0	2538	Shalomsalvi	2003
13983	0	0	0	2538	Ivakuzmanoska	2004
13984	0	0	0	2538	Letiziacorsini	2003
13985	0	0	0	2538	Sofiesiem	2003
13986	0	0	0	2538	Sofikozeikidze	2004
13987	0	0	0	2538	Alexis Larachua	2005
13988	0	0	0	2538	Hannahwalpole	2003
13989	0	0	0	2538	Paulinewalpot	2004
13990	0	0	0	2538	Camilacorrales	2004
13991	0	0	0	2538	Islaythornicroft	2003
13992	0	0	0	2538	Mirandayuan	2002
13993	0	0	0	2538	Serenabryan	2005
13994	0	0	0	2538	Luanaplaza	2005
13995	0	0	0	2538	Katyabalin	2004
13996	0	0	0	2538	Donyaameri	2004

13997	0	0	0	2538	Francabucco paolasso	2002
13998	0	0	0	2566	Charlyegger	2004
13999	0	0	0	2566	Yukinakubo	2004
14000	0	0	0	2566	Sanaahewitt	2002
14001	0	0	0	2569	Andreasydow	2003
14002	0	0	0	2569	Sabrinasanchez	2003
14003	0	0	0	2569	Rachele Elisazingale	2002
14004	0	0	0	2569	Danielaaguilar	2006
14005	0	0	0	2569	Ivajovanova	2006
14006	0	0	0	2569	Nadejdamaslova	2002
14007	0	0	0	2569	Thekliachristodoulou	2004
14008	0	0	0	2569	Sararakim	2003
14009	0	0	0	2569	Josefinaramirez	2003
14010	0	0	0	2578	Vanshitamalhotra	2002
14011	0	0	0	2578	Muslimaakhunova	2002
14012	0	0	0	2578	Nicolelukesova	2006
14013	0	0	0	2578	DANIELAchaves	2004
14014	0	0	0	2578	Mayaperkucin	2002
14015	0	0	0	2583	Ayabychenko	2005
14016	0	0	0	2583	Orianagrott	2003
14017	0	0	0	2583	Min Juma	2003
14018	0	0	0	2583	Madeleinejoffe	2005
14019	0	0	0	2583	Lararossetto diniz de souza	2004
14020	0	0	0	2583	Xiaotongzhao	2004
14021	0	0	0	2583	Anjastankovic	2005
14022	0	0	0	2583	Mialjubas	2002
14023	0	0	0	2583	Mayaarksey	2002
14024	0	0	0	2583	Andjelinagnjdic	2004
14025	0	0	0	2583	Nanahoyokota	2003
14026	0	0	0	2583	Shania Kaurgadhia	2003
14027	0	0	0	2583	Valentinatorres da silva	2004
14028	0	0	0	2583	Clairejaramba	2004
14029	0	0	0	2583	Ioana Teodorasava	2005
14030	0	0	0	2583	Meganlombardt	2002
14031	0	0	0	2583	Jadaani	2003
14032	0	0	0	2600	Fatmamornagui	2004
14033	0	0	0	2601	Alanarepanich	2002
14034	0	0	0	2601	Emmacohen	2005
14035	0	0	0	2601	Simoanerodgers	2004
14036	0	0	0	2601	Anastasiaparaskevoudi	2002
14037	0	0	0	2601	Elenasamuelsson	2005
14038	0	0	0	2601	Ana Mariatornean	2004
14039	0	0	0	2601	Danielaluciano	2003
14040	0	0	0	2601	Suhyunlim	2002
14041	0	0	0	2601	Alanarepanich	2002
14042	0	0	0	2601	Alinagavrish	2003
14043	0	0	0	2601	Svevabernardi	2004
14044	0	0	0	2601	Marinahristova	2003
14045	0	0	0	2601	nataliewagner	2003
14046	0	0	0	2601	Chutnareeboonklang	2004

14047	0	0	0	2601	Elananasr	2003
14048	0	0	0	2601	Randy Ikoriantsoarakotoarilala	2005
14049	0	0	0	2601	Sin Yuche	2005
14050	0	0	0	2601	Nahyeonggwak	2003
14051	0	0	0	2601	Nasibaespolova	2002
14052	0	0	0	2601	Puckapornjirachavala	2004
14053	0	0	0	2601	Patriciabordas	2002
14054	0	0	0	2601	Sofia Constanzakarlau	2003
14055	0	0	0	2601	Violetanikolayeva	2004
14056	0	0	0	2601	Maria Victoriagraff	2003
14057	0	0	0	2601	Anaisperalta criado	2002
14058	0	0	0	2601	Jomkwanvettayawaikoon	2005
14059	0	0	0	2627	Sofiaciobica	2005
14060	0	0	0	2627	Selmamoustaadil	2006
14061	0	0	0	2627	Angelinamelki	2002
14062	0	0	0	2627	Ninawalter	2003
14063	0	0	0	2627	Inesbollecker	2006
14064	0	0	0	2627	Darisakhanova	2003
14065	0	0	0	2633	Andrasutherland	2004
14066	0	0	0	2633	Malakarafat	2004
14067	0	0	0	2633	Laurafuchs	2005
14068	0	0	0	2633	Isabellachhiv	2005
14069	0	0	0	2633	Emelinehonfoga	2003
14070	0	0	0	2633	Anushkaashar	2004
14071	0	0	0	2633	Katehuizenga	2002
14072	0	0	0	2633	Ji Hyunkang	2005
14073	0	0	0	2633	Kategardiner	2002
14074	0	0	0	2633	Ivadaneva	2005
14075	0	0	0	2633	Toffeeghorab	2007
14076	0	0	0	2644	Elizabethtkachenko	2004
14077	0	0	0	2644	Lunamorini	2003
14078	0	0	0	2644	Vanyaarora	2005
14079	0	0	0	2644	Lenacouto	2007
14080	0	0	0	2644	Diana Mariailie	2006
14081	0	0	0	2644	Veronikapodobed	2004
14082	0	0	0	2644	Kelly Marierichter	2005
14083	0	0	0	2644	Taldekel	2003
14084	0	0	0	2644	Elsapascual	2002
14085	0	0	0	2644	Alexia-Sharaiancu	2006
14086	0	0	0	2644	Florencejoule	2004
14087	0	0	0	2655	Sarahrokusek	2006
14088	0	0	0	2655	Yaraalhaqbani	2004
14089	0	0	0	2655	Giuliabarberini	2003
14090	0	0	0	2655	Joannahee	2007
14091	0	0	0	2655	Randaalqudsi	2004
14092	0	0	0	2655	Yukikawa	2006
14093	0	0	0	2655	Abigailthurmer	2003
14094	0	0	0	2655	Xinyuhe	2002
14095	0	0	0	2655	Sarah-Annewong anqi	2006
14096	0	0	0	2655	Taewatanabe	2004

14097	0	0	0	2655	Caglateoman	2002
14098	0	0	0	2655	Cynthiawanjala	2005
14099	0	0	0	2655	Aminasuleymanova	2004
14100	0	0	0	2655	KESARINunsiri	2004
14101	0	0	0	2655	Siddhikhot	2005
14102	0	0	0	2655	Adrianatabares	2002
14103	0	0	0	2655	Rinahosni	2002
14104	0	0	0	2655	Nicolbenesova	2004
14105	0	0	0	2655	Jovananikolic	2003
14106	0	0	0	2655	Annawise	2005
14107	0	0	0	2655	Sofyakashchenko	2005
14108	0	0	0	2655	Paigegilbert	2003
14109	0	0	0	2655	Raeganpine	2003
14110	0	0	0	2655	Estherromero garcia	2004
14111	0	0	0	2655	Elavakaukamea	2002
14112	0	0	0	2655	Pearliezhang	2006
14113	0	0	0	2655	Lironloiter	2006
14114	0	0	0	2655	Laimavladson	2007
14115	0	0	0	2655	Maholyrazakaniaina	2005
14116	0	0	0	2655	Evabaziv	2003
14117	0	0	0	2655	E Wenwee	2006
14118	0	0	0	2655	Tanyaradzwakaome	2002
14119	0	0	0	2655	Alyssarichter	2003
14120	0	0	0	2655	Janakhatib	2003
14121	0	0	0	2655	Danielaben hamo	2004
14122	0	0	0	2655	Lucianamendoza	2005
14123	0	0	0	2655	Kynadecruy	2005
14124	0	0	0	2655	Dorakurdi	2002
14125	0	0	0	2655	Salomesierra	2002
14126	0	0	0	2655	Deliagaillard	2006
14127	0	0	0	2655	Lenathomas iglesias	2006
14128	0	0	0	2655	Architamahalwal	2002
14129	0	0	0	2655	Saramilanese	2004
14130	0	0	0	2655	Bhumikatripathi	2004
14131	0	0	0	2655	Malenacanon	2003
14132	0	0	0	2655	Anastasiiakhriapko	2003
14133	0	0	0	2655	Irelandsimme	2004
14134	0	0	0	2655	Ekaterinadoroshenko	2005
14135	0	0	0	2655	Giuliabarberini	2003
14136	0	0	0	2655	Raeganpine	2003
14137	0	0	0	2655	Xinyuhe	2002
14138	0	0	0	2655	Dorakurdi	2002
14139	0	0	0	2655	Himadrikashyap	2002
14140	0	0	0	2655	Joannahee	2007
14141	0	0	0	2655	Aidaoviedo	2006
14142	0	0	0	2710	Dariagrigorenko	2003
14143	0	0	0	2710	Chems Dohazeghlouli	2004
14144	0	0	0	2710	Ajabroqi	2005
14145	0	0	0	2710	Alexandrabarcikova	2004
14146	0	0	0	2710	Elliealdrich	2002

14147	0	0	0	2710	Adeliyamukhutdinova	2003
14148	0	0	0	2710	Abigalecotuna	2003
14149	0	0	0	2710	Ekaterinaburenina	2004
14150	0	0	0	2710	Remikaohashi	2003
14151	0	0	0	2710	Estefaniagonzalez	2003
14152	0	0	0	2710	Ellafaessler	2004
14153	0	0	0	2710	Patriciaungure rogozoveca	2005
14154	0	0	0	2722	kristinavergazova	2002
14155	0	0	0	2722	Kikirijk	2003
14156	0	0	0	2722	Annikahakovirta	2002
14157	0	0	0	2722	Isabellaflodin	2002
14158	0	0	0	2722	Katesharabura	2003
14159	0	0	0	2722	Simranchhabra	2002
14160	0	0	0	2722	Zsofiamihalka	2004
14161	0	0	0	2722	Daryavelikova	2004
14162	0	0	0	2722	Anaristevska	2002
14163	0	0	0	2722	Ariannanepomuceno	2002
14164	0	0	0	2722	Annamaliugina	2004
14165	0	0	0	2722	Irisatursunova	2003
14166	0	0	0	2722	Hannavasconez teran	2004
14167	0	0	0	2722	Yifeihe	2002
14168	0	0	0	2722	Nairagevorkian	2006
14169	0	0	0	2722	Hana Aymanelsherif	2004
14170	0	0	0	2722	Dartavorslava	2004
14171	0	0	0	2722	Paloma Florenciacaceres villalb	2004
14172	0	0	0	2722	Isabellanunez	2002
14173	0	0	0	2722	Serenateluwo	2006
14174	0	0	0	2722	Evdokiiapopova	2004
14175	0	0	0	2743	Natthanichakinnaree	2002
14176	0	0	0	2743	Olesyaborozdenko	2004
14177	0	0	0	2743	Kristynafantova	2002
14178	0	0	0	2743	Sie Dingchai	2002
14179	0	0	0	2743	Polinagubina	2002
14180	0	0	0	2743	Sylviekalouskova	2002
14181	0	0	0	2743	Luciekubikova	2003
14182	0	0	0	2743	Daphneykoutsofta	2004
14183	0	0	0	2743	Emiliadurska	2004
14184	0	0	0	2752	Doramiskovic	2007
14185	0	0	0	2752	Smiltebakyte	2004
14186	0	0	0	2752	Lucyfurness	2002
14187	0	0	0	2752	Eleanornobbs	2003
14188	0	0	0	2752	Oliwiaorlinska	2005
14189	0	0	0	2752	Mariataneva	2004
14190	0	0	0	2752	Victoriasubev	2002
14191	0	0	0	2752	Alexandramcdonald	2004
14192	0	0	0	2752	Mialeshem	2004
14193	0	0	0	2752	Olgalesnikova	2002
14194	0	0	0	2752	Annaaagaard nielsen	2002
14195	0	0	0	2752	Bentebierma	2004
14196	0	0	0	2752	Lucianagalvez llanes	2003

14197	0	0	0	2752	Dalidaqadaria	2002
14198	0	0	0	2752	Nikitasalkar	2002
14199	0	0	0	2752	Beatricestagno	2003
14200	0	0	0	2752	Anipapyan	2003
14201	0	0	0	2752	Ruslanakovalenko	2003
14202	0	0	0	2752	Kseniamamontova	2003
14203	0	0	0	2752	Zeynepilker	2005
14204	0	0	0	2752	Mariamunhoz	2005
14205	0	0	0	2752	Simonaangelova	2004
14206	0	0	0	2752	Zoispyrou	2003
14207	0	0	0	2752	Juminkong	2004
14208	0	0	0	2752	Janicdurocher	2005
14209	0	0	0	2752	Sharvanishrestha	2005
14210	0	0	0	2752	Yunamiyauchi	2004
14211	0	0	0	2752	Feiluo	2004
14212	0	0	0	2752	Alexandrazimmer	2006
14213	0	0	0	2752	Ekaterinastrizhkova	2004
14214	0	0	0	2752	Annatang	2004
14215	0	0	0	2752	Ceciliewiche	2002
14216	0	0	0	2752	Naomischraeder	2005
14217	0	0	0	2752	Alexandraalafinova	2004
14218	0	0	0	2752	Malanniavashkevich	2004
14219	0	0	0	2752	Momokofujita	2004
14220	0	0	0	2752	Carla Lorenatorrico	2002
14221	0	0	0	2752	Ema Albinablahova	2003
14222	0	0	0	2752	Florencefedeli	2003
14223	0	0	0	2752	Aengikuoni	2004
14224	0	0	0	2752	Moira Ellesilva	2004
14225	0	0	0	2752	Noelle Vanassewilbert	2004
14226	0	0	0	2752	Fatmamathlouthi	2004
14227	0	0	0	2752	Daria Mariatudor	2004
14228	0	0	0	2752	Parisayoub	2002
14229	0	0	0	2752	Sahithyavederea	2002
14230	0	0	0	2752	Chiarali yaw hay	2004
14231	0	0	0	2752	Wing Yan Emilychan	2004
14232	0	0	0	2752	Valeriaporras buezo	2004
14233	0	0	0	2752	Oleksandrasmirnova	2003
14234	0	0	0	2752	Wing Yan Emilychan	2004
14235	0	0	0	2752	Beatricestagno	2003
14236	0	0	0	2752	Mei Lingjoensen-lee	2006
14237	0	0	0	2752	Inesbergaoui	2004
14238	0	0	0	2752	Varvaraalexeyeva	2005
14239	0	0	0	2752	Alexandraalafinova	2004
14240	0	0	0	2752	Yixinzhang	2004
14241	0	0	0	2752	Yvonnesubev	2005
14242	0	0	0	2752	Mariyavyshkina	2004
14243	0	0	0	2752	Srishtidhir	2002
14244	0	0	0	2752	Naomischraeder	2005
14245	0	0	0	2752	Yuzurihanakanii	2005
14246	0	0	0	2752	Rubyyoung	2006

14247	0	0	0	2752	Simonaangelova	2004
14248	0	0	0	2752	Nandinidixit	2005
14249	0	0	0	2752	Medareimoryte	2003
14250	0	0	0	2752	Samanthahorwood	2003
14251	0	0	0	2752	Alana Norahseneviratne	2003
14252	0	0	0	2752	Paula Ysabelleuy	2002
14253	0	0	0	2752	Amreensidhu	2003
14254	0	0	0	2752	Praewajongjit	2004
14255	0	0	0	2752	Luzmary Caribaydiaz castillo	2003
14256	0	0	0	2752	Jazzminconrad	2004
14257	0	0	0	2752	Sirenawaas	2006
14258	0	0	0	2752	Veronikapoliakova	2003
14259	0	0	0	2752	Tomimain	2003
14260	0	0	0	2752	Anastasijaradojevic	2002
14261	0	0	0	2752	Mariakarachaliou	2004
14262	0	0	0	2752	Charlotteready	2005
14263	0	0	0	2752	Sharvanishrestha	2005
14264	0	0	0	2752	Constanzagomez	2002
14265	0	0	0	2752	Ayse Bilgesuyurdakul	2004
14266	0	0	0	2752	Clarissaflach seibel	2004
14267	0	0	0	2752	Katherinedibbs	2004
14268	0	0	0	2752	Anastasiasafyanova	2004
14269	0	0	0	2752	Parisayoub	2002
14270	0	0	0	2752	Camilamontoya galvis	2006
14271	0	0	0	2752	Juliacaffarena	2006
14272	0	0	0	2752	Anipapyan	2003
14273	0	0	0	2752	Daniela Valentinatijaro	2006
14274	0	0	0	2752	Chiarali yaw hay	2004
14275	0	0	0	2752	Daria Mariatudor	2004
14276	0	0	0	2752	Victoriasubev	2002
14277	0	0	0	2752	An Sofiebeckers	2004
14278	0	0	0	2752	Doramiskovic	2007
14279	0	0	0	2752	janaabdul razzak	2004
14280	0	0	0	2752	Janicdurocher	2005
14281	0	0	0	2752	Keila Mangestipaath	2005
14282	0	0	0	2752	Aoishiro	2004
14283	0	0	0	2752	Cheyennehoukes	2003
14284	0	0	0	2752	Annaaagaard nielsen	2002
14285	0	0	0	2752	Valentinaurraco	2003
14286	0	0	0	2752	Mialeshem	2004
14287	0	0	0	2752	Beatricecampomori	2003
14288	0	0	0	2752	Wing Yinlaw	2002
14289	0	0	0	2752	Carla Lorenatorrico	2002
14290	0	0	0	2752	Moira Ellesilva	2004
14291	0	0	0	2752	Oliwiaorlinska	2005
14292	0	0	0	2752	Hannalevy	2005
14293	0	0	0	2752	Varvarapanshina	2006
14294	0	0	0	2752	Ioannapapastamouli	2006
14295	0	0	0	2752	Valentinaponce serrano	2006
14296	0	0	0	2752	Maria Fernandahatem arias	2004

14297	0	0	0	2752	Alexisbruce	2003
14298	0	0	0	2752	Valeriaporras buezo	2004
14299	0	0	0	2752	Ruvarashe Lyndahshoko	2005
14300	0	0	0	2752	Nazwasyamsabilla	2005
14301	0	0	0	2752	Andreavargas lau	2005
14302	0	0	0	2752	Erufujii	2004
14303	0	0	0	2752	Momokofujita	2004
14304	0	0	0	2752	Feiluo	2004
14305	0	0	0	2752	Bentebierma	2004
14306	0	0	0	2752	Zeynepilker	2005
14307	0	0	0	2875	Shakiravarese	2006
14308	0	0	0	2875	Reginaalcobe garibay	2006
14309	0	0	0	2875	Florencebreton	2003
14310	0	0	0	2875	Alexia Laviniapuiac	2005
14311	0	0	0	2875	Gehadel kashef	2005
14312	0	0	0	2875	Hannabougouffa	2004
14313	0	0	0	2875	Yuliiakhmara	2005
14314	0	0	0	2875	Aalishaalexis	2004
14315	0	0	0	2875	Celiabastin	2004
14316	0	0	0	2875	Biancabulat	2005
14317	0	0	0	2875	DANIELA GUADALUPEherrera c	2002
14318	0	0	0	2875	Andreamrvic	2003
14319	0	0	0	2875	Alisagolynker	2002
14320	0	0	0	2875	Anaitarutyunyan	2003
14321	0	0	0	2875	Astamiettinen	2004
14322	0	0	0	2875	Charlottabuss	2004
14323	0	0	0	2891	Georgiahosking	2002
14324	0	0	0	2891	Maria Cristinadiaz-nazario	2003
14325	0	0	0	2891	Erikadi muzio	2004
14326	0	0	0	2891	Elengrab	2004
14327	0	0	0	2891	Lingsu	2002
14328	0	0	0	2891	Metamarenk	2003
14329	0	0	0	2891	Oliviathiemke	2003
14330	0	0	0	2891	Habibafarouk	2004
14331	0	0	0	2891	Carlota Mariapavia sanchez	2002
14332	0	0	0	2891	Thamarafrasser kawaratani	2005
14333	0	0	0	2891	Aliyaabbas	2005
14334	0	0	0	2891	Hsin-Yili	2004
14335	0	0	0	2891	Thamarafrasser kawaratani	2005
14336	0	0	0	2891	Karolinarajca	2005
14337	0	0	0	2891	Marianacampos	2004
14338	0	0	0	2891	Katiaandre	2002
14339	0	0	0	2891	Viola Hajnajeck	2007
14340	0	0	0	2891	Selenekentish	2003
14341	0	0	0	2891	Chiaradi genova	2004
14342	0	0	0	2891	Milanachandelier	2006
14343	0	0	0	2891	Ting-Anchen	2003
14344	0	0	0	2891	Palomakeresztes	2002
14345	0	0	0	2913	Alanasubasic	2007
14346	0	0	0	2913	Charnemulder	2006

14347	0	0	0	2913	Mishelyfernandez	2005
14348	0	0	0	2913	Reesecalvo	2007
14349	0	0	0	2913	Kyrayip	2004
14350	0	0	0	2913	Tanatswatanyongana	2005
14351	0	0	0	2913	HARUNAhamada	2005
14352	0	0	0	2913	Katieoliver	2006
14353	0	0	0	2913	Mikaelahiu	2007
14354	0	0	0	2913	Kristinaradovic	2003
14355	0	0	0	2913	Andrijanalepojevic	2004
14356	0	0	0	2913	Hamsmetwali	2006
14357	0	0	0	2913	Nadaelhussein yousry	2004
14358	0	0	0	2913	Ameiasorey	2007
14359	0	0	0	2913	Julietterobinson	2003
14360	0	0	0	2913	Iman Syuhadaabdullah	2005
14361	0	0	0	2913	Nataliaturczuk	2004
14362	0	0	0	2913	Rositsadencheva	2007
14363	0	0	0	2913	Carolinraschdorf	2006
14364	0	0	0	2913	Joy Nicolelim	2005
14365	0	0	0	2913	Genevievedoe	2004
14366	0	0	0	2913	MARCELAtorres ceron	2002
14367	0	0	0	2913	Satiaubakirova	2006
14368	0	0	0	2913	Youmnafayssal	2006
14369	0	0	0	2913	Santamonransibrahmanakul	2005
14370	0	0	0	2913	Nawalyoussef	2006
14371	0	0	0	2913	Ananyamadduri	2005
14372	0	0	0	2913	Akihomaruyama	2004
14373	0	0	0	2913	Claraschoen	2006
14374	0	0	0	2913	Sabiramohamed	2003
14375	0	0	0	2913	Elleedryer	2004
14376	0	0	0	2913	Vittoriabaccino	2004
14377	0	0	0	2913	Aleksandrakushnareva	2005
14378	0	0	0	2913	Dariabibanina	2007
14379	0	0	0	2913	Sofiaberdecio flores	2005
14380	0	0	0	2913	Kaylentolliver	2003
14381	0	0	0	2913	Alyssacameron	2003
14382	0	0	0	2913	Jingjinghuang	2005
14383	0	0	0	2913	Shizukasakurada	2004
14384	0	0	0	2913	Wen Chunchung	2004
14385	0	0	0	2913	Ting Chihchang	2004
14386	0	0	0	2913	Aline Midorida silva	2004
14387	0	0	0	2913	Helin Alessandraalvarez prado	2005
14388	0	0	0	2913	Romicohen	2004
14389	0	0	0	2913	Milleechug	2006
14390	0	0	0	2913	Namratadas	2006
14391	0	0	0	2913	Ilinajha	2003
14392	0	0	0	2913	Virginiazanotti	2002
14393	0	0	0	2913	Ananyamanchanda	2004
14394	0	0	0	2913	Anjanimaheshkumar	2004
14395	0	0	0	2913	Marianaarguedas scobernatti	2003
14396	0	0	0	2913	Larissakastrati	2004

14397	0	0	0	2913	Ariannade bernardo	2004
14398	0	0	0	2913	Valentinanustes cadena	2003
14399	0	0	0	2913	Luizarodrigues daud	2002
14400	0	0	0	2913	Roisinmurphy	2003
14401	0	0	0	2913	Clionawalsh	2004
14402	0	0	0	2913	Mariahonore	2004
14403	0	0	0	2913	Valentinasuarez berrezueta	2005
14404	0	0	0	2913	Rhidaimyang lay	2003
14405	0	0	0	2913	MAHIpanchal	2003
14406	0	0	0	2913	muduozhou	2004
14407	0	0	0	2913	Isabelabraham	2004
14408	0	0	0	2913	Ireneverzin	2004
14409	0	0	0	2913	Daria Ioanapastoric	2004
14410	0	0	0	2913	Sophiasappa	2005
14411	0	0	0	2913	Sofiasokolova	2004
14412	0	0	0	2913	Miramanchanda	2002
14413	0	0	0	2913	Rotemsabag	2005
14414	0	0	0	2913	Pak Yingkok	2006
14415	0	0	0	2913	Mayashilo	2004
14416	0	0	0	2913	Noemibasiletti	2006
14417	0	0	0	2913	Camillefisette	2006
14418	0	0	0	2913	Anastasiarozhkova	2007
14419	0	0	0	2913	Eszterkis czako	2006
14420	0	0	0	2913	Kinaagraham	2005
14421	0	0	0	2913	Miruthula Mahalakshmikrishna	2004
14422	0	0	0	2913	Milica Vasilikimilosavljevic	2005
14423	0	0	0	2913	Kagisoledwaba	2004
14424	0	0	0	2913	Priyanshichauhan	2005
14425	0	0	0	2913	Luciakrivosudska	2003
14426	0	0	0	2913	Laracharles	2004
14427	0	0	0	2913	Martina Mariabovio	2005
14428	0	0	0	2913	Ana Sophiaariza corredor	2004
14429	0	0	0	2913	Alexiazarate valdez	2005
14430	0	0	0	2913	Tadisachamisa	2005
14431	0	0	0	2913	ASPASIAkatsouda taka	2002
14432	0	0	0	2913	Danielade haseth	2003
14433	0	0	0	2913	Minjichoi	2007
14434	0	0	0	2913	Marilouisevan zyl	2006
14435	0	0	0	2913	Isabellezumpft	2005
14436	0	0	0	2913	thi thanh loannguyen	2004
14437	0	0	0	2913	Ichinohorikawa	2007
14438	0	0	0	2913	Freya Athallahzayyan	2006
14439	0	0	0	2913	Sarahjenoudet	2004
14440	0	0	0	2913	Tengfei	2004
14441	0	0	0	2913	Chofifahparaswati	2003
14442	0	0	0	2913	Alessia Karolterlizzi	2003
14443	0	0	0	2913	Isabellezhu	2005
14444	0	0	0	2913	Hannahnagpal	2005
14445	0	0	0	2913	Brashadyer	2006
14446	0	0	0	2913	DANIELArodriguez	2006

14447	0	0	0	2913	MERIEMmakhlouf	2007
14448	0	0	0	2913	Maria Joserodriguez chanto	2005
14449	0	0	0	2913	Kupakwashe Diliakanyore	2007
14450	0	0	0	2913	Chiedzanyamakura	2003
14451	0	0	0	2913	Joy ocholongwasunday samuel	2002
14452	0	0	0	2913	asaljavidan	2005
14453	0	0	0	2913	Naimaluthra	2007
14454	0	0	0	2913	Dibamoharer	2003
14455	0	0	0	2913	Ariliboshoff	2005
14456	0	0	0	2913	Meriemben ezzedine	2006
14457	0	0	0	2913	Keshetshmueli	2005
14458	0	0	0	2913	Victoriaaguirre	2003
14459	0	0	0	2913	Habibaelanany	2006
14460	0	0	0	2913	Sarrazghal	2005
14461	0	0	0	2913	IRANAzarrinkolah	2005
14462	0	0	0	2913	Tam Truc Thiho	2004
14463	0	0	0	2913	Maykayla Cramos	2004
14464	0	0	0	2913	Akua Akoma EJhansen	2007
14465	0	0	0	2913	Chizitemokonjo	2005
14466	0	0	0	2913	YUXINzhang	2004
14467	0	0	0	2913	Sarahlouati	2007
14468	0	0	0	2913	Marikashimohara	2004
14469	0	0	0	2913	Nagisatakahashi	2003
14470	0	0	0	2913	Veronikapodrez	2007
14471	0	0	0	2913	Julialares	2005
14472	0	0	0	2913	Krystallim	2007
14473	0	0	0	2913	Maria Josecallejas hernandez	2003
14474	0	0	0	2913	Diana Virginiasiblesz brandt	2005
14475	0	0	0	2913	antonellabenavides	2002
14476	0	0	0	2913	Yennlee	2006
14477	0	0	0	2913	Sepideharghavan	2003
14478	0	0	0	2913	Marian Camilaangel velasquez	2004
14479	0	0	0	2913	Khushalimodi	2005
14480	0	0	0	2913	Martinawenz castillo	2003
14481	0	0	0	2913	Lucianabraga	2003
14482	0	0	0	2913	Ximenaku	2004
14483	0	0	0	2913	Judithattias	2005
14484	0	0	0	2913	Riyamatharoo	2005
14485	0	0	0	2913	Aurianelunain	2005
14486	0	0	0	2913	Anastasiakouchnareva	2003
14487	0	0	0	2913	Heshani Imangahewa thalagaha	2005
14488	0	0	0	2913	Yuen Tunglam	2005
14489	0	0	0	2913	Lauraberesova	2002
14490	0	0	0	2913	Vitoriasolis urdiales	2004
14491	0	0	0	2913	Kenzakamri	2005
14492	0	0	0	2913	Yu Yangjin	2003
14493	0	0	0	2913	Nadia Machtyngierpaz	2003
14494	0	0	0	2913	Leticiamatuszewski	2005
14495	0	0	0	2913	Doroteamilosavljevic	2004
14496	0	0	0	2913	Elizabethshaykis	2005

14497	0	0	0	2913	Weronikadomanska	2005
14498	0	0	0	2913	Giuliadal pozzo	2005
14499	0	0	0	2913	Liepasataite	2006
14500	0	0	0	2913	Aliesiareva	2006
14501	0	0	0	2913	Rosaliemolbaleh	2002
14502	0	0	0	2913	Cherineamiet	2004
14503	0	0	0	2913	Samrahamidovic	2004
14504	0	0	0	2913	Martakurmaz	2006
14505	0	0	0	2913	Dariaknysh	2004
14506	0	0	0	2913	Vitoriade brito francisco	2002
14507	0	0	0	2913	Fionapepper	2002
14508	0	0	0	2913	Sheilahemerenciana	2004
14509	0	0	0	2913	Sumejjaisic	2003
14510	0	0	0	2913	Ulyanaromanova	2005
14511	0	0	0	2913	Anshuokamura	2002
14512	0	0	0	2913	Laurenfullerton	2003
14513	0	0	0	2913	Orianaparkins-godwin	2003
14514	0	0	0	2913	Yubeanlee	2002
14515	0	0	0	2913	Yeon Soopark	2003
14516	0	0	0	2913	Mehrsachavoushi hosseini	2004
14517	0	0	0	2913	Polabatkiewicz	2003
14518	0	0	0	2913	Parmidahajjar	2003
14519	0	0	0	2913	Paularodriguez gregoris	2004
14520	0	0	0	2913	Yaimaperez wilson	2005
14521	0	0	0	2913	Alexandrajansson	2002
14522	0	0	0	2913	Zoecvitanovic	2004
14523	0	0	0	2913	Minnemardrosians	2003
14524	0	0	0	2913	Serenahatakka	2005
14525	0	0	0	2913	Tabathasuarez	2005
14526	0	0	0	2913	Karlagrobbelaar	2002
14527	0	0	0	2913	Agamhilel	2003
14528	0	0	0	2913	Daniellashved	2003
14529	0	0	0	2913	Victoria Mariamandma	2005
14530	0	0	0	2913	Melissalandres	2002
14531	0	0	0	2913	Mayelabassega	2004
14532	0	0	0	2913	Karinkratochvilova	2004
14533	0	0	0	2913	Reshma V S Schallarapu	2002
14534	0	0	0	2913	Maria Gaiameneguzzo	2005
14535	0	0	0	2913	Izem Elifolcer	2005
14536	0	0	0	2913	Lizidolidze	2005
14537	0	0	0	2913	Aleksandraaverianova	2005
14538	0	0	0	2913	Anamariakurtsikashvili	2005
14539	0	0	0	2913	Andreea Stefaniadinca	2004
14540	0	0	0	2913	Ruyamavi	2004
14541	0	0	0	2913	Neleniermann	2005
14542	0	0	0	2913	Cezarapencea	2004
14543	0	0	0	2913	Daryadarashkevich	2004
14544	0	0	0	2913	Carlalazzaroni	2002
14545	0	0	0	2913	Anastasiapesic	2002
14546	0	0	0	2913	Alessandraanghel	2002

14547	0	0	0	2913	Polinadrobysheva	2004
14548	0	0	0	2913	Micheleadamczewska	2002
14549	0	0	0	2913	Anna Charlotteo farrell	2002
14550	0	0	0	2913	Angelinasubotic	2003
14551	0	0	0	2913	Kimhansen	2002
14552	0	0	0	2913	Doheeyim	2002
14553	0	0	0	2913	Mariapenalver aguilo	2002
14554	0	0	0	2913	Ivadivac	2003
14555	0	0	0	2913	Valentinashleyn	2002
14556	0	0	0	2913	Stellawiesemann	2002
14557	0	0	0	2913	Inez Mariakotlikova	2003
14558	0	0	0	2913	Leamouton	2004
14559	0	0	0	2913	Adithivenkatakrishnan	2003
14560	0	0	0	2913	Matildecoen	2004
14561	0	0	0	2913	Rachaelphelan	2004
14562	0	0	0	2913	Charlottemalmio	2003
14563	0	0	0	2913	Evelinakivite	2004
14564	0	0	0	2913	Justynajodlowska	2004
14565	0	0	0	2913	Ayriskilic	2004
14566	0	0	0	2913	Aleina Elizabethalex	2002
14567	0	0	0	2913	Anizeikidze	2003
14568	0	0	0	2913	Madalenatanqueiro	2002
14569	0	0	0	2913	Ekaterinapavlova	2003
14570	0	0	0	2913	Constancaazinhaga	2004
14571	0	0	0	2913	Minseokim	2002
14572	0	0	0	2913	Su Jinkim	2003
14573	0	0	0	2913	Michellewetherall	2004
14574	0	0	0	2913	Ilariagrudino	2004
14575	0	0	0	2913	Milanakulayeva	2004
14576	0	0	0	2913	Oshiniherath	2003
14577	0	0	0	2913	Andreaperez bordon	2004
14578	0	0	0	3146	Alicevolpe	2002
14579	0	0	0	3146	Antonialawson	2002
14580	0	0	0	3146	Magdalenapaszkowiak	2002
14581	0	0	0	3146	Arinadobrygina	2003
14582	0	0	0	3146	Mariaandre	2003
14583	0	0	0	3146	Mayakeidar	2002
14584	0	0	0	3146	Majaradisic	2003
14585	0	0	0	3146	Pattarawadeeatidphokin	2002
14586	0	0	0	3146	Narinesargsyan	2002
14587	0	0	0	3146	Elizavetagnilozubova	2004
14588	0	0	0	3146	Nikolinegullacksen	2002
14589	0	0	0	3146	Paolapanova	2002
14590	0	0	0	3146	Sanyasingh	2002
14591	0	0	0	3146	Emmarenard	2002
14592	0	0	0	3146	Jessica Luisaalsola	2004
14593	0	0	0	3146	Ela Denizalpgiray	2003
14594	0	0	0	3146	Yee Chingwai	2004
14595	0	0	0	3146	Annaborovinskaya	2003
14596	0	0	0	3146	Runasaito	2002

14597	0	0	0	3146	Gavriellasmith	2003
14598	0	0	0	3146	Luciezasterova	2005
14599	0	0	0	3146	Eloisede mooij	2003
14600	0	0	0	3146	Avantikasivanand	2004
14601	0	0	0	3146	Lucieivanova	2004
14602	0	0	0	3146	Klarakajabova	2004
14603	0	0	0	3146	Angelamarnika	2004
14604	0	0	0	3146	Nadezhdapetkova	2004
14605	0	0	0	3146	Noraparik	2005
14606	0	0	0	3146	Esmeraldabrunner	2005
14607	0	0	0	3146	Tanivlainic	2004
14608	0	0	0	3146	Terezazelinova	2002
14609	0	0	0	3146	Brynjohnson	2003
14610	0	0	0	3146	Sofijazarina	2006
14611	0	0	0	3146	Alexandraokorokova	2005
14612	0	0	0	3146	Angela Mariabasto parra	2003
14613	0	0	0	3146	Elizavetashkryleva	2002
14614	0	0	0	3146	Claudiamiller	2002
14615	0	0	0	3146	Madisonschwarz	2004
14616	0	0	0	3146	Celinanoltenius	2004
14617	0	0	0	3146	Ruiruizou	2006
14618	0	0	0	3146	Serenagar	2003
14619	0	0	0	3146	Raquel Alejandrasantos marmo	2005
14620	0	0	0	3146	DIANA GABRIELAcraciun	2006
14621	0	0	0	3146	Tonirachal	2005
14622	0	0	0	3146	Tzu-Yingtseng	2006
14623	0	0	0	3146	Lenapeyer	2004
14624	0	0	0	3146	Sofievan reek	2004
14625	0	0	0	3146	Richardlynefrancois	2005
14626	0	0	0	3146	Chloebrown	2002
14627	0	0	0	3146	Nguyen Phanyen tich	2003
14628	0	0	0	3146	Natalieblock	2004
14629	0	0	0	3146	Cenanliu	2005
14630	0	0	0	3146	Luizaestrada	2003
14631	0	0	0	3146	Emaharajda	2007
14632	0	0	0	3146	Ambermcginnis	2003
14633	0	0	0	3146	Learebreyend	2002
14634	0	0	0	3146	Maria Ceciliagomez yanes	2004
14635	0	0	0	3146	Malakwalaa mohamed	2004
14636	0	0	0	3146	Mariaaldea wesselly	2005
14637	0	0	0	3146	Thereseharboe holst	2005
14638	0	0	0	3146	Celinede baeremaeker	2004
14639	0	0	0	3146	Orianaizarra	2004
14640	0	0	0	3146	Fernandarain contreras	2004
14641	0	0	0	3146	Florajohnson	2007
14642	0	0	0	3146	Annanovratidou	2005
14643	0	0	0	3146	Hanaminisy	2003
14644	0	0	0	3146	Manuelasanchez cortes	2003
14645	0	0	0	3146	Elianahanna	2004
14646	0	0	0	3146	Jelsyvette Kaylyncroes	2003

14647	0	0	0	3146	Chu Niwang	2003
14648	0	0	0	3146	Meribeljarna	2005
14649	0	0	0	3146	Taimahassiba	2004
14650	0	0	0	3146	Malakahmed	2004
14651	0	0	0	3146	Loginkhairy	2004
14652	0	0	0	3146	Yahuihu	2006
14653	0	0	0	3221	Fatima Aliraja	2007
14654	0	0	0	3221	Kai Ying Ericalim	2006
14655	0	0	0	3221	Zarakhan	2007
14656	0	0	0	3221	Nehandathomias	2006
14657	0	0	0	3221	Gabrielladanielsson	2004
14658	0	0	0	3221	Hannatelzynska	2005
14659	0	0	0	3221	Yuinauchida	2003
14660	0	0	0	3221	Taematsumoto	2005
14661	0	0	0	3221	Paulinajurkowska	2005
14662	0	0	0	3221	Klaravaja	2006
14663	0	0	0	3221	Sanjadjurdjevic	2005
14664	0	0	0	3221	Charliecroteau	2003
14665	0	0	0	3221	Eun Bilee	2005
14666	0	0	0	3221	Safijanizamic	2002
14667	0	0	0	3221	Handeulkim	2002
14668	0	0	0	3221	Sachisharma	2003
14669	0	0	0	3221	Kylinsadler	2003
14670	0	0	0	3221	Tamilagadamauri	2005
14671	0	0	0	3221	Hong-Yuchen	2004
14672	0	0	0	3221	Khymberlymckenzie	2005
14673	0	0	0	3221	Nataliatrigosso	2006
14674	0	0	0	3221	Mizukiyamashita	2003
14675	0	0	0	3221	Vivien Noemifaludi	2004
14676	0	0	0	3221	Omoladeaderemi	2002
14677	0	0	0	3221	Martinabernal	2002
14678	0	0	0	3221	Monanarita	2006
14679	0	0	0	3221	Emiliacosatto corzo	2003
14680	0	0	0	3221	Marielafortuny	2005
14681	0	0	0	3221	Inesvandekerckhove	2007
14682	0	0	0	3221	Amishishukla	2005
14683	0	0	0	3221	Mianyiji	2006
14684	0	0	0	3221	Jesychea	2006
14685	0	0	0	3221	Ellajames	2005
14686	0	0	0	3221	Kayleighlim	2007
14687	0	0	0	3221	loganvoeks	2003
14688	0	0	0	3221	Shihotsujioka	2007
14689	0	0	0	3221	Sofiagomez guerrero	2002
14690	0	0	0	3221	Marianajimenez bustillo	2004
14691	0	0	0	3221	Nataliazamora caprirolo	2003
14692	0	0	0	3221	Maria Gabrielamejia	2005
14693	0	0	0	3221	Gretalangiu	2003
14694	0	0	0	3221	Ewapodulka	2003
14695	0	0	0	3221	Hoi Yiu Michellewong	2005
14696	0	0	0	3221	Aadainna	2004

14697	0	0	0	3221	Madisonmills	2002
14698	0	0	0	3221	Eevaristola	2003
14699	0	0	0	3221	Belendeus	2002
14700	0	0	0	3221	Heewonkil	2004
14701	0	0	0	3221	Emmabelluomini	2004
14702	0	0	0	3221	Subekshasuren raj	2003
14703	0	0	0	3221	Victoriakoylass	2002
14704	0	0	0	3221	Shatavisaghosh	2003
14705	0	0	0	3221	Madalinajosan	2004
14706	0	0	0	3221	Nicolemikulan	2002
14707	0	0	0	3221	Milanamaydanova	2002
14708	0	0	0	3221	Jekaterinasagejeva	2002
14709	0	0	0	3221	Anitaivanova	2002
14710	0	0	0	3221	Gabriellegeolier	2002
14711	0	0	0	3221	Mariabezmenova	2002
14712	0	0	0	3221	Melenebadenhorst	2003
14713	0	0	0	3221	Gloriacontrino	2003
14714	0	0	0	3221	Sedefbilgic	2004
14715	0	0	0	3221	Katerina Noruphansen	2002
14716	0	0	0	3221	Vanessatcaci	2003
14717	0	0	0	3221	Katerynayemshanova	2003
14718	0	0	0	3221	Mariasimonenko	2003
14719	0	0	0	3221	Khaliahalemai	2004
14720	0	0	0	3221	Iulia Andreeaionescu	2005
14721	0	0	0	3221	Marezunic	2003
14722	0	0	0	3221	Carinasommer	2003
14723	0	0	0	3221	Deepshikasriram	2003
14724	0	0	0	3221	Veronikakarimbayeva	2004
14725	0	0	0	3221	Teodorastefanovic	2003
14726	0	0	0	3221	Anamarijakovacevic	2003
14727	0	0	0	3221	Sarayigin	2002
14728	0	0	0	3221	Katrinstefanova	2002
14729	0	0	0	3297	Yasminimamniyazova	2004
14730	0	0	0	3297	Maria Teresafrias serrano	2002
14731	0	0	0	3297	Yunawatanabe	2003
14732	0	0	0	3297	Michellekorpys	2004
14733	0	0	0	3297	Oana Biancamarinescu	2004
14734	0	0	0	3297	Coralrodriguez	2002
14735	0	0	0	3297	Harivarshinemurugesan	2002
14736	0	0	0	3297	Ylva Frafjordlanda	2002
14737	0	0	0	3297	Madelinefrediani	2002
14738	0	0	0	3297	Mathildesarcelet	2003
14739	0	0	0	3297	Veronicasirci	2004
14740	0	0	0	3297	Letiziamigani	2004
14741	0	0	0	3297	Babetteburgersdijk	2002
14742	0	0	0	3297	Laurapliksa	2003
14743	0	0	0	3297	Orlacooper	2003
14744	0	0	0	3297	Amanda Gabriellezoleta	2003
14745	0	0	0	3297	Anahitavatisyan	2004
14746	0	0	0	3297	Catherinehodge	2004

14747	0	0	0	3297	Hanasokolovic	2004
14748	0	0	0	3297	Rebeccasuarez	2004
14749	0	0	0	3297	Shambhavitiwari	2003
14750	0	0	0	3297	Mariiahurieva	2003
14751	0	0	0	3297	Kyliemerlevede	2004
14752	0	0	0	3297	Kyliemisdorp	2003
14753	0	0	0	3297	Ivaylagradinarova	2002
14754	0	0	0	3297	Angelinakister	2003
14755	0	0	0	3297	Anastasiazolotareva	2002
14756	0	0	0	3297	Agnesececcarelli	2002
14757	0	0	0	3297	Martagarcia reboredo montesd	2003
14758	0	0	0	3297	Alicebenvegnu	2003
14759	0	0	0	3297	Sandragines de la fuente	2002
14760	0	0	0	3297	Nicoleross	2003
14761	0	0	0	3297	Myrzaiymsherikulova	2002
14762	0	0	0	3297	Daimypaula	2002
14763	0	0	0	3297	Aishwaryakailaje	2003
14764	0	0	0	3297	Jeanette Angelahartono	2003
14765	0	0	0	3297	Maddalenagiordano	2003
14766	0	0	0	3297	Tharubphethomkrun	2002
14767	0	0	0	3297	Aleksandrabuslaeva	2002
14768	0	0	0	3297	Soniacassani	2002
14769	0	0	0	3297	Kashmiramanohar	2003
14770	0	0	0	3297	Rara Sima Kusumawardani	2003
14771	0	0	0	3297	Maeva Samranroguet	2005
14772	0	0	0	3297	Tenarajki	2005
14773	0	0	0	3297	Emmarutherford	2003
14774	0	0	0	3297	Ka Wengkuong	2003
14775	0	0	0	3297	Alexandra Ioanamoldovan	2003
14776	0	0	0	3297	Rose Marienijkamp	2006
14777	0	0	0	3297	Olgasorochkina	2004
14778	0	0	0	3297	Likabolkvadze	2005
14779	0	0	0	3297	Mathildegilles	2004
14780	0	0	0	3297	Ernabrdarevic	2003
14781	0	0	0	3297	Anastasiarachkovskaya	2005
14782	0	0	0	3297	Athenakomodromou	2004
14783	0	0	0	3297	Simaheren	2002
14784	0	0	0	3297	Min Kyojung	2004
14785	0	0	0	3297	Dominiquejansen van vuuren	2003
14786	0	0	0	3297	Annamcbride	2003
14787	0	0	0	3297	Raniatajina	2005
14788	0	0	0	3297	Isil Gulferazkara	2002
14789	0	0	0	3297	Wing Man Jennyleung	2005
14790	0	0	0	3297	Kasandra Leonidjekic	2004
14791	0	0	0	3297	Raissapetrovskaya	2005
14792	0	0	0	3297	Valentinaefremova	2004
14793	0	0	0	3297	Millafalster	2003
14794	0	0	0	3297	Alexavolkov	2005
14795	0	0	0	3297	Elasekmen	2006
14796	0	0	0	3297	Yumakomatsu	2004

14797	0	0	0	3297	Aayanaevanson	2002
14798	0	0	0	3297	Magdawawrowska	2002
14799	0	0	0	3297	Menuhapopovici	2004
14800	0	0	0	3297	Elisgergel	2005
14801	0	0	0	3297	Isha Shreeshah	2005
14802	0	0	0	3297	Charlottecatherwood	2002
14803	0	0	0	3297	Daniela Tarakotara	2005
14804	0	0	0	3297	Marielapluutus	2004
14805	0	0	0	3297	Yuzumiyoshi	2004
14806	0	0	0	3297	Masamitic	2004
14807	0	0	0	3297	Annagiuliadanesi	2003
14808	0	0	0	3297	Maria Ioanasecara	2005
14809	0	0	0	3297	Nataliazalewska	2004
14810	0	0	0	3297	Annadomingo garcia	2002
14811	0	0	0	3297	Amiraellouzi	2004
14812	0	0	0	3297	Jelenagojkovic	2005
14813	0	0	0	3297	Mishrageetam	2003
14814	0	0	0	3297	Dariagiansante	2003
14815	0	0	0	3297	Snigdhaallada	2003
14816	0	0	0	3297	Jillschalk	2004
14817	0	0	0	3297	Mariaivankovic	2006
14818	0	0	0	3297	Sahbavalaei	2005
14819	0	0	0	3297	Yael Azulbarrios	2003
14820	0	0	0	3297	Yazmin Abrilbarrios	2003
14821	0	0	0	3297	Martinnaroldan cardenas	2003
14822	0	0	0	3297	Giaposito	2004
14823	0	0	0	3297	Melissatamariz zedeno	2002
14824	0	0	0	3297	Sofiavillarroel llobet	2003
14825	0	0	0	3297	Valeriasan miguel soria	2004
14826	0	0	0	3297	Belisavilla torres	2004
14827	0	0	0	3297	Alicebarmashyna	2003
14828	0	0	0	3297	Anastasiiapriadkina	2003
14829	0	0	0	3297	Jaidesmith	2003
14830	0	0	0	3297	Lauraalmagro	2004
14831	0	0	0	3297	Delfinayanez	2003
14832	0	0	0	3297	Katiecodd	2004
14833	0	0	0	3297	Nishka Eshwaryvivekanandan	2006
14834	0	0	0	3297	Abigailelham	2007
14835	0	0	0	3297	Valeriamontano	2002
14836	0	0	0	3297	Renadayman maamoun	2005
14837	0	0	0	3297	Perihanauf	2003
14838	0	0	0	3297	Rosearnastairmand	2005
14839	0	0	0	3297	Ekaterinalazareva	2005
14840	0	0	0	3297	Aminataibi garcia	2004
14841	0	0	0	3297	Paigegrice	2003
14842	0	0	0	3297	Ellao'malley	2003
14843	0	0	0	3297	Sophiacisse-ignatiev	2005
14844	0	0	0	3297	Alexamartinez mendoza	2005
14845	0	0	0	3297	Anaalvarez storni	2002
14846	0	0	0	3297	Pamelabadillo hernandez	2006

14847	0	0	0	3297	Faridamoussa	2006
14848	0	0	0	3297	Ziyigao	2005
14849	0	0	0	3297	Warapornmuangpho	2005
14850	0	0	0	3297	Artinagashi	2007
14851	0	0	0	3297	Hailey Robyntroup	2005
14852	0	0	0	3297	Justinapenna	2003
14853	0	0	0	3297	Knoozfouad	2005
14854	0	0	0	3297	Nicoleobando marin	2004
14855	0	0	0	3297	JADEali	2004
14856	0	0	0	3297	Adaliabadroe	2004
14857	0	0	0	3297	Ariadnazegarra	2004
14858	0	0	0	3297	Shaloomdelgado forero	2004
14859	0	0	0	3297	Mariameltaweel	2006
14860	0	0	0	3297	Georgiaplunkett	2004
14861	0	0	0	3297	Siya Ushakakkar	2004
14862	0	0	0	3297	Sedraelkhurashy	2005
14863	0	0	0	3297	Natsukiyamagami	2007
14864	0	0	0	3297	Noralouko	2006
14865	0	0	0	3297	Aichamegdiche	2006
14866	0	0	0	3297	Yumitoulegenova	2003
14867	0	0	0	3297	Fatmajammoussi	2005
14868	0	0	0	3297	Mayamoussa	2005
14869	0	0	0	3297	Roualimem	2005
14870	0	0	0	3297	Nadiarodewald	2006
14871	0	0	0	3297	Hannahsutherland-smith	2002
14872	0	0	0	3297	Linda Fernandamarino	2005
14873	0	0	0	3297	Mariapazospina	2006
14874	0	0	0	3297	Andreaelaez miranda	2004
14875	0	0	0	3297	Giorgiaroselli	2004
14876	0	0	0	3297	Jieonoh	2005
14877	0	0	0	3297	Isabellasaul	2003
14878	0	0	0	3297	Mariiapetrova	2005
14879	0	0	0	3297	Sarahben aba	2007
14880	0	0	0	3297	Annasydorenko	2003
14881	0	0	0	3297	Sofia Isabelancieta cortez	2004
14882	0	0	0	3297	Ariana Fernandarodriguez	2005
14883	0	0	0	3297	Natalia Gabrielanavarro ramire	2003
14884	0	0	0	3297	Ju-Yunjeng	2004
14885	0	0	0	3297	Ana Mihaelagavrilescu	2004
14886	0	0	0	3297	Marineboutros	2006
14887	0	0	0	3297	Shreya Sudheerabhat	2004
14888	0	0	0	3297	Ketingchen	2007
14889	0	0	0	3297	Nicolematukutire	2005
14890	0	0	0	3297	Kannchayachungwatana	2007
14891	0	0	0	3297	Inbarpony	2005
14892	0	0	0	3297	Munashebera	2005
14893	0	0	0	3297	Naomiwidiaman	2004
14894	0	0	0	3297	Rachel Meganpeters	2004
14895	0	0	0	3297	Juanitacarrascal mendez	2002
14896	0	0	0	3297	Es Megantay	2004

14897	0	0	0	3297	FABIOLA VALENTINAAbsereni rel	2003
14898	0	0	0	3297	Lok Yanwong	2006
14899	0	0	0	3297	Isabellapita	2002
14900	0	0	0	3297	Jessicachurch	2004
14901	0	0	0	3297	Kayladean	2003
14902	0	0	0	3297	Giulia Sophystefan	2003
14903	0	0	0	3297	Iremturemisoglu	2004
14904	0	0	0	3297	Natsukiirie	2004
14905	0	0	0	3297	Justineazarak quebengco	2003
14906	0	0	0	3297	Renatagregorutti	2002
14907	0	0	0	3297	Nataliyaslipchenko	2002
14908	0	0	0	3297	Alexandramartinakova	2003
14909	0	0	0	3297	Pragnyakumar	2003
14910	0	0	0	3297	Mandiportegys	2003
14911	0	0	0	3297	Prakritishrestha	2004
14912	0	0	0	3297	Dahamna Methsandilamappula	2005
14913	0	0	0	3297	Maria Victoriaromero scattone	2005
14914	0	0	0	3297	Barhaham	2002
14915	0	0	0	3297	Aisanakojonazarova	2006
14916	0	0	0	3297	Ylanduong	2004
14917	0	0	0	3297	Linaguendouz	2006
14918	0	0	0	3297	Arianakolbassina	2007
14919	0	0	0	3297	Angelaperez barro	2003
14920	0	0	0	3297	Anamariacoroiu	2005
14921	0	0	0	3297	Yukowadazumi	2004
14922	0	0	0	3297	Ozumkavran	2005
14923	0	0	0	3297	Elizavetapetrova	2002
14924	0	0	0	3297	Lisettealjas	2006
14925	0	0	0	3297	Kalennagai	2005
14926	0	0	0	3297	Pelincolak	2005
14927	0	0	0	3297	Gresibajri	2006
14928	0	0	0	3297	Despoinabaxopoulou	2004
14929	0	0	0	3297	Saibhoyar	2004
14930	0	0	0	3297	Valentinagomez	2002
14931	0	0	0	3297	Xinyi	2003
14932	0	0	0	3297	Alinacherniaieva	2005
14933	0	0	0	3297	Apsarachhieu	2004
14934	0	0	0	3297	Angelika Johannafenyves	2003
14935	0	0	0	3297	Eun Seokim	2005
14936	0	0	0	3297	Danijeladanilovic	2003
14937	0	0	0	3297	Hibamerida	2005
14938	0	0	0	3297	Nataliemasinda	2004
14939	0	0	0	3297	Sarahjemail	2007
14940	0	0	0	3297	Tanakagarikai	2006
14941	0	0	0	3297	Danielaborruel	2006
14942	0	0	0	3297	Preethikaachandramohan	2004
14943	0	0	0	3297	Malakrzanic-sullivan	2007
14944	0	0	0	3297	Annabelleloutit	2006
14945	0	0	0	3297	Janahossam salah	2007
14946	0	0	0	3297	Sabrinalin	2007

14947	0	0	0	3297	Khadijaben salem	2007
14948	1	1	1	1	Dianeparry	2002
14949	1	1	1	2	Dariasnigur	2002
14950	1	1	1	3	Emmanavarro	2001
14951	1	1	1	4	Alexanoel	2002
14952	1	1	1	5	Natsumikawaguchi	2002
14953	1	1	1	6	Elsajacquemot	2003
14954	1	1	1	7	Robinmontgomery	2004
14955	1	1	1	8	Kamillabartone	2002
14956	1	1	1	9	Alexandraeala	2005
14957	1	1	1	10	Lindafruhvirtova	2005
14958	1	1	1	11	Mariabondarenko	2003
14959	1	1	1	12	Alinacharaeva	2002
14960	0	1	1	13	Hurricane Tyrablack	2001
14961	0	1	1	14	Oksanaselekhmeteva	2003
14962	0	1	1	15	Alexandrayepifanova	2003
14963	0	1	1	16	Sadanahimana	2001
14964	0	1	1	17	Abigailforbes	2001
14965	0	1	1	18	Qinwenzheng	2002
14966	0	1	1	19	Melodiecollard	2003
14967	0	1	1	20	Selenajanicijevic	2002
14968	0	1	1	21	Polinakudermetova	2003
14969	0	1	1	22	Victoriajimenez kasintseva	2005
14970	0	1	1	23	Mai Napattnirundorn	2002
14971	0	1	1	24	Anemintegi del olmo	2003
14972	0	1	1	25	Zhuoxuanbai	2002
14973	0	1	1	26	Mell Elizabethreasco gonzalez	2002
14974	0	1	1	27	Priska Madelynnugroho	2003
14975	0	1	1	28	Hong Yi Codywong	2002
14976	0	0	1	29	Carolemonnet	2001
14977	0	0	1	30	Aubanedroguet	2002
14978	0	0	1	31	Savannahbroadus	2002
14979	0	0	1	32	Matildamutavdzic	2004
14980	0	0	1	33	Adriennnagy	2001
14981	0	0	1	34	Elinaavanesyan	2002
14982	0	0	1	35	Mariatimofeeva	2003
14983	0	0	1	36	Alexandravecic	2002
14984	0	0	1	37	Maraguth	2003
14985	0	0	1	38	Shavitkimchi	2002
14986	0	0	1	39	Katrinascott	2004
14987	0	0	1	40	Amarnibanks	2002
14988	0	0	1	41	Caijsa Wildahennemann	2001
14989	0	0	1	42	Pialovric	2002
14990	0	0	1	43	Giuliamorlet	2002
14991	0	0	1	44	Anastasiatikhonova	2001
14992	0	0	1	45	Yeonwooku	2003
14993	0	0	1	46	Dariafrayman	2002
14994	0	0	1	47	Sohyunpark	2002
14995	0	0	1	48	Weronikabaszak	2002
14996	0	0	1	49	Darjasemenistaja	2002

14997	0	0	1	50	Angelinawirges	2002
14998	0	0	1	51	Juliebelgraver	2002
14999	0	0	1	52	Helenepellicano	2002
15000	0	0	1	53	Zivafalkner	2002
15001	0	0	1	54	Dianashnaider	2004
15002	0	0	1	55	Liubovkostenko	2003
15003	0	0	1	56	Guillerminagrant	2002
15004	0	0	0	57	Martacustic	2002
15005	0	0	0	58	Anageller	2002
15006	0	0	0	59	Joannagarland	2001
15007	0	0	0	60	Elizabethcoleman	2003
15008	0	0	0	61	Celia Bellemohr	2002
15009	0	0	0	62	Charlottechavatipon	2002
15010	0	0	0	63	Annerlygeorgopoulos	2003
15011	0	0	0	64	Melaniadelai	2002
15012	0	0	0	65	Madisonsieg	2003
15013	0	0	0	66	Antoniasamudio	2001
15014	0	0	0	67	Elvinakalieva	2003
15015	0	0	0	68	Skyler Marie Gracegrishuk	2002
15016	0	0	0	69	Rominaccuno	2002
15017	0	0	0	70	Chloebeck	2001
15018	0	0	0	71	Charlotteowensby	2003
15019	0	0	0	72	Romanacisovska	2002
15020	0	0	0	73	Emmajackson	2002
15021	0	0	0	74	Saraziodato	2002
15022	0	0	0	75	Sakiimamura	2002
15023	0	0	0	76	Mylenehalemai	2001
15024	0	0	0	77	Janicetjen	2002
15025	0	0	0	78	Ana Luizacruz	2001
15026	0	0	0	79	Kaileyevans	2003
15027	0	0	0	80	Michaelakadleckova	2001
15028	0	0	0	81	Jiaqiwang	2002
15029	0	0	0	82	Federicarossi	2001
15030	0	0	0	83	Hibahshaikh	2002
15031	0	0	0	84	Taramalik	2002
15032	0	0	0	85	Martynakubka	2001
15033	0	0	0	86	Laurenanzalotta kynoch	2002
15034	0	0	0	87	Carlotamartinez cirez	2001
15035	0	0	0	88	Lisapigato	2003
15036	0	0	0	89	Valentinaryser	2001
15037	0	0	0	90	Carol Young Suhlee	2001
15038	0	0	0	91	Lucienguyen tan	2003
15039	0	0	0	92	Danaguzman	2003
15040	0	0	0	93	Tina Nadinesmith	2002
15041	0	0	0	94	BoYoungjeong	2003
15042	0	0	0	95	Mananchayasawangkaew	2002
15043	0	0	0	96	Noakrznaric uygungul	2003
15044	0	0	0	97	Kirapavlova	2004
15045	0	0	0	98	Viktoriyapetrenko	2001
15046	0	0	0	99	Danielladimitrova	2002

15047	0	0	0	100	Erikamatsuda	2003
15048	0	0	0	101	Leyreromero gormaz	2002
15049	0	0	0	102	Mariiatkacheva	2001
15050	0	0	0	103	Veronikapepelyaeva	2001
15051	0	0	0	104	Kyliecollins	2002
15052	0	0	0	105	Funakozaki	2001
15053	0	0	0	106	Thasapornnaklo	2001
15054	0	0	0	107	Hinainoue	2003
15055	0	0	0	108	Zoziyakardava	2001
15056	0	0	0	109	Dakotafordham	2002
15057	0	0	0	110	Rebeccalynn	2004
15058	0	0	0	111	Meganbasson	2002
15059	1	1	1	1	Claraburel	2001
15060	1	1	1	2	Cocogauff	2004
15061	1	1	1	3	Xiyuwang	2001
15062	1	1	1	4	Claratauson	2002
15063	1	1	1	5	Camilaosorio	2001
15064	1	1	1	6	En Shuoliang	2000
15065	1	1	1	7	Xinyuwang	2001
15066	1	1	1	8	Catymcnally	2001
15067	1	1	1	9	Qinwenzheng	2002
15068	0	1	1	10	Eleonoramolinaro	2000
15069	0	1	1	11	Yukinaito	2001
15070	0	1	1	12	Dianeparry	2002
15071	0	1	1	13	Leylahfernandez	2002
15072	0	1	1	14	Nahosato	2001
15073	0	1	1	15	Elisabettacocciaretto	2001
15074	0	1	1	16	Alexanoel	2002
15075	0	1	1	17	Hurricane Tyrablack	2001
15076	0	1	1	18	Elizabethmandlik	2001
15077	0	1	1	19	Viktoriiadema	2000
15078	0	1	1	20	Mananchayasawangkaew	2002
15079	0	1	1	21	Leama	2001
15080	0	1	1	22	Emmaraducanu	2002
15081	0	1	1	23	Lulusun	2001
15082	0	0	1	24	Sohyunpark	2002
15083	0	0	1	25	Kamillabartone	2002
15084	0	0	1	26	Joannagarland	2001
15085	0	0	1	27	Hong Yi Codywong	2002
15086	0	0	1	28	Adriennnagy	2001
15087	0	0	1	29	Oksanaselekhmeteva	2003
15088	0	0	1	30	Katievolynets	2001
15089	0	0	1	31	Thasapornnaklo	2001
15090	0	0	1	32	Sadanahimana	2001
15091	0	0	1	33	Martacustic	2002
15092	0	0	1	34	Loudmillabencheikh	2001
15093	0	0	1	35	Kamillarakhimova	2001
15094	0	0	1	36	Georgiadrummy	2000
15095	0	0	1	37	Anastasiatikhonova	2001
15096	0	0	1	38	Gabriellaprice	2003

15097	0	0	1	39	Margarytabilokin	2001
15098	0	0	1	40	Dariasnigur	2002
15099	0	0	1	41	Natsumikawaguchi	2002
15100	0	0	1	42	Viktoryiakanapatskaya	2002
15101	0	0	1	43	Lenkastara	2000
15102	0	0	1	44	Maria Lourdescarle	2000
15103	0	0	1	45	Anageller	2002
15104	0	0	0	46	Elinaavanesyan	2002
15105	0	0	0	47	Caijsa Wildahennemann	2001
15106	0	0	0	48	Selinovunc	2001
15107	0	0	0	49	Mylenehalemai	2001
15108	0	0	0	50	Kacieharvey	2001
15109	0	0	0	51	Francescacurmi	2002
15110	0	0	0	52	Chloebeck	2001
15111	0	0	0	53	Stefaniarogozinska dzik	2001
15112	0	0	0	54	Dariafrayman	2002
15113	0	0	0	55	Savannahbroadus	2002
15114	0	0	0	56	Dalaynahewitt	2000
15115	0	0	0	57	Yasminemansouri	2001
15116	0	0	0	58	Maria Gabrielarivera corado	2001
15117	0	0	0	59	Danielladimitrova	2002
15118	0	0	0	60	Manakawamura	2001
15119	0	0	0	61	Anamakatsaria	2000
15120	0	0	0	62	Manonleonard	2001
15121	0	0	0	63	Giuliamorlet	2002
15122	0	0	0	64	Danielavismane	2000
15123	0	0	0	65	Himarisato	2002
15124	0	0	0	66	Violetapisah	2000
15125	0	0	0	67	Martinabiagianti	2001
15126	0	0	0	68	Annahertel	2000
15127	0	0	0	69	Alexandravagramov	2001
15128	0	0	0	70	Joannezuger	2000
15129	0	0	0	71	Federicarossi	2001
15130	0	0	0	72	Federicasacco	2002
15131	0	0	0	73	Peytonstearns	2001
15132	0	0	0	74	Rutgalindo	2001
15133	0	0	0	75	Robinmontgomery	2004
15134	0	0	0	76	Gerganatopalova	2000
15135	0	0	0	77	Elsajacquemot	2003
15136	0	0	0	78	Andreeaprisacariu	2000
15137	0	0	0	79	Anrinagata	2000
15138	0	0	0	80	Laura Sofiarico garcia	2000
15139	0	0	0	81	Natashasubhash	2001
15140	0	0	0	82	Mariamdalakishvili	2001
15141	0	0	0	83	Valentinaivanov	2001
15142	0	0	0	84	Charlotteowensby	2003
15143	0	0	0	85	Carol Young Suhlee	2001
15144	0	0	0	86	Zivafalkner	2002
15145	0	0	0	87	Hannahviller moller	2001
15146	0	0	0	88	Viktoriamorvayova	2001

15147	0	0	0	89	Alexandrayepifanova	2003
15148	0	0	0	90	Nikaradisic	2000
15149	1	1	1	1	Whitneyosuigwe	2002
15150	1	1	1	2	Martakostyuk	2002
15151	1	1	1	3	Elenarybakina	1999
15152	1	1	1	4	Xinyuwang	2001
15153	1	1	1	5	Olgadanilovic	2001
15154	1	1	1	6	En Shuoliang	2000
15155	1	1	1	7	Emilianaarango	2000
15156	1	1	1	8	Camilaosorio	2001
15157	1	1	1	9	Simonawaltert	2000
15158	0	1	1	10	Carsonbranstine	2000
15159	0	1	1	11	Maria Lourdescarle	2000
15160	0	1	1	12	Annli	2000
15161	0	1	1	13	Emilyappleton	1999
15162	0	1	1	14	Sofyalansere	2000
15163	0	1	1	15	Nahosato	2001
15164	0	1	1	16	Kajajuvan	2000
15165	0	1	1	17	Joannagarland	2001
15166	0	1	1	18	Anastasiakharitonova	2000
15167	0	1	1	19	Alexanoel	2002
15168	0	1	1	20	Sofiasewing	1999
15169	0	1	1	21	Xiyuwang	2001
15170	0	1	1	22	Lulusun	2001
15171	0	1	1	23	Kamillarakhimova	2001
15172	0	0	1	24	Yukinaito	2001
15173	0	0	1	25	Elysiabolton	2000
15174	0	0	1	26	Danielavismane	2000
15175	0	0	1	27	Catymcnally	2001
15176	0	0	1	28	Yasminemansouri	2001
15177	0	0	1	29	Natashasubhash	2001
15178	0	0	1	30	Juleniemeier	1999
15179	0	0	1	31	Alinacharaeva	2002
15180	0	0	1	32	Ylenain-albon	1999
15181	0	0	1	33	Zeeldesai	1999
15182	0	0	1	34	Taylorjohnson	2000
15183	0	0	1	35	Hurricane Tyrablack	2001
15184	0	0	1	36	Violetapisah	2000
15185	0	0	1	37	Laynesleeth	2001
15186	0	0	1	38	Victoriaflores	1999
15187	0	0	1	39	Sadanahimana	2001
15188	0	0	1	40	Haileybaptiste	2001
15189	0	0	1	41	Viktoriiadema	2000
15190	0	0	1	42	Mahakjain	2001
15191	0	0	1	43	Oonaorpana	2001
15192	0	0	1	44	Mihikayadav	1999
15193	0	0	1	45	Anhzelikaisaeva	2000
15194	0	0	0	46	Draginjavukovic	2000
15195	0	0	0	47	Annahertel	2000
15196	0	0	0	48	Tatianapieri	1999

15197	0	0	0	49	Himarisato	2002
15198	0	0	0	50	Elliedouglas	2000
15199	0	0	0	51	Viktoriamorvayova	2001
15200	0	0	0	52	Pei Hsuanchen	2000
15201	0	0	0	53	Paulaarias manjon	2000
15202	0	0	0	54	Diaeel jardi	2000
15203	0	0	0	55	Peytonstearns	2001
15204	0	0	0	56	Leaboskovic	1999
15205	0	0	0	57	Nicolemossmer	2000
15206	0	0	0	58	Thasapornnaklo	2001
15207	0	0	0	59	Alicollins	2000
15208	0	0	0	60	Dalaynahewitt	2000
15209	0	0	0	61	Fernandalabrana	1999
15210	0	0	0	62	Mihaela Lorenamarculescu	2000
15211	0	0	0	63	Maihontama	1999
15212	0	0	0	64	Thaisa Granapedretti	1999
15213	0	0	0	65	Denisahindova	2002
15214	0	0	0	66	Vanessaong	2002
15215	0	0	0	67	Ecaterinavisnevscaia	1999
15216	0	0	0	68	Elizabethmandlik	2001
15217	0	0	0	69	Katievolynets	2001
15218	0	0	0	70	Sofiamunera sanchez	1999
15219	0	0	0	71	Leama	2001
15220	0	0	0	72	Anastasiaiamachkine	2000
15221	0	0	0	73	I-Hsuancho	2000
15222	0	0	0	74	Anrinagata	2000
15223	0	0	0	75	Sinazuger	1999
15224	0	0	0	76	Ayumimiyamoto	2000
15225	0	0	0	77	Moyukauchijima	2001
15226	0	0	0	78	Malenehelgo	1999
15227	0	0	0	79	Majachwalinska	2001
15228	0	0	0	80	Barboramatusova	1999
15229	0	0	0	81	Tiffanylagarde	2000
15230	0	0	0	82	Funakozaki	2001
15231	0	0	0	83	Katyatownsend	2000
15232	0	0	0	84	Lexiestevens	1999
15233	0	0	0	85	Astrid Wanjabrune olsen	1999
15234	0	0	0	86	Maria Joseportillo ramirez	1999
15235	0	0	0	87	Victoriahu	2002
15236	0	0	0	88	Vitoriaokuyama	1999
15237	0	0	0	89	Camilletownsend	2000
15238	0	0	0	90	Yanglee	2000
15239	1	1	1	1	Anastasiapotapova	2001
15240	1	1	1	2	Olesyapervushina	2000
15241	1	1	1	3	Kayladay	1999
15242	1	1	1	4	Rebekamasarova	1999
15243	1	1	1	5	Amandaanisimova	2001
15244	1	1	1	6	Kajajuvan	2000
15245	1	1	1	7	Aminaanshba	1999
15246	1	1	1	8	Dayanayastremska	2000

15247	1	1	1	9	Claireliu	2000
15248	0	1	1	10	Taylorjohnson	2000
15249	0	1	1	11	Xiyuwang	2001
15250	0	1	1	12	Usue Maitanearconada	1998
15251	0	1	1	13	Olgadanilovic	2001
15252	0	1	1	14	Igaswiatek	2001
15253	0	1	1	15	Jodie Annaburrage	1999
15254	0	1	1	16	Pannaudvardy	1998
15255	0	1	1	17	Katarinazavatska	2000
15256	0	1	1	18	Elliedouglas	2000
15257	0	1	1	19	Ayumimiyamoto	2000
15258	0	1	1	20	Emilyappleton	1999
15259	0	1	1	21	Varvaragracheva	2000
15260	0	1	1	22	Maihontama	1999
15261	0	0	1	23	Catymcnally	2001
15262	0	0	1	24	Maria Lourdescarle	2000
15263	0	0	1	25	Carsonbranstine	2000
15264	0	0	1	26	Yukinaito	2001
15265	0	0	1	27	Evaguerrero alvarez	1999
15266	0	0	1	28	Ioanaminca	1998
15267	0	0	1	29	Dominiqueschaefer	1999
15268	0	0	1	30	Natashasubhash	2001
15269	0	0	1	31	Malenehelgo	1999
15270	0	0	1	32	Paulaarias manjon	2000
15271	0	0	1	33	Tatianapieri	1999
15272	0	0	1	34	Biancaandreescu	2000
15273	0	0	1	35	Zhimadu	1999
15274	0	0	1	36	Laraescauriza	1998
15275	0	0	1	37	Elenarybakina	1999
15276	0	0	1	38	Georgia Andreeacraciun	1999
15277	0	0	1	39	Leaboskovic	1999
15278	0	0	1	40	I-Hsuancho	2000
15279	0	0	1	41	Monikakilnarova	1999
15280	0	0	1	42	Ylenain-albon	1999
15281	0	0	1	43	Baijinglin	2000
15282	0	0	1	44	Miraantonitsch	1998
15283	0	0	0	45	Federicabilardo	1999
15284	0	0	0	46	Irinacantos siemers	2000
15285	0	0	0	47	Melany Solangekrywoj	1998
15286	0	0	0	48	Maria Joseportillo ramirez	1999
15287	0	0	0	49	Luciekankova	2000
15288	0	0	0	50	Alexandrasanford	1999
15289	0	0	0	51	Mirjambjorklund	1998
15290	0	0	0	52	Morgancoppoc	1999
15291	0	0	0	53	Larasalden	1999
15292	0	0	0	54	Emilianaarango	2000
15293	0	0	0	55	Liudmilasamsonova	1998
15294	0	0	0	56	Mariamateas	1999
15295	0	0	0	57	Shellykrolitzky	1999
15296	0	0	0	58	Lucreziastefanini	1998

15297	0	0	0	59	Fernandalabrana	1999
15298	0	0	0	60	Hurricane Tyrablack	2001
15299	0	0	0	61	Chihiromuramatsu	1998
15300	0	0	0	62	Nicolemossmer	2000
15301	0	0	0	63	Shuyuema	1999
15302	0	0	0	64	Whitneyosuigwe	2002
15303	0	0	0	65	Haileybaptiste	2001
15304	0	0	0	66	Camilaromero	1998
15305	0	0	0	67	Sadanahimana	2001
15306	0	0	0	68	Charlotterobillard-millette	1999
15307	0	0	0	69	Annli	2000
15308	0	0	0	70	Selma Stefaniacadar	2000
15309	0	0	0	71	Oanagavrila	1998
15310	0	0	0	72	Elenichristofi	1998
15311	0	0	0	73	Siqicao	1999
15312	0	0	0	74	Mayukaaikawa	1998
15313	0	0	0	75	Abigaildesiatnikov	2001
15314	0	0	0	76	Jia Qiren	2000
15315	0	0	0	77	Anastasiazarycka	1998
15316	0	0	0	78	Anhzelikaisaeva	2000
15317	0	0	0	79	Wushuangzheng	1998
15318	0	0	0	80	Katieswan	1999
15319	0	0	0	81	Ninakruijer	1998
15320	0	0	0	82	Jimenarodriguez-benito	2000
15321	0	0	0	83	Jaimeefourlis	1999
15322	0	0	0	84	Yardenakler	1999
15323	0	0	0	85	Mariatyrina	1999
15324	0	0	0	86	Destaneeaiava	2000
15325	0	0	0	87	Berfucengiz	1999
15326	1	1	1	1	Dalmagalfi	1998
15327	1	1	1	2	Marketavondrousova	1999
15328	1	1	1	3	Katieswan	1999
15329	1	1	1	4	Biancaandreescu	2000
15330	1	1	1	5	Annablinkova	1998
15331	1	1	1	6	Terezamihalikova	1998
15332	1	1	1	7	Usue Maitanearconada	1998
15333	1	1	1	8	Sofiakenin	1998
15334	1	1	1	9	Charlotterobillard-millette	1999
15335	1	1	1	10	Kayladay	1999
15336	0	1	1	11	Miriamskoch	1997
15337	0	1	1	12	Annakalinskaya	1998
15338	0	1	1	13	Francescadi lorenzo	1997
15339	0	1	1	14	Evgeniyalevashova	1999
15340	0	1	1	15	Veralapko	1998
15341	0	1	1	16	Katharinahobgarski	1997
15342	0	1	1	17	Wushuangzheng	1998
15343	0	1	1	18	Julieta Laraestable	1997
15344	0	1	1	19	Pranjalayadlapalli	1999
15345	0	1	1	20	Sofyazhuk	1999
15346	0	1	1	21	Olesyapervushina	2000

15347	0	1	1	22	Anastasiapotapova	2001
15348	0	1	1	23	Luisastefani	1997
15349	0	0	1	24	Rebekamasarova	1999
15350	0	0	1	25	Aleksandrapospelova	1998
15351	0	0	1	26	Fannistollar	1998
15352	0	0	1	27	Michaelagordon	1999
15353	0	0	1	28	Claireliu	2000
15354	0	0	1	29	Viktoriahruncakova	1998
15355	0	0	1	30	Inesibbou	1999
15356	0	0	1	31	Alexandrasanford	1999
15357	0	0	1	32	Emilyarbuthnott	1997
15358	0	0	1	33	Chihiromuramatsu	1998
15359	0	0	1	34	Katherinesebov	1999
15360	0	0	1	35	Jaquelinecristian	1998
15361	0	0	1	36	Biancaturati	1997
15362	0	0	1	37	Ioanaminca	1998
15363	0	0	1	38	Mariamateas	1999
15364	0	0	1	39	Amandaanisimova	2001
15365	0	0	1	40	Tessahandrianjafitrimo	1998
15366	0	0	1	41	Beatricetorelli	1997
15367	0	0	1	42	Maria Fernandaherazo gonzale:	1997
15368	0	0	1	43	Maialumsden	1998
15369	0	0	1	44	Carolinedolehide	1998
15370	0	0	1	45	Jessicahinojosa gomez	1997
15371	0	0	1	46	Ioana Dianapietroiu	1998
15372	0	0	0	47	Thaisa Granapedretti	1999
15373	0	0	0	48	Mayukaaikawa	1998
15374	0	0	0	49	Oanagavrila	1998
15375	0	0	0	50	Jilteichmann	1997
15376	0	0	0	51	Emiliefrancati	1997
15377	0	0	0	52	Oliviatjandramulia	1997
15378	0	0	0	53	Jadelewis	1998
15379	0	0	0	54	Elenichristofi	1998
15380	0	0	0	55	Derianur haliza	1997
15381	0	0	0	56	Pannaudvardy	1998
15382	0	0	0	57	Mihikayadav	1999
15383	0	0	0	58	Mancapislak	1997
15384	0	0	0	59	Destaneeaiava	2000
15385	0	0	0	60	Yueyuan	1998
15386	0	0	0	61	Sofiasewing	1999
15387	0	0	0	62	Yulianamonroy	1998
15388	0	0	0	63	Katarinazavatska	2000
15389	0	0	0	64	Greetminnen	1997
15390	0	0	0	65	Kimberlybirrell	1998
15391	0	0	0	66	Liudmilasamsonova	1998
15392	0	0	0	67	Monikakilnarova	1999
15393	0	0	0	68	Ornellagaravani	1998
15394	0	0	0	69	Maddisoninglis	1998
15395	0	0	0	70	Ingridneel	1998
15396	0	0	0	71	Naikthabains	1997

15397	0	0	0	72	Shuyuema	1999
15398	0	0	0	73	Vanessawong	1999
15399	0	0	0	74	Karola Patriciabejenaru	1997
15400	0	0	0	75	Abigaildesiatnikov	2001
15401	0	0	0	76	Emilianaarango	2000
15402	0	0	0	77	Melany Solangekrywoj	1998
15403	0	0	0	78	Seonemendez	1999
15404	0	0	0	79	Maria Joseportillo ramirez	1999
15405	0	0	0	80	Raveenakingsley	1998
15406	0	0	0	81	Raquelpedraza	1998
15407	0	0	0	82	Mirjambjorklund	1998
15408	0	0	0	83	Dominiqueschaefer	1999
15409	0	0	0	84	Sophiachow	1997
15410	0	0	0	85	Eugeniaganga	1998
15411	0	0	0	86	Oliviahauger	1997
15412	0	0	0	87	Luciewargnier	1999
15413	0	0	0	88	Yanniliu	1999
15414	0	0	0	89	Danielamorales beckmann	1998
15415	0	0	0	90	Natsuhoarakawa	1997
15416	0	0	0	91	Priscillahon	1998
15417	0	0	0	92	Miahorvit	1997
15418	1	1	1	1	Catherinebellis	1999
15419	1	1	1	2	Shilinxu	1998
15420	1	1	1	3	Irynashymanovich	1997
15421	1	1	1	4	Jilteichmann	1997
15422	1	1	1	5	Dariakasatkina	1997
15423	1	1	1	6	Kristinaschmiedlova	1997
15424	1	1	1	7	Mariebouzkova	1998
15425	1	1	1	8	Jelenaostapenko	1997
15426	1	1	1	9	Paulabadosa	1997
15427	1	1	1	10	Alionabolsova	1997
15428	0	1	1	11	Anhelinakalinina	1997
15429	0	1	1	12	Marketavondrousova	1999
15430	0	1	1	13	Elena Gabrielaruse	1997
15431	0	1	1	14	Dalmagalfi	1998
15432	0	1	1	15	Annakalinskaya	1998
15433	0	1	1	16	Fannistollar	1998
15434	0	1	1	17	Sofiakenin	1998
15435	0	1	1	18	Ioana Loredanarosca	1996
15436	0	1	1	19	Kimberlybirrell	1998
15437	0	1	1	20	Nataliavikhlyantseva	1997
15438	0	1	1	21	Anastasiyakomardina	1997
15439	0	1	1	22	Naikthabains	1997
15440	0	1	1	23	Olgafridman	1998
15441	0	1	1	24	Raveenakingsley	1998
15442	0	1	1	25	Usue Maitanearconada	1998
15443	0	0	1	26	Katieboulter	1996
15444	0	0	1	27	Ziyuesun	1996
15445	0	0	1	28	Annablinkova	1998
15446	0	0	1	29	Annabondar	1997

15447	0	0	1	30	Luisastefani	1997
15448	0	0	1	31	Seonemendez	1999
15449	0	0	1	32	Greetminnen	1997
15450	0	0	1	33	Evgeniyalevashova	1999
15451	0	0	1	34	Akvileparazinskaite	1996
15452	0	0	1	35	Wushuangzheng	1998
15453	0	0	1	36	Katieswan	1999
15454	0	0	1	37	Sandrasamir	1997
15455	0	0	1	38	Viktoriahruncakova	1998
15456	0	0	1	39	Tamigrende	1997
15457	0	0	1	40	Luciewargnier	1999
15458	0	0	1	41	Renatazarazua	1997
15459	0	0	1	42	Kamonwanbuayam	1996
15460	0	0	1	43	Biancaturati	1997
15461	0	0	1	44	Simonaheinova	1997
15462	0	0	1	45	Michaelagordon	1999
15463	0	0	1	46	Dashaivanova	1996
15464	0	0	1	47	Maialumsden	1998
15465	0	0	1	48	Gabriellataylor	1998
15466	0	0	1	49	Destaneeaiava	2000
15467	0	0	1	50	Terezamihalikova	1998
15468	0	0	0	51	Priscillahon	1998
15469	0	0	0	52	Fionaferro	1997
15470	0	0	0	53	Tessahandrianjafitrimo	1998
15471	0	0	0	54	Adeliyazabirova	1998
15472	0	0	0	55	Katherinesebov	1999
15473	0	0	0	56	Katarinajokic	1998
15474	0	0	0	57	Helenploskina	1997
15475	0	0	0	58	Ioanaducu	1996
15476	0	0	0	59	Margotyerolymos	1997
15477	0	0	0	60	Leticiagarcia vidal	1997
15478	0	0	0	61	Jessicaho	1997
15479	0	0	0	62	Isabellewallace	1996
15480	0	0	0	63	Domenicagonzalez	1996
15481	0	0	0	64	Oliviatjandramulia	1997
15482	0	0	0	65	Maria Fernandaherazo gonzalez	1997
15483	0	0	0	66	Miahorvit	1997
15484	0	0	0	67	Raquelpedraza	1998
15485	0	0	0	68	Ojasvineesingh	1998
15486	0	0	0	69	Juliagrabher	1996
15487	0	0	0	70	Emiliefrancati	1997
15488	0	0	0	71	Emilyarbuthnott	1997
15489	0	0	0	72	Freyachristie	1997
15490	0	0	0	73	Charlotterobillard-millette	1999
15491	0	0	0	74	Oliviahauger	1997
15492	0	0	0	75	Fernandaastete	1997
15493	0	0	0	76	Qiu Yuye	1997
15494	0	0	0	77	Madisonbourguignon	1997
15495	0	0	0	78	Verenahofer	1997
15496	0	0	0	79	Yolimarogando	1996

15497	0	0	0	80	Aleksandrapospelova	1998
15498	0	0	0	81	Zhanlanwei	1998
15499	0	0	0	82	Georgiabrescia	1996
15500	0	0	0	83	Xiaodiyou	1996
15501	0	0	0	84	Jaquelinecristian	1998
15502	0	0	0	85	Justinamikulskyte	1996
15503	0	0	0	86	Kellychen	1999
15504	0	0	0	87	Veronikamiroshnichenko	1997
15505	0	0	0	88	Lesedi Sheyajacobs	1997
15506	0	0	0	89	Anastasiamikheeva	1999
15507	0	0	0	90	Mayukaaikawa	1998
15508	0	0	0	91	Rosiejohanson	1998
15509	0	0	0	92	Vanessawong	1999
15510	0	0	0	93	Katrine Isabelsteffensen	1996
15511	0	0	0	94	Emmanuellesalas	1998
15512	0	0	0	95	Alexandrasanford	1999
15513	0	0	0	96	Jennafriedel	1997
15514	0	0	0	97	Jodie Annaburrage	1999
15515	0	0	0	98	Lizettecabrera	1997
15516	0	0	0	99	Reginaclark	1996
15517	0	0	0	100	Emilysmith	1997
15518	1	1	1	1	Belindabencic	1997
15519	1	1	1	2	Anakonjuh	1997
15520	1	1	1	3	Varvaraflink	1996
15521	1	1	1	4	Barborakrejcikova	1995
15522	1	1	1	5	Ivanajorovic	1997
15523	1	1	1	6	Katerinasiniakova	1996
15524	1	1	1	7	Tornado Aliciablack	1998
15525	1	1	1	8	Dariakasatkina	1997
15526	1	1	1	9	Louisachirico	1996
15527	0	1	1	10	Elisemertens	1995
15528	0	1	1	11	Camilagiangreco campiz	1996
15529	0	1	1	12	Anhelinakalinina	1997
15530	0	1	1	13	Irynashymanovich	1997
15531	0	1	1	14	Shilinxu	1998
15532	0	1	1	15	Jelenaostapenko	1997
15533	0	1	1	16	Ipeksoylu	1996
15534	0	1	1	17	Ioanaducu	1996
15535	0	1	1	18	Ninastojanovic	1996
15536	0	1	1	19	Alejandracisneros	1995
15537	0	1	1	20	Constanzavega	1995
15538	0	1	1	21	Mariamarfutina	1997
15539	0	1	1	22	Mariebouzkova	1998
15540	0	1	1	23	Alicematteucci	1995
15541	0	0	1	24	Ziyuesun	1996
15542	0	0	1	25	Veronikakudermetova	1997
15543	0	0	1	26	Victoriarodriguez	1995
15544	0	0	1	27	Akvileparazinskaite	1996
15545	0	0	1	28	Helenploskina	1997
15546	0	0	1	29	Elizavetakulichkova	1996

15547	0	0	1	30	Sandrasamir	1997
15548	0	0	1	31	Karinkennel	1995
15549	0	0	1	32	Kristinaschmiedlova	1997
15550	0	0	1	33	Christinamakarova	1996
15551	0	0	1	34	Beatrizhaddad maia	1996
15552	0	0	1	35	Katieboulter	1996
15553	0	0	1	36	Viktoriyalushkova	1996
15554	0	0	1	37	Katrine Isabelsteffensen	1996
15555	0	0	1	38	Johnniserenaud	1996
15556	0	0	1	39	Anastasiyakomardina	1997
15557	0	0	1	40	Domenicagonzalez	1996
15558	0	0	1	41	Alinasilich	1996
15559	0	0	1	42	Ching-Wenhsu	1996
15560	0	0	1	43	Xiaodiyou	1996
15561	0	0	1	44	Saratomic	1998
15562	0	0	1	45	Renatazarazua	1997
15563	0	0	0	46	Fionaferro	1997
15564	0	0	0	47	Carolzhao	1995
15565	0	0	0	48	Lauraucros	1995
15566	0	0	0	49	Isabellewallace	1996
15567	0	0	0	50	Janafett	1996
15568	0	0	0	51	Carolinaalves	1996
15569	0	0	0	52	Dashaivanova	1996
15570	0	0	0	53	Asiyadair	1996
15571	0	0	0	54	Kamonwanbuayam	1996
15572	0	0	0	55	Michaelagordon	1999
15573	0	0	0	56	Jilteichmann	1997
15574	0	0	0	57	Marikaakkerman	1995
15575	0	0	0	58	Margotyerolymos	1997
15576	0	0	0	59	Beatricelombardo	1996
15577	0	0	0	60	Ilkacsoregi	1996
15578	0	0	0	61	Katherineip	1995
15579	0	0	0	62	Jamieloeb	1995
15580	0	0	0	63	Harrietdart	1996
15581	0	0	0	64	Naikthabains	1997
15582	0	0	0	65	Katydunne	1995
15583	0	0	0	66	Riannavaldes	1996
15584	0	0	0	67	Kyokaokamura	1995
15585	0	0	0	68	Usue Maitanearconada	1998
15586	0	0	0	69	Hikariyamamoto	1995
15587	0	0	0	70	Gabrielapantuckova	1995
15588	0	0	0	71	Szabinaszlavikovics	1995
15589	0	0	0	72	Madisonbourguignon	1997
15590	0	0	0	73	Giuliapairone	1996
15591	0	0	0	74	Brittgeukens	1996
15592	0	0	0	75	Cassandravazquez	1996
15593	0	0	0	76	Vasilisaaponasenko	1996
15594	0	0	0	77	Deborahchiesa	1996
15595	0	0	0	78	Maria Paulinaperez-garcia	1996
15596	0	0	0	79	Mamiadachi	1996

15597	0	0	0	80	Emilyarbuthnott	1997
15598	0	0	0	81	Miraruder-hook	1996
15599	0	0	0	82	Constanzagorches	1998
15600	0	0	0	83	Lizettecabrera	1997
15601	0	0	0	84	Ana Paulaneffa de los rios	1997
15602	0	0	0	85	Saraottomano	1996
15603	0	0	0	86	Emma Christinehiguchi	1998
15604	0	0	0	87	Ingridmartins	1996
15605	0	0	0	88	Hanamortagy	1998
15606	0	0	0	89	Elyssegraci	1996
15607	1	1	1	1	Taylortownsend	1996
15608	1	1	1	2	Katerinasiniakova	1996
15609	1	1	1	3	Eugeniebouchard	1994
15610	1	1	1	4	Anakonjuh	1997
15611	1	1	1	5	Antonialottner	1996
15612	1	1	1	6	Francoiseabanda	1997
15613	1	1	1	7	Marcelazacarias	1994
15614	1	1	1	8	Petrauberalova	1995
15615	1	1	1	9	Belindabencic	1997
15616	0	1	1	10	Elizavetakulichkova	1996
15617	0	1	1	11	Carolzhao	1995
15618	0	1	1	12	Annadanilina	1995
15619	0	1	1	13	Ching-Wenhsu	1996
15620	0	1	1	14	Chalenascholl	1995
15621	0	1	1	15	Sachiavickery	1995
15622	0	1	1	16	Samanthacrawford	1995
15623	0	1	1	17	Maria Inesdeheza	1994
15624	0	1	1	18	Christinamakarova	1996
15625	0	1	1	19	Elisemertens	1995
15626	0	1	1	20	Anettkontaveit	1995
15627	0	1	1	21	Beatrizhaddad maia	1996
15628	0	1	1	22	Dariasalnikova	1994
15629	0	0	1	23	Kylemcphillips	1994
15630	0	0	1	24	Barborakrejcikova	1995
15631	0	0	1	25	Laurapigossi	1994
15632	0	0	1	26	Katydunne	1995
15633	0	0	1	27	Erinroutliffe	1995
15634	0	0	1	28	Alliekiick	1995
15635	0	0	1	29	Barbarahaas	1996
15636	0	0	1	30	Oleksandrakorashvili	1996
15637	0	0	1	31	Ilkacsoregi	1996
15638	0	0	1	32	Montserratgonzalez	1994
15639	0	0	1	33	Constanzavega	1995
15640	0	0	1	34	Ayakaokuno	1995
15641	0	0	1	35	Jenniferbrady	1995
15642	0	0	1	36	Camillarosatello	1995
15643	0	0	1	37	Adrijanalekaj	1995
15644	0	0	1	38	Domenicagonzalez	1996
15645	0	0	1	39	Rebeccapeterson	1995
15646	0	0	1	40	Ellenallgurin	1994

15647	0	0	1	41	Gabrielle Faithandrews	1996
15648	0	0	1	42	Jelenaostapenko	1997
15649	0	0	1	43	Ipeksoylu	1996
15650	0	0	1	44	Kimberleyzimmermann	1995
15651	0	0	0	45	Ioana Loredanarosca	1996
15652	0	0	0	46	Victoriarodriguez	1995
15653	0	0	0	47	Hikariyamamoto	1995
15654	0	0	0	48	Aldilasutjiadi	1995
15655	0	0	0	49	Elkelemmens	1994
15656	0	0	0	50	Lauraucros	1995
15657	0	0	0	51	Fionaferro	1997
15658	0	0	0	52	Nataliavajdova	1995
15659	0	0	0	53	Sabinasharipova	1994
15660	0	0	0	54	Olgadoroshina	1994
15661	0	0	0	55	Victoriabosio	1994
15662	0	0	0	56	Dominikapaterova	1995
15663	0	0	0	57	Zarahrazafimahatratra	1994
15664	0	0	0	58	Mariebenoit	1995
15665	0	0	0	59	Maiarsherif ahmed abdelaziz	1996
15666	0	0	0	60	Fernyangeles paz	1995
15667	0	0	0	61	Mamiadachi	1996
15668	0	0	0	62	Elenamaltseva	1994
15669	0	0	0	63	Varvaraflink	1996
15670	0	0	0	64	Valeriapatiuk	1996
15671	0	0	0	65	Emilyfanning	1995
15672	0	0	0	66	Klaartjeliebens	1995
15673	0	0	0	67	Katrine Isabelsteffensen	1996
15674	0	0	0	68	Tornado Aliciablack	1998
15675	0	0	0	69	Justinede sutter	1995
15676	0	0	0	70	Kathinkavon deichmann	1994
15677	0	0	0	71	Zuzannamaciejewska	1995
15678	0	0	0	72	Michellewerbrouck	1995
15679	0	0	0	73	Alejandracisneros	1995
15680	0	0	0	74	Blairshankle	1994
15681	0	0	0	75	Denisestarr	1995
15682	0	0	0	76	Indyde vroome	1996
15683	0	0	0	77	Ivamekovec	1995
15684	0	0	0	78	Louisachirico	1996
15685	0	0	0	79	Naikthabains	1997
15686	0	0	0	80	Isabellewallace	1996
15687	0	0	0	81	Marikaakkerman	1995
15688	0	0	0	82	Maielkamash	1994
15689	0	0	0	83	Kanikavaidya	1995
15690	0	0	0	84	Deborahkerfs	1995
15691	0	0	0	85	Carmenblanco	1995
15692	0	0	0	86	Junelee	1995
15693	0	0	0	87	Sujeongjang	1995
15694	1	1	1	1	Irinakhromacheva	1995
15695	1	1	1	2	Ashleighbarty	1996
15696	1	1	1	3	An-Sophiemestach	1994

15697	1	1	1	4	Eugeniebouchard	1994
15698	1	1	1	5	Carolinegarcia	1993
15699	1	1	1	6	Gracemin	1994
15700	1	1	1	7	Dankakovinic	1994
15701	1	1	1	8	Montserratgonzalez	1994
15702	1	1	1	9	Anettkontaveit	1995
15703	0	1	1	10	Indyde vroome	1996
15704	0	1	1	11	Yuliaputintseva	1995
15705	0	1	1	12	Jovanajovic	1993
15706	0	1	1	13	Victoriabosio	1994
15707	0	1	1	14	Anna Karolinaschmiedlova	1994
15708	0	1	1	15	Victoriakan	1995
15709	0	1	1	16	Zuzannamaciejewska	1995
15710	0	1	1	17	Jesikamaleckova	1994
15711	0	1	1	18	Sabinasharipova	1994
15712	0	1	1	19	Donnavekic	1996
15713	0	1	1	20	Victoriaduval	1995
15714	0	1	1	21	Domenicagonzalez	1996
15715	0	0	1	22	Natalijastevanovic	1994
15716	0	0	1	23	Klarafabikova	1993
15717	0	0	1	24	Gannapoznikhirenko	1994
15718	0	0	1	25	Sofiyakovalets	1994
15719	0	0	1	26	Patriciaku flores	1993
15720	0	0	1	27	Ellenallgurin	1994
15721	0	0	1	28	Miyukato	1994
15722	0	0	1	29	Demischuurs	1993
15723	0	0	1	30	Zarahrazafimahatratra	1994
15724	0	0	1	31	Petrarohanova	1995
15725	0	0	1	32	Aliaksandrasasnovich	1994
15726	0	0	1	33	Elizavetakulichkova	1996
15727	0	0	1	34	Carolzhao	1995
15728	0	0	1	35	Makotoninomiya	1994
15729	0	0	1	36	Christinamakarova	1996
15730	0	0	1	37	Ilonakremen	1994
15731	0	0	1	38	Emilyfanning	1995
15732	0	0	1	39	Abbiemyers	1994
15733	0	0	1	40	Stephanienauta	1994
15734	0	0	1	41	Naohibino	1994
15735	0	0	1	42	Marie Elisecasares	1993
15736	0	0	0	43	Barbarahaas	1996
15737	0	0	0	44	Risaozaki	1994
15738	0	0	0	45	Varvaraflink	1996
15739	0	0	0	46	Ayakaokuno	1995
15740	0	0	0	47	Viktoriamalova	1993
15741	0	0	0	48	Mihokowase	1995
15742	0	0	0	49	Elena-Teodoracadar	1994
15743	0	0	0	50	Kelseylaurente	1994
15744	0	0	0	51	Beatrizhaddad maia	1996
15745	0	0	0	52	Ana Sofiasanchez	1994
15746	0	0	0	53	Viktoriyatomova	1995

15747	0	0	0	54	Deborahkerfs	1995
15748	0	0	0	55	Mamiadachi	1996
15749	0	0	0	56	Meganebianco	1994
15750	0	0	0	57	Victoriarodriguez	1995
15751	0	0	0	58	Rikosawayanagi	1994
15752	0	0	0	59	Katydunne	1995
15753	0	0	0	60	Catherineharrison	1994
15754	0	0	0	61	Marcelazacarias	1994
15755	0	0	0	62	Laurapigossi	1994
15756	0	0	0	63	Juliaelbaba	1994
15757	0	0	0	64	Olgaianchuk	1995
15758	0	0	0	65	Katharinalehnert	1994
15759	0	0	0	66	Nadiaecheverria alam	1996
15760	0	0	0	67	Blairshankle	1994
15761	0	0	0	68	Chantalskamlova	1993
15762	0	0	0	69	Kanaminishimura	1996
15763	0	0	0	70	Dariasaville	1994
15764	0	0	0	71	Denisestarr	1995
15765	0	0	0	72	Erihozumi	1994
15766	0	0	0	73	Dariasalnikova	1994
15767	0	0	0	74	Tristen Zdewar	1994
15768	0	0	0	75	Kimberley-Annsurin	1994
15769	0	0	0	76	Luciabutkovska	1993
15770	0	0	0	77	Ekaterinasemenova	1994
15771	0	0	0	78	Ximenasiles luna	1994
15772	0	0	0	79	Valeriasalazar	1995
15773	0	0	0	80	Kriszinakapitany	1993
15774	0	0	0	81	Elizaveta Annanemchinov	1993
15775	0	0	0	82	Lauradeigman	1993
15776	0	0	0	83	Verenascott	1994
15777	0	0	0	84	Anaevepain	1993
15778	1	1	1	1	Dariasaville	1994
15779	1	1	1	2	Elinasvitolina	1994
15780	1	1	1	3	Laurendavis	1993
15781	1	1	1	4	Monicapuig	1993
15782	1	1	1	5	Irinakhromacheva	1995
15783	1	1	1	6	Kristynapliskova	1992
15784	1	1	1	7	Timeababos	1993
15785	1	1	1	8	Karolinapliskova	1992
15786	1	1	1	9	Saisaizheng	1994
15787	0	1	1	10	An-Sophiemestach	1994
15788	0	1	1	11	Sachieishizu	1992
15789	0	1	1	12	Janacepelova	1993
15790	0	1	1	13	Yuliaputintseva	1995
15791	0	1	1	14	Onsjabeur	1994
15792	0	1	1	15	Veronicacepede royg	1992
15793	0	1	1	16	Sofiyakovalets	1994
15794	0	1	1	17	Carolinegarcia	1993
15795	0	1	1	18	Cristinadinu	1993
15796	0	1	1	19	Chantalskamlova	1993

15797	0	1	1	20	Hao Chentang	1994
15798	0	1	1	21	Dariasalnikova	1994
15799	0	1	1	22	Ilonakremen	1994
15800	0	0	1	23	Gracemin	1994
15801	0	0	1	24	Nastjakolar	1994
15802	0	0	1	25	Zarahrazafimahatratra	1994
15803	0	0	1	26	Tamaracurovic	1994
15804	0	0	1	27	Madisonkeys	1995
15805	0	0	1	28	Gabrieladabrowski	1992
15806	0	0	1	29	Denisasatralova	1993
15807	0	0	1	30	Kristahardebeck	1994
15808	0	0	1	31	Agustina Soleskenazi	1993
15809	0	0	1	32	Eugeniebouchard	1994
15810	0	0	1	33	Rantian	1994
15811	0	0	1	34	Andreagamiz	1992
15812	0	0	1	35	Dorotejaeric	1992
15813	0	0	1	36	Anna Karolinaschmiedlova	1994
15814	0	0	1	37	Dankakovinic	1994
15815	0	0	1	38	Silvianjiric	1993
15816	0	0	1	39	Adrianaperez	1992
15817	0	0	1	40	Alisonvan uytvanck	1994
15818	0	0	1	41	Ceciliacosta melgar	1992
15819	0	0	1	42	Miyukato	1994
15820	0	0	1	43	Luksikakumkhum	1993
15821	0	0	1	44	Ellenallgurin	1994
15822	0	0	0	45	Erihozumi	1994
15823	0	0	0	46	Ulrikkeeikeri	1992
15824	0	0	0	47	Katarenapaliivets	1992
15825	0	0	0	48	Luciabutkovska	1993
15826	0	0	0	49	Leoliajeanjean	1995
15827	0	0	0	50	Fernandabrito	1992
15828	0	0	0	51	Chanellevan nguyen	1994
15829	0	0	0	52	Grace Sariysidora	1993
15830	0	0	0	53	Viktoriyatomova	1995
15831	0	0	0	54	Demischuurs	1993
15832	0	0	0	55	Maigrage	1992
15833	0	0	0	56	Sabinevan der sar	1992
15834	0	0	0	57	Laurenherring	1993
15835	0	0	0	58	Aliaksandrasasnovich	1994
15836	0	0	0	59	Jessicapegula	1994
15837	0	0	0	60	Monicaturewicz	1993
15838	0	0	0	61	Jenniferren	1993
15839	0	0	0	62	Emimutaguchi	1993
15840	0	0	0	63	Polinaprudnikava	1992
15841	0	0	0	64	Saskagavrilovska	1995
15842	0	0	0	65	Victoriabosio	1994
15843	0	0	0	66	Rishikasunkara	1993
15844	0	0	0	67	Anna-Lenafriedsam	1994
15845	0	0	0	68	Risaozaki	1994
15846	0	0	0	69	Clothildede bernardi	1994

15847	0	0	0	70	Caitlynwilliams	1993
15848	0	0	0	71	Nourabbes	1993
15849	0	0	0	72	Riokitagawa	1993
15850	0	0	0	73	Estergoldfeld	1993
15851	0	0	0	74	Anniemulholland	1993
15852	0	0	0	75	Lucybrown	1993
15853	0	0	0	76	Sachiavickery	1995
15854	0	0	0	77	Elisabethabanda	1994
15855	0	0	0	78	Katerinapopova	1993
15856	0	0	0	79	Santashumilina	1992
15857	0	0	0	80	Marcelazacarias	1994
15858	0	0	0	81	Melissezer	1993
15859	0	0	0	82	Kseniakirillova	1993
15860	0	0	0	83	Stefanietan	1992
15861	0	0	0	84	Mariannejodoin	1993
15862	0	0	0	85	Kerriecartwright	1992
15863	0	0	0	86	Akikoomae	1993
15864	0	0	0	87	Polinaleykina	1994
15865	0	0	0	88	Noelscott	1993
15866	1	1	1	1	Kristinamladenovic	1993
15867	1	1	1	2	Noppawanlertcheewakarn	1991
15868	1	1	1	3	Timeababos	1993
15869	1	1	1	4	Heatherwatson	1992
15870	1	1	1	5	Dariasaville	1994
15871	1	1	1	6	Miyabiinoue	1991
15872	1	1	1	7	Silvianjiric	1993
15873	1	1	1	8	Ajlatomljanovic	1993
15874	1	1	1	9	Camilasilva	1992
15875	0	1	1	10	Sloanestephens	1993
15876	0	1	1	11	Nastjakolar	1994
15877	0	1	1	12	Gabrieladabrowski	1992
15878	0	1	1	13	Yanabuchina	1992
15879	0	1	1	14	Marynazanevska	1993
15880	0	1	1	15	Yuliaputintseva	1995
15881	0	1	1	16	Richelhogenkamp	1992
15882	0	1	1	17	Valeriasolovyeva	1992
15883	0	1	1	18	Zsofiasusanyi	1992
15884	0	1	1	19	Janacepelova	1993
15885	0	1	1	20	Irinakhromacheva	1995
15886	0	1	1	21	Ulrikkeeikeri	1992
15887	0	0	1	22	Chanelsimmonds	1992
15888	0	0	1	23	Saisaizheng	1994
15889	0	0	1	24	Laurarobson	1994
15890	0	0	1	25	Anabogdan	1992
15891	0	0	1	26	Polinaleykina	1994
15892	0	0	1	27	Madisonkeys	1995
15893	0	0	1	28	Kseniakirillova	1993
15894	0	0	1	29	Aleksandrakrunic	1993
15895	0	0	1	30	Tamaracurovic	1994
15896	0	0	1	31	Akikoomae	1993

15897	0	0	1	32	Magdalinette	1992
15898	0	0	1	33	Annaorlik	1993
15899	0	0	1	34	Veronicacepede royg	1992
15900	0	0	1	35	Grace Sariysidora	1993
15901	0	0	1	36	Eugeniebouchard	1994
15902	0	0	1	37	Katarenapaliivets	1992
15903	0	0	1	38	Nicolegibbs	1993
15904	0	0	1	39	Paulaormaechea	1992
15905	0	0	1	40	Beatricecapra	1992
15906	0	0	1	41	Estergoldfeld	1993
15907	0	0	0	42	Emimutaguchi	1993
15908	0	0	0	43	Elenabogdan	1992
15909	0	0	0	44	Nataliyapintusova	1992
15910	0	0	0	45	Sinahaas	1992
15911	0	0	0	46	Fatmaal nabhani	1991
15912	0	0	0	47	Anna Arinamarenko	1992
15913	0	0	0	48	Vivienjuhaszova	1993
15914	0	0	0	49	Luksikakumkhum	1993
15915	0	0	0	50	Martinatrevisan	1993
15916	0	0	0	51	Adrianaperez	1992
15917	0	0	0	52	Ekaterinanikitina	1993
15918	0	0	0	53	Chantalskamlova	1993
15919	0	0	0	54	Gracemin	1994
15920	0	0	0	55	Polinavinogradova	1993
15921	0	0	0	56	Stephaniecornish	1991
15922	0	0	0	57	Ilincastoica	1993
15923	0	0	0	58	Laurenembree	1991
15924	0	0	0	59	Noelscott	1993
15925	0	0	0	60	Malouejdesgaard	1991
15926	0	0	0	61	Ting-Feijuan	1992
15927	0	0	0	62	Hannahjames	1991
15928	0	0	0	63	Alexandracercone	1991
15929	0	0	0	64	Malloryburdette	1991
15930	0	0	0	65	Lesleypattinama kerkhove	1991
15931	0	0	0	66	Vasziliszabulgakova	1993
15932	0	0	0	67	Brookebolender	1992
15933	0	0	0	68	Sachiavickery	1995
15934	0	0	0	69	Kyrashroff	1992
15935	0	0	0	70	Ziyang	1993
15936	0	0	0	71	Quirinelemoine	1991
15937	0	0	0	72	Rashmiteltumbde	1992
15938	0	0	0	73	Jessicapegula	1994
15939	0	0	0	74	Elishagabb	1993
15940	0	0	0	75	Kerriecartwright	1992
15941	0	0	0	76	Daneikaborthwick	1993
15942	0	0	0	77	Alejandragranillo	1991
15943	0	0	0	78	Michelacasanova	1992
15944	0	0	0	79	Monicayajima	1992
15945	0	0	0	80	Isabellaholland	1992
15946	0	0	0	81	Fernandafaria	1991

15947	0	0	0	82	Andreatabachnik	1992
15948	1	1	1	1	Noppawanlertcheewakarn	1991
15949	1	1	1	2	Arantxarus	1990
15950	1	1	1	3	Simonahalep	1991
15951	1	1	1	4	Elenabogdan	1992
15952	1	1	1	5	Laurarobson	1994
15953	1	1	1	6	Anabogdan	1992
15954	1	1	1	7	Kuruminara	1991
15955	1	1	1	8	Melanieoudin	1991
15956	1	1	1	9	Bojanajovanovski petrovic	1991
15957	0	1	1	10	Kristinamladenovic	1993
15958	0	1	1	11	Jessyrompies	1990
15959	0	1	1	12	Akiyamasoto	1991
15960	0	1	1	13	Lesleypattinama kerkhove	1991
15961	0	1	1	14	Ajlatomljanovic	1993
15962	0	1	1	15	Cindychala	1991
15963	0	1	1	16	Viktoriakamenskaya	1991
15964	0	1	1	17	Timeababos	1993
15965	0	1	1	18	Zarinadiyas	1993
15966	0	1	1	19	Heatherwatson	1992
15967	0	1	1	20	Johannakonta	1991
15968	0	1	1	21	Lindaberlinecke	1991
15969	0	1	1	22	Alexandradamaschin	1991
15970	0	0	1	23	Ksenialykina	1990
15971	0	0	1	24	Isabellaholland	1992
15972	0	0	1	25	Yanabuchina	1992
15973	0	0	1	26	Beatricegumulya	1991
15974	0	0	1	27	Richelhogenkamp	1992
15975	0	0	1	28	Miyabiinoue	1991
15976	0	0	1	29	Aranzasalut	1991
15977	0	0	1	30	Nastassjaburnett	1992
15978	0	0	1	31	Valeriasolovyeva	1992
15979	0	0	1	32	Zuzanaluknarova	1991
15980	0	0	1	33	Elenachernyakova	1990
15981	0	0	1	34	Reka Lucajani	1991
15982	0	0	1	35	Malloryburdette	1991
15983	0	0	1	36	Anastasiagrymalska	1990
15984	0	0	1	37	Romanatabak	1991
15985	0	0	1	38	Sandrazaniewska	1992
15986	0	0	1	39	Zi-Junyang	1990
15987	0	0	1	40	Annaorlik	1993
15988	0	0	1	41	Monicagorny	1991
15989	0	0	1	42	Martasirotkina	1991
15990	0	0	1	43	Pamelamontez	1991
15991	0	0	0	44	Misakidoi	1991
15992	0	0	0	45	Zsofiasusanyi	1992
15993	0	0	0	46	Silvianjiric	1993
15994	0	0	0	47	Zuzanalinhova	1990
15995	0	0	0	48	Monikatumova	1991
15996	0	0	0	49	Amandinehesse	1993

15997	0	0	0	50	Karinapimkina	1991
15998	0	0	0	51	Biancaswanepoel	1991
15999	0	0	0	52	Martinaborecka	1991
16000	0	0	0	53	Milanaspremo	1991
16001	0	0	0	54	Biancabotto	1991
16002	0	0	0	55	Sallypeers	1991
16003	0	0	0	56	Nadialalami	1990
16004	0	0	0	57	Kanyapatnarattana	1992
16005	0	0	0	58	Katarzynapiter	1991
16006	0	0	0	59	Valeriasavinykh	1991
16007	0	0	0	60	Bistraotashliyska	1991
16008	0	0	0	61	Alejandragranillo	1991
16009	0	0	0	62	Hannahjames	1991
16010	0	0	0	63	Annaberta	1991
16011	0	0	0	64	Paulaormaechea	1992
16012	0	0	0	65	Martinatrevisan	1993
16013	0	0	0	66	Stephaniecornish	1991
16014	0	0	0	67	Jadecurtis	1990
16015	0	0	0	68	Nicolebartnik	1991
16016	0	0	0	69	Marynazanevska	1993
16017	0	0	0	70	Nichalertpitaksinchai	1991
16018	0	0	0	71	Katarenapaliivets	1992
16019	0	0	0	72	Dianamarcu	1991
16020	0	0	0	73	Gabrieladabrowski	1992
16021	0	0	0	74	Alexaguarachi	1990
16022	0	0	0	75	Rebeccamarino	1990
16023	0	0	0	76	Monicayajima	1992
16024	0	0	0	77	Laura-Aliciaaguilar	1990
16025	0	0	0	78	Victoriabrook	1990
16026	0	0	0	79	Francescasegarelli	1990
16027	0	0	0	80	Malouejdesgaard	1991
16028	0	0	0	81	Taramoore	1992
16029	0	0	0	82	Poojashreevenkatesha	1990
16030	0	0	0	83	Lauraslater	1991
16031	0	0	0	84	Daniellemills	1991
16032	0	0	0	85	Hao-Chingchan	1993
16033	1	1	1	1	Urszularadwanska	1990
16034	1	1	1	2	Anastasiapavlyuchenkova	1991
16035	1	1	1	3	Kristinakucova	1990
16036	1	1	1	4	Melanieoudin	1991
16037	1	1	1	5	Anastasiapivovarova	1990
16038	1	1	1	6	Kseniapervak	1991
16039	1	1	1	7	Kuruminara	1991
16040	1	1	1	8	Ksenialykina	1990
16041	1	1	1	9	Nikolahofmanova	1991
16042	0	1	1	10	Madisonbrengle	1990
16043	0	1	1	11	Bojanajovanovski petrovic	1991
16044	0	1	1	12	Oksanakalashnikova	1990
16045	0	1	1	13	Juliaglushko	1990
16046	0	1	1	14	Noppawanlertcheewakarn	1991

16047	0	1	1	15	Katarzynapiter	1991
16048	0	1	1	16	Tamarynhendler	1992
16049	0	1	1	17	Elenachernyakova	1990
16050	0	1	1	18	Michellelarcher de brito	1993
16051	0	1	1	19	Juliacohen	1989
16052	0	1	1	20	Mallorycecil	1990
16053	0	1	1	21	Kai-Chenchang	1991
16054	0	0	1	22	Irina-Cameliabegu	1990
16055	0	0	1	23	Simonahalep	1991
16056	0	0	1	24	Lenkajurikova	1990
16057	0	0	1	25	Yi-Miaozhou	1991
16058	0	0	1	26	Cindychala	1991
16059	0	0	1	27	Arantxarus	1990
16060	0	0	1	28	Tatianabua	1990
16061	0	0	1	29	Stephanievogt	1990
16062	0	0	1	30	Rekazsilinszka	1989
16063	0	0	1	31	Jadecurtis	1990
16064	0	0	1	32	Gailbrodsky	1991
16065	0	0	1	33	Malenagordo	1990
16066	0	0	1	34	Tyracalderwood	1990
16067	0	0	1	35	Arinarodionova	1989
16068	0	0	1	36	Johannakonta	1991
16069	0	0	1	37	Klaudiaboczova	1990
16070	0	0	1	38	Romanatabak	1991
16071	0	0	1	39	Nellyciolkowski	1990
16072	0	0	1	40	Jessicamoore	1990
16073	0	0	1	41	Alexandradamaschin	1991
16074	0	0	1	42	Veronica Ruo Qili	1989
16075	0	0	0	43	Sandraroma	1990
16076	0	0	0	44	Lyudmylakichenok	1992
16077	0	0	0	45	Ioanaivan	1990
16078	0	0	0	46	Shihoakita	1990
16079	0	0	0	47	Misakidoi	1991
16080	0	0	0	48	Valeriasolovyeva	1992
16081	0	0	0	49	Nadialalami	1990
16082	0	0	0	50	Sung-Heehan	1990
16083	0	0	0	51	Sallypeers	1991
16084	0	0	0	52	Martasirotkina	1991
16085	0	0	0	53	Synakayser	1990
16086	0	0	0	54	Victorialarriere	1991
16087	0	0	0	55	Valeriapulido-velasco	1990
16088	0	0	0	56	Monicagorny	1991
16089	0	0	0	57	Yanabuchina	1992
16090	0	0	0	58	Taramoore	1992
16091	0	0	0	59	Kristyfrilling	1990
16092	0	0	0	60	Juliaboserup	1991
16093	0	0	0	61	Yuukitanaka	1990
16094	0	0	0	62	Biancaswanepoel	1991
16095	0	0	0	63	Pamelamontez	1991
16096	0	0	0	64	Laura-Aliciaaguilar	1990

16097	0	0	0	65	Nadiiakichenok	1992
16098	0	0	0	66	Claudinepaulson	1990
16099	0	0	0	67	Anne-Christinevoicu	1990
16100	0	0	0	68	Alisonbai	1990
16101	0	0	0	69	Chenastrogo	1990
16102	0	0	0	70	Jeannineprentner	1990
16103	0	0	0	71	Alenkahubacek	1990
16104	0	0	0	72	Nanulipipiya	1992
16105	0	0	0	73	Nadejdaguskova	1992
16106	0	0	0	74	Gioiabarbieri	1991
16107	0	0	0	75	Karinapimkina	1991
16108	0	0	0	76	Francescasegarelli	1990
16109	0	0	0	77	Alejandragranillo	1991
16110	0	0	0	78	Paulaormaechea	1992
16111	0	0	0	79	Shivikaburman	1989
16112	0	0	0	80	Carolinemagnusson	1990
16113	0	0	0	81	Lauraslater	1991
16114	0	0	0	82	Giadagiovannad'ortona	1991
16115	0	0	0	83	Nicoleherzog	1991
16116	0	0	0	84	Paula Cristinagoncalves	1990
16117	1	1	1	1	Anastasiapavlyuchenkova	1991
16118	1	1	1	2	Carolinewozniacki	1990
16119	1	1	1	3	Kseniamilevskaya	1990
16120	1	1	1	4	Ayumimorita	1990
16121	1	1	1	5	Sharonfichman	1990
16122	1	1	1	6	Kristinaantoniychuk	1990
16123	1	1	1	7	Juliacohen	1989
16124	1	1	1	8	Urszularadwanska	1990
16125	1	1	1	9	Soranacirstea	1990
16126	1	1	1	10	Nikolahofmanova	1991
16127	0	1	1	11	Katerinavankova	1989
16128	0	1	1	12	Sachahughes	1990
16129	0	1	1	13	Tamirapaszek	1990
16130	0	1	1	14	Telianapereira	1988
16131	0	1	1	15	Alizecornet	1990
16132	0	1	1	16	Rekazsilinszka	1989
16133	0	1	1	17	Anastasiapivovarova	1990
16134	0	1	1	18	Ralucaolaru	1989
16135	0	1	1	19	Kristinakucova	1990
16136	0	1	1	20	Corinnadentoni	1989
16137	0	1	1	21	Dominiceripoll	1989
16138	0	1	1	22	Alexandradulgheru	1989
16139	0	1	1	23	Roxanevaisemberg	1989
16140	0	0	1	24	Madisonbrengle	1990
16141	0	0	1	25	Kseniapervak	1991
16142	0	0	1	26	Jessicamoore	1990
16143	0	0	1	27	Maria-Fernandaalvarez-teran	1989
16144	0	0	1	28	Naomicavaday	1989
16145	0	0	1	29	Alexandrapanova	1989
16146	0	0	1	30	Valeriapulido-velasco	1990

16147	0	0	1	31	Klaudiaboczova	1990
16148	0	0	1	32	Terezamrdeza	1990
16149	0	0	1	33	Aminarakhim	1989
16150	0	0	1	34	Kimberlycouts	1989
16151	0	0	1	35	Elenachernyakova	1990
16152	0	0	1	36	Katerinakramperova	1988
16153	0	0	1	37	Tamarynhendler	1992
16154	0	0	1	38	Lenalitvak	1988
16155	0	0	1	39	Marritboonstra	1988
16156	0	0	1	40	Kai-Chenchang	1991
16157	0	0	1	41	Michaelapochabova	1989
16158	0	0	1	42	Yaninawickmayer	1989
16159	0	0	1	43	Kseniapalkina	1989
16160	0	0	1	44	Ana-Claraduarte	1989
16161	0	0	1	45	Chelseygullickson	1990
16162	0	0	1	46	Mayagaverova	1989
16163	0	0	0	47	Emikoito	1989
16164	0	0	0	48	Venisechan	1989
16165	0	0	0	49	Dilyarasaidkhodjaeva	1990
16166	0	0	0	50	Evgeniyarodina	1989
16167	0	0	0	51	Klaudiamalenovska	1989
16168	0	0	0	52	Veronica Ruo Qili	1989
16169	0	0	0	53	Katharinakilli	1989
16170	0	0	0	54	Ksenialykina	1990
16171	0	0	0	55	Anouktigu	1989
16172	0	0	0	56	Denisedy	1989
16173	0	0	0	57	Andreaberkova	1990
16174	0	0	0	58	Lingzhang	1989
16175	0	0	0	59	Mailenauroux	1988
16176	0	0	0	60	Lindsayburdette	1988
16177	0	0	0	61	Melanieklaffner	1990
16178	0	0	0	62	Tyracalderwood	1990
16179	0	0	0	63	Ashleyweinhold	1989
16180	0	0	0	64	Sanaabhambri	1988
16181	0	0	0	65	Melanieoudin	1991
16182	0	0	0	66	Natasazoric	1989
16183	0	0	0	67	Jadecurtis	1990
16184	0	0	0	68	Bibianeschoofs	1988
16185	0	0	0	69	Monikakochanova	1989
16186	0	0	0	70	Aiyamamoto	1989
16187	0	0	0	71	Erikazanchetta	1989
16188	0	0	0	72	Courtneyclayton	1990
16189	0	0	0	73	Chloejones	1990
16190	0	0	0	74	Gailbrodsky	1991
16191	0	0	0	75	Laurenalbanese	1989
16192	0	0	0	76	Akariinoue	1988
16193	0	0	0	77	Kristyfrilling	1990
16194	0	0	0	78	Kim-Alicegrajdek	1991
16195	0	0	0	79	Jasminatinjic	1991
16196	0	0	0	80	Erinakikuchi	1989

16197	0	0	0	81	Andreea-Roxanavaideanu	1990
16198	0	0	0	82	Yurinakoshino	1988
16199	0	0	0	83	Irina-Alexandraparaschiv	1990
16200	0	0	0	84	Marinagiral lores	1990
16201	0	0	0	85	Katariinatuohimaa	1988
16202	0	0	0	86	Brittanyaugustine	1991
16203	0	0	0	87	Nadinefahoum	1989
16204	0	0	0	88	Francescasegarelli	1990
16205	0	0	0	89	Stefanienunic	1989
16206	0	0	0	90	Annafitzpatrick	1989
16207	0	0	0	91	Maxiehmer	1990
16208	1	1	1	1	Victoriaazarenka	1989
16209	1	1	1	2	Agnesszavay	1988
16210	1	1	1	3	Agnieszkaradwanska	1989
16211	1	1	1	4	Carolinewozniacki	1990
16212	1	1	1	5	Dominikacibulkova	1989
16213	1	1	1	6	Ralucaolaru	1989
16214	1	1	1	7	Alexandradulgheru	1989
16215	1	1	1	8	Alexaglatch	1989
16216	1	1	1	9	Latishachan	1989
16217	0	1	1	10	Mihaelabuzarnescu	1988
16218	0	1	1	11	Alisakleybanova	1989
16219	0	1	1	12	Ayumimorita	1990
16220	0	1	1	13	Aleksandrawozniak	1987
16221	0	1	1	14	Vaniaking	1989
16222	0	1	1	15	Bibianeschoofs	1988
16223	0	1	1	16	Sharonfichman	1990
16224	0	1	1	17	Soranacirstea	1990
16225	0	1	1	18	Yaroslavashvedova	1987
16226	0	1	1	19	Aminarakhim	1989
16227	0	1	1	20	Marinaerakovic	1988
16228	0	1	1	21	Tamirapaszek	1990
16229	0	1	1	22	Nikolafrankova	1988
16230	0	1	1	23	Wen-Hsinhsu	1988
16231	0	1	1	24	Annatatishvili	1990
16232	0	0	1	25	Evgeniyarodina	1989
16233	0	0	1	26	Ellenbarry	1989
16234	0	0	1	27	Ekaterinamakarova	1988
16235	0	0	1	28	Valerietetreault	1988
16236	0	0	1	29	Roxanevaisemberg	1989
16237	0	0	1	30	Jennifer-Leeheinser	1987
16238	0	0	1	31	Reneereinhard	1990
16239	0	0	1	32	Juliacohen	1989
16240	0	0	1	33	Katerinakramperova	1988
16241	0	0	1	34	Anastasiapavlyuchenkova	1991
16242	0	0	1	35	Corinnadentoni	1989
16243	0	0	1	36	Saraerrani	1987
16244	0	0	1	37	Florenciamolinero	1988
16245	0	0	1	38	Kristinaantoniychuk	1990
16246	0	0	1	39	Elizabethplotkin	1988

16247	0	0	1	40	Laurenalbanese	1989
16248	0	0	1	41	Anastasiapivovarova	1990
16249	0	0	1	42	Marritboonstra	1988
16250	0	0	1	43	Urszularadwanska	1990
16251	0	0	1	44	Lisetbrito-herrera	1988
16252	0	0	1	45	Irenapavlovic	1988
16253	0	0	1	46	Erikasema	1988
16254	0	0	1	47	Alexandrapanova	1989
16255	0	0	1	48	Mayagaverova	1989
16256	0	0	0	49	Olivialukaszewicz	1988
16257	0	0	0	50	Ellahnze	1988
16258	0	0	0	51	Stephanieherz	1990
16259	0	0	0	52	Magdalenarybarikova	1988
16260	0	0	0	53	Ana-Claraduarte	1989
16261	0	0	0	54	Dominiceripoll	1989
16262	0	0	0	55	Jadecurtis	1990
16263	0	0	0	56	Lindsayburdette	1988
16264	0	0	0	57	Bianca-Ioanabonifate	1988
16265	0	0	0	58	Polonarebersak	1987
16266	0	0	0	59	Michaelajohansson	1988
16267	0	0	0	60	Jessicasweeting	1988
16268	0	0	0	61	Fernandahermenegildo	1988
16269	0	0	0	62	Eugeniagrebenyuk	1988
16270	0	0	0	63	Sabinelisicki	1989
16271	0	0	0	64	Agnesszatmari	1987
16272	0	0	0	65	Maria-Fernandaalvarez-teran	1989
16273	0	0	0	66	Yvettehyndman	1988
16274	0	0	0	67	Ekaterinakosminskaya	1988
16275	0	0	0	68	Veronica Ruo Qili	1989
16276	0	0	0	69	Barborahodinarova	1989
16277	0	0	0	70	Jenniferstevens	1988
16278	0	0	0	71	Ashleyweinhold	1989
16279	0	0	0	72	Kimberlycouts	1989
16280	0	0	0	73	Larafakhoury	1988
16281	0	0	0	74	Shaynamcdowell	1989
16282	0	0	0	75	Vesnadolonc	1989
16283	0	0	0	76	Yasminschnack	1988
16284	0	0	0	77	Magyaziz	1987
16285	0	0	0	78	Andrearemynse	1988
16286	0	0	0	79	Jelenadurisic	1989
16287	0	0	0	80	Saorikarikomi	1988
16288	0	0	0	81	Ekaterinashulaeva	1987
16289	0	0	0	82	Elizabethkobak	1988
16290	1	1	1	1	Michaellakrajicek	1989
16291	1	1	1	2	Katerynavolodko	1986
16292	1	1	1	3	Shaharpeer	1987
16293	1	1	1	4	Victoriaazarenka	1989
16294	1	1	1	5	Latishachan	1989
16295	1	1	1	6	Monicaniculescu	1987
16296	1	1	1	7	Marinaerakovic	1988

16297	1	1	1	8	Olgagovortsova	1988
16298	1	1	1	9	Irinakotkina	1986
16299	1	1	1	10	Wen-Hsinhsu	1988
16300	0	1	1	11	Juliacohen	1989
16301	0	1	1	12	Agnesszavay	1988
16302	0	1	1	13	Timeabacsinszky	1989
16303	0	1	1	14	Dominikacibulkova	1989
16304	0	1	1	15	Aleksandrawozniak	1987
16305	0	1	1	16	Carolinewozniacki	1990
16306	0	1	1	17	Madalinagojnea	1987
16307	0	1	1	18	Allakudryavtseva	1987
16308	0	1	1	19	Alisakleybanova	1989
16309	0	1	1	20	Pichittrathongdach	1987
16310	0	1	1	21	Reginakulikova	1989
16311	0	1	1	22	Evgeniyarodina	1989
16312	0	1	1	23	Jennifer-Leeheinser	1987
16313	0	1	1	24	Nikolafrankova	1988
16314	0	0	1	25	Verdianaverardi	1987
16315	0	0	1	26	Magdalenarybarikova	1988
16316	0	0	1	27	Yaroslavashvedova	1987
16317	0	0	1	28	Bibianeschoofs	1988
16318	0	0	1	29	Sheng-Nansun	1987
16319	0	0	1	30	Maria-Fernandaalvarez-teran	1989
16320	0	0	1	31	Agnesszatmari	1987
16321	0	0	1	32	Ayumimorita	1990
16322	0	0	1	33	Elizabethplotkin	1988
16323	0	0	1	34	Angeliquekerber	1988
16324	0	0	1	35	Eugeniagrebenyuk	1988
16325	0	0	1	36	Florenciamolinero	1988
16326	0	0	1	37	Estefaniabalda-alvarez	1987
16327	0	0	1	38	Masazec-peskiric	1987
16328	0	0	1	39	Tatjanamaria	1987
16329	0	0	1	40	Anajerman	1986
16330	0	0	1	41	Martalesniak	1988
16331	0	0	1	42	Yasminschnack	1988
16332	0	0	1	43	Giuliagabba	1987
16333	0	0	1	44	Vojislavalukic	1987
16334	0	0	1	45	Michaelajohansson	1988
16335	0	0	1	46	Telianapereira	1988
16336	0	0	1	47	Marygambale	1988
16337	0	0	1	48	Katerinakramperova	1988
16338	0	0	0	49	Vanessapinto	1987
16339	0	0	0	50	Irenapavlovic	1988
16340	0	0	0	51	Krystinamarcio	1988
16341	0	0	0	52	Maraikebiglmaier	1987
16342	0	0	0	53	Veronikachvojkova	1987
16343	0	0	0	54	Teodoramircic	1988
16344	0	0	0	55	Magdalenakiszczynska	1988
16345	0	0	0	56	Erikasema	1988
16346	0	0	0	57	Sharonfichman	1990

16347	0	0	0	58	Ekaterinakosminskaya	1988
16348	0	0	0	59	Vaniaking	1989
16349	0	0	0	60	Natalietanevska	1987
16350	0	0	0	61	Nicolethyssen	1988
16351	0	0	0	62	Roxanevaisemberg	1989
16352	0	0	0	63	Valerietetreault	1988
16353	0	0	0	64	Ekaterinamakarova	1988
16354	0	0	0	65	Taraiyer	1988
16355	0	0	0	66	Lisetbrito-herrera	1988
16356	0	0	0	67	Daniellajeflea	1987
16357	0	0	0	68	Stephaniedubois	1986
16358	0	0	0	69	Ekaterinashulaeva	1987
16359	0	0	0	70	Janajuricova	1987
16360	0	0	0	71	Shaynamcdowell	1989
16361	0	0	0	72	Sanjaancic	1988
16362	0	0	0	73	Melaniegloria	1987
16363	0	0	0	74	Betinajozami	1988
16364	0	0	0	75	Amandaavedissian	1987
16365	0	0	0	76	Elizabethkobak	1988
16366	0	0	0	77	Michellebrycki	1989
16367	0	0	0	78	Lindsayburdette	1988
16368	0	0	0	79	Stephaniewetmore	1988
16369	0	0	0	80	Annatatishvili	1990
16370	0	0	0	81	Joannamatuszczyk	1989
16371	0	0	0	82	Larafakhoury	1988
16372	0	0	0	83	Katrinatsang	1988
16373	0	0	0	84	Ellahnze	1988
16374	0	0	0	85	Fernandahermenegildo	1988
16375	0	0	0	86	Giraschofield	1986
16376	0	0	0	87	Katarinazoricic	1987
16377	0	0	0	88	Anna-Mariazubori	1986
16378	0	0	0	89	Wan-Tingliu	1989
16379	0	0	0	90	Christystriplin	1987
16380	0	0	0	91	Carlatamborini	1987
16381	0	0	0	92	Yvettehyndman	1988
16382	0	0	0	93	Lenalitvak	1988
16383	0	0	0	94	Liegevieira	1988
16384	0	0	0	95	Angelinagabueva	1988
16385	0	0	0	96	Jenniferelie	1986
16386	1	1	1	1	Nicolaibudkov kjaer	2006
16387	1	1	1	2	Meesrottgering	2007
16388	1	1	1	3	Kaylanbigun	2006
16389	1	1	1	4	Rafaeljodar	2006
16390	1	1	1	5	Reisakamoto	2006
16391	1	1	1	6	Maximmrva	2007
16392	1	1	1	7	Lucapreda	2006
16393	1	1	1	8	Charlierobertson	2006
16394	1	1	1	9	Jankumstat	2007
16395	1	1	1	10	Hoyoungroh	2006
16396	1	1	1	11	Migueltobon	2006

16397	0	1	1	12	Thomasfaurel	2006
16398	0	1	1	13	Haydenjones	2006
16399	0	1	1	14	Amiromarkhanov	2007
16400	0	1	1	15	Naoyahonda	2006
16401	0	1	1	16	Andressantamarta roig	2007
16402	0	1	1	17	Jangjunkim	2006
16403	0	1	1	18	Theopapamalamis	2006
16404	0	1	1	19	Jackkennedy	2008
16405	0	1	1	20	Nathantrouve	2006
16406	0	1	1	21	Jaggerleach	2007
16407	0	1	1	22	Oliverbonding	2007
16408	0	1	1	23	Petrbrunclik	2006
16409	0	1	1	24	Cooperwoestendick	2006
16410	0	1	1	25	Oskaripaldanius	2007
16411	0	1	1	26	Henrybernet	2007
16412	0	1	1	27	Markomaksimovic	2006
16413	0	1	1	28	Ianmayew	2006
16414	0	0	1	29	Redabennani	2007
16415	0	0	1	30	Timofeiderepasko	2007
16416	0	0	1	31	Maxwellexsted	2007
16417	0	0	1	32	Flynnthomas	2008
16418	0	0	1	33	Maxschoenhaus	2007
16419	0	0	1	34	Andreade marchi	2007
16420	0	0	1	35	Janklimas	2006
16421	0	0	1	36	Moisekouame	2009
16422	0	0	1	37	Williamrejchtman vinciguerra	2007
16423	0	0	1	38	Atakankarahan	2006
16424	0	0	1	39	Ognjenmilic	2007
16425	0	0	1	40	Alexanderrazeghi	2006
16426	0	0	1	41	Alexandervasilev	2007
16427	0	0	1	42	Benjaminwillwerth	2007
16428	0	0	1	43	Dominickmosejczuk	2007
16429	0	0	1	44	Andreastimini	2006
16430	0	0	1	45	Gustavo Ribeirode almeida	2006
16431	0	0	1	46	Izanalmazan valiente	2006
16432	0	0	1	47	Valentingaray	2007
16433	0	0	1	48	Diegodedura-palomero	2008
16434	0	0	1	49	Maximozeitune	2006
16435	0	0	1	50	Mikapetkovic	2006
16436	0	0	1	51	Noahjohnston	2007
16437	0	0	1	52	Stefan Horiahaita	2007
16438	0	0	1	53	Nielsmcdonald	2008
16439	0	0	1	54	Kaseschinnerer	2006
16440	0	0	1	55	Kriishtyagi	2007
16441	0	0	0	56	Danielerapagnetta	2006
16442	0	0	0	57	Lorenzoberaldo	2006
16443	0	0	0	58	Glibsekachov	2006
16444	0	0	0	59	Lorenzoangelini	2006
16445	0	0	0	60	Luis Gutomiguel	2009
16446	0	0	0	61	Denispetak	2006

16447	0	0	0	62	Daniilsarksian	2006
16448	0	0	0	63	Ludvighede	2007
16449	0	0	0	64	Karimbennani	2007
16450	0	0	0	65	Matissefarzam	2007
16451	0	0	0	66	Carlescordoba	2006
16452	0	0	0	67	Anasmazdrashki	2006
16453	0	0	0	68	Yeonsujeong	2007
16454	0	0	0	69	Nikitabilozertsev	2008
16455	0	0	0	70	Vojtechvales	2006
16456	0	0	0	71	Egorpleshivtsev	2007
16457	0	0	0	72	Benjamingusic wan	2007
16458	0	0	0	73	Maximusdussault	2007
16459	0	0	0	74	Alaatrifi	2007
16460	0	0	0	75	Dmitrybessonov	2006
16461	0	0	0	76	Sergioplanella hernandez	2007
16462	0	0	0	77	Viktorfrydrych	2006
16463	0	0	0	78	Justinengel	2007
16464	0	0	0	79	Joaquinguilleme	2006
16465	0	0	0	80	Tomsickenberger	2007
16466	0	0	0	81	Joao Vitorscramin do lago	2006
16467	0	0	0	82	Stilesbrockett	2006
16468	0	0	0	83	Rafaelbotran neutze	2006
16469	0	0	0	84	Donghyunhwang	2007
16470	0	0	0	85	Fuminjiang	2007
16471	0	0	0	86	Ronitkarki	2007
16472	0	0	0	87	Calvinbaierl	2007
16473	0	0	0	88	Matthewforbes	2006
16474	0	0	0	89	Danialrakhmatullayev	2006
16475	0	0	0	90	Keeganrice	2006
16476	0	0	0	91	Diego Eduardomunoz navia	2006
16477	0	0	0	92	Hughwinter	2006
16478	0	0	0	93	Tianhuizhang	2006
16479	0	0	0	94	Pablomartinez gomez	2006
16480	0	0	0	95	Enzokohlmann de freitas	2006
16481	0	0	0	96	Leonardocattaneo	2006
16482	0	0	0	97	Gabrielghetu	2007
16483	0	0	0	98	Pavlemarinkov	2006
16484	0	0	0	99	Maxstenzer	2006
16485	0	0	0	100	Brunokokot	2006
16486	0	0	0	101	Radu Davidturcanu	2006
16487	0	0	0	102	Jacobolar	2007
16488	0	0	0	103	Josephoyebog jr	2007
16489	0	0	0	104	Billchan	2006
16490	0	0	0	105	Rafaelsegado esteve	2006
16491	0	0	0	106	Luis Josenakamine	2006
16492	0	0	0	107	Bernardomunk mesa	2006
16493	0	0	0	108	Dillonbeckles	2006
16494	0	0	0	109	Karelfroemel	2006
16495	0	0	0	110	Makmikovic	2006

P10, P25, and P50 denote the number of players classified within the top 10%, 25%, and 50% perf
coded dichotomously, where a value of 1 indicates that the player achieved the respective percen
Data for the years 2020 and 2021 should be interpreted with caution due to substantial disruption
compressed ranking point distributions.
All data were obtained from publicly available records provided by the International Tennis Federa

004–2024).

Nation	Singles Played	Doubles Played	Total Singles Points
Brazil	10	7	2550
Mexico	10	9	2350
Austria	16	15	2310
Russia	9	8	1760
USA	7	7	1760
Great Britain	11	9	1880
Italy	14	12	1675
Bolivia	10	7	1800
USA	13	12	1740
Bulgaria	16	13	1730
China, P.R.	11	10	1670
Serbia	16	11	1650
Norway	22	18	1430
France	9	7	1485
Poland	13	11	1390
Italy	14	13	1190
Japan	15	14	1180
Estonia	12	12	1080
USA	22	16	1240
Sweden	21	17	1130
USA	16	12	1260
Russia	13	13	1180
Spain	12	10	1085
USA	14	12	1020
USA	22	14	1030
Czechia	16	15	920
Italy	17	16	740
Great Britain	17	16	1010
Czechia	18	15	800
Korea, Rep.	23	23	920
Czechia	17	15	920
France	25	25	890
Germany	21	19	940
Sweden	14	12	745
Morocco	17	16	1020
USA	16	15	850
China, P.R.	19	14	790
Australia	17	13	940
Czechia	15	14	720
Brazil	18	13	880
Bulgaria	13	12	1020
Netherlands	11	10	930
Australia	25	23	830
Italy	11	10	760
Italy	17	15	820
Netherlands	19	19	830

Great Britain	23	21	820
Russia	24	20	760
Switzerland	11	11	770
India	13	12	860
Italy	15	13	730
Great Britain	20	18	780
Armenia	23	20	730
France	16	13	840
France	21	17	750
Turkiye	23	22	680
Korea, Rep.	20	19	680
France	13	9	745
Belgium	9	7	840
Sweden	17	14	690
Netherlands	12	10	735
USA	17	14	590
Slovenia	16	16	580
Korea, Rep.	17	18	730
Germany	16	8	770
USA	23	21	571
Brazil	15	11	600
India	13	9	610
Sweden	16	14	660
Czechia	21	19	550
Estonia	16	12	690
Venezuela	18	12	690
Peru	23	19	580
Japan	10	9	660
Korea, Rep.	20	14	600
Brazil	29	21	591
Canada	15	12	585
Serbia	15	14	510
Italy	13	13	510
Argentina	21	20	520
Argentina	14	13	622
USA	18	14	520
Morocco	18	15	590
France	11	8	681
Spain	12	10	570
Argentina	24	18	550
Ukraine	20	16	501
USA	6	6	570
Spain	22	18	510
Russia	12	10	496
Australia	22	22	480
Romania	10	9	500
Brazil	22	16	496
Uzbekistan	14	12	480
Germany	10	10	370
Colombia	24	22	440

USA	13	10	510
Japan	15	12	455
Brazil	21	17	440
Canada	13	11	376
Canada	26	20	450
Ukraine	13	11	406
Serbia	12	11	436
Spain	21	19	422
Argentina	19	18	440
India	26	24	441
Spain	12	10	420
Thailand	21	17	360
USA	16	13	400
India	13	8	436
USA	19	15	441
Brazil	20	19	344
USA	17	13	362
Colombia	20	17	320
Italy	12	9	328
Argentina	10	6	300
USA	14	12	235
Australia	12	11	190
Japan	14	12	252
Japan	15	11	207
Denmark	18	14	184
Italy	10	8	195
Japan	21	17	70
Japan	12	8	78
Belgium	15	14	2440
Spain	14	9	2400
Korea, Rep.	18	16	2200
Paraguay	7	7	1930
USA	10	9	1700
Mexico	15	15	1760
Switzerland	10	9	1500
Russia	19	19	1280
Peru	10	9	1210
Argentina	13	13	1380
France	7	7	1410
Croatia	8	7	1330
Belgium	13	12	1190
Lithuania	10	9	1040
Czechia	7	6	1215
USA	14	14	950
Czechia	9	10	1160
Hong Kong, China	8	8	955
France	19	19	1160
Slovenia	12	12	1160
France	16	13	980
Czechia	13	12	1070

Poland	9	3	1180
Bolivia	16	14	950
USA	9	9	990
France	9	7	1080
South Africa	13	13	1010
Peru	12	11	780
USA	8	7	820
Japan	21	19	990
Japan	20	16	920
Slovakia	16	16	870
France	19	18	930
USA	8	8	810
Russia	17	16	970
Slovakia	13	11	1000
Spain	10	10	890
USA	20	18	880
Brazil	13	11	850
New Zealand	19	16	830
Australia	19	17	930
Canada	13	12	690
Croatia	5	5	670
Sweden	25	21	790
USA	12	10	700
Switzerland	12	12	670
USA	7	7	850
Switzerland	11	10	700
Spain	18	17	780
Paraguay	12	13	700
USA	15	13	500
France	14	11	710
Australia	12	11	730
USA	23	21	700
USA	13	11	700
Czechia	14	8	716
France	13	12	680
USA	22	14	690
Mexico	20	17	670
Czechia	17	15	640
Serbia	21	22	590
USA	18	15	620
Serbia	18	15	560
USA	17	16	621
Japan	15	12	600
Italy	15	12	530
Korea, Rep.	19	19	610
Thailand	12	11	346
Turkiye	15	15	530
Switzerland	25	22	440
Australia	17	15	510
Germany	20	20	580

Sweden	21	16	520
Ukraine	24	19	441
Cyprus	15	14	505
Japan	19	14	472
Namibia	14	14	540
Chile	21	15	520
Germany	27	24	510
USA	17	17	465
Bulgaria	11	8	516
USA	16	14	530
Great Britain	22	20	496
Japan	6	4	480
USA	21	19	430
Italy	12	9	526
Canada	23	20	400
Japan	25	20	401
Turkiye	11	10	370
Portugal	20	18	355
Great Britain	10	11	416
Belgium	15	13	370
USA	15	14	420
Great Britain	17	14	450
Thailand	23	21	426
Poland	12	11	400
Kazakhstan	18	14	381
France	21	16	406
Poland	9	8	400
Netherlands	18	15	368
Croatia	15	9	256
Germany	8	7	295
Italy	12	11	396
Chile	24	15	350
USA	23	19	313
Italy	14	9	337
Argentina	10	10	286
USA	15	13	320
Lithuania	6	5	250
Japan	22	20	226
North Macedonia	6	4	230
Great Britain	10	9	122
Italy	12	8	126
Korea, Rep.	8	5	110
China, P.R.	10	9	2590
Spain	8	7	2210
USA	11	10	1920
USA	12	12	1620
Ukraine	12	10	1440
USA	13	11	1430
Croatia	17	17	1390
Paraguay	13	13	1410

France	13	12	1330
Switzerland	8	6	1370
Czechia	10	10	1360
France	6	6	1360
Estonia	11	10	1280
Peru	15	15	1120
Sweden	18	16	1220
Peru	14	14	1030
Spain	15	15	880
France	12	9	1140
Lithuania	20	20	680
Great Britain	7	5	1040
France	4	3	960
Australia	16	15	990
Poland	18	14	900
USA	12	12	820
Brazil	6	6	1020
France	3	3	890
Spain	11	11	960
Mexico	19	17	950
North Macedonia	18	17	960
USA	9	8	790
Switzerland	17	16	970
Bulgaria	12	12	710
Hong Kong, China	10	9	700
Lithuania	23	22	720
USA	14	12	780
Slovakia	18	16	680
Croatia	12	8	910
USA	10	10	680
Ecuador	12	11	700
Israel	16	15	770
Slovenia	23	21	700
Argentina	11	9	750
France	6	5	790
Poland	12	11	650
Poland	10	9	720
Belarus	13	12	656
Slovenia	19	19	755
Belarus	12	12	670
USA	12	12	760
Korea, Rep.	20	17	730
Russia	15	14	615
France	8	8	496
Jordan	10	10	425
Bolivia	18	17	700
Germany	8	8	620
Germany	14	13	570
Germany	16	13	700
Germany	16	12	582

Russia	17	17	560
Belgium	17	14	630
Czechia	16	15	516
Czechia	11	11	615
Brazil	14	13	540
Czechia	11	10	560
Russia	15	14	530
Spain	12	9	670
Namibia	10	10	620
Argentina	19	19	540
USA	10	9	420
Czechia	16	14	456
Poland	16	10	536
Uzbekistan	16	14	620
Morocco	12	12	522
Croatia	18	15	481
Morocco	10	10	626
Serbia	19	16	480
Belgium	5	3	626
Poland	22	20	542
Russia	14	12	446
Morocco	14	14	610
Canada	13	11	492
Korea, Rep.	15	14	470
Great Britain	8	8	490
USA	13	9	470
Italy	16	15	506
Belgium	12	12	366
Ukraine	7	6	426
Belgium	15	14	480
Switzerland	12	11	492
Czechia	16	13	446
Spain	13	11	520
Colombia	8	7	556
Kazakhstan	24	24	504
Russia	15	12	496
USA	17	17	425
Cyprus	21	21	440
India	11	11	484
Israel	16	13	480
Portugal	12	12	470
France	11	11	485
France	15	12	470
Russia	15	15	413
Poland	16	12	456
Bulgaria	15	14	440
Mexico	18	18	425
USA	20	19	430
Germany	10	9	396
USA	21	16	440

Turkiye	17	16	400
Thailand	13	13	310
Argentina	12	8	360
France	17	12	337
Sweden	17	14	352
Germany	7	5	380
USA	18	18	385
Portugal	14	13	381
Sweden	10	6	480
Peru	15	15	380
Russia	16	13	392
Japan	17	13	456
Belgium	26	21	352
USA	22	17	249
Austria	16	14	276
USA	11	7	400
Great Britain	16	16	416
USA	22	20	377
France	15	12	446
Bulgaria	19	17	328
Switzerland	20	17	425
Peru	8	8	370
Ireland	14	11	368
Australia	5	5	410
Canada	18	18	328
Great Britain	16	10	342
Japan	15	11	352
Austria	26	24	352
Paraguay	17	15	306
Argentina	23	23	322
USA	6	5	345
Belgium	14	14	344
India	7	7	348
India	17	16	340
USA	24	20	312
USA	9	9	220
Bosnia and Herzegov	14	12	356
France	15	14	352
Switzerland	23	20	336
Serbia	16	13	362
Great Britain	15	13	358
Russia	16	13	306
Czechia	17	13	320
Serbia	14	13	362
Slovakia	15	14	288
Portugal	12	8	346
Italy	12	10	318
USA	7	7	300
USA	21	16	306
Great Britain	17	11	328

USA	11	9	280
Ukraine	13	12	262
Russia	15	11	350
USA	24	22	312
France	10	9	242
Poland	7	6	332
Colombia	9	9	316
Italy	6	6	336
Czechia	21	21	286
France	19	15	318
France	11	10	318
Cyprus	10	10	336
Australia	8	8	316
Great Britain	7	6	328
Belarus	8	7	332
Russia	16	16	322
Italy	18	14	237
Croatia	18	18	272
Colombia	17	17	248
USA	4	3	288
Italy	13	13	231
Canada	8	7	215
Poland	19	16	272
USA	23	21	191
Czechia	6	6	232
USA	19	18	300
Italy	15	13	254
Argentina	18	15	288
Italy	16	16	282
USA	19	16	252
Netherlands	18	16	256
Brazil	13	12	224
USA	15	12	285
Slovenia	16	13	282
USA	5	0	320
USA	19	12	268
Ukraine	13	12	214
Turkiye	17	16	262
Israel	16	16	226
Czechia	21	18	216
Romania	20	17	241
Argentina	23	21	258
South Africa	11	9	258
Mexico	22	22	241
USA	10	6	272
Lithuania	14	14	262
USA	16	15	212
Bulgaria	12	7	268
Romania	16	14	166
Turkiye	7	5	245

Greece	18	17	230
France	15	12	232
Romania	12	10	250
Great Britain	18	15	236
Colombia	20	18	202
Japan	13	13	224
USA	5	4	240
Sweden	8	8	250
Australia	5	5	260
Serbia	14	11	202
Czechia	16	14	206
Russia	13	12	181
Serbia	18	16	208
Spain	4	4	200
Uzbekistan	16	14	224
Bulgaria	16	13	200
India	11	11	220
USA	17	14	182
India	14	13	226
Czechia	17	14	200
Argentina	22	21	201
Switzerland	23	20	206
Portugal	27	25	140
Tunisia	12	11	196
Bulgaria	12	12	226
Chile	18	17	196
El Salvador	14	14	212
Czechia	9	6	211
Germany	10	9	185
Italy	14	10	192
USA	10	7	160
Mexico	10	7	218
India	8	8	235
USA	19	16	214
Spain	12	8	216
Brazil	7	6	182
India	12	10	172
Israel	16	14	174
Austria	17	12	196
France	19	18	176
Estonia	19	14	208
Croatia	15	14	210
Great Britain	17	12	182
North Macedonia	13	13	168
Switzerland	14	10	216
Israel	11	10	170
Russia	21	17	190
Ukraine	13	12	192
France	19	13	152
Nigeria	8	8	204

Germany	11	11	224
Russia	20	18	154
New Zealand	8	6	201
USA	17	15	121
Serbia	9	8	132
USA	19	13	152
Chile	11	11	202
Slovakia	9	8	185
USA	12	11	168
France	16	12	178
China, P.R.	26	23	138
Belgium	23	18	174
Turkiye	18	16	176
Kazakhstan	16	15	166
Germany	18	17	132
Netherlands	13	10	199
Madagascar	5	5	162
Switzerland	15	11	188
Great Britain	17	17	172
Korea, Rep.	7	7	186
Serbia	18	15	182
USA	27	25	154
Japan	13	10	163
Chile	11	10	199
France	19	19	166
Russia	12	12	169
Hungary	14	14	160
Italy	11	8	179
Brazil	11	7	164
Japan	4	4	60
Austria	16	12	172
Egypt	7	6	192
Australia	10	10	166
USA	14	13	132
Chile	17	17	170
Great Britain	17	16	168
USA	13	11	151
Romania	12	11	180
Argentina	12	11	146
Czechia	22	19	148
Australia	10	10	154
Cyprus	10	9	158
Switzerland	7	7	152
Switzerland	10	8	186
USA	14	11	165
Poland	10	9	145
Japan	10	8	126
Spain	10	8	131
Great Britain	8	6	150
Greece	15	14	156

Egypt	18	12	146
USA	14	14	144
Poland	17	17	144
USA	15	15	146
France	8	8	161
Mexico	15	13	142
Ireland	13	12	180
Sweden	7	6	75
Thailand	6	6	142
Ecuador	10	10	154
Italy	10	7	181
Serbia	3	1	150
Ukraine	16	15	136
Bulgaria	12	11	120
Ukraine	8	6	167
USA	16	13	83
Germany	18	16	101
Switzerland	11	9	143
Japan	6	6	148
Spain	7	3	168
Italy	10	6	131
Poland	9	8	140
Venezuela	16	14	146
Malaysia	5	5	156
USA	4	4	130
South Africa	12	12	134
Mexico	17	15	140
Serbia	15	10	126
Brazil	12	8	123
Russia	14	13	128
Malaysia	4	4	159
Egypt	15	12	135
Great Britain	9	8	135
Portugal	11	9	135
Austria	12	12	142
Argentina	12	10	142
Italy	16	15	138
Uzbekistan	8	5	144
Togo	14	13	130
Denmark	5	3	165
Lithuania	14	13	86
Thailand	10	7	114
Indonesia	7	4	131
Kazakhstan	13	13	114
USA	22	15	120
Kazakhstan	19	18	99
Switzerland	14	9	114
Netherlands	18	16	122
Czechia	15	12	129
Morocco	15	11	104

Spain	15	10	93
France	9	8	141
Great Britain	20	18	134
New Zealand	3	3	132
Montenegro	16	14	138
Guatemala	17	17	109
Egypt	17	14	120
Great Britain	14	10	114
France	8	8	148
France	16	13	110
Peru	12	10	149
Tunisia	16	15	111
Russia	18	9	144
France	16	15	112
Turkiye	21	19	115
USA	16	13	118
Russia	18	13	108
Netherlands	16	11	130
Belgium	17	15	111
Slovakia	10	11	122
Italy	1	1	150
Great Britain	6	6	114
Canada	3	3	132
South Africa	18	17	94
Italy	8	6	100
Korea, Rep.	8	8	133
Canada	17	14	94
Czechia	13	12	122
Slovakia	16	12	121
Serbia	16	13	78
Belgium	17	15	93
Chile	10	10	100
Russia	15	11	100
USA	4	4	120
USA	5	5	118
Ireland	9	9	132
Great Britain	7	7	97
Kazakhstan	12	12	102
Bolivia	6	6	101
Slovenia	8	4	124
Germany	16	14	124
Mexico	9	8	118
Czechia	17	14	96
USA	21	14	115
Canada	11	10	119
India	14	14	115
Japan	6	6	112
Russia	15	14	102
Tunisia	11	11	74
USA	9	8	105

Korea, Rep.	4	4	92
Poland	18	15	69
India	10	10	87
France	19	16	96
Estonia	21	18	87
Hong Kong, China	4	4	113
Finland	12	12	108
China, P.R.	9	8	113
Sweden	17	13	66
France	22	21	68
Russia	4	3	120
Czechia	20	18	55
Italy	1	1	90
India	8	4	100
Romania	11	8	88
Australia	15	13	79
Japan	4	4	110
Japan	9	8	110
Switzerland	25	19	78
Iran	4	4	96
Malaysia	15	14	66
Canada	11	11	91
Mexico	13	8	114
Germany	14	11	104
Japan	7	7	85
Great Britain	14	11	85
Cyprus	12	11	75
Egypt	7	4	93
Belgium	13	13	71
Great Britain	9	9	81
Brazil	5	4	110
Chile	14	13	99
Netherlands	16	14	94
France	12	7	109
Spain	12	12	85
Finland	14	13	99
USA	10	9	99
Armenia	17	14	86
Russia	14	10	105
Ukraine	19	18	86
Lebanon	16	12	95
Spain	12	10	100
Iran	10	6	104
Thailand	8	8	103
Ukraine	15	13	75
Thailand	5	5	61
Turkiye	11	11	82
Latvia	22	12	99
Slovenia	20	15	63
Greece	10	9	94

China, P.R.	22	19	84
Greece	5	2	108
Costa Rica	9	9	79
South Africa	14	11	88
France	20	13	52
Bolivia	11	8	88
Brazil	9	8	95
Romania	8	6	85
Belgium	23	19	63
South Africa	8	6	104
Ecuador	11	11	99
Great Britain	8	6	79
Spain	18	11	84
Ukraine	11	10	84
India	19	16	95
Korea, Rep.	7	7	76
Portugal	17	15	84
Germany	16	11	85
Slovenia	17	15	63
Japan	7	6	94
Mexico	10	10	88
Poland	9	9	79
Korea, Rep.	7	7	78
Israel	14	10	97
New Zealand	6	5	72
Turkiye	13	10	87
Slovakia	15	13	96
Uzbekistan	18	18	67
Australia	6	6	80
Russia	15	12	72
France	16	13	80
Mexico	10	8	68
USA	11	9	72
USA	2	2	90
USA	9	4	75
Colombia	4	4	70
Colombia	7	7	70
Thailand	7	7	71
Venezuela	14	12	85
Kazakhstan	12	11	73
Argentina	7	6	96
Belgium	7	7	75
Romania	10	9	86
Italy	9	8	91
Uzbekistan	14	14	75
Australia	3	4	78
Germany	16	13	60
Singapore	20	20	60
Kazakhstan	9	9	75
Poland	10	8	75

Korea, Rep.	4	4	73
Great Britain	16	16	47
Great Britain	13	11	77
Sweden	14	13	88
Israel	14	13	81
Ecuador	10	10	72
Thailand	12	10	56
France	15	14	67
Latvia	14	14	53
Japan	12	13	87
Austria	4	2	92
Germany	9	5	65
Ukraine	14	10	64
Canada	6	6	69
Latvia	16	12	66
Azerbaijan	10	4	92
USA	11	7	79
Peru	7	6	74
Italy	14	13	44
Brazil	9	5	75
Czechia	19	18	66
Slovenia	11	9	71
Germany	7	4	71
Russia	11	7	86
Slovenia	18	17	65
Great Britain	9	9	57
Denmark	4	3	88
Chile	8	6	71
Croatia	9	7	73
Canada	23	18	55
Belarus	12	11	62
Chile	12	12	59
Poland	11	7	80
Great Britain	19	17	61
Venezuela	9	5	85
Uzbekistan	7	6	87
Australia	7	7	45
Belarus	17	15	45
Bulgaria	9	8	82
India	8	8	40
Sweden	11	7	69
Great Britain	15	14	73
USA	12	9	30
USA	14	12	56
Ukraine	9	7	83
Israel	12	11	53
Nigeria	10	10	62
Romania	13	11	78
Kosovo	9	8	47
France	18	16	68

Great Britain	11	7	73
Belarus	19	16	64
Portugal	10	8	65
France	18	17	60
India	9	9	70
Switzerland	6	5	82
Chile	17	12	63
Great Britain	19	16	48
Senegal	9	9	80
Belgium	20	19	62
Romania	15	13	65
Great Britain	12	9	69
Slovakia	12	10	42
Japan	12	12	60
Portugal	10	9	63
Great Britain	18	13	49
Serbia	17	13	44
Japan	4	4	63
Botswana	10	8	78
India	4	3	79
Greece	7	5	60
Morocco	6	4	81
Netherlands	17	15	61
Romania	7	7	55
Spain	20	15	65
China, P.R.	7	6	71
Mexico	11	11	56
Ukraine	10	6	80
Portugal	4	4	60
Czechia	17	11	49
Sweden	8	4	74
Japan	8	6	54
USA	8	7	66
USA	6	6	51
Egypt	17	14	60
Spain	11	9	59
Poland	9	7	71
Denmark	10	7	56
USA	14	12	61
USA	15	11	65
Australia	4	4	61
Canada	15	8	60
USA	15	10	57
Colombia	7	7	52
Russia	9	8	51
Turkiye	9	7	49
Mexico	8	8	60
Zimbabwe	17	15	66
Spain	12	11	73
Cote D'Ivoire	5	4	76

Germany	14	13	38
Peru	10	10	58
Mexico	10	9	63
Portugal	5	4	74
Great Britain	13	12	52
Cyprus	13	13	45
Switzerland	12	10	37
Spain	8	5	70
Latvia	11	10	65
Benin	7	6	58
Estonia	9	7	58
Spain	9	5	73
Ghana	13	12	39
Canada	6	6	58
Finland	15	15	37
France	10	6	64
Bulgaria	12	7	74
USA	6	4	68
Thailand	6	6	45
Czechia	12	11	57
Canada	4	4	70
Spain	11	10	66
Turkiye	16	16	49
Spain	10	2	70
Hungary	12	7	71
Spain	12	10	67
Poland	7	5	44
Poland	7	6	62
Sweden	6	5	51
Great Britain	13	12	50
France	7	5	73
Egypt	5	5	20
Brazil	12	12	57
Korea, Rep.	15	15	58
Great Britain	10	8	40
Germany	20	14	57
Montenegro	7	6	60
Kazakhstan	15	12	58
Italy	8	4	71
Singapore	8	8	61
USA	4	4	63
Sweden	15	8	63
Kazakhstan	7	7	23
Australia	2	2	70
Romania	10	9	61
USA	6	6	60
Canada	13	9	63
Israel	16	15	58
Thailand	6	6	51
France	20	19	42

Zimbabwe	8	8	53
Mexico	14	13	42
Germany	11	1	70
Russia	10	8	48
Serbia	13	12	31
USA	11	10	53
Canada	6	6	55
Italy	19	17	50
Kazakhstan	14	14	57
South Africa	8	8	42
Italy	5	3	66
Slovenia	10	9	61
Great Britain	10	9	39
Mexico	16	11	40
USA	19	19	35
Switzerland	1	1	30
USA	10	9	27
Australia	8	8	43
India	12	12	45
France	8	9	43
France	14	14	44
Russia	10	10	62
Greece	17	13	44
Chile	6	4	50
India	8	7	46
Japan	9	9	35
USA	11	8	50
Belarus	10	9	24
Russia	5	3	55
Sweden	17	13	34
Brazil	14	7	60
USA	3	3	30
Australia	2	2	30
Russia	13	12	45
Australia	9	7	48
Brazil	14	12	51
India	10	8	55
Czechia	5	4	38
Ecuador	11	11	56
Venezuela	7	7	52
Kazakhstan	12	11	46
USA	9	9	60
Lithuania	20	16	39
Sweden	9	2	66
Colombia	9	9	29
Ukraine	10	9	45
New Zealand	6	6	45
Austria	24	10	52
Serbia	13	10	54
Spain	12	10	51

Hungary	15	11	63
Qatar	12	10	45
Kenya	15	13	27
Czechia	6	4	58
Kazakhstan	12	7	48
Australia	5	5	55
Costa Rica	20	19	35
India	4	1	60
Sweden	4	0	63
Portugal	14	12	48
Japan	4	4	35
India	5	5	46
Sweden	14	11	39
Belgium	14	11	51
Switzerland	9	7	35
USA	15	13	52
Great Britain	12	9	47
Hong Kong, China	4	4	53
Kazakhstan	15	13	34
Denmark	10	10	47
South Africa	14	13	44
Israel	8	7	20
Japan	13	13	20
Kazakhstan	17	15	33
Slovakia	7	3	55
Sweden	14	14	57
Spain	3	0	60
Norway	7	7	49
Colombia	13	13	43
El Salvador	15	15	39
Guadeloupe	12	7	52
Russia	9	3	55
Belarus	6	5	32
France	17	15	30
Slovenia	9	9	43
USA	8	8	35
Turkiye	15	14	52
Spain	15	7	49
Bosnia and Herzegov	14	8	50
South Africa	10	9	37
Turkiye	16	15	33
Bulgaria	9	3	40
Russia	7	6	47
Israel	16	14	43
France	9	9	30
Korea, Rep.	5	5	53
Argentina	9	8	40
Sweden	14	10	32
USA	17	14	47
Turkiye	10	7	47

Hong Kong, China	5	5	40
Chile	10	10	43
India	6	6	51
Australia	2	1	56
Great Britain	10	10	37
Russia	13	11	17
India	10	10	44
Venezuela	4	4	45
Brazil	12	12	33
Cyprus	13	11	48
Argentina	3	2	28
Germany	6	5	27
South Africa	18	16	33
Italy	13	8	36
Serbia	4	4	45
Australia	1	1	36
Great Britain	11	9	47
Sweden	8	8	40
Austria	15	10	50
Netherlands	11	7	34
Japan	3	2	46
Brazil	7	6	48
Slovakia	13	11	28
Canada	4	3	42
Great Britain	9	6	48
Guatemala	14	14	39
Georgia	4	1	54
Jamaica	4	4	40
Egypt	22	19	40
USA	10	8	43
Spain	8	7	39
India	7	7	45
France	16	15	41
Portugal	11	9	32
Slovenia	18	14	14
Japan	8	8	48
India	9	9	43
Slovenia	21	19	28
Germany	6	6	37
Russia	14	10	41
Mexico	14	12	42
Switzerland	9	9	35
Latvia	10	8	38
Mexico	6	6	14
Austria	18	15	36
India	11	11	45
France	6	5	48
Russia	12	11	22
Romania	7	6	23
USA	2	2	48

Portugal	3	3	30
Russia	15	12	31
Great Britain	10	9	35
France	13	13	28
South Africa	10	8	52
Nepal	14	13	35
Poland	9	11	46
USA	16	15	35
Iran	3	3	42
India	6	6	41
Iran	8	7	38
Bulgaria	4	4	27
South Africa	9	6	42
USA	2	2	28
USA	3	1	39
USA	4	2	50
Spain	6	4	37
Bosnia and Herzegov	10	7	45
Lithuania	13	11	31
Ecuador	7	7	45
Romania	14	10	36
Korea, Rep.	6	6	31
Russia	16	14	37
Romania	11	11	27
Turkiye	13	12	24
India	11	10	24
Japan	11	9	40
Bulgaria	11	8	25
Brazil	4	4	34
Thailand	7	3	39
France	11	8	39
Serbia	8	6	46
Venezuela	2	2	36
Hong Kong, China	14	13	24
Greece	3	2	41
Nigeria	5	4	34
Sweden	5	5	38
France	14	10	37
Czechia	9	6	30
Czechia	9	9	12
Kenya	9	8	27
Russia	10	8	32
Austria	16	12	32
New Zealand	4	4	34
Malta	9	9	35
India	8	7	37
Korea, Rep.	6	6	38
Hong Kong, China	5	5	44
Dominican Republic	10	10	30
Spain	8	5	26

Netherlands	8	7	30
Colombia	4	4	42
Spain	6	6	45
Brazil	5	2	42
Canada	7	6	39
Czechia	5	4	24
Great Britain	4	4	43
Great Britain	9	9	25
Australia	3	3	29
Ukraine	11	8	40
Bolivia	7	7	28
Great Britain	8	5	41
Germany	6	4	40
Denmark	12	12	35
USA	2	2	36
Poland	8	8	25
Ecuador	9	8	30
Nigeria	6	5	39
Malaysia	24	17	24
Poland	11	3	45
Brazil	14	15	29
Latvia	9	3	37
Spain	3	3	37
Peru	7	7	36
Kazakhstan	14	11	38
Switzerland	14	13	30
New Zealand	6	4	25
Ecuador	10	7	40
Zimbabwe	13	11	22
Mexico	6	6	33
Great Britain	12	10	26
Turkiye	16	15	24
Colombia	15	14	19
Denmark	16	13	36
Slovakia	9	8	24
Poland	9	5	41
Italy	7	6	34
Belarus	5	3	40
Spain	15	12	34
France	6	5	43
Argentina	2	2	39
Russia	15	13	37
Slovenia	7	5	34
USA	13	12	31
Austria	8	7	25
Hong Kong, China	4	4	32
Romania	23	19	30
Peru	12	11	34
Australia	1	1	36
Turkiye	11	9	17

Greece	6	6	33
Brazil	10	7	38
Italy	6	4	30
Russia	4	4	36
France	18	15	17
Thailand	6	5	17
Russia	9	9	24
Serbia	10	10	14
USA	3	2	39
Iran	9	7	23
Great Britain	9	7	34
Brazil	11	10	32
Sweden	5	5	12
Brazil	3	2	38
Belarus	11	9	28
Montenegro	16	8	28
Japan	2	2	36
Denmark	8	6	30
Spain	4	2	41
Romania	8	7	35
Great Britain	7	4	37
Brazil	13	12	26
Egypt	7	6	35
Indonesia	3	3	20
Canada	4	4	30
Germany	10	7	26
Croatia	7	6	24
Japan	7	7	24
Australia	3	2	25
Germany	4	2	36
Israel	11	9	39
Argentina	6	6	34
Russia	7	5	33
Argentina	15	14	17
USA	1	2	0
Japan	10	9	35
Brazil	7	4	36
Russia	10	8	38
Austria	2	1	38
Poland	14	10	30
Russia	13	11	24
Bolivia	6	4	34
Germany	10	5	33
France	7	7	27
France	15	8	24
Korea, Rep.	7	6	17
Brazil	4	2	35
Venezuela	6	6	37
Romania	11	9	35
Colombia	14	14	21

Spain	8	5	35
USA	6	6	38
Turkiye	19	13	31
Australia	7	7	24
Ukraine	7	6	25
India	8	2	33
Japan	6	5	34
El Salvador	12	10	25
Austria	14	13	21
Canada	21	12	21
Czechia	5	4	27
Italy	9	9	12
Ghana	11	8	28
Netherlands	11	6	23
Mexico	12	7	29
Cyprus	9	8	31
Great Britain	4	4	28
Brazil	10	6	25
Czechia	7	7	24
Nicaragua	20	17	29
Montenegro	15	12	31
Mexico	16	12	12
Egypt	10	9	19
Poland	8	2	35
Spain	16	10	26
Israel	9	8	27
South Africa	4	4	29
France	11	7	20
Great Britain	1	1	30
Algeria	1	1	36
Poland	15	15	10
Argentina	1	0	36
Egypt	14	7	26
Thailand	6	3	35
USA	4	4	29
Netherlands	7	6	25
New Zealand	5	5	17
Italy	5	2	35
USA	6	5	30
Kazakhstan	10	6	32
Kazakhstan	12	12	13
Uzbekistan	12	6	34
Peru	16	13	29
Israel	14	13	25
Nigeria	12	12	24
Romania	11	7	30
Slovakia	18	10	19
Lithuania	16	14	15
Great Britain	9	9	28
Poland	9	5	22

Dominican Republic	6	6	20
Canada	4	3	20
India	15	14	21
Japan	6	5	12
France	6	3	35
Germany	12	10	29
Germany	6	5	15
USA	12	11	34
Costa Rica	12	10	28
New Zealand	3	3	28
Russia	12	11	34
China, P.R.	16	12	21
USA	8	7	19
Montenegro	11	11	33
Guatemala	17	15	24
USA	4	4	29
Kazakhstan	14	11	21
Czechia	9	6	32
India	8	7	30
Spain	11	9	12
Israel	18	13	25
Sweden	13	11	21
Korea, Rep.	4	4	33
China, P.R.	6	6	28
Netherlands	10	9	17
Italy	11	10	14
Poland	9	3	2
Paraguay	7	5	0
Italy	4	3	0
Singapore	5	5	19
Brazil	6	4	19
Venezuela	6	6	14
Estonia	13	10	5
Mexico	11	8	22
New Zealand	3	3	29
Brazil	15	14	15
Denmark	2	2	30
Bulgaria	6	5	25
USA	4	4	20
Belgium	9	7	28
Brazil	16	14	21
Belarus	15	11	16
Czechia	9	8	15
Colombia	17	13	26
Sweden	14	13	18
Serbia	15	11	7
Venezuela	7	7	26
Slovenia	12	8	5
Russia	18	14	20
New Zealand	3	3	28

France	11	8	25
Egypt	11	8	20
Russia	6	6	21
Ukraine	10	9	17
Argentina	7	6	27
Brazil	4	0	32
Russia	10	6	27
Korea, Rep.	2	1	32
Germany	6	4	23
Slovakia	8	7	26
USA	5	2	30
India	2	2	28
India	11	11	16
Czechia	2	1	30
USA	1	1	30
Uzbekistan	11	10	28
Spain	3	2	20
Australia	7	6	27
Belgium	8	7	27
USA	12	11	26
Indonesia	9	9	26
Australia	10	10	23
Nigeria	6	5	24
Belarus	7	5	12
Korea, Rep.	6	6	29
Italy	11	8	22
New Zealand	6	6	22
USA	4	4	23
Korea, Rep.	8	8	18
Czechia	10	6	23
Australia	3	3	24
USA	6	6	20
Canada	5	6	12
Dominican Republic	10	7	27
Spain	6	3	28
Italy	9	6	27
Bulgaria	10	7	20
Hungary	8	6	7
Paraguay	6	5	13
Mexico	6	5	30
USA	1	1	30
Norway	1	1	30
USA	1	1	30
USA	2	1	30
USA	1	0	30
Russia	6	2	30
Japan	9	9	19
USA	2	2	0
Austria	7	4	29
India	3	3	28

USA	7	6	20
Colombia	10	8	19
El Salvador	14	13	19
Russia	17	10	16
France	7	7	28
Brazil	10	6	22
Mexico	10	6	22
Singapore	4	4	23
Costa Rica	13	13	18
Brazil	13	11	12
Iran	5	5	25
Israel	19	17	21
Great Britain	5	5	17
Czechia	13	7	10
France	10	9	9
Netherlands	9	6	15
Czechia	11	9	18
Australia	9	7	27
Russia	8	6	26
Czechia	7	5	19
Tajikistan	4	4	9
Netherlands	4	3	23
Jordan	6	5	7
Czechia	6	6	7
USA	17	10	24
Canada	5	5	20
Montenegro	4	0	28
India	9	6	17
India	2	2	15
Russia	8	6	16
Kazakhstan	5	5	19
Russia	10	8	26
Colombia	7	6	16
Sweden	14	11	16
USA	11	9	16
Slovakia	15	14	15
Tunisia	11	11	6
Italy	9	8	21
Bulgaria	10	8	14
Brazil	7	5	9
Poland	11	8	7
Colombia	10	9	4
South Africa	7	7	19
Japan	13	13	16
France	3	2	27
Spain	4	1	20
Costa Rica	11	9	19
Italy	5	5	22
Romania	15	9	21
Germany	7	7	20

Zimbabwe	9	8	10
Israel	2	2	23
South Africa	2	2	23
Australia	2	2	23
Korea, Rep.	4	4	25
Italy	8	7	24
Canada	21	17	11
Switzerland	12	7	9
Australia	6	6	20
Egypt	17	17	17
USA	1	1	0
Germany	3	2	0
Russia	11	9	26
France	4	3	19
New Zealand	6	6	20
France	7	7	20
Italy	6	4	26
Belgium	9	7	21
Croatia	14	14	12
Nigeria	5	4	12
Greece	9	9	17
Brazil	7	5	12
Latvia	4	2	7
Belarus	7	7	21
Israel	10	8	15
Korea, Rep.	6	6	22
Korea, Rep.	6	5	10
Lebanon	10	8	17
Sweden	3	2	14
Italy	5	5	22
Czechia	15	13	13
Namibia	5	5	4
Canada	5	5	17
Mexico	15	9	20
Austria	3	2	25
France	4	3	25
Switzerland	11	9	18
Spain	6	6	15
Mexico	3	3	18
Mexico	3	1	18
Japan	3	3	23
New Zealand	1	1	18
Canada	9	7	17
Korea, Rep.	6	5	19
Korea, Rep.	7	7	14
Hong Kong, China	4	4	12
Colombia	5	4	12
Portugal	5	3	24
Czechia	11	8	19
Turkiye	16	11	16

Ghana	7	7	12
Hong Kong, China	5	4	23
Venezuela	3	3	19
Cameroon	5	3	18
China, P.R.	9	7	19
Colombia	7	7	14
Spain	8	7	12
Brazil	7	3	14
North Macedonia	8	8	16
Brazil	6	4	22
USA	10	6	19
Thailand	7	7	17
Great Britain	10	8	20
Italy	10	6	19
Singapore	9	8	21
France	13	11	16
Poland	11	7	10
Colombia	3	3	15
Singapore	6	6	17
Mexico	5	4	16
France	2	1	23
Paraguay	7	7	12
Australia	2	2	10
USA	2	2	20
Chile	9	6	20
Great Britain	5	3	22
Canada	8	7	18
Venezuela	5	5	18
Croatia	8	6	10
Bulgaria	12	7	16
Peru	7	5	14
Hungary	8	7	5
Venezuela	4	4	17
Mexico	6	6	23
Romania	9	8	19
Ecuador	10	10	12
Poland	15	6	16
Greece	6	3	23
France	12	10	19
Venezuela	7	7	19
Brazil	11	10	7
Czechia	6	5	18
USA	12	8	12
Slovenia	11	11	15
Hong Kong, China	4	4	17
Hong Kong, China	3	3	20
Kazakhstan	11	7	12
Russia	6	6	7
Russia	8	7	2
USA	4	2	0

New Zealand	3	3	19
Ukraine	9	5	19
France	11	8	7
Croatia	7	4	22
Australia	6	5	22
Australia	3	3	22
Croatia	9	7	10
USA	7	4	15
Peru	3	3	15
Venezuela	5	5	16
Australia	4	4	7
Australia	2	2	20
Germany	4	4	20
South Africa	18	17	18
Netherlands	7	6	12
Netherlands	12	9	12
Australia	6	6	12
Russia	26	17	18
Great Britain	7	7	19
Slovakia	4	3	19
Iran	5	5	15
Australia	6	5	10
Sweden	2	1	10
Croatia	4	4	10
France	7	6	10
Czechia	5	4	18
Canada	10	8	17
Russia	13	7	17
Portugal	10	9	14
Finland	5	4	16
Sweden	8	7	7
USA	3	2	9
Chile	8	6	4
Austria	5	1	20
Finland	6	6	11
Germany	15	9	7
Portugal	4	2	18
Germany	5	4	15
Thailand	6	5	17
Czechia	9	7	19
Iran	4	4	14
India	7	7	12
Poland	9	5	7
Argentina	3	2	18
Ukraine	4	4	17
France	6	4	17
Netherlands	4	4	15
Japan	2	2	15
USA	5	4	10
Nigeria	3	3	18

Japan	3	3	20
Russia	2	2	20
India	6	4	20
Japan	4	2	20
Singapore	5	3	20
Argentina	3	3	15
Bolivia	3	3	12
Egypt	8	6	10
Brazil	10	8	17
France	16	15	11
Japan	4	4	16
USA	5	4	2
Zimbabwe	8	6	11
Bosnia and Herzegov	9	7	19
France	10	6	14
Bolivia	3	3	14
Italy	4	3	19
Finland	10	6	16
Colombia	5	5	15
Australia	4	4	14
Israel	14	7	10
Finland	10	7	17
Pakistan	2	2	18
Venezuela	6	6	14
Kazakhstan	14	12	8
Venezuela	6	6	7
Romania	10	7	5
Poland	4	2	2
Italy	7	6	17
Uzbekistan	13	13	14
Russia	6	3	16
Japan	9	8	5
Czechia	8	8	13
India	12	10	6
Germany	3	0	19
Australia	3	3	12
USA	13	8	16
Russia	8	4	11
Venezuela	7	7	16
USA	14	14	15
Vietnam	2	1	18
Thailand	2	1	12
Hong Kong, China	1	1	18
Venezuela	7	7	15
Great Britain	7	5	13
Israel	10	7	7
Nepal	8	8	4
Chile	2	2	15
Israel	6	5	15
Argentina	2	2	15

Thailand	7	6	12
Brazil	7	6	9
Turkiye	8	6	14
USA	8	6	15
Bolivia	3	2	14
Ukraine	12	8	12
Botswana	7	5	7
Mexico	3	3	18
USA	5	5	14
Spain	1	1	18
New Zealand	3	3	12
Tunisia	6	6	12
North Macedonia	15	14	9
Nigeria	11	11	9
France	3	2	18
Denmark	1	0	18
Italy	1	1	18
USA	5	5	15
Egypt	8	6	14
Venezuela	7	7	11
Latvia	14	11	6
Hungary	1	2	0
Australia	2	2	0
Israel	13	6	17
Sweden	5	5	16
Romania	11	9	10
Serbia	4	4	17
Egypt	7	5	17
Canada	3	2	17
Hong Kong, China	4	3	16
Thailand	7	7	10
Spain	11	8	17
Ecuador	8	4	9
Finland	9	9	7
USA	7	4	5
South Africa	3	3	12
Romania	5	5	12
Ecuador	9	7	15
Singapore	4	4	10
USA	9	6	10
Korea, Rep.	6	5	14
Montenegro	4	2	14
Mexico	14	13	5
Canada	3	3	16
Brazil	11	9	16
Switzerland	5	4	16
Lithuania	10	6	11
New Zealand	6	6	7
Botswana	8	7	0
Latvia	13	7	14

Japan	9	9	12
Israel	3	3	10
eSwatini	7	4	10
Japan	4	4	7
Hungary	16	12	14
USA	4	2	11
Mexico	10	6	10
Germany	6	3	17
Egypt	13	11	12
Chile	2	2	10
Argentina	6	5	12
Brazil	8	4	7
Singapore	11	9	7
Kazakhstan	14	7	7
South Africa	4	2	10
Finland	9	8	4
Italy	1	1	0
Australia	4	4	15
India	6	3	12
USA	8	3	15
USA	3	2	14
Russia	19	13	11
Argentina	4	4	10
Denmark	9	7	12
Venezuela	6	5	11
Colombia	12	7	10
Jordan	17	13	5
Hungary	5	5	0
Russia	8	5	0
Spain	5	4	14
Slovakia	6	3	12
USA	8	5	12
Egypt	8	11	12
Canada	6	6	7
Argentina	1	1	5
Tunisia	8	6	5
Romania	9	7	5
Netherlands	9	8	4
Russia	6	5	16
Serbia	11	5	16
Canada	9	5	12
Japan	5	5	12
New Zealand	5	5	7
Spain	9	3	12
Colombia	3	3	12
Venezuela	5	5	9
Romania	10	8	8
Finland	10	9	7
Poland	7	4	6
Egypt	15	14	6

Sweden	5	4	15
India	6	6	12
Malta	23	20	9
New Zealand	3	3	7
Costa Rica	6	6	5
Czechia	3	3	15
Cameroon	3	2	11
Cyprus	8	6	14
Cote D'Ivoire	5	3	12
Korea, Rep.	4	4	12
Germany	6	4	10
France	2	2	10
Israel	15	9	9
USA	7	6	14
Ecuador	5	4	13
Romania	10	6	9
Sweden	11	7	7
Germany	13	11	9
Belarus	3	3	10
Argentina	2	2	5
Venezuela	4	4	9
Spain	11	7	9
Russia	11	9	2
Germany	3	1	15
Germany	3	0	15
Serbia	5	4	15
Argentina	2	2	15
Israel	11	5	15
Peru	6	6	10
Israel	9	4	9
India	12	11	9
Colombia	10	9	9
India	3	3	7
Australia	2	3	0
Romania	5	5	10
Portugal	10	8	14
Uzbekistan	8	6	14
Japan	6	6	9
Czechia	4	3	9
Great Britain	9	6	11
Serbia	18	10	10
Great Britain	14	9	9
Denmark	6	6	9
Mexico	9	7	7
Egypt	12	6	2
Chile	3	3	12
France	6	5	9
Turkiye	13	10	13
Italy	6	6	9
Australia	5	5	7

Switzerland	7	6	12
Canada	13	12	7
Spain	6	6	12
India	4	4	11
Korea, Rep.	2	2	11
Australia	6	6	9
Spain	7	6	14
Netherlands	9	8	10
Sweden	10	10	10
Russia	7	6	10
Finland	11	10	9
Thailand	6	6	7
Israel	6	5	5
Serbia	6	2	14
Norway	2	0	14
Morocco	2	2	11
Australia	3	2	12
Canada	5	5	12
Canada	2	2	10
Australia	4	4	10
Tunisia	6	4	10
Mexico	8	6	12
Mexico	5	1	10
Argentina	6	5	12
Hungary	9	6	12
Bulgaria	9	5	12
Thailand	7	4	12
Latvia	4	2	10
Colombia	11	10	9
Japan	6	5	7
Egypt	11	9	7
USA	7	6	10
Hong Kong, China	2	2	9
Belgium	8	6	9
Bolivia	6	5	7
Russia	15	11	6
Croatia	9	6	0
Canada	4	2	12
Poland	7	2	12
Israel	11	9	12
Thailand	6	3	12
Japan	3	3	10
China, P.R.	1	1	10
Hungary	7	4	10
Argentina	2	2	10
Thailand	1	2	10
Japan	7	6	12
Peru	10	9	12
Egypt	8	10	5
Great Britain	4	4	2

Slovenia	12	12	0
Japan	3	3	10
Bolivia	13	12	11
Brazil	8	4	5
Portugal	5	5	5
Italy	6	2	11
Ukraine	6	5	9
Zimbabwe	9	9	7
Sweden	2	1	7
Slovakia	6	5	9
Switzerland	7	5	5
Estonia	4	3	12
Zimbabwe	5	5	12
Tunisia	9	4	12
Russia	2	2	12
Great Britain	5	3	12
Cyprus	8	7	5
Hong Kong, China	4	4	7
France	6	5	5
Singapore	8	8	10
Israel	10	6	10
Bolivia	3	2	10
Switzerland	8	4	10
Panama	3	3	10
Germany	4	2	10
Denmark	8	7	10
Israel	9	4	10
Australia	6	6	11
Great Britain	8	3	7
Poland	9	6	7
Croatia	12	8	11
Kazakhstan	14	12	11
Russia	15	14	8
Slovakia	11	10	7
Mexico	9	7	4
Tunisia	9	7	0
France	3	3	10
Russia	4	4	9
Russia	8	5	9
Romania	9	7	9
Korea, Rep.	5	5	9
India	6	4	10
India	6	4	10
Russia	7	6	9
Sweden	4	3	5
Brazil	11	9	4
Switzerland	4	4	4
Australia	3	3	12
Latvia	8	0	12
Tunisia	5	4	12

Netherlands	7	5	12
Japan	2	2	7
Switzerland	3	2	7
Chile	7	5	7
Japan	2	2	5
Egypt	14	10	9
India	12	5	9
Venezuela	5	4	9
Russia	3	2	5
Belgium	5	4	4
Mexico	8	5	2
Australia	1	1	10
Germany	9	4	10
USA	1	1	10
Estonia	2	2	10
Thailand	4	2	10
Argentina	5	3	10
India	6	4	10
India	5	3	10
Spain	5	1	10
Canada	5	4	10
Bosnia and Herzegov	11	8	7
Great Britain	1	1	5
Hungary	3	1	5
Georgia	6	3	11
Italy	4	2	11
Ghana	7	4	11
Korea, Rep.	6	5	10
Israel	15	12	6
Thailand	7	6	10
USA	3	2	10
Hungary	8	4	10
Japan	3	3	9
Colombia	8	5	9
Great Britain	5	3	10
Mexico	14	9	6
Italy	5	5	2
Montenegro	6	5	9
Great Britain	15	11	9
Kazakhstan	15	7	9
Hong Kong, China	4	3	7
Belgium	8	8	7
Singapore	4	4	9
USA	2	1	5
Egypt	15	15	2
Canada	4	4	2
Bolivia	2	2	0
Germany	1	1	0
Hungary	1	1	0
South Africa	10	9	0

Hungary	1	1	0
USA	1	1	0
Poland	4	1	0
Tunisia	8	3	0
Russia	8	3	11
Great Britain	13	8	7
China, P.R.	8	6	7
Kazakhstan	12	6	8
New Zealand	4	4	7
Venezuela	2	2	4
Germany	6	6	0
Mexico	16	10	0
Australia	3	3	10
Paraguay	7	4	10
Greece	3	3	10
Great Britain	5	3	10
Italy	6	5	10
Sweden	3	1	10
Singapore	5	5	9
Sweden	5	4	5
Sweden	5	3	10
Australia	16	13	9
Nigeria	4	4	7
Italy	5	3	7
North Macedonia	8	6	7
Russia	8	6	6
Sweden	4	3	6
Egypt	9	6	4
Brazil	7	6	0
Japan	5	5	9
Japan	6	4	9
Korea, Rep.	4	4	7
Chile	4	3	7
Serbia	11	7	7
Uzbekistan	6	7	7
Czechia	5	2	9
Czechia	2	2	9
Sweden	1	1	9
Italy	5	3	9
Japan	1	1	9
Germany	5	2	4
USA	9	8	2
USA	9	8	2
Morocco	8	8	2
Egypt	12	9	2
Spain	2	2	7
Sweden	4	4	7
Greece	11	9	7
Denmark	10	10	7
Turkiye	11	8	7

Argentina	4	3	7
Hong Kong, China	4	3	7
India	4	4	5
Azerbaijan	7	6	8
Poland	8	4	7
Romania	13	8	4
Brazil	8	7	4
Canada	4	4	2
Canada	4	4	2
Australia	2	2	10
Ukraine	6	4	10
Spain	1	1	10
France	3	1	10
Ukraine	1	0	10
Kazakhstan	7	4	10
Egypt	5	2	10
Poland	1	0	10
Australia	1	0	10
China, P.R.	4	3	10
Hong Kong, China	1	0	10
USA	1	0	10
Thailand	5	3	10
Croatia	3	2	10
USA	1	1	10
Bulgaria	2	0	10
Poland	9	3	10
France	2	2	10
India	1	1	10
Czechia	2	1	10
USA	2	2	10
Argentina	2	2	10
Latvia	10	5	10
India	4	2	10
Portugal	3	3	10
France	1	1	10
USA	2	1	10
Colombia	2	2	10
Bolivia	3	2	10
Germany	9	5	5
Czechia	4	3	5
USA	3	3	5
South Africa	7	7	5
India	4	1	10
Brazil	7	2	10
South Africa	7	5	7
Spain	6	4	7
Chile	5	5	5
Sweden	3	3	5
USA	11	4	2
Bolivia	11	4	9

Turkiye	11	6	9
Bolivia	5	3	9
France	7	6	9
Germany	5	5	5
Serbia	4	2	9
USA	3	2	9
Nigeria	2	2	9
France	10	9	9
New Zealand	2	2	9
Finland	7	4	9
Spain	1	1	9
Spain	7	6	5
Brazil	14	12	4
Great Britain	3	3	0
USA	8	5	7
Ecuador	4	3	7
Singapore	5	5	7
Sweden	6	5	4
Tajikistan	7	6	2
Iran	5	5	7
Germany	4	4	5
South Africa	10	5	5
Cyprus	6	5	5
Korea, Rep.	2	2	7
Cameroon	2	2	7
USA	2	3	7
Bosnia and Herzegov	4	4	7
Romania	11	9	6
Finland	5	4	6
Poland	12	10	6
Finland	10	9	2
France	8	5	5
France	3	0	9
Cameroon	2	1	9
Japan	6	6	9
Germany	8	4	9
Belgium	5	4	9
Finland	4	1	9
France	1	1	9
Colombia	3	1	9
Czechia	3	2	9
Australia	3	3	9
Cameroon	2	1	9
Hong Kong, China	3	3	5
Ecuador	5	4	4
Egypt	4	3	2
Korea, Rep.	2	2	2
Austria	13	8	7
Romania	3	2	7
Serbia	5	3	7

India	6	3	7
India	5	4	7
Morocco	5	3	7
Israel	7	3	7
Belgium	4	3	5
Russia	10	7	4
Bolivia	5	4	2
Croatia	13	9	0
Mexico	9	6	7
Sweden	3	1	7
Ghana	6	5	7
Turkiye	5	5	7
USA	2	1	5
Egypt	5	3	5
Austria	12	5	5
Austria	4	2	5
Austria	3	1	5
Netherlands	3	3	5
Slovenia	10	7	5
USA	1	1	5
Great Britain	1	1	5
Brazil	3	2	5
Venezuela	6	5	7
Nigeria	2	2	7
Hong Kong, China	3	3	7
France	2	2	7
Japan	4	2	7
Turkiye	5	4	7
Italy	7	4	7
Russia	4	3	5
Australia	4	3	4
Ecuador	7	6	2
Slovakia	9	5	2
Switzerland	3	3	2
Argentina	2	2	0
Ukraine	10	4	5
Colombia	4	4	5
Argentina	4	3	5
Peru	4	4	5
Korea, Rep.	6	6	5
Great Britain	6	2	5
Togo	7	5	5
China, P.R.	9	6	5
USA	5	3	6
Zimbabwe	7	6	6
Estonia	13	3	5
France	4	2	2
Germany	8	7	2
USA	2	1	2
France	12	10	4

USA	7	6	4
Korea, Rep.	6	6	2
Japan	7	7	2
Hong Kong, China	2	2	0
France	5	2	0
Estonia	2	3	0
Bosnia and Herzegov	10	5	7
Estonia	9	5	7
Benin	4	4	7
Canada	6	2	7
Italy	5	2	7
Venezuela	4	4	7
Italy	5	1	7
Egypt	3	2	7
Ukraine	4	1	7
Syria	7	5	7
Brazil	3	3	7
France	4	3	7
France	10	6	6
Turkiye	8	8	4
Australia	8	8	2
France	8	8	2
Sweden	5	3	2
Belarus	7	3	0
Netherlands	5	3	0
Chile	3	3	5
USA	4	4	5
New Zealand	3	3	5
Austria	9	3	6
Israel	10	8	6
Tunisia	9	6	6
Lebanon	6	4	5
Netherlands	7	4	5
Luxembourg	3	2	5
Monaco	5	5	2
Italy	8	6	2
Portugal	5	5	0
Poland	11	8	0
Japan	5	5	0
Ukraine	7	4	0
Chile	8	6	5
Turkiye	11	7	5
Turkiye	11	7	5
Poland	9	5	5
Egypt	12	6	5
Spain	7	2	5
Russia	7	3	5
USA	4	3	5
Australia	3	2	5
Estonia	5	2	4

Hong Kong, China	3	1	4
Australia	7	5	4
Mexico	15	9	4
Thailand	6	4	2
Turkiye	16	11	2
Serbia	10	8	2
Ecuador	5	1	7
New Zealand	2	2	7
France	4	2	7
South Africa	5	1	7
Brazil	6	3	7
Greece	5	1	7
Japan	7	4	7
Czechia	4	3	7
India	2	2	7
Egypt	7	3	7
Pakistan	2	2	7
Mexico	16	7	7
Hong Kong, China	3	2	7
Peru	5	1	7
Peru	5	3	7
Brazil	6	3	7
Germany	6	1	7
France	2	2	7
Finland	7	0	7
Belgium	7	4	7
Italy	7	2	7
Germany	3	3	7
Egypt	5	4	2
Brazil	2	2	2
Finland	12	7	2
Great Britain	3	3	2
Finland	5	5	0
Poland	5	3	0
Bahamas	2	2	0
France	5	4	0
Romania	2	1	5
Canada	8	8	5
Spain	5	2	5
Germany	8	2	5
France	6	5	5
Thailand	1	1	5
New Zealand	3	3	5
Canada	4	3	5
Greece	3	2	5
Chile	2	2	5
Germany	4	3	5
Estonia	5	3	5
USA	2	1	5
Chile	2	2	5

Canada	2	1	5
USA	1	1	5
Canada	6	3	5
Germany	5	5	5
Egypt	4	6	5
Mexico	10	8	5
Chile	1	1	5
Venezuela	3	1	5
USA	6	3	5
Australia	6	4	6
India	9	5	6
Brazil	10	6	6
Switzerland	10	7	6
Greece	17	12	6
Israel	9	7	5
Poland	8	1	5
Russia	11	7	2
Venezuela	5	5	2
South Africa	5	1	0
Mexico	7	4	0
Mexico	5	5	0
Egypt	4	2	0
USA	3	2	0
Egypt	4	2	0
Hong Kong, China	4	4	5
USA	4	4	5
Ghana	5	4	5
Bulgaria	8	5	5
Greece	5	5	5
Chile	3	3	5
Moldova	1	1	5
Switzerland	3	2	5
Nigeria	4	4	5
Australia	3	3	5
USA	4	4	5
Italy	1	1	5
Russia	8	4	5
Barbados	4	4	5
Italy	4	4	5
North Macedonia	4	3	5
Norway	4	2	5
Italy	2	1	5
Russia	8	4	5
USA	4	4	5
Italy	9	6	4
Switzerland	6	5	4
Egypt	11	8	4
Egypt	9	8	4
South Africa	4	4	4
Russia	15	9	4

France	7	5	4
Russia	7	6	2
Egypt	15	11	2
Ukraine	5	4	2
Colombia	11	10	4
Uzbekistan	11	6	4
USA	8	6	4
France	5	4	4
Venezuela	4	4	4
Morocco	4	4	4
Nicaragua	5	5	4
Russia	7	6	4
Czechia	5	2	4
Pakistan	4	4	4
India	10	7	2
India	3	2	2
Italy	8	2	0
Russia	7	1	0
USA	1	1	0
Hong Kong, China	1	1	0
France	3	2	0
South Africa	4	3	0
USA	4	4	6
Russia	15	11	6
Great Britain	9	8	2
USA	7	5	2
India	10	5	2
Zimbabwe	6	5	0
Czechia	8	3	0
USA	3	2	5
Egypt	7	7	5
USA	3	3	5
India	2	2	5
Togo	5	3	5
Switzerland	4	2	5
Japan	8	6	5
Qatar	4	3	5
USA	3	2	5
Canada	3	3	5
Benin	3	3	5
Italy	4	3	5
India	6	2	5
Netherlands	7	3	5
Spain	4	3	5
Hong Kong, China	4	2	5
Luxembourg	1	1	5
Croatia	5	1	5
USA	2	1	5
Serbia	4	2	5
Spain	5	3	5

USA	1	1	5
France	3	3	5
New Zealand	4	4	5
Slovakia	3	3	5
Argentina	3	4	5
Nigeria	7	7	4
Japan	4	4	4
Chile	4	4	4
Hong Kong, China	4	4	4
Israel	10	6	4
Spain	6	5	2
Great Britain	6	6	2
Singapore	4	3	4
Turkiye	6	3	4
Korea, Rep.	2	2	4
Australia	2	2	4
Belgium	7	6	4
Italy	3	3	4
USA	4	3	4
Kyrgyzstan	4	4	4
Poland	11	4	2
Canada	3	2	2
Australia	4	4	2
Italy	3	3	2
Israel	13	6	2
Australia	4	4	0
Austria	10	7	0
Korea, Rep.	6	6	0
Canada	5	5	2
France	3	2	2
Spain	2	1	2
Chile	10	6	2
Netherlands	7	4	2
Denmark	7	5	2
Uzbekistan	6	3	2
Australia	6	5	2
Argentina	4	4	2
Ukraine	6	2	2
Ukraine	3	2	0
Russia	9	7	0
Israel	9	8	0
New Zealand	2	2	0
New Zealand	2	2	0
Israel	9	8	0
Australia	7	4	0
Canada	2	2	5
Romania	3	1	5
France	7	2	5
Canada	2	2	5
Austria	2	0	5

Canada	1	1	5
Canada	3	3	5
Croatia	9	1	5
Germany	1	1	5
Indonesia	5	3	5
Singapore	3	1	5
Russia	2	1	5
Chile	1	1	5
New Zealand	2	2	5
Thailand	1	1	5
Sweden	5	1	5
South Africa	2	1	5
Canada	3	3	5
Singapore	5	3	5
Slovenia	8	5	5
Argentina	2	1	5
Zimbabwe	4	2	5
France	5	2	5
USA	1	0	5
Greece	3	2	5
Uzbekistan	3	2	5
Denmark	1	1	5
Argentina	2	2	5
South Africa	5	1	5
Germany	5	4	5
Korea, Rep.	5	4	5
India	3	1	5
Iran	4	0	5
Switzerland	3	2	5
Mexico	3	3	5
Argentina	2	1	5
Australia	11	8	5
Hong Kong, China	3	2	5
Peru	1	1	5
Great Britain	2	1	5
USA	3	1	5
Serbia	6	6	5
Japan	3	2	5
Spain	2	0	5
Egypt	6	6	5
USA	1	1	5
Germany	5	3	5
Hong Kong, China	2	0	5
Mexico	8	6	5
New Zealand	1	0	5
Germany	1	0	5
USA	1	1	5
Burkina Faso	2	2	5
Bolivia	2	2	5
India	3	0	5

Thailand	1	1	5
Hong Kong, China	4	0	5
Chile	2	2	5
Japan	3	3	5
Argentina	2	0	5
Qatar	2	2	5
Chile	2	1	5
Thailand	4	3	5
Denmark	4	3	5
Egypt	5	2	5
Japan	3	1	5
Great Britain	5	3	5
Cote D'Ivoire	3	0	5
Korea, Rep.	2	2	5
Korea, Rep.	2	2	5
New Zealand	1	1	5
Croatia	6	3	5
USA	3	0	5
Great Britain	6	3	5
Germany	3	2	5
Greece	6	3	5
Uzbekistan	12	8	5
India	2	2	5
Paraguay	2	1	5
Bulgaria	2	2	5
USA	1	1	5
Bulgaria	3	1	5
Mexico	4	4	5
Romania	4	3	5
Russia	1	1	5
France	4	2	5
Russia	1	0	5
Brazil	1	0	5
Spain	5	3	5
Romania	6	3	5
Russia	6	1	5
France	2	2	5
Japan	1	1	5
Russia	7	3	5
Romania	9	6	2
Costa Rica	12	7	2
Sweden	5	4	2
Sweden	5	4	2
Portugal	9	6	0
Cyprus	7	6	0
Great Britain	5	4	0
Mexico	4	4	0
Lebanon	2	2	4
Egypt	3	4	4
Bulgaria	10	4	4

Bulgaria	4	2	4
Russia	5	4	4
Iran	3	1	4
Slovakia	6	4	4
Hong Kong, China	2	2	4
Australia	2	1	4
Finland	5	5	4
Honduras	5	3	4
Turkiye	8	5	4
Russia	6	5	4
India	5	1	4
USA	8	6	4
Paraguay	7	5	4
USA	6	4	4
India	11	6	4
Uzbekistan	16	6	4
Australia	2	2	4
Venezuela	7	5	4
Colombia	6	5	0
Peru	2	2	0
Nigeria	5	4	0
Switzerland	5	4	0
Thailand	6	6	2
Hong Kong, China	4	4	2
Namibia	5	4	2
Turkiye	7	6	2
Turkiye	10	7	2
Ghana	5	4	2
Brazil	7	4	2
Kosovo	11	5	2
Brazil	13	13	2
Egypt	5	6	2
Latvia	9	4	2
Austria	11	3	2
Austria	5	4	0
Serbia	15	11	0
USA	15	10	0
Turkiye	11	8	0
Dominican Republic	10	5	0
Iran	5	5	2
Russia	5	3	2
Serbia	11	7	2
USA	4	2	2
Australia	2	2	2
Sweden	7	3	2
Russia	9	5	2
Botswana	5	5	2
Ghana	9	8	0
Spain	12	10	0
Ecuador	3	2	0

USA	4	4	0
Slovenia	13	9	0
France	3	2	0
India	5	5	0
Ukraine	11	7	0
Brazil	12	6	4
France	3	3	4
Uzbekistan	5	4	4
Czechia	6	4	4
Ukraine	8	2	4
Belgium	7	4	4
Russia	11	3	4
Pakistan	2	2	4
Colombia	6	4	4
India	8	4	4
India	7	3	4
Uzbekistan	12	4	4
Estonia	13	7	4
India	6	6	4
Iran	4	2	4
USA	4	3	4
South Africa	2	0	4
Czechia	2	1	4
Serbia	8	3	0
Russia	2	2	0
Russia	2	2	0
Spain	6	4	0
Korea, Rep.	5	5	0
Italy	8	5	0
Great Britain	6	5	2
New Zealand	2	2	2
Thailand	6	5	2
Thailand	3	3	2
Bosnia and Herzegov	6	3	2
Czechia	3	3	2
Slovakia	6	4	2
Singapore	5	5	2
Ukraine	3	2	0
India	7	1	0
Austria	5	3	0
France	1	1	0
Australia	4	3	0
France	1	1	0
Australia	2	2	0
Australia	3	2	0
Germany	5	1	0
Germany	2	1	0
Canada	4	3	0
Ukraine	3	2	0
Canada	2	2	0

Australia	4	3	0
Austria	5	3	0
Canada	2	1	0
Australia	4	3	0
Spain	5	4	2
Thailand	7	6	2
Finland	6	5	2
New Zealand	3	3	2
Russia	1	1	2
Venezuela	4	3	2
India	4	3	2
Poland	5	1	2
Bulgaria	8	3	2
Georgia	9	6	2
India	6	3	2
Great Britain	7	4	2
Brazil	8	4	2
Italy	4	1	2
Denmark	2	1	2
India	7	4	2
Bulgaria	12	7	2
Colombia	5	3	2
Kazakhstan	18	10	2
Portugal	2	2	2
India	6	4	2
Japan	2	1	2
Tunisia	10	7	2
Peru	4	3	2
Russia	2	1	2
Great Britain	5	3	2
Tunisia	8	4	2
Serbia	7	2	2
Italy	5	2	2
Singapore	3	3	2
Kosovo	9	8	2
Pakistan	4	4	2
Finland	7	6	2
Italy	1	1	2
Nigeria	4	4	2
USA	2	2	2
Bulgaria	3	2	0
Russia	3	2	0
Slovenia	3	1	0
Portugal	3	3	0
Netherlands	8	3	0
New Zealand	3	3	0
Argentina	2	2	0
Hungary	8	5	0
Argentina	2	2	0
Hungary	11	5	0

India	5	1	0
Germany	2	1	0
Mexico	11	8	0
Germany	5	2	0
USA	3	2	0
Greece	3	3	0
USA	1	1	0
Italy	3	1	0
Hong Kong, China	3	2	0
Slovenia	2	1	0
Bolivia	3	3	0
Slovenia	5	1	0
Singapore	7	2	0
Hong Kong, China	1	1	0
Australia	4	3	0
Australia	4	4	0
Argentina	2	2	0
Australia	3	1	0
Italy	7	5	0
Belgium	9	7	0
USA	12	6	0
Austria	2	3	0
USA	8	7	0
Canada	3	2	0
USA	1	1	0
Sweden	2	1	0
Bolivia	1	1	0
Finland	3	1	0
Israel	3	3	0
Hungary	14	6	0
USA	12	6	0
Sweden	2	2	0
Thailand	3	3	0
Cameroon	2	2	0
France	9	6	0
Kazakhstan	11	6	0
Sweden	9	6	0
Poland	6	3	0
South Africa	6	4	0
Venezuela	5	4	2
India	9	7	2
Australia	1	1	2
Egypt	3	3	2
Venezuela	5	4	2
Iran	4	3	2
Honduras	7	4	2
Sweden	2	1	2
Bolivia	3	2	2
Greece	7	4	2
Belgium	6	3	2

USA	3	3	2
France	5	3	2
Great Britain	4	2	2
Egypt	9	7	2
India	6	3	2
Switzerland	7	3	2
Turkiye	9	3	2
USA	4	3	2
India	8	4	2
Turkiye	6	6	2
Japan	2	2	2
Hong Kong, China	6	3	2
Romania	3	3	2
Iran	2	2	2
Nigeria	1	1	2
South Africa	9	6	2
Brazil	12	3	2
Israel	10	5	2
Poland	4	2	2
Singapore	3	2	2
Austria	10	2	2
Lithuania	4	2	2
Nigeria	2	2	2
Lithuania	5	3	2
Italy	3	2	2
Romania	4	2	2
India	9	7	2
USA	10	4	2
Portugal	8	5	2
France	5	5	2
Cameroon	3	3	2
Venezuela	2	2	2
Brazil	2	1	2
Chinese Taipei	1	1	2
Venezuela	6	5	2
Ecuador	4	4	2
Finland	7	4	2
Germany	7	3	2
Canada	7	4	2
Japan	7	3	2
Hong Kong, China	2	2	2
USA	5	1	2
Czechia	5	3	2
Iran	5	4	2
Spain	9	4	2
USA	2	1	2
Serbia	7	2	2
Romania	11	5	2
Great Britain	6	5	2
Canada	9	2	2

Korea, Rep.	2	2	2
Canada	5	4	2
Belgium	6	2	2
Portugal	5	4	2
Korea, Rep.	2	2	2
Romania	6	5	0
Dominican Republic	10	8	0
Bolivia	7	4	0
Canada	2	2	0
Korea, Rep.	4	4	0
Israel	7	5	0
Italy	1	2	0
Romania	2	2	0
Belarus	9	6	0
Great Britain	5	4	0
Italy	8	5	0
Austria	13	10	0
Romania	3	2	0
Egypt	6	7	0
Thailand	7	6	0
South Africa	11	5	0
Russia	9	5	0
Turkiye	8	6	0
Denmark	6	4	0
Australia	6	6	0
Brazil	11	8	0
Kazakhstan	11	4	0
Venezuela	2	2	0
Korea, Rep.	2	2	0
Great Britain	8	3	0
India	9	7	0
Venezuela	2	2	0
Finland	4	2	0
Mexico	9	6	0
Moldova	8	1	2
Croatia	9	3	2
Spain	1	0	2
France	1	1	2
Kazakhstan	13	3	2
Japan	5	2	2
Croatia	8	1	2
Russia	3	0	2
Austria	5	2	2
Bosnia and Herzegov	16	5	2
Romania	4	2	2
Tunisia	10	7	2
Ukraine	4	1	2
Peru	5	3	2
France	4	2	2
Hong Kong, China	2	2	2

Kazakhstan	7	5	2
Colombia	1	1	2
Colombia	7	3	2
Panama	6	6	2
Brazil	3	2	2
Kyrgyzstan	6	2	2
India	2	1	2
Russia	3	0	2
Bolivia	5	2	2
Norway	2	0	2
Iran	4	4	2
El Salvador	6	2	2
Peru	1	1	2
Venezuela	4	4	2
Russia	4	0	2
Great Britain	2	2	2
Bolivia	6	5	2
India	3	2	2
Venezuela	4	1	2
USA	1	1	2
Ireland	3	3	2
Spain	2	1	2
Switzerland	7	3	2
Poland	7	3	2
Brazil	1	1	2
Venezuela	5	4	2
France	1	1	2
USA	1	1	2
Colombia	6	2	2
Japan	5	4	2
Czechia	2	1	2
Serbia	10	5	2
Ecuador	4	4	2
Brazil	2	1	2
Japan	5	2	2
Cameroon	2	1	2
Colombia	5	2	2
Zimbabwe	3	2	2
Thailand	2	2	2
Italy	5	1	2
Brazil	6	1	2
Russia	10	2	2
Iran	3	1	2
Japan	2	0	2
Venezuela	4	4	2
Poland	9	4	2
Ghana	9	3	2
Slovakia	2	1	2
Paraguay	2	1	2
Serbia	4	2	2

Germany	2	1	2
New Zealand	1	1	2
Egypt	3	2	2
Venezuela	2	1	2
Croatia	9	3	2
Colombia	4	1	2
Lebanon	4	2	2
Spain	8	4	2
Germany	1	0	2
Cameroon	2	1	2
Russia	7	3	2
France	1	1	2
Pakistan	4	4	2
Romania	2	1	2
Italy	2	1	2
Portugal	4	3	2
Poland	5	2	2
Russia	7	4	2
Bosnia and Herzegov	1	1	2
Iran	5	3	2
Turkiye	4	1	2
Turkiye	6	3	2
Mexico	5	1	2
Korea, Rep.	2	2	2
Bulgaria	2	1	2
Turkiye	3	1	2
USA	1	1	2
Argentina	1	0	2
India	2	1	2
Russia	4	3	2
Mexico	5	3	2
Croatia	8	1	2
Venezuela	4	3	2
Kazakhstan	13	3	2
Australia	4	4	2
Serbia	1	1	2
India	3	0	2
Tunisia	6	1	2
South Africa	2	1	2
Italy	2	1	2
Bolivia	1	1	2
Bulgaria	5	2	2
India	3	2	2
Poland	9	4	2
Brazil	1	0	2
Serbia	4	2	2
Venezuela	5	4	2
Germany	1	0	2
Mexico	5	3	2
Norway	1	0	2

Netherlands	1	1	2
France	1	1	2
Iran	3	1	2
Spain	8	4	2
Brazil	6	1	2
Argentina	1	0	2
Paraguay	2	1	2
India	3	0	2
Lebanon	4	2	2
Serbia	1	1	2
Iran	1	0	2
Portugal	4	3	2
Venezuela	3	1	2
Spain	1	1	2
Peru	1	1	2
Iran	4	4	2
Austria	6	3	2
Italy	2	1	2
Brazil	3	2	2
Norway	2	1	2
Colombia	1	2	0
New Zealand	3	1	0
Bolivia	3	1	0
Sweden	2	2	0
Sweden	3	1	0
Botswana	2	2	0
USA	5	2	0
USA	1	1	0
USA	4	3	0
Estonia	1	1	0
Israel	8	4	0
Hungary	5	1	0
Great Britain	5	1	0
Germany	3	1	0
Australia	6	5	0
Germany	6	3	0
Romania	2	1	0
Portugal	6	5	0
Turkiye	5	2	0
Netherlands	7	2	0
Great Britain	3	3	0
Ukraine	4	2	0
Australia	1	1	0
USA	1	1	0
Chile	2	2	0
Germany	8	2	0
Chile	4	4	0
Italy	6	4	0
Mexico	6	4	0
Great Britain	9	5	0

Argentina	2	2	0
Romania	4	3	0
Netherlands	4	2	0
Thailand	3	2	0
India	2	1	0
New Zealand	3	3	0
USA	1	1	0
Netherlands	7	2	0
USA	2	1	0
Zimbabwe	3	3	0
Germany	3	2	0
Japan	2	2	0
Saudi Arabia	6	2	0
Mexico	4	1	0
Egypt	6	6	0
Thailand	2	2	0
France	4	2	0
Spain	5	3	0
Croatia	1	1	0
USA	2	1	0
Great Britain	3	1	0
Canada	5	3	0
Hungary	3	2	0
Australia	2	1	0
Romania	4	1	0
Canada	3	3	0
Iran	2	2	0
Greece	3	3	0
Togo	2	1	0
South Africa	6	2	0
Thailand	6	3	0
USA	2	1	0
Argentina	1	1	0
Australia	2	2	0
Canada	1	1	0
Slovakia	6	3	0
Poland	2	2	0
Benin	3	1	0
New Zealand	2	2	0
Australia	5	3	0
Switzerland	1	1	0
France	4	2	0
USA	5	3	0
Australia	2	2	0
Thailand	6	3	0
Switzerland	1	1	0
Argentina	1	1	0
New Zealand	2	2	0
Estonia	1	1	0
Romania	6	5	0

Romania	4	1	0
France	1	1	0
Great Britain	3	1	0
Canada	1	1	0
Slovakia	6	3	0
Sweden	3	1	0
USA	4	1	0
Nigeria	6	5	0
Ukraine	6	2	0
Romania	3	2	0
Sweden	7	2	0
Russia	4	3	0
Brazil	2	2	0
New Zealand	1	1	0
Spain	5	2	0
Pakistan	4	4	0
Netherlands	1	1	0
Georgia	7	5	0
Slovakia	3	1	0
Mexico	11	4	0
Spain	3	2	0
Canada	2	1	0
Sweden	2	1	0
Colombia	5	3	0
Russia	5	3	0
Azerbaijan	2	2	0
Great Britain	4	2	0
Finland	4	3	0
Spain	4	3	0
Spain	4	2	0
Great Britain	1	1	0
Mexico	6	6	0
Italy	2	2	0
San Marino	1	1	0
Georgia	6	5	0
Sweden	4	3	0
Guatemala	5	3	0
Czechia	5	3	0
Portugal	5	5	0
Spain	5	2	0
France	6	3	0
France	4	1	0
Canada	2	1	0
France	4	1	0
France	8	2	0
China, P.R.	8	7	0
Russia	10	6	0
Japan	3	2	0
Egypt	7	4	0
Switzerland	3	2	0

Korea, Rep.	3	3	0
Moldova	1	1	0
Italy	5	3	0
Mexico	8	4	0
Mexico	11	2	0
Italy	2	2	0
Israel	8	4	0
Pakistan	4	3	0
Germany	1	1	0
Italy	2	1	0
Czechia	1	1	0
Cyprus	5	4	0
Italy	2	1	0
Finland	2	1	0
France	4	2	0
Venezuela	4	4	0
Tajikistan	4	4	0
Iran	5	5	0
Czechia	3	1	0
Spain	3	1	0
Hong Kong, China	2	1	0
Czechia	1	1	0
Hong Kong, China	3	2	0
Korea, Rep.	6	6	0
Great Britain	5	2	0
Switzerland	3	1	0
Georgia	7	4	0
India	2	1	0
Mexico	8	5	0
India	3	1	0
Egypt	11	8	0
Mexico	1	1	0
Ukraine	12	3	0
Estonia	4	1	0
Italy	2	1	0
Japan	1	1	0
USA	7	2	0
Norway	2	1	0
Pakistan	4	3	0
Venezuela	4	3	0
Bolivia	3	2	0
Belgium	2	2	0
Japan	1	1	0
Venezuela	4	3	0
Serbia	1	1	0
Iran	1	1	0
France	1	1	0
El Salvador	10	5	0
Iran	4	3	0
Australia	2	2	0

Peru	4	1	0
Bulgaria	5	2	0
India	5	2	0
USA	3	1	0
Tunisia	8	2	0
Romania	6	2	0
Italy	3	3	0
India	3	2	0
France	1	1	0
Portugal	3	3	0
Estonia	4	1	0
USA	2	1	0
Spain	1	1	0
Poland	8	2	0
Bolivia	3	3	0
Norway	1	1	0
Australia	2	2	0
Norway	2	1	0
Belgium	2	2	0
Venezuela	2	2	0
Japan	1	1	0
USA	4	3	0
Bolivia	3	2	0
Senegal	4	1	0
Australia	4	3	0
Belarus	3	3	0
Great Britain	1	1	0
Uzbekistan	1	1	0
Japan	1	1	0
Japan	7	4	0
Montenegro	5	1	0
Russia	3	2	0
Australia	4	4	0
Italy	2	1	0
Korea, Rep.	5	5	0
Romania	2	2	0
Nigeria	1	1	0
Japan	5	1	0
Japan	1	1	0
Thailand	4	2	0
Australia	3	3	0
Bosnia and Herzegov	3	1	0
Germany	6	4	0
Latvia	9	3	0
Turkiye	4	1	0
Japan	1	1	0
Israel	5	2	0
Peru	4	2	0
Nigeria	2	2	0
Czechia	4	2	0

Ukraine	6	2	0
Jordan	1	1	0
USA	7	2	0
Ecuador	3	2	0
Spain	2	1	0
Venezuela	3	2	0
Nigeria	3	3	0
Cameroon	2	1	0
Great Britain	2	2	0
Belgium	5	2	0
Croatia	7	3	0
Paraguay	3	3	0
Brazil	6	3	0
Mexico	7	2	0
Croatia	7	3	0
Bolivia	3	2	0
Great Britain	2	1	0
Korea, Rep.	5	4	0
Nepal	6	4	0
France	4	1	0
Belgium	2	2	0
Bulgaria	4	2	0
Serbia	3	1	0
USA	5	4	0
Italy	8	5	0
Bosnia and Herzegov	4	2	0
France	4	3	0
Mexico	1	1	0
Mexico	15	7	0
Cameroon	3	1	0
Turkiye	3	1	0
Russia	2	1	0
Russia	6	2	0
Argentina	3	1	0
Mexico	1	1	0
Russia	2	1	0
India	5	1	0
Finland	7	7	0
Czechia	2	2	0
Russia	2	1	0
Italy	2	1	0
Armenia	3	3	0
USA	4	3	0
Japan	7	4	0
Japan	5	1	0
Japan	1	1	0
North Macedonia	10	6	0
El Salvador	10	5	0
USA	3	1	0
USA	5	1	0

USA	2	1	0
Venezuela	3	2	0
Peru	4	2	0
Bulgaria	4	2	0
Russia	3	2	0
Thailand	4	2	0
Iran	1	1	0
Mexico	7	2	0
Germany	5	4	0
South Africa	4	1	0
Tunisia	8	2	0
Egypt	8	7	0
Turkiye	5	3	0
Bosnia and Herzegov	3	1	0
Czechia	4	2	0
Colombia	4	1	0
Brazil	1	1	0
Norway	1	1	0
Senegal	4	1	0
Australia	2	2	0
India	3	2	0
Romania	6	2	0
Japan	1	1	0
Tunisia	4	1	0
Peru	4	1	0
Bulgaria	5	2	0
Portugal	3	3	0
Lebanon	3	1	0
Tunisia	2	1	0
France	2	2	0
Venezuela	2	2	0
Pakistan	4	3	0
France	1	1	0
Russia	1	1	0
Spain	2	1	0
Brazil	6	3	0
Japan	1	1	0
Poland	8	2	0
India	3	1	0
Japan	8	5	0
Nigeria	3	3	0
Venezuela	1	1	0
Spain	5	1	0
Cameroon	3	1	0
Australia	2	2	0
Australia	4	3	0
Australia	4	4	0
Australia	3	3	0
Nigeria	2	2	0
Spain	2	1	0

France	1	1	0
Spain	1	1	0
Japan	1	1	0
Japan	1	1	0
Nigeria	1	1	0
Cameroon	2	1	0
Italy	3	3	0
France	16	15	2700
Denmark	9	6	2510
Switzerland	17	16	1905
Japan	11	9	2030
France	17	12	1825
Switzerland	14	10	1725
USA	11	8	1455
Brazil	20	19	1190
Latvia	22	19	1230
Italy	20	18	1170
China, P.R.	23	22	1215
Great Britain	21	21	1045
Argentina	14	14	1185
Hungary	21	17	1080
South Africa	17	19	1140
Switzerland	20	18	1025
France	19	14	1090
Brazil	12	11	920
Italy	19	18	795
Netherlands	18	16	970
France	23	22	890
USA	8	8	850
USA	32	30	720
France	18	15	880
Argentina	18	17	865
Argentina	24	24	920
Great Britain	15	14	880
Czechia	13	13	810
Czechia	17	14	785
Austria	21	21	850
Belgium	20	17	766
Belgium	24	23	730
France	22	21	735
Great Britain	22	18	655
Cote D'Ivoire	19	18	750
France	21	19	705
Serbia	12	10	755
France	19	16	746
Switzerland	20	13	531
Romania	15	14	520
Japan	17	17	555
China, P.R.	15	13	635
Poland	20	18	710

France	15	14	778
Belgium	19	16	600
Brazil	27	26	425
China, P.R.	16	13	680
Sweden	23	20	680
Poland	22	19	405
Hong Kong, China	12	11	665
Russia	14	13	630
Spain	12	12	700
Russia	20	19	550
Italy	15	14	590
Brazil	11	9	625
USA	17	17	650
Italy	18	18	515
USA	19	18	586
Denmark	21	10	635
France	18	14	622
Poland	22	19	630
Belgium	16	16	610
Spain	16	13	470
Hungary	31	23	470
Italy	17	15	526
Romania	19	18	475
Moldova	23	21	480
Mexico	24	23	510
China, P.R.	15	15	516
Germany	22	19	470
Germany	22	20	455
USA	17	11	470
France	21	18	465
Italy	22	21	385
France	17	14	456
USA	15	15	456
USA	17	15	456
India	21	13	440
Japan	19	19	400
Italy	13	11	411
Spain	10	10	440
Italy	16	14	480
France	21	21	451
Thailand	14	11	516
Great Britain	22	22	405
Spain	5	1	560
Paraguay	16	15	441
Bulgaria	22	20	450
Russia	22	21	392
Spain	17	18	475
Russia	21	16	475
China, P.R.	20	18	432
Australia	17	16	480

Italy	19	18	400
Slovakia	17	15	420
Belgium	24	23	414
Australia	25	21	406
Russia	27	23	362
Russia	14	10	416
France	16	13	478
Belgium	26	25	400
Italy	13	13	345
Germany	21	15	457
Ukraine	18	15	416
Switzerland	21	21	371
Argentina	9	9	325
Jordan	18	16	392
USA	12	12	430
Bulgaria	19	17	376
USA	22	19	370
Colombia	13	13	361
Brazil	20	20	376
Brazil	21	16	345
Canada	14	11	390
Russia	10	8	410
Switzerland	22	19	402
Spain	25	23	346
Ecuador	15	15	347
Great Britain	19	19	361
Poland	26	25	346
Spain	16	14	346
Brazil	13	13	372
USA	20	20	361
Germany	15	14	392
Croatia	22	22	336
France	19	16	361
Austria	29	26	350
Finland	23	22	352
Russia	24	21	392
Russia	18	16	376
USA	16	16	322
Guatemala	20	19	336
France	13	13	396
Sweden	18	16	371
Germany	22	20	313
Great Britain	25	23	288
Russia	22	18	306
Slovakia	22	18	298
Germany	26	24	328
Netherlands	20	16	326
Japan	19	18	275
USA	13	12	326
Czechia	26	24	302

Slovakia	23	20	282
Chinese Taipei	11	11	316
Netherlands	25	22	252
Australia	17	17	306
Spain	20	18	328
New Zealand	20	16	300
France	25	22	312
Ukraine	20	18	306
Israel	22	22	322
Morocco	15	11	298
China, P.R.	19	18	258
Poland	21	18	328
Italy	26	21	248
Czechia	20	16	270
Argentina	8	8	285
Bulgaria	18	17	258
Serbia	23	18	260
Great Britain	33	27	249
Uruguay	19	17	281
Spain	10	9	306
Philippines	23	19	280
Russia	15	15	258
Estonia	22	19	246
China, P.R.	21	19	276
USA	5	2	290
Russia	14	14	266
USA	16	9	282
Switzerland	23	20	236
Portugal	16	13	257
Belarus	22	15	210
Croatia	19	18	226
Cyprus	22	21	248
Madagascar	17	17	268
Spain	22	20	282
Ecuador	18	15	271
Korea, Rep.	16	16	282
Montenegro	18	18	276
Argentina	17	15	245
Czechia	21	19	228
Canada	13	12	284
Serbia	22	16	264
USA	16	15	224
Portugal	18	15	236
USA	13	13	230
France	19	18	231
Czechia	21	17	267
Czechia	12	10	286
Poland	18	17	205
Australia	14	14	210
USA	7	6	223

Mozambique	15	13	230
Slovakia	21	20	172
Germany	24	22	240
Poland	19	16	252
Ukraine	23	18	242
France	28	25	222
Tunisia	20	19	234
China, P.R.	19	19	218
USA	22	19	198
Morocco	15	13	240
Tunisia	23	23	252
USA	10	9	265
Sweden	19	19	242
France	22	18	248
Czechia	18	12	200
Korea, Rep.	6	4	245
Slovenia	17	14	246
Morocco	15	13	238
Mexico	16	15	216
Mexico	20	20	226
Spain	14	13	238
New Zealand	9	9	230
Ireland	24	23	193
Netherlands	17	17	172
Portugal	14	14	182
Netherlands	21	17	196
New Zealand	15	14	194
Korea, Rep.	24	24	180
Slovakia	20	18	206
USA	22	18	222
USA	16	16	192
Italy	13	11	176
Latvia	35	32	156
Australia	3	3	220
Great Britain	28	23	180
France	12	6	218
Colombia	18	16	191
Uzbekistan	16	16	212
Russia	24	23	182
Bulgaria	22	17	206
Colombia	11	11	210
Germany	26	21	183
Chinese Taipei	11	11	213
Uzbekistan	11	11	176
Russia	8	6	226
Australia	17	17	129
Poland	17	15	198
Thailand	21	21	120
Colombia	25	24	196
USA	15	11	146

Spain	18	8	198
France	21	16	218
Thailand	20	17	200
Spain	16	9	216
North Macedonia	22	18	186
Denmark	18	18	148
France	13	13	174
Canada	14	13	189
China, P.R.	17	14	150
Italy	18	14	184
Argentina	11	9	186
Spain	18	13	204
Great Britain	17	15	166
Japan	24	24	175
Croatia	21	16	171
USA	17	17	133
Brazil	11	11	148
Kazakhstan	27	24	164
South Africa	14	13	169
Italy	14	12	158
Benin	13	13	182
Australia	11	10	205
Russia	18	18	145
Belgium	15	9	188
Great Britain	22	18	146
Canada	18	15	151
Japan	12	12	184
India	16	15	160
Serbia	11	4	204
USA	15	15	148
Namibia	14	14	154
Israel	22	21	160
Canada	17	14	116
Russia	20	18	162
Australia	18	18	111
Bulgaria	30	24	168
Israel	22	21	103
South Africa	17	17	127
Australia	22	22	143
Chinese Taipei	16	16	141
Brazil	13	12	145
Poland	24	20	148
Lithuania	22	15	164
Japan	16	16	124
USA	12	10	141
Netherlands	22	17	150
Argentina	12	12	124
Sweden	15	13	174
Egypt	17	13	145
Mexico	12	12	171

Turkiye	21	14	138
Japan	13	13	158
Turkiye	20	20	126
USA	3	2	185
Poland	22	18	130
China, P.R.	17	15	134
Argentina	10	10	162
USA	9	7	176
USA	21	18	133
Czechia	11	9	138
Ireland	16	15	142
Portugal	16	16	142
USA	4	4	168
Australia	10	9	98
Canada	18	16	134
Lithuania	20	18	142
Mexico	15	15	126
Egypt	15	15	144
India	13	13	139
Great Britain	19	14	159
Lithuania	26	22	144
India	13	12	135
China, P.R.	11	9	139
Argentina	16	15	113
Venezuela	9	9	148
Mexico	13	13	131
Romania	10	11	133
Japan	26	24	116
Germany	21	18	109
Slovenia	20	17	160
Japan	20	20	134
Belarus	21	19	119
Tunisia	21	21	138
Romania	7	4	135
Norway	18	11	126
Seychelles	14	13	133
Czechia	18	15	128
Korea, Rep.	15	14	138
Russia	14	11	124
Tunisia	16	15	123
France	8	7	141
Russia	12	11	144
Pakistan	6	6	147
North Macedonia	20	19	124
Germany	26	21	120
Mexico	7	7	115
USA	25	20	106
USA	18	13	147
Uruguay	11	11	121
Chile	22	21	134

France	12	10	110
Kazakhstan	18	18	124
USA	8	6	145
Russia	24	19	114
Poland	25	21	126
Mexico	17	16	115
Greece	21	17	115
Japan	15	15	109
Korea, Rep.	12	11	129
Egypt	18	16	127
China, P.R.	6	4	126
Japan	16	16	138
USA	15	15	114
Thailand	17	15	117
Bulgaria	22	19	136
Great Britain	23	22	130
Bulgaria	20	16	112
Finland	10	10	132
Switzerland	22	17	125
Spain	13	11	118
Belgium	13	9	126
New Zealand	12	12	114
Australia	17	17	98
Thailand	22	20	132
Israel	16	15	131
Peru	25	23	125
South Africa	19	19	96
Great Britain	22	16	118
Macau	19	18	109
Slovenia	22	20	120
Brazil	9	6	151
Norway	16	16	113
Poland	14	12	134
Russia	13	10	90
Spain	10	7	95
New Zealand	19	19	118
Australia	15	13	110
Argentina	15	13	97
India	13	13	103
Switzerland	17	15	120
Vanuatu	10	10	124
Israel	21	21	107
Great Britain	23	19	111
Germany	26	25	102
Brazil	24	24	101
Thailand	13	12	120
Sweden	17	16	110
Netherlands	22	20	98
France	16	14	107
Russia	12	12	124

Portugal	8	8	118
Belgium	18	14	116
Israel	22	18	112
Chile	11	11	116
South Africa	10	10	114
USA	12	12	99
Hungary	11	11	73
France	18	15	121
Morocco	12	10	104
Czechia	26	18	99
Colombia	15	15	102
Austria	13	6	134
Portugal	18	16	108
USA	22	19	70
Morocco	19	16	103
Spain	7	5	125
Bolivia	16	13	118
Russia	20	20	88
Belarus	14	12	93
USA	12	10	105
Lithuania	28	24	93
Peru	10	10	107
Singapore	27	27	107
Spain	12	9	119
India	17	15	103
Latvia	24	19	92
Croatia	9	7	118
Israel	18	16	102
Chile	19	17	114
Bulgaria	16	14	88
France	16	12	115
Cyprus	20	18	96
Great Britain	20	15	92
Malaysia	12	11	125
USA	14	14	104
Egypt	12	9	122
Russia	12	11	120
Italy	17	13	103
Bolivia	12	11	78
Cyprus	16	15	97
USA	3	2	125
USA	8	8	97
France	16	11	90
France	23	20	116
USA	19	17	110
Croatia	21	18	79
Belarus	11	11	101
USA	14	14	101
Netherlands	24	21	85
Jamaica	24	22	81

Sweden	22	22	85
Japan	23	20	98
Canada	20	17	96
Venezuela	12	12	98
Namibia	11	10	70
Bolivia	20	19	96
Japan	18	15	110
Romania	16	15	88
Russia	17	17	86
USA	12	10	73
Philippines	10	10	102
Nigeria	11	10	112
Brazil	10	8	89
Morocco	22	18	89
USA	13	11	94
Czechia	19	19	73
Chile	20	19	92
France	11	11	115
Russia	9	8	73
Chinese Taipei	13	13	85
Mauritius	19	15	67
Belarus	18	9	78
Australia	19	15	101
Great Britain	21	18	95
India	15	14	102
Korea, Rep.	6	6	109
Korea, Rep.	10	10	106
France	11	8	112
Poland	19	17	95
USA	12	3	110
Brazil	12	10	86
China, P.R.	15	14	70
Cote D'Ivoire	13	13	98
Pakistan	10	9	98
Italy	14	12	103
Poland	14	11	93
Italy	20	17	97
Australia	13	13	96
Japan	5	3	115
Thailand	28	27	97
Austria	22	15	84
Czechia	23	19	76
Thailand	4	6	111
Greece	17	17	69
Chinese Taipei	13	12	84
Poland	23	21	86
Lithuania	21	18	81
Ukraine	12	11	97
Slovenia	16	11	77
Great Britain	18	14	66

Serbia	20	12	76
Great Britain	20	12	102
Tunisia	23	22	62
New Zealand	18	17	88
Romania	16	14	75
Turkiye	21	17	93
Argentina	25	24	88
Czechia	13	10	63
Netherlands	15	15	76
USA	18	17	78
Colombia	12	12	76
Czechia	19	16	74
Canada	11	11	65
Hungary	14	14	88
Sweden	21	19	76
Belarus	6	4	81
Argentina	12	11	76
Brazil	4	4	95
Brazil	12	9	87
Israel	10	10	65
USA	20	18	85
Brazil	11	10	82
Kazakhstan	17	13	90
Israel	14	12	96
Latvia	20	16	70
Finland	18	15	86
Russia	14	13	76
Israel	20	17	79
Indonesia	8	8	76
Slovakia	15	11	84
Romania	19	13	66
Sweden	9	6	79
Great Britain	11	11	74
China, P.R.	16	14	78
Germany	20	13	81
Greece	17	17	66
Czechia	16	14	89
Germany	13	12	90
Slovenia	26	16	86
India	16	9	82
USA	25	22	73
Egypt	13	11	79
Ukraine	10	8	79
India	8	5	97
USA	10	10	56
Peru	10	9	88
Chinese Taipei	16	15	67
Italy	1	0	100
Czechia	16	11	65
Canada	13	13	55

USA	16	14	88
USA	9	10	43
Slovakia	23	22	63
Switzerland	21	14	87
USA	4	3	83
Colombia	17	16	72
Tunisia	21	19	53
Italy	14	5	93
USA	15	11	69
China, P.R.	17	11	89
Great Britain	14	13	63
India	11	10	74
Spain	10	8	83
Serbia	9	6	89
Russia	19	18	71
Hong Kong, China	10	10	58
Serbia	17	15	73
Paraguay	14	13	54
Montenegro	14	13	52
Bosnia and Herzegov	5	4	89
Great Britain	17	15	83
Croatia	17	15	79
Australia	18	12	75
Australia	11	11	61
Peru	13	13	74
Russia	21	17	52
France	18	18	59
USA	8	7	83
Japan	15	13	65
South Africa	6	6	61
Bulgaria	15	13	59
Romania	18	14	67
Italy	18	15	77
Germany	16	14	70
Turkiye	16	13	77
Chinese Taipei	7	5	72
India	8	8	76
Spain	9	7	86
Zimbabwe	14	13	89
Russia	11	9	71
Italy	13	9	57
Portugal	22	20	41
USA	15	15	61
Switzerland	14	11	83
India	17	17	61
Canada	20	14	64
Cyprus	12	10	69
Poland	13	12	37
Kazakhstan	19	19	55
Canada	11	11	55

Spain	11	5	86
Mexico	14	14	64
Great Britain	20	18	68
Bulgaria	23	21	63
Philippines	17	17	44
USA	7	7	74
Indonesia	12	11	75
Great Britain	12	9	68
USA	13	12	61
Netherlands	13	11	51
Bermuda	13	12	39
Vietnam	12	11	78
Slovenia	17	15	52
Israel	12	9	82
New Zealand	11	10	41
Netherlands	26	23	44
Colombia	15	13	59
Brazil	8	7	80
Uzbekistan	17	14	66
Uzbekistan	5	5	25
Austria	20	17	58
France	7	5	77
Romania	8	6	78
Dominican Republic	12	12	61
Portugal	17	14	43
Belgium	10	5	60
Canada	20	16	66
Netherlands	15	14	57
Malaysia	17	17	46
USA	19	18	61
France	8	6	72
Costa Rica	8	8	65
Spain	14	12	50
Netherlands	14	13	37
Great Britain	9	9	52
Germany	12	9	65
Spain	8	7	69
France	6	6	68
USA	12	11	66
Ukraine	6	4	65
Korea, Rep.	9	9	71
Japan	11	11	50
Estonia	10	10	25
Portugal	11	11	54
Argentina	9	9	44
Canada	19	15	63
Thailand	8	10	25
Hungary	12	9	58
Japan	8	8	60
India	11	11	47

Italy	10	9	70
Tunisia	25	24	35
Australia	14	12	70
France	12	9	68
France	18	16	59
Russia	13	14	45
Germany	12	11	64
Germany	11	9	71
India	14	14	48
Croatia	13	10	64
Germany	13	12	62
Germany	15	12	71
Argentina	18	17	62
USA	18	16	58
Portugal	14	12	46
China, P.R.	5	3	69
Venezuela	6	6	64
Qatar	15	15	68
Korea, Rep.	12	10	50
Korea, Rep.	16	13	51
USA	5	5	64
Kenya	18	17	48
India	13	9	75
Paraguay	12	12	65
Israel	21	19	49
Chile	22	22	48
Great Britain	12	11	60
USA	8	7	50
Italy	14	8	61
Kazakhstan	21	20	50
Switzerland	7	7	65
Czechia	10	10	55
South Africa	18	17	49
India	12	11	56
Japan	15	14	51
Italy	12	11	60
Spain	9	8	60
Bolivia	19	19	49
Spain	18	14	65
Slovakia	15	13	38
Chinese Taipei	12	11	43
Great Britain	9	8	53
Bulgaria	25	19	51
Peru	3	3	57
Hungary	11	9	49
Azerbaijan	21	18	39
India	8	7	57
Algeria	7	6	65
India	10	6	45
Brazil	12	11	44

Colombia	14	11	55
Belgium	20	17	52
France	15	10	52
Great Britain	16	15	45
China, P.R.	18	17	52
Benin	8	8	55
USA	9	7	66
Russia	13	10	53
Serbia	18	15	45
Russia	10	9	53
Russia	11	8	32
Montenegro	12	9	60
Malaysia	13	12	49
Italy	18	14	60
France	5	2	60
Zimbabwe	14	14	42
Czechia	13	10	34
Uzbekistan	10	10	59
Peru	12	12	55
Great Britain	20	15	53
Canada	14	12	48
Barbados	7	6	55
Turkiye	12	6	57
France	7	7	55
Romania	11	8	61
India	11	11	64
Argentina	7	7	43
USA	6	4	30
Spain	18	10	49
Australia	14	13	51
Belgium	15	11	48
Great Britain	12	11	55
Romania	13	11	53
Israel	25	23	37
Togo	9	9	53
Australia	9	6	63
Brazil	18	15	39
France	20	16	35
Russia	11	11	55
Malaysia	10	10	49
Bosnia and Herzegov	10	2	66
Kyrgyzstan	8	7	53
Switzerland	10	6	56
France	4	4	41
Czechia	22	16	50
Mexico	14	14	47
Estonia	12	10	53
Egypt	14	14	22
Bosnia and Herzegov	15	10	45
China, P.R.	11	11	42

Chile	9	9	35
Croatia	15	16	35
Japan	7	6	37
Estonia	12	10	45
Turkiye	23	19	44
France	13	7	30
Israel	21	7	62
India	17	17	44
Colombia	17	12	51
Kenya	12	12	44
Great Britain	20	18	43
Slovenia	16	13	53
Serbia	10	9	36
France	12	9	58
Japan	8	6	54
Uzbekistan	15	14	45
France	6	5	59
Estonia	12	7	37
Russia	11	10	37
Cote D'Ivoire	7	7	35
Croatia	17	13	47
USA	7	5	45
USA	5	4	62
Russia	23	17	48
Mexico	16	16	48
Italy	8	8	47
Chile	8	7	44
Serbia	15	12	49
Korea, Rep.	16	13	56
Italy	10	6	57
Greece	15	13	43
Poland	19	15	43
Mexico	11	11	44
China, P.R.	11	9	40
Argentina	16	15	47
Israel	11	9	51
Argentina	9	8	42
France	13	10	34
Australia	8	5	56
France	6	5	46
USA	3	1	60
Germany	14	11	57
Kyrgyzstan	17	12	46
USA	10	10	48
Poland	17	12	51
Sweden	6	4	58
Korea, Rep.	8	8	44
Finland	7	5	48
Belarus	18	15	40
India	9	9	32

Mexico	12	12	40
Russia	18	16	39
Kazakhstan	16	16	39
Japan	7	6	53
USA	9	9	25
Italy	13	11	27
Chile	12	12	34
Argentina	11	9	45
Czechia	15	12	21
Korea, Rep.	13	10	47
Brazil	11	10	5
China, P.R.	13	11	30
Czechia	17	16	24
Mexico	15	14	30
Chile	12	9	49
Bulgaria	14	12	31
Portugal	13	13	24
Senegal	15	12	31
South Africa	14	12	42
Kazakhstan	15	10	51
Kazakhstan	4	4	48
South Africa	12	11	40
Indonesia	15	15	43
Nigeria	2	2	48
Croatia	28	23	30
France	16	16	35
USA	15	14	40
Lebanon	25	22	30
Czechia	16	13	47
Germany	17	16	31
Argentina	19	14	34
Serbia	21	15	36
Sweden	14	14	30
Italy	8	7	45
France	17	16	34
Belgium	17	14	44
Denmark	19	17	26
Spain	14	10	42
Uzbekistan	3	2	46
Korea, Rep.	13	11	40
Germany	18	16	35
Australia	16	16	34
France	13	13	34
Brazil	6	6	50
Thailand	12	7	45
Russia	13	11	25
El Salvador	15	15	30
Sweden	10	7	46
Russia	12	12	33
Brazil	7	6	49

Great Britain	12	12	36
New Zealand	16	16	39
Turkiye	16	14	40
Mexico	11	10	41
India	5	4	41
Great Britain	13	12	34
Bolivia	10	8	39
Brazil	18	14	10
Argentina	16	14	37
Canada	8	8	39
USA	12	9	39
Slovenia	11	10	20
France	13	11	38
Colombia	6	6	41
Great Britain	29	29	27
Korea, Rep.	10	9	39
Armenia	10	8	38
Poland	9	0	51
Kenya	14	13	31
Brazil	18	18	43
India	6	6	47
Japan	9	9	37
Turkiye	17	16	44
Ukraine	7	6	15
Vietnam	9	8	41
Ukraine	11	11	41
Russia	16	14	41
El Salvador	13	13	35
USA	4	4	40
Serbia	8	7	45
Great Britain	7	5	43
Great Britain	17	16	29
Thailand	21	17	36
Spain	7	3	48
Slovakia	7	6	41
Germany	19	10	38
Egypt	14	11	41
Australia	8	8	35
USA	16	11	37
Bahamas	8	8	35
Greece	23	22	35
Slovakia	15	13	19
Ecuador	10	10	39
Italy	15	12	10
Spain	15	10	24
Hong Kong, China	14	14	33
Ukraine	18	8	46
Korea, Rep.	7	6	34
Belarus	13	11	29
Colombia	6	5	43

Croatia	8	6	29
Egypt	19	16	32
Chinese Taipei	15	15	31
Nigeria	10	10	35
Brazil	2	2	35
Guatemala	15	14	40
Belarus	14	14	31
Chinese Taipei	14	14	17
Great Britain	16	14	39
Romania	17	17	35
Portugal	14	12	39
USA	4	4	15
Moldova	27	19	39
Turkiye	19	15	27
USA	9	7	41
Indonesia	5	5	41
Iran	11	9	34
Argentina	6	5	40
Colombia	14	14	27
Switzerland	7	6	20
Korea, Rep.	9	9	24
Latvia	14	13	35
Finland	9	6	38
Portugal	19	16	21
Switzerland	24	19	28
USA	7	5	35
Tunisia	11	9	37
India	7	5	30
USA	5	5	35
Russia	19	17	32
Australia	14	10	30
France	17	14	35
Japan	11	11	27
Chinese Taipei	11	9	32
Argentina	10	6	22
Kazakhstan	13	12	35
USA	11	9	39
Israel	15	12	40
France	12	12	37
Canada	13	11	24
Austria	10	9	37
Belgium	17	13	40
Sweden	10	10	40
Egypt	20	16	38
France	24	21	38
Bosnia and Herzegov	8	7	37
Korea, Rep.	19	19	21
Egypt	12	11	24
Korea, Rep.	4	6	38
Serbia	18	12	34

Pakistan	6	6	32
USA	6	6	32
Chinese Taipei	3	3	42
Uzbekistan	14	14	34
Serbia	13	7	39
Russia	7	5	27
Ukraine	7	4	41
Canada	12	11	32
Ukraine	16	13	14
Romania	8	7	42
Japan	21	17	35
USA	10	8	33
France	14	13	21
Romania	14	13	31
Poland	25	14	29
Argentina	9	9	21
Chinese Taipei	15	14	24
Mexico	21	20	27
Serbia	10	5	32
Russia	13	11	32
Sweden	15	12	39
Thailand	14	13	30
France	15	13	29
Australia	12	10	26
Chinese Taipei	14	14	16
Turkiye	14	10	34
Lithuania	22	16	7
Venezuela	11	9	34
Switzerland	15	10	27
France	15	12	34
Chinese Taipei	5	4	28
Turkiye	15	13	29
Croatia	21	16	24
Kazakhstan	15	13	25
China, P.R.	13	10	34
Italy	20	15	33
Spain	10	9	30
Argentina	7	6	36
China, P.R.	16	13	12
Japan	14	10	18
Great Britain	9	6	37
Japan	19	17	31
Korea, Rep.	5	6	16
Israel	18	13	28
Italy	13	12	21
USA	8	7	12
China, P.R.	12	12	29
Poland	12	9	26
France	9	8	25
Bulgaria	21	16	29

Bolivia	7	7	23
Poland	9	7	29
Romania	11	9	26
France	23	23	30
Great Britain	14	10	32
Great Britain	17	14	31
USA	12	9	28
Bulgaria	19	14	24
Great Britain	10	10	22
Mexico	18	18	24
USA	10	8	27
Chinese Taipei	9	9	22
Netherlands	16	14	21
Serbia	18	14	30
China, P.R.	11	10	19
Indonesia	15	15	16
Netherlands	18	14	14
Argentina	3	2	35
Korea, Rep.	15	15	31
Czechia	12	7	35
Italy	7	7	35
Spain	9	7	30
Great Britain	12	12	31
France	9	4	29
Argentina	9	9	35
Australia	6	5	25
Canada	6	4	24
Colombia	12	12	32
USA	15	10	21
Spain	4	3	36
Spain	9	6	34
Australia	11	11	24
Brazil	10	9	31
Algeria	9	7	26
China, P.R.	15	14	24
Estonia	22	20	21
Slovakia	13	7	31
Mexico	2	2	15
Indonesia	8	8	21
Canada	14	9	29
Chile	6	4	28
USA	14	9	28
Singapore	6	4	29
Italy	17	10	34
USA	5	4	25
Japan	6	5	31
Morocco	9	6	29
Greece	11	9	19
Bulgaria	10	5	32
Russia	6	4	32

USA	11	9	26
Portugal	18	16	27
Russia	4	3	35
Thailand	11	7	18
Uzbekistan	11	10	31
Canada	8	7	30
Russia	10	8	27
Thailand	6	8	17
South Africa	11	10	14
Italy	12	11	20
Brazil	10	8	25
Netherlands	18	12	21
Netherlands	14	12	12
Korea, Rep.	12	9	26
Uzbekistan	9	9	24
Australia	9	9	19
Zimbabwe	15	13	13
Serbia	8	7	19
Great Britain	14	12	22
Japan	14	12	26
Belgium	28	22	14
Nigeria	2	2	35
Malaysia	8	8	32
Austria	11	10	25
Montenegro	26	20	25
France	7	7	24
Venezuela	7	7	26
Belarus	10	8	22
Peru	9	8	23
Slovenia	22	19	21
Egypt	12	9	28
Mexico	11	11	20
Romania	10	8	28
China, P.R.	9	7	33
Finland	8	6	29
Korea, Rep.	6	6	15
Slovakia	19	18	27
Slovenia	13	9	27
Brazil	16	11	14
Italy	9	6	32
Belarus	12	9	20
USA	3	2	23
Bolivia	9	9	30
Indonesia	12	10	20
Russia	15	13	29
Canada	7	6	26
Montenegro	7	6	20
Chinese Taipei	5	5	25
Germany	12	7	25
Czechia	13	9	17

Kazakhstan	20	12	12
France	9	7	25
France	19	16	21
Austria	22	18	12
Russia	10	7	4
Kazakhstan	14	9	24
Canada	8	7	29
France	12	10	12
Netherlands	12	10	5
Austria	15	10	15
Mexico	12	10	24
Spain	7	4	24
Tunisia	22	14	26
Algeria	11	4	25
Spain	9	7	12
USA	19	15	24
Portugal	8	3	22
Kuwait	8	8	18
Argentina	9	9	24
Turkiye	12	10	28
Italy	22	18	24
Israel	12	11	20
France	12	8	29
Japan	7	6	24
Russia	8	7	26
Germany	13	8	25
Sweden	12	8	20
Denmark	18	16	21
Thailand	21	18	24
Japan	2	1	30
Japan	13	10	10
Australia	8	7	19
Switzerland	12	7	21
Brazil	15	12	19
USA	13	13	22
Israel	16	13	22
USA	1	0	30
Portugal	15	10	21
Mexico	17	13	16
Greece	9	8	26
Canada	13	10	23
Turkiye	16	8	16
Argentina	6	3	2
Serbia	7	6	28
Australia	9	7	22
Japan	6	5	15
Latvia	25	18	22
Spain	5	3	29
Israel	22	15	18
France	13	12	15

Korea, Rep.	6	6	28
Austria	17	12	10
Australia	7	7	10
Slovakia	12	9	24
Belarus	19	12	24
Belarus	9	7	22
Poland	8	5	25
USA	12	7	25
Morocco	13	9	20
Canada	5	4	9
Serbia	6	3	19
Japan	12	10	21
Ecuador	7	7	14
Tunisia	16	12	12
Germany	10	3	29
Hungary	8	4	29
South Africa	12	11	25
Zimbabwe	15	12	22
Serbia	2	1	28
Spain	9	7	27
Czechia	7	5	15
Italy	9	4	27
New Zealand	9	8	28
Spain	8	6	20
Italy	7	5	22
Italy	11	9	15
Egypt	16	14	18
Israel	15	15	21
Czechia	12	6	17
Tunisia	10	8	20
Canada	11	10	12
Romania	18	13	12
USA	9	9	22
Italy	8	4	14
Australia	15	13	19
Georgia	11	1	28
Portugal	17	15	23
Uzbekistan	5	5	24
Japan	4	3	20
Benin	5	3	18
Tunisia	18	11	21
Cyprus	18	11	19
France	5	2	23
Greece	14	13	18
Finland	12	3	20
Japan	7	7	13
China, P.R.	10	10	24
South Africa	8	7	26
Barbados	7	7	12
Chile	9	9	12

Australia	4	4	23
Guadeloupe	13	8	20
Denmark	13	11	22
USA	6	5	18
India	15	11	21
France	5	5	23
Slovenia	12	11	10
Ecuador	9	7	11
Latvia	11	7	20
Indonesia	7	7	12
Ukraine	12	7	15
USA	4	1	23
France	4	3	23
USA	11	10	19
France	16	14	16
Venezuela	4	4	20
Slovenia	19	8	24
Tunisia	10	6	19
Spain	4	2	23
Colombia	5	4	23
Colombia	9	9	7
Brazil	7	2	0
Bosnia and Herzegov	7	5	19
Denmark	13	11	21
Russia	11	10	16
Great Britain	6	5	21
Romania	11	9	20
North Macedonia	6	6	16
India	6	4	20
Australia	11	6	20
Italy	6	5	22
Spain	5	1	22
Slovakia	21	16	11
Slovakia	5	0	25
Mexico	11	11	22
Germany	6	4	20
Guadeloupe	10	9	21
Australia	7	5	15
Australia	4	4	22
China, P.R.	5	3	20
Iran	2	2	18
Chile	3	3	11
USA	5	3	10
India	12	10	18
Singapore	15	10	10
Indonesia	10	10	16
Kazakhstan	24	20	18
Chinese Taipei	15	14	12
Argentina	9	7	22
Australia	9	6	21

USA	16	11	21
Portugal	15	12	10
USA	12	10	22
Cameroon	14	12	17
Italy	7	3	24
Bulgaria	11	8	24
Romania	6	4	19
Austria	5	4	17
Sweden	11	10	14
Netherlands	6	4	12
USA	14	11	21
Belarus	11	9	18
Serbia	24	19	13
Czechia	16	12	16
Vietnam	15	11	18
Israel	15	10	12
Turkiye	18	10	6
Mexico	11	8	19
Spain	3	2	20
USA	16	11	15
Sweden	7	4	15
Austria	9	6	12
India	13	11	18
USA	16	16	12
Japan	6	5	9
France	12	12	19
Canada	17	13	12
Australia	12	9	15
Italy	11	11	14
USA	20	17	18
Belgium	9	9	14
Great Britain	22	18	10
Bolivia	5	5	18
Bulgaria	5	5	19
Egypt	8	7	19
Italy	6	5	10
Morocco	7	5	18
USA	8	6	14
Morocco	4	3	18
Chinese Taipei	13	9	14
Russia	7	5	17
France	8	7	17
Cyprus	10	8	12
Belgium	15	11	16
Sweden	9	5	14
Sweden	7	5	12
Thailand	15	10	15
South Africa	19	18	11
Argentina	13	9	11
Turkiye	13	13	11

Zimbabwe	10	10	11
Mexico	11	10	16
Tunisia	12	5	20
Romania	9	7	17
Belarus	19	14	18
Finland	11	6	15
Sweden	11	7	13
Germany	14	11	10
Thailand	17	16	13
France	7	4	2
USA	2	2	18
Bulgaria	13	8	14
Spain	7	6	14
Serbia	10	7	19
Sweden	2	2	20
Venezuela	7	7	9
Egypt	9	6	16
Kazakhstan	11	10	17
Kenya	15	10	17
Israel	9	7	18
Korea, Rep.	12	9	22
Germany	17	14	15
Great Britain	6	3	15
Austria	13	7	21
Nigeria	4	4	17
Korea, Rep.	6	6	21
Australia	6	6	16
Argentina	13	12	14
Finland	8	4	14
New Zealand	10	9	10
Switzerland	11	5	19
Spain	7	3	20
Finland	6	5	12
Russia	5	5	0
Brazil	9	8	19
Chile	12	10	19
China, P.R.	12	10	10
Russia	11	9	14
Bulgaria	9	8	7
Bulgaria	15	6	5
Hungary	7	6	19
Thailand	7	7	18
Great Britain	10	6	10
USA	14	10	11
France	13	8	21
Sweden	10	3	21
Czechia	10	7	14
Saudi Arabia	12	10	12
Brazil	6	6	17
Croatia	4	2	20

Romania	14	6	19
Egypt	4	5	7
Serbia	20	15	13
Curacao	7	7	11
Egypt	17	13	7
Canada	12	12	6
Italy	4	4	0
Romania	11	8	19
USA	5	4	19
Serbia	16	12	19
Malaysia	21	19	15
Algeria	4	2	18
Great Britain	15	14	7
Chinese Taipei	12	12	4
Canada	3	3	17
Croatia	12	9	15
Serbia	7	6	14
Australia	4	4	10
Canada	11	8	12
Lithuania	10	9	9
USA	7	6	5
Hungary	6	6	18
North Macedonia	10	8	6
South Africa	7	4	20
South Africa	4	4	20
Ukraine	1	0	20
China, P.R.	6	4	15
Kazakhstan	13	8	17
Ukraine	8	6	20
Mauritius	2	2	15
France	10	8	16
Singapore	6	4	14
New Zealand	12	13	12
USA	10	9	10
Kazakhstan	4	4	15
Colombia	9	8	16
Canada	8	4	19
Canada	14	12	14
USA	4	4	10
Greece	8	7	14
Tunisia	14	12	9
Chinese Taipei	9	8	11
Netherlands	10	10	8
Netherlands	16	13	7
Russia	10	7	12
Denmark	9	3	16
Croatia	5	4	5
Germany	9	8	15
Turkiye	18	12	11
Brazil	3	3	7

Great Britain	4	5	14
Japan	6	4	14
Austria	11	10	13
Italy	8	3	19
USA	3	3	12
Lithuania	11	9	7
Hong Kong, China	15	11	6
Ukraine	8	6	6
France	8	6	14
Netherlands	12	7	15
Japan	12	11	15
Switzerland	8	6	18
France	5	2	18
Spain	2	2	18
USA	6	6	18
India	3	3	18
Thailand	5	4	14
France	5	3	11
Brazil	6	3	2
France	5	3	0
Mexico	4	4	15
Tunisia	6	5	17
Italy	15	8	14
Serbia	7	6	17
Italy	13	10	14
Colombia	16	13	13
Costa Rica	11	11	5
Austria	13	5	2
USA	15	11	15
Romania	9	7	15
Spain	7	6	7
Germany	8	3	16
India	8	7	16
Russia	6	6	12
Serbia	13	8	10
Turkiye	12	11	7
Germany	25	16	6
Czechia	5	0	18
Ghana	5	4	10
Kazakhstan	14	9	14
Latvia	11	7	14
Serbia	4	4	7
Sweden	10	8	4
Germany	21	14	2
Ukraine	7	5	17
Vietnam	6	5	12
USA	6	6	10
Switzerland	25	13	16
India	10	6	13
Germany	14	13	7

USA	2	2	10
Egypt	11	6	15
Sweden	15	12	14
Greece	5	3	16
Switzerland	8	4	16
Belgium	8	6	9
Russia	2	2	5
USA	6	5	12
Korea, Rep.	5	5	14
China, P.R.	7	6	12
Chinese Taipei	5	4	14
Lithuania	12	8	7
Great Britain	8	7	14
Peru	8	7	12
Dominican Republic	9	5	2
Latvia	9	4	17
Sweden	7	5	12
Tunisia	11	7	12
Czechia	8	7	10
Tunisia	13	7	12
Turkiye	13	12	7
Chinese Taipei	11	11	13
Chinese Taipei	8	7	7
Czechia	9	8	0
Japan	1	1	10
Japan	1	1	10
Spain	5	2	15
USA	3	3	10
Madagascar	17	16	11
Brazil	4	1	16
Belarus	9	7	5
USA	5	5	0
Brazil	9	5	0
Kazakhstan	7	6	15
Russia	10	5	15
Uzbekistan	7	7	12
Russia	6	4	14
Cyprus	13	10	11
Great Britain	14	10	10
Chile	12	7	12
Belgium	14	13	11
Nepal	4	4	7
China, P.R.	4	4	5
Korea, Rep.	4	3	13
Canada	17	14	10
Japan	6	5	7
Italy	5	4	5
Australia	7	7	2
Czechia	11	10	0
Germany	13	8	12

Italy	6	2	16
Brazil	4	3	12
Romania	15	10	11
Czechia	8	6	10
Finland	7	1	16
Belarus	15	8	12
Denmark	10	6	12
Greece	6	4	9
Australia	5	4	7
Egypt	7	6	14
Uzbekistan	6	6	15
Kazakhstan	8	6	12
France	10	4	14
Canada	12	9	9
Sweden	12	9	12
France	12	10	11
Thailand	13	6	4
Poland	10	9	0
Colombia	8	7	9
USA	3	1	14
USA	4	3	14
USA	4	2	14
Germany	12	8	14
Chinese Taipei	10	8	14
South Africa	10	8	11
Belgium	5	4	11
El Salvador	14	11	9
Russia	7	6	2
Czechia	10	7	12
Mexico	9	9	9
Costa Rica	8	8	9
Switzerland	6	4	12
Great Britain	5	5	11
Netherlands	9	9	9
Hungary	8	5	9
USA	4	3	9
Norway	11	8	0
USA	1	1	15
USA	1	1	15
Italy	1	1	15
Uzbekistan	15	8	12
Poland	3	3	10
Sweden	10	3	15
France	6	4	5
France	5	4	12
Australia	5	5	11
Finland	10	9	2
Austria	4	3	2
Ireland	3	3	2
Romania	12	7	14

Portugal	8	8	10
USA	15	14	5
Tunisia	18	8	9
Benin	3	3	5
Australia	12	10	5
France	11	8	11
Hong Kong, China	8	6	2
Russia	13	11	2
Belarus	5	5	10
Iran	12	8	13
Japan	15	14	13
Pakistan	6	6	10
Ukraine	15	7	9
Israel	13	7	9
Germany	12	9	9
Indonesia	5	4	9
Great Britain	7	6	2
Malta	15	12	12
France	7	6	12
Italy	9	9	11
Nigeria	2	2	11
Ukraine	10	3	14
Italy	17	11	10
Dominican Republic	6	6	11
Dominican Republic	17	12	7
France	3	3	14
Venezuela	7	6	14
Iran	4	3	7
Great Britain	5	4	7
Russia	9	4	6
Japan	3	3	4
China, P.R.	10	10	4
Mexico	7	6	12
Kazakhstan	8	6	10
Romania	9	4	12
South Africa	8	8	10
Norway	10	7	12
Israel	10	7	7
Australia	6	5	9
Japan	7	7	2
Russia	11	7	12
Switzerland	1	1	10
Slovenia	6	5	10
Japan	10	9	10
Canada	4	4	12
Canada	6	4	12
Venezuela	9	7	7
Turkiye	14	8	12
Colombia	4	3	12
Great Britain	14	11	9

Iran	8	7	9
New Zealand	11	11	7
Korea, Rep.	9	7	5
Nepal	2	2	4
South Africa	10	9	0
Peru	3	3	7
France	13	3	10
Czechia	4	3	10
Portugal	11	8	7
Canada	16	11	11
Ukraine	12	9	11
Guatemala	4	4	10
France	13	7	10
Hong Kong, China	12	8	9
Ghana	4	4	7
Czechia	2	2	7
India	9	7	11
India	10	9	11
France	12	10	10
Israel	13	9	10
Denmark	4	2	10
Turkiye	15	14	7
China, P.R.	16	14	4
Korea, Rep.	8	7	4
Brazil	6	4	2
Venezuela	8	6	10
Russia	13	9	9
New Zealand	8	7	13
Thailand	14	4	13
Hungary	6	4	10
USA	4	4	9
Ireland	4	2	12
Uzbekistan	5	3	12
Colombia	7	3	12
Australia	5	5	12
Russia	6	2	12
Poland	4	1	12
Tunisia	6	5	12
Japan	4	4	9
France	9	5	7
Iran	3	3	9
Ukraine	19	17	5
China, P.R.	10	6	0
Tunisia	9	4	10
Hungary	9	4	10
Finland	6	3	10
Spain	5	2	5
Lithuania	15	6	11
Hungary	5	5	11
Pakistan	2	2	11

Tajikistan	6	4	11
Great Britain	10	6	11
Germany	10	3	11
Great Britain	3	3	11
Ghana	3	2	11
USA	5	4	7
Hungary	13	9	7
USA	22	19	6
Romania	15	11	5
Germany	12	8	10
Mauritius	6	6	10
Morocco	12	9	9
Singapore	4	4	10
Chile	3	3	10
Israel	13	9	9
India	8	8	9
Korea, Rep.	3	3	9
Finland	7	4	9
Slovenia	8	6	4
USA	7	7	2
Vietnam	6	5	12
Japan	3	1	12
Benin	6	2	12
USA	4	2	12
Indonesia	11	10	7
Canada	8	5	5
France	6	4	9
Norway	6	3	9
New Zealand	8	7	6
Zimbabwe	3	3	5
China, P.R.	11	10	2
Haiti	5	4	10
Japan	13	6	10
France	3	2	10
Slovenia	8	4	10
Sweden	5	2	10
Russia	6	2	10
Guatemala	13	11	11
Spain	17	7	10
Denmark	5	4	10
Luxembourg	9	7	7
Latvia	18	13	5
India	8	3	11
Nigeria	3	3	11
Thailand	6	4	11
Ecuador	13	13	11
Great Britain	4	3	7
Switzerland	10	4	7
Kazakhstan	17	9	4
Greece	9	4	10

Russia	18	11	9
Belgium	9	8	9
Cyprus	10	6	9
USA	8	7	10
Italy	5	2	10
China, P.R.	6	4	10
Russia	9	9	6
Switzerland	11	8	4
Argentina	11	10	2
India	8	7	9
Greece	7	4	7
India	6	3	5
Bulgaria	4	2	5
Pakistan	5	5	9
Japan	11	8	9
Iran	3	2	9
India	9	5	9
Belgium	14	10	9
USA	6	3	0
Great Britain	15	4	11
Canada	19	15	11
Great Britain	10	5	5
Kosovo	5	5	7
Poland	2	2	7
Botswana	3	3	7
Turkiye	8	5	4
Morocco	4	4	10
Brazil	8	6	10
Turkiye	9	8	10
Russia	7	5	10
Italy	3	3	10
Tunisia	7	5	9
China, P.R.	5	5	7
India	9	8	10
New Zealand	7	5	7
Greece	9	7	7
Russia	11	6	7
Netherlands	10	9	4
Costa Rica	5	5	2
Bolivia	5	5	9
Bosnia and Herzegov	5	3	9
Spain	10	6	9
Austria	8	5	7
Morocco	6	3	7
France	4	2	9
USA	5	5	9
Serbia	8	6	9
Romania	8	6	7
Venezuela	3	3	5
Syria	12	9	5

Croatia	9	5	7
Latvia	8	5	7
Korea, Rep.	6	6	7
Luxembourg	9	8	7
Guatemala	11	8	8
Egypt	25	14	8
Chinese Taipei	10	9	7
Canada	8	8	5
Israel	2	1	4
Austria	7	7	2
Israel	11	8	2
Serbia	5	1	10
France	5	3	10
Russia	3	0	10
Italy	4	1	10
Russia	5	2	10
Mexico	6	4	10
USA	4	2	10
Germany	4	3	10
USA	3	1	10
Great Britain	3	3	10
Lithuania	3	3	10
France	4	3	10
Greece	2	2	10
North Macedonia	3	2	5
France	7	5	5
Ireland	8	5	5
Lithuania	10	6	5
Israel	11	3	10
Japan	3	2	10
Russia	10	10	7
France	9	9	7
Italy	3	3	6
Lebanon	6	6	6
Italy	9	6	6
Romania	9	7	2
Indonesia	5	5	9
USA	10	6	9
Jordan	22	11	5
Ukraine	2	1	9
Austria	12	4	9
USA	2	1	9
Australia	7	4	9
Venezuela	5	5	9
Korea, Rep.	9	6	9
Poland	11	6	6
France	15	11	5
Austria	10	8	7
Thailand	17	7	8
Venezuela	4	4	7

Russia	7	5	7
Belgium	10	6	7
Poland	7	3	7
Morocco	16	13	4
Australia	9	7	4
New Zealand	8	8	4
Poland	12	6	0
Greece	4	4	7
USA	2	2	5
India	6	4	7
France	5	2	7
Finland	3	2	7
Romania	8	7	7
Russia	8	6	7
Italy	12	8	4
Russia	3	3	0
Switzerland	10	8	0
India	11	10	0
Costa Rica	7	6	0
Peru	6	4	9
Croatia	7	0	9
Poland	9	5	5
Latvia	6	6	5
Belgium	7	1	9
Germany	2	1	9
Hong Kong, China	7	5	9
Russia	6	2	9
Korea, Rep.	5	2	9
Bulgaria	12	4	9
Spain	2	0	9
Serbia	4	1	9
Italy	5	3	9
Korea, Rep.	3	2	9
India	2	1	9
Jordan	7	7	6
Bulgaria	17	14	6
Russia	4	3	6
Egypt	9	8	5
Israel	8	6	2
Mexico	1	1	5
Russia	10	4	7
Canada	7	3	7
Croatia	2	2	7
South Africa	4	4	7
Cyprus	4	4	7
China, P.R.	9	6	7
New Zealand	5	4	5
Italy	3	2	5
Canada	11	7	8
Italy	11	11	5

France	10	5	5
Costa Rica	8	7	4
Guatemala	12	10	4
New Zealand	7	7	4
New Zealand	8	7	4
Dominican Republic	12	8	4
Egypt	10	7	4
Italy	8	6	7
Mexico	13	9	5
Russia	7	4	7
Netherlands	8	5	7
Chile	8	7	7
Togo	9	6	7
Spain	6	4	7
Israel	11	7	5
USA	2	1	5
Germany	6	5	5
Croatia	4	3	5
Australia	2	2	5
Netherlands	2	2	5
Great Britain	8	8	7
Botswana	8	7	7
Portugal	8	5	5
Russia	5	4	5
Portugal	6	6	5
New Zealand	16	15	4
Poland	16	11	4
Venezuela	8	7	2
Colombia	12	10	2
Canada	21	14	2
Great Britain	7	6	5
Slovakia	19	10	5
Netherlands	7	4	5
Chile	7	5	5
Thailand	10	6	6
Northern Mariana Isl	6	5	6
Hungary	5	4	6
Dominican Republic	12	10	6
USA	6	3	5
France	5	3	2
Turkiye	9	7	2
New Zealand	4	3	2
Vietnam	6	3	5
Mexico	11	7	5
Italy	9	6	5
Canada	10	6	5
Serbia	8	8	5
Japan	4	4	5
India	10	7	4
Hungary	19	11	4

Korea, Rep.	3	3	4
USA	13	11	0
Dominican Republic	7	7	7
Bulgaria	7	4	7
USA	2	1	7
Tunisia	7	5	7
Denmark	11	7	7
USA	2	1	7
Nigeria	2	2	7
USA	4	3	7
Spain	8	6	7
Korea, Rep.	5	4	7
Ecuador	4	4	7
Korea, Rep.	9	7	7
South Africa	13	8	4
Estonia	11	7	2
Vietnam	4	4	2
Thailand	10	8	0
Bulgaria	22	6	5
Belgium	4	2	5
Hungary	6	5	5
Italy	4	2	5
Egypt	12	11	5
Ukraine	14	6	6
Israel	11	8	6
Hungary	5	3	6
Hong Kong, China	9	5	6
France	12	6	5
Georgia	5	5	2
France	13	10	2
Kazakhstan	15	9	0
Italy	5	3	0
Kazakhstan	9	6	0
Uzbekistan	4	4	0
Sweden	10	8	5
Romania	14	8	5
South Africa	2	2	5
Zimbabwe	13	12	5
Turkiye	8	5	5
Nigeria	2	2	5
Colombia	6	3	5
Australia	7	4	5
Russia	8	3	5
Tunisia	6	5	4
Netherlands	7	3	4
Spain	16	10	4
Australia	8	6	4
Hong Kong, China	9	9	4
Cyprus	10	6	2
Slovenia	14	8	2

Sri Lanka	8	8	2
Brazil	12	6	0
Chinese Taipei	8	7	0
Czechia	4	0	7
Colombia	3	1	7
Colombia	13	8	7
Germany	9	6	7
Spain	4	2	7
Germany	2	1	7
Italy	6	3	7
Switzerland	7	2	7
Germany	6	2	7
Croatia	7	4	7
Greece	3	3	7
USA	5	4	7
Thailand	4	1	7
India	7	3	7
Bolivia	4	3	7
Singapore	3	3	7
Serbia	5	1	7
Italy	9	7	7
Russia	3	0	7
Singapore	3	3	7
Pakistan	5	5	4
Indonesia	10	8	4
Great Britain	10	7	4
Mauritius	4	2	2
Israel	8	3	0
New Zealand	6	5	0
Czechia	8	8	0
Chinese Taipei	5	4	0
Korea, Rep.	10	6	0
France	7	4	0
China, P.R.	9	7	0
Mexico	7	6	0
USA	5	3	5
Mexico	5	5	5
USA	3	1	5
Switzerland	11	7	5
Slovenia	6	3	5
Slovenia	7	3	5
USA	8	6	5
Italy	2	2	5
Norway	4	2	5
Spain	5	1	5
Croatia	2	2	5
Netherlands	5	1	5
Estonia	5	1	5
Iran	5	4	6
Zimbabwe	7	7	6

France	9	4	6
Great Britain	4	4	5
Turkiye	7	7	5
Russia	14	8	2
Romania	15	9	2
Hungary	8	6	2
Germany	5	3	0
China, P.R.	6	4	0
Slovenia	11	7	0
Germany	8	3	0
Uzbekistan	5	4	0
Australia	9	7	0
Germany	5	3	0
USA	4	1	0
China, P.R.	2	2	0
Montenegro	7	5	5
Germany	6	5	5
Tunisia	8	4	5
Canada	4	2	5
France	6	3	5
Korea, Rep.	10	3	5
Italy	8	7	5
Nicaragua	1	1	5
USA	7	4	4
Egypt	7	7	2
Russia	7	5	0
Poland	12	9	0
Singapore	13	6	4
France	9	8	4
Honduras	5	5	4
Turkiye	12	10	4
Thailand	11	9	4
Russia	15	8	4
Australia	18	8	4
Belgium	6	4	4
Brazil	4	3	4
India	3	2	4
Finland	4	3	4
South Africa	11	10	2
Estonia	10	6	2
USA	7	5	2
Great Britain	6	4	2
South Africa	4	4	0
Bulgaria	4	4	0
Hong Kong, China	5	5	0
Russia	9	2	6
France	5	4	6
Iran	7	6	2
Colombia	8	6	2
Morocco	6	5	2

Egypt	16	12	2
Israel	9	5	2
El Salvador	9	8	2
France	9	8	0
USA	3	3	0
Slovakia	22	15	5
USA	5	4	5
Bosnia and Herzegov	7	2	5
Bosnia and Herzegov	8	4	5
Tunisia	9	3	5
Tunisia	16	9	5
Australia	9	8	5
Morocco	7	4	5
Germany	9	6	5
Israel	15	8	5
India	12	10	5
Hungary	3	2	5
Greece	5	5	5
Korea, Rep.	7	6	5
Ecuador	1	1	5
Slovakia	7	4	5
Hong Kong, China	10	5	5
Poland	7	4	5
Singapore	1	1	5
India	10	5	5
India	15	12	5
Hungary	4	3	5
Hungary	4	2	5
USA	1	1	5
Russia	4	4	5
Sri Lanka	4	4	5
Sri Lanka	3	3	5
Curacao	2	1	5
Japan	3	3	5
Chile	7	4	5
Monaco	4	3	5
Australia	2	1	5
USA	10	10	4
Tunisia	12	8	4
Morocco	8	5	4
Spain	3	2	2
Colombia	10	8	2
Iran	5	5	2
Korea, Rep.	9	7	2
Serbia	11	9	2
Ireland	2	2	0
Switzerland	10	9	0
Finland	2	2	4
USA	10	5	4
Thailand	9	4	4

Sri Lanka	4	4	4
Singapore	4	4	4
China, P.R.	14	7	4
Georgia	9	8	4
Peru	11	10	4
Mexico	8	7	4
Korea, Rep.	8	5	4
USA	4	3	4
China, P.R.	14	7	4
Finland	2	2	4
Singapore	4	4	4
Sri Lanka	4	4	4
Pakistan	4	4	4
Korea, Rep.	8	5	4
India	8	6	4
Australia	7	5	4
Mexico	8	7	4
Costa Rica	8	4	4
Nigeria	2	2	4
China, P.R.	9	7	2
China, P.R.	8	6	2
Bulgaria	7	4	2
Belarus	5	4	2
France	6	6	0
Czechia	6	2	0
Serbia	5	3	0
Switzerland	14	12	0
USA	3	3	2
Venezuela	2	2	2
Bolivia	3	3	2
Estonia	6	6	2
Argentina	3	3	2
Belgium	3	1	2
Sweden	10	5	2
Egypt	13	10	2
USA	14	13	2
Turkiye	4	2	2
Italy	4	4	2
Kazakhstan	11	8	2
Korea, Rep.	6	4	2
Mexico	9	8	2
USA	5	3	2
Japan	9	7	2
Serbia	4	2	2
Bolivia	4	3	2
Bulgaria	7	6	2
Poland	8	5	2
Korea, Rep.	8	7	0
Spain	10	5	0
Mexico	9	8	0

Serbia	5	2	0
Sweden	10	5	0
Germany	7	4	5
Mexico	8	6	5
Mexico	2	1	5
Mexico	2	0	5
Italy	6	3	5
Dominican Republic	4	4	5
Mexico	6	5	5
Dominican Republic	4	1	5
Malaysia	7	5	5
Mexico	6	6	5
Mexico	5	2	5
Austria	4	0	5
Australia	4	4	5
Slovenia	19	5	5
USA	3	3	5
Switzerland	6	5	5
USA	3	1	5
USA	5	2	5
Switzerland	4	4	5
Slovenia	2	1	5
Great Britain	1	0	5
Russia	5	2	5
China, P.R.	2	2	5
Czechia	10	4	5
Brazil	2	2	5
Italy	2	1	5
Sweden	7	3	5
Croatia	6	0	5
Trinidad & Tobago	4	2	5
Canada	2	1	5
Italy	4	1	5
USA	1	1	5
Serbia	7	4	5
Spain	3	0	5
Romania	6	5	5
South Africa	5	4	5
Germany	2	1	5
USA	1	0	5
Bulgaria	3	0	5
USA	1	1	5
Austria	7	6	5
Italy	4	3	5
Japan	4	1	5
Belgium	6	1	5
Romania	5	2	5
USA	5	1	5
USA	1	0	5
USA	3	3	5

Slovenia	3	0	5
Italy	3	2	5
Great Britain	1	0	5
USA	1	1	5
Czechia	3	2	5
Romania	4	0	5
China, P.R.	6	5	5
Austria	7	6	5
USA	3	2	5
France	3	0	5
Italy	1	1	5
USA	2	0	5
Mexico	4	4	5
Ghana	1	1	5
Venezuela	1	1	5
Japan	1	0	5
New Zealand	8	8	5
Indonesia	5	5	5
Spain	4	1	5
Spain	4	3	5
Brazil	1	1	5
USA	1	1	5
Chile	1	0	5
Hungary	2	2	5
Russia	7	5	5
Argentina	6	2	5
Venezuela	4	4	5
Bulgaria	7	2	5
Austria	9	4	5
Colombia	2	0	5
Peru	3	3	5
USA	1	0	5
USA	5	3	5
Peru	3	2	5
Cyprus	15	9	2
France	9	5	2
USA	7	6	2
Sweden	5	2	2
Japan	7	5	2
Romania	6	4	2
Peru	7	7	2
France	11	7	2
Spain	6	6	2
USA	2	2	0
USA	2	2	0
Sweden	10	6	0
Israel	11	9	0
USA	8	5	0
USA	1	1	0
Canada	9	6	0

Argentina	2	2	4
Nicaragua	11	6	4
Indonesia	3	3	4
Turkiye	2	2	4
Argentina	7	4	4
Japan	11	10	4
Hong Kong, China	6	4	4
Bolivia	7	7	4
Argentina	4	4	4
Syria	3	3	4
Korea, Rep.	6	4	4
Albania	4	4	4
Barbados	7	7	4
Spain	9	5	4
Uzbekistan	7	3	4
Israel	14	11	4
Botswana	4	4	4
South Africa	12	11	4
Thailand	2	2	4
Germany	15	6	4
Indonesia	11	9	0
Belarus	10	6	0
Russia	7	4	0
France	4	2	0
Malaysia	13	10	2
Poland	10	6	2
Ghana	6	4	2
Italy	10	6	2
New Zealand	8	8	2
Bosnia and Herzegov	4	3	2
Russia	4	4	2
Japan	8	5	2
Hungary	6	5	2
Italy	12	9	0
Japan	3	2	0
Romania	7	2	0
Romania	12	4	0
Austria	5	3	0
Singapore	3	3	2
Colombia	6	6	2
Thailand	7	6	2
Algeria	8	3	2
New Zealand	13	12	2
Armenia	3	2	2
Serbia	14	7	2
Austria	7	4	2
Ukraine	13	8	2
Japan	2	2	2
France	7	5	2
Great Britain	2	2	2

Italy	3	3	2
USA	12	6	2
Nigeria	2	2	2
Iran	2	2	2
Costa Rica	8	4	2
Chinese Taipei	7	3	2
India	10	6	2
France	7	5	2
Algeria	8	3	2
Iran	2	2	2
Tunisia	17	6	2
Morocco	6	3	0
Nigeria	2	2	4
Canada	3	2	4
Argentina	3	3	4
India	6	4	4
Pakistan	5	4	4
Australia	9	7	4
Thailand	2	2	4
Tajikistan	11	5	4
Switzerland	8	3	4
Russia	11	2	4
Mauritius	6	3	4
New Zealand	3	3	4
Cote D'Ivoire	5	2	4
Kuwait	5	5	4
France	8	5	4
Great Britain	8	4	4
Netherlands	8	5	4
Singapore	3	2	4
Russia	15	2	4
Belgium	3	2	4
Martinique	4	2	4
France	2	2	4
USA	5	3	4
Portugal	7	4	4
Lebanon	6	2	4
Russia	8	4	4
New Zealand	4	2	4
Russia	7	1	4
Georgia	3	1	4
France	3	1	4
Sri Lanka	3	3	4
Spain	12	6	4
Italy	8	5	0
Netherlands	7	5	0
Hungary	4	3	0
Slovakia	5	5	0
Brazil	4	5	0
Egypt	8	7	2

Chile	8	8	2
Australia	14	6	2
Tajikistan	4	2	2
Egypt	11	8	2
Costa Rica	13	6	2
Portugal	12	8	2
Venezuela	5	2	2
Tunisia	13	5	2
USA	5	4	2
France	5	3	2
Sweden	3	4	2
Bolivia	9	4	2
eSwatini	3	3	0
Argentina	4	3	0
Egypt	8	4	0
Tunisia	5	1	0
Russia	1	1	0
Italy	2	1	0
Tunisia	3	2	0
Russia	2	1	0
Turkiye	7	6	0
Great Britain	8	8	0
Czechia	3	2	0
Romania	4	2	2
Nigeria	2	2	2
Korea, Rep.	2	1	2
Italy	9	7	2
Poland	15	9	2
Kosovo	4	3	2
Kazakhstan	13	9	2
Thailand	13	10	2
Canada	3	1	2
Argentina	3	3	2
Mexico	10	5	2
Morocco	1	1	2
Egypt	13	13	2
Thailand	12	7	2
Colombia	15	6	2
India	14	9	2
Venezuela	1	1	2
China, P.R.	10	7	2
Hungary	7	4	2
Finland	2	2	2
Canada	2	1	2
South Africa	10	6	2
Colombia	5	3	2
Hong Kong, China	7	5	2
Philippines	2	2	2
India	4	1	2
Chinese Taipei	7	2	2

Canada	4	4	2
Uzbekistan	7	5	2
Kuwait	5	5	2
Venezuela	3	3	2
Singapore	3	3	2
France	1	1	2
Zimbabwe	4	4	2
Zimbabwe	8	7	2
Morocco	1	1	2
Thailand	13	10	2
Canada	3	1	2
Singapore	3	3	2
Mexico	10	5	2
Hong Kong, China	7	5	2
Egypt	13	13	2
India	14	9	2
Kuwait	5	5	2
Chinese Taipei	7	2	2
Venezuela	3	3	2
Spain	11	6	2
Australia	6	6	0
Romania	7	5	0
Israel	8	3	0
Australia	7	7	0
USA	2	3	0
USA	4	1	0
Canada	1	1	0
Paraguay	5	4	0
Canada	8	3	0
Austria	2	2	0
Belarus	2	3	0
Czechia	4	4	0
Brazil	4	2	0
Greece	7	6	0
USA	1	1	0
Iran	5	4	0
China, P.R.	8	6	0
Romania	6	3	0
Venezuela	2	2	0
Russia	10	6	0
New Zealand	11	10	0
Japan	18	11	0
Japan	5	4	0
Poland	11	5	0
Thailand	11	9	0
Great Britain	8	4	0
Poland	9	4	0
China, P.R.	8	5	0
Korea, Rep.	8	4	0
Peru	6	1	0

Bosnia and Herzegov	5	3	2
Canada	4	2	2
USA	1	1	2
Canada	8	4	2
Korea, Rep.	4	1	2
Montenegro	4	4	2
India	2	1	2
Korea, Rep.	5	4	2
Egypt	11	10	2
Korea, Rep.	6	4	2
Botswana	3	2	2
Argentina	4	3	2
Zimbabwe	5	5	2
Philippines	7	3	2
Paraguay	1	1	2
Venezuela	1	1	2
India	5	4	2
Japan	7	3	2
Greece	1	1	2
Montenegro	2	2	2
Cyprus	5	3	2
Belgium	3	1	2
Paraguay	1	1	2
Hungary	4	2	2
Rwanda	2	2	2
India	7	5	2
Italy	4	2	2
Norway	7	5	2
Costa Rica	11	5	2
India	10	5	2
India	1	1	2
Finland	2	3	2
Mexico	10	6	2
Trinidad & Tobago	5	5	2
Hungary	5	2	2
Bulgaria	7	3	2
Italy	5	1	2
Ukraine	3	1	2
Japan	2	1	2
Canada	7	5	2
Italy	1	1	2
Cuba	1	1	2
Finland	2	3	2
Switzerland	2	1	2
Zimbabwe	5	4	2
Serbia	11	7	2
Bolivia	4	3	2
Mexico	11	7	2
Great Britain	4	2	2
USA	8	4	2

Algeria	3	1	2
Turkiye	1	1	2
Spain	1	1	2
Bosnia and Herzegov	2	2	2
Serbia	9	5	2
Australia	9	6	2
USA	3	3	2
Spain	1	1	2
Greece	1	1	2
USA	8	4	2
Great Britain	4	2	2
Hungary	5	2	2
Greece	1	1	2
Australia	1	1	2
Algeria	3	1	2
Italy	4	3	2
Paraguay	1	1	2
Argentina	6	2	2
Iran	2	1	2
Japan	2	1	2
USA	6	6	2
Venezuela	3	2	2
Canada	7	5	2
Great Britain	4	3	2
India	1	1	2
Trinidad & Tobago	5	5	2
USA	1	1	2
Finland	2	3	2
Mexico	10	6	2
Barbados	7	6	2
Egypt	9	7	2
Japan	3	1	2
Costa Rica	11	5	2
India	2	1	2
Japan	5	3	2
USA	6	5	2
Italy	1	1	2
Germany	10	8	2
India	3	3	2
Thailand	7	3	2
Italy	5	1	2
Turkiye	1	1	2
France	3	2	2
Dominican Republic	3	2	2
Zimbabwe	5	4	2
Turkiye	5	2	2
China, P.R.	2	2	2
Rwanda	2	2	2
Argentina	3	3	2
Serbia	11	6	2

Japan	9	5	2
Serbia	11	7	2
Argentina	5	3	2
Singapore	4	3	2
Nigeria	1	1	2
Tunisia	10	4	2
USA	5	3	2
Armenia	4	2	2
Nigeria	1	1	2
Bosnia and Herzegov	2	2	2
Italy	6	4	2
Korea, Rep.	6	4	2
Israel	8	5	2
Morocco	7	2	2
Sweden	6	3	2
Japan	7	3	2
Colombia	3	1	2
Great Britain	4	1	2
Thailand	5	3	2
North Macedonia	3	3	0
Canada	5	3	0
Serbia	5	2	0
Ukraine	11	9	0
Croatia	3	2	0
USA	5	3	0
Great Britain	5	5	0
Sweden	10	4	0
St. Lucia	8	4	0
Estonia	9	4	0
Uzbekistan	3	3	0
Germany	6	2	0
South Africa	4	3	0
Egypt	8	5	0
Portugal	10	4	0
Bulgaria	11	5	0
Mexico	6	4	0
Cyprus	6	5	0
Ghana	5	3	0
Great Britain	5	5	0
Switzerland	14	11	0
Great Britain	5	5	0
Lithuania	5	3	0
Chile	7	6	0
Ukraine	9	8	0
Korea, Rep.	7	4	0
Japan	3	3	0
Switzerland	8	6	0
Germany	13	7	0
Korea, Rep.	7	4	0
Chinese Taipei	9	8	0

Belgium	7	5	0
South Africa	4	4	0
Singapore	4	4	0
Korea, Rep.	1	1	2
Austria	4	2	2
Pakistan	2	2	2
Indonesia	6	3	2
Colombia	3	3	2
Canada	4	3	2
Iran	3	1	2
Russia	1	1	2
Serbia	8	4	2
Australia	1	1	2
Uzbekistan	4	2	2
France	2	0	2
Venezuela	1	1	2
Guam	2	1	2
New Zealand	2	1	2
Germany	6	3	2
Ukraine	6	2	2
Singapore	4	3	2
Kosovo	4	2	2
Korea, Rep.	7	6	2
Peru	9	7	2
Mexico	4	2	2
Austria	5	1	2
Austria	6	3	2
Slovakia	6	3	2
Iran	3	2	2
Turkiye	7	4	2
Pakistan	2	2	2
Australia	1	1	2
Colombia	2	1	2
Austria	4	2	2
Korea, Rep.	4	2	2
Hong Kong, China	4	3	2
Mozambique	2	2	2
Norway	5	3	2
Russia	1	1	2
Australia	2	2	2
Indonesia	6	3	2
Morocco	4	2	2
Belgium	10	1	2
Russia	4	0	2
Italy	2	0	2
Kazakhstan	7	4	2
Japan	2	0	2
Uzbekistan	6	0	2
Ukraine	1	1	2
Russia	13	3	2

Denmark	1	0	2
Northern Mariana Isl	2	2	2
Finland	4	2	2
South Africa	9	5	2
France	10	7	2
Switzerland	5	1	2
Colombia	4	1	2
Poland	8	3	2
Netherlands	8	5	2
Colombia	4	2	2
Finland	2	2	2
Czechia	8	5	2
USA	6	4	2
Ukraine	1	1	2
India	3	3	2
France	3	0	2
France	3	1	2
Spain	1	1	2
France	2	0	2
Grenada	2	2	2
Pakistan	6	6	2
France	6	3	2
USA	1	1	2
Norway	2	2	2
Russia	2	1	2
Peru	1	1	2
Israel	18	4	2
South Africa	6	2	2
South Africa	2	1	2
Italy	3	0	2
Singapore	3	2	2
Spain	4	2	2
Russia	1	1	2
Venezuela	1	1	2
Belarus	5	0	2
Uzbekistan	4	1	2
USA	3	2	2
France	2	2	2
Canada	6	3	2
Norway	4	2	2
Tunisia	8	3	2
Honduras	2	2	2
Hungary	2	1	2
Chile	3	2	2
Morocco	8	3	2
Croatia	10	5	2
Great Britain	3	1	2
Romania	6	1	2
Bolivia	6	4	2
Canada	6	4	2

Canada	7	5	2
South Africa	5	2	2
Italy	2	1	2
Belarus	8	4	2
Uzbekistan	7	3	2
USA	8	1	2
Canada	3	1	2
Egypt	3	2	2
Chile	3	2	2
Canada	7	5	2
Russia	1	1	2
Colombia	4	4	2
Kazakhstan	7	4	2
Australia	1	1	2
Norway	4	2	2
Mexico	4	1	2
Russia	1	1	2
Singapore	4	3	2
Korea, Rep.	4	2	2
India	4	2	2
Korea, Rep.	7	6	2
Portugal	9	2	2
Romania	6	1	2
Peru	9	6	2
Russia	2	1	2
Singapore	4	1	2
Austria	6	3	2
Iran	2	1	2
France	2	0	2
Armenia	3	1	2
Norway	5	3	2
Japan	2	1	2
Pakistan	2	2	2
USA	1	1	2
Germany	6	3	2
Finland	3	2	2
Ukraine	1	1	2
Japan	5	2	2
Great Britain	3	1	2
Cuba	1	1	2
Thailand	2	1	2
Iran	2	2	2
Ukraine	6	2	2
Cuba	1	1	2
France	6	3	2
Bosnia and Herzegov	5	2	2
Uzbekistan	7	3	2
Puerto Rico	3	0	2
China, P.R.	7	5	2
Singapore	2	0	2

Slovakia	6	3	2
Japan	1	0	2
Uzbekistan	6	0	2
Colombia	2	1	2
Belgium	10	1	2
Peru	1	1	2
Brazil	1	1	2
Bolivia	6	4	2
Italy	2	0	2
Venezuela	1	1	2
Netherlands	8	5	2
Canada	3	1	2
Denmark	1	0	2
Honduras	2	2	2
Spain	5	2	2
Bolivia	4	2	2
Mozambique	2	2	2
India	2	1	2
Iran	3	1	2
Canada	7	1	2
Russia	4	0	2
Venezuela	5	5	2
Australia	3	3	2
France	6	3	2
Iran	3	2	2
Philippines	4	2	2
Austria	5	1	2
Finland	1	1	2
Turkiye	7	4	2
Ghana	4	2	2
Mexico	7	1	2
Panama	5	4	2
Uzbekistan	4	1	2
China, P.R.	7	5	2
Switzerland	5	1	2
Indonesia	2	2	2
India	1	0	2
Belarus	5	0	2
Peru	3	2	2
Argentina	1	1	2
Nigeria	2	2	2
Hong Kong, China	3	1	2
New Zealand	2	1	2
Zimbabwe	3	3	2
Italy	2	1	2
Belarus	8	4	2
El Salvador	6	5	2
Thailand	1	0	2
South Africa	1	1	2
Croatia	10	5	2

Nigeria	2	1	2
Israel	18	4	2
Indonesia	1	0	2
Germany	8	2	2
Croatia	4	1	2
Italy	3	0	2
Czechia	3	1	2
Czechia	3	1	2
Tunisia	1	1	2
Canada	2	0	2
Paraguay	4	3	2
Serbia	8	4	2
Uzbekistan	3	1	2
Brazil	11	2	2
Great Britain	3	1	2
Great Britain	1	1	0
Switzerland	8	2	0
Canada	4	2	0
USA	2	1	0
Mexico	9	8	0
Dominican Republic	5	4	0
Greece	2	1	0
Jamaica	1	1	0
Serbia	5	1	0
Italy	3	3	0
Netherlands	2	1	0
USA	1	1	0
China, P.R.	2	2	0
Switzerland	2	1	0
Hong Kong, China	10	3	0
Poland	1	1	0
USA	1	2	0
Slovenia	5	3	0
Greece	1	1	0
Honduras	7	6	0
Russia	6	4	0
Bosnia and Herzegov	2	1	0
USA	8	6	0
Mexico	9	4	0
Russia	9	4	0
Norway	6	3	0
Mexico	4	4	0
Great Britain	2	1	0
Colombia	8	5	0
Sweden	4	1	0
Russia	3	2	0
Spain	5	2	0
Vietnam	4	2	0
Spain	4	1	0
Spain	3	1	0

Italy	6	1	0
Austria	3	1	0
Estonia	1	1	0
Trinidad & Tobago	1	1	0
Mexico	9	5	0
Brazil	3	2	0
New Zealand	3	3	0
Tunisia	5	2	0
Romania	2	1	0
Greece	3	2	0
Estonia	1	1	0
Belgium	5	2	0
Mexico	6	5	0
Slovenia	4	2	0
Egypt	9	6	0
USA	0	1	0
Germany	5	3	0
India	4	2	0
Portugal	10	3	0
Sweden	4	2	0
Italy	4	3	0
Netherlands	2	1	0
Costa Rica	6	1	0
Belgium	2	1	0
Norway	6	2	0
Norway	4	2	0
Spain	7	3	0
Netherlands	3	1	0
Cyprus	4	2	0
Croatia	9	5	0
Slovenia	0	1	0
Iran	6	6	0
China, P.R.	9	5	0
Norway	8	3	0
Sweden	3	3	0
Kazakhstan	4	1	0
Malaysia	9	7	0
Sweden	11	6	0
Costa Rica	6	3	0
Poland	6	1	0
Czechia	4	1	0
Ukraine	2	1	0
Croatia	5	3	0
Egypt	3	3	0
Poland	9	4	0
Japan	2	1	0
Sweden	2	1	0
Colombia	6	5	0
India	5	4	0
Canada	5	2	0

Germany	4	2	0
Mexico	7	5	0
USA	11	7	0
Czechia	5	2	0
Kuwait	5	5	0
Colombia	3	3	0
Venezuela	2	2	0
Venezuela	1	1	0
Israel	5	3	0
Venezuela	3	3	0
Brazil	14	6	0
Panama	7	4	0
Argentina	5	4	0
Brazil	8	2	0
Korea, Rep.	6	4	0
Russia	6	3	0
Chile	4	4	0
Russia	3	1	0
Chinese Taipei	8	8	0
India	1	3	0
Japan	9	6	0
Trinidad & Tobago	7	6	0
El Salvador	10	7	0
Pakistan	4	2	0
Thailand	6	3	0
Trinidad & Tobago	4	3	0
Mexico	7	4	0
Kuwait	5	5	0
Israel	4	2	0
Thailand	7	6	0
Thailand	6	2	0
Germany	4	2	0
South Africa	8	7	0
Serbia	14	6	0
Colombia	3	3	0
Venezuela	2	2	0
Slovakia	1	1	0
Tunisia	6	2	0
USA	4	2	0
Great Britain	8	5	0
Sweden	4	3	0
India	5	4	0
India	10	3	0
Finland	5	3	0
Great Britain	3	2	0
Slovakia	15	5	0
South Africa	4	4	0
Ireland	3	1	0
USA	11	7	0
Iran	2	2	0

Czechia	5	2	0
Serbia	8	3	0
Serbia	5	3	0
Russia	10	4	0
Italy	5	3	0
Israel	13	6	0
Turkiye	5	3	0
Mexico	7	5	0
Spain	4	4	0
Great Britain	1	1	0
Hungary	7	3	0
India	12	8	0
Russia	5	3	0
Italy	2	1	0
Italy	6	3	0
Ireland	4	3	0
Nepal	2	2	0
China, P.R.	9	7	0
China, P.R.	5	4	0
Tunisia	7	1	0
Sweden	12	5	0
Romania	4	2	0
Portugal	7	3	0
Italy	1	1	0
Ukraine	2	2	0
Ukraine	3	2	0
Japan	1	1	0
Japan	7	5	0
Czechia	5	2	0
Israel	5	3	0
Uzbekistan	5	2	0
Sweden	3	2	0
Russia	4	3	0
Slovenia	4	1	0
Serbia	3	2	0
Hungary	3	2	0
Bulgaria	3	2	0
Serbia	5	2	0
Turkiye	9	6	0
Serbia	3	2	0
Russia	6	2	0
Ukraine	3	2	0
Italy	7	3	0
Romania	7	3	0
Slovenia	4	3	0
Thailand	4	6	0
India	1	1	0
Indonesia	5	3	0
North Macedonia	8	3	0
Italy	9	5	0

Cyprus	12	5	0
South Africa	4	3	0
Great Britain	4	3	0
USA	7	2	0
Latvia	7	2	0
France	7	1	0
Portugal	4	2	0
France	2	2	0
Portugal	3	2	0
China, P.R.	5	7	0
Curacao	6	4	0
Ukraine	15	8	0
Sri Lanka	3	2	0
Latvia	4	3	0
Slovakia	4	1	0
Moldova	4	3	0
Russia	1	1	0
Ireland	8	2	0
Switzerland	5	1	0
Serbia	8	4	0
Belarus	10	2	0
Canada	2	3	0
USA	4	3	0
Russia	6	4	0
India	5	2	0
Russia	3	2	0
Italy	4	2	0
Russia	2	2	0
Indonesia	5	4	0
Israel	14	7	0
Bosnia and Herzegov	3	2	0
Romania	6	4	0
Slovakia	5	4	0
Lebanon	9	4	0
Moldova	8	3	0
Serbia	7	1	0
Cyprus	7	2	0
Serbia	3	3	0
Iran	3	3	0
Norway	11	7	0
Norway	10	7	0
Denmark	7	4	0
Bulgaria	4	2	0
Greece	1	1	0
Ukraine	20	9	0
Ukraine	1	1	0
USA	4	1	0
Germany	3	1	0
Greece	2	1	0
Colombia	5	2	0

Israel	2	1	0
India	4	2	0
Italy	4	3	0
Kazakhstan	2	1	0
Spain	5	3	0
Ireland	2	1	0
China, P.R.	6	4	0
India	18	11	0
Israel	6	2	0
Germany	12	4	0
Trinidad & Tobago	6	6	0
Ireland	3	1	0
Greece	4	3	0
India	0	1	0
Great Britain	7	7	0
Iran	4	2	0
China, P.R.	5	1	0
Korea, Rep.	4	3	0
Korea, Rep.	6	2	0
South Africa	5	2	0
Bangladesh	2	2	0
Morocco	6	4	0
Morocco	7	3	0
Cyprus	7	4	0
Ukraine	5	1	0
Brazil	5	3	0
Paraguay	6	3	0
Ecuador	3	3	0
Brazil	5	4	0
Bolivia	14	6	0
Brazil	10	3	0
Chile	4	2	0
Brazil	2	1	0
Paraguay	10	8	0
Peru	2	1	0
Paraguay	1	1	0
Turkiye	5	4	0
Tunisia	12	4	0
Spain	3	2	0
Japan	3	2	0
USA	5	1	0
USA	8	6	0
Sweden	3	1	0
Costa Rica	8	3	0
Thailand	6	5	0
India	8	5	0
USA	4	2	0
India	1	1	0
Great Britain	2	2	0
Venezuela	1	1	0

France	2	1	0
Puerto Rico	3	1	0
Italy	7	1	0
Colombia	5	2	0
Zimbabwe	6	6	0
Great Britain	8	5	0
USA	1	1	0
India	7	4	0
Indonesia	3	3	0
Russia	12	5	0
Russia	1	1	0
Japan	1	1	0
Costa Rica	9	4	0
USA	8	4	0
Botswana	6	5	0
Japan	5	3	0
Brazil	7	2	0
Ecuador	3	2	0
Switzerland	1	1	0
China, P.R.	7	4	0
China, P.R.	2	2	0
Dominican Republic	8	6	0
Australia	4	4	0
USA	5	2	0
Chinese Taipei	5	3	0
Great Britain	3	2	0
France	2	2	0
Uzbekistan	4	1	0
USA	3	1	0
Madagascar	1	1	0
Mexico	14	9	0
Bahamas	2	2	0
Chinese Taipei	7	4	0
Korea, Rep.	3	2	0
Serbia	2	1	0
USA	1	1	0
India	5	4	0
Togo	6	4	0
Japan	5	3	0
Pakistan	1	1	0
Venezuela	1	1	0
Colombia	2	2	0
Chile	16	13	0
Australia	4	4	0
Pakistan	2	2	0
India	2	2	0
Spain	6	4	0
Paraguay	1	1	0
Argentina	5	2	0
Bulgaria	15	4	0

Canada	11	6	0
Iran	3	3	0
Great Britain	8	8	0
India	3	2	0
Chile	2	2	0
Venezuela	4	3	0
USA	2	2	0
New Zealand	4	3	0
Korea, Rep.	8	3	0
India	6	4	0
New Zealand	4	4	0
Guatemala	2	2	0
USA	2	1	0
Finland	1	1	0
Honduras	3	3	0
Indonesia	1	1	0
Zimbabwe	4	4	0
Ghana	3	1	0
Vanuatu	2	2	0
New Zealand	4	4	0
New Zealand	5	3	0
Pakistan	2	2	0
Zimbabwe	3	3	0
Sweden	3	1	0
Japan	1	1	0
Hungary	3	1	0
Finland	2	2	0
Thailand	3	2	0
Thailand	6	4	0
Tunisia	4	1	0
Tunisia	7	6	0
Belarus	6	2	0
Nigeria	1	1	0
Vanuatu	1	1	0
Brazil	4	3	0
France	2	2	0
Japan	1	1	0
Honduras	1	1	0
Australia	3	3	0
Ukraine	3	1	0
USA	5	2	0
South Africa	6	2	0
Chinese Taipei	2	2	0
Togo	5	4	0
South Africa	4	1	0
Venezuela	1	1	0
Russia	3	2	0
Russia	2	1	0
India	7	5	0
Ghana	3	2	0

Bolivia	2	1	0
Bahamas	1	1	0
Ecuador	2	2	0
India	3	2	0
Argentina	14	11	2360
Denmark	15	12	2510
Czechia	9	8	2145
Japan	15	12	2250
France	18	16	1870
USA	13	12	1565
USA	7	2	1390
France	14	12	1230
USA	12	12	1105
Japan	19	19	855
Czechia	7	7	945
Switzerland	22	19	1025
France	18	13	945
Switzerland	15	10	1025
Switzerland	17	16	725
Canada	10	8	810
South Africa	19	19	940
Italy	19	18	730
Belgium	15	13	765
Czechia	14	14	730
Argentina	17	17	805
Ukraine	11	11	870
Hungary	17	16	780
Cote D'Ivoire	19	18	790
USA	7	7	745
Latvia	24	21	800
China, P.R.	23	21	680
Australia	17	16	620
China, P.R.	14	12	690
Spain	10	8	705
Brazil	10	7	560
Italy	12	12	690
Great Britain	21	21	605
Belarus	13	13	540
Spain	8	2	760
USA	15	15	350
Sweden	18	18	677
Spain	14	14	610
Poland	17	14	555
France	22	17	620
Canada	15	13	620
Argentina	19	18	575
USA	15	13	325
Czechia	11	11	385
Romania	17	16	520
Poland	25	21	411

USA	25	23	405
Japan	12	11	450
Austria	21	20	580
Great Britain	20	16	485
Italy	16	15	510
Netherlands	22	18	540
Australia	9	9	380
Ukraine	9	9	480
Algeria	12	11	565
Romania	21	19	475
USA	17	15	410
Hong Kong, China	19	18	469
France	18	16	470
Argentina	13	13	440
Japan	18	15	495
Brazil	18	18	345
Germany	21	19	431
Great Britain	19	12	440
France	15	13	440
Japan	12	11	436
Germany	23	19	400
USA	20	18	436
USA	15	10	350
France	10	7	325
Australia	27	23	342
Romania	15	12	320
France	22	21	285
Australia	18	17	300
Australia	8	7	330
Brazil	26	26	205
Switzerland	22	18	278
France	9	7	255
USA	14	12	310
Germany	22	21	307
USA	11	10	316
Netherlands	13	11	286
Jamaica	20	15	282
Japan	14	13	315
Brazil	18	14	211
South Africa	10	7	256
Germany	15	14	198
Japan	19	19	164
Brazil	11	10	185
Finland	6	5	230
Italy	18	16	180
Switzerland	24	21	181
France	19	15	224
Uruguay	18	17	181
Spain	20	18	166
Romania	12	9	145

Great Britain	8	7	141
Netherlands	13	11	110
Argentina	13	11	97
USA	22	20	85
South Africa	8	7	70
Vietnam	7	6	90
Chinese Taipei	9	7	3620
France	10	9	2080
Argentina	11	10	2050
Bulgaria	16	13	1560
USA	9	7	1810
Argentina	16	16	1435
Colombia	16	14	1460
Italy	17	16	1565
USA	5	1	1490
Brazil	12	10	1240
Finland	18	16	975
Great Britain	8	6	1100
Romania	21	19	950
Australia	17	16	820
Japan	13	13	730
Spain	15	15	910
France	12	12	535
Belgium	19	14	820
Dominican Republic	13	13	865
Turkiye	16	14	630
Czechia	15	14	805
Great Britain	16	13	685
Spain	12	12	890
Czechia	17	16	770
China, P.R.	14	13	825
Czechia	9	9	480
Belgium	18	16	810
USA	10	10	780
France	17	15	775
Netherlands	12	13	765
France	13	13	760
Switzerland	15	15	655
USA	13	13	755
USA	15	12	580
France	17	16	730
Canada	18	17	645
Great Britain	6	6	670
Uzbekistan	19	17	585
USA	23	21	630
USA	19	11	700
Netherlands	17	15	510
Japan	14	12	704
USA	20	17	500
Poland	11	9	725

Brazil	15	13	675
Argentina	15	12	630
USA	16	16	505
Italy	14	13	590
Argentina	23	19	521
Argentina	20	17	560
USA	20	20	520
Japan	16	13	565
Chinese Taipei	11	10	480
Brazil	21	21	480
Great Britain	18	16	596
Great Britain	16	15	495
Korea, Rep.	16	14	555
Kazakhstan	8	6	475
Switzerland	12	12	495
Belgium	19	13	450
South Africa	13	13	495
Japan	17	16	371
Brazil	10	9	450
India	12	9	421
Netherlands	19	16	395
Poland	13	11	430
Canada	17	17	440
Germany	10	7	480
Brazil	12	8	435
USA	8	8	380
Germany	18	16	405
Italy	17	14	421
Croatia	22	20	310
Italy	16	12	400
Argentina	14	14	410
USA	15	15	410
Argentina	16	11	431
Hong Kong, China	22	18	416
Germany	13	10	175
Argentina	14	13	285
Brazil	17	12	310
Turkiye	22	21	360
Poland	22	18	325
Netherlands	21	18	376
Romania	15	15	370
USA	12	11	340
France	11	10	335
Croatia	18	14	252
France	19	17	335
France	17	14	325
Peru	16	16	190
Korea, Rep.	16	16	286
USA	14	13	276
Argentina	11	10	230

Estonia	26	20	290
Brazil	19	14	300
USA	18	17	281
Kazakhstan	7	6	316
Philippines	16	15	224
USA	20	17	165
Malaysia	20	20	153
USA	15	13	260
Sweden	15	12	137
USA	8	8	165
Venezuela	18	16	100
Romania	8	7	125
Brazil	9	7	80
USA	17	9	138
USA	12	7	35
Argentina	15	13	1110
China, P.R.	7	5	920
Australia	6	5	987,5
Kazakhstan	14	14	860
Finland	10	6	872,5
Hungary	6	5	790
Serbia	11	10	805
Austria	10	8	670
Japan	12	12	680
Korea, Rep.	23	22	625
Argentina	14	12	680
France	7	4	740
France	17	15	662,5
USA	11	11	577,5
USA	14	11	610
Chinese Taipei	18	18	655
Great Britain	10	9	645
Chinese Taipei	11	9	385
Colombia	18	18	412,5
Brazil	10	10	580
Czechia	15	14	460
France	19	18	485
Argentina	17	17	560
Argentina	19	15	520
Russia	14	14	475
USA	17	14	470
France	9	9	515
Czechia	19	19	412,5
Japan	19	18	475
USA	10	8	492,5
Czechia	16	15	420
Israel	8	7	500
Mexico	17	17	475
USA	22	19	480
Belgium	12	11	440

USA	15	13	435
Romania	20	16	430
Portugal	9	9	407,5
USA	8	8	390
USA	10	10	430
USA	15	14	380
USA	11	10	350
Great Britain	17	14	470
USA	10	10	395
Argentina	13	12	462,5
USA	19	18	435
Dominican Republic	19	19	412,5
Cyprus	10	9	415
Great Britain	17	16	427,5
Spain	19	18	410
Italy	17	15	375
Ireland	12	12	400
Brazil	25	19	390
Brazil	20	19	345
Germany	6	4	392,5
France	11	10	330
Tunisia	15	14	365
Romania	17	16	380
Japan	18	14	355
USA	13	13	330
India	18	17	305
Russia	11	10	370
Russia	7	6	300
Brazil	25	22	300
Netherlands	16	15	245
Australia	20	17	290
Bulgaria	24	20	275
Great Britain	14	13	310
Japan	15	13	260
USA	17	13	282,5
Brazil	19	14	305
Australia	13	11	280
USA	20	18	250
Great Britain	16	11	285
Netherlands	18	14	210
USA	19	18	280
Korea, Rep.	17	17	275
Japan	11	9	245
France	8	7	280
South Africa	15	15	260
Russia	8	8	270
France	14	8	250
France	15	15	200
USA	17	16	240
Chile	13	11	185

Mexico	18	17	220
Great Britain	19	16	235
USA	13	12	190
Italy	15	13	180
Colombia	13	11	210
Germany	23	18	215
New Zealand	13	11	180
Chinese Taipei	13	12	180
USA	20	19	180
Japan	9	9	110
Venezuela	25	23	140
USA	9	7	195
Brazil	21	17	150
USA	19	15	150
Brazil	6	5	120
USA	20	15	125
USA	9	8	135
USA	17	9	105
Serbia	14	12	1312,5
Greece	8	7	1092,5
Japan	13	11	1000
Canada	7	6	920
USA	13	12	885
China, P.R.	15	15	905
France	10	10	865
Uzbekistan	8	7	777,5
Canada	13	12	610
Germany	11	8	732,5
Australia	6	5	650
Japan	17	17	560
Estonia	12	12	555
Austria	10	8	640
USA	10	9	615
Germany	15	15	600
Spain	10	10	585
Israel	20	15	455
Germany	16	14	480
Argentina	16	14	550
Italy	18	17	565
Portugal	18	17	520
Germany	15	14	540
Argentina	16	15	530
Brazil	15	15	520
Australia	16	16	507,5
Canada	22	19	400
USA	10	10	485
Great Britain	16	14	510
Egypt	12	12	480
Uzbekistan	13	13	490
Japan	15	13	442,5

Chinese Taipei	12	10	420
Argentina	24	23	445
Japan	17	17	342,5
France	18	18	410
Poland	16	14	370
Morocco	13	13	390
USA	14	14	450
Poland	17	14	360
Turkiye	20	18	360
China, P.R.	20	16	375
USA	22	21	387,5
USA	13	12	312,5
Great Britain	14	11	382,5
Australia	12	10	332,5
Philippines	11	11	395
Brazil	14	13	320
USA	15	11	425
Hungary	18	12	395
Argentina	21	21	360
Great Britain	17	13	380
Great Britain	15	13	345
France	19	18	355
France	10	10	365
Spain	13	12	317,5
Russia	14	12	320
Japan	15	14	325
Great Britain	21	19	315
France	16	14	290
France	11	9	330
USA	9	8	305
Hong Kong, China	11	10	300
Romania	21	19	320
Russia	11	10	325
USA	11	10	300
Belgium	12	10	295
Hungary	6	5	330
USA	19	15	295
Mexico	17	16	310
USA	20	19	280
Italy	12	10	290
Slovakia	9	8	190
Chinese Taipei	9	8	280
USA	19	17	200
Estonia	14	10	240
USA	11	9	245
Brazil	16	13	270
Brazil	13	11	270
China, P.R.	13	7	230
Japan	16	12	220
Great Britain	8	8	190

Puerto Rico	13	11	210
Russia	9	9	180
Italy	14	12	225
USA	9	8	170
USA	12	12	180
Canada	12	11	140
Great Britain	17	13	190
Italy	11	9	125
USA	8	7	1275
Norway	18	15	860
Hungary	16	14	830
Korea, Rep.	8	7	940
Chile	19	17	800
Brazil	7	7	837,5
Sweden	9	8	850
Serbia	14	14	702,5
USA	11	10	705
Spain	11	10	680
France	11	10	730
Australia	8	7	665
Vietnam	10	9	635
Greece	14	14	660
Korea, Rep.	15	15	635
Australia	20	18	622,5
Argentina	16	16	580
Slovakia	11	9	545
Philippines	18	16	510
USA	6	6	555
Argentina	19	17	535
Uzbekistan	14	14	555
Great Britain	21	21	490
Finland	14	13	490
USA	21	20	495
Argentina	20	18	510
France	18	16	460
Egypt	16	16	480
USA	13	12	480
Italy	16	15	452,5
Japan	16	13	420
France	18	17	410
Canada	18	17	440
China, P.R.	18	16	440
Estonia	20	16	475
Canada	14	14	412,5
Slovakia	16	14	390
Russia	17	16	430
Chinese Taipei	17	16	402,5
Portugal	21	20	320
Argentina	16	11	365
Kazakhstan	8	7	415

Korea, Rep.	11	11	395
Portugal	15	15	390
Italy	10	5	410
Poland	12	10	322,5
USA	14	14	372,5
Japan	11	10	360
India	6	4	365
Denmark	16	15	325
Germany	15	14	270
Bulgaria	17	14	365
Brazil	13	11	330
Australia	18	17	315
Japan	11	9	330
Spain	14	12	300
Estonia	18	15	310
Russia	25	22	310
Germany	22	20	250
Japan	12	12	310
Israel	16	14	320
Australia	6	5	260
Peru	12	12	250
Switzerland	14	13	295
Poland	17	15	260
Italy	15	12	332,5
USA	13	11	290
Japan	13	10	275
USA	15	15	260
Chinese Taipei	18	15	245
Italy	14	7	270
Australia	13	13	180
Turkiye	23	19	245
Poland	23	18	240
Tunisia	10	10	270
Brazil	20	20	230
Italy	16	12	245
USA	18	17	285
Finland	13	12	220
Brazil	19	18	240
Ireland	18	14	220
Brazil	19	18	240
Great Britain	20	19	220
Great Britain	19	17	230
Argentina	18	15	220
Paraguay	24	20	212,5
Russia	6	6	225
Canada	21	17	190
Italy	13	9	230
France	24	23	200
USA	12	9	200
Australia	12	11	190

USA	18	16	180
USA	18	14	170
Morocco	25	18	170
Chile	9	9	90
Brazil	12	10	110
Switzerland	26	16	160
Switzerland	9	9	145
Japan	9	8	120
India	18	16	140
USA	8	6	170
USA	8	7	105
Brazil	20	13	90
Russia	14	11	1170
Brazil	16	15	1055
USA	7	6	1112,5
France	8	8	990
Spain	11	11	900
USA	13	12	840
Australia	10	10	790
Korea, Rep.	19	19	777,5
USA	13	10	785
France	8	7	690
Japan	13	13	700
Korea, Rep.	13	11	740
Brazil	11	11	650
Poland	8	9	665
Korea, Rep.	22	21	620
Argentina	17	17	630
Great Britain	15	14	615
Japan	16	16	552,5
Russia	5	5	617,5
Sweden	17	14	605
Peru	16	15	540
Cyprus	12	12	565
France	13	11	552,5
Netherlands	17	15	520
USA	15	15	545
Brazil	15	14	440
Poland	14	15	467,5
Italy	13	13	525
USA	6	5	585
Russia	14	14	500
Spain	11	11	445
Peru	20	20	422,5
Greece	18	16	475
Korea, Rep.	19	18	440
Belgium	9	8	460
Australia	14	14	400
Hungary	16	13	442,5
Australia	10	8	460

USA	11	11	385
Austria	8	8	305
Poland	17	17	395
Sweden	12	11	420
Romania	13	13	355
Japan	27	26	365
Kazakhstan	17	16	355
USA	10	10	405
Italy	16	16	375
Croatia	13	13	340
Chile	20	18	355
USA	14	12	395
South Africa	18	16	352,5
Serbia	16	13	355
Australia	13	13	315
Peru	21	19	350
Japan	13	14	332,5
France	15	13	330
USA	17	16	310
Vietnam	14	12	332,5
Slovakia	15	15	307,5
Slovakia	13	13	312,5
Uzbekistan	13	12	310
USA	14	13	320
Brazil	10	10	275
Germany	16	8	310
Serbia	13	8	365
Australia	26	24	295
Russia	13	12	265
China, P.R.	10	9	270
Russia	7	7	250
Denmark	21	19	260
Italy	15	15	270
Brazil	20	18	275
Switzerland	13	11	290
Argentina	19	18	240
Great Britain	13	12	200
Chinese Taipei	24	23	245
Japan	12	11	235
Korea, Rep.	12	12	240
Finland	25	21	220
Colombia	13	13	210
Japan	17	14	240
Italy	6	6	180
USA	20	17	210
France	19	18	210
Dominican Republic	12	10	215
USA	21	17	200
Japan	14	11	160
USA	21	20	182,5

Brazil	23	20	180
Great Britain	20	16	190
Canada	17	15	180
USA	14	13	170
USA	24	23	160
USA	30	23	150
Denmark	7	5	180
Brazil	19	14	125
Australia	10	5	105
Germany	16	14	1162,5
Croatia	10	8	1085
Italy	8	5	1085
Serbia	11	6	970
USA	13	9	835
Chile	6	6	845
France	13	12	765
USA	12	12	742,5
USA	15	14	650
Italy	11	11	560
Argentina	11	11	640
Australia	5	4	630
Russia	15	15	620
France	15	14	562,5
Chile	19	19	570
France	11	11	640
Chile	15	15	550
Russia	14	13	630
Japan	20	20	600
Poland	15	14	552,5
Korea, Rep.	15	15	590
Russia	16	16	540
Serbia	8	8	600
Russia	14	14	555
France	7	7	510
Peru	16	16	545
Belgium	15	14	555
Japan	10	9	522,5
Austria	16	15	417,5
Japan	15	14	530
Brazil	22	21	452,5
Korea, Rep.	7	7	495
Great Britain	12	11	450
Mexico	17	18	465
Germany	9	8	392,5
South Africa	8	8	475
Australia	12	10	360
Italy	16	13	447,5
France	14	12	390
Italy	8	8	370
Japan	18	13	390

USA	15	14	380
USA	16	14	325
Australia	19	18	370
Korea, Rep.	16	16	345
Denmark	17	13	330
Brazil	23	21	320
Korea, Rep.	16	16	345
Korea, Rep.	18	14	360
Germany	14	13	262,5
Great Britain	11	9	325
Germany	15	13	240
Australia	11	10	365
France	18	17	345
Brazil	19	13	340
Tunisia	16	14	310
Chile	20	17	280
Brazil	22	18	260
USA	12	12	210
Germany	13	10	315
USA	7	5	275
Colombia	15	13	300
Italy	6	5	280
Brazil	17	13	250
Sweden	11	9	315
Chile	13	8	310
China, P.R.	18	17	292,5
Japan	15	15	280
Japan	15	13	280
Great Britain	10	9	302,5
Japan	12	11	260
USA	17	16	280
Canada	10	10	250
Turkiye	10	9	285
Peru	17	16	282,5
Thailand	11	6	260
France	15	13	240
Slovakia	12	12	270
USA	23	23	260
Colombia	14	12	240
Mexico	22	19	200
USA	8	8	225
Mexico	19	17	240
Great Britain	22	21	200
USA	16	15	190
Burundi	19	18	230
USA	20	18	195
Canada	7	7	225
USA	11	9	230
Sweden	9	9	230
Brazil	14	10	180

Mexico	23	22	170
Venezuela	15	14	155
Canada	6	4	170
Brazil	21	17	145
Egypt	8	8	130
USA	14	12	145
USA	22	17	115
Paraguay	21	13	130
Colombia	20	16	115
Poland	9	9	1590
Belgium	12	10	1240
Italy	11	10	850
Australia	8	8	670
Australia	5	4	890
Portugal	15	13	575
Serbia	14	12	710
Serbia	16	13	660
Japan	14	14	660
Sweden	17	12	620
Argentina	12	12	580
USA	11	10	630
Chile	16	14	510
Great Britain	8	7	490
Italy	12	12	555
Korea, Rep.	15	15	550
USA	7	7	530
South Africa	15	14	500
Germany	17	17	520
Croatia	16	16	460
USA	20	19	505
USA	17	13	520
Argentina	15	15	475
USA	15	15	470
Australia	14	13	430
Germany	15	15	440
Netherlands	9	8	450
Japan	13	13	430
Great Britain	10	9	250
Italy	14	13	375
Brazil	19	18	420
Canada	15	15	385
Great Britain	8	6	380
Great Britain	16	16	390
Serbia	14	14	420
Denmark	17	15	385
Sweden	14	14	350
France	6	6	450
Canada	17	16	355
Argentina	16	16	350
Peru	15	15	350

Germany	15	14	395
France	9	10	380
France	12	10	390
Russia	21	21	340
Belgium	8	6	370
Italy	18	17	345
Egypt	13	11	360
Croatia	15	14	340
France	13	10	350
Uzbekistan	13	13	370
France	10	10	330
Great Britain	25	20	305
France	11	10	350
Korea, Rep.	16	14	355
Peru	15	15	260
USA	13	12	320
Australia	16	15	285
Finland	18	16	300
Great Britain	19	17	275
Czechia	9	9	300
Mexico	24	23	290
USA	16	15	295
Australia	14	11	290
Germany	16	13	290
Slovakia	15	14	320
Lithuania	21	18	230
Australia	6	5	100
Chile	19	16	250
Italy	16	14	250
Australia	18	17	220
Brazil	17	11	275
Israel	13	13	245
France	11	9	230
Turkiye	22	19	250
Israel	16	15	240
USA	16	13	260
Spain	10	7	230
USA	16	15	220
USA	14	14	220
USA	8	4	220
Mexico	18	14	230
Japan	17	15	210
Japan	18	17	215
Greece	12	12	220
Colombia	19	18	210
USA	9	7	195
Venezuela	16	13	200
Russia	11	7	200
Vietnam	21	18	190
USA	6	6	200

Chinese Taipei	20	19	155
USA	17	12	215
Burundi	15	13	180
Switzerland	26	22	190
India	11	11	160
Brazil	6	4	180
Brazil	17	12	180
France	12	11	150
Slovakia	13	11	135
Brazil	23	19	160
Mexico	25	23	150
Great Britain	26	22	120
Brazil	11	9	80
Great Britain	14	11	70
Czechia	9	9	1240
Australia	13	12	1140
Great Britain	7	7	950
Austria	13	11	910
USA	6	6	910
Bolivia	16	15	820
Brazil	15	15	840
Slovakia	12	10	705
Great Britain	9	8	670
Austria	17	14	735
Germany	17	14	530
Japan	18	17	555
USA	16	16	540
Brazil	16	14	570
Chile	20	21	540
Australia	10	8	490
Serbia	17	12	540
Great Britain	8	8	390
Belgium	19	17	460
Brazil	16	15	480
Great Britain	16	14	510
Ecuador	18	19	440
Belarus	17	17	420
USA	8	8	530
Czechia	19	16	460
Belgium	19	18	440
China, P.R.	13	12	465
Australia	14	11	450
Croatia	9	9	350
Egypt	17	15	435
Belarus	22	22	390
France	15	13	435
Philippines	8	8	430
Poland	18	17	390
Spain	6	5	350
USA	14	12	400

Serbia	18	17	410
Ukraine	11	11	325
Portugal	17	15	370
Chile	19	15	350
Chile	17	16	350
Mexico	20	20	310
Germany	12	11	290
Russia	18	18	350
France	6	5	380
Croatia	9	9	370
South Africa	15	14	365
Serbia	12	12	345
Belgium	8	7	360
Burundi	18	16	345
Italy	11	7	360
Great Britain	21	20	280
USA	20	20	315
Germany	15	14	350
Slovakia	16	13	350
Russia	15	15	310
France	13	10	325
USA	11	7	360
Austria	16	13	305
New Zealand	18	20	280
Spain	8	8	270
Czechia	15	14	310
USA	11	11	300
Zimbabwe	13	10	280
USA	10	9	300
Slovenia	16	11	280
Brazil	12	10	310
Spain	8	8	240
USA	10	9	190
Peru	17	15	260
India	24	16	245
Great Britain	20	18	220
Peru	16	13	240
Australia	9	7	220
Korea, Rep.	14	12	240
Argentina	17	13	245
Sweden	13	12	210
Brazil	17	17	220
Vietnam	24	18	225
Great Britain	24	24	200
Israel	11	10	220
USA	15	10	215
Czechia	8	8	200
Brazil	7	6	200
Great Britain	18	17	200
Mexico	15	14	200

Great Britain	20	16	180
Brazil	16	16	190
Mexico	13	11	175
Chile	18	17	160
USA	19	17	150
USA	22	14	150
Switzerland	12	10	160
Japan	15	12	130
Portugal	18	16	140
Brazil	10	8	80
Colombia	18	18	1070
Hungary	9	8	920
Czechia	16	14	740
Bosnia and Herzegov	13	13	820
Austria	21	19	820
Brazil	9	9	790
Ecuador	21	20	490
Peru	13	13	470
Argentina	5	6	740
Croatia	26	25	620
Australia	9	6	680
Russia	9	9	560
Argentina	12	10	640
Great Britain	15	15	585
Great Britain	17	16	500
Hungary	23	23	590
China, P.R.	12	12	540
Russia	12	11	505
Japan	15	13	510
USA	5	5	590
Italy	18	17	565
Netherlands	12	11	450
Australia	16	16	430
Slovakia	18	16	430
Bolivia	19	19	450
Slovakia	16	17	420
Philippines	23	21	470
France	14	11	490
Venezuela	13	11	460
Australia	12	11	410
Japan	13	13	415
Germany	12	10	310
USA	12	10	355
Netherlands	6	5	320
Germany	20	17	310
China, P.R.	12	11	375
Brazil	10	10	330
USA	18	17	325
Ireland	16	16	280
Argentina	16	13	305

Russia	13	13	290
USA	15	15	300
Ireland	16	15	260
Great Britain	10	9	300
Paraguay	21	17	285
Italy	17	14	320
USA	13	13	310
Mexico	15	14	300
Sweden	12	12	280
Brazil	20	17	290
USA	18	17	250
Brazil	19	14	220
Philippines	11	10	250
Germany	18	14	270
Germany	10	9	195
USA	7	6	240
Chinese Taipei	6	6	250
Argentina	11	10	255
Japan	14	14	200
Germany	24	24	250
France	15	11	275
Monaco	9	9	245
China, P.R.	17	16	165
India	20	17	220
USA	7	5	270
Tunisia	11	10	245
Mexico	12	11	250
Korea, Rep.	6	5	215
Ecuador	12	9	180
Brazil	7	7	210
Mexico	19	13	210
Brazil	20	18	205
Great Britain	16	14	205
Chile	22	16	220
Colombia	12	11	180
USA	13	9	210
Canada	16	13	190
France	14	14	170
Poland	12	12	165
Finland	8	7	170
New Zealand	18	17	160
USA	11	10	175
Italy	10	9	175
Chile	22	18	170
Barbados	15	14	140
Puerto Rico	14	13	150
Canada	12	11	130
Chile	15	14	160
Poland	16	12	110
Great Britain	10	9	150

Great Britain	10	9	90
New Zealand	8	7	100
Colombia	15	10	100
France	10	6	70
Chile	18	18	60
Sweden	14	13	950
France	16	14	900
Argentina	15	14	690
Australia	9	8	740
France	12	12	660
USA	15	12	680
Chinese Taipei	19	18	590
Argentina	16	16	630
France	18	18	520
Russia	5	3	710
Argentina	14	14	640
Germany	15	12	520
Chinese Taipei	17	17	390
USA	14	13	550
Venezuela	10	7	570
Germany	12	11	540
Belgium	17	14	620
Brazil	17	15	515
Peru	13	12	485
Japan	24	20	500
Italy	13	12	565
Japan	18	15	460
Slovakia	17	16	500
USA	9	9	490
Japan	20	19	450
Switzerland	11	9	535
Belgium	12	9	510
Brazil	19	18	470
Germany	12	11	450
Brazil	6	5	510
Argentina	11	11	430
USA	9	9	440
Croatia	11	11	330
Slovakia	19	17	430
USA	18	16	415
Argentina	19	19	385
Guatemala	12	12	440
Ukraine	13	13	405
New Zealand	20	20	360
Greece	12	10	395
South Africa	15	14	375
Switzerland	10	10	375
Sweden	12	10	395
Finland	16	15	370
Ireland	23	18	375

Philippines	18	18	230
Sweden	17	16	270
USA	15	16	350
USA	9	9	325
Russia	11	9	320
Great Britain	15	12	350
Great Britain	15	15	330
Italy	16	15	310
Austria	15	13	230
Japan	22	20	290
Austria	14	12	255
Italy	16	14	330
France	7	6	270
USA	8	5	335
New Zealand	15	14	290
Egypt	12	11	290
Italy	17	17	280
Belgium	16	14	320
India	15	14	320
Austria	14	14	260
Great Britain	19	19	230
Spain	7	6	270
Belgium	14	12	190
Croatia	9	8	140
Canada	26	24	245
Italy	15	13	230
Mexico	17	14	240
Canada	23	23	210
Belarus	10	10	210
Czechia	7	6	190
Ecuador	14	13	195
Latvia	24	19	170
India	12	10	220
Italy	14	10	150
Spain	6	6	210
Great Britain	17	14	185
Argentina	13	11	150
Great Britain	15	11	190
Brazil	10	9	150
Brazil	11	9	180
Brazil	22	16	150
Bahamas	18	17	140
Hong Kong, China	13	12	75
Mexico	8	8	75
Mexico	8	6	70
Chinese Taipei	8	8	930
India	15	15	980
Australia	6	4	930
Finland	13	13	660
Germany	17	16	630

France	17	12	720
Mexico	9	8	640
Brazil	12	10	690
Belgium	17	15	645
Bosnia and Herzegov	14	13	585
Argentina	16	16	605
El Salvador	15	15	560
Serbia	9	7	610
USA	9	8	580
Great Britain	15	15	505
Brazil	8	8	510
Indonesia	13	10	380
USA	9	9	490
Croatia	14	13	460
Belgium	16	15	495
Austria	20	15	360
Japan	9	8	500
Russia	6	4	480
USA	9	9	420
Croatia	13	12	400
Great Britain	10	10	420
Thailand	13	13	375
Chinese Taipei	15	13	365
USA	8	8	375
France	16	16	375
Australia	11	9	390
Japan	15	12	400
Thailand	13	12	365
Italy	16	15	370
Croatia	15	15	350
Australia	18	18	330
Chinese Taipei	13	15	200
Slovenia	17	15	370
Australia	11	9	360
USA	15	13	375
Great Britain	12	11	355
China, P.R.	12	11	350
Colombia	14	13	370
Dominican Republic	17	16	330
Austria	23	19	340
Russia	11	9	360
USA	8	8	330
USA	20	18	350
Canada	10	9	300
Chile	16	16	355
Zimbabwe	11	11	320
Guatemala	22	21	320
USA	13	10	340
France	16	13	290
USA	11	11	260

Italy	11	10	330
Finland	7	6	320
Great Britain	18	16	310
Great Britain	15	12	310
Netherlands	10	10	270
Russia	14	13	290
Argentina	8	7	300
Great Britain	18	16	240
Belgium	19	15	310
Australia	14	12	280
France	9	8	270
USA	12	12	270
Puerto Rico	16	14	250
India	24	18	285
Great Britain	7	7	260
France	25	16	235
South Africa	16	15	245
USA	16	15	210
Guatemala	19	17	220
USA	16	15	240
Israel	16	12	240
Canada	9	8	210
Belarus	21	20	210
North Macedonia	15	11	255
Great Britain	14	13	240
Australia	11	11	140
Venezuela	11	9	195
Canada	25	24	215
Mexico	24	23	200
Mexico	14	13	210
Great Britain	17	13	195
Italy	17	17	195
Australia	8	6	200
Croatia	13	12	180
Austria	23	19	150
Great Britain	12	9	195
Italy	12	12	155
Great Britain	20	16	150
Japan	7	6	180
Brazil	14	11	160
Guatemala	16	16	120
Canada	10	8	110
Lithuania	14	12	1290
Belarus	18	18	1210
Italy	10	10	870
France	9	9	690
Brazil	15	15	740
France	15	13	700
Italy	10	10	580
Czechia	16	16	550

Chile	20	20	570
Australia	13	12	590
Brazil	19	18	590
Poland	14	10	630
Portugal	11	11	610
Bulgaria	17	15	540
Chile	19	17	510
Australia	12	11	500
Australia	6	4	590
Moldova	16	15	530
USA	11	8	540
Great Britain	16	15	480
Mexico	17	16	490
Australia	11	7	510
Australia	19	19	480
France	19	18	380
USA	11	11	460
Netherlands	15	14	425
USA	10	8	430
Great Britain	15	13	375
Chinese Taipei	11	12	420
Paraguay	10	9	350
Belarus	8	6	325
Slovenia	16	15	390
USA	8	8	350
Ecuador	16	15	380
Thailand	17	17	410
Colombia	16	15	330
Chile	18	18	405
Belgium	18	15	380
Australia	15	13	345
Slovenia	12	11	415
Australia	16	14	345
South Africa	14	14	365
North Macedonia	13	12	390
Russia	18	16	355
France	13	11	320
USA	10	9	305
Australia	19	12	320
Japan	11	7	350
Indonesia	12	12	290
Czechia	11	11	290
Russia	16	15	250
Great Britain	20	20	280
Italy	22	17	325
USA	15	15	260
Romania	12	12	300
Russia	13	13	250
Slovakia	16	15	240
Croatia	19	16	290

Croatia	17	13	310
Korea, Rep.	15	13	260
El Salvador	12	12	300
USA	13	13	250
Netherlands	15	12	245
Thailand	19	19	260
Hungary	16	11	275
Italy	17	15	260
France	19	17	245
Finland	8	8	190
India	12	13	180
USA	18	17	260
Netherlands	19	14	265
Serbia	25	20	250
USA	9	5	280
Great Britain	23	22	215
USA	11	11	200
Finland	24	20	220
Chile	16	15	235
Italy	21	18	230
Slovenia	15	12	270
Great Britain	11	11	160
Dominican Republic	21	19	230
Israel	18	14	240
USA	13	11	210
Netherlands	11	10	200
Croatia	18	13	230
Croatia	15	8	210
USA	11	10	195
Venezuela	7	7	130
Ireland	11	10	200
Slovakia	10	9	150
Great Britain	20	17	200
Russia	17	15	185
France	6	4	180
Madagascar	13	12	205
Romania	12	11	190
Puerto Rico	22	18	160
Croatia	8	6	175
Philippines	9	5	170
France	9	7	150
Mexico	13	10	125
Poland	9	8	85
Great Britain	22	18	85
USA	7	6	90
Brazil	10	3	20
Netherlands	9	6	1350
Brazil	19	17	910
Slovakia	14	14	800
France	14	12	785

Romania	16	15	750
Czechia	5	4	790
France	6	7	710
Croatia	13	12	630
USA	16	15	520
Russia	17	16	630
Czechia	19	19	540
Russia	14	13	575
Australia	19	18	560
Portugal	15	15	550
Spain	10	8	570
Belgium	18	17	510
Estonia	14	14	510
Brazil	16	14	540
Slovakia	15	15	440
Lebanon	17	17	490
India	16	16	480
Bolivia	19	17	520
Russia	18	17	440
Chinese Taipei	14	14	440
USA	15	14	190
Chile	15	15	390
Czechia	15	15	420
Poland	12	12	480
Croatia	11	9	400
India	15	15	410
Italy	18	16	430
Italy	17	17	370
Canada	6	6	430
Lithuania	19	17	415
Japan	12	11	390
USA	13	13	260
Ukraine	9	9	410
Paraguay	15	15	365
Portugal	12	13	395
Brazil	16	15	380
USA	16	16	350
India	14	14	370
Netherlands	13	13	350
Argentina	7	7	300
Venezuela	15	16	330
Chile	18	19	330
Finland	21	19	380
USA	14	14	345
Spain	12	10	360
Croatia	9	10	300
USA	23	19	310
USA	16	15	340
Chile	24	21	300
China, P.R.	11	9	355

Ecuador	15	13	330
Australia	12	10	310
Canada	6	2	370
Estonia	14	12	325
Australia	11	11	270
Japan	17	15	240
Australia	11	7	290
Croatia	12	9	285
Netherlands Antilles	13	9	300
Tunisia	16	13	295
China, P.R.	12	9	280
France	9	10	260
Switzerland	9	7	290
Kenya	11	11	240
Colombia	13	10	270
Japan	5	3	300
Greece	13	13	255
Great Britain	9	9	260
Colombia	12	11	215
France	12	10	255
Russia	18	17	250
Great Britain	19	15	240
Switzerland	13	12	185
Great Britain	19	15	250
Denmark	16	13	230
Ukraine	21	14	235
New Zealand	12	10	215
Australia	16	15	200
USA	21	19	210
Ecuador	11	11	225
USA	16	10	200
Great Britain	14	10	190
Czechia	13	11	160
France	14	9	140
Switzerland	9	6	185
Great Britain	17	14	130
Austria	10	8	80
USA	13	11	1190
Croatia	10	9	1210
USA	16	14	1040
France	7	6	980
Argentina	16	14	850
Netherlands	6	6	660
Switzerland	15	11	645
Belgium	12	8	675
Netherlands	16	16	560
Brazil	17	17	590
Australia	12	11	570
Korea, Rep.	6	6	530
Venezuela	13	13	470

Brazil	10	10	520
Netherlands	10	10	510
Czechia	11	11	510
USA	8	8	560
Russia	10	10	470
Croatia	9	9	530
Austria	9	9	525
Italy	12	11	510
Canada	14	10	480
India	16	16	455
USA	19	19	475
France	16	15	495
Ukraine	8	6	490
Japan	16	15	440
India	16	16	430
Canada	8	9	350
Switzerland	12	12	415
Venezuela	9	9	360
Russia	15	14	385
Germany	8	8	435
Argentina	13	11	290
Brazil	13	13	400
Japan	15	12	390
USA	9	9	280
Australia	20	18	370
Germany	16	14	350
Slovakia	6	6	370
Russia	15	16	300
Greece	16	16	365
USA	11	11	320
Bahamas	7	7	350
USA	15	14	330
Hong Kong, China	11	9	370
USA	8	7	200
Venezuela	6	5	300
Japan	17	16	245
Canada	17	15	230
Japan	15	12	280
Kuwait	10	6	320
Ireland	15	14	230
USA	19	19	240
USA	6	6	260
USA	6	5	280
Australia	15	13	240
USA	13	13	260
Morocco	9	6	310
Czechia	22	20	260
USA	18	16	210
Australia	10	10	235
Namibia	16	13	225

Panama	13	13	245
Ukraine	10	9	220
Slovakia	6	6	220
Great Britain	7	7	190
Denmark	11	11	210
Switzerland	16	12	220
USA	10	8	200
USA	9	7	210
USA	24	22	170
Mexico	10	8	185
USA	11	11	200
Japan	9	9	200
Chile	22	21	170
Australia	9	7	220
USA	11	8	170
Italy	12	12	170
Belarus	11	11	170
Switzerland	17	14	165
Mexico	20	19	160
Great Britain	11	7	120
Croatia	11	7	130
USA	10	8	100
Mexico	15	13	60
Peru	12	8	50
USA	7	7	0
France	6	4	2050
Argentina	9	8	850
USA	9	9	520
Korea, Rep.	13	13	740
Korea, Rep.	16	16	720
Germany	11	10	680
Spain	14	13	560
USA	9	9	300
Slovakia	12	13	620
El Salvador	16	16	520
China, P.R.	18	18	510
Serbia	15	14	500
USA	11	9	560
Netherlands	14	14	530
Italy	15	14	550
France	15	13	580
Spain	11	10	535
Netherlands	13	13	510
Bahamas	17	16	480
Ukraine	16	15	460
USA	6	5	540
Great Britain	9	7	510
Argentina	12	12	465
Hong Kong, China	14	12	410
Germany	9	9	430

USA	14	14	455
Netherlands	14	13	390
Great Britain	12	9	420
USA	11	11	425
Brazil	14	13	435
South Africa	14	14	385
USA	7	6	400
Great Britain	8	8	430
Belgium	16	11	400
New Zealand	15	15	380
Italy	16	12	420
Venezuela	18	17	340
Great Britain	15	14	330
New Zealand	8	8	380
Argentina	9	9	340
Croatia	18	16	335
Czechia	17	16	330
Italy	14	13	330
Croatia	14	13	320
Netherlands	15	15	300
Russia	10	8	300
Australia	11	10	300
Kazakhstan	13	12	330
Great Britain	22	16	300
France	6	5	320
Canada	16	14	270
Germany	15	12	275
Belarus	20	18	295
Croatia	17	12	310
India	14	14	265
Brazil	12	12	290
Ireland	14	12	250
USA	25	23	230
Russia	10	9	240
Ghana	15	12	250
USA	13	11	290
Great Britain	24	21	275
India	15	14	235
Croatia	9	9	260
Slovakia	11	12	185
Australia	10	9	220
Brazil	13	12	250
USA	9	9	190
Netherlands	14	11	210
Austria	9	8	240
Mexico	14	13	215
India	18	14	220
Brazil	7	7	250
Venezuela	12	11	180
Japan	16	13	205

USA	13	11	220
Australia	13	10	140
USA	20	16	200
Finland	8	7	225
Kazakhstan	13	12	200
France	13	12	185
Austria	12	12	170
Great Britain	20	18	185
Brazil	11	10	190
USA	20	17	150
USA	7	6	130
Portugal	12	10	95
Germany	8	7	130
USA	14	11	150
Great Britain	19	17	140
USA	12	9	120
USA	19	12	140
USA	7	6	150
Ecuador	9	7	120
Japan	12	8	125
Peru	17	15	105
Japan	14	11	130
Canada	8	7	110
Venezuela	6	5	60
Portugal	8	6	70
USA	14	8	65
Australia	11	10	3230
USA	9	6	2040
USA	9	6	1990
Great Britain	12	8	1990
Belgium	16	15	1890
Japan	12	11	1980
Serbia	17	14	1850
USA	15	14	1690
Czechia	18	17	1550
Bulgaria	15	15	1660
Great Britain	10	9	1270
Bulgaria	11	9	1520
Chile	15	15	1390
Kazakhstan	13	12	1170
Czechia	17	17	1060
Bulgaria	11	10	1190
USA	14	13	1160
Czechia	14	12	1350
Great Britain	11	8	1020
Israel	19	16	1180
Poland	14	12	1180
Japan	9	5	1270
Ukraine	17	13	1140
Italy	12	12	900

Morocco	15	13	890
Czechia	17	14	710
Czechia	14	14	970
Germany	16	14	680
USA	16	15	1000
Norway	11	10	765
Sweden	17	14	950
Argentina	20	20	940
USA	22	18	930
Japan	22	21	890
Italy	13	14	820
Australia	16	15	950
Netherlands	17	16	720
France	12	10	910
Lithuania	23	20	820
Czechia	21	19	830
USA	15	15	790
USA	10	7	990
Germany	13	11	870
Bulgaria	17	14	750
Canada	15	12	850
Brazil	14	14	740
Japan	16	14	880
Sweden	26	24	770
Slovakia	12	12	760
Austria	13	7	815
USA	18	16	720
USA	15	13	720
Netherlands	8	8	690
Argentina	14	14	780
USA	20	14	600
France	17	16	670
Canada	14	8	640
France	16	16	660
Turkiye	18	17	645
USA	23	21	620
Russia	9	8	725
France	19	17	670
Spain	14	14	605
USA	19	16	610
Great Britain	20	20	640
Brazil	14	14	490
USA	13	10	610
China, P.R.	17	16	590
Japan	13	10	590
Belarus	16	16	490
Russia	14	11	590
Dominican Republic	21	19	536
Italy	17	14	486
Czechia	10	9	490

Egypt	13	11	550
Spain	15	12	560
USA	18	12	515
China, P.R.	20	17	500
USA	13	12	550
Great Britain	25	25	520
India	16	16	510
France	21	17	510
Japan	16	14	506
Russia	7	6	525
Czechia	23	20	440
Denmark	16	12	440
Ukraine	14	14	410
Ukraine	29	17	390
Czechia	28	26	361
USA	17	15	426
Sweden	27	25	401
India	16	12	395
Jamaica	18	13	361
China, P.R.	16	14	328
Kazakhstan	13	13	126
USA	9	9	305
Argentina	15	14	347
Romania	12	10	313
Czechia	17	15	336
Great Britain	12	9	360
China, P.R.	21	20	291
Japan	18	15	258
Germany	14	10	218
USA	8	6	300
Korea, Rep.	6	5	250
Benin	7	6	135
USA	12	8	160
USA	8	4	100
Russia	5	4	3380
USA	7	3	2400
Slovakia	10	9	2140
Czechia	19	15	1910
Peru	12	12	1960
Japan	11	10	1590
Spain	13	12	1730
Japan	8	7	1680
Great Britain	14	14	1590
Czechia	9	6	1830
Australia	15	13	1670
Czechia	18	16	1290
Russia	6	6	1300
USA	17	14	1200
Japan	8	7	1220
Spain	12	12	1370

Italy	8	8	1040
Great Britain	10	9	1120
Serbia	15	14	1280
Japan	17	16	1080
Russia	10	10	1240
Japan	7	7	1220
Russia	13	13	1080
Denmark	11	10	1150
Romania	16	16	830
Bulgaria	20	18	980
Russia	11	11	970
Australia	13	12	960
Argentina	17	12	950
Bulgaria	17	16	990
Great Britain	6	6	920
Benin	19	16	890
Morocco	9	8	910
Slovakia	9	7	860
Bulgaria	14	15	920
Japan	10	10	600
Israel	17	15	860
Italy	12	12	865
Poland	20	19	880
Italy	15	12	800
Italy	16	15	820
Norway	23	21	840
Brazil	17	15	890
USA	14	14	670
USA	18	18	740
Chile	22	19	780
Great Britain	13	12	740
Italy	16	15	690
Poland	13	14	680
Bulgaria	22	18	710
Czechia	11	10	760
Morocco	17	17	680
Slovenia	12	10	790
Austria	18	17	740
Romania	21	17	740
Kazakhstan	16	14	690
USA	20	19	680
USA	17	16	645
Ukraine	17	16	730
Italy	17	12	660
USA	16	13	640
Japan	17	15	690
Kazakhstan	11	11	636
Italy	14	10	575
Italy	19	16	520
Argentina	12	12	560

USA	13	13	510
Germany	22	20	600
Austria	13	11	680
Poland	15	15	515
Korea, Rep.	15	10	620
Australia	18	14	512
Great Britain	22	20	580
Australia	7	5	640
Israel	16	13	570
Serbia	16	16	560
Romania	14	14	385
USA	12	10	580
Australia	20	19	511
Italy	13	10	510
Great Britain	15	12	416
France	18	16	570
USA	15	13	392
USA	8	8	385
USA	21	20	470
Ukraine	23	21	406
USA	12	10	465
France	15	15	511
Slovakia	7	6	320
Germany	25	21	432
USA	16	14	416
Denmark	19	16	321
Argentina	16	12	410
Romania	17	15	432
Great Britain	7	6	300
India	21	21	400
USA	14	13	330
Japan	17	15	382
China, P.R.	22	16	352
USA	11	10	380
Canada	21	16	278
USA	19	12	361
USA	17	15	345
Russia	12	11	216
Great Britain	11	10	315
Kazakhstan	16	15	226
Czechia	9	9	240
Romania	10	7	270
Korea, Rep.	15	10	348
France	18	14	313
Canada	18	14	225
Italy	17	13	225
USA	20	15	258
USA	13	12	192
Russia	16	12	181
USA	15	12	45

Czechia	9	7	3010
Belgium	10	10	2600
USA	8	9	1840
Switzerland	10	10	1740
Japan	13	13	1400
Canada	9	9	1270
Czechia	13	12	1340
USA	5	2	1340
Great Britain	17	17	1240
Slovakia	17	17	1200
Australia	14	10	1250
Japan	15	15	1210
Argentina	19	17	1190
Slovakia	21	18	1100
Hungary	19	19	1010
Germany	9	9	950
Peru	19	18	960
Spain	22	22	1030
Great Britain	11	11	890
Great Britain	15	15	840
USA	12	10	870
Ukraine	16	12	930
Slovakia	16	15	850
France	14	13	820
Canada	9	9	980
Argentina	15	16	910
Canada	9	9	690
Japan	14	13	820
Canada	10	8	810
Denmark	10	10	770
USA	11	11	830
Czechia	22	18	710
USA	14	14	840
Chinese Taipei	19	18	600
USA	13	12	760
Germany	12	12	670
USA	24	24	790
Chinese Taipei	23	18	810
Slovenia	17	11	790
Slovakia	15	13	600
Czechia	7	7	740
USA	13	10	790
Germany	10	9	670
USA	9	9	630
Belgium	6	5	700
Canada	20	20	760
Serbia	25	17	750
Spain	12	10	740
Australia	7	6	690
Russia	14	14	600

Poland	14	13	660
Czechia	13	13	700
Australia	12	12	651
USA	16	14	660
USA	14	12	700
USA	15	14	610
India	13	10	640
Poland	26	24	470
Czechia	13	12	610
USA	18	18	572
Great Britain	17	17	630
Morocco	14	12	620
Great Britain	15	14	620
Kazakhstan	20	19	570
Italy	13	12	601
Belgium	19	18	540
USA	10	9	580
Kenya	11	10	580
South Africa	15	12	575
Kazakhstan	20	18	560
Russia	9	9	521
Canada	18	15	520
Turkiye	14	13	595
China, P.R.	24	23	460
Denmark	16	16	460
Australia	18	17	431
Czechia	21	19	510
Netherlands	16	13	496
Russia	7	6	530
Italy	17	16	416
Belgium	11	10	430
Croatia	6	6	325
Greece	8	5	540
Switzerland	10	9	360
Bulgaria	8	8	525
Great Britain	22	21	437
Iran	9	8	440
Kazakhstan	19	18	401
Benin	10	8	475
Morocco	10	11	412
Serbia	9	8	310
Denmark	19	13	362
USA	20	18	314
Kazakhstan	19	16	282
Brazil	9	7	356
Norway	22	17	306
USA	20	19	282
Italy	13	12	255
Lithuania	13	12	182
USA	12	10	274

Croatia	14	13	2000
Czechia	11	12	1940
Russia	13	13	1740
Czechia	15	15	1820
Andorra	6	6	1690
Belarus	6	6	1430
Russia	12	11	1300
USA	2	2	1300
Spain	6	5	1380
Philippines	6	6	1035
Argentina	12	9	1270
USA	14	13	1090
Greece	10	10	1110
Russia	13	11	1110
Czechia	3	3	1140
Hungary	10	8	1110
Denmark	19	18	950
Czechia	10	10	1020
Germany	10	9	950
Belgium	9	9	830
Switzerland	15	14	990
Poland	13	13	950
France	16	11	872
USA	11	11	790
Croatia	19	19	770
Czechia	11	10	870
France	8	7	970
Germany	5	3	1020
Belgium	17	17	780
USA	8	7	910
Germany	8	7	790
Canada	15	14	760
Russia	10	10	870
Finland	14	14	700
USA	8	7	630
USA	5	5	700
Russia	10	10	740
Russia	17	15	790
Czechia	18	18	830
Canada	10	10	770
Russia	13	12	790
Belgium	18	17	710
Switzerland	7	6	850
USA	19	17	620
Canada	15	15	605
Slovakia	12	10	722
USA	17	15	710
Czechia	11	11	620
Canada	17	17	560
USA	6	6	400

Switzerland	21	17	560
Czechia	8	7	640
Germany	15	10	630
Russia	13	12	600
Bulgaria	21	21	696
Mexico	9	9	590
Slovakia	13	12	510
Czechia	12	12	535
Great Britain	12	14	641
Germany	14	13	610
Belgium	21	20	517
Canada	15	14	610
Serbia	20	18	550
Russia	1	1	700
France	14	14	550
Belarus	14	14	620
Russia	1	1	490
Japan	6	6	564
USA	5	5	550
Russia	9	9	546
Germany	10	10	496
Serbia	11	11	510
Russia	7	6	540
Turkiye	20	20	520
Peru	13	13	520
Slovakia	17	15	520
Romania	12	11	520
Morocco	13	12	520
Kenya	13	13	506
Switzerland	10	9	502
Germany	11	10	520
Kazakhstan	18	17	520
USA	11	11	384
Spain	11	11	520
Czechia	21	20	425
USA	25	20	480
Russia	7	6	477
Lithuania	20	20	472
Russia	10	9	480
Russia	15	10	297
Argentina	15	14	425
Hungary	5	5	356
Czechia	7	4	536
USA	7	7	370
Slovakia	16	15	440
Romania	16	13	487
Russia	11	10	419
USA	11	8	456
Slovakia	21	19	456
Australia	11	9	476

Italy	20	18	366
Russia	2	2	390
Great Britain	4	4	450
India	10	10	486
USA	16	15	436
Russia	15	15	385
Czechia	16	16	432
Argentina	12	12	428
Japan	16	15	361
USA	15	13	441
Italy	18	15	313
Iran	15	12	452
Thailand	5	5	406
Paraguay	14	12	386
USA	17	13	348
Belgium	21	18	404
Hungary	16	15	367
Ukraine	10	10	295
Turkiye	14	14	361
Poland	16	16	392
Spain	14	14	380
Germany	11	10	336
Romania	15	13	352
Denmark	22	21	342
Mexico	10	9	367
Japan	10	9	300
Canada	8	8	360
France	13	12	328
Denmark	17	17	361
Russia	16	14	360
Japan	8	8	361
USA	21	16	345
Tunisia	13	13	352
Brazil	18	16	342
Kazakhstan	13	10	312
Ukraine	11	8	370
Slovenia	16	14	370
Bulgaria	8	6	336
Kazakhstan	15	12	296
Ireland	19	18	328
Serbia	11	6	376
Poland	14	13	288
Brazil	17	17	338
Canada	9	7	330
Chinese Taipei	15	14	306
Russia	15	13	328
Japan	5	5	338
Italy	17	12	326
South Africa	12	12	304
Kazakhstan	13	12	328

Germany	7	7	276
Ukraine	16	12	328
Switzerland	16	14	258
Colombia	6	5	380
Russia	16	14	328
USA	14	13	308
Lithuania	13	12	296
Chinese Taipei	9	9	292
Austria	17	14	336
Australia	6	6	306
Italy	17	15	312
USA	11	10	328
Greece	13	11	286
Italy	2	2	330
Bolivia	8	8	250
Great Britain	2	2	360
Poland	15	13	296
Poland	14	14	264
Belgium	18	18	264
Croatia	13	9	266
Great Britain	7	5	315
Egypt	15	14	270
Netherlands	13	12	292
China, P.R.	11	11	246
Spain	14	10	264
USA	15	13	260
Ukraine	16	11	256
Russia	11	10	244
India	10	10	226
Slovakia	12	11	232
Russia	11	12	238
Czechia	3	3	270
USA	8	8	228
Lithuania	19	18	266
Mexico	15	13	273
Ecuador	10	10	280
Germany	8	6	252
Canada	7	7	255
Kazakhstan	16	16	238
USA	17	16	240
Canada	12	11	266
Croatia	1	1	300
Israel	16	10	296
Poland	18	15	252
Kazakhstan	16	16	242
Thailand	6	1	294
Poland	16	14	251
Poland	21	21	208
Great Britain	13	13	234
Ukraine	12	12	206

Great Britain	14	13	195
Great Britain	7	5	236
Romania	14	12	234
Belgium	16	12	266
Spain	7	6	270
Portugal	15	14	236
Uzbekistan	11	9	214
Japan	15	12	201
Japan	7	6	170
France	11	10	244
Austria	19	19	222
Montenegro	15	15	214
USA	17	14	216
Hungary	14	13	214
Zimbabwe	16	14	212
Italy	2	2	140
Italy	16	11	205
Ukraine	11	9	252
Morocco	12	10	208
Paraguay	17	13	138
Great Britain	7	6	235
USA	12	10	172
Spain	12	8	236
Belarus	2	1	240
Kazakhstan	13	12	198
USA	9	9	181
Ukraine	11	7	214
South Africa	12	12	176
France	7	6	116
Canada	13	13	170
Czechia	14	13	170
USA	11	9	188
Czechia	8	8	200
Kazakhstan	14	14	172
Serbia	17	13	236
Romania	12	10	206
South Africa	12	12	184
Netherlands	10	10	187
Italy	14	12	196
Germany	13	13	186
Poland	6	6	210
Peru	5	5	200
Russia	6	5	209
USA	9	8	201
Spain	16	12	208
France	15	11	216
Russia	13	13	157
Netherlands	13	12	156
Great Britain	14	12	216
Australia	13	12	154

Serbia	10	6	222
Bulgaria	14	12	206
Poland	11	9	210
Canada	16	13	210
Brazil	17	15	162
USA	3	3	156
USA	13	10	146
Romania	7	5	216
Russia	19	14	208
Australia	4	4	200
Russia	14	11	204
Thailand	7	4	210
Spain	5	4	230
Zimbabwe	8	8	201
USA	5	5	150
USA	20	16	180
Belarus	3	3	190
Israel	12	11	184
USA	3	3	160
Russia	13	11	186
Turkiye	14	14	177
Russia	12	10	196
Sweden	16	14	162
USA	15	15	170
Turkiye	11	10	164
Germany	6	5	126
Nigeria	8	8	174
Russia	2	2	90
Russia	4	4	200
Croatia	21	13	136
Austria	20	19	166
France	15	9	188
Australia	7	7	170
Canada	3	3	200
Australia	2	2	200
Italy	8	8	85
Kyrgyzstan	12	12	160
Slovakia	17	16	152
South Africa	11	10	178
Bolivia	11	10	153
El Salvador	9	9	125
Canada	6	6	203
Russia	6	5	186
Canada	15	14	170
Russia	9	9	172
Lithuania	19	17	138
Slovakia	9	9	156
Egypt	17	14	148
USA	12	11	124
St. Kitts & Nevis	21	17	122

Romania	15	13	123
Sweden	5	3	120
USA	17	16	179
India	6	6	144
Italy	14	14	164
Romania	9	7	180
Slovakia	23	20	130
Brazil	10	7	173
USA	9	7	168
Romania	9	8	138
Dominican Republic	13	13	158
Israel	12	11	152
India	7	5	171
Slovakia	7	6	150
Russia	12	12	116
Russia	10	10	140
France	11	11	153
Germany	13	8	162
Japan	10	7	116
Slovakia	21	17	154
Cyprus	9	3	164
Australia	10	7	140
Israel	17	15	128
Great Britain	14	13	146
Chile	11	9	152
Chile	21	20	128
Argentina	15	10	150
India	8	2	154
Croatia	16	15	118
Switzerland	14	14	120
Lithuania	17	16	138
Estonia	13	12	140
USA	8	8	150
Bolivia	25	22	124
Netherlands	9	7	138
USA	10	9	133
France	3	2	130
Netherlands	9	8	129
Bulgaria	12	11	127
Slovakia	15	13	133
USA	10	8	133
France	15	12	121
Slovakia	14	13	121
Indonesia	3	3	120
Belarus	2	2	90
Singapore	7	7	141
Bolivia	10	10	124
Mexico	21	21	122
Belgium	20	16	96
India	11	9	125

Germany	10	9	126
Belgium	13	14	123
Russia	11	8	103
Estonia	17	15	128
New Zealand	4	4	116
France	8	7	130
Netherlands	11	11	120
Finland	17	17	118
Algeria	3	2	140
France	11	11	122
Benin	9	9	118
USA	6	6	120
Russia	12	7	140
Australia	8	8	114
USA	6	6	108
Great Britain	12	9	127
Netherlands	14	13	98
Russia	6	4	143
Peru	5	5	100
Italy	9	6	120
USA	15	12	132
Bulgaria	15	14	132
Germany	18	16	114
Netherlands	13	11	96
Kazakhstan	12	12	94
Morocco	14	14	118
Portugal	10	10	114
USA	4	4	115
Japan	14	14	103
Spain	4	2	139
Denmark	12	10	96
Slovenia	15	15	122
Mexico	11	10	125
Botswana	8	7	125
Hong Kong, China	5	5	143
France	1	1	140
Tunisia	12	12	121
Bolivia	6	6	146
Bulgaria	7	7	144
France	14	13	96
Bolivia	18	17	108
Brazil	13	11	100
New Zealand	5	5	131
Turkiye	15	15	83
Estonia	17	15	108
Czechia	14	13	86
Romania	7	5	90
Russia	7	4	133
Russia	18	16	100
Guatemala	20	20	102

Colombia	7	5	129
Brazil	11	10	106
Morocco	11	8	133
Switzerland	16	12	104
Netherlands	5	5	104
Argentina	15	13	78
Thailand	6	6	91
France	4	4	120
Zimbabwe	13	13	104
Australia	8	8	105
Israel	15	14	102
Romania	10	7	126
Peru	10	8	108
Slovenia	7	1	137
Bolivia	10	10	119
Russia	14	11	108
Slovenia	17	16	96
Austria	13	12	113
Czechia	11	10	85
Russia	4	3	121
Israel	14	14	91
USA	13	11	102
USA	15	9	112
Singapore	9	9	110
Chile	6	5	108
Norway	16	16	86
Russia	6	6	107
India	11	11	99
Colombia	11	9	109
Korea, Rep.	2	1	120
France	19	11	111
Kazakhstan	14	15	75
Great Britain	14	12	82
Czechia	14	10	88
Australia	10	10	90
Chile	19	16	98
Australia	6	6	106
Belarus	15	15	68
Netherlands	11	11	92
Slovakia	10	8	96
Japan	8	8	116
Brazil	18	16	89
Canada	16	9	102
Italy	5	4	90
Canada	15	15	94
Italy	6	5	113
Italy	4	4	0
Germany	7	7	99
Bulgaria	8	8	77
Colombia	12	12	79

Portugal	10	9	66
Russia	8	5	74
Belgium	15	15	92
Iran	7	6	105
Venezuela	4	4	108
Morocco	4	4	82
North Macedonia	13	11	87
Russia	11	10	74
Guatemala	15	14	79
Serbia	20	14	86
Hungary	12	10	94
Germany	13	7	97
Italy	13	6	122
USA	7	7	94
Italy	9	8	95
Latvia	11	10	107
USA	11	11	47
Great Britain	12	10	93
Poland	18	12	92
Slovenia	15	11	101
Poland	12	9	87
Puerto Rico	10	9	103
India	8	8	112
Poland	16	10	76
Argentina	5	4	96
Slovakia	16	16	82
USA	4	1	118
Ecuador	15	14	99
Ecuador	11	10	80
India	12	12	89
Kazakhstan	12	12	62
Egypt	15	13	83
France	10	7	110
Japan	4	3	116
USA	7	7	84
Tunisia	7	7	97
Hungary	11	10	94
Mexico	9	9	84
Serbia	15	11	106
Brazil	11	11	61
Italy	16	13	86
Korea, Rep.	6	6	99
Canada	9	6	101
Bulgaria	9	5	107
Netherlands	10	9	64
USA	12	8	83
Latvia	14	10	74
USA	13	5	113
Turkiye	11	7	109
Russia	13	11	91

USA	20	16	88
USA	6	5	88
USA	6	1	112
Bosnia and Herzegov	10	7	91
Bulgaria	21	18	86
India	11	9	95
Russia	5	5	86
Poland	16	14	71
Germany	6	0	111
Kazakhstan	15	14	90
Canada	6	6	70
Latvia	8	6	80
Belgium	22	20	78
Ukraine	8	7	87
Russia	9	7	105
Russia	17	14	88
Australia	4	3	104
Russia	9	8	95
Greece	13	8	86
Egypt	11	12	91
USA	9	7	96
Japan	9	7	84
Mexico	11	10	92
Argentina	3	3	70
Italy	16	14	88
Malaysia	13	10	73
Korea, Rep.	5	5	104
Thailand	7	7	58
Kazakhstan	9	8	97
Italy	6	4	90
Greece	16	13	81
Japan	6	6	76
Kazakhstan	16	15	65
Bolivia	11	10	65
Korea, Rep.	6	6	77
Russia	17	11	85
USA	12	9	46
Ukraine	12	11	37
Tunisia	12	9	76
Israel	8	7	79
Russia	8	9	29
Germany	15	13	79
Israel	10	10	67
Colombia	13	12	79
Israel	12	10	89
USA	3	2	90
Poland	12	11	71
Zimbabwe	15	15	58
Italy	12	11	84
Serbia	17	9	75

Cameroon	9	9	77
Croatia	15	11	49
Dominican Republic	13	11	58
Egypt	11	3	92
Iran	13	13	61
Great Britain	16	7	78
Slovenia	13	8	92
USA	11	8	64
Turkiye	7	7	60
Germany	5	2	95
New Caledonia	7	7	64
USA	15	15	71
USA	11	10	71
Bolivia	11	9	54
USA	6	6	70
Italy	8	5	59
Colombia	6	6	86
Germany	4	3	76
Korea, Rep.	12	11	61
Ukraine	13	11	38
France	7	7	82
South Africa	8	8	75
Guatemala	13	13	50
Russia	13	12	47
USA	12	10	66
Brazil	6	6	70
France	13	11	79
Australia	7	6	80
Ecuador	9	9	65
Poland	11	8	82
Russia	12	3	82
Czechia	14	13	58
Portugal	9	9	43
USA	10	8	65
Russia	5	2	80
Australia	6	6	68
Belgium	10	10	82
Venezuela	6	6	76
Canada	19	18	69
Hungary	9	6	82
Denmark	16	13	63
Serbia	13	11	14
Mexico	10	9	66
Denmark	9	9	78
New Zealand	2	2	70
Romania	16	14	71
USA	11	10	68
Estonia	14	13	56
Poland	6	6	73
Australia	9	9	40

India	9	7	37
Canada	6	4	64
Italy	5	2	30
Russia	11	13	74
Romania	11	11	61
USA	18	15	81
Spain	6	5	76
China, P.R.	18	17	62
Slovenia	13	11	60
Serbia	10	1	85
USA	3	2	72
Ukraine	7	6	58
Australia	6	6	50
Spain	8	4	84
Italy	9	8	75
USA	11	10	47
Barbados	13	13	66
Ukraine	20	10	58
India	3	3	65
Canada	16	14	69
USA	8	6	79
USA	4	3	60
Austria	14	8	54
Germany	15	14	37
Costa Rica	21	21	59
France	4	0	82
USA	6	5	61
Thailand	7	6	57
Russia	12	10	72
Austria	11	9	62
Madagascar	11	10	71
Kazakhstan	14	13	39
USA	16	12	65
Argentina	2	2	78
Kazakhstan	11	11	53
Russia	7	6	69
Thailand	7	7	53
USA	16	15	64
Kazakhstan	8	6	80
Turkiye	13	11	70
Great Britain	13	12	50
Thailand	11	11	56
USA	8	7	63
Romania	6	6	63
Brazil	7	6	65
Czechia	3	3	78
Ukraine	7	6	55
Canada	5	4	54
Egypt	15	15	52
Great Britain	7	4	57

USA	4	2	60
Austria	8	6	30
Chile	7	6	56
Dominican Republic	12	12	55
USA	11	9	53
Korea, Rep.	5	5	60
Australia	7	5	54
Thailand	7	7	63
Uzbekistan	9	9	63
Italy	10	8	65
Ukraine	4	4	71
Germany	13	9	66
Nepal	9	9	51
Norway	19	16	45
Turkiye	20	16	50
Canada	12	9	56
Azerbaijan	15	9	61
USA	10	9	68
Sweden	5	5	49
Colombia	7	5	65
South Africa	14	12	65
Venezuela	6	6	66
Nepal	6	6	62
Czechia	7	5	66
Latvia	12	10	62
Great Britain	7	7	47
Japan	7	7	73
New Zealand	3	3	51
Great Britain	8	7	52
Ecuador	6	6	46
Israel	15	14	63
Korea, Rep.	6	5	73
USA	13	11	63
USA	7	7	61
South Africa	11	10	53
Russia	16	12	60
USA	15	12	40
Cote D'Ivoire	11	10	57
USA	15	15	25
Brazil	11	11	53
Denmark	11	11	40
Japan	5	5	63
Mexico	13	10	66
USA	3	3	58
Austria	17	13	56
Singapore	17	16	54
Australia	7	7	36
France	14	11	63
Germany	2	2	65
Egypt	19	18	52

Austria	15	11	22
Hong Kong, China	3	3	53
Austria	10	8	30
USA	4	1	60
Poland	3	2	60
USA	10	6	61
Australia	6	6	45
Lithuania	10	9	57
Spain	9	7	60
Slovenia	12	11	42
Mexico	7	6	46
Hong Kong, China	4	4	54
Fiji	12	10	45
Russia	10	7	63
Russia	16	11	54
Kazakhstan	13	11	56
Great Britain	4	3	61
South Africa	10	9	60
Latvia	11	10	58
France	12	10	57
Poland	8	6	59
Ukraine	6	5	59
Ukraine	12	11	12
Venezuela	7	5	53
Croatia	16	15	45
Poland	9	8	33
Togo	12	12	53
Serbia	10	8	25
Korea, Rep.	2	2	60
Great Britain	4	4	0
Canada	4	4	50
Netherlands	6	6	54
France	7	6	48
Russia	3	2	66
Guatemala	16	16	43
Tunisia	8	5	64
Japan	7	7	57
Germany	12	10	45
Serbia	22	18	41
Czechia	9	7	38
Switzerland	8	5	46
USA	12	9	44
New Zealand	3	3	46
Kosovo	10	8	56
Czechia	21	20	40
Sweden	10	7	44
Japan	5	5	32
Japan	13	10	54
Great Britain	14	9	40
Spain	6	2	54

Russia	11	8	53
Egypt	4	3	60
Romania	13	12	43
Cyprus	13	13	35
Russia	16	12	35
Romania	7	3	63
Slovenia	14	13	40
India	6	6	39
Latvia	12	12	39
France	15	10	52
Russia	8	7	57
Slovakia	6	6	45
Hong Kong, China	4	4	50
Russia	12	9	58
China, P.R.	9	8	44
South Africa	11	11	47
Italy	5	5	47
Cyprus	9	9	55
Austria	7	5	45
Jamaica	10	9	50
Russia	13	8	54
USA	3	3	59
Japan	7	7	29
Great Britain	14	14	30
Sweden	9	7	47
Portugal	8	7	45
Kazakhstan	14	14	41
France	9	7	53
Israel	3	2	54
Russia	10	9	47
Sweden	7	6	45
Peru	10	9	56
Egypt	9	4	56
France	13	10	45
Spain	11	8	40
USA	3	2	60
USA	2	1	60
USA	2	0	60
USA	3	1	60
India	7	1	60
USA	2	0	60
France	11	10	48
Estonia	12	10	25
India	7	6	53
Botswana	11	8	32
Greece	4	4	49
USA	7	7	40
Great Britain	11	8	50
Slovakia	14	12	48
Finland	4	4	53

USA	3	3	48
Czechia	10	5	50
Kenya	14	14	35
Russia	10	8	57
Belgium	8	7	49
Czechia	7	6	37
Spain	11	4	52
Canada	5	5	50
Germany	13	6	53
Mexico	14	13	38
USA	5	5	39
France	11	10	48
France	9	10	39
USA	8	8	52
New Zealand	4	4	42
Canada	6	4	46
Russia	19	10	45
Chinese Taipei	2	2	30
Russia	5	5	51
Kazakhstan	13	10	45
USA	19	13	21
Poland	10	7	24
Slovakia	15	13	30
Belgium	10	8	44
Croatia	18	12	33
Estonia	7	6	39
Peru	6	4	30
Venezuela	10	9	24
South Africa	14	10	50
Bulgaria	11	9	30
Peru	9	9	40
France	12	6	45
Italy	10	10	40
Greece	6	6	43
Sweden	5	3	38
Nigeria	7	7	36
Italy	8	8	35
China, P.R.	4	1	48
Argentina	15	9	48
Romania	5	4	33
Belarus	12	10	27
Canada	9	8	22
Germany	5	5	37
New Zealand	3	3	29
Korea, Rep.	6	6	30
Brazil	11	7	18
USA	3	3	45
Russia	11	8	48
Peru	7	7	32
Czechia	8	6	51

Turkiye	12	11	39
Slovakia	11	9	26
USA	5	4	50
Brazil	10	10	36
Italy	7	6	32
USA	6	6	35
Spain	8	6	44
Hong Kong, China	4	4	43
Bolivia	7	7	37
Italy	11	11	23
Germany	8	4	44
India	4	4	51
Czechia	7	5	37
Italy	12	10	45
Ukraine	9	7	34
Austria	10	7	35
Sweden	9	9	25
USA	4	4	46
Colombia	11	11	44
Russia	12	10	40
Turkiye	11	11	31
El Salvador	12	11	44
Turkiye	15	9	37
Romania	5	4	36
Great Britain	8	7	22
Poland	18	13	20
Egypt	4	2	45
Colombia	8	7	26
Greece	11	10	28
USA	6	6	28
Singapore	5	5	35
Great Britain	7	6	37
Israel	11	7	43
South Africa	13	11	29
Spain	9	8	44
Slovakia	12	10	33
Japan	3	3	46
Slovakia	10	6	42
Italy	7	6	32
Estonia	10	10	34
Switzerland	6	6	34
Brazil	6	4	34
Russia	6	6	38
Peru	6	6	37
Russia	9	6	36
USA	1	1	30
USA	3	3	45
Great Britain	5	4	38
Great Britain	6	5	39
France	13	8	35

Bolivia	4	3	46
New Zealand	3	2	46
Mexico	8	8	25
Singapore	5	5	30
USA	5	5	37
Palestine	15	15	29
Bulgaria	14	10	26
France	19	18	26
Venezuela	5	5	42
Italy	7	5	36
Great Britain	8	6	43
Tunisia	11	10	40
Israel	11	11	34
Kazakhstan	13	12	33
Botswana	7	5	45
Slovakia	13	10	30
USA	7	7	42
Belgium	17	7	20
Romania	8	8	30
Japan	6	6	33
Germany	16	10	22
New Zealand	3	3	37
Italy	12	11	32
Egypt	11	8	16
Venezuela	3	3	32
Belarus	8	6	29
Great Britain	5	5	17
Ukraine	14	13	32
Kazakhstan	10	7	44
El Salvador	10	9	39
USA	19	16	30
Thailand	7	7	25
France	11	9	34
Germany	6	4	34
USA	4	4	38
France	10	9	40
USA	8	8	37
Brazil	7	5	36
Hong Kong, China	4	4	40
Russia	4	3	38
Austria	13	8	34
Ukraine	4	4	41
Romania	14	5	39
Argentina	4	3	41
Lithuania	15	14	39
France	14	13	37
Mexico	9	9	4
Colombia	12	11	31
Korea, Rep.	6	6	34
Iran	5	4	39

Mexico	10	9	18
Australia	5	5	40
Israel	4	4	40
Australia	6	6	35
Russia	12	9	32
USA	10	9	15
Montenegro	12	7	39
Greece	12	10	40
Hong Kong, China	6	6	29
Mexico	9	9	15
Bulgaria	2	1	36
Ukraine	6	6	33
Saudi Arabia	8	8	31
Japan	7	7	28
Sweden	7	7	15
France	9	8	24
South Africa	5	5	41
Ukraine	3	3	35
South Africa	5	5	26
Serbia	9	10	18
Romania	11	10	32
USA	13	12	32
Sweden	10	9	19
Kazakhstan	14	13	14
USA	8	6	32
Russia	8	5	40
Russia	3	4	38
Israel	2	2	28
Russia	4	4	36
France	2	2	30
USA	3	3	30
Singapore	3	3	25
France	15	15	29
France	11	10	34
USA	12	10	21
Ukraine	19	17	21
Egypt	14	13	26
Turkiye	4	3	28
Romania	15	12	27
Belgium	11	10	31
Moldova	4	3	34
India	11	10	27
Great Britain	9	8	32
Slovakia	12	8	32
Korea, Rep.	4	4	30
Belgium	6	4	37
Finland	11	11	19
Ukraine	7	2	40
Denmark	11	11	30
Spain	7	6	39

Armenia	6	5	36
Spain	10	8	37
Czechia	7	6	20
South Africa	3	3	25
Finland	7	7	18
Switzerland	6	6	18
Australia	8	7	29
USA	9	8	30
Chile	8	8	30
Slovakia	4	4	35
Nepal	8	8	30
Iran	4	4	27
France	10	10	22
Bulgaria	6	2	37
India	11	11	27
Croatia	7	5	35
Netherlands	4	4	15
Bulgaria	13	10	17
Italy	5	3	23
Turkiye	5	5	18
Israel	9	7	33
Sweden	8	7	15
Venezuela	3	3	27
Argentina	2	2	36
Russia	6	4	28
Sweden	5	5	31
Great Britain	12	8	24
New Zealand	5	5	25
Thailand	7	7	27
Poland	9	9	24
Turkiye	10	8	17
Russia	9	6	32
Japan	12	11	28
USA	4	1	30
Czechia	2	2	30
Kazakhstan	8	4	36
Mexico	2	2	18
India	12	12	25
USA	17	13	17
Egypt	10	9	15
Netherlands	14	11	12
Sweden	7	6	27
Greece	1	1	36
Thailand	1	0	36
USA	2	2	36
Great Britain	8	7	28
India	6	5	10
Bosnia and Herzegov	6	4	31
Spain	10	9	17
Mexico	14	9	25

USA	10	7	27
Russia	12	10	27
Venezuela	4	3	34
USA	4	4	28
Czechia	12	5	29
Moldova	9	1	35
Ukraine	6	5	24
Kazakhstan	16	15	24
Czechia	5	5	27
Canada	7	5	27
Switzerland	9	5	27
Lithuania	14	10	26
Bolivia	3	3	19
Germany	5	2	34
Egypt	10	9	30
Australia	4	4	25
Romania	11	9	22
France	10	8	33
Serbia	9	7	22
Poland	9	3	33
USA	2	2	30
Turkiye	11	9	25
Turkiye	15	11	21
Croatia	7	6	20
Czechia	8	6	24
Japan	6	6	29
India	5	3	29
Brazil	13	10	24
Romania	5	3	23
Singapore	6	6	29
Zimbabwe	3	3	28
Tunisia	8	6	29
Portugal	7	4	24
South Africa	8	7	15
USA	14	6	32
Germany	8	4	26
India	13	12	22
Serbia	8	7	20
Russia	13	10	9
China, P.R.	15	12	24
South Africa	14	11	22
India	11	7	29
Brazil	6	6	10
Russia	2	2	10
Sweden	8	8	15
Venezuela	2	2	23
Venezuela	2	2	23
Russia	12	9	26
Portugal	7	5	29
Montenegro	4	1	29

USA	8	1	32
Iran	6	6	25
Venezuela	7	7	21
Canada	2	2	30
USA	18	16	21
Romania	2	2	27
Egypt	12	10	18
Croatia	16	9	28
Great Britain	5	4	25
Colombia	8	6	22
Egypt	12	10	15
Uzbekistan	8	8	25
Russia	14	11	25
Italy	7	7	16
Finland	11	9	15
Japan	8	8	12
India	11	10	13
Colombia	2	2	30
Singapore	3	2	24
Korea, Rep.	5	5	21
Ukraine	6	4	4
India	3	2	25
Brazil	11	9	27
USA	4	4	17
Uzbekistan	6	6	28
Czechia	6	4	30
Israel	2	1	30
Antigua & Barbuda	2	1	30
USA	2	1	30
USA	2	1	30
North Macedonia	6	5	30
USA	6	3	30
Romania	15	13	20
Great Britain	2	2	23
Romania	9	4	22
Ukraine	5	5	0
Netherlands	2	2	0
Great Britain	4	3	22
Ukraine	9	8	28
Brazil	2	2	20
USA	4	4	15
Sweden	2	2	15
Spain	5	4	19
Georgia	9	9	27
Great Britain	8	7	24
Romania	9	7	12
Brazil	4	4	28
Canada	4	4	15
Singapore	5	4	20
Hungary	9	4	21

Russia	8	6	21
Poland	8	7	21
Great Britain	7	6	22
Greece	10	9	25
Belgium	11	7	20
Korea, Rep.	6	5	12
Korea, Rep.	5	5	10
Austria	10	7	25
Russia	8	8	24
Dominican Republic	11	9	14
Canada	15	13	22
Slovenia	12	9	7
France	5	4	27
Poland	9	8	14
USA	6	6	22
Spain	8	4	25
Russia	9	6	25
Serbia	13	11	19
Singapore	5	5	14
Japan	7	7	11
Canada	5	4	20
Indonesia	3	3	20
Turkiye	13	12	15
India	4	4	14
Germany	7	6	18
Kazakhstan	12	9	16
Russia	6	5	23
Australia	2	2	27
India	8	6	20
Zimbabwe	12	12	15
Great Britain	8	3	24
Poland	11	9	17
Ireland	2	2	5
Russia	13	13	18
Venezuela	2	2	18
Germany	12	10	13
USA	4	3	23
Japan	7	6	24
Serbia	11	4	22
Thailand	7	7	17
Brazil	9	6	25
Finland	7	7	25
Slovakia	6	5	2
Australia	6	6	15
Great Britain	10	7	24
France	8	3	20
Japan	2	2	20
Japan	6	6	22
Czechia	7	6	17
Israel	8	7	15

Singapore	5	5	10
Czechia	1	1	20
Germany	13	10	20
Switzerland	8	8	12
Nigeria	4	4	23
Russia	9	7	19
Mexico	9	8	25
Czechia	7	5	25
France	9	8	25
Mexico	7	4	23
Portugal	7	5	19
India	7	6	17
Italy	7	4	9
Colombia	7	6	7
Spain	5	2	20
Turkiye	7	6	19
Netherlands	5	5	22
Spain	7	5	20
India	2	1	15
Egypt	6	3	19
Romania	6	5	19
Spain	5	1	23
Brazil	6	5	22
Guatemala	5	5	20
South Africa	8	7	26
Brazil	8	7	21
Great Britain	8	1	26
Hong Kong, China	3	3	21
Czechia	17	12	11
Russia	14	9	22
USA	8	8	21
Bulgaria	10	10	14
USA	15	15	9
Korea, Rep.	6	6	7
Mexico	9	6	23
Australia	6	6	15
Latvia	12	10	17
Spain	9	4	23
Spain	7	6	15
Australia	3	3	20
USA	7	5	22
Japan	7	7	7
Kazakhstan	8	7	14
Brazil	10	9	14
Great Britain	14	10	15
Israel	6	5	20
India	4	4	20
USA	9	7	20
Korea, Rep.	4	3	25
Great Britain	11	11	21

Russia	3	2	18
Turkiye	16	14	16
Czechia	1	1	18
Thailand	1	2	10
Bulgaria	10	8	23
Italy	5	4	21
Russia	7	7	15
South Africa	8	4	20
Switzerland	3	1	23
Russia	10	8	21
South Africa	5	5	18
South Africa	11	7	12
Brazil	8	4	19
Japan	7	6	12
Israel	10	10	20
Egypt	4	4	15
India	10	9	10
Greece	8	8	17
Russia	8	5	17
Switzerland	8	6	15
Australia	4	2	20
France	5	5	11
Australia	2	2	20
Ukraine	4	3	20
South Africa	4	3	20
Germany	3	3	5
Brazil	4	2	23
Thailand	6	6	17
Ukraine	9	7	14
Hungary	13	7	0
Korea, Rep.	3	3	22
Egypt	7	7	20
Cyprus	7	7	17
Italy	6	4	22
Ecuador	4	3	22
Bosnia and Herzegov	9	6	18
Venezuela	7	7	15
Venezuela	2	2	14
Russia	9	7	7
Austria	11	8	17
Israel	9	7	19
Sweden	3	3	9
Mexico	4	3	23
Mexico	10	5	23
Great Britain	3	3	10
France	6	4	10
Argentina	2	1	23
France	11	9	20
Bolivia	3	2	10
Finland	6	6	12

India	3	3	5
Argentina	2	2	5
Hong Kong, China	2	1	23
Czechia	4	3	23
Mexico	10	9	14
Russia	9	8	15
Finland	9	9	19
India	6	6	21
USA	6	6	20
Bolivia	9	6	17
Belgium	8	8	12
Venezuela	4	4	17
USA	3	2	0
USA	3	3	0
Hong Kong, China	6	5	19
Egypt	10	10	19
USA	3	3	13
Venezuela	7	7	13
Turkiye	15	14	9
Hong Kong, China	3	3	9
Australia	4	4	22
Germany	4	2	12
Germany	11	6	7
Uzbekistan	9	7	17
Japan	10	7	17
Japan	4	4	14
Germany	8	6	5
Venezuela	7	7	18
Nigeria	5	3	14
Venezuela	7	7	14
Hong Kong, China	5	4	12
Bulgaria	11	8	20
South Africa	4	3	17
India	1	1	18
Great Britain	12	6	19
Japan	2	2	19
Poland	9	8	15
Togo	8	6	14
USA	10	8	14
USA	9	6	11
Kenya	9	8	7
Uzbekistan	13	11	7
Russia	8	5	20
Great Britain	6	6	12
Nigeria	2	2	9
Australia	7	7	12
Czechia	14	7	12
Malaysia	4	4	18
France	8	5	15
USA	2	2	10

Korea, Rep.	4	4	19
Singapore	3	3	5
Serbia	4	2	18
France	4	3	18
Brazil	7	6	13
Thailand	6	6	15
India	6	4	17
India	8	9	7
Turkiye	11	9	7
Turkiye	6	6	10
France	17	10	17
Spain	12	11	16
France	11	7	12
France	5	3	20
Ukraine	14	13	4
Russia	11	4	17
Netherlands	7	7	14
India	3	3	19
Great Britain	14	11	13
Colombia	9	9	9
Russia	9	9	15
Latvia	11	10	16
Slovakia	5	3	15
Japan	5	5	10
Chile	2	2	10
Turkiye	11	8	14
Germany	11	8	10
Uzbekistan	8	8	11
Italy	2	1	20
Italy	1	0	20
Chile	2	2	20
Romania	4	2	20
Spain	7	5	17
USA	4	4	12
Russia	13	11	14
Italy	6	5	16
Turkiye	14	11	8
Australia	4	4	19
Australia	5	5	12
USA	3	2	18
Greece	3	2	18
France	1	1	18
Portugal	9	9	9
Netherlands	2	2	5
Venezuela	7	6	16
Mexico	13	11	10
Egypt	10	10	17
Greece	8	6	15
Thailand	4	4	12
Poland	11	7	12

Colombia	7	6	18
Nigeria	6	6	15
Korea, Rep.	6	5	12
Cameroon	2	2	7
Russia	14	9	17
Israel	4	4	15
New Zealand	4	4	15
Spain	4	3	9
Czechia	3	1	19
Ukraine	5	4	15
Italy	6	5	4
Egypt	12	8	13
Russia	7	4	7
Latvia	6	3	18
Austria	4	3	18
Poland	12	12	6
Australia	4	4	0
Russia	1	1	0
India	10	6	17
Slovakia	9	8	17
India	4	4	15
Indonesia	5	5	12
Argentina	4	2	10
Nepal	4	4	10
Great Britain	7	4	17
Nigeria	6	6	14
Great Britain	13	11	12
Belgium	4	4	12
Italy	4	2	15
Australia	4	3	12
Turkiye	4	3	15
Italy	3	2	10
Serbia	10	3	7
Hong Kong, China	4	4	4
Egypt	13	14	4
Turkiye	10	7	18
Paraguay	3	3	15
India	1	1	18
Spain	5	0	18
USA	3	2	18
Japan	7	6	14
Korea, Rep.	6	6	12
Israel	9	8	12
Italy	3	1	18
Spain	3	0	18
Nigeria	4	3	18
USA	1	0	18
Russia	2	2	0
USA	4	2	0
Tunisia	5	4	12

New Zealand	5	5	17
Peru	8	7	17
Guatemala	10	7	16
Lithuania	8	8	12
Bolivia	5	4	12
Tunisia	8	7	14
Kazakhstan	8	5	2
Italy	8	5	15
Netherlands	5	2	15
Iran	4	4	15
Korea, Rep.	6	6	14
USA	6	4	10
Russia	10	7	16
Russia	12	8	16
Great Britain	6	6	12
Spain	7	5	16
Norway	6	5	2
Switzerland	9	8	9
Japan	6	6	9
India	7	6	13
Russia	7	7	9
Colombia	4	4	17
Australia	4	4	12
Romania	10	8	14
Ecuador	6	5	12
Tunisia	10	4	12
USA	5	5	12
USA	5	3	10
India	2	2	10
Romania	5	2	12
Spain	9	4	14
Poland	11	5	14
Turkiye	10	7	15
Canada	4	3	15
Australia	6	5	15
Germany	3	2	10
Finland	4	4	5
Netherlands	8	5	2
France	8	7	0
Australia	4	4	12
Australia	4	4	12
Guatemala	17	16	14
Singapore	4	4	14
Belgium	12	10	15
USA	3	2	14
Slovakia	5	4	15
Uzbekistan	10	10	2
Russia	9	5	12
Slovakia	2	2	10
Spain	8	6	7

Colombia	5	5	7
Germany	12	6	7
Russia	10	5	16
Latvia	11	11	12
Thailand	7	7	10
Egypt	9	8	5
India	8	5	13
Korea, Rep.	3	3	12
Brazil	6	5	7
Canada	10	6	15
Belarus	4	2	15
Zimbabwe	5	5	12
Hong Kong, China	4	4	15
Israel	12	7	14
Russia	5	4	11
Georgia	10	10	9
Peru	6	6	7
Belgium	8	7	4
Japan	8	8	4
Israel	5	4	10
South Africa	7	6	10
Mexico	6	2	14
Guatemala	6	6	14
South Africa	7	7	9
Czechia	4	3	14
Nigeria	3	3	14
Russia	5	4	13
Russia	11	7	10
Denmark	11	11	9
Canada	3	3	5
Argentina	2	2	10
Canada	2	1	9
Russia	4	2	9
Slovakia	13	13	7
Venezuela	6	6	6
Canada	4	3	15
Canada	4	4	15
Canada	4	4	15
Turkiye	4	2	15
Slovakia	4	1	15
Argentina	2	2	15
Argentina	2	2	15
Portugal	6	3	15
Mexico	8	8	10
Paraguay	11	10	11
Czechia	12	11	11
Austria	12	6	9
Hungary	6	3	5
Ukraine	5	3	10
Guatemala	13	10	8

Austria	7	5	2
Romania	5	5	14
Singapore	5	5	14
USA	12	11	14
Pakistan	4	4	14
Peru	2	2	14
Mexico	5	3	14
Russia	11	11	10
Australia	6	6	12
Mexico	9	6	12
Slovenia	10	5	12
Czechia	11	8	12
Egypt	15	11	12
Turkiye	8	7	9
USA	15	10	8
Argentina	5	3	10
Thailand	7	7	10
Greece	7	8	7
Paraguay	7	6	12
Turkiye	7	6	12
Serbia	6	5	11
USA	4	3	9
Belarus	7	6	9
El Salvador	16	15	7
Belgium	8	8	7
Finland	5	0	14
Russia	5	3	11
Venezuela	5	5	11
USA	8	8	10
Norway	7	3	2
Canada	4	3	10
India	4	3	12
Canada	3	3	12
France	4	4	10
Kazakhstan	12	9	9
USA	5	5	10
Bulgaria	5	5	9
Australia	4	4	7
Mexico	12	11	5
Spain	8	5	12
Israel	5	5	10
Spain	4	2	12
Israel	15	11	12
Argentina	2	1	10
New Zealand	2	2	10
Austria	5	4	10
Japan	2	2	10
Argentina	2	1	10
Argentina	2	2	10
Croatia	11	8	10

Korea, Rep.	5	3	10
India	4	4	10
USA	7	6	10
Argentina	2	2	5
Albania	5	5	8
Russia	14	10	6
Greece	7	7	7
Austria	9	7	7
Great Britain	10	8	5
USA	4	3	2
Russia	7	6	2
Denmark	8	6	9
Spain	7	2	13
Czechia	5	3	13
Morocco	4	4	10
Switzerland	7	5	9
France	8	5	9
Brazil	7	5	2
China, P.R.	7	6	0
Poland	6	5	12
Romania	11	8	12
USA	5	5	9
Georgia	4	3	12
USA	3	2	12
USA	5	4	12
Singapore	5	4	12
Serbia	3	3	12
USA	8	4	12
Denmark	5	4	12
Belarus	5	2	12
Singapore	3	2	12
Guatemala	8	5	11
Cameroon	2	2	9
Chinese Taipei	4	3	7
Argentina	4	4	7
Croatia	7	4	10
France	8	8	10
Benin	5	5	10
India	5	3	10
El Salvador	8	8	7
Ukraine	6	6	7
Germany	2	1	11
France	3	2	11
Poland	8	7	2
USA	10	7	0
Germany	9	7	10
Germany	7	5	9
Italy	7	5	9
Switzerland	5	2	10
Australia	4	4	7

Great Britain	5	5	4
Serbia	7	6	2
Austria	9	6	12
Russia	10	4	12
Sweden	4	2	12
India	2	1	12
Turkiye	7	5	12
Slovakia	10	4	12
Korea, Rep.	6	6	7
Sweden	5	3	5
Japan	3	3	5
Poland	6	4	9
Israel	9	9	0
Luxembourg	2	1	10
Japan	1	1	10
USA	2	2	10
Great Britain	4	3	10
Great Britain	2	1	10
USA	3	1	10
Morocco	4	3	10
Argentina	2	2	10
Colombia	14	8	11
Australia	4	4	10
Germany	5	3	10
Egypt	11	10	7
Latvia	10	5	5
Greece	3	3	11
Switzerland	9	7	5
Slovenia	9	5	5
Egypt	5	6	5
USA	6	4	4
Romania	7	7	4
Italy	6	4	2
Poland	13	7	2
Romania	7	5	10
Russia	5	5	10
South Africa	10	7	10
Great Britain	5	3	10
Togo	7	5	9
Poland	11	9	9
France	8	8	9
USA	5	4	5
Cameroon	2	2	10
Armenia	3	2	10
Cameroon	2	2	10
Serbia	13	4	9
Singapore	5	5	5
Serbia	10	6	4
Russia	4	4	2
Italy	5	3	2

Italy	5	3	2
Singapore	5	5	7
Belgium	6	3	5
Bosnia and Herzegov	4	2	9
Canada	5	3	9
Turkiye	11	8	9
Spain	3	2	5
Egypt	5	6	2
Uzbekistan	8	8	2
Slovakia	9	6	2
Kazakhstan	12	8	2
Italy	2	1	0
Germany	4	2	0
USA	1	1	0
USA	3	1	0
Sweden	1	1	0
Russia	4	4	0
USA	1	1	0
USA	1	1	0
Hungary	9	2	0
Bolivia	2	1	0
Germany	4	1	0
Bolivia	4	1	0
Philippines	4	3	0
Germany	3	1	0
Canada	6	4	11
Iran	8	8	7
Turkiye	14	10	7
Ghana	10	10	5
Croatia	12	9	5
Norway	2	1	11
Argentina	2	2	7
Poland	14	8	2
Australia	4	4	10
Thailand	6	2	10
Ghana	9	9	9
Ghana	9	9	9
USA	9	5	7
Zimbabwe	13	10	7
Germany	8	8	6
Czechia	13	10	2
Switzerland	4	4	2
Germany	10	9	7
Germany	3	2	5
Peru	8	7	9
USA	4	3	9
Mexico	7	4	7
France	5	3	7
Germany	8	5	5
USA	5	2	9

Russia	6	5	9
Poland	7	3	9
Czechia	4	2	9
New Zealand	1	1	9
Serbia	6	2	9
France	5	5	9
Venezuela	3	3	9
Bulgaria	15	9	9
Kazakhstan	16	14	6
USA	9	7	7
Japan	8	8	7
Bolivia	5	4	7
Italy	4	3	7
Lithuania	9	6	7
Portugal	6	6	7
Canada	5	4	7
USA	4	4	5
Uzbekistan	3	3	2
Australia	1	1	10
Belarus	1	1	10
Uzbekistan	7	5	10
Serbia	2	1	10
Australia	2	2	10
Russia	2	0	10
Poland	4	1	10
South Africa	3	0	10
Portugal	2	1	10
France	1	1	10
India	3	0	10
India	2	2	10
Australia	1	0	10
Russia	5	1	10
Zimbabwe	5	3	10
Germany	1	0	10
Mexico	11	5	10
USA	1	1	10
Morocco	3	3	10
Spain	1	1	10
Thailand	2	1	10
Argentina	2	2	10
Japan	3	1	10
Chile	4	0	10
Austria	1	1	10
India	3	3	10
Russia	2	2	10
Australia	2	2	10
Benin	3	2	10
Belarus	2	2	10
Estonia	6	3	10
Turkiye	4	2	10

Kenya	11	11	5
Cameroon	2	2	10
USA	3	3	10
Finland	2	0	10
Venezuela	4	3	10
Canada	8	6	7
France	8	5	6
Korea, Rep.	6	6	6
Canada	3	2	5
Kazakhstan	10	6	4
South Africa	8	5	9
France	11	7	9
Zimbabwe	6	5	9
Peru	5	2	9
Spain	6	4	9
Korea, Rep.	4	4	9
India	9	8	9
Bosnia and Herzegov	8	8	5
Italy	4	2	9
USA	4	2	9
USA	3	2	9
Latvia	7	4	9
Kazakhstan	10	6	9
Brazil	8	7	4
China, P.R.	8	4	2
Russia	6	6	2
USA	6	5	2
Ukraine	4	3	7
Singapore	5	5	7
Austria	4	4	7
Turkiye	7	4	7
Australia	5	4	5
Uzbekistan	9	5	8
Egypt	6	5	7
USA	4	3	4
Kazakhstan	10	7	2
Austria	6	4	2
Germany	13	12	2
Great Britain	8	7	2
Bolivia	4	4	7
Egypt	12	4	5
France	2	2	7
Turkiye	8	5	7
Spain	6	4	7
Great Britain	2	2	7
Korea, Rep.	6	6	5
Egypt	13	11	2
France	4	3	9
Thailand	7	4	9
Singapore	5	5	5

Japan	1	0	9
Singapore	1	1	9
Brazil	5	3	9
USA	1	0	9
Ukraine	4	3	9
Czechia	2	0	9
France	8	2	9
Lebanon	3	3	9
Denmark	11	9	9
New Zealand	1	1	9
Canada	4	3	6
Japan	5	4	6
Hong Kong, China	4	3	5
Romania	5	3	5
Great Britain	3	2	5
Italy	5	4	4
Egypt	11	6	4
Australia	4	4	2
Ecuador	5	4	2
Canada	5	4	5
Thailand	6	5	7
India	7	4	7
India	11	10	7
Iran	4	2	7
Tunisia	9	5	7
Turkiye	7	6	5
Colombia	6	6	4
Paraguay	9	7	4
Czechia	2	2	2
Romania	7	2	5
USA	3	2	7
Egypt	6	2	7
Finland	7	7	7
Thailand	1	1	5
Argentina	2	2	5
Slovenia	5	4	5
Israel	4	4	5
Russia	4	1	5
Canada	7	3	5
Thailand	5	4	5
Thailand	1	1	5
India	1	1	5
Turkiye	8	7	5
Tanzania	3	3	5
Australia	2	2	5
Argentina	2	2	5
Croatia	9	7	5
Spain	6	3	7
India	2	2	7
Italy	9	7	7

Italy	9	7	7
India	2	2	7
Serbia	7	2	7
USA	2	2	7
India	7	7	2
Egypt	12	8	0
Hong Kong, China	4	4	5
Bulgaria	13	9	6
Venezuela	5	5	6
Peru	10	10	6
Russia	7	6	6
India	11	7	6
New Zealand	3	3	5
Mexico	12	7	5
Czechia	2	2	5
Sweden	1	1	5
Serbia	6	1	5
Puerto Rico	1	1	5
Russia	2	1	2
Australia	4	4	2
France	7	5	2
Australia	4	4	2
Cyprus	12	12	0
India	5	5	0
Russia	5	2	8
Russia	3	3	5
Bolivia	10	8	5
Colombia	5	3	5
Sweden	7	5	4
Lebanon	2	2	4
South Africa	5	3	4
Kyrgyzstan	4	4	7
Georgia	4	4	7
Japan	8	8	7
Belgium	4	3	7
Switzerland	3	3	7
Sweden	4	2	7
Poland	9	4	7
India	2	2	7
Kazakhstan	12	8	7
USA	5	2	7
France	3	2	7
Serbia	4	2	7
Bulgaria	6	4	7
Germany	4	1	7
Russia	9	3	7
Switzerland	6	5	2
Greece	8	6	2
Kazakhstan	9	5	2
Poland	7	6	2

Canada	9	8	2
Poland	6	6	0
Latvia	7	6	0
Germany	11	6	5
Mexico	11	4	5
Hong Kong, China	4	4	5
France	9	6	6
Kazakhstan	14	7	6
Finland	6	5	6
New Zealand	4	3	5
Serbia	10	5	5
Mexico	4	3	4
Great Britain	9	9	2
Australia	4	4	2
Slovakia	7	5	0
Dominican Republic	6	5	0
Sweden	3	2	5
Mexico	4	3	5
Colombia	2	2	5
Russia	5	2	4
Mexico	2	1	4
Sweden	8	5	4
Mali	3	3	4
South Africa	8	6	4
Japan	5	5	4
Italy	2	2	2
Estonia	9	6	2
Mexico	8	6	2
Spain	6	3	2
Czechia	13	6	7
Thailand	5	3	7
USA	7	7	7
New Zealand	2	1	7
India	3	2	7
Germany	3	1	7
Brazil	6	2	7
Panama	8	6	7
Colombia	3	3	7
Netherlands	8	6	7
Czechia	7	4	7
Namibia	2	2	7
Iran	5	5	7
Latvia	7	4	7
Iran	2	2	7
Portugal	2	2	7
Togo	2	2	7
Romania	2	1	7
Brazil	3	1	7
Brazil	7	3	7
Kazakhstan	2	2	7

Togo	2	2	7
Croatia	8	7	4
Romania	2	2	2
Belgium	2	2	2
Canada	15	8	2
Czechia	7	4	0
Russia	9	4	0
Chile	4	2	0
Romania	4	1	5
Egypt	6	2	5
Hong Kong, China	4	3	5
Argentina	1	1	5
Sweden	5	2	5
USA	2	1	5
Thailand	1	1	5
Russia	2	1	5
Argentina	1	1	5
Japan	3	3	5
Sweden	1	1	5
France	2	1	5
Iran	2	1	5
India	3	2	5
Thailand	2	1	5
Sweden	2	1	5
Romania	5	3	5
Hungary	6	2	5
New Zealand	2	2	5
Thailand	2	2	5
Turkiye	2	2	5
New Zealand	4	4	5
Denmark	5	4	5
USA	3	2	5
Sweden	2	2	5
France	5	2	5
Argentina	2	2	5
Kenya	3	2	5
Japan	3	2	5
Kenya	9	6	5
Japan	4	4	5
China, P.R.	6	2	5
Singapore	5	4	6
Kazakhstan	19	5	6
Bosnia and Herzegov	14	8	6
New Zealand	3	2	5
Canada	6	4	5
USA	8	7	5
Egypt	5	3	5
Bulgaria	3	2	5
Belgium	10	6	5
Poland	2	2	2

Cameroon	2	2	2
Spain	5	4	2
Mexico	3	3	0
Mexico	7	5	0
Romania	1	1	0
Ukraine	1	1	0
Australia	1	1	0
Ukraine	2	1	0
Egypt	4	3	0
Brazil	3	2	5
Ecuador	4	3	5
Belgium	4	3	5
Pakistan	4	4	5
Nigeria	5	5	5
Germany	1	1	5
Nigeria	2	2	5
USA	1	1	5
Netherlands	7	4	5
France	1	1	5
Sweden	3	3	5
India	2	2	5
Spain	7	5	5
Poland	6	4	5
USA	5	4	5
Pakistan	4	4	5
Czechia	4	4	2
Finland	4	3	4
Germany	7	5	4
Uzbekistan	11	8	4
Serbia	2	2	4
Belgium	6	3	4
India	3	3	4
Luxembourg	3	2	4
Venezuela	3	3	4
Estonia	8	7	4
Great Britain	3	2	4
Australia	4	4	4
France	6	6	4
India	7	7	4
Australia	4	4	2
Hungary	9	7	2
South Africa	9	7	2
USA	6	5	0
Bulgaria	2	1	0
Croatia	3	3	0
Korea, Rep.	1	1	0
Russia	2	1	0
Bulgaria	11	9	6
Hong Kong, China	4	3	6
Turkiye	8	3	6

Turkiye	8	3	6
Chile	10	5	6
Czechia	3	3	2
Serbia	8	8	2
Brazil	8	5	2
Croatia	6	5	0
India	8	3	0
Korea, Rep.	6	4	5
USA	5	3	5
Canada	2	1	5
Great Britain	4	2	5
Sweden	3	2	5
India	8	7	5
Ecuador	3	3	5
Peru	4	3	5
Serbia	8	3	5
USA	6	4	5
India	4	3	5
Brazil	3	1	5
Colombia	2	2	5
Ecuador	3	2	5
Sweden	5	3	5
Italy	1	1	5
Switzerland	8	4	5
Egypt	8	7	5
Spain	4	2	5
Mexico	4	3	5
Serbia	5	1	5
Italy	2	1	5
France	5	2	5
Australia	2	2	5
Cameroon	2	2	5
Spain	6	4	5
Mexico	1	1	5
Brazil	3	1	5
Argentina	1	1	5
USA	5	1	2
Costa Rica	6	5	2
Morocco	2	2	4
Venezuela	4	3	4
Czechia	5	2	4
Latvia	12	6	4
Bulgaria	10	7	4
Venezuela	4	3	4
Croatia	11	6	2
Australia	4	4	0
North Macedonia	8	7	0
Ukraine	6	6	0
Belarus	11	6	0
Israel	6	4	0

France	2	1	2
Morocco	3	2	2
Iran	5	4	2
Latvia	12	8	2
Turkiye	5	4	2
Great Britain	9	8	2
Ecuador	2	2	2
Great Britain	4	3	2
Russia	3	3	2
Russia	7	7	2
Australia	6	6	2
Russia	1	1	2
Denmark	3	3	2
Serbia	6	2	2
Poland	10	4	2
Iran	5	5	2
USA	3	2	2
Bosnia and Herzegov	8	4	2
Ukraine	3	3	0
USA	6	3	0
Romania	7	4	5
Canada	4	1	5
Romania	2	0	5
Canada	3	3	5
Germany	3	1	5
Mexico	2	2	5
Germany	10	2	5
France	2	2	5
Russia	1	1	5
Germany	6	0	5
Germany	13	6	5
Netherlands	5	1	5
Russia	1	1	5
Thailand	2	1	5
Russia	3	3	5
Czechia	2	1	5
Thailand	1	1	5
USA	2	1	5
Brazil	7	4	5
India	5	1	5
Peru	2	2	5
Chile	4	4	5
Korea, Rep.	4	3	5
Czechia	4	2	5
USA	3	1	5
Thailand	6	5	5
Bolivia	5	4	5
Latvia	8	3	5
India	1	1	5
Uzbekistan	4	4	5

Russia	2	2	5
Czechia	4	2	5
Thailand	7	1	5
Portugal	6	3	5
Latvia	9	4	5
Bolivia	5	4	5
Latvia	8	3	5
USA	3	1	5
India	5	1	5
Great Britain	1	1	5
Brazil	3	2	5
Cyprus	7	3	5
Uzbekistan	4	4	5
New Zealand	1	1	5
Hong Kong, China	4	3	5
Germany	1	0	5
Israel	4	4	5
France	2	2	5
France	3	1	5
Tunisia	3	1	5
Germany	6	0	5
Bosnia and Herzegov	2	1	5
Chile	4	1	5
Italy	1	1	5
Netherlands	5	1	5
Czechia	2	1	5
Croatia	5	3	5
Korea, Rep.	2	2	5
Thailand	6	5	5
Korea, Rep.	2	2	5
Korea, Rep.	5	4	5
Egypt	4	3	5
Ukraine	9	5	5
Peru	2	1	5
USA	3	3	5
New Zealand	2	2	5
Argentina	1	1	5
Zimbabwe	3	3	5
USA	2	1	5
Samoa	1	1	5
Colombia	5	2	5
USA	4	1	5
Brazil	5	2	5
South Africa	7	5	5
USA	2	1	5
Poland	3	1	5
Netherlands	1	0	5
Greece	2	1	5
India	1	1	5
Iran	5	5	5

Tunisia	1	1	5
Hong Kong, China	4	0	5
USA	1	1	5
Japan	3	2	5
Tunisia	6	2	5
Singapore	2	2	5
USA	5	1	5
USA	2	1	5
USA	2	1	5
Chile	1	1	5
Ghana	5	1	5
Ghana	8	6	5
Russia	2	1	5
Spain	2	1	5
Nigeria	5	2	5
Spain	1	1	5
North Macedonia	10	8	5
USA	9	3	5
Spain	4	3	5
France	2	2	5
Japan	6	1	5
Nigeria	2	2	5
Russia	1	1	5
Norway	2	2	5
USA	6	3	5
China, P.R.	5	3	5
New Zealand	1	1	5
Japan	6	4	5
France	3	1	5
Spain	1	0	5
Germany	2	1	5
Norway	2	2	5
USA	2	0	5
Italy	4	2	5
Nepal	4	4	5
Russia	3	1	5
Germany	3	1	5
Canada	2	2	5
USA	1	1	5
Italy	3	3	5
New Zealand	1	1	5
Italy	1	1	5
Hong Kong, China	3	3	2
Turkiye	7	6	2
Italy	5	3	2
Spain	7	4	2
France	4	2	2
Colombia	2	2	2
Turkiye	10	8	2
Spain	5	3	2

New Zealand	3	2	0
Tunisia	6	4	0
Australia	3	3	0
Brazil	6	4	0
Mexico	3	2	0
Bulgaria	6	4	4
India	4	2	4
Czechia	7	5	4
India	5	2	4
France	6	4	4
Zimbabwe	4	4	4
Finland	6	5	4
Cameroon	2	2	4
Bolivia	4	3	4
Peru	5	2	4
Bolivia	4	2	4
Hong Kong, China	3	1	4
Italy	7	1	4
Poland	8	7	4
Korea, Rep.	5	5	0
Estonia	6	4	2
Russia	4	5	2
South Africa	8	5	2
Israel	3	2	2
Switzerland	4	4	2
Great Britain	10	8	2
Egypt	11	11	2
India	8	5	2
Poland	3	2	2
Korea, Rep.	6	5	2
Georgia	7	7	2
New Zealand	3	3	2
Great Britain	5	4	2
Great Britain	7	4	2
USA	11	11	2
Russia	4	3	0
Australia	2	2	2
Canada	2	2	2
Great Britain	4	3	2
India	2	2	2
Spain	3	3	2
Estonia	13	8	2
Tajikistan	4	4	2
Ukraine	3	3	2
Canada	7	3	2
Egypt	11	11	2
Nepal	5	4	2
Latvia	9	5	0
Australia	3	4	0
Belarus	4	3	0

Croatia	9	4	0
Australia	3	3	0
Australia	4	4	0
Slovakia	4	2	4
France	3	2	4
Colombia	5	4	4
Finland	4	3	4
Latvia	11	5	4
Iran	3	2	4
Serbia	9	0	4
Japan	4	3	4
Mexico	2	2	4
Finland	5	2	4
Korea, Rep.	5	4	4
Great Britain	6	1	4
Bolivia	4	2	4
Venezuela	3	3	4
Brazil	3	2	4
Iran	4	2	4
Japan	3	2	4
Mexico	11	5	4
Kazakhstan	6	2	4
Russia	7	2	4
Canada	3	3	4
Venezuela	3	2	4
Nepal	4	4	0
Russia	12	8	0
Morocco	7	7	0
Belgium	7	7	0
Hong Kong, China	4	4	0
New Zealand	3	3	0
USA	3	3	0
USA	2	2	0
Great Britain	8	4	2
Algeria	4	3	2
South Africa	6	4	2
Egypt	7	7	2
Finland	7	6	2
Brazil	7	4	2
Georgia	6	3	2
Mexico	5	4	2
Greece	3	3	2
Latvia	12	5	2
Korea, Rep.	4	4	2
Australia	4	3	2
Zimbabwe	5	3	0
Kazakhstan	6	2	0
Russia	10	2	0
Romania	3	3	0
USA	6	4	0

Australia	2	2	0
Russia	10	2	0
Romania	3	3	0
Kazakhstan	6	2	0
Estonia	3	2	0
Ukraine	1	1	0
South Africa	7	6	0
USA	6	4	0
Zimbabwe	5	3	0
Australia	2	1	2
Hong Kong, China	2	2	2
Denmark	4	2	2
Belgium	2	2	2
Finland	6	3	2
France	4	2	2
Mexico	5	5	2
South Africa	7	4	2
USA	1	1	2
Peru	3	2	2
Latvia	10	5	2
Sweden	3	3	2
Belgium	4	2	2
Pakistan	4	4	2
Kazakhstan	2	2	2
USA	3	2	2
USA	9	3	2
Czechia	6	4	2
Israel	6	5	2
Turkiye	5	5	2
Peru	5	4	2
Belgium	11	6	2
Kazakhstan	3	3	2
Cameroon	2	2	2
Germany	3	3	2
Belgium	6	6	2
Sweden	4	2	2
USA	1	1	2
Peru	3	2	2
Great Britain	6	3	2
USA	2	2	2
India	5	3	2
Great Britain	2	1	2
Colombia	10	6	2
Slovakia	3	1	2
Slovakia	5	1	2
Portugal	2	2	2
Poland	7	2	2
Venezuela	3	3	2
Italy	3	2	2
Venezuela	3	3	2

Turkiye	5	4	2
India	7	6	2
Venezuela	2	2	2
Bosnia and Herzegov	12	7	2
Uzbekistan	5	5	2
Mexico	5	3	0
Singapore	3	3	0
Brazil	4	3	0
Belarus	2	2	0
Poland	1	1	0
Argentina	2	2	0
Australia	2	2	0
Slovenia	7	3	0
Mexico	4	3	0
Australia	2	2	0
India	15	11	0
Belgium	4	4	0
Portugal	3	1	0
Argentina	2	2	0
Russia	1	1	0
India	10	5	0
Denmark	2	1	0
Australia	2	2	0
USA	1	1	0
Great Britain	4	3	0
Great Britain	5	4	0
Great Britain	1	2	0
Czechia	6	2	0
New Zealand	2	2	0
Mexico	9	2	0
Italy	8	5	0
Montenegro	7	4	0
Sweden	2	1	0
USA	3	3	0
Great Britain	8	5	0
Costa Rica	3	3	0
Belgium	3	2	0
Netherlands	4	4	0
Great Britain	3	3	0
Russia	6	3	0
Romania	11	5	0
Denmark	6	6	0
Morocco	4	3	2
USA	5	3	2
Great Britain	2	2	2
Brazil	7	4	2
Australia	2	2	2
Belarus	4	2	2
Cote D'Ivoire	2	2	2
Kosovo	3	1	2

Morocco	1	1	2
Mexico	9	5	2
North Macedonia	10	9	2
Brazil	7	4	2
Italy	2	1	2
Spain	2	2	2
Zimbabwe	4	4	2
Germany	2	1	2
Great Britain	2	2	2
Belarus	4	2	2
Great Britain	2	2	2
India	1	1	2
Morocco	4	3	2
Thailand	1	1	2
South Africa	3	2	2
Hong Kong, China	4	4	2
Colombia	2	2	2
Italy	1	1	2
Portugal	7	5	2
Hong Kong, China	5	1	2
USA	5	3	2
Egypt	3	1	2
Slovakia	7	6	2
Ukraine	12	6	2
Netherlands	9	4	2
Cote D'Ivoire	2	2	2
Kazakhstan	8	1	2
Moldova	6	3	2
Togo	4	4	2
Peru	4	1	2
Czechia	4	3	2
Venezuela	4	4	2
Bosnia and Herzegov	10	4	2
Japan	1	1	2
Colombia	5	3	2
Korea, Rep.	3	2	2
Iran	5	4	2
Iran	6	5	2
Romania	6	3	2
Australia	4	4	2
Australia	2	2	2
Spain	7	5	2
Hungary	3	1	2
Kosovo	3	1	2
Kenya	3	2	2
Greece	2	2	2
Australia	2	2	2
Australia	1	1	2
USA	2	2	2
Russia	1	1	2

Uzbekistan	4	3	2
Egypt	12	3	2
El Salvador	12	9	2
Netherlands	6	2	2
Japan	5	4	2
Greece	1	1	2
Italy	4	1	2
Bolivia	3	2	2
Czechia	4	2	2
Czechia	3	2	2
Australia	4	4	2
Finland	3	2	2
USA	1	1	2
Russia	2	1	2
Brazil	1	1	2
Vietnam	2	2	2
Peru	6	5	2
Jamaica	3	2	2
Japan	2	2	2
Thailand	4	2	0
New Zealand	2	2	0
Australia	5	4	0
Togo	2	2	0
Russia	4	3	0
Great Britain	6	5	0
Serbia	3	2	0
Bulgaria	7	4	0
Serbia	12	9	0
Belgium	8	4	0
France	9	7	0
Mexico	8	6	0
Austria	7	5	0
India	3	3	0
India	9	4	0
Morocco	4	3	0
Russia	4	3	0
Czechia	10	5	0
Australia	4	4	0
Russia	5	5	0
Netherlands	7	5	0
Korea, Rep.	5	4	0
Venezuela	6	5	0
USA	2	1	2
Lithuania	1	1	2
Belgium	2	2	2
Brazil	1	1	2
Venezuela	2	1	2
Honduras	7	2	2
Russia	5	2	2
Slovakia	2	1	2

Brazil	1	1	2
Australia	2	1	2
Kazakhstan	2	1	2
Russia	5	2	2
Korea, Rep.	4	4	2
USA	4	1	2
Singapore	4	2	2
France	11	7	2
Netherlands	5	2	2
Australia	5	3	2
Slovakia	2	1	2
Chile	1	1	2
Ukraine	6	1	2
Ecuador	3	3	2
Turkiye	4	3	2
El Salvador	8	7	2
Netherlands	4	3	2
USA	2	1	2
Dominican Republic	9	2	2
Hong Kong, China	4	4	2
Bolivia	2	1	2
Switzerland	1	0	2
Finland	3	0	2
Turkiye	4	2	2
South Africa	3	2	2
Mexico	5	2	2
New Zealand	5	0	2
Ukraine	1	1	2
Spain	2	0	2
Egypt	10	8	2
Turkiye	5	2	2
Georgia	3	0	2
Finland	6	1	2
Mexico	4	3	2
Norway	1	0	2
Poland	7	3	2
Bolivia	4	3	2
Greece	3	1	2
India	7	2	2
Belgium	4	3	2
Finland	2	2	2
Japan	4	1	2
Japan	2	2	2
Ecuador	3	0	2
Portugal	3	2	2
Brazil	1	0	2
France	7	5	2
Mexico	1	1	2
France	2	2	2
New Zealand	1	1	2

Iran	3	1	2
Germany	8	2	2
Latvia	7	4	2
USA	1	1	2
Brazil	3	1	2
Poland	3	1	2
Cyprus	1	0	2
Australia	2	0	2
USA	1	1	2
Lithuania	3	1	2
Great Britain	8	2	2
Switzerland	2	1	2
Great Britain	3	3	2
USA	2	1	2
Great Britain	4	2	2
Lithuania	5	2	2
France	2	1	2
Belgium	2	2	2
Colombia	3	2	2
Austria	6	1	2
Latvia	13	9	2
Peru	2	1	2
Austria	2	1	2
USA	8	5	2
Latvia	2	1	2
Czechia	6	1	2
Ireland	4	3	2
Venezuela	2	2	2
Iran	2	0	2
Japan	3	1	2
France	1	0	2
Nigeria	4	2	2
Japan	5	0	2
Russia	3	2	2
Russia	4	4	2
Romania	2	1	2
Brazil	3	1	2
USA	9	2	2
Bulgaria	4	3	2
Canada	2	2	2
Lithuania	1	1	2
Romania	1	1	2
Norway	4	2	2
Mexico	9	6	2
Iran	2	1	2
Syria	2	1	2
Poland	4	1	2
Germany	2	1	2
Bosnia and Herzegov	4	0	2
Ukraine	2	1	2

Norway	4	2	2
Great Britain	3	3	2
Netherlands	4	3	2
Iran	2	0	2
Australia	3	2	2
Costa Rica	6	2	2
Canada	2	2	2
Brazil	3	1	2
Switzerland	2	1	2
Latvia	13	9	2
Netherlands	5	2	2
Iran	2	1	2
India	3	2	2
Italy	2	0	2
Germany	2	1	2
South Africa	6	4	2
Russia	4	4	2
Italy	3	0	2
Germany	5	3	2
Slovakia	4	0	2
Tunisia	6	3	2
Greece	1	1	2
Ecuador	4	4	2
Romania	11	4	2
Japan	3	0	2
Peru	3	0	2
Mexico	9	6	2
Cameroon	2	2	2
Great Britain	5	1	2
Ukraine	1	0	2
Great Britain	4	3	2
Colombia	1	1	2
Canada	5	1	2
Tunisia	3	1	2
France	1	0	2
Iran	4	4	2
Russia	5	2	2
Brazil	2	1	2
Japan	5	0	2
Nicaragua	2	1	2
Paraguay	1	1	2
Costa Rica	4	3	2
France	7	5	2
Venezuela	1	1	2
Pakistan	2	2	2
Iran	1	1	2
New Zealand	1	1	2
Nepal	2	2	2
Venezuela	4	2	2
USA	5	3	2

Great Britain	4	0	2
Brazil	1	0	2
India	2	0	2
USA	3	2	2
Australia	2	1	2
Canada	2	1	0
Slovenia	7	4	0
New Zealand	1	1	0
Japan	4	2	0
Germany	3	1	0
Zimbabwe	4	3	0
New Zealand	1	1	0
Canada	1	1	0
Slovakia	3	3	0
Luxembourg	5	1	0
New Zealand	2	2	0
Egypt	6	6	0
Korea, Rep.	3	2	0
USA	1	1	0
USA	2	2	0
Canada	4	2	0
Romania	3	1	0
Croatia	2	2	0
Egypt	8	8	0
USA	1	1	0
Germany	2	1	0
Chile	6	4	0
Colombia	4	4	0
Kenya	1	1	0
Bolivia	2	1	0
Egypt	4	2	0
Israel	5	2	0
Japan	3	2	0
Belgium	2	2	0
Lithuania	12	4	0
Croatia	3	2	0
Mexico	3	3	0
Romania	1	1	0
Sweden	3	1	0
USA	2	2	0
Romania	4	3	0
Great Britain	2	3	0
Great Britain	4	1	0
Canada	4	4	0
Kenya	4	4	0
Sweden	3	2	0
Israel	7	4	0
Belarus	2	2	0
Russia	2	2	0
Sweden	5	3	0

Israel	5	2	0
Zimbabwe	4	3	0
Canada	2	1	0
Czechia	2	1	0
Zambia	4	3	0
Canada	4	4	0
Luxembourg	5	1	0
Great Britain	2	1	0
Bulgaria	3	2	0
USA	1	1	0
Kenya	3	2	0
Russia	1	1	0
USA	1	1	0
Canada	2	1	0
Canada	5	4	0
France	7	6	0
Hungary	11	8	0
Slovakia	3	3	0
Australia	1	1	0
Bosnia and Herzegov	4	1	0
Australia	1	2	0
Romania	3	1	0
India	4	2	0
France	6	1	0
Germany	3	1	0
Romania	1	1	0
USA	1	1	0
Sweden	3	2	0
Sweden	5	3	0
Sweden	3	2	0
Germany	5	2	0
Great Britain	2	3	0
Thailand	1	1	0
Bolivia	2	1	0
Latvia	4	1	0
Sweden	3	1	0
Ecuador	2	1	0
Slovenia	7	4	0
Great Britain	4	1	0
Thailand	2	2	0
Canada	2	2	0
Chile	6	4	0
Russia	1	1	0
Cyprus	6	1	0
USA	11	6	0
Russia	5	2	0
Greece	2	2	0
Egypt	4	2	0
Slovakia	3	1	0
Cyprus	4	4	0

Ukraine	5	3	0
Brazil	2	1	0
Ecuador	3	1	0
Romania	4	1	0
France	3	3	0
Israel	7	4	0
Australia	2	3	0
Bulgaria	4	2	0
USA	3	2	0
Australia	4	4	0
Czechia	1	1	0
Kenya	9	6	0
Nigeria	2	1	0
Venezuela	3	3	0
Slovakia	3	1	0
France	2	1	0
Russia	2	1	0
Nepal	4	3	0
Finland	6	5	0
Cameroon	2	2	0
Turkiye	6	2	0
Colombia	4	2	0
Turkiye	8	3	0
Italy	3	2	0
Egypt	9	7	0
Bolivia	4	3	0
Nigeria	2	2	0
Panama	4	4	0
Egypt	9	8	0
Spain	1	1	0
Slovakia	3	3	0
Brazil	5	1	0
Kazakhstan	3	1	0
Korea, Rep.	6	5	0
USA	4	3	0
Czechia	3	1	0
Finland	6	5	0
Venezuela	5	5	0
Estonia	3	3	0
Japan	9	7	0
Italy	4	2	0
Colombia	5	4	0
Austria	6	2	0
Australia	1	1	0
Bolivia	8	6	0
Peru	2	2	0
Japan	2	1	0
Hungary	8	5	0
Spain	2	2	0
Zimbabwe	4	1	0

Spain	4	1	0
Bulgaria	12	9	0
Estonia	10	3	0
Montenegro	3	1	0
Turkiye	6	2	0
Slovakia	3	1	0
Korea, Rep.	6	5	0
Colombia	5	4	0
Zimbabwe	4	1	0
Czechia	3	3	0
India	4	1	0
France	2	1	0
Italy	3	2	0
Hungary	8	5	0
Czechia	7	3	0
Russia	2	1	0
Finland	6	5	0
Turkiye	10	6	0
Turkiye	8	3	0
Czechia	4	3	0
Germany	1	1	0
Slovakia	3	3	0
Bolivia	4	3	0
Spain	2	2	0
Italy	4	2	0
Nepal	4	3	0
France	2	2	0
Germany	5	3	0
Estonia	3	3	0
USA	3	3	0
Russia	2	1	0
Austria	6	2	0
Bulgaria	7	1	0
Finland	2	2	0
Sweden	2	2	0
Russia	5	3	0
Serbia	4	3	0
Russia	4	2	0
Serbia	6	3	0
Serbia	7	4	0
Portugal	7	5	0
Kazakhstan	11	5	0
France	1	1	0
Georgia	4	4	0
Germany	2	2	0
Italy	1	1	0
Denmark	4	3	0
Italy	1	1	0
Croatia	7	5	0
Bulgaria	4	2	0

Belgium	5	2	0
Austria	6	3	0
Poland	7	3	0
Korea, Rep.	4	3	0
Serbia	3	2	0
Japan	1	1	0
Colombia	2	2	0
Colombia	4	2	0
Peru	2	2	0
Venezuela	3	2	0
Colombia	2	1	0
Dominican Republic	8	2	0
Russia	8	4	0
Korea, Rep.	3	2	0
Korea, Rep.	4	4	0
Japan	3	2	0
Austria	5	2	0
Hong Kong, China	4	4	0
Russia	3	1	0
Russia	4	1	0
Ukraine	4	4	0
Russia	1	1	0
Serbia	3	1	0
Georgia	4	3	0
Kosovo	4	2	0
Australia	1	1	0
Korea, Rep.	1	1	0
Russia	11	5	0
Slovakia	2	1	0
France	2	2	0
Russia	6	4	0
Thailand	6	6	0
Egypt	6	3	0
Tunisia	10	5	0
Brazil	2	1	0
Spain	1	1	0
India	5	2	0
India	1	1	0
Zimbabwe	8	5	0
Cote D'Ivoire	3	2	0
Greece	1	1	0
Brazil	2	1	0
Switzerland	2	1	0
Italy	1	1	0
Czechia	5	2	0
Peru	2	1	0
Italy	2	1	0
Romania	4	3	0
Russia	2	1	0
Switzerland	2	1	0

Switzerland	3	1	0
USA	1	1	0
Japan	7	2	0
Switzerland	4	1	0
USA	2	1	0
Togo	4	4	0
El Salvador	9	6	0
Hong Kong, China	3	2	0
Colombia	2	2	0
Singapore	2	1	0
Peru	10	4	0
South Africa	8	5	0
Denmark	5	3	0
USA	3	1	0
Bolivia	3	3	0
Italy	2	1	0
Greece	2	2	0
Czechia	1	1	0
Peru	5	4	0
Italy	1	1	0
Poland	7	3	0
Argentina	3	2	0
Greece	2	1	0
Czechia	16	3	0
Romania	5	3	0
Denmark	1	1	0
Ecuador	3	3	0
Sweden	5	3	0
USA	10	5	0
Cote D'Ivoire	2	2	0
Kazakhstan	7	1	0
Kazakhstan	18	8	0
Egypt	6	5	0
Colombia	3	2	0
Russia	2	1	0
Italy	4	1	0
USA	2	2	0
Belgium	8	5	0
Hong Kong, China	3	3	0
India	5	4	0
Germany	6	2	0
Cyprus	5	2	0
Japan	3	2	0
Russia	6	3	0
Brazil	4	2	0
Venezuela	3	1	0
Italy	3	1	0
Nepal	4	2	0
Brazil	3	3	0
Brazil	7	2	0

Nepal	2	2	0
Mexico	6	3	0
Russia	10	8	0
Colombia	5	1	0
Mexico	10	4	0
Japan	3	2	0
USA	3	1	0
Japan	7	3	0
India	4	2	0
Brazil	4	2	0
Kazakhstan	14	3	0
Denmark	5	3	0
Canada	2	1	0
USA	8	3	0
USA	4	2	0
Brazil	1	1	0
Poland	8	3	0
Russia	7	2	0
USA	4	2	0
Great Britain	5	2	0
Belgium	5	2	0
Sweden	4	2	0
Ecuador	2	2	0
Tajikistan	1	2	0
USA	3	1	0
Russia	12	9	0
Ukraine	6	2	0
Nepal	2	2	0
Mexico	6	4	0
France	2	1	0
Spain	1	1	0
Peru	2	2	0
Peru	2	2	0
Venezuela	4	3	0
Japan	6	1	0
Venezuela	3	3	0
Hong Kong, China	4	3	0
Korea, Rep.	3	2	0
USA	6	4	0
Mexico	5	3	0
USA	5	5	0
Turkiye	4	1	0
Spain	1	1	0
Spain	2	1	0
USA	1	1	0
Brazil	1	1	0
Pakistan	3	3	0
Benin	3	2	0
Venezuela	4	3	0
Paraguay	2	1	0

Mexico	2	2	0
South Africa	7	4	0
Brazil	2	1	0
Venezuela	3	2	0
Australia	1	1	0
India	2	2	0
Great Britain	5	3	0
Costa Rica	8	1	0
South Africa	2	1	0
Cote D'Ivoire	8	6	0
Czechia	2	2	0
India	2	1	0
Russia	4	2	0
Mexico	4	2	0
Paraguay	1	1	0
Brazil	5	3	0
India	3	2	0
Uzbekistan	6	3	0
Japan	5	4	0
Brazil	6	2	0
Thailand	2	1	0
Italy	1	1	0
Cameroon	2	2	0
Italy	3	2	0
Thailand	4	3	0
Denmark	1	1	0
Brazil	2	1	0
Australia	2	2	0
Canada	6	6	0
Ecuador	1	1	0
Iran	1	1	0
Japan	6	5	0
Canada	3	1	0
Canada	5	3	0
Russia	2	2	0
Ecuador	1	1	0
Ethiopia	8	6	0
France	15	9	2430
Andorra	21	20	2210
Philippines	13	13	1755
France	7	6	1815
Ukraine	4	0	1930
Russia	13	10	1670
USA	13	13	1450
Germany	15	11	1390
Belarus	15	15	1220
USA	5	3	1415
Latvia	12	11	845
Poland	23	19	1195
Russia	11	10	850

Russia	11	11	1105
Russia	14	14	960
Canada	2	2	1280
Indonesia	13	14	950
Czechia	13	14	935
Peru	14	11	1010
Great Britain	17	17	855
Czechia	12	10	970
Hong Kong, China	16	16	1000
China, P.R.	13	13	955
Ecuador	18	17	925
Belgium	19	17	880
Russia	20	20	800
Belarus	14	11	890
China, P.R.	5	4	985
USA	16	14	965
Italy	16	13	846
Italy	12	9	895
USA	14	10	910
France	20	19	850
Uruguay	18	17	630
Czechia	12	10	920
Canada	11	10	750
Belarus	15	13	780
Thailand	13	13	750
USA	21	21	580
France	17	12	545
USA	24	23	715
Japan	9	7	605
Spain	16	15	810
Armenia	14	13	820
Spain	14	13	775
Hungary	21	18	790
Slovenia	19	18	555
Mexico	18	18	730
Russia	15	12	760
France	18	16	681
Belarus	13	12	585
Switzerland	15	14	716
Argentina	13	12	670
Korea, Rep.	9	9	690
Hungary	17	15	650
France	14	12	395
Slovakia	18	15	645
Germany	18	17	655
Finland	21	21	606
Russia	16	14	642
Italy	13	12	640
Italy	13	14	410
Spain	12	12	575

France	18	18	495
Italy	12	10	420
Chinese Taipei	13	13	546
Germany	16	6	636
Australia	13	13	645
Slovakia	21	21	550
Korea, Rep.	15	12	505
USA	9	9	660
Czechia	14	12	595
USA	7	5	636
Russia	17	14	540
France	22	16	450
Australia	27	21	571
Korea, Rep.	9	8	465
France	20	19	500
Croatia	18	18	570
Russia	19	18	510
Belgium	20	20	580
Puerto Rico	24	22	500
USA	13	13	205
Germany	20	17	510
Great Britain	17	10	575
Ukraine	17	16	561
Canada	14	14	505
Slovenia	15	16	495
Japan	20	21	385
Ukraine	14	13	400
France	18	19	400
Italy	17	17	496
Latvia	22	21	446
Kazakhstan	24	24	560
Cyprus	10	11	506
Germany	16	14	470
Australia	7	7	475
USA	13	13	516
Russia	19	17	456
Germany	14	13	400
Canada	17	17	480
Croatia	17	16	480
Russia	20	19	510
China, P.R.	20	19	387
Peru	10	10	400
Argentina	8	5	472
France	22	19	400
Russia	9	8	480
Japan	23	23	392
Great Britain	26	25	401
Venezuela	18	18	408
Russia	22	22	400
Romania	17	12	316

USA	25	22	401
Mexico	21	21	406
Slovakia	23	21	440
USA	19	13	440
Australia	21	21	385
USA	19	16	466
Thailand	16	13	386
Germany	17	15	440
Argentina	16	16	417
Czechia	24	17	350
Belgium	19	19	371
Japan	12	10	310
Czechia	20	18	416
USA	19	16	346
Croatia	27	20	366
China, P.R.	16	16	449
Serbia	21	21	386
Great Britain	11	11	420
Malta	9	7	380
Sweden	16	16	366
Switzerland	20	19	370
Spain	12	10	300
USA	6	5	305
China, P.R.	14	11	390
Indonesia	11	12	356
Morocco	17	17	336
Switzerland	19	18	350
Great Britain	16	15	346
Greece	16	14	376
Poland	17	16	352
Bolivia	20	20	341
Australia	19	19	340
USA	22	17	318
USA	15	15	357
Australia	13	8	380
Nigeria	11	11	330
Slovakia	18	13	391
Algeria	13	12	359
Russia	24	19	326
USA	13	7	330
Poland	12	11	352
Chile	15	15	325
Germany	17	16	360
USA	10	9	358
Croatia	10	10	275
China, P.R.	16	15	310
Chinese Taipei	14	14	212
Australia	11	11	320
France	20	15	328
Canada	15	15	298

USA	7	7	375
Israel	17	13	230
Belgium	22	22	266
Germany	18	17	298
Bulgaria	17	15	346
Latvia	17	16	281
Thailand	5	5	358
USA	19	19	316
Austria	27	22	273
Peru	10	10	282
Russia	16	16	346
Russia	14	14	274
Australia	16	15	320
China, P.R.	15	15	217
Korea, Rep.	13	13	255
Italy	17	14	308
USA	16	16	291
Thailand	14	7	295
Netherlands	21	20	301
USA	18	16	266
USA	28	22	250
Kazakhstan	23	18	306
Chinese Taipei	13	11	240
Russia	14	14	316
China, P.R.	12	8	280
Serbia	21	14	308
Russia	18	18	288
USA	13	10	276
Canada	16	13	292
Chinese Taipei	16	15	313
Japan	16	16	276
Russia	8	7	284
Canada	17	14	262
Peru	13	13	232
India	10	9	270
Turkiye	29	28	250
Egypt	23	20	296
Japan	14	14	264
Portugal	16	16	243
Tunisia	19	18	276
USA	11	9	285
Netherlands	10	10	263
Mexico	20	20	270
Czechia	14	12	290
Poland	19	18	248
Lithuania	21	20	260
Germany	16	16	290
Russia	24	18	228
Latvia	26	23	252
Spain	20	15	224

Israel	21	22	266
Czechia	25	17	202
Slovakia	19	16	266
Thailand	6	4	259
Italy	9	8	138
Russia	17	16	240
France	20	18	186
Belgium	21	18	256
Cameroon	12	12	225
Japan	14	12	231
Thailand	18	5	298
Canada	14	13	216
China, P.R.	4	4	255
Egypt	13	11	260
Burundi	14	13	216
Greece	11	10	252
Italy	10	8	270
Great Britain	16	11	188
El Salvador	20	20	230
Tunisia	17	16	228
Egypt	19	19	220
Italy	16	13	249
Bulgaria	21	20	243
USA	12	10	251
Switzerland	18	18	203
Russia	12	5	225
USA	9	9	158
Czechia	13	10	262
USA	16	16	212
Australia	10	10	214
USA	6	6	190
Czechia	17	12	224
Serbia	21	3	272
Romania	18	15	222
Latvia	15	13	236
Poland	22	22	212
USA	12	11	218
Russia	22	22	218
Slovakia	17	17	230
USA	16	12	205
France	17	17	228
France	17	12	252
Turkiye	20	15	236
Russia	17	13	156
Romania	16	13	240
Germany	13	8	221
USA	8	7	240
Italy	11	10	175
Belarus	9	9	194
Morocco	11	8	188

USA	18	16	226
China, P.R.	16	15	234
Russia	13	13	160
Brazil	14	14	195
Slovakia	18	16	166
Italy	11	8	182
Czechia	18	17	178
Kenya	12	10	165
Belgium	10	10	218
Ecuador	16	16	198
Brazil	18	17	206
Japan	22	21	156
Botswana	15	15	206
South Africa	16	14	210
Brazil	18	17	194
Sweden	7	7	194
Russia	17	17	171
Bulgaria	14	10	200
Russia	19	19	220
Czechia	7	7	165
Turkiye	22	22	198
USA	13	12	130
Hungary	21	20	198
USA	8	8	146
Hong Kong, China	8	9	151
Romania	22	16	212
Finland	22	21	187
Czechia	14	10	176
Italy	14	13	149
Czechia	18	12	190
USA	5	4	216
Austria	25	22	142
Croatia	18	17	186
USA	12	10	218
Russia	18	17	186
Thailand	7	7	176
Russia	15	10	184
Brazil	18	17	133
Belgium	18	16	170
Belarus	9	9	175
Russia	17	17	204
France	15	14	185
USA	14	13	104
France	17	13	170
Morocco	13	11	157
Czechia	4	4	200
Canada	7	6	202
Russia	20	18	132
Thailand	9	1	221
Hungary	13	12	176

Japan	16	16	164
Canada	14	12	184
France	20	17	182
South Africa	8	8	184
Switzerland	12	7	184
Chinese Taipei	12	12	179
Kazakhstan	21	20	178
New Zealand	18	16	164
China, P.R.	12	11	185
Spain	13	8	198
Switzerland	24	18	165
Paraguay	16	14	171
South Africa	16	16	163
Ukraine	10	10	165
USA	6	6	164
Netherlands	16	11	173
New Zealand	18	18	156
Canada	12	12	172
Croatia	13	13	184
Colombia	18	15	130
Argentina	13	12	192
Russia	3	1	190
China, P.R.	11	11	161
Israel	17	17	170
Georgia	14	12	160
France	14	12	180
USA	8	6	200
Bulgaria	14	13	164
Canada	6	6	140
Spain	13	6	189
France	9	7	196
Japan	23	23	105
USA	14	14	150
Netherlands	11	10	125
Romania	15	12	154
Canada	15	15	167
Uzbekistan	10	10	171
Germany	18	16	166
Egypt	11	10	162
Australia	8	7	154
Japan	21	21	152
Hong Kong, China	21	17	109
China, P.R.	9	9	80
Czechia	19	18	152
Serbia	20	20	100
Australia	14	13	114
New Caledonia	18	17	164
Canada	13	12	156
Estonia	13	6	177
Colombia	19	13	180

Morocco	9	7	163
Australia	12	12	134
Korea, Rep.	6	6	164
Russia	20	14	168
Spain	13	12	122
France	22	20	138
Netherlands	12	12	154
China, P.R.	14	13	144
Spain	15	12	148
Georgia	15	14	138
Brazil	11	10	158
Belarus	5	4	115
Denmark	17	16	152
India	10	9	132
Russia	18	17	136
Mexico	18	17	144
Lithuania	24	23	117
USA	15	14	124
Sweden	10	10	151
USA	1	1	170
USA	7	7	135
India	13	11	138
Tunisia	8	6	153
St. Kitts & Nevis	23	19	144
Russia	6	5	162
Russia	16	15	114
Spain	15	10	159
Mexico	13	12	104
China, P.R.	17	17	145
Poland	15	15	131
Japan	17	15	120
Bolivia	13	12	128
Russia	21	18	148
USA	12	4	165
USA	15	14	136
Ukraine	19	19	109
Moldova	16	14	113
Colombia	10	10	114
Turkiye	17	16	84
USA	5	5	160
Austria	5	5	120
Italy	16	13	45
Brazil	8	7	98
USA	7	6	50
Malta	12	8	135
Brazil	6	5	139
Canada	9	5	160
USA	13	10	134
India	8	8	150
China, P.R.	13	12	155

Serbia	13	9	151
Russia	13	12	104
Germany	8	5	125
South Africa	11	11	118
Czechia	22	21	104
South Africa	14	14	132
China, P.R.	11	11	144
Spain	11	10	136
France	17	13	126
Slovakia	19	19	125
Russia	7	6	151
Bulgaria	11	9	143
Chinese Taipei	13	12	103
Netherlands	12	12	92
Argentina	13	10	153
France	8	6	113
Turkiye	16	14	138
Bulgaria	19	15	100
Russia	12	11	82
Venezuela	14	14	128
Slovakia	20	18	122
Czechia	3	3	146
China, P.R.	12	12	133
Spain	7	7	111
Czechia	17	15	116
Chinese Taipei	15	12	128
Canada	18	12	123
Ukraine	7	4	134
Japan	16	12	98
Korea, Rep.	14	13	124
Japan	12	10	112
Belarus	17	16	67
USA	19	16	116
Thailand	11	11	132
Japan	4	4	110
North Macedonia	20	18	126
China, P.R.	18	17	49
Italy	17	17	93
France	14	14	128
Russia	18	17	100
Ukraine	22	22	95
Latvia	7	7	110
Turkiye	12	11	120
Ukraine	9	9	104
Kazakhstan	19	19	110
Russia	15	14	123
Egypt	13	13	129
Russia	24	15	114
Turkiye	20	19	110
Luxembourg	14	13	115

China, P.R.	11	11	111
Italy	12	8	119
Iran	13	13	119
France	17	16	112
Tunisia	15	12	96
Egypt	15	14	100
Czechia	17	17	96
Colombia	11	11	114
Kazakhstan	12	12	93
USA	6	4	90
Sweden	13	10	130
Norway	15	15	119
Poland	17	15	104
Romania	21	15	115
Japan	6	6	118
Dominican Republic	19	18	96
Serbia	13	12	130
Canada	9	9	85
Romania	9	8	114
Germany	19	17	104
Austria	14	13	78
USA	9	9	116
Barbados	14	13	114
USA	12	11	104
Czechia	18	14	112
USA	12	10	106
Russia	4	4	65
Germany	16	15	121
Russia	16	16	106
Singapore	20	18	114
Kazakhstan	19	12	130
Ukraine	9	3	132
Russia	9	9	60
Canada	12	7	120
Italy	10	6	121
Israel	17	15	124
Kazakhstan	18	17	94
Switzerland	10	7	91
Ukraine	8	6	100
South Africa	11	11	102
Romania	10	9	109
Korea, Rep.	8	8	103
Switzerland	14	13	92
Canada	12	11	100
Netherlands	18	18	84
Japan	13	13	81
Chile	11	10	84
Czechia	15	16	110
Brazil	16	15	91
Colombia	10	8	114

Kazakhstan	13	13	110
Australia	14	14	77
China, P.R.	13	12	113
New Zealand	11	11	110
Australia	9	9	97
Brazil	17	16	106
USA	5	4	120
Chinese Taipei	9	9	78
USA	16	15	84
Sweden	11	11	73
India	13	6	121
Estonia	21	18	95
Lithuania	19	15	112
Bulgaria	14	11	93
Romania	17	7	110
Russia	4	4	110
Sweden	23	21	76
Italy	15	14	86
Great Britain	16	15	92
Belarus	14	13	110
Australia	6	4	123
China, P.R.	11	11	97
Italy	18	14	93
India	15	14	94
USA	21	18	81
Thailand	17	17	103
China, P.R.	10	9	109
Bulgaria	17	12	120
Portugal	12	11	96
Germany	9	6	91
France	14	11	86
Canada	27	24	101
Russia	7	7	80
Czechia	9	8	73
South Africa	17	17	98
France	8	8	94
Kazakhstan	12	12	92
India	10	9	99
China, P.R.	8	8	112
Poland	3	3	120
Ukraine	19	16	68
Bulgaria	14	11	77
Croatia	13	10	87
USA	9	9	84
USA	14	9	96
Italy	16	11	81
China, P.R.	15	14	98
Slovakia	16	12	94
China, P.R.	10	10	104
Romania	15	13	81

Mexico	13	12	102
Russia	24	20	104
India	11	10	100
Lithuania	24	24	68
Montenegro	15	14	84
Kazakhstan	25	24	70
Colombia	6	5	93
Russia	7	5	114
Bulgaria	13	11	95
Italy	11	10	70
South Africa	5	1	95
France	13	13	86
USA	15	14	77
China, P.R.	3	3	80
Australia	6	4	105
Croatia	22	19	55
Bulgaria	23	19	94
Madagascar	17	15	83
Russia	13	11	75
Finland	15	14	65
Thailand	8	9	78
USA	4	4	91
France	18	15	86
Russia	17	14	87
Venezuela	11	11	87
Switzerland	20	17	84
Russia	18	16	104
Italy	11	8	52
Romania	13	12	75
Estonia	17	13	81
Puerto Rico	5	4	97
Ukraine	14	4	110
Korea, Rep.	12	11	92
Ukraine	10	10	60
Brazil	9	8	47
France	18	16	72
Great Britain	17	17	89
Canada	11	10	73
Austria	26	19	76
Great Britain	16	12	61
Paraguay	13	13	68
Slovakia	22	20	80
Brazil	15	14	82
Czechia	15	11	70
South Africa	8	8	50
Slovakia	15	10	71
Thailand	16	15	77
Portugal	1	1	100
USA	8	7	58
USA	16	11	94

Bosnia and Herzegov	12	11	77
Slovakia	12	11	82
Russia	15	13	85
France	14	13	80
Japan	6	4	101
Slovenia	23	18	78
USA	17	16	66
Italy	22	15	79
USA	8	8	61
China, P.R.	14	12	81
China, P.R.	11	10	63
Thailand	17	16	56
Chinese Taipei	9	9	63
Russia	16	14	82
Hong Kong, China	8	8	79
Lithuania	22	15	68
China, P.R.	8	8	82
USA	23	21	60
Mexico	16	16	68
Russia	14	12	63
Slovenia	13	10	71
USA	4	4	85
China, P.R.	13	11	92
Turkiye	20	19	69
Romania	7	7	40
Austria	5	5	75
Hong Kong, China	18	16	47
Slovenia	20	18	77
Canada	3	3	95
Tunisia	8	8	74
USA	15	15	78
Romania	20	17	69
China, P.R.	13	13	48
Serbia	24	12	82
India	11	11	74
Bosnia and Herzegov	13	7	69
Venezuela	15	12	87
Serbia	12	7	96
Chinese Taipei	20	20	58
Nigeria	10	10	81
Chinese Taipei	12	11	61
Slovakia	4	4	60
Russia	21	21	64
Argentina	13	13	80
Estonia	23	21	65
France	17	15	74
Estonia	10	10	60
Greece	4	4	88
Great Britain	22	16	73
Hungary	15	10	80

Italy	12	7	86
Australia	8	8	38
Ukraine	16	15	68
Belarus	17	17	53
Germany	13	11	76
Romania	20	19	57
USA	13	11	62
Romania	17	13	52
Canada	21	21	67
Czechia	16	11	57
Japan	8	7	50
Italy	17	15	74
Poland	16	11	69
Poland	11	11	61
Poland	22	19	66
Italy	9	7	55
Denmark	16	15	70
South Africa	6	5	83
Ukraine	14	9	59
Romania	22	17	59
Argentina	9	9	75
Egypt	15	13	78
USA	10	9	65
Croatia	24	23	63
Iran	15	13	77
Italy	13	10	57
Russia	8	8	65
Russia	9	8	50
Sweden	14	9	81
USA	16	6	88
Japan	18	17	85
Italy	3	3	55
Guatemala	14	14	48
India	9	9	48
Germany	9	6	69
Kazakhstan	17	15	65
Netherlands	18	14	63
Slovenia	17	14	73
Israel	17	14	80
Ukraine	1	0	90
Russia	4	4	80
Russia	14	12	71
Russia	11	10	66
USA	9	5	77
Israel	10	5	77
Slovakia	9	9	56
Greece	9	9	75
Great Britain	7	6	65
Norway	12	11	73
Russia	12	12	61

USA	13	10	74
Turkiye	17	17	63
Luxembourg	21	21	62
USA	12	12	66
USA	9	8	72
USA	15	12	66
Austria	21	20	65
Poland	15	14	78
Korea, Rep.	6	6	63
Germany	14	8	44
Sweden	7	6	81
Mexico	16	17	53
India	13	13	44
USA	8	8	15
France	9	9	65
Russia	14	9	69
France	19	15	74
Russia	4	2	78
Morocco	15	11	70
Russia	10	10	45
Sweden	11	9	74
USA	5	5	61
USA	12	7	76
Peru	5	4	65
Italy	12	10	60
Great Britain	14	10	50
China, P.R.	9	9	63
Thailand	4	4	60
Netherlands	12	11	48
India	9	9	72
Nepal	15	11	63
Zimbabwe	15	15	64
China, P.R.	7	7	63
Germany	20	16	60
USA	21	18	53
USA	4	3	53
Kazakhstan	22	19	50
Spain	10	11	70
Korea, Rep.	7	7	53
Turkiye	12	12	68
Great Britain	7	7	76
Slovenia	14	11	66
Spain	8	5	75
Russia	6	5	30
India	9	7	68
Czechia	8	7	72
Serbia	15	11	58
Bolivia	7	7	60
Australia	9	9	60
Colombia	5	5	63

USA	12	7	79
Canada	10	9	58
Peru	10	9	52
Romania	14	12	70
Germany	15	11	56
Belarus	24	22	35
Russia	18	14	60
Czechia	8	5	80
Ukraine	21	16	61
USA	12	10	61
USA	20	19	56
France	17	17	55
Korea, Rep.	14	14	68
Philippines	4	4	75
Belarus	15	11	41
Australia	8	7	53
Puerto Rico	8	8	48
China, P.R.	14	11	55
China, P.R.	14	13	53
Belgium	13	12	53
Czechia	7	7	52
Chinese Taipei	11	10	28
South Africa	13	13	59
Italy	6	5	56
Mexico	16	16	60
Slovakia	15	12	70
USA	10	9	44
Indonesia	9	9	65
Norway	14	12	68
USA	5	4	51
Mexico	13	12	43
France	13	13	52
France	13	11	65
Portugal	10	10	32
Chinese Taipei	13	13	35
Korea, Rep.	5	5	62
Italy	7	6	53
India	10	9	68
Cyprus	11	0	75
Belgium	10	9	49
Poland	19	17	58
Estonia	20	19	53
Australia	10	10	52
Spain	13	8	57
Cameroon	8	8	52
New Zealand	9	9	58
USA	4	4	60
Egypt	14	13	62
France	9	8	45
Hungary	14	12	54

Russia	10	8	43
Great Britain	4	4	35
Russia	4	4	20
Greece	16	15	52
Netherlands	23	20	45
Mexico	13	12	48
Armenia	6	5	63
Colombia	14	14	43
France	19	15	48
Sri Lanka	6	4	67
Kazakhstan	13	10	60
Brazil	11	11	47
Uzbekistan	10	7	40
Denmark	15	9	60
France	14	12	57
Mexico	19	19	45
Brazil	10	9	54
China, P.R.	16	16	52
Great Britain	13	12	53
Great Britain	9	9	52
China, P.R.	10	8	44
Guatemala	20	18	35
Russia	10	9	54
Great Britain	10	7	62
Russia	10	8	54
Belgium	15	12	43
USA	10	9	54
France	16	15	47
Russia	10	7	64
Tunisia	15	13	55
Canada	13	12	53
Japan	7	6	47
Ireland	13	10	56
Great Britain	16	14	47
Germany	11	10	54
Russia	15	14	48
Russia	11	8	46
Switzerland	22	19	38
Korea, Rep.	10	10	53
Germany	1	1	15
Serbia	7	4	51
Mexico	10	10	48
Venezuela	14	14	42
Argentina	10	10	42
Germany	15	15	36
Japan	17	15	52
Turkiye	18	17	44
Russia	6	6	45
South Africa	8	8	45
Great Britain	10	9	34

Bolivia	7	7	55
Australia	6	6	48
Great Britain	9	8	54
Canada	7	7	39
Russia	2	1	62
Italy	13	8	55
Romania	6	5	55
Romania	11	8	60
Latvia	15	9	50
Egypt	17	16	27
Germany	7	3	61
Sweden	9	9	39
Great Britain	8	8	40
Netherlands	7	6	25
Belgium	13	11	50
Italy	6	6	30
Romania	10	9	55
Russia	15	14	42
Hungary	4	5	35
Norway	21	17	53
France	10	9	46
Switzerland	16	11	35
Belgium	13	13	40
Czechia	18	15	51
Argentina	16	14	42
USA	9	6	50
Spain	8	6	50
USA	11	7	52
Italy	12	10	34
China, P.R.	8	8	42
Chinese Taipei	16	14	31
Japan	4	2	60
Poland	23	22	45
Sweden	11	11	40
Japan	7	7	52
Fiji	9	8	49
Nigeria	14	12	51
Great Britain	11	10	37
Israel	13	9	56
Bosnia and Herzegov	12	6	59
France	10	9	52
Peru	7	6	43
France	19	17	37
South Africa	4	4	47
China, P.R.	9	6	55
Ukraine	1	1	60
Poland	17	14	32
Bolivia	8	8	21
France	18	15	31
Cote D'Ivoire	7	7	44

Portugal	8	8	46
Japan	13	12	38
Greece	15	12	45
Austria	18	15	42
Belarus	6	6	32
Canada	8	8	47
Canada	12	12	47
USA	10	9	48
Belgium	19	13	50
USA	12	11	36
Korea, Rep.	8	8	35
Japan	8	7	39
Argentina	6	6	48
Russia	15	15	43
Russia	9	9	39
Slovenia	13	9	45
Ukraine	9	8	44
Greece	14	13	22
Kazakhstan	21	21	39
USA	4	2	51
Russia	12	9	45
Great Britain	3	3	53
Bolivia	5	5	54
Croatia	11	12	48
Slovakia	19	13	45
Great Britain	8	7	35
Chinese Taipei	9	9	41
Poland	15	15	48
Poland	19	16	33
Slovenia	11	9	55
Slovenia	16	14	25
Australia	9	8	53
Norway	8	6	35
Japan	9	9	46
Bolivia	7	7	45
Kazakhstan	18	17	23
France	15	15	45
South Africa	9	8	37
Belarus	14	11	28
Russia	22	20	39
Cyprus	15	14	29
Russia	6	4	49
Kazakhstan	13	13	38
Venezuela	11	11	32
New Zealand	3	2	54
Canada	6	6	38
Spain	12	6	51
Egypt	14	8	53
Burundi	11	11	36
Thailand	14	13	32

Australia	13	13	18
Kazakhstan	5	5	32
Latvia	27	24	44
Slovenia	17	15	33
Venezuela	5	5	42
France	13	10	48
India	12	11	34
Russia	15	14	48
Chinese Taipei	7	6	48
Tunisia	15	8	36
Switzerland	15	7	47
Germany	9	6	50
Egypt	13	12	39
Palestine	10	10	48
France	8	8	25
Russia	18	15	39
Romania	22	18	29
Madagascar	16	14	39
Ukraine	10	7	45
Luxembourg	11	10	38
Croatia	15	9	36
Italy	10	10	34
Great Britain	14	14	22
Philippines	16	15	44
India	13	12	35
Turkiye	20	18	28
Uzbekistan	9	8	36
France	18	18	40
Korea, Rep.	7	7	37
USA	5	4	37
New Caledonia	4	3	38
Turkiye	16	16	41
Russia	11	11	34
Ireland	21	17	34
Poland	15	9	31
Korea, Rep.	11	11	38
South Africa	14	14	42
Great Britain	13	12	37
Finland	6	5	45
USA	15	13	42
Croatia	15	10	31
Indonesia	4	4	49
Austria	10	9	39
Guatemala	9	9	39
USA	12	12	38
Croatia	12	12	29
France	17	16	38
Japan	9	7	50
Finland	4	4	20
Colombia	11	10	39

Spain	14	11	22
Armenia	12	11	26
Solomon Islands	16	14	35
Sweden	12	12	28
Romania	10	8	37
Thailand	5	6	29
Slovenia	18	16	30
Brazil	6	6	25
New Zealand	11	11	31
Japan	2	2	40
Romania	13	13	34
Bosnia and Herzegov	6	3	42
Austria	15	13	38
South Africa	14	14	29
Slovakia	11	5	44
Finland	9	7	39
Ukraine	20	17	37
New Zealand	8	7	48
Malaysia	7	8	31
Tunisia	12	9	34
Greece	12	10	21
USA	6	5	28
Israel	18	15	31
Bulgaria	7	6	38
Argentina	9	9	30
Turkiye	11	11	28
South Africa	8	9	25
South Africa	7	7	33
Germany	5	4	42
Brazil	10	8	25
Russia	4	4	30
Japan	13	10	28
New Zealand	9	7	37
Serbia	14	5	41
Egypt	12	8	39
USA	5	5	35
Great Britain	12	12	35
Japan	10	9	34
Brazil	10	10	35
Israel	19	17	29
Germany	14	13	25
Russia	4	4	28
Hungary	14	9	43
Great Britain	10	8	40
Kazakhstan	10	7	34
Sweden	7	6	40
Italy	12	8	30
Slovenia	14	10	32
Australia	4	3	42
USA	9	8	39

Poland	19	16	37
Turkiye	13	12	25
Serbia	14	9	39
Russia	10	10	34
Russia	16	16	29
Bulgaria	11	6	39
Sweden	10	7	36
China, P.R.	14	13	26
Belgium	7	7	36
Chinese Taipei	10	9	34
Portugal	6	6	14
Egypt	12	11	29
Argentina	7	7	24
Netherlands	12	11	10
India	9	9	42
Argentina	7	7	34
Chinese Taipei	11	11	26
Russia	9	9	24
Russia	4	1	44
Thailand	9	9	27
Germany	7	7	40
China, P.R.	11	11	22
USA	6	6	30
USA	16	14	29
Egypt	12	12	28
France	8	8	26
Japan	7	7	40
Egypt	12	9	36
Korea, Rep.	6	6	31
Austria	9	6	36
Romania	7	4	36
USA	3	3	41
Algeria	11	9	34
Spain	16	13	35
Israel	14	9	29
Serbia	21	15	31
Ukraine	11	11	37
Greece	11	9	37
Colombia	5	5	41
Tunisia	9	8	32
Montenegro	11	8	32
India	16	15	34
Canada	9	8	39
Finland	5	3	35
Serbia	7	3	36
Serbia	18	16	21
Venezuela	3	3	32
India	9	8	26
Costa Rica	22	22	27
USA	3	0	41

USA	7	3	41
Tunisia	15	8	37
Germany	9	8	40
Russia	7	7	27
USA	14	9	22
Spain	11	10	28
Turkiye	23	18	25
Spain	9	5	30
Poland	18	16	27
Armenia	6	6	29
Great Britain	3	1	35
Togo	7	6	36
USA	11	6	24
Great Britain	8	8	21
Belarus	5	4	39
France	13	11	34
Belarus	14	9	26
Russia	23	14	30
Tunisia	10	7	20
Germany	6	5	39
USA	14	9	32
Sweden	7	6	33
Japan	8	7	23
China, P.R.	12	8	28
Cameroon	7	7	20
Kazakhstan	16	12	25
France	10	10	29
Ukraine	12	11	12
Italy	10	5	34
Singapore	3	3	29
France	8	8	26
USA	9	8	25
Morocco	5	1	35
Venezuela	7	7	35
USA	2	2	33
Slovakia	16	7	33
Italy	8	7	31
Poland	10	8	25
Bulgaria	3	2	35
Great Britain	4	4	11
Czechia	5	2	36
Canada	7	7	26
Turkiye	25	20	18
Bolivia	5	3	33
Korea, Rep.	3	3	37
Hungary	13	12	17
India	9	9	30
USA	6	4	28
Turkiye	5	5	23
Finland	14	13	19

Slovakia	7	5	29
Samoa	5	5	29
France	11	11	27
USA	10	7	24
Korea, Rep.	6	6	26
Canada	7	6	29
Latvia	18	16	32
Australia	5	5	28
Zimbabwe	9	8	32
Sweden	10	10	12
Russia	20	12	22
Korea, Rep.	6	6	31
Peru	6	6	27
Serbia	22	13	32
Russia	19	17	22
Poland	3	3	15
China, P.R.	6	6	32
South Africa	10	10	13
Romania	9	5	34
Peru	11	11	30
Ukraine	8	6	22
Mexico	15	14	23
Austria	19	13	17
USA	3	3	36
Russia	13	7	30
Italy	4	3	27
Russia	11	10	25
Guatemala	14	14	24
Netherlands	10	10	16
Korea, Rep.	7	7	22
Venezuela	3	3	29
USA	4	3	28
Slovakia	4	4	30
France	18	12	31
Germany	19	11	17
Korea, Rep.	8	8	31
France	8	6	27
Guatemala	9	9	19
Romania	14	6	34
Canada	8	8	21
Japan	15	14	19
Russia	11	9	27
Canada	10	10	30
USA	8	7	20
Romania	14	9	7
Great Britain	3	0	35
USA	2	1	30
USA	7	1	35
Singapore	4	4	32
USA	13	7	25

Japan	1	1	0
Egypt	15	8	31
Germany	6	5	28
Belarus	9	5	28
Bulgaria	13	8	25
Indonesia	12	12	27
Hungary	4	3	19
China, P.R.	12	10	30
Czechia	3	3	20
New Zealand	3	3	33
Slovenia	13	11	22
China, P.R.	9	7	22
Tunisia	18	12	27
Bulgaria	20	19	12
Morocco	7	7	27
Nepal	3	2	32
France	5	3	32
USA	4	3	32
Slovakia	14	12	25
Germany	4	3	20
Czechia	9	8	24
India	11	11	21
Estonia	16	13	13
Brazil	6	6	30
Germany	9	6	27
USA	6	5	25
Italy	14	11	32
Australia	8	8	24
Russia	14	13	24
China, P.R.	12	11	23
Uzbekistan	16	16	19
Spain	4	3	25
USA	4	4	23
Russia	10	5	30
Slovakia	14	7	26
Venezuela	7	6	20
Argentina	17	15	20
Uzbekistan	14	7	27
Russia	7	6	33
Brazil	13	10	27
Uzbekistan	9	9	20
Mexico	10	9	17
USA	2	3	18
Switzerland	9	6	28
Belgium	13	13	16
Spain	7	6	12
Kosovo	8	4	28
Hong Kong, China	8	7	23
Korea, Rep.	6	6	27
USA	12	11	19

Brazil	2	2	10
Switzerland	5	5	27
South Africa	8	8	22
USA	8	5	29
Mexico	21	19	18
El Salvador	13	13	24
New Zealand	14	14	18
India	7	6	28
Hong Kong, China	14	13	20
Hong Kong, China	12	12	18
Malaysia	4	4	32
Indonesia	2	0	32
France	16	10	20
Chinese Taipei	5	5	19
Spain	17	15	22
China, P.R.	12	10	31
Romania	9	5	20
Singapore	4	4	16
USA	7	6	26
Netherlands	8	8	10
Colombia	7	7	16
Egypt	10	8	24
Malaysia	14	14	24
Zimbabwe	15	14	14
Korea, Rep.	5	4	29
France	6	6	25
Spain	6	0	31
Lithuania	14	14	24
China, P.R.	14	12	17
France	14	14	28
Russia	5	3	28
Tunisia	17	15	17
Czechia	16	10	10
Spain	8	4	28
Chinese Taipei	10	8	24
Israel	13	10	10
Russia	4	4	16
China, P.R.	7	7	15
Belarus	7	6	22
Japan	7	6	21
Korea, Rep.	6	5	28
Great Britain	2	0	30
USA	1	1	30
Brazil	8	7	30
South Africa	6	6	27
China, P.R.	9	7	19
USA	6	5	17
USA	5	1	30
Korea, Rep.	3	3	30
Mali	10	9	26

Bulgaria	15	12	17
Austria	15	11	15
Morocco	5	3	23
USA	11	10	18
USA	4	4	5
China, P.R.	8	6	27
Canada	8	7	12
Turkiye	14	12	18
Australia	11	8	12
Japan	7	7	24
Pakistan	6	6	23
China, P.R.	11	9	15
Mexico	11	10	27
Russia	6	6	20
Sweden	11	8	24
USA	10	10	18
Venezuela	5	5	23
Israel	8	8	18
Korea, Rep.	17	15	13
Belarus	10	6	12
USA	6	4	20
Australia	3	2	20
USA	7	5	26
Colombia	16	16	19
Egypt	20	17	18
Italy	6	4	12
Singapore	13	13	22
USA	6	5	26
Estonia	3	3	17
Barbados	8	8	15
France	9	9	25
Italy	5	3	19
Egypt	10	9	18
Egypt	10	9	12
Malaysia	9	9	22
USA	13	9	19
Serbia	13	7	19
Croatia	4	4	17
Thailand	13	12	19
Great Britain	10	9	12
South Africa	9	8	25
Venezuela	5	4	20
Mexico	10	10	17
Bulgaria	8	7	24
South Africa	6	5	19
Thailand	8	5	21
Ukraine	13	8	7
USA	8	6	24
Czechia	11	9	12
Romania	14	13	20

Canada	17	12	14
Great Britain	17	15	16
Russia	20	14	26
Sweden	11	11	21
Chile	4	3	20
USA	14	9	16
Korea, Rep.	9	6	25
El Salvador	8	8	24
China, P.R.	10	8	25
Poland	15	12	21
Japan	10	9	19
USA	7	5	17
USA	11	7	25
South Africa	7	7	11
Russia	4	4	15
Russia	18	11	22
Lithuania	16	12	17
USA	14	9	23
Colombia	10	9	24
Hong Kong, China	9	9	21
India	9	9	20
Great Britain	5	2	22
Israel	12	9	19
Egypt	13	9	10
Mexico	6	6	18
Kyrgyzstan	7	7	18
Romania	9	9	19
Serbia	21	19	18
Belgium	11	4	24
Ukraine	17	10	25
Russia	10	9	15
Latvia	17	9	21
Chile	9	9	17
France	3	3	25
Kazakhstan	17	14	2
India	7	6	19
Turkiye	19	12	16
Estonia	2	2	20
Russia	2	2	15
Hungary	4	3	10
Aruba	5	5	21
Great Britain	7	6	22
Switzerland	8	7	17
USA	2	2	10
Uruguay	6	5	21
Singapore	4	3	20
Venezuela	4	4	14
Argentina	7	7	9
Malaysia	13	13	19
Belarus	10	10	19

Switzerland	5	4	14
Morocco	16	13	11
Korea, Rep.	5	3	18
Lebanon	9	8	15
Thailand	11	8	4
Guatemala	17	13	18
Great Britain	5	5	19
Germany	19	15	11
France	16	13	22
Dominican Republic	12	11	16
Japan	10	10	21
USA	4	3	14
Romania	8	8	12
Israel	5	4	16
Argentina	7	4	18
Australia	11	10	12
Austria	18	13	12
Korea, Rep.	3	3	5
Belgium	17	15	5
Argentina	2	2	18
Spain	8	8	14
Estonia	13	12	14
Ukraine	12	9	15
China, P.R.	12	10	19
Bulgaria	7	3	15
Australia	5	5	15
Argentina	3	3	20
Great Britain	5	5	9
Denmark	17	13	0
Czechia	8	4	15
Switzerland	15	6	17
France	15	14	12
Italy	12	9	7
Russia	4	4	20
Ukraine	15	11	18
USA	7	7	11
Serbia	6	6	22
Japan	5	4	19
Netherlands	6	6	22
Poland	27	18	18
Finland	7	2	22
Colombia	10	9	14
Egypt	12	7	9
France	11	9	9
Thailand	13	8	21
Italy	9	5	20
Turkiye	7	5	12
Switzerland	7	4	12
Colombia	10	10	18
Greece	8	7	12

Zimbabwe	7	5	18
Czechia	18	13	9
Kazakhstan	15	9	20
Mexico	9	9	17
Indonesia	11	11	15
Switzerland	10	7	20
Belgium	11	8	19
New Zealand	6	6	19
Morocco	10	9	15
Italy	3	2	18
USA	7	6	16
Great Britain	7	6	14
Belarus	7	4	11
Brazil	10	8	9
Russia	5	5	17
Japan	4	3	7
Slovakia	14	10	19
Iran	3	2	18
China, P.R.	9	8	7
USA	3	1	20
Czechia	6	4	12
India	6	1	21
Spain	6	2	15
Tunisia	5	5	16
Germany	18	15	13
Russia	15	14	12
Brazil	13	10	9
India	7	7	20
India	2	2	14
Netherlands	10	9	12
Venezuela	6	6	12
USA	6	6	17
Bulgaria	7	4	20
Canada	10	9	17
Mexico	4	4	12
Russia	11	8	19
Australia	8	6	10
Romania	6	5	14
USA	4	3	5
Iran	5	5	17
Uzbekistan	12	12	7
Japan	5	5	19
USA	2	2	10
Ireland	7	7	19
Russia	20	17	13
France	13	6	19
Russia	2	1	18
Russia	8	6	15
Ukraine	13	8	13
Israel	8	5	12

Puerto Rico	7	7	9
Estonia	6	5	5
Dominican Republic	13	13	18
Guatemala	8	8	10
Russia	12	8	18
Nigeria	4	4	17
Ukraine	4	4	14
Russia	7	7	14
India	14	14	10
Mexico	13	12	8
Spain	12	12	0
Ukraine	6	4	20
Russia	9	4	20
Latvia	3	3	20
USA	3	1	19
Switzerland	14	12	16
Argentina	3	3	16
Italy	8	8	16
USA	4	4	14
Ukraine	7	6	14
Belgium	9	8	14
Belarus	5	2	13
Chinese Taipei	9	9	6
Russia	4	4	19
Mexico	10	9	10
Mexico	12	9	15
USA	5	3	19
USA	9	2	19
Russia	13	10	18
USA	5	5	15
Kazakhstan	22	16	18
Venezuela	4	4	15
Russia	9	8	15
Great Britain	5	4	7
South Africa	5	2	20
USA	2	2	15
USA	1	1	15
Germany	2	0	20
Germany	7	5	20
Japan	4	3	20
Colombia	6	6	15
Malaysia	5	4	7
France	5	5	14
Sweden	4	4	10
Egypt	10	6	19
Egypt	10	7	19
Israel	10	7	12
China, P.R.	3	3	5
Czechia	2	2	5
Indonesia	7	5	12

Greece	6	6	17
Romania	12	10	18
India	12	12	14
Chile	2	2	18
Russia	1	1	18
Korea, Rep.	5	5	18
Israel	14	7	11
Germany	6	5	17
Canada	3	3	12
Tunisia	12	9	16
Morocco	7	7	16
Australia	6	3	12
Thailand	7	7	12
Brazil	11	9	10
Russia	12	10	15
Czechia	4	4	10
Canada	11	11	14
Russia	13	9	15
Russia	5	4	15
China, P.R.	4	3	14
India	3	2	9
Slovakia	6	4	17
Great Britain	16	12	15
Greece	9	8	14
Great Britain	3	3	14
Israel	3	3	11
Denmark	1	1	0
Malaysia	13	13	16
USA	3	1	15
Kenya	9	8	17
Sweden	7	6	10
Peru	4	4	17
Russia	10	6	16
Ukraine	15	8	14
Serbia	15	13	5
Ireland	5	4	15
Barbados	4	4	12
Denmark	9	6	11
Thailand	2	3	15
Czechia	11	7	15
Poland	10	7	14
USA	2	1	18
USA	2	0	18
Netherlands	4	2	18
Great Britain	1	1	18
Russia	4	3	14
Sweden	5	5	12
Bulgaria	7	6	12
Croatia	10	7	14
Russia	4	4	5

Ecuador	2	2	18
Canada	2	1	18
Turkiye	5	1	18
Netherlands	1	1	18
India	3	3	18
Estonia	11	8	15
Canada	10	9	11
Switzerland	10	9	10
Belgium	12	9	6
USA	4	3	0
Greece	7	5	16
Egypt	13	8	11
Australia	7	6	9
Greece	12	12	14
France	12	10	14
Singapore	10	9	14
Poland	12	8	11
Russia	4	4	9
Kazakhstan	6	4	4
Italy	8	6	15
Serbia	17	11	16
USA	8	8	7
South Africa	8	8	16
Hong Kong, China	8	8	13
Estonia	12	8	6
Mexico	5	5	14
Japan	5	4	10
Turkiye	13	11	11
Netherlands	2	1	11
Bolivia	4	4	11
China, P.R.	10	6	11
Australia	11	11	0
Uzbekistan	7	6	17
Romania	15	9	14
USA	13	10	13
Slovakia	12	7	7
Russia	11	11	14
Austria	14	12	10
USA	10	8	15
Canada	6	5	15
France	6	3	16
Chile	8	7	15
Thailand	9	6	13
Cote D'Ivoire	6	6	11
Turkiye	17	10	11
Poland	5	5	5
Namibia	12	11	5
Spain	10	7	16
Bolivia	5	4	12
Greece	9	8	11

Croatia	3	2	9
Italy	15	10	9
Dominican Republic	7	7	2
USA	5	3	15
Peru	5	5	14
Ecuador	6	6	12
Ecuador	4	4	14
Egypt	6	3	14
Turkiye	17	8	11
Germany	6	4	10
Portugal	16	11	10
Puerto Rico	6	5	12
Romania	10	5	12
Brazil	3	3	5
USA	4	1	5
USA	2	1	5
Korea, Rep.	9	9	14
Great Britain	7	6	10
Switzerland	9	5	0
USA	2	2	0
Indonesia	6	6	12
Poland	18	8	12
Belgium	11	8	12
Belarus	6	5	12
Slovakia	10	6	10
India	10	10	12
El Salvador	11	11	8
Great Britain	8	8	2
Kazakhstan	8	4	12
Mexico	5	4	15
Austria	12	7	15
Korea, Rep.	6	5	15
France	7	5	11
Belarus	9	6	11
India	9	8	8
USA	13	11	6
Czechia	10	6	10
Spain	14	10	10
Denmark	8	3	14
Tunisia	11	6	12
Greece	9	4	12
Latvia	19	11	12
Russia	7	6	12
Romania	11	8	10
France	12	10	14
Great Britain	2	2	14
Russia	6	6	14
Peru	3	3	14
Finland	8	6	14
India	12	12	11

Denmark	15	15	11
Morocco	6	4	10
Zimbabwe	3	3	10
USA	5	4	12
Colombia	8	7	9
Great Britain	8	7	12
Italy	9	5	12
China, P.R.	7	5	12
Australia	9	6	10
Netherlands	3	3	5
Ukraine	10	7	13
Russia	11	7	6
Korea, Rep.	9	7	5
Italy	2	1	15
Hungary	12	3	15
Israel	5	2	15
Germany	2	1	15
Romania	8	5	12
Colombia	9	9	12
Sweden	5	0	15
Greece	2	1	15
Ghana	8	7	12
Russia	10	3	10
Czechia	3	3	10
Romania	9	8	5
Iran	3	1	15
USA	4	4	15
China, P.R.	10	8	12
Indonesia	5	5	12
Korea, Rep.	9	8	12
USA	6	6	9
Austria	4	2	14
Turkiye	14	5	14
Great Britain	6	6	10
Slovakia	7	2	10
Switzerland	4	4	9
Slovenia	10	8	7
Kazakhstan	5	5	14
Canada	7	2	14
Thailand	7	5	7
Russia	7	6	7
Korea, Rep.	8	8	2
Portugal	11	11	5
Canada	4	3	12
Kazakhstan	7	4	12
USA	8	5	12
Cyprus	9	8	12
Thailand	4	2	13
Poland	13	13	9
Russia	9	7	9

Bolivia	5	5	9
Japan	4	3	9
Australia	9	8	9
Egypt	7	7	2
Azerbaijan	12	11	0
USA	5	5	10
Moldova	11	3	12
Russia	16	9	11
France	15	11	10
Germany	17	9	12
India	7	6	11
Turkiye	8	6	10
India	15	13	9
India	10	8	9
USA	8	7	4
Ireland	11	11	4
Russia	8	7	11
Argentina	3	3	10
Australia	5	3	10
Tunisia	10	9	10
Ukraine	10	8	7
Russia	11	10	7
Czechia	6	3	5
Kazakhstan	4	2	14
Finland	7	1	14
Malaysia	15	15	11
Czechia	7	7	5
Australia	7	6	5
Japan	10	8	4
USA	4	4	12
Brazil	8	4	12
Ukraine	5	3	10
USA	2	1	10
Poland	7	3	12
Mexico	3	1	10
Philippines	3	3	12
Serbia	8	4	12
Germany	5	4	12
Colombia	10	9	9
Togo	7	7	9
Chile	5	5	9
Ecuador	6	6	9
Colombia	5	5	9
India	3	2	9
Argentina	10	9	7
Mexico	5	5	10
Japan	3	3	10
Italy	9	5	12
Finland	4	3	10
Russia	6	4	10

Israel	14	11	7
Great Britain	3	2	5
Japan	8	7	12
Canada	6	6	8
Morocco	6	4	7
USA	2	2	0
China, P.R.	4	4	10
Estonia	5	4	10
Thailand	4	4	11
Belgium	8	4	10
Nigeria	2	2	10
Romania	9	6	7
Italy	7	6	7
Serbia	9	8	2
Denmark	8	7	2
Spain	5	4	0
Mexico	10	10	9
India	8	7	10
Bosnia and Herzegov	14	7	7
Argentina	2	2	5
Paraguay	3	3	5
Mexico	5	5	5
South Africa	5	5	12
Tunisia	8	3	12
Peru	3	3	12
Serbia	8	3	7
Australia	8	8	9
USA	9	9	8
Indonesia	9	9	7
Mexico	9	9	10
Canada	8	7	10
Israel	7	7	10
Mexico	7	5	10
New Zealand	7	7	11
Mexico	8	8	10
Spain	5	4	11
Serbia	7	5	11
India	4	4	11
Colombia	7	7	11
USA	5	5	11
Estonia	7	4	10
USA	5	4	10
USA	6	6	9
Bulgaria	11	5	7
Brazil	10	9	5
Mexico	2	2	10
Ecuador	2	2	9
Mexico	7	6	9
Thailand	13	10	7
Australia	9	9	5

New Zealand	17	16	4
Egypt	11	8	4
South Africa	4	4	2
Tunisia	9	8	2
Russia	15	7	12
Spain	6	1	12
Portugal	10	5	12
Mexico	11	8	7
Estonia	14	8	12
Italy	9	7	12
Denmark	11	6	12
North Macedonia	3	3	12
Australia	5	5	12
Australia	9	8	12
Japan	9	7	12
Slovenia	12	9	7
USA	7	7	9
Sweden	11	7	6
Switzerland	7	4	5
Brazil	9	7	4
Poland	10	6	2
Germany	3	2	10
Netherlands	3	2	10
USA	3	2	10
China, P.R.	2	2	10
USA	2	2	10
USA	2	2	10
Morocco	7	6	7
Russia	14	10	7
Serbia	11	3	11
Colombia	5	4	11
Russia	6	5	11
USA	4	4	11
Ecuador	8	8	8
Japan	8	6	8
Germany	7	5	4
Dominican Republic	5	5	2
Egypt	10	11	0
Austria	8	4	10
Canada	12	7	9
Norway	10	10	10
Philippines	4	4	10
Latvia	12	4	10
Australia	8	7	7
India	9	8	7
Pakistan	2	1	10
Pakistan	2	2	10
Hungary	11	4	10
Botswana	11	8	10
Colombia	3	3	10

Madagascar	4	4	7
Singapore	7	6	7
Kazakhstan	17	14	6
Moldova	8	8	6
Hungary	9	9	5
Belgium	3	3	2
Mexico	5	4	7
Romania	14	10	9
Australia	3	3	7
Australia	6	6	7
Canada	5	5	9
India	6	4	9
Korea, Rep.	7	7	9
France	14	13	6
Serbia	9	9	2
Sweden	3	3	0
Costa Rica	1	1	0
USA	6	4	0
Costa Rica	1	1	0
Nepal	5	5	7
Austria	11	9	5
Spain	2	2	11
India	10	7	11
Russia	10	9	8
Paraguay	5	5	7
USA	6	6	7
Croatia	18	16	6
Austria	6	4	5
Egypt	7	5	4
Russia	2	2	10
Mexico	8	7	10
Sri Lanka	5	3	10
Indonesia	6	6	9
USA	7	4	10
Bolivia	4	3	10
Nepal	4	4	7
New Zealand	7	6	7
Chile	9	7	6
Morocco	10	7	4
Australia	16	7	4
Mexico	9	7	2
Ukraine	13	9	2
Netherlands	16	12	0
Italy	6	4	9
Chile	4	4	7
Sweden	13	11	5
China, P.R.	2	2	9
Singapore	3	3	9
USA	2	1	9
Peru	6	5	9

Israel	6	5	9
Nigeria	2	2	9
Australia	6	5	2
France	6	4	7
USA	12	11	7
Benin	4	4	7
Italy	3	3	7
Ukraine	4	4	7
USA	6	4	7
Poland	4	4	7
Romania	11	8	7
Romania	6	5	7
Bulgaria	9	4	7
South Africa	9	9	7
Israel	18	13	6
Brazil	11	8	5
France	17	13	5
Israel	5	4	4
Dominican Republic	8	8	2
New Zealand	5	5	2
Australia	6	5	0
Russia	2	2	10
Brazil	2	1	10
Turkiye	2	1	10
Australia	2	2	10
USA	4	3	10
Uzbekistan	4	3	10
Turkiye	5	3	10
Germany	3	0	10
USA	10	7	5
Brazil	4	4	5
Switzerland	2	1	10
Croatia	9	2	10
USA	2	0	10
Croatia	1	1	10
Russia	1	0	10
Spain	5	3	10
Chile	3	2	10
Croatia	9	2	10
USA	6	3	10
Croatia	1	1	10
Spain	5	3	10
Sweden	7	2	10
USA	3	2	10
Norway	4	4	10
Slovenia	7	5	10
Russia	1	0	10
USA	2	0	10
USA	2	0	10
Turkiye	5	4	10

Canada	4	4	10
Spain	5	1	10
Montenegro	8	2	10
Sweden	2	1	10
Israel	4	4	7
USA	7	7	5
China, P.R.	5	2	10
South Africa	7	6	10
Pakistan	2	2	10
USA	6	4	10
Poland	11	5	7
Russia	10	9	7
Australia	5	4	7
Laos	9	7	6
Bulgaria	11	6	6
France	6	5	6
Puerto Rico	5	4	5
USA	5	3	5
Great Britain	9	6	4
Poland	6	2	0
Poland	8	6	0
India	9	6	9
Luxembourg	10	9	9
Tunisia	6	4	9
USA	3	3	9
South Africa	9	9	9
USA	1	1	9
Norway	4	1	9
Hungary	6	4	9
New Zealand	8	8	9
Germany	1	1	9
New Zealand	7	7	9
Philippines	4	4	9
Great Britain	6	4	5
Costa Rica	7	7	4
Australia	9	7	2
Israel	10	7	2
Greece	5	5	7
Portugal	6	6	7
Japan	10	6	8
India	6	5	8
Canada	6	4	7
Israel	8	6	4
Poland	7	5	4
USA	12	12	4
Belgium	6	5	2
Canada	5	5	0
Curacao	6	6	7
Japan	8	7	7
Canada	8	4	5

Russia	9	7	7
France	9	8	7
Sri Lanka	3	3	7
Iran	3	3	7
China, P.R.	4	2	7
Colombia	4	4	7
Colombia	5	5	7
Bulgaria	14	5	6
South Africa	6	5	0
Armenia	4	2	9
Kenya	9	7	9
Venezuela	4	4	9
Russia	2	0	9
Croatia	12	2	9
Colombia	3	3	9
Colombia	4	3	9
Lebanon	4	4	9
France	4	1	9
USA	2	1	9
Germany	8	2	9
USA	1	1	9
France	1	0	9
Serbia	8	5	9
Madagascar	5	4	9
Italy	3	1	9
Greece	10	7	9
Canada	5	3	9
Singapore	2	1	9
Korea, Rep.	7	1	9
Turkiye	8	5	6
Lebanon	5	5	6
South Africa	4	4	6
India	9	6	6
Korea, Rep.	10	9	6
Russia	8	7	6
Israel	9	5	5
Japan	7	4	2
Czechia	10	2	7
Russia	2	1	7
Russia	4	4	7
New Zealand	6	5	7
USA	5	2	7
Portugal	4	4	5
Czechia	10	4	5
El Salvador	12	11	8
Thailand	7	5	5
Hungary	7	6	4
Tunisia	10	7	4
Zimbabwe	5	5	4
Great Britain	4	4	4

India	10	7	4
Colombia	5	4	4
Portugal	5	4	2
South Africa	3	2	0
Argentina	4	4	7
Mexico	4	4	7
Spain	13	10	7
Turkiye	7	5	7
Spain	4	2	5
China, P.R.	4	4	5
USA	5	5	5
Slovenia	10	6	5
Japan	4	3	7
Canada	3	2	7
Peru	7	5	7
USA	3	2	7
Russia	13	7	7
Romania	11	7	7
Great Britain	4	3	7
Denmark	4	3	7
Japan	10	3	7
Zimbabwe	12	12	7
Argentina	4	3	7
Australia	8	8	6
Italy	4	2	5
Japan	12	8	4
USA	6	6	4
Portugal	15	12	0
Czechia	2	2	0
Tunisia	4	4	5
Germany	12	8	5
Italy	3	3	5
Germany	7	3	5
Brazil	2	2	5
USA	5	5	6
Spain	5	5	5
Mexico	6	4	5
Russia	17	13	4
Spain	3	2	2
Romania	12	9	2
Iran	1	1	2
China, P.R.	10	8	0
Greece	3	3	5
Pakistan	2	2	5
Nigeria	4	3	5
Netherlands	12	9	4
Russia	6	4	4
Sweden	12	7	4
Sri Lanka	6	4	4
Uzbekistan	5	4	2

Turkiye	11	8	2
USA	7	7	0
Austria	4	3	0
Switzerland	8	4	0
Greece	4	3	7
Poland	9	4	7
USA	6	4	7
Romania	9	1	7
Hong Kong, China	5	5	7
Russia	4	2	7
Slovenia	10	9	7
Russia	7	2	7
USA	2	2	7
South Africa	5	4	7
Peru	7	6	7
Finland	3	2	7
USA	3	2	7
Peru	3	2	7
South Africa	11	9	4
China, P.R.	10	8	2
Russia	6	5	5
Norway	6	6	5
Bosnia and Herzegov	2	2	5
Canada	4	4	5
Mexico	4	4	5
China, P.R.	8	6	5
Philippines	10	6	6
Australia	6	6	6
India	5	3	6
India	7	5	6
Costa Rica	8	6	5
Belgium	8	7	5
USA	2	2	5
China, P.R.	9	7	5
Bolivia	4	4	4
Korea, Rep.	7	5	2
Slovenia	10	5	5
Slovenia	10	5	5
Turkiye	10	3	5
Egypt	4	5	5
Venezuela	3	3	5
USA	5	4	5
Bulgaria	6	3	5
Colombia	14	10	4
USA	2	2	4
Bulgaria	7	6	4
Tunisia	9	4	4
USA	5	5	4
Russia	12	10	2
Kazakhstan	8	5	0

Spain	7	3	0
Colombia	4	4	7
Montenegro	2	0	7
Bosnia and Herzegov	2	2	7
France	6	3	7
USA	3	3	7
Latvia	8	4	7
Bolivia	2	2	7
Germany	4	1	7
Turkiye	11	2	7
Jamaica	2	2	7
Ukraine	7	5	7
Spain	8	3	7
Norway	12	5	7
New Zealand	5	5	7
Philippines	4	3	7
Russia	4	0	7
Mauritius	2	2	7
Australia	7	5	7
Russia	4	3	7
France	4	3	7
Colombia	2	1	7
India	6	3	7
Latvia	5	2	7
Israel	3	3	7
Thailand	3	1	7
Canada	4	2	7
Cote D'Ivoire	2	2	7
Spain	5	1	7
Finland	2	0	7
Singapore	3	3	7
Slovakia	3	3	4
USA	7	6	4
Korea, Rep.	8	8	4
Hungary	9	6	4
Argentina	10	9	4
Japan	9	8	4
Turkiye	5	4	2
Netherlands	12	9	2
Togo	11	11	2
India	4	4	2
France	4	3	2
Germany	1	1	5
Great Britain	2	2	5
Germany	10	3	5
Spain	2	1	5
Ireland	4	3	5
Croatia	9	5	5
Bulgaria	4	3	5
Aruba	1	1	5

Kyrgyzstan	5	4	5
Czechia	5	5	5
Germany	7	5	5
Czechia	3	1	5
Cyprus	4	3	5
Serbia	8	2	5
Germany	5	3	5
India	6	3	6
Russia	9	3	6
Uzbekistan	6	5	6
Ukraine	5	5	6
Philippines	10	8	6
Korea, Rep.	5	5	6
USA	5	2	5
Canada	7	3	5
Honduras	10	10	2
Hong Kong, China	4	4	2
Netherlands	7	5	0
Australia	2	1	0
Uzbekistan	4	3	0
Germany	7	2	0
Ukraine	4	2	5
Czechia	9	5	5
France	3	1	5
Spain	2	2	5
Korea, Rep.	9	6	5
Zimbabwe	7	7	5
Jordan	3	3	5
Australia	4	4	5
Korea, Rep.	1	1	5
Pakistan	1	1	5
North Macedonia	4	4	5
Russia	3	2	5
Japan	6	5	5
Brazil	3	3	5
Brazil	2	2	5
Puerto Rico	3	1	5
France	8	7	4
Greece	7	4	4
Cyprus	3	3	4
Canada	4	4	4
Colombia	6	6	4
India	14	12	4
Russia	12	11	4
Venezuela	8	8	4
USA	8	4	4
Mexico	2	2	4
India	7	6	4
Belgium	10	6	4
France	12	9	4

Russia	4	2	4
Chinese Taipei	9	8	4
Mauritius	8	8	4
Thailand	5	5	4
Russia	14	6	4
Thailand	7	6	4
Chinese Taipei	10	9	4
Belgium	13	10	4
India	3	3	4
Ukraine	7	5	4
Great Britain	14	10	4
Germany	8	6	2
Egypt	10	10	2
Algeria	3	3	0
Germany	2	2	0
Korea, Rep.	8	2	0
Portugal	1	1	0
Russia	6	1	6
Russia	10	10	2
Zimbabwe	8	8	2
USA	9	7	2
France	4	2	2
Egypt	9	7	0
USA	5	5	0
Kazakhstan	6	4	0
Colombia	2	2	5
USA	5	4	5
USA	7	4	5
Bulgaria	4	2	5
South Africa	9	7	5
Mexico	7	7	5
Georgia	2	2	5
Switzerland	9	6	5
Turkiye	13	8	5
Spain	7	3	5
Brazil	7	7	5
Chinese Taipei	4	4	5
China, P.R.	7	4	5
Russia	4	2	5
Singapore	2	2	5
Canada	8	4	5
India	8	5	5
USA	1	1	5
India	1	1	5
Spain	2	1	5
New Zealand	4	4	5
Serbia	6	5	5
USA	3	3	5
Argentina	4	3	5
Latvia	6	5	5

USA	2	2	5
Canada	7	5	5
Japan	12	6	5
Belarus	2	1	5
Germany	7	5	5
Venezuela	3	3	5
Sweden	11	7	5
Argentina	8	3	4
Sweden	7	4	4
Guatemala	5	5	4
USA	8	4	4
Morocco	8	6	4
Croatia	6	4	4
Finland	5	5	2
Argentina	10	8	2
Germany	7	5	2
New Zealand	9	7	4
Antigua & Barbuda	7	6	4
Belgium	9	8	4
Iran	3	2	4
Hungary	9	5	4
New Zealand	5	5	4
Japan	7	5	4
Hong Kong, China	3	2	4
Singapore	4	4	4
USA	6	4	4
Japan	6	4	4
Israel	4	4	4
Finland	7	5	4
China, P.R.	15	11	4
Thailand	7	6	4
Indonesia	2	1	4
Thailand	4	4	4
Lebanon	4	4	4
Mexico	5	4	4
India	3	2	4
USA	4	4	2
New Zealand	3	3	2
Italy	4	2	2
Israel	6	3	2
Ukraine	2	1	2
Bulgaria	4	2	2
Nigeria	2	2	2
USA	2	1	2
Japan	7	6	2
Great Britain	3	2	2
Argentina	2	2	2
Italy	7	4	2
Korea, Rep.	9	9	2
Bulgaria	4	2	2

Japan	3	2	0
Netherlands	6	2	0
Czechia	7	6	0
USA	14	11	0
Brazil	5	2	0
Czechia	4	4	0
Russia	6	2	0
Romania	10	7	0
Romania	4	3	0
China, P.R.	9	7	5
Germany	4	3	5
Portugal	6	4	5
Colombia	1	1	5
Mexico	7	4	5
Mexico	5	5	5
Mexico	2	2	5
USA	2	1	5
India	4	3	5
India	2	2	5
USA	1	1	5
Italy	3	3	5
Bosnia and Herzegov	3	2	5
Denmark	3	1	5
Benin	1	1	5
Norway	3	1	5
Israel	15	1	5
Estonia	3	3	5
Montenegro	1	1	5
Canada	3	3	5
Brazil	3	1	5
Czechia	8	4	5
Tunisia	2	2	5
USA	2	0	5
Benin	1	1	5
Serbia	3	3	5
Canada	1	0	5
USA	1	0	5
Netherlands	8	1	5
Sweden	6	3	5
Turkiye	2	1	5
Colombia	2	2	5
Brazil	11	7	5
Brazil	5	2	5
Canada	3	1	5
Ukraine	3	1	5
Russia	7	3	5
South Africa	9	7	5
India	2	0	5
Netherlands	7	5	5
Bosnia and Herzegov	7	5	5

Greece	1	1	5
Sweden	2	1	5
Dominican Republic	8	8	5
Ireland	5	4	5
India	4	0	5
South Africa	2	1	5
Greece	4	4	5
Estonia	5	2	5
Israel	5	2	5
Serbia	12	2	5
Russia	4	1	5
USA	1	1	5
Brazil	11	3	5
Russia	2	2	5
India	1	0	5
Estonia	3	1	5
Bolivia	4	3	5
Russia	1	0	5
China, P.R.	5	4	5
USA	1	0	5
India	9	7	5
Russia	2	2	5
Spain	2	1	5
India	6	4	5
Ukraine	1	0	5
India	1	0	5
Bosnia and Herzegov	6	2	5
Japan	1	1	5
Paraguay	3	1	5
France	1	1	5
France	1	1	5
Trinidad & Tobago	4	4	5
USA	1	0	5
Japan	4	4	5
Estonia	3	3	5
Romania	1	0	5
Paraguay	1	1	5
Finland	3	0	5
Israel	8	4	5
USA	1	0	5
India	6	4	5
Romania	3	2	5
Finland	3	2	5
Japan	2	1	5
Venezuela	1	1	5
Paraguay	3	1	5
France	1	1	5
Australia	2	2	5
Japan	1	1	5
Paraguay	1	1	5

Bolivia	1	1	5
Estonia	3	3	5
USA	14	7	5
USA	1	0	5
Romania	1	0	5
Ukraine	1	0	5
Italy	5	1	5
Russia	1	1	5
Bosnia and Herzegov	6	2	5
Finland	5	3	2
USA	2	2	2
Switzerland	10	7	2
Greece	5	5	2
Brazil	3	3	2
Uruguay	7	7	2
Russia	10	11	2
Italy	7	4	0
Greece	6	6	0
Germany	6	1	0
USA	2	2	0
USA	1	1	0
Italy	6	4	0
Germany	5	1	0
Canada	2	2	4
Zimbabwe	5	1	4
Russia	9	7	4
Zimbabwe	7	5	4
Peru	2	2	4
Thailand	3	3	4
Germany	10	5	4
Argentina	4	3	4
Hungary	10	3	4
USA	6	3	4
Australia	7	6	4
Lithuania	5	4	4
Italy	3	2	4
North Macedonia	6	5	4
Italy	9	4	4
Norway	7	6	4
Georgia	8	5	4
Singapore	5	3	4
New Zealand	2	2	4
Belgium	11	6	4
Bolivia	2	2	4
Great Britain	4	1	4
New Zealand	4	4	4
Barbados	3	3	4
Brazil	6	5	4
USA	5	3	4
Iran	4	4	4

Argentina	5	4	4
Austria	2	2	0
Japan	6	5	0
Barbados	5	5	0
Mexico	9	8	2
Morocco	5	4	2
Italy	4	2	2
El Salvador	9	8	2
North Macedonia	4	3	2
USA	3	2	2
Cyprus	9	9	2
Morocco	4	3	2
Mexico	3	3	2
India	2	2	0
Uzbekistan	5	4	0
Czechia	2	3	0
Costa Rica	7	7	0
Canada	6	4	0
Israel	5	4	2
Poland	13	8	2
Korea, Rep.	6	4	2
France	12	7	2
Brazil	8	7	2
China, P.R.	10	8	2
Serbia	2	2	2
USA	9	9	2
Canada	12	7	2
Australia	4	2	2
Japan	5	4	2
Kenya	7	6	2
Brazil	9	8	2
South Africa	12	11	2
Romania	10	8	2
Namibia	6	6	2
Great Britain	6	5	2
Tunisia	14	7	0
Australia	2	0	4
Great Britain	3	3	4
USA	6	6	4
Greece	11	6	4
Sweden	10	4	4
Romania	7	6	4
Dominican Republic	8	6	4
Korea, Rep.	4	3	4
Australia	2	0	4
Azerbaijan	3	0	4
Italy	9	5	4
Bulgaria	6	3	4
Great Britain	8	6	4
Thailand	3	2	4

Australia	8	5	4
Madagascar	4	4	4
Hong Kong, China	10	0	4
Korea, Rep.	6	5	4
Uzbekistan	3	2	4
Thailand	7	6	4
Hungary	8	4	4
Argentina	2	2	4
Ukraine	7	2	4
Argentina	4	4	4
Spain	7	5	4
Thailand	2	2	4
Romania	16	13	0
France	2	2	0
Sweden	2	2	0
Austria	7	6	0
France	3	2	0
Russia	17	8	0
Great Britain	10	6	2
Egypt	2	2	2
Austria	9	6	2
USA	4	2	2
Benin	3	2	2
USA	5	1	2
Ireland	6	4	2
Korea, Rep.	9	9	2
Ireland	5	3	2
North Macedonia	2	2	2
Egypt	6	5	2
USA	1	1	0
Argentina	1	1	0
India	1	1	0
Portugal	2	1	0
USA	5	5	0
Canada	4	1	0
Germany	5	3	0
Israel	15	9	0
France	1	1	0
Romania	2	2	0
France	1	1	0
Australia	2	1	2
Saudi Arabia	4	5	2
Italy	3	1	2
Singapore	3	2	2
Jordan	2	2	2
Japan	8	4	2
USA	4	3	2
China, P.R.	2	2	2
Singapore	4	4	2
Japan	5	4	2

Turkiye	5	4	2
Kenya	8	6	2
Kyrgyzstan	5	4	2
Thailand	6	6	2
India	1	1	2
Spain	3	3	2
Egypt	7	5	2
Czechia	16	4	2
Serbia	5	3	2
South Africa	6	5	2
Russia	8	2	2
USA	1	1	2
New Zealand	4	4	2
Spain	3	3	2
New Zealand	2	2	2
USA	2	2	2
Singapore	6	5	2
Lithuania	3	2	2
Madagascar	2	2	2
Ukraine	9	5	2
Singapore	2	2	2
Zimbabwe	8	7	2
USA	5	4	2
Jordan	6	6	2
Israel	11	5	2
Peru	2	2	2
New Zealand	12	8	2
Hungary	2	1	2
Colombia	7	5	2
France	4	3	2
France	6	3	2
India	2	2	2
Italy	3	2	2
India	4	2	2
Argentina	2	2	2
Ukraine	11	5	2
USA	2	2	2
Russia	3	2	2
Italy	3	1	2
New Zealand	4	4	2
China, P.R.	2	2	2
Hungary	2	1	2
India	7	4	2
Singapore	3	2	2
USA	2	2	2
Russia	1	1	0
Morocco	4	1	0
Kosovo	1	1	0
Slovakia	1	1	0
Great Britain	2	2	0

Russia	8	6	0
USA	1	1	0
Russia	1	1	0
Japan	2	2	0
Venezuela	3	3	0
USA	1	1	0
Latvia	8	6	0
Uzbekistan	4	3	0
Netherlands	4	4	0
Finland	6	2	0
USA	1	1	0
USA	6	5	0
USA	1	1	0
Hungary	13	7	0
Bulgaria	6	3	0
North Macedonia	4	4	0
Switzerland	7	5	0
Russia	4	2	0
Uzbekistan	3	3	0
Ecuador	1	1	0
China, P.R.	9	8	0
Armenia	3	2	0
Egypt	5	2	0
Latvia	17	11	0
Paraguay	3	3	0
Great Britain	20	17	0
Nigeria	3	3	0
Russia	1	1	0
Thailand	2	1	0
Russia	4	4	0
Czechia	1	1	0
Malaysia	12	12	0
Ukraine	2	2	0
Czechia	2	1	0
Czechia	2	1	0
Cyprus	11	10	0
Poland	9	8	0
Croatia	1	1	2
Lithuania	8	5	2
France	3	1	2
Great Britain	1	1	2
Poland	5	1	2
Bulgaria	5	4	2
Great Britain	7	4	2
Great Britain	2	2	2
South Africa	6	1	2
Russia	1	1	2
Denmark	7	6	2
Netherlands	7	5	2
Colombia	4	3	2

Georgia	4	1	2
India	3	2	2
Italy	6	3	2
Belgium	4	2	2
Ukraine	7	3	2
Russia	9	6	2
Turkiye	5	3	2
Brazil	2	2	2
North Macedonia	5	5	2
Greece	7	6	2
Korea, Rep.	6	5	2
Canada	3	2	2
Nepal	2	2	2
Japan	5	4	2
China, P.R.	2	2	2
Austria	1	1	2
Russia	5	4	2
Denmark	9	5	2
Denmark	9	5	2
Canada	2	2	2
Russia	3	1	2
Belarus	5	2	2
Japan	1	1	2
Bolivia	2	2	2
Czechia	3	1	2
Belgium	11	5	2
Switzerland	11	8	2
USA	3	2	2
Hong Kong, China	3	2	2
Tunisia	9	6	2
Romania	6	1	2
Australia	1	1	2
India	3	2	2
Mauritius	2	2	2
Hong Kong, China	7	5	2
Guatemala	4	3	2
Ukraine	5	2	2
Hong Kong, China	7	5	2
Italy	6	3	2
New Zealand	1	1	2
Tunisia	7	5	2
Kazakhstan	6	3	2
Russia	3	1	2
China, P.R.	10	8	2
Great Britain	8	4	2
Ukraine	3	1	2
India	15	7	2
Canada	2	2	2
Japan	2	1	2
New Zealand	5	5	2

North Macedonia	5	5	2
India	6	2	2
Lithuania	10	5	2
Canada	4	4	2
Sri Lanka	3	2	2
Philippines	7	3	2
India	5	3	2
Thailand	1	1	2
Venezuela	3	3	2
Netherlands	15	8	2
Great Britain	5	5	2
Ukraine	9	3	2
USA	1	1	2
Serbia	9	3	2
Greece	4	2	2
Trinidad & Tobago	1	1	2
Nepal	2	2	2
Costa Rica	6	4	2
Turkiye	5	4	2
Brazil	3	3	2
Jamaica	6	6	2
Russia	3	1	2
Australia	1	1	2
Colombia	6	3	2
Argentina	2	2	2
Belgium	4	2	2
Colombia	2	2	2
Mauritius	2	2	2
Romania	6	1	2
Great Britain	7	4	2
Belgium	13	7	2
Croatia	1	1	2
Palestine	5	2	2
Canada	3	2	2
Indonesia	5	5	2
Japan	3	2	2
Netherlands	3	2	2
Denmark	7	6	2
Argentina	2	1	2
South Africa	6	1	2
Great Britain	1	1	2
Hong Kong, China	2	1	2
Bolivia	2	2	2
USA	3	2	2
Poland	5	1	2
USA	2	1	2
Russia	1	1	2
Australia	2	1	2
Ecuador	1	1	2
Mexico	8	6	2

Trinidad & Tobago	1	1	2
Guatemala	4	3	2
Zimbabwe	6	5	2
Indonesia	6	4	2
Peru	4	4	2
Japan	6	3	2
Japan	1	1	2
China, P.R.	2	2	2
Netherlands	7	5	2
Turkiye	5	3	2
Ethiopia	7	7	0
USA	6	6	0
Canada	3	3	0
Romania	11	6	0
Egypt	7	8	0
France	11	3	0
Ukraine	2	3	0
Trinidad & Tobago	5	5	0
Belgium	12	7	0
Romania	7	2	0
Mexico	3	3	0
Serbia	13	4	0
Israel	8	6	0
Russia	5	5	0
Finland	5	3	0
Germany	5	3	0
South Africa	5	5	0
Puerto Rico	8	7	0
Italy	8	4	0
Ukraine	8	3	0
China, P.R.	12	9	0
Slovenia	10	6	0
Denmark	6	5	0
Egypt	10	8	0
Paraguay	5	5	0
Australia	10	8	0
Moldova	7	3	0
Chinese Taipei	7	5	0
Australia	10	8	0
Poland	5	3	0
Mexico	7	6	0
Belgium	4	3	0
Hungary	3	3	0
Antigua & Barbuda	7	7	0
Argentina	7	5	0
France	4	3	0
Chinese Taipei	7	5	0
Hungary	7	5	0
Australia	3	2	2
South Africa	1	1	2

Bolivia	2	0	2
USA	1	1	2
Singapore	2	1	2
Zimbabwe	3	3	2
Japan	1	1	2
New Zealand	2	2	2
Singapore	2	1	2
Serbia	4	1	2
Serbia	7	2	2
Egypt	3	3	2
Egypt	2	0	2
USA	3	3	2
Australia	4	3	2
Malaysia	4	4	2
Poland	3	0	2
Bulgaria	1	1	2
Germany	2	0	2
Singapore	2	0	2
Great Britain	2	1	2
Guatemala	1	1	2
Kazakhstan	7	4	2
Lebanon	2	2	2
Thailand	9	3	2
Canada	2	1	2
Hong Kong, China	1	0	2
Japan	3	2	2
Germany	4	2	2
USA	6	6	2
USA	9	1	2
Italy	1	0	2
Russia	5	4	2
Finland	2	0	2
Bolivia	2	2	2
USA	2	2	2
Australia	2	2	2
China, P.R.	7	5	2
Japan	2	2	2
Chinese Taipei	4	4	2
Chinese Taipei	6	5	2
Brazil	3	3	2
Venezuela	1	1	2
Israel	12	4	2
India	3	1	2
India	1	0	2
India	6	2	2
Argentina	2	2	2
India	8	3	2
India	14	8	2
Costa Rica	7	5	2
Switzerland	4	1	2

Venezuela	3	2	2
Colombia	1	0	2
Brazil	6	3	2
Ireland	5	2	2
Ireland	2	1	2
Trinidad & Tobago	4	3	2
Ecuador	2	2	2
Peru	5	3	2
India	2	2	2
China, P.R.	4	3	2
Trinidad & Tobago	2	2	2
Italy	7	5	2
Romania	5	2	2
USA	3	1	2
Russia	8	5	2
India	7	1	2
Israel	8	3	2
Hong Kong, China	3	2	2
Israel	6	1	2
Italy	1	1	2
Belgium	1	1	2
Russia	1	0	2
Hungary	2	2	2
USA	1	1	2
India	2	2	2
Serbia	4	1	2
South Africa	8	7	2
India	2	2	2
Slovakia	11	4	2
Australia	3	3	2
Argentina	1	1	2
Colombia	2	0	2
Mexico	2	1	2
Zimbabwe	8	8	2
Greece	6	2	2
Curacao	2	2	2
Korea, Rep.	2	1	2
South Africa	5	3	2
USA	5	4	2
Vietnam	2	1	2
Japan	3	1	2
Indonesia	2	1	2
New Caledonia	2	2	2
China, P.R.	2	2	2
Indonesia	2	1	2
Italy	4	1	2
Canada	2	2	2
India	3	1	2
Antigua & Barbuda	1	1	2
Costa Rica	2	0	2

Tunisia	6	4	2
Costa Rica	5	4	2
Zimbabwe	3	3	2
Zimbabwe	5	3	2
Nigeria	2	2	2
Iran	1	1	2
Australia	2	0	2
Iran	4	1	2
South Africa	2	1	2
Tunisia	2	1	2
Israel	3	2	2
USA	4	3	2
Egypt	4	5	2
Tunisia	5	0	2
Iran	1	0	2
Vietnam	4	3	2
US Virgin Islands	2	2	2
Germany	5	2	2
Nigeria	3	2	2
China, P.R.	4	3	2
Tunisia	1	1	2
Japan	2	1	2
Japan	4	1	2
Ukraine	2	2	2
USA	2	2	2
Singapore	3	3	2
Mexico	3	2	2
Venezuela	3	3	2
USA	2	2	2
Singapore	1	1	2
Iran	2	1	2
Colombia	3	3	2
India	4	3	2
Chile	1	1	2
Brazil	6	0	2
Peru	2	2	2
France	4	2	2
India	2	1	2
France	3	1	2
Spain	1	0	2
Sri Lanka	2	1	2
Hong Kong, China	2	1	2
Slovakia	4	2	2
Spain	1	1	2
Morocco	2	2	2
China, P.R.	2	0	2
Bolivia	1	0	2
Poland	10	7	2
Serbia	4	2	2
Israel	7	2	2

Poland	1	1	2
Italy	1	0	2
Lithuania	5	2	2
Ukraine	1	1	2
Vanuatu	2	2	2
Switzerland	2	1	2
Germany	10	4	2
Russia	6	2	2
Russia	4	4	2
Brazil	9	6	2
Peru	1	1	2
Belgium	9	2	2
Bosnia and Herzegov	1	0	2
Kazakhstan	7	4	2
Japan	6	1	2
Cayman Islands	1	1	2
Great Britain	2	0	2
Korea, Rep.	3	3	2
Korea, Rep.	4	3	2
Iran	4	2	2
Poland	13	8	2
Iran	2	1	2
Spain	2	2	2
Italy	3	1	2
Sweden	10	5	2
France	4	3	2
Iran	1	1	2
Finland	4	1	2
Spain	6	4	2
South Africa	5	4	2
Israel	1	0	2
Israel	5	2	2
Estonia	7	7	2
Guadeloupe	2	0	2
Great Britain	7	3	2
Slovakia	4	3	2
India	7	4	2
Italy	2	0	2
Turkiye	12	7	2
Georgia	2	2	2
Russia	5	3	2
Georgia	4	4	2
Romania	7	2	2
Sweden	8	5	2
Germany	3	1	2
Romania	7	3	2
Belarus	6	3	2
Italy	2	1	2
South Africa	2	1	2
Italy	4	1	2

Azerbaijan	4	3	2
Germany	4	0	2
Ireland	3	3	2
Australia	5	4	2
Netherlands	4	1	2
Korea, Rep.	7	5	2
Spain	2	1	2
Serbia	14	3	2
Uzbekistan	3	1	2
Germany	1	0	2
Czechia	5	3	2
Belgium	3	1	2
India	2	1	2
Italy	5	2	2
Canada	6	4	2
Finland	6	1	2
Latvia	3	2	2
Poland	10	6	2
Turkiye	1	1	2
India	2	2	2
Georgia	4	1	2
Portugal	2	1	2
Russia	4	2	2
Portugal	5	4	2
Korea, Rep.	4	2	2
Korea, Rep.	4	2	2
Romania	7	0	2
Belarus	4	1	2
Kazakhstan	6	3	2
Sri Lanka	3	2	2
Spain	2	2	2
Italy	6	2	0
New Zealand	5	5	0
Poland	1	1	0
Russia	2	2	0
Portugal	6	6	0
Israel	6	4	0
Slovenia	12	6	0
Thailand	4	2	0
Armenia	1	1	0
Kazakhstan	10	5	0
Denmark	3	3	0
Bulgaria	3	2	0
India	1	1	0
France	8	4	0
Canada	1	1	0
Turkiye	6	4	0
Hong Kong, China	1	1	0
USA	3	2	0
Japan	1	1	0

USA	1	1	0
Czechia	3	2	0
Netherlands	9	7	0
Australia	5	5	0
Czechia	10	4	0
Czechia	5	2	0
Croatia	3	2	0
Bulgaria	10	3	0
Estonia	3	2	0
Switzerland	7	1	0
Croatia	4	1	0
Slovakia	4	1	0
USA	3	2	0
Latvia	5	4	0
Russia	1	1	0
Colombia	3	2	0
Russia	1	1	0
USA	1	1	0
Australia	8	7	0
El Salvador	1	1	0
China, P.R.	2	2	0
USA	3	2	0
El Salvador	6	6	0
Canada	2	2	0
USA	3	3	0
Chinese Taipei	8	4	0
Switzerland	1	1	0
Aruba	1	1	0
Haiti	2	2	0
USA	3	3	0
Vietnam	4	1	0
USA	2	2	0
Canada	2	1	0
Brazil	9	6	0
Czechia	0	1	0
USA	4	1	0
France	3	3	0
Mexico	2	2	0
Egypt	3	3	0
Romania	2	1	0
Denmark	3	1	0
Belgium	8	5	0
Venezuela	1	1	0
Chile	2	2	0
Great Britain	1	1	0
Greece	4	1	0
Egypt	8	7	0
Colombia	9	7	0
USA	3	2	0
Aruba	3	3	0

China, P.R.	5	4	0
Estonia	3	2	0
Qatar	12	9	0
Egypt	4	4	0
Egypt	5	4	0
China, P.R.	2	2	0
Pakistan	2	2	0
Singapore	2	2	0
Pakistan	2	2	0
France	2	2	0
Sweden	6	1	0
Poland	7	3	0
Japan	3	1	0
Japan	8	4	0
Great Britain	2	1	0
Serbia	3	2	0
Serbia	4	4	0
Canada	7	4	0
Korea, Rep.	8	5	0
USA	6	3	0
Korea, Rep.	6	5	0
India	3	1	0
USA	5	3	0
Belgium	6	3	0
Chinese Taipei	9	6	0
Philippines	11	6	0
Bolivia	1	1	0
Japan	8	3	0
Hungary	5	4	0
Nigeria	1	1	0
Argentina	3	2	0
Japan	1	1	0
Mexico	2	2	0
Guatemala	6	6	0
France	2	2	0
India	1	1	0
China, P.R.	2	2	0
Singapore	2	2	0
Canada	3	2	0
Singapore	3	2	0
USA	2	1	0
Japan	2	2	0
Colombia	5	4	0
Colombia	5	4	0
Bolivia	2	2	0
Colombia	3	3	0
Italy	9	7	0
Poland	9	7	0
Hong Kong, China	6	5	0
Finland	6	2	0

USA	5	2	0
Finland	4	1	0
Uruguay	1	1	0
Korea, Rep.	6	1	0
Italy	7	2	0
Australia	4	3	0
Trinidad & Tobago	6	6	0
India	4	4	0
Moldova	1	1	0
Austria	5	3	0
Kazakhstan	3	2	0
Latvia	9	5	0
Bulgaria	8	7	0
Canada	4	3	0
Russia	2	2	0
South Africa	6	4	0
Italy	6	4	0
Turkiye	7	4	0
Denmark	6	1	0
Moldova	15	3	0
Ukraine	14	9	0
Russia	9	5	0
France	3	3	0
Romania	6	5	0
Croatia	6	2	0
Germany	3	3	0
India	2	1	0
Kazakhstan	7	2	0
Serbia	7	3	0
Serbia	6	4	0
Germany	8	5	0
Bulgaria	9	5	0
Kazakhstan	16	8	0
Spain	3	2	0
Japan	6	4	0
Germany	6	2	0
Romania	2	1	0
Spain	1	1	0
India	2	1	0
Norway	7	1	0
Great Britain	2	1	0
France	6	1	0
Italy	5	3	0
Italy	6	2	0
Netherlands	1	1	0
Latvia	16	5	0
Great Britain	1	1	0
Philippines	2	2	0
Armenia	3	1	0
Great Britain	6	4	0

Croatia	6	5	0
USA	7	2	0
India	2	1	0
Ukraine	4	2	0
Luxembourg	2	2	0
South Africa	6	5	0
Bulgaria	4	4	0
Kazakhstan	6	4	0
Russia	0	2	0
Italy	5	2	0
Spain	4	1	0
Italy	2	2	0
Spain	1	1	0
Malaysia	4	4	0
Kyrgyzstan	7	5	0
Netherlands	9	4	0
India	2	1	0
Indonesia	3	2	0
Italy	3	2	0
New Zealand	1	1	0
Russia	6	4	0
Italy	6	2	0
India	1	1	0
Indonesia	3	3	0
Switzerland	2	2	0
Croatia	4	1	0
Canada	1	1	0
Macau	1	1	0
Romania	8	1	0
Netherlands	3	2	0
Russia	3	1	0
Georgia	3	2	0
France	7	4	0
Luxembourg	4	3	0
Russia	8	4	0
Cyprus	8	6	0
Sweden	5	4	0
Korea, Rep.	7	5	0
South Africa	6	4	0
Great Britain	7	2	0
Tunisia	4	2	0
Turkiye	2	1	0
Hong Kong, China	4	3	0
Austria	6	3	0
Kazakhstan	9	3	0
Belarus	1	1	0
Norway	6	2	0
Germany	5	3	0
Turkiye	1	1	0
Japan	2	1	0

Antigua & Barbuda	1	1	0
Poland	12	2	0
Romania	2	1	0
Russia	3	2	0
Nepal	2	2	0
New Zealand	1	1	0
Slovakia	5	4	0
Estonia	13	7	0
Japan	3	1	0
Serbia	1	1	0
Italy	1	1	0
Romania	7	4	0
Poland	8	3	0
Spain	2	1	0
Morocco	6	3	0
Serbia	4	3	0
India	1	1	0
Italy	3	1	0
Great Britain	3	3	0
Netherlands	5	2	0
Croatia	3	3	0
Iran	2	2	0
Argentina	2	2	0
Argentina	3	3	0
Ecuador	1	1	0
Peru	4	4	0
Ecuador	2	2	0
Bolivia	2	2	0
Bolivia	0	1	0
Colombia	3	3	0
Ukraine	1	1	0
Russia	7	5	0
Australia	2	2	0
Spain	3	3	0
Argentina	1	1	0
USA	2	2	0
Sri Lanka	2	2	0
Indonesia	2	2	0
Colombia	7	6	0
Egypt	8	8	0
Egypt	5	2	0
New Zealand	1	1	0
Belarus	6	2	0
Spain	1	1	0
Canada	2	1	0
New Zealand	6	2	0
USA	2	2	0
Mexico	2	2	0
Argentina	1	1	0
Mexico	7	6	0

Egypt	4	6	0
China, P.R.	3	2	0
Thailand	2	2	0
Great Britain	4	2	0
South Africa	1	1	0
Argentina	3	2	0
Egypt	6	7	0
Costa Rica	5	3	0
Trinidad & Tobago	3	3	0
Trinidad & Tobago	3	3	0
Peru	2	1	0
Colombia	2	2	0
Egypt	4	7	0
Australia	8	7	0
India	6	4	0
Egypt	9	6	0
Japan	1	1	0
Sweden	1	1	0
Tunisia	7	2	0
USA	3	1	0
Tunisia	6	3	0
Egypt	7	4	0
Tunisia	6	3	0
USA	1	1	0
New Zealand	2	2	0
Colombia	2	1	0
Colombia	1	1	0
Peru	2	1	0
Italy	2	2	0
Korea, Rep.	6	3	0
USA	3	3	0
Ukraine	6	3	0
Tunisia	2	2	0
Ukraine	4	3	0
Bolivia	1	1	0
Peru	2	2	0
Honduras	4	4	0
Chinese Taipei	4	3	0
Romania	3	2	0
Egypt	7	6	0
India	5	1	0
China, P.R.	3	1	0
Zimbabwe	2	1	0
Thailand	1	1	0
Israel	8	6	0
Zimbabwe	5	5	0
USA	6	5	0
Great Britain	3	3	0
Colombia	2	2	0
Singapore	2	1	0

Venezuela	5	4	0
Hong Kong, China	6	3	0
Ecuador	5	5	0
Canada	6	5	0
USA	4	2	0
Italy	1	1	0
Turkiye	2	1	0
Japan	5	1	0
Venezuela	3	3	0
Argentina	2	1	0
Canada	4	1	0
Slovakia	6	3	0
USA	3	2	0
New Zealand	3	2	0
Nepal	2	2	0
Sri Lanka	1	1	0
Bolivia	1	1	0
Israel	13	4	0
Germany	6	1	0
USA	1	1	0
Morocco	1	2	0
Kazakhstan	5	2	0
Spain	2	1	0
Romania	4	4	0
Japan	1	1	0
Turkiye	8	4	0
Russia	3	2	0
Estonia	3	2	0
Japan	4	2	0
Turkiye	4	2	0
Albania	1	1	0
Greece	4	1	0
India	2	2	0
Argentina	4	4	0
China, P.R.	7	5	0
Ukraine	8	3	0
Cambodia	4	2	0
Hungary	6	3	0
Korea, Rep.	6	4	0
Serbia	2	3	0
Morocco	3	1	0
Zimbabwe	3	3	0
Tunisia	2	1	0
Zimbabwe	3	3	0
USA	1	1	0
Sri Lanka	4	4	0
New Zealand	1	1	0
New Zealand	3	3	0
Egypt	2	2	0
USA	1	1	0

Tunisia	5	5	0
France	11	7	2185
Ukraine	6	1	2330
USA	8	7	1445
USA	8	6	1725
Japan	13	11	925
France	11	7	1380
USA	11	11	1250
Latvia	11	10	985
Philippines	11	11	1235
Czechia	14	14	1015
Russia	14	13	960
Russia	12	12	935
USA	13	12	1040
Russia	9	8	715
USA	20	18	1015
Burundi	14	13	900
USA	9	9	820
China, P.R.	7	5	1060
Canada	14	13	875
France	15	13	700
Russia	13	11	876
Andorra	16	14	890
Thailand	13	13	815
Spain	13	12	855
China, P.R.	18	18	760
Ecuador	18	17	775
Indonesia	15	16	870
Hong Kong, China	16	17	750
France	18	18	725
France	16	13	485
USA	13	13	425
Great Britain	15	15	605
Hungary	12	11	280
Armenia	15	14	660
Russia	14	12	610
Germany	16	13	660
Germany	18	18	615
Israel	21	17	400
USA	11	11	700
Great Britain	20	13	640
Sweden	9	9	600
Slovenia	17	17	575
France	22	15	450
Russia	9	9	480
Korea, Rep.	15	12	481
Cyprus	12	13	530
Korea, Rep.	10	10	465
Poland	20	16	475
Latvia	22	21	546

Germany	22	21	516
France	22	22	475
Malta	12	10	510
Slovenia	20	19	425
Russia	14	14	495
Ukraine	13	12	405
Uruguay	16	15	460
Spain	15	13	440
Argentina	14	13	505
Chinese Taipei	7	5	515
USA	16	16	410
France	15	13	492
USA	14	11	515
Australia	14	14	435
Italy	13	10	485
USA	20	19	425
Colombia	15	15	380
USA	11	9	430
USA	18	12	440
Peru	14	14	380
USA	9	9	110
USA	16	13	455
Slovakia	15	13	375
USA	12	10	410
Italy	14	12	441
Japan	16	14	250
France	9	7	335
Indonesia	14	13	356
Brazil	16	13	345
USA	17	17	376
Slovakia	17	17	275
China, P.R.	17	15	380
Italy	8	6	270
USA	23	20	292
USA	26	21	330
Poland	13	9	255
Puerto Rico	23	21	290
Spain	14	12	325
Italy	12	8	340
Switzerland	13	13	280
USA	16	15	310
France	18	16	305
Peru	13	10	275
Australia	26	18	310
Korea, Rep.	16	16	215
Thailand	6	7	275
Croatia	20	13	302
Russia	20	17	272
Ukraine	10	9	220
Bulgaria	16	13	210

Japan	17	16	215
Spain	14	13	210
Russia	6	6	220
Russia	8	7	150
USA	10	9	150
Japan	12	8	176
Thailand	8	8	150
USA	17	14	166
Georgia	10	7	161
USA	17	16	134
USA	11	8	172
South Africa	8	4	24
France	10	7	2950
USA	8	9	2280
China, P.R.	8	5	2045
Denmark	13	11	1960
Colombia	13	9	2030
Chinese Taipei	7	6	1745
China, P.R.	7	5	1415
USA	6	6	1100
China, P.R.	16	10	1540
Luxembourg	11	10	1455
Japan	10	9	1140
France	16	15	1220
Canada	10	8	1355
Japan	12	12	870
Italy	13	13	955
USA	16	16	840
USA	15	14	840
USA	17	14	920
Ukraine	15	15	910
Thailand	16	15	840
USA	15	14	920
Great Britain	7	0	980
New Zealand	17	14	705
Korea, Rep.	15	14	695
Latvia	17	17	855
Chinese Taipei	14	14	680
Hong Kong, China	20	16	735
Hungary	22	22	580
Russia	12	11	645
USA	11	11	740
Thailand	15	12	675
Burundi	18	18	660
Spain	21	19	595
France	20	17	535
Russia	16	16	570
Ireland	12	12	575
Russia	19	19	500
USA	14	13	630

Ukraine	13	13	510
Ukraine	16	6	700
Japan	16	14	555
Belarus	13	13	465
Slovakia	18	18	520
Argentina	10	9	420
Argentina	15	13	530
Armenia	10	10	475
Sweden	22	20	530
Turkiye	18	16	455
France	16	15	435
USA	21	20	356
Malta	14	14	480
USA	14	13	395
Poland	15	14	465
Cyprus	18	15	475
USA	13	12	430
USA	7	7	345
France	14	13	450
Guatemala	16	16	451
Bulgaria	21	19	435
Japan	12	11	311
Georgia	14	12	410
France	17	16	360
France	25	18	341
Latvia	7	7	475
Japan	18	18	240
Papua New Guinea	8	8	305
Italy	20	16	295
Poland	18	14	265
Canada	17	16	350
Switzerland	16	12	380
Italy	17	15	265
Italy	15	12	355
USA	13	12	250
Guatemala	24	23	336
USA	11	10	355
Bulgaria	11	11	320
France	12	9	325
Romania	12	11	255
Japan	8	5	250
Colombia	12	10	271
USA	11	11	140
Georgia	12	12	190
New Zealand	14	11	205
USA	14	12	245
USA	17	13	245
Slovenia	19	17	202
Denmark	15	8	188
Slovakia	11	11	145

USA	13	9	160
Slovenia	7	8	60
USA	15	13	1377,5
Ukraine	9	8	1137,5
Kazakhstan	11	10	972,5
China, P.R.	12	10	810
Serbia	9	8	605
Chinese Taipei	16	16	675
Colombia	9	9	610
Colombia	12	10	640
Switzerland	12	11	655
Canada	12	9	480
Argentina	13	10	610
USA	10	10	610
Great Britain	13	13	550
Russia	16	14	565
Japan	20	19	475
Slovenia	6	5	535
Chinese Taipei	17	16	505
Russia	15	14	525
USA	11	11	550
USA	11	8	460
China, P.R.	12	10	420
New Zealand	21	20	520
Russia	13	12	510
Japan	17	17	442,5
Australia	13	13	510
Latvia	16	15	470
USA	12	11	332,5
France	18	16	432,5
USA	15	14	425
Germany	13	11	425
Russia	11	11	450
Switzerland	7	5	465
India	8	5	480
USA	10	10	375
USA	19	18	402,5
Papua New Guinea	13	11	390
Canada	14	11	412,5
USA	16	12	410
Burundi	17	14	382,5
USA	14	13	385
Ukraine	20	19	350
India	9	9	440
Finland	20	18	382,5
India	12	12	425
Russia	13	11	410
Serbia	17	15	360
Poland	24	24	340
Italy	11	10	372,5

Japan	17	17	307,5
USA	11	10	305
Slovakia	18	16	350
Chinese Taipei	11	11	300
Spain	14	13	345
Morocco	19	19	330
USA	16	16	260
Croatia	9	9	310
USA	14	14	280
Thailand	18	17	300
Great Britain	17	14	300
USA	14	13	270
Chile	13	13	260
Romania	20	19	307,5
Japan	9	7	305
Brazil	11	11	240
Czechia	16	15	275
USA	10	10	290
Moldova	14	13	275
USA	17	16	275
USA	10	10	300
Colombia	13	12	235
USA	19	17	220
Peru	19	17	230
Chinese Taipei	11	9	200
Japan	17	15	200
Switzerland	25	20	250
Japan	16	12	190
Japan	10	9	240
Norway	10	7	210
Poland	7	7	180
Slovakia	16	11	220
Canada	20	17	210
Japan	15	14	180
USA	22	22	195
Netherlands	14	12	200
Norway	10	7	140
Mexico	10	7	80
USA	17	9	160
Brazil	14	12	115
USA	20	20	87,5
Chinese Taipei	9	9	30
Russia	12	12	1330
Russia	10	9	1092,5
USA	7	7	1040
Switzerland	10	3	1005
USA	11	8	950
Slovenia	13	12	792,5
Russia	16	16	810
Ukraine	6	4	727,5

USA	11	8	612,5
USA	13	13	657,5
China, P.R.	14	13	595
USA	5	4	620
Serbia	14	13	535
Poland	12	9	570
Great Britain	18	12	575
Hungary	16	15	530
Ukraine	8	6	645
USA	13	8	590
Japan	16	15	485
Great Britain	20	20	490
France	15	14	575
Japan	14	13	480
USA	11	10	430
Argentina	21	21	525
Canada	12	10	520
Japan	18	18	470
Spain	12	11	510
Romania	16	16	462,5
Peru	14	14	470
USA	13	11	402,5
Norway	21	19	380
Spain	15	13	390
Italy	17	16	380
Canada	6	3	470
China, P.R.	17	16	450
Paraguay	17	17	445
Kazakhstan	9	10	420
Romania	11	8	480
Croatia	16	15	380
Chinese Taipei	16	15	365
Czechia	11	11	420
Switzerland	14	14	387,5
Australia	14	11	380
Austria	13	11	385
Italy	18	17	320
Germany	19	15	342,5
Argentina	16	16	370
Mexico	20	18	305
Czechia	14	14	330
USA	6	6	380
Sweden	20	18	310
USA	20	19	320
Belgium	20	19	350
Colombia	15	9	332,5
Russia	12	11	345
USA	12	10	320
Israel	17	14	320
Italy	15	14	275

Chile	21	21	275
USA	14	11	280
Japan	7	7	265
USA	13	12	290
China, P.R.	13	10	280
USA	13	12	270
USA	12	12	265
Ecuador	15	12	210
Burundi	16	16	245
Canada	7	7	212,5
USA	10	9	220
Romania	18	17	225
Romania	9	9	210
Greece	9	9	175
China, P.R.	12	10	210
Japan	12	12	160
USA	11	6	230
China, P.R.	17	13	170
Czechia	8	8	155
Russia	8	2	210
China, P.R.	8	8	160
Great Britain	6	4	155
Netherlands	11	10	130
USA	14	13	170
Australia	6	5	130
Israel	21	17	135
Russia	20	16	120
Australia	6	3	100
Turkiye	7	5	70
Hungary	10	10	995
Czechia	9	8	832,5
Great Britain	12	11	850
Canada	15	14	837,5
Russia	13	12	750
Slovakia	14	13	717,5
USA	12	12	730
USA	11	11	715
Canada	14	14	770
USA	15	14	735
Czechia	11	10	475
Russia	10	10	630
USA	12	12	542,5
Russia	15	14	560
Belarus	11	10	550
Germany	8	6	570
China, P.R.	18	18	550
Argentina	10	10	600
India	16	16	510
Russia	8	7	615
Russia	11	10	555

Russia	13	7	585
Brazil	11	10	482,5
Switzerland	11	9	560
Russia	11	11	510
Hungary	7	6	505
USA	9	9	505
USA	8	8	495
Slovakia	8	8	470
Algeria	16	14	470
USA	18	18	442,5
Great Britain	16	16	395
Japan	16	15	472,5
Canada	13	11	430
Romania	13	12	407,5
Italy	9	9	462,5
Romania	17	16	437,5
USA	14	12	445
USA	8	8	455
France	11	10	395
Italy	15	15	395
Colombia	12	12	350
Great Britain	15	14	340
USA	6	4	332,5
Mexico	18	17	350
Romania	14	9	395
Brazil	21	19	320
Japan	14	12	310
Romania	16	15	280
Switzerland	6	5	320
Denmark	13	13	240
Australia	7	7	260
New Zealand	24	20	295
Greece	11	11	250
Indonesia	12	11	295
Hungary	25	24	255
India	13	5	335
Slovenia	9	7	325
Australia	9	9	270
China, P.R.	14	12	290
USA	18	11	290
Colombia	19	19	305
Ukraine	14	13	300
Belgium	6	5	270
Australia	6	4	285
Russia	14	9	300
Czechia	11	9	307,5
Argentina	18	18	260
Australia	9	7	245
Estonia	7	7	240
Great Britain	11	8	250

China, P.R.	19	16	255
Canada	15	13	245
Romania	14	13	260
USA	13	9	280
Colombia	13	12	220
Argentina	15	15	230
Australia	8	8	260
Mexico	15	14	220
USA	7	3	260
USA	17	16	180
Sweden	21	19	225
Peru	15	15	195
Brazil	11	10	255
Argentina	17	13	225
USA	10	8	200
France	11	10	155
China, P.R.	20	17	180
Mexico	16	15	160
Japan	15	13	180
Australia	7	2	190
USA	10	7	30
USA	11	10	1042,5
China, P.R.	9	9	1022,5
Belarus	11	11	827,5
Switzerland	10	10	692,5
Russia	5	4	875
Slovakia	11	10	820
Czechia	9	4	815
Latvia	9	10	767,5
Spain	10	10	735
Spain	13	13	740
Ukraine	8	7	647,5
Czechia	15	14	662,5
Romania	14	14	705
Hungary	15	15	665
Russia	16	15	650
Hungary	13	14	560
USA	13	14	612,5
Romania	9	8	550
Australia	12	10	655
Russia	15	15	590
Russia	9	9	522,5
Great Britain	14	14	502,5
Ukraine	7	5	605
USA	16	15	595
USA	17	16	482,5
Great Britain	9	9	470
China, P.R.	8	8	490
Russia	17	15	450
Hungary	13	12	490

Brazil	20	20	450
Australia	16	14	495
Belgium	15	15	515
Russia	17	16	460
Lithuania	9	7	482,5
China, P.R.	15	11	505
Great Britain	15	14	485
Egypt	13	12	475
Slovakia	13	12	415
Indonesia	13	12	380
France	17	15	465
Mexico	13	14	380
Thailand	9	9	410
Italy	15	14	440
Czechia	12	11	412,5
USA	13	12	422,5
USA	14	14	412,5
Great Britain	15	14	347,5
Great Britain	16	16	425
Australia	12	11	415
Slovakia	16	14	305
Australia	11	12	260
France	8	3	420
France	16	11	370
Russia	20	19	360
Canada	14	13	365
Serbia	7	7	350
Ukraine	10	8	370
Romania	12	11	272,5
France	13	12	355
Brazil	14	12	310
USA	18	16	305
Australia	14	14	310
Ecuador	9	9	302,5
Australia	14	11	300
Colombia	15	14	300
USA	15	15	300
USA	21	17	315
India	16	14	285
Austria	13	13	315
Denmark	21	16	275
Great Britain	18	16	230
Great Britain	14	13	210
Canada	14	14	270
USA	14	12	300
Chile	21	20	275
China, P.R.	11	11	165
USA	16	12	295
Italy	19	15	295
Puerto Rico	12	11	280

Russia	15	14	242,5
China, P.R.	22	19	255
Italy	11	9	275
China, P.R.	9	9	200
Romania	13	13	200
Lithuania	15	13	220
USA	10	8	240
Russia	16	15	220
Namibia	20	20	230
Great Britain	18	12	250
Japan	16	14	160
Canada	15	13	220
Canada	13	11	220
USA	10	10	190
France	14	8	195
USA	14	13	170
USA	19	16	180
Great Britain	16	14	180
Australia	10	8	150
Mexico	16	16	140
Great Britain	13	10	60
Switzerland	8	8	1862,5
Croatia	6	5	1170
Russia	11	11	1122,5
Czechia	12	12	820
Serbia	12	11	905
Czechia	7	7	655
USA	14	13	790
Russia	10	8	795
USA	8	7	810
Belgium	11	10	670
Paraguay	20	19	650
Ukraine	11	11	575
Belarus	13	13	555
China, P.R.	13	13	555
Latvia	9	9	602,5
Turkiye	11	11	520
Romania	15	15	512,5
Serbia	16	16	490
Mexico	18	18	507,5
Argentina	12	12	520
Italy	10	10	510
Czechia	15	12	525
Italy	17	17	470
China, P.R.	10	10	515
Russia	10	10	485
Mexico	14	14	437,5
Lithuania	22	18	470
Ukraine	15	13	510
Russia	6	7	480

Egypt	15	15	492,5
Switzerland	6	4	495
Slovakia	14	11	505
USA	10	10	460
Brazil	6	5	440
Great Britain	17	16	435
Ukraine	20	20	432,5
USA	19	17	420
USA	15	15	435
Russia	16	13	412,5
Ecuador	12	12	350
Russia	11	11	395
Chinese Taipei	11	11	395
China, P.R.	14	13	320
Australia	16	16	370
Mexico	17	15	422,5
France	13	13	347,5
Canada	6	6	300
Colombia	15	15	330
Australia	20	19	342,5
Croatia	13	13	310
Brazil	21	20	315
USA	17	16	295
Kazakhstan	16	13	335
Thailand	9	9	300
USA	14	11	345
Switzerland	15	15	290
Canada	19	17	330
France	13	12	270
Italy	12	12	325
Hungary	12	11	265
Hong Kong, China	12	13	290
USA	7	7	300
Great Britain	19	18	270
Great Britain	16	14	205
Great Britain	7	7	315
USA	15	13	280
Japan	16	13	240
USA	16	16	270
Japan	12	12	240
Czechia	12	12	250
Hungary	16	15	245
USA	14	13	250
Italy	13	11	222,5
Belgium	22	17	180
Mexico	16	16	215
Russia	24	19	245
Italy	11	10	185
Colombia	19	18	210
Japan	10	10	195

Great Britain	14	11	220
USA	23	18	200
Mexico	17	16	170
Australia	14	11	160
Paraguay	12	8	190
Switzerland	14	13	165
USA	13	13	160
Brazil	14	12	110
Egypt	8	6	145
USA	21	16	130
USA	10	10	1020
Czechia	16	14	940
Canada	6	4	880
Croatia	13	12	880
Germany	14	12	750
Canada	12	8	680
Mexico	17	17	630
Slovakia	20	19	670
Switzerland	14	14	640
Russia	12	12	610
Canada	17	16	600
Kazakhstan	8	8	570
Chinese Taipei	18	16	635
USA	11	11	590
USA	11	10	500
USA	5	5	570
Bolivia	14	14	515
USA	17	17	480
Belgium	23	23	470
Estonia	7	5	530
Brazil	14	12	465
Russia	11	9	475
USA	14	14	450
Czechia	15	14	460
Brazil	13	13	380
Great Britain	19	17	430
New Zealand	20	19	425
USA	7	7	470
Austria	14	12	465
Ukraine	15	14	440
Hungary	15	15	400
Paraguay	11	11	410
Argentina	15	15	405
Japan	20	17	410
USA	13	12	390
Italy	16	15	380
Kosovo	16	16	390
Ecuador	16	15	350
Sweden	16	15	395
Sweden	9	9	400

USA	8	8	85
Latvia	10	10	350
Turkiye	16	14	335
Belgium	13	13	330
Romania	14	14	320
Mexico	17	16	300
Japan	13	9	250
Indonesia	18	18	310
Belgium	12	11	310
Colombia	16	16	320
France	14	10	365
Slovakia	22	19	295
Uzbekistan	6	6	360
Russia	11	9	305
Argentina	9	9	320
Czechia	17	16	270
Madagascar	15	13	270
Belgium	12	11	280
Egypt	12	12	270
Peru	14	11	240
Japan	13	12	190
Russia	14	10	300
Russia	13	9	300
Israel	17	13	270
New Zealand	17	16	250
Belgium	18	17	260
USA	23	21	230
USA	10	10	260
Belgium	17	14	190
Liechtenstein	10	8	230
Poland	11	11	170
Belgium	16	14	220
Mexico	14	13	215
USA	14	13	180
USA	11	11	210
Netherlands	9	4	220
Croatia	9	8	210
USA	12	11	210
Great Britain	14	12	165
Australia	17	14	170
Canada	23	18	165
Egypt	10	6	170
India	11	9	170
Belgium	11	11	160
Venezuela	14	14	140
USA	9	8	140
Korea, Rep.	6	5	115
Russia	10	10	1000
Australia	11	11	980
Belgium	5	5	920

Canada	14	11	860
France	6	1	820
USA	6	3	680
Montenegro	10	10	650
Paraguay	18	17	635
Estonia	13	13	735
Netherlands	16	13	680
Kazakhstan	9	3	690
Serbia	17	14	585
Argentina	18	18	570
Slovakia	13	13	570
Russia	12	12	470
Poland	15	13	505
Czechia	14	14	480
Uzbekistan	12	10	460
Croatia	11	9	515
USA	9	5	520
Ecuador	16	15	370
Serbia	8	8	480
Czechia	14	14	415
Ukraine	19	19	390
Ukraine	9	9	450
Peru	17	17	430
Sweden	18	16	430
Japan	13	12	325
Netherlands	9	9	140
Madagascar	17	17	425
Czechia	11	10	440
Belarus	17	17	365
Russia	15	13	410
Canada	17	17	380
Japan	15	13	320
USA	16	16	360
Belarus	9	9	335
New Zealand	15	14	330
Australia	14	14	310
USA	21	21	320
Japan	15	13	300
Ecuador	17	17	350
Austria	16	16	335
Japan	13	11	280
Russia	12	11	335
Japan	19	19	270
Slovakia	10	9	350
Japan	15	14	230
Romania	16	16	300
USA	12	10	275
Brazil	18	16	230
Mexico	15	13	250
Bulgaria	11	11	245

Belgium	19	17	240
Japan	12	11	210
Switzerland	22	18	200
Mexico	18	15	240
Japan	10	9	185
Great Britain	14	14	255
USA	11	10	210
Mexico	16	16	215
Brazil	17	15	210
USA	13	13	215
Ukraine	10	9	210
Philippines	11	10	180
Venezuela	14	12	210
USA	20	20	200
Slovakia	6	6	200
Japan	12	9	180
Australia	7	4	220
USA	16	14	180
Japan	12	11	100
Russia	6	3	170
USA	16	15	180
Canada	16	12	170
Slovakia	7	7	155
Russia	12	10	140
Peru	10	8	115
Mexico	16	10	120
Hungary	16	14	105
USA	11	9	130
Great Britain	17	15	120
Great Britain	23	18	100
France	6	5	45
Australia	9	8	1320
Ukraine	13	12	930
USA	11	7	930
Puerto Rico	17	11	870
Russia	14	12	800
Czechia	7	7	850
Hungary	10	8	530
Czechia	7	7	840
China, P.R.	13	13	630
Belgium	13	13	640
Japan	7	6	710
Slovakia	13	10	540
Kazakhstan	15	10	680
Tunisia	13	13	635
Paraguay	18	16	590
Ukraine	17	15	585
France	15	13	550
Romania	17	17	530
Slovakia	12	12	370

China, P.R.	7	7	460
Russia	16	13	485
Belarus	19	17	470
USA	11	9	470
Slovenia	8	7	440
Madagascar	17	17	465
Serbia	8	8	430
USA	8	4	410
Canada	9	9	360
Czechia	17	13	390
USA	6	6	450
Argentina	17	16	395
Canada	15	13	350
China, P.R.	10	8	360
Venezuela	16	15	350
Serbia	12	11	360
Slovakia	18	17	375
Montenegro	14	14	375
Croatia	8	8	330
Venezuela	15	14	340
Belgium	15	14	340
Chile	16	14	340
Japan	18	18	330
Thailand	13	13	300
Sweden	14	11	330
Japan	14	13	300
Norway	11	10	305
Canada	11	11	360
Slovakia	16	15	320
France	14	12	320
Chile	17	16	330
USA	10	7	320
Indonesia	14	13	310
Bulgaria	15	13	325
Netherlands	20	18	220
Denmark	10	10	270
Netherlands	15	13	260
USA	10	9	220
Belarus	13	11	280
USA	9	6	330
USA	10	6	270
Great Britain	16	16	275
Japan	16	15	270
Belarus	14	14	260
Serbia	16	16	280
Argentina	15	14	270
India	14	12	245
Germany	12	10	230
Japan	16	14	220
France	14	14	230

USA	12	12	215
Tunisia	15	14	230
Japan	13	8	260
USA	9	7	250
USA	14	11	200
Great Britain	19	19	210
USA	9	8	250
Canada	11	11	220
Russia	11	9	225
Russia	11	10	225
Mexico	16	16	160
Turkiye	10	10	180
Russia	7	4	160
Singapore	15	12	140
Canada	9	8	80
Bahamas	11	8	140
Japan	11	9	135
Russia	6	5	100
USA	10	8	100
France	12	11	1360
Thailand	8	6	900
Hungary	16	16	870
Great Britain	12	12	860
Australia	12	8	730
Japan	10	10	650
Croatia	18	17	610
Australia	9	8	560
Chile	15	15	640
USA	5	5	650
Slovenia	15	12	625
Canada	12	10	565
Russia	14	11	560
Belgium	14	10	470
Kazakhstan	12	8	630
Netherlands	10	9	575
Russia	12	11	420
Hungary	19	19	510
Slovakia	14	14	520
Russia	16	16	515
Norway	17	17	500
South Africa	12	12	540
China, P.R.	16	15	480
Great Britain	6	6	520
Romania	7	7	500
Russia	15	15	470
USA	10	4	475
Russia	12	11	420
Serbia	10	10	420
Serbia	12	11	420
Japan	17	14	440

Poland	11	11	355
Belarus	10	10	320
Paraguay	18	18	360
Indonesia	21	18	400
Canada	13	12	410
Canada	18	19	370
USA	9	9	370
Argentina	12	11	360
USA	12	12	350
USA	13	6	410
Japan	14	12	370
Romania	6	5	290
Belarus	14	14	325
Germany	16	16	360
Oman	13	13	295
Russia	14	13	290
Slovakia	12	12	290
Thailand	19	16	310
Italy	8	8	305
Venezuela	15	14	315
Russia	18	18	270
Slovakia	15	13	200
USA	10	10	265
Russia	11	10	265
Great Britain	11	11	255
Romania	17	13	275
USA	6	5	260
USA	19	17	205
Denmark	18	18	195
Chinese Taipei	10	8	255
Great Britain	10	10	220
USA	13	10	220
USA	7	7	205
Netherlands	8	8	230
Hungary	17	15	150
USA	12	11	195
USA	9	7	240
India	22	19	185
China, P.R.	15	10	220
Netherlands	6	5	190
India	11	10	195
USA	8	5	200
Great Britain	21	20	190
Bahamas	18	17	185
Great Britain	17	15	170
Mexico	12	11	180
Switzerland	22	17	140
USA	18	13	120
Australia	6	4	90
Brazil	6	5	75

Mexico	17	14	20
Thailand	16	14	900
Netherlands	7	7	870
Romania	4	4	970
Romania	17	16	870
Great Britain	12	11	890
Romania	13	11	870
Japan	9	5	700
USA	6	7	710
Serbia	12	12	640
France	18	17	535
Indonesia	19	19	540
Japan	16	14	520
Netherlands	13	13	440
Australia	15	14	480
France	15	12	510
Russia	10	10	510
Hungary	13	13	430
Kazakhstan	13	12	505
Great Britain	15	15	465
Great Britain	11	10	480
Germany	16	14	480
Romania	11	10	450
Russia	9	7	365
Australia	16	16	390
Russia	16	12	445
Indonesia	20	19	365
Netherlands	14	13	345
Japan	9	9	360
Argentina	10	9	410
Italy	11	6	400
Russia	10	9	355
Slovakia	17	17	375
Russia	10	12	360
Hungary	13	13	340
USA	16	14	330
Italy	14	13	380
Slovakia	11	11	360
Poland	13	12	360
Hong Kong, China	13	13	370
Belarus	11	11	320
South Africa	12	12	350
Russia	10	10	350
USA	11	9	340
Japan	12	11	275
Hungary	13	12	360
Croatia	15	15	315
Czechia	12	12	310
Czechia	17	17	320
France	15	14	280

Russia	20	16	290
South Africa	18	18	260
Czechia	17	15	280
Serbia	17	16	300
Peru	14	13	320
Australia	12	12	240
Morocco	12	12	285
Thailand	17	16	285
Poland	7	5	310
Russia	11	11	245
Bulgaria	17	14	310
Mexico	16	15	290
Great Britain	21	19	260
Sweden	14	11	245
Argentina	12	10	270
Italy	12	10	260
Great Britain	14	11	250
Great Britain	8	8	180
USA	15	14	250
Belgium	8	6	180
Thailand	14	12	220
Canada	18	16	190
Romania	13	13	200
Canada	8	8	230
Chile	16	13	180
Canada	7	7	200
USA	22	19	150
Mexico	18	18	160
Great Britain	12	10	175
Dominican Republic	12	10	170
Denmark	19	19	140
Great Britain	8	6	120
India	6	5	110
Great Britain	15	13	130
USA	13	7	120
Chinese Taipei	8	7	65
Poland	6	6	970
Russia	7	4	960
Slovakia	11	11	870
USA	10	9	820
Russia	7	7	710
Russia	11	11	720
Japan	14	14	660
Russia	13	12	640
Austria	17	17	670
USA	9	9	690
Serbia	16	15	665
Georgia	12	12	535
Israel	16	14	590
Thailand	16	14	560

Poland	11	10	555
Belgium	12	10	580
Russia	25	25	490
Portugal	11	10	590
USA	8	6	510
USA	13	13	490
Chinese Taipei	12	12	510
Romania	12	12	480
Romania	13	12	500
Slovakia	14	14	445
China, P.R.	11	11	455
France	9	9	520
Netherlands	12	10	450
Argentina	17	17	360
Liechtenstein	11	8	390
USA	14	14	420
Great Britain	12	12	390
USA	7	7	450
Argentina	19	19	340
Australia	18	18	340
Australia	8	8	280
Great Britain	19	16	380
Slovakia	11	11	275
Slovakia	14	14	370
France	10	10	370
Australia	8	7	330
Romania	17	15	340
USA	15	15	290
Sweden	14	13	300
Ukraine	17	17	280
Romania	16	16	270
Japan	12	12	300
Japan	16	14	230
Russia	14	13	260
Morocco	19	17	265
Korea, Rep.	9	7	290
Australia	16	15	260
Russia	14	14	260
Germany	12	11	260
France	8	6	280
Mexico	6	6	220
South Africa	14	14	250
Russia	15	14	250
Great Britain	11	11	255
USA	9	9	165
USA	11	9	255
Japan	12	11	255
South Africa	16	14	250
USA	15	14	240
Mexico	20	20	230

Ukraine	17	17	200
Ecuador	11	10	200
Canada	16	13	240
Australia	9	7	230
Israel	18	17	220
Austria	18	14	210
Australia	12	9	220
Russia	13	12	190
Russia	15	13	170
Italy	12	11	195
Russia	19	16	180
Dominican Republic	18	16	190
Mexico	19	17	190
Argentina	17	14	180
India	22	17	180
Sweden	18	16	170
Great Britain	15	12	135
Italy	23	18	105
Brazil	7	6	90
Brazil	8	6	40
Russia	11	9	1630
Denmark	7	6	1060
Belarus	13	13	930
Japan	13	7	840
Canada	9	8	580
Ukraine	9	9	680
USA	19	16	670
Poland	7	7	650
Romania	13	12	600
Austria	17	16	710
Czechia	19	19	610
Australia	18	18	630
Austria	6	5	640
Brazil	13	13	560
France	6	6	570
USA	14	12	560
Russia	11	11	540
Romania	5	5	480
Slovakia	13	13	485
Italy	12	11	530
Germany	18	15	510
Romania	9	9	440
Brazil	11	11	460
USA	14	13	460
Russia	12	10	475
Australia	16	16	425
Bolivia	10	9	495
Great Britain	9	8	475
Russia	11	11	360
Mexico	18	18	380

Slovakia	15	14	345
Croatia	14	10	440
Kazakhstan	9	9	390
USA	14	13	380
Russia	18	17	375
Czechia	13	13	325
Belgium	11	9	390
USA	16	15	365
Netherlands	9	9	320
Chinese Taipei	13	12	300
Slovakia	14	14	350
Belgium	12	11	345
Kyrgyzstan	12	12	340
Brazil	14	14	320
USA	11	8	350
Russia	8	6	360
Japan	23	19	300
Hong Kong, China	13	13	330
Uzbekistan	15	15	320
Russia	8	8	280
Slovakia	15	13	330
USA	11	11	320
Germany	14	11	320
Russia	15	15	250
Netherlands	10	10	270
Philippines	18	18	290
Czechia	15	15	255
Hong Kong, China	14	14	280
Argentina	10	8	300
USA	7	6	280
Austria	13	13	230
Australia	15	14	220
USA	7	7	245
India	8	8	240
USA	13	12	300
Serbia	9	7	255
Great Britain	19	19	200
Netherlands	6	4	230
Slovakia	12	10	210
Japan	16	11	220
Italy	8	6	270
USA	13	12	270
USA	15	14	250
USA	13	12	210
USA	6	4	250
Japan	14	11	160
USA	11	10	155
Germany	18	15	190
Bosnia and Herzegov	15	12	170
Japan	13	11	140

Italy	10	9	170
Japan	9	6	130
Romania	22	19	155
Venezuela	10	9	160
Finland	8	7	170
USA	14	13	140
Israel	18	13	120
Dominican Republic	13	11	115
USA	14	14	80
Great Britain	22	18	90
Germany	17	14	50
Belarus	6	5	1800
Hungary	5	5	1040
Poland	12	12	1010
Denmark	17	17	870
Slovakia	14	13	860
Romania	16	14	810
Romania	14	13	800
USA	11	11	760
Chinese Taipei	12	12	650
Romania	17	16	690
Russia	10	9	610
Japan	12	12	640
Canada	6	5	630
USA	11	11	560
Netherlands	14	12	540
Canada	9	9	530
Romania	16	16	560
Kazakhstan	11	10	590
Kazakhstan	16	16	520
New Zealand	7	6	450
Austria	8	8	520
Czechia	9	9	350
Chinese Taipei	9	9	460
USA	11	10	460
Russia	11	11	425
New Zealand	18	11	490
Russia	7	6	440
Canada	16	14	460
Brazil	14	14	410
USA	12	12	400
Netherlands	14	14	420
USA	18	15	430
Czechia	18	18	350
Russia	10	10	405
Italy	13	11	425
Italy	8	7	405
Argentina	13	13	365
Ukraine	12	12	350
USA	12	12	320

USA	14	13	380
Russia	14	14	380
Netherlands	13	12	330
Poland	13	13	300
Chile	15	14	340
France	12	10	330
Japan	15	13	260
Russia	11	10	310
Russia	18	16	330
Australia	16	11	310
USA	25	25	300
Netherlands	16	13	295
Slovakia	8	7	310
Brazil	19	19	295
Germany	13	10	295
Great Britain	18	18	265
USA	16	13	275
Romania	14	14	220
Slovenia	12	9	270
Sweden	8	7	260
Bahamas	20	19	270
Brazil	18	17	230
Russia	9	8	250
Germany	8	4	280
Romania	8	8	210
Bolivia	13	13	230
USA	18	18	230
Russia	6	5	240
USA	16	14	210
Czechia	17	15	190
USA	17	16	220
USA	12	11	185
USA	14	13	185
Great Britain	11	8	190
Australia	12	10	180
Serbia	15	10	170
USA	9	8	140
Egypt	9	6	170
USA	8	7	190
Slovenia	16	13	140
Japan	20	18	100
Canada	8	7	110
USA	9	8	60
Netherlands	10	9	1140
Ukraine	12	8	1070
Israel	8	7	1030
Belarus	13	13	750
Chinese Taipei	12	11	710
Romania	15	15	710
New Zealand	18	18	560

Belarus	13	12	640
Russia	11	10	620
Chinese Taipei	17	17	615
USA	18	18	630
Hungary	12	11	615
Switzerland	8	8	620
Slovakia	20	18	620
Canada	12	10	590
Denmark	14	13	600
Romania	11	10	470
Russia	8	6	500
Russia	5	5	450
Thailand	15	15	450
Russia	17	10	465
Russia	17	16	430
USA	15	14	415
Czechia	14	13	375
Italy	13	12	400
Slovakia	16	15	385
Kazakhstan	12	11	360
Netherlands	13	12	315
China, P.R.	10	10	320
Bolivia	20	19	350
Romania	19	19	380
Japan	13	12	390
USA	17	14	340
Germany	8	7	355
Russia	13	11	375
Argentina	13	13	320
Ecuador	17	17	340
Slovenia	9	9	370
Germany	12	12	330
Slovenia	15	11	325
Poland	9	8	340
USA	15	15	270
Italy	12	10	320
Serbia	18	13	350
Sweden	15	13	355
Brazil	9	9	350
USA	10	10	330
Czechia	16	16	280
Germany	16	16	335
France	16	15	295
USA	15	13	300
Germany	16	15	280
Czechia	9	7	240
Serbia	19	18	280
Poland	14	13	280
Japan	14	13	285
Canada	11	11	295

Russia	12	12	250
USA	11	11	240
Australia	13	9	260
Netherlands	13	13	260
Brazil	16	15	245
Canada	12	10	275
Russia	9	7	290
India	14	12	280
Chile	18	17	205
Australia	13	10	230
Canada	6	5	240
Canada	17	15	255
Slovakia	17	17	220
Australia	17	16	230
Croatia	9	8	260
Canada	15	14	240
Argentina	8	8	210
USA	14	12	200
USA	17	14	200
Australia	13	12	180
USA	17	14	185
Canada	16	13	180
USA	13	11	150
Poland	19	15	170
Great Britain	15	14	180
USA	9	8	180
USA	18	17	170
Brazil	15	13	160
USA	15	12	185
Canada	12	8	140
France	10	9	155
China, P.R.	10	7	130
USA	16	13	140
USA	22	22	125
USA	15	13	110
USA	17	14	140
Brazil	10	8	120
Russia	15	14	85
USA	8	8	50
Norway	9	5	2990,00
Netherlands	9	7	2680,00
USA	8	6	2540,00
Spain	7	6	2680,00
Japan	5	5	2150,00
Czechia	15	13	1890,00
Romania	10	8	1970,00
Great Britain	16	13	1750,00
Czechia	10	9	1620,00
Korea, Rep.	19	16	1490,00
Colombia	19	19	1520,00

France	16	14	1470,00
Australia	15	9	1570,00
Kazakhstan	19	19	1320,00
Japan	15	15	1470,00
Spain	19	19	1490,00
Korea, Rep.	18	17	1410,00
France	12	11	1380,00
USA	15	14	1460,00
France	17	16	1260,00
USA	19	15	1180,00
Great Britain	18	17	1280,00
Czechia	12	12	1170,00
USA	15	13	960,00
Finland	24	21	1200,00
Switzerland	16	15	1140,00
Serbia	10	11	1190,00
USA	14	13	1165,00
Morocco	11	8	1240,00
Russia	22	19	760,00
USA	21	20	720,00
Switzerland	14	12	1010,00
Germany	13	13	770,00
Italy	14	14	1020,00
Czechia	20	19	770,00
France	17	14	1080,00
Sweden	29	24	950,00
Turkiye	12	12	950,00
Serbia	20	17	930,00
USA	11	11	620,00
Bulgaria	15	15	850,00
USA	19	17	790,00
USA	17	16	860,00
Cyprus	19	19	780,00
Brazil	17	17	730,00
Spain	16	16	770,00
Argentina	16	13	760,00
Germany	8	6	800,00
Argentina	12	12	675,00
Germany	21	16	730,00
USA	20	16	650,00
Romania	21	13	696,00
Germany	20	15	710,00
USA	21	16	710,00
India	20	17	700,00
Italy	13	11	700,00
Italy	20	18	660,00
Ukraine	25	20	700,00
Italy	17	16	670,00
Brazil	14	11	680,00
Czechia	17	11	541,00

Armenia	21	19	590,00
Sweden	23	17	660,00
Morocco	18	13	680,00
USA	14	12	660,00
Spain	18	13	680,00
Bulgaria	13	10	670,00
Korea, Rep.	17	13	580,00
Ukraine	15	13	630,00
Czechia	21	19	480,00
Russia	19	17	570,00
Great Britain	13	11	690,00
USA	23	20	560,00
Tunisia	18	16	546,00
Russia	19	16	536,00
Spain	18	17	520,00
Great Britain	17	15	300,00
Germany	11	8	535,00
Nicaragua	21	11	470,00
Germany	23	20	432,00
Brazil	24	21	525,00
USA	13	11	502,00
Guatemala	20	17	456,00
Korea, Rep.	20	19	440,00
China, P.R.	21	17	425,00
USA	21	17	440,00
USA	19	15	456,00
USA	9	8	430,00
Kazakhstan	13	9	430,00
Canada	8	8	445,00
Bolivia	19	15	472,00
Australia	17	12	420,00
China, P.R.	16	14	366,00
Spain	14	11	391,00
Brazil	14	13	380,00
Italy	25	14	368,00
Romania	14	10	345,00
Australia	7	6	380,00
Germany	10	8	270,00
Poland	23	18	304,00
Romania	11	8	385,00
USA	30	26	273,00
USA	20	15	230,00
Singapore	18	14	264,00
Spain	12	11	252,00
Peru	11	7	265,00
Spain	10	7	245,00
USA	26	17	162,00
Czechia	19	10	178,00
Slovenia	15	7	18,00

ormance percentiles, respectively, based on year-end Total Ranking Points (TRP) distribu
tile threshold in a given year, and a value of 0 indicates that the player did not reach the
ıs to the international junior competition calendar caused by the COVID-19 pandemic, re

ation (ITF) and were analysed in anonymised and aggregated form.

Total Doubles Points	TRP	Year
1409	2902,25	2023
2009	2852,25	2023
1762	2750,5	2023
2091	2282,75	2023
1725	2191,25	2023
1027	2136,75	2023
1499	2049,75	2023
876	2019	2023
1019	1994,75	2023
816	1934	2023
944	1906	2023
877	1869,25	2023
1109	1707,25	2023
727	1666,75	2023
1012	1643	2023
1792	1638	2023
1341	1515,25	2023
1724	1511	2023
989	1487,25	2023
1177	1424,25	2023
569	1402,25	2023
749	1367,25	2023
930	1317,5	2023
1190	1317,5	2023
1049	1292,25	2023
1251	1232,75	2023
1964	1231	2023
869	1227,25	2023
1567	1191,75	2023
1072	1188	2023
1065	1186,25	2023
1161	1180,25	2023
922	1170,5	2023
1642	1155,5	2023
524	1151	2023
1190	1147,5	2023
1364	1131	2023
750	1127,5	2023
1536	1104	2023
794	1078,5	2023
225	1076,25	2023
532	1063	2023
929	1062,25	2023
1154	1048,5	2023
816	1024	2023
734	1013,5	2023

	757	1009,25	2023
	989	1007,25	2023
	862	985,5	2023
	412	963	2023
	929	962,25	2023
	727	961,75	2023
	921	960,25	2023
	472	958	2023
	802	950,5	2023
	1026	936,5	2023
	981	925,25	2023
	682	915,5	2023
	299	914,75	2023
	779	884,75	2023
	599	884,75	2023
	1124	871	2023
	1116	859	2023
	472	848	2023
	251	832,75	2023
	1042	831,5	2023
	922	830,5	2023
	817	814,25	2023
	592	808	2023
	1027	806,75	2023
	465	806,25	2023
	382	785,5	2023
	780	775	2023
	412	763	2023
	652	763	2023
	674	759,5	2023
	697	759,25	2023
	989	757,25	2023
	929	742,25	2023
	877	739,25	2023
	457	736,25	2023
	809	722,25	2023
	517	719,25	2023
	0	681	2023
	420	675	2023
	456	664	2023
	630	658,5	2023
	300	645	2023
	495	633,75	2023
	547	632,75	2023
	607	631,75	2023
	509	627,25	2023
	510	623,5	2023
	574	623,5	2023
	974	613,5	2023
	622	595,5	2023

	315	588,75	2023
	494	578,5	2023
	539	574,75	2023
	765	567,25	2023
	450	562,5	2023
	585	552,25	2023
	464	552	2023
	510	549,5	2023
	426	546,5	2023
	330	523,5	2023
	382	515,5	2023
	569	502,25	2023
	382	495,5	2023
	202	486,5	2023
	162	481,5	2023
	487	465,75	2023
	330	444,5	2023
	487	441,75	2023
	300	403	2023
	292	373	2023
	509	362,25	2023
	644	351	2023
	330	334,5	2023
	364	298	2023
	330	266,5	2023
	150	232,5	2023
	234	128,5	2023
	184	124	2023
	682	2610,5	2022
	735	2583,75	2022
	861	2415,25	2022
	1072	2198	2022
	1432	2058	2022
	937	1994,25	2022
	1146	1786,5	2022
	1462	1645,5	2022
	1634	1618,5	2022
	696	1554	2022
	529	1542,25	2022
	525	1461,25	2022
	854	1403,5	2022
	1380	1385	2022
	545	1351,25	2022
	1537	1334,25	2022
	672	1328	2022
	1462	1320,5	2022
	621	1315,25	2022
	562	1300,5	2022
	1266	1296,5	2022
	877	1289,25	2022

	367	1271,75	2022
	1169	1242,25	2022
	982	1235,5	2022
	570	1222,5	2022
	779	1204,75	2022
	1664	1196	2022
	1462	1185,5	2022
	779	1184,75	2022
	1026	1176,5	2022
	1124	1151	2022
	847	1141,75	2022
	1320	1140	2022
	651	1132,75	2022
	509	1127,25	2022
	922	1120,5	2022
	936	1114	2022
	810	1052,5	2022
	847	1041,75	2022
	404	1031	2022
	1349	1027,25	2022
	1275	988,75	2022
	741	975,25	2022
	1078	969,5	2022
	1192	968	2022
	442	960,5	2022
	1011	952,75	2022
	660	945	2022
	808	902	2022
	1582	895,5	2022
	681	880,25	2022
	585	876,25	2022
	704	876	2022
	592	848	2022
	487	837,75	2022
	622	835,5	2022
	487	811,75	2022
	562	810,5	2022
	674	808,5	2022
	779	784,75	2022
	630	777,5	2022
	764	751	2022
	502	746,5	2022
	539	734,75	2022
	817	734,25	2022
	494	733,5	2022
	1402	696,5	2022
	629	687,25	2022
	981	685,25	2022
	682	680,5	2022
	382	675,5	2022

	600	670	2022
	899	665,75	2022
	622	660,5	2022
	742	657,5	2022
	469	657,25	2022
	495	643,75	2022
	525	641,25	2022
	704	641	2022
	457	630,25	2022
	390	627,5	2022
	517	625,25	2022
	532	613	2022
	727	611,75	2022
	300	601	2022
	749	587,25	2022
	741	586,25	2022
	854	583,5	2022
	892	578	2022
	585	562,25	2022
	765	561,25	2022
	547	556,75	2022
	419	554,75	2022
	464	542	2022
	540	535	2022
	614	534,5	2022
	502	531,5	2022
	487	521,75	2022
	540	503	2022
	943	491,75	2022
	772	488	2022
	307	472,75	2022
	449	462,25	2022
	480	433	2022
	375	430,75	2022
	491	408,75	2022
	340	405	2022
	292	323	2022
	292	299	2022
	150	267,5	2022
	532	255	2022
	330	208,5	2022
	165	151,25	2022
	607	2741,75	2021
	1170	2502,5	2021
	1034	2178,5	2021
	951	1857,75	2021
	1484	1811	2021
	1191	1727,75	2021
	1033	1648,25	2021
	854	1623,5	2021

	780	1525	2021
	547	1506,75	2021
	569	1502,25	2021
	465	1476,25	2021
	630	1437,5	2021
	1191	1417,75	2021
	764	1411	2021
	845	1241,25	2021
	1350	1217,5	2021
	284	1211	2021
	2100	1205	2021
	495	1163,75	2021
	795	1158,75	2021
	525	1121,25	2021
	877	1119,25	2021
	1161	1110,25	2021
	337	1104,25	2021
	795	1088,75	2021
	487	1081,75	2021
	516	1079	2021
	472	1078	2021
	1041	1050,25	2021
	300	1045	2021
	1312	1038	2021
	1319	1029,75	2021
	1147	1006,75	2021
	877	999,25	2021
	1266	996,5	2021
	225	966,25	2021
	1140	965	2021
	952	938	2021
	562	910,5	2021
	810	902,5	2021
	597	899,25	2021
	435	898,75	2021
	952	888	2021
	660	885	2021
	907	882,75	2021
	502	880,5	2021
	795	868,75	2021
	409	862,25	2021
	502	855,5	2021
	862	830,5	2021
	1327	827,75	2021
	1575	818,75	2021
	472	818	2021
	689	792,25	2021
	876	789	2021
	356	789	2021
	779	776,75	2021

	802	760,5	2021
	509	757,25	2021
	915	744,75	2021
	517	744,25	2021
	749	727,25	2021
	667	726,75	2021
	779	724,75	2021
	210	722,5	2021
	392	718	2021
	670	707,5	2021
	1140	705	2021
	974	699,5	2021
	645	697,25	2021
	294	693,5	2021
	682	692,5	2021
	831	688,75	2021
	225	682,25	2021
	765	671,25	2021
	150	663,5	2021
	480	662	2021
	847	657,75	2021
	163	650,75	2021
	629	649,25	2021
	712	648	2021
	615	643,75	2021
	686	641,5	2021
	540	641	2021
	1005	617,25	2021
	750	613,5	2021
	525	611,25	2021
	472	610	2021
	652	609	2021
	337	604,25	2021
	129	588,25	2021
	324	585	2021
	342	581,5	2021
	622	580,5	2021
	562	580,5	2021
	382	579,5	2021
	390	577,5	2021
	420	575	2021
	360	575	2021
	420	575	2021
	615	566,75	2021
	420	561	2021
	477	559,25	2021
	524	556	2021
	494	553,5	2021
	630	553,5	2021
	420	545	2021

	570	542,5	2021
	915	538,75	2021
	690	532,5	2021
	765	528,25	2021
	704	528	2021
	562	520,5	2021
	532	518	2021
	547	517,75	2021
	147	516,75	2021
	547	516,75	2021
	487	513,75	2021
	223	511,75	2021
	630	509,5	2021
	1041	509,25	2021
	921	506,25	2021
	394	498,5	2021
	330	498,5	2021
	457	491,25	2021
	180	491	2021
	652	491	2021
	242	485,5	2021
	438	479,5	2021
	411	470,75	2021
	229	467,25	2021
	522	458,5	2021
	412	445	2021
	357	441,25	2021
	354	440,5	2021
	532	439	2021
	465	438,25	2021
	352	433	2021
	330	426,5	2021
	308	425	2021
	330	422,5	2021
	435	420,75	2021
	794	418,5	2021
	249	418,25	2021
	264	418	2021
	311	413,75	2021
	203	412,75	2021
	214	411,5	2021
	420	411	2021
	351	407,75	2021
	178	406,5	2021
	472	406	2021
	237	405,25	2021
	349	405,25	2021
	412	403	2021
	382	401,5	2021
	293	401,25	2021

	472	398	2021
	532	395	2021
	180	395	2021
	324	393	2021
	600	392	2021
	232	390	2021
	294	389,5	2021
	205	387,25	2021
	360	376	2021
	216	372	2021
	210	370,5	2021
	125	367,25	2021
	200	366	2021
	150	365,5	2021
	134	365,5	2021
	172	365	2021
	510	364,5	2021
	346	358,5	2021
	420	353	2021
	252	351	2021
	472	349	2021
	532	348	2021
	294	345,5	2021
	614	344,5	2021
	450	344,5	2021
	163	340,75	2021
	334	337,5	2021
	192	336	2021
	214	335,5	2021
	324	333	2021
	300	331	2021
	424	330	2021
	169	327,25	2021
	156	321	2021
	0	320	2021
	208	320	2021
	424	320	2021
	198	311,5	2021
	342	311,5	2021
	381	311,25	2021
	268	308	2021
	194	306,5	2021
	189	305,25	2021
	256	305	2021
	124	303	2021
	162	302,5	2021
	360	302	2021
	117	297,25	2021
	495	289,75	2021
	174	288,5	2021

	234	288,5	2021
	222	287,5	2021
	145	286,25	2021
	194	284,5	2021
	325	283,25	2021
	232	282	2021
	165	281,25	2021
	117	279,25	2021
	71	277,75	2021
	291	274,75	2021
	274	274,5	2021
	367	272,75	2021
	251	270,75	2021
	270	267,5	2021
	170	266,5	2021
	246	261,5	2021
	161	260,25	2021
	312	260	2021
	131	258,75	2021
	234	258,5	2021
	226	257,5	2021
	202	256,5	2021
	465	256,25	2021
	240	256	2021
	116	255	2021
	234	254,5	2021
	169	254,25	2021
	159	250,75	2021
	262	250,5	2021
	232	250	2021
	356	249	2021
	119	247,75	2021
	49	247,25	2021
	131	246,75	2021
	119	245,75	2021
	253	245,25	2021
	291	244,75	2021
	282	244,5	2021
	192	244	2021
	270	243,5	2021
	142	243,5	2021
	133	243,25	2021
	245	243,25	2021
	294	241,5	2021
	101	241,25	2021
	282	240,5	2021
	196	239	2021
	186	238,5	2021
	342	237,5	2021
	130	236,5	2021

	47	235,75	2021
	327	235,75	2021
	137	235,25	2021
	450	233,5	2021
	405	233,25	2021
	324	233	2021
	120	232	2021
	181	230,25	2021
	241	228,25	2021
	196	227	2021
	354	226,5	2021
	204	225	2021
	196	225	2021
	232	224	2021
	367	223,75	2021
	99	223,75	2021
	240	222	2021
	131	220,75	2021
	191	219,75	2021
	135	219,75	2021
	150	219,5	2021
	252	217	2021
	215	216,75	2021
	68	216	2021
	198	215,5	2021
	183	214,75	2021
	215	213,75	2021
	138	213,5	2021
	193	212,25	2021
	607	211,75	2021
	159	211,75	2021
	79	211,75	2021
	178	210,5	2021
	312	210	2021
	152	208	2021
	153	206,25	2021
	221	206,25	2021
	97	204,25	2021
	230	203,5	2021
	216	202	2021
	192	202	2021
	170	200,5	2021
	192	200	2021
	52	199	2021
	133	198,25	2021
	210	197,5	2021
	282	196,5	2021
	260	196	2021
	180	195	2021
	156	195	2021

	195	194,75	2021
	194	192,5	2021
	188	191	2021
	176	190	2021
	113	189,25	2021
	184	188	2021
	31	187,75	2021
	450	187,5	2021
	182	187,5	2021
	132	187	2021
	15	184,75	2021
	135	183,75	2021
	183	181,75	2021
	246	181,5	2021
	57	181,25	2021
	390	180,5	2021
	312	179	2021
	144	179	2021
	118	177,5	2021
	36	177	2021
	184	177	2021
	147	176,75	2021
	121	176,25	2021
	79	175,75	2021
	180	175	2021
	164	175	2021
	137	174,25	2021
	191	173,75	2021
	202	173,5	2021
	181	173,25	2021
	52	172	2021
	147	171,75	2021
	144	171	2021
	142	170,5	2021
	112	170	2021
	106	168,5	2021
	116	167	2021
	87	165,75	2021
	142	165,5	2021
	0	165	2021
	316	165	2021
	201	164,25	2021
	128	163	2021
	194	162,5	2021
	170	162,5	2021
	252	162	2021
	190	161,5	2021
	158	161,5	2021
	129	161,25	2021
	222	159,5	2021

	262	158,5	2021
	69	158,25	2021
	97	158,25	2021
	102	157,5	2021
	77	157,25	2021
	192	157	2021
	144	156	2021
	167	155,75	2021
	30	155,5	2021
	182	155,5	2021
	24	155	2021
	174	154,5	2021
	40	154	2021
	166	153,5	2021
	153	153,25	2021
	134	151,5	2021
	173	151,25	2021
	82	150,5	2021
	158	150,5	2021
	113	150,25	2021
	0	150	2021
	144	150	2021
	66	148,5	2021
	214	147,5	2021
	190	147,5	2021
	58	147,5	2021
	208	146	2021
	92	145	2021
	96	145	2021
	266	144,5	2021
	201	143,25	2021
	171	142,75	2021
	171	142,75	2021
	90	142,5	2021
	98	142,5	2021
	41	142,25	2021
	180	142	2021
	159	141,75	2021
	162	141,5	2021
	66	140,5	2021
	61	139,25	2021
	83	138,75	2021
	171	138,75	2021
	94	138,5	2021
	78	138,5	2021
	94	138,5	2021
	100	137	2021
	136	136	2021
	248	136	2021
	123	135,75	2021

	174	135,5	2021
	264	135	2021
	189	134,25	2021
	147	132,75	2021
	182	132,5	2021
	68	130	2021
	86	129,5	2021
	63	128,75	2021
	244	127	2021
	234	126,5	2021
	22	125,5	2021
	282	125,5	2021
	135	123,75	2021
	95	123,75	2021
	142	123,5	2021
	174	122,5	2021
	47	121,75	2021
	47	121,75	2021
	172	121	2021
	98	120,5	2021
	216	120	2021
	113	119,25	2021
	20	119	2021
	57	118,25	2021
	132	118	2021
	130	117,5	2021
	170	117,5	2021
	97	117,25	2021
	184	117	2021
	144	117	2021
	28	117	2021
	71	116,75	2021
	89	116,25	2021
	28	116	2021
	124	116	2021
	68	116	2021
	66	115,5	2021
	118	115,5	2021
	41	115,25	2021
	116	115	2021
	79	114,75	2021
	55	113,75	2021
	39	113,75	2021
	42	113,5	2021
	151	112,75	2021
	207	112,75	2021
	122	112,5	2021
	53	112,25	2021
	196	112	2021
	71	111,75	2021

	110	111,5	2021
	14	111,5	2021
	123	109,75	2021
	86	109,5	2021
	228	109	2021
	83	108,75	2021
	55	108,75	2021
	94	108,5	2021
	182	108,5	2021
	17	108,25	2021
	37	108,25	2021
	115	107,75	2021
	95	107,75	2021
	93	107,25	2021
	48	107	2021
	122	106,5	2021
	89	106,25	2021
	84	106	2021
	171	105,75	2021
	47	105,75	2021
	70	105,5	2021
	105	105,25	2021
	108	105	2021
	30	104,5	2021
	130	104,5	2021
	64	103	2021
	26	102,5	2021
	141	102,25	2021
	87	101,75	2021
	119	101,75	2021
	87	101,75	2021
	135	101,75	2021
	118	101,5	2021
	45	101,25	2021
	104	101	2021
	120	100	2021
	120	100	2021
	115	99,75	2021
	58	99,5	2021
	105	99,25	2021
	13	99,25	2021
	95	98,75	2021
	50	98,5	2021
	30	98,5	2021
	92	98	2021
	80	98	2021
	151	97,75	2021
	151	97,75	2021
	90	97,5	2021
	90	97,5	2021

	98	97,5	2021
	201	97,25	2021
	79	96,75	2021
	35	96,75	2021
	62	96,5	2021
	98	96,5	2021
	161	96,25	2021
	117	96,25	2021
	172	96	2021
	35	95,75	2021
	15	95,75	2021
	122	95,5	2021
	125	95,25	2021
	104	95	2021
	114	94,5	2021
	9	94,25	2021
	60	94	2021
	79	93,75	2021
	199	93,75	2021
	74	93,5	2021
	110	93,5	2021
	89	93,25	2021
	89	93,25	2021
	29	93,25	2021
	112	93	2021
	144	93	2021
	16	92	2021
	84	92	2021
	74	91,5	2021
	145	91,25	2021
	116	91	2021
	128	91	2021
	43	90,75	2021
	118	90,5	2021
	22	90,5	2021
	13	90,25	2021
	181	90,25	2021
	180	90	2021
	32	90	2021
	198	89,5	2021
	82	89,5	2021
	65	89,25	2021
	237	89,25	2021
	133	89,25	2021
	25	89,25	2021
	143	88,75	2021
	104	88	2021
	39	87,75	2021
	163	87,75	2021
	78	87,5	2021

	58	87,5	2021
	93	87,25	2021
	88	87	2021
	108	87	2021
	68	87	2021
	19	86,75	2021
	93	86,25	2021
	153	86,25	2021
	24	86	2021
	94	85,5	2021
	82	85,5	2021
	64	85	2021
	171	84,75	2021
	99	84,75	2021
	86	84,5	2021
	141	84,25	2021
	161	84,25	2021
	84	84	2021
	23	83,75	2021
	19	83,75	2021
	94	83,5	2021
	10	83,5	2021
	89	83,25	2021
	113	83,25	2021
	73	83,25	2021
	48	83	2021
	108	83	2021
	10	82,5	2021
	90	82,5	2021
	134	82,5	2021
	32	82	2021
	112	82	2021
	63	81,75	2021
	123	81,75	2021
	87	81,75	2021
	91	81,75	2021
	42	81,5	2021
	102	81,5	2021
	81	81,25	2021
	65	81,25	2021
	80	81	2021
	83	80,75	2021
	95	80,75	2021
	115	80,75	2021
	119	80,75	2021
	126	80,5	2021
	81	80,25	2021
	56	80	2021
	28	80	2021
	16	80	2021

	168	80	2021
	87	79,75	2021
	66	79,5	2021
	22	79,5	2021
	110	79,5	2021
	138	79,5	2021
	170	79,5	2021
	37	79,25	2021
	56	79	2021
	83	78,75	2021
	83	78,75	2021
	23	78,75	2021
	159	78,75	2021
	82	78,5	2021
	166	78,5	2021
	58	78,5	2021
	16	78	2021
	40	78	2021
	130	77,5	2021
	80	77	2021
	27	76,75	2021
	43	76,75	2021
	110	76,5	2021
	25	76,25	2021
	21	76,25	2021
	36	76	2021
	128	76	2021
	55	75,75	2021
	99	75,75	2021
	102	75,5	2021
	10	75,5	2021
	222	75,5	2021
	74	75,5	2021
	68	75	2021
	140	75	2021
	72	75	2021
	59	74,75	2021
	66	74,5	2021
	14	74,5	2021
	53	74,25	2021
	45	74,25	2021
	44	74	2021
	204	74	2021
	15	73,75	2021
	51	73,75	2021
	52	73	2021
	40	73	2021
	58	72,5	2021
	85	72,25	2021
	120	72	2021

	76	72	2021
	120	72	2021
	7	71,75	2021
	95	71,75	2021
	163	71,75	2021
	73	71,25	2021
	64	71	2021
	84	71	2021
	56	71	2021
	115	70,75	2021
	19	70,75	2021
	36	70	2021
	123	69,75	2021
	119	69,75	2021
	138	69,5	2021
	157	69,25	2021
	169	69,25	2021
	105	69,25	2021
	97	69,25	2021
	105	69,25	2021
	101	69,25	2021
	28	69	2021
	100	69	2021
	75	68,75	2021
	91	68,75	2021
	135	68,75	2021
	73	68,25	2021
	177	68,25	2021
	53	68,25	2021
	136	68	2021
	31	67,75	2021
	150	67,5	2021
	150	67,5	2021
	90	67,5	2021
	77	67,25	2021
	65	67,25	2021
	47	66,75	2021
	115	66,75	2021
	43	66,75	2021
	59	66,75	2021
	82	66,5	2021
	26	66,5	2021
	108	66	2021
	0	66	2021
	148	66	2021
	83	65,75	2021
	83	65,75	2021
	54	65,5	2021
	45	65,25	2021
	57	65,25	2021

	9	65,25	2021
	79	64,75	2021
	151	64,75	2021
	26	64,5	2021
	64	64	2021
	36	64	2021
	116	64	2021
	15	63,75	2021
	0	63	2021
	59	62,75	2021
	110	62,5	2021
	66	62,5	2021
	93	62,25	2021
	44	62	2021
	107	61,75	2021
	39	61,75	2021
	58	61,5	2021
	34	61,5	2021
	109	61,25	2021
	56	61	2021
	67	60,75	2021
	163	60,75	2021
	162	60,5	2021
	110	60,5	2021
	21	60,25	2021
	13	60,25	2021
	0	60	2021
	44	60	2021
	67	59,75	2021
	83	59,75	2021
	28	59	2021
	16	59	2021
	107	58,75	2021
	115	58,75	2021
	63	58,75	2021
	94	58,5	2021
	25	58,25	2021
	37	58,25	2021
	32	58	2021
	83	57,75	2021
	99	57,75	2021
	70	57,5	2021
	41	57,25	2021
	56	57	2021
	108	57	2021
	16	57	2021
	67	56,75	2021
	99	56,75	2021
	38	56,5	2021
	37	56,25	2021

	65	56,25	2021
	53	56,25	2021
	21	56,25	2021
	0	56	2021
	76	56	2021
	156	56	2021
	48	56	2021
	44	56	2021
	91	55,75	2021
	31	55,75	2021
	111	55,75	2021
	115	55,75	2021
	90	55,5	2021
	77	55,25	2021
	40	55	2021
	75	54,75	2021
	31	54,75	2021
	59	54,75	2021
	18	54,5	2021
	82	54,5	2021
	34	54,5	2021
	26	54,5	2021
	106	54,5	2021
	50	54,5	2021
	25	54,25	2021
	60	54	2021
	0	54	2021
	55	53,75	2021
	55	53,75	2021
	43	53,75	2021
	59	53,75	2021
	34	53,5	2021
	50	53,5	2021
	86	53,5	2021
	158	53,5	2021
	21	53,25	2021
	41	53,25	2021
	101	53,25	2021
	65	53,25	2021
	49	53,25	2021
	44	53	2021
	72	53	2021
	60	53	2021
	156	53	2021
	67	52,75	2021
	31	52,75	2021
	19	52,75	2021
	123	52,75	2021
	119	52,75	2021
	19	52,75	2021

	90	52,5	2021
	86	52,5	2021
	69	52,25	2021
	97	52,25	2021
	0	52	2021
	67	51,75	2021
	23	51,75	2021
	66	51,5	2021
	38	51,5	2021
	40	51	2021
	52	51	2021
	95	50,75	2021
	34	50,5	2021
	90	50,5	2021
	45	50,25	2021
	0	50	2021
	52	50	2021
	20	50	2021
	76	50	2021
	20	50	2021
	55	49,75	2021
	75	49,75	2021
	51	49,75	2021
	90	49,5	2021
	102	49,5	2021
	102	49,5	2021
	37	49,25	2021
	96	49	2021
	60	49	2021
	39	48,75	2021
	39	48,75	2021
	10	48,5	2021
	50	48,5	2021
	97	48,25	2021
	28	48	2021
	55	47,75	2021
	39	47,75	2021
	43	47,75	2021
	70	47,5	2021
	142	47,5	2021
	82	47,5	2021
	62	47,5	2021
	61	47,25	2021
	53	47,25	2021
	48	47	2021
	40	47	2021
	36	47	2021
	12	47	2021
	67	46,75	2021
	83	46,75	2021

	67	46,75	2021
	19	46,75	2021
	6	46,5	2021
	18	46,5	2021
	29	46,25	2021
	89	46,25	2021
	12	46	2021
	84	46	2021
	67	45,75	2021
	23	45,75	2021
	71	45,75	2021
	19	45,75	2021
	22	45,5	2021
	42	45,5	2021
	38	45,5	2021
	82	45,5	2021
	61	45,25	2021
	25	45,25	2021
	84	45	2021
	0	45	2021
	64	45	2021
	31	44,75	2021
	31	44,75	2021
	35	44,75	2021
	26	44,5	2021
	58	44,5	2021
	78	44,5	2021
	17	44,25	2021
	89	44,25	2021
	45	44,25	2021
	73	44,25	2021
	81	44,25	2021
	101	44,25	2021
	32	44	2021
	80	44	2021
	12	44	2021
	40	44	2021
	15	43,75	2021
	39	43,75	2021
	3	43,75	2021
	19	43,75	2021
	26	43,5	2021
	38	43,5	2021
	50	43,5	2021
	73	43,25	2021
	45	43,25	2021
	52	43	2021
	36	43	2021
	27	42,75	2021
	102	42,5	2021

	38	42,5	2021
	18	42,5	2021
	49	42,25	2021
	25	42,25	2021
	101	42,25	2021
	101	42,25	2021
	72	42	2021
	112	42	2021
	12	42	2021
	76	42	2021
	31	41,75	2021
	39	41,75	2021
	119	41,75	2021
	14	41,5	2021
	54	41,5	2021
	54	41,5	2021
	21	41,25	2021
	45	41,25	2021
	0	41	2021
	24	41	2021
	16	41	2021
	59	40,75	2021
	23	40,75	2021
	83	40,75	2021
	42	40,5	2021
	58	40,5	2021
	65	40,25	2021
	65	40,25	2021
	60	40	2021
	15	39,75	2021
	3	39,75	2021
	23	39,75	2021
	25	39,25	2021
	89	39,25	2021
	157	39,25	2021
	16	39	2021
	12	39	2021
	3	38,75	2021
	3	38,75	2021
	35	38,75	2021
	59	38,75	2021
	19	38,75	2021
	22	38,5	2021
	46	38,5	2021
	58	38,5	2021
	86	38,5	2021
	14	38,5	2021
	6	38,5	2021
	13	38,25	2021
	69	38,25	2021

	13	38,25	2021
	0	38	2021
	28	38	2021
	56	38	2021
	52	38	2021
	20	38	2021
	16	38	2021
	51	37,75	2021
	66	37,5	2021
	66	37,5	2021
	41	37,25	2021
	101	37,25	2021
	36	37	2021
	56	37	2021
	32	37	2021
	23	36,75	2021
	35	36,75	2021
	47	36,75	2021
	50	36,5	2021
	30	36,5	2021
	22	36,5	2021
	98	36,5	2021
	70	36,5	2021
	6	36,5	2021
	42	36,5	2021
	37	36,25	2021
	29	36,25	2021
	65	36,25	2021
	25	36,25	2021
	0	36	2021
	104	36	2021
	0	36	2021
	40	36	2021
	3	35,75	2021
	27	35,75	2021
	43	35,75	2021
	75	35,75	2021
	3	35,75	2021
	23	35,75	2021
	15	35,75	2021
	91	35,75	2021
	6	35,5	2021
	26	35,5	2021
	42	35,5	2021
	46	35,5	2021
	21	35,25	2021
	65	35,25	2021
	81	35,25	2021
	29	35,25	2021
	53	35,25	2021

	60	35	2021
	60	35	2021
	56	35	2021
	92	35	2021
	0	35	2021
	23	34,75	2021
	79	34,75	2021
	3	34,75	2021
	27	34,75	2021
	27	34,75	2021
	3	34,75	2021
	54	34,5	2021
	62	34,5	2021
	6	34,5	2021
	42	34,5	2021
	22	34,5	2021
	54	34,5	2021
	9	34,25	2021
	17	34,25	2021
	88	34	2021
	36	34	2021
	52	34	2021
	3	33,75	2021
	23	33,75	2021
	67	33,75	2021
	79	33,75	2021
	127	33,75	2021
	135	33,75	2021
	135	33,75	2021
	58	33,5	2021
	58	33,5	2021
	78	33,5	2021
	114	33,5	2021
	45	33,25	2021
	17	33,25	2021
	73	33,25	2021
	13	33,25	2021
	33	33,25	2021
	52	33	2021
	20	33	2021
	48	33	2021
	68	33	2021
	72	33	2021
	27	32,75	2021
	59	32,75	2021
	103	32,75	2021
	27	32,75	2021
	110	32,5	2021
	50	32,5	2021
	17	32,25	2021

	29	32,25	2021
	49	32,25	2021
	45	32,25	2021
	60	32	2021
	20	32	2021
	0	32	2021
	20	32	2021
	0	32	2021
	35	31,75	2021
	23	31,75	2021
	6	31,5	2021
	14	31,5	2021
	62	31,5	2021
	6	31,5	2021
	6	31,5	2021
	13	31,25	2021
	45	31,25	2021
	17	31,25	2021
	17	31,25	2021
	20	31	2021
	20	31	2021
	32	31	2021
	28	31	2021
	75	30,75	2021
	7	30,75	2021
	35	30,75	2021
	35	30,75	2021
	31	30,75	2021
	51	30,75	2021
	31	30,75	2021
	26	30,5	2021
	42	30,5	2021
	74	30,5	2021
	13	30,25	2021
	9	30,25	2021
	13	30,25	2021
	41	30,25	2021
	93	30,25	2021
	69	30,25	2021
	0	30	2021
	0	30	2021
	0	30	2021
	0	30	2021
	0	30	2021
	0	30	2021
	0	30	2021
	44	30	2021
	120	30	2021
	3	29,75	2021
	7	29,75	2021

	39	29,75	2021
	43	29,75	2021
	43	29,75	2021
	55	29,75	2021
	6	29,5	2021
	30	29,5	2021
	30	29,5	2021
	26	29,5	2021
	45	29,25	2021
	68	29	2021
	16	29	2021
	32	29	2021
	48	29	2021
	76	29	2021
	80	29	2021
	55	28,75	2021
	42	28,5	2021
	6	28,5	2021
	10	28,5	2021
	38	28,5	2021
	78	28,5	2021
	21	28,25	2021
	85	28,25	2021
	85	28,25	2021
	16	28	2021
	32	28	2021
	0	28	2021
	44	28	2021
	52	28	2021
	48	28	2021
	35	27,75	2021
	7	27,75	2021
	47	27,75	2021
	47	27,75	2021
	47	27,75	2021
	51	27,75	2021
	87	27,75	2021
	26	27,5	2021
	54	27,5	2021
	74	27,5	2021
	82	27,5	2021
	94	27,5	2021
	33	27,25	2021
	44	27	2021
	0	27	2021
	27	26,75	2021
	31	26,75	2021
	19	26,75	2021
	23	26,75	2021
	26	26,5	2021

	66	26,5	2021
	14	26,5	2021
	14	26,5	2021
	14	26,5	2021
	6	26,5	2021
	10	26,5	2021
	62	26,5	2021
	70	26,5	2021
	25	26,25	2021
	37	26,25	2021
	105	26,25	2021
	105	26,25	2021
	0	26	2021
	28	26	2021
	24	26	2021
	24	26	2021
	0	26	2021
	20	26	2021
	56	26	2021
	56	26	2021
	35	25,75	2021
	55	25,75	2021
	75	25,75	2021
	19	25,75	2021
	42	25,5	2021
	13	25,25	2021
	61	25,25	2021
	33	25,25	2021
	45	25,25	2021
	13	25,25	2021
	49	25,25	2021
	85	25,25	2021
	32	25	2021
	20	25	2021
	0	25	2021
	0	25	2021
	28	25	2021
	40	25	2021
	27	24,75	2021
	27	24,75	2021
	7	24,75	2021
	27	24,75	2021
	31	24,75	2021
	23	24,75	2021
	43	24,75	2021
	51	24,75	2021
	51	24,75	2021
	3	24,75	2021
	23	24,75	2021
	34	24,5	2021

	50	24,5	2021
	6	24,5	2021
	22	24,5	2021
	26	24,5	2021
	21	24,25	2021
	41	24,25	2021
	49	24,25	2021
	41	24,25	2021
	33	24,25	2021
	9	24,25	2021
	20	24	2021
	28	24	2021
	16	24	2021
	20	24	2021
	12	24	2021
	32	24	2021
	56	24	2021
	35	23,75	2021
	27	23,75	2021
	31	23,75	2021
	3	23,75	2021
	47	23,75	2021
	54	23,5	2021
	14	23,5	2021
	14	23,5	2021
	6	23,5	2021
	22	23,5	2021
	22	23,5	2021
	53	23,25	2021
	29	23,25	2021
	37	23,25	2021
	73	23,25	2021
	25	23,25	2021
	0	23	2021
	16	23	2021
	44	23	2021
	28	23	2021
	0	23	2021
	16	23	2021
	16	23	2021
	64	23	2021
	19	22,75	2021
	43	22,75	2021
	31	22,75	2021
	23	22,75	2021
	10	22,5	2021
	42	22,5	2021
	62	22,5	2021
	82	22,5	2021
	90	22,5	2021

	13	22,25	2021
	13	22,25	2021
	61	22,25	2021
	0	22	2021
	0	22	2021
	0	22	2021
	48	22	2021
	28	22	2021
	28	22	2021
	24	22	2021
	60	22	2021
	7	21,75	2021
	7	21,75	2021
	15	21,75	2021
	39	21,75	2021
	39	21,75	2021
	39	21,75	2021
	15	21,75	2021
	10	21,5	2021
	10	21,5	2021
	26	21,5	2021
	45	21,25	2021
	45	21,25	2021
	45	21,25	2021
	45	21,25	2021
	13	21,25	2021
	16	21	2021
	16	21	2021
	28	21	2021
	20	21	2021
	56	21	2021
	48	21	2021
	68	21	2021
	3	20,75	2021
	39	20,75	2021
	55	20,75	2021
	10	20,5	2021
	22	20,5	2021
	14	20,5	2021
	6	20,5	2021
	26	20,5	2021
	34	20,5	2021
	54	20,5	2021
	10	20,5	2021
	13	20,25	2021
	13	20,25	2021
	21	20,25	2021
	21	20,25	2021
	41	20,25	2021
	9	20,25	2021

	0	20	2021
	0	20	2021
	0	20	2021
	0	20	2021
	0	20	2021
	20	20	2021
	32	20	2021
	40	20	2021
	12	20	2021
	36	20	2021
	16	20	2021
	72	20	2021
	35	19,75	2021
	3	19,75	2021
	23	19,75	2021
	23	19,75	2021
	3	19,75	2021
	15	19,75	2021
	19	19,75	2021
	22	19,5	2021
	38	19,5	2021
	10	19,5	2021
	6	19,5	2021
	22	19,5	2021
	46	19,5	2021
	50	19,5	2021
	58	19,5	2021
	70	19,5	2021
	9	19,25	2021
	21	19,25	2021
	13	19,25	2021
	57	19,25	2021
	25	19,25	2021
	53	19,25	2021
	0	19	2021
	28	19	2021
	12	19	2021
	32	19	2021
	12	19	2021
	16	19	2021
	3	18,75	2021
	27	18,75	2021
	3	18,75	2021
	15	18,75	2021
	23	18,75	2021
	47	18,75	2021
	59	18,75	2021
	14	18,5	2021
	14	18,5	2021
	14	18,5	2021

	26	18,5	2021
	38	18,5	2021
	18	18,5	2021
	13	18,25	2021
	17	18,25	2021
	25	18,25	2021
	45	18,25	2021
	0	18	2021
	16	18	2021
	0	18	2021
	24	18	2021
	24	18	2021
	36	18	2021
	36	18	2021
	0	18	2021
	0	18	2021
	0	18	2021
	12	18	2021
	16	18	2021
	28	18	2021
	48	18	2021
	72	18	2021
	72	18	2021
	3	17,75	2021
	7	17,75	2021
	31	17,75	2021
	3	17,75	2021
	3	17,75	2021
	3	17,75	2021
	7	17,75	2021
	31	17,75	2021
	3	17,75	2021
	35	17,75	2021
	43	17,75	2021
	51	17,75	2021
	22	17,5	2021
	22	17,5	2021
	10	17,5	2021
	30	17,5	2021
	30	17,5	2021
	14	17,5	2021
	14	17,5	2021
	50	17,5	2021
	6	17,5	2021
	6	17,5	2021
	6	17,5	2021
	26	17,5	2021
	42	17,5	2021
	70	17,5	2021
	13	17,25	2021

	21	17,25	2021
	29	17,25	2021
	29	17,25	2021
	41	17,25	2021
	13	17,25	2021
	25	17,25	2021
	28	17	2021
	0	17	2021
	20	17	2021
	28	17	2021
	20	17	2021
	40	17	2021
	40	17	2021
	40	17	2021
	27	16,75	2021
	51	16,75	2021
	67	16,75	2021
	6	16,5	2021
	18	16,5	2021
	6	16,5	2021
	10	16,5	2021
	22	16,5	2021
	26	16,5	2021
	18	16,5	2021
	22	16,5	2021
	26	16,5	2021
	46	16,5	2021
	66	16,5	2021
	66	16,5	2021
	9	16,25	2021
	17	16,25	2021
	17	16,25	2021
	17	16,25	2021
	37	16,25	2021
	45	16,25	2021
	45	16,25	2021
	45	16,25	2021
	49	16,25	2021
	0	16	2021
	0	16	2021
	16	16	2021
	16	16	2021
	36	16	2021
	16	16	2021
	16	16	2021
	28	16	2021
	32	16	2021
	36	16	2021
	40	16	2021
	40	16	2021

	3	15,75	2021
	15	15,75	2021
	27	15,75	2021
	35	15,75	2021
	43	15,75	2021
	3	15,75	2021
	19	15,75	2021
	6	15,5	2021
	14	15,5	2021
	14	15,5	2021
	22	15,5	2021
	22	15,5	2021
	26	15,5	2021
	6	15,5	2021
	10	15,5	2021
	26	15,5	2021
	34	15,5	2021
	25	15,25	2021
	21	15,25	2021
	41	15,25	2021
	25	15,25	2021
	25	15,25	2021
	53	15,25	2021
	0	15	2021
	0	15	2021
	0	15	2021
	0	15	2021
	0	15	2021
	20	15	2021
	24	15	2021
	24	15	2021
	24	15	2021
	32	15	2021
	60	15	2021
	19	14,75	2021
	3	14,75	2021
	3	14,75	2021
	23	14,75	2021
	23	14,75	2021
	15	14,75	2021
	19	14,75	2021
	23	14,75	2021
	23	14,75	2021
	31	14,75	2021
	51	14,75	2021
	10	14,5	2021
	22	14,5	2021
	6	14,5	2021
	22	14,5	2021
	30	14,5	2021

	9	14,25	2021
	29	14,25	2021
	9	14,25	2021
	13	14,25	2021
	13	14,25	2021
	21	14,25	2021
	0	14	2021
	16	14	2021
	16	14	2021
	16	14	2021
	20	14	2021
	28	14	2021
	36	14	2021
	0	14	2021
	0	14	2021
	12	14	2021
	7	13,75	2021
	7	13,75	2021
	15	13,75	2021
	15	13,75	2021
	15	13,75	2021
	7	13,75	2021
	15	13,75	2021
	7	13,75	2021
	7	13,75	2021
	7	13,75	2021
	7	13,75	2021
	15	13,75	2021
	19	13,75	2021
	27	13,75	2021
	27	13,75	2021
	15	13,75	2021
	19	13,75	2021
	19	13,75	2021
	27	13,75	2021
	31	13,75	2021
	55	13,75	2021
	6	13,5	2021
	6	13,5	2021
	6	13,5	2021
	6	13,5	2021
	14	13,5	2021
	14	13,5	2021
	14	13,5	2021
	14	13,5	2021
	14	13,5	2021
	6	13,5	2021
	6	13,5	2021
	34	13,5	2021
	46	13,5	2021

	54	13,5	2021
	13	13,25	2021
	9	13,25	2021
	33	13,25	2021
	33	13,25	2021
	9	13,25	2021
	17	13,25	2021
	25	13,25	2021
	25	13,25	2021
	16	13	2021
	32	13	2021
	3	12,75	2021
	3	12,75	2021
	3	12,75	2021
	3	12,75	2021
	3	12,75	2021
	31	12,75	2021
	23	12,75	2021
	31	12,5	2021
	10	12,5	2021
	10	12,5	2021
	10	12,5	2021
	10	12,5	2021
	10	12,5	2021
	10	12,5	2021
	10	12,5	2021
	10	12,5	2021
	6	12,5	2021
	22	12,5	2021
	22	12,5	2021
	6	12,5	2021
	6	12,5	2021
	18	12,5	2021
	22	12,5	2021
	34	12,5	2021
	50	12,5	2021
	9	12,25	2021
	13	12,25	2021
	13	12,25	2021
	13	12,25	2021
	13	12,25	2021
	9	12,25	2021
	9	12,25	2021
	13	12,25	2021
	29	12,25	2021
	33	12,25	2021
	33	12,25	2021
	0	12	2021
	0	12	2021
	0	12	2021

	0	12	2021
	20	12	2021
	20	12	2021
	20	12	2021
	28	12	2021
	12	12	2021
	12	12	2021
	12	12	2021
	28	12	2021
	32	12	2021
	40	12	2021
	7	11,75	2021
	7	11,75	2021
	7	11,75	2021
	7	11,75	2021
	7	11,75	2021
	7	11,75	2021
	7	11,75	2021
	7	11,75	2021
	7	11,75	2021
	7	11,75	2021
	19	11,75	2021
	27	11,75	2021
	27	11,75	2021
	3	11,75	2021
	3	11,75	2021
	3	11,75	2021
	7	11,75	2021
	23	11,75	2021
	6	11,5	2021
	6	11,5	2021
	6	11,5	2021
	10	11,5	2021
	10	11,5	2021
	6	11,5	2021
	22	11,5	2021
	38	11,5	2021
	9	11,25	2021
	9	11,25	2021
	9	11,25	2021
	17	11,25	2021
	17	11,25	2021
	9	11,25	2021
	25	11,25	2021
	37	11,25	2021
	37	11,25	2021
	45	11,25	2021
	45	11,25	2021
	45	11,25	2021
	45	11,25	2021

	45	11,25	2021
	45	11,25	2021
	45	11,25	2021
	45	11,25	2021
	0	11	2021
	16	11	2021
	16	11	2021
	12	11	2021
	16	11	2021
	28	11	2021
	44	11	2021
	44	11	2021
	3	10,75	2021
	3	10,75	2021
	3	10,75	2021
	3	10,75	2021
	3	10,75	2021
	3	10,75	2021
	7	10,75	2021
	23	10,75	2021
	3	10,75	2021
	7	10,75	2021
	15	10,75	2021
	15	10,75	2021
	15	10,75	2021
	19	10,75	2021
	19	10,75	2021
	27	10,75	2021
	43	10,75	2021
	6	10,5	2021
	6	10,5	2021
	14	10,5	2021
	14	10,5	2021
	14	10,5	2021
	14	10,5	2021
	6	10,5	2021
	6	10,5	2021
	6	10,5	2021
	6	10,5	2021
	6	10,5	2021
	26	10,5	2021
	34	10,5	2021
	34	10,5	2021
	34	10,5	2021
	34	10,5	2021
	13	10,25	2021
	13	10,25	2021
	13	10,25	2021
	13	10,25	2021
	13	10,25	2021

	13	10,25	2021
	13	10,25	2021
	21	10,25	2021
	9	10,25	2021
	13	10,25	2021
	25	10,25	2021
	25	10,25	2021
	33	10,25	2021
	33	10,25	2021
	0	10	2021
	0	10	2021
	0	10	2021
	0	10	2021
	0	10	2021
	0	10	2021
	0	10	2021
	0	10	2021
	0	10	2021
	0	10	2021
	0	10	2021
	0	10	2021
	0	10	2021
	0	10	2021
	0	10	2021
	0	10	2021
	0	10	2021
	0	10	2021
	0	10	2021
	0	10	2021
	0	10	2021
	0	10	2021
	0	10	2021
	0	10	2021
	0	10	2021
	0	10	2021
	0	10	2021
	0	10	2021
	0	10	2021
	20	10	2021
	20	10	2021
	20	10	2021
	20	10	2021
	0	10	2021
	0	10	2021
	12	10	2021
	12	10	2021
	20	10	2021
	20	10	2021
	32	10	2021
	3	9,75	2021

	3	9,75	2021
	3	9,75	2021
	3	9,75	2021
	19	9,75	2021
	3	9,75	2021
	3	9,75	2021
	3	9,75	2021
	3	9,75	2021
	3	9,75	2021
	3	9,75	2021
	3	9,75	2021
	19	9,75	2021
	23	9,75	2021
	39	9,75	2021
	10	9,5	2021
	10	9,5	2021
	10	9,5	2021
	22	9,5	2021
	30	9,5	2021
	9	9,25	2021
	17	9,25	2021
	17	9,25	2021
	17	9,25	2021
	9	9,25	2021
	9	9,25	2021
	9	9,25	2021
	9	9,25	2021
	13	9,25	2021
	13	9,25	2021
	13	9,25	2021
	29	9,25	2021
	16	9	2021
	0	9	2021
	0	9	2021
	0	9	2021
	0	9	2021
	0	9	2021
	0	9	2021
	0	9	2021
	0	9	2021
	0	9	2021
	0	9	2021
	0	9	2021
	16	9	2021
	20	9	2021
	28	9	2021
	28	9	2021
	7	8,75	2021
	7	8,75	2021
	7	8,75	2021

	7	8,75	2021
	7	8,75	2021
	7	8,75	2021
	7	8,75	2021
	15	8,75	2021
	19	8,75	2021
	27	8,75	2021
	35	8,75	2021
	6	8,5	2021
	6	8,5	2021
	6	8,5	2021
	6	8,5	2021
	14	8,5	2021
	14	8,5	2021
	14	8,5	2021
	14	8,5	2021
	14	8,5	2021
	14	8,5	2021
	14	8,5	2021
	14	8,5	2021
	14	8,5	2021
	14	8,5	2021
	6	8,5	2021
	6	8,5	2021
	6	8,5	2021
	6	8,5	2021
	6	8,5	2021
	6	8,5	2021
	6	8,5	2021
	14	8,5	2021
	18	8,5	2021
	26	8,5	2021
	26	8,5	2021
	26	8,5	2021
	34	8,5	2021
	13	8,25	2021
	13	8,25	2021
	13	8,25	2021
	13	8,25	2021
	13	8,25	2021
	13	8,25	2021
	13	8,25	2021
	13	8,25	2021
	9	8,25	2021
	9	8,25	2021
	13	8,25	2021
	25	8,25	2021
	25	8,25	2021
	25	8,25	2021
	16	8	2021

	16	8	2021
	24	8	2021
	24	8	2021
	32	8	2021
	32	8	2021
	32	8	2021
	3	7,75	2021
	3	7,75	2021
	3	7,75	2021
	3	7,75	2021
	3	7,75	2021
	3	7,75	2021
	3	7,75	2021
	3	7,75	2021
	3	7,75	2021
	3	7,75	2021
	3	7,75	2021
	3	7,75	2021
	7	7,75	2021
	15	7,75	2021
	23	7,75	2021
	23	7,75	2021
	23	7,75	2021
	31	7,75	2021
	31	7,75	2021
	10	7,5	2021
	10	7,5	2021
	10	7,5	2021
	6	7,5	2021
	6	7,5	2021
	6	7,5	2021
	10	7,5	2021
	10	7,5	2021
	10	7,5	2021
	22	7,5	2021
	22	7,5	2021
	30	7,5	2021
	30	7,5	2021
	30	7,5	2021
	30	7,5	2021
	9	7,25	2021
	9	7,25	2021
	9	7,25	2021
	9	7,25	2021
	9	7,25	2021
	9	7,25	2021
	9	7,25	2021
	9	7,25	2021
	9	7,25	2021
	13	7,25	2021

	13	7,25	2021
	13	7,25	2021
	13	7,25	2021
	21	7,25	2021
	21	7,25	2021
	21	7,25	2021
	0	7	2021
	0	7	2021
	0	7	2021
	0	7	2021
	0	7	2021
	0	7	2021
	0	7	2021
	0	7	2021
	0	7	2021
	0	7	2021
	0	7	2021
	0	7	2021
	0	7	2021
	0	7	2021
	0	7	2021
	0	7	2021
	0	7	2021
	0	7	2021
	0	7	2021
	0	7	2021
	0	7	2021
	0	7	2021
	20	7	2021
	20	7	2021
	20	7	2021
	20	7	2021
	28	7	2021
	28	7	2021
	28	7	2021
	28	7	2021
	7	6,75	2021
	7	6,75	2021
	7	6,75	2021
	7	6,75	2021
	7	6,75	2021
	7	6,75	2021
	7	6,75	2021
	7	6,75	2021
	7	6,75	2021
	7	6,75	2021
	7	6,75	2021
	7	6,75	2021
	7	6,75	2021
	7	6,75	2021

	7	6,75	2021
	7	6,75	2021
	7	6,75	2021
	7	6,75	2021
	7	6,75	2021
	7	6,75	2021
	7	6,75	2021
	7	6,75	2021
	7	6,75	2021
	3	6,75	2021
	3	6,75	2021
	3	6,75	2021
	3	6,75	2021
	3	6,75	2021
	7	6,75	2021
	7	6,75	2021
	19	6,75	2021
	19	6,75	2021
	27	6,75	2021
	27	6,75	2021
	27	6,75	2021
	27	6,75	2021
	27	6,75	2021
	27	6,75	2021
	6	6,5	2021
	6	6,5	2021
	6	6,5	2021
	6	6,5	2021
	6	6,5	2021
	6	6,5	2021
	6	6,5	2021
	6	6,5	2021
	6	6,5	2021
	6	6,5	2021
	6	6,5	2021
	6	6,5	2021
	6	6,5	2021
	6	6,5	2021
	6	6,5	2021
	6	6,5	2021
	6	6,5	2021
	6	6,5	2021
	6	6,5	2021
	6	6,5	2021
	10	6,5	2021
	10	6,5	2021
	10	6,5	2021
	10	6,5	2021
	10	6,5	2021
	10	6,5	2021

	10	6,5	2021
	18	6,5	2021
	18	6,5	2021
	18	6,5	2021
	9	6,25	2021
	9	6,25	2021
	9	6,25	2021
	9	6,25	2021
	9	6,25	2021
	9	6,25	2021
	9	6,25	2021
	9	6,25	2021
	9	6,25	2021
	9	6,25	2021
	17	6,25	2021
	17	6,25	2021
	25	6,25	2021
	25	6,25	2021
	25	6,25	2021
	25	6,25	2021
	25	6,25	2021
	25	6,25	2021
	0	6	2021
	0	6	2021
	16	6	2021
	16	6	2021
	16	6	2021
	24	6	2021
	24	6	2021
	3	5,75	2021
	3	5,75	2021
	3	5,75	2021
	3	5,75	2021
	3	5,75	2021
	3	5,75	2021
	3	5,75	2021
	3	5,75	2021
	3	5,75	2021
	3	5,75	2021
	3	5,75	2021
	3	5,75	2021
	3	5,75	2021
	3	5,75	2021
	3	5,75	2021
	3	5,75	2021
	3	5,75	2021
	3	5,75	2021
	3	5,75	2021
	3	5,75	2021
	3	5,75	2021

	3	5,75	2021
	3	5,75	2021
	3	5,75	2021
	3	5,75	2021
	3	5,75	2021
	7	5,75	2021
	7	5,75	2021
	7	5,75	2021
	7	5,75	2021
	7	5,75	2021
	15	5,75	2021
	15	5,75	2021
	6	5,5	2021
	6	5,5	2021
	6	5,5	2021
	6	5,5	2021
	6	5,5	2021
	6	5,5	2021
	6	5,5	2021
	6	5,5	2021
	14	5,5	2021
	14	5,5	2021
	14	5,5	2021
	14	5,5	2021
	14	5,5	2021
	22	5,5	2021
	22	5,5	2021
	22	5,5	2021
	13	1,5	2021
	13	1,5	2021
	13	1,5	2021
	13	1,5	2021
	13	1,5	2021
	13	1,5	2021
	13	1,5	2021
	13	1,5	2021
	13	1,5	2021
	13	1,5	2021
	21	1,5	2021
	21	1,5	2021
	21	1,5	2021
	21	1,5	2021
	21	1,5	2021
	21	1,5	2021
	21	1,5	2021
	0	5	2021
	0	5	2021
	0	5	2021
	0	5	2021
	0	5	2021

0	5	2021
0	5	2021
0	5	2021
0	5	2021
0	5	2021
0	5	2021
0	5	2021
0	5	2021
0	5	2021
0	5	2021
0	5	2021
0	5	2021
0	5	2021
0	5	2021
0	5	2021
0	5	2021
0	5	2021
0	5	2021
0	5	2021
0	5	2021
0	5	2021
0	5	2021
0	5	2021
0	5	2021
0	5	2021
0	5	2021
0	5	2021
0	5	2021
0	5	2021
0	5	2021
0	5	2021
0	5	2021
0	5	2021
0	5	2021
0	5	2021
0	5	2021
0	5	2021
0	5	2021
0	5	2021
0	5	2021
0	5	2021
0	5	2021
0	5	2021
0	5	2021
0	5	2021
0	5	2021
0	5	2021
0	5	2021
0	5	2021
0	5	2021

	0	5	2021
	0	5	2021
	0	5	2021
	0	5	2021
	0	5	2021
	0	5	2021
	0	5	2021
	0	5	2021
	0	5	2021
	0	5	2021
	0	5	2021
	0	5	2021
	0	5	2021
	0	5	2021
	0	5	2021
	0	5	2021
	0	5	2021
	0	5	2021
	0	5	2021
	0	5	2021
	0	5	2021
	0	5	2021
	0	5	2021
	0	5	2021
	0	5	2021
	0	5	2021
	0	5	2021
	0	5	2021
	0	5	2021
	0	5	2021
	0	5	2021
	0	5	2021
	0	5	2021
	0	5	2021
	0	5	2021
	0	5	2021
	0	5	2021
	0	5	2021
	0	5	2021
	12	5	2021
	12	5	2021
	12	5	2021
	12	5	2021
	20	5	2021
	20	5	2021
	20	5	2021
	20	5	2021
	3	4,75	2021
	3	4,75	2021
	3	4,75	2021

	3	4,75	2021
	3	4,75	2021
	3	4,75	2021
	3	4,75	2021
	3	4,75	2021
	3	4,75	2021
	3	4,75	2021
	3	4,75	2021
	3	4,75	2021
	3	4,75	2021
	3	4,75	2021
	3	4,75	2021
	3	4,75	2021
	3	4,75	2021
	3	4,75	2021
	3	4,75	2021
	3	4,75	2021
	3	4,75	2021
	19	4,75	2021
	19	4,75	2021
	19	4,75	2021
	19	4,75	2021
	10	4,5	2021
	10	4,5	2021
	10	4,5	2021
	10	4,5	2021
	10	4,5	2021
	10	4,5	2021
	10	4,5	2021
	10	4,5	2021
	10	4,5	2021
	10	4,5	2021
	10	4,5	2021
	10	4,5	2021
	18	4,5	2021
	18	4,5	2021
	18	4,5	2021
	18	4,5	2021
	18	4,5	2021
	9	4,25	2021
	9	4,25	2021
	9	4,25	2021
	9	4,25	2021
	9	4,25	2021
	9	4,25	2021
	9	4,25	2021
	9	4,25	2021
	17	4,25	2021
	17	4,25	2021
	17	4,25	2021

	17	4,25	2021
	17	4,25	2021
	17	4,25	2021
	17	4,25	2021
	17	4,25	2021
	0	4	2021
	0	4	2021
	0	4	2021
	0	4	2021
	0	4	2021
	0	4	2021
	0	4	2021
	0	4	2021
	0	4	2021
	0	4	2021
	0	4	2021
	0	4	2021
	0	4	2021
	0	4	2021
	0	4	2021
	0	4	2021
	0	4	2021
	0	4	2021
	16	4	2021
	16	4	2021
	16	4	2021
	16	4	2021
	16	4	2021
	16	4	2021
	7	3,75	2021
	7	3,75	2021
	7	3,75	2021
	7	3,75	2021
	7	3,75	2021
	7	3,75	2021
	7	3,75	2021
	7	3,75	2021
	15	3,75	2021
	15	3,75	2021
	15	3,75	2021
	15	3,75	2021
	15	3,75	2021
	15	3,75	2021
	15	3,75	2021
	15	3,75	2021
	15	3,75	2021
	15	3,75	2021
	15	3,75	2021
	15	3,75	2021
	15	3,75	2021

	15	3,75	2021
	15	3,75	2021
	15	3,75	2021
	15	3,75	2021
	6	3,5	2021
	6	3,5	2021
	6	3,5	2021
	6	3,5	2021
	6	3,5	2021
	6	3,5	2021
	6	3,5	2021
	6	3,5	2021
	6	3,5	2021
	6	3,5	2021
	6	3,5	2021
	6	3,5	2021
	6	3,5	2021
	6	3,5	2021
	6	3,5	2021
	6	3,5	2021
	6	3,5	2021
	6	3,5	2021
	6	3,5	2021
	6	3,5	2021
	6	3,5	2021
	6	3,5	2021
	6	3,5	2021
	6	3,5	2021
	6	3,5	2021
	6	3,5	2021
	6	3,5	2021
	6	3,5	2021
	6	3,5	2021
	6	3,5	2021
	6	3,5	2021
	6	3,5	2021
	6	3,5	2021
	6	3,5	2021
	6	3,5	2021
	6	3,5	2021
	14	3,5	2021
	14	3,5	2021
	14	3,5	2021
	14	3,5	2021
	14	3,5	2021
	14	3,5	2021
	14	3,5	2021
	14	3,5	2021
	14	3,5	2021
	14	3,5	2021

	14	3,5	2021
	14	3,5	2021
	14	3,5	2021
	14	3,5	2021
	14	3,5	2021
	14	3,5	2021
	14	3,5	2021
	14	3,5	2021
	14	3,5	2021
	14	3,5	2021
	14	3,5	2021
	14	3,5	2021
	14	3,5	2021
	14	3,5	2021
	14	3,5	2021
	14	3,5	2021
	14	3,5	2021
	14	3,5	2021
	13	3,25	2021
	13	3,25	2021
	13	3,25	2021
	13	3,25	2021
	13	3,25	2021
	13	3,25	2021
	13	3,25	2021
	13	3,25	2021
	13	3,25	2021
	13	3,25	2021
	13	3,25	2021
	13	3,25	2021
	13	3,25	2021
	13	3,25	2021
	13	3,25	2021
	12	3	2021
	12	3	2021
	12	3	2021
	12	3	2021
	12	3	2021
	12	3	2021
	3	2,75	2021
	3	2,75	2021
	3	2,75	2021
	3	2,75	2021
	3	2,75	2021
	3	2,75	2021
	3	2,75	2021
	3	2,75	2021
	3	2,75	2021
	3	2,75	2021
	3	2,75	2021

3	2,75	2021
3	2,75	2021
3	2,75	2021
3	2,75	2021
3	2,75	2021
3	2,75	2021
3	2,75	2021
3	2,75	2021
3	2,75	2021
3	2,75	2021
3	2,75	2021
3	2,75	2021
3	2,75	2021
3	2,75	2021
3	2,75	2021
3	2,75	2021
3	2,75	2021
3	2,75	2021
3	2,75	2021
3	2,75	2021
3	2,75	2021
3	2,75	2021
3	2,75	2021
3	2,75	2021
3	2,75	2021
3	2,75	2021
3	2,75	2021
3	2,75	2021
3	2,75	2021
3	2,75	2021
3	2,75	2021
3	2,75	2021
3	2,75	2021
3	2,75	2021
3	2,75	2021
3	2,75	2021
3	2,75	2021
3	2,75	2021
3	2,75	2021
3	2,75	2021
3	2,75	2021
3	2,75	2021
3	2,75	2021
3	2,75	2021
3	2,75	2021
3	2,75	2021
3	2,75	2021
3	2,75	2021
3	2,75	2021
3	2,75	2021

	3	2,75	2021
	3	2,75	2021
	3	2,75	2021
	3	2,75	2021
	3	2,75	2021
	10	2,5	2021
	10	2,5	2021
	10	2,5	2021
	10	2,5	2021
	10	2,5	2021
	10	2,5	2021
	10	2,5	2021
	10	2,5	2021
	10	2,5	2021
	10	2,5	2021
	10	2,5	2021
	10	2,5	2021
	10	2,5	2021
	10	2,5	2021
	10	2,5	2021
	10	2,5	2021
	10	2,5	2021
	10	2,5	2021
	10	2,5	2021
	9	2,25	2021
	9	2,25	2021
	9	2,25	2021
	9	2,25	2021
	9	2,25	2021
	9	2,25	2021
	9	2,25	2021
	9	2,25	2021
	9	2,25	2021
	9	2,25	2021
	0	2	2021
	0	2	2021
	0	2	2021
	0	2	2021
	0	2	2021
	0	2	2021
	0	2	2021
	0	2	2021
	0	2	2021
	0	2	2021
	0	2	2021
	0	2	2021
	0	2	2021
	0	2	2021
	0	2	2021
	0	2	2021

0	2	2021
0	2	2021
0	2	2021
0	2	2021
0	2	2021
0	2	2021
0	2	2021
0	2	2021
0	2	2021
0	2	2021
0	2	2021
0	2	2021
0	2	2021
0	2	2021
0	2	2021
0	2	2021
0	2	2021
0	2	2021
0	2	2021
0	2	2021
0	2	2021
0	2	2021
0	2	2021
0	2	2021
0	2	2021
0	2	2021
0	2	2021
0	2	2021
0	2	2021
0	2	2021
0	2	2021
0	2	2021
0	2	2021
0	2	2021
0	2	2021
0	2	2021
0	2	2021
0	2	2021
0	2	2021
0	2	2021
0	2	2021
0	2	2021
0	2	2021
0	2	2021
0	2	2021
0	2	2021
0	2	2021
0	2	2021
0	2	2021
0	2	2021

0	2	2021
0	2	2021
0	2	2021
0	2	2021
0	2	2021
0	2	2021
0	2	2021
0	2	2021
0	2	2021
0	2	2021
0	2	2021
0	2	2021
0	2	2021
0	2	2021
0	2	2021
0	2	2021
0	2	2021
0	2	2021
0	2	2021
0	2	2021
0	2	2021
0	2	2021
0	2	2021
0	2	2021
0	2	2021
0	2	2021
0	2	2021
0	2	2021
0	2	2021
0	2	2021
0	2	2021
0	2	2021
0	2	2021
0	2	2021
0	2	2021
0	2	2021
0	2	2021
0	2	2021
0	2	2021
0	2	2021
0	2	2021
0	2	2021
0	2	2021
0	2	2021
0	2	2021
0	2	2021
0	2	2021
0	2	2021
0	2	2021
0	2	2021

0	2	2021
0	2	2021
0	2	2021
0	2	2021
0	2	2021
0	2	2021
0	2	2021
0	2	2021
0	2	2021
0	2	2021
0	2	2021
0	2	2021
0	2	2021
0	2	2021
0	2	2021
0	2	2021
0	2	2021
0	2	2021
0	2	2021
0	2	2021
7	1,75	2021
7	1,75	2021
7	1,75	2021
7	1,75	2021
7	1,75	2021
7	1,75	2021
7	1,75	2021
7	1,75	2021
7	1,75	2021
7	1,75	2021
7	1,75	2021
7	1,75	2021
7	1,75	2021
7	1,75	2021
7	1,75	2021
7	1,75	2021
7	1,75	2021
7	1,75	2021
7	1,75	2021
7	1,75	2021
7	1,75	2021
7	1,75	2021
7	1,75	2021
7	1,75	2021
7	1,75	2021
7	1,75	2021
7	1,75	2021
7	1,75	2021
7	1,75	2021
7	1,75	2021

7	1,75	2021
7	1,75	2021
7	1,75	2021
7	1,75	2021
7	1,75	2021
7	1,75	2021
7	1,75	2021
7	1,75	2021
7	1,75	2021
7	1,75	2021
7	1,75	2021
7	1,75	2021
7	1,75	2021
7	1,75	2021
7	1,75	2021
7	1,75	2021
7	1,75	2021
7	1,75	2021
7	1,75	2021
7	1,75	2021
7	1,75	2021
7	1,75	2021
7	1,75	2021
7	1,75	2021
7	1,75	2021
7	1,75	2021
7	1,75	2021
7	1,75	2021
7	1,75	2021
7	1,75	2021
7	1,75	2021
7	1,75	2021
7	1,75	2021
7	1,75	2021
7	1,75	2021
7	1,75	2021
7	1,75	2021
7	1,75	2021
7	1,75	2021
7	1,75	2021
7	1,75	2021
7	1,75	2021
7	1,75	2021
7	1,75	2021
7	1,75	2021
7	1,75	2021
7	1,75	2021
7	1,75	2021
7	1,75	2021
7	1,75	2021

	7	1,75	2021
	7	1,75	2021
	7	1,75	2021
	7	1,75	2021
	7	1,75	2021
	7	1,75	2021
	6	1,5	2021
	6	1,5	2021
	6	1,5	2021
	6	1,5	2021
	6	1,5	2021
	6	1,5	2021
	6	1,5	2021
	6	1,5	2021
	6	1,5	2021
	6	1,5	2021
	6	1,5	2021
	6	1,5	2021
	6	1,5	2021
	6	1,5	2021
	6	1,5	2021
	6	1,5	2021
	6	1,5	2021
	6	1,5	2021
	6	1,5	2021
	6	1,5	2021
	6	1,5	2021
	6	1,5	2021
	6	1,5	2021
	6	1,5	2021
	6	1,5	2021
	6	1,5	2021
	6	1,5	2021
	6	1,5	2021
	6	1,5	2021
	6	1,5	2021
	6	1,5	2021
	6	1,5	2021
	6	1,5	2021
	6	1,5	2021
	6	1,5	2021
	6	1,5	2021
	6	1,5	2021
	6	1,5	2021
	6	1,5	2021
	6	1,5	2021
	6	1,5	2021
	6	1,5	2021
	6	1,5	2021
	6	1,5	2021

	6	1,5	2021
	6	1,5	2021
	6	1,5	2021
	6	1,5	2021
	6	1,5	2021
	6	1,5	2021
	6	1,5	2021
	6	1,5	2021
	6	1,5	2021
	6	1,5	2021
	6	1,5	2021
	6	1,5	2021
	6	1,5	2021
	6	1,5	2021
	6	1,5	2021
	6	1,5	2021
	6	1,5	2021
	6	1,5	2021
	6	1,5	2021
	6	1,5	2021
	6	1,5	2021
	6	1,5	2021
	6	1,5	2021
	6	1,5	2021
	6	1,5	2021
	6	1,5	2021
	6	1,5	2021
	6	1,5	2021
	6	1,5	2021
	6	1,5	2021
	6	1,5	2021
	3	0,75	2021
	3	0,75	2021
	3	0,75	2021
	3	0,75	2021
	3	0,75	2021
	3	0,75	2021
	3	0,75	2021
	3	0,75	2021
	3	0,75	2021
	3	0,75	2021
	3	0,75	2021
	3	0,75	2021
	3	0,75	2021
	3	0,75	2021
	3	0,75	2021
	3	0,75	2021
	3	0,75	2021
	3	0,75	2021
	3	0,75	2021

	3	0,75	2021
	3	0,75	2021
	3	0,75	2021
	3	0,75	2021
	3	0,75	2021
	3	0,75	2021
	3	0,75	2021
	3	0,75	2021
	3	0,75	2021
	3	0,75	2021
	3	0,75	2021
	3	0,75	2021
	3	0,75	2021
	3	0,75	2021
	3	0,75	2021
	3	0,75	2021
	3	0,75	2021
	3	0,75	2021
	3	0,75	2021
	3	0,75	2021
	3	0,75	2021
	3	0,75	2021
	3	0,75	2021
	3	0,75	2021
	3	0,75	2021
	3	0,75	2021
	3	0,75	2021
	3	0,75	2021
	3	0,75	2021
	3	0,75	2021
	3	0,75	2021
	3	0,75	2021
	3	0,75	2021
	3	0,75	2021
	3	0,75	2021
	3	0,75	2021
	3	0,75	2021
	3	0,75	2021
	3	0,75	2021
	3	0,75	2021
	3	0,75	2021
	3	0,75	2021
	3	0,75	2021
	3	0,75	2021
	3	0,75	2021
	3	0,75	2021
	3	0,75	2021
	3	0,75	2021
	3	0,75	2021
	3	0,75	2021

	3	0,75	2021
	3	0,75	2021
	3	0,75	2021
	3	0,75	2021
	3	0,75	2021
	3	0,75	2021
	3	0,75	2021
	3	0,75	2021
	3	0,75	2021
	3	0,75	2021
	3	0,75	2021
	3	0,75	2021
	3	0,75	2021
	3	0,75	2021
	3	0,75	2021
	3	0,75	2021
	3	0,75	2021
	3	0,75	2021
	3	0,75	2021
	3	0,75	2021
	3	0,75	2021
	3	0,75	2021
	3	0,75	2021
	3	0,75	2021
	3	0,75	2021
	3	0,75	2021
	3	0,75	2021
	3	0,75	2021
	3	0,75	2021
	3	0,75	2021
	3	0,75	2021
	3	0,75	2021
	3	0,75	2021
	3	0,75	2021
	3	0,75	2021
	3	0,75	2021
	3	0,75	2021
	3	0,75	2021
	3	0,75	2021
	3	0,75	2021
	3	0,75	2021
	3	0,75	2021
	3	0,75	2021
	3	0,75	2021
	3	0,75	2021
	3	0,75	2021
	3	0,75	2021
	3	0,75	2021
	3	0,75	2021
	3	0,75	2021

	3	0,75	2021
	3	0,75	2021
	3	0,75	2021
	3	0,75	2021
	3	0,75	2021
	3	0,75	2021
	3	0,75	2021
	3	0,75	2021
	3	0,75	2021
	3	0,75	2021
	3	0,75	2021
	3	0,75	2021
	3	0,75	2021
	3	0,75	2021
	3	0,75	2021
	3	0,75	2021
	3	0,75	2021
	3	0,75	2021
	3	0,75	2021
	3	0,75	2021
	3	0,75	2021
	3	0,75	2021
	3	0,75	2021
	3	0,75	2021
	3	0,75	2021
	3	0,75	2021
	3	0,75	2021
	3	0,75	2021
	3	0,75	2021
	3	0,75	2021
	3	0,75	2021
	3	0,75	2021
	3	0,75	2021
	3	0,75	2021
	3	0,75	2021
	3	0,75	2021
	3	0,75	2021
	3	0,75	2021
	3	0,75	2021
	3	0,75	2021
	3	0,75	2021
	3	0,75	2021
	3	0,75	2021
	3	0,75	2021
	3	0,75	2021
	3	0,75	2021
	3	0,75	2021
	3	0,75	2021
	3	0,75	2021
	3	0,75	2021

	3	0,75	2021
	3	0,75	2021
	3	0,75	2021
	3	0,75	2021
	3	0,75	2021
	3	0,75	2021
	3	0,75	2021
	1005	2951,25	2020
	615	2663,75	2020
	1989	2402,25	2020
	625	2186,25	2020
	955	2063,75	2020
	1210	2027,5	2020
	925	1686,25	2020
	1544	1576	2020
	1125	1511,25	2020
	1135	1453,75	2020
	850	1427,5	2020
	1364	1386	2020
	590	1332,5	2020
	975	1323,75	2020
	530	1272,5	2020
	860	1240	2020
	395	1188,75	2020
	945	1156,25	2020
	1420	1150	2020
	577	1114,25	2020
	824	1096	2020
	845	1061,25	2020
	1289	1042,25	2020
	642	1040,5	2020
	692	1038	2020
	457	1034,25	2020
	596	1029	2020
	762	1000,5	2020
	742	970,5	2020
	310	927,5	2020
	615	919,75	2020
	750	917,5	2020
	720	915	2020
	1037	914,25	2020
	600	900	2020
	734	888,5	2020
	525	886,25	2020
	547	882,75	2020
	1379	875,75	2020
	1385	866,25	2020
	1222	860,5	2020
	870	852,5	2020
	540	845	2020

	263	843,75	2020
	875	818,75	2020
	1544	811	2020
	516	809	2020
	515	808,75	2020
	1585	801,25	2020
	475	783,75	2020
	585	776,25	2020
	241	760,25	2020
	815	753,75	2020
	625	746,25	2020
	460	740	2020
	312	728	2020
	805	716,25	2020
	510	713,5	2020
	310	712,5	2020
	351	709,75	2020
	304	706	2020
	337	694,25	2020
	847	681,75	2020
	810	672,5	2020
	537	660,25	2020
	660	640	2020
	622	635,5	2020
	427	616,75	2020
	367	607,75	2020
	547	606,75	2020
	605	606,25	2020
	537	604,25	2020
	552	603	2020
	867	601,75	2020
	562	596,5	2020
	559	595,75	2020
	540	591	2020
	585	586,25	2020
	719	579,75	2020
	675	579,75	2020
	520	570	2020
	355	568,75	2020
	457	565,25	2020
	184	562	2020
	627	561,75	2020
	0	560	2020
	469	558,25	2020
	405	551,25	2020
	637	551,25	2020
	292	548	2020
	280	545	2020
	445	543,25	2020
	243	540,75	2020

	520	530	2020
	417	524,25	2020
	435	522,75	2020
	465	522,25	2020
	637	521,25	2020
	390	513,5	2020
	136	512	2020
	445	511,25	2020
	655	508,75	2020
	196	506	2020
	330	498,5	2020
	510	498,5	2020
	692	498	2020
	422	497,5	2020
	260	495	2020
	474	494,5	2020
	475	488,75	2020
	506	487,5	2020
	442	486,5	2020
	555	483,75	2020
	375	483,75	2020
	275	478,75	2020
	294	475,5	2020
	502	471,5	2020
	496	471	2020
	427	467,75	2020
	465	462,25	2020
	462	461,5	2020
	327	453,75	2020
	360	451	2020
	201	442,25	2020
	424	442	2020
	312	439	2020
	350	437,5	2020
	342	437,5	2020
	180	437	2020
	232	434	2020
	438	431,5	2020
	377	430,25	2020
	137	430,25	2020
	232	429	2020
	420	418	2020
	502	413,5	2020
	420	411	2020
	420	403	2020
	276	397	2020
	280	396	2020
	465	391,25	2020
	245	387,25	2020
	337	386,25	2020

	417	386,25	2020
	280	386	2020
	510	379,5	2020
	290	378,5	2020
	192	376	2020
	300	375	2020
	250	374,5	2020
	252	369	2020
	176	366	2020
	270	365,5	2020
	427	364,75	2020
	144	364	2020
	435	356,75	2020
	342	355,5	2020
	281	355,25	2020
	387	354,75	2020
	369	352,25	2020
	412	352	2020
	280	351	2020
	179	350,75	2020
	282	350,5	2020
	367	349,75	2020
	390	343,5	2020
	262	341,5	2020
	195	338,75	2020
	282	336,5	2020
	212	335	2020
	390	333,5	2020
	292	330	2020
	480	330	2020
	412	329	2020
	324	329	2020
	241	328,25	2020
	177	326,25	2020
	216	325	2020
	172	325	2020
	182	321,5	2020
	304	321	2020
	372	321	2020
	132	317	2020
	202	314,5	2020
	354	312,5	2020
	293	309,25	2020
	316	309	2020
	312	309	2020
	147	303,75	2020
	67	302,75	2020
	387	301,75	2020
	360	300	2020
	295	296,75	2020

	264	296	2020
	495	295,75	2020
	216	294	2020
	154	290,5	2020
	192	290	2020
	264	288	2020
	216	288	2020
	270	285,5	2020
	350	285,5	2020
	179	284,75	2020
	119	281,75	2020
	61	280,25	2020
	152	280	2020
	119	277,75	2020
	306	276,5	2020
	122	275,5	2020
	110	273,5	2020
	138	272,5	2020
	224	272	2020
	177	270,25	2020
	129	270,25	2020
	160	270	2020
	306	269,5	2020
	390	269,5	2020
	349	269,25	2020
	292	269	2020
	290	266,5	2020
	342	265,5	2020
	234	264,5	2020
	166	263,5	2020
	286	263,5	2020
	345	262,25	2020
	424	262	2020
	160	260	2020
	320	260	2020
	166	259,5	2020
	267	257,75	2020
	176	256	2020
	294	255,5	2020
	193	254,25	2020
	160	250	2020
	268	250	2020
	142	248,5	2020
	290	248,5	2020
	89	248,25	2020
	475	247,75	2020
	196	247	2020
	495	243,75	2020
	188	243	2020
	387	242,75	2020

	177	242,25	2020
	83	238,75	2020
	149	237,25	2020
	76	235	2020
	194	234,5	2020
	345	234,25	2020
	239	233,75	2020
	164	230	2020
	312	228	2020
	173	227,25	2020
	152	224	2020
	78	223,5	2020
	229	223,25	2020
	192	223	2020
	206	222,5	2020
	358	222,5	2020
	288	220	2020
	216	218	2020
	195	217,75	2020
	235	216,75	2020
	139	216,75	2020
	43	215,75	2020
	281	215,25	2020
	103	213,75	2020
	264	212	2020
	244	212	2020
	104	210	2020
	197	209,25	2020
	20	209	2020
	244	209	2020
	219	208,75	2020
	191	207,75	2020
	357	205,25	2020
	173	205,25	2020
	375	204,75	2020
	144	204	2020
	402	203,5	2020
	303	202,75	2020
	232	201	2020
	240	201	2020
	221	200,25	2020
	208	200	2020
	142	199,5	2020
	300	199	2020
	227	197,75	2020
	191	197,75	2020
	294	197,5	2020
	94	197,5	2020
	209	197,25	2020
	104	197	2020

	236	197	2020
	155	196,75	2020
	282	196,5	2020
	45	196,25	2020
	264	196	2020
	244	195	2020
	130	194,5	2020
	67	192,75	2020
	237	192,25	2020
	216	192	2020
	189	189,25	2020
	187	188,75	2020
	81	188,25	2020
	360	188	2020
	216	188	2020
	182	187,5	2020
	244	187	2020
	167	185,75	2020
	181	184,25	2020
	101	184,25	2020
	154	182,5	2020
	189	182,25	2020
	172	182	2020
	267	179,75	2020
	119	177,75	2020
	184	177	2020
	168	175	2020
	234	174,5	2020
	262	174,5	2020
	55	173,75	2020
	158	173,5	2020
	216	173	2020
	137	172,25	2020
	142	170,5	2020
	178	170,5	2020
	147	169,75	2020
	158	167,5	2020
	117	167,25	2020
	173	167,25	2020
	176	167	2020
	101	166,25	2020
	87	165,75	2020
	75	165,75	2020
	166	165,5	2020
	178	164,5	2020
	197	164,25	2020
	232	164	2020
	67	163,75	2020
	170	163,5	2020
	116	163	2020

	210	162,5	2020
	152	162	2020
	67	161,75	2020
	191	161,75	2020
	139	160,75	2020
	182	160,5	2020
	182	160,5	2020
	205	160,25	2020
	125	160,25	2020
	133	160,25	2020
	135	159,75	2020
	87	159,75	2020
	182	159,5	2020
	170	159,5	2020
	94	159,5	2020
	118	159,5	2020
	188	159	2020
	107	158,75	2020
	134	158,5	2020
	162	158,5	2020
	127	157,75	2020
	174	157,5	2020
	236	157	2020
	95	155,75	2020
	95	154,75	2020
	118	154,5	2020
	230	153,5	2020
	141	153,25	2020
	176	153	2020
	132	153	2020
	7	152,75	2020
	159	152,75	2020
	75	152,75	2020
	250	152,5	2020
	230	152,5	2020
	138	152,5	2020
	165	151,25	2020
	216	151	2020
	192	151	2020
	122	150,5	2020
	103	149,75	2020
	169	149,25	2020
	150	148,5	2020
	183	147,75	2020
	185	147,25	2020
	108	147	2020
	147	146,75	2020
	194	146,5	2020
	154	145,5	2020
	85	145,25	2020

	108	145	2020
	116	145	2020
	128	144	2020
	110	143,5	2020
	117	143,25	2020
	176	143	2020
	280	143	2020
	87	142,75	2020
	152	142	2020
	169	141,25	2020
	157	141,25	2020
	20	139	2020
	122	138,5	2020
	274	138,5	2020
	142	138,5	2020
	51	137,75	2020
	78	137,5	2020
	198	137,5	2020
	177	137,25	2020
	128	137	2020
	176	137	2020
	117	136,25	2020
	113	135,25	2020
	65	135,25	2020
	129	135,25	2020
	169	134,25	2020
	65	134,25	2020
	128	134	2020
	79	133,75	2020
	183	133,75	2020
	75	133,75	2020
	149	133,25	2020
	165	133,25	2020
	32	133	2020
	116	133	2020
	43	132,75	2020
	51	132,75	2020
	117	132,25	2020
	216	132	2020
	136	131	2020
	20	130	2020
	131	129,75	2020
	158	129,5	2020
	54	129,5	2020
	74	128,5	2020
	197	128,25	2020
	109	128,25	2020
	106	127,5	2020
	166	126,5	2020
	182	126,5	2020

	166	126,5	2020
	112	126	2020
	120	126	2020
	112	126	2020
	220	125	2020
	116	125	2020
	59	124,75	2020
	143	123,75	2020
	150	123,5	2020
	201	123,25	2020
	84	123	2020
	44	123	2020
	131	121,75	2020
	130	121,5	2020
	110	121,5	2020
	192	121	2020
	115	120,75	2020
	23	120,75	2020
	190	120,5	2020
	142	120,5	2020
	214	120,5	2020
	165	119,25	2020
	73	119,25	2020
	97	119,25	2020
	69	119,25	2020
	40	119	2020
	49	118,25	2020
	23	117,75	2020
	90	117,5	2020
	29	117,25	2020
	123	116,75	2020
	187	116,75	2020
	74	116,5	2020
	73	116,25	2020
	53	116,25	2020
	92	116	2020
	75	115,75	2020
	79	115,75	2020
	3	115,75	2020
	74	115,5	2020
	125	115,25	2020
	157	115,25	2020
	14	114,5	2020
	182	114,5	2020
	119	113,75	2020
	109	113,25	2020
	129	113,25	2020
	64	113	2020
	139	111,75	2020
	183	111,75	2020

	142	111,5	2020
	36	111	2020
	196	111	2020
	91	110,75	2020
	143	110,75	2020
	70	110,5	2020
	89	110,25	2020
	188	110	2020
	136	110	2020
	127	109,75	2020
	135	109,75	2020
	140	109	2020
	175	108,75	2020
	83	108,75	2020
	130	108,5	2020
	110	108,5	2020
	129	108,25	2020
	52	108	2020
	83	107,75	2020
	171	107,75	2020
	90	107,5	2020
	100	107	2020
	64	106	2020
	40	106	2020
	143	105,75	2020
	77	105,25	2020
	115	104,75	2020
	103	104,75	2020
	115	104,75	2020
	80	104	2020
	151	103,75	2020
	99	103,75	2020
	119	103,75	2020
	102	103,5	2020
	90	103,5	2020
	150	103,5	2020
	58	103,5	2020
	52	103	2020
	68	103	2020
	83	102,75	2020
	118	102,5	2020
	94	102,5	2020
	93	102,25	2020
	19	101,75	2020
	181	101,25	2020
	49	100,25	2020
	133	100,25	2020
	0	100	2020
	139	99,75	2020
	177	99,25	2020

	44	99	2020
	223	98,75	2020
	141	98,25	2020
	44	98	2020
	59	97,75	2020
	103	97,75	2020
	179	97,75	2020
	18	97,5	2020
	113	97,25	2020
	31	96,75	2020
	135	96,75	2020
	89	96,25	2020
	53	96,25	2020
	28	96	2020
	100	96	2020
	150	95,5	2020
	90	95,5	2020
	164	95	2020
	171	94,75	2020
	22	94,5	2020
	45	94,25	2020
	59	93,75	2020
	75	93,75	2020
	131	93,75	2020
	79	93,75	2020
	165	93,25	2020
	137	93,25	2020
	40	93	2020
	112	93	2020
	127	92,75	2020
	135	92,75	2020
	103	92,75	2020
	63	92,75	2020
	90	92,5	2020
	62	92,5	2020
	82	92,5	2020
	64	92	2020
	23	91,75	2020
	10	91,5	2020
	81	91,25	2020
	137	91,25	2020
	200	91	2020
	120	91	2020
	31	90,75	2020
	119	90,75	2020
	106	90,5	2020
	86	90,5	2020
	214	90,5	2020
	140	90	2020
	140	90	2020

	15	89,75	2020
	103	89,75	2020
	86	89,5	2020
	106	89,5	2020
	182	89,5	2020
	62	89,5	2020
	58	89,5	2020
	85	89,25	2020
	113	89,25	2020
	150	88,5	2020
	198	88,5	2020
	39	87,75	2020
	143	87,75	2020
	22	87,5	2020
	184	87	2020
	170	86,5	2020
	110	86,5	2020
	25	86,25	2020
	81	86,25	2020
	245	86,25	2020
	113	86,25	2020
	37	86,25	2020
	32	86	2020
	100	86	2020
	170	85,5	2020
	102	85,5	2020
	77	85,25	2020
	113	85,25	2020
	156	85	2020
	96	85	2020
	52	85	2020
	79	84,75	2020
	139	84,75	2020
	190	84,5	2020
	130	84,5	2020
	77	84,25	2020
	60	84	2020
	63	83,75	2020
	69	83,25	2020
	72	83	2020
	47	82,75	2020
	131	82,75	2020
	231	82,75	2020
	114	82,5	2020
	153	82,25	2020
	77	82,25	2020
	229	82,25	2020
	96	82	2020
	87	81,75	2020
	138	81,5	2020

	45	81,25	2020
	184	81	2020
	42	80,5	2020
	49	80,25	2020
	84	80	2020
	139	79,75	2020
	62	79,5	2020
	32	79	2020
	123	78,75	2020
	58	78,5	2020
	66	78,5	2020
	29	78,25	2020
	64	78	2020
	80	78	2020
	127	77,75	2020
	34	77,5	2020
	53	77,25	2020
	37	77,25	2020
	109	77,25	2020
	104	77	2020
	51	76,75	2020
	115	76,75	2020
	6	76,5	2020
	46	76,5	2020
	109	76,25	2020
	113	76,25	2020
	64	76	2020
	103	75,75	2020
	57	75,25	2020
	101	75,25	2020
	41	75,25	2020
	81	75,25	2020
	104	75	2020
	75	74,75	2020
	95	74,75	2020
	58	74,5	2020
	57	74,25	2020
	100	74	2020
	36	74	2020
	142	73,5	2020
	119	72,75	2020
	79	72,75	2020
	87	72,75	2020
	63	72,75	2020
	95	72,75	2020
	133	72,25	2020
	60	72	2020
	27	71,75	2020
	107	71,75	2020
	110	71,5	2020

	66	71,5	2020
	75	70,75	2020
	74	70,5	2020
	101	70,25	2020
	73	70,25	2020
	61	70,25	2020
	16	70	2020
	68	70	2020
	100	70	2020
	68	70	2020
	152	70	2020
	39	69,75	2020
	82	69,5	2020
	38	69,5	2020
	38	69,5	2020
	109	69,25	2020
	140	69	2020
	40	69	2020
	56	69	2020
	64	69	2020
	83	68,75	2020
	55	68,75	2020
	46	68,5	2020
	54	68,5	2020
	28	68	2020
	16	68	2020
	99	67,75	2020
	150	67,5	2020
	74	67,5	2020
	66	67,5	2020
	76	67	2020
	48	67	2020
	55	66,75	2020
	119	66,75	2020
	55	66,75	2020
	13	66,25	2020
	109	66,25	2020
	125	66,25	2020
	45	66,25	2020
	68	66	2020
	0	66	2020
	52	66	2020
	40	66	2020
	100	66	2020
	63	65,75	2020
	75	65,75	2020
	50	65,5	2020
	174	65,5	2020
	81	65,25	2020
	92	65	2020

	120	65	2020
	119	64,75	2020
	110	64,5	2020
	78	64,5	2020
	82	64,5	2020
	137	64,25	2020
	9	64,25	2020
	81	64,25	2020
	53	64,25	2020
	80	64	2020
	84	64	2020
	42	63,5	2020
	110	63,5	2020
	21	63,25	2020
	37	63,25	2020
	72	63	2020
	15	62,75	2020
	103	62,75	2020
	103	62,75	2020
	110	62,5	2020
	62	62,5	2020
	68	62	2020
	0	62	2020
	54	61,5	2020
	54	61,5	2020
	58	61,5	2020
	69	61,25	2020
	48	61	2020
	20	61	2020
	15	60,75	2020
	71	60,75	2020
	71	60,75	2020
	66	60,5	2020
	82	60,5	2020
	54	60,5	2020
	38	60,5	2020
	73	60,25	2020
	105	60,25	2020
	17	60,25	2020
	57	60,25	2020
	0	60	2020
	12	60	2020
	55	59,75	2020
	46	59,5	2020
	34	59,5	2020
	6	59,5	2020
	62	59,5	2020
	45	59,25	2020
	76	59	2020
	108	59	2020

	76	59	2020
	80	59	2020
	76	58	2020
	20	58	2020
	131	57,75	2020
	123	57,75	2020
	93	57,25	2020
	48	57	2020
	143	56,75	2020
	38	56,5	2020
	206	56,5	2020
	105	56,25	2020
	129	56,25	2020
	104	56	2020
	27	55,75	2020
	99	55,75	2020
	127	55,75	2020
	99	55,75	2020
	54	55,5	2020
	18	55,5	2020
	30	55,5	2020
	62	55,5	2020
	50	55,5	2020
	28	55	2020
	99	54,75	2020
	79	54,75	2020
	58	54,5	2020
	97	54,25	2020
	29	54,25	2020
	93	54,25	2020
	80	54	2020
	71	53,75	2020
	95	53,75	2020
	35	53,75	2020
	79	53,75	2020
	39	53,75	2020
	111	53,75	2020
	47	53,75	2020
	30	53,5	2020
	54	53,5	2020
	73	53,25	2020
	77	53,25	2020
	77	53,25	2020
	12	53	2020
	32	53	2020
	112	53	2020
	92	53	2020
	28	53	2020
	80	53	2020
	15	52,75	2020

	67	52,75	2020
	54	52,5	2020
	49	52,25	2020
	45	52,25	2020
	45	52,25	2020
	73	52,25	2020
	51	51,75	2020
	166	51,5	2020
	57	51,25	2020
	49	51,25	2020
	49	51,25	2020
	125	51,25	2020
	53	51,25	2020
	41	51,25	2020
	97	51,25	2020
	48	51	2020
	52	51	2020
	0	51	2020
	80	51	2020
	31	50,75	2020
	15	50,75	2020
	54	50,5	2020
	26	50,5	2020
	142	50,5	2020
	37	50,25	2020
	37	50,25	2020
	37	50,25	2020
	61	50,25	2020
	40	50	2020
	20	50	2020
	28	50	2020
	84	50	2020
	56	50	2020
	7	49,75	2020
	35	49,75	2020
	46	49,5	2020
	34	49,5	2020
	58	49,5	2020
	49	49,25	2020
	57	49,25	2020
	56	49	2020
	119	48,75	2020
	39	48,75	2020
	154	48,5	2020
	98	48,5	2020
	61	48,25	2020
	9	48,25	2020
	56	48	2020
	76	48	2020
	20	48	2020

	76	48	2020
	63	47,75	2020
	67	47,75	2020
	50	47,5	2020
	50	47,5	2020
	29	47,25	2020
	65	47,25	2020
	120	47	2020
	32	47	2020
	48	47	2020
	31	46,75	2020
	127	46,75	2020
	30	46,5	2020
	78	46,5	2020
	22	46,5	2020
	22	46,5	2020
	50	46,5	2020
	25	46,25	2020
	77	46,25	2020
	104	46	2020
	88	46	2020
	43	45,75	2020
	31	45,75	2020
	99	45,75	2020
	71	45,75	2020
	41	45,25	2020
	33	45,25	2020
	61	45,25	2020
	40	45	2020
	52	45	2020
	60	45	2020
	40	45	2020
	72	45	2020
	51	44,75	2020
	91	44,75	2020
	39	44,75	2020
	23	44,75	2020
	19	44,75	2020
	31	44,75	2020
	82	44,5	2020
	30	44,5	2020
	17	44,25	2020
	17	44,25	2020
	25	44,25	2020
	25	44,25	2020
	28	44	2020
	92	44	2020
	79	43,75	2020
	23	43,75	2020
	39	43,75	2020

	47	43,75	2020
	47	43,75	2020
	6	43,5	2020
	38	43,5	2020
	18	43,5	2020
	65	43,25	2020
	9	43,25	2020
	44	43	2020
	116	43	2020
	3	42,75	2020
	31	42,75	2020
	39	42,75	2020
	87	42,75	2020
	47	42,75	2020
	55	42,75	2020
	87	42,75	2020
	74	42,5	2020
	61	42,25	2020
	40	42	2020
	39	41,75	2020
	10	41,5	2020
	46	41,5	2020
	50	41,5	2020
	61	41,25	2020
	101	41,25	2020
	29	41,25	2020
	136	41	2020
	28	41	2020
	55	40,75	2020
	27	40,75	2020
	51	40,75	2020
	46	40,5	2020
	66	40,5	2020
	61	40,25	2020
	25	40,25	2020
	29	40,25	2020
	41	40,25	2020
	16	40	2020
	112	40	2020
	88	40	2020
	12	40	2020
	36	40	2020
	96	40	2020
	47	39,75	2020
	75	39,75	2020
	111	39,75	2020
	42	39,5	2020
	54	39,5	2020
	58	39,5	2020
	42	39,5	2020

	66	39,5	2020
	41	39,25	2020
	53	39,25	2020
	37	39,25	2020
	29	39,25	2020
	33	39,25	2020
	45	39,25	2020
	60	39	2020
	68	39	2020
	59	38,75	2020
	47	38,75	2020
	66	38,5	2020
	70	38,5	2020
	34	38,5	2020
	78	38,5	2020
	90	38,5	2020
	98	38,5	2020
	13	38,25	2020
	29	38,25	2020
	13	38,25	2020
	13	38,25	2020
	33	38,25	2020
	29	38,25	2020
	37	38,25	2020
	12	38	2020
	52	38	2020
	56	38	2020
	24	38	2020
	68	38	2020
	7	37,75	2020
	15	37,75	2020
	55	37,75	2020
	27	37,75	2020
	47	37,75	2020
	55	37,75	2020
	67	37,75	2020
	26	37,5	2020
	90	37,5	2020
	66	37,5	2020
	34	37,5	2020
	38	37,5	2020
	38	37,5	2020
	34	37,5	2020
	13	37,25	2020
	48	37	2020
	24	37	2020
	32	37	2020
	71	36,75	2020
	19	36,75	2020
	19	36,75	2020

	43	36,75	2020
	38	36,5	2020
	6	36,5	2020
	74	36,5	2020
	21	36,25	2020
	24	36	2020
	36	36	2020
	76	36	2020
	88	36	2020
	64	36	2020
	43	35,75	2020
	59	35,75	2020
	95	35,75	2020
	38	35,5	2020
	46	35,5	2020
	66	35,5	2020
	90	35,5	2020
	65	35,25	2020
	52	35	2020
	36	35	2020
	84	35	2020
	0	35	2020
	12	35	2020
	39	34,75	2020
	38	34,5	2020
	42	34,5	2020
	34	34,5	2020
	49	34,25	2020
	45	34,25	2020
	53	34,25	2020
	25	34,25	2020
	56	34	2020
	24	34	2020
	3	33,75	2020
	19	33,75	2020
	75	33,75	2020
	26	33,5	2020
	26	33,5	2020
	78	33,5	2020
	6	33,5	2020
	54	33,5	2020
	41	33,25	2020
	12	33	2020
	52	33	2020
	15	32,75	2020
	27	32,75	2020
	51	32,75	2020
	31	32,75	2020
	30	32,5	2020
	62	32,5	2020

	82	32,5	2020
	30	32,5	2020
	46	32,5	2020
	82	32,5	2020
	114	32,5	2020
	33	32,25	2020
	13	32,25	2020
	81	32,25	2020
	109	32,25	2020
	68	32	2020
	31	31,75	2020
	31	31,75	2020
	23	31,75	2020
	27	31,75	2020
	79	31,75	2020
	30	31,5	2020
	38	31,5	2020
	54	31,5	2020
	29	31,25	2020
	13	31,25	2020
	29	31,25	2020
	45	31,25	2020
	9	31,25	2020
	28	31	2020
	20	31	2020
	23	30,75	2020
	43	30,75	2020
	39	30,75	2020
	27	30,75	2020
	3	30,75	2020
	83	30,75	2020
	46	30,5	2020
	38	30,5	2020
	46	30,5	2020
	34	30,5	2020
	33	30,25	2020
	0	30	2020
	36	30	2020
	56	30	2020
	16	30	2020
	28	30	2020
	56	30	2020
	112	30	2020
	7	29,75	2020
	31	29,75	2020
	59	29,75	2020
	31	29,75	2020
	3	29,75	2020
	47	29,75	2020
	59	29,75	2020

	6	29,5	2020
	78	29,5	2020
	78	29,5	2020
	22	29,5	2020
	22	29,5	2020
	30	29,5	2020
	17	29,25	2020
	17	29,25	2020
	37	29,25	2020
	81	29,25	2020
	41	29,25	2020
	32	29	2020
	60	29	2020
	68	29	2020
	0	29	2020
	0	29	2020
	16	29	2020
	28	29	2020
	3	28,75	2020
	7	28,75	2020
	55	28,75	2020
	7	28,75	2020
	3	28,75	2020
	35	28,75	2020
	27	28,75	2020
	55	28,75	2020
	42	28,5	2020
	30	28,5	2020
	45	28,25	2020
	33	28,25	2020
	65	28,25	2020
	65	28,25	2020
	25	28,25	2020
	57	28,25	2020
	36	28	2020
	0	28	2020
	20	28	2020
	15	27,75	2020
	31	27,75	2020
	39	27,75	2020
	27	27,75	2020
	35	27,75	2020
	19	27,75	2020
	39	27,75	2020
	31	27,75	2020
	59	27,75	2020
	14	27,5	2020
	6	27,5	2020
	62	27,5	2020
	62	27,5	2020

	17	27,25	2020
	29	27,25	2020
	20	27	2020
	36	27	2020
	24	27	2020
	16	27	2020
	68	27	2020
	64	27	2020
	27	26,75	2020
	59	26,75	2020
	47	26,75	2020
	14	26,5	2020
	14	26,5	2020
	30	26,5	2020
	42	26,5	2020
	26	26,5	2020
	9	26,25	2020
	29	26,25	2020
	13	26,25	2020
	13	26,25	2020
	77	26,25	2020
	105	26,25	2020
	28	26	2020
	20	26	2020
	40	26	2020
	19	25,75	2020
	23	25,75	2020
	39	25,75	2020
	21	25,25	2020
	21	25,25	2020
	13	25,25	2020
	13	25,25	2020
	57	25,25	2020
	0	25	2020
	12	25	2020
	20	25	2020
	16	25	2020
	40	25	2020
	12	25	2020
	20	25	2020
	28	25	2020
	56	25	2020
	59	24,75	2020
	27	24,75	2020
	59	24,75	2020
	34	24,5	2020
	26	24,5	2020
	50	24,5	2020
	9	24,25	2020
	13	24,25	2020

	13	24,25	2020
	57	24,25	2020
	9	24,25	2020
	29	24,25	2020
	0	24	2020
	0	24	2020
	20	24	2020
	28	24	2020
	40	24	2020
	48	24	2020
	12	24	2020
	24	24	2020
	44	24	2020
	31	23,75	2020
	23	23,75	2020
	47	23,75	2020
	71	23,75	2020
	18	23,5	2020
	14	23,5	2020
	34	23,5	2020
	34	23,5	2020
	46	23,5	2020
	22	23,5	2020
	46	23,5	2020
	58	23,5	2020
	18	23,5	2020
	46	23,5	2020
	33	23,25	2020
	37	23,25	2020
	21	23,25	2020
	37	23,25	2020
	53	23,25	2020
	20	23	2020
	16	23	2020
	16	23	2020
	52	23	2020
	20	23	2020
	36	23	2020
	20	23	2020
	36	23	2020
	23	22,75	2020
	23	22,75	2020
	43	22,75	2020
	27	22,75	2020
	35	22,75	2020
	43	22,75	2020
	31	22,75	2020
	47	22,75	2020
	47	22,75	2020
	47	22,75	2020

	47	22,75	2020
	26	22,5	2020
	10	22,5	2020
	22	22,5	2020
	18	22,5	2020
	30	22,5	2020
	38	22,5	2020
	50	22,5	2020
	38	22,5	2020
	82	22,5	2020
	17	22,25	2020
	33	22,25	2020
	33	22,25	2020
	13	22,25	2020
	9	22,25	2020
	53	22,25	2020
	24	22	2020
	20	22	2020
	20	22	2020
	16	22	2020
	0	22	2020
	28	22	2020
	27	21,75	2020
	3	21,75	2020
	19	21,75	2020
	3	21,75	2020
	23	21,75	2020
	31	21,75	2020
	31	21,75	2020
	47	21,75	2020
	10	21,5	2020
	6	21,5	2020
	38	21,5	2020
	86	21,5	2020
	9	21,25	2020
	9	21,25	2020
	45	21,25	2020
	29	21,25	2020
	57	21,25	2020
	65	21,25	2020
	9	21,25	2020
	12	21	2020
	44	21	2020
	40	21	2020
	0	21	2020
	0	21	2020
	28	21	2020
	35	20,75	2020
	15	20,75	2020
	3	20,75	2020

	7	20,75	2020
	55	20,75	2020
	31	20,75	2020
	39	20,75	2020
	55	20,75	2020
	59	20,75	2020
	83	20,75	2020
	6	20,5	2020
	6	20,5	2020
	6	20,5	2020
	22	20,5	2020
	10	20,5	2020
	54	20,5	2020
	66	20,5	2020
	13	20,25	2020
	21	20,25	2020
	25	20,25	2020
	41	20,25	2020
	33	20,25	2020
	45	20,25	2020
	61	20,25	2020
	9	20,25	2020
	57	20,25	2020
	0	20	2020
	0	20	2020
	0	20	2020
	20	20	2020
	12	20	2020
	0	20	2020
	20	20	2020
	16	20	2020
	24	20	2020
	32	20	2020
	40	20	2020
	19	19,75	2020
	15	19,75	2020
	3	19,75	2020
	23	19,75	2020
	39	19,75	2020
	23	19,75	2020
	43	19,75	2020
	35	19,75	2020
	47	19,75	2020
	51	19,75	2020
	30	19,5	2020
	14	19,5	2020
	58	19,5	2020
	18	19,5	2020
	34	19,5	2020
	50	19,5	2020

	21	19,25	2020
	21	19,25	2020
	25	19,25	2020
	0	19	2020
	28	19	2020
	48	19	2020
	52	19	2020
	52	19	2020
	19	18,75	2020
	15	18,75	2020
	15	18,75	2020
	3	18,75	2020
	3	18,75	2020
	3	18,75	2020
	3	18,75	2020
	3	18,75	2020
	19	18,75	2020
	31	18,75	2020
	67	18,75	2020
	75	18,75	2020
	14	18,5	2020
	6	18,5	2020
	18	18,5	2020
	6	18,5	2020
	18	18,5	2020
	22	18,5	2020
	54	18,5	2020
	66	18,5	2020
	13	18,25	2020
	13	18,25	2020
	45	18,25	2020
	9	18,25	2020
	9	18,25	2020
	25	18,25	2020
	33	18,25	2020
	45	18,25	2020
	49	18,25	2020
	0	18	2020
	32	18	2020
	16	18	2020
	16	18	2020
	44	18	2020
	56	18	2020
	64	18	2020
	3	17,75	2020
	23	17,75	2020
	31	17,75	2020
	7	17,75	2020
	19	17,75	2020
	43	17,75	2020

	31	17,75	2020
	10	17,5	2020
	14	17,5	2020
	6	17,5	2020
	6	17,5	2020
	34	17,5	2020
	50	17,5	2020
	21	17,25	2020
	13	17,25	2020
	21	17,25	2020
	13	17,25	2020
	41	17,25	2020
	13	17,25	2020
	21	17,25	2020
	61	17,25	2020
	0	17	2020
	20	17	2020
	20	17	2020
	28	17	2020
	20	17	2020
	40	17	2020
	16	17	2020
	40	17	2020
	68	17	2020
	27	16,75	2020
	27	16,75	2020
	7	16,75	2020
	27	16,75	2020
	23	16,75	2020
	3	16,75	2020
	47	16,75	2020
	67	16,75	2020
	67	16,75	2020
	6	16,5	2020
	6	16,5	2020
	18	16,5	2020
	10	16,5	2020
	22	16,5	2020
	26	16,5	2020
	18	16,5	2020
	22	16,5	2020
	38	16,5	2020
	45	16,25	2020
	13	16,25	2020
	25	16,25	2020
	37	16,25	2020
	45	16,25	2020
	57	16,25	2020
	65	16,25	2020
	16	16	2020

	0	16	2020
	16	16	2020
	20	16	2020
	24	16	2020
	0	16	2020
	16	16	2020
	16	16	2020
	28	16	2020
	36	16	2020
	7	15,75	2020
	3	15,75	2020
	15	15,75	2020
	7	15,75	2020
	27	15,75	2020
	15	15,75	2020
	19	15,75	2020
	47	15,75	2020
	63	15,75	2020
	26	15,5	2020
	6	15,5	2020
	6	15,5	2020
	6	15,5	2020
	6	15,5	2020
	6	15,5	2020
	18	15,5	2020
	18	15,5	2020
	26	15,5	2020
	54	15,5	2020
	13	15,25	2020
	25	15,25	2020
	25	15,25	2020
	13	15,25	2020
	17	15,25	2020
	25	15,25	2020
	25	15,25	2020
	25	15,25	2020
	61	15,25	2020
	0	15	2020
	0	15	2020
	0	15	2020
	12	15	2020
	20	15	2020
	0	15	2020
	40	15	2020
	12	15	2020
	16	15	2020
	52	15	2020
	52	15	2020
	52	15	2020
	3	14,75	2020

	19	14,75	2020
	39	14,75	2020
	23	14,75	2020
	39	14,75	2020
	39	14,75	2020
	15	14,75	2020
	51	14,75	2020
	51	14,75	2020
	18	14,5	2020
	6	14,5	2020
	6	14,5	2020
	18	14,5	2020
	22	14,5	2020
	22	14,5	2020
	22	14,5	2020
	22	14,5	2020
	50	14,5	2020
	9	14,25	2020
	9	14,25	2020
	13	14,25	2020
	13	14,25	2020
	0	14	2020
	16	14	2020
	12	14	2020
	28	14	2020
	0	14	2020
	0	14	2020
	28	14	2020
	28	14	2020
	32	14	2020
	40	14	2020
	40	14	2020
	7	13,75	2020
	15	13,75	2020
	7	13,75	2020
	15	13,75	2020
	7	13,75	2020
	27	13,75	2020
	19	13,75	2020
	47	13,75	2020
	6	13,5	2020
	14	13,5	2020
	14	13,5	2020
	14	13,5	2020
	6	13,5	2020
	6	13,5	2020
	26	13,5	2020
	6	13,5	2020
	6	13,5	2020
	18	13,5	2020

	18	13,5	2020
	26	13,5	2020
	34	13,5	2020
	38	13,5	2020
	54	13,5	2020
	25	13,25	2020
	13	13,25	2020
	13	13,25	2020
	25	13,25	2020
	9	13,25	2020
	9	13,25	2020
	13	13,25	2020
	13	13,25	2020
	17	13,25	2020
	25	13,25	2020
	25	13,25	2020
	9	13,25	2020
	9	13,25	2020
	13	13,25	2020
	13	13,25	2020
	13	13,25	2020
	25	13,25	2020
	37	13,25	2020
	37	13,25	2020
	45	13,25	2020
	12	13	2020
	16	13	2020
	0	13	2020
	0	13	2020
	12	13	2020
	16	13	2020
	3	12,25	2020
	3	12,25	2020
	3	12,25	2020
	3	12,25	2020
	3	12,25	2020
	3	12,25	2020
	3	12,25	2020
	15	12,25	2020
	23	12,25	2020
	15	12,25	2020
	31	12,25	2020
	51	12,25	2020
	10	12,5	2020
	10	12,5	2020
	10	12,5	2020
	30	12,5	2020
	6	12,5	2020
	6	12,5	2020
	6	12,5	2020

	6	12,5	2020
	6	12,5	2020
	6	12,5	2020
	6	12,5	2020
	6	12,5	2020
	22	12,5	2020
	22	12,5	2020
	26	12,5	2020
	30	12,5	2020
	9	12,25	2020
	9	12,25	2020
	13	12,25	2020
	9	12,25	2020
	9	12,25	2020
	13	12,25	2020
	13	12,25	2020
	13	12,25	2020
	13	12,25	2020
	33	12,25	2020
	41	12,25	2020
	0	12	2020
	0	12	2020
	0	12	2020
	0	12	2020
	20	12	2020
	28	12	2020
	12	12	2020
	12	12	2020
	24	12	2020
	28	12	2020
	40	12	2020
	7	11,75	2020
	7	11,75	2020
	7	11,75	2020
	7	11,75	2020
	7	11,75	2020
	7	11,75	2020
	3	11,75	2020
	7	11,75	2020
	7	11,75	2020
	19	11,75	2020
	27	11,75	2020
	3	11,75	2020
	3	11,75	2020
	3	11,75	2020
	3	11,75	2020
	19	11,75	2020
	19	11,75	2020
	31	11,75	2020
	6	11,5	2020

	10	11,5	2020
	10	11,5	2020
	10	11,5	2020
	6	11,5	2020
	6	11,5	2020
	6	11,5	2020
	22	11,5	2020
	30	11,5	2020
	38	11,5	2020
	9	11,25	2020
	17	11,25	2020
	25	11,25	2020
	25	11,25	2020
	9	11,25	2020
	9	11,25	2020
	9	11,25	2020
	9	11,25	2020
	9	11,25	2020
	45	11,25	2020
	0	11	2020
	0	11	2020
	24	11	2020
	16	11	2020
	16	11	2020
	16	11	2020
	28	11	2020
	3	10,75	2020
	3	10,75	2020
	3	10,75	2020
	3	10,75	2020
	3	10,75	2020
	7	10,75	2020
	15	10,75	2020
	3	10,75	2020
	15	10,75	2020
	15	10,75	2020
	15	10,75	2020
	27	10,75	2020
	35	10,75	2020
	6	10,5	2020
	6	10,5	2020
	6	10,5	2020
	14	10,5	2020
	14	10,5	2020
	6	10,5	2020
	6	10,5	2020
	6	10,5	2020
	14	10,5	2020
	22	10,5	2020
	22	10,5	2020

	13	10,25	2020
	13	10,25	2020
	13	10,25	2020
	13	10,25	2020
	9	10,25	2020
	9	10,25	2020
	13	10,25	2020
	21	10,25	2020
	25	10,25	2020
	33	10,25	2020
	33	10,25	2020
	0	10	2020
	0	10	2020
	0	10	2020
	0	10	2020
	0	10	2020
	0	10	2020
	0	10	2020
	0	10	2020
	0	10	2020
	0	10	2020
	0	10	2020
	0	10	2020
	0	10	2020
	20	10	2020
	20	10	2020
	20	10	2020
	20	10	2020
	0	10	2020
	0	10	2020
	12	10	2020
	12	10	2020
	16	10	2020
	16	10	2020
	16	10	2020
	32	10	2020
	3	9,75	2020
	3	9,75	2020
	19	9,75	2020
	3	9,75	2020
	3	9,75	2020
	3	9,75	2020
	3	9,75	2020
	3	9,75	2020
	3	9,75	2020
	15	9,75	2020
	19	9,75	2020
	10	9,5	2020
	6	9,5	2020
	10	9,5	2020

	10	9,5	2020
	10	9,5	2020
	10	9,5	2020
	22	9,5	2020
	22	9,5	2020
	22	9,5	2020
	38	9,5	2020
	9	9,25	2020
	17	9,25	2020
	9	9,25	2020
	9	9,25	2020
	9	9,25	2020
	9	9,25	2020
	9	9,25	2020
	21	9,25	2020
	37	9,25	2020
	37	9,25	2020
	37	9,25	2020
	37	9,25	2020
	0	9	2020
	0	9	2020
	16	9	2020
	16	9	2020
	0	9	2020
	0	9	2020
	0	9	2020
	0	9	2020
	0	9	2020
	0	9	2020
	0	9	2020
	0	9	2020
	0	9	2020
	0	9	2020
	0	9	2020
	12	9	2020
	12	9	2020
	12	9	2020
	16	9	2020
	28	9	2020
	15	8,75	2020
	7	8,75	2020
	7	8,75	2020
	7	8,75	2020
	7	8,75	2020
	7	8,75	2020
	7	8,75	2020
	15	8,75	2020
	15	8,75	2020
	3	8,75	2020
	15	8,75	2020

	15	8,75	2020
	19	8,75	2020
	19	8,75	2020
	19	8,75	2020
	19	8,75	2020
	19	8,75	2020
	19	8,75	2020
	6	8,5	2020
	14	8,5	2020
	6	8,5	2020
	6	8,5	2020
	6	8,5	2020
	6	8,5	2020
	6	8,5	2020
	14	8,5	2020
	14	8,5	2020
	14	8,5	2020
	14	8,5	2020
	14	8,5	2020
	14	8,5	2020
	6	8,5	2020
	6	8,5	2020
	14	8,5	2020
	14	8,5	2020
	14	8,5	2020
	18	8,5	2020
	18	8,5	2020
	26	8,5	2020
	26	8,5	2020
	26	8,5	2020
	13	8,25	2020
	13	8,25	2020
	13	8,25	2020
	13	8,25	2020
	9	8,25	2020
	9	8,25	2020
	9	8,25	2020
	9	8,25	2020
	13	8,25	2020
	25	8,25	2020
	25	8,25	2020
	25	8,25	2020
	12	8	2020
	12	8	2020
	12	8	2020
	12	8	2020
	12	8	2020
	12	8	2020
	16	8	2020
	16	8	2020

	16	8	2020
	32	8	2020
	3	7,75	2020
	3	7,75	2020
	3	7,75	2020
	3	7,75	2020
	3	7,75	2020
	3	7,75	2020
	3	7,75	2020
	3	7,75	2020
	3	7,75	2020
	3	7,75	2020
	3	7,75	2020
	3	7,75	2020
	15	7,75	2020
	23	7,75	2020
	23	7,75	2020
	31	7,75	2020
	10	7,5	2020
	10	7,5	2020
	10	7,5	2020
	10	7,5	2020
	10	7,5	2020
	6	7,5	2020
	6	7,5	2020
	6	7,5	2020
	6	7,5	2020
	10	7,5	2020
	22	7,5	2020
	22	7,5	2020
	30	7,5	2020
	30	7,5	2020
	30	7,5	2020
	30	7,5	2020
	9	7,25	2020
	9	7,25	2020
	9	7,25	2020
	9	7,25	2020
	9	7,25	2020
	9	7,25	2020
	9	7,25	2020
	9	7,25	2020
	9	7,25	2020
	13	7,25	2020
	13	7,25	2020
	13	7,25	2020
	13	7,25	2020
	13	7,25	2020
	21	7,25	2020
	21	7,25	2020

	21	7,25	2020
	29	7,25	2020
	29	7,25	2020
	0	7	2020
	0	7	2020
	0	7	2020
	0	7	2020
	0	7	2020
	0	7	2020
	0	7	2020
	0	7	2020
	0	7	2020
	0	7	2020
	0	7	2020
	0	7	2020
	0	7	2020
	0	7	2020
	0	7	2020
	0	7	2020
	0	7	2020
	0	7	2020
	0	7	2020
	0	7	2020
	12	7	2020
	12	7	2020
	12	7	2020
	20	7	2020
	28	7	2020
	28	7	2020
	28	7	2020
	28	7	2020
	28	7	2020
	28	7	2020
	28	7	2020
	28	7	2020
	7	6,75	2020
	7	6,75	2020
	7	6,75	2020
	7	6,75	2020
	7	6,75	2020
	7	6,75	2020
	7	6,75	2020
	7	6,75	2020
	7	6,75	2020
	7	6,75	2020
	7	6,75	2020
	7	6,75	2020
	7	6,75	2020
	3	6,75	2020
	3	6,75	2020

	3	6,75	2020
	7	6,75	2020
	7	6,75	2020
	19	6,75	2020
	19	6,75	2020
	19	6,75	2020
	27	6,75	2020
	27	6,75	2020
	27	6,75	2020
	27	6,75	2020
	27	6,75	2020
	27	6,75	2020
	27	6,75	2020
	27	6,75	2020
	27	6,75	2020
	6	6,5	2020
	6	6,5	2020
	6	6,5	2020
	6	6,5	2020
	6	6,5	2020
	6	6,5	2020
	6	6,5	2020
	6	6,5	2020
	10	6,5	2020
	18	6,5	2020
	26	6,5	2020
	26	6,5	2020
	9	6,25	2020
	9	6,25	2020
	9	6,25	2020
	9	6,25	2020
	9	6,25	2020
	9	6,25	2020
	9	6,25	2020
	9	6,25	2020
	9	6,25	2020
	9	6,25	2020
	9	6,25	2020
	17	6,25	2020
	17	6,25	2020
	17	6,25	2020
	17	6,25	2020
	25	6,25	2020
	25	6,25	2020
	25	6,25	2020
	0	6	2020
	0	6	2020
	16	6	2020
	16	6	2020
	16	6	2020

16	6	2020
16	6	2020
16	6	2020
24	6	2020
24	6	2020
3	5,75	2020
3	5,75	2020
3	5,75	2020
3	5,75	2020
3	5,75	2020
3	5,75	2020
3	5,75	2020
3	5,75	2020
3	5,75	2020
3	5,75	2020
3	5,75	2020
3	5,75	2020
3	5,75	2020
3	5,75	2020
3	5,75	2020
3	5,75	2020
3	5,75	2020
3	5,75	2020
3	5,75	2020
3	5,75	2020
3	5,75	2020
3	5,75	2020
3	5,75	2020
3	5,75	2020
3	5,75	2020
3	5,75	2020
3	5,75	2020
3	5,75	2020
3	5,75	2020
3	5,75	2020
3	5,75	2020
3	5,75	2020
7	5,75	2020
7	5,75	2020
7	5,75	2020
15	5,75	2020
15	5,75	2020
15	5,75	2020
15	5,75	2020
15	5,75	2020
23	5,75	2020
23	5,75	2020
6	5,5	2020
6	5,5	2020
6	5,5	2020

	6	5,5	2020
	6	5,5	2020
	6	5,5	2020
	6	5,5	2020
	6	5,5	2020
	6	5,5	2020
	6	5,5	2020
	6	5,5	2020
	6	5,5	2020
	6	5,5	2020
	6	5,5	2020
	6	5,5	2020
	6	5,5	2020
	6	5,5	2020
	6	5,5	2020
	6	5,5	2020
	6	5,5	2020
	6	5,5	2020
	6	5,5	2020
	14	5,5	2020
	14	5,5	2020
	14	5,5	2020
	14	5,5	2020
	22	5,5	2020
	22	5,5	2020
	22	5,5	2020
	22	5,5	2020
	13	1,5	2020
	13	1,5	2020
	13	1,5	2020
	13	1,5	2020
	13	1,5	2020
	13	1,5	2020
	13	1,5	2020
	13	1,5	2020
	13	1,5	2020
	13	1,5	2020
	13	1,5	2020
	13	1,5	2020
	13	1,5	2020
	13	1,5	2020
	13	1,5	2020
	13	1,5	2020
	13	1,5	2020
	13	1,5	2020
	13	1,5	2020
	13	1,5	2020
	21	1,5	2020
	21	1,5	2020
	21	1,5	2020

21	1,5	2020
21	1,5	2020
0	5	2020
0	5	2020
0	5	2020
0	5	2020
0	5	2020
0	5	2020
0	5	2020
0	5	2020
0	5	2020
0	5	2020
0	5	2020
0	5	2020
0	5	2020
0	5	2020
0	5	2020
0	5	2020
0	5	2020
0	5	2020
0	5	2020
0	5	2020
0	5	2020
0	5	2020
0	5	2020
0	5	2020
0	5	2020
0	5	2020
0	5	2020
0	5	2020
0	5	2020
0	5	2020
0	5	2020
0	5	2020
0	5	2020
0	5	2020
0	5	2020
0	5	2020
0	5	2020
0	5	2020
0	5	2020
0	5	2020
0	5	2020
0	5	2020
0	5	2020
0	5	2020
0	5	2020
0	5	2020
0	5	2020
0	5	2020

0	5	2020
0	5	2020
0	5	2020
0	5	2020
0	5	2020
0	5	2020
0	5	2020
0	5	2020
0	5	2020
0	5	2020
0	5	2020
0	5	2020
0	5	2020
0	5	2020
0	5	2020
0	5	2020
0	5	2020
0	5	2020
0	5	2020
0	5	2020
0	5	2020
0	5	2020
0	5	2020
0	5	2020
0	5	2020
0	5	2020
0	5	2020
0	5	2020
0	5	2020
0	5	2020
0	5	2020
0	5	2020
0	5	2020
0	5	2020
12	5	2020
12	5	2020
12	5	2020
12	5	2020
12	5	2020
12	5	2020
12	5	2020
12	5	2020
12	5	2020
20	5	2020
20	5	2020
20	5	2020
20	5	2020
20	5	2020
20	5	2020
20	5	2020

	3	4,75	2020
	3	4,75	2020
	3	4,75	2020
	3	4,75	2020
	3	4,75	2020
	3	4,75	2020
	3	4,75	2020
	3	4,75	2020
	3	4,75	2020
	3	4,75	2020
	3	4,75	2020
	3	4,75	2020
	3	4,75	2020
	3	4,75	2020
	3	4,75	2020
	3	4,75	2020
	3	4,75	2020
	3	4,75	2020
	3	4,75	2020
	3	4,75	2020
	19	4,75	2020
	19	4,75	2020
	19	4,75	2020
	19	4,75	2020
	10	4,5	2020
	10	4,5	2020
	10	4,5	2020
	10	4,5	2020
	10	4,5	2020
	10	4,5	2020
	10	4,5	2020
	10	4,5	2020
	10	4,5	2020
	18	4,5	2020
	18	4,5	2020
	18	4,5	2020
	18	4,5	2020
	18	4,5	2020
	9	4,25	2020
	9	4,25	2020
	9	4,25	2020
	9	4,25	2020
	9	4,25	2020
	9	4,25	2020
	9	4,25	2020
	9	4,25	2020
	9	4,25	2020
	9	4,25	2020
	9	4,25	2020
	9	4,25	2020

	9	4,25	2020
	9	4,25	2020
	9	4,25	2020
	9	4,25	2020
	9	4,25	2020
	9	4,25	2020
	9	4,25	2020
	9	4,25	2020
	9	4,25	2020
	9	4,25	2020
	9	4,25	2020
	17	4,25	2020
	0	4	2020
	0	4	2020
	0	4	2020
	0	4	2020
	0	4	2020
	0	4	2020
	0	4	2020
	0	4	2020
	0	4	2020
	0	4	2020
	0	4	2020
	0	4	2020
	0	4	2020
	0	4	2020
	0	4	2020
	0	4	2020
	0	4	2020
	0	4	2020
	0	4	2020
	0	4	2020
	0	4	2020
	0	4	2020
	0	4	2020
	0	4	2020
	0	4	2020
	0	4	2020
	0	4	2020
	0	4	2020
	0	4	2020
	0	4	2020
	0	4	2020
	0	4	2020
	16	4	2020
	16	4	2020
	16	4	2020
	16	4	2020
	16	4	2020
	7	3,75	2020

	7	3,75	2020
	7	3,75	2020
	7	3,75	2020
	7	3,75	2020
	7	3,75	2020
	7	3,75	2020
	7	3,75	2020
	7	3,75	2020
	7	3,75	2020
	7	3,75	2020
	7	3,75	2020
	7	3,75	2020
	15	3,75	2020
	15	3,75	2020
	15	3,75	2020
	15	3,75	2020
	15	3,75	2020
	15	3,75	2020
	15	3,75	2020
	15	3,75	2020
	15	3,75	2020
	15	3,75	2020
	15	3,75	2020
	6	3,5	2020
	6	3,5	2020
	6	3,5	2020
	6	3,5	2020
	6	3,5	2020
	6	3,5	2020
	6	3,5	2020
	6	3,5	2020
	6	3,5	2020
	6	3,5	2020
	6	3,5	2020
	6	3,5	2020
	6	3,5	2020
	6	3,5	2020
	6	3,5	2020
	6	3,5	2020
	6	3,5	2020
	6	3,5	2020
	6	3,5	2020
	6	3,5	2020
	6	3,5	2020
	6	3,5	2020
	6	3,5	2020
	6	3,5	2020
	6	3,5	2020
	6	3,5	2020
	6	3,5	2020

	6	3,5	2020
	6	3,5	2020
	6	3,5	2020
	6	3,5	2020
	6	3,5	2020
	6	3,5	2020
	6	3,5	2020
	6	3,5	2020
	6	3,5	2020
	6	3,5	2020
	6	3,5	2020
	6	3,5	2020
	6	3,5	2020
	6	3,5	2020
	6	3,5	2020
	6	3,5	2020
	6	3,5	2020
	6	3,5	2020
	6	3,5	2020
	6	3,5	2020
	14	3,5	2020
	14	3,5	2020
	14	3,5	2020
	14	3,5	2020
	14	3,5	2020
	14	3,5	2020
	14	3,5	2020
	14	3,5	2020
	14	3,5	2020
	14	3,5	2020
	14	3,5	2020
	13	3,25	2020
	13	3,25	2020
	13	3,25	2020
	13	3,25	2020
	13	3,25	2020
	13	3,25	2020
	13	3,25	2020
	13	3,25	2020
	13	3,25	2020
	13	3,25	2020
	13	3,25	2020
	13	3,25	2020
	12	3	2020
	12	3	2020
	12	3	2020
	12	3	2020
	12	3	2020
	12	3	2020
	12	3	2020

3	2,75	2020
3	2,75	2020
3	2,75	2020
3	2,75	2020
3	2,75	2020
3	2,75	2020
3	2,75	2020
3	2,75	2020
3	2,75	2020
3	2,75	2020
3	2,75	2020
3	2,75	2020
3	2,75	2020
3	2,75	2020
3	2,75	2020
3	2,75	2020
3	2,75	2020
3	2,75	2020
3	2,75	2020
3	2,75	2020
3	2,75	2020
3	2,75	2020
3	2,75	2020
3	2,75	2020
3	2,75	2020
3	2,75	2020
3	2,75	2020
3	2,75	2020
3	2,75	2020
3	2,75	2020
3	2,75	2020
3	2,75	2020
3	2,75	2020
3	2,75	2020
3	2,75	2020
3	2,75	2020
3	2,75	2020
3	2,75	2020
3	2,75	2020
3	2,75	2020
3	2,75	2020
3	2,75	2020
3	2,75	2020
3	2,75	2020
3	2,75	2020
3	2,75	2020
3	2,75	2020
3	2,75	2020
3	2,75	2020
3	2,75	2020

3	2,75	2020
3	2,75	2020
3	2,75	2020
3	2,75	2020
3	2,75	2020
3	2,75	2020
3	2,75	2020
3	2,75	2020
3	2,75	2020
3	2,75	2020
3	2,75	2020
3	2,75	2020
3	2,75	2020
3	2,75	2020
3	2,75	2020
3	2,75	2020
3	2,75	2020
3	2,75	2020
3	2,75	2020
3	2,75	2020
3	2,75	2020
3	2,75	2020
3	2,75	2020
3	2,75	2020
3	2,75	2020
3	2,75	2020
3	2,75	2020
3	2,75	2020
3	2,75	2020
3	2,75	2020
3	2,75	2020
3	2,75	2020
3	2,75	2020
3	2,75	2020
3	2,75	2020
3	2,75	2020
3	2,75	2020
3	2,75	2020
3	2,75	2020
3	2,75	2020
3	2,75	2020
3	2,75	2020
3	2,75	2020
3	2,75	2020
3	2,75	2020
3	2,75	2020
3	2,75	2020
3	2,75	2020
3	2,75	2020
3	2,75	2020

3	2,75	2020
3	2,75	2020
3	2,75	2020
3	2,75	2020
3	2,75	2020
3	2,75	2020
3	2,75	2020
3	2,75	2020
3	2,75	2020
3	2,75	2020
3	2,75	2020
3	2,75	2020
3	2,75	2020
3	2,75	2020
3	2,75	2020
3	2,75	2020
3	2,75	2020
3	2,75	2020
3	2,75	2020
10	2,5	2020
10	2,5	2020
10	2,5	2020
10	2,5	2020
10	2,5	2020
10	2,5	2020
10	2,5	2020
10	2,5	2020
10	2,5	2020
10	2,5	2020
10	2,5	2020
10	2,5	2020
10	2,5	2020
10	2,5	2020
10	2,5	2020
10	2,5	2020
10	2,5	2020
10	2,5	2020
10	2,5	2020
10	2,5	2020
10	2,5	2020
10	2,5	2020
9	2,25	2020
9	2,25	2020
9	2,25	2020
9	2,25	2020
9	2,25	2020
9	2,25	2020
9	2,25	2020
9	2,25	2020
9	2,25	2020

	9	2,25	2020
	9	2,25	2020
	9	2,25	2020
	0	2	2020
	0	2	2020
	0	2	2020
	0	2	2020
	0	2	2020
	0	2	2020
	0	2	2020
	0	2	2020
	0	2	2020
	0	2	2020
	0	2	2020
	0	2	2020
	0	2	2020
	0	2	2020
	0	2	2020
	0	2	2020
	0	2	2020
	0	2	2020
	0	2	2020
	0	2	2020
	0	2	2020
	0	2	2020
	0	2	2020
	0	2	2020
	0	2	2020
	0	2	2020
	0	2	2020
	0	2	2020
	0	2	2020
	0	2	2020
	0	2	2020
	0	2	2020
	0	2	2020
	0	2	2020
	0	2	2020
	0	2	2020
	0	2	2020
	0	2	2020
	0	2	2020
	0	2	2020
	0	2	2020
	0	2	2020
	0	2	2020
	0	2	2020
	0	2	2020
	0	2	2020
	0	2	2020

0	2	2020
0	2	2020
0	2	2020
0	2	2020
0	2	2020
0	2	2020
0	2	2020
0	2	2020
0	2	2020
0	2	2020
0	2	2020
0	2	2020
0	2	2020
0	2	2020
0	2	2020
0	2	2020
0	2	2020
0	2	2020
0	2	2020
0	2	2020
0	2	2020
0	2	2020
0	2	2020
0	2	2020
0	2	2020
0	2	2020
0	2	2020
0	2	2020
0	2	2020
0	2	2020
0	2	2020
0	2	2020
0	2	2020
0	2	2020
0	2	2020
0	2	2020
0	2	2020
0	2	2020
0	2	2020
0	2	2020
0	2	2020
0	2	2020
0	2	2020
0	2	2020
0	2	2020
0	2	2020
0	2	2020
0	2	2020
0	2	2020
0	2	2020

0	2	2020
0	2	2020
0	2	2020
0	2	2020
0	2	2020
0	2	2020
0	2	2020
0	2	2020
0	2	2020
0	2	2020
0	2	2020
0	2	2020
0	2	2020
0	2	2020
0	2	2020
0	2	2020
0	2	2020
0	2	2020
0	2	2020
0	2	2020
0	2	2020
0	2	2020
0	2	2020
0	2	2020
0	2	2020
0	2	2020
0	2	2020
0	2	2020
0	2	2020
0	2	2020
0	2	2020
0	2	2020
0	2	2020
0	2	2020
0	2	2020
0	2	2020
0	2	2020
0	2	2020
0	2	2020
0	2	2020
0	2	2020
0	2	2020
0	2	2020
0	2	2020
0	2	2020
0	2	2020
0	2	2020
0	2	2020
0	2	2020
0	2	2020

0	2	2020
0	2	2020
0	2	2020
0	2	2020
0	2	2020
0	2	2020
0	2	2020
0	2	2020
0	2	2020
0	2	2020
0	2	2020
0	2	2020
0	2	2020
0	2	2020
0	2	2020
0	2	2020
0	2	2020
0	2	2020
0	2	2020
0	2	2020
0	2	2020
0	2	2020
0	2	2020
0	2	2020
0	2	2020
0	2	2020
0	2	2020
0	2	2020
0	2	2020
0	2	2020
0	2	2020
0	2	2020
0	2	2020
0	2	2020
0	2	2020
0	2	2020
0	2	2020
0	2	2020
0	2	2020
0	2	2020
0	2	2020
0	2	2020
0	2	2020
0	2	2020
0	2	2020
0	2	2020
0	2	2020
0	2	2020
0	2	2020
0	2	2020

	0	2	2020
	0	2	2020
	0	2	2020
	0	2	2020
	0	2	2020
	0	2	2020
	0	2	2020
	0	2	2020
	0	2	2020
	0	2	2020
	0	2	2020
	0	2	2020
	0	2	2020
	0	2	2020
	0	2	2020
	7	1,75	2020
	7	1,75	2020
	7	1,75	2020
	7	1,75	2020
	7	1,75	2020
	7	1,75	2020
	7	1,75	2020
	7	1,75	2020
	7	1,75	2020
	7	1,75	2020
	7	1,75	2020
	7	1,75	2020
	7	1,75	2020
	7	1,75	2020
	7	1,75	2020
	7	1,75	2020
	7	1,75	2020
	7	1,75	2020
	7	1,75	2020
	7	1,75	2020
	7	1,75	2020
	7	1,75	2020
	7	1,75	2020
	7	1,75	2020
	7	1,75	2020
	7	1,75	2020
	7	1,75	2020
	7	1,75	2020
	7	1,75	2020
	7	1,75	2020
	7	1,75	2020
	7	1,75	2020
	7	1,75	2020
	7	1,75	2020
	7	1,75	2020

	7	1,75	2020
	7	1,75	2020
	7	1,75	2020
	7	1,75	2020
	7	1,75	2020
	7	1,75	2020
	7	1,75	2020
	7	1,75	2020
	7	1,75	2020
	7	1,75	2020
	7	1,75	2020
	7	1,75	2020
	7	1,75	2020
	7	1,75	2020
	7	1,75	2020
	7	1,75	2020
	7	1,75	2020
	7	1,75	2020
	7	1,75	2020
	7	1,75	2020
	7	1,75	2020
	7	1,75	2020
	7	1,75	2020
	7	1,75	2020
	7	1,75	2020
	7	1,75	2020
	7	1,75	2020
	7	1,75	2020
	7	1,75	2020
	7	1,75	2020
	7	1,75	2020
	7	1,75	2020
	7	1,75	2020
	7	1,75	2020
	7	1,75	2020
	7	1,75	2020
	7	1,75	2020
	7	1,75	2020
	7	1,75	2020
	7	1,75	2020
	7	1,75	2020
	7	1,75	2020
	7	1,75	2020
	7	1,75	2020
	7	1,75	2020
	7	1,75	2020
	7	1,75	2020
	7	1,75	2020
	6	1,5	2020
	6	1,5	2020

	6	1,5	2020
	6	1,5	2020
	6	1,5	2020
	6	1,5	2020
	6	1,5	2020
	6	1,5	2020
	6	1,5	2020
	6	1,5	2020
	6	1,5	2020
	6	1,5	2020
	6	1,5	2020
	6	1,5	2020
	6	1,5	2020
	6	1,5	2020
	6	1,5	2020
	6	1,5	2020
	6	1,5	2020
	6	1,5	2020
	6	1,5	2020
	6	1,5	2020
	6	1,5	2020
	6	1,5	2020
	6	1,5	2020
	6	1,5	2020
	6	1,5	2020
	6	1,5	2020
	6	1,5	2020
	6	1,5	2020
	6	1,5	2020
	6	1,5	2020
	6	1,5	2020
	6	1,5	2020
	6	1,5	2020
	6	1,5	2020
	6	1,5	2020
	6	1,5	2020
	6	1,5	2020
	6	1,5	2020
	6	1,5	2020
	6	1,5	2020
	6	1,5	2020
	6	1,5	2020
	6	1,5	2020
	6	1,5	2020
	6	1,5	2020
	6	1,5	2020
	6	1,5	2020
	6	1,5	2020
	6	1,5	2020
	6	1,5	2020

	6	1,5	2020
	6	1,5	2020
	6	1,5	2020
	6	1,5	2020
	6	1,5	2020
	6	1,5	2020
	6	1,5	2020
	6	1,5	2020
	6	1,5	2020
	6	1,5	2020
	6	1,5	2020
	6	1,5	2020
	3	0,75	2020
	3	0,75	2020
	3	0,75	2020
	3	0,75	2020
	3	0,75	2020
	3	0,75	2020
	3	0,75	2020
	3	0,75	2020
	3	0,75	2020
	3	0,75	2020
	3	0,75	2020
	3	0,75	2020
	3	0,75	2020
	3	0,75	2020
	3	0,75	2020
	3	0,75	2020
	3	0,75	2020
	3	0,75	2020
	3	0,75	2020
	3	0,75	2020
	3	0,75	2020
	3	0,75	2020
	3	0,75	2020
	3	0,75	2020
	3	0,75	2020
	3	0,75	2020
	3	0,75	2020
	3	0,75	2020
	3	0,75	2020
	3	0,75	2020
	3	0,75	2020
	3	0,75	2020
	3	0,75	2020
	3	0,75	2020
	3	0,75	2020
	3	0,75	2020
	3	0,75	2020
	3	0,75	2020

3	0,75	2020
3	0,75	2020
3	0,75	2020
3	0,75	2020
3	0,75	2020
3	0,75	2020
3	0,75	2020
3	0,75	2020
3	0,75	2020
3	0,75	2020
3	0,75	2020
3	0,75	2020
3	0,75	2020
3	0,75	2020
3	0,75	2020
3	0,75	2020
3	0,75	2020
3	0,75	2020
3	0,75	2020
3	0,75	2020
3	0,75	2020
3	0,75	2020
3	0,75	2020
3	0,75	2020
3	0,75	2020
3	0,75	2020
3	0,75	2020
3	0,75	2020
3	0,75	2020
3	0,75	2020
3	0,75	2020
3	0,75	2020
3	0,75	2020
3	0,75	2020
3	0,75	2020
3	0,75	2020
3	0,75	2020
3	0,75	2020
3	0,75	2020
3	0,75	2020
3	0,75	2020
3	0,75	2020
3	0,75	2020
3	0,75	2020
3	0,75	2020
3	0,75	2020
3	0,75	2020
3	0,75	2020
3	0,75	2020
3	0,75	2020

	3	0,75	2020
	3	0,75	2020
	3	0,75	2020
	3	0,75	2020
	3	0,75	2020
	3	0,75	2020
	3	0,75	2020
	3	0,75	2020
	3	0,75	2020
	3	0,75	2020
	3	0,75	2020
	3	0,75	2020
	3	0,75	2020
	3	0,75	2020
	3	0,75	2020
	3	0,75	2020
	3	0,75	2020
	3	0,75	2020
	3	0,75	2020
	3	0,75	2020
	3	0,75	2020
	3	0,75	2020
	3	0,75	2020
	3	0,75	2020
	3	0,75	2020
	3	0,75	2020
	3	0,75	2020
	3	0,75	2020
	3	0,75	2020
	3	0,75	2020
	3	0,75	2020
	3	0,75	2020
	3	0,75	2020
	3	0,75	2020
	3	0,75	2020
	3	0,75	2020
	3	0,75	2020
	3	0,75	2020
	3	0,75	2020
	3	0,75	2020
	3	0,75	2020
	3	0,75	2020
	3	0,75	2020
	3	0,75	2020
	3	0,75	2020
	3	0,75	2020
	3	0,75	2020
	3	0,75	2020
	3	0,75	2020
	3	0,75	2020

	3	0,75	2020
	3	0,75	2020
	3	0,75	2020
	3	0,75	2020
	3	0,75	2020
	3	0,75	2020
	3	0,75	2020
	3	0,75	2020
	3	0,75	2020
	3	0,75	2020
	3	0,75	2020
	3	0,75	2020
	3	0,75	2020
	3	0,75	2020
	3	0,75	2020
	3	0,75	2020
	3	0,75	2020
	3	0,75	2020
	3	0,75	2020
	3	0,75	2020
	3	0,75	2020
	3	0,75	2020
	3	0,75	2020
	3	0,75	2020
	3	0,75	2020
	3	0,75	2020
	3	0,75	2020
	3	0,75	2020
	3	0,75	2020
	3	0,75	2020
	3	0,75	2020
	3	0,75	2020
	3	0,75	2020
	3	0,75	2020
	3	0,75	2020
	3	0,75	2020
	3	0,75	2020
	3	0,75	2020
	3	0,75	2020
	3	0,75	2020
	3	0,75	2020
	3	0,75	2020
	3	0,75	2020
	3	0,75	2020
	3	0,75	2020
	3	0,75	2020
	3	0,75	2020
	3	0,75	2020
	3	0,75	2020
	3	0,75	2020

3	0,75	2020
3	0,75	2020
3	0,75	2020
3	0,75	2020
3	0,75	2020
3	0,75	2020
3	0,75	2020
3	0,75	2020
3	0,75	2020
3	0,75	2020
3	0,75	2020
3	0,75	2020
3	0,75	2020
3	0,75	2020
3	0,75	2020
3	0,75	2020
3	0,75	2020
3	0,75	2020
3	0,75	2020
3	0,75	2020
3	0,75	2020
3	0,75	2020
3	0,75	2020
3	0,75	2020
3	0,75	2020
3	0,75	2020
3	0,75	2020
3	0,75	2020
3	0,75	2020
3	0,75	2020
3	0,75	2020
3	0,75	2020
3	0,75	2020
3	0,75	2020
3	0,75	2020
3	0,75	2020
3	0,75	2020
3	0,75	2020
3	0,75	2020
3	0,75	2020
3	0,75	2020
3	0,75	2020
3	0,75	2020
3	0,75	2020
3	0,75	2020
3	0,75	2020
3	0,75	2020
3	0,75	2020
3	0,75	2020
3	0,75	2020

	3	0,75	2020
	3	0,75	2020
	3	0,75	2020
	3	0,75	2020
	1580	2755	2019
	820	2715	2019
	1790	2592,5	2019
	760	2440	2019
	940	2105	2019
	1130	1847,5	2019
	525	1521,25	2019
	870	1447,5	2019
	1065	1371,25	2019
	1530	1237,5	2019
	1140	1230	2019
	620	1180	2019
	925	1176,25	2019
	425	1131,25	2019
	1415	1078,75	2019
	1045	1071,25	2019
	485	1061,25	2019
	1150	1017,5	2019
	960	1005	2019
	965	971,25	2019
	645	966,25	2019
	310	947,5	2019
	620	935	2019
	570	932,5	2019
	675	913,75	2019
	435	908,75	2019
	790	877,5	2019
	1015	873,75	2019
	720	870	2019
	490	827,5	2019
	1010	812,5	2019
	485	811,25	2019
	810	807,5	2019
	1005	791,25	2019
	75	778,75	2019
	1630	757,5	2019
	305	753,25	2019
	570	752,5	2019
	775	748,75	2019
	465	736,25	2019
	425	726,25	2019
	560	715	2019
	1450	687,5	2019
	1190	682,5	2019
	650	682,5	2019
	1030	668,5	2019

	995	653,75	2019
	810	652,5	2019
	290	652,5	2019
	645	646,25	2019
	525	641,25	2019
	400	640	2019
	985	626,25	2019
	572	623	2019
	217	619,25	2019
	570	617,5	2019
	730	592,5	2019
	405	570,25	2019
	360	560	2019
	475	558,75	2019
	202	545,5	2019
	780	540	2019
	425	537,25	2019
	360	530	2019
	280	510	2019
	295	509,75	2019
	415	503,75	2019
	237	495,25	2019
	507	476,75	2019
	590	472,5	2019
	495	465,75	2019
	555	458,75	2019
	675	453,75	2019
	535	433,75	2019
	360	420	2019
	825	411,25	2019
	512	406	2019
	555	393,75	2019
	330	392,5	2019
	290	379,5	2019
	200	366	2019
	315	364,75	2019
	330	364,5	2019
	190	362,5	2019
	605	362,25	2019
	315	334,75	2019
	525	329,25	2019
	650	326,5	2019
	525	316,25	2019
	285	301,25	2019
	385	276,25	2019
	380	276	2019
	174	267,5	2019
	250	243,5	2019
	300	241	2019
	305	221,25	2019

	270	208,5	2019
	220	165	2019
	130	129,5	2019
	140	120	2019
	165	111,25	2019
	75	108,75	2019
	950	3857,5	2018
	1870	2547,5	2018
	1760	2490	2018
	1465	1926,25	2018
	300	1885	2018
	1315	1763,75	2018
	1165	1751,25	2018
	695	1738,75	2018
	150	1527,5	2018
	910	1467,5	2018
	1095	1248,75	2018
	405	1201,25	2018
	980	1195	2018
	1405	1171,25	2018
	1530	1112,5	2018
	615	1063,75	2018
	2095	1058,75	2018
	930	1052,5	2018
	615	1018,75	2018
	1390	977,5	2018
	675	973,75	2018
	1140	970	2018
	300	965	2018
	745	956,25	2018
	520	955	2018
	1870	947,5	2018
	475	928,75	2018
	587	926,75	2018
	565	916,25	2018
	540	900	2018
	560	900	2018
	860	870	2018
	395	853,75	2018
	1080	850	2018
	425	836,25	2018
	655	808,75	2018
	550	807,5	2018
	885	806,25	2018
	705	806,25	2018
	370	792,5	2018
	1115	788,75	2018
	330	786,5	2018
	1140	785	2018
	205	776,25	2018

	390	772,5	2018
	530	762,5	2018
	995	753,75	2018
	530	722,5	2018
	805	722,25	2018
	615	713,75	2018
	715	698,75	2018
	495	688,75	2018
	775	673,75	2018
	750	667,5	2018
	260	661	2018
	565	636,25	2018
	280	625	2018
	550	612,5	2018
	390	592,5	2018
	515	578,75	2018
	334	578,5	2018
	810	573,5	2018
	480	570	2018
	582	566,5	2018
	675	563,75	2018
	520	560	2018
	480	560	2018
	245	541,25	2018
	395	533,75	2018
	570	522,5	2018
	465	521,25	2018
	390	518,5	2018
	775	503,75	2018
	415	503,75	2018
	335	493,75	2018
	270	477,5	2018
	180	476	2018
	227	472,75	2018
	1185	471,25	2018
	735	468,75	2018
	605	461,25	2018
	385	456,25	2018
	480	445	2018
	246	437,5	2018
	270	437,5	2018
	360	430	2018
	360	425	2018
	635	410,75	2018
	302	410,5	2018
	330	407,5	2018
	755	378,75	2018
	327	367,75	2018
	360	366	2018
	540	365	2018

	300	365	2018
	220	355	2018
	275	349,75	2018
	110	343,5	2018
	385	320,25	2018
	615	318,75	2018
	630	310,5	2018
	165	301,25	2018
	532	270	2018
	415	268,75	2018
	350	187,5	2018
	230	182,5	2018
	360	170	2018
	105	164,25	2018
	84	56	2018
	770	1302,5	2017
	502,5	1045,63	2017
	150	1025	2017
	245	921,25	2017
	170	915	2017
	395	888,75	2017
	102,5	830,63	2017
	637,5	829,38	2017
	497,5	804,38	2017
	645	786,25	2017
	375	773,75	2017
	110	767,5	2017
	357,5	751,88	2017
	675	746,25	2017
	530	742,5	2017
	297,5	729,38	2017
	307,5	721,88	2017
	1102,5	660,63	2017
	915	641,25	2017
	220	635	2017
	650	622,5	2017
	550	622,5	2017
	240	620	2017
	330	602,5	2017
	480	595	2017
	500	595	2017
	315	593,75	2017
	670	580	2017
	415	578,75	2017
	307,5	569,38	2017
	550	557,5	2017
	195	548,75	2017
	287,5	546,88	2017
	250	542,5	2017
	395	538,75	2017

	407,5	536,88	2017
	380	525	2017
	465	523,75	2017
	505	516,25	2017
	340	515	2017
	515	508,75	2017
	625	506,25	2017
	117,5	499,38	2017
	405	496,25	2017
	120	492,5	2017
	207,5	486,88	2017
	287,5	484,38	2017
	275	483,75	2017
	210	480	2017
	255	473,75	2017
	370	467,5	2017
	235	458,75	2017
	265	456,25	2017
	405	446,25	2017
	150	430	2017
	395	428,75	2017
	247,5	426,88	2017
	170	422,5	2017
	230	412,5	2017
	315	408,75	2017
	415	408,75	2017
	80	390	2017
	297,5	374,38	2017
	290	372,5	2017
	500	370	2017
	275	358,75	2017
	325	356,25	2017
	150	347,5	2017
	345	346,25	2017
	252,5	345,63	2017
	160	345	2017
	252,5	343,13	2017
	350	337,5	2017
	205	336,25	2017
	500	335	2017
	180	325	2017
	180	320	2017
	280	315	2017
	140	315	2017
	210	312,5	2017
	107,5	296,88	2017
	180	295	2017
	375	293,75	2017
	212,5	293,13	2017
	410	287,5	2017

	220	275	2017
	160	275	2017
	330	272,5	2017
	357,5	269,38	2017
	235	268,75	2017
	197,5	264,38	2017
	315	258,75	2017
	285	251,25	2017
	277,5	249,38	2017
	532,5	243,13	2017
	360	230	2017
	80	215	2017
	230	207,5	2017
	212,5	203,13	2017
	220	175	2017
	170	167,5	2017
	105	161,25	2017
	50	117,5	2017
	695	1486,25	2016
	477,5	1211,88	2016
	457,5	1114,38	2016
	455	1033,75	2016
	595	1033,75	2016
	375	998,75	2016
	205	916,25	2016
	225	833,75	2016
	825	816,25	2016
	237,5	791,88	2016
	485	771,25	2016
	690	732,5	2016
	692,5	728,13	2016
	240	700	2016
	337,5	699,38	2016
	330	682,5	2016
	350	672,5	2016
	750	642,5	2016
	640	640	2016
	322,5	630,63	2016
	217,5	619,38	2016
	332,5	603,13	2016
	250	602,5	2016
	285	601,25	2016
	257,5	584,38	2016
	300	582,5	2016
	727,5	581,88	2016
	347,5	571,88	2016
	247,5	571,88	2016
	355	568,75	2016
	265	556,25	2016
	395	541,25	2016

	450	532,5	2016
	325	526,25	2016
	735	526,25	2016
	437,5	519,38	2016
	570	512,5	2016
	480	510	2016
	217,5	504,38	2016
	570	502,5	2016
	550	497,5	2016
	430	482,5	2016
	357,5	476,88	2016
	635	471,25	2016
	342,5	468,13	2016
	535	466,25	2016
	275	463,75	2016
	557,5	459,38	2016
	100	450	2016
	200	445	2016
	310	437,5	2016
	230	437,5	2016
	337,5	429,38	2016
	292,5	428,13	2016
	225	421,25	2016
	395	416,25	2016
	380	415	2016
	340	410	2016
	347,5	401,88	2016
	412,5	393,13	2016
	245	391,25	2016
	332,5	388,13	2016
	325	381,25	2016
	225	376,25	2016
	200	375	2016
	292,5	373,13	2016
	310	372,5	2016
	120	360	2016
	260	360	2016
	200	360	2016
	262,5	345,63	2016
	195	338,75	2016
	560	330	2016
	182,5	325,63	2016
	490	322,5	2016
	315	318,75	2016
	260	310	2016
	140	305	2016
	137,5	304,38	2016
	230	287,5	2016
	210	272,5	2016
	302,5	265,63	2016

	215	263,75	2016
	305	256,25	2016
	110	252,5	2016
	230	227,5	2016
	180	225	2016
	337,5	224,38	2016
	120	220	2016
	20	130	2016
	327,5	1356,88	2015
	500	985	2015
	590	977,5	2015
	150	977,5	2015
	600	950	2015
	412,5	940,63	2015
	240	910	2015
	715	881,25	2015
	542,5	840,63	2015
	560	820	2015
	355	818,75	2015
	505	791,25	2015
	585	781,25	2015
	477,5	779,38	2015
	380	730	2015
	417,5	726,88	2015
	450	692,5	2015
	497,5	669,38	2015
	590	657,5	2015
	380	650	2015
	450	647,5	2015
	327,5	636,88	2015
	505	616,25	2015
	490	612,5	2015
	355	583,75	2015
	272,5	578,13	2015
	465	576,25	2015
	370	572,5	2015
	350	567,5	2015
	387,5	549,38	2015
	500	545	2015
	505	536,25	2015
	375	533,75	2015
	365	531,25	2015
	177,5	519,38	2015
	410	515	2015
	435	498,75	2015
	245	491,25	2015
	330	485	2015
	630	477,5	2015
	430	472,5	2015
	215	468,75	2015

	262,5	460,63	2015
	250	452,5	2015
	155	448,75	2015
	487,5	444,38	2015
	272,5	440,63	2015
	300	435	2015
	270	432,5	2015
	425	431,25	2015
	640	430	2015
	207,5	416,88	2015
	327,5	411,88	2015
	347,5	401,88	2015
	285	401,25	2015
	405	401,25	2015
	357,5	399,38	2015
	350	397,5	2015
	585	396,25	2015
	290	382,5	2015
	242,5	380,63	2015
	470	377,5	2015
	510	377,5	2015
	300	370	2015
	430	367,5	2015
	130	365	2015
	280	360	2015
	330	357,5	2015
	372,5	353,13	2015
	430	352,5	2015
	330	352,5	2015
	675	348,75	2015
	385	341,25	2015
	385	336,25	2015
	235	328,75	2015
	360	320	2015
	290	317,5	2015
	130	317,5	2015
	340	305	2015
	235	298,75	2015
	277,5	289,38	2015
	180	285	2015
	220	275	2015
	170	272,5	2015
	202,5	270,63	2015
	230	270	2015
	175	268,75	2015
	310	267,5	2015
	142,5	265,63	2015
	240	260	2015
	215	253,75	2015
	220	245	2015

	207,5	231,88	2015
	237,5	229,38	2015
	220	225	2015
	537,5	224,38	2015
	420	215	2015
	180	205	2015
	210	197,5	2015
	275	188,75	2015
	180	185	2015
	0	170	2015
	90	127,5	2015
	150	127,5	2015
	830	1377,5	2014
	915	1283,75	2014
	675	1281,25	2014
	925	1221,25	2014
	595	1048,75	2014
	515	968,75	2014
	605	941,25	2014
	580	922,5	2014
	532,5	918,13	2014
	730	872,5	2014
	645	861,25	2014
	432,5	848,13	2014
	695	823,75	2014
	575	808,75	2014
	555	758,75	2014
	425	736,25	2014
	340	700	2014
	510	680	2014
	195	666,25	2014
	217,5	659,38	2014
	450	652,5	2014
	340	650	2014
	370	645	2014
	487,5	641,88	2014
	370	637,5	2014
	700	615	2014
	575	611,25	2014
	337,5	609,38	2014
	65	601,25	2014
	357,5	589,38	2014
	505	571,25	2014
	520	552,5	2014
	272,5	543,13	2014
	385	536,25	2014
	285	531,25	2014
	520	530	2014
	287,5	514,38	2014
	202,5	510,63	2014

	465	501,25	2014
	782,5	500,63	2014
	330	477,5	2014
	220	475	2014
	480	475	2014
	397,5	464,38	2014
	430	462,5	2014
	190	452,5	2014
	295	448,75	2014
	410	442,5	2014
	327,5	436,88	2014
	135	428,75	2014
	297,5	426,88	2014
	282,5	425,63	2014
	437,5	424,38	2014
	272,5	418,13	2014
	310	410	2014
	300	405	2014
	367,5	401,88	2014
	272,5	400,63	2014
	332,5	390,63	2014
	312,5	390,63	2014
	310	387,5	2014
	257,5	384,38	2014
	417,5	379,38	2014
	260	375	2014
	30	372,5	2014
	270	362,5	2014
	332,5	348,13	2014
	272,5	338,13	2014
	337,5	334,38	2014
	270	327,5	2014
	200	320	2014
	177,5	319,38	2014
	100	315	2014
	295	313,75	2014
	435	308,75	2014
	252,5	308,13	2014
	255	298,75	2014
	235	298,75	2014
	297,5	294,38	2014
	315	288,75	2014
	190	287,5	2014
	402,5	280,63	2014
	240	270	2014
	225	266,25	2014
	160	255	2014
	215	253,75	2014
	290	232,5	2014
	180	227,5	2014

	187,5	226,88	2014
	115	218,75	2014
	140	215	2014
	145	206,25	2014
	180	205	2014
	190	197,5	2014
	50	192,5	2014
	102,5	150,63	2014
	15	108,75	2014
	620	1317,5	2013
	280	1155	2013
	75	1103,75	2013
	297,5	1044,38	2013
	437,5	944,38	2013
	310	922,5	2013
	295	838,75	2013
	380	837,5	2013
	430	757,5	2013
	702,5	735,63	2013
	375	733,75	2013
	365	721,25	2013
	395	718,75	2013
	610	715	2013
	575	713,75	2013
	265	706,25	2013
	610	702,5	2013
	290	702,5	2013
	395	698,75	2013
	585	698,75	2013
	410	692,5	2013
	575	683,75	2013
	295	673,75	2013
	430	662,5	2013
	557,5	649,38	2013
	400	645	2013
	350	642,5	2013
	475	641,25	2013
	865	633,75	2013
	325	611,25	2013
	505	578,75	2013
	290	567,5	2013
	402,5	550,63	2013
	290	537,5	2013
	557,5	531,88	2013
	210	527,5	2013
	575	503,75	2013
	225	503,75	2013
	432,5	498,13	2013
	502,5	495,63	2013
	365	481,25	2013

	380	475	2013
	550	462,5	2013
	367,5	461,88	2013
	397,5	444,38	2013
	417,5	434,38	2013
	425	426,25	2013
	322,5	425,63	2013
	262,5	425,63	2013
	645	423,75	2013
	387,5	421,88	2013
	720	420	2013
	215	418,75	2013
	280	415	2013
	220	395	2013
	267,5	376,88	2013
	380	375	2013
	445	371,25	2013
	595	358,75	2013
	170	357,5	2013
	315	353,75	2013
	215	353,75	2013
	287,5	351,88	2013
	395	348,75	2013
	130	347,5	2013
	150	347,5	2013
	210	345	2013
	260	345	2013
	255	343,75	2013
	160	342,5	2013
	317,5	339,38	2013
	230	337,5	2013
	322,5	330,63	2013
	175	328,75	2013
	150	320	2013
	235	318,75	2013
	310	317,5	2013
	185	316,25	2013
	220	315	2013
	285	311,25	2013
	375	293,75	2013
	265	291,25	2013
	200	290	2013
	347,5	286,88	2013
	367,5	281,88	2013
	150	267,5	2013
	285	266,25	2013
	160	265	2013
	122,5	260,63	2013
	120	260	2013
	180	225	2013

	205	221,25	2013
	230	212,5	2013
	85	191,25	2013
	170	187,5	2013
	205	181,25	2013
	110	172,5	2013
	150	152,5	2013
	90	152,5	2013
	110	142,5	2013
	310	1667,5	2012
	280	1310	2012
	485	971,25	2012
	1140	955	2012
	230	947,5	2012
	775	768,75	2012
	225	766,25	2012
	325	741,25	2012
	290	732,5	2012
	250	682,5	2012
	410	682,5	2012
	160	670	2012
	630	667,5	2012
	700	665	2012
	400	655	2012
	315	628,75	2012
	320	610	2012
	440	610	2012
	240	580	2012
	465	576,25	2012
	260	570	2012
	170	562,5	2012
	320	555	2012
	290	542,5	2012
	450	542,5	2012
	350	527,5	2012
	270	517,5	2012
	325	511,25	2012
	1030	507,5	2012
	520	505	2012
	335	503,75	2012
	425	491,25	2012
	420	485	2012
	370	482,5	2012
	230	477,5	2012
	360	475	2012
	480	470	2012
	60	465	2012
	425	461,25	2012
	390	447,5	2012
	370	442,5	2012

	180	440	2012
	235	438,75	2012
	185	436,25	2012
	375	433,75	2012
	230	427,5	2012
	310	422,5	2012
	235	418,75	2012
	305	416,25	2012
	260	415	2012
	170	412,5	2012
	300	405	2012
	365	396,25	2012
	180	395	2012
	150	392,5	2012
	495	383,75	2012
	240	380	2012
	370	377,5	2012
	265	366,25	2012
	355	363,75	2012
	245	361,25	2012
	280	360	2012
	250	357,5	2012
	225	346,25	2012
	225	346,25	2012
	100	345	2012
	425	336,25	2012
	905	326,25	2012
	305	326,25	2012
	295	323,75	2012
	380	315	2012
	150	312,5	2012
	265	311,25	2012
	310	307,5	2012
	215	303,75	2012
	255	303,75	2012
	140	295	2012
	250	292,5	2012
	280	290	2012
	260	285	2012
	260	285	2012
	210	282,5	2012
	250	272,5	2012
	225	271,25	2012
	155	258,75	2012
	190	257,5	2012
	240	255	2012
	205	251,25	2012
	205	251,25	2012
	220	245	2012
	180	245	2012

	355	243,75	2012
	80	235	2012
	210	232,5	2012
	160	230	2012
	250	222,5	2012
	160	220	2012
	160	220	2012
	260	215	2012
	320	215	2012
	180	205	2012
	155	188,75	2012
	190	167,5	2012
	120	110	2012
	160	110	2012
	890	1462,5	2011
	475	1258,75	2011
	580	1095	2011
	475	1028,75	2011
	250	972,5	2011
	550	957,5	2011
	385	936,25	2011
	695	878,75	2011
	595	818,75	2011
	190	782,5	2011
	735	713,75	2011
	625	711,25	2011
	600	690	2011
	425	676,25	2011
	350	627,5	2011
	375	583,75	2011
	170	582,5	2011
	740	575	2011
	445	571,25	2011
	360	570	2011
	240	570	2011
	505	566,25	2011
	525	551,25	2011
	80	550	2011
	350	547,5	2011
	400	540	2011
	270	532,5	2011
	310	527,5	2011
	640	510	2011
	250	497,5	2011
	410	492,5	2011
	180	480	2011
	175	473,75	2011
	330	472,5	2011
	460	465	2011
	230	457,5	2011

	185	456,25	2011
	525	456,25	2011
	305	446,25	2011
	385	446,25	2011
	385	446,25	2011
	515	438,75	2011
	595	438,75	2011
	330	432,5	2011
	180	425	2011
	220	425	2011
	225	421,25	2011
	280	415	2011
	200	410	2011
	245	406,25	2011
	180	405	2011
	455	393,75	2011
	295	388,75	2011
	140	385	2011
	140	385	2011
	300	385	2011
	225	381,25	2011
	70	377,5	2011
	260	370	2011
	350	367,5	2011
	375	363,75	2011
	215	363,75	2011
	250	362,5	2011
	295	353,75	2011
	180	345	2011
	260	345	2011
	115	338,75	2011
	375	333,75	2011
	540	325	2011
	245	321,25	2011
	295	318,75	2011
	360	310	2011
	245	301,25	2011
	305	296,25	2011
	225	296,25	2011
	200	295	2011
	250	272,5	2011
	200	270	2011
	170	267,5	2011
	260	265	2011
	170	262,5	2011
	180	260	2011
	215	253,75	2011
	190	247,5	2011
	190	247,5	2011
	115	228,75	2011

	185	226,25	2011
	145	226,25	2011
	130	207,5	2011
	180	205	2011
	220	205	2011
	140	185	2011
	90	182,5	2011
	210	182,5	2011
	140	175	2011
	165	121,25	2011
	405	1171,25	2010
	170	962,5	2010
	730	922,5	2010
	375	913,75	2010
	305	896,25	2010
	290	862,5	2010
	1400	840	2010
	1375	813,75	2010
	170	782,5	2010
	605	771,25	2010
	300	755	2010
	580	705	2010
	235	698,75	2010
	425	691,25	2010
	705	676,25	2010
	295	663,75	2010
	470	657,5	2010
	535	638,75	2010
	510	637,5	2010
	160	630	2010
	240	625	2010
	600	600	2010
	500	555	2010
	495	553,75	2010
	405	551,25	2010
	495	543,75	2010
	240	530	2010
	120	520	2010
	160	500	2010
	305	486,25	2010
	210	467,5	2010
	505	436,25	2010
	320	435	2010
	420	425	2010
	425	416,25	2010
	160	415	2010
	270	397,5	2010
	245	386,25	2010
	415	383,75	2010
	285	376,25	2010

	345	376,25	2010
	300	375	2010
	450	372,5	2010
	265	366,25	2010
	320	365	2010
	170	362,5	2010
	200	360	2010
	190	347,5	2010
	265	346,25	2010
	200	340	2010
	320	330	2010
	435	328,75	2010
	310	327,5	2010
	200	320	2010
	480	315	2010
	280	310	2010
	235	308,75	2010
	195	303,75	2010
	405	301,25	2010
	200	300	2010
	100	300	2010
	205	296,25	2010
	490	287,5	2010
	240	280	2010
	30	277,5	2010
	130	277,5	2010
	65	266,25	2010
	190	262,5	2010
	325	261,25	2010
	190	257,5	2010
	190	257,5	2010
	190	252,5	2010
	170	247,5	2010
	70	237,5	2010
	205	231,25	2010
	80	230	2010
	160	230	2010
	235	228,75	2010
	250	227,5	2010
	225	226,25	2010
	250	222,5	2010
	170	217,5	2010
	160	215	2010
	150	207,5	2010
	270	207,5	2010
	220	205	2010
	290	202,5	2010
	140	195	2010
	310	187,5	2010
	130	182,5	2010

	205	141,25	2010
	90	122,5	2010
	50	112,5	2010
	120	100	2010
	100	85	2010
	420	1055	2009
	230	957,5	2009
	485	811,25	2009
	280	810	2009
	470	777,5	2009
	295	753,75	2009
	620	745	2009
	455	743,75	2009
	885	741,25	2009
	100	735	2009
	315	718,75	2009
	780	715	2009
	1280	710	2009
	500	675	2009
	395	668,75	2009
	490	662,5	2009
	130	652,5	2009
	410	617,5	2009
	520	615	2009
	455	613,75	2009
	170	607,5	2009
	475	578,75	2009
	310	577,5	2009
	350	577,5	2009
	480	570	2009
	130	567,5	2009
	210	562,5	2009
	240	530	2009
	310	527,5	2009
	20	515	2009
	330	512,5	2009
	280	510	2009
	700	505	2009
	290	502,5	2009
	315	493,75	2009
	360	475	2009
	105	466,25	2009
	235	463,75	2009
	380	455	2009
	230	452,5	2009
	295	448,75	2009
	285	446,25	2009
	190	442,5	2009
	290	442,5	2009
	270	442,5	2009

	835	438,75	2009
	615	423,75	2009
	290	422,5	2009
	330	407,5	2009
	320	400	2009
	180	395	2009
	245	391,25	2009
	290	382,5	2009
	605	381,25	2009
	335	373,75	2009
	475	373,75	2009
	170	372,5	2009
	410	372,5	2009
	90	357,5	2009
	255	353,75	2009
	255	353,75	2009
	290	352,5	2009
	130	352,5	2009
	120	350	2009
	280	330	2009
	380	325	2009
	160	310	2009
	470	307,5	2009
	645	301,25	2009
	210	297,5	2009
	245	291,25	2009
	190	287,5	2009
	310	287,5	2009
	280	280	2009
	305	266,25	2009
	265	261,25	2009
	325	251,25	2009
	75	238,75	2009
	335	233,75	2009
	90	232,5	2009
	190	232,5	2009
	300	225	2009
	135	223,75	2009
	245	211,25	2009
	90	202,5	2009
	180	195	2009
	190	187,5	2009
	260	140	2009
	80	95	2009
	20	75	2009
	975	1173,75	2008
	520	1110	2008
	200	980	2008
	860	875	2008
	650	792,5	2008

	260	785	2008
	480	760	2008
	140	725	2008
	295	718,75	2008
	385	681,25	2008
	290	677,5	2008
	435	668,75	2008
	100	635	2008
	200	630	2008
	445	616,25	2008
	340	595	2008
	830	587,5	2008
	360	580	2008
	445	571,25	2008
	280	565	2008
	760	550	2008
	180	545	2008
	145	516,25	2008
	360	510	2008
	360	490	2008
	265	486,25	2008
	440	485	2008
	475	483,75	2008
	410	477,5	2008
	390	472,5	2008
	325	471,25	2008
	285	471,25	2008
	370	457,5	2008
	295	443,75	2008
	375	443,75	2008
	450	442,5	2008
	970	442,5	2008
	280	440	2008
	290	432,5	2008
	225	431,25	2008
	285	426,25	2008
	300	425	2008
	180	415	2008
	315	408,75	2008
	275	408,75	2008
	190	407,5	2008
	300	405	2008
	200	400	2008
	390	397,5	2008
	170	397,5	2008
	310	397,5	2008
	310	397,5	2008
	210	392,5	2008
	390	387,5	2008
	465	376,25	2008

	180	375	2008
	190	367,5	2008
	215	363,75	2008
	205	361,25	2008
	350	357,5	2008
	265	356,25	2008
	200	350	2008
	425	346,25	2008
	140	345	2008
	240	340	2008
	260	335	2008
	250	332,5	2008
	310	327,5	2008
	160	325	2008
	255	323,75	2008
	355	323,75	2008
	295	318,75	2008
	420	315	2008
	355	308,75	2008
	260	305	2008
	250	302,5	2008
	360	300	2008
	355	298,75	2008
	110	282,5	2008
	160	280	2008
	540	275	2008
	315	273,75	2008
	210	267,5	2008
	265	266,25	2008
	210	262,5	2008
	265	261,25	2008
	265	261,25	2008
	235	258,75	2008
	310	257,5	2008
	340	235	2008
	130	227,5	2008
	220	210	2008
	225	206,25	2008
	50	192,5	2008
	100	185	2008
	150	157,5	2008
	175	153,75	2008
	360	1380	2007
	460	1325	2007
	615	1023,75	2007
	845	901,25	2007
	445	851,25	2007
	355	788,75	2007
	635	738,75	2007
	700	725	2007

	525	701,25	2007
	430	697,5	2007
	385	686,25	2007
	160	670	2007
	230	667,5	2007
	425	646,25	2007
	500	635	2007
	505	626,25	2007
	140	625	2007
	335	613,75	2007
	275	608,75	2007
	405	581,25	2007
	340	575	2007
	200	560	2007
	305	556,25	2007
	675	548,75	2007
	335	543,75	2007
	420	530	2007
	340	515	2007
	555	513,75	2007
	360	510	2007
	595	498,75	2007
	615	478,75	2007
	340	475	2007
	450	462,5	2007
	330	462,5	2007
	210	462,5	2007
	520	460	2007
	200	455	2007
	240	440	2007
	375	438,75	2007
	70	432,5	2007
	305	421,25	2007
	220	420	2007
	80	410	2007
	205	406,25	2007
	330	402,5	2007
	380	400	2007
	295	393,75	2007
	170	392,5	2007
	390	387,5	2007
	335	373,75	2007
	495	373,75	2007
	370	372,5	2007
	180	370	2007
	425	366,25	2007
	225	356,25	2007
	405	351,25	2007
	440	350	2007
	220	345	2007

	140	345	2007
	330	342,5	2007
	160	340	2007
	360	340	2007
	370	337,5	2007
	245	321,25	2007
	180	320	2007
	225	316,25	2007
	285	316,25	2007
	500	315	2007
	530	312,5	2007
	210	312,5	2007
	190	312,5	2007
	205	301,25	2007
	80	300	2007
	330	297,5	2007
	390	297,5	2007
	310	297,5	2007
	245	296,25	2007
	245	291,25	2007
	60	285	2007
	490	282,5	2007
	190	277,5	2007
	105	266,25	2007
	210	262,5	2007
	225	256,25	2007
	100	255	2007
	165	251,25	2007
	210	247,5	2007
	445	241,25	2007
	150	237,5	2007
	350	237,5	2007
	140	235	2007
	170	227,5	2007
	180	225	2007
	80	225	2007
	100	215	2007
	180	205	2007
	95	198,75	2007
	110	197,5	2007
	100	175	2007
	130	157,5	2007
	220	140	2007
	165	126,25	2007
	100	115	2007
	30	27,5	2007
	385	1446,25	2006
	350	997,5	2006
	550	937,5	2006
	460	900	2006

	315	828,75	2006
	80	810	2006
	295	783,75	2006
	455	743,75	2006
	850	732,5	2006
	310	707,5	2006
	610	692,5	2006
	455	688,75	2006
	405	661,25	2006
	260	615	2006
	160	610	2006
	390	607,5	2006
	380	605	2006
	230	597,5	2006
	575	583,75	2006
	340	575	2006
	365	571,25	2006
	205	571,25	2006
	505	566,25	2006
	500	565	2006
	1440	550	2006
	575	533,75	2006
	425	526,25	2006
	130	512,5	2006
	425	506,25	2006
	365	501,25	2006
	270	497,5	2006
	475	488,75	2006
	230	487,5	2006
	290	487,5	2006
	385	486,25	2006
	905	486,25	2006
	290	482,5	2006
	405	466,25	2006
	260	460	2006
	310	457,5	2006
	415	453,75	2006
	330	452,5	2006
	375	443,75	2006
	575	443,75	2006
	430	437,5	2006
	410	432,5	2006
	185	426,25	2006
	310	422,5	2006
	230	417,5	2006
	430	407,5	2006
	380	405	2006
	255	403,75	2006
	380	395	2006
	120	385	2006

	205	381,25	2006
	275	378,75	2006
	30	377,5	2006
	205	376,25	2006
	400	370	2006
	495	363,75	2006
	265	356,25	2006
	280	355	2006
	180	345	2006
	200	345	2006
	230	337,5	2006
	305	336,25	2006
	145	326,25	2006
	320	320	2006
	185	316,25	2006
	60	315	2006
	230	312,5	2006
	185	306,25	2006
	355	303,75	2006
	190	302,5	2006
	195	298,75	2006
	215	293,75	2006
	390	282,5	2006
	100	275	2006
	170	272,5	2006
	150	272,5	2006
	205	266,25	2006
	265	266,25	2006
	170	252,5	2006
	50	237,5	2006
	115	228,75	2006
	125	221,25	2006
	220	215	2006
	245	201,25	2006
	50	197,5	2006
	265	196,25	2006
	145	116,25	2006
	830	1397,5	2005
	605	1361,25	2005
	545	1176,25	2005
	390	1077,5	2005
	840	1060	2005
	340	745	2005
	355	733,75	2005
	230	732,5	2005
	525	691,25	2005
	400	690	2005
	410	672,5	2005
	440	640	2005
	655	633,75	2005

	445	631,25	2005
	480	630	2005
	430	617,5	2005
	230	617,5	2005
	545	606,25	2005
	270	597,5	2005
	240	585	2005
	290	582,5	2005
	385	576,25	2005
	430	562,5	2005
	335	558,75	2005
	220	550	2005
	230	547,5	2005
	375	533,75	2005
	385	526,25	2005
	700	525	2005
	425	521,25	2005
	570	502,5	2005
	470	502,5	2005
	260	500	2005
	795	488,75	2005
	355	488,75	2005
	315	468,75	2005
	675	448,75	2005
	240	430	2005
	320	430	2005
	180	415	2005
	460	415	2005
	170	407,5	2005
	340	405	2005
	180	395	2005
	250	392,5	2005
	80	390	2005
	690	372,5	2005
	205	351,25	2005
	425	351,25	2005
	425	336,25	2005
	220	335	2005
	50	332,5	2005
	410	332,5	2005
	315	318,75	2005
	230	317,5	2005
	150	317,5	2005
	310	317,5	2005
	225	316,25	2005
	20	315	2005
	180	305	2005
	340	295	2005
	240	295	2005
	255	288,75	2005

	175	288,75	2005
	180	265	2005
	180	265	2005
	290	262,5	2005
	200	260	2005
	155	258,75	2005
	210	252,5	2005
	140	245	2005
	290	242,5	2005
	220	240	2005
	150	237,5	2005
	150	237,5	2005
	270	237,5	2005
	60	235	2005
	250	232,5	2005
	250	232,5	2005
	205	221,25	2005
	140	200	2005
	135	193,75	2005
	100	145	2005
	50	142,5	2005
	100	125	2005
	125	91,25	2005
	155	88,75	2005
	60	15	2005
	130	2082,5	2004
	380	945	2004
	1660	935	2004
	420	845	2004
	465	836,25	2004
	525	811,25	2004
	735	743,75	2004
	1660	715	2004
	380	715	2004
	530	652,5	2004
	540	645	2004
	530	632,5	2004
	265	626,25	2004
	380	625	2004
	285	621,25	2004
	150	617,5	2004
	285	606,25	2004
	380	605	2004
	450	592,5	2004
	490	582,5	2004
	150	577,5	2004
	205	561,25	2004
	270	532,5	2004
	450	522,5	2004
	320	510	2004

	200	505	2004
	420	495	2004
	290	492,5	2004
	260	490	2004
	205	486,25	2004
	405	486,25	2004
	290	472,5	2004
	170	472,5	2004
	275	468,75	2004
	305	456,25	2004
	130	452,5	2004
	415	443,75	2004
	455	443,75	2004
	165	421,25	2004
	290	412,5	2004
	285	406,25	2004
	285	401,25	2004
	235	388,75	2004
	260	385	2004
	330	382,5	2004
	325	381,25	2004
	320	380	2004
	160	370	2004
	260	365	2004
	130	352,5	2004
	330	352,5	2004
	310	352,5	2004
	215	348,75	2004
	155	348,75	2004
	320	345	2004
	215	343,75	2004
	360	340	2004
	405	331,25	2004
	355	328,75	2004
	300	325	2004
	130	322,5	2004
	180	320	2004
	330	317,5	2004
	180	305	2004
	475	303,75	2004
	320	300	2004
	160	290	2004
	390	287,5	2004
	310	287,5	2004
	180	285	2004
	260	280	2004
	240	280	2004
	95	273,75	2004
	370	272,5	2004
	255	268,75	2004

	190	267,5	2004
	475	258,75	2004
	225	256,25	2004
	110	252,5	2004
	190	247,5	2004
	230	242,5	2004
	280	240	2004
	190	232,5	2004
	165	231,25	2004
	185	196,25	2004
	240	190	2004
	355	183,75	2004
	205	181,25	2004
	120	180	2004
	130	172,5	2004
	190	167,5	2004
	110	167,5	2004
	50	162,5	2004
	150	157,5	2004
	120	155	2004
	195	153,75	2004
	70	147,5	2004
	60	125	2004
	255	123,75	2004
	85	91,25	2004
	100	90	2004
	1124	3511	2024
	2512	2668	2024
	2512	2618	2024
	1447	2351,75	2024
	1582	2285,5	2024
	1109	2257,25	2024
	1334	2183,5	2024
	891	1912,75	2024
	1085	1821,25	2024
	606	1811,5	2024
	1791	1717,75	2024
	472	1638	2024
	876	1609	2024
	1394	1518,5	2024
	1829	1517,25	2024
	1267	1506,75	2024
	1349	1497,25	2024
	585	1496,25	2024
	1565	1411,25	2024
	771	1372,75	2024
	652	1343	2024
	247	1331,75	2024
	622	1295,5	2024
	1401	1250,25	2024

	1394	1238,5	2024
	2091	1232,75	2024
	982	1215,5	2024
	2061	1195,25	2024
	750	1187,5	2024
	1664	1181	2024
	869	1167,25	2024
	861	1155,25	2024
	885	1151,25	2024
	861	1105,25	2024
	1011	1072,75	2024
	487	1071,75	2024
	1342	1055,5	2024
	517	1039,25	2024
	846	1031,5	2024
	802	1030,5	2024
	959	1029,75	2024
	142	1025,5	2024
	584	1016	2024
	1003	1000,75	2024
	592	998	2024
	1011	992,75	2024
	450	992,5	2024
	884	991	2024
	772	953	2024
	525	946,25	2024
	810	922,5	2024
	802	920,5	2024
	921	920,25	2024
	555	918,75	2024
	906	826,5	2024
	539	804,75	2024
	615	793,75	2024
	532	793	2024
	584	791	2024
	660	785	2024
	224	781	2024
	412	773	2024
	667	771,75	2024
	577	754,25	2024
	442	750,5	2024
	1020	745	2024
	517	739,25	2024
	562	730,5	2024
	540	725	2024
	922	720,5	2024
	495	713,75	2024
	697	710,25	2024
	875	704,75	2024
	847	701,75	2024

	600	700	2024
	524	691	2024
	524	646	2024
	561	640,25	2024
	352	638	2024
	472	638	2024
	509	637,25	2024
	472	628	2024
	472	624	2024
	375	618,75	2024
	689	612,25	2024
	652	603	2024
	652	573	2024
	555	528,75	2024
	667	527,75	2024
	374	519,5	2024
	396	500	2024
	412	498	2024
	517	490,25	2024
	592	476	2024
	1394	474,5	2024
	652	468	2024
	480	467	2024
	607	464,75	2024
	427	442,75	2024
	315	438,75	2024
	360	381	2024
	312	336	2024
	405	319,25	2024
	75	318,75	2024
	210	302,5	2024
	569	277,25	2024
	289	232,25	2024
	382	195,5	2024
	1117	3659,25	2023
	1200	2700	2023
	2218	2694,5	2023
	2062	2425,5	2023
	907	2186,75	2023
	2137	2124,25	2023
	1551	2117,75	2023
	1356	2019	2023
	1672	2008	2023
	525	1961,25	2023
	690	1842,5	2023
	2062	1805,5	2023
	1649	1712,25	2023
	1994	1698,5	2023
	1356	1559	2023
	592	1518	2023

	1731	1472,75	2023
	1387	1466,75	2023
	741	1465,25	2023
	1498	1454,5	2023
	382	1335,5	2023
	322	1300,5	2023
	802	1280,5	2023
	517	1279,25	2023
	1769	1272,25	2023
	840	1190	2023
	727	1151,75	2023
	742	1145,5	2023
	779	1144,75	2023
	546	1126,5	2023
	795	1118,75	2023
	870	1107,5	2023
	772	1103	2023
	885	1081,25	2023
	644	1081	2023
	1874	1068,5	2023
	741	1045,25	2023
	682	1035,5	2023
	592	1028	2023
	905	1026,25	2023
	801	1020,25	2023
	674	1008,5	2023
	382	985,5	2023
	1220	975	2023
	922	970,5	2023
	697	954,25	2023
	779	934,75	2023
	975	933,75	2023
	1004	931	2023
	823	915,75	2023
	577	904,25	2023
	802	880,5	2023
	352	878	2023
	532	873	2023
	525	871,25	2023
	705	866,25	2023
	741	865,25	2023
	847	856,75	2023
	502	855,5	2023
	772	853	2023
	802	840,5	2023
	569	832,25	2023
	647	797,75	2023
	861	790,25	2023
	1080	790	2023
	907	786,75	2023

	1049	772,25	2023
	675	768,75	2023
	255	743,75	2023
	869	732,25	2023
	449	732,25	2023
	862	727,5	2023
	585	726,25	2023
	330	722,5	2023
	524	701	2023
	554	698,5	2023
	1214	688,5	2023
	375	673,75	2023
	630	668,5	2023
	600	660	2023
	966	657,5	2023
	180	615	2023
	877	611,25	2023
	847	596,75	2023
	457	584,25	2023
	615	559,75	2023
	362	555,5	2023
	177	555,25	2023
	930	552,5	2023
	480	552	2023
	540	551	2023
	854	534,5	2023
	419	514,75	2023
	322	512,5	2023
	847	511,75	2023
	420	505	2023
	675	498,75	2023
	442	492,5	2023
	510	479,5	2023
	315	458,75	2023
	697	452,25	2023
	267	427,75	2023
	330	427,5	2023
	816	420	2023
	375	408,75	2023
	705	402,25	2023
	592	388	2023
	472	388	2023
	150	385,5	2023
	270	380,5	2023
	517	354,25	2023
	502	350,5	2023
	281	328,25	2023
	487	313,75	2023
	120	211	2023
	262	110,5	2023

	2272	3578	2022
	1942	3085,5	2022
	1334	2173,5	2022
	1071	2007,75	2022
	1267	1716,75	2022
	1499	1644,75	2022
	1139	1624,75	2022
	937	1574,25	2022
	1297	1564,25	2022
	1401	1550,25	2022
	1176	1544	2022
	1026	1466,5	2022
	929	1422,25	2022
	1110	1377,5	2022
	1432	1368	2022
	1229	1257,25	2022
	892	1183	2022
	599	1179,75	2022
	1026	1146,5	2022
	1147	1126,75	2022
	989	1117,25	2022
	712	1108	2022
	997	1099,25	2022
	1101	1095,25	2022
	457	1094,25	2022
	711	1087,75	2022
	1499	1064,75	2022
	922	1050,5	2022
	869	1027,25	2022
	1004	1021	2022
	682	1000,5	2022
	1138	994,5	2022
	577	984,25	2022
	1462	965,5	2022
	809	962,25	2022
	1169	962,25	2022
	622	945,5	2022
	464	926	2022
	539	924,75	2022
	1236	909	2022
	667	906,75	2022
	457	904,25	2022
	907	896,75	2022
	1064	896	2022
	772	893	2022
	510	887,5	2022
	547	886,75	2022
	532	873	2022
	659	854,75	2022
	1012	853	2022

	667	826,75	2022
	502	825,5	2022
	607	802,75	2022
	524	791	2022
	322	780,5	2022
	674	778,5	2022
	510	767,5	2022
	1169	762,25	2022
	599	759,75	2022
	727	753,75	2022
	487	751,75	2022
	502	745,5	2022
	502	745,5	2022
	697	744,25	2022
	540	736	2022
	763	730,75	2022
	600	730	2022
	555	718,75	2022
	561	715,25	2022
	562	700,5	2022
	712	699	2022
	697	694,25	2022
	367	686,75	2022
	779	654,75	2022
	765	651,25	2022
	824	637	2022
	450	622,5	2022
	495	619,75	2022
	345	616,25	2022
	742	601,5	2022
	681	600,25	2022
	1072	593	2022
	210	592,5	2022
	817	564,25	2022
	50	537,5	2022
	367	528,75	2022
	345	526,25	2022
	449	513,25	2022
	96	499	2022
	304	488	2022
	704	486	2022
	478	481,5	2022
	651	476,75	2022
	731	464,75	2022
	292	429	2022
	352	394	2022
	442	392,5	2022
	492	378	2022
	727	363,75	2022
	72	292	2022

	1469	2367,25	2021
	1461	2305,25	2021
	2137	2274,25	2021
	1424	2176	2021
	846	1901,5	2021
	1237	1739,25	2021
	1079	1569,75	2021
	750	1487,5	2021
	412	1483	2021
	1784	1481	2021
	614	1423,5	2021
	1025	1346,25	2021
	869	1327,25	2021
	772	1303	2021
	569	1282,25	2021
	660	1275	2021
	1222	1255,5	2021
	720	1200	2021
	945	1186,25	2021
	1417	1184,25	2021
	712	1168	2021
	727	1131,75	2021
	1011	1124,75	2021
	1274	1108,5	2021
	1311	1097,75	2021
	907	1096,75	2021
	442	1080,5	2021
	90	1042,5	2021
	1049	1042,25	2021
	486	1031,5	2021
	945	1026,25	2021
	1056	1024	2021
	510	997,5	2021
	1184	996	2021
	1442	990,5	2021
	1042	960,5	2021
	869	957,25	2021
	667	956,75	2021
	480	950	2021
	652	933	2021
	547	926,75	2021
	772	903	2021
	142	885,5	2021
	1004	871	2021
	1004	856	2021
	487	843,75	2021
	532	843	2021
	772	813	2021
	967	801,75	2021
	1597	799,25	2021

	936	794	2021
	570	782,5	2021
	540	765	2021
	660	765	2021
	276	765	2021
	674	758,5	2021
	982	755,5	2021
	840	745	2021
	412	744	2021
	525	741,25	2021
	809	719,25	2021
	434	718,5	2021
	622	705,5	2021
	0	700	2021
	600	700	2021
	300	695	2021
	750	677,5	2021
	450	676,5	2021
	495	673,75	2021
	495	669,75	2021
	690	668,5	2021
	614	663,5	2021
	457	654,25	2021
	525	651,25	2021
	517	649,25	2021
	517	649,25	2021
	502	645,5	2021
	502	645,5	2021
	547	642,75	2021
	502	627,5	2021
	420	625	2021
	420	625	2021
	960	624	2021
	330	602,5	2021
	674	593,5	2021
	442	590,5	2021
	449	589,25	2021
	465	588,25	2021
	402	580,5	2021
	1131	579,75	2021
	589	572,25	2021
	855	569,75	2021
	129	568,25	2021
	787	566,75	2021
	480	560	2021
	264	553	2021
	532	552	2021
	382	551,5	2021
	375	549,75	2021
	277	545,25	2021

	705	542,25	2021
	592	538	2021
	345	536,25	2021
	184	532	2021
	382	531,5	2021
	585	531,25	2021
	390	529,5	2021
	390	525,5	2021
	629	518,25	2021
	300	516	2021
	810	515,5	2021
	246	513,5	2021
	394	504,5	2021
	466	502,5	2021
	599	497,75	2021
	360	494	2021
	457	481,25	2021
	742	480,5	2021
	465	477,25	2021
	330	474,5	2021
	342	465,5	2021
	517	465,25	2021
	450	464,5	2021
	487	463,75	2021
	330	449,5	2021
	577	444,25	2021
	327	441,75	2021
	435	436,75	2021
	300	436	2021
	300	435	2021
	286	432,5	2021
	346	431,5	2021
	312	430	2021
	339	426,75	2021
	435	420,75	2021
	203	420,75	2021
	191	417,75	2021
	327	417,75	2021
	474	414,5	2021
	342	413,5	2021
	135	409,75	2021
	480	408	2021
	276	407	2021
	301	405,25	2021
	382	401,5	2021
	294	401,5	2021
	236	397	2021
	282	396,5	2021
	367	395,75	2021
	269	395,25	2021

	450	388,5	2021
	233	386,25	2021
	511	385,75	2021
	15	383,75	2021
	222	383,5	2021
	300	383	2021
	330	378,5	2021
	330	374,5	2021
	154	374,5	2021
	274	374,5	2021
	234	370,5	2021
	168	370	2021
	312	364	2021
	135	363,75	2021
	450	362,5	2021
	0	360	2021
	246	357,5	2021
	372	357	2021
	360	354	2021
	339	350,75	2021
	142	350,5	2021
	322	350,5	2021
	233	350,25	2021
	382	341,5	2021
	300	339	2021
	284	331	2021
	300	331	2021
	343	329,75	2021
	414	329,5	2021
	390	329,5	2021
	360	328	2021
	225	326,25	2021
	390	325,5	2021
	234	324,5	2021
	204	324	2021
	174	323,5	2021
	274	320,5	2021
	260	320	2021
	316	317	2021
	300	315	2021
	184	312	2021
	45	311,25	2021
	58	310,5	2021
	232	310	2021
	262	307,5	2021
	45	305,25	2021
	204	302	2021
	372	301	2021
	262	299,5	2021
	372	299	2021

	412	298	2021
	247	297,75	2021
	239	293,75	2021
	88	288	2021
	60	285	2021
	180	281	2021
	257	278,25	2021
	303	276,75	2021
	427	276,75	2021
	128	276	2021
	214	275,5	2021
	244	275	2021
	232	274	2021
	238	273,5	2021
	246	273,5	2021
	517	269,25	2021
	255	268,75	2021
	65	268,25	2021
	239	267,75	2021
	512	266	2021
	109	262,25	2021
	359	261,75	2021
	96	260	2021
	75	258,75	2021
	242	258,5	2021
	298	255,5	2021
	165	255,25	2021
	317	255,25	2021
	555	254,75	2021
	337	254,25	2021
	330	252,5	2021
	256	252	2021
	208	252	2021
	316	251	2021
	55	249,75	2021
	175	249,75	2021
	260	249	2021
	246	248,5	2021
	203	246,75	2021
	238	245,5	2021
	142	245,5	2021
	180	245	2021
	143	244,75	2021
	173	244,25	2021
	140	243	2021
	107	242,75	2021
	342	242,5	2021
	345	242,25	2021
	100	241	2021
	342	239,5	2021

	65	238,25	2021
	124	237	2021
	105	236,25	2021
	102	235,5	2021
	294	235,5	2021
	314	234,5	2021
	352	234	2021
	72	234	2021
	102	233,5	2021
	133	233,25	2021
	116	233	2021
	82	230,5	2021
	0	230	2021
	112	229	2021
	315	228,75	2021
	188	227	2021
	145	226,25	2021
	167	225,75	2021
	262	225,5	2021
	157	225,25	2021
	190	224,5	2021
	112	224	2021
	246	223,5	2021
	214	223,5	2021
	234	222,5	2021
	382	221,5	2021
	190	221,5	2021
	525	221,25	2021
	72	218	2021
	324	217	2021
	201	216,25	2021
	112	216	2021
	172	213	2021
	45	211,25	2021
	45	211,25	2021
	502	210,5	2021
	202	210,5	2021
	222	207,5	2021
	116	207	2021
	214	206,5	2021
	322	205,5	2021
	7	204,75	2021
	75	204,75	2021
	138	204,5	2021
	127	203,75	2021
	262	203,5	2021
	188	203	2021
	212	201	2021
	304	200	2021
	300	197	2021

	292	196	2021
	300	195	2021
	62	194,5	2021
	202	194,5	2021
	117	193,25	2021
	47	191,75	2021
	246	191,5	2021
	73	191,25	2021
	92	191	2021
	201	188,25	2021
	121	188,25	2021
	145	188,25	2021
	66	187,5	2021
	149	187,25	2021
	281	186,25	2021
	180	185	2021
	120	183	2021
	82	182,5	2021
	265	182,25	2021
	112	182	2021
	66	180,5	2021
	159	179,75	2021
	204	179	2021
	129	178,25	2021
	103	177,75	2021
	190	175,5	2021
	102	175,5	2021
	82	174,5	2021
	226	174,5	2021
	216	174	2021
	144	174	2021
	131	172,75	2021
	89	172,25	2021
	192	172	2021
	134	171,5	2021
	148	170	2021
	157	169,25	2021
	158	168,5	2021
	165	168,25	2021
	140	168	2021
	138	167,5	2021
	182	166,5	2021
	180	166	2021
	180	165	2021
	300	165	2021
	95	164,75	2021
	162	164,5	2021
	164	163	2021
	264	162	2021
	146	161,5	2021

	141	161,25	2021
	152	161	2021
	232	161	2021
	131	160,75	2021
	178	160,5	2021
	116	159	2021
	156	159	2021
	164	159	2021
	75	158,75	2021
	146	158,5	2021
	162	158,5	2021
	150	157,5	2021
	70	157,5	2021
	174	157,5	2021
	198	157,5	2021
	119	156,75	2021
	234	156,5	2021
	54	156,5	2021
	225	156,25	2021
	144	156	2021
	92	155	2021
	92	155	2021
	161	154,25	2021
	232	154	2021
	240	154	2021
	144	154	2021
	160	154	2021
	155	153,75	2021
	203	153,75	2021
	58	153,5	2021
	230	153,5	2021
	122	152,5	2021
	108	152	2021
	107	151,75	2021
	34	151,5	2021
	45	151,25	2021
	120	151	2021
	19	150,75	2021
	20	149	2021
	210	148,5	2021
	158	147,5	2021
	189	147,25	2021
	61	146,25	2021
	252	146	2021
	151	145,75	2021
	234	144,5	2021
	217	144,25	2021
	43	143,75	2021
	172	143	2021
	158	141,5	2021

	47	140,75	2021
	139	140,75	2021
	28	140	2021
	143	139,75	2021
	143	139,75	2021
	244	139	2021
	192	139	2021
	75	138,75	2021
	139	138,75	2021
	134	138,5	2021
	146	138,5	2021
	49	138,25	2021
	118	137,5	2021
	0	137	2021
	72	137	2021
	114	136,5	2021
	160	136	2021
	89	135,25	2021
	197	134,25	2021
	52	134	2021
	172	134	2021
	124	133	2021
	84	133	2021
	92	133	2021
	99	132,75	2021
	186	132,5	2021
	100	132	2021
	131	131,75	2021
	90	131,5	2021
	45	131,25	2021
	81	131,25	2021
	224	131	2021
	194	130,5	2021
	169	130,25	2021
	160	130	2021
	127	129,75	2021
	94	129,5	2021
	246	129,5	2021
	150	129,5	2021
	128	128	2021
	46	127,5	2021
	153	127,25	2021
	99	126,75	2021
	146	126,5	2021
	129	126,25	2021
	52	126	2021
	502	125,5	2021
	105	125,25	2021
	191	124,75	2021
	181	124,25	2021

	232	124	2021
	199	123,75	2021
	127	123,75	2021
	74	123,5	2021
	62	123,5	2021
	165	123,25	2021
	145	123,25	2021
	196	123	2021
	174	122,5	2021
	145	122,25	2021
	113	122,25	2021
	101	122,25	2021
	0	122	2021
	108	121	2021
	104	121	2021
	52	120	2021
	292	120	2021
	108	120	2021
	109	119,25	2021
	73	119,25	2021
	129	119,25	2021
	64	119	2021
	27	118,75	2021
	171	118,75	2021
	90	118,5	2021
	145	118,25	2021
	0	118	2021
	76	118	2021
	152	118	2021
	112	117	2021
	220	117	2021
	134	116,5	2021
	25	116,25	2021
	0	116	2021
	126	115,5	2021
	73	115,25	2021
	84	115	2021
	122	114,5	2021
	34	114,5	2021
	214	114,5	2021
	113	114,25	2021
	61	114,25	2021
	52	114	2021
	28	114	2021
	200	114	2021
	123	113,75	2021
	159	113,75	2021
	0	113	2021
	15	112,75	2021
	87	112,75	2021

	98	112,5	2021
	96	112	2021
	0	112	2021
	84	112	2021
	103	111,75	2021
	66	111,5	2021
	101	111,25	2021
	160	111	2021
	0	111	2021
	82	110,5	2021
	161	110,25	2021
	120	110	2021
	127	109,75	2021
	90	109,5	2021
	16	109	2021
	84	109	2021
	19	108,75	2021
	55	108,75	2021
	91	108,75	2021
	70	108,5	2021
	48	108	2021
	94	107,5	2021
	61	107,25	2021
	147	106,75	2021
	75	106,75	2021
	135	106,75	2021
	9	106,25	2021
	192	106	2021
	34	105,5	2021
	61	105,25	2021
	97	105,25	2021
	117	105,25	2021
	160	105	2021
	156	104	2021
	108	104	2021
	74	103,5	2021
	228	103	2021
	263	102,75	2021
	106	102,5	2021
	93	102,25	2021
	292	102	2021
	91	101,75	2021
	139	101,75	2021
	90	101,5	2021
	50	101,5	2021
	45	101,25	2021
	121	101,25	2021
	173	101,25	2021
	68	101	2021
	103	100,75	2021

	90	99,5	2021
	200	99	2021
	164	99	2021
	26	98,5	2021
	150	98,5	2021
	81	98,25	2021
	23	97,75	2021
	134	97,5	2021
	150	97,5	2021
	7	96,75	2021
	131	96,75	2021
	100	96	2021
	99	95,75	2021
	167	95,75	2021
	102	95,5	2021
	145	95,25	2021
	35	94,75	2021
	73	94,25	2021
	133	94,25	2021
	224	94	2021
	48	94	2021
	74	93,5	2021
	172	93	2021
	184	93	2021
	107	92,75	2021
	90	92,5	2021
	53	92,25	2021
	48	92	2021
	108	92	2021
	39	91,75	2021
	39	91,75	2021
	134	91,5	2021
	190	90,5	2021
	102	90,5	2021
	41	90,25	2021
	87	89,75	2021
	31	89,75	2021
	54	89,5	2021
	81	89,25	2021
	28	89	2021
	104	89	2021
	299	88,75	2021
	90	88,5	2021
	40	88	2021
	72	88	2021
	64	87	2021
	76	87	2021
	124	87	2021
	55	86,75	2021
	187	86,75	2021

	199	86,75	2021
	90	86,5	2021
	225	86,25	2021
	49	86,25	2021
	100	86	2021
	19	85,75	2021
	39	85,75	2021
	94	85,5	2021
	101	85,25	2021
	0	85	2021
	52	85	2021
	107	84,75	2021
	139	84,75	2021
	3	84,75	2021
	38	84,5	2021
	149	84,25	2021
	73	84,25	2021
	103	83,75	2021
	72	83	2021
	56	83	2021
	16	83	2021
	90	82,5	2021
	114	82,5	2021
	182	82,5	2021
	93	82,25	2021
	0	82	2021
	83	81,75	2021
	99	81,75	2021
	39	81,75	2021
	79	81,75	2021
	42	81,5	2021
	170	81,5	2021
	66	81,5	2021
	14	81,5	2021
	112	81	2021
	48	81	2021
	112	81	2021
	68	81	2021
	3	80,75	2021
	41	80,25	2021
	120	80	2021
	96	80	2021
	67	79,75	2021
	66	79,5	2021
	58	79,5	2021
	6	79,5	2021
	96	79	2021
	100	79	2021
	108	79	2021
	88	79	2021

	75	78,75	2021
	195	78,75	2021
	91	78,75	2021
	95	78,75	2021
	103	78,75	2021
	75	78,75	2021
	98	78,5	2021
	61	78,25	2021
	60	78	2021
	52	78	2021
	27	77,75	2021
	47	77,75	2021
	107	77,75	2021
	131	77,75	2021
	110	77,5	2021
	86	77,5	2021
	66	77,5	2021
	37	77,25	2021
	113	77,25	2021
	47	76,75	2021
	47	76,75	2021
	43	76,75	2021
	59	76,75	2021
	40	76	2021
	55	75,75	2021
	115	75,75	2021
	10	75,5	2021
	97	75,25	2021
	93	75,25	2021
	116	75	2021
	47	74,75	2021
	7	74,75	2021
	45	74,25	2021
	52	74	2021
	84	74	2021
	56	74	2021
	135	73,75	2021
	67	73,75	2021
	194	73,5	2021
	81	73,25	2021
	132	73	2021
	40	73	2021
	27	72,75	2021
	59	72,75	2021
	66	72,5	2021
	74	72,5	2021
	145	72,25	2021
	37	72,25	2021
	27	71,75	2021
	79	71,75	2021

	199	71,75	2021
	75	71,75	2021
	166	71,5	2021
	45	71,25	2021
	45	71,25	2021
	40	71	2021
	103	70,75	2021
	54	70,5	2021
	42	70,5	2021
	114	70,5	2021
	97	70,25	2021
	65	70,25	2021
	100	70	2021
	26	69,5	2021
	62	69,5	2021
	53	69,25	2021
	33	69,25	2021
	36	69	2021
	44	69	2021
	47	68,75	2021
	39	68,75	2021
	38	68,5	2021
	226	68,5	2021
	62	68,5	2021
	93	68,25	2021
	141	68,25	2021
	60	68	2021
	172	68	2021
	31	67,75	2021
	270	67,5	2021
	69	67,25	2021
	53	67,25	2021
	76	67	2021
	3	66,75	2021
	94	66,5	2021
	9	66,25	2021
	37	66,25	2021
	84	66	2021
	100	66	2021
	111	65,75	2021
	79	65,75	2021
	87	65,75	2021
	78	65,5	2021
	38	65,5	2021
	101	65,25	2021
	85	65,25	2021
	133	65,25	2021
	43	64,75	2021
	99	64,75	2021
	42	64,5	2021

	46	64,5	2021
	17	64,25	2021
	84	64	2021
	116	64	2021
	116	64	2021
	3	63,75	2021
	94	63,5	2021
	97	63,25	2021
	97	63,25	2021
	45	63,25	2021
	25	63,25	2021
	72	63	2021
	52	63	2021
	18	62,5	2021
	73	62,25	2021
	60	62	2021
	60	62	2021
	27	61,75	2021
	66	61,5	2021
	46	61,5	2021
	30	61,5	2021
	10	61,5	2021
	130	61,5	2021
	125	61,25	2021
	57	61,25	2021
	63	60,75	2021
	79	60,75	2021
	31	60,75	2021
	27	60,75	2021
	55	60,75	2021
	62	60,5	2021
	18	60,5	2021
	17	60,25	2021
	61	60,25	2021
	81	60,25	2021
	0	60	2021
	0	60	2021
	0	60	2021
	0	60	2021
	0	60	2021
	0	60	2021
	48	60	2021
	139	59,75	2021
	27	59,75	2021
	110	59,5	2021
	42	59,5	2021
	73	58,25	2021
	33	58,25	2021
	41	58,25	2021
	20	58	2021

	39	57,75	2021
	31	57,75	2021
	91	57,75	2021
	3	57,75	2021
	35	57,75	2021
	83	57,75	2021
	21	57,25	2021
	29	57,25	2021
	17	57,25	2021
	77	57,25	2021
	72	57	2021
	36	57	2021
	71	56,75	2021
	19	56,75	2021
	59	56,75	2021
	42	56,5	2021
	46	56,5	2021
	105	56,25	2021
	21	56,25	2021
	45	56,25	2021
	141	56,25	2021
	128	56	2021
	103	55,75	2021
	47	55,75	2021
	91	55,75	2021
	66	55,5	2021
	102	55,5	2021
	126	55,5	2021
	21	55,25	2021
	100	55	2021
	60	55	2021
	40	55	2021
	58	54,5	2021
	45	54,25	2021
	65	54,25	2021
	73	54,25	2021
	77	54,25	2021
	25	54,25	2021
	24	54	2021
	84	54	2021
	108	54	2021
	127	53,75	2021
	66	53,5	2021
	97	53,25	2021
	93	53,25	2021
	141	53,25	2021
	32	53	2021
	20	53	2021
	84	53	2021
	7	52,75	2021

	55	52,75	2021
	107	52,75	2021
	10	52,5	2021
	66	52,5	2021
	82	52,5	2021
	70	52,5	2021
	32	52	2021
	36	52	2021
	59	51,75	2021
	115	51,75	2021
	31	51,75	2021
	3	51,75	2021
	58	51,5	2021
	26	51,5	2021
	70	51,5	2021
	66	51,5	2021
	106	51,5	2021
	21	51,25	2021
	29	51,25	2021
	45	51,25	2021
	81	51,25	2021
	28	51	2021
	56	51	2021
	59	50,75	2021
	115	50,75	2021
	123	50,75	2021
	22	50,5	2021
	98	50,5	2021
	90	50,5	2021
	90	50,5	2021
	61	50,25	2021
	53	50,25	2021
	27	49,75	2021
	83	49,75	2021
	23	49,75	2021
	66	49,5	2021
	14	49,5	2021
	30	49,5	2021
	70	49,5	2021
	61	49,25	2021
	61	49,25	2021
	61	49,25	2021
	44	49	2021
	48	49	2021
	52	49	2021
	75	48,75	2021
	13	48,25	2021
	40	48	2021
	36	48	2021
	52	48	2021

	7	47,75	2021
	7	47,75	2021
	91	47,75	2021
	71	47,75	2021
	42	47,5	2021
	74	47,5	2021
	86	47,5	2021
	86	47,5	2021
	22	47,5	2021
	45	47,25	2021
	17	47,25	2021
	29	47,25	2021
	53	47,25	2021
	56	47	2021
	7	46,75	2021
	67	46,75	2021
	19	46,75	2021
	107	46,75	2021
	66	46,5	2021
	54	46,5	2021
	98	46,5	2021
	38	46,5	2021
	57	46,25	2021
	121	46,25	2021
	56	46	2021
	67	45,75	2021
	115	45,75	2021
	55	45,75	2021
	6	45,5	2021
	26	45,5	2021
	62	45,5	2021
	82	45,5	2021
	45	45,25	2021
	45	45,25	2021
	29	45,25	2021
	20	45	2021
	32	45	2021
	36	45	2021
	20	45	2021
	27	44,75	2021
	43	44,75	2021
	14	44,5	2021
	22	44,5	2021
	14	44,5	2021
	22	44,5	2021
	29	44,25	2021
	161	44,25	2021
	52	44	2021
	39	43,75	2021
	19	43,75	2021

	102	43,5	2021
	14	43,5	2021
	14	43,5	2021
	34	43,5	2021
	46	43,5	2021
	114	43,5	2021
	18	43,5	2021
	13	43,25	2021
	57	43,25	2021
	112	43	2021
	27	42,75	2021
	39	42,75	2021
	47	42,75	2021
	59	42,75	2021
	111	42,75	2021
	75	42,75	2021
	7	42,75	2021
	31	42,75	2021
	66	42,5	2021
	97	42,25	2021
	41	42,25	2021
	41	42,25	2021
	93	42,25	2021
	113	42,25	2021
	39	41,75	2021
	6	41,5	2021
	14	41,5	2021
	54	41,5	2021
	22	41,5	2021
	45	41,25	2021
	45	41,25	2021
	65	41,25	2021
	49	41,25	2021
	29	41,25	2021
	81	41,25	2021
	81	41,25	2021
	60	41	2021
	52	41	2021
	56	41	2021
	40	41	2021
	28	41	2021
	54	40,5	2021
	34	40,5	2021
	34	40,5	2021
	42	40,5	2021
	13	40,25	2021
	85	40,25	2021
	0	40	2021
	40	40	2021
	3	39,75	2021

	14	39,5	2021
	9	39,25	2021
	77	39,25	2021
	57	39,25	2021
	85	39,25	2021
	84	39	2021
	39	38,75	2021
	35	38,75	2021
	35	38,75	2021
	15	38,75	2021
	35	38,75	2021
	47	38,75	2021
	66	38,5	2021
	6	38,5	2021
	46	38,5	2021
	13	38,25	2021
	93	38,25	2021
	85	38,25	2021
	61	38,25	2021
	81	38,25	2021
	20	38	2021
	92	38	2021
	44	38	2021
	7	37,75	2021
	39	37,75	2021
	26	37,5	2021
	54	37,5	2021
	49	37,25	2021
	41	37,25	2021
	53	37,25	2021
	81	37,25	2021
	20	37	2021
	36	37	2021
	27	36,75	2021
	27	36,75	2021
	3	36,75	2021
	75	36,75	2021
	46	36,5	2021
	78	36,5	2021
	86	36,5	2021
	97	36,25	2021
	37	36,25	2021
	0	36	2021
	0	36	2021
	0	36	2021
	32	36	2021
	104	36	2021
	20	36	2021
	75	35,75	2021
	43	35,75	2021

	34	35,5	2021
	34	35,5	2021
	6	35,5	2021
	29	35,25	2021
	25	35,25	2021
	0	35	2021
	44	35	2021
	43	34,75	2021
	30	34,5	2021
	29	34,25	2021
	29	34,25	2021
	33	34,25	2021
	61	34,25	2021
	0	34	2021
	16	34	2021
	36	34	2021
	48	34	2021
	3	33,75	2021
	47	33,75	2021
	3	33,75	2021
	14	33,5	2021
	34	33,5	2021
	50	33,5	2021
	54	33,5	2021
	37	33,25	2021
	17	33,25	2021
	17	33,25	2021
	37	33,25	2021
	41	33,25	2021
	16	33	2021
	20	33	2021
	16	33	2021
	36	33	2021
	72	33	2021
	3	32,75	2021
	27	32,75	2021
	43	32,75	2021
	51	32,75	2021
	95	32,75	2021
	34	32,5	2021
	42	32,5	2021
	14	32,5	2021
	90	32,5	2021
	90	32,5	2021
	70	32,5	2021
	38	32,5	2021
	38	32,5	2021
	25	32,25	2021
	13	32,25	2021
	13	32,25	2021

	0	32	2021
	28	32	2021
	44	32	2021
	7	31,75	2021
	43	31,75	2021
	19	31,75	2021
	54	31,5	2021
	14	31,5	2021
	25	31,25	2021
	37	31,25	2021
	65	31,25	2021
	25	31,25	2021
	24	31	2021
	60	31	2021
	63	30,75	2021
	75	30,75	2021
	71	30,75	2021
	3	30,75	2021
	26	30,5	2021
	38	30,5	2021
	106	30,5	2021
	21	30,25	2021
	13	30,25	2021
	53	30,25	2021
	9	30,25	2021
	0	30	2021
	0	30	2021
	0	30	2021
	0	30	2021
	0	30	2021
	0	30	2021
	0	30	2021
	40	30	2021
	28	30	2021
	32	30	2021
	120	30	2021
	120	30	2021
	31	29,75	2021
	7	29,75	2021
	39	29,75	2021
	59	29,75	2021
	59	29,75	2021
	42	29,5	2021
	10	29,5	2021
	22	29,5	2021
	70	29,5	2021
	6	29,5	2021
	56	29	2021
	36	29	2021
	32	29	2021

	32	29	2021
	32	29	2021
	27	28,75	2021
	15	28,75	2021
	35	28,75	2021
	67	28,75	2021
	75	28,75	2021
	15	28,75	2021
	18	28,5	2021
	58	28,5	2021
	26	28,5	2021
	86	28,5	2021
	6	28,5	2021
	58	28,5	2021
	25	28,25	2021
	13	28,25	2021
	13	28,25	2021
	37	28,25	2021
	57	28,25	2021
	69	28,25	2021
	32	28	2021
	32	28	2021
	52	28	2021
	56	28	2021
	40	28	2021
	48	28	2021
	19	27,75	2021
	3	27,75	2021
	31	27,75	2021
	51	27,75	2021
	14	27,5	2021
	42	27,5	2021
	90	27,5	2021
	38	27,5	2021
	38	27,5	2021
	58	27,5	2021
	17	27,25	2021
	13	27,25	2021
	21	27,25	2021
	41	27,25	2021
	9	27,25	2021
	9	27,25	2021
	101	27,25	2021
	48	27	2021
	12	27	2021
	28	27	2021
	28	27	2021
	20	27	2021
	40	27	2021
	48	27	2021

	68	27	2021
	27	26,75	2021
	27	26,75	2021
	59	26,75	2021
	15	26,75	2021
	31	26,75	2021
	6	26,5	2021
	6	26,5	2021
	6	26,5	2021
	14	26,5	2021
	30	26,5	2021
	38	26,5	2021
	70	26,5	2021
	78	26,5	2021
	25	26,25	2021
	29	26,25	2021
	17	26,25	2021
	25	26,25	2021
	45	26,25	2021
	29	26,25	2021
	29	26,25	2021
	13	26,25	2021
	17	26,25	2021
	24	26	2021
	0	26	2021
	20	26	2021
	0	26	2021
	20	26	2021
	60	26	2021
	15	25,75	2021
	19	25,75	2021
	47	25,75	2021
	67	25,75	2021
	75	25,75	2021
	10	25,5	2021
	42	25,5	2021
	34	25,5	2021
	10	25,5	2021
	42	25,5	2021
	21	25,25	2021
	13	25,25	2021
	73	25,25	2021
	45	25,25	2021
	45	25,25	2021
	40	25	2021
	20	25	2021
	20	25	2021
	20	25	2021
	0	25	2021
	16	25	2021

	28	25	2021
	36	25	2021
	27	24,75	2021
	59	24,75	2021
	7	24,75	2021
	15	24,75	2021
	39	24,75	2021
	18	24,5	2021
	6	24,5	2021
	14	24,5	2021
	26	24,5	2021
	50	24,5	2021
	22	24,5	2021
	50	24,5	2021
	17	24,25	2021
	37	24,25	2021
	57	24,25	2021
	28	24	2021
	28	24	2021
	36	24	2021
	16	24	2021
	52	24	2021
	15	23,75	2021
	15	23,75	2021
	15	23,75	2021
	75	23,75	2021
	3	23,75	2021
	27	23,75	2021
	39	23,75	2021
	95	23,75	2021
	6	23,5	2021
	14	23,5	2021
	26	23,5	2021
	6	23,5	2021
	6	23,5	2021
	22	23,5	2021
	34	23,5	2021
	38	23,5	2021
	66	23,5	2021
	25	23,25	2021
	17	23,25	2021
	57	23,25	2021
	0	23	2021
	0	23	2021
	52	23	2021
	52	23	2021
	0	23	2021
	12	23	2021
	52	23	2021
	44	23	2021

	72	23	2021
	72	23	2021
	0	23	2021
	0	23	2021
	35	22,75	2021
	31	22,75	2021
	15	22,75	2021
	6	22,5	2021
	10	22,5	2021
	22	22,5	2021
	42	22,5	2021
	22	22,5	2021
	90	22,5	2021
	90	22,5	2021
	13	22,25	2021
	13	22,25	2021
	37	22,25	2021
	37	22,25	2021
	53	22,25	2021
	53	22,25	2021
	0	22	2021
	40	22	2021
	60	22	2021
	19	21,75	2021
	19	21,75	2021
	31	21,75	2021
	67	21,75	2021
	15	21,75	2021
	31	21,75	2021
	31	21,75	2021
	39	21,75	2021
	6	21,5	2021
	18	21,5	2021
	14	21,5	2021
	10	21,5	2021
	10	21,5	2021
	26	21,5	2021
	30	21,5	2021
	30	21,5	2021
	42	21,5	2021
	58	21,5	2021
	58	21,5	2021
	6	21,5	2021
	38	21,5	2021
	50	21,5	2021
	37	21,25	2021
	37	21,25	2021
	13	21,25	2021
	25	21,25	2021
	45	21,25	2021

	9	21,25	2021
	65	21,25	2021
	13	21,25	2021
	13	21,25	2021
	33	21,25	2021
	24	21	2021
	16	21	2021
	56	21	2021
	56	21	2021
	43	20,75	2021
	15	20,75	2021
	19	20,75	2021
	35	20,75	2021
	3	20,75	2021
	67	20,75	2021
	14	20,5	2021
	26	20,5	2021
	6	20,5	2021
	30	20,5	2021
	46	20,5	2021
	22	20,5	2021
	17	20,25	2021
	21	20,25	2021
	41	20,25	2021
	41	20,25	2021
	25	20,25	2021
	41	20,25	2021
	37	20,25	2021
	0	20	2021
	0	20	2021
	0	20	2021
	0	20	2021
	12	20	2021
	32	20	2021
	24	20	2021
	16	20	2021
	48	20	2021
	3	19,75	2021
	31	19,75	2021
	7	19,75	2021
	7	19,75	2021
	7	19,75	2021
	43	19,75	2021
	59	19,75	2021
	15	19,75	2021
	39	19,75	2021
	10	19,5	2021
	18	19,5	2021
	30	19,5	2021
	30	19,5	2021

	6	19,5	2021
	18	19,5	2021
	30	19,5	2021
	50	19,5	2021
	9	19,25	2021
	17	19,25	2021
	17	19,25	2021
	41	19,25	2021
	0	19	2021
	16	19	2021
	60	19	2021
	23	18,75	2021
	47	18,75	2021
	3	18,75	2021
	3	18,75	2021
	51	18,75	2021
	75	18,75	2021
	75	18,75	2021
	6	18,5	2021
	6	18,5	2021
	14	18,5	2021
	26	18,5	2021
	34	18,5	2021
	34	18,5	2021
	6	18,5	2021
	18	18,5	2021
	26	18,5	2021
	26	18,5	2021
	13	18,25	2021
	25	18,25	2021
	13	18,25	2021
	33	18,25	2021
	45	18,25	2021
	57	18,25	2021
	57	18,25	2021
	0	18	2021
	12	18	2021
	0	18	2021
	0	18	2021
	0	18	2021
	16	18	2021
	24	18	2021
	24	18	2021
	0	18	2021
	0	18	2021
	0	18	2021
	0	18	2021
	72	18	2021
	72	18	2021
	23	17,75	2021

	3	17,75	2021
	3	17,75	2021
	7	17,75	2021
	23	17,75	2021
	23	17,75	2021
	15	17,75	2021
	63	17,75	2021
	10	17,5	2021
	10	17,5	2021
	10	17,5	2021
	14	17,5	2021
	30	17,5	2021
	6	17,5	2021
	6	17,5	2021
	22	17,5	2021
	6	17,5	2021
	62	17,5	2021
	33	17,25	2021
	33	17,25	2021
	17	17,25	2021
	33	17,25	2021
	0	17	2021
	20	17	2021
	12	17	2021
	20	17	2021
	20	17	2021
	20	17	2021
	28	17	2021
	28	17	2021
	20	17	2021
	12	17	2021
	12	17	2021
	7	16,75	2021
	7	16,75	2021
	7	16,75	2021
	27	16,75	2021
	47	16,75	2021
	59	16,75	2021
	67	16,75	2021
	18	16,5	2021
	18	16,5	2021
	10	16,5	2021
	10	16,5	2021
	6	16,5	2021
	10	16,5	2021
	6	16,5	2021
	58	16,5	2021
	17	16,25	2021
	25	16,25	2021
	37	16,25	2021

	37	16,25	2021
	37	16,25	2021
	0	16	2021
	16	16	2021
	24	16	2021
	44	16	2021
	12	16	2021
	16	16	2021
	36	16	2021
	3	15,75	2021
	3	15,75	2021
	15	15,75	2021
	3	15,75	2021
	7	15,75	2021
	19	15,75	2021
	27	15,75	2021
	35	15,75	2021
	47	15,75	2021
	47	15,75	2021
	22	15,5	2021
	22	15,5	2021
	6	15,5	2021
	6	15,5	2021
	26	15,5	2021
	6	15,5	2021
	6	15,5	2021
	10	15,5	2021
	22	15,5	2021
	25	15,25	2021
	41	15,25	2021
	21	15,25	2021
	25	15,25	2021
	25	15,25	2021
	33	15,25	2021
	37	15,25	2021
	0	15	2021
	0	15	2021
	0	15	2021
	0	15	2021
	0	15	2021
	0	15	2021
	0	15	2021
	0	15	2021
	20	15	2021
	16	15	2021
	16	15	2021
	24	15	2021
	40	15	2021
	20	15	2021
	28	15	2021

	52	15	2021
	3	14,75	2021
	3	14,75	2021
	3	14,75	2021
	3	14,75	2021
	3	14,75	2021
	3	14,75	2021
	19	14,75	2021
	10	14,5	2021
	10	14,5	2021
	10	14,5	2021
	10	14,5	2021
	10	14,5	2021
	22	14,5	2021
	26	14,5	2021
	17	14,25	2021
	17	14,25	2021
	29	14,25	2021
	9	14,25	2021
	9	14,25	2021
	13	14,25	2021
	21	14,25	2021
	21	14,25	2021
	29	14,25	2021
	29	14,25	2021
	0	14	2021
	12	14	2021
	12	14	2021
	16	14	2021
	48	14	2021
	15	13,75	2021
	7	13,75	2021
	7	13,75	2021
	15	13,75	2021
	19	13,75	2021
	15	13,75	2021
	19	13,75	2021
	27	13,75	2021
	35	13,75	2021
	6	13,5	2021
	14	13,5	2021
	6	13,5	2021
	6	13,5	2021
	14	13,5	2021
	14	13,5	2021
	14	13,5	2021
	14	13,5	2021
	14	13,5	2021
	14	13,5	2021
	14	13,5	2021

	14	13,5	2021
	14	13,5	2021
	14	13,5	2021
	34	13,5	2021
	22	13,5	2021
	30	13,5	2021
	25	13,25	2021
	25	13,25	2021
	33	13,25	2021
	45	13,25	2021
	45	13,25	2021
	16	13	2021
	0	13	2021
	0	13	2021
	12	13	2021
	16	13	2021
	16	13	2021
	44	13	2021
	52	13	2021
	3	12,25	2021
	3	12,25	2021
	15	12,25	2021
	3	12,25	2021
	3	12,25	2021
	3	12,25	2021
	3	12,25	2021
	3	12,25	2021
	3	12,25	2021
	3	12,25	2021
	3	12,25	2021
	3	12,25	2021
	7	12,25	2021
	15	12,25	2021
	23	12,25	2021
	23	12,25	2021
	10	12,5	2021
	10	12,5	2021
	10	12,5	2021
	10	12,5	2021
	22	12,5	2021
	22	12,5	2021
	6	12,5	2021
	6	12,5	2021
	42	12,5	2021
	50	12,5	2021
	9	12,25	2021
	13	12,25	2021
	13	12,25	2021
	9	12,25	2021
	21	12,25	2021

	33	12,25	2021
	41	12,25	2021
	0	12	2021
	0	12	2021
	0	12	2021
	0	12	2021
	0	12	2021
	0	12	2021
	20	12	2021
	28	12	2021
	28	12	2021
	12	12	2021
	48	12	2021
	7	11,75	2021
	7	11,75	2021
	7	11,75	2021
	7	11,75	2021
	7	11,75	2021
	7	11,75	2021
	7	11,75	2021
	7	11,75	2021
	3	11,75	2021
	7	11,75	2021
	7	11,75	2021
	19	11,75	2021
	27	11,75	2021
	3	11,75	2021
	27	11,75	2021
	27	11,75	2021
	27	11,75	2021
	31	11,75	2021
	31	11,75	2021
	39	11,75	2021
	39	11,75	2021
	6	11,5	2021
	6	11,5	2021
	6	11,5	2021
	6	11,5	2021
	10	11,5	2021
	10	11,5	2021
	10	11,5	2021
	26	11,5	2021
	6	11,5	2021
	6	11,5	2021
	6	11,5	2021
	10	11,5	2021
	26	11,5	2021
	30	11,5	2021
	38	11,5	2021
	38	11,5	2021

	38	11,5	2021
	17	11,25	2021
	25	11,25	2021
	9	11,25	2021
	9	11,25	2021
	9	11,25	2021
	25	11,25	2021
	37	11,25	2021
	37	11,25	2021
	37	11,25	2021
	37	11,25	2021
	45	11,25	2021
	45	11,25	2021
	45	11,25	2021
	45	11,25	2021
	45	11,25	2021
	45	11,25	2021
	45	11,25	2021
	45	11,25	2021
	45	11,25	2021
	45	11,25	2021
	45	11,25	2021
	45	11,25	2021
	45	11,25	2021
	45	11,25	2021
	0	11	2021
	16	11	2021
	16	11	2021
	24	11	2021
	24	11	2021
	0	11	2021
	16	11	2021
	36	11	2021
	3	10,75	2021
	3	10,75	2021
	7	10,75	2021
	7	10,75	2021
	15	10,75	2021
	15	10,75	2021
	19	10,75	2021
	35	10,75	2021
	35	10,75	2021
	14	10,5	2021
	22	10,5	2021
	6	10,5	2021
	6	10,5	2021
	14	10,5	2021
	14	10,5	2021
	22	10,5	2021
	6	10,5	2021

	6	10,5	2021
	6	10,5	2021
	6	10,5	2021
	6	10,5	2021
	6	10,5	2021
	6	10,5	2021
	6	10,5	2021
	6	10,5	2021
	18	10,5	2021
	13	10,25	2021
	13	10,25	2021
	13	10,25	2021
	13	10,25	2021
	13	10,25	2021
	13	10,25	2021
	13	10,25	2021
	21	10,25	2021
	33	10,25	2021
	0	10	2021
	0	10	2021
	0	10	2021
	0	10	2021
	0	10	2021
	0	10	2021
	0	10	2021
	0	10	2021
	0	10	2021
	0	10	2021
	0	10	2021
	0	10	2021
	0	10	2021
	0	10	2021
	0	10	2021
	0	10	2021
	0	10	2021
	0	10	2021
	0	10	2021
	0	10	2021
	0	10	2021
	0	10	2021
	0	10	2021
	0	10	2021
	0	10	2021
	0	10	2021
	0	10	2021
	0	10	2021
	0	10	2021
	0	10	2021
	0	10	2021
	0	10	2021

	20	10	2021
	0	10	2021
	0	10	2021
	0	10	2021
	0	10	2021
	12	10	2021
	16	10	2021
	16	10	2021
	20	10	2021
	24	10	2021
	3	9,75	2021
	3	9,75	2021
	3	9,75	2021
	3	9,75	2021
	3	9,75	2021
	3	9,75	2021
	3	9,75	2021
	19	9,75	2021
	3	9,75	2021
	3	9,75	2021
	3	9,75	2021
	3	9,75	2021
	3	9,75	2021
	23	9,75	2021
	31	9,75	2021
	31	9,75	2021
	31	9,75	2021
	10	9,5	2021
	10	9,5	2021
	10	9,5	2021
	10	9,5	2021
	18	9,5	2021
	6	9,5	2021
	10	9,5	2021
	22	9,5	2021
	30	9,5	2021
	30	9,5	2021
	30	9,5	2021
	30	9,5	2021
	9	9,25	2021
	17	9,25	2021
	9	9,25	2021
	9	9,25	2021
	9	9,25	2021
	9	9,25	2021
	17	9,25	2021
	29	9,25	2021
	0	9	2021
	0	9	2021
	16	9	2021

	0	9	2021
	0	9	2021
	0	9	2021
	0	9	2021
	0	9	2021
	0	9	2021
	0	9	2021
	0	9	2021
	0	9	2021
	0	9	2021
	12	9	2021
	12	9	2021
	16	9	2021
	16	9	2021
	16	9	2021
	20	9	2021
	20	9	2021
	28	9	2021
	28	9	2021
	15	8,75	2021
	7	8,75	2021
	7	8,75	2021
	7	8,75	2021
	7	8,75	2021
	7	8,75	2021
	15	8,75	2021
	19	8,75	2021
	19	8,75	2021
	27	8,75	2021
	14	8,5	2021
	6	8,5	2021
	6	8,5	2021
	6	8,5	2021
	14	8,5	2021
	14	8,5	2021
	14	8,5	2021
	14	8,5	2021
	14	8,5	2021
	14	8,5	2021
	14	8,5	2021
	14	8,5	2021
	14	8,5	2021
	14	8,5	2021
	14	8,5	2021
	14	8,5	2021
	14	8,5	2021
	14	8,5	2021
	6	8,5	2021
	6	8,5	2021
	6	8,5	2021

	6	8,5	2021
	6	8,5	2021
	6	8,5	2021
	6	8,5	2021
	26	8,5	2021
	34	8,5	2021
	13	8,25	2021
	9	8,25	2021
	9	8,25	2021
	9	8,25	2021
	9	8,25	2021
	9	8,25	2021
	13	8,25	2021
	13	8,25	2021
	13	8,25	2021
	13	8,25	2021
	13	8,25	2021
	13	8,25	2021
	25	8,25	2021
	25	8,25	2021
	25	8,25	2021
	25	8,25	2021
	33	8,25	2021
	33	8,25	2021
	0	8	2021
	12	8	2021
	12	8	2021
	12	8	2021
	16	8	2021
	16	8	2021
	16	8	2021
	3	7,75	2021
	3	7,75	2021
	3	7,75	2021
	3	7,75	2021
	3	7,75	2021
	3	7,75	2021
	3	7,75	2021
	3	7,75	2021
	3	7,75	2021
	3	7,75	2021
	3	7,75	2021
	3	7,75	2021
	3	7,75	2021
	3	7,75	2021
	3	7,75	2021
	23	7,75	2021
	23	7,75	2021
	23	7,75	2021
	23	7,75	2021

	23	7,75	2021
	31	7,75	2021
	31	7,75	2021
	10	7,5	2021
	10	7,5	2021
	10	7,5	2021
	6	7,5	2021
	6	7,5	2021
	6	7,5	2021
	10	7,5	2021
	10	7,5	2021
	14	7,5	2021
	22	7,5	2021
	22	7,5	2021
	30	7,5	2021
	30	7,5	2021
	9	7,25	2021
	9	7,25	2021
	9	7,25	2021
	13	7,25	2021
	13	7,25	2021
	13	7,25	2021
	13	7,25	2021
	13	7,25	2021
	13	7,25	2021
	21	7,25	2021
	21	7,25	2021
	21	7,25	2021
	21	7,25	2021
	0	7	2021
	0	7	2021
	0	7	2021
	0	7	2021
	0	7	2021
	0	7	2021
	0	7	2021
	0	7	2021
	0	7	2021
	0	7	2021
	0	7	2021
	0	7	2021
	0	7	2021
	0	7	2021
	0	7	2021
	0	7	2021
	0	7	2021
	0	7	2021
	0	7	2021
	0	7	2021
	0	7	2021

0	7	2021
12	7	2021
20	7	2021
20	7	2021
20	7	2021
28	7	2021
28	7	2021
28	7	2021
7	6,75	2021
7	6,75	2021
7	6,75	2021
7	6,75	2021
7	6,75	2021
7	6,75	2021
7	6,75	2021
7	6,75	2021
7	6,75	2021
7	6,75	2021
7	6,75	2021
7	6,75	2021
7	6,75	2021
7	6,75	2021
7	6,75	2021
7	6,75	2021
7	6,75	2021
7	6,75	2021
7	6,75	2021
7	6,75	2021
7	6,75	2021
7	6,75	2021
7	6,75	2021
7	6,75	2021
7	6,75	2021
7	6,75	2021
7	6,75	2021
7	6,75	2021
7	6,75	2021
7	6,75	2021
7	6,75	2021
7	6,75	2021
3	6,75	2021
3	6,75	2021
3	6,75	2021
7	6,75	2021
7	6,75	2021
7	6,75	2021
7	6,75	2021
7	6,75	2021
7	6,75	2021
19	6,75	2021

	19	6,75	2021
	19	6,75	2021
	27	6,75	2021
	27	6,75	2021
	27	6,75	2021
	27	6,75	2021
	27	6,75	2021
	27	6,75	2021
	27	6,75	2021
	6	6,5	2021
	6	6,5	2021
	6	6,5	2021
	6	6,5	2021
	6	6,5	2021
	6	6,5	2021
	6	6,5	2021
	6	6,5	2021
	6	6,5	2021
	6	6,5	2021
	6	6,5	2021
	6	6,5	2021
	6	6,5	2021
	6	6,5	2021
	6	6,5	2021
	6	6,5	2021
	18	6,5	2021
	9	6,25	2021
	9	6,25	2021
	9	6,25	2021
	9	6,25	2021
	9	6,25	2021
	9	6,25	2021
	9	6,25	2021
	9	6,25	2021
	9	6,25	2021
	9	6,25	2021
	9	6,25	2021
	9	6,25	2021
	9	6,25	2021
	17	6,25	2021
	17	6,25	2021
	17	6,25	2021
	25	6,25	2021
	25	6,25	2021
	25	6,25	2021
	25	6,25	2021
	25	6,25	2021
	0	6	2021
	0	6	2021
	0	6	2021

	0	6	2021
	0	6	2021
	16	6	2021
	16	6	2021
	16	6	2021
	24	6	2021
	24	6	2021
	3	5,75	2021
	3	5,75	2021
	3	5,75	2021
	3	5,75	2021
	3	5,75	2021
	3	5,75	2021
	3	5,75	2021
	3	5,75	2021
	3	5,75	2021
	3	5,75	2021
	3	5,75	2021
	3	5,75	2021
	3	5,75	2021
	3	5,75	2021
	3	5,75	2021
	3	5,75	2021
	3	5,75	2021
	3	5,75	2021
	3	5,75	2021
	3	5,75	2021
	3	5,75	2021
	3	5,75	2021
	3	5,75	2021
	3	5,75	2021
	3	5,75	2021
	3	5,75	2021
	3	5,75	2021
	3	5,75	2021
	3	5,75	2021
	3	5,75	2021
	15	5,75	2021
	15	5,75	2021
	6	5,5	2021
	6	5,5	2021
	6	5,5	2021
	6	5,5	2021
	6	5,5	2021
	6	5,5	2021
	14	5,5	2021
	22	5,5	2021
	22	5,5	2021
	22	5,5	2021
	22	5,5	2021
	22	5,5	2021

	13	1,5	2021
	13	1,5	2021
	13	1,5	2021
	13	1,5	2021
	13	1,5	2021
	13	1,5	2021
	13	1,5	2021
	13	1,5	2021
	13	1,5	2021
	13	1,5	2021
	13	1,5	2021
	13	1,5	2021
	13	1,5	2021
	13	1,5	2021
	13	1,5	2021
	13	1,5	2021
	13	1,5	2021
	13	1,5	2021
	21	1,5	2021
	21	1,5	2021
	0	5	2021
	0	5	2021
	0	5	2021
	0	5	2021
	0	5	2021
	0	5	2021
	0	5	2021
	0	5	2021
	0	5	2021
	0	5	2021
	0	5	2021
	0	5	2021
	0	5	2021
	0	5	2021
	0	5	2021
	0	5	2021
	0	5	2021
	0	5	2021
	0	5	2021
	0	5	2021
	0	5	2021
	0	5	2021
	0	5	2021
	0	5	2021
	0	5	2021
	0	5	2021
	0	5	2021
	0	5	2021
	0	5	2021
	0	5	2021

0	5	2021
0	5	2021
0	5	2021
0	5	2021
0	5	2021
0	5	2021
0	5	2021
0	5	2021
0	5	2021
0	5	2021
0	5	2021
0	5	2021
0	5	2021
0	5	2021
0	5	2021
0	5	2021
0	5	2021
0	5	2021
0	5	2021
0	5	2021
0	5	2021
0	5	2021
0	5	2021
0	5	2021
0	5	2021
0	5	2021
0	5	2021
0	5	2021
0	5	2021
0	5	2021
0	5	2021
0	5	2021
0	5	2021
0	5	2021
0	5	2021
0	5	2021
0	5	2021
0	5	2021
0	5	2021
0	5	2021
0	5	2021
0	5	2021
0	5	2021
0	5	2021
0	5	2021
0	5	2021
0	5	2021
0	5	2021
0	5	2021
0	5	2021

0	5	2021
0	5	2021
0	5	2021
0	5	2021
0	5	2021
0	5	2021
0	5	2021
0	5	2021
0	5	2021
0	5	2021
0	5	2021
0	5	2021
0	5	2021
0	5	2021
0	5	2021
0	5	2021
0	5	2021
0	5	2021
0	5	2021
0	5	2021
0	5	2021
0	5	2021
0	5	2021
0	5	2021
0	5	2021
0	5	2021
0	5	2021
0	5	2021
0	5	2021
0	5	2021
0	5	2021
0	5	2021
0	5	2021
0	5	2021
0	5	2021
0	5	2021
0	5	2021
0	5	2021
0	5	2021
0	5	2021
0	5	2021
0	5	2021
12	5	2021
12	5	2021
12	5	2021
12	5	2021
12	5	2021
12	5	2021
12	5	2021
12	5	2021

	20	5	2021
	20	5	2021
	20	5	2021
	20	5	2021
	20	5	2021
	3	4,75	2021
	3	4,75	2021
	3	4,75	2021
	3	4,75	2021
	3	4,75	2021
	3	4,75	2021
	3	4,75	2021
	3	4,75	2021
	3	4,75	2021
	3	4,75	2021
	3	4,75	2021
	3	4,75	2021
	3	4,75	2021
	3	4,75	2021
	19	4,75	2021
	10	4,5	2021
	10	4,5	2021
	10	4,5	2021
	10	4,5	2021
	10	4,5	2021
	10	4,5	2021
	10	4,5	2021
	10	4,5	2021
	10	4,5	2021
	10	4,5	2021
	10	4,5	2021
	10	4,5	2021
	10	4,5	2021
	10	4,5	2021
	10	4,5	2021
	18	4,5	2021
	9	4,25	2021
	9	4,25	2021
	9	4,25	2021
	9	4,25	2021
	9	4,25	2021
	9	4,25	2021
	9	4,25	2021
	9	4,25	2021
	9	4,25	2021
	9	4,25	2021
	9	4,25	2021
	17	4,25	2021
	17	4,25	2021
	17	4,25	2021

	17	4,25	2021
	17	4,25	2021
	17	4,25	2021
	0	4	2021
	0	4	2021
	0	4	2021
	0	4	2021
	0	4	2021
	0	4	2021
	0	4	2021
	0	4	2021
	0	4	2021
	0	4	2021
	0	4	2021
	0	4	2021
	0	4	2021
	0	4	2021
	0	4	2021
	0	4	2021
	0	4	2021
	0	4	2021
	0	4	2021
	0	4	2021
	0	4	2021
	0	4	2021
	16	4	2021
	16	4	2021
	16	4	2021
	16	4	2021
	16	4	2021
	16	4	2021
	16	4	2021
	16	4	2021
	7	3,75	2021
	7	3,75	2021
	7	3,75	2021
	7	3,75	2021
	7	3,75	2021
	7	3,75	2021
	7	3,75	2021
	7	3,75	2021
	7	3,75	2021
	7	3,75	2021
	7	3,75	2021
	7	3,75	2021
	15	3,75	2021
	15	3,75	2021
	15	3,75	2021
	15	3,75	2021
	15	3,75	2021

	15	3,75	2021
	15	3,75	2021
	15	3,75	2021
	15	3,75	2021
	15	3,75	2021
	15	3,75	2021
	15	3,75	2021
	15	3,75	2021
	15	3,75	2021
	6	3,5	2021
	6	3,5	2021
	6	3,5	2021
	6	3,5	2021
	6	3,5	2021
	6	3,5	2021
	6	3,5	2021
	6	3,5	2021
	6	3,5	2021
	6	3,5	2021
	6	3,5	2021
	6	3,5	2021
	6	3,5	2021
	6	3,5	2021
	6	3,5	2021
	6	3,5	2021
	6	3,5	2021
	6	3,5	2021
	6	3,5	2021
	6	3,5	2021
	6	3,5	2021
	6	3,5	2021
	6	3,5	2021
	6	3,5	2021
	6	3,5	2021
	6	3,5	2021
	6	3,5	2021
	6	3,5	2021
	6	3,5	2021
	6	3,5	2021
	6	3,5	2021
	6	3,5	2021
	6	3,5	2021
	6	3,5	2021
	6	3,5	2021
	6	3,5	2021
	6	3,5	2021
	6	3,5	2021
	6	3,5	2021
	6	3,5	2021
	6	3,5	2021

	6	3,5	2021
	6	3,5	2021
	6	3,5	2021
	6	3,5	2021
	6	3,5	2021
	14	3,5	2021
	14	3,5	2021
	14	3,5	2021
	14	3,5	2021
	14	3,5	2021
	14	3,5	2021
	14	3,5	2021
	14	3,5	2021
	14	3,5	2021
	14	3,5	2021
	14	3,5	2021
	14	3,5	2021
	14	3,5	2021
	14	3,5	2021
	14	3,5	2021
	14	3,5	2021
	14	3,5	2021
	14	3,5	2021
	14	3,5	2021
	14	3,5	2021
	14	3,5	2021
	14	3,5	2021
	13	3,25	2021
	13	3,25	2021
	13	3,25	2021
	13	3,25	2021
	13	3,25	2021
	13	3,25	2021
	13	3,25	2021
	13	3,25	2021
	13	3,25	2021
	13	3,25	2021
	13	3,25	2021
	12	3	2021
	12	3	2021
	12	3	2021
	12	3	2021
	3	2,75	2021
	3	2,75	2021
	3	2,75	2021
	3	2,75	2021
	3	2,75	2021
	3	2,75	2021
	3	2,75	2021
	3	2,75	2021

	3	2,75	2021
	3	2,75	2021
	3	2,75	2021
	3	2,75	2021
	3	2,75	2021
	3	2,75	2021
	3	2,75	2021
	3	2,75	2021
	3	2,75	2021
	3	2,75	2021
	3	2,75	2021
	3	2,75	2021
	3	2,75	2021
	3	2,75	2021
	3	2,75	2021
	3	2,75	2021
	3	2,75	2021
	3	2,75	2021
	3	2,75	2021
	3	2,75	2021
	3	2,75	2021
	3	2,75	2021
	3	2,75	2021
	3	2,75	2021
	3	2,75	2021
	3	2,75	2021
	3	2,75	2021
	3	2,75	2021
	3	2,75	2021
	3	2,75	2021
	3	2,75	2021
	3	2,75	2021
	3	2,75	2021
	3	2,75	2021
	3	2,75	2021
	3	2,75	2021
	3	2,75	2021
	3	2,75	2021
	3	2,75	2021
	3	2,75	2021
	3	2,75	2021
	3	2,75	2021
	3	2,75	2021
	3	2,75	2021
	3	2,75	2021
	3	2,75	2021
	3	2,75	2021
	3	2,75	2021
	3	2,75	2021
	3	2,75	2021

	3	2,75	2021
	3	2,75	2021
	3	2,75	2021
	3	2,75	2021
	3	2,75	2021
	3	2,75	2021
	3	2,75	2021
	3	2,75	2021
	3	2,75	2021
	3	2,75	2021
	3	2,75	2021
	3	2,75	2021
	3	2,75	2021
	3	2,75	2021
	3	2,75	2021
	3	2,75	2021
	3	2,75	2021
	3	2,75	2021
	3	2,75	2021
	10	2,5	2021
	10	2,5	2021
	10	2,5	2021
	10	2,5	2021
	10	2,5	2021
	10	2,5	2021
	10	2,5	2021
	10	2,5	2021
	10	2,5	2021
	10	2,5	2021
	10	2,5	2021
	10	2,5	2021
	10	2,5	2021
	10	2,5	2021
	10	2,5	2021
	10	2,5	2021
	9	2,25	2021
	9	2,25	2021
	9	2,25	2021
	9	2,25	2021
	9	2,25	2021
	9	2,25	2021
	9	2,25	2021
	0	2	2021
	0	2	2021
	0	2	2021
	0	2	2021
	0	2	2021
	0	2	2021
	0	2	2021
	0	2	2021

0	2	2021
0	2	2021
0	2	2021
0	2	2021
0	2	2021
0	2	2021
0	2	2021
0	2	2021
0	2	2021
0	2	2021
0	2	2021
0	2	2021
0	2	2021
0	2	2021
0	2	2021
0	2	2021
0	2	2021
0	2	2021
0	2	2021
0	2	2021
0	2	2021
0	2	2021
0	2	2021
0	2	2021
0	2	2021
0	2	2021
0	2	2021
0	2	2021
0	2	2021
0	2	2021
0	2	2021
0	2	2021
0	2	2021
0	2	2021
0	2	2021
0	2	2021
0	2	2021
0	2	2021
0	2	2021
0	2	2021
0	2	2021
0	2	2021
0	2	2021
0	2	2021
0	2	2021
0	2	2021
0	2	2021
0	2	2021
0	2	2021
0	2	2021

0	2	2021
0	2	2021
0	2	2021
0	2	2021
0	2	2021
0	2	2021
0	2	2021
0	2	2021
0	2	2021
0	2	2021
0	2	2021
0	2	2021
0	2	2021
0	2	2021
0	2	2021
0	2	2021
0	2	2021
0	2	2021
0	2	2021
0	2	2021
0	2	2021
0	2	2021
0	2	2021
0	2	2021
0	2	2021
0	2	2021
0	2	2021
0	2	2021
0	2	2021
0	2	2021
0	2	2021
0	2	2021
0	2	2021
0	2	2021
0	2	2021
0	2	2021
0	2	2021
0	2	2021
0	2	2021
0	2	2021
0	2	2021
0	2	2021
0	2	2021
0	2	2021
0	2	2021
0	2	2021
0	2	2021
0	2	2021
0	2	2021
0	2	2021

0	2	2021
0	2	2021
0	2	2021
0	2	2021
0	2	2021
0	2	2021
0	2	2021
0	2	2021
0	2	2021
0	2	2021
0	2	2021
0	2	2021
0	2	2021
0	2	2021
0	2	2021
0	2	2021
0	2	2021
0	2	2021
0	2	2021
0	2	2021
0	2	2021
0	2	2021
0	2	2021
0	2	2021
0	2	2021
0	2	2021
0	2	2021
0	2	2021
0	2	2021
0	2	2021
0	2	2021
0	2	2021
0	2	2021
0	2	2021
0	2	2021
0	2	2021
0	2	2021
0	2	2021
0	2	2021
0	2	2021
0	2	2021
0	2	2021
0	2	2021
0	2	2021
0	2	2021
0	2	2021
0	2	2021
0	2	2021
0	2	2021
0	2	2021

0	2	2021
0	2	2021
0	2	2021
0	2	2021
0	2	2021
7	1,75	2021
7	1,75	2021
7	1,75	2021
7	1,75	2021
7	1,75	2021
7	1,75	2021
7	1,75	2021
7	1,75	2021
7	1,75	2021
7	1,75	2021
7	1,75	2021
7	1,75	2021
7	1,75	2021
7	1,75	2021
7	1,75	2021
7	1,75	2021
7	1,75	2021
7	1,75	2021
7	1,75	2021
7	1,75	2021
7	1,75	2021
7	1,75	2021
7	1,75	2021
7	1,75	2021
7	1,75	2021
7	1,75	2021
7	1,75	2021
7	1,75	2021
7	1,75	2021
7	1,75	2021
7	1,75	2021
7	1,75	2021
7	1,75	2021
7	1,75	2021
7	1,75	2021
7	1,75	2021
7	1,75	2021
7	1,75	2021
7	1,75	2021
7	1,75	2021
7	1,75	2021
7	1,75	2021
7	1,75	2021
7	1,75	2021
7	1,75	2021

7	1,75	2021
7	1,75	2021
7	1,75	2021
7	1,75	2021
7	1,75	2021
7	1,75	2021
7	1,75	2021
7	1,75	2021
7	1,75	2021
7	1,75	2021
7	1,75	2021
7	1,75	2021
7	1,75	2021
7	1,75	2021
7	1,75	2021
7	1,75	2021
7	1,75	2021
7	1,75	2021
7	1,75	2021
7	1,75	2021
7	1,75	2021
7	1,75	2021
7	1,75	2021
7	1,75	2021
7	1,75	2021
7	1,75	2021
7	1,75	2021
7	1,75	2021
7	1,75	2021
7	1,75	2021
7	1,75	2021
7	1,75	2021
7	1,75	2021
7	1,75	2021
7	1,75	2021
7	1,75	2021
7	1,75	2021
7	1,75	2021
7	1,75	2021
7	1,75	2021
7	1,75	2021
7	1,75	2021
7	1,75	2021
7	1,75	2021
7	1,75	2021
7	1,75	2021
7	1,75	2021
7	1,75	2021
7	1,75	2021
7	1,75	2021

	7	1,75	2021
	7	1,75	2021
	7	1,75	2021
	7	1,75	2021
	7	1,75	2021
	7	1,75	2021
	7	1,75	2021
	7	1,75	2021
	7	1,75	2021
	7	1,75	2021
	7	1,75	2021
	7	1,75	2021
	6	1,5	2021
	6	1,5	2021
	6	1,5	2021
	6	1,5	2021
	6	1,5	2021
	6	1,5	2021
	6	1,5	2021
	6	1,5	2021
	6	1,5	2021
	6	1,5	2021
	6	1,5	2021
	6	1,5	2021
	6	1,5	2021
	6	1,5	2021
	6	1,5	2021
	6	1,5	2021
	6	1,5	2021
	6	1,5	2021
	6	1,5	2021
	6	1,5	2021
	6	1,5	2021
	6	1,5	2021
	6	1,5	2021
	6	1,5	2021
	6	1,5	2021
	6	1,5	2021
	6	1,5	2021
	6	1,5	2021
	6	1,5	2021
	6	1,5	2021
	6	1,5	2021
	6	1,5	2021
	6	1,5	2021
	6	1,5	2021
	6	1,5	2021
	6	1,5	2021
	6	1,5	2021
	6	1,5	2021

	6	1,5	2021
	6	1,5	2021
	6	1,5	2021
	6	1,5	2021
	6	1,5	2021
	6	1,5	2021
	6	1,5	2021
	6	1,5	2021
	6	1,5	2021
	6	1,5	2021
	6	1,5	2021
	6	1,5	2021
	6	1,5	2021
	6	1,5	2021
	6	1,5	2021
	6	1,5	2021
	6	1,5	2021
	6	1,5	2021
	6	1,5	2021
	6	1,5	2021
	6	1,5	2021
	6	1,5	2021
	6	1,5	2021
	6	1,5	2021
	6	1,5	2021
	6	1,5	2021
	6	1,5	2021
	6	1,5	2021
	6	1,5	2021
	6	1,5	2021
	6	1,5	2021
	6	1,5	2021
	6	1,5	2021
	3	0,75	2021
	3	0,75	2021
	3	0,75	2021
	3	0,75	2021
	3	0,75	2021
	3	0,75	2021
	3	0,75	2021
	3	0,75	2021
	3	0,75	2021
	3	0,75	2021
	3	0,75	2021
	3	0,75	2021
	3	0,75	2021
	3	0,75	2021
	3	0,75	2021
	3	0,75	2021
	3	0,75	2021

3	0,75	2021
3	0,75	2021
3	0,75	2021
3	0,75	2021
3	0,75	2021
3	0,75	2021
3	0,75	2021
3	0,75	2021
3	0,75	2021
3	0,75	2021
3	0,75	2021
3	0,75	2021
3	0,75	2021
3	0,75	2021
3	0,75	2021
3	0,75	2021
3	0,75	2021
3	0,75	2021
3	0,75	2021
3	0,75	2021
3	0,75	2021
3	0,75	2021
3	0,75	2021
3	0,75	2021
3	0,75	2021
3	0,75	2021
3	0,75	2021
3	0,75	2021
3	0,75	2021
3	0,75	2021
3	0,75	2021
3	0,75	2021
3	0,75	2021
3	0,75	2021
3	0,75	2021
3	0,75	2021
3	0,75	2021
3	0,75	2021
3	0,75	2021
3	0,75	2021
3	0,75	2021
3	0,75	2021
3	0,75	2021
3	0,75	2021
3	0,75	2021
3	0,75	2021
3	0,75	2021
3	0,75	2021
3	0,75	2021
3	0,75	2021

	3	0,75	2021
	3	0,75	2021
	3	0,75	2021
	3	0,75	2021
	3	0,75	2021
	3	0,75	2021
	3	0,75	2021
	3	0,75	2021
	3	0,75	2021
	3	0,75	2021
	3	0,75	2021
	3	0,75	2021
	3	0,75	2021
	3	0,75	2021
	3	0,75	2021
	3	0,75	2021
	3	0,75	2021
	3	0,75	2021
	3	0,75	2021
	3	0,75	2021
	3	0,75	2021
	3	0,75	2021
	3	0,75	2021
	3	0,75	2021
	3	0,75	2021
	3	0,75	2021
	3	0,75	2021
	3	0,75	2021
	3	0,75	2021
	3	0,75	2021
	3	0,75	2021
	3	0,75	2021
	3	0,75	2021
	3	0,75	2021
	3	0,75	2021
	3	0,75	2021
	3	0,75	2021
	3	0,75	2021
	3	0,75	2021
	3	0,75	2021
	3	0,75	2021
	3	0,75	2021
	3	0,75	2021
	3	0,75	2021
	3	0,75	2021
	3	0,75	2021
	3	0,75	2021
	3	0,75	2021
	3	0,75	2021
	3	0,75	2021

3	0,75	2021
3	0,75	2021
3	0,75	2021
3	0,75	2021
3	0,75	2021
3	0,75	2021
3	0,75	2021
3	0,75	2021
3	0,75	2021
3	0,75	2021
3	0,75	2021
3	0,75	2021
3	0,75	2021
3	0,75	2021
3	0,75	2021
3	0,75	2021
3	0,75	2021
3	0,75	2021
3	0,75	2021
3	0,75	2021
3	0,75	2021
3	0,75	2021
3	0,75	2021
3	0,75	2021
3	0,75	2021
3	0,75	2021
3	0,75	2021
3	0,75	2021
3	0,75	2021
3	0,75	2021
3	0,75	2021
3	0,75	2021
3	0,75	2021
3	0,75	2021
3	0,75	2021
3	0,75	2021
3	0,75	2021
3	0,75	2021
3	0,75	2021
3	0,75	2021
3	0,75	2021
3	0,75	2021
3	0,75	2021
3	0,75	2021
3	0,75	2021
3	0,75	2021
3	0,75	2021
3	0,75	2021
3	0,75	2021
3	0,75	2021

	3	0,75	2021
	3	0,75	2021
	3	0,75	2021
	3	0,75	2021
	3	0,75	2021
	3	0,75	2021
	3	0,75	2021
	3	0,75	2021
	3	0,75	2021
	3	0,75	2021
	3	0,75	2021
	3	0,75	2021
	3	0,75	2021
	3	0,75	2021
	3	0,75	2021
	3	0,75	2021
	3	0,75	2021
	3	0,75	2021
	3	0,75	2021
	3	0,75	2021
	3	0,75	2021
	3	0,75	2021
	3	0,75	2021
	3	0,75	2021
	3	0,75	2021
	3	0,75	2021
	3	0,75	2021
	3	0,75	2021
	3	0,75	2021
	3	0,75	2021
	3	0,75	2021
	3	0,75	2021
	3	0,75	2021
	3	0,75	2021
	3	0,75	2021
	3	0,75	2021
	3	0,75	2021
	662	2595,5	2020
	535	2343,75	2020
	1575	2148,75	2020
	610	1967,5	2020
	0	1930	2020
	635	1828,75	2020
	870	1667,5	2020
	270	1457,5	2020
	937	1454,25	2020
	150	1452,5	2020
	2354	1433,5	2020
	870	1412,5	2020
	2132	1383	2020

	990	1352,5	2020
	1455	1323,75	2020
	120	1310	2020
	1040	1210	2020
	1027	1191,75	2020
	707	1186,75	2020
	1245	1166,25	2020
	775	1163,75	2020
	635	1158,75	2020
	770	1147,5	2020
	787	1121,75	2020
	877	1099,25	2020
	1145	1086,25	2020
	642	1050,5	2020
	195	1033,75	2020
	240	1025	2020
	712	1024	2020
	459	1009,75	2020
	390	1007,5	2020
	615	1003,75	2020
	1469	997,25	2020
	270	987,5	2020
	930	982,5	2020
	757	969,25	2020
	780	945	2020
	1397	929,25	2020
	1520	925	2020
	795	913,75	2020
	1215	908,75	2020
	382	905,5	2020
	320	900	2020
	435	883,75	2020
	354	878,5	2020
	1290	877,5	2020
	479	849,75	2020
	347	846,75	2020
	635	839,75	2020
	975	828,75	2020
	432	824	2020
	590	817,5	2020
	425	796,25	2020
	580	795	2020
	1595	793,75	2020
	580	790	2020
	495	778,75	2020
	645	767,25	2020
	480	762	2020
	444	751	2020
	1349	747,25	2020
	629	732,25	2020

	905	721,25	2020
	1199	719,75	2020
	660	711	2020
	285	707,25	2020
	242	705,5	2020
	614	703,5	2020
	785	701,25	2020
	140	695	2020
	385	691,25	2020
	184	682	2020
	555	678,75	2020
	910	677,5	2020
	424	677	2020
	845	676,25	2020
	704	676	2020
	420	675	2020
	645	671,25	2020
	360	670	2020
	670	667,5	2020
	1842	665,5	2020
	590	657,5	2020
	325	656,25	2020
	357	650,25	2020
	510	632,5	2020
	510	622,5	2020
	899	609,75	2020
	830	607,5	2020
	817	604,25	2020
	422	601,5	2020
	615	599,75	2020
	154	598,5	2020
	332	589	2020
	460	585	2020
	435	583,75	2020
	270	583,5	2020
	510	583,5	2020
	727	581,75	2020
	390	577,5	2020
	390	577,5	2020
	246	571,5	2020
	685	558,25	2020
	630	557,5	2020
	335	555,75	2020
	592	548	2020
	270	547,5	2020
	615	545,75	2020
	569	543,25	2020
	540	543	2020
	570	542,5	2020
	890	538,5	2020

	517	530,25	2020
	494	529,5	2020
	346	526,5	2020
	317	519,25	2020
	525	516,25	2020
	182	511,5	2020
	475	504,75	2020
	252	503	2020
	342	502,5	2020
	600	500	2020
	502	496,5	2020
	745	496,25	2020
	309	493,25	2020
	555	484,75	2020
	431	473,75	2020
	99	473,75	2020
	340	471	2020
	198	469,5	2020
	340	465	2020
	387	462,75	2020
	364	461	2020
	615	453,75	2020
	580	450	2020
	240	450	2020
	350	443,5	2020
	420	441	2020
	360	440	2020
	367	437,75	2020
	242	436,5	2020
	324	433	2020
	364	432	2020
	367	431,75	2020
	454	431,5	2020
	290	429,5	2020
	150	417,5	2020
	340	415	2020
	89	413,25	2020
	212	412	2020
	330	408,5	2020
	302	405,5	2020
	206	403,5	2020
	312	403	2020
	172	403	2020
	171	400,75	2020
	500	400	2020
	360	400	2020
	750	399,5	2020
	315	398,75	2020
	282	398,5	2020
	390	395,5	2020

	80	395	2020
	655	393,75	2020
	502	391,5	2020
	374	391,5	2020
	176	390	2020
	435	389,75	2020
	120	388	2020
	286	387,5	2020
	449	385,25	2020
	411	384,75	2020
	153	384,25	2020
	435	382,75	2020
	247	381,75	2020
	645	378,25	2020
	487	376,75	2020
	264	374	2020
	322	371,5	2020
	292	368	2020
	244	362	2020
	379	360,75	2020
	442	360,5	2020
	216	360	2020
	475	358,75	2020
	170	358,5	2020
	310	357,5	2020
	190	355,5	2020
	264	354	2020
	310	353,5	2020
	231	349,75	2020
	145	349,25	2020
	292	349	2020
	255	347,75	2020
	342	347,5	2020
	436	341	2020
	281	340,25	2020
	360	340	2020
	159	335,75	2020
	270	331,5	2020
	352	331	2020
	220	331	2020
	170	327,5	2020
	253	326,25	2020
	222	325,5	2020
	138	324,5	2020
	294	321,5	2020
	246	321,5	2020
	117	319,25	2020
	360	318	2020
	262	317,5	2020
	372	317	2020

	200	316	2020
	450	314,5	2020
	194	314,5	2020
	220	314	2020
	697	312,25	2020
	270	307,5	2020
	480	306	2020
	199	305,75	2020
	315	303,75	2020
	282	301,5	2020
	12	301	2020
	337	300,25	2020
	180	300	2020
	158	299,5	2020
	324	297	2020
	163	292,75	2020
	90	292,5	2020
	417	292,25	2020
	242	290,5	2020
	250	290,5	2020
	276	289	2020
	157	288,25	2020
	179	287,75	2020
	142	286,5	2020
	331	285,75	2020
	240	285	2020
	505	284,25	2020
	88	284	2020
	282	282,5	2020
	272	282	2020
	365	281,25	2020
	229	281,25	2020
	34	280,5	2020
	231	279,75	2020
	173	279,25	2020
	264	278	2020
	236	277	2020
	234	276,5	2020
	184	276	2020
	277	274,25	2020
	180	273	2020
	81	272,25	2020
	142	271,5	2020
	460	271	2020
	119	269,75	2020
	192	269	2020
	115	268,75	2020
	372	268	2020
	292	267	2020
	312	266	2020

	158	265,5	2020
	126	265,5	2020
	420	265	2020
	274	263,5	2020
	387	262,75	2020
	309	259,25	2020
	324	259	2020
	374	258,5	2020
	161	258,25	2020
	240	258	2020
	208	258	2020
	407	257,75	2020
	201	256,25	2020
	182	255,5	2020
	244	255	2020
	233	252,25	2020
	322	251,5	2020
	205	251,25	2020
	119	249,75	2020
	335	248,75	2020
	198	247,5	2020
	469	247,25	2020
	194	246,5	2020
	399	245,75	2020
	375	244,75	2020
	123	242,75	2020
	222	242,5	2020
	261	241,25	2020
	365	240,25	2020
	200	240	2020
	95	239,75	2020
	390	239,5	2020
	214	239,5	2020
	80	238	2020
	203	236,75	2020
	235	234,75	2020
	200	234	2020
	402	233,5	2020
	252	233	2020
	231	232,75	2020
	113	232,25	2020
	187	231,75	2020
	504	230	2020
	232	228	2020
	282	227,5	2020
	102	225,5	2020
	93	225,25	2020
	372	225	2020
	14	224,5	2020
	185	222,25	2020

	232	222	2020
	151	221,75	2020
	154	220,5	2020
	143	219,75	2020
	142	219,5	2020
	154	217,5	2020
	157	217,25	2020
	212	217	2020
	127	216,75	2020
	74	216,5	2020
	205	216,25	2020
	180	216	2020
	212	216	2020
	201	215,25	2020
	202	214,5	2020
	166	214,5	2020
	230	213,5	2020
	161	212,25	2020
	109	211,25	2020
	324	211	2020
	71	209,75	2020
	75	208,75	2020
	191	208,75	2020
	152	208	2020
	192	208	2020
	109	207,25	2020
	28	207	2020
	172	207	2020
	266	206,5	2020
	67	205,75	2020
	27	202,75	2020
	390	202,5	2020
	210	202,5	2020
	307	201,75	2020
	190	201,5	2020
	137	201,25	2020
	116	200	2020
	133	199,25	2020
	149	199,25	2020
	181	199,25	2020
	188	199	2020
	360	199	2020
	474	198,5	2020
	183	197,75	2020
	390	197,5	2020
	334	197,5	2020
	130	196,5	2020
	150	193,5	2020
	66	193,5	2020
	51	192,75	2020

	117	192,25	2020
	232	192	2020
	100	189	2020
	84	189	2020
	264	188	2020
	198	187,5	2020
	131	186,75	2020
	169	186,25	2020
	153	186,25	2020
	192	186	2020
	111	185,75	2020
	280	185	2020
	132	185	2020
	206	183,5	2020
	190	183,5	2020
	155	182,75	2020
	262	182,5	2020
	232	182	2020
	123	181,75	2020
	45	181,25	2020
	185	181,25	2020
	170	180,5	2020
	110	180,5	2020
	142	179,5	2020
	66	178,5	2020
	256	178	2020
	75	177,75	2020
	294	177,5	2020
	129	177,25	2020
	184	177	2020
	224	176	2020
	191	175,75	2020
	110	175,5	2020
	40	175	2020
	154	174,5	2020
	261	174,25	2020
	245	174,25	2020
	240	174	2020
	357	173,25	2020
	52	173	2020
	210	172,5	2020
	510	172,5	2020
	296	172	2020
	485	171,25	2020
	145	171,25	2020
	126	170,5	2020
	42	170,5	2020
	146	170,5	2020
	81	170,25	2020
	61	170,25	2020

	74	169,5	2020
	260	169	2020
	174	168,5	2020
	197	167,25	2020
	250	166,5	2020
	135	165,75	2020
	84	165	2020
	114	164,5	2020
	153	164,25	2020
	157	164,25	2020
	52	164	2020
	79	162,75	2020
	237	162,25	2020
	280	162	2020
	35	161,75	2020
	195	161,75	2020
	94	161,5	2020
	244	161	2020
	315	160,75	2020
	130	160,5	2020
	154	160,5	2020
	56	160	2020
	106	159,5	2020
	194	159,5	2020
	173	159,25	2020
	118	157,5	2020
	138	157,5	2020
	90	156,5	2020
	232	156	2020
	123	154,75	2020
	171	154,75	2020
	349	154,25	2020
	153	154,25	2020
	89	154,25	2020
	175	153,75	2020
	109	153,25	2020
	411	151,75	2020
	232	151	2020
	91	150,75	2020
	201	150,25	2020
	216	149	2020
	155	148,75	2020
	115	148,75	2020
	178	148,5	2020
	154	148,5	2020
	100	148	2020
	74	147,5	2020
	134	147,5	2020
	149	147,25	2020
	125	146,25	2020

	140	146	2020
	108	146	2020
	107	145,75	2020
	134	145,5	2020
	196	145	2020
	178	144,5	2020
	194	144,5	2020
	121	144,25	2020
	205	144,25	2020
	215	143,75	2020
	55	143,75	2020
	95	142,75	2020
	155	142,75	2020
	108	142	2020
	95	141,75	2020
	182	141,5	2020
	45	141,25	2020
	224	141	2020
	104	140	2020
	144	140	2020
	246	139,5	2020
	94	139,5	2020
	100	139	2020
	140	139	2020
	108	139	2020
	131	138,75	2020
	295	138,75	2020
	70	138,5	2020
	129	138,25	2020
	96	138	2020
	31	137,75	2020
	20	137	2020
	307	136,75	2020
	67	136,75	2020
	62	136,5	2020
	50	136,5	2020
	163	134,75	2020
	174	134,5	2020
	136	134	2020
	126	133,5	2020
	97	133,25	2020
	121	133,25	2020
	165	133,25	2020
	132	133	2020
	196	133	2020
	206	132,5	2020
	194	132,5	2020
	89	132,25	2020
	163	131,75	2020
	70	131,5	2020

	86	131,5	2020
	213	130,25	2020
	68	130	2020
	80	130	2020
	130	129,5	2020
	92	129	2020
	36	129	2020
	201	128,25	2020
	176	128	2020
	220	128	2020
	28	128	2020
	132	128	2020
	61	127,25	2020
	136	127	2020
	67	126,75	2020
	65	126,25	2020
	198	125,5	2020
	158	125,5	2020
	133	125,25	2020
	60	125	2020
	7	124,75	2020
	111	124,75	2020
	126	124,5	2020
	122	124,5	2020
	174	124,5	2020
	85	124,25	2020
	60	124	2020
	16	124	2020
	111	123,75	2020
	130	123,5	2020
	148	123	2020
	87	122,75	2020
	170	122,5	2020
	196	122	2020
	94	121,5	2020
	109	121,25	2020
	116	121	2020
	85	120,25	2020
	32	120	2020
	0	120	2020
	208	120	2020
	172	120	2020
	131	119,75	2020
	141	119,25	2020
	91	118,75	2020
	151	118,75	2020
	82	118,5	2020
	97	118,25	2020
	54	117,5	2020
	145	117,25	2020

	61	117,25	2020
	47	115,75	2020
	63	115,75	2020
	191	115,75	2020
	126	115,5	2020
	180	115	2020
	87	114,75	2020
	3	114,75	2020
	78	114,5	2020
	176	114	2020
	75	113,75	2020
	111	113,75	2020
	147	113,75	2020
	134	113,5	2020
	34	113,5	2020
	234	113,5	2020
	77	113,25	2020
	121	113,25	2020
	152	113	2020
	192	113	2020
	139	112,75	2020
	86	112,5	2020
	106	112,5	2020
	101	112,25	2020
	101	112,25	2020
	113	112,25	2020
	33	112,25	2020
	240	112	2020
	147	111,75	2020
	120	111	2020
	54	110,5	2020
	0	110	2020
	71	109,75	2020
	199	109,75	2020
	250	109,5	2020
	150	109,5	2020
	79	108,75	2020
	143	108,75	2020
	130	108,5	2020
	189	108,25	2020
	160	108	2020
	112	108	2020
	103	107,75	2020
	150	107,5	2020
	229	107,25	2020
	145	107,25	2020
	119	106,75	2020
	27	106,75	2020
	194	106,5	2020
	50	106,5	2020

	117	106,25	2020
	96	106	2020
	84	106	2020
	102	105,5	2020
	18	105,5	2020
	109	105,25	2020
	156	105	2020
	103	104,75	2020
	174	104,5	2020
	93	104,25	2020
	164	104	2020
	192	104	2020
	163	103,75	2020
	86	103,5	2020
	95	102,75	2020
	139	102,75	2020
	82	102,5	2020
	169	102,25	2020
	136	102	2020
	155	101,75	2020
	123	101,75	2020
	66	101,5	2020
	38	101,5	2020
	128	101	2020
	242	100,5	2020
	102	100,5	2020
	214	100,5	2020
	93	100,25	2020
	20	100	2020
	104	100	2020
	86	99,5	2020
	122	99,5	2020
	204	99	2020
	67	98,75	2020
	98	98,5	2020
	117	98,25	2020
	45	98,25	2020
	9	98,25	2020
	161	98,25	2020
	69	98,25	2020
	148	98	2020
	150	97,5	2020
	134	97,5	2020
	70	97,5	2020
	130	97,5	2020
	92	97	2020
	147	96,75	2020
	35	96,75	2020
	94	96,5	2020
	66	96,5	2020

	41	96,25	2020
	232	96	2020
	112	96	2020
	171	95,75	2020
	79	95,75	2020
	155	95,75	2020
	134	95,5	2020
	174	95,5	2020
	113	95,25	2020
	151	94,75	2020
	178	94,5	2020
	81	94,25	2020
	101	94,25	2020
	133	94,25	2020
	112	94	2020
	155	93,75	2020
	95	93,75	2020
	41	93,25	2020
	137	93,25	2020
	137	93,25	2020
	73	93,25	2020
	60	93	2020
	111	92,75	2020
	118	92,5	2020
	62	92,5	2020
	142	92,5	2020
	109	92,25	2020
	169	92,25	2020
	44	92	2020
	14	91,5	2020
	25	91,25	2020
	145	91,25	2020
	172	91	2020
	172	91	2020
	87	90,75	2020
	102	90,5	2020
	110	90,5	2020
	69	90,25	2020
	41	90,25	2020
	0	90	2020
	40	90	2020
	76	90	2020
	95	89,75	2020
	51	89,75	2020
	50	89,5	2020
	134	89,5	2020
	57	89,25	2020
	97	89,25	2020
	65	89,25	2020
	112	89	2020

	60	89	2020
	104	89	2020
	106	88,5	2020
	90	88,5	2020
	66	88,5	2020
	87	87,75	2020
	91	87,75	2020
	38	87,5	2020
	97	87,25	2020
	173	87,25	2020
	24	87	2020
	136	87	2020
	170	86,5	2020
	286	86,5	2020
	85	86,25	2020
	67	85,75	2020
	46	85,5	2020
	30	85,5	2020
	61	85,25	2020
	161	85,25	2020
	44	85	2020
	94	84,5	2020
	34	84,5	2020
	78	84,5	2020
	97	84,25	2020
	136	84	2020
	84	84	2020
	95	83,75	2020
	143	83,75	2020
	47	83,75	2020
	83	83,75	2020
	77	83,25	2020
	81	83,25	2020
	93	83,25	2020
	121	83,25	2020
	120	83	2020
	131	82,75	2020
	51	82,75	2020
	118	82,5	2020
	57	82,25	2020
	25	82,25	2020
	62	81,5	2020
	25	81,25	2020
	205	81,25	2020
	52	81	2020
	36	81	2020
	92	81	2020
	84	81	2020
	84	81	2020
	71	80,75	2020

	7	80,75	2020
	91	80,75	2020
	114	80,5	2020
	42	80,5	2020
	98	80,5	2020
	181	80,25	2020
	80	80	2020
	0	80	2020
	76	80	2020
	76	80	2020
	95	79,75	2020
	97	79,25	2020
	44	79	2020
	16	79	2020
	151	78,75	2020
	102	78,5	2020
	121	78,25	2020
	93	78,25	2020
	100	78	2020
	100	78	2020
	103	77,75	2020
	199	77,75	2020
	74	77,5	2020
	86	77,5	2020
	69	77,25	2020
	29	77,25	2020
	132	77	2020
	48	77	2020
	36	77	2020
	103	76,75	2020
	134	76,5	2020
	96	76	2020
	42	75,5	2020
	174	75,5	2020
	161	75,25	2020
	53	75,25	2020
	88	75	2020
	28	75	2020
	0	75	2020
	103	74,75	2020
	66	74,5	2020
	86	74,5	2020
	89	74,25	2020
	69	74,25	2020
	89	74,25	2020
	64	74	2020
	55	73,75	2020
	47	73,75	2020
	114	73,5	2020
	78	73,5	2020

	120	73	2020
	150	72,5	2020
	210	72,5	2020
	82	72,5	2020
	108	72	2020
	96	72	2020
	36	72	2020
	114	71,5	2020
	94	71,5	2020
	18	71,5	2020
	43	70,75	2020
	95	70,75	2020
	123	70,75	2020
	42	70,5	2020
	53	70,25	2020
	99	69,75	2020
	63	69,75	2020
	71	69,75	2020
	67	69,75	2020
	71	69,75	2020
	102	69,5	2020
	138	69,5	2020
	61	69,25	2020
	29	69,25	2020
	60	69	2020
	104	69	2020
	59	68,75	2020
	87	68,75	2020
	17	68,25	2020
	53	68,25	2020
	61	68,25	2020
	84	68	2020
	48	68	2020
	84	68	2020
	55	67,75	2020
	79	67,75	2020
	87	67,75	2020
	119	67,75	2020
	59	67,75	2020
	210	67,5	2020
	65	67,25	2020
	75	66,75	2020
	99	66,75	2020
	98	66,5	2020
	122	66,5	2020
	58	66,5	2020
	90	66,5	2020
	84	66	2020
	84	66	2020
	128	66	2020

	44	66	2020
	72	66	2020
	46	65,5	2020
	105	65,25	2020
	13	65,25	2020
	40	65	2020
	40	65	2020
	20	65	2020
	59	64,75	2020
	151	64,75	2020
	14	64,5	2020
	102	64,5	2020
	97	64,25	2020
	156	64	2020
	55	63,75	2020
	135	63,75	2020
	35	63,75	2020
	87	63,75	2020
	114	63,5	2020
	42	63,5	2020
	69	63,25	2020
	112	63	2020
	91	62,75	2020
	46	62,5	2020
	80	62	2020
	48	62	2020
	48	62	2020
	39	61,75	2020
	111	61,75	2020
	79	61,75	2020
	123	61,75	2020
	6	61,5	2020
	66	61,5	2020
	86	61,5	2020
	37	61,25	2020
	49	61,25	2020
	41	61,25	2020
	96	61	2020
	19	60,75	2020
	6	60,5	2020
	34	60,5	2020
	70	60,5	2020
	94	60,5	2020
	53	60,25	2020
	21	60,25	2020
	0	60	2020
	112	60	2020
	156	60	2020
	116	60	2020
	63	59,75	2020

	54	59,5	2020
	85	59,25	2020
	57	59,25	2020
	69	59,25	2020
	108	59	2020
	48	59	2020
	48	59	2020
	43	58,75	2020
	35	58,75	2020
	91	58,75	2020
	95	58,75	2020
	78	58,5	2020
	42	58,5	2020
	62	58,5	2020
	78	58,5	2020
	53	58,25	2020
	56	58	2020
	144	58	2020
	76	58	2020
	27	57,75	2020
	51	57,75	2020
	19	57,75	2020
	15	57,75	2020
	37	57,25	2020
	49	57,25	2020
	89	57,25	2020
	65	57,25	2020
	36	57	2020
	96	57	2020
	7	56,75	2020
	127	56,75	2020
	15	56,75	2020
	86	56,5	2020
	42	56,5	2020
	46	56,5	2020
	133	56,25	2020
	45	56,25	2020
	76	56	2020
	112	56	2020
	68	56	2020
	108	56	2020
	27	55,75	2020
	71	55,75	2020
	95	55,75	2020
	6	55,5	2020
	70	55,5	2020
	17	55,25	2020
	9	55,25	2020
	77	55,25	2020
	91	54,75	2020

	147	54,75	2020
	90	54,5	2020
	42	54,5	2020
	86	54,5	2020
	50	54,5	2020
	25	54,25	2020
	81	54,25	2020
	24	54	2020
	24	54	2020
	72	54	2020
	27	53,75	2020
	14	53,5	2020
	58	53,5	2020
	21	53,25	2020
	111	52,75	2020
	55	52,75	2020
	95	52,75	2020
	55	52,75	2020
	30	52,5	2020
	58	52,5	2020
	65	52,25	2020
	73	52,25	2020
	121	52,25	2020
	33	52,25	2020
	68	52	2020
	95	51,75	2020
	62	51,5	2020
	46	51,5	2020
	58	51,5	2020
	57	51,25	2020
	53	51,25	2020
	41	51,25	2020
	69	51,25	2020
	68	51	2020
	80	51	2020
	52	51	2020
	35	50,75	2020
	55	50,75	2020
	23	50,75	2020
	34	50,5	2020
	78	50,5	2020
	6	50,5	2020
	45	50,25	2020
	45	50,25	2020
	49	50,25	2020
	85	50,25	2020
	49	50,25	2020
	0	50	2020
	120	50	2020
	43	49,75	2020

	111	49,75	2020
	95	49,75	2020
	58	49,5	2020
	86	49,5	2020
	50	49,5	2020
	82	49,5	2020
	77	49,25	2020
	97	49,25	2020
	73	49,25	2020
	35	48,75	2020
	59	48,75	2020
	26	48,5	2020
	42	48,5	2020
	78	48,5	2020
	17	48,25	2020
	37	48,25	2020
	44	48	2020
	0	48	2020
	68	48	2020
	56	48	2020
	108	48	2020
	79	47,75	2020
	66	47,5	2020
	37	47,25	2020
	69	47,25	2020
	77	47,25	2020
	88	47	2020
	56	47	2020
	19	46,75	2020
	86	46,5	2020
	66	46,5	2020
	74	46,5	2020
	38	46,5	2020
	21	46,25	2020
	29	46,25	2020
	45	46,25	2020
	45	46,25	2020
	48	46	2020
	44	46	2020
	68	46	2020
	84	46	2020
	72	46	2020
	12	46	2020
	24	46	2020
	47	45,75	2020
	22	45,5	2020
	62	45,5	2020
	54	45,5	2020
	13	45,25	2020
	25	45,25	2020

	33	45,25	2020
	81	45,25	2020
	24	45	2020
	44	45	2020
	64	45	2020
	23	44,75	2020
	35	44,75	2020
	75	44,75	2020
	34	44,5	2020
	42	44,5	2020
	122	44,5	2020
	61	44,25	2020
	81	44,25	2020
	137	44,25	2020
	9	44,25	2020
	40	44	2020
	72	44	2020
	80	44	2020
	0	44	2020
	67	43,75	2020
	14	43,5	2020
	86	43,5	2020
	54	43,5	2020
	58	43,5	2020
	61	43,25	2020
	69	43,25	2020
	13	43,25	2020
	28	43	2020
	48	43	2020
	28	43	2020
	27	42,75	2020
	7	42,75	2020
	35	42,75	2020
	30	42,5	2020
	54	42,5	2020
	46	42,5	2020
	21	42,25	2020
	20	42	2020
	3	41,75	2020
	39	41,75	2020
	39	41,75	2020
	31	41,75	2020
	10	41,5	2020
	26	41,5	2020
	21	41,25	2020
	81	41,25	2020
	37	41,25	2020
	60	41	2020
	56	41	2020
	0	41	2020

	0	41	2020
	16	41	2020
	3	40,75	2020
	55	40,75	2020
	75	40,75	2020
	51	40,75	2020
	62	40,5	2020
	42	40,5	2020
	53	40,25	2020
	45	40,25	2020
	20	40	2020
	16	40	2020
	64	40	2020
	75	39,75	2020
	3	39,75	2020
	22	39,5	2020
	53	39,25	2020
	37	39,25	2020
	77	39,25	2020
	0	39	2020
	28	39	2020
	24	39	2020
	64	39	2020
	44	39	2020
	76	39	2020
	55	38,75	2020
	39	38,75	2020
	107	38,75	2020
	19	38,75	2020
	38	38,5	2020
	50	38,5	2020
	53	38,25	2020
	13	38,25	2020
	12	38	2020
	20	38	2020
	20	38	2020
	28	38	2020
	52	38	2020
	12	38	2020
	108	38	2020
	7	37,75	2020
	47	37,75	2020
	79	37,75	2020
	19	37,75	2020
	3	37,75	2020
	83	37,75	2020
	29	37,25	2020
	37	37,25	2020
	57	37,25	2020
	73	37,25	2020

	32	37	2020
	32	37	2020
	40	37	2020
	52	37	2020
	43	36,75	2020
	31	36,75	2020
	19	36,75	2020
	35	36,75	2020
	19	36,75	2020
	99	36,75	2020
	58	36,5	2020
	22	36,5	2020
	38	36,5	2020
	17	36,25	2020
	57	36,25	2020
	85	36,25	2020
	17	36,25	2020
	93	36,25	2020
	9	36,25	2020
	24	36	2020
	56	36	2020
	52	36	2020
	76	36	2020
	0	36	2020
	24	36	2020
	36	36	2020
	44	36	2020
	48	36	2020
	80	36	2020
	55	35,75	2020
	27	35,75	2020
	31	35,75	2020
	23	35,75	2020
	19	35,75	2020
	75	35,75	2020
	19	35,75	2020
	34	35,5	2020
	66	35,5	2020
	6	35,5	2020
	58	35,5	2020
	66	35,5	2020
	33	35,25	2020
	21	35,25	2020
	61	35,25	2020
	113	35,25	2020
	0	35	2020
	20	35	2020
	0	35	2020
	12	35	2020
	40	35	2020

	140	35	2020
	15	34,75	2020
	27	34,75	2020
	27	34,75	2020
	39	34,75	2020
	31	34,75	2020
	63	34,75	2020
	18	34,5	2020
	58	34,5	2020
	6	34,5	2020
	50	34,5	2020
	50	34,5	2020
	30	34,5	2020
	89	34,25	2020
	29	34,25	2020
	9	34,25	2020
	9	34,25	2020
	9	34,25	2020
	37	34,25	2020
	56	34	2020
	40	34	2020
	52	34	2020
	84	34	2020
	15	33,75	2020
	27	33,75	2020
	34	33,5	2020
	6	33,5	2020
	38	33,5	2020
	38	33,5	2020
	42	33,5	2020
	58	33,5	2020
	34	33,5	2020
	41	33,25	2020
	13	33,25	2020
	29	33,25	2020
	53	33,25	2020
	53	33,25	2020
	24	33	2020
	0	33	2020
	23	32,75	2020
	51	32,75	2020
	63	32,75	2020
	59	32,75	2020
	19	32,75	2020
	67	32,75	2020
	83	32,75	2020
	19	32,75	2020
	39	32,75	2020
	22	32,5	2020
	54	32,5	2020

	90	32,5	2020
	22	32,5	2020
	41	32,25	2020
	13	32,25	2020
	57	32,25	2020
	33	32,25	2020
	57	32,25	2020
	17	32,25	2020
	49	32,25	2020
	57	32,25	2020
	0	32	2020
	0	32	2020
	48	32	2020
	52	32	2020
	40	32	2020
	3	31,75	2020
	47	31,75	2020
	63	31,75	2020
	22	31,5	2020
	86	31,5	2020
	62	31,5	2020
	29	31,25	2020
	29	31,25	2020
	69	31,25	2020
	9	31,25	2020
	25	31,25	2020
	0	31	2020
	28	31	2020
	56	31	2020
	12	31	2020
	12	31	2020
	56	31	2020
	83	30,75	2020
	10	30,5	2020
	26	30,5	2020
	82	30,5	2020
	58	30,5	2020
	62	30,5	2020
	33	30,25	2020
	37	30,25	2020
	9	30,25	2020
	0	30	2020
	0	30	2020
	0	30	2020
	12	30	2020
	44	30	2020
	52	30	2020
	0	30	2020
	0	30	2020
	16	30	2020

	52	30	2020
	59	29,75	2020
	27	29,75	2020
	47	29,75	2020
	99	29,75	2020
	10	29,5	2020
	70	29,5	2020
	46	29,5	2020
	70	29,5	2020
	22	29,5	2020
	26	29,5	2020
	57	29,25	2020
	9	29,25	2020
	37	29,25	2020
	21	29,25	2020
	45	29,25	2020
	25	29,25	2020
	45	29,25	2020
	65	29,25	2020
	69	29,25	2020
	36	29	2020
	34	28,5	2020
	10	28,5	2020
	38	28,5	2020
	42	28,5	2020
	65	28,25	2020
	25	28,25	2020
	9	28,25	2020
	45	28,25	2020
	53	28,25	2020
	12	28	2020
	36	28	2020
	40	28	2020
	63	27,75	2020
	23	27,75	2020
	35	27,75	2020
	34	27,5	2020
	42	27,5	2020
	34	27,5	2020
	62	27,5	2020
	9	27,25	2020
	29	27,25	2020
	41	27,25	2020
	13	27,25	2020
	33	27,25	2020
	25	27,25	2020
	81	27,25	2020
	12	27	2020
	60	27	2020
	28	27	2020

	51	26,75	2020
	43	26,75	2020
	3	26,75	2020
	23	26,75	2020
	27	26,75	2020
	43	26,75	2020
	6	26,5	2020
	10	26,5	2020
	6	26,5	2020
	22	26,5	2020
	30	26,5	2020
	38	26,5	2020
	6	26,5	2020
	62	26,5	2020
	45	26,25	2020
	17	26,25	2020
	37	26,25	2020
	13	26,25	2020
	9	26,25	2020
	21	26,25	2020
	25	26,25	2020
	16	26	2020
	28	26	2020
	64	26	2020
	32	26	2020
	32	26	2020
	27	25,75	2020
	31	25,75	2020
	7	25,75	2020
	3	25,75	2020
	43	25,75	2020
	19	25,75	2020
	35	25,75	2020
	3	25,75	2020
	95	25,75	2020
	25	25,25	2020
	37	25,25	2020
	20	25	2020
	40	25	2020
	60	25	2020
	16	25	2020
	12	25	2020
	32	25	2020
	59	24,75	2020
	15	24,75	2020
	19	24,75	2020
	43	24,75	2020
	63	24,75	2020
	22	24,5	2020
	22	24,5	2020

	42	24,5	2020
	54	24,5	2020
	26	24,5	2020
	38	24,5	2020
	82	24,5	2020
	25	24,25	2020
	21	24,25	2020
	53	24,25	2020
	9	24,25	2020
	32	24	2020
	12	24	2020
	40	24	2020
	48	24	2020
	32	24	2020
	23	23,75	2020
	47	23,75	2020
	47	23,75	2020
	75	23,75	2020
	75	23,75	2020
	22	23,5	2020
	38	23,5	2020
	38	23,5	2020
	33	23,25	2020
	17	23,25	2020
	33	23,25	2020
	33	23,25	2020
	13	23,25	2020
	57	23,25	2020
	93	23,25	2020
	32	23	2020
	24	23	2020
	44	23	2020
	64	23	2020
	12	23	2020
	20	23	2020
	48	23	2020
	3	22,75	2020
	15	22,75	2020
	3	22,75	2020
	19	22,75	2020
	3	22,75	2020
	35	22,75	2020
	55	22,75	2020
	55	22,75	2020
	6	22,5	2020
	10	22,5	2020
	42	22,5	2020
	42	22,5	2020
	18	22,5	2020
	42	22,5	2020

	18	22,5	2020
	54	22,5	2020
	9	22,25	2020
	21	22,25	2020
	29	22,25	2020
	9	22,25	2020
	13	22,25	2020
	13	22,25	2020
	29	22,25	2020
	17	22,25	2020
	25	22,25	2020
	33	22,25	2020
	45	22,25	2020
	53	22,25	2020
	20	22	2020
	60	22	2020
	12	22	2020
	16	22	2020
	60	22	2020
	7	21,75	2020
	39	21,75	2020
	3	21,75	2020
	27	21,75	2020
	23	21,75	2020
	35	21,75	2020
	39	21,75	2020
	51	21,75	2020
	7	21,75	2020
	31	21,75	2020
	39	21,75	2020
	39	21,75	2020
	18	21,5	2020
	6	21,5	2020
	18	21,5	2020
	38	21,5	2020
	10	21,5	2020
	46	21,5	2020
	30	21,5	2020
	66	21,5	2020
	18	21,5	2020
	58	21,5	2020
	9	21,25	2020
	45	21,25	2020
	9	21,25	2020
	33	21,25	2020
	9	21,25	2020
	13	21,25	2020
	25	21,25	2020
	33	21,25	2020
	37	21,25	2020

	49	21,25	2020
	65	21,25	2020
	12	21	2020
	44	21	2020
	12	21	2020
	16	21	2020
	28	21	2020
	28	21	2020
	44	21	2020
	52	21	2020
	84	21	2020
	3	20,75	2020
	3	20,75	2020
	3	20,75	2020
	7	20,75	2020
	19	20,75	2020
	19	20,75	2020
	19	20,75	2020
	27	20,75	2020
	27	20,75	2020
	27	20,75	2020
	31	20,75	2020
	59	20,75	2020
	6	20,5	2020
	42	20,5	2020
	22	20,5	2020
	6	20,5	2020
	6	20,5	2020
	10	20,5	2020
	21	20,25	2020
	9	20,25	2020
	21	20,25	2020
	21	20,25	2020
	53	20,25	2020
	0	20	2020
	20	20	2020
	20	20	2020
	0	20	2020
	0	20	2020
	0	20	2020
	20	20	2020
	52	20	2020
	24	20	2020
	40	20	2020
	3	19,75	2020
	3	19,75	2020
	31	19,75	2020
	59	19,75	2020
	59	19,75	2020
	31	19,75	2020

	10	19,5	2020
	6	19,5	2020
	22	19,5	2020
	6	19,5	2020
	6	19,5	2020
	6	19,5	2020
	34	19,5	2020
	9	19,25	2020
	29	19,25	2020
	13	19,25	2020
	13	19,25	2020
	29	19,25	2020
	28	19	2020
	36	19	2020
	16	19	2020
	36	19	2020
	20	19	2020
	16	19	2020
	16	19	2020
	20	19	2020
	40	19	2020
	7	18,75	2020
	15	18,75	2020
	19	18,75	2020
	19	18,75	2020
	31	18,75	2020
	75	18,75	2020
	10	18,5	2020
	14	18,5	2020
	6	18,5	2020
	34	18,5	2020
	6	18,5	2020
	10	18,5	2020
	18	18,5	2020
	54	18,5	2020
	13	18,25	2020
	25	18,25	2020
	29	18,25	2020
	12	18	2020
	12	18	2020
	16	18	2020
	0	18	2020
	0	18	2020
	0	18	2020
	0	18	2020
	16	18	2020
	24	18	2020
	24	18	2020
	16	18	2020
	52	18	2020

	0	18	2020
	0	18	2020
	0	18	2020
	0	18	2020
	0	18	2020
	12	18	2020
	28	18	2020
	32	18	2020
	48	18	2020
	72	18	2020
	7	17,75	2020
	27	17,75	2020
	35	17,75	2020
	15	17,75	2020
	15	17,75	2020
	15	17,75	2020
	27	17,75	2020
	35	17,75	2020
	55	17,75	2020
	10	17,5	2020
	6	17,5	2020
	42	17,5	2020
	6	17,5	2020
	18	17,5	2020
	46	17,5	2020
	13	17,25	2020
	29	17,25	2020
	25	17,25	2020
	25	17,25	2020
	25	17,25	2020
	25	17,25	2020
	69	17,25	2020
	0	17	2020
	12	17	2020
	16	17	2020
	40	17	2020
	12	17	2020
	28	17	2020
	7	16,75	2020
	7	16,75	2020
	3	16,75	2020
	7	16,75	2020
	15	16,75	2020
	23	16,75	2020
	23	16,75	2020
	47	16,75	2020
	47	16,75	2020
	3	16,75	2020
	19	16,75	2020
	23	16,75	2020

	31	16,75	2020
	31	16,75	2020
	59	16,75	2020
	6	16,5	2020
	10	16,5	2020
	18	16,5	2020
	10	16,5	2020
	10	16,5	2020
	22	16,5	2020
	25	16,25	2020
	25	16,25	2020
	17	16,25	2020
	17	16,25	2020
	45	16,25	2020
	45	16,25	2020
	45	16,25	2020
	9	16,25	2020
	25	16,25	2020
	65	16,25	2020
	65	16,25	2020
	16	16	2020
	16	16	2020
	16	16	2020
	16	16	2020
	24	16	2020
	16	16	2020
	32	16	2020
	56	16	2020
	15	15,75	2020
	3	15,75	2020
	3	15,75	2020
	3	15,75	2020
	19	15,75	2020
	19	15,75	2020
	31	15,75	2020
	39	15,75	2020
	22	15,5	2020
	22	15,5	2020
	6	15,5	2020
	14	15,5	2020
	14	15,5	2020
	14	15,5	2020
	14	15,5	2020
	22	15,5	2020
	6	15,5	2020
	6	15,5	2020
	6	15,5	2020
	6	15,5	2020
	6	15,5	2020
	18	15,5	2020

	18	15,5	2020
	22	15,5	2020
	22	15,5	2020
	13	15,25	2020
	25	15,25	2020
	13	15,25	2020
	13	15,25	2020
	13	15,25	2020
	21	15,25	2020
	41	15,25	2020
	9	15,25	2020
	37	15,25	2020
	41	15,25	2020
	0	15	2020
	0	15	2020
	0	15	2020
	0	15	2020
	12	15	2020
	12	15	2020
	0	15	2020
	0	15	2020
	12	15	2020
	20	15	2020
	20	15	2020
	40	15	2020
	0	15	2020
	0	15	2020
	12	15	2020
	12	15	2020
	12	15	2020
	24	15	2020
	3	14,75	2020
	3	14,75	2020
	19	14,75	2020
	19	14,75	2020
	23	14,75	2020
	31	14,75	2020
	3	14,75	2020
	3	14,75	2020
	31	14,75	2020
	31	14,75	2020
	51	14,75	2020
	38	14,5	2020
	10	14,5	2020
	10	14,5	2020
	10	14,5	2020
	10	14,5	2020
	6	14,5	2020
	22	14,5	2020
	22	14,5	2020

	22	14,5	2020
	22	14,5	2020
	22	14,5	2020
	50	14,5	2020
	58	14,5	2020
	17	14,25	2020
	9	14,25	2020
	13	14,25	2020
	17	14,25	2020
	9	14,25	2020
	13	14,25	2020
	17	14,25	2020
	21	14,25	2020
	21	14,25	2020
	41	14,25	2020
	41	14,25	2020
	12	14	2020
	16	14	2020
	16	14	2020
	16	14	2020
	28	14	2020
	28	14	2020
	36	14	2020
	0	14	2020
	0	14	2020
	12	14	2020
	36	14	2020
	36	14	2020
	40	14	2020
	7	13,75	2020
	7	13,75	2020
	15	13,75	2020
	15	13,75	2020
	7	13,75	2020
	15	13,75	2020
	7	13,75	2020
	7	13,75	2020
	7	13,75	2020
	19	13,75	2020
	19	13,75	2020
	19	13,75	2020
	19	13,75	2020
	19	13,75	2020
	19	13,75	2020
	26	13,5	2020
	14	13,5	2020
	14	13,5	2020
	6	13,5	2020
	14	13,5	2020
	14	13,5	2020

	26	13,5	2020
	34	13,5	2020
	6	13,5	2020
	22	13,5	2020
	26	13,5	2020
	54	13,5	2020
	13	13,25	2020
	13	13,25	2020
	9	13,25	2020
	13	13,25	2020
	13	13,25	2020
	25	13,25	2020
	25	13,25	2020
	45	13,25	2020
	45	13,25	2020
	53	13,25	2020
	16	13	2020
	12	13	2020
	24	13	2020
	32	13	2020
	32	13	2020
	31	12,25	2020
	3	12,25	2020
	3	12,25	2020
	3	12,25	2020
	23	12,25	2020
	15	12,25	2020
	19	12,25	2020
	23	12,25	2020
	10	12,5	2020
	10	12,5	2020
	10	12,5	2020
	10	12,5	2020
	6	12,5	2020
	10	12,5	2020
	6	12,5	2020
	6	12,5	2020
	6	12,5	2020
	6	12,5	2020
	6	12,5	2020
	9	12,25	2020
	9	12,25	2020
	13	12,25	2020
	21	12,25	2020
	29	12,25	2020
	9	12,25	2020
	13	12,25	2020
	13	12,25	2020
	21	12,25	2020
	29	12,25	2020

	33	12,25	2020
	33	12,25	2020
	41	12,25	2020
	41	12,25	2020
	0	12	2020
	0	12	2020
	0	12	2020
	20	12	2020
	0	12	2020
	0	12	2020
	0	12	2020
	0	12	2020
	0	12	2020
	0	12	2020
	0	12	2020
	20	12	2020
	12	12	2020
	24	12	2020
	28	12	2020
	32	12	2020
	40	12	2020
	7	11,75	2020
	7	11,75	2020
	7	11,75	2020
	7	11,75	2020
	7	11,75	2020
	7	11,75	2020
	19	11,75	2020
	19	11,75	2020
	3	11,75	2020
	3	11,75	2020
	3	11,75	2020
	3	11,75	2020
	15	11,75	2020
	15	11,75	2020
	31	11,75	2020
	39	11,75	2020
	47	11,75	2020
	6	11,5	2020
	10	11,5	2020
	6	11,5	2020
	6	11,5	2020
	6	11,5	2020
	18	11,5	2020
	18	11,5	2020
	6	11,5	2020
	6	11,5	2020
	6	11,5	2020
	6	11,5	2020
	6	11,5	2020

	18	11,5	2020
	18	11,5	2020
	22	11,5	2020
	22	11,5	2020
	26	11,5	2020
	38	11,5	2020
	17	11,25	2020
	9	11,25	2020
	17	11,25	2020
	17	11,25	2020
	9	11,25	2020
	9	11,25	2020
	9	11,25	2020
	21	11,25	2020
	37	11,25	2020
	45	11,25	2020
	45	11,25	2020
	45	11,25	2020
	45	11,25	2020
	16	11	2020
	24	11	2020
	0	11	2020
	0	11	2020
	12	11	2020
	16	11	2020
	16	11	2020
	20	11	2020
	24	11	2020
	28	11	2020
	3	10,75	2020
	3	10,75	2020
	3	10,75	2020
	7	10,75	2020
	3	10,75	2020
	3	10,75	2020
	15	10,75	2020
	15	10,75	2020
	19	10,75	2020
	27	10,75	2020
	27	10,75	2020
	35	10,75	2020
	35	10,75	2020
	43	10,75	2020
	6	10,5	2020
	14	10,5	2020
	22	10,5	2020
	6	10,5	2020
	6	10,5	2020
	6	10,5	2020
	6	10,5	2020

	6	10,5	2020
	6	10,5	2020
	34	10,5	2020
	13	10,25	2020
	13	10,25	2020
	13	10,25	2020
	13	10,25	2020
	13	10,25	2020
	13	10,25	2020
	13	10,25	2020
	13	10,25	2020
	13	10,25	2020
	13	10,25	2020
	13	10,25	2020
	17	10,25	2020
	21	10,25	2020
	21	10,25	2020
	25	10,25	2020
	33	10,25	2020
	33	10,25	2020
	41	10,25	2020
	0	10	2020
	0	10	2020
	0	10	2020
	0	10	2020
	0	10	2020
	0	10	2020
	0	10	2020
	0	10	2020
	20	10	2020
	20	10	2020
	0	10	2020
	0	10	2020
	0	10	2020
	0	10	2020
	0	10	2020
	0	10	2020
	0	10	2020
	0	10	2020
	0	10	2020
	0	10	2020
	0	10	2020
	0	10	2020
	0	10	2020
	0	10	2020
	0	10	2020
	0	10	2020
	0	10	2020
	0	10	2020
	0	10	2020

	0	10	2020
	0	10	2020
	0	10	2020
	0	10	2020
	12	10	2020
	20	10	2020
	0	10	2020
	0	10	2020
	0	10	2020
	0	10	2020
	12	10	2020
	12	10	2020
	12	10	2020
	16	10	2020
	16	10	2020
	16	10	2020
	20	10	2020
	20	10	2020
	24	10	2020
	40	10	2020
	40	10	2020
	3	9,75	2020
	3	9,75	2020
	3	9,75	2020
	3	9,75	2020
	3	9,75	2020
	3	9,75	2020
	3	9,75	2020
	3	9,75	2020
	3	9,75	2020
	3	9,75	2020
	3	9,75	2020
	3	9,75	2020
	19	9,75	2020
	23	9,75	2020
	31	9,75	2020
	31	9,75	2020
	10	9,5	2020
	10	9,5	2020
	6	9,5	2020
	6	9,5	2020
	10	9,5	2020
	22	9,5	2020
	22	9,5	2020
	22	9,5	2020
	30	9,5	2020
	38	9,5	2020
	9	9,25	2020
	9	9,25	2020
	17	9,25	2020

	9	9,25	2020
	9	9,25	2020
	9	9,25	2020
	9	9,25	2020
	9	9,25	2020
	9	9,25	2020
	9	9,25	2020
	13	9,25	2020
	37	9,25	2020
	0	9	2020
	0	9	2020
	0	9	2020
	0	9	2020
	0	9	2020
	0	9	2020
	0	9	2020
	0	9	2020
	0	9	2020
	0	9	2020
	0	9	2020
	0	9	2020
	0	9	2020
	0	9	2020
	0	9	2020
	0	9	2020
	0	9	2020
	0	9	2020
	0	9	2020
	0	9	2020
	12	9	2020
	12	9	2020
	12	9	2020
	12	9	2020
	12	9	2020
	12	9	2020
	16	9	2020
	28	9	2020
	7	8,75	2020
	7	8,75	2020
	7	8,75	2020
	7	8,75	2020
	7	8,75	2020
	15	8,75	2020
	15	8,75	2020
	3	8,75	2020
	15	8,75	2020
	19	8,75	2020
	19	8,75	2020
	19	8,75	2020
	19	8,75	2020

	19	8,75	2020
	19	8,75	2020
	27	8,75	2020
	35	8,75	2020
	6	8,5	2020
	6	8,5	2020
	6	8,5	2020
	6	8,5	2020
	14	8,5	2020
	14	8,5	2020
	14	8,5	2020
	14	8,5	2020
	6	8,5	2020
	6	8,5	2020
	6	8,5	2020
	6	8,5	2020
	6	8,5	2020
	6	8,5	2020
	6	8,5	2020
	6	8,5	2020
	6	8,5	2020
	6	8,5	2020
	6	8,5	2020
	10	8,5	2020
	14	8,5	2020
	18	8,5	2020
	18	8,5	2020
	34	8,5	2020
	34	8,5	2020
	13	8,25	2020
	13	8,25	2020
	13	8,25	2020
	13	8,25	2020
	13	8,25	2020
	9	8,25	2020
	13	8,25	2020
	13	8,25	2020
	17	8,25	2020
	25	8,25	2020
	25	8,25	2020
	25	8,25	2020
	33	8,25	2020
	12	8	2020
	12	8	2020
	12	8	2020
	16	8	2020
	16	8	2020
	16	8	2020
	16	8	2020
	24	8	2020

	24	8	2020
	32	8	2020
	32	8	2020
	32	8	2020
	3	7,75	2020
	3	7,75	2020
	3	7,75	2020
	3	7,75	2020
	3	7,75	2020
	3	7,75	2020
	3	7,75	2020
	3	7,75	2020
	3	7,75	2020
	3	7,75	2020
	3	7,75	2020
	3	7,75	2020
	3	7,75	2020
	3	7,75	2020
	15	7,75	2020
	23	7,75	2020
	10	7,5	2020
	10	7,5	2020
	10	7,5	2020
	10	7,5	2020
	10	7,5	2020
	10	7,5	2020
	6	7,5	2020
	6	7,5	2020
	6	7,5	2020
	6	7,5	2020
	10	7,5	2020
	10	7,5	2020
	10	7,5	2020
	10	7,5	2020
	14	7,5	2020
	22	7,5	2020
	9	7,25	2020
	9	7,25	2020
	9	7,25	2020
	9	7,25	2020
	9	7,25	2020
	9	7,25	2020
	9	7,25	2020
	13	7,25	2020
	13	7,25	2020
	13	7,25	2020
	13	7,25	2020
	13	7,25	2020
	21	7,25	2020
	29	7,25	2020

29	7,25	2020
0	7	2020
0	7	2020
0	7	2020
0	7	2020
0	7	2020
0	7	2020
0	7	2020
0	7	2020
0	7	2020
0	7	2020
0	7	2020
0	7	2020
0	7	2020
0	7	2020
0	7	2020
0	7	2020
0	7	2020
0	7	2020
0	7	2020
0	7	2020
0	7	2020
0	7	2020
0	7	2020
0	7	2020
0	7	2020
0	7	2020
0	7	2020
0	7	2020
0	7	2020
0	7	2020
12	7	2020
12	7	2020
12	7	2020
12	7	2020
12	7	2020
12	7	2020
20	7	2020
20	7	2020
20	7	2020
20	7	2020
20	7	2020
7	6,75	2020
7	6,75	2020
7	6,75	2020
7	6,75	2020
7	6,75	2020
7	6,75	2020
7	6,75	2020
7	6,75	2020

	7	6,75	2020
	7	6,75	2020
	7	6,75	2020
	7	6,75	2020
	7	6,75	2020
	7	6,75	2020
	7	6,75	2020
	3	6,75	2020
	3	6,75	2020
	3	6,75	2020
	3	6,75	2020
	3	6,75	2020
	3	6,75	2020
	7	6,75	2020
	7	6,75	2020
	19	6,75	2020
	19	6,75	2020
	27	6,75	2020
	27	6,75	2020
	27	6,75	2020
	27	6,75	2020
	6	6,5	2020
	6	6,5	2020
	6	6,5	2020
	6	6,5	2020
	6	6,5	2020
	6	6,5	2020
	6	6,5	2020
	6	6,5	2020
	6	6,5	2020
	6	6,5	2020
	6	6,5	2020
	6	6,5	2020
	6	6,5	2020
	6	6,5	2020
	6	6,5	2020
	6	6,5	2020
	10	6,5	2020
	10	6,5	2020
	10	6,5	2020
	10	6,5	2020
	10	6,5	2020
	10	6,5	2020
	10	6,5	2020
	9	6,25	2020
	9	6,25	2020
	9	6,25	2020
	9	6,25	2020
	9	6,25	2020
	9	6,25	2020

	9	6,25	2020
	9	6,25	2020
	9	6,25	2020
	9	6,25	2020
	9	6,25	2020
	9	6,25	2020
	9	6,25	2020
	9	6,25	2020
	9	6,25	2020
	9	6,25	2020
	9	6,25	2020
	17	6,25	2020
	17	6,25	2020
	25	6,25	2020
	25	6,25	2020
	25	6,25	2020
	25	6,25	2020
	0	6	2020
	16	6	2020
	16	6	2020
	16	6	2020
	16	6	2020
	24	6	2020
	24	6	2020
	24	6	2020
	3	5,75	2020
	3	5,75	2020
	3	5,75	2020
	3	5,75	2020
	3	5,75	2020
	3	5,75	2020
	3	5,75	2020
	3	5,75	2020
	3	5,75	2020
	3	5,75	2020
	3	5,75	2020
	3	5,75	2020
	3	5,75	2020
	3	5,75	2020
	3	5,75	2020
	3	5,75	2020
	3	5,75	2020
	3	5,75	2020
	3	5,75	2020
	3	5,75	2020
	3	5,75	2020
	3	5,75	2020
	3	5,75	2020
	3	5,75	2020
	3	5,75	2020

	3	5,75	2020
	3	5,75	2020
	3	5,75	2020
	3	5,75	2020
	3	5,75	2020
	3	5,75	2020
	3	5,75	2020
	7	5,75	2020
	7	5,75	2020
	7	5,75	2020
	7	5,75	2020
	7	5,75	2020
	7	5,75	2020
	15	5,75	2020
	15	5,75	2020
	15	5,75	2020
	6	5,5	2020
	6	5,5	2020
	6	5,5	2020
	6	5,5	2020
	6	5,5	2020
	6	5,5	2020
	6	5,5	2020
	6	5,5	2020
	6	5,5	2020
	6	5,5	2020
	6	5,5	2020
	6	5,5	2020
	6	5,5	2020
	6	5,5	2020
	6	5,5	2020
	6	5,5	2020
	6	5,5	2020
	6	5,5	2020
	6	5,5	2020
	6	5,5	2020
	14	5,5	2020
	13	1,5	2020
	13	1,5	2020
	13	1,5	2020
	13	1,5	2020
	13	1,5	2020
	13	1,5	2020
	13	1,5	2020
	13	1,5	2020
	13	1,5	2020
	13	1,5	2020
	13	1,5	2020
	13	1,5	2020
	13	1,5	2020

	21	1,5	2020
	21	1,5	2020
	21	1,5	2020
	21	1,5	2020
	21	1,5	2020
	21	1,5	2020
	21	1,5	2020
	21	1,5	2020
	21	1,5	2020
	0	5	2020
	0	5	2020
	0	5	2020
	0	5	2020
	0	5	2020
	0	5	2020
	0	5	2020
	0	5	2020
	0	5	2020
	0	5	2020
	0	5	2020
	0	5	2020
	0	5	2020
	0	5	2020
	0	5	2020
	0	5	2020
	0	5	2020
	0	5	2020
	0	5	2020
	0	5	2020
	0	5	2020
	0	5	2020
	0	5	2020
	0	5	2020
	0	5	2020
	0	5	2020
	0	5	2020
	0	5	2020
	0	5	2020
	0	5	2020
	0	5	2020
	0	5	2020
	0	5	2020
	0	5	2020
	0	5	2020
	0	5	2020
	0	5	2020
	0	5	2020
	0	5	2020
	0	5	2020

0	5	2020
0	5	2020
0	5	2020
0	5	2020
0	5	2020
0	5	2020
0	5	2020
0	5	2020
0	5	2020
0	5	2020
0	5	2020
0	5	2020
0	5	2020
0	5	2020
0	5	2020
0	5	2020
0	5	2020
0	5	2020
0	5	2020
0	5	2020
0	5	2020
0	5	2020
0	5	2020
0	5	2020
0	5	2020
0	5	2020
0	5	2020
0	5	2020
0	5	2020
0	5	2020
0	5	2020
0	5	2020
0	5	2020
0	5	2020
0	5	2020
0	5	2020
0	5	2020
0	5	2020
0	5	2020
0	5	2020
0	5	2020
0	5	2020
0	5	2020
0	5	2020
0	5	2020
0	5	2020
0	5	2020
0	5	2020
0	5	2020
0	5	2020

0	5	2020
0	5	2020
0	5	2020
0	5	2020
0	5	2020
0	5	2020
0	5	2020
0	5	2020
0	5	2020
12	5	2020
12	5	2020
12	5	2020
12	5	2020
12	5	2020
12	5	2020
12	5	2020
20	5	2020
20	5	2020
20	5	2020
20	5	2020
20	5	2020
20	5	2020
20	5	2020
3	4,75	2020
3	4,75	2020
3	4,75	2020
3	4,75	2020
3	4,75	2020
3	4,75	2020
3	4,75	2020
3	4,75	2020
3	4,75	2020
3	4,75	2020
3	4,75	2020
3	4,75	2020
3	4,75	2020
3	4,75	2020
3	4,75	2020
3	4,75	2020
3	4,75	2020
3	4,75	2020
3	4,75	2020
3	4,75	2020
3	4,75	2020
3	4,75	2020
3	4,75	2020
3	4,75	2020
3	4,75	2020
3	4,75	2020
3	4,75	2020

3	4,75	2020
19	4,75	2020
19	4,75	2020
19	4,75	2020
10	4,5	2020
10	4,5	2020
10	4,5	2020
10	4,5	2020
10	4,5	2020
10	4,5	2020
10	4,5	2020
10	4,5	2020
10	4,5	2020
18	4,5	2020
18	4,5	2020
18	4,5	2020
18	4,5	2020
18	4,5	2020
9	4,25	2020
9	4,25	2020
9	4,25	2020
9	4,25	2020
9	4,25	2020
9	4,25	2020
9	4,25	2020
9	4,25	2020
9	4,25	2020
9	4,25	2020
9	4,25	2020
9	4,25	2020
9	4,25	2020
9	4,25	2020
9	4,25	2020
9	4,25	2020
9	4,25	2020
17	4,25	2020
0	4	2020
0	4	2020
0	4	2020
0	4	2020
0	4	2020
0	4	2020
0	4	2020
0	4	2020
0	4	2020
0	4	2020
0	4	2020
0	4	2020
0	4	2020
0	4	2020

	0	4	2020
	0	4	2020
	0	4	2020
	0	4	2020
	0	4	2020
	0	4	2020
	0	4	2020
	0	4	2020
	0	4	2020
	0	4	2020
	0	4	2020
	0	4	2020
	16	4	2020
	16	4	2020
	16	4	2020
	16	4	2020
	16	4	2020
	16	4	2020
	7	3,75	2020
	7	3,75	2020
	7	3,75	2020
	7	3,75	2020
	7	3,75	2020
	7	3,75	2020
	7	3,75	2020
	7	3,75	2020
	7	3,75	2020
	7	3,75	2020
	7	3,75	2020
	15	3,75	2020
	15	3,75	2020
	15	3,75	2020
	15	3,75	2020
	15	3,75	2020
	15	3,75	2020
	15	3,75	2020
	15	3,75	2020
	15	3,75	2020
	15	3,75	2020
	15	3,75	2020
	6	3,5	2020
	6	3,5	2020
	6	3,5	2020
	6	3,5	2020
	6	3,5	2020
	6	3,5	2020
	6	3,5	2020
	6	3,5	2020
	6	3,5	2020
	6	3,5	2020

	6	3,5	2020
	6	3,5	2020
	6	3,5	2020
	6	3,5	2020
	6	3,5	2020
	6	3,5	2020
	6	3,5	2020
	6	3,5	2020
	6	3,5	2020
	6	3,5	2020
	6	3,5	2020
	6	3,5	2020
	6	3,5	2020
	6	3,5	2020
	6	3,5	2020
	6	3,5	2020
	6	3,5	2020
	6	3,5	2020
	6	3,5	2020
	6	3,5	2020
	6	3,5	2020
	6	3,5	2020
	6	3,5	2020
	6	3,5	2020
	6	3,5	2020
	6	3,5	2020
	6	3,5	2020
	6	3,5	2020
	6	3,5	2020
	6	3,5	2020
	6	3,5	2020
	6	3,5	2020
	6	3,5	2020
	6	3,5	2020
	6	3,5	2020
	6	3,5	2020
	6	3,5	2020
	6	3,5	2020
	6	3,5	2020
	6	3,5	2020
	6	3,5	2020
	6	3,5	2020
	6	3,5	2020
	6	3,5	2020
	6	3,5	2020
	14	3,5	2020
	14	3,5	2020
	14	3,5	2020
	14	3,5	2020
	14	3,5	2020

	14	3,5	2020
	14	3,5	2020
	14	3,5	2020
	14	3,5	2020
	14	3,5	2020
	14	3,5	2020
	14	3,5	2020
	13	3,25	2020
	13	3,25	2020
	13	3,25	2020
	13	3,25	2020
	13	3,25	2020
	13	3,25	2020
	13	3,25	2020
	13	3,25	2020
	13	3,25	2020
	13	3,25	2020
	13	3,25	2020
	13	3,25	2020
	13	3,25	2020
	13	3,25	2020
	13	3,25	2020
	13	3,25	2020
	13	3,25	2020
	13	3,25	2020
	13	3,25	2020
	13	3,25	2020
	13	3,25	2020
	12	3	2020
	12	3	2020
	12	3	2020
	12	3	2020
	12	3	2020
	12	3	2020
	12	3	2020
	12	3	2020
	12	3	2020
	3	2,75	2020
	3	2,75	2020
	3	2,75	2020
	3	2,75	2020
	3	2,75	2020
	3	2,75	2020
	3	2,75	2020
	3	2,75	2020
	3	2,75	2020
	3	2,75	2020
	3	2,75	2020
	3	2,75	2020
	3	2,75	2020

3	2,75	2020
3	2,75	2020
3	2,75	2020
3	2,75	2020
3	2,75	2020
3	2,75	2020
3	2,75	2020
3	2,75	2020
3	2,75	2020
3	2,75	2020
3	2,75	2020
3	2,75	2020
3	2,75	2020
3	2,75	2020
3	2,75	2020
3	2,75	2020
3	2,75	2020
3	2,75	2020
3	2,75	2020
3	2,75	2020
3	2,75	2020
3	2,75	2020
3	2,75	2020
3	2,75	2020
3	2,75	2020
3	2,75	2020
3	2,75	2020
3	2,75	2020
3	2,75	2020
3	2,75	2020
3	2,75	2020
3	2,75	2020
3	2,75	2020
3	2,75	2020
3	2,75	2020
3	2,75	2020
3	2,75	2020
3	2,75	2020
3	2,75	2020
3	2,75	2020
3	2,75	2020
3	2,75	2020
3	2,75	2020
3	2,75	2020
3	2,75	2020
3	2,75	2020
3	2,75	2020
3	2,75	2020
3	2,75	2020
3	2,75	2020

3	2,75	2020
3	2,75	2020
3	2,75	2020
3	2,75	2020
3	2,75	2020
3	2,75	2020
3	2,75	2020
3	2,75	2020
3	2,75	2020
3	2,75	2020
3	2,75	2020
3	2,75	2020
3	2,75	2020
3	2,75	2020
3	2,75	2020
3	2,75	2020
3	2,75	2020
3	2,75	2020
3	2,75	2020
3	2,75	2020
3	2,75	2020
3	2,75	2020
3	2,75	2020
3	2,75	2020
3	2,75	2020
3	2,75	2020
3	2,75	2020
3	2,75	2020
3	2,75	2020
3	2,75	2020
3	2,75	2020
3	2,75	2020
3	2,75	2020
3	2,75	2020
3	2,75	2020
3	2,75	2020
3	2,75	2020
3	2,75	2020
3	2,75	2020
3	2,75	2020
3	2,75	2020
3	2,75	2020
3	2,75	2020
3	2,75	2020
3	2,75	2020
3	2,75	2020
3	2,75	2020
3	2,75	2020
3	2,75	2020
3	2,75	2020

	3	2,75	2020
	3	2,75	2020
	3	2,75	2020
	3	2,75	2020
	3	2,75	2020
	3	2,75	2020
	3	2,75	2020
	3	2,75	2020
	3	2,75	2020
	3	2,75	2020
	10	2,5	2020
	10	2,5	2020
	10	2,5	2020
	10	2,5	2020
	10	2,5	2020
	10	2,5	2020
	10	2,5	2020
	10	2,5	2020
	10	2,5	2020
	10	2,5	2020
	10	2,5	2020
	10	2,5	2020
	10	2,5	2020
	10	2,5	2020
	10	2,5	2020
	10	2,5	2020
	9	2,25	2020
	9	2,25	2020
	9	2,25	2020
	9	2,25	2020
	9	2,25	2020
	9	2,25	2020
	9	2,25	2020
	9	2,25	2020
	9	2,25	2020
	9	2,25	2020
	9	2,25	2020
	9	2,25	2020
	9	2,25	2020
	9	2,25	2020
	9	2,25	2020
	9	2,25	2020
	9	2,25	2020
	9	2,25	2020
	9	2,25	2020
	9	2,25	2020
	9	2,25	2020
	9	2,25	2020
	0	2	2020
	0	2	2020

0	2	2020
0	2	2020
0	2	2020
0	2	2020
0	2	2020
0	2	2020
0	2	2020
0	2	2020
0	2	2020
0	2	2020
0	2	2020
0	2	2020
0	2	2020
0	2	2020
0	2	2020
0	2	2020
0	2	2020
0	2	2020
0	2	2020
0	2	2020
0	2	2020
0	2	2020
0	2	2020
0	2	2020
0	2	2020
0	2	2020
0	2	2020
0	2	2020
0	2	2020
0	2	2020
0	2	2020
0	2	2020
0	2	2020
0	2	2020
0	2	2020
0	2	2020
0	2	2020
0	2	2020
0	2	2020
0	2	2020
0	2	2020
0	2	2020
0	2	2020
0	2	2020
0	2	2020
0	2	2020
0	2	2020
0	2	2020
0	2	2020
0	2	2020

0	2	2020
0	2	2020
0	2	2020
0	2	2020
0	2	2020
0	2	2020
0	2	2020
0	2	2020
0	2	2020
0	2	2020
0	2	2020
0	2	2020
0	2	2020
0	2	2020
0	2	2020
0	2	2020
0	2	2020
0	2	2020
0	2	2020
0	2	2020
0	2	2020
0	2	2020
0	2	2020
0	2	2020
0	2	2020
0	2	2020
0	2	2020
0	2	2020
0	2	2020
0	2	2020
0	2	2020
0	2	2020
0	2	2020
0	2	2020
0	2	2020
0	2	2020
0	2	2020
0	2	2020
0	2	2020
0	2	2020
0	2	2020
0	2	2020
0	2	2020
0	2	2020
0	2	2020
0	2	2020
0	2	2020
0	2	2020
0	2	2020
0	2	2020

0	2	2020
0	2	2020
0	2	2020
0	2	2020
0	2	2020
0	2	2020
0	2	2020
0	2	2020
0	2	2020
0	2	2020
0	2	2020
0	2	2020
0	2	2020
0	2	2020
0	2	2020
0	2	2020
0	2	2020
0	2	2020
0	2	2020
0	2	2020
0	2	2020
0	2	2020
0	2	2020
0	2	2020
0	2	2020
0	2	2020
0	2	2020
0	2	2020
0	2	2020
0	2	2020
0	2	2020
0	2	2020
0	2	2020
0	2	2020
0	2	2020
0	2	2020
0	2	2020
0	2	2020
0	2	2020
0	2	2020
0	2	2020
0	2	2020
0	2	2020
0	2	2020
0	2	2020
0	2	2020
0	2	2020
0	2	2020
0	2	2020
0	2	2020

0	2	2020
0	2	2020
0	2	2020
0	2	2020
0	2	2020
0	2	2020
0	2	2020
0	2	2020
0	2	2020
0	2	2020
0	2	2020
0	2	2020
0	2	2020
0	2	2020
0	2	2020
0	2	2020
0	2	2020
0	2	2020
0	2	2020
0	2	2020
0	2	2020
0	2	2020
0	2	2020
0	2	2020
0	2	2020
0	2	2020
0	2	2020
0	2	2020
0	2	2020
0	2	2020
0	2	2020
0	2	2020
0	2	2020
0	2	2020
0	2	2020
0	2	2020
0	2	2020
0	2	2020
0	2	2020
0	2	2020
0	2	2020
0	2	2020
0	2	2020
0	2	2020
0	2	2020
0	2	2020
0	2	2020
0	2	2020
0	2	2020
0	2	2020

0	2	2020
0	2	2020
0	2	2020
0	2	2020
0	2	2020
0	2	2020
0	2	2020
0	2	2020
0	2	2020
0	2	2020
0	2	2020
0	2	2020
0	2	2020
0	2	2020
0	2	2020
0	2	2020
0	2	2020
0	2	2020
0	2	2020
0	2	2020
0	2	2020
0	2	2020
0	2	2020
0	2	2020
0	2	2020
0	2	2020
0	2	2020
0	2	2020
0	2	2020
0	2	2020
0	2	2020
7	1,75	2020
7	1,75	2020
7	1,75	2020
7	1,75	2020
7	1,75	2020
7	1,75	2020
7	1,75	2020
7	1,75	2020
7	1,75	2020
7	1,75	2020
7	1,75	2020
7	1,75	2020
7	1,75	2020
7	1,75	2020
7	1,75	2020
7	1,75	2020
7	1,75	2020
7	1,75	2020
7	1,75	2020

	7	1,75	2020
	7	1,75	2020
	7	1,75	2020
	7	1,75	2020
	7	1,75	2020
	7	1,75	2020
	7	1,75	2020
	7	1,75	2020
	7	1,75	2020
	7	1,75	2020
	7	1,75	2020
	7	1,75	2020
	7	1,75	2020
	7	1,75	2020
	7	1,75	2020
	7	1,75	2020
	7	1,75	2020
	7	1,75	2020
	7	1,75	2020
	7	1,75	2020
	7	1,75	2020
	7	1,75	2020
	7	1,75	2020
	7	1,75	2020
	7	1,75	2020
	7	1,75	2020
	7	1,75	2020
	7	1,75	2020
	7	1,75	2020
	7	1,75	2020
	7	1,75	2020
	7	1,75	2020
	7	1,75	2020
	7	1,75	2020
	7	1,75	2020
	7	1,75	2020
	7	1,75	2020
	7	1,75	2020
	7	1,75	2020
	7	1,75	2020
	7	1,75	2020
	7	1,75	2020
	7	1,75	2020
	7	1,75	2020
	7	1,75	2020
	7	1,75	2020
	7	1,75	2020
	7	1,75	2020
	7	1,75	2020
	7	1,75	2020

	7	1,75	2020
	7	1,75	2020
	7	1,75	2020
	7	1,75	2020
	7	1,75	2020
	7	1,75	2020
	6	1,5	2020
	6	1,5	2020
	6	1,5	2020
	6	1,5	2020
	6	1,5	2020
	6	1,5	2020
	6	1,5	2020
	6	1,5	2020
	6	1,5	2020
	6	1,5	2020
	6	1,5	2020
	6	1,5	2020
	6	1,5	2020
	6	1,5	2020
	6	1,5	2020
	6	1,5	2020
	6	1,5	2020
	6	1,5	2020
	6	1,5	2020
	6	1,5	2020
	6	1,5	2020
	6	1,5	2020
	6	1,5	2020
	6	1,5	2020
	6	1,5	2020
	6	1,5	2020
	6	1,5	2020
	6	1,5	2020
	6	1,5	2020
	6	1,5	2020
	6	1,5	2020
	6	1,5	2020
	6	1,5	2020
	6	1,5	2020
	6	1,5	2020
	6	1,5	2020
	6	1,5	2020
	6	1,5	2020
	6	1,5	2020
	6	1,5	2020
	6	1,5	2020
	6	1,5	2020
	6	1,5	2020
	6	1,5	2020

	6	1,5	2020
	6	1,5	2020
	6	1,5	2020
	6	1,5	2020
	6	1,5	2020
	6	1,5	2020
	6	1,5	2020
	6	1,5	2020
	6	1,5	2020
	6	1,5	2020
	6	1,5	2020
	6	1,5	2020
	6	1,5	2020
	6	1,5	2020
	6	1,5	2020
	6	1,5	2020
	6	1,5	2020
	6	1,5	2020
	6	1,5	2020
	6	1,5	2020
	6	1,5	2020
	6	1,5	2020
	6	1,5	2020
	6	1,5	2020
	6	1,5	2020
	6	1,5	2020
	6	1,5	2020
	6	1,5	2020
	6	1,5	2020
	6	1,5	2020
	6	1,5	2020
	6	1,5	2020
	3	0,75	2020
	3	0,75	2020
	3	0,75	2020
	3	0,75	2020
	3	0,75	2020
	3	0,75	2020
	3	0,75	2020
	3	0,75	2020
	3	0,75	2020
	3	0,75	2020
	3	0,75	2020
	3	0,75	2020
	3	0,75	2020
	3	0,75	2020
	3	0,75	2020
	3	0,75	2020
	3	0,75	2020
	3	0,75	2020

3	0,75	2020
3	0,75	2020
3	0,75	2020
3	0,75	2020
3	0,75	2020
3	0,75	2020
3	0,75	2020
3	0,75	2020
3	0,75	2020
3	0,75	2020
3	0,75	2020
3	0,75	2020
3	0,75	2020
3	0,75	2020
3	0,75	2020
3	0,75	2020
3	0,75	2020
3	0,75	2020
3	0,75	2020
3	0,75	2020
3	0,75	2020
3	0,75	2020
3	0,75	2020
3	0,75	2020
3	0,75	2020
3	0,75	2020
3	0,75	2020
3	0,75	2020
3	0,75	2020
3	0,75	2020
3	0,75	2020
3	0,75	2020
3	0,75	2020
3	0,75	2020
3	0,75	2020
3	0,75	2020
3	0,75	2020
3	0,75	2020
3	0,75	2020
3	0,75	2020
3	0,75	2020
3	0,75	2020
3	0,75	2020
3	0,75	2020
3	0,75	2020
3	0,75	2020
3	0,75	2020
3	0,75	2020
3	0,75	2020
3	0,75	2020

3	0,75	2020
3	0,75	2020
3	0,75	2020
3	0,75	2020
3	0,75	2020
3	0,75	2020
3	0,75	2020
3	0,75	2020
3	0,75	2020
3	0,75	2020
3	0,75	2020
3	0,75	2020
3	0,75	2020
3	0,75	2020
3	0,75	2020
3	0,75	2020
3	0,75	2020
3	0,75	2020
3	0,75	2020
3	0,75	2020
3	0,75	2020
3	0,75	2020
3	0,75	2020
3	0,75	2020
3	0,75	2020
3	0,75	2020
3	0,75	2020
3	0,75	2020
3	0,75	2020
3	0,75	2020
3	0,75	2020
3	0,75	2020
3	0,75	2020
3	0,75	2020
3	0,75	2020
3	0,75	2020
3	0,75	2020
3	0,75	2020
3	0,75	2020
3	0,75	2020
3	0,75	2020
3	0,75	2020
3	0,75	2020
3	0,75	2020
3	0,75	2020
3	0,75	2020
3	0,75	2020
3	0,75	2020
3	0,75	2020
3	0,75	2020

	3	0,75	2020
	3	0,75	2020
	3	0,75	2020
	3	0,75	2020
	3	0,75	2020
	3	0,75	2020
	3	0,75	2020
	3	0,75	2020
	3	0,75	2020
	3	0,75	2020
	3	0,75	2020
	3	0,75	2020
	3	0,75	2020
	3	0,75	2020
	3	0,75	2020
	3	0,75	2020
	3	0,75	2020
	3	0,75	2020
	3	0,75	2020
	3	0,75	2020
	3	0,75	2020
	3	0,75	2020
	3	0,75	2020
	3	0,75	2020
	3	0,75	2020
	3	0,75	2020
	3	0,75	2020
	3	0,75	2020
	3	0,75	2020
	3	0,75	2020
	3	0,75	2020
	3	0,75	2020
	3	0,75	2020
	3	0,75	2020
	3	0,75	2020
	3	0,75	2020
	3	0,75	2020
	3	0,75	2020
	3	0,75	2020
	3	0,75	2020
	3	0,75	2020
	3	0,75	2020
	3	0,75	2020
	3	0,75	2020
	3	0,75	2020
	3	0,75	2020
	3	0,75	2020
	3	0,75	2020
	3	0,75	2020
	3	0,75	2020

	3	0,75	2020
	3	0,75	2020
	3	0,75	2020
	3	0,75	2020
	3	0,75	2020
	3	0,75	2020
	3	0,75	2020
	3	0,75	2020
	3	0,75	2020
	3	0,75	2020
	3	0,75	2020
	3	0,75	2020
	3	0,75	2020
	3	0,75	2020
	3	0,75	2020
	3	0,75	2020
	3	0,75	2020
	3	0,75	2020
	3	0,75	2020
	3	0,75	2020
	3	0,75	2020
	3	0,75	2020
	3	0,75	2020
	3	0,75	2020
	3	0,75	2020
	3	0,75	2020
	3	0,75	2020
	3	0,75	2020
	3	0,75	2020
	3	0,75	2020
	3	0,75	2020
	3	0,75	2020
	3	0,75	2020
	3	0,75	2020
	3	0,75	2020
	3	0,75	2020
	3	0,75	2020
	3	0,75	2020
	3	0,75	2020
	3	0,75	2020
	3	0,75	2020
	3	0,75	2020
	3	0,75	2020
	3	0,75	2020
	3	0,75	2020
	3	0,75	2020
	3	0,75	2020
	3	0,75	2020
	3	0,75	2020
	3	0,75	2020

	3	0,75	2020
	685	2356,25	2019
	75	2348,75	2019
	1595	1843,75	2019
	205	1776,25	2019
	2260	1490	2019
	350	1467,5	2019
	870	1467,5	2019
	1900	1460	2019
	870	1452,5	2019
	835	1223,75	2019
	975	1203,75	2019
	1045	1196,25	2019
	550	1177,5	2019
	1820	1170	2019
	470	1132,5	2019
	930	1132,5	2019
	1195	1118,75	2019
	195	1108,75	2019
	930	1107,5	2019
	1475	1068,75	2019
	740	1061	2019
	435	998,75	2019
	690	987,5	2019
	420	960	2019
	710	937,5	2019
	640	935	2019
	205	921,25	2019
	665	916,25	2019
	495	848,75	2019
	1385	831,25	2019
	1605	826,25	2019
	735	788,75	2019
	1935	763,75	2019
	405	761,25	2019
	565	751,25	2019
	350	747,5	2019
	495	738,75	2019
	1345	736,25	2019
	140	735	2019
	325	721,25	2019
	435	708,75	2019
	495	698,75	2019
	985	696,25	2019
	845	691,25	2019
	840	691	2019
	635	688,75	2019
	875	683,75	2019
	825	681,25	2019
	525	677,25	2019

	620	671	2019
	760	665	2019
	565	651,25	2019
	885	646,25	2019
	605	646,25	2019
	965	646,25	2019
	735	643,75	2019
	760	630	2019
	405	606,25	2019
	340	600	2019
	745	596,25	2019
	407	593,75	2019
	280	585	2019
	550	572,5	2019
	260	550	2019
	465	541,25	2019
	610	532,5	2019
	390	527,5	2019
	347	526,75	2019
	560	520	2019
	1640	520	2019
	250	517,5	2019
	520	505	2019
	345	496,25	2019
	217	495,25	2019
	935	483,75	2019
	545	471,25	2019
	390	453,5	2019
	415	448,75	2019
	290	448,5	2019
	690	447,5	2019
	260	445	2019
	650	432,5	2019
	535	425,75	2019
	330	412,5	2019
	615	408,75	2019
	462	405,5	2019
	320	405	2019
	225	396,25	2019
	445	391,25	2019
	325	391,25	2019
	340	390	2019
	395	373,75	2019
	247	371,75	2019
	625	371,25	2019
	375	368,75	2019
	169	344,25	2019
	260	337	2019
	450	332,5	2019
	365	301,25	2019

	340	300	2019
	350	297,5	2019
	270	287,5	2019
	530	282,5	2019
	492	273	2019
	385	272,25	2019
	400	250	2019
	247	227,75	2019
	260	226	2019
	310	211,5	2019
	100	197	2019
	35	32,75	2019
	600	3100	2018
	1710	2707,5	2018
	1375	2388,75	2018
	1170	2252,5	2018
	860	2245	2018
	945	1981,25	2018
	1940	1900	2018
	2595	1748,75	2018
	635	1698,75	2018
	487	1576,75	2018
	1700	1565	2018
	845	1431,25	2018
	245	1416,25	2018
	1970	1362,5	2018
	695	1128,75	2018
	1000	1090	2018
	950	1077,5	2018
	610	1072,5	2018
	630	1067,5	2018
	830	1047,5	2018
	450	1032,5	2018
	0	980	2018
	1020	960	2018
	950	932,5	2018
	300	930	2018
	885	901,25	2018
	630	892,5	2018
	1180	875	2018
	895	868,75	2018
	515	868,75	2018
	495	798,75	2018
	495	783,75	2018
	730	777,5	2018
	870	752,5	2018
	715	748,75	2018
	685	746,25	2018
	985	746,25	2018
	445	741,25	2018

	795	708,75	2018
	0	700	2018
	560	695	2018
	895	688,75	2018
	650	682,5	2018
	930	652,5	2018
	405	631,25	2018
	610	627,5	2018
	345	616,25	2018
	600	605	2018
	645	596,25	2018
	945	592,25	2018
	420	585	2018
	750	582,5	2018
	465	581,25	2018
	420	580	2018
	587	576,75	2018
	915	573,75	2018
	415	553,75	2018
	360	541	2018
	415	538,75	2018
	870	528,5	2018
	470	527,5	2018
	625	516,25	2018
	660	506	2018
	110	502,5	2018
	1000	490	2018
	710	482,5	2018
	690	467,5	2018
	810	467,5	2018
	445	461,25	2018
	324	461	2018
	735	448,75	2018
	340	440	2018
	750	437,5	2018
	330	418,5	2018
	162	395,5	2018
	275	388,75	2018
	255	388,75	2018
	530	387,5	2018
	485	371,25	2018
	350	358,5	2018
	750	327,5	2018
	545	326,25	2018
	465	321,25	2018
	200	295	2018
	190	292,5	2018
	290	274,5	2018
	259	252,75	2018
	395	243,75	2018

	80	180	2018
	320	140	2018
	845	1588,75	2017
	705	1313,75	2017
	337,5	1056,88	2017
	520	940	2017
	785	801,25	2017
	470	792,5	2017
	460	725	2017
	337,5	724,38	2017
	252,5	718,13	2017
	910	707,5	2017
	355	698,75	2017
	347,5	696,88	2017
	500	675	2017
	332,5	648,13	2017
	650	637,5	2017
	395	633,75	2017
	512,5	633,13	2017
	380	620	2017
	265	616,25	2017
	557,5	599,38	2017
	690	592,5	2017
	280	590	2017
	317,5	589,38	2017
	575	586,25	2017
	285	581,25	2017
	400	570	2017
	845	543,75	2017
	430	540	2017
	445	536,25	2017
	405	526,25	2017
	235	508,75	2017
	152,5	503,13	2017
	45	491,25	2017
	442,5	485,63	2017
	317,5	481,88	2017
	345	476,25	2017
	242,5	473,13	2017
	250	472,5	2017
	357,5	471,88	2017
	337,5	469,38	2017
	475	468,75	2017
	95	463,75	2017
	302,5	458,13	2017
	125	456,25	2017
	175	453,75	2017
	362,5	450,63	2017
	440	450	2017
	280	442,5	2017

	505	433,75	2017
	492,5	428,13	2017
	310	427,5	2017
	460	415	2017
	240	405	2017
	287,5	401,88	2017
	550	397,5	2017
	342,5	395,63	2017
	450	392,5	2017
	335	383,75	2017
	330	382,5	2017
	427,5	376,88	2017
	457,5	374,38	2017
	260	372,5	2017
	240	365	2017
	497,5	364,38	2017
	337,5	359,38	2017
	267,5	356,88	2017
	270	342,5	2017
	252,5	338,13	2017
	150	337,5	2017
	385	331,25	2017
	415	323,75	2017
	367,5	321,88	2017
	450	312,5	2017
	445	311,25	2017
	240	310	2017
	460	305	2017
	220	295	2017
	325	291,25	2017
	392,5	278,13	2017
	225	276,25	2017
	177,5	254,38	2017
	290	252,5	2017
	207,5	246,88	2017
	170	242,5	2017
	335	223,75	2017
	457,5	194,38	2017
	110	187,5	2017
	275	183,75	2017
	180	132,5	2017
	145	66,25	2017
	945	1566,25	2016
	705	1268,75	2016
	470	1157,5	2016
	135	1038,75	2016
	300	1025	2016
	640	952,5	2016
	505	936,25	2016
	422,5	833,13	2016

	727,5	794,38	2016
	357,5	746,88	2016
	605	746,25	2016
	470	737,5	2016
	770	727,5	2016
	477,5	689,38	2016
	400	675	2016
	567,5	671,88	2016
	87,5	666,88	2016
	180	635	2016
	565	626,25	2016
	535	623,75	2016
	175	618,75	2016
	480	600	2016
	642,5	590,63	2016
	252,5	588,13	2016
	255	583,75	2016
	450	582,5	2016
	282,5	580,63	2016
	370	555	2016
	310	547,5	2016
	542,5	538,13	2016
	590	527,5	2016
	545	526,25	2016
	550	517,5	2016
	150	507,5	2016
	220	505	2016
	227,5	501,88	2016
	320	500	2016
	65	496,25	2016
	435	488,75	2016
	470	482,5	2016
	237,5	479,38	2016
	325	468,75	2016
	330	462,5	2016
	305	461,25	2016
	505	446,25	2016
	395	441,25	2016
	265	436,25	2016
	505	431,25	2016
	400	430	2016
	180	425	2016
	450	422,5	2016
	387,5	416,88	2016
	265	416,25	2016
	282,5	403,13	2016
	215	398,75	2016
	247,5	381,88	2016
	200	370	2016
	350	362,5	2016

	297,5	349,38	2016
	260	345	2016
	282,5	335,63	2016
	175	333,75	2016
	172,5	323,13	2016
	182,5	315,63	2016
	187,5	311,88	2016
	355	298,75	2016
	210	297,5	2016
	295	286,25	2016
	242,5	280,63	2016
	210	277,5	2016
	250	272,5	2016
	360	265	2016
	200	260	2016
	400	260	2016
	87,5	251,88	2016
	302,5	245,63	2016
	340	240	2016
	30	217,5	2016
	195	208,75	2016
	205	206,25	2016
	280	200	2016
	120	200	2016
	260	195	2016
	145	171,25	2016
	115	148,75	2016
	85	121,25	2016
	110	97,5	2016
	695	1168,75	2015
	1285	1153,75	2015
	540	985	2015
	420	942,5	2015
	550	887,5	2015
	655	881,25	2015
	532,5	863,13	2015
	565	856,25	2015
	257,5	834,38	2015
	375	828,75	2015
	1285	796,25	2015
	487,5	751,88	2015
	675	711,25	2015
	575	703,75	2015
	575	693,75	2015
	410	672,5	2015
	487,5	671,88	2015
	240	660	2015
	582,5	655,63	2015
	157,5	654,38	2015
	395	653,75	2015

	265	651,25	2015
	675	651,25	2015
	337,5	644,38	2015
	527,5	641,88	2015
	445	616,25	2015
	385	601,25	2015
	277,5	564,38	2015
	320	550	2015
	297,5	544,38	2015
	395	541,25	2015
	570	537,5	2015
	212,5	525,63	2015
	345	516,25	2015
	430	515	2015
	185	508,75	2015
	242,5	498,13	2015
	170	487,5	2015
	60	470	2015
	270	462,5	2015
	200	445	2015
	327,5	431,88	2015
	365	431,25	2015
	380	427,5	2015
	297,5	424,38	2015
	90	417,5	2015
	367,5	411,88	2015
	340	395	2015
	437,5	389,38	2015
	270	387,5	2015
	565	381,25	2015
	470	377,5	2015
	322,5	375,63	2015
	460	365	2015
	277,5	364,38	2015
	412,5	358,13	2015
	80	355	2015
	110	352,5	2015
	320	350	2015
	235	348,75	2015
	215	343,75	2015
	150	342,5	2015
	167,5	341,88	2015
	285	341,25	2015
	215	338,75	2015
	155	338,75	2015
	122,5	338,13	2015
	302,5	335,63	2015
	325	326,25	2015
	340	325	2015
	295	323,75	2015

	260	320	2015
	295	318,75	2015
	215	313,75	2015
	110	307,5	2015
	340	305	2015
	262,5	295,63	2015
	137,5	294,38	2015
	230	277,5	2015
	50	272,5	2015
	350	267,5	2015
	167,5	266,88	2015
	265	261,25	2015
	20	260	2015
	112,5	253,13	2015
	185	246,25	2015
	282,5	225,63	2015
	140	215	2015
	220	215	2015
	120	210	2015
	75	208,75	2015
	30	37,5	2015
	590	1190	2014
	450	1135	2014
	587,5	974,38	2014
	1110	970	2014
	362,5	965,63	2014
	502,5	945,63	2014
	260	880	2014
	445	878,75	2014
	475	853,75	2014
	450	852,5	2014
	765	838,75	2014
	680	832,5	2014
	437,5	814,38	2014
	585	811,25	2014
	505	776,25	2014
	695	733,75	2014
	320	692,5	2014
	570	692,5	2014
	80	675	2014
	325	671,25	2014
	575	666,25	2014
	550	640	2014
	135	638,75	2014
	115	623,75	2014
	520	612,5	2014
	560	610	2014
	475	608,75	2014
	580	595	2014
	402,5	590,63	2014

	545	586,25	2014
	320	575	2014
	230	572,5	2014
	445	571,25	2014
	347,5	569,38	2014
	250	567,5	2014
	285	556,25	2014
	300	550	2014
	532,5	548,13	2014
	595	528,75	2014
	242,5	525,63	2014
	580	525	2014
	400	510	2014
	215	493,75	2014
	280	482,5	2014
	225	478,75	2014
	262,5	478,13	2014
	482,5	468,13	2014
	160	465	2014
	192,5	463,13	2014
	585	451,25	2014
	762,5	450,63	2014
	65	436,25	2014
	245	431,25	2014
	260	425	2014
	237,5	424,38	2014
	265	416,25	2014
	177,5	414,38	2014
	557,5	411,88	2014
	220	410	2014
	397,5	409,38	2014
	400	405	2014
	320	390	2014
	347,5	389,38	2014
	355	388,75	2014
	327,5	381,88	2014
	302,5	375,63	2014
	197,5	364,38	2014
	282,5	355,63	2014
	160	355	2014
	290	347,5	2014
	465	346,25	2014
	545	346,25	2014
	297,5	344,38	2014
	170	342,5	2014
	265	341,25	2014
	700	340	2014
	170	337,5	2014
	120	325	2014
	180	325	2014

	305	318,75	2014
	250	317,5	2014
	130	307,5	2014
	415	303,75	2014
	412,5	303,13	2014
	305	296,25	2014
	217,5	294,38	2014
	297,5	294,38	2014
	255	293,75	2014
	110	277,5	2014
	410	262,5	2014
	160	260	2014
	157,5	259,38	2014
	260	255	2014
	165	236,25	2014
	232,5	228,13	2014
	160	220	2014
	155	218,75	2014
	260	215	2014
	232,5	198,13	2014
	130	92,5	2014
	620	2017,5	2013
	570	1312,5	2013
	330	1205	2013
	1390	1167,5	2013
	320	985	2013
	1260	970	2013
	480	910	2013
	237,5	854,38	2013
	100	835	2013
	650	832,5	2013
	310	727,5	2013
	480	695	2013
	555	693,75	2013
	550	692,5	2013
	225	658,75	2013
	545	656,25	2013
	525	643,75	2013
	615	643,75	2013
	542,5	643,13	2013
	445	631,25	2013
	330	592,5	2013
	267,5	591,88	2013
	482,5	590,63	2013
	290	587,5	2013
	382,5	580,63	2013
	560	577,5	2013
	425	576,25	2013
	252,5	573,13	2013
	305	556,25	2013

	242,5	553,13	2013
	230	552,5	2013
	190	552,5	2013
	365	551,25	2013
	440	550	2013
	455	548,75	2013
	425	538,75	2013
	417,5	524,38	2013
	240	495	2013
	310	490	2013
	530	482,5	2013
	340	480	2013
	332,5	478,13	2013
	620	475	2013
	405	471,25	2013
	190	470	2013
	465	463,75	2013
	595	448,75	2013
	465	446,25	2013
	345	428,75	2013
	435	418,75	2013
	405	416,25	2013
	412,5	398,13	2013
	235	393,75	2013
	365	391,25	2013
	177,5	389,38	2013
	387,5	386,88	2013
	220	385	2013
	450	382,5	2013
	215	378,75	2013
	437,5	374,38	2013
	330	372,5	2013
	255	363,75	2013
	370	362,5	2013
	630	362,5	2013
	147,5	351,88	2013
	230	337,5	2013
	370	332,5	2013
	240	330	2013
	340	325	2013
	292,5	323,13	2013
	295	318,75	2013
	260	315	2013
	295	296,25	2013
	430	287,5	2013
	275	283,75	2013
	150	282,5	2013
	387,5	281,88	2013
	285	281,25	2013
	340	280	2013

	175	263,75	2013
	192,5	248,13	2013
	235	228,75	2013
	250	222,5	2013
	117,5	219,38	2013
	187,5	211,88	2013
	202,5	210,63	2013
	325	191,25	2013
	110	172,5	2013
	160	170	2013
	1640	1430	2012
	475	1058,75	2012
	590	1027,5	2012
	380	975	2012
	335	833,75	2012
	510	807,5	2012
	660	795	2012
	420	775	2012
	465	756,25	2012
	530	742,5	2012
	445	711,25	2012
	510	697,5	2012
	240	695	2012
	270	657,5	2012
	535	633,75	2012
	200	620	2012
	380	610	2012
	480	600	2012
	450	582,5	2012
	190	577,5	2012
	450	577,5	2012
	335	558,75	2012
	405	551,25	2012
	350	547,5	2012
	660	545	2012
	455	543,75	2012
	475	543,75	2012
	290	542,5	2012
	290	537,5	2012
	330	522,5	2012
	455	513,75	2012
	410	512,5	2012
	380	500	2012
	355	498,75	2012
	345	476,25	2012
	360	470	2012
	310	467,5	2012
	430	457,5	2012
	220	450	2012
	125	431,25	2012

	1335	418,75	2012
	265	416,25	2012
	305	411,25	2012
	290	402,5	2012
	325	401,25	2012
	380	395	2012
	580	395	2012
	340	395	2012
	330	392,5	2012
	285	391,25	2012
	70	382,5	2012
	335	378,75	2012
	50	372,5	2012
	255	368,75	2012
	140	355	2012
	335	353,75	2012
	330	352,5	2012
	290	352,5	2012
	315	348,75	2012
	430	347,5	2012
	630	347,5	2012
	170	342,5	2012
	100	325	2012
	220	325	2012
	290	322,5	2012
	200	310	2012
	285	301,25	2012
	160	300	2012
	375	283,75	2012
	210	282,5	2012
	440	280	2012
	180	265	2012
	170	257,5	2012
	305	256,25	2012
	150	247,5	2012
	80	240	2012
	100	235	2012
	100	235	2012
	220	220	2012
	180	215	2012
	160	205	2012
	140	205	2012
	115	198,75	2012
	140	195	2012
	210	192,5	2012
	100	165	2012
	80	135	2012
	1380	1345	2011
	500	1105	2011
	570	1062,5	2011

	760	1050	2011
	30	827,5	2011
	440	790	2011
	530	782,5	2011
	555	773,75	2011
	150	772,5	2011
	290	752,5	2011
	50	702,5	2011
	295	658,75	2011
	240	630	2011
	200	620	2011
	485	591,25	2011
	265	571,25	2011
	345	566,25	2011
	410	562,5	2011
	180	560	2011
	140	555	2011
	675	538,75	2011
	210	532,5	2011
	430	522,5	2011
	515	518,75	2011
	240	510	2011
	260	495	2011
	245	491,25	2011
	610	477,5	2011
	1350	477,5	2011
	185	471,25	2011
	100	465	2011
	385	461,25	2011
	200	460	2011
	300	455	2011
	465	436,25	2011
	280	430	2011
	365	426,25	2011
	325	411,25	2011
	390	407,5	2011
	345	406,25	2011
	410	402,5	2011
	200	400	2011
	250	397,5	2011
	435	388,75	2011
	160	375	2011
	410	372,5	2011
	80	370	2011
	550	367,5	2011
	190	347,5	2011
	210	327,5	2011
	380	325	2011
	285	321,25	2011
	305	321,25	2011

	290	312,5	2011
	375	303,75	2011
	410	302,5	2011
	240	300	2011
	405	286,25	2011
	120	285	2011
	285	281,25	2011
	260	280	2011
	220	265	2011
	190	262,5	2011
	170	252,5	2011
	280	250	2011
	140	245	2011
	170	242,5	2011
	170	242,5	2011
	240	240	2011
	50	232,5	2011
	200	230	2011
	500	225	2011
	210	222,5	2011
	170	222,5	2011
	180	215	2011
	185	201,25	2011
	235	198,75	2011
	185	161,25	2011
	155	158,75	2011
	215	158,75	2011
	110	157,5	2011
	120	150	2011
	150	137,5	2011
	90	67,5	2011
	300	1395	2010
	320	1010	2010
	170	972,5	2010
	370	962,5	2010
	505	926,25	2010
	255	913,75	2010
	1520	910	2010
	255	903,75	2010
	790	827,5	2010
	620	795	2010
	210	762,5	2010
	880	760	2010
	290	752,5	2010
	435	743,75	2010
	525	721,25	2010
	255	648,75	2010
	360	640	2010
	430	637,5	2010
	930	602,5	2010

	530	592,5	2010
	400	585	2010
	375	563,75	2010
	330	552,5	2010
	405	541,25	2010
	295	538,75	2010
	405	531,25	2010
	380	505	2010
	505	486,25	2010
	380	485	2010
	90	472,5	2010
	285	466,25	2010
	430	457,5	2010
	315	438,75	2010
	350	437,5	2010
	280	430	2010
	220	430	2010
	210	427,5	2010
	375	423,75	2010
	330	422,5	2010
	310	417,5	2010
	305	416,25	2010
	340	415	2010
	455	413,75	2010
	335	413,75	2010
	420	405	2010
	380	400	2010
	150	397,5	2010
	310	397,5	2010
	310	397,5	2010
	265	396,25	2010
	215	373,75	2010
	250	372,5	2010
	180	370	2010
	580	365	2010
	335	353,75	2010
	360	350	2010
	505	346,25	2010
	245	341,25	2010
	40	340	2010
	280	340	2010
	250	337,5	2010
	260	335	2010
	290	332,5	2010
	210	332,5	2010
	200	320	2010
	295	318,75	2010
	340	315	2010
	350	307,5	2010
	280	300	2010

	315	293,75	2010
	255	293,75	2010
	125	291,25	2010
	160	290	2010
	355	288,75	2010
	270	277,5	2010
	70	267,5	2010
	180	265	2010
	140	260	2010
	90	247,5	2010
	275	228,75	2010
	150	217,5	2010
	130	192,5	2010
	205	191,25	2010
	390	177,5	2010
	130	172,5	2010
	130	167,5	2010
	80	120	2010
	75	118,75	2010
	775	1553,75	2009
	1020	1155	2009
	655	1033,75	2009
	490	982,5	2009
	345	816,25	2009
	490	772,5	2009
	600	760	2009
	770	752,5	2009
	280	710	2009
	210	702,5	2009
	310	702,5	2009
	435	673,75	2009
	340	645	2009
	695	643,75	2009
	40	640	2009
	250	637,5	2009
	845	631,25	2009
	455	623,75	2009
	410	622,5	2009
	405	616,25	2009
	450	612,5	2009
	270	607,5	2009
	360	570	2009
	155	558,75	2009
	210	552,5	2009
	265	536,25	2009
	150	512,5	2009
	360	510	2009
	350	507,5	2009
	330	502,5	2009
	230	497,5	2009

	535	488,75	2009
	635	478,75	2009
	475	478,75	2009
	315	478,75	2009
	270	477,5	2009
	410	472,5	2009
	320	450	2009
	335	443,75	2009
	370	442,5	2009
	80	430	2009
	230	427,5	2009
	550	427,5	2009
	360	415	2009
	200	410	2009
	395	393,75	2009
	385	386,25	2009
	360	380	2009
	250	372,5	2009
	260	370	2009
	180	360	2009
	290	342,5	2009
	545	336,25	2009
	265	331,25	2009
	250	327,5	2009
	270	322,5	2009
	180	320	2009
	200	310	2009
	400	305	2009
	425	301,25	2009
	180	300	2009
	310	297,5	2009
	300	295	2009
	330	287,5	2009
	230	287,5	2009
	450	262,5	2009
	265	261,25	2009
	70	257,5	2009
	285	256,25	2009
	80	240	2009
	180	235	2009
	160	235	2009
	130	232,5	2009
	160	230	2009
	180	230	2009
	145	206,25	2009
	90	202,5	2009
	140	175	2009
	150	157,5	2009
	200	140	2009
	30	82,5	2009

	100	45	2009
	730	1082,5	2008
	560	1010	2008
	70	987,5	2008
	445	981,25	2008
	360	980	2008
	380	965	2008
	360	790	2008
	190	757,5	2008
	430	747,5	2008
	640	695	2008
	555	678,75	2008
	405	621,25	2008
	645	601,25	2008
	475	598,75	2008
	300	585	2008
	285	581,25	2008
	555	568,75	2008
	195	553,75	2008
	330	547,5	2008
	215	533,75	2008
	190	527,5	2008
	280	520	2008
	615	518,75	2008
	455	503,75	2008
	235	503,75	2008
	525	496,25	2008
	495	468,75	2008
	405	461,25	2008
	180	455	2008
	205	451,25	2008
	380	450	2008
	295	448,75	2008
	350	447,5	2008
	425	446,25	2008
	450	442,5	2008
	250	442,5	2008
	320	440	2008
	315	438,75	2008
	265	436,25	2008
	450	432,5	2008
	320	430	2008
	300	425	2008
	295	413,75	2008
	525	406,25	2008
	180	405	2008
	285	386,25	2008
	285	381,25	2008
	220	375	2008
	375	373,75	2008

	325	371,25	2008
	430	367,5	2008
	335	363,75	2008
	255	363,75	2008
	160	360	2008
	475	358,75	2008
	285	356,25	2008
	265	351,25	2008
	150	347,5	2008
	405	346,25	2008
	125	341,25	2008
	160	330	2008
	280	330	2008
	335	328,75	2008
	210	322,5	2008
	200	310	2008
	240	310	2008
	440	290	2008
	140	285	2008
	405	281,25	2008
	170	262,5	2008
	290	262,5	2008
	190	247,5	2008
	70	247,5	2008
	245	241,25	2008
	120	230	2008
	270	217,5	2008
	220	215	2008
	160	215	2008
	180	215	2008
	240	200	2008
	195	168,75	2008
	220	165	2008
	80	150	2008
	90	142,5	2008
	140	100	2008
	1485	1341,25	2007
	510	1087,5	2007
	560	1010	2007
	515	948,75	2007
	470	827,5	2007
	335	803,75	2007
	505	786,25	2007
	540	775	2007
	380	765	2007
	290	762,5	2007
	160	705	2007
	640	695	2007
	280	660	2007
	345	646,25	2007

	340	640	2007
	230	637,5	2007
	575	633,75	2007
	110	617,5	2007
	430	617,5	2007
	510	617,5	2007
	360	600	2007
	465	596,25	2007
	235	558,75	2007
	400	545	2007
	335	538,75	2007
	60	535	2007
	240	510	2007
	555	498,75	2007
	420	495	2007
	290	492,5	2007
	375	483,75	2007
	130	482,5	2007
	495	463,75	2007
	410	442,5	2007
	600	430	2007
	200	430	2007
	585	421,25	2007
	180	415	2007
	160	410	2007
	315	408,75	2007
	170	382,5	2007
	360	380	2007
	315	378,75	2007
	375	373,75	2007
	390	367,5	2007
	265	366,25	2007
	505	356,25	2007
	375	353,75	2007
	310	342,5	2007
	200	340	2007
	315	338,75	2007
	285	331,25	2007
	250	322,5	2007
	150	317,5	2007
	385	316,25	2007
	265	316,25	2007
	255	313,75	2007
	205	306,25	2007
	555	303,75	2007
	170	297,5	2007
	160	295	2007
	170	292,5	2007
	190	287,5	2007
	230	287,5	2007

	325	281,25	2007
	315	278,75	2007
	140	275	2007
	145	266,25	2007
	160	260	2007
	200	260	2007
	120	250	2007
	240	250	2007
	305	246,25	2007
	170	237,5	2007
	220	235	2007
	180	235	2007
	170	232,5	2007
	140	215	2007
	125	211,25	2007
	150	207,5	2007
	150	172,5	2007
	115	133,75	2007
	50	102,5	2007
	50	52,5	2007
	1535	2013,75	2006
	560	1200	2006
	480	1050	2006
	620	995	2006
	1115	858,75	2006
	470	797,5	2006
	450	782,5	2006
	485	771,25	2006
	670	767,5	2006
	220	765	2006
	600	760	2006
	310	707,5	2006
	255	703,75	2006
	520	690	2006
	400	670	2006
	410	662,5	2006
	405	641,25	2006
	640	640	2006
	555	623,75	2006
	290	602,5	2006
	305	586,25	2006
	575	583,75	2006
	455	573,75	2006
	380	555	2006
	305	551,25	2006
	480	545	2006
	190	542,5	2006
	255	538,75	2006
	620	515	2006
	500	505	2006

	630	502,5	2006
	210	492,5	2006
	400	490	2006
	290	452,5	2006
	310	452,5	2006
	470	442,5	2006
	205	441,25	2006
	290	437,5	2006
	450	432,5	2006
	470	417,5	2006
	270	417,5	2006
	290	417,5	2006
	295	413,75	2006
	340	405	2006
	210	402,5	2006
	135	393,75	2006
	350	387,5	2006
	230	387,5	2006
	260	385	2006
	405	381,25	2006
	200	380	2006
	220	375	2006
	195	368,75	2006
	470	367,5	2006
	355	358,75	2006
	270	357,5	2006
	405	356,25	2006
	260	345	2006
	150	337,5	2006
	210	332,5	2006
	405	331,25	2006
	440	330	2006
	295	318,75	2006
	285	311,25	2006
	40	310	2006
	210	307,5	2006
	405	301,25	2006
	255	293,75	2006
	335	293,75	2006
	285	291,25	2006
	80	290	2006
	80	290	2006
	150	287,5	2006
	250	272,5	2006
	50	262,5	2006
	350	247,5	2006
	300	230	2006
	150	227,5	2006
	150	207,5	2006
	260	205	2006

	110	197,5	2006
	270	197,5	2006
	160	195	2006
	100	185	2006
	40	180	2006
	130	172,5	2006
	190	167,5	2006
	150	152,5	2006
	195	128,75	2006
	140	125	2006
	100	75	2006
	1385	2146,25	2005
	970	1282,5	2005
	475	1128,75	2005
	575	1013,75	2005
	395	958,75	2005
	505	936,25	2005
	490	922,5	2005
	540	895	2005
	655	813,75	2005
	465	806,25	2005
	765	801,25	2005
	515	768,75	2005
	365	721,25	2005
	570	702,5	2005
	560	680	2005
	580	675	2005
	455	673,75	2005
	300	665	2005
	530	652,5	2005
	630	607,5	2005
	225	576,25	2005
	900	575	2005
	400	560	2005
	355	548,75	2005
	460	540	2005
	200	540	2005
	370	532,5	2005
	290	532,5	2005
	430	517,5	2005
	465	516,25	2005
	380	515	2005
	265	496,25	2005
	540	485	2005
	295	478,75	2005
	210	477,5	2005
	255	468,75	2005
	325	446,25	2005
	360	440	2005
	465	436,25	2005

	220	435	2005
	200	430	2005
	380	425	2005
	425	406,25	2005
	260	405	2005
	255	393,75	2005
	490	382,5	2005
	290	382,5	2005
	190	377,5	2005
	250	372,5	2005
	275	368,75	2005
	265	361,25	2005
	205	361,25	2005
	260	360	2005
	245	356,25	2005
	355	353,75	2005
	270	342,5	2005
	435	328,75	2005
	220	325	2005
	240	320	2005
	170	312,5	2005
	330	312,5	2005
	220	305	2005
	80	300	2005
	350	297,5	2005
	260	295	2005
	250	292,5	2005
	150	277,5	2005
	260	275	2005
	300	265	2005
	170	262,5	2005
	300	260	2005
	225	241,25	2005
	170	232,5	2005
	180	225	2005
	215	223,75	2005
	310	217,5	2005
	125	201,25	2005
	30	197,5	2005
	150	177,5	2005
	180	145	2005
	110	137,5	2005
	50	72,5	2005
	1100	1415	2004
	405	1171,25	2004
	360	1120	2004
	920	980	2004
	855	923,75	2004
	740	895	2004
	1240	870	2004

	920	870	2004
	505	746,25	2004
	405	716,25	2004
	330	712,5	2004
	335	698,75	2004
	280	690	2004
	210	672,5	2004
	310	667,5	2004
	180	645	2004
	575	613,75	2004
	230	557,5	2004
	380	545	2004
	330	532,5	2004
	265	531,25	2004
	330	512,5	2004
	375	508,75	2004
	430	482,5	2004
	315	478,75	2004
	355	473,75	2004
	395	458,75	2004
	535	448,75	2004
	510	447,5	2004
	380	445	2004
	245	441,25	2004
	200	440	2004
	395	438,75	2004
	255	418,75	2004
	165	416,25	2004
	380	415	2004
	290	412,5	2004
	140	405	2004
	290	402,5	2004
	310	402,5	2004
	245	401,25	2004
	520	400	2004
	315	398,75	2004
	190	397,5	2004
	160	395	2004
	170	392,5	2004
	220	385	2004
	400	380	2004
	180	380	2004
	320	375	2004
	220	355	2004
	295	353,75	2004
	455	353,75	2004
	295	353,75	2004
	290	352,5	2004
	265	351,25	2004
	200	345	2004

	370	342,5	2004
	380	335	2004
	295	333,75	2004
	285	331,25	2004
	330	327,5	2004
	210	327,5	2004
	125	321,25	2004
	140	315	2004
	425	311,25	2004
	295	303,75	2004
	240	300	2004
	180	300	2004
	310	297,5	2004
	270	297,5	2004
	130	292,5	2004
	140	275	2004
	260	275	2004
	205	251,25	2004
	200	250	2004
	260	245	2004
	210	237,5	2004
	220	235	2004
	310	227,5	2004
	200	220	2004
	150	217,5	2004
	100	205	2004
	125	201,25	2004
	150	197,5	2004
	35	193,75	2004
	180	185	2004
	100	180	2004
	185	176,25	2004
	125	171,25	2004
	160	165	2004
	200	160	2004
	65	156,25	2004
	70	137,5	2004
	145	121,25	2004
	100	75	2004
	1612,00	3393,00	2024
	757,00	2869,25	2024
	1229,00	2847,25	2024
	607,00	2831,75	2024
	1852,00	2613,00	2024
	1634,00	2298,50	2024
	1153,00	2258,25	2024
	1085,00	2021,25	2024
	1507,00	1996,75	2024
	1387,00	1836,75	2024
	1191,00	1817,75	2024

1183,00	1765,75	2024
674,00	1738,50	2024
1612,00	1723,00	2024
990,00	1717,50	2024
876,00	1709,00	2024
1049,00	1672,25	2024
831,00	1587,75	2024
464,00	1576,00	2024
1169,00	1552,25	2024
1453,00	1543,25	2024
1003,00	1530,75	2024
1341,00	1505,25	2024
2167,00	1501,75	2024
966,00	1441,50	2024
1199,00	1439,75	2024
914,00	1418,50	2024
720,00	1345,00	2024
352,00	1328,00	2024
2016,00	1264,00	2024
2167,00	1261,75	2024
982,00	1255,50	2024
1897,00	1244,25	2024
719,00	1199,75	2024
1612,00	1173,00	2024
329,00	1162,25	2024
779,00	1144,75	2024
765,00	1141,25	2024
666,00	1096,50	2024
1859,00	1084,75	2024
861,00	1065,25	2024
1012,00	1043,00	2024
630,00	1017,50	2024
869,00	997,25	2024
1027,00	986,75	2024
749,00	957,25	2024
756,00	949,00	2024
570,00	942,50	2024
1042,00	935,50	2024
817,00	934,25	2024
1080,00	920,00	2024
892,00	919,00	2024
810,00	912,50	2024
750,00	897,50	2024
735,00	883,75	2024
712,00	878,00	2024
862,00	875,50	2024
682,00	870,50	2024
794,00	868,50	2024
704,00	856,00	2024
1259,00	855,75	2024

974,00	833,50	2024
630,00	817,50	2024
540,00	815,00	2024
555,00	798,75	2024
450,00	792,50	2024
482,00	790,50	2024
817,00	784,25	2024
584,00	776,00	2024
1168,00	772,00	2024
733,00	753,25	2024
252,00	753,00	2024
742,00	745,50	2024
607,00	697,75	2024
570,00	678,50	2024
629,00	677,25	2024
1499,00	674,75	2024
367,00	626,75	2024
622,00	625,50	2024
735,00	615,75	2024
330,00	607,50	2024
412,00	605,00	2024
510,00	583,50	2024
525,00	571,25	2024
585,00	571,25	2024
517,00	569,25	2024
434,00	564,50	2024
525,00	561,25	2024
524,00	561,00	2024
434,00	553,50	2024
264,00	538,00	2024
405,00	521,25	2024
614,00	519,50	2024
487,00	512,75	2024
517,00	509,25	2024
525,00	499,25	2024
510,00	472,50	2024
322,00	460,50	2024
719,00	449,75	2024
562,00	444,50	2024
232,00	443,00	2024
525,00	404,25	2024
569,00	372,25	2024
322,00	344,50	2024
225,00	308,25	2024
90,00	287,50	2024
142,00	280,50	2024
292,00	235,00	2024
147,00	214,75	2024
132,00	51,00	2024

tions. For each player, these variables were
specified percentile.
sulting in atypical participation rates and
